# Bacterial calpains and the evolution of the calpain (C2) family of peptidases

**DOI:** 10.1186/s13062-015-0095-0

**Published:** 2015-11-02

**Authors:** Neil D. Rawlings

**Affiliations:** Wellcome Trust Sanger Institute and the EMBL-European Bioinformatics Institute, Wellcome Trust Genome Campus, Hinxton, Cambridgeshire, CB10 1SA UK

**Keywords:** Calpain, Calpamodulin, Androglobin, Peptidase, Evolution, Alignment, Phylogenetic tree, Bacteria

## Abstract

Homologues of calpain, often thought to be an essential, cytoplasmic, calcium-dependent cysteine endopeptidase found exclusively in eukaryotes, have been found in bacterial proteomes. The homologues lack calcium-binding sites, have differing domain architectures, and can be secreted or membrane-associated. Homologues are rare and occur in a minority of bacterial phyla and often in a minority of species in a genus. However, the differences in domain architecture argue against a recent, horizontal gene transfer from a eukaryote. From analysis of a phylogenetic tree and absence of homologues in archaea, calpains in eukaryotes may be derived from genes horizontally transferred from a bacterium.

**Reviewers:** This article was reviewed by L. Aravind and Frank Eisenhaber.

**Electronic supplementary material:**

The online version of this article (doi:10.1186/s13062-015-0095-0) contains supplementary material, which is available to authorized users.

## Findings

Calpains are cysteine endopeptidases from peptidase family C2 [1]. Chicken (*Gallus gallus)* calpain, a typical example, is a heterodimer of a large and a small subunit. The peptidase active site is present on the large subunit and consists of a Cys, His, Asn catalytic triad in which the cysteine bears the nucleophilic thiol group and the histidine is the general base, with its ring orientated in the optimal conformation by the asparagine. A glutamine N-terminal to the cysteine is an important residue that helps stabilize the acyl-intermediate formed during substrate hydrolysis by forming a structure known as the “oxyanion hole” [2]. The large subunit is a multidomain protein, and both the large and small subunits can bind calcium ions via domains known as EF hand structures [3]. The peptidase domain, which bears the active site residues, also binds calcium via two binding sites. The protein is synthesized as a precursor, and activation occurs when intracellular calcium increases and calpain undergoes autodigestion [4]. In humans (*Homo sapiens*) and many other mammals, there are calpains sensitive to different concentrations of intracellular calcium. In the human, there are fifteen proteins with domains homologous to the peptidase domain of chicken calpain. Two of these homologues, calpamodulin and androglobin, are not peptidases because the active site cysteines have been replaced [5, 6].

Of the fifteen human calpains, eight are ubiquitous in cells, whereas six have a restricted distribution. Calpain-3 is found in skeletal muscle, calpains 8 and 9 in the gastrointestinal tract, calpain-11 and androglobin in the testis, calpain-12 in the hair follicle, and calpamodulin in embryonic muscle. Deficiency in a calpain can lead to disease: platelet dysfunction (calpain-1), muscular dystrophy (calpain-3), stress-induced gastric ulcer (calpains 8 and 9), and calpain-2 deficiency is embryonically lethal [7]. Calpains are associated with digestion of cytoskeletal, muscle and lens proteins such as vimentin [8], myelin basic protein [9], aggrecan core protein [10], tropomyosin alpha-1 chain [11], amphiphysin [12], alpha-actinin-1 [13], filaggrin-2 [14], microtubule-associated protein tau [15], beta crystallins [16, 17] and spectrin beta chain [18].

The structure of the peptidase domain of rat (*Rattus norvegicus*) calpain-2 was solved and the fold was found to be similar to that of cysteine peptidases of the papain family (peptidase family C1) [4]. Because the folds are similar and the active site residues are the same and occur in the same order within the protein sequence both families C1 and C2 are included in the same peptidase clan (or superfamily) CA [1].

Calpain homologues have been found in a variety of non-chordate species, including *Drosophila melanogaster* (four homologues), *Caenorhabditis elegans* (fourteen homologues), *Schistosoma mansoni* (seven homologues), plants (phytocalpain), fungi (PalB peptidase), yeast (Rim13 peptidase), *Leishmania* and *Trypanosoma* spp. (approximately twenty homologues, depending on the species) and bacteria. The domain architecture of many of these proteins differs considerably from the chordate calpains, with only the peptidase domain being in common [19].

The only bacterial calpain homologue that has been subject to any biochemical characterization is the Tpr protein from *Porphyromonas gingivalis* [20, 21]. The only substrates identified were skimmed milk and the synthetic substrate Pz-Pro-Leu-Gly-Pro-d-Arg (where Pz is *N*α-(4-phenylazo)benzyloxycarbonyl), where cleavage occurs between Leu and Gly. Tos-LysCH_2_Cl (TLCK), Tos-PheCH_2_Cl (TPCK), ethylenediaminetetraacetic acid (EDTA), and iodoacetate were found to be inhibitory, which is suggestive of a calcium-dependent cysteine peptidase. However phenylmethane sulfonylfluoride (PMSF) was also found to be inhibitory, and given that PMSF is predominantly a serine peptidase inhibitor, it has been argued that the characterized entity may have been a mixture of peptidases [11]. If this is the case, then no bacterial calpain homologue has been biochemically characterized.

The evolution of the calpain family had been studied by Jékely & Friedrich [22] when few genomes had been completely sequenced and no bacterial homologues were known. Given the distribution of calpain homologues and the variety of domains that can embellish the peptidase domain, the purpose of this study is to determine in what kind of organism the first calpain-like homologue appeared, to determine when and in which order non-peptidase domains were incorporated into the proteins and to re-examine the conclusions of Jékely & Friedrich [22].

## Methods

### Detection of homologues

The chicken calpain sequence was chosen to represent the family, and homologues were found by using the peptidase domain from the known structure to search for homologues in the NCBI non-redundant protein sequence database using BlastP [23, 24]. TBlastN searches were performed against complete genome sequences of organisms where homologues were expected but not detected. In addition, individual proteomes were downloaded from the NCBI Genomes site [25] and submitted to the *MEROPS* batch Blast [26]. Because only counts and occurrences of genes were to be analysed, only the longest splice variant of a calpain homologue was retained. An alignment was made of the peptidase domain sequences using MUSCLE [27]. Sequences were extracted from this alignment to make a second alignment containing only one sequence from each phylum of organisms (see Additional file 1: Figure S1). This alignment was submitted to the HMMER server [28], which creates a HMMER model from the alignment, and the NCBI non-redundant protein sequence database was searched to find more distant homologues.

### Prediction of domain architecture, signal peptides and transmembrane domains

Complete protein sequences of all homologues detected by the method above were submitted to PfamScan [29]. The results file was analysed using a bespoke Perl program to create an “architecture string” for each sequence, which lists all the domain names in sequence order. Where more than one domain was predicted to occur in the same region of the sequence, the domain with the lowest E value was selected. In order to predict signal peptides and transmembrane domains, complete protein sequences of selected calpain homologues were submitted to the HMMER server [28, 30] as implemented at the EMBL-European Bioinfomatics Insitute (www.ebi.ac.uk/Tools/hmmer).

### Alignment and phylogenetic tree

Because so many homologues were detected, a full sequence alignment of peptidase domains and a phylogenetic tree determined from this alignment would be too large to present here. Instead, sequences were selected from the family. To ensure that the selection was representative, the following criteria were used: 1) sequences corresponding to characterized peptidases; 2) all homologues from the following model organisms: human, mouse, chicken, zebrafish, *Branchiostoma floridae*, *Ciona intestinalis*, *Drosophila melanogaster*, *Caenorhabditis elegans*, *Arabidopsis thaliana*, *Saccharomyces cerevisiae* and *Plasmodium falciparum*; 3) all homologues from prokaryotes; and 4) a representative sequence from each phylum possessing a homologue. In order to root the phylogenetic tree, the peptidase domain of papain, a member of the structurally-related cysteine endopeptidases from family C1, was included in the selection to provide an outgroup.

An alignment of peptidase domains from these selected homologues was made using ClustalW [31]. A neighbour-joining tree was generated from the FastA file using FastTree 2.1 [32] using default parameters. In order to assess the robustness of the branching pattern, the FastTree tree was compared to trees generated from 1000 bootstraps alignment. The program SeqBoot.exe from the Phylip package [33] was used to generate alignments by randomly sampling the ClustalW alignment, and the program CompareToBootStrap.pl (from the FastTree package) was used to compare trees generated from these alignments with the FastTree tree.

## Results and Discussion

### Homologues found

In total, 3795 calpain homologues were detected (see Additional file 2: Table S1 for the complete list). These were found in eukaryotes and bacteria, but no homologue was detected in any archaeon. Of these homologues, 637 are missing one or more active site residues because they are fragments, and a further 337 are considered not to be peptidases because one or more active site residues are not conserved.

All completely sequenced animal and plant genomes possessed a homologue except those of the three-toed sloth (*Choloepus hoffmani*), the sea lamprey (*Petromyzon marinus*), the sea squirt *Ciona savigni*, the chlorophytes *Chlorella variabilis* and *Coccomyxa subellipsoidea* and the rhodophyte *Cyanidioschyzon merolae*. The absences from animals are most likely the results of errors in sequencing and assembly rather than because genes have been lost, especially in the case of *C. savigni* because homologues are known from *C. intestinalis*. The absences in the algae may result from genuine gene losses. Absences of homologues in fungi and protozoa were more widespread: no homologues were detected in the ascomycetes *Eremothecium cymbalariae*, *Passalora fulva*, *Schizosaccharomyces japonicas* and *S. pombe*; the basidiomycetes *Postia placenta*, *Rhodotorula glutinis* and *Tremella mesenterica*; the apicomplexan *Cryptosporidian hominis*; any cercozoan, diplomonad, microsporidian or mycetozoan. Although no homologous protein has been identified in the basidiomycete *Dichomitus squalens*, a fragment could be detected with a TBlastN search against the genome sequence.

Homologues in bacteria were few, with most bacterial genomes lacking a homologue. Sixty-seven homologues were found in species in the following seven phyla: Actinobacteria (1 species of 3 with completely sequenced genomes), Bacteroidetes (8 of 173), Chloroflexi (1 of 17), Cyanobacteria (14 of 53), Firmicutes (19 of 681), Planctomyces (1, at the time of writing there were no species with a completely sequenced genome) and Proteobacteria (11 of 815). Homologues were found in a minority of species in a phylum, and in some cases examples were found in only a minority of species in a genus (for example, *Streptomyces* where three species are each known to possess a homologue out of 30 species with completely sequenced genomes). With the exception of *Porphyromonas gingivalis*, no homologues were found in well-known bacteria such as *Escherichia coli*, *Vibrio cholera*, *Bacillus subtilis, Staphylococcus aureus*, or *Streptomyces griseus*.

**Fig. 1:**
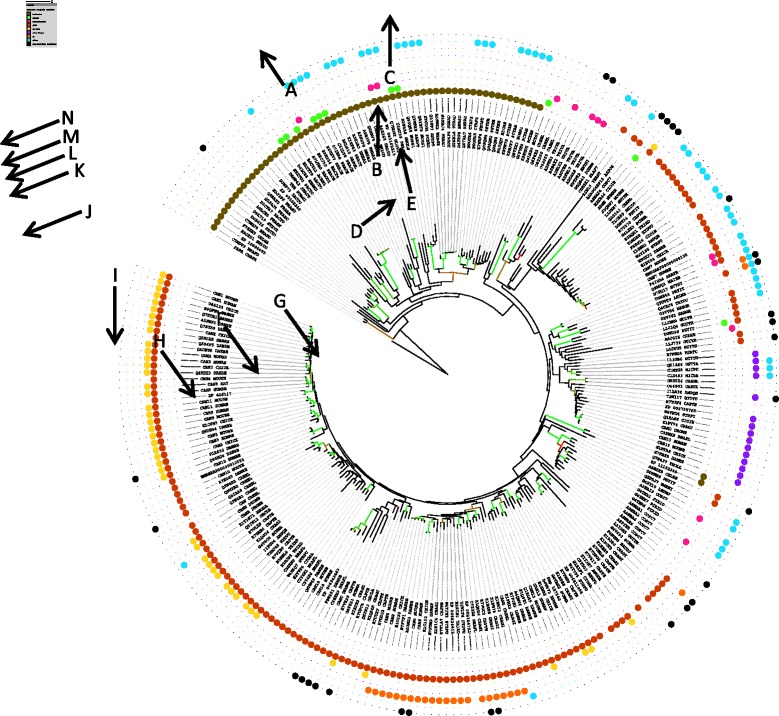
Phylogenetic tree for selected members of the calpain family. The tree generated by FastTree is shown. Coloured branches indicate significant bootstrap values where light green is 100 %, dark green is >90 %, brown is >85 % and red >80 %. Tips are labelled with UniProt identifiers (where possible). Coloured dots indicate classification, additional domains and non-peptidase homologues, where brown is Bacteria, green is a signal peptide, pink is a transmembrane region, red is a CIII domain, yellow is an EF-hand structure domain, blue is a zinc-finger domain, orange is a C2 domain, turquoise is another additional domain, and black is a non-peptidase homologue. For the key to sequences, see Additional file 4: Table S2

The peptidase domains from 267 members were selected from the family for further analysis (see Methods for the criteria used). The alignment, plus the peptidase domain from papain, is shown in Additional file 3: Figure S2. The phylogenetic tree derived from this alignment is shown in Fig. 1 ﻿(the key to which is shown in Additional file 4: Table S2) . It is only these 267 homologues that are considered in the discussion below.

### Active site residues

Of the 267 homologues, 29 are non-peptidase homologues because one or more of the active site residues has been replaced and the protein presumably no longer functions as a peptidase. The most widely studied of these is calpamodulin. This situation is not uncommon with enzymes, and these non-peptidase homologues presumably perform other protein-binding functions. There are 29 non-peptidase homologues included in the phylogenetic tree. The nucleophilic cysteine is missing in calpamodulin, androglobin, and the clp-3 protein from *Caenorhabditis elegans*. The histidine that acts as a general base is also replaced not only in these sequences, but additionally in calpain C from *Drosophila melanogaster*. The asparagine that orientates the active site histidine ring can be replaced by aspartic acid and the protein may still be a functional peptidase, as in the case of the PalB peptidase from *Saccharomyces cerevisiae* [34]. There are other homologues with such a replacement, including calpain C from *D. melanogaster* and a calpain 3-like protein from zebrafish. In addition to the other non-peptidase homologues, sequences without the asparagine conserved are a homologue from *Frankia* sp. EUN1f and the F47F6.5 protein from *C. elegans*.

### Calcium-binding sites

From the structure of the complex of rat calpain 2 with calpastatin, the calcium binding sites in the peptidase domain were found to consist of Ile89, Gly91, Asp96, Glu175 (site 1) and Glu292, Asp299, Gln319, Glu323 (site 2) [4]. All of these residues are conserved in more than half of the sequences in the alignment (Additional file 3: Figure S2) with the exception of Ile89, which can be replaced by any aliphatic hydrophobic residue (Leu, Val, Met or Ala). As noted by Franz et al. [35], neither calcium-binding site is conserved in human calpain-7, and only site 1 is found in some of the bacterial calpain homologues (from *Frankia* spp, *Streptomyces viridochromogenes* and cyanobacteria).

### Domains found in calpain homologues

**Fig. 2:**
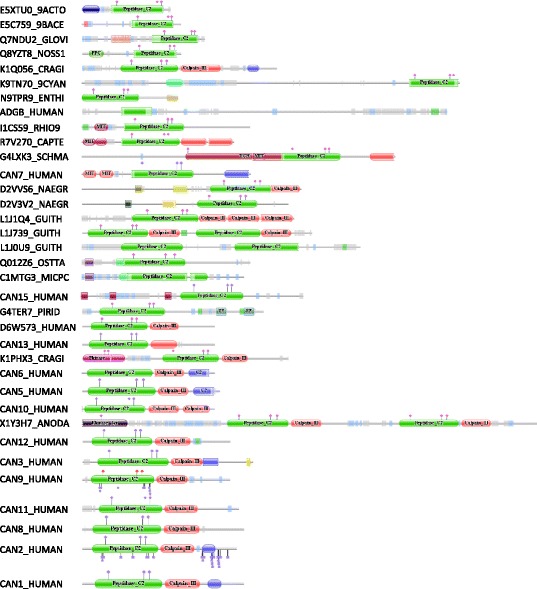
Representative domain architectures for calpain homologues. Domain architecture diagrams were downloaded from the Pfam website (http://pfam.xfam.org). Diagrams are shown in the same order as they occur in Fig. 1, starting with homologues from bacteria and proceeding in a clockwise direction. The UniProt identifier for each protein is shown on the left. In each domain architecture diagram sequence length is indicated by the horizontal grey line (the “string”): sequence lengths are approximately proportional to one another. Domains are shown in the order they appear in the sequence as “beads on a string”. A domain with rounded ends is complete; a domain with a ragged end matches only a fragment of the complete domain as defined in Pfam. All calpain domains are shown in green and labelled “peptidase C2”; other domains are shown in other colours. Small rectangles indicate transmembrane regions (pink), disordered regions (grey), regions of low complexity (pale blue), and regions of coiled-coil (green). Blue “lollipops” at the top edge of a domain indicate active site residues (in the order Cys, His, Asn). Carbohydrate attachment sites are shown as square-topped lollipops at the bottom edge of a domain (where known). Key to sequences (unp = unnamed protein, domains are shown as strings with each domain separated by a tilde, pC2 = calpain domain): E5XTU0_9ACTO, unp (*Segniliparus rugosus* strain ATCC BAA-974), pC2; E5C759_9BACE, unp (*Bacteroides* sp. D2), pC2; Q7NDU2_GLOVI, Glr4140 protein (*Gloeobacter violaceus* strain PCC 7421), HemolysinCabind x 8 ~ pC2 Q8YZT8_NOSS1, Alr0369 protein (*Nostoc* sp. strain PCC 7120), PPC ~ pC2; K1Q056_CRAGI, calpain-A (*Crassostrea gigas* (Pacific oyster)), pC2 ~ Calpain III x 2 ~ EF hand 8; K9TN70_9CYAN, unp (*Oscillatoria acuminata* strain PCC 6304), DUF4114 ~ pC2; N9TPR9_ENTHI, unp (*Entamoeba histolytica* strain HM-1:IMSS-A), pC2 ~ LIM; ADGB_HUMAN, androglobin (human), pC2; I1CS59_RHIO9, unp (*Rhizopus delemar*), MIT ~ pC2; R7V270_CAPTE, unp (*Capitella teleta*), MIT x 2 ~ pC2 ~ Calpain III x 2; G4LXK3_SCHMA, calpain-7 (*Schistosoma mansoni*), UCH ~ MIT ~ pC2 ~ Calpain III; CAN7_HUMAN, calpain-7 (human), MIT ~ pC2 ~ Calpain III; D2VVS6_NAEGR, unp (*Naegleria gruberi*), TPR 1 ~ TPR 11 ~ pC2 ~ Calpain III; D2V3V2_NAEGR, unp (*Naegleria gruberi*), TPR 8 ~ TPR 11 ~ pC2; L1J1Q4_GUITH, unp (*Guillardia theta* strain CCMP2712), pC2 ~ Calpain III x 3; L1J739_GUITH, unp (*Guillardia theta* strain CCMP2712), pC2 ~ Calpain III ~ pC2 ~ Calpain III; L1J0U9_GUITH, unp (*Guillardia theta* strain CCMP2712), pC2 x 2; Q012Z6_OSTTA, CG1391-PB, isoform B (*Ostreococcus tauri*), UBA 4 ~ zf GRF ~ pC2; C1MTG3_MICPC, unp (*Micromonas pusilla* strain CCMP1545), UBA 4 ~ zf GRF ~ pC2~; CAN15_HUMAN, calpain-15 (human), zf RANBP x 3 ~ pC2; G4TER7_PIRID, unp (*Piriformospora indica* strain DSM 11827), pC2 ~ ZZ x 2; D6W573_HUMAN, HCG1988128 isoform CRA_b (human), pC2 ~ Calpain III; CAN13_HUMAN, calpain-13 (human), pC2 ~ Calpain III; K1PHX3_CRAGI, calpain-9 (*Crassostrea gigas* (Pacific oyster)), Pkinase x 2 ~ pC2 ~ Calpain III; CAN6_HUMAN, calpain-6 (human), pC2 ~ Calpain III ~ C2; CAN5_HUMAN, calpain-5 (human), pC2 ~ Calpain III ~ C2; CAN10_HUMAN, calpain-10 (human), pC2 ~ Calpain III x 2; X1Y3H7_ANODA, unp (*Anopheles darlingi*), Pkinase Tyr ~ pC2 ~ Calpain III ~ pC2 ~ Calpain III; CAN12_HUMAN, calpain-12 (human), pC2 ~ Calpain III; CAN3_HUMAN, calpain-3 (human), pC2 ~ Calpain III ~ Calpain u2 ~ EF-hand 5; CAN9_HUMAN, calpain-9 (human), pC2 ~ Calpain III; CAN11_HUMAN, calpain-11 (human), pC2 ~ Calpain III; CAN8_HUMAN, calpain-8 (human), pC2 ~ Calpain III; CAN2_HUMAN, calpain-2 (human), pC2 ~ Calpain III ~ EF-hand 8; CAN1_HUMAN, calpain-1 (human), pC2 ~ Calpain III ~ EF-hand 8

Many calpain homologues are multidomain proteins. Figure 2 shows examples of the different domain architectures known for calpains; all examples are taken from the 267 homologues used in this analysis. All calpain homologues by definition possess the “peptidase_C2” domain, the domain that bears the active site residues and performs the proteolytic activity. For a number of hypothetical or uncharacterized homologues the only predicted domain is the peptidase_C2 domain, and examples include the T11A5.6 and W05G11.4 gene products from *Caenorhabditis elegans*.

Most eukaryotic calpain homologues possess a CIII domain, the function of which is unknown [19].

Many calpains bind calcium through EF hand structure domains, and a variety of different EF-hand domains are found, with the commonest being EF-hand_8 and EF-hand_6 (others are EF_hand_1, EF_hand_5 and EF_hand_7). However, whether an EF hand structure is a member of one family or another is often debatable because the E values are very similar. All EF hand structure domains are structurally related and members of the same clan [3]. EF hand structures occur in other proteins, notably the ciliary protein EFCAB7 (“EF-hand calcium-binding domain-containing protein 7”) which has been shown to be a positive regulator of hedgehog signalling. In addition to the EF hand structures, EFCAB7 also contains two globular domains that have been shown to be homologous to the CIII domains of calpains and which interact with the protein IQCE, which in turn is membrane-associated [36]. CIII domains may therefore be a means by which calpains can associate with membranes.

Calpain-15 possesses zinc-finger domains (zf-RanBP domains) which interact with nucleic acids [37]. The number of zinc fingers varies from two (e.g., the water flea *Daphnia pulex)* to seven (e.g., some bees from the genera *Apis* and *Bombus*). Zinc fingers from the zf-H2 and zf-H2_2 families are found in calpain-15 from the gnat *Culex pipiens quinquefasciatus*. Zinc fingers are also found in hypothetical proteins from the cryptophyte *Guillardia theta* and the chlorophytes *Micromonas* and *Ostreococcus*, which are zf-GRF domains.

A C2 domain targets a protein to a cell membrane [38]. C2 domains are found in calpains 5 and tra-3 and in an unnamed homologue found in marsupials, monotremes, birds and reptiles. MIT (microtubule interacting and transport) domains, which are found in vacuolar sorting proteins, characterize calpain-7 and are also found in the Rim13 peptidase. Phytocalpain possesses laminin domains. A UCH (ubiquitin carboxy-terminal hydrolase) domain is found in a single calpain-7 orthologue from *Schistosoma mansoni*, and a SNARE (soluble *N*-ethylmaleimide-sensitive factor attachment protein receptor) domain is found in a single calpain-3 orthologue.

A variety of other domains are predicted to occur in other, uncharacterized calpain homologues. These include: WXG100 (three homologues from *Herpetosiphon aurantiacus* and one from *Segniliparus rugosus*); Brix (a homologue from *Ichthyophthirius multifiliis*); CARDB (nine domains in a homologue from *Cyanothece* sp. PCC 7822); Abhydrolase_3 (*Emericella nidulans*); PPC (homologues from *Microcoleus* sp. PCC 7113, *Microcystis aeruginosa*, *Fischerella* sp. JSC-11, *Cyanothece* sp. PCC 7822, *Anabaena variabilis* and *Nostoc* sp. PCC 7120); Collagen (*Leptosphaeria maculans*); TPR_1, TPR_8 and TPR_11 (homologues from *Naegleria gruberi*); YwqJ-deaminase and Tox-PL (*Salinispora tropica*); Pkinase_Tyr (*Anopheles darlingi*); ZZ (three homologues from *Piriformospora indica*), DUF1935 (*Leishmania* and *Trypanosoma* species); F5_F8_type_C (*Bacteroides* species); LIM (*Entamoeba* species); and HemolysinCabind (eight domains in a homologue from *Gloeobacter violaceus*). It is entirely possible that the association with the peptidase domain for any of these is the result of erroneous gene assembly, where an exon from one gene has been incorrectly assigned to the gene up- or downstream.

### Origin of the family

The phylogenetic tree (Fig. 1) appears to represent an origin for the calpain gene within the bacteria, followed by vertical gene transfer into eukaryotes. This is supported by the facts that most of the bacterial homologues are very divergent from the eukaryote homologues and that many bacterial calpain homologues have only the peptidase domain; the embellishment with other domains happened mainly in the eukaryotes. However, there are two arguments against this view. First of all, there are no archaeon calpain homologues, and second, the limited distribution of calpain homologues amongst the bacteria is difficult to explain. Homologues are found in a minority of bacterial phyla (mainly Bacteroidetes, Cyanobacteria, Firmicutes and Proteobacteria, with single homologues in Chloroflexi and Planctomycetes), which argues against an origin in the ancestral bacterium, and in a minority of bacteria for which the genomes have been completely sequenced. Even within a genus, bacteria with homologues are very much in a minority (*Streptomyces* is a good example). For the Tpr peptidase, not all strains of *Porphyromonas gingivalis* have a homologue: it is known from strains W83 and TDC60, but not ATCC 33277. This distribution is reminiscent of multiple (and very recent, in evolutionary terms) horizontal gene transfers, and the alternative explanation, multiple gene losses, is unlikely. It would also seem unlikely that horizontal gene transfer occurred from a eukaryote to a bacterium because the eukaryote and bacterial sequences are so divergent. A much more likely scenario would be multiple horizontal gene transfers within the bacteria, then a horizontal gene transfer from a bacterium to a eukaryote, perhaps accompanying the development of the mitochondrion (or chloroplast) from an endosymbiont bacterium.

It has been estimated that *E. coli* and *Salmonella typhimurium* diverged between 100 and 160 million years ago. A study of 16S RNA sequences has shown that in some species of bacteria from the same genus, the sequence divergence can be as great (*e.g., Buchnera*) or greater (*e.g., Pseudomonas*) than that between *Escherichia* and *Salmonella* [39]. For the calpain gene to be present in some species of a genus and not others, any horizontal transfers would have occurred within the last 100 million years or so.

It is clear from the phylogenetic tree that there is not a constant rate of evolution within the family, because the branches to *Saccharomyces* Rim13 peptidase and the androglobins are much longer than any of the other branches, indicating a faster rate of mutation for these sequences. However, it is also clear that the divergences between sequences from closely related bacteria are frequently much greater than between calpain orthologues of human and chicken (which diverged approximately 310 million years ago [40]). It could be argued that the bacterial sequences are evolving more rapidly than those from eukaryotes, but there still seems to be an anomaly between sequence divergence and distribution amongst bacteria.

In support of the hypothesis that horizontal gene transfer occurred between bacteria, the phylogenetic tree (Fig. 1) shows multiple, distinct groups of homologues from bacteria in the phyla Firmicutes, Bacteriodetes and Cyanobacteria. If the tree matched the organism evolution, then all homologues from bacteria in the Firmicutes, for example, should be clustered together. There are also two bacterial sequences, from *Photorhabdus* and *Diplorickettsia*, that cluster with the eukaryote sequences: these are presumably derived from horizontal gene transfers from eukaryote to prokaryote.

Arguments against the hypothesis of horizontal gene transfers are the degree of dissimilarity, the different domain architectures and length between the sequences. This diversity in sequences argues against a recent origin. However, the bacterial species that have retained (or gained) a calpain homologue are from a variety of environments, being free-living or symbiotic, aerobic, anaerobic or autotrophic. Without knowing the function of a calpain homologue in a bacterium it is perhaps impossible to understand the distribution.

It might be expected that the original calpain was a simple protein containing just a peptidase domain, and that the source organism was free-living, perhaps in an unusual, and poorly researched, environment. A reconstructed tree of life based on 31 orthologous domains from 191 organisms whose genomes had been completely sequenced shows a fundamental divergence amongst the bacteria into Gram-positive (Firmicutes and Actinobacteria) and Gram-negative, with the Firmicutes as the most ancient of the bacterial phyla [41]. So it is possible that the last common ancestor of the bacteria with a calpain homologue was a member of the Firmicutes. If the calpain homologue was derived by vertical gene transfer, then it would be expected that the gene would have been retained in most of the species closely related to the source organism.

The rooted tree shows that thirteen sequences are derived from nodes that predate the bacterial-eukaryote split (Fig. 1, node A). These sequences are from Gram positive bacteria that are members of the phyla Firmicutes (*Brachybacterium*, *Streptomyces* and *Frankia*) and Actinobacteria (*Leucobacter*). It is possible that any of these sequences might be representative of the ancestral calpain. The most divergent sequences are from *Brachybacterium faecium* and *B. paraconglomeratum*. Both sequences are short, containing only the peptidase domain. However, neither *Brachybacterium* species is free-living: both were isolated from poultry litter [42], implying that they are symbionts living in the avian gastrointestinal duct. *Frankia alni* is a nitrogen-fixing symbiont associated with alder and myrtle [43]. The other *Frankia* spp are uncharacterized bacteria, but these may also not be free-living. The calpain homologue from *Leucobacter* is a C-terminal fragment bearing only the peptidase domain, so it is impossible to tell what the complete domain architecture is. *Leucobacter* is a soil organism tolerant to high levels of chromium [44]. Homologues are not present in the four other *Leucobacter* species with completely-sequenced genomes.

### Bacterial calpains

All bacterial homologues of calpain are predicted to be peptidases, with the single exception of the homologue from *Frankia* sp. EUN1f. All genes for bacterial calpain homologues are found on the chromosome: none are on plasmids.

The sequence length of bacterial homologues varies immensely from 258 residues (*Brachybacterium faecium*) to 3,754 residues (*Salinispora tropica*). A homologue from *Nocardia brasiliensis* is predicted to be 15,203 residues, but only the N-terminal half of the peptidase domain can be detected. In most bacterial homologues the peptidase domain is the C-terminal domain, but the homologue from *Granulibacter bethesdensis* is an exception where the peptidase domain is N-terminal (residues 49–247 in a sequence of 681 residues).

Most eukaryote calpain homologues are known to be intracellular, but this may not be the case for the bacterial homologues. Of the 66 bacterial calpain homologues identified, only three are predicted to have signal peptides: homologues from *Geobacter lovleyi* (UniProt:B3E5A8), *Segniliparus rotundus* (D6ZC85) and *Bacteroides thetaiotaomicron* (Q8A0R1). These may be periplasmic proteins. However, other mechanisms for protein secretion exist in bacteria. The *Gloeobacter violaceus* calpain homologue possesses HemolysinCaBind domains, which are known to be important in metallopeptidases known as serralysins for secretion outside of the cell wall and which bind calcium [45]. Unlike in serralysin where the HemolysinCaBind domains are at the C-terminus, in the *Gloeobacter* calpain homologue the eight HemolysinCaBind domains are N-terminal to the peptidase domain. If these domains are used for secretion, then it is possible that the calpain homologue functions extracellularly.

Three bacterial calpains, from *Bacteroides* sp. D2, *Salinispora tropica* and *S. pacifica*, are predicted to have transmembrane helices and therefore to be membrane-associated.

Most bacterial calpain homologues are simple proteins with only the peptidase domain. However, some sequences are multidomain. Domains that occur just in just one homologue are YwqJ-deaminase (*Salinispora*). The deaminase domain is also found in a putative ribonuclease YwqJ from *Bacillus subtilis* (Uniprot: P96722), but no protein with this domain has been characterized biochemically. The *Salinispora* calpain homologue is also the only one to have Tox-PL domains. As mentioned above, the calpain homologue from *Gloeobacter* is the only one to possess HemolysinCaBind domains. One homologue from *Cyanothece* has a CARDB domain, which is predicted to be a cell-adhesion domain also found in glycoyl hydrolases and peptidases, and may indicate that the protein is attached to the cell membrane. Homologues from *Herpetosiphon* and *Segniliparus* have WXG100 domains. This is a domain of approximately 100 residues containing a Trp-Xaa-Gly motif. Proteins with a WXG100 domain may be toxins and be exported by the poorly characterized TS77/ESX/ESAT-6 secretion system [46, 47]. The domain had been thought restricted to Firmicutes, but *Segniliparus* is a member of the phylum Chloroflexi. All of the homologues from *Bacteroides* have a discoidin (or F5_F8_type_C) domain, originally identified in human blood coagulation proteins. Proteins with a discoidin domain are predominantly extracellular. Homologues from cyanobacteria may have PPC domains, which are C-terminal propeptides associated predominantly with secreted peptidases such as vibriolysin [48]. Unusually, the PPC domains are N-terminal to the peptidase domain in the calpain homologues from cyanobacteria.

### Eukaryote calpains

Most eukaryotic calpain homologues are cytoplasmic, but the following eight homologues are predicted to be membrane-associated because transmembrane helices are predicted: clec-119 from *Caenorhabditis elegans*; TVAG_023460, TVAG_183710, TVAG_256510 and TVAG_423090 from *Trichomonas vaginalis*; and phytocalpains from *Arabidopsis thaliana* and *Zea mays*.

Only a single calpain homologue is found in any bacterial species, but multiple genes are found in eukaryotes, which are the result of many gene duplications. The number of paralogues varies with species: for example, there is only one homologue in *Arabidopsis thaliana*, but 15 in human and 44 in the dinoflagellate *Symbiodinium minutum*. Considering the duplications that gave rise to the human calpains, the first duplication occurred at node B (see Fig. 1). This presumably occurred early in eukaryote evolution. Calpain-7 and androglobin are derived from a duplication at node C. Calpain-15 is derived from a gene duplication that occurred at node D. Both of these duplications also occurred in early eukaryote evolution because all branches derived from these nodes contain calpains from protozoa, plants or fungi.

Calpains −13 and −14 are derived from a gene duplication at node G. This duplication event occurred in early vertebrate evolution, because invertebrate sequences (from *Schistosoma*) are ancestral. Calpain-5 and calpamodulin arose via a gene duplication at node H. This occurred early in vertebrate evolution, because both calpains are present in zebrafish. A further gene duplication at node I in early metazoan evolution, gave rise to calpain-10 (and its equivalent in *Drosophila*, calpain-C.

All the remaining gene duplications took place in early vertebrate evolution. A gene duplication at node J gave rise to calpain-12; one at node K gave rise to calpain-3; one at node L gave rise to calpain-9; one at node M gave rise to calpain-11; one at node N saw the origins of calpains −2 and −8; and the final duplication one at node O gave rise to calpain-1.

Comparing this evolution scheme to that of Jékely & Friedrich [22], who used the complete sequences rather than the peptidase domains, it is clear that the gene duplications are in a different order. Jékely & Friedrich [22] concluded that the calpain family in chordates resulted from two gene duplications before the divergence of the protostomes and deuterostomes, *i.e.,* before 670 million years ago (MYA), a single fusion event and two genome duplications - the first in an ancestral chordate approximately 550–450 MYA and the second 450–400 MYA. The genome duplications are based on the ‘2R hypothesis’ of Ohno [49]. The order of divergence was calpain 15 (most divergent); calpains 5 and 6; calpains 3 and the calpain small subunit; and finally calpains 1, 2, 8 and 9.

In the present study, the presence of paralogues of calpains 5, 6, 7, 10 and 15 in *Hydra* confirms that the gene duplications that gave rise to the CAPN15 and CAPN5/6 genes occurred before the divergence of the protostomes and deuterostomes, and dates the event even older to before the divergence of the Radiata and Bilatera. The duplication that gave rise to the genes ancestral to CAPN14/14 and CAPN1/2/3/8/9/11/12 also occurred before the divergence of Protostomia and Deuterostomia. There is no evidence from Fig. 1 to suggest that calpains 1, 2, 3, 8, 9, 11 and 12, which all have distribution restricted to chordates, were generated by any events other than a series of gene duplications. One must be aware, however, that the tree-drawing method is bound to produce bifurcations rather than the simultaneous multiple duplications that tetraploidization would generate, and the branch lengths for gene duplications in Fig. 1 are short. Putnam et al. [50], in their analysis of the amphioxus (*Branchiostoma floridae*) genome concluded, from comparison of the synteny between the amphioxus and human genomes, that two rounds of genome duplication occurred in the vertebrate lineage after the divergence of cephalochordates, and identified 17 ancient chordate linkage groups. Table 1 shows the human calpain homologues and their chromosome locations, from which it is possible to map to which linkage group and duplication product each gene belongs. For two genes to be derived by genome duplication, each should be a member of a different linkage group and duplication product. From Table 1 it is immediately apparent that the CAPN2, CANP8, CANP9, CANP13 and CANP14 genes are all members of the same linkage group (11) and duplication product (A), and must therefore be the result of gene duplication events and not genome duplications (as had been suggested by Jékely & Friedrich). The ancestral CANP2/8/9/13/14 (11A), CANP3 (11B), CANP1 (11C) and CANP11 (11D), therefore, ought to be products of two rounds of genome duplications. However, the close similarity between calpains 1 and 2, argues for a much more recent gene duplication event. The CAPN5 (9C) and CAPN6 (9A) genes, and the CAPN6 (13B) and CAPN7 (13C) genes could also be the product of genome duplications, but any calpain genes representing 9B, 9D, 13A and 13D would have been lost. The six other calpain homologue genes are from different linkage groups and two tetraploidization events would have resulted in three calpain gene losses for each, which would have been a surprising large loss (18 genes) compared to the six genes that were retained.

**Table 1:** Human calpain genes

Gene	Chromosome location	Segment ID	Paralogy group	Duplication product
ADGB	6q24.3	6.2	4	C
CANP7	3p25.1	3.1	13	C
CANP15	16p13.3	16.1	15	B
CANP14	2p23-21	2.2	11	A
CANP13	2p21-22	2.2	11	A
CANP6	Xq23	X.7	9, 13	A, B
CANP5	11q13.5	11.7	9	C
CANP10	2q37.3	2.9	10	A
CANP12	19q13.2	19.2	1, 2	C, D
CANP3	15q15.1	15.1	11	B
CANP9	1q42.2	1.16	11	A
CANP11	6p21.1	6.4-5	11	D
CANP8	1q41	1.16	11	A
CANP2	1q41	1.16	11	A
CANP1	11q13.1	11.4-5	11, 17	C, D

Genes for human calpains are shown in divergence order with the gene derived from the most ancient divergence first. Segment IDs and paralogy groups are taken from Supplementary Table 14 from Putnam et al., [50]. Note that the chromosome locations for the CANP6, CANP12 and CANP1 genes overlap more than one paralogy group

Amphioxus was not included in the set of model organisms from which calpain sequences were selected because there are questions about the gene assembly. Of the eleven sequences showing homology to the calpain peptidase domain, four were partial and did not overlap all of the active site residues. One sequence (XP_002586006) matched a region between the active site Cys and His and thus did not overlap any of the active site residues; the other three overlapped the N-terminal two residues only. Of the seven sequences with complete calpain peptidase domains, two are more closely related to protostome sequences: one (EEN62485) is most closely related to sequences from in the annelid *Capitella* and the Pacific oyster (*Crassostrea gigas*); and another (XP_002590876) is most closely related to *Drosophila* calpains A and B. The others are orthologues and paralogues of vertebrate calpains: one (XP_002611732) is an orthologue of calpain 15; one (EEN46888) an orthologue of calpain 10; one (XP_002603439) is paralogous to calpains 5 and 6; and the other two (XP_002607996 and XP_002607981) are closely related to each other and calpains 1, 2, 3, 8, 9, 11 and 12. The latter three lend support to the vertebrate genome duplication hypothesis described above.

A variety of additional domains have been appended to the peptidase domain of eukaryote calpains and the nodes where these were acquired (or lost) are indicated by letters on the phylogenetic tree (Fig. 1).

Homologues from the sarcomastigophoran *Naegleria* possess TPR (tetratricopeptide repeat) domains, which are involved in protein-protein interactions [51]. Calpain-7 and calpains from fungi have an MIT (microtubule interacting and transport) domain. This domain is involved in protein sorting and transport [52]. Other domains are found in fungi. A calpain from *Emericella* has a fragment resembling the region around the catalytic serine of α/β hydrolase domain, but with the serine replaced by histidine.

CIII domains are found in almost all eukaryote calpains, and were acquired early in eukaryote evolution (node B): they are found in calpains from the apicomplexan *Plasmodium* but not the heterokontophyte *Ectocarpus*. Calpain-III domains were lost in calpain-15 (node E) and other calpains with zinc fingers.

Zinc fingers are associated with binding nucleotides, and are associated with calpain-15 (node E). Deficiency of this enzyme leads to degeneration of the cells forming the small optic lobes in *Drosophila* [53]. Surprisingly, zinc fingers are also associated with calpain homologues from fungi and single-celled organisms. Homologues from the basidiomycete *Piriformospora* have ZZ zinc finger domains and chlorophytes such as *Ostreococcus* have zf_GRF zinc-finger domains. In addition, the chlorophyte calpains possess a UBA (ubiquitin-associated) domain. Other protozoa possess domains that interact with nucleotides. A homologue from *Entamoeba* has a LIM domain, which consists of two zinc finger-like domains. A homologue from *Ichthyophthirius* has a Brix domain which may be important for binding single-stranded RNA [54].

C2 domains are found in calpain-5, calpamodulin and tra-3. These were acquired at node D. Calcium-binding EF hand domains are present in “classical” calpains and were acquired at node I.

## Conclusions

Calpains had been thought to be exclusively eukaryotic. However, proteins with calpain-like cysteine endopeptidase domains occur in both bacteria and eukaryotes.

Homologues have not been found in archaea. The presence of sequences in bacteria apparently derived from a node that predates the bacteria-eukaryota divergence and absence in archaea raises the possibility that the original eukaryote calpain gene was horizontally transferred from a bacterium.

Calpains were thought to be essential proteins in eukaryotes, however homologues are absent in a number of single-celled eukaryotes, including algae and *Schizosaccharomyces*, and some basidiomycetes.

Calpains were also thought to be exclusively cytoplasmic, however some bacterial homologues may be secreted because they are predicted to possess signal peptides. Some bacterial and eukaryote calpain homologues are predicted to possess transmembrane regions, suggesting that activity occurs in close proximity to plasma membranes.

Calpains were thought to be exclusively calcium-dependent, but although some bacterial homologues retain one of the two calcium-binding sites found in the peptidase domain, none possess the EF-hand domain, and most are predicted not to be calcium-dependent.

The distribution of calpain homologues amongst bacteria is unusual. Although homologues are found in several phyla, most phyla do not possess homologues. Within a phylum, only a small minority of species with completely sequenced genomes possess a homologue, and in some cases, e.g., *Streptomyces*, only a small minority of species within a genus possess homologues. Such a limited distribution could be taken as evidence of recent, multiple horizontal gene transfers, however, the diversity in sequence, differences in length and domain architecture are arguments against this. Bacteria with homologues do not occur in similar environments, or share similar unusual metabolic pathways, so what the evolutionary pressure is to maintain a homologue is unknown.

A bacterium possesses only one calpain homologue, but a eukaryote frequently possesses more than one paralogue. Gene duplication in eukaryotes is frequently associated with acquisition of an additional domain, such as a CIII domain, zinc-finger, C2 domain or EF-hand structure. Most vertebrate calpain genes are derived via gene duplication, but there is some limited evidence that some calpain genes result from genome duplication.

## Reviewers’ comments

### Reviewer’s report 1: L. Aravind, National Center for Biotechnology Information, USA

#### Reviewer comments

Neil Rawlings describes the presence of calpains in bacteria and makes the case for their ultimate origin in the bacterial superkingdom. I am convinced by both the calpain affinities of the bacterial proteins and the essence of the proposed evolutionary scenario. Hence, I recommend publication of this work.

The author might want to consider the following points in the revision of the manuscript: the versions fused to the WXG domains are likely to be secreted toxins of the T7SS. Such toxins including those with several types of papain-like fold peptidase domains have been described before here: PMID 22731697. The author lists several domains fused to the peptidase catalytic domain in different types of calpains. However, description of the C-termini of several eukaryotic calpains is unclear: two widespread conserved domains might have bearing on the membrane interactions of calpains (see figure 7 in PMID: 24582806).

The singular of archaea is archaeon rather archaean which could convey a different sense.

Author’s response: *I thank the reviewer for pointing out these two references, and I have incorporated both into the text. However, I would prefer the paper to be more focused on the evolution of the calpain domain and I have chosen not to go into depth concerning additional domains.*

The singular of archaea has been corrected.

### Reviewer’s report 2: Frank Eisenhaber, Bioinformatics Institute A*STAR, Singapore

#### Reviewer comments

The proposed review about the calpain family is timely and it is very carefully and well done.

I suggest to add files containing the lists of calpains together with annotations (taxonomy, complete/fragment, etc.) as well as the reasons for inclusions (e.g., E-values in Blast searches) for future reference. The problem is that any repeated search will result in at least slightly changed outcomes. Further, I suggest an additional figure showing representative domain architecture of calpains.

Author’s response: *To be able to repeat the search exactly, not only are a list of hits required, but also a copy of the seed alignment used for the HMMER3 search and a copy of the UniProtKB sequence library that was searched against. I have provided an additional figure (Additional file**1**: Figure S1) which shows the seed alignment used for the search, and an additional table (Additional file**2**: Table S1) which lists all the hits returned from the HMMER3 search, with their E-values. Note that the table includes more hits than mentioned in the paper, because the UniProtKB database is not non-redundant and in my list only one of a pair of sequences from the same organism was selected if the sequences were identical or more than 94 % identical. I am unable to provide a copy of the UniProtKB library for obvious reasons. Note also that the existing Supplementary Table and Figure have been renumbered.*

A figure (Fig. 2) to show representative domain architectures has been added.

## Additional files

Additional file 1: Figure S1. Protein sequence alignment used for HMMER3 search. The alignment generated by ClustalW is shown. This alignment consists of a representative sequence from every phylum with a known calpain homologue. (DOCX 3054 kb) Additional file 2: Table S1. Homologues detected by HMMER3 search. The homologues detected by searching the UniProt database with the Clustal sequence alignment shown in Additional file 1: Figure S1. The columns are: UniProt identifier and residue range, the E-value, and the organism name. (DOCX 31 kb) Additional file 3: Figure S2. Protein sequence alignment for selected members of the calpain family. The alignment generated by ClustalW is shown. Residues conserved in 80 % of sequences are highlighted in light yellow. Residues that are similar in 80 % of sequences are highlighted in dark yellow. Active site residues are indicated by an asterisk. Calcium-binding residues are indicated by a plus sign. Residue numbers are shown on the right hand side. For the key to sequences, see Additional file 4: Table S2. (TXT 12 kb) Additional file 4: Table S2. Key to sequences. The names of the 267 proteins, the species they are derived from, and the identifier used in Fig. 1 and Additional file 2: Figure S2 are given. The exponent from the expect value from the HMMER search is also shown (“E-value”). (DOCX 199 kb)

## Abbreviations

Abhydrolase: alpha-beta hydrolase
CAPN: calcium-activated neutral peptidase
CARDB: cell adhesion related domain found in bacteria
DUF: domain of unknown function
EDTA: ethylenediaminetetraacetic acid
F5_F8_type_C: F5/8 type C domain
HemolysinCabind: Hemolysin-type calcium-binding repeat
LIM: **L**in11, **I**sl-1 and **M**ec-3 domain
MIT: microtubule interacting and transport
MYA: million years ago
Pkinase_Tyr: protein tyrosine kinase
PMSF: phenylmethane sulfonylfluoride
Pz: *N*α-(4-phenylazo)benzyloxycarbonyl
SNARE: soluble *N*-ethylmaleimide-sensitive factor attachment protein receptor
TLCK: Tos-LysCH_2_Cl
Tos: tosyl
Tox-PL: papain-like toxin
TPCK: Tos-PheCH_2_Cl
TPR: tetratricopeptide repeat
UBA: ubiquitin-associated
UCH: ubiquitin carboxy-terminal hydrolase
Zf: zinc finger
zf_GRF: Gly, Arg, Phe zinc finger
ZZ: zinc finger binding two zinc ions
