# Supplementary material for: Bacterial calpains and the evolution of the calpain (C2) family of peptidases
Source: Biol Direct. 2015 Nov 2;10:66. doi: 10.1186/s13062-015-0095-0 (PMC4631099; doi:10.1186/s13062-015-0095-0)
Supplement: Additional file 2: Table S1. — Homologues detected by HMMER3 search. The homologues detected by searching the UniProt database with the Clustal sequence alignment shown in Additional file 1: Figure S1. The columns are: UniProt identifier and residue range, the E-value, and the organism name. (DOCX 31 kb) [file 13062_2015_95_MOESM2_ESM.docx]

| ***Identifier*** | ***Organism*** | ***Protein/gene name*** |
| --- | --- | --- |
| A0A064B913_ASPOZ | *Aspergillus oryzae* | Rim13 peptidase |
| A0JMG3_DANRE | *Brachydanio rerio* | capn5a |
| A2D9A8_TRIVA | *Trichomonas vaginalis* | TVAG_183710 |
| A2DTE8_TRIVA | *Trichomonas vaginalis* | TVAG_423090 |
| A2E1X1_TRIVA | *Trichomonas vaginalis* | TVAG_164300 |
| A2E4X2_TRIVA | *Trichomonas vaginalis* | TVAG_161170 |
| A2FEL7_TRIVA | *Trichomonas vaginalis* | TVAG_023460 |
| A2FHB3_TRIVA | *Trichomonas vaginalis* | TVAG_256510 |
| A4QNV6_DANRE | *Brachydanio rerio* | zgc:162184 |
| A4X268_SALTO | *Salinispora tropica* | Strop_0483 |
| A4YRP8_BRASO | *Bradyrhizobium* sp. ORS278 | BRADO2763 |
| A5EMK2_BRASB | *Bradyrhizobium* sp. BTAi1 | BBta_5424 |
| A5PMP1_DANRE | *Brachydanio rerio* | capn1b |
| A7E2G3_DANRE | *Brachydanio rerio* | capn12 |
| A7LTS0_BACO1 | *Bacteroides ovatus* | BACOVA_01216 |
| A8HZK8_CHLRE | *Chlamydomonas reinhardtii* | phytocalpain |
| A8J6T6_CHLRE | *Chlamydomonas reinhardtii* | FAP226 |
| A8LCE5_FRASN | *Frankia* sp. EAN1pec | Franean1_0117 |
| A8N4X4_COPC7 | *Coprinopsis cinerea* | CC1G_04674 |
| A8NAE6_COPC7 | *Coprinopsis cinerea* | CC1G_05897 |
| A8NAI9_COPC7 | *Coprinopsis cinerea* | CC1G_05940 |
| A8NBA1_COPC7 | *Coprinopsis cinerea* | CC1G_07471 |
| A8NBA2_COPC7 | *Coprinopsis cinerea* | CC1G_07472 |
| A8NBA6_COPC7 | *Coprinopsis cinerea* | CC1G_07476 |
| A8PIE5_COPC7 | *Coprinopsis cinerea* | CC1G_05906/CC1G_15138 |
| A9B2A3_HERA2 | *Herpetosiphon aurantiacus* | Haur_2853 |
| A9B2P0_HERA2 | *Herpetosiphon aurantiacus* | Haur_2853 |
| A9B3U8_HERA2 | *Herpetosiphon aurantiacus* | Haur_3448 |
| ADGB_HUMAN | *Homo sapiens* | androglobin |
| B0C8L1_ACAM1 | *Acaryochloris marina* | AM1_6343 |
| B0JUQ7_MICAN | *Microcystis aeruginosa* | MAE_46830 |
| B3E5A8_GEOLS | *Geobacter lovleyi* | Glov_2379 |
| B3RIE5_TRIAD | *Trichoplax adhaerens* | TRIADDRAFT_36865 |
| B3RJS5_TRIAD | *Trichoplax adhaerens* | TRIADDRAFT_63582 |
| B3RNL8_TRIAD | *Trichoplax adhaerens* | TRIADDRAFT_21543 |
| B3RQK1_TRIAD | *Trichoplax adhaerens* | calpain-7 |
| B7FWX6_PHATC | *Phaeodactylum tricornutum* | PHATRDRAFT_45234 |
| C0H5K1_PLAF7 | *Plasmodium falciparum* | calpain |
| C1E493_MICSR | *Micromonas* sp. RCC299 | MICPUN_57955 |
| C1MTG3_MICPC | *Micromonas pusilla* | MICPUCDRAFT_58059 |
| C3XWK8_BRAFL | *Branchiostoma floridae* | BRAFLDRAFT_98697 |
| C3Y6N1_BRAFL | *Branchiostoma floridae* | BRAFLDRAFT_74930 |
| C3Y6P6_BRAFL | *Branchiostoma floridae* | BRAFLDRAFT_213643 |
| C3YB23_BRAFL | *Branchiostoma floridae* | BRAFLDRAFT_174202 |
| C3YJZ3_BRAFL | *Branchiostoma floridae* | BRAFLDRAFT_122588 |
| C3ZKN8_BRAFL | *Branchiostoma floridae* | BRAFLDRAFT_129583 |
| C3ZKN9_BRAFL | *Branchiostoma floridae* | BRAFLDRAFT_101136 |
| C6ILT8_9BACE | *Bacteroides* sp. 1_1_6 | BSIG_02709 |
| C7MDV9_BRAFD | *Brachybacterium faecium* | Bfae_19510 |
| C7MGJ8_BRAFD | *Brachybacterium faecium* | Bfae_03130 |
| CAN_CAEEL | *Caenorhabditis elegans* | clp-1 |
| CAN1_HUMAN | *Homo sapiens* | calpain-1 |
| CAN1_MOUSE | *Mus musculus* | calpain-1 |
| CAN10_HUMAN | *Homo sapiens* | calpain-10 |
| CAN10_MOUSE | *Mus musculus* | calpain-10 |
| CAN11_HUMAN | *Homo sapiens* | calpain-11 |
| CAN11_MOUSE | *Mus musculus* | calpain-11 |
| CAN12_HUMAN | *Homo sapiens* | calpain-12 |
| CAN12_MOUSE | *Mus musculus* | calpain-12 |
| CAN13_HUMAN | *Homo sapiens* | calpain-13 |
| CAN13_MOUSE | *Mus musculus* | calpain-13 |
| CAN15_HUMAN | *Homo sapiens* | calpain-15 |
| CAN15_MOUSE | *Mus musculus* | calpain-15 |
| CAN2_CHICK | *Gallus gallus* | calpain-2 |
| CAN2_HUMAN | *Homo sapiens* | calpain-2 |
| CAN2_MOUSE | *Mus musculus* | calpain-2 |
| CAN3_CHICK | *Gallus gallus* | calpain-3 |
| CAN3_HUMAN | *Homo sapiens* | calpain-3 |
| CAN3_MOUSE | *Mus musculus* | calpain-3 |
| CAN5_CAEEL | *Caenorhabditis elegans* | calpain tra-3 |
| CAN5_HUMAN | *Homo sapiens* | calpain-5 |
| CAN5_MOUSE | *Mus musculus* | calpain-5 |
| CAN6_HUMAN | *Homo sapiens* | calpamodulin |
| CAN6_MOUSE | *Mus musculus* | calpamodulin |
| CAN7_HUMAN | *Homo sapiens* | calpain-7 |
| CAN7_MOUSE | *Mus musculus* | calpain-7 |
| CAN8_HUMAN | *Homo sapiens* | calpain-8 |
| CAN8_MOUSE | *Mus musculus* | calpain-8 |
| CAN8_RAT | *Rattus norvegicus* | calpain-8 |
| CAN9_HUMAN | *Homo sapiens* | calpain-9 |
| CAN9_MOUSE | *Mus musculus* | calpain-9 |
| CANA_DROME | *Drosophila melanogaster* | calpain A |
| CANB_DROME | *Drosophila melanogaster* | calpain B |
| CANC_DROME | *Drosophila melanogaster* | Calpain-C |
| CAND_DROME | *Drosophila melanogaster* | Calpain-D |
| CANX_CHICK | *Gallus gallus* | calpain C |
| D0MWB9_PHYIT | *Phytophthora infestans* | PITG_02442 |
| D0MZ44_PHYIT | *Phytophthora infestans* | PITG_02997 |
| D0ND88_PHYIT | *Phytophthora infestans* | PITG_08806 |
| D0NT34_PHYIT | *Phytophthora infestans* | calpain-7 |
| D0WP73_9ACTO | *Actinomyces* sp. oral taxon 848 | HMPREF0972_01594 |
| D2V3V2_NAEGR | *Naegleria gruberi* | NAEGRDRAFT_63500 |
| D2VVS6_NAEGR | *Naegleria gruberi* | NAEGRDRAFT_52656 |
| D3CT47_9ACTO | *Frankia* sp. EUN1f | FrEUN1fDRAFT_0714 |
| D4YQ94_9MICO | *Brevibacterium mcbrellneri* | HMPREF0183_2104 |
| D5MF42_9BACT | *Candidatus Methylomirabilis oxyfera* | DAMO_1311 |
| D6B0I6_9ACTO | *Streptomyces albus* | SSHG_03621 |
| D6W573_HUMAN | *Homo sapiens* | calpain-14 |
| D6ZC85_SEGRD | *Segniliparus rotundus* | Srot_2619 |
| D7FQ17_ECTSI | *Ectocarpus siliculosus* | Esi_0002_0104 |
| D7IBA4_9BACE | *Bacteroides* sp. 1_1_14 | HMPREF9007_01542 |
| D8LB20_ECTSI | *Ectocarpus siliculosus* | Esi_0000_0184 |
| D9X719_STRVR | *Streptomyces viridochromogenes* | SSQG_04604 |
| E0UGX6_CYAP2 | *Cyanothece* sp. PCC 7822 | Cyan7822_2485 |
| E0UGY5_CYAP2 | *Cyanothece* sp. PCC 7822 | Cyan7822_2494 |
| E1BRR5_CHICK | *Gallus gallus* | calpain-13 |
| E1BTA8_CHICK | *Gallus gallus* | calpain-15 |
| E1BY84_CHICK | *Gallus gallus* | calpain-7 |
| E1C292_CHICK | *Gallus gallus* | calpain-5 |
| E1C312_CHICK | *Gallus gallus* | calpain-6 |
| E1C6M3_CHICK | *Gallus gallus* | calpain-9 |
| E3J3F6_FRASU | *Frankia* sp. EuI1c | FraEuI1c_0070 |
| E4ZY10_LEPMJ | *Leptosphaeria maculans* | LEMA_P111740.1 |
| E5C759_9BACE | *Bacteroides* sp. D2 | BacD2_08405 |
| E5XTT9_9ACTO | *Segniliparus rugosus* | HMPREF9336_02911 |
| E5XTU0_9ACTO | *Segniliparus rugosus* | HMPREF9336_02912 |
| E7F0H3_DANRE | *Brachydanio rerio* | si:ch211-202f3.4 |
| E7F7F1_DANRE | *Brachydanio rerio* | capn5b |
| E7FAX5_DANRE | *Brachydanio rerio* | solh |
| E7FDN3_DANRE | *Brachydanio rerio* | LOC798614 |
| E9QFN0_DANRE | *Brachydanio rerio* | si dkeyp-50d11.2 |
| ENSSARP00000008135 | *Sorex araneus* | calpain-7 |
| ENSSARP00000010553 | *Sorex araneus* |  |
| F1Q9Z1_DANRE | *Brachydanio rerio* | si:ch211-202f3.3 |
| F1R632_DANRE | *Brachydanio rerio* | capn3b |
| F4I0A4_ARATH | *Arabidopsis thaliana* | phytocalpain |
| F4NTM9_BATDJ | *Batrachochytrium dendrobatidis* | BATDEDRAFT_85291 |
| F6SGI6_CIOIN | *Ciona intestinalis* | LOC100181402 |
| F7ZZH9_CELGA | *Leucobacter chromiiresistens* | LchrJ3_00270 |
| F8W4K1_DANRE | *Brachydanio rerio* | capn10 |
| G0QS43_ICHMG | *Ichthyophthirius multifiliis* | IMG5_099180 |
| G4LXK3_SCHMA | *Schistosoma mansoni* | calpain-7 |
| G4TER6_PIRID | *Piriformospora indica* | PIIN_03746 |
| G4TER7_PIRID | *Piriformospora indica* | PIIN_03747 |
| G4TNA1_PIRID | *Piriformospora indica* | PIIN_06730 |
| G4V623_SCHMA | *Schistosoma mansoni* | calpain-14 |
| G4V6C0_SCHMA | *Schistosoma mansoni* | Smp_003980 |
| G4VAG2_SCHMA | *Schistosoma mansoni* | calpain |
| G4VAG2_SCHMA | *Schistosoma mansoni* | Smp_157500 |
| G5EEZ6_CAEEL | *Caenorhabditis elegans* | T11A5.6 |
| G6FUE0_9CYAN | *Fischerella* sp. JSC-11 | FJSC11DRAFT_2487 |
| H0S4P6_9BRAD | *Bradyrhizobium* sp. ORS 285 | BRAO285_570023 |
| H0SNL4_9BRAD | *Bradyrhizobium* sp. ORS 375 | BRAO375_4710021 |
| H0TAL7_9BRAD | *Bradyrhizobium* sp. STM 3809 | BRAS3809_7540004 |
| H2XR46_CIOIN | *Ciona intestinalis* | adgb |
| H2YVF0_CIOSA | *Ciona intestinalis* | Csa.4609 |
| H2ZIF1_CIOSA | *Ciona intestinalis* | LOC100182750 |
| H6L010_SAPGL | *Saprospira grandis* | SGRA_3289 |
| H9GX91_DANRE | *Brachydanio rerio* | capn7 |
| I1CS59_RHIO9 | *Rhizopus oryzae* | calpain-7 |
| I1EA30_AMPQE | *Amphimedon queenslandica* | LOC100636838 |
| I1FGV4_AMPQE | *Amphimedon queenslandica* | LOC100632502 |
| I1GER3_AMPQE | *Amphimedon queenslandica* | LOC100636231 |
| I6AT95_9BACT | *Gemmata obscuriglobus* | GobsU_010100014824 |
| J3QMP3_MOUSE | *Mus musculus* | androglobin |
| K1PCX8_CRAGI | *Crassostrea gigas* | CGI_10003593 |
| K1PHX3_CRAGI | *Crassostrea gigas* | CGI_10012113 |
| K1PJ80_CRAGI | *Crassostrea gigas* | CGI_10003594 |
| K1PV74_CRAGI | *Crassostrea gigas* | CGI_10006557 |
| K1PW75_CRAGI | *Crassostrea gigas* | CGI_10009508 |
| K1Q056_CRAGI | *Crassostrea gigas* | CGI_10026186 |
| K1Q505_CRAGI | *Crassostrea gigas* | CGI_10008277 |
| K1QU26_CRAGI | *Crassostrea gigas* | CGI_10004929 |
| K1R1C6_CRAGI | *Crassostrea gigas* | CGI_10008828 |
| K1R5E8_CRAGI | *Crassostrea gigas* | CGI_10024255 |
| K1RCZ2_CRAGI | *Crassostrea gigas* | CGI_10002395 |
| K1REZ8_CRAGI | *Crassostrea gigas* | CGI_10014855 |
| K1RSB8_CRAGI | *Crassostrea gigas* | CGI_10023863 |
| K1RWI3_CRAGI | *Crassostrea gigas* | CGI_10006438 |
| K1ZP83_9BACT | uncultured bacterium | ACD_62C00241G0006 |
| K7WKF4_9NOST | *Anabaena* sp. 90 | ANA_C13379 |
| K9PHG5_9CYAN | *Calothrix* sp. PCC 7507 | Cal7507_1508 |
| K9TN70_9CYAN | *Oscillatoria acuminata* | Oscil6304_4312 |
| K9UIG2_9CHRO | *Chamaesiphon minutus* | Cha6605_3446 |
| K9WJ87_9CYAN | *Microcoleus* sp. PCC 7113 | Mic7113_4571 |
| L1I9H5_GUITH | *Guillardia theta* | GUITHDRAFT_120920 |
| L1IGB6_GUITH | *Guillardia theta* | GUITHDRAFT_118521 |
| L1J0U9_GUITH | *Guillardia theta* | GUITHDRAFT_141718 |
| L1J1Q4_GUITH | *Guillardia theta* | GUITHDRAFT_164093 |
| L1J739_GUITH | *Guillardia theta* | GUITHDRAFT_163621 |
| L1JBM0_GUITH | *Guillardia theta* | GUITHDRAFT_139068 |
| L1JZN7_GUITH | *Guillardia theta* | calpain-7 |
| M4ZBJ4_9BRAD | *Agromonas oligotrophica* | S58_48570 |
| N9TPR9_ENTHI | *Entamoeba histolytica* | EHI_045290 |
| O02259_CAEEL | *Caenorhabditis elegans* | F44F1.1 |
| O02260_CAEEL | *Caenorhabditis elegans* | F44F1.3 |
| O42133_CHICK | *Gallus gallus* | calpain-1 |
| O44903_CAEEL | *Caenorhabditis elegans* | W05G11.4 |
| Q012Z6_OSTTA | *Ostreococcus tauri* | Ot08g02960 |
| Q05QW2_9SYNE | *Synechococcus* sp. RS9916 | RS9916_38921 |
| Q05QW3_9SYNE | *Synechococcus* sp. RS9916 | RS9916_38916 |
| Q05QW4_9SYNE | *Synechococcus* sp. RS9916 | RS9916_38911 |
| Q07SP7_RHOP5 | *Rhodopseudomonas palustris* | RPE_1084 |
| Q0BRV8_GRABC | *Granulibacter bethesdensis* | GbCGDNIH1_1546 |
| Q0RAZ2_FRAAA | *Frankia alni* | FRAAL6773 |
| Q1RL55_CIOIN | *Ciona intestinalis* | Ci-SOL |
| Q1RLS9_DANRE | *Brachydanio rerio* | zgc:136872 |
| Q22143_CAEEL | *Caenorhabditis elegans* | calpain-7 |
| Q2J4J6_FRASC | *Frankia* sp. CcI3 | Francci3_4450 |
| Q3MBX8_ANAVT | *Anabaena variabilis* | Ava_1886 |
| Q4CXJ8_TRYCC | *Trypanosoma cruzi* | Tc00.1047053510337.20 |
| Q4VBH3_DANRE | *Brachydanio rerio* | capn8 |
| Q504F5_DANRE | *Brachydanio rerio* | capn2b |
| Q5B5J7_EMENI | *Emericella nidulans* | ANIA_10513 |
| Q5BLH5_DANRE | *Brachydanio rerio* | capn2a |
| Q5IW12_GECLA | *Gecarcinus lateralis* | calpain B |
| Q5IW13_GECLA | *Gecarcinus lateralis* | calpain M |
| Q66HU4_DANRE | *Brachydanio rerio* | capn3a |
| Q6DFZ8_DANRE | *Brachydanio rerio* | zgc:112420 |
| Q6DG44_DANRE | *Brachydanio rerio* | capn9 |
| Q6F6K5_CAEEL | *Caenorhabditis elegans* | clec-119 |
| Q6NW69_DANRE | *Brachydanio rerio* | zgc:85932 |
| Q7N497_PHOLL | *Photorhabdus luminescens* | plu2455 |
| Q7NDU2_GLOVI | *Gloeobacter violaceus* | glr4140 |
| Q7SYA9_DANRE | *Brachydanio rerio* | capn1a |
| Q7ZUR1_DANRE | *Brachydanio rerio* | zgc:55262 |
| Q8IAA8_CAEEL | *Caenorhabditis elegans* | clp-4 |
| Q8RUQ1_MAIZE | *Zea mays* | phytocalpain |
| Q8YQK8_NOSS1 | *Nostoc* sp. PCC 7120 | alr3815 |
| Q8YXX3_NOSS1 | *Nostoc* sp. PCC 7120 | all1084 |
| Q8YZT8_NOSS1 | *Nostoc* sp. PCC 7120 | alr0369 |
| Q9N4B1_CAEEL | *Caenorhabditis elegans* | clp-7 |
| Q9N4B2_CAEEL | *Caenorhabditis elegans* | clp-6 |
| Q9U0T9_LEIMA | *Leishmania major* | LMJF_04_0450 |
| Q9U2B2_CAEEL | *Caenorhabditis elegans* | clp-7 |
| R6UXV3_9BACE | *Bacteroides faecis* | BN607_02600 |
| R7TJF2_CAPTE | *Capitella capitata* | CAPTEDRAFT_191770 |
| R7TWH9_CAPTE | *Capitella capitata* | CAPTEDRAFT_97820 |
| R7UAB6_CAPTE | *Capitella capitata* | CAPTEDRAFT_151195 |
| R7UBC6_CAPTE | *Capitella capitata* | CAPTEDRAFT_62348 |
| R7UD02_CAPTE | *Capitella capitata* | CAPTEDRAFT_182806 |
| R7ULX9_CAPTE | *Capitella capitata* | CAPTEDRAFT_167979 |
| R7UNL8_CAPTE | *Capitella capitata* | CAPTEDRAFT_90023 |
| R7UPU6_CAPTE | *Capitella capitata* | CAPTEDRAFT_218884 |
| R7UUX7_CAPTE | *Capitella capitata* | CAPTEDRAFT_98040 |
| R7V270_CAPTE | *Capitella capitata* | CAPTEDRAFT_220858 |
| R7V817_CAPTE | *Capitella capitata* | CAPTEDRAFT_53459 |
| R7VGC2_CAPTE | *Capitella capitata* | CAPTEDRAFT_155770 |
| R7VIB6_CAPTE | *Capitella capitata* | CAPTEDRAFT_174522 |
| R7VK39_CAPTE | *Capitella capitata* | CAPTEDRAFT_224123 |
| R7VLA7_CAPTE | *Capitella capitata* | CAPTEDRAFT_228346 |
| R9H254_BACT4 | *Bacteroides thetaiotaomicron* | BT_3960 |
| RIM13_YEAST | *Saccharomyces cerevisiae* | RIM13 peptidase |
| Smp_157500 | *Schistosoma mansoni* | Smp_159550 |
| T2M2K0_HYDVU | *Hydra magnipapillata* | LOC100215853 |
| T2MI17_HYDVU | *Hydra magnipapillata* | LOC100198393 |
| T2MIT4_HYDVU | *Hydra magnipapillata* | LOC100207933 |
| T2MJ45_HYDVU | *Hydra magnipapillata* | LOC100206735 |
| TPR_PORGI | *Porphyromonas gingivalis* | Tpr peptidase |
| W4Y195_STRPU | *Strongylocentrotus purpuratus* | Rim13 peptidase |
| W4YJN2_STRPU | *Strongylocentrotus purpuratus* | Sp-Capn5 |
| W4YMU4_STRPU | *Strongylocentrotus purpuratus* | calpain-15 |
| W4YV93_STRPU | *Strongylocentrotus purpuratus* | LOC100888469 |
| W4Z5Q7_STRPU | *Strongylocentrotus purpuratus* | Sp-Calpain |
| WP_043162213 | *Salinispora pacifica* |  |
| X1Y3H7_ANODA | *Anopheles darlingi* | AND_20430 |
| X1Y3H71_ANODA | *Anopheles darlingi* | AND_20430 |
| XP_002168617 | *Hydra magnipapillata* | LOC100211449 |
| XP_002735765 | *Saccoglossus kowalevskii* | LOC100374474 |
| XP_004943443 | *Gallus gallus* | calpain-10 |
| XP_006811804 | *Saccoglossus kowalevskii* | LOC102800862 |
| XP_006824412 | *Saccoglossus kowalevskii* | LOC100368095 |
| XP_426117 | *Gallus gallus* | calpain-8 |
| Z9JR51_9MICO | *Brachybacterium paraconglomeratum* | BparL_010100003073 |
| ZP_09168240 | *Frankia* sp. CN3 | FrCN3DRAFT_2911 |
| ZP_10094989 | *Brachybacterium paraconglomeratum* | BparL_010100002468 |
| ZP_11152369 | *Diplorickettsia massiliensis* | Dmas2_04705 |
