# Supplementary material for: Bacterial calpains and the evolution of the calpain (C2) family of peptidases
Source: Biol Direct. 2015 Nov 2;10:66. doi: 10.1186/s13062-015-0095-0 (PMC4631099; doi:10.1186/s13062-015-0095-0)
Supplement: Additional file 4: Table S2. — Key to sequences. The names of the 267 proteins, the species they are derived from, and the identifier used in Fig. 1 and Additional file 2: Figure S2 are given. The exponent from the expect value from the HMMER search is also shown (“E-value”). (DOCX 199 kb) [file 13062_2015_95_MOESM4_ESM.docx]

| **Identifier/residues** | **E-value** | **Species** |
| --- | --- | --- |
| A0A0D3F9J8_9ORYZ/1664-1908 | 3.4e-78 | Oryza barthii |
| A0A0D3F9J8_9ORYZ/3660-3904 | 3.4e-78 | Oryza barthii |
| A0A0D3F9J7_9ORYZ/1664-1908 | 3.4e-78 | Oryza barthii |
| A0A0D3F9J7_9ORYZ/3674-3918 | 3.4e-78 | Oryza barthii |
| G4VAG2_SCHMA/152-405 | 1.4e-71 | Schistosoma mansoni |
| G4VAG2_SCHMA/879-1134 | 1.7e-82 | Schistosoma mansoni |
| A0A094ZH52_SCHHA/760-990 | 4.2e-65 | Schistosoma haematobium |
| A0A094ZH52_SCHHA/1375-1630 | 7.1e-82 | Schistosoma haematobium |
| W5JJM5_ANODA/94-338 | 2.4e-50 | Anopheles darlingi |
| W5JJM5_ANODA/881-1143 | 1.1e-74 | Anopheles darlingi |
| L1J739_GUITH/73-323 | 2.5e-64 | Guillardia theta CCMP2712 |
| L1J739_GUITH/656-903 | 3.6e-50 | Guillardia theta CCMP2712 |
| A0A0D2XSI3_FUSO4/153-428 | 5.7e-54 | Fusarium oxysporum f. sp. lycopersici |
| A0A0D2XSI3_FUSO4/594-869 | 5.7e-54 | Fusarium oxysporum f. sp. lycopersici |
| N1RQW9_FUSC4/146-374 | 2.3e-43 | Fusarium oxysporum f. sp. cubense |
| N1RQW9_FUSC4/392-665 | 1.4e-60 | Fusarium oxysporum f. sp. cubense |
| C3Y6N1_BRAFL/8-80 | 1.6e-21 | Branchiostoma floridae |
| C3Y6N1_BRAFL/476-709 | 7.2e-70 | Branchiostoma floridae |
| Q4SYK3_TETNG/75-270 | 1.7e-52 | Tetraodon nigroviridis |
| Q4SYK3_TETNG/571-708 | 7.2e-33 | Tetraodon nigroviridis |
| A0A096N342_PAPAN/51-325 | 8.4e-90 | Papio anubis |
| H9EUZ1_MACMU/51-325 | 8.4e-90 | Macaca mulatta |
| A0A0D9QV50_CHLSB/51-325 | 8.5e-90 | Chlorocebus sabaeus |
| G1S4D9_NOMLE/51-325 | 8.4e-90 | Nomascus leucogenys |
| K6ZPM2_PANTR/51-325 | 8.7e-90 | Pan troglodytes |
| G3QYF9_GORGO/91-365 | 1.0e-89 | Gorilla gorilla gorilla |
| F1SUK6_PIG/84-358 | 1.1e-89 | Sus scrofa |
| W4Z5Q7_STRPU/51-300 | 1.4e-89 | Strongylocentrotus purpuratus |
| A0A093GSY6_PICPB/50-324 | 1.9e-89 | Picoides pubescens |
| F7HJT8_CALJA/51-325 | 2.1e-89 | Callithrix jacchus |
| U3DU82_CALJA/51-325 | 2.1e-89 | Callithrix jacchus |
| F7DS40_CALJA/91-365 | 2.4e-89 | Callithrix jacchus |
| A0A093IC27_EURHL/50-324 | 2.5e-89 | Eurypyga helias |
| A0A0A0A9T5_CHAVO/50-324 | 3.1e-89 | Charadrius vociferus |
| U3JYN9_FICAL/78-352 | 3.0e-89 | Ficedula albicollis |
| A0A091PBH6_HALAL/50-324 | 3.5e-89 | Haliaeetus albicilla |
| I3MW90_SPETR/51-325 | 3.6e-89 | Spermophilus tridecemlineatus |
| A0A093BQW3_CHAPE/50-324 | 3.2e-89 | Chaetura pelagica |
| A0A091MNV5_CARIC/50-324 | 3.4e-89 | Cariama cristata |
| CAN5_HUMAN/51-325 | 3.1e-89 | Homo sapiens |
| A0A091SWT8_9AVES/50-324 | 3.5e-89 | Pelecanus crispus |
| A0A093NLP2_PYGAD/50-324 | 3.5e-89 | Pygoscelis adeliae |
| A0A087RFB4_APTFO/50-324 | 3.5e-89 | Aptenodytes forsteri |
| A0A091GGL9_9AVES/50-324 | 3.5e-89 | Cuculus canorus |
| Q59GM2_HUMAN/89-363 | 3.5e-89 | Homo sapiens |
| A0A091URJ9_NIPNI/50-324 | 3.8e-89 | Nipponia nippon |
| G3V7U6_RAT/51-325 | 4.1e-89 | Rattus norvegicus |
| G1NQT4_MELGA/50-324 | 4.0e-89 | Meleagris gallopavo |
| G3SPW7_LOXAF/51-325 | 4.0e-89 | Loxodonta africana |
| H0XGA0_OTOGA/89-363 | 4.6e-89 | Otolemur garnettii |
| A0A091J420_9AVES/50-324 | 4.4e-89 | Egretta garzetta |
| A0A093Q5U3_9PASS/50-324 | 5.0e-89 | Manacus vitellinus |
| Q3TDS7_MOUSE/51-325 | 5.5e-89 | Mus musculus |
| Q3U2P3_MOUSE/51-325 | 5.5e-89 | Mus musculus |
| CAN5_MOUSE/51-325 | 5.5e-89 | Mus musculus |
| G3I7G1_CRIGR/54-328 | 5.6e-89 | Cricetulus griseus |
| R0JLR5_ANAPL/50-324 | 5.6e-89 | Anas platyrhynchos |
| A0A091E4X5_FUKDA/167-441 | 6.4e-89 | Fukomys damarensis |
| U3INP6_ANAPL/86-360 | 6.3e-89 | Anas platyrhynchos |
| A0A091MY21_APAVI/50-325 | 6.8e-89 | Apaloderma vittatum |
| G1U496_RABIT/174-448 | 9.9e-89 | Oryctolagus cuniculus |
| A0A093JDN0_FULGA/50-324 | 1.2e-88 | Fulmarus glacialis |
| F1MQD1_BOVIN/51-325 | 1.1e-88 | Bos taurus |
| L8IUH0_9CETA/57-331 | 1.1e-88 | Bos mutus |
| A0A091I089_CALAN/50-324 | 1.1e-88 | Calypte anna |
| L7N319_XENTR/51-325 | 1.1e-88 | Xenopus tropicalis |
| Q6DIP0_XENTR/51-325 | 1.2e-88 | Xenopus tropicalis |
| A0A091H9A0_BUCRH/50-324 | 1.3e-88 | Buceros rhinoceros silvestris |
| A0A091RIN3_9GRUI/50-325 | 1.4e-88 | Mesitornis unicolor |
| K7FS80_PELSI/40-314 | 1.7e-88 | Pelodiscus sinensis |
| E7F7F1_DANRE/51-325 | 1.6e-88 | Danio rerio |
| E1C292_CHICK/50-324 | 1.9e-88 | Gallus gallus |
| M3WKQ6_FELCA/51-325 | 2.4e-88 | Felis catus |
| A0A091VQP9_OPIHO/50-324 | 2.3e-88 | Opisthocomus hoazin |
| M3YAX7_MUSPF/51-325 | 2.1e-88 | Mustela putorius furo |
| G5AW79_HETGA/60-334 | 1.6e-88 | Heterocephalus glaber |
| H2MJC7_ORYLA/59-333 | 2.2e-88 | Oryzias latipes |
| I3JLQ6_ORENI/48-322 | 2.3e-88 | Oreochromis niloticus |
| D2I3E3_AILME/56-330 | 2.8e-88 | Ailuropoda melanoleuca |
| Q6ZRM8_HUMAN/91-365 | 2.6e-88 | Homo sapiens |
| F1PB20_CANFA/51-325 | 2.8e-88 | Canis familiaris |
| G1LRF4_AILME/93-367 | 3.2e-88 | Ailuropoda melanoleuca |
| L5LVX4_MYODS/51-325 | 2.2e-88 | Myotis davidii |
| I3JT03_ORENI/51-325 | 3.2e-88 | Oreochromis niloticus |
| A0A087YGB5_POEFO/51-325 | 3.9e-88 | Poecilia formosa |
| M4A9S0_XIPMA/51-325 | 3.9e-88 | Xiphophorus maculatus |
| A0A093BZQ1_TAUER/50-324 | 3.5e-88 | Tauraco erythrolophus |
| F6VDH6_HORSE/51-325 | 5.8e-88 | Equus caballus |
| F6V5B8_HORSE/57-331 | 5.9e-88 | Equus caballus |
| H2TPV0_TAKRU/70-344 | 5.4e-88 | Takifugu rubripes |
| Q6NU10_XENLA/51-325 | 8.1e-88 | Xenopus laevis |
| B3KSR7_HUMAN/93-365 | 7.6e-88 | Homo sapiens |
| H2TPU9_TAKRU/52-326 | 8.6e-88 | Takifugu rubripes |
| J3RYM5_CROAD/50-324 | 1.2e-87 | Crotalus adamanteus |
| H3CDI8_TETNG/52-326 | 1.1e-87 | Tetraodon nigroviridis |
| E7EV01_HUMAN/93-365 | 1.2e-87 | Homo sapiens |
| L5JLY9_PTEAL/368-642 | 1.0e-87 | Pteropus alecto |
| B4HPG1_DROSE/128-384 | 1.5e-87 | Drosophila sechellia |
| CANA_DROME/113-369 | 1.7e-87 | Drosophila melanogaster |
| H3CKJ2_TETNG/49-321 | 1.6e-87 | Tetraodon nigroviridis |
| B3NJU8_DROER/128-384 | 1.8e-87 | Drosophila erecta |
| A0A0B4LG26_DROME/128-384 | 1.8e-87 | Drosophila melanogaster |
| B4QE26_DROSI/128-384 | 1.8e-87 | Drosophila simulans |
| B4PB86_DROYA/113-369 | 2.0e-87 | Drosophila yakuba |
| A0A087VKF2_BALRE/1-272 | 2.0e-87 | Balearica regulorum gibbericeps |
| A0A091KHI8_9GRUI/2-272 | 1.9e-87 | Chlamydotis macqueenii |
| H0ZS50_TAEGU/2-272 | 2.4e-87 | Taeniopygia guttata |
| Q7ZWK6_XENLA/51-325 | 2.4e-87 | Xenopus laevis |
| A0A093FBX5_TYTAL/1-272 | 2.5e-87 | Tyto alba |
| A0A094KLD4_9AVES/2-272 | 2.7e-87 | Podiceps cristatus |
| A0A093CR08_9AVES/2-272 | 3.0e-87 | Pterocles gutturalis |
| W5K6T7_ASTMX/51-324 | 3.4e-87 | Astyanax mexicanus |
| A0A099ZX59_TINGU/2-271 | 3.3e-87 | Tinamus guttatus |
| A0A094LGG5_ANTCR/2-272 | 4.2e-87 | Antrostomus carolinensis |
| A0A093HZF1_STRCA/2-272 | 3.9e-87 | Struthio camelus australis |
| G3W892_SARHA/52-326 | 4.8e-87 | Sarcophilus harrisii |
| B3MGG4_DROAN/113-369 | 5.1e-87 | Drosophila ananassae |
| A0A091UI08_PHORB/2-272 | 5.5e-87 | Phoenicopterus ruber ruber |
| A0A069DWP5_9HEMI/96-353 | 7.0e-87 | Panstrongylus megistus |
| H2SMY0_TAKRU/51-323 | 7.3e-87 | Takifugu rubripes |
| W5K4R9_ASTMX/80-335 | 8.8e-87 | Astyanax mexicanus |
| W5MI53_LEPOC/51-325 | 1.1e-86 | Lepisosteus oculatus |
| W5MI74_LEPOC/59-333 | 1.1e-86 | Lepisosteus oculatus |
| G3PIY2_GASAC/48-322 | 1.3e-86 | Gasterosteus aculeatus |
| G3QC69_GASAC/52-326 | 1.6e-86 | Gasterosteus aculeatus |
| V9KM33_CALMI/51-325 | 1.7e-86 | Callorhinchus milii |
| I3ISA7_DANRE/51-325 | 1.8e-86 | Danio rerio |
| A0A023F577_TRIIF/96-353 | 1.9e-86 | Triatoma infestans |
| H2SX02_TAKRU/44-296 | 2.4e-86 | Takifugu rubripes |
| H2SX01_TAKRU/53-305 | 2.5e-86 | Takifugu rubripes |
| K7FSA4_PELSI/1-270 | 2.5e-86 | Pelodiscus sinensis |
| H2SWZ8_TAKRU/80-332 | 2.9e-86 | Takifugu rubripes |
| F1Q7N2_DANRE/51-325 | 2.8e-86 | Danio rerio |
| K9J3G6_DESRO/67-341 | 2.3e-86 | Desmodus rotundus |
| H2L9Q3_ORYLA/51-325 | 4.0e-86 | Oryzias latipes |
| F6YNP0_MONDO/121-395 | 4.1e-86 | Monodelphis domestica |
| B4KME2_DROMO/113-369 | 4.9e-86 | Drosophila mojavensis |
| B3RJS5_TRIAD/54-307 | 7.6e-86 | Trichoplax adhaerens |
| A0A087Y317_POEFO/48-322 | 5.0e-86 | Poecilia formosa |
| A0A096LQS5_POEFO/68-342 | 5.4e-86 | Poecilia formosa |
| H2SWZ9_TAKRU/72-325 | 6.6e-86 | Takifugu rubripes |
| B4LNX1_DROVI/113-369 | 7.3e-86 | Drosophila virilis |
| B4J629_DROGR/113-369 | 7.5e-86 | Drosophila grimshawi |
| M3ZXS6_XIPMA/58-332 | 7.8e-86 | Xiphophorus maculatus |
| F7DRZ9_CALJA/51-324 | 1.0e-85 | Callithrix jacchus |
| T1PG94_MUSDO/114-370 | 1.1e-85 | Musca domestica |
| H3CYV6_TETNG/80-335 | 1.5e-85 | Tetraodon nigroviridis |
| A0A087X0C8_HUMAN/51-324 | 1.4e-85 | Homo sapiens |
| A0A084WJ00_9DIPT/314-571 | 2.0e-85 | Anopheles sinensis |
| Q66HU4_DANRE/80-335 | 2.5e-85 | Danio rerio |
| B4GCQ6_DROPE/113-369 | 2.5e-85 | Drosophila persimilis |
| A0A026WP62_CERBI/115-371 | 2.7e-85 | Cerapachys biroi |
| CAN5_RAT/51-325 | 3.7e-85 | Rattus norvegicus |
| B5E1I7_DROPS/113-369 | 5.2e-85 | Drosophila pseudoobscura pseudoobscura |
| A0JMG3_DANRE/51-325 | 5.3e-85 | Danio rerio |
| B4MRQ4_DROWI/113-369 | 6.5e-85 | Drosophila willistoni |
| C3YJZ3_BRAFL/53-330 | 8.2e-85 | Branchiostoma floridae |
| A0A091NE86_9PASS/50-326 | 8.5e-85 | Acanthisitta chloris |
| V5V0P1_CYPCA/80-335 | 1.1e-84 | Cyprinus carpio |
| F7A288_MONDO/51-325 | 6.5e-85 | Monodelphis domestica |
| A0A0A1XNE6_BACCU/113-369 | 9.4e-85 | Bactrocera cucurbitae |
| A0NGM4_ANOGA/176-433 | 1.3e-84 | Anopheles gambiae |
| H2V187_TAKRU/51-324 | 2.5e-84 | Takifugu rubripes |
| H3CXF9_TETNG/52-325 | 2.2e-84 | Tetraodon nigroviridis |
| Q4SFI9_TETNG/51-324 | 2.3e-84 | Tetraodon nigroviridis |
| J9K494_ACYPI/98-354 | 2.9e-84 | Acyrthosiphon pisum |
| Q8IAA8_CAEEL/265-518 | 2.9e-84 | Caenorhabditis elegans |
| G3VME1_SARHA/52-326 | 3.2e-84 | Sarcophilus harrisii |
| W5JKC2_ANODA/134-391 | 3.5e-84 | Anopheles darlingi |
| F4WIE1_ACREC/56-312 | 3.4e-84 | Acromyrmex echinatior |
| D8LB20_ECTSI/97-378 | 3.1e-84 | Ectocarpus siliculosus |
| A0A0A9Y6U0_LYGHE/95-351 | 3.2e-84 | Lygus hesperus |
| Q8IAA9_CAEEL/265-518 | 4.2e-84 | Caenorhabditis elegans |
| W5N8X1_LEPOC/51-325 | 5.5e-84 | Lepisosteus oculatus |
| U4U1X6_DENPD/361-617 | 5.5e-84 | Dendroctonus ponderosae |
| N6TX50_DENPD/361-617 | 5.9e-84 | Dendroctonus ponderosae |
| A0A087ZX44_APIME/90-346 | 7.1e-84 | Apis mellifera |
| A0A034V5G3_BACDO/113-369 | 6.4e-84 | Bactrocera dorsalis |
| B4L097_DROMO/279-535 | 6.5e-84 | Drosophila mojavensis |
| A0A034V4U7_BACDO/113-369 | 6.8e-84 | Bactrocera dorsalis |
| F7EDU0_MACMU/51-316 | 7.9e-84 | Macaca mulatta |
| E2B6V2_HARSA/55-311 | 7.4e-84 | Harpegnathos saltator |
| F7EDT3_MACMU/90-355 | 9.0e-84 | Macaca mulatta |
| V9I6M4_APICE/81-337 | 9.0e-84 | Apis cerana |
| V9I6P4_APICE/81-337 | 9.4e-84 | Apis cerana |
| V9I6P7_APICE/90-346 | 9.2e-84 | Apis cerana |
| V9I8N5_APICE/90-346 | 9.6e-84 | Apis cerana |
| E2ADL6_CAMFO/79-335 | 9.6e-84 | Camponotus floridanus |
| G3NNM2_GASAC/56-311 | 1.1e-83 | Gasterosteus aculeatus |
| G3NNN5_GASAC/80-335 | 1.2e-83 | Gasterosteus aculeatus |
| Q17E34_AEDAE/155-412 | 1.1e-83 | Aedes aegypti |
| A0A023EVZ7_AEDAL/116-373 | 1.2e-83 | Aedes albopictus |
| H9IS71_BOMMO/246-502 | 1.2e-83 | Bombyx mori |
| E9IT45_SOLIN/122-378 | 1.3e-83 | Solenopsis invicta |
| A0A087XLW4_POEFO/80-335 | 1.6e-83 | Poecilia formosa |
| A0A034V3R5_BACDO/113-369 | 1.5e-83 | Bactrocera dorsalis |
| W5PV56_SHEEP/69-334 | 1.7e-83 | Ovis aries |
| A0A023EXW9_AEDAL/116-373 | 1.9e-83 | Aedes albopictus |
| G0NAB1_CAEBE/329-582 | 2.2e-83 | Caenorhabditis brenneri |
| H2ZV34_LATCH/56-330 | 2.3e-83 | Latimeria chalumnae |
| B0WSM3_CULQU/155-412 | 2.4e-83 | Culex quinquefasciatus |
| T2MJ45_HYDVU/88-336 | 2.7e-83 | Hydra vulgaris |
| A8PB44_BRUMA/243-496 | 2.7e-83 | Brugia malayi |
| G0NBQ1_CAEBE/278-531 | 3.3e-83 | Caenorhabditis brenneri |
| A0A060WQD0_ONCMY/87-375 | 5.7e-83 | Oncorhynchus mykiss |
| Q9N4B1_CAEEL/335-588 | 4.3e-83 | Caenorhabditis elegans |
| A8NMH2_BRUMA/431-684 | 4.3e-83 | Brugia malayi |
| A8PB45_BRUMA/431-684 | 4.3e-83 | Brugia malayi |
| T1FNH3_HELRO/15-272 | 4.6e-83 | Helobdella robusta |
| CANB_DROME/284-540 | 4.8e-83 | Drosophila melanogaster |
| CAN_SCHMA/124-379 | 5.2e-83 | Schistosoma mansoni |
| G3QBW1_GASAC/51-324 | 7.4e-83 | Gasterosteus aculeatus |
| H2VS51_CAEJA/265-518 | 5.9e-83 | Caenorhabditis japonica |
| B4MLX9_DROWI/278-534 | 5.9e-83 | Drosophila willistoni |
| B4PDY0_DROYA/283-539 | 6.6e-83 | Drosophila yakuba |
| B3NGN5_DROER/285-541 | 6.6e-83 | Drosophila erecta |
| B4QNQ9_DROSI/284-540 | 6.6e-83 | Drosophila simulans |
| B4LGL7_DROVI/277-533 | 7.0e-83 | Drosophila virilis |
| I3IV71_ORENI/3-254 | 7.5e-83 | Oreochromis niloticus |
| M4AE78_XIPMA/50-319 | 7.9e-83 | Xiphophorus maculatus |
| H2SX00_TAKRU/56-226 | 3.1e-53 | Takifugu rubripes |
| H2SX00_TAKRU/255-331 | 8.4e-24 | Takifugu rubripes |
| E0W233_PEDHC/66-316 | 8.0e-83 | Pediculus humanus subsp. corporis |
| H2SWZ7_TAKRU/80-250 | 3.2e-53 | Takifugu rubripes |
| H2SWZ7_TAKRU/279-355 | 8.5e-24 | Takifugu rubripes |
| Q9N4B2_CAEEL/182-434 | 1.1e-82 | Caenorhabditis elegans |
| Q5IW12_GECLA/118-374 | 9.3e-83 | Gecarcinus lateralis |
| H3CLD3_TETNG/65-317 | 1.1e-82 | Tetraodon nigroviridis |
| K7IZ40_NASVI/103-359 | 9.5e-83 | Nasonia vitripennis |
| H2S159_TAKRU/41-293 | 2.0e-82 | Takifugu rubripes |
| B3M575_DROAN/288-544 | 1.0e-82 | Drosophila ananassae |
| I1GIY1_AMPQE/48-323 | 1.1e-82 | Amphimedon queenslandica |
| A0A093HVA8_STRCA/51-325 | 1.4e-82 | Struthio camelus australis |
| B4HLR2_DROSE/284-540 | 1.1e-82 | Drosophila sechellia |
| Q4T7F6_TETNG/51-104 | 3.1e-10 | Tetraodon nigroviridis |
| Q4T7F6_TETNG/255-473 | 7.7e-67 | Tetraodon nigroviridis |
| A0A044UQX4_ONCVO/248-501 | 1.3e-82 | Onchocerca volvulus |
| A5A6F7_SCHHA/124-379 | 1.4e-82 | Schistosoma haematobium |
| F7BAV6_ORNAN/51-325 | 1.4e-82 | Ornithorhynchus anatinus |
| A0A085LV26_9BILA/394-647 | 1.5e-82 | Trichuris suis |
| M4ANX3_XIPMA/65-317 | 1.8e-82 | Xiphophorus maculatus |
| A0A087Y3J5_POEFO/52-324 | 2.3e-82 | Poecilia formosa |
| H2S160_TAKRU/41-293 | 2.1e-82 | Takifugu rubripes |
| H2S153_TAKRU/41-293 | 2.0e-82 | Takifugu rubripes |
| H2S152_TAKRU/42-294 | 2.1e-82 | Takifugu rubripes |
| H2S157_TAKRU/66-318 | 2.2e-82 | Takifugu rubripes |
| H2S155_TAKRU/65-317 | 2.2e-82 | Takifugu rubripes |
| H2S161_TAKRU/72-324 | 2.1e-82 | Takifugu rubripes |
| H2S154_TAKRU/77-329 | 2.3e-82 | Takifugu rubripes |
| A0A0D8XXK2_DICVI/337-590 | 2.2e-82 | Dictyocaulus viviparus |
| A8XJ10_CAEBR/334-587 | 2.7e-82 | Caenorhabditis briggsae |
| A0A094L2F6_ANTCR/51-325 | 2.1e-82 | Antrostomus carolinensis |
| J9F664_WUCBA/214-467 | 2.9e-82 | Wuchereria bancrofti |
| A0A093P089_PYGAD/51-325 | 2.2e-82 | Pygoscelis adeliae |
| S6CVW8_9VIRU/105-361 | 3.1e-82 | Cotesia congregata bracovirus |
| B4J2T4_DROGR/295-551 | 3.4e-82 | Drosophila grimshawi |
| E1C312_CHICK/51-325 | 3.9e-82 | Gallus gallus |
| K1Q056_CRAGI/225-481 | 4.0e-82 | Crassostrea gigas |
| R7ULX9_CAPTE/89-346 | 3.8e-82 | Capitella teleta |
| V5G6A1_ANOGL/91-347 | 3.7e-82 | Anoplophora glabripennis |
| A0A091T4S3_9AVES/51-325 | 2.9e-82 | Pelecanus crispus |
| A0A0D6LXK7_9BILA/301-554 | 4.6e-82 | Ancylostoma ceylanicum |
| B3RIE5_TRIAD/54-326 | 1.3e-81 | Trichoplax adhaerens |
| I3ITW3_ORENI/54-306 | 4.9e-82 | Oreochromis niloticus |
| B4H3H2_DROPE/284-540 | 4.4e-82 | Drosophila persimilis |
| Q2LZQ6_DROPS/290-546 | 4.4e-82 | Drosophila pseudoobscura pseudoobscura |
| A0A087YNH1_POEFO/51-323 | 5.6e-82 | Poecilia formosa |
| E9G628_DAPPU/75-331 | 5.0e-82 | Daphnia pulex |
| A0A016UMK2_9BILA/288-541 | 5.3e-82 | Ancylostoma ceylanicum |
| F1MYC8_BOVIN/79-331 | 6.0e-82 | Bos taurus |
| A8XLV6_CAEBR/266-519 | 6.3e-82 | Caenorhabditis briggsae |
| A0A016UMS3_9BILA/375-628 | 6.6e-82 | Ancylostoma ceylanicum |
| D6WGT1_TRICA/317-573 | 8.2e-82 | Tribolium castaneum |
| U6P005_HAECO/307-560 | 8.6e-82 | Haemonchus contortus |
| B5AFM5_SHEEP/79-331 | 8.5e-82 | Ovis aries |
| R0LVR4_ANAPL/51-325 | 1.1e-81 | Anas platyrhynchos |
| A0A034WDZ3_BACDO/276-532 | 8.7e-82 | Bactrocera dorsalis |
| W4YJN2_STRPU/54-325 | 8.4e-82 | Strongylocentrotus purpuratus |
| K7G0J2_PELSI/51-325 | 7.4e-82 | Pelodiscus sinensis |
| A0A099YYZ4_TINGU/51-325 | 1.3e-81 | Tinamus guttatus |
| H2MV83_ORYLA/80-250 | 5.6e-53 | Oryzias latipes |
| H2MV83_ORYLA/274-378 | 3.1e-23 | Oryzias latipes |
| Q9XSJ2_PIG/79-331 | 1.4e-81 | Sus scrofa |
| V3ZFZ8_LOTGI/88-344 | 1.3e-81 | Lottia gigantea |
| F1SI20_PIG/79-331 | 1.4e-81 | Sus scrofa |
| H0Z763_TAEGU/51-324 | 1.3e-81 | Taeniopygia guttata |
| Q0VGP9_MOUSE/79-331 | 1.7e-81 | Mus musculus |
| O08702_RAT/79-331 | 1.7e-81 | Rattus norvegicus |
| F8W8F5_HUMAN/99-351 | 1.8e-81 | Homo sapiens |
| B3RNL8_TRIAD/53-306 | 1.6e-81 | Trichoplax adhaerens |
| A2AVV5_MOUSE/79-331 | 1.8e-81 | Mus musculus |
| O70376_RAT/79-331 | 1.8e-81 | Rattus norvegicus |
| A0A0E2M4Q0_PORGN/194-463 | 1.8e-81 | Porphyromonas gingivalis F0570 |
| W1R5Y7_PORGN/194-463 | 1.8e-81 | Porphyromonas gingivalis SJD2 |
| U2JLM7_PORGN/194-463 | 1.8e-81 | Porphyromonas gingivalis F0566 |
| TPR_PORGI/194-463 | 1.8e-81 | Porphyromonas gingivalis |
| G1N408_MELGA/51-325 | 1.9e-81 | Meleagris gallopavo |
| A0A0A1XHT6_BACCU/277-533 | 1.9e-81 | Bactrocera cucurbitae |
| M0ZYS9_SOLTU/57-303 | 1.8e-81 | Solanum tuberosum |
| F1RCJ4_DANRE/65-317 | 1.9e-81 | Danio rerio |
| A0A091T746_PHALP/51-325 | 1.5e-81 | Phaethon lepturus |
| A0A0B1PM85_9BILA/346-598 | 2.0e-81 | Trichuris suis |
| O96071_SCHJA/124-379 | 2.0e-81 | Schistosoma japonicum |
| R7UAB6_CAPTE/133-389 | 2.1e-81 | Capitella teleta |
| H2LRZ3_ORYLA/53-326 | 2.1e-81 | Oryzias latipes |
| I3IV72_ORENI/80-250 | 6.4e-53 | Oreochromis niloticus |
| I3IV72_ORENI/298-374 | 1.5e-22 | Oreochromis niloticus |
| H2YVE4_CIOSA/5-253 | 2.2e-81 | Ciona savignyi |
| A0A060XR55_ONCMY/51-324 | 1.6e-81 | Oncorhynchus mykiss |
| A0A085NIS3_9BILA/394-646 | 2.2e-81 | Trichuris suis |
| H2YVF0_CIOSA/36-284 | 2.4e-81 | Ciona savignyi |
| I3JCJ7_ORENI/51-324 | 2.2e-81 | Oreochromis niloticus |
| Q6DG44_DANRE/65-317 | 2.6e-81 | Danio rerio |
| Q9XSJ3_RABIT/79-331 | 2.8e-81 | Oryctolagus cuniculus |
| G3Q8B2_GASAC/53-304 | 2.7e-81 | Gasterosteus aculeatus |
| G3Q8A9_GASAC/67-318 | 2.8e-81 | Gasterosteus aculeatus |
| A0A087QI31_APTFO/51-325 | 6.8e-81 | Aptenodytes forsteri |
| I3JCJ6_ORENI/53-326 | 3.2e-81 | Oreochromis niloticus |
| F7G8T9_MACMU/99-351 | 4.3e-81 | Macaca mulatta |
| H2SWZ6_TAKRU/14-182 | 6.6e-52 | Takifugu rubripes |
| H2SWZ6_TAKRU/211-288 | 2.0e-23 | Takifugu rubripes |
| A0A091JZ84_COLST/51-325 | 4.7e-81 | Colius striatus |
| A0A093J9P8_EURHL/51-325 | 5.4e-81 | Eurypyga helias |
| O45033_SCHJA/126-381 | 4.6e-81 | Schistosoma japonicum |
| W5N7I7_LEPOC/90-260 | 6.0e-53 | Lepisosteus oculatus |
| W5N7I7_LEPOC/312-392 | 3.9e-22 | Lepisosteus oculatus |
| A0A091UIU1_NIPNI/51-325 | 7.1e-81 | Nipponia nippon |
| A0A091FLQ6_9AVES/51-325 | 6.5e-81 | Cuculus canorus |
| H2VHD7_CAEJA/332-586 | 5.9e-81 | Caenorhabditis japonica |
| A0A091ITC1_9AVES/51-325 | 6.9e-81 | Egretta garzetta |
| A0A093F3Z7_GAVST/51-325 | 7.2e-81 | Gavia stellata |
| A0A093IXQ5_FULGA/51-325 | 4.6e-81 | Fulmarus glacialis |
| A0A0B2W2E3_TOXCA/125-377 | 7.1e-81 | Toxocara canis |
| A0A093QPZ1_PHACA/51-325 | 4.7e-81 | Phalacrocorax carbo |
| M4A4P3_XIPMA/80-250 | 1.4e-52 | Xiphophorus maculatus |
| M4A4P3_XIPMA/294-369 | 1.6e-22 | Xiphophorus maculatus |
| W5N7J4_LEPOC/90-260 | 5.6e-53 | Lepisosteus oculatus |
| W5N7J4_LEPOC/305-384 | 6.4e-22 | Lepisosteus oculatus |
| G7YU18_CLOSI/122-376 | 7.5e-81 | Clonorchis sinensis |
| O88977_MOUSE/79-331 | 7.8e-81 | Mus musculus |
| G1KA77_ANOCA/67-319 | 8.2e-81 | Anolis carolinensis |
| A0A068VCY9_COFCA/1780-2026 | 9.5e-81 | Coffea canephora |
| A0A0D8Y8I5_DICVI/233-485 | 8.1e-81 | Dictyocaulus viviparus |
| O96072_SCHJA/124-379 | 8.1e-81 | Schistosoma japonicum |
| A0A0A0AQB8_CHAVO/51-325 | 6.6e-81 | Charadrius vociferus |
| H2MZK5_ORYLA/49-301 | 1.0e-80 | Oryzias latipes |
| V9K9G3_CALMI/68-320 | 1.2e-80 | Callorhinchus milii |
| A0A091ND73_APAVI/51-325 | 1.2e-80 | Apaloderma vittatum |
| Q9XSJ1_BOVIN/79-331 | 1.1e-80 | Bos taurus |
| A0A093BWM9_9AVES/51-325 | 7.8e-81 | Pterocles gutturalis |
| A0A093PVV3_9PASS/51-325 | 1.4e-80 | Manacus vitellinus |
| G3VZS2_SARHA/99-351 | 1.2e-80 | Sarcophilus harrisii |
| A0A093IYL1_PICPB/67-319 | 1.4e-80 | Picoides pubescens |
| A0A091VNJ0_OPIHO/51-325 | 1.3e-80 | Opisthocomus hoazin |
| F1L021_ASCSU/266-518 | 1.3e-80 | Ascaris suum |
| T1E1P9_CUPSA/69-325 | 2.0e-80 | Cupiennius salei |
| A0A096LQ44_POEFO/51-317 | 1.1e-80 | Poecilia formosa |
| T1JCQ1_STRMM/81-340 | 1.6e-80 | Strigamia maritima |
| G4VDT5_SCHMA/146-403 | 1.6e-80 | Schistosoma mansoni |
| Q4SDQ1_TETNG/80-345 | 2.0e-80 | Tetraodon nigroviridis |
| G4VDT6_SCHMA/146-403 | 1.6e-80 | Schistosoma mansoni |
| A0A093Q2Q4_9PASS/67-319 | 2.0e-80 | Manacus vitellinus |
| H2YVE3_CIOSA/50-300 | 1.9e-80 | Ciona savignyi |
| Q596C5_ONCMY/53-305 | 1.9e-80 | Oncorhynchus mykiss |
| A0A060WUQ8_ONCMY/53-305 | 1.9e-80 | Oncorhynchus mykiss |
| A0A060WGS8_ONCMY/53-305 | 1.9e-80 | Oncorhynchus mykiss |
| H2YVE6_CIOSA/42-292 | 1.9e-80 | Ciona savignyi |
| C6EVS4_HUMAN/14-264 | 2.2e-80 | Homo sapiens |
| E9GZ67_DAPPU/70-326 | 1.9e-80 | Daphnia pulex |
| H2YVE8_CIOSA/38-288 | 2.1e-80 | Ciona savignyi |
| A0A087V7Q6_BALRE/51-325 | 1.6e-80 | Balearica regulorum gibbericeps |
| K4DI57_SOLLC/1686-1932 | 2.4e-80 | Solanum lycopersicum |
| A0A0B7AGY3_9EUPU/185-441 | 2.3e-80 | Arion vulgaris |
| A0A0B7AEW7_9EUPU/185-441 | 2.3e-80 | Arion vulgaris |
| F1KTE0_ASCSU/413-665 | 2.3e-80 | Ascaris suum |
| A0A0B7AGL1_9EUPU/185-441 | 2.5e-80 | Arion vulgaris |
| R0I1G2_9BRAS/1736-1980 | 3.3e-80 | Capsella rubella |
| DEK1_ARATH/1736-1979 | 3.4e-80 | Arabidopsis thaliana |
| F4I0A4_ARATH/1764-2007 | 3.5e-80 | Arabidopsis thaliana |
| A0A091NXK6_HALAL/51-325 | 2.8e-80 | Haliaeetus albicilla |
| F1KZB1_ASCSU/281-534 | 2.7e-80 | Ascaris suum |
| A0A091LKJ9_CATAU/51-325 | 2.1e-80 | Cathartes aura |
| A0A0B2VEU6_TOXCA/283-535 | 3.0e-80 | Toxocara canis |
| C3ZKN8_BRAFL/47-305 | 3.2e-80 | Branchiostoma floridae |
| CAN_CAEEL/341-593 | 3.2e-80 | Caenorhabditis elegans |
| G3Q8A6_GASAC/67-325 | 4.6e-80 | Gasterosteus aculeatus |
| A0A091RZK4_NESNO/67-319 | 3.8e-80 | Nestor notabilis |
| M5WS03_PRUPE/1742-1988 | 4.4e-80 | prunus_persica |
| A0A087U142_9ARAC/140-396 | 3.8e-80 | Stegodyphus mimosarum |
| M5W795_PRUPE/1742-1988 | 4.7e-80 | prunus_persica |
| A0A016UIH3_9BILA/237-489 | 4.4e-80 | Ancylostoma ceylanicum |
| A0A016UJ10_9BILA/239-491 | 4.4e-80 | Ancylostoma ceylanicum |
| A0A093CBI1_TAUER/51-325 | 4.4e-80 | Tauraco erythrolophus |
| D7KM88_ARALL/1736-1979 | 6.5e-80 | Arabidopsis lyrata subsp. lyrata |
| R7VLA7_CAPTE/49-324 | 5.3e-80 | Capitella teleta |
| V8NSD3_OPHHA/137-389 | 7.5e-80 | Ophiophagus hannah |
| A0A016UMS7_9BILA/293-549 | 5.4e-80 | Ancylostoma ceylanicum |
| A0A016UL99_9BILA/293-549 | 5.5e-80 | Ancylostoma ceylanicum |
| A0A016UNT0_9BILA/296-552 | 5.5e-80 | Ancylostoma ceylanicum |
| A0A0B7AEG2_9EUPU/114-370 | 5.5e-80 | Arion vulgaris |
| A0A023FWJ4_9ACAR/73-326 | 4.9e-80 | Amblyomma parvum |
| A0A0B7ADU6_9EUPU/114-370 | 5.5e-80 | Arion vulgaris |
| A0A0B7AGX8_9EUPU/114-370 | 5.5e-80 | Arion vulgaris |
| A0A0B7AGY8_9EUPU/114-370 | 5.6e-80 | Arion vulgaris |
| A0A016ULV4_9BILA/293-549 | 5.9e-80 | Ancylostoma ceylanicum |
| A0A016UML0_9BILA/296-552 | 6.0e-80 | Ancylostoma ceylanicum |
| U6J063_ECHGR/141-394 | 5.8e-80 | Echinococcus granulosus |
| A0A068Y9K5_ECHMU/141-394 | 5.8e-80 | Echinococcus multilocularis |
| U3K956_FICAL/51-325 | 6.5e-80 | Ficedula albicollis |
| A0A016UJT1_9BILA/389-641 | 6.6e-80 | Ancylostoma ceylanicum |
| F6WTS6_XENTR/2-271 | 5.0e-80 | Xenopus tropicalis |
| H2YVE9_CIOSA/37-291 | 7.3e-80 | Ciona savignyi |
| A0A022RK49_ERYGU/1736-1977 | 8.4e-80 | Erythranthe guttata |
| A0A022QEI4_ERYGU/1742-1983 | 8.4e-80 | Erythranthe guttata |
| E5SH48_TRISP/345-598 | 7.3e-80 | Trichinella spiralis |
| V4CR31_LOTGI/55-330 | 8.3e-80 | Lottia gigantea |
| W4WI34_ATTCE/136-405 | 2.8e-79 | Atta cephalotes |
| Q387D9_TRYB2/50-277 | 5.5e-27 | Trypanosoma brucei brucei |
| Q387D9_TRYB2/663-891 | 9.0e-27 | Trypanosoma brucei brucei |
| Q387D9_TRYB2/5569-5768 | 3.9e-14 | Trypanosoma brucei brucei |
| Q6SSJ2_NICBE/1726-1970 | 1.1e-79 | Nicotiana benthamiana |
| W6NAM0_HAECO/383-635 | 9.9e-80 | Haemonchus contortus |
| Q762C4_MOUSE/28-275 | 1.0e-79 | Mus musculus |
| A0A091NKV5_APAVI/67-319 | 1.2e-79 | Apaloderma vittatum |
| D0A5Q3_TRYB9/50-277 | 5.8e-27 | Trypanosoma brucei gambiense |
| D0A5Q3_TRYB9/663-891 | 9.6e-27 | Trypanosoma brucei gambiense |
| D0A5Q3_TRYB9/5569-5768 | 4.1e-14 | Trypanosoma brucei gambiense |
| V9KEP7_CALMI/76-326 | 1.1e-79 | Callorhinchus milii |
| A0A093BVN6_9AVES/67-319 | 1.2e-79 | Pterocles gutturalis |
| A0A0B1T1B1_OESDE/258-510 | 1.0e-79 | Oesophagostomum dentatum |
| H9GJR3_ANOCA/53-327 | 9.3e-80 | Anolis carolinensis |
| A0A091PSA9_HALAL/52-304 | 1.6e-79 | Haliaeetus albicilla |
| K7GWB6_CAEJA/380-632 | 1.4e-79 | Caenorhabditis japonica |
| A0A087V8Z4_BALRE/67-319 | 1.7e-79 | Balearica regulorum gibbericeps |
| K7GWB7_CAEJA/424-676 | 1.5e-79 | Caenorhabditis japonica |
| M7BBW7_CHEMY/2-271 | 1.2e-79 | Chelonia mydas |
| T2MIT4_HYDVU/49-323 | 1.6e-79 | Hydra vulgaris |
| E9AD28_LEIMA/48-275 | 1.8e-27 | Leishmania major |
| E9AD28_LEIMA/666-887 | 8.5e-27 | Leishmania major |
| E9AD28_LEIMA/4765-4933 | 2.4e-13 | Leishmania major |
| A0A093J3U0_STRCA/57-309 | 2.0e-79 | Struthio camelus australis |
| A0A091KKZ7_9GRUI/67-319 | 2.0e-79 | Chlamydotis macqueenii |
| A0A091S5X1_NESNO/51-325 | 1.5e-79 | Nestor notabilis |
| B2GUG2_XENTR/67-319 | 2.1e-79 | Xenopus tropicalis |
| A0A068XWF4_HYMMI/142-396 | 2.3e-79 | Hymenolepis microstoma |
| H0ZI51_TAEGU/67-319 | 2.5e-79 | Taeniopygia guttata |
| T1KHM5_TETUR/65-324 | 2.2e-79 | Tetranychus urticae |
| A0A091KQM6_9GRUI/3-270 | 2.8e-79 | Chlamydotis macqueenii |
| A4HFH6_LEIBR/47-274 | 4.8e-26 | Leishmania braziliensis |
| A4HFH6_LEIBR/667-887 | 6.2e-27 | Leishmania braziliensis |
| A4HFH6_LEIBR/5579-5793 | 1.5e-14 | Leishmania braziliensis |
| V4MF78_EUTSA/1736-1980 | 3.1e-79 | Eutrema salsugineum |
| A0A091Q4D9_LEPDC/51-325 | 1.8e-79 | Leptosomus discolor |
| A0A088SCJ7_9TRYP/47-274 | 4.8e-26 | Leishmania panamensis |
| A0A088SCJ7_9TRYP/667-887 | 6.5e-27 | Leishmania panamensis |
| A0A088SCJ7_9TRYP/5579-5793 | 1.5e-14 | Leishmania panamensis |
| Q762C5_MOUSE/28-275 | 3.0e-79 | Mus musculus |
| B9GMB8_POPTR/1705-1951 | 3.3e-79 | Populus trichocarpa |
| A0A067EGZ4_CITSI/1748-1989 | 3.4e-79 | Citrus sinensis |
| V4TYY8_9ROSI/1678-1919 | 3.4e-79 | Citrus clementina |
| U5DBI3_AMBTC/1713-1955 | 4.3e-79 | Amborella trichopoda |
| M4FA81_BRARP/1737-1980 | 3.8e-79 | Brassica rapa subsp. pekinensis |
| A0A091JKI9_9AVES/67-319 | 3.5e-79 | Egretta garzetta |
| A0A067EH92_CITSI/1748-1989 | 3.6e-79 | Citrus sinensis |
| G0N3H0_CAEBE/398-650 | 2.9e-79 | Caenorhabditis brenneri |
| K1R1C6_CRAGI/53-328 | 3.3e-79 | Crassostrea gigas |
| A0A091H363_9AVES/67-319 | 3.8e-79 | Cuculus canorus |
| A5BD62_VITVI/1090-1336 | 3.7e-79 | Vitis vinifera |
| G0P3W6_CAEBE/278-553 | 3.5e-79 | Caenorhabditis brenneri |
| A0A0A0AYD4_CHAVO/67-319 | 3.9e-79 | Charadrius vociferus |
| V9KMA3_CALMI/76-326 | 3.4e-79 | Callorhinchus milii |
| A0A091R6S2_MERNU/67-319 | 3.9e-79 | Merops nubicus |
| E3MNY5_CAERE/352-604 | 3.6e-79 | Caenorhabditis remanei |
| A0A091QA21_MERNU/3-270 | 4.4e-79 | Merops nubicus |
| G1Q0P0_MYOLU/76-333 | 4.2e-79 | Myotis lucifugus |
| C5WML7_SORBI/1745-1988 | 6.0e-79 | Sorghum bicolor |
| L1J0U9_GUITH/245-480 | 1.6e-39 | Guillardia theta CCMP2712 |
| L1J0U9_GUITH/804-1059 | 7.9e-34 | Guillardia theta CCMP2712 |
| Q5DC34_SCHJA/146-403 | 4.2e-79 | Schistosoma japonicum |
| C1LFG3_SCHJA/146-403 | 4.3e-79 | Schistosoma japonicum |
| A0A091THL1_PHALP/35-287 | 4.7e-79 | Phaethon lepturus |
| A0A0D3CR01_BRAOL/1756-1999 | 5.9e-79 | Brassica oleracea var. oleracea |
| CAN9_MOUSE/67-319 | 4.6e-79 | Mus musculus |
| A0A091EBU7_CORBR/67-319 | 5.6e-79 | Corvus brachyrhynchos |
| A8Y3L5_CAEBR/480-732 | 4.8e-79 | Caenorhabditis briggsae |
| A4I2N6_LEIIN/45-273 | 7.7e-26 | Leishmania infantum |
| A4I2N6_LEIIN/664-887 | 1.3e-26 | Leishmania infantum |
| A4I2N6_LEIIN/5583-5787 | 1.0e-14 | Leishmania infantum |
| D7SLL2_VITVI/1741-1987 | 6.2e-79 | Vitis vinifera |
| L7LYW7_9ACAR/72-325 | 5.0e-79 | Rhipicephalus pulchellus |
| K4A4N5_SETIT/1746-1989 | 8.6e-79 | Setaria italica |
| A0A023EXU5_AEDAL/94-350 | 5.9e-79 | Aedes albopictus |
| A0A023EV80_AEDAL/94-350 | 6.1e-79 | Aedes albopictus |
| A0A091MJL0_9PASS/3-271 | 7.1e-79 | Acanthisitta chloris |
| A0A0B0MTZ6_GOSAR/1716-1957 | 6.9e-79 | Gossypium arboreum |
| Q6PDE6_MOUSE/67-319 | 6.3e-79 | Mus musculus |
| A0A090MZ41_STRRB/391-643 | 6.1e-79 | Strongyloides ratti |
| DEK1_MAIZE/1744-1987 | 9.6e-79 | Zea mays |
| A0A096T7S4_MAIZE/1744-1987 | 9.6e-79 | Zea mays |
| A0A0E0K491_ORYPU/1840-2084 | 7.0e-79 | oryza_punctata |
| A0A0E0K492_ORYPU/1843-2087 | 7.0e-79 | oryza_punctata |
| L7MBK8_9ACAR/135-388 | 6.1e-79 | Rhipicephalus pulchellus |
| Q8GT31_9POAL/745-988 | 5.1e-79 | Saccharum hybrid cultivar |
| J3LGC8_ORYBR/1747-1990 | 1.0e-78 | Oryza brachyantha |
| H2S158_TAKRU/41-306 | 1.3e-78 | Takifugu rubripes |
| Q8RUQ1_MAIZE/1745-1987 | 1.2e-78 | Zea mays |
| G3Q5H4_GASAC/81-332 | 8.1e-79 | Gasterosteus aculeatus |
| H2ZUV0_LATCH/67-319 | 3.2e-78 | Latimeria chalumnae |
| Q17E35_AEDAE/94-350 | 9.3e-79 | Aedes aegypti |
| A0A093GAS8_PICPB/3-270 | 6.2e-79 | Picoides pubescens |
| A0A0D9VK29_9ORYZ/1701-1944 | 9.9e-79 | leersia_perrieri |
| A0A087X9C1_POEFO/85-336 | 9.1e-79 | Poecilia formosa |
| W5NF88_LEPOC/85-336 | 9.5e-79 | Lepisosteus oculatus |
| W5KVG2_ASTMX/51-326 | 1.1e-78 | Astyanax mexicanus |
| A0A074ZHY8_9TREM/122-377 | 1.0e-78 | Opisthorchis viverrini |
| G3TLI8_LOXAF/99-271 | 3.5e-53 | Loxodonta africana |
| G3TLI8_LOXAF/321-399 | 1.6e-19 | Loxodonta africana |
| A0A0E0CQH3_9ORYZ/1465-1709 | 1.2e-78 | oryza_meridionalis |
| DEK1_ORYSJ/1746-1990 | 1.5e-78 | Oryza sativa subsp. japonica |
| A0A091IFX0_CALAN/59-311 | 1.3e-78 | Calypte anna |
| A0A067K6C9_JATCU/926-1164 | 1.3e-78 | Jatropha curcas |
| A0A0E0NJY1_ORYRU/1586-1830 | 1.4e-78 | oryza_rufipogon |
| G7YHP4_CLOSI/134-389 | 1.2e-78 | Clonorchis sinensis |
| B9F2B3_ORYSJ/1662-1906 | 1.4e-78 | Oryza sativa subsp. japonica |
| B8AHB8_ORYSI/1662-1906 | 1.4e-78 | Oryza sativa subsp. indica |
| A0A0D9RDG1_CHLSB/67-319 | 1.4e-78 | Chlorocebus sabaeus |
| I1FGV4_AMPQE/73-327 | 1.3e-78 | Amphimedon queenslandica |
| A0A0E0GC02_ORYNI/1761-2005 | 1.5e-78 | oryza_nivara |
| A0A0D9YX79_9ORYZ/1586-1830 | 1.6e-78 | oryza_glumaepatula |
| Q17E36_AEDAE/91-343 | 1.5e-78 | Aedes aegypti |
| A7S7W7_NEMVE/44-304 | 1.6e-78 | Nematostella vectensis |
| A0A023GP13_9ACAR/83-336 | 1.4e-78 | Amblyomma triste |
| A0A061GQC2_THECC/1020-1261 | 1.9e-78 | theobroma_cacao |
| A0A0B7B7Z1_9EUPU/53-328 | 2.2e-78 | Arion vulgaris |
| A0A087YG43_POEFO/78-329 | 1.7e-78 | Poecilia formosa |
| M0T5I0_MUSAM/1749-1991 | 2.4e-78 | Musa acuminata subsp. malaccensis |
| M0YWZ5_HORVD/271-517 | 1.4e-78 | Hordeum vulgare var. distichum |
| V5HD85_IXORI/59-312 | 1.9e-78 | Ixodes ricinus |
| A6QP82_BOVIN/99-271 | 2.8e-53 | Bos taurus |
| A6QP82_BOVIN/323-400 | 3.1e-19 | Bos taurus |
| H2T142_TAKRU/75-326 | 2.1e-78 | Takifugu rubripes |
| Q5IW11_GECLA/53-325 | 2.3e-78 | Gecarcinus lateralis |
| A8J6T6_CHLRE/3-316 | 1.1e-45 | Chlamydomonas reinhardtii |
| A8J6T6_CHLRE/425-705 | 1.1e-26 | Chlamydomonas reinhardtii |
| H2T137_TAKRU/78-329 | 2.2e-78 | Takifugu rubripes |
| G3TVX4_LOXAF/99-271 | 3.5e-53 | Loxodonta africana |
| G3TVX4_LOXAF/321-401 | 3.6e-19 | Loxodonta africana |
| A0A024R9M1_HUMAN/99-271 | 2.4e-52 | Homo sapiens |
| A0A024R9M1_HUMAN/322-399 | 4.1e-20 | Homo sapiens |
| G7MWY9_MACMU/99-271 | 1.0e-52 | Macaca mulatta |
| G7MWY9_MACMU/321-399 | 1.1e-19 | Macaca mulatta |
| U3BUK4_CALJA/85-336 | 4.6e-76 | Callithrix jacchus |
| H2T138_TAKRU/98-349 | 2.4e-78 | Takifugu rubripes |
| A0A061GQN2_THECC/1743-1984 | 3.2e-78 | theobroma_cacao |
| I3JUL7_ORENI/75-326 | 2.7e-78 | Oreochromis niloticus |
| A0A096NFY3_PAPAN/67-319 | 2.7e-78 | Papio anubis |
| U3CDJ6_CALJA/85-336 | 4.6e-76 | Callithrix jacchus |
| U3FKM9_CALJA/85-336 | 4.6e-76 | Callithrix jacchus |
| G7NXK9_MACFA/67-319 | 2.7e-78 | Macaca fascicularis |
| G7MFR3_MACMU/67-319 | 2.7e-78 | Macaca mulatta |
| F6TLF5_MACMU/67-319 | 2.7e-78 | Macaca mulatta |
| A0A061GQB8_THECC/1743-1984 | 3.4e-78 | theobroma_cacao |
| A0A059AEU0_EUCGR/1737-1979 | 3.0e-78 | Eucalyptus grandis |
| S8CMX5_9LAMI/1773-2019 | 3.2e-78 | Genlisea aurea |
| A0A091UKZ4_NIPNI/67-319 | 3.5e-78 | Nipponia nippon |
| Q9R113_RAT/79-251 | 1.0e-52 | Rattus norvegicus |
| Q9R113_RAT/301-379 | 1.2e-19 | Rattus norvegicus |
| E9AD27_LEIMA/45-273 | 1.8e-25 | Leishmania major |
| E9AD27_LEIMA/664-887 | 1.6e-26 | Leishmania major |
| E9AD27_LEIMA/5579-5782 | 2.0e-14 | Leishmania major |
| D2IKJ9_HIPHI/102-356 | 4.0e-78 | Hippoglossus hippoglossus |
| R0JTT5_ANAPL/67-319 | 3.7e-78 | Anas platyrhynchos |
| A0A0D2QCX5_GOSRA/1738-1978 | 3.8e-78 | Gossypium raimondii |
| A0A093BSR7_CHAPE/67-319 | 3.5e-78 | Chaetura pelagica |
| A0A091QS89_9GRUI/67-319 | 3.5e-78 | Mesitornis unicolor |
| I3K5P4_ORENI/82-333 | 3.8e-78 | Oreochromis niloticus |
| K1RWI3_CRAGI/55-326 | 3.6e-78 | Crassostrea gigas |
| S7PJR0_MYOBR/51-324 | 3.3e-78 | Myotis brandtii |
| W9SJ89_9ROSA/1742-1987 | 4.5e-78 | Morus notabilis |
| H2T140_TAKRU/47-298 | 3.6e-78 | Takifugu rubripes |
| O70482_RAT/79-251 | 7.7e-53 | Rattus norvegicus |
| O70482_RAT/301-379 | 2.2e-19 | Rattus norvegicus |
| A0A091S9F5_NESNO/95-394 | 1.3e-76 | Nestor notabilis |
| I3JUI6_ORENI/86-337 | 4.0e-78 | Oreochromis niloticus |
| A2AVV7_MOUSE/79-251 | 7.7e-53 | Mus musculus |
| A2AVV7_MOUSE/301-379 | 2.3e-19 | Mus musculus |
| I3JFK8_ORENI/102-356 | 5.0e-78 | Oreochromis niloticus |
| Q9QZF9_RAT/79-251 | 8.2e-53 | Rattus norvegicus |
| Q9QZF9_RAT/301-379 | 2.4e-19 | Rattus norvegicus |
| CAN3_BOVIN/99-271 | 6.7e-53 | Bos taurus |
| CAN3_BOVIN/323-400 | 3.1e-19 | Bos taurus |
| A2AVV6_MOUSE/79-251 | 8.2e-53 | Mus musculus |
| A2AVV6_MOUSE/301-379 | 2.4e-19 | Mus musculus |
| E9QFN0_DANRE/80-331 | 4.8e-78 | Danio rerio |
| CAN3_HUMAN/99-271 | 3.9e-52 | Homo sapiens |
| CAN3_HUMAN/322-399 | 6.0e-20 | Homo sapiens |
| X1WC14_DANRE/90-341 | 5.0e-78 | Danio rerio |
| K7G6Z7_PELSI/75-326 | 4.6e-78 | Pelodiscus sinensis |
| M0YWZ6_HORVD/631-877 | 3.5e-78 | Hordeum vulgare var. distichum |
| B9SBQ0_RICCO/1747-1986 | 5.7e-78 | Ricinus communis |
| CAN9_HUMAN/67-319 | 5.3e-78 | Homo sapiens |
| G3RAS2_GORGO/67-319 | 5.3e-78 | Gorilla gorilla gorilla |
| E3MIU5_CAERE/270-540 | 3.8e-76 | Caenorhabditis remanei |
| G3NLY6_GASAC/72-323 | 5.3e-78 | Gasterosteus aculeatus |
| J9JU00_ACYPI/56-307 | 5.5e-78 | Acyrthosiphon pisum |
| G3NM11_GASAC/78-329 | 5.4e-78 | Gasterosteus aculeatus |
| H3CKC4_TETNG/75-326 | 5.3e-78 | Tetraodon nigroviridis |
| H3DGU2_TETNG/75-326 | 5.3e-78 | Tetraodon nigroviridis |
| H3C151_TETNG/78-329 | 5.5e-78 | Tetraodon nigroviridis |
| H3BX34_TETNG/81-332 | 5.5e-78 | Tetraodon nigroviridis |
| H0XKK2_OTOGA/99-271 | 2.6e-52 | Otolemur garnettii |
| H0XKK2_OTOGA/322-399 | 1.3e-19 | Otolemur garnettii |
| A0A091H4M0_BUCRH/2-248 | 9.0e-78 | Buceros rhinoceros silvestris |
| A0A091PF54_LEPDC/67-319 | 6.6e-78 | Leptosomus discolor |
| A8PPB7_BRUMA/225-477 | 5.6e-78 | Brugia malayi |
| B2RDI5_HUMAN/85-336 | 3.3e-75 | Homo sapiens |
| H3C6V4_TETNG/102-353 | 5.8e-78 | Tetraodon nigroviridis |
| A0A0D6LMM3_9BILA/172-427 | 5.9e-78 | Ancylostoma ceylanicum |
| A0A0A0RAU7_SHEEP/92-264 | 1.5e-52 | Ovis aries |
| A0A0A0RAU7_SHEEP/315-393 | 1.7e-19 | Ovis aries |
| A0A0A0R8C1_SHEEP/92-264 | 1.5e-52 | Ovis aries |
| A0A0A0R8C1_SHEEP/315-393 | 1.7e-19 | Ovis aries |
| A0A024R5A3_HUMAN/85-336 | 3.2e-75 | Homo sapiens |
| G1QME7_NOMLE/67-319 | 6.4e-78 | Nomascus leucogenys |
| A0A024R580_HUMAN/85-336 | 3.3e-75 | Homo sapiens |
| A8PNE4_BRUMA/225-477 | 5.9e-78 | Brugia malayi |
| CAN1_HUMAN/85-336 | 3.3e-75 | Homo sapiens |
| V5GXY9_IXORI/59-312 | 6.4e-78 | Ixodes ricinus |
| B6C9J6_HORVD/1742-1988 | 1.0e-77 | Hordeum vulgare var. distichum |
| W5QH59_SHEEP/99-271 | 1.6e-52 | Ovis aries |
| W5QH59_SHEEP/322-400 | 1.8e-19 | Ovis aries |
| Q6GLE3_XENTR/75-326 | 6.5e-78 | Xenopus tropicalis |
| A0A0B2VN55_TOXCA/302-554 | 6.2e-78 | Toxocara canis |
| G3H0X3_CRIGR/79-251 | 1.1e-52 | Cricetulus griseus |
| G3H0X3_CRIGR/301-379 | 3.0e-19 | Cricetulus griseus |
| A8NBA6_COPC7/108-387 | 7.3e-78 | Coprinopsis cinerea |
| A4HFH5_LEIBR/48-271 | 2.0e-24 | Leishmania braziliensis |
| A4HFH5_LEIBR/671-896 | 1.4e-25 | Leishmania braziliensis |
| A4HFH5_LEIBR/4400-4604 | 5.5e-16 | Leishmania braziliensis |
| E1C6M3_CHICK/67-319 | 7.9e-78 | Gallus gallus |
| L8I1L7_9CETA/99-271 | 1.3e-52 | Bos mutus |
| L8I1L7_9CETA/323-400 | 2.5e-19 | Bos mutus |
| I1KSP1_SOYBN/1739-1979 | 9.0e-78 | Glycine max |
| G3GX11_CRIGR/67-319 | 8.1e-78 | Cricetulus griseus |
| J7GU62_BOSMU/99-271 | 1.3e-52 | Bos mutus grunniens |
| J7GU62_BOSMU/323-400 | 2.5e-19 | Bos mutus grunniens |
| A0A0B2SN56_GLYSO/1722-1962 | 9.1e-78 | Glycine soja |
| L5K1Q9_PTEAL/99-271 | 1.6e-52 | Pteropus alecto |
| L5K1Q9_PTEAL/321-399 | 2.4e-19 | Pteropus alecto |
| I1ID37_BRADI/1748-1991 | 9.7e-78 | Brachypodium distachyon |
| A0A0A0MXZ1_RAT/67-319 | 7.7e-78 | Rattus norvegicus |
| CAN9_RAT/67-319 | 7.7e-78 | Rattus norvegicus |
| A0A087YMN7_POEFO/102-356 | 6.5e-78 | Poecilia formosa |
| M3YEJ8_MUSPF/99-271 | 1.7e-52 | Mustela putorius furo |
| M3YEJ8_MUSPF/321-399 | 2.3e-19 | Mustela putorius furo |
| M0YWZ4_HORVD/1033-1279 | 5.9e-78 | Hordeum vulgare var. distichum |
| A0A0A0MXU0_RAT/75-326 | 7.3e-78 | Rattus norvegicus |
| V3Z6F4_LOTGI/52-322 | 7.8e-78 | Lottia gigantea |
| H2M8N5_ORYLA/85-336 | 8.3e-78 | Oryzias latipes |
| Q6P415_XENLA/75-326 | 8.6e-78 | Xenopus laevis |
| B0WSM1_CULQU/91-343 | 8.9e-78 | Culex quinquefasciatus |
| F6ZEJ6_MONDO/75-326 | 1.1e-77 | Monodelphis domestica |
| H2RBT6_PANTR/99-271 | 2.5e-52 | Pan troglodytes |
| H2RBT6_PANTR/322-399 | 1.7e-19 | Pan troglodytes |
| D2GW72_AILME/99-271 | 1.6e-52 | Ailuropoda melanoleuca |
| D2GW72_AILME/321-399 | 2.7e-19 | Ailuropoda melanoleuca |
| F7EDP7_MONDO/67-319 | 9.1e-78 | Monodelphis domestica |
| A0A093P2W2_PYGAD/67-319 | 1.1e-77 | Pygoscelis adeliae |
| Q66IH0_XENTR/85-336 | 8.9e-77 | Xenopus tropicalis |
| H2Q1B8_PANTR/67-319 | 9.4e-78 | Pan troglodytes |
| G1MBZ6_AILME/99-271 | 1.7e-52 | Ailuropoda melanoleuca |
| G1MBZ6_AILME/321-399 | 2.8e-19 | Ailuropoda melanoleuca |
| M4ALQ1_XIPMA/51-326 | 9.1e-78 | Xiphophorus maculatus |
| V5IF45_IXORI/73-326 | 1.0e-77 | Ixodes ricinus |
| E2R8T7_CANFA/99-271 | 1.7e-52 | Canis familiaris |
| E2R8T7_CANFA/321-399 | 2.8e-19 | Canis familiaris |
| G1QWI5_NOMLE/99-271 | 2.5e-52 | Nomascus leucogenys |
| G1QWI5_NOMLE/322-399 | 2.0e-19 | Nomascus leucogenys |
| A0A096NTR8_PAPAN/99-271 | 4.6e-52 | Papio anubis |
| A0A096NTR8_PAPAN/321-399 | 1.1e-19 | Papio anubis |
| CAN3_PIG/98-270 | 1.4e-52 | Sus scrofa |
| CAN3_PIG/322-399 | 3.4e-19 | Sus scrofa |
| F7G8S2_MACMU/99-271 | 4.6e-52 | Macaca mulatta |
| F7G8S2_MACMU/321-399 | 1.1e-19 | Macaca mulatta |
| CAN3_MACFA/99-271 | 4.6e-52 | Macaca fascicularis |
| CAN3_MACFA/321-399 | 1.1e-19 | Macaca fascicularis |
| G7PB02_MACFA/99-271 | 4.6e-52 | Macaca fascicularis |
| G7PB02_MACFA/321-399 | 1.1e-19 | Macaca fascicularis |
| F1SI23_PIG/99-271 | 1.4e-52 | Sus scrofa |
| F1SI23_PIG/323-400 | 3.4e-19 | Sus scrofa |
| F7G923_MACMU/99-271 | 4.6e-52 | Macaca mulatta |
| F7G923_MACMU/321-399 | 1.1e-19 | Macaca mulatta |
| J9E1S9_WUCBA/29-281 | 9.0e-78 | Wuchereria bancrofti |
| W5GMM4_WHEAT/1654-1897 | 1.8e-77 | Triticum aestivum |
| A2VDQ9_BOVIN/75-326 | 9.9e-78 | Bos taurus |
| Q6GN47_XENLA/67-319 | 1.3e-77 | Xenopus laevis |
| J0XGW2_LOALO/221-473 | 1.1e-77 | Loa loa |
| W5G959_WHEAT/1655-1898 | 2.0e-77 | Triticum aestivum |
| A0A094LDF5_9AVES/67-319 | 1.4e-77 | Podiceps cristatus |
| M8C6S9_AEGTA/1714-1957 | 2.1e-77 | aegilops_tauschii |
| A0A091EVW4_CORBR/3-278 | 1.4e-77 | Corvus brachyrhynchos |
| H2T6V7_TAKRU/72-323 | 1.3e-77 | Takifugu rubripes |
| W5GTX8_WHEAT/1745-1988 | 2.1e-77 | Triticum aestivum |
| H2T6V6_TAKRU/77-328 | 1.3e-77 | Takifugu rubripes |
| Q5IW13_GECLA/112-371 | 1.3e-77 | Gecarcinus lateralis |
| H2T6V5_TAKRU/85-336 | 1.3e-77 | Takifugu rubripes |
| M7AJJ8_CHEMY/75-326 | 1.4e-77 | Chelonia mydas |
| K7GD78_PELSI/67-319 | 1.5e-77 | Pelodiscus sinensis |
| E2RSU9_CANFA/67-319 | 1.5e-77 | Canis familiaris |
| E2RSU5_CANFA/67-319 | 1.5e-77 | Canis familiaris |
| A0A091H3E3_BUCRH/94-393 | 1.1e-76 | Buceros rhinoceros silvestris |
| K1PW75_CRAGI/87-357 | 2.1e-77 | Crassostrea gigas |
| M3WNS8_FELCA/99-271 | 1.7e-52 | Felis catus |
| M3WNS8_FELCA/321-399 | 4.4e-19 | Felis catus |
| A0A060VYZ7_ONCMY/75-326 | 1.3e-77 | Oncorhynchus mykiss |
| A0A093PL42_9PASS/94-393 | 1.8e-76 | Manacus vitellinus |
| H2NMZ5_PONAB/99-271 | 2.9e-52 | Pongo abelii |
| H2NMZ5_PONAB/323-399 | 2.6e-19 | Pongo abelii |
| H2T139_TAKRU/53-304 | 1.4e-77 | Takifugu rubripes |
| F7G830_ORNAN/75-326 | 2.0e-77 | Ornithorhynchus anatinus |
| G1TSG0_RABIT/99-271 | 4.4e-52 | Oryctolagus cuniculus |
| G1TSG0_RABIT/321-399 | 1.5e-19 | Oryctolagus cuniculus |
| M3ZVA7_XIPMA/82-333 | 1.6e-77 | Xiphophorus maculatus |
| A0A0D9R873_CHLSB/99-271 | 3.4e-52 | Chlorocebus sabaeus |
| A0A0D9R873_CHLSB/321-399 | 2.2e-19 | Chlorocebus sabaeus |
| A0A091K398_COLST/95-394 | 2.6e-76 | Colius striatus |
| T1IQ80_STRMM/137-397 | 1.6e-77 | Strigamia maritima |
| E9AHC1_LEIIN/48-275 | 7.4e-27 | Leishmania infantum |
| E9AHC1_LEIIN/666-887 | 3.9e-26 | Leishmania infantum |
| E9AHC1_LEIIN/3750-3918 | 1.2e-12 | Leishmania infantum |
| A0A091LE34_CATAU/94-393 | 1.2e-76 | Cathartes aura |
| T0M4Y4_9CETA/79-344 | 2.2e-77 | Camelus ferus |
| A0A093I3D6_STRCA/94-393 | 6.3e-77 | Struthio camelus australis |
| Q762C7_HUMAN/14-183 | 1.7e-51 | Homo sapiens |
| Q762C7_HUMAN/235-312 | 2.6e-20 | Homo sapiens |
| A0A091IEH6_CALAN/94-393 | 2.8e-76 | Calypte anna |
| A0A087XZ47_POEFO/51-326 | 1.8e-77 | Poecilia formosa |
| M3ZUY6_XIPMA/78-329 | 1.8e-77 | Xiphophorus maculatus |
| A0A091WEF6_OPIHO/60-312 | 1.9e-77 | Opisthocomus hoazin |
| H2UH72_TAKRU/72-347 | 1.7e-77 | Takifugu rubripes |
| B6V3H9_CHICK/93-392 | 1.7e-76 | Gallus gallus |
| B8Y8S8_CHICK/93-392 | 1.7e-76 | Gallus gallus |
| F1NJ45_CHICK/93-392 | 1.7e-76 | Gallus gallus |
| A0A099ZE15_TINGU/50-301 | 2.1e-77 | Tinamus guttatus |
| W5NF49_LEPOC/85-336 | 2.0e-77 | Lepisosteus oculatus |
| A0A0A0LX51_CUCSA/1750-1990 | 2.8e-77 | Cucumis sativus |
| A0A091M244_CARIC/94-393 | 2.6e-76 | Cariama cristata |
| F1LSQ2_RAT/99-271 | 4.2e-52 | Rattus norvegicus |
| F1LSQ2_RAT/321-399 | 2.6e-19 | Rattus norvegicus |
| Q8MQV0_HOMAM/111-371 | 1.9e-77 | Homarus americanus |
| CAN3_MOUSE/99-271 | 4.2e-52 | Mus musculus |
| CAN3_MOUSE/321-399 | 2.6e-19 | Mus musculus |
| F6RX19_HORSE/99-271 | 2.4e-52 | Equus caballus |
| F6RX19_HORSE/321-399 | 5.2e-19 | Equus caballus |
| F6RX57_HORSE/99-271 | 2.5e-52 | Equus caballus |
| F6RX57_HORSE/321-399 | 5.2e-19 | Equus caballus |
| A0A091X854_OPIHO/94-393 | 2.6e-76 | Opisthocomus hoazin |
| E3MBL7_CAERE/72-282 | 2.3e-40 | Caenorhabditis remanei |
| E3MBL7_CAERE/593-807 | 3.0e-31 | Caenorhabditis remanei |
| G3P5X1_GASAC/52-306 | 2.4e-77 | Gasterosteus aculeatus |
| B8A6G0_DANRE/78-329 | 2.2e-77 | Danio rerio |
| R0LRK8_ANAPL/75-326 | 2.4e-77 | Anas platyrhynchos |
| G3SSD8_LOXAF/67-319 | 2.5e-77 | Loxodonta africana |
| A0A093JER8_EURHL/94-393 | 7.3e-77 | Eurypyga helias |
| A0A087VIS6_BALRE/94-393 | 2.6e-76 | Balearica regulorum gibbericeps |
| W5KKQ5_ASTMX/77-328 | 2.4e-77 | Astyanax mexicanus |
| A0A091K1R4_COLST/67-319 | 2.8e-77 | Colius striatus |
| Q672H2_ONCMY/80-331 | 2.7e-77 | Oncorhynchus mykiss |
| A0A093FAT3_TYTAL/34-286 | 3.0e-77 | Tyto alba |
| H2UH71_TAKRU/51-326 | 2.4e-77 | Takifugu rubripes |
| I3M895_SPETR/85-336 | 2.9e-76 | Spermophilus tridecemlineatus |
| F6YKN2_XENTR/1-166 | 5.3e-50 | Xenopus tropicalis |
| F6YKN2_XENTR/215-292 | 2.3e-21 | Xenopus tropicalis |
| Q7ZXQ4_XENLA/85-336 | 3.4e-77 | Xenopus laevis |
| H2UH74_TAKRU/51-326 | 2.4e-77 | Takifugu rubripes |
| R7VIB6_CAPTE/75-331 | 2.8e-77 | Capitella teleta |
| V9L9Q8_CALMI/42-293 | 2.2e-77 | Callorhinchus milii |
| H2UH70_TAKRU/53-328 | 2.5e-77 | Takifugu rubripes |
| G1NHS3_MELGA/67-319 | 3.0e-77 | Meleagris gallopavo |
| U3IJ17_ANAPL/110-361 | 3.0e-77 | Anas platyrhynchos |
| I3N5R2_SPETR/99-271 | 6.0e-52 | Spermophilus tridecemlineatus |
| I3N5R2_SPETR/321-399 | 2.5e-19 | Spermophilus tridecemlineatus |
| D2HXR9_AILME/67-319 | 3.6e-77 | Ailuropoda melanoleuca |
| B0WSM2_CULQU/119-375 | 2.7e-77 | Culex quinquefasciatus |
| H2YVE5_CIOSA/48-223 | 3.6e-51 | Ciona savignyi |
| H2YVE5_CIOSA/267-342 | 3.3e-20 | Ciona savignyi |
| A4QPE6_MOUSE/99-271 | 5.8e-52 | Mus musculus |
| A4QPE6_MOUSE/321-399 | 2.6e-19 | Mus musculus |
| A7SN59_NEMVE/51-327 | 3.1e-77 | Nematostella vectensis |
| A0A091QYG0_MERNU/94-393 | 6.0e-76 | Merops nubicus |
| S4RFH7_PETMA/8-258 | 3.4e-77 | Petromyzon marinus |
| G1LTD2_AILME/67-319 | 4.0e-77 | Ailuropoda melanoleuca |
| M4AT99_XIPMA/103-357 | 4.0e-77 | Xiphophorus maculatus |
| F7F9H3_ORNAN/75-326 | 3.4e-77 | Ornithorhynchus anatinus |
| G7L8S3_MEDTR/1744-1983 | 3.8e-77 | Medicago truncatula |
| T1EE39_HELRO/55-323 | 3.3e-77 | Helobdella robusta |
| F6VR06_HORSE/67-319 | 3.8e-77 | Equus caballus |
| A0A0A6YVU9_MOUSE/75-326 | 3.1e-77 | Mus musculus |
| CAN8_MOUSE/75-326 | 3.2e-77 | Mus musculus |
| H3CPR3_TETNG/86-337 | 3.4e-77 | Tetraodon nigroviridis |
| A0A091GP17_9AVES/94-393 | 7.7e-76 | Cuculus canorus |
| A0A091K095_9AVES/94-393 | 2.8e-76 | Egretta garzetta |
| Q4SQP6_TETNG/82-333 | 3.5e-77 | Tetraodon nigroviridis |
| A0A0A0MY53_RAT/75-326 | 3.5e-77 | Rattus norvegicus |
| CAN8_RAT/75-326 | 3.5e-77 | Rattus norvegicus |
| U3IYT9_ANAPL/94-393 | 3.0e-76 | Anas platyrhynchos |
| R0L2L3_ANAPL/94-393 | 3.1e-76 | Anas platyrhynchos |
| A0A093LKQ9_FULGA/94-393 | 5.5e-76 | Fulmarus glacialis |
| A0A093NUE0_PYGAD/94-393 | 4.5e-76 | Pygoscelis adeliae |
| V7CN28_PHAVU/1734-1979 | 4.5e-77 | Phaseolus vulgaris |
| M4A255_XIPMA/82-333 | 4.0e-77 | Xiphophorus maculatus |
| V9KIG1_CALMI/75-325 | 4.1e-77 | Callorhinchus milii |
| F7FGW6_ORNAN/66-318 | 4.1e-77 | Ornithorhynchus anatinus |
| H9G0C4_MACMU/85-336 | 1.3e-75 | Macaca mulatta |
| CAN1_MACFA/85-336 | 1.3e-75 | Macaca fascicularis |
| H2YVE1_CIOSA/48-218 | 8.3e-51 | Ciona savignyi |
| H2YVE1_CIOSA/270-347 | 2.3e-20 | Ciona savignyi |
| F6ZLR4_MACMU/85-336 | 1.3e-75 | Macaca mulatta |
| G7PPI8_MACFA/85-336 | 1.3e-75 | Macaca fascicularis |
| Q9I8T0_XENLA/75-326 | 4.1e-77 | Xenopus laevis |
| A0A091NFB1_APAVI/94-393 | 8.7e-76 | Apaloderma vittatum |
| D2H4Q7_AILME/85-336 | 6.6e-76 | Ailuropoda melanoleuca |
| A0A093FTU0_GAVST/94-393 | 5.4e-76 | Gavia stellata |
| S7P8F9_MYOBR/79-251 | 2.3e-52 | Myotis brandtii |
| S7P8F9_MYOBR/296-374 | 1.0e-18 | Myotis brandtii |
| I3MFK2_SPETR/85-336 | 4.7e-77 | Spermophilus tridecemlineatus |
| A0A096NCM9_PAPAN/114-365 | 1.5e-75 | Papio anubis |
| A0A093DDR1_CHAPE/94-393 | 4.5e-76 | Chaetura pelagica |
| A0A0A0AMJ3_CHAVO/94-393 | 5.9e-76 | Charadrius vociferus |
| B2R942_HUMAN/67-319 | 5.2e-77 | Homo sapiens |
| E1BKJ3_BOVIN/104-355 | 5.0e-77 | Bos taurus |
| Q7SYA9_DANRE/78-329 | 5.1e-77 | Danio rerio |
| A0A093T705_PHACA/94-393 | 3.9e-76 | Phalacrocorax carbo |
| Q6AX93_XENLA/85-336 | 6.5e-77 | Xenopus laevis |
| A0A091H0L1_BUCRH/3-270 | 3.8e-77 | Buceros rhinoceros silvestris |
| B7ZQQ8_XENLA/75-326 | 5.2e-77 | Xenopus laevis |
| C0HAB5_SALSA/80-331 | 5.8e-77 | Salmo salar |
| G1MH21_AILME/117-368 | 7.5e-76 | Ailuropoda melanoleuca |
| A0A061I2B5_CRIGR/75-326 | 5.6e-77 | Cricetulus griseus |
| G1NG52_MELGA/93-392 | 2.3e-76 | Meleagris gallopavo |
| G3UP61_MELGA/93-392 | 2.3e-76 | Meleagris gallopavo |
| A0A091NV51_HALAL/94-393 | 6.5e-76 | Haliaeetus albicilla |
| H0ZLH4_TAEGU/93-392 | 8.7e-76 | Taeniopygia guttata |
| M1FSS0_ICTPU/80-331 | 5.9e-77 | Ictalurus punctatus |
| CAN3_RAT/99-271 | 1.2e-51 | Rattus norvegicus |
| CAN3_RAT/321-399 | 2.6e-19 | Rattus norvegicus |
| B7ZQR0_XENLA/75-326 | 5.8e-77 | Xenopus laevis |
| Q6AZS6_XENLA/75-326 | 5.8e-77 | Xenopus laevis |
| A0A094K6H4_ANTCR/94-393 | 9.9e-76 | Antrostomus carolinensis |
| CAN3_CHICK/93-392 | 5.4e-76 | Gallus gallus |
| G3VZS1_SARHA/99-269 | 3.5e-52 | Sarcophilus harrisii |
| G3VZS1_SARHA/320-397 | 8.2e-19 | Sarcophilus harrisii |
| A0A091F467_CORBR/93-392 | 1.0e-75 | Corvus brachyrhynchos |
| A0A091VXH3_NIPNI/94-393 | 6.5e-76 | Nipponia nippon |
| H2LRH5_ORYLA/51-321 | 6.6e-77 | Oryzias latipes |
| C6EVS3_HUMAN/14-184 | 4.3e-51 | Homo sapiens |
| C6EVS3_HUMAN/235-312 | 5.1e-20 | Homo sapiens |
| A0A0B2PFT0_GLYSO/1664-1904 | 8.5e-77 | Glycine soja |
| A4II92_XENTR/75-326 | 6.7e-77 | Xenopus tropicalis |
| F6TCZ2_XENTR/76-327 | 6.8e-77 | Xenopus tropicalis |
| H2YVE2_CIOSA/48-217 | 1.2e-50 | Ciona savignyi |
| H2YVE2_CIOSA/273-348 | 2.2e-20 | Ciona savignyi |
| Q28C80_XENTR/76-327 | 6.9e-77 | Xenopus tropicalis |
| M3XZU8_MUSPF/68-320 | 7.7e-77 | Mustela putorius furo |
| I1K484_SOYBN/1738-1978 | 8.9e-77 | Glycine max |
| A0A074YYZ2_9TREM/116-378 | 6.9e-77 | Opisthorchis viverrini |
| F6S8G4_XENTR/90-341 | 7.2e-77 | Xenopus tropicalis |
| A0A0D9R5U5_CHLSB/151-402 | 1.8e-75 | Chlorocebus sabaeus |
| A0A087QYR8_APTFO/94-393 | 4.5e-76 | Aptenodytes forsteri |
| A0A096MDV5_POEFO/75-326 | 8.2e-77 | Poecilia formosa |
| H0V233_CAVPO/85-336 | 6.8e-76 | Cavia porcellus |
| K7FYC7_PELSI/93-263 | 7.1e-50 | Pelodiscus sinensis |
| K7FYC7_PELSI/314-392 | 4.6e-21 | Pelodiscus sinensis |
| G3NLU3_GASAC/78-329 | 8.2e-77 | Gasterosteus aculeatus |
| Q762C8_HUMAN/14-183 | 4.9e-51 | Homo sapiens |
| Q762C8_HUMAN/235-312 | 5.7e-20 | Homo sapiens |
| C3Y6P6_BRAFL/56-325 | 8.4e-77 | Branchiostoma floridae |
| A0A093H2I4_TYTAL/94-393 | 9.4e-76 | Tyto alba |
| H2VAL5_TAKRU/100-351 | 1.1e-76 | Takifugu rubripes |
| A0A091TSX4_PHALP/94-393 | 7.6e-76 | Phaethon lepturus |
| G3ILW1_CRIGR/85-336 | 9.3e-77 | Cricetulus griseus |
| CAN1_PONAB/85-336 | 3.3e-75 | Pongo abelii |
| U6DJ14_NEOVI/1-246 | 9.2e-77 | Neovison vison |
| A0A091DR68_FUKDA/135-307 | 1.4e-52 | Fukomys damarensis |
| A0A091DR68_FUKDA/357-435 | 3.1e-18 | Fukomys damarensis |
| U6JR38_ECHGR/117-384 | 9.6e-77 | Echinococcus granulosus |
| G1SJ23_RABIT/85-336 | 1.9e-75 | Oryctolagus cuniculus |
| L9JC24_TUPCH/173-345 | 4.1e-53 | Tupaia chinensis |
| L9JC24_TUPCH/418-522 | 9.7e-18 | Tupaia chinensis |
| D2SSQ4_NEPNO/109-369 | 9.3e-77 | Nephrops norvegicus |
| E7FFQ8_DANRE/80-331 | 1.1e-76 | Danio rerio |
| W5N9G2_LEPOC/51-327 | 9.5e-77 | Lepisosteus oculatus |
| F1QI24_DANRE/80-331 | 1.1e-76 | Danio rerio |
| Q7ZUR1_DANRE/80-331 | 1.1e-76 | Danio rerio |
| G3QXN8_GORGO/85-336 | 3.3e-75 | Gorilla gorilla gorilla |
| H2Q425_PANTR/85-336 | 3.3e-75 | Pan troglodytes |
| G5BM28_HETGA/99-271 | 5.3e-52 | Heterocephalus glaber |
| G5BM28_HETGA/321-399 | 1.0e-18 | Heterocephalus glaber |
| G3S9I8_GORGO/85-336 | 3.3e-75 | Gorilla gorilla gorilla |
| K7CEN7_PANTR/85-336 | 3.3e-75 | Pan troglodytes |
| A0A093IP99_FULGA/57-309 | 1.2e-76 | Fulmarus glacialis |
| H2NCW8_PONAB/85-336 | 3.3e-75 | Pongo abelii |
| A0A093IHI2_PICPB/93-262 | 1.7e-49 | Picoides pubescens |
| A0A093IHI2_PICPB/313-392 | 2.6e-21 | Picoides pubescens |
| K7FYT0_PELSI/93-263 | 9.2e-50 | Pelodiscus sinensis |
| K7FYT0_PELSI/314-392 | 5.6e-21 | Pelodiscus sinensis |
| M3VWM5_FELCA/85-336 | 1.5e-75 | Felis catus |
| D2IKJ7_HIPHI/83-334 | 1.2e-76 | Hippoglossus hippoglossus |
| V8NX99_OPHHA/946-1195 | 1.6e-76 | Ophiophagus hannah |
| A0A096MRQ5_PAPAN/75-326 | 1.1e-76 | Papio anubis |
| T1EFK4_HELRO/54-329 | 1.6e-76 | Helobdella robusta |
| H2VAL1_TAKRU/100-354 | 2.0e-76 | Takifugu rubripes |
| H2VAL4_TAKRU/100-354 | 1.9e-76 | Takifugu rubripes |
| H2VAL0_TAKRU/100-354 | 2.0e-76 | Takifugu rubripes |
| H2VAK8_TAKRU/100-354 | 2.0e-76 | Takifugu rubripes |
| A5PMP1_DANRE/78-329 | 1.6e-76 | Danio rerio |
| A0A091E012_FUKDA/93-344 | 7.1e-76 | Fukomys damarensis |
| W5UC05_ICTPU/78-329 | 1.6e-76 | Ictalurus punctatus |
| H0VJZ2_CAVPO/67-319 | 1.6e-76 | Cavia porcellus |
| A9JT02_DANRE/75-324 | 1.6e-76 | Danio rerio |
| A0A093GXP5_STRCA/44-295 | 1.8e-76 | Struthio camelus australis |
| A0A091XIF8_OPIHO/75-326 | 2.1e-76 | Opisthocomus hoazin |
| Q9I8G2_DANRE/80-331 | 1.9e-76 | Danio rerio |
| A0A098M1D6_9SAUR/85-336 | 1.1e-74 | Hypsiglena sp. JMG-2014 |
| Q3HV56_SANVI/75-327 | 1.8e-76 | Sander vitreus vitreus |
| G3VKY8_SARHA/85-336 | 2.3e-76 | Sarcophilus harrisii |
| A0A068XZA3_ECHMU/117-384 | 2.0e-76 | Echinococcus multilocularis |
| B1B154_HUMAN/75-326 | 1.9e-76 | Homo sapiens |
| E7FBY5_DANRE/75-324 | 2.2e-76 | Danio rerio |
| I3JFK9_ORENI/63-233 | 1.3e-52 | Oreochromis niloticus |
| I3JFK9_ORENI/266-364 | 7.9e-18 | Oreochromis niloticus |
| H2VAK9_TAKRU/100-358 | 2.9e-76 | Takifugu rubripes |
| H0XI10_OTOGA/85-336 | 1.4e-75 | Otolemur garnettii |
| A5PMN9_DANRE/75-324 | 2.2e-76 | Danio rerio |
| U5YRM2_SPAAU/81-332 | 2.3e-76 | Sparus aurata |
| B7ZV15_DANRE/75-324 | 2.3e-76 | Danio rerio |
| H0WZX4_OTOGA/75-326 | 5.7e-76 | Otolemur garnettii |
| Q4RQ30_TETNG/73-324 | 2.7e-76 | Tetraodon nigroviridis |
| H3DGT9_TETNG/84-335 | 2.7e-76 | Tetraodon nigroviridis |
| W5UKL8_ICTPU/75-326 | 2.7e-76 | Ictalurus punctatus |
| H2VAL2_TAKRU/100-353 | 3.6e-76 | Takifugu rubripes |
| H2VAL3_TAKRU/100-353 | 3.6e-76 | Takifugu rubripes |
| G1SDE0_RABIT/67-319 | 2.9e-76 | Oryctolagus cuniculus |
| A0A091GN64_9AVES/75-326 | 3.3e-76 | Cuculus canorus |
| A0A087YGJ4_POEFO/75-326 | 2.9e-76 | Poecilia formosa |
| A0A096LXI1_POEFO/80-331 | 2.9e-76 | Poecilia formosa |
| H0WM10_OTOGA/67-319 | 3.1e-76 | Otolemur garnettii |
| L8IQY6_9CETA/85-336 | 8.0e-76 | Bos mutus |
| I3KRY8_ORENI/51-326 | 3.5e-76 | Oreochromis niloticus |
| CAN1_BOVIN/85-336 | 8.0e-76 | Bos taurus |
| A1Z1N7_BOSMU/85-336 | 8.0e-76 | Bos mutus grunniens |
| B1B153_HUMAN/75-326 | 2.8e-76 | Homo sapiens |
| A9T6E2_PHYPA/1754-2000 | 3.5e-76 | Physcomitrella patens subsp. patens |
| W5NF66_LEPOC/75-326 | 3.3e-76 | Lepisosteus oculatus |
| W5NF65_LEPOC/75-326 | 3.3e-76 | Lepisosteus oculatus |
| A0A093PMH9_9PASS/75-326 | 3.3e-76 | Manacus vitellinus |
| A0A093DRG0_TAUER/41-292 | 3.2e-76 | Tauraco erythrolophus |
| L8Y487_TUPCH/75-326 | 3.4e-76 | Tupaia chinensis |
| A4I2N5_LEIIN/49-273 | 1.6e-25 | Leishmania infantum |
| A4I2N5_LEIIN/670-892 | 3.7e-25 | Leishmania infantum |
| A4I2N5_LEIIN/5408-5612 | 1.1e-13 | Leishmania infantum |
| G3WZT3_SARHA/67-319 | 3.8e-76 | Sarcophilus harrisii |
| Q4VBH3_DANRE/75-326 | 3.8e-76 | Danio rerio |
| A0A091D2Z4_FUKDA/75-326 | 3.6e-76 | Fukomys damarensis |
| E7FA31_DANRE/80-329 | 4.1e-76 | Danio rerio |
| Q80Y25_MOUSE/85-336 | 7.7e-76 | Mus musculus |
| M3WJ14_FELCA/67-319 | 7.7e-76 | Felis catus |
| A0A093L1C2_EURHL/67-319 | 4.0e-76 | Eurypyga helias |
| Q3TB79_MOUSE/85-336 | 8.0e-76 | Mus musculus |
| G3NMK9_GASAC/78-329 | 4.0e-76 | Gasterosteus aculeatus |
| L5KPW1_PTEAL/85-336 | 1.1e-75 | Pteropus alecto |
| CAN1_MOUSE/85-336 | 8.4e-76 | Mus musculus |
| G3NML5_GASAC/86-337 | 4.1e-76 | Gasterosteus aculeatus |
| H3CDE4_TETNG/36-291 | 6.5e-76 | Tetraodon nigroviridis |
| A0A0B7B6M7_9EUPU/53-323 | 4.6e-76 | Arion vulgaris |
| G3SKW9_GORGO/75-326 | 4.0e-76 | Gorilla gorilla gorilla |
| M7AZA5_CHEMY/30-281 | 4.3e-76 | Chelonia mydas |
| H3CDE3_TETNG/39-294 | 6.7e-76 | Tetraodon nigroviridis |
| H3C0K2_TETNG/37-292 | 6.7e-76 | Tetraodon nigroviridis |
| G1KC29_ANOCA/75-326 | 8.7e-76 | Anolis carolinensis |
| G3Q8Y1_GASAC/87-338 | 4.5e-76 | Gasterosteus aculeatus |
| U3F907_MICFL/85-336 | 2.7e-74 | Micrurus fulvius |
| I3KRY7_ORENI/51-326 | 4.5e-76 | Oreochromis niloticus |
| CAN3_SHEEP/99-271 | 1.2e-50 | Ovis aries |
| CAN3_SHEEP/322-400 | 1.8e-19 | Ovis aries |
| A0A087R6V3_APTFO/67-319 | 5.8e-76 | Aptenodytes forsteri |
| Q4D6J0_TRYCC/51-277 | 6.4e-27 | Trypanosoma cruzi |
| Q4D6J0_TRYCC/668-890 | 1.1e-22 | Trypanosoma cruzi |
| Q4D6J0_TRYCC/4025-4186 | 1.4e-14 | Trypanosoma cruzi |
| U3BYX7_CALJA/75-326 | 4.9e-76 | Callithrix jacchus |
| F1RG55_PIG/67-319 | 6.5e-76 | Sus scrofa |
| A0A084WJ03_9DIPT/86-347 | 5.2e-76 | Anopheles sinensis |
| A5WWC4_DANRE/75-326 | 5.5e-76 | Danio rerio |
| F1R4X7_DANRE/75-326 | 5.5e-76 | Danio rerio |
| F1DAU3_CAPHI/85-336 | 1.6e-75 | Capra hircus |
| A0A088RTE8_9TRYP/50-276 | 5.8e-27 | Leishmania panamensis |
| A0A088RTE8_9TRYP/669-889 | 2.1e-25 | Leishmania panamensis |
| A0A088RTE8_9TRYP/5577-5763 | 9.7e-12 | Leishmania panamensis |
| W5L2L6_ASTMX/157-408 | 5.6e-76 | Astyanax mexicanus |
| H0X767_OTOGA/102-353 | 6.1e-76 | Otolemur garnettii |
| A0A060VX40_ONCMY/75-324 | 5.7e-76 | Oncorhynchus mykiss |
| W5PVG1_SHEEP/98-349 | 1.7e-75 | Ovis aries |
| K9IMG8_DESRO/85-336 | 1.2e-75 | Desmodus rotundus |
| M3YLA6_MUSPF/85-336 | 1.6e-75 | Mustela putorius furo |
| Q9N0M7_PIG/85-336 | 1.6e-75 | Sus scrofa |
| F1LS29_RAT/85-336 | 1.3e-75 | Rattus norvegicus |
| CAN1_RAT/85-336 | 1.3e-75 | Rattus norvegicus |
| F7F719_MONDO/76-327 | 6.9e-76 | Monodelphis domestica |
| F6TJS4_XENTR/85-338 | 6.9e-75 | Xenopus tropicalis |
| A0A096N3H3_PAPAN/75-326 | 7.2e-76 | Papio anubis |
| A0A091EI09_CORBR/43-294 | 7.3e-76 | Corvus brachyrhynchos |
| L9KQQ3_TUPCH/307-558 | 1.3e-75 | Tupaia chinensis |
| G3Q6L8_GASAC/51-324 | 7.3e-76 | Gasterosteus aculeatus |
| H3CN15_TETNG/51-322 | 7.3e-76 | Tetraodon nigroviridis |
| M3XXX1_MUSPF/77-330 | 6.9e-76 | Mustela putorius furo |
| G1RSC4_NOMLE/75-326 | 7.3e-76 | Nomascus leucogenys |
| D2HJD9_AILME/75-326 | 1.5e-75 | Ailuropoda melanoleuca |
| W5L2R3_ASTMX/67-317 | 8.1e-76 | Astyanax mexicanus |
| W5NF60_LEPOC/75-324 | 8.2e-76 | Lepisosteus oculatus |
| G3WPB8_SARHA/77-328 | 9.6e-76 | Sarcophilus harrisii |
| Q5TYH7_ANOGA/39-301 | 7.2e-76 | Anopheles gambiae |
| W5NF61_LEPOC/81-330 | 8.6e-76 | Lepisosteus oculatus |
| CAN1_PIG/85-336 | 2.0e-75 | Sus scrofa |
| CAN8_HUMAN/75-326 | 8.6e-76 | Homo sapiens |
| G1MBH6_AILME/85-336 | 1.6e-75 | Ailuropoda melanoleuca |
| Q6DFZ8_DANRE/75-326 | 8.8e-76 | Danio rerio |
| A8WH62_XENLA/64-315 | 9.5e-76 | Xenopus laevis |
| A0A091NLU1_APAVI/75-326 | 9.5e-76 | Apaloderma vittatum |
| G1N8W2_MELGA/89-340 | 1.0e-75 | Meleagris gallopavo |
| A0A0B1TMF9_OESDE/229-499 | 1.3e-75 | Oesophagostomum dentatum |
| U3D7R8_CALJA/75-326 | 1.2e-75 | Callithrix jacchus |
| H2M898_ORYLA/84-337 | 1.2e-75 | Oryzias latipes |
| F7A7W5_HORSE/77-328 | 1.2e-75 | Equus caballus |
| F7BLU8_HORSE/75-326 | 1.2e-75 | Equus caballus |
| CAN2_BOVIN/75-326 | 1.4e-75 | Bos taurus |
| W5K7N0_ASTMX/75-324 | 1.3e-75 | Astyanax mexicanus |
| Q561U0_DANRE/75-326 | 1.3e-75 | Danio rerio |
| Q4RQ27_TETNG/75-332 | 1.3e-75 | Tetraodon nigroviridis |
| G7Y893_CLOSI/116-378 | 1.6e-75 | Clonorchis sinensis |
| U3KBJ6_FICAL/75-326 | 1.2e-74 | Ficedula albicollis |
| G3QYC6_GORGO/75-326 | 1.6e-75 | Gorilla gorilla gorilla |
| G7MEH1_MACMU/29-280 | 1.6e-75 | Macaca mulatta |
| H2YVE7_CIOSA/41-300 | 1.6e-75 | Ciona savignyi |
| Q5R1Y9_ONCMY/75-324 | 1.6e-75 | Oncorhynchus mykiss |
| I3M2A0_SPETR/67-319 | 1.6e-75 | Spermophilus tridecemlineatus |
| U3JT66_FICAL/93-392 | 2.5e-74 | Ficedula albicollis |
| H2Q170_PANTR/75-326 | 1.7e-75 | Pan troglodytes |
| E0VHV1_PEDHC/45-302 | 1.5e-75 | Pediculus humanus subsp. corporis |
| CAN2_HUMAN/75-326 | 1.7e-75 | Homo sapiens |
| I3JJX0_ORENI/75-323 | 1.7e-75 | Oreochromis niloticus |
| G3RDP2_GORGO/104-355 | 1.8e-75 | Gorilla gorilla gorilla |
| Q504F5_DANRE/75-324 | 1.6e-75 | Danio rerio |
| G5B6L3_HETGA/85-336 | 2.1e-75 | Heterocephalus glaber |
| T1EM95_HELRO/63-312 | 1.7e-75 | Helobdella robusta |
| Q3UDG8_MOUSE/85-336 | 3.6e-75 | Mus musculus |
| F6TJJ5_MACMU/75-326 | 1.8e-75 | Macaca mulatta |
| K1RSB8_CRAGI/50-318 | 1.8e-75 | Crassostrea gigas |
| CAN2_MACFA/75-326 | 1.9e-75 | Macaca fascicularis |
| H9EQA5_MACMU/75-326 | 1.9e-75 | Macaca mulatta |
| A0A0D9RUD1_CHLSB/75-326 | 1.9e-75 | Chlorocebus sabaeus |
| A0A091M5H3_CARIC/75-326 | 2.0e-75 | Cariama cristata |
| F7CP55_XENTR/78-329 | 2.0e-75 | Xenopus tropicalis |
| A0A091TQH2_PHALP/33-284 | 2.1e-75 | Phaethon lepturus |
| R7TWH9_CAPTE/61-306 | 1.8e-75 | Capitella teleta |
| A8IF71_SHEEP/75-326 | 2.5e-75 | Ovis aries |
| H0W1N7_CAVPO/1-168 | 3.3e-50 | Cavia porcellus |
| H0W1N7_CAVPO/218-296 | 3.1e-19 | Cavia porcellus |
| J3S8A4_CROAD/75-326 | 4.2e-75 | Crotalus adamanteus |
| Q8BPV9_MOUSE/75-326 | 2.1e-75 | Mus musculus |
| A0A091R3K3_MERNU/56-307 | 2.3e-75 | Merops nubicus |
| E7FDN3_DANRE/51-325 | 2.3e-75 | Danio rerio |
| S7PEY8_MYOBR/85-336 | 3.4e-75 | Myotis brandtii |
| G5BQI6_HETGA/67-319 | 2.3e-75 | Heterocephalus glaber |
| A0A093GFF0_PICPB/75-326 | 2.4e-75 | Picoides pubescens |
| D2IKJ6_HIPHI/78-329 | 2.5e-75 | Hippoglossus hippoglossus |
| I3JJT7_ORENI/85-336 | 2.6e-75 | Oreochromis niloticus |
| B6V3H8_CHICK/75-326 | 1.3e-74 | Gallus gallus |
| U3KBV3_FICAL/78-329 | 2.6e-75 | Ficedula albicollis |
| D8SWW6_SELML/1656-1898 | 2.9e-75 | Selaginella moellendorffii |
| D8T469_SELML/1672-1914 | 3.0e-75 | Selaginella moellendorffii |
| A0A093CE25_9AVES/75-326 | 3.1e-75 | Pterocles gutturalis |
| K7G2C5_PELSI/75-326 | 3.3e-75 | Pelodiscus sinensis |
| A0A0A0A048_CHAVO/24-275 | 3.0e-75 | Charadrius vociferus |
| E1BR22_CHICK/75-326 | 1.3e-74 | Gallus gallus |
| H0V2U3_CAVPO/60-328 | 3.6e-75 | Cavia porcellus |
| CAN2_CHICK/75-326 | 1.3e-74 | Gallus gallus |
| B7FWX6_PHATC/320-608 | 3.6e-75 | Phaeodactylum tricornutum |
| A0A060X593_ONCMY/39-309 | 3.7e-75 | Oncorhynchus mykiss |
| A0A091DUT6_FUKDA/91-343 | 3.4e-75 | Fukomys damarensis |
| A0A093RBR2_PHACA/60-311 | 3.7e-75 | Phalacrocorax carbo |
| Q3U8S5_MOUSE/75-326 | 3.9e-75 | Mus musculus |
| CAN2_MOUSE/75-326 | 3.9e-75 | Mus musculus |
| Q3U3A8_MOUSE/75-326 | 3.9e-75 | Mus musculus |
| A0A093BNS1_TAUER/2-296 | 3.9e-74 | Tauraco erythrolophus |
| A0A091NDB5_9PASS/3-247 | 1.1e-73 | Acanthisitta chloris |
| R7UWC1_CAPTE/653-878 | 1.9e-57 | Capitella teleta |
| R7UWC1_CAPTE/1005-1056 | 9.0e-12 | Capitella teleta |
| A0A093NPM1_PYGAD/75-326 | 4.2e-75 | Pygoscelis adeliae |
| A8KC46_DANRE/75-326 | 4.3e-75 | Danio rerio |
| A6YNL5_PIG/75-326 | 6.0e-75 | Sus scrofa |
| C3YB23_BRAFL/32-294 | 4.5e-75 | Branchiostoma floridae |
| A0A091JBY5_9AVES/75-326 | 5.2e-75 | Egretta garzetta |
| M3XXY5_MUSPF/205-456 | 9.3e-75 | Mustela putorius furo |
| U3JLJ3_FICAL/67-237 | 3.9e-46 | Ficedula albicollis |
| U3JLJ3_FICAL/279-362 | 7.5e-23 | Ficedula albicollis |
| V8NKS8_OPHHA/75-326 | 5.9e-75 | Ophiophagus hannah |
| J9LGL6_ACYPI/59-311 | 7.5e-75 | Acyrthosiphon pisum |
| G1N9J6_MELGA/12-263 | 1.0e-74 | Meleagris gallopavo |
| CAN2_RAT/75-326 | 7.8e-75 | Rattus norvegicus |
| A0A087QRJ8_APTFO/75-326 | 6.4e-75 | Aptenodytes forsteri |
| F1P052_CHICK/75-324 | 6.1e-75 | Gallus gallus |
| H0YWP3_TAEGU/77-328 | 7.6e-75 | Taeniopygia guttata |
| W5JMN9_ANODA/101-364 | 7.8e-75 | Anopheles darlingi |
| F6ZUN8_CALJA/67-319 | 8.1e-75 | Callithrix jacchus |
| A0A094L5Q8_9AVES/53-304 | 8.0e-75 | Podiceps cristatus |
| A0A0F5D196_PRIPA/56-337 | 7.4e-75 | Pristionchus pacificus |
| A0A093BWQ7_9AVES/3-247 | 8.4e-74 | Pterocles gutturalis |
| Q8C2J1_MOUSE/85-336 | 1.8e-74 | Mus musculus |
| R0LRI5_ANAPL/2-251 | 1.3e-74 | Anas platyrhynchos |
| C0HAF6_SALSA/80-331 | 9.9e-75 | Salmo salar |
| U3IQF8_ANAPL/2-251 | 1.4e-74 | Anas platyrhynchos |
| A0A077Z2M0_TRITR/49-328 | 1.2e-74 | Trichuris trichiura |
| A0A091WVY0_NIPNI/71-322 | 1.0e-74 | Nipponia nippon |
| B2ZA85_SHEEP/85-336 | 3.2e-74 | Ovis aries |
| A0A091KR10_9GRUI/75-326 | 1.0e-74 | Chlamydotis macqueenii |
| J0XG50_LOALO/50-325 | 1.3e-74 | Loa loa |
| H0W2Y7_CAVPO/75-326 | 1.3e-74 | Cavia porcellus |
| T1E719_CROHD/85-336 | 3.5e-73 | Crotalus horridus |
| J3S431_CROAD/85-336 | 3.5e-73 | Crotalus adamanteus |
| G3P5S8_GASAC/69-322 | 1.9e-74 | Gasterosteus aculeatus |
| G3P5U0_GASAC/72-325 | 1.9e-74 | Gasterosteus aculeatus |
| C1LGC2_SCHJA/80-339 | 1.7e-74 | Schistosoma japonicum |
| U3IC53_ANAPL/49-300 | 1.6e-74 | Anas platyrhynchos |
| C0H9F2_SALSA/94-340 | 2.2e-74 | Salmo salar |
| G1KRC7_ANOCA/93-263 | 7.0e-49 | Anolis carolinensis |
| G1KRC7_ANOCA/311-388 | 1.2e-19 | Anolis carolinensis |
| H2T9A5_TAKRU/78-329 | 2.0e-74 | Takifugu rubripes |
| H2T9A3_TAKRU/78-329 | 2.0e-74 | Takifugu rubripes |
| H2T9A7_TAKRU/86-337 | 2.0e-74 | Takifugu rubripes |
| H2T9A6_TAKRU/86-337 | 2.0e-74 | Takifugu rubripes |
| H2T9A8_TAKRU/85-336 | 2.0e-74 | Takifugu rubripes |
| A0A091F4A2_CORBR/44-293 | 1.7e-74 | Corvus brachyrhynchos |
| A0A068WYE1_ECHGR/120-374 | 2.7e-74 | Echinococcus granulosus |
| F6SU04_HORSE/85-334 | 5.3e-73 | Equus caballus |
| A0A090MTS7_STRRB/56-333 | 2.3e-74 | Strongyloides ratti |
| G3NNP2_GASAC/48-281 | 2.5e-74 | Gasterosteus aculeatus |
| H2N3F0_PONAB/67-317 | 2.3e-74 | Pongo abelii |
| A0A0B8RSA7_BOIIR/85-336 | 1.3e-72 | Boiga irregularis |
| F1PMS4_CANFA/14-262 | 3.7e-74 | Canis familiaris |
| Q59EF6_HUMAN/104-355 | 2.3e-74 | Homo sapiens |
| D2IKJ8_HIPHI/75-323 | 2.7e-74 | Hippoglossus hippoglossus |
| Q7PZT1_ANOGA/94-356 | 2.6e-74 | Anopheles gambiae |
| A0A044SLC1_ONCVO/209-469 | 2.7e-74 | Onchocerca volvulus |
| F1P975_CANFA/45-292 | 5.4e-74 | Canis familiaris |
| H9GNF9_ANOCA/85-336 | 1.0e-73 | Anolis carolinensis |
| U3EU26_MICFL/78-329 | 3.0e-74 | Micrurus fulvius |
| G7YXL6_CLOSI/191-455 | 2.9e-74 | Clonorchis sinensis |
| K1RCZ2_CRAGI/126-394 | 3.4e-74 | Crassostrea gigas |
| W5NI00_LEPOC/75-326 | 3.3e-74 | Lepisosteus oculatus |
| W5NHZ8_LEPOC/75-326 | 3.4e-74 | Lepisosteus oculatus |
| H2S156_TAKRU/60-287 | 4.0e-74 | Takifugu rubripes |
| F6YA81_XENTR/67-320 | 3.5e-74 | Xenopus tropicalis |
| F6STM7_XENTR/86-360 | 3.5e-74 | Xenopus tropicalis |
| G1TKY3_RABIT/96-347 | 4.7e-74 | Oryctolagus cuniculus |
| A0A091NR85_APAVI/44-292 | 3.2e-74 | Apaloderma vittatum |
| G1K9E5_ANOCA/78-329 | 3.9e-74 | Anolis carolinensis |
| V4AV81_LOTGI/32-292 | 2.0e-73 | Lottia gigantea |
| A8Y0F3_CAEBR/51-333 | 4.4e-74 | Caenorhabditis briggsae |
| A0A091LWA5_CARIC/2-249 | 4.4e-74 | Cariama cristata |
| G3P5W5_GASAC/73-245 | 3.0e-51 | Gasterosteus aculeatus |
| G3P5W5_GASAC/278-366 | 6.8e-17 | Gasterosteus aculeatus |
| K7FM40_PELSI/85-336 | 1.0e-73 | Pelodiscus sinensis |
| G4V6C0_SCHMA/80-339 | 4.6e-74 | Schistosoma mansoni |
| A0A068YBR8_ECHMU/120-374 | 6.5e-74 | Echinococcus multilocularis |
| O42133_CHICK/85-336 | 5.5e-74 | Gallus gallus |
| G1KBL7_ANOCA/83-375 | 3.9e-72 | Anolis carolinensis |
| E9AYV2_LEIMU/47-274 | 1.2e-25 | Leishmania mexicana |
| E9AYV2_LEIMU/666-886 | 4.2e-24 | Leishmania mexicana |
| E9AYV2_LEIMU/3748-3934 | 2.0e-12 | Leishmania mexicana |
| A0A099ZYL7_CHAVO/44-293 | 4.6e-74 | Charadrius vociferus |
| A0A093FXX0_TYTAL/53-304 | 5.8e-74 | Tyto alba |
| A0A093EUM4_TAUER/44-292 | 4.7e-74 | Tauraco erythrolophus |
| G7MEH2_MACMU/75-325 | 5.5e-74 | Macaca mulatta |
| H3CJC4_TETNG/75-325 | 6.1e-74 | Tetraodon nigroviridis |
| F1R898_DANRE/75-324 | 5.9e-74 | Danio rerio |
| A0A093BSX3_CHAPE/73-324 | 5.5e-74 | Chaetura pelagica |
| R7UUX7_CAPTE/42-295 | 5.9e-74 | Capitella teleta |
| T1DNM9_CROHD/42-288 | 1.6e-73 | Crotalus horridus |
| U3J373_ANAPL/41-211 | 3.8e-46 | Anas platyrhynchos |
| U3J373_ANAPL/252-332 | 7.3e-22 | Anas platyrhynchos |
| E9AD26_LEIMA/49-273 | 6.6e-25 | Leishmania major |
| E9AD26_LEIMA/669-892 | 9.1e-24 | Leishmania major |
| E9AD26_LEIMA/3979-4184 | 2.2e-13 | Leishmania major |
| A0A075AB26_9TREM/140-404 | 7.1e-74 | Opisthorchis viverrini |
| G7NTW8_MACFA/75-325 | 8.6e-74 | Macaca fascicularis |
| M1FS51_ICTPU/73-322 | 9.9e-74 | Ictalurus punctatus |
| L8I222_9CETA/27-273 | 1.1e-73 | Bos mutus |
| K1R5E8_CRAGI/121-368 | 1.0e-73 | Crassostrea gigas |
| K7GG17_PELSI/78-329 | 1.1e-73 | Pelodiscus sinensis |
| T1E6A5_CROHD/78-329 | 1.2e-73 | Crotalus horridus |
| G0N6W6_CAEBE/53-335 | 1.4e-73 | Caenorhabditis brenneri |
| M7B962_CHEMY/72-324 | 1.2e-73 | Chelonia mydas |
| Q5BLH5_DANRE/75-324 | 1.2e-73 | Danio rerio |
| H9GI64_ANOCA/74-329 | 1.6e-73 | Anolis carolinensis |
| A0A0B2V0G8_TOXCA/50-325 | 1.3e-73 | Toxocara canis |
| B6V3H7_CHICK/78-329 | 1.3e-73 | Gallus gallus |
| A0A093ND15_PYGAD/44-293 | 1.1e-73 | Pygoscelis adeliae |
| CANX_CHICK/78-329 | 1.3e-73 | Gallus gallus |
| G1NEF6_MELGA/83-334 | 1.3e-73 | Meleagris gallopavo |
| M7ANZ1_CHEMY/78-329 | 1.4e-73 | Chelonia mydas |
| B2RCM3_HUMAN/75-326 | 1.4e-73 | Homo sapiens |
| H2W7C9_CAEJA/54-336 | 1.6e-73 | Caenorhabditis japonica |
| B4DN77_HUMAN/2-248 | 1.5e-73 | Homo sapiens |
| A4ZZ65_CHICK/78-329 | 1.4e-73 | Gallus gallus |
| H2T6V8_TAKRU/58-228 | 7.7e-48 | Takifugu rubripes |
| H2T6V8_TAKRU/283-358 | 1.0e-19 | Takifugu rubripes |
| F6XC76_XENTR/46-298 | 1.5e-73 | Xenopus tropicalis |
| M7AKZ7_CHEMY/85-336 | 2.1e-73 | Chelonia mydas |
| A0A084WJ02_9DIPT/99-361 | 2.0e-73 | Anopheles sinensis |
| E3N746_CAERE/53-335 | 2.3e-73 | Caenorhabditis remanei |
| G3MR51_9ACAR/68-317 | 1.9e-73 | Amblyomma maculatum |
| G3TSC4_LOXAF/2-249 | 2.0e-73 | Loxodonta africana |
| M4A315_XIPMA/75-323 | 1.9e-73 | Xiphophorus maculatus |
| J9NUY5_CANFA/99-323 | 1.7e-73 | Canis familiaris |
| G3UKD2_LOXAF/2-249 | 2.0e-73 | Loxodonta africana |
| H0Z2N5_TAEGU/44-293 | 1.7e-73 | Taeniopygia guttata |
| A0A091QU76_MERNU/44-293 | 1.7e-73 | Merops nubicus |
| A0A068XBD0_HYMMI/359-626 | 2.3e-73 | Hymenolepis microstoma |
| A0A091IE01_CALAN/73-324 | 1.9e-73 | Calypte anna |
| A0A093GV14_STRCA/44-292 | 1.7e-73 | Struthio camelus australis |
| S7MU25_MYOBR/1-247 | 2.7e-73 | Myotis brandtii |
| R0KNG7_ANAPL/46-296 | 2.3e-73 | Anas platyrhynchos |
| A0A061I368_CRIGR/60-307 | 3.2e-73 | Cricetulus griseus |
| W5L346_ASTMX/79-249 | 6.2e-46 | Astyanax mexicanus |
| W5L346_ASTMX/295-378 | 1.6e-21 | Astyanax mexicanus |
| A0A087YL00_POEFO/75-323 | 3.1e-73 | Poecilia formosa |
| Q8AVE8_XENLA/75-325 | 2.5e-73 | Xenopus laevis |
| A0A060XE50_ONCMY/62-315 | 2.5e-73 | Oncorhynchus mykiss |
| A0A087RE86_APTFO/44-293 | 2.2e-73 | Aptenodytes forsteri |
| V5GUD5_ANOGL/44-298 | 2.9e-73 | Anoplophora glabripennis |
| H3CHJ6_TETNG/103-351 | 2.9e-73 | Tetraodon nigroviridis |
| A8PPC0_BRUMA/51-326 | 3.3e-73 | Brugia malayi |
| A0A091H4J1_BUCRH/44-293 | 2.8e-73 | Buceros rhinoceros silvestris |
| A0A095CCW5_SCHHA/80-339 | 3.4e-73 | Schistosoma haematobium |
| A0A023GM04_9ACAR/68-317 | 3.6e-73 | Amblyomma triste |
| A0A091R8W2_9GRUI/78-329 | 3.4e-73 | Mesitornis unicolor |
| M3WDP4_FELCA/75-328 | 3.3e-73 | Felis catus |
| G3NQ89_GASAC/75-325 | 4.0e-73 | Gasterosteus aculeatus |
| G7NTW7_MACFA/2-246 | 4.1e-73 | Macaca fascicularis |
| F6Y3D2_MACMU/44-292 | 4.7e-73 | Macaca mulatta |
| H2U5V4_TAKRU/75-323 | 1.5e-72 | Takifugu rubripes |
| L7M767_9ACAR/67-316 | 4.2e-73 | Rhipicephalus pulchellus |
| H2T9A9_TAKRU/29-275 | 4.6e-73 | Takifugu rubripes |
| A0A084WJ01_9DIPT/110-372 | 4.8e-73 | Anopheles sinensis |
| V5V1L2_CYPCA/75-324 | 4.8e-73 | Cyprinus carpio |
| A0A060WCK1_ONCMY/51-329 | 4.3e-73 | Oncorhynchus mykiss |
| G3S0X4_GORGO/71-324 | 7.4e-73 | Gorilla gorilla gorilla |
| U5YQL7_SPAAU/75-323 | 5.0e-73 | Sparus aurata |
| Q66JB4_XENTR/78-329 | 4.9e-73 | Xenopus tropicalis |
| G3Q947_GASAC/75-329 | 4.9e-73 | Gasterosteus aculeatus |
| G3Q945_GASAC/80-334 | 5.0e-73 | Gasterosteus aculeatus |
| A0A093R479_PHACA/78-329 | 5.2e-73 | Phalacrocorax carbo |
| D2A484_TRICA/98-345 | 5.5e-73 | Tribolium castaneum |
| H2MAH1_ORYLA/59-229 | 2.1e-49 | Oryzias latipes |
| H2MAH1_ORYLA/240-353 | 1.6e-17 | Oryzias latipes |
| Q672G9_ONCMY/75-325 | 5.7e-73 | Oncorhynchus mykiss |
| G3QKK1_GORGO/71-318 | 6.0e-73 | Gorilla gorilla gorilla |
| G3RYI2_GORGO/71-318 | 6.0e-73 | Gorilla gorilla gorilla |
| L7M9U7_9ACAR/67-316 | 5.8e-73 | Rhipicephalus pulchellus |
| H2RBJ8_PANTR/71-318 | 6.0e-73 | Pan troglodytes |
| H2RDS4_PANTR/71-318 | 6.0e-73 | Pan troglodytes |
| Q9YIC1_COTCO/78-329 | 5.8e-73 | Coturnix coturnix |
| A0A091MWN8_APAVI/78-329 | 5.8e-73 | Apaloderma vittatum |
| A0A023FNA0_9ACAR/68-317 | 6.3e-73 | Amblyomma cajennense |
| A0A091W1A9_NIPNI/78-329 | 6.3e-73 | Nipponia nippon |
| A0A093FRR5_GAVST/44-293 | 5.5e-73 | Gavia stellata |
| H2U5V5_TAKRU/75-323 | 6.2e-73 | Takifugu rubripes |
| Q5U594_XENLA/78-329 | 6.7e-73 | Xenopus laevis |
| A0A091EVG4_CORBR/78-329 | 7.2e-73 | Corvus brachyrhynchos |
| A0A093T2K2_9PASS/78-329 | 7.1e-73 | Manacus vitellinus |
| Q6PAF2_XENLA/78-329 | 7.6e-73 | Xenopus laevis |
| H2RCY6_PANTR/109-359 | 7.3e-73 | Pan troglodytes |
| A0A091L627_CATAU/78-331 | 7.4e-73 | Cathartes aura |
| A0A091P1M8_HALAL/44-294 | 6.6e-73 | Haliaeetus albicilla |
| CAN5_CAEEL/53-335 | 8.1e-73 | Caenorhabditis elegans |
| Q4SVI4_TETNG/65-336 | 1.1e-72 | Tetraodon nigroviridis |
| Q8UW96_XENLA/78-329 | 7.8e-73 | Xenopus laevis |
| A0A087YKW2_POEFO/76-327 | 7.9e-73 | Poecilia formosa |
| H0ZWD5_TAEGU/85-336 | 8.0e-73 | Taeniopygia guttata |
| U3K9T7_FICAL/78-329 | 8.1e-73 | Ficedula albicollis |
| M3WC24_FELCA/91-342 | 1.2e-72 | Felis catus |
| A0A096M747_POEFO/87-338 | 8.2e-73 | Poecilia formosa |
| T1IFQ4_RHOPR/60-315 | 9.3e-73 | Rhodnius prolixus |
| A0A093JLS6_EURHL/44-293 | 7.2e-73 | Eurypyga helias |
| U3KA02_FICAL/75-324 | 9.7e-73 | Ficedula albicollis |
| W5NBL4_LEPOC/62-309 | 8.2e-73 | Lepisosteus oculatus |
| W2TPJ0_NECAM/4-277 | 9.8e-73 | Necator americanus |
| A0A096NIK2_PAPAN/103-354 | 8.3e-73 | Papio anubis |
| A0A060YF89_ONCMY/75-325 | 9.0e-73 | Oncorhynchus mykiss |
| D6W573_HUMAN/71-318 | 1.1e-72 | Homo sapiens |
| CAN14_HUMAN/71-318 | 1.1e-72 | Homo sapiens |
| V9KFX7_CALMI/75-326 | 1.1e-72 | Callorhinchus milii |
| E1BRR5_CHICK/123-371 | 1.2e-72 | Gallus gallus |
| S4RFJ9_PETMA/3-170 | 6.0e-47 | Petromyzon marinus |
| S4RFJ9_PETMA/230-306 | 8.9e-20 | Petromyzon marinus |
| A0A0A9WMK7_LYGHE/45-304 | 1.2e-72 | Lygus hesperus |
| A0A093S247_9PASS/44-293 | 1.1e-72 | Manacus vitellinus |
| B0DNX2_LACBS/163-441 | 1.4e-72 | Laccaria bicolor |
| A0A087RAR2_APTFO/78-329 | 1.5e-72 | Aptenodytes forsteri |
| R7UD02_CAPTE/44-299 | 1.6e-72 | Capitella teleta |
| CAN11_HUMAN/109-360 | 1.6e-72 | Homo sapiens |
| A0A0C9XXK6_9AGAR/167-445 | 1.5e-72 | Laccaria amethystina LaAM-08-1 |
| H2U5V2_TAKRU/85-333 | 1.6e-72 | Takifugu rubripes |
| H2U5V3_TAKRU/80-328 | 1.6e-72 | Takifugu rubripes |
| A0A0D9RII6_CHLSB/108-359 | 1.7e-72 | Chlorocebus sabaeus |
| A0A091Q7F8_LEPDC/44-293 | 1.4e-72 | Leptosomus discolor |
| A0A060W802_ONCMY/84-253 | 1.3e-47 | Oncorhynchus mykiss |
| A0A060W802_ONCMY/278-366 | 4.9e-19 | Oncorhynchus mykiss |
| G1QUF7_NOMLE/104-355 | 1.8e-72 | Nomascus leucogenys |
| G3UNU1_MELGA/2-172 | 5.8e-48 | Meleagris gallopavo |
| G3UNU1_MELGA/199-300 | 1.5e-18 | Meleagris gallopavo |
| F6UZV8_CANFA/85-336 | 1.9e-71 | Canis familiaris |
| H0VWP6_CAVPO/75-326 | 2.3e-72 | Cavia porcellus |
| U6D156_NEOVI/2-234 | 1.7e-72 | Neovison vison |
| H3AP62_LATCH/46-306 | 1.9e-72 | Latimeria chalumnae |
| W6UGM0_ECHGR/72-322 | 1.6e-72 | Echinococcus granulosus |
| G7N9S1_MACMU/71-318 | 2.5e-72 | Macaca mulatta |
| G7PLZ3_MACFA/71-318 | 2.5e-72 | Macaca fascicularis |
| H2ZIF1_CIOSA/102-354 | 2.5e-72 | Ciona savignyi |
| A0A091GGM5_9AVES/44-293 | 2.1e-72 | Cuculus canorus |
| A0A093GD57_PICPB/78-329 | 2.5e-72 | Picoides pubescens |
| F6VTX5_CALJA/71-318 | 2.8e-72 | Callithrix jacchus |
| A0A091JX81_COLST/78-329 | 2.6e-72 | Colius striatus |
| A0A093RMG7_PYGAD/78-329 | 2.7e-72 | Pygoscelis adeliae |
| A0A091FLK7_9AVES/78-329 | 3.0e-72 | Cuculus canorus |
| G2HI81_PANTR/72-323 | 3.1e-72 | Pan troglodytes |
| F7I3J8_CALJA/71-325 | 3.0e-72 | Callithrix jacchus |
| A0A091I1W0_CALAN/78-329 | 3.1e-72 | Calypte anna |
| A0A093HXF0_STRCA/78-324 | 3.2e-72 | Struthio camelus australis |
| A0A091K271_COLST/44-293 | 2.9e-72 | Colius striatus |
| A0A091WCT8_OPIHO/44-293 | 3.3e-72 | Opisthocomus hoazin |
| H3ASA4_LATCH/44-294 | 3.6e-72 | Latimeria chalumnae |
| G7MPG0_MACMU/72-323 | 4.1e-72 | Macaca mulatta |
| G7P404_MACFA/72-323 | 4.1e-72 | Macaca fascicularis |
| I3J735_ORENI/63-316 | 4.5e-72 | Oreochromis niloticus |
| H0X7K8_OTOGA/108-359 | 4.5e-72 | Otolemur garnettii |
| L8IAN1_9CETA/80-331 | 5.1e-72 | Bos mutus |
| A0A099ZJ81_TINGU/78-330 | 5.1e-72 | Tinamus guttatus |
| M3ZD34_XIPMA/75-325 | 6.2e-72 | Xiphophorus maculatus |
| D2GZA7_AILME/81-332 | 5.9e-72 | Ailuropoda melanoleuca |
| H3C248_TETNG/78-327 | 8.2e-72 | Tetraodon nigroviridis |
| H2T9A2_TAKRU/78-360 | 3.1e-70 | Takifugu rubripes |
| F1P959_CANFA/75-328 | 6.6e-72 | Canis familiaris |
| S7NST1_MYOBR/71-319 | 8.6e-72 | Myotis brandtii |
| H2MXP5_ORYLA/73-323 | 8.1e-72 | Oryzias latipes |
| B7Z467_HUMAN/71-318 | 7.9e-72 | Homo sapiens |
| E5SK75_TRISP/49-328 | 1.1e-71 | Trichinella spiralis |
| M3ZD92_XIPMA/75-325 | 1.1e-71 | Xiphophorus maculatus |
| A0A091EAI4_CORBR/2-249 | 9.6e-72 | Corvus brachyrhynchos |
| T1FYK4_HELRO/42-310 | 1.2e-71 | Helobdella robusta |
| Q4T1A2_TETNG/75-327 | 9.9e-72 | Tetraodon nigroviridis |
| G3VKY7_SARHA/85-253 | 2.4e-46 | Sarcophilus harrisii |
| G3VKY7_SARHA/311-383 | 2.9e-19 | Sarcophilus harrisii |
| A0A091S3U6_NESNO/44-292 | 9.3e-72 | Nestor notabilis |
| H3AP61_LATCH/42-303 | 1.0e-71 | Latimeria chalumnae |
| A0A0D9RFQ8_CHLSB/71-318 | 1.3e-71 | Chlorocebus sabaeus |
| G3Q938_GASAC/75-323 | 1.1e-71 | Gasterosteus aculeatus |
| G3Q932_GASAC/75-323 | 1.2e-71 | Gasterosteus aculeatus |
| L5LPC2_MYODS/71-318 | 1.4e-71 | Myotis davidii |
| G1N1G7_MELGA/75-323 | 1.4e-71 | Meleagris gallopavo |
| G1R0I6_NOMLE/85-335 | 4.4e-70 | Nomascus leucogenys |
| A0A093IIZ5_FULGA/44-293 | 1.1e-71 | Fulmarus glacialis |
| K9HIC0_AGABB/85-361 | 1.6e-71 | Agaricus bisporus var. bisporus |
| H0ZCN4_TAEGU/78-329 | 1.4e-71 | Taeniopygia guttata |
| S9X338_9CETA/67-312 | 1.5e-71 | Camelus ferus |
| T1J9T8_STRMM/793-1030 | 2.5e-71 | Strigamia maritima |
| K5X336_AGABU/85-361 | 1.6e-71 | Agaricus bisporus var. burnettii |
| A0A077YVW3_TRITR/153-398 | 1.5e-71 | Trichuris trichiura |
| A0A099Z2N5_TINGU/44-291 | 1.4e-71 | Tinamus guttatus |
| A0A091TF80_PHALP/4-245 | 1.5e-71 | Phaethon lepturus |
| G1S025_NOMLE/71-318 | 1.9e-71 | Nomascus leucogenys |
| A0A087XXC3_POEFO/75-325 | 2.3e-71 | Poecilia formosa |
| A0A091KWC2_9GRUI/4-245 | 1.8e-71 | Chlamydotis macqueenii |
| Q7ZYF3_XENLA/75-325 | 2.4e-71 | Xenopus laevis |
| A0A091LZA0_CARIC/44-293 | 2.0e-71 | Cariama cristata |
| A0A091ULT9_PHALP/78-329 | 2.4e-71 | Phaethon lepturus |
| A0A023GM01_9ACAR/67-317 | 3.0e-71 | Amblyomma triste |
| A0A023GNR6_9ACAR/67-317 | 3.1e-71 | Amblyomma triste |
| A0A087VQU8_BALRE/78-323 | 2.7e-71 | Balearica regulorum gibbericeps |
| L8INI5_9CETA/69-317 | 3.7e-71 | Bos mutus |
| H3BG10_LATCH/81-332 | 3.0e-71 | Latimeria chalumnae |
| T1GQC8_MEGSC/223-461 | 1.6e-69 | Megaselia scalaris |
| K1PCX8_CRAGI/85-330 | 4.0e-71 | Crassostrea gigas |
| V2Y845_MONRO/94-369 | 4.0e-71 | Moniliophthora roreri |
| A0A094K4K6_ANTCR/44-293 | 3.8e-71 | Antrostomus carolinensis |
| V9K9S9_CALMI/77-330 | 5.5e-71 | Callorhinchus milii |
| E2R821_CANFA/75-323 | 6.6e-71 | Canis familiaris |
| CAN11_RAT/85-336 | 8.0e-71 | Rattus norvegicus |
| H2LQK8_ORYLA/70-323 | 7.5e-71 | Oryzias latipes |
| F7B5Q9_HORSE/77-328 | 7.0e-71 | Equus caballus |
| F7AD60_HORSE/79-330 | 7.0e-71 | Equus caballus |
| A0A0C9XAL5_9AGAR/94-369 | 8.7e-71 | Laccaria amethystina LaAM-08-1 |
| H2TKT9_TAKRU/56-309 | 8.4e-71 | Takifugu rubripes |
| H3ACY1_LATCH/5-254 | 8.4e-71 | Latimeria chalumnae |
| L8HS37_9CETA/75-326 | 7.5e-71 | Bos mutus |
| G1P491_MYOLU/106-356 | 7.9e-71 | Myotis lucifugus |
| H2VAL6_TAKRU/100-329 | 1.2e-70 | Takifugu rubripes |
| H2TKT7_TAKRU/60-313 | 9.2e-71 | Takifugu rubripes |
| L9KS52_TUPCH/72-329 | 8.7e-71 | Tupaia chinensis |
| F1M7U2_RAT/72-323 | 1.0e-70 | Rattus norvegicus |
| H2TKT8_TAKRU/56-309 | 9.0e-71 | Takifugu rubripes |
| D3ZXF4_RAT/85-336 | 1.1e-70 | Rattus norvegicus |
| H2RUW4_TAKRU/60-304 | 9.3e-71 | Takifugu rubripes |
| U6D7H8_NEOVI/2-234 | 8.6e-71 | Neovison vison |
| F6TLE9_MACMU/67-309 | 1.0e-70 | Macaca mulatta |
| A0A091M2T5_CARIC/78-323 | 9.7e-71 | Cariama cristata |
| Q4RRV7_TETNG/67-319 | 9.8e-71 | Tetraodon nigroviridis |
| H2RUW2_TAKRU/83-327 | 1.0e-70 | Takifugu rubripes |
| H2RUW3_TAKRU/81-325 | 1.0e-70 | Takifugu rubripes |
| H3DFF7_TETNG/65-317 | 1.2e-70 | Tetraodon nigroviridis |
| G3MLV0_9ACAR/67-317 | 1.3e-70 | Amblyomma maculatum |
| A0A093HBJ7_TYTAL/44-293 | 1.0e-70 | Tyto alba |
| A0A091RSH6_MERNU/78-339 | 1.2e-70 | Merops nubicus |
| H2RZA3_TAKRU/4-246 | 1.7e-70 | Takifugu rubripes |
| M4AW54_XIPMA/72-325 | 1.3e-70 | Xiphophorus maculatus |
| A0A091UCX6_PHORB/44-293 | 1.1e-70 | Phoenicopterus ruber ruber |
| H2RZA4_TAKRU/5-247 | 1.7e-70 | Takifugu rubripes |
| A0A091VQM9_NIPNI/44-293 | 1.2e-70 | Nipponia nippon |
| A0A091TQ93_9AVES/44-293 | 1.2e-70 | Pelecanus crispus |
| H0XNU9_OTOGA/44-292 | 1.5e-70 | Otolemur garnettii |
| A0A093QGL0_PHACA/44-293 | 1.2e-70 | Phalacrocorax carbo |
| V2XCT2_MONRO/91-366 | 1.4e-70 | Moniliophthora roreri |
| Q1RLS9_DANRE/75-326 | 1.8e-70 | Danio rerio |
| A0A093C634_9AVES/4-245 | 1.4e-70 | Pterocles gutturalis |
| F7EGL4_XENTR/74-323 | 1.9e-70 | Xenopus tropicalis |
| A0A094L7T9_9AVES/4-245 | 1.5e-70 | Podiceps cristatus |
| G1NXK1_MYOLU/71-319 | 1.8e-70 | Myotis lucifugus |
| F1N121_BOVIN/75-326 | 1.8e-70 | Bos taurus |
| Q5TMZ6_ANOGA/89-351 | 1.8e-70 | Anopheles gambiae |
| H2RJN1_TAKRU/76-330 | 2.5e-70 | Takifugu rubripes |
| A0A0B1T7P5_OESDE/2-247 | 1.8e-70 | Oesophagostomum dentatum |
| B0DNX1_LACBS/156-434 | 2.0e-70 | Laccaria bicolor |
| F1MB09_BOVIN/71-318 | 2.5e-70 | Bos taurus |
| H2RJN2_TAKRU/76-330 | 2.6e-70 | Takifugu rubripes |
| H2RJM4_TAKRU/80-334 | 2.6e-70 | Takifugu rubripes |
| H2RJM6_TAKRU/76-330 | 2.6e-70 | Takifugu rubripes |
| A0A091PZC2_HALAL/3-251 | 2.0e-70 | Haliaeetus albicilla |
| M3Z250_MUSPF/75-323 | 2.7e-70 | Mustela putorius furo |
| G7NNI9_MACMU/72-320 | 3.1e-70 | Macaca mulatta |
| E7F6J9_DANRE/75-326 | 2.9e-70 | Danio rerio |
| F1QW40_DANRE/75-326 | 2.9e-70 | Danio rerio |
| S4RSU5_PETMA/91-260 | 1.1e-44 | Petromyzon marinus |
| S4RSU5_PETMA/309-386 | 7.7e-20 | Petromyzon marinus |
| S7MJG2_MYOBR/119-366 | 3.1e-70 | Myotis brandtii |
| A0A087XXL8_POEFO/64-317 | 3.2e-70 | Poecilia formosa |
| A0A096MHY5_POEFO/77-330 | 3.4e-70 | Poecilia formosa |
| A0A091KV00_9GRUI/78-333 | 3.2e-70 | Chlamydotis macqueenii |
| T1IP60_STRMM/353-609 | 3.3e-70 | Strigamia maritima |
| M3WPF6_FELCA/77-331 | 5.3e-70 | Felis catus |
| F7HSF8_MACMU/75-323 | 4.8e-70 | Macaca mulatta |
| F7BNM0_HORSE/3-249 | 3.2e-70 | Equus caballus |
| R0LQS4_ANAPL/65-318 | 3.9e-70 | Anas platyrhynchos |
| I3LD38_PIG/71-319 | 3.9e-70 | Sus scrofa |
| W5PGZ1_SHEEP/79-330 | 4.7e-70 | Ovis aries |
| A0A087Y2K7_POEFO/63-235 | 6.2e-50 | Poecilia formosa |
| A0A087Y2K7_POEFO/251-321 | 2.3e-14 | Poecilia formosa |
| A0A091S5V3_NESNO/78-320 | 4.8e-70 | Nestor notabilis |
| G3NLY9_GASAC/72-300 | 5.3e-70 | Gasterosteus aculeatus |
| L5KRN5_PTEAL/71-318 | 5.4e-70 | Pteropus alecto |
| F1RI38_PIG/75-323 | 5.4e-70 | Sus scrofa |
| G3QB60_GASAC/80-330 | 7.7e-70 | Gasterosteus aculeatus |
| H2RZA5_TAKRU/2-241 | 6.4e-70 | Takifugu rubripes |
| A8I338_CHLRE/118-383 | 6.4e-70 | Chlamydomonas reinhardtii |
| U3INS7_ANAPL/75-323 | 6.3e-70 | Anas platyrhynchos |
| A0A096NZZ9_PAPAN/71-321 | 6.8e-70 | Papio anubis |
| G5AMA9_HETGA/75-323 | 6.5e-70 | Heterocephalus glaber |
| B0DNX0_LACBS/149-422 | 6.4e-70 | Laccaria bicolor |
| H2RZA2_TAKRU/3-240 | 8.0e-70 | Takifugu rubripes |
| W5PZ25_SHEEP/75-327 | 6.8e-70 | Ovis aries |
| H2RZA8_TAKRU/3-240 | 7.8e-70 | Takifugu rubripes |
| K1PHX3_CRAGI/445-697 | 7.5e-70 | Crassostrea gigas |
| H2NYP7_PONAB/75-323 | 8.0e-70 | Pongo abelii |
| D2VKJ8_NAEGR/33-285 | 7.1e-70 | Naegleria gruberi |
| G7YKC1_CLOSI/115-370 | 9.3e-70 | Clonorchis sinensis |
| A0A0D9QVH3_CHLSB/75-323 | 9.1e-70 | Chlorocebus sabaeus |
| F1RQU6_PIG/81-332 | 7.5e-70 | Sus scrofa |
| H2RZA9_TAKRU/3-241 | 8.7e-70 | Takifugu rubripes |
| A0A0C2XKS2_HEBCY/97-373 | 1.2e-69 | Hebeloma cylindrosporum h7 |
| CAN12_HUMAN/75-323 | 9.5e-70 | Homo sapiens |
| A0A087U600_9ARAC/163-417 | 7.2e-70 | Stegodyphus mimosarum |
| G5B8M3_HETGA/53-300 | 8.7e-70 | Heterocephalus glaber |
| F7BHE3_CALJA/75-322 | 9.7e-70 | Callithrix jacchus |
| I3M224_SPETR/75-326 | 8.9e-70 | Spermophilus tridecemlineatus |
| A0A074ZJ10_9TREM/74-329 | 9.5e-70 | Opisthorchis viverrini |
| I3LGE0_PIG/80-323 | 1.0e-69 | Sus scrofa |
| V9KMM3_CALMI/1-228 | 1.2e-69 | Callorhinchus milii |
| G3QKQ1_GORGO/109-360 | 1.2e-69 | Gorilla gorilla gorilla |
| A0A0B7G1C8_THACB/104-377 | 1.1e-69 | Thanatephorus cucumeris |
| H2T141_TAKRU/75-303 | 1.3e-69 | Takifugu rubripes |
| B7PB22_IXOSC/532-773 | 1.6e-69 | Ixodes scapularis |
| M5CF86_THACB/104-377 | 1.3e-69 | Thanatephorus cucumeris |
| I3JDK3_ORENI/81-329 | 1.4e-69 | Oreochromis niloticus |
| H2XSK0_CIOIN/76-317 | 2.2e-69 | Ciona intestinalis |
| CAN11_MOUSE/85-336 | 1.4e-69 | Mus musculus |
| A0A023FKC9_9ACAR/67-317 | 1.3e-69 | Amblyomma cajennense |
| T1G630_HELRO/52-305 | 1.6e-69 | Helobdella robusta |
| A0A091IPF7_9AVES/44-293 | 1.2e-69 | Egretta garzetta |
| G1TDX0_RABIT/85-336 | 1.5e-69 | Oryctolagus cuniculus |
| Q1LUX4_DANRE/58-311 | 1.4e-69 | Danio rerio |
| F1MXQ5_BOVIN/109-362 | 1.6e-69 | Bos taurus |
| A7SE78_NEMVE/90-332 | 1.7e-69 | Nematostella vectensis |
| G1LC77_AILME/77-332 | 1.6e-69 | Ailuropoda melanoleuca |
| W5UDJ5_ICTPU/62-315 | 1.6e-69 | Ictalurus punctatus |
| F1QL66_DANRE/58-311 | 1.6e-69 | Danio rerio |
| Q3B7N8_BOVIN/74-314 | 1.5e-69 | Bos taurus |
| I3JDL2_ORENI/84-338 | 1.7e-69 | Oreochromis niloticus |
| V2WLX8_MONRO/99-374 | 1.7e-69 | Moniliophthora roreri |
| T1IBD5_RHOPR/56-312 | 1.8e-69 | Rhodnius prolixus |
| H0VQJ4_CAVPO/75-323 | 1.7e-69 | Cavia porcellus |
| H0W2G7_CAVPO/73-324 | 1.8e-69 | Cavia porcellus |
| H2L5T8_ORYLA/60-310 | 1.9e-69 | Oryzias latipes |
| S9YSC4_9CETA/75-323 | 2.1e-69 | Camelus ferus |
| H9U0E8_MESVI/2-230 | 1.8e-69 | Mesostigma viride |
| E0V998_PEDHC/1051-1297 | 3.5e-69 | Pediculus humanus subsp. corporis |
| M3VVH9_FELCA/75-323 | 2.1e-69 | Felis catus |
| V4B2J8_LOTGI/33-287 | 1.9e-69 | Lottia gigantea |
| F1MVZ7_BOVIN/74-314 | 2.1e-69 | Bos taurus |
| E2QWM8_CANFA/71-319 | 2.6e-69 | Canis familiaris |
| I3NAY5_SPETR/71-319 | 2.7e-69 | Spermophilus tridecemlineatus |
| A0A091E1K9_FUKDA/178-426 | 4.5e-69 | Fukomys damarensis |
| H2RZA6_TAKRU/2-236 | 3.6e-69 | Takifugu rubripes |
| A0A067PGC3_9HOMO/122-386 | 3.2e-69 | Jaapia argillacea MUCL 33604 |
| E9QL26_MOUSE/75-323 | 3.0e-69 | Mus musculus |
| CAN12_MOUSE/75-323 | 3.0e-69 | Mus musculus |
| G1LZX8_AILME/81-333 | 2.9e-69 | Ailuropoda melanoleuca |
| H2RZA7_TAKRU/2-236 | 3.6e-69 | Takifugu rubripes |
| H2P6K3_PONAB/71-324 | 3.1e-69 | Pongo abelii |
| I3JNF9_ORENI/86-336 | 3.5e-69 | Oreochromis niloticus |
| A7E2G3_DANRE/58-311 | 3.2e-69 | Danio rerio |
| T1L551_TETUR/489-736 | 5.4e-69 | Tetranychus urticae |
| E1BI66_BOVIN/74-314 | 3.8e-69 | Bos taurus |
| L8IQZ0_9CETA/74-314 | 3.8e-69 | Bos mutus |
| L5M104_MYODS/73-316 | 4.1e-69 | Myotis davidii |
| W5QEJ1_SHEEP/81-320 | 5.6e-69 | Ovis aries |
| J9NXF4_CANFA/75-327 | 4.7e-69 | Canis familiaris |
| X8JDC0_9HOMO/104-377 | 4.3e-69 | Rhizoctonia solani AG-3 Rhs1AP |
| U3KA85_FICAL/46-298 | 5.2e-69 | Ficedula albicollis |
| M3YMH6_MUSPF/82-333 | 4.7e-69 | Mustela putorius furo |
| D2V6M1_NAEGR/66-306 | 5.1e-69 | Naegleria gruberi |
| M1FRG3_ICTPU/104-356 | 6.3e-69 | Ictalurus punctatus |
| G1LTR8_AILME/71-319 | 5.4e-69 | Ailuropoda melanoleuca |
| A7RJ83_NEMVE/49-309 | 5.8e-69 | Nematostella vectensis |
| A0A0A0MW93_PAPAN/75-326 | 6.8e-69 | Papio anubis |
| M5C932_THACB/104-377 | 6.1e-69 | Thanatephorus cucumeris |
| F6QAB0_XENTR/82-333 | 6.4e-69 | Xenopus tropicalis |
| X8JBU7_9HOMO/82-351 | 6.8e-69 | Rhizoctonia solani AG-3 Rhs1AP |
| A0A044SXR9_ONCVO/6-266 | 7.5e-69 | Onchocerca volvulus |
| F5XA00_PORGT/1-233 | 6.1e-69 | Porphyromonas gingivalis |
| A0A074RU16_9HOMO/112-388 | 7.7e-69 | Rhizoctonia solani 123E |
| H2RJN0_TAKRU/84-336 | 1.0e-68 | Takifugu rubripes |
| L5LEP2_MYODS/85-325 | 1.3e-68 | Myotis davidii |
| H2RJM3_TAKRU/92-344 | 1.0e-68 | Takifugu rubripes |
| H2RJM5_TAKRU/84-336 | 9.7e-69 | Takifugu rubripes |
| W5LZZ3_LEPOC/83-308 | 7.1e-69 | Lepisosteus oculatus |
| F1Q9Z1_DANRE/117-362 | 1.0e-68 | Danio rerio |
| H2RJM8_TAKRU/84-336 | 1.0e-68 | Takifugu rubripes |
| H2RJM9_TAKRU/84-336 | 1.0e-68 | Takifugu rubripes |
| Q58EB1_DANRE/108-353 | 1.0e-68 | Danio rerio |
| D3ZJZ8_RAT/75-323 | 9.4e-69 | Rattus norvegicus |
| W5K4F4_ASTMX/75-333 | 1.2e-68 | Astyanax mexicanus |
| H2RJM7_TAKRU/84-336 | 1.0e-68 | Takifugu rubripes |
| W5P728_SHEEP/75-326 | 9.0e-69 | Ovis aries |
| A0A074RGP9_9HOMO/104-377 | 9.0e-69 | Rhizoctonia solani 123E |
| L5M894_MYODS/66-307 | 1.0e-68 | Myotis davidii |
| I3JJZ3_ORENI/84-335 | 9.7e-69 | Oreochromis niloticus |
| A0A067SLH8_9AGAR/93-369 | 1.4e-68 | Galerina marginata CBS 339.88 |
| H2N3L9_PONAB/75-302 | 1.0e-68 | Pongo abelii |
| Q1RL55_CIOIN/125-367 | 2.1e-68 | Ciona intestinalis |
| F6TZD9_CIOIN/125-367 | 2.1e-68 | Ciona intestinalis |
| H2NEQ9_PONAB/105-327 | 1.4e-68 | Pongo abelii |
| E7F947_DANRE/74-325 | 1.3e-68 | Danio rerio |
| A4QNV6_DANRE/74-325 | 1.3e-68 | Danio rerio |
| X1WHQ9_DANRE/74-325 | 1.3e-68 | Danio rerio |
| A0A0D6LWY6_9BILA/182-412 | 1.7e-67 | Ancylostoma ceylanicum |
| E9QE31_DANRE/90-342 | 1.4e-68 | Danio rerio |
| A0A078B7R3_STYLE/205-446 | 1.9e-68 | Stylonychia lemnae |
| A0A066VG47_9HOMO/114-381 | 2.0e-68 | Rhizoctonia solani AG-8 WAC10335 |
| V2X4Z7_MONRO/102-377 | 1.7e-68 | Moniliophthora roreri |
| A0A0D2P2Z7_9AGAR/96-372 | 2.1e-68 | Hypholoma sublateritium FD-334 SS-4 |
| G3QB61_GASAC/80-322 | 2.9e-68 | Gasterosteus aculeatus |
| A0A0D2N738_9AGAR/45-320 | 2.3e-68 | Hypholoma sublateritium FD-334 SS-4 |
| A0A0B7FYL5_THACB/124-387 | 2.4e-68 | Thanatephorus cucumeris |
| V4AA66_LOTGI/165-426 | 3.5e-68 | Lottia gigantea |
| W5QEG6_SHEEP/74-314 | 2.4e-68 | Ovis aries |
| A0A091WB90_OPIHO/44-291 | 2.0e-68 | Opisthocomus hoazin |
| F1R632_DANRE/90-342 | 2.8e-68 | Danio rerio |
| M7B8E3_CHEMY/75-323 | 2.5e-68 | Chelonia mydas |
| H2MXN5_ORYLA/73-325 | 2.9e-68 | Oryzias latipes |
| X8JJZ6_9HOMO/106-378 | 2.8e-68 | Rhizoctonia solani AG-3 Rhs1AP |
| A0A074RP62_9HOMO/106-378 | 2.8e-68 | Rhizoctonia solani 123E |
| A0A074RKL4_9HOMO/114-381 | 3.4e-68 | Rhizoctonia solani 123E |
| X8JBX3_9HOMO/114-381 | 3.4e-68 | Rhizoctonia solani AG-3 Rhs1AP |
| F6TMZ0_MONDO/99-271 | 5.5e-52 | Monodelphis domestica |
| F6TMZ0_MONDO/349-401 | 4.8e-10 | Monodelphis domestica |
| A0A088AVY4_APIME/808-1048 | 3.5e-68 | Apis mellifera |
| F7FU37_CALJA/1-224 | 3.1e-68 | Callithrix jacchus |
| L9KRF0_TUPCH/21-247 | 4.1e-68 | Tupaia chinensis |
| F6TDC2_XENTR/1-224 | 3.5e-68 | Xenopus tropicalis |
| H2YK54_CIOSA/10-258 | 4.3e-68 | Ciona savignyi |
| H2YK53_CIOSA/19-269 | 4.7e-68 | Ciona savignyi |
| W5NAJ1_LEPOC/18-268 | 4.7e-68 | Lepisosteus oculatus |
| D2HYF5_AILME/62-313 | 4.0e-68 | Ailuropoda melanoleuca |
| B4DWH5_HUMAN/59-282 | 2.7e-65 | Homo sapiens |
| A0A0A9WJH6_LYGHE/353-591 | 5.4e-68 | Lygus hesperus |
| K1QU26_CRAGI/911-1184 | 5.5e-68 | Crassostrea gigas |
| E7F0H3_DANRE/73-324 | 4.7e-68 | Danio rerio |
| G1LYW6_AILME/62-313 | 4.5e-68 | Ailuropoda melanoleuca |
| J9HMY1_9SPIT/205-446 | 5.3e-68 | Oxytricha trifallax |
| I3JNG1_ORENI/86-336 | 5.3e-68 | Oreochromis niloticus |
| Q66JI2_XENTR/75-325 | 5.7e-68 | Xenopus tropicalis |
| A0A091RZY9_NESNO/10-227 | 6.0e-68 | Nestor notabilis |
| S4RHV4_PETMA/416-658 | 5.8e-68 | Petromyzon marinus |
| F1PAA8_CANFA/73-322 | 6.1e-68 | Canis familiaris |
| S4RHU4_PETMA/515-757 | 7.0e-68 | Petromyzon marinus |
| G3WGA7_SARHA/51-303 | 8.6e-68 | Sarcophilus harrisii |
| S4RHT9_PETMA/529-771 | 7.1e-68 | Petromyzon marinus |
| I3JDJ8_ORENI/46-315 | 2.0e-66 | Oreochromis niloticus |
| T1PH76_MUSDO/513-754 | 8.6e-68 | Musca domestica |
| I3IYD1_ORENI/79-329 | 7.9e-68 | Oreochromis niloticus |
| B4JNF8_DROGR/714-955 | 1.4e-67 | Drosophila grimshawi |
| A0A067RV60_ZOONE/1053-1290 | 1.1e-67 | Zootermopsis nevadensis |
| F7D8R2_MONDO/73-321 | 7.9e-68 | Monodelphis domestica |
| K1PJ80_CRAGI/110-355 | 9.4e-68 | Crassostrea gigas |
| A0A091MJD0_9PASS/44-291 | 7.2e-68 | Acanthisitta chloris |
| A0A0C9QYG1_9HYME/830-1071 | 9.5e-68 | Fopius arisanus |
| F3YDK6_DROME/468-709 | 1.3e-67 | Drosophila melanogaster |
| F3YDJ2_DROME/466-707 | 1.3e-67 | Drosophila melanogaster |
| C7TZP3_SCHJA/124-360 | 9.2e-68 | Schistosoma japonicum |
| A0A087YI68_POEFO/80-330 | 1.1e-67 | Poecilia formosa |
| K7GJ53_PELSI/48-296 | 1.1e-67 | Pelodiscus sinensis |
| A0A091R8K5_MERNU/44-291 | 9.2e-68 | Merops nubicus |
| L5KNG0_PTEAL/73-316 | 1.1e-67 | Pteropus alecto |
| G1N1T1_MELGA/76-324 | 1.5e-67 | Meleagris gallopavo |
| A0A091K1D5_COLST/44-291 | 9.9e-68 | Colius striatus |
| A0A093IKS0_FULGA/78-331 | 1.2e-67 | Fulmarus glacialis |
| C3XWK8_BRAFL/434-677 | 1.5e-67 | Branchiostoma floridae |
| J9GEK4_9SPIT/205-446 | 1.4e-67 | Oxytricha trifallax |
| A0A0C3BAA9_9HOMO/119-394 | 1.4e-67 | Serendipita vermifera MAFF 305830 |
| S9WML3_9CETA/28-251 | 1.4e-67 | Camelus ferus |
| CAND_DROME/1062-1303 | 2.7e-67 | Drosophila melanogaster |
| E1JJ98_DROME/1061-1302 | 2.7e-67 | Drosophila melanogaster |
| B4R3R3_DROSI/1057-1298 | 2.6e-67 | Drosophila simulans |
| I3M943_SPETR/2-246 | 1.7e-67 | Spermophilus tridecemlineatus |
| A0A091SNC7_9AVES/44-292 | 1.7e-67 | Pelecanus crispus |
| B4N1Z9_DROWI/1191-1432 | 2.9e-67 | Drosophila willistoni |
| B4PZ32_DROYA/1063-1304 | 2.7e-67 | Drosophila yakuba |
| I3NDM1_SPETR/2-246 | 2.1e-67 | Spermophilus tridecemlineatus |
| A0NEY9_ANOGA/342-589 | 2.8e-67 | Anopheles gambiae |
| G3WGA6_SARHA/69-316 | 2.1e-67 | Sarcophilus harrisii |
| B0WUP5_CULQU/645-886 | 2.5e-67 | Culex quinquefasciatus |
| W4X7P6_ATTCE/838-1079 | 2.7e-67 | Atta cephalotes |
| K1PV74_CRAGI/547-789 | 2.5e-67 | Crassostrea gigas |
| F6YBE6_HORSE/72-315 | 2.2e-67 | Equus caballus |
| A0A091QHY2_MERNU/13-226 | 2.1e-67 | Merops nubicus |
| A0A091KV79_9GRUI/44-291 | 1.8e-67 | Chlamydotis macqueenii |
| E2BZN4_HARSA/845-1086 | 2.9e-67 | Harpegnathos saltator |
| F4X630_ACREC/834-1075 | 2.7e-67 | Acromyrmex echinatior |
| A0A022T5G8_9HYME/910-1151 | 2.4e-67 | Microplitis demolitor |
| G0QPS2_ICHMG/403-645 | 1.6e-67 | Ichthyophthirius multifiliis |
| M3XZM4_MUSPF/47-218 | 6.7e-42 | Mustela putorius furo |
| M3XZM4_MUSPF/281-362 | 1.3e-19 | Mustela putorius furo |
| A0A060X116_ONCMY/24-274 | 2.3e-67 | Oncorhynchus mykiss |
| V8NLI7_OPHHA/79-328 | 3.5e-67 | Ophiophagus hannah |
| A0A093CFN1_9AVES/44-291 | 2.2e-67 | Pterocles gutturalis |
| A0A094K684_ANTCR/1-225 | 3.0e-67 | Antrostomus carolinensis |
| F6ZUQ6_CALJA/67-309 | 3.1e-67 | Callithrix jacchus |
| B3NYD6_DROER/1058-1299 | 5.2e-67 | Drosophila erecta |
| A0A067SIT6_9AGAR/88-364 | 3.0e-67 | Galerina marginata CBS 339.88 |
| A0A091HBB1_BUCRH/44-291 | 2.5e-67 | Buceros rhinoceros silvestris |
| A0A093H427_STRCA/1-224 | 3.0e-67 | Struthio camelus australis |
| E2A3Y1_CAMFO/850-1091 | 4.1e-67 | Camponotus floridanus |
| A0A060WTQ3_ONCMY/84-307 | 3.3e-67 | Oncorhynchus mykiss |
| G4TER7_PIRID/119-395 | 3.3e-67 | Piriformospora indica |
| F5HLY4_ANOGA/669-916 | 4.9e-67 | Anopheles gambiae |
| F1NGQ0_CHICK/74-328 | 4.1e-67 | Gallus gallus |
| A0A0C3FDX8_9HOMO/116-387 | 4.4e-67 | Piloderma croceum F 1598 |
| S4RZ10_PETMA/74-326 | 3.7e-67 | Petromyzon marinus |
| A0A0B7A6W7_9EUPU/238-489 | 4.5e-67 | Arion vulgaris |
| A0A091E0L9_FUKDA/72-323 | 3.6e-67 | Fukomys damarensis |
| A0A026WNK6_CERBI/800-1044 | 5.7e-67 | Cerapachys biroi |
| A0A091N7R4_APAVI/44-291 | 3.2e-67 | Apaloderma vittatum |
| S7MWQ7_MYOBR/75-302 | 3.6e-67 | Myotis brandtii |
| B4MAZ5_DROVI/1158-1399 | 6.2e-67 | Drosophila virilis |
| A0A091WVV7_NIPNI/44-291 | 3.7e-67 | Nipponia nippon |
| A0A093F3F9_GAVST/8-218 | 4.9e-67 | Gavia stellata |
| A0A084VH02_9DIPT/630-875 | 6.9e-67 | Anopheles sinensis |
| E7FC17_DANRE/64-314 | 5.5e-67 | Danio rerio |
| A0A0C3BD17_9HOMO/100-370 | 6.1e-67 | Piloderma croceum F 1598 |
| F6Q180_CALJA/1-223 | 5.2e-67 | Callithrix jacchus |
| A0A087XXC9_POEFO/73-325 | 5.9e-67 | Poecilia formosa |
| A0A078H6M4_BRANA/1697-1824 | 2.0e-39 | Brassica napus |
| A0A078H6M4_BRANA/1825-1909 | 8.3e-22 | Brassica napus |
| G1PAJ2_MYOLU/73-314 | 5.9e-67 | Myotis lucifugus |
| F1R0V4_DANRE/64-314 | 7.6e-67 | Danio rerio |
| L5LMV0_MYODS/73-314 | 6.3e-67 | Myotis davidii |
| M3ZD93_XIPMA/75-328 | 7.0e-67 | Xiphophorus maculatus |
| A0A0D7BQZ4_9HOMO/109-383 | 7.6e-67 | Cylindrobasidium torrendii FP15055 ss-10 |
| K7ISI6_NASVI/1580-1821 | 7.6e-67 | Nasonia vitripennis |
| W5JH01_ANODA/794-1039 | 1.0e-66 | Anopheles darlingi |
| F7GHV0_CALJA/88-348 | 8.7e-67 | Callithrix jacchus |
| B4L5D9_DROMO/1210-1451 | 1.4e-66 | Drosophila mojavensis |
| CAN13_MOUSE/69-311 | 8.0e-67 | Mus musculus |
| A0A067ST29_9AGAR/121-394 | 9.9e-67 | Galerina marginata CBS 339.88 |
| H2T9A4_TAKRU/58-297 | 9.0e-67 | Takifugu rubripes |
| A0A086TIG2_9FUNG/111-355 | 9.6e-67 | Mortierella verticillata NRRL 6337 |
| I3MHC5_SPETR/71-313 | 9.3e-67 | Spermophilus tridecemlineatus |
| I3N0A2_SPETR/71-313 | 9.3e-67 | Spermophilus tridecemlineatus |
| I3NCP5_SPETR/73-315 | 1.1e-66 | Spermophilus tridecemlineatus |
| G3RTH4_GORGO/109-360 | 1.1e-66 | Gorilla gorilla gorilla |
| A0A085MW32_9BILA/63-317 | 1.2e-66 | Trichuris suis |
| G1KGI9_ANOCA/90-340 | 1.0e-66 | Anolis carolinensis |
| A0A093FRS2_GAVST/44-304 | 1.3e-66 | Gavia stellata |
| A0A078IGL8_BRANA/2421-2548 | 2.8e-39 | Brassica napus |
| A0A078IGL8_BRANA/2549-2633 | 1.2e-21 | Brassica napus |
| T1FB33_HELRO/209-456 | 1.7e-66 | Helobdella robusta |
| Q24DF0_TETTS/775-1021 | 1.8e-66 | Tetrahymena thermophila |
| B3MXY5_DROAN/1165-1406 | 1.4e-66 | Drosophila ananassae |
| W8B644_CERCA/466-708 | 2.1e-66 | Ceratitis capitata |
| A0A087RE84_APTFO/44-291 | 1.2e-66 | Aptenodytes forsteri |
| A0A091RJ01_9GRUI/1-225 | 1.6e-66 | Mesitornis unicolor |
| W8BGV2_CERCA/366-608 | 1.8e-66 | Ceratitis capitata |
| W8B1X7_CERCA/869-1111 | 3.8e-66 | Ceratitis capitata |
| K3WE14_PYTUL/111-375 | 1.6e-66 | Pythium ultimum DAOM BR144 |
| W8AUJ6_CERCA/498-740 | 2.3e-66 | Ceratitis capitata |
| A0A0C3LBD0_9HOMO/81-349 | 2.1e-66 | Tulasnella calospora MUT 4182 |
| A0A091P2A9_HALAL/44-304 | 1.1e-65 | Haliaeetus albicilla |
| I3LMV5_PIG/15-186 | 2.0e-41 | Sus scrofa |
| I3LMV5_PIG/230-311 | 2.7e-19 | Sus scrofa |
| A0A0A1XFI8_BACCU/370-611 | 2.0e-66 | Bactrocera cucurbitae |
| U3J800_ANAPL/115-410 | 7.4e-65 | Anas platyrhynchos |
| M5FYJ8_DACSP/92-369 | 1.7e-66 | Dacryopinax sp. DJM 731 |
| A0A093FC91_GAVST/1-224 | 1.9e-66 | Gavia stellata |
| A0A091GZU3_BUCRH/1-225 | 1.9e-66 | Buceros rhinoceros silvestris |
| A8NBA1_COPC7/93-361 | 2.0e-66 | Coprinopsis cinerea |
| G5B8M1_HETGA/62-313 | 2.0e-66 | Heterocephalus glaber |
| A0A067PLU6_9HOMO/117-381 | 2.2e-66 | Jaapia argillacea MUCL 33604 |
| A0A0A1WPH4_BACCU/479-720 | 2.5e-66 | Bactrocera cucurbitae |
| H2UH73_TAKRU/52-318 | 7.0e-64 | Takifugu rubripes |
| E2LG92_MONPE/37-300 | 5.2e-66 | Moniliophthora perniciosa |
| A0A091RBV0_MERNU/1-224 | 2.2e-66 | Merops nubicus |
| A0A099Z9E1_TINGU/7-230 | 2.4e-66 | Tinamus guttatus |
| A8NAF5_COPC7/172-449 | 2.6e-66 | Coprinopsis cinerea |
| R7TJF2_CAPTE/109-353 | 2.5e-66 | Capitella teleta |
| A0A093NPJ0_PYGAD/44-291 | 2.2e-66 | Pygoscelis adeliae |
| U4L2S3_PYROM/125-394 | 3.4e-66 | Pyronema omphalodes |
| CAN13_RAT/63-313 | 3.0e-66 | Rattus norvegicus |
| A0BI61_PARTE/181-423 | 3.9e-66 | Paramecium tetraurelia |
| A0A023EVG6_AEDAL/132-374 | 3.5e-66 | Aedes albopictus |
| G3SNL0_LOXAF/3-226 | 3.4e-66 | Loxodonta africana |
| A0A034W488_BACDO/370-611 | 4.5e-66 | Bactrocera dorsalis |
| A0A0A1XHQ8_BACCU/901-1142 | 4.4e-66 | Bactrocera cucurbitae |
| A0A087XVY0_POEFO/77-247 | 2.5e-42 | Poecilia formosa |
| A0A087XVY0_POEFO/301-379 | 7.1e-18 | Poecilia formosa |
| V3ZTI1_LOTGI/51-308 | 5.9e-66 | Lottia gigantea |
| I3JSI8_ORENI/400-638 | 6.1e-66 | Oreochromis niloticus |
| Q23RI7_TETTS/155-397 | 1.7e-65 | Tetrahymena thermophila |
| A0A087URU6_9ARAC/2-235 | 4.7e-66 | Stegodyphus mimosarum |
| A0A093PTT3_9PASS/1-224 | 4.9e-66 | Manacus vitellinus |
| A0A034W046_BACDO/901-1142 | 9.8e-66 | Bactrocera dorsalis |
| G1KGC4_ANOCA/79-329 | 6.4e-66 | Anolis carolinensis |
| A0A094NIY7_9AVES/1-224 | 6.8e-66 | Podiceps cristatus |
| A0A074ZUH1_9TREM/249-459 | 2.1e-42 | Opisthorchis viverrini |
| A0A074ZUH1_9TREM/494-603 | 1.4e-17 | Opisthorchis viverrini |
| A0A091EJ98_CORBR/44-304 | 6.9e-66 | Corvus brachyrhynchos |
| A0A096M905_POEFO/1-256 | 7.2e-66 | Poecilia formosa |
| I3MDF9_SPETR/75-327 | 1.3e-65 | Spermophilus tridecemlineatus |
| A0A0C3C0E7_HEBCY/90-366 | 6.6e-66 | Hebeloma cylindrosporum h7 |
| M7AHQ8_CHEMY/123-366 | 7.4e-66 | Chelonia mydas |
| A0A093PKU5_9PASS/44-304 | 8.0e-66 | Manacus vitellinus |
| I3IYD2_ORENI/19-263 | 8.2e-66 | Oreochromis niloticus |
| A0A067PIS2_9HOMO/116-384 | 7.2e-66 | Jaapia argillacea MUCL 33604 |
| T0M7V2_9CETA/32-256 | 9.4e-66 | Camelus ferus |
| A0A093ND50_PYGAD/1-224 | 8.0e-66 | Pygoscelis adeliae |
| H2L3M8_ORYLA/14-250 | 8.7e-66 | Oryzias latipes |
| Q5C278_SCHJA/80-314 | 7.5e-66 | Schistosoma japonicum |
| E2QWT7_CANFA/62-311 | 1.0e-65 | Canis familiaris |
| H2RUW1_TAKRU/74-342 | 1.3e-65 | Takifugu rubripes |
| K7FD40_PELSI/56-301 | 1.1e-65 | Pelodiscus sinensis |
| A0A060Y275_ONCMY/18-248 | 9.9e-66 | Oncorhynchus mykiss |
| W5LLH3_ASTMX/8-231 | 1.1e-65 | Astyanax mexicanus |
| H2PJ68_PONAB/109-339 | 1.0e-65 | Pongo abelii |
| M3XZJ5_MUSPF/72-314 | 1.1e-65 | Mustela putorius furo |
| A0A091PMG8_LEPDC/78-323 | 9.3e-66 | Leptosomus discolor |
| A2D9A8_TRIVA/990-1239 | 1.4e-65 | Trichomonas vaginalis |
| A0A091KUW2_9GRUI/1-224 | 1.1e-65 | Chlamydotis macqueenii |
| A0A0B7G1E3_THACB/116-382 | 1.2e-65 | Thanatephorus cucumeris |
| L8WNC3_THACA/121-395 | 1.2e-65 | Thanatephorus cucumeris |
| A0A0A1NPG1_9FUNG/301-560 | 1.5e-65 | Rhizopus microsporus |
| A0A067SVS7_9AGAR/183-459 | 1.2e-65 | Galerina marginata CBS 339.88 |
| H3CM28_TETNG/4-254 | 1.5e-65 | Tetraodon nigroviridis |
| H3CM27_TETNG/3-253 | 1.5e-65 | Tetraodon nigroviridis |
| M5CG70_THACB/112-379 | 1.5e-65 | Thanatephorus cucumeris |
| W4H6S9_9STRA/101-367 | 1.7e-65 | Aphanomyces astaci |
| G4TNA1_PIRID/127-400 | 2.6e-65 | Piriformospora indica |
| V3ZVX2_LOTGI/63-335 | 1.8e-65 | Lottia gigantea |
| G3SPH3_LOXAF/1-224 | 1.8e-65 | Loxodonta africana |
| A0A072PKE6_9EURO/186-469 | 2.1e-65 | Exophiala aquamarina CBS 119918 |
| F2UEB3_SALR5/154-427 | 2.0e-65 | Salpingoeca rosetta |
| A0A093IPA8_FULGA/44-304 | 2.0e-65 | Fulmarus glacialis |
| R1C8G2_EMIHU/62-291 | 3.4e-65 | Emiliania huxleyi CCMP1516 |
| C1MT99_MICPC/116-395 | 2.3e-65 | Micromonas pusilla |
| M5C8M8_THACB/112-379 | 2.4e-65 | Thanatephorus cucumeris |
| A0A0B7FZM1_THACB/112-379 | 2.5e-65 | Thanatephorus cucumeris |
| T1IN91_STRMM/60-309 | 3.2e-65 | Strigamia maritima |
| A0A060XHR8_ONCMY/57-271 | 3.3e-65 | Oncorhynchus mykiss |
| F6YYV5_CALJA/72-317 | 2.9e-65 | Callithrix jacchus |
| D2A1T4_TRICA/710-949 | 4.3e-65 | Tribolium castaneum |
| T1I9F8_RHOPR/318-555 | 4.3e-65 | Rhodnius prolixus |
| A7E6D7_SCLS1/182-453 | 5.0e-65 | Sclerotinia sclerotiorum |
| J9K0L5_ACYPI/406-650 | 4.9e-65 | Acyrthosiphon pisum |
| A0A099ZWQ2_CHAVO/44-304 | 4.0e-65 | Charadrius vociferus |
| A0A093EEK6_TAUER/44-304 | 5.0e-65 | Tauraco erythrolophus |
| A0A093J6T7_EURHL/44-306 | 6.3e-65 | Eurypyga helias |
| Q4ST20_TETNG/52-325 | 8.8e-62 | Tetraodon nigroviridis |
| A0A0C2Y808_HEBCY/117-389 | 5.5e-65 | Hebeloma cylindrosporum h7 |
| F7I7I7_CALJA/72-317 | 5.9e-65 | Callithrix jacchus |
| G1TYB0_RABIT/71-322 | 5.7e-65 | Oryctolagus cuniculus |
| W9C927_9HELO/185-456 | 9.0e-65 | Sclerotinia borealis F-4157 |
| F6PGW8_HORSE/1-223 | 5.8e-65 | Equus caballus |
| M7THF8_BOTF1/184-455 | 9.6e-65 | Botryotinia fuckeliana |
| W2T578_NECAM/189-405 | 5.7e-65 | Necator americanus |
| A0A024UVW5_9STRA/114-380 | 6.6e-65 | Aphanomyces invadans |
| G2XV69_BOTF4/205-476 | 9.9e-65 | Botryotinia fuckeliana |
| A0A015J9I4_9GLOM/53-364 | 9.1e-65 | Rhizophagus irregularis DAOM 197198w |
| U9UIW0_RHIID/53-364 | 9.1e-65 | Rhizophagus irregularis |
| A0A0C7BXB3_9FUNG/120-379 | 7.6e-65 | Rhizopus microsporus |
| M3W3D8_FELCA/2-241 | 7.7e-65 | Felis catus |
| Q6NW69_DANRE/64-314 | 9.7e-65 | Danio rerio |
| A0A091DKJ4_FUKDA/62-313 | 8.0e-65 | Fukomys damarensis |
| G3PIF7_GASAC/21-271 | 8.1e-65 | Gasterosteus aculeatus |
| M2SA86_COCSN/159-430 | 9.0e-65 | Cochliobolus sativus |
| A0A0C7BXB8_9FUNG/175-434 | 8.7e-65 | Rhizopus microsporus |
| A0A0D1XP22_9PEZI/180-451 | 1.1e-64 | Verruconis gallopava |
| M4A2U1_XIPMA/1-224 | 8.8e-65 | Xiphophorus maculatus |
| W4KKZ4_9HOMO/69-338 | 1.1e-64 | Heterobasidion irregulare TC 32-1 |
| A0A0C9U8E5_9HOMO/101-376 | 1.1e-64 | Sphaerobolus stellatus SS14 |
| V9KVL4_CALMI/75-318 | 3.2e-64 | Callorhinchus milii |
| F1KY41_ASCSU/1-220 | 9.8e-65 | Ascaris suum |
| F1QMV0_DANRE/2-222 | 1.3e-64 | Danio rerio |
| F7GPX3_CALJA/75-326 | 3.1e-64 | Callithrix jacchus |
| H3BAM4_LATCH/532-771 | 4.2e-64 | Latimeria chalumnae |
| G1TCB5_RABIT/84-335 | 1.5e-64 | Oryctolagus cuniculus |
| A0A0C9UZW5_9HOMO/85-363 | 1.7e-64 | Sphaerobolus stellatus SS14 |
| G1RZX0_NOMLE/72-315 | 1.6e-64 | Nomascus leucogenys |
| D0NT34_PHYIT/196-489 | 1.7e-64 | Phytophthora infestans |
| T0QXK2_9STRA/82-348 | 1.6e-64 | Saprolegnia diclina VS20 |
| L5M1J4_MYODS/34-256 | 1.9e-64 | Myotis davidii |
| A0A0D1WQ48_9EURO/185-468 | 2.3e-64 | Exophiala mesophila |
| S8BH70_DACHA/185-452 | 2.7e-64 | Dactylellina haptotyla |
| W9WCB4_9EURO/221-504 | 2.6e-64 | Cladophialophora yegresii CBS 114405 |
| A0A0D7A591_9AGAR/95-370 | 2.3e-64 | Fistulina hepatica ATCC 64428 |
| L8Y8M7_TUPCH/12-239 | 9.0e-64 | Tupaia chinensis |
| I7MG72_TETTS/142-374 | 3.2e-64 | Tetrahymena thermophila |
| A0A091DRE5_FUKDA/58-281 | 3.8e-64 | Fukomys damarensis |
| A0A023F4R8_TRIIF/782-1018 | 4.0e-64 | Triatoma infestans |
| A0A0C9LU94_9FUNG/325-584 | 3.6e-64 | Mucor ambiguus |
| Q23YD5_TETTS/102-335 | 4.3e-64 | Tetrahymena thermophila |
| F6SFW8_MACMU/72-302 | 3.7e-64 | Macaca mulatta |
| L5KK31_PTEAL/596-834 | 4.8e-64 | Pteropus alecto |
| G1XCE2_ARTOA/185-454 | 4.1e-64 | Arthrobotrys oligospora |
| E7FAX5_DANRE/570-813 | 5.3e-64 | Danio rerio |
| R0ITF3_SETT2/159-430 | 4.0e-64 | Setosphaeria turcica |
| H0WIW5_OTOGA/62-313 | 4.1e-64 | Otolemur garnettii |
| F6RWK0_ORNAN/1-224 | 4.3e-64 | Ornithorhynchus anatinus |
| H2RR02_TAKRU/15-250 | 4.6e-64 | Takifugu rubripes |
| A0A0D2FX42_9EURO/214-497 | 5.2e-64 | Capronia semiimmersa |
| F7AGH7_XENTR/1-142 | 6.7e-39 | Xenopus tropicalis |
| F7AGH7_XENTR/190-271 | 3.0e-19 | Xenopus tropicalis |
| G7Q029_MACFA/334-576 | 6.0e-64 | Macaca fascicularis |
| S4R4M3_PETMA/1-228 | 4.1e-64 | Petromyzon marinus |
| A0A084FUG2_9PEZI/156-428 | 6.0e-64 | Scedosporium apiospermum |
| A0A0D2IIP4_9EURO/229-512 | 5.9e-64 | Fonsecaea multimorphosa CBS 102226 |
| H9G7D7_ANOCA/611-849 | 2.0e-63 | Anolis carolinensis |
| V9DC29_9EURO/221-504 | 6.5e-64 | Cladophialophora carrionii CBS 160.54 |
| A0A067MU31_9HOMO/108-391 | 7.4e-64 | Botryobasidium botryosum FD-172 SS1 |
| J9ITG9_9SPIT/91-324 | 6.6e-64 | Oxytricha trifallax |
| S9WKE5_9CETA/72-218 | 2.6e-42 | Camelus ferus |
| S9WKE5_9CETA/218-290 | 6.0e-16 | Camelus ferus |
| A0A0D2G5Y7_9EURO/234-517 | 7.2e-64 | Fonsecaea pedrosoi CBS 271.37 |
| Q4SUI7_TETNG/35-278 | 6.5e-64 | Tetraodon nigroviridis |
| H3A395_LATCH/80-371 | 1.7e-63 | Latimeria chalumnae |
| W6YRH2_COCMI/159-430 | 7.1e-64 | Bipolaris oryzae ATCC 44560 |
| H2LRZ7_ORYLA/62-280 | 5.0e-64 | Oryzias latipes |
| F6ZW91_MONDO/606-847 | 1.1e-62 | Monodelphis domestica |
| H2QHQ0_PANTR/72-315 | 6.6e-64 | Pan troglodytes |
| W7F3D3_COCVI/159-430 | 7.6e-64 | Bipolaris victoriae FI3 |
| H2P6K6_PONAB/72-315 | 7.6e-64 | Pongo abelii |
| G3N349_BOVIN/446-684 | 9.4e-64 | Bos taurus |
| F7C364_MONDO/73-321 | 8.3e-64 | Monodelphis domestica |
| A2FHB3_TRIVA/1018-1269 | 9.4e-64 | Trichomonas vaginalis |
| U6NUT8_HAECO/424-641 | 8.7e-64 | Haemonchus contortus |
| F6VYQ3_MONDO/44-215 | 1.5e-38 | Monodelphis domestica |
| F6VYQ3_MONDO/261-347 | 2.0e-19 | Monodelphis domestica |
| D3ZJJ4_RAT/536-777 | 1.1e-63 | Rattus norvegicus |
| H2QG90_PANTR/1-226 | 9.9e-64 | Pan troglodytes |
| M3XDT9_FELCA/538-778 | 9.9e-64 | Felis catus |
| E1B6Y0_BOVIN/523-761 | 1.1e-63 | Bos taurus |
| M3YX37_MUSPF/598-840 | 1.1e-63 | Mustela putorius furo |
| W6YGR9_COCCA/159-430 | 9.8e-64 | Bipolaris zeicola 26-R-13 |
| L1JBM0_GUITH/40-273 | 1.0e-63 | Guillardia theta CCMP2712 |
| CAN15_MOUSE/544-785 | 1.2e-63 | Mus musculus |
| H0V3Z7_CAVPO/71-312 | 1.1e-63 | Cavia porcellus |
| A0A0F5REM5_9PORP/194-468 | 9.1e-64 | Porphyromonas gulae |
| A0A0A2G291_9PORP/194-468 | 9.1e-64 | Porphyromonas gulae |
| A8WGH7_XENTR/432-671 | 1.9e-63 | Xenopus tropicalis |
| W5KG96_ASTMX/94-264 | 3.3e-51 | Astyanax mexicanus |
| W5KG96_ASTMX/317-395 | 1.4e-06 | Astyanax mexicanus |
| G1R9H7_NOMLE/54-293 | 8.9e-64 | Nomascus leucogenys |
| A0A067CRY7_SAPPC/91-357 | 1.0e-63 | Saprolegnia parasitica CBS 223.65 |
| A0A0B7BIK0_9EUPU/42-298 | 1.2e-63 | Arion vulgaris |
| G3VY79_SARHA/466-707 | 1.0e-62 | Sarcophilus harrisii |
| G7NQD5_MACMU/533-775 | 1.2e-63 | Macaca mulatta |
| A0A0D2CA82_9EURO/229-512 | 1.2e-63 | Cladophialophora immunda |
| G3VY80_SARHA/466-707 | 1.0e-62 | Sarcophilus harrisii |
| F1RGT7_PIG/510-747 | 1.3e-63 | Sus scrofa |
| F1RGT8_PIG/517-754 | 1.3e-63 | Sus scrofa |
| G3RHW3_GORGO/72-315 | 1.1e-63 | Gorilla gorilla gorilla |
| G3RUL3_GORGO/72-315 | 1.1e-63 | Gorilla gorilla gorilla |
| M4AX88_XIPMA/538-781 | 2.2e-63 | Xiphophorus maculatus |
| Q23JW9_TETTS/279-529 | 1.1e-63 | Tetrahymena thermophila |
| A0A096NKF4_PAPAN/601-843 | 1.3e-63 | Papio anubis |
| K1Q505_CRAGI/49-308 | 1.1e-63 | Crassostrea gigas |
| I2CVD0_MACMU/533-775 | 1.3e-63 | Macaca mulatta |
| K6ZR76_PANTR/536-775 | 1.3e-63 | Pan troglodytes |
| K7AP34_PANTR/536-775 | 1.3e-63 | Pan troglodytes |
| F7EBB5_MACMU/72-315 | 1.1e-63 | Macaca mulatta |
| CAN15_HUMAN/536-775 | 1.3e-63 | Homo sapiens |
| A0A0D9RK01_CHLSB/601-840 | 1.4e-63 | Chlorocebus sabaeus |
| J9IEQ0_9SPIT/93-340 | 1.4e-63 | Oxytricha trifallax |
| A0A093IT86_EURHL/63-311 | 1.2e-63 | Eurypyga helias |
| A0A0D2F4U6_9EURO/229-512 | 1.3e-63 | Cladophialophora bantiana CBS 173.52 |
| F6QC28_CALJA/528-766 | 1.6e-63 | Callithrix jacchus |
| R7VGC2_CAPTE/48-320 | 1.7e-63 | Capitella teleta |
| M3ZLN5_XIPMA/6-254 | 1.6e-63 | Xiphophorus maculatus |
| G0P7C1_CAEBE/96-325 | 8.1e-22 | Caenorhabditis brenneri |
| G0P7C1_CAEBE/675-903 | 6.9e-37 | Caenorhabditis brenneri |
| G1Q947_MYOLU/42-213 | 1.0e-40 | Myotis lucifugus |
| G1Q947_MYOLU/273-354 | 8.3e-17 | Myotis lucifugus |
| W9X4X3_9EURO/229-512 | 1.6e-63 | Cladophialophora psammophila CBS 110553 |
| A0A093BKW4_CHAPE/530-768 | 4.9e-63 | Chaetura pelagica |
| U3CBX1_CALJA/528-766 | 1.8e-63 | Callithrix jacchus |
| T0S7R4_9STRA/91-357 | 1.6e-63 | Saprolegnia diclina VS20 |
| E9FU36_DAPPU/404-668 | 2.0e-63 | Daphnia pulex |
| Q4T4R2_TETNG/50-292 | 1.6e-63 | Tetraodon nigroviridis |
| M7BS27_CHEMY/736-974 | 4.7e-63 | Chelonia mydas |
| G1PFD3_MYOLU/529-767 | 2.6e-63 | Myotis lucifugus |
| W2JS81_PHYPR/291-565 | 2.1e-63 | Phytophthora parasitica |
| G1LKP8_AILME/517-755 | 2.3e-63 | Ailuropoda melanoleuca |
| F6Y261_MACMU/533-775 | 2.1e-63 | Macaca mulatta |
| S2KGD1_MUCC1/239-498 | 1.9e-63 | Mucor circinelloides f. circinelloides |
| D2HDW3_AILME/533-771 | 2.4e-63 | Ailuropoda melanoleuca |
| A0A0D2J2S9_9EURO/226-509 | 2.1e-63 | Rhinocladiella mackenziei CBS 650.93 |
| A0A078AZ10_STYLE/204-450 | 2.7e-63 | Stylonychia lemnae |
| A0A096LTX4_POEFO/535-778 | 4.1e-63 | Poecilia formosa |
| H3CF83_TETNG/376-618 | 2.8e-63 | Tetraodon nigroviridis |
| S3C030_OPHP1/150-422 | 3.1e-63 | Ophiostoma piceae |
| I3N5U9_SPETR/525-765 | 3.6e-63 | Spermophilus tridecemlineatus |
| L5LQR1_MYODS/566-804 | 2.9e-63 | Myotis davidii |
| A0A087YH20_POEFO/649-890 | 4.8e-63 | Poecilia formosa |
| A0A091GAS6_9AVES/546-784 | 6.2e-63 | Cuculus canorus |
| B2R6T2_HUMAN/51-325 | 3.2e-63 | Homo sapiens |
| CAN6_HUMAN/51-325 | 3.2e-63 | Homo sapiens |
| G7N9R9_MACMU/72-315 | 3.2e-63 | Macaca mulatta |
| G7PLZ1_MACFA/72-315 | 3.2e-63 | Macaca fascicularis |
| A0A096NFQ7_PAPAN/72-315 | 3.2e-63 | Papio anubis |
| F7EBC1_MACMU/72-315 | 3.2e-63 | Macaca mulatta |
| A2QVX3_ASPNC/141-392 | 4.1e-63 | Aspergillus niger |
| B4USK8_OTOGA/524-763 | 5.0e-63 | Otolemur garnettii |
| A0BL14_PARTE/72-313 | 4.6e-63 | Paramecium tetraurelia |
| H2UHV7_TAKRU/538-781 | 7.4e-63 | Takifugu rubripes |
| F1PB63_CANFA/51-325 | 4.0e-63 | Canis familiaris |
| H2TH86_TAKRU/7-256 | 5.0e-63 | Takifugu rubripes |
| C1E493_MICSR/277-555 | 4.4e-63 | Micromonas sp. |
| CAN13_HUMAN/72-315 | 4.4e-63 | Homo sapiens |
| H2UHV6_TAKRU/538-781 | 8.1e-63 | Takifugu rubripes |
| J9NVT7_CANFA/521-760 | 5.9e-63 | Canis familiaris |
| S7N040_MYOBR/1327-1565 | 6.2e-63 | Myotis brandtii |
| G3U8K8_LOXAF/93-329 | 5.4e-63 | Loxodonta africana |
| E2R1S3_CANFA/534-773 | 6.0e-63 | Canis familiaris |
| F6VYM2_MONDO/73-317 | 5.0e-63 | Monodelphis domestica |
| A0A023FW73_9ACAR/68-293 | 4.5e-63 | Amblyomma parvum |
| Q0U7G1_PHANO/130-401 | 6.1e-63 | Phaeosphaeria |
| A0A081AZ73_PHYPR/291-565 | 6.3e-63 | Phytophthora parasitica P1976 |
| W2P2L6_PHYPR/291-565 | 6.3e-63 | Phytophthora parasitica |
| W2XRZ6_PHYPR/291-565 | 6.3e-63 | Phytophthora parasitica CJ01A1 |
| V9FYI2_PHYPR/291-565 | 6.3e-63 | Phytophthora parasitica P1569 |
| W2LW34_PHYPR/291-565 | 6.3e-63 | Phytophthora parasitica |
| W3A2B2_PHYPR/291-565 | 6.3e-63 | Phytophthora parasitica P10297 |
| W2QU61_PHYPN/291-565 | 6.3e-63 | Phytophthora parasitica |
| A8I337_CHLRE/75-333 | 6.0e-63 | Chlamydomonas reinhardtii |
| E3QBR6_COLGM/131-402 | 6.1e-63 | Colletotrichum graminicola |
| G2X5V8_VERDV/121-392 | 6.9e-63 | Verticillium dahliae |
| R7UNL8_CAPTE/6-254 | 5.1e-63 | Capitella teleta |
| A0A091R5Z0_9GRUI/546-784 | 1.1e-62 | Mesitornis unicolor |
| F1RWU4_PIG/51-325 | 6.0e-63 | Sus scrofa |
| A0A093PEB8_9PASS/436-674 | 1.7e-62 | Manacus vitellinus |
| A0A078ACE3_STYLE/202-445 | 7.1e-63 | Stylonychia lemnae |
| I3KVT1_ORENI/439-682 | 1.3e-62 | Oreochromis niloticus |
| E3RN87_PYRTT/158-429 | 7.4e-63 | Pyrenophora teres f. teres |
| C1E5L8_MICSR/119-412 | 1.0e-62 | Micromonas sp. |
| H2LS81_ORYLA/543-781 | 1.3e-62 | Oryzias latipes |
| A0A093JC02_EURHL/546-784 | 1.4e-62 | Eurypyga helias |
| K2S7D6_MACPH/125-395 | 8.7e-63 | Macrophomina phaseolina) |
| A0A091II72_CALAN/484-722 | 1.8e-62 | Calypte anna |
| E6ZGY8_DICLA/597-840 | 1.3e-62 | Dicentrarchus labrax |
| I3KVT2_ORENI/539-782 | 1.6e-62 | Oreochromis niloticus |
| J9ISJ7_9SPIT/140-395 | 9.5e-63 | Oxytricha trifallax |
| A0A099Z394_TINGU/546-784 | 1.5e-62 | Tinamus guttatus |
| J9IKT3_9SPIT/140-395 | 9.5e-63 | Oxytricha trifallax |
| A0A091Q5U9_LEPDC/541-779 | 1.9e-62 | Leptosomus discolor |
| A0A023F4D4_TRIIF/80-336 | 9.2e-63 | Triatoma infestans |
| I3KVT0_ORENI/598-841 | 1.7e-62 | Oreochromis niloticus |
| Q0CKC6_ASPTN/143-422 | 1.3e-62 | Aspergillus terreus |
| A0A066XBM9_COLSU/165-436 | 1.1e-62 | Colletotrichum sublineola |
| A4S6M5_OSTLU/110-391 | 1.0e-62 | Ostreococcus lucimarinus |
| I3MDM7_SPETR/51-325 | 1.1e-62 | Spermophilus tridecemlineatus |
| E1BTA8_CHICK/533-771 | 2.0e-62 | Gallus gallus |
| A0A0A0AZG8_CHAVO/546-784 | 2.4e-62 | Charadrius vociferus |
| A0A093P4X1_PYGAD/546-784 | 2.0e-62 | Pygoscelis adeliae |
| A0A091UQN0_NIPNI/535-773 | 2.2e-62 | Nipponia nippon |
| A0A0D2EAB4_9EURO/217-502 | 1.4e-62 | Exophiala xenobiotica |
| W8APN2_CERCA/285-499 | 1.1e-62 | Ceratitis capitata |
| A0A087QI89_APTFO/546-784 | 2.2e-62 | Aptenodytes forsteri |
| G1RWN6_NOMLE/51-325 | 1.3e-62 | Nomascus leucogenys |
| R1G6R2_BOTPV/123-393 | 1.5e-62 | Botryosphaeria parva |
| A0A0C3DUJ1_9PEZI/127-399 | 1.9e-62 | Oidiodendron maius Zn |
| G1P5T0_MYOLU/51-325 | 1.4e-62 | Myotis lucifugus |
| A0A090D541_PODAN/234-479 | 1.7e-62 | Podospora anserina |
| B2B4M7_PODAN/234-479 | 1.7e-62 | Podospora anserina |
| A0A094AA96_9PEZI/158-429 | 2.0e-62 | Pseudogymnoascus pannorum VKM F-3775 |
| A0A090MCL3_9HYPO/205-478 | 1.9e-62 | Fusarium sp. FIESC_5 CS3069 |
| R7V817_CAPTE/3-255 | 1.3e-62 | Capitella teleta |
| G3QXW3_GORGO/52-326 | 1.7e-62 | Gorilla gorilla gorilla |
| G3RMF5_GORGO/536-775 | 2.1e-62 | Gorilla gorilla gorilla |
| D2HEA2_AILME/51-325 | 1.7e-62 | Ailuropoda melanoleuca |
| H0ZYX1_TAEGU/526-764 | 3.9e-62 | Taeniopygia guttata |
| H0W573_CAVPO/529-773 | 2.3e-62 | Cavia porcellus |
| K1Q0W4_CRAGI/1583-1819 | 1.1e-48 | Crassostrea gigas |
| E4ZY10_LEPMJ/369-640 | 2.0e-62 | Leptosphaeria maculans |
| H2QZ04_PANTR/51-325 | 1.8e-62 | Pan troglodytes |
| F6ZMP5_HORSE/51-325 | 1.8e-62 | Equus caballus |
| Q012Z6_OSTTA/236-502 | 2.2e-62 | Ostreococcus tauri |
| B8N4G8_ASPFN/141-421 | 2.2e-62 | Aspergillus flavus |
| A0A0C3QH63_9HOMO/84-359 | 1.9e-62 | Tulasnella calospora MUT 4182 |
| V4CDZ5_LOTGI/2-249 | 1.7e-62 | Lottia gigantea |
| A0A0B7N4J0_9FUNG/328-587 | 2.5e-62 | Parasitella parasitica |
| A0A0D9RFG0_CHLSB/72-315 | 2.0e-62 | Chlorocebus sabaeus |
| U3JQ19_FICAL/607-845 | 4.4e-62 | Ficedula albicollis |
| A0A093HAW1_STRCA/546-784 | 4.3e-62 | Struthio camelus australis |
| A7RJ84_NEMVE/10-180 | 4.6e-45 | Nematostella vectensis |
| A7RJ84_NEMVE/184-237 | 3.1e-11 | Nematostella vectensis |
| W5PRQ3_SHEEP/493-730 | 2.7e-62 | Ovis aries |
| A0A0D2B4K0_9PEZI/182-496 | 3.2e-62 | Verruconis gallopava |
| A1DMK0_NEOFI/141-421 | 2.6e-62 | Neosartorya fischeri |
| T0MER7_9CETA/379-615 | 1.3e-59 | Camelus ferus |
| T1G4S5_HELRO/62-225 | 2.5e-43 | Helobdella robusta |
| T1G4S5_HELRO/227-286 | 1.1e-12 | Helobdella robusta |
| R8BVV9_TOGMI/189-460 | 4.5e-62 | Togninia minima |
| A0A091DRM3_FUKDA/547-784 | 3.7e-62 | Fukomys damarensis |
| M3WF83_FELCA/51-325 | 2.9e-62 | Felis catus |
| G1TQN1_RABIT/51-325 | 2.9e-62 | Oryctolagus cuniculus |
| U3AT41_CALJA/51-325 | 3.1e-62 | Callithrix jacchus |
| F7CEG2_CALJA/52-326 | 3.1e-62 | Callithrix jacchus |
| T1HE54_RHOPR/89-319 | 5.0e-62 | Rhodnius prolixus |
| H9F082_MACMU/51-325 | 3.2e-62 | Macaca mulatta |
| H6C004_EXODN/275-558 | 4.0e-62 | Exophiala dermatitidis |
| U4UMU3_DENPD/483-726 | 4.7e-62 | Dendroctonus ponderosae |
| W5Q0H3_SHEEP/1-224 | 3.5e-62 | Ovis aries |
| I8IU94_ASPO3/141-421 | 5.0e-62 | Aspergillus oryzae |
| Q2UM54_ASPOR/141-421 | 5.0e-62 | Aspergillus oryzae) |
| A0A064B7W4_ASPOZ/141-421 | 5.0e-62 | Aspergillus oryzae 100-8 |
| R7UPU6_CAPTE/347-602 | 4.0e-62 | Capitella teleta |
| F7C609_CALJA/85-314 | 8.0e-60 | Callithrix jacchus |
| R1FKT8_EMIHU/237-482 | 2.9e-22 | Emiliania huxleyi CCMP1516 |
| R1FKT8_EMIHU/986-1259 | 1.7e-33 | Emiliania huxleyi CCMP1516 |
| G3W098_SARHA/2-244 | 4.5e-62 | Sarcophilus harrisii |
| Q4WN14_ASPFU/151-428 | 4.5e-62 | Neosartorya fumigata |
| A0A084BLI4_ASPFM/151-428 | 4.5e-62 | Aspergillus fumigatus var. RP-2014 |
| B0Y7I8_ASPFC/151-428 | 4.5e-62 | Neosartorya fumigata |
| S9Z1N2_9CETA/535-778 | 5.4e-62 | Camelus ferus |
| H0Z2G3_TAEGU/44-295 | 4.8e-62 | Taeniopygia guttata |
| G4N9K0_MAGO7/132-392 | 6.4e-62 | Magnaporthe oryzae |
| G7Q3H2_MACFA/51-325 | 4.5e-62 | Macaca fascicularis |
| G7NT58_MACMU/51-325 | 4.5e-62 | Macaca mulatta |
| A0A096N760_PAPAN/51-325 | 4.5e-62 | Papio anubis |
| A0A0D9R8W1_CHLSB/51-325 | 4.5e-62 | Chlorocebus sabaeus |
| A0A0B7BJ11_9EUPU/42-296 | 5.6e-62 | Arion vulgaris |
| A0A095A057_SCHHA/3-217 | 5.0e-62 | Schistosoma haematobium |
| W9XHG5_9EURO/185-468 | 6.3e-62 | Capronia epimyces CBS 606.96 |
| A0A067RD29_ZOONE/2-263 | 5.4e-62 | Zootermopsis nevadensis |
| H2TH82_TAKRU/7-261 | 7.2e-62 | Takifugu rubripes |
| A0A0F0IFG6_ASPPA/141-421 | 7.9e-62 | Aspergillus parasiticus SU-1 |
| A0A0D1YQT5_9EURO/222-505 | 6.5e-62 | Exophiala sideris |
| G4ZGX2_PHYSP/214-491 | 6.2e-62 | Phytophthora sojae |
| C9SGX3_VERA1/124-395 | 7.7e-62 | Verticillium alfalfae |
| H2TH80_TAKRU/35-289 | 7.6e-62 | Takifugu rubripes |
| M3XY49_MUSPF/51-325 | 6.5e-62 | Mustela putorius furo |
| A0A096P8B8_OSTTA/108-389 | 7.0e-62 | Ostreococcus tauri |
| A0A094EZX4_9PEZI/154-432 | 8.1e-62 | Pseudogymnoascus pannorum VKM F-4516 (FW-969) |
| G3YCG1_ASPNA/119-399 | 9.4e-62 | Aspergillus niger |
| F7VTW2_SORMK/216-492 | 5.7e-62 | Sordaria macrospora |
| G4MXS6_MAGO7/202-475 | 8.4e-62 | Magnaporthe oryzae |
| A0A093ZZK6_9PEZI/158-429 | 1.0e-61 | Pseudogymnoascus pannorum VKM F-4281 (FW-2241) |
| R1CY28_EMIHU/330-563 | 1.0e-61 | Emiliania huxleyi CCMP1516 |
| A0A093ZI28_9PEZI/158-429 | 1.1e-61 | Pseudogymnoascus pannorum VKM F-4246 |
| S8B537_PENO1/144-422 | 1.5e-61 | Penicillium oxalicum |
| Q7S7N5_NEUCR/225-501 | 1.4e-61 | Neurospora crassa |
| A0A094BCU1_9PEZI/158-429 | 1.1e-61 | Pseudogymnoascus pannorum VKM F-4513 (FW-928) |
| H1UZA5_COLHI/164-435 | 9.6e-62 | Colletotrichum higginsianum |
| A0A0C3KE31_9HOMO/81-353 | 1.1e-61 | Tulasnella calospora MUT 4182 |
| H3FP35_PRIPA/23-290 | 9.6e-62 | Pristionchus pacificus |
| A0DLL4_PARTE/107-357 | 1.3e-61 | Paramecium tetraurelia |
| F6ZTC0_HORSE/506-742 | 1.2e-61 | Equus caballus |
| A0A0F2MC66_SPOSC/185-456 | 1.1e-61 | Sporothrix schenckii 1099-18 |
| I1CS59_RHIO9/236-493 | 1.3e-61 | Rhizopus delemar |
| A0A0D2BPL7_9EURO/183-468 | 1.3e-61 | Exophiala spinifera |
| A8NBA2_COPC7/89-357 | 1.1e-61 | Coprinopsis cinerea |
| A0A0D8XKQ4_DICVI/50-373 | 1.6e-61 | Dictyocaulus viviparus |
| V9DNE5_9EURO/149-430 | 9.0e-62 | Cladophialophora carrionii CBS 160.54 |
| A0A094H6G2_9PEZI/154-432 | 1.3e-61 | Pseudogymnoascus pannorum VKM F-4519 (FW-2642) |
| A0A094EXQ7_9PEZI/154-432 | 1.3e-61 | Pseudogymnoascus pannorum VKM F-103 |
| L7IW05_MAGOP/128-388 | 1.7e-61 | Magnaporthe oryzae |
| L7HUB3_MAGOY/128-388 | 1.7e-61 | Magnaporthe oryzae |
| A8PIE5_COPC7/138-404 | 1.5e-61 | Coprinopsis cinerea |
| H3G7S8_PHYRM/80-357 | 1.3e-61 | Phytophthora ramorum |
| A0A093YA24_9PEZI/158-429 | 1.7e-61 | Pseudogymnoascus pannorum VKM F-3808 |
| G3W097_SARHA/2-247 | 1.6e-61 | Sarcophilus harrisii |
| A0A0D2EFK4_9EURO/149-430 | 1.1e-61 | Capronia semiimmersa |
| A0A093X6T5_9PEZI/158-429 | 2.0e-61 | Pseudogymnoascus pannorum VKM F-3557 |
| A0A094FDJ7_9PEZI/158-429 | 2.0e-61 | Pseudogymnoascus pannorum VKM F-4514 (FW-929) |
| R7Z225_CONA1/156-427 | 1.8e-61 | Coniosporium apollinis |
| A0A078B814_STYLE/140-395 | 2.6e-61 | Stylonychia lemnae |
| L5KC29_PTEAL/35-306 | 1.8e-61 | Pteropus alecto |
| A0A094HHE4_9PEZI/180-451 | 2.5e-61 | Pseudogymnoascus pannorum VKM F-4518 (FW-2643) |
| W9XUH4_9EURO/252-535 | 2.4e-61 | Capronia coronata CBS 617.96 |
| A0A010RNR1_9PEZI/164-435 | 2.2e-61 | Colletotrichum fioriniae PJ7 |
| G4YLK6_PHYSP/162-418 | 5.6e-61 | Phytophthora sojae |
| A0A094BXB1_9PEZI/154-432 | 2.1e-61 | Pseudogymnoascus pannorum VKM F-4513 (FW-928) |
| E1BJ18_BOVIN/51-325 | 2.3e-61 | Bos taurus |
| L8I5P9_9CETA/51-325 | 2.3e-61 | Bos mutus |
| W5Q8J4_SHEEP/51-325 | 2.2e-61 | Ovis aries |
| K2H5P2_ENTNP/28-264 | 2.2e-61 | Entamoeba nuttalli |
| T0MCG1_COLGC/164-435 | 2.6e-61 | Colletotrichum gloeosporioides |
| A0A060YXE1_ONCMY/19-233 | 4.0e-61 | Oncorhynchus mykiss |
| A0A094FG22_9PEZI/158-429 | 2.8e-61 | Pseudogymnoascus pannorum VKM F-4515 (FW-2607) |
| A8K2N3_HUMAN/72-315 | 2.4e-61 | Homo sapiens |
| A0A094FZD1_9PEZI/158-429 | 2.9e-61 | Pseudogymnoascus pannorum VKM F-4517 (FW-2822) |
| W7N122_GIBM7/197-470 | 3.9e-61 | Gibberella moniliformis |
| W9HHJ1_FUSOX/197-470 | 4.1e-61 | Fusarium oxysporum FOSC 3-a |
| A0A0D2Y4D5_FUSO4/197-470 | 3.2e-61 | Fusarium oxysporum f. sp. lycopersici |
| A0A093ZQ15_9PEZI/154-432 | 2.9e-61 | Pseudogymnoascus pannorum VKM F-4246 |
| W9M4Z0_FUSOX/197-470 | 4.3e-61 | Fusarium oxysporum f. sp. lycopersici MN25 |
| W9L5A8_FUSOX/197-470 | 4.3e-61 | Fusarium oxysporum Fo47 |
| C8V4M8_EMENI/147-427 | 3.2e-61 | Emericella nidulans |
| H0UUE1_CAVPO/51-325 | 6.6e-61 | Cavia porcellus |
| W9WIL8_9EURO/149-430 | 2.5e-61 | Cladophialophora yegresii CBS 114405 |
| L2FEP5_COLGN/164-435 | 4.1e-61 | Colletotrichum gloeosporioides |
| G3UN93_LOXAF/493-729 | 3.8e-61 | Loxodonta africana |
| A0A072PKV3_9EURO/148-426 | 4.1e-61 | Exophiala aquamarina CBS 119918 |
| W5K713_ASTMX/65-317 | 5.6e-61 | Astyanax mexicanus |
| X0FFT5_FUSOX/197-470 | 6.4e-61 | Fusarium oxysporum f. sp. radicis-lycopersici 26381 |
| X0ASK6_FUSOX/197-470 | 6.4e-61 | Fusarium oxysporum f. sp. melonis 26406 |
| X0LT89_FUSOX/197-470 | 6.4e-61 | Fusarium oxysporum f. sp. vasinfectum 25433 |
| W9QA72_FUSOX/197-470 | 6.4e-61 | Fusarium oxysporum f. sp. pisi HDV247 |
| X0CHJ3_FUSOX/197-470 | 6.4e-61 | Fusarium oxysporum f. sp. raphani 54005 |
| N4UUY8_FUSC1/134-407 | 6.6e-61 | Fusarium oxysporum f. sp. cubense |
| A0A094GA94_9PEZI/154-432 | 4.2e-61 | Pseudogymnoascus pannorum VKM F-4517 (FW-2822) |
| S0DME5_GIBF5/182-455 | 6.2e-61 | Gibberella fujikuroi |
| F7C571_CALJA/85-232 | 9.5e-44 | Callithrix jacchus |
| F7C571_CALJA/231-335 | 7.4e-12 | Callithrix jacchus |
| D2VFD5_NAEGR/359-592 | 6.4e-61 | Naegleria gruberi |
| A0A094HHM7_9PEZI/158-429 | 5.7e-61 | Pseudogymnoascus pannorum VKM F-4520 (FW-2644) |
| L5JLI8_PTEAL/94-235 | 2.5e-40 | Pteropus alecto |
| L5JLI8_PTEAL/237-307 | 6.0e-15 | Pteropus alecto |
| W3XEY0_9PEZI/168-439 | 5.9e-61 | Pestalotiopsis fici W106-1 |
| D0A5Q1_TRYB9/51-278 | 1.3e-27 | Trypanosoma brucei gambiense |
| D0A5Q1_TRYB9/666-894 | 2.2e-27 | Trypanosoma brucei gambiense |
| A0A084ASG5_STACH/165-435 | 6.5e-61 | Stachybotrys chartarum IBT 7711 |
| A0A084RP14_STACH/165-435 | 6.5e-61 | Stachybotrys chartarum IBT 40288 |
| A0A084PIL5_STACH/165-435 | 6.5e-61 | Stachybotrys chartarum IBT 40293 |
| A0A0C3LB94_9HOMO/91-370 | 6.1e-61 | Tulasnella calospora MUT 4182 |
| A1CTD8_ASPCL/142-422 | 6.2e-61 | Aspergillus clavatus |
| V3ZT32_LOTGI/45-306 | 5.1e-61 | Lottia gigantea |
| A0A061RGX9_9CHLO/46-290 | 6.6e-61 | Tetraselmis sp. GSL018 |
| Q5B5J7_EMENI/147-427 | 7.1e-61 | Emericella nidulans |
| F6S9S2_ORNAN/499-738 | 7.5e-61 | Ornithorhynchus anatinus |
| A0A0B7JU04_BIOOC/148-425 | 8.7e-61 | Bionectria ochroleuca |
| F6S9U2_ORNAN/521-760 | 7.9e-61 | Ornithorhynchus anatinus |
| L5LSY7_MYODS/118-389 | 6.7e-61 | Myotis davidii |
| A0A0D2KE51_9EURO/151-428 | 8.6e-61 | Fonsecaea multimorphosa CBS 102226 |
| X0LMU1_FUSOX/197-470 | 1.1e-60 | Fusarium oxysporum f. sp. cubense |
| J9ITY9_9SPIT/202-446 | 7.9e-61 | Oxytricha trifallax |
| F9FSI1_FUSOF/197-470 | 1.1e-60 | Fusarium oxysporum |
| X0IG43_FUSOX/197-470 | 1.1e-60 | Fusarium oxysporum f. sp. conglutinans |
| A0A0B1TG78_OESDE/50-138 | 8.6e-22 | Oesophagostomum dentatum |
| A0A0B1TG78_OESDE/140-300 | 1.7e-33 | Oesophagostomum dentatum |
| F7CKQ7_CALJA/85-335 | 8.4e-59 | Callithrix jacchus |
| A4S1N6_OSTLU/235-492 | 8.5e-61 | Ostreococcus lucimarinus |
| A0A0C4E595_MAGP6/73-344 | 9.4e-61 | Magnaporthiopsis poae |
| G5C191_HETGA/104-272 | 1.7e-46 | Heterocephalus glaber |
| G5C191_HETGA/279-326 | 1.8e-08 | Heterocephalus glaber |
| J3NI38_GAGT3/199-470 | 9.0e-61 | Gaeumannomyces graminis var. tritici |
| L8G936_PSED2/158-429 | 1.2e-60 | Pseudogymnoascus destructans |
| I7MFB0_TETTS/538-780 | 1.5e-60 | Tetrahymena thermophila |
| F0XXB9_AURAN/38-288 | 8.7e-61 | Aureococcus anophagefferens |
| A0A017SDV3_9EURO/141-421 | 1.0e-60 | Aspergillus ruber CBS 135680 |
| A0A091SX99_9AVES/546-784 | 1.8e-60 | Pelecanus crispus |
| A0A084QT64_9HYPO/165-435 | 1.4e-60 | Stachybotrys chlorohalonata IBT 40285 |
| J9IGC9_9SPIT/94-326 | 1.1e-60 | Oxytricha trifallax |
| B4I6H0_DROSE/1051-1332 | 2.6e-60 | Drosophila sechellia |
| G3SWI3_LOXAF/51-325 | 1.2e-60 | Loxodonta africana |
| CAN6_RAT/51-325 | 1.2e-60 | Rattus norvegicus |
| E3NCA4_CAERE/345-544 | 1.3e-60 | Caenorhabditis remanei |
| G3V6M4_RAT/51-325 | 1.2e-60 | Rattus norvegicus |
| A0A084GFF4_9PEZI/268-541 | 1.5e-60 | Scedosporium apiospermum |
| L1IGB6_GUITH/174-413 | 1.5e-60 | Guillardia theta CCMP2712 |
| A0A067NU04_PLEOS/100-259 | 1.1e-34 | Pleurotus ostreatus PC15 |
| A0A067NU04_PLEOS/261-331 | 3.8e-20 | Pleurotus ostreatus PC15 |
| J9I4B8_9SPIT/78-318 | 1.3e-60 | Oxytricha trifallax |
| H2PWI0_PONAB/51-325 | 1.2e-60 | Pongo abelii |
| W9X0I5_9EURO/150-428 | 1.7e-60 | Cladophialophora psammophila CBS 110553 |
| A0A0D2HBE6_9EURO/150-428 | 1.7e-60 | Cladophialophora bantiana CBS 173.52 |
| S9Y4T9_9CETA/114-320 | 2.3e-60 | Camelus ferus |
| C4LTE2_ENTHI/28-264 | 1.6e-60 | Entamoeba histolytica |
| N9TPR9_ENTHI/28-264 | 1.6e-60 | Entamoeba histolytica HM-1:IMSS-A |
| M3TQM8_ENTHI/28-264 | 1.6e-60 | Entamoeba histolytica HM-1:IMSS-B |
| M7W874_ENTHI/28-264 | 1.6e-60 | Entamoeba histolytica HM-3:IMSS |
| M2NKT7_BAUCO/163-439 | 1.9e-60 | Baudoinia compniacensis |
| M2Q4E2_ENTHI/28-264 | 1.6e-60 | Entamoeba histolytica KU27 |
| E3QVA5_COLGM/228-500 | 2.3e-60 | Colletotrichum graminicola |
| E5A5Z3_LEPMJ/401-676 | 3.0e-60 | Leptosphaeria maculans |
| A0A0D2CW25_9EURO/150-429 | 2.1e-60 | Cladophialophora immunda |
| Q80VZ1_MOUSE/51-325 | 2.0e-60 | Mus musculus |
| H1V1N5_COLHI/228-501 | 2.1e-60 | Colletotrichum higginsianum |
| CAN6_MOUSE/51-325 | 2.0e-60 | Mus musculus |
| A0A0B7G1H1_THACB/120-389 | 2.1e-60 | Thanatephorus cucumeris |
| T2MI17_HYDVU/209-458 | 3.0e-60 | Hydra vulgaris |
| W4FAD7_9STRA/141-399 | 6.2e-60 | Aphanomyces astaci |
| K9G622_PEND1/141-421 | 3.0e-60 | Penicillium digitatum |
| A0A0D2KJV9_9AGAR/123-400 | 2.5e-60 | Hypholoma sublateritium FD-334 SS-4 |
| A0A068XN81_HYMMI/120-355 | 5.3e-59 | Hymenolepis microstoma |
| G3GW74_CRIGR/109-342 | 2.9e-60 | Cricetulus griseus |
| F0XGL8_GROCL/137-409 | 3.1e-60 | Grosmannia clavigera |
| H0WQR6_OTOGA/51-325 | 2.8e-60 | Otolemur garnettii |
| K8F4A3_9CHLO/146-456 | 3.0e-60 | Bathycoccus prasinos |
| G1NCL8_MELGA/444-681 | 5.7e-60 | Meleagris gallopavo |
| A0A0D9LLD2_9EURO/141-421 | 3.5e-60 | Penicillium solitum |
| E9BIY6_LEIDB/48-275 | 7.5e-28 | Leishmania donovani |
| E9BIY6_LEIDB/666-887 | 2.0e-26 | Leishmania donovani |
| A0A0D2GBQ3_9EURO/152-429 | 4.1e-60 | Fonsecaea pedrosoi CBS 271.37 |
| A0A094K8R2_9PEZI/158-429 | 4.0e-60 | Pseudogymnoascus pannorum VKM F-4519 (FW-2642) |
| A0A094ENX8_9PEZI/158-429 | 4.0e-60 | Pseudogymnoascus pannorum VKM F-103 |
| A0A091D1M9_FUKDA/51-325 | 3.7e-60 | Fukomys damarensis |
| A0A077ZRQ4_STYLE/1447-1714 | 6.8e-60 | Stylonychia lemnae |
| G1NCM0_MELGA/531-768 | 6.6e-60 | Meleagris gallopavo |
| G1NCL4_MELGA/533-770 | 6.6e-60 | Meleagris gallopavo |
| D0MWB9_PHYIT/158-416 | 7.8e-60 | Phytophthora infestans |
| A0A0A2KY04_PENIT/141-421 | 4.9e-60 | Penicillium italicum |
| A0A0F4YXW4_TALEM/139-419 | 5.4e-60 | Rasamsonia emersonii CBS 393.64 |
| H2ZXK7_LATCH/1-209 | 5.1e-60 | Latimeria chalumnae |
| K3VT92_FUSPC/202-475 | 6.1e-60 | Fusarium pseudograminearum |
| R8BWB2_TOGMI/186-462 | 6.2e-60 | Togninia minima |
| A0A0D9MPZ7_ASPFL/141-418 | 7.8e-60 | Aspergillus flavus AF70 |
| A0A078AMF6_STYLE/1942-2232 | 1.4e-59 | Stylonychia lemnae |
| L5M1A3_MYODS/75-242 | 2.9e-45 | Myotis davidii |
| L5M1A3_MYODS/243-290 | 3.1e-09 | Myotis davidii |
| A0A078APY7_STYLE/94-326 | 9.0e-60 | Stylonychia lemnae |
| B6GZZ4_PENCW/141-392 | 7.3e-60 | Penicillium chrysogenum |
| D6WUX3_TRICA/64-323 | 6.1e-60 | Tribolium castaneum |
| A0A0D2BLS6_9EURO/219-504 | 7.8e-60 | Exophiala oligosperma |
| A0A066XIY0_COLSU/224-497 | 8.7e-60 | Colletotrichum sublineola |
| G0R1Z6_ICHMG/64-312 | 7.5e-60 | Ichthyophthirius multifiliis |
| V8P380_OPHHA/80-329 | 1.3e-58 | Ophiophagus hannah |
| C7YW62_NECH7/133-406 | 1.2e-59 | Nectria haematococca |
| A0A0D2LUS7_9AGAR/80-359 | 7.5e-60 | Hypholoma sublateritium FD-334 SS-4 |
| A0A066VAN1_9HOMO/112-395 | 1.5e-59 | Rhizoctonia solani AG-8 WAC10335 |
| G2QQK2_THITE/134-412 | 1.2e-59 | Thielavia terrestris |
| Q387E1_TRYB2/51-278 | 5.5e-27 | Trypanosoma brucei brucei |
| Q387E1_TRYB2/666-894 | 9.0e-27 | Trypanosoma brucei brucei |
| H2LWA2_ORYLA/2-242 | 9.7e-60 | Oryzias latipes |
| T0KSN1_COLGC/234-507 | 1.1e-59 | Colletotrichum gloeosporioides |
| A0A0B2VG92_TOXCA/265-502 | 1.1e-59 | Toxocara canis |
| K3X588_PYTUL/314-594 | 1.5e-59 | Pythium ultimum DAOM BR144 |
| B8M361_TALSN/143-423 | 1.3e-59 | Talaromyces stipitatus |
| J9J954_9SPIT/222-463 | 1.2e-59 | Oxytricha trifallax |
| M7AXP6_CHEMY/71-274 | 2.0e-59 | Chelonia mydas |
| G0QT44_ICHMG/183-426 | 1.6e-59 | Ichthyophthirius multifiliis |
| S7Q9N3_MYOBR/63-287 | 1.5e-59 | Myotis brandtii |
| G3JFS8_CORMM/130-401 | 1.5e-59 | Cordyceps militaris |
| U1LSZ3_ASCSU/265-503 | 1.5e-59 | Ascaris suum |
| F1KW88_ASCSU/265-503 | 1.5e-59 | Ascaris suum |
| A8NAI9_COPC7/158-418 | 2.9e-59 | Coprinopsis cinerea |
| A0A093VHT8_PENMA/143-423 | 1.8e-59 | Talaromyces marneffei PM1 |
| B6Q9R8_PENMQ/143-423 | 1.8e-59 | Penicillium marneffei |
| A8XST3_CAEBR/268-528 | 1.9e-59 | Caenorhabditis briggsae |
| A0A093V158_PENMA/259-539 | 2.3e-59 | Talaromyces marneffei PM1 |
| C7ZA42_NECH7/119-391 | 2.4e-59 | Nectria haematococca |
| F2U474_SALR5/349-585 | 6.0e-59 | Salpingoeca rosetta |
| Q22143_CAEEL/268-526 | 3.2e-59 | Caenorhabditis elegans |
| A0A0D2IRT1_9EURO/149-427 | 3.8e-59 | Rhinocladiella mackenziei CBS 650.93 |
| C5K6D5_PERM5/312-559 | 3.8e-59 | Perkinsus marinus |
| A0A024S926_HYPJE/138-411 | 4.6e-59 | Trichoderma reesei RUT C-30 |
| M7SM61_EUTLA/161-433 | 4.3e-59 | Eutypa lata |
| A0A0A2JTG4_PENEN/141-421 | 4.5e-59 | Penicillium expansum |
| A0A0A2KC76_PENEN/141-421 | 4.5e-59 | Penicillium expansum |
| A0A094LEV8_9AVES/61-308 | 3.9e-59 | Podiceps cristatus |
| A0A0D2UL67_CAPO3/97-355 | 2.7e-58 | Capsaspora owczarzaki ATCC 30864 |
| H2TH83_TAKRU/35-288 | 4.7e-59 | Takifugu rubripes |
| H2TH81_TAKRU/35-288 | 5.0e-59 | Takifugu rubripes |
| E3MCU2_CAERE/267-527 | 5.0e-59 | Caenorhabditis remanei |
| G0SDL8_CHATD/238-518 | 6.0e-59 | Chaetomium thermophilum |
| U1HRS8_ENDPU/154-436 | 6.6e-59 | Endocarpon pusillum) |
| A0A0D2E5A3_9EURO/147-425 | 3.5e-59 | Exophiala xenobiotica |
| A0A0D2EQ63_9EURO/147-425 | 3.6e-59 | Exophiala xenobiotica |
| C6HDJ0_AJECH/133-416 | 3.6e-59 | Ajellomyces capsulatus |
| G0QZK7_ICHMG/305-555 | 5.5e-59 | Ichthyophthirius multifiliis |
| A0A077ZPJ5_STYLE/65-318 | 7.4e-59 | Stylonychia lemnae |
| J9IU68_9SPIT/1651-1907 | 8.9e-59 | Oxytricha trifallax |
| X6PF56_RETFI/126-391 | 6.0e-59 | Reticulomyxa filosa |
| I7MCL9_TETTS/218-472 | 1.2e-58 | Tetrahymena thermophila |
| E4WY71_OIKDI/76-297 | 6.3e-59 | Oikopleura dioica |
| A0A010S4W9_9PEZI/221-494 | 7.0e-59 | Colletotrichum fioriniae PJ7 |
| G3GRB1_CRIGR/103-302 | 1.0e-58 | Cricetulus griseus |
| N6UHV2_DENPD/299-552 | 8.5e-59 | Dendroctonus ponderosae |
| A0A0D2C5L7_9EURO/150-428 | 9.7e-59 | Exophiala oligosperma |
| G0RN45_HYPJQ/138-411 | 9.3e-59 | Hypocrea jecorina |
| A0A093YXA7_9PEZI/75-328 | 7.8e-52 | Pseudogymnoascus pannorum VKM F-3808 |
| G9MQ56_HYPVG/136-407 | 9.5e-59 | Hypocrea virens |
| W2QVV5_PHYPN/160-415 | 1.9e-58 | Phytophthora parasitica |
| N4VIS8_COLOR/221-494 | 9.2e-59 | Colletotrichum orbiculare |
| K9J5S4_DESRO/3-206 | 9.3e-59 | Desmodus rotundus |
| W2QWQ4_PHYPN/160-415 | 2.0e-58 | Phytophthora parasitica |
| G0MED9_CAEBE/284-526 | 1.1e-58 | Caenorhabditis brenneri |
| J9P2E8_CANFA/85-305 | 9.1e-59 | Canis familiaris |
| F6Q6Z5_MACMU/51-324 | 1.0e-58 | Macaca mulatta |
| W2FVU6_PHYPR/160-415 | 2.8e-58 | Phytophthora parasitica |
| W2ME90_PHYPR/160-415 | 2.8e-58 | Phytophthora parasitica |
| W2K886_PHYPR/160-415 | 2.8e-58 | Phytophthora parasitica |
| W2W1B6_PHYPR/160-415 | 2.8e-58 | Phytophthora parasitica CJ01A1 |
| V9E781_PHYPR/160-415 | 2.8e-58 | Phytophthora parasitica P1569 |
| W2I243_PHYPR/160-415 | 2.8e-58 | Phytophthora parasitica |
| W2YBI8_PHYPR/160-415 | 2.8e-58 | Phytophthora parasitica P10297 |
| G9M038_MESVI/8-211 | 1.2e-58 | Mesostigma viride |
| W2W1R2_PHYPR/160-415 | 3.0e-58 | Phytophthora parasitica CJ01A1 |
| W2MEC8_PHYPR/160-415 | 3.0e-58 | Phytophthora parasitica |
| W2KAL6_PHYPR/160-415 | 3.0e-58 | Phytophthora parasitica |
| W2FVG0_PHYPR/160-415 | 3.0e-58 | Phytophthora parasitica |
| V9E812_PHYPR/160-415 | 3.0e-58 | Phytophthora parasitica P1569 |
| W2I267_PHYPR/160-415 | 3.0e-58 | Phytophthora parasitica |
| A0A074ZCY2_9TREM/62-234 | 1.1e-36 | Opisthorchis viverrini |
| A0A074ZCY2_9TREM/241-410 | 7.6e-16 | Opisthorchis viverrini |
| W2YDZ9_PHYPR/160-415 | 3.0e-58 | Phytophthora parasitica P10297 |
| W3X5V0_9PEZI/203-476 | 1.5e-58 | Pestalotiopsis fici W106-1 |
| V5FP14_BYSSN/244-524 | 1.5e-58 | Byssochlamys spectabilis |
| A0A080Z973_PHYPR/450-705 | 3.3e-58 | Phytophthora parasitica P1976 |
| G3VKC9_SARHA/3-221 | 1.9e-58 | Sarcophilus harrisii |
| E4XVE4_OIKDI/125-390 | 1.6e-58 | Oikopleura dioica |
| J3PCH4_GAGT3/179-452 | 2.0e-58 | Gaeumannomyces graminis var. tritici |
| A0A080Z972_PHYPR/450-705 | 3.5e-58 | Phytophthora parasitica P1976 |
| A0A0D1YI29_9EURO/149-427 | 1.9e-58 | Exophiala sideris |
| W7ENP8_COCVI/174-450 | 3.6e-58 | Bipolaris victoriae FI3 |
| R0JFH7_ANAPL/231-492 | 2.1e-58 | Anas platyrhynchos |
| I3JJW9_ORENI/3-203 | 1.9e-58 | Oreochromis niloticus |
| W6Y662_COCCA/174-450 | 3.7e-58 | Bipolaris zeicola 26-R-13 |
| A0A0C4DLV2_MAGP6/178-451 | 2.4e-58 | Magnaporthiopsis poae |
| M2SL48_COCH5/174-452 | 3.1e-58 | Cochliobolus heterostrophus |
| A0A0D2B9Y3_9EURO/147-425 | 2.2e-58 | Exophiala spinifera |
| Q1K520_NEUCR/184-454 | 2.8e-58 | Neurospora crassa |
| E3N747_CAERE/53-221 | 4.8e-43 | Caenorhabditis remanei |
| E3N747_CAERE/227-280 | 7.1e-09 | Caenorhabditis remanei |
| Q8NIS6_NEUCS/184-454 | 2.9e-58 | Neurospora crassa |
| U6NQ05_HAECO/257-491 | 2.5e-58 | Haemonchus contortus |
| I7ME41_TETTS/1433-1677 | 3.5e-58 | Tetrahymena thermophila) |
| M2SJ68_COCSN/174-452 | 3.6e-58 | Cochliobolus sativus |
| E4YR76_OIKDI/125-390 | 2.4e-58 | Oikopleura dioica |
| A0A0D3BN76_BRAOL/952-1065 | 4.1e-35 | Brassica oleracea var. oleracea |
| A0A0D3BN76_BRAOL/1066-1140 | 1.9e-17 | Brassica oleracea var. oleracea |
| F7VZA0_SORMK/164-434 | 3.6e-58 | Sordaria macrospora |
| T1I8H2_RHOPR/56-302 | 3.9e-58 | Rhodnius prolixus |
| A0A078AK08_STYLE/89-342 | 2.5e-58 | Stylonychia lemnae |
| B0EE72_ENTDS/41-265 | 2.6e-58 | Entamoeba dispar |
| F8MRL8_NEUT8/189-459 | 3.8e-58 | Neurospora tetrasperma |
| G0QS43_ICHMG/50-295 | 5.0e-58 | Ichthyophthirius multifiliis |
| F7CD99_CALJA/75-328 | 4.0e-58 | Callithrix jacchus |
| F7BYI1_CALJA/75-323 | 3.6e-58 | Callithrix jacchus |
| V9KM05_CALMI/1-188 | 4.0e-58 | Callorhinchus milii |
| G9NYA4_HYPAI/137-407 | 4.9e-58 | Hypocrea atroviridis |
| G1NFR6_MELGA/232-493 | 4.9e-58 | Meleagris gallopavo |
| A0A016T8D9_9BILA/31-266 | 4.4e-58 | Ancylostoma ceylanicum |
| U3K377_FICAL/217-478 | 5.3e-58 | Ficedula albicollis |
| A0CF53_PARTE/339-584 | 6.3e-58 | Paramecium tetraurelia |
| A0A0B7K3S3_BIOOC/138-411 | 3.6e-58 | Bionectria ochroleuca |
| A0A024FZZ2_9STRA/974-1224 | 1.5e-57 | Albugo candida |
| A0A093R6V9_PHACA/1-185 | 6.4e-58 | Phalacrocorax carbo |
| A0A0B1SME5_OESDE/145-380 | 7.7e-58 | Oesophagostomum dentatum |
| A0A067QUQ1_ZOONE/281-544 | 7.8e-58 | Zootermopsis nevadensis |
| A0A016Q2K1_GIBZA/140-411 | 7.0e-58 | Gibberella zeae |
| K3VUA5_FUSPC/140-411 | 7.0e-58 | Fusarium pseudograminearum |
| A0A091DR09_FUKDA/217-478 | 6.9e-58 | Fukomys damarensis |
| A0A024UC93_9STRA/141-396 | 3.1e-57 | Aphanomyces invadans |
| A0A024UD12_9STRA/141-396 | 3.1e-57 | Aphanomyces invadans |
| A0A024UAR5_9STRA/141-396 | 3.1e-57 | Aphanomyces invadans |
| G0S3K1_CHATD/160-435 | 7.6e-58 | Chaetomium thermophilum |
| A0A088RVX6_9TRYP/48-271 | 1.1e-25 | Leishmania panamensis |
| A0A088RVX6_9TRYP/671-898 | 3.5e-26 | Leishmania panamensis |
| G0UZA3_TRYCI/56-278 | 1.5e-27 | Trypanosoma congolense |
| G0UZA3_TRYCI/671-891 | 2.5e-24 | Trypanosoma congolense |
| G3VKD0_SARHA/1-222 | 9.4e-58 | Sarcophilus harrisii |
| W6YSA0_COCMI/174-452 | 1.4e-57 | Bipolaris oryzae ATCC 44560 |
| A0A0D8XKD9_DICVI/261-522 | 9.0e-58 | Dictyocaulus viviparus |
| A0A016T8A9_9BILA/310-545 | 1.0e-57 | Ancylostoma ceylanicum |
| G1SSR4_RABIT/261-522 | 9.4e-58 | Oryctolagus cuniculus |
| D2VVS6_NAEGR/688-945 | 1.2e-57 | Naegleria gruberi |
| A8PU52_BRUMA/198-427 | 1.0e-57 | Brugia malayi |
| I3MNV8_SPETR/23-284 | 9.3e-58 | Spermophilus tridecemlineatus |
| I3LBF0_PIG/261-522 | 9.9e-58 | Sus scrofa |
| A0A016T7G1_9BILA/346-581 | 1.1e-57 | Ancylostoma ceylanicum |
| K7G8Q3_PELSI/217-478 | 1.0e-57 | Pelodiscus sinensis |
| G4LXK2_SCHMA/1021-1280 | 1.6e-57 | Schistosoma mansoni |
| G4LXK3_SCHMA/1046-1305 | 1.6e-57 | Schistosoma mansoni |
| A2FEL7_TRIVA/1079-1331 | 1.2e-57 | Trichomonas vaginalis |
| K7G8R7_PELSI/261-522 | 1.2e-57 | Pelodiscus sinensis |
| G1SUQ2_RABIT/75-316 | 3.0e-57 | Oryctolagus cuniculus |
| A0A0F4GF91_9PEZI/152-422 | 1.5e-57 | Zymoseptoria brevis |
| H0XA63_OTOGA/261-522 | 1.4e-57 | Otolemur garnettii |
| E0VKU5_PEDHC/121-381 | 1.3e-57 | Pediculus humanus subsp. corporis |
| U6DGD5_NEOVI/261-522 | 1.4e-57 | Neovison vison |
| Q499T5_RAT/261-522 | 1.5e-57 | Rattus norvegicus |
| F7AE26_HORSE/261-523 | 1.4e-57 | Equus caballus |
| H0VTP5_CAVPO/260-521 | 1.4e-57 | Cavia porcellus |
| E1BIX3_BOVIN/261-522 | 1.5e-57 | Bos taurus |
| G5BUL7_HETGA/227-488 | 1.5e-57 | Heterocephalus glaber |
| L5L0F2_PTEAL/261-522 | 1.5e-57 | Pteropus alecto |
| W5Q043_SHEEP/217-478 | 1.5e-57 | Ovis aries |
| E4YQT3_OIKDI/92-354 | 1.6e-57 | Oikopleura dioica |
| F7H3Z3_MACMU/261-522 | 1.6e-57 | Macaca mulatta |
| H2PBE5_PONAB/259-520 | 1.6e-57 | Pongo abelii |
| E2R0F7_CANFA/261-522 | 1.6e-57 | Canis familiaris |
| D2H1G2_AILME/261-522 | 1.6e-57 | Ailuropoda melanoleuca |
| L8HMG7_9CETA/228-489 | 1.6e-57 | Bos mutus |
| M3XEV5_FELCA/261-522 | 1.6e-57 | Felis catus |
| F7D1T2_CALJA/261-522 | 1.6e-57 | Callithrix jacchus |
| A0A0D9RCS0_CHLSB/261-522 | 1.6e-57 | Chlorocebus sabaeus |
| A0A096MKK7_PAPAN/261-522 | 1.6e-57 | Papio anubis |
| U3FH04_CALJA/265-526 | 1.6e-57 | Callithrix jacchus |
| CAN7_MOUSE/261-522 | 1.7e-57 | Mus musculus |
| M1EHK5_MUSPF/192-453 | 1.6e-57 | Mustela putorius furo |
| D2V2W6_NAEGR/234-508 | 1.7e-57 | Naegleria gruberi |
| G0R592_ICHMG/15-257 | 2.2e-57 | Ichthyophthirius multifiliis |
| B0FGU2_PIG/261-522 | 1.8e-57 | Sus scrofa ussuricus |
| A0A060Z092_ONCMY/14-205 | 1.5e-57 | Oncorhynchus mykiss |
| R1F5H3_EMIHU/176-411 | 1.9e-57 | Emiliania huxleyi CCMP1516 |
| G7YT17_CLOSI/67-331 | 1.5e-57 | Clonorchis sinensis |
| M3XRN3_MUSPF/261-522 | 2.0e-57 | Mustela putorius furo |
| G7NY08_MACFA/259-520 | 2.0e-57 | Macaca fascicularis |
| H9ESM2_MACMU/261-522 | 2.0e-57 | Macaca mulatta |
| X0ABI2_FUSOX/153-282 | 1.6e-12 | Fusarium oxysporum f. sp. melonis 26406 |
| X0ABI2_FUSOX/276-503 | 2.8e-40 | Fusarium oxysporum f. sp. melonis 26406 |
| E3RQG6_PYRTT/181-457 | 1.4e-57 | Pyrenophora teres f. teres |
| R1G650_BOTPV/177-448 | 2.3e-57 | Botryosphaeria parva |
| G1KB21_ANOCA/259-520 | 2.2e-57 | Anolis carolinensis |
| A0A0B7JWG9_BIOOC/124-397 | 2.5e-57 | Bionectria ochroleuca |
| S0E9C4_GIBF5/147-419 | 2.5e-57 | Gibberella fujikuroi |
| A7RRP7_NEMVE/262-523 | 2.3e-57 | Nematostella vectensis |
| A0A044UNL6_ONCVO/198-427 | 2.5e-57 | Onchocerca volvulus |
| G1QME6_NOMLE/261-522 | 2.6e-57 | Nomascus leucogenys |
| W9IGA6_FUSOX/112-384 | 2.7e-57 | Fusarium oxysporum FOSC 3-a |
| A0A094ZP27_SCHHA/205-459 | 2.6e-57 | Schistosoma haematobium |
| H2WQA0_CAEJA/210-453 | 3.3e-57 | Caenorhabditis japonica |
| X0MNM1_FUSOX/147-419 | 2.9e-57 | Fusarium oxysporum f. sp. vasinfectum 25433 |
| W9K9A7_FUSOX/132-404 | 2.9e-57 | Fusarium oxysporum Fo47 |
| X0AJ79_FUSOX/132-404 | 2.9e-57 | Fusarium oxysporum f. sp. melonis 26406 |
| N4V0G0_FUSC1/147-419 | 2.9e-57 | Fusarium oxysporum f. sp. cubense |
| X0H8V5_FUSOX/132-404 | 2.9e-57 | Fusarium oxysporum f. sp. radicis-lycopersici |
| W9LII2_FUSOX/132-404 | 2.9e-57 | Fusarium oxysporum f. sp. lycopersici MN25 |
| X0HRL6_FUSOX/147-419 | 2.9e-57 | Fusarium oxysporum f. sp. conglutinans |
| X0DBL9_FUSOX/147-419 | 2.9e-57 | Fusarium oxysporum f. sp. raphani 54005 |
| W9PLY4_FUSOX/147-419 | 2.9e-57 | Fusarium oxysporum f. sp. pisi HDV247 |
| W9IB56_FUSOX/147-419 | 2.9e-57 | Fusarium oxysporum FOSC 3-a |
| W9K716_FUSOX/147-419 | 2.9e-57 | Fusarium oxysporum Fo47 |
| X0AIE7_FUSOX/147-419 | 2.9e-57 | Fusarium oxysporum f. sp. melonis 26406 |
| X0G3A1_FUSOX/147-419 | 2.9e-57 | Fusarium oxysporum f. sp. radicis-lycopersici |
| F9F4I6_FUSOF/182-454 | 3.2e-57 | Fusarium oxysporum |
| W9LQI3_FUSOX/147-419 | 2.9e-57 | Fusarium oxysporum f. sp. lycopersici MN25 |
| S7MRR1_MYOBR/313-574 | 3.7e-57 | Myotis brandtii |
| I7MGN1_TETTS/154-396 | 4.2e-57 | Tetrahymena thermophila |
| W7MZU0_GIBM7/163-435 | 3.8e-57 | Gibberella moniliformis |
| B2B059_PODAN/192-463 | 4.8e-57 | Podospora anserina |
| V9KPY7_CALMI/1-184 | 4.5e-57 | Callorhinchus milii |
| X0JCV6_FUSOX/99-371 | 4.4e-57 | Fusarium oxysporum f. sp. cubense |
| A0A0C2IUQ4_9PEZI/173-448 | 5.0e-57 | Sporothrix brasiliensis 5110 |
| A0A090D694_PODAN/250-521 | 5.4e-57 | Podospora anserina |
| X0B6Z6_FUSOX/167-437 | 4.3e-57 | Fusarium oxysporum f. sp. raphani 54005 |
| X0GZ53_FUSOX/167-437 | 4.3e-57 | Fusarium oxysporum f. sp. conglutinans |
| A0A0C3H0B9_9PEZI/81-362 | 4.4e-57 | Oidiodendron maius Zn |
| X0JCY2_FUSOX/147-419 | 4.8e-57 | Fusarium oxysporum f. sp. cubense |
| A0A0F2MEZ5_SPOSC/173-448 | 5.4e-57 | Sporothrix schenckii 1099-18 |
| N1S7L9_FUSC4/149-421 | 4.9e-57 | Fusarium oxysporum f. sp. cubense |
| CAN7_PIG/261-522 | 4.7e-57 | Sus scrofa |
| U7PNU6_SPOS1/173-448 | 5.5e-57 | Sporothrix schenckii |
| Q6DCQ4_XENLA/66-345 | 1.8e-55 | Xenopus laevis |
| R0JZ45_SETT2/135-414 | 6.7e-57 | Setosphaeria turcica |
| A0A0B7KF04_BIOOC/118-384 | 5.5e-57 | Bionectria ochroleuca |
| J9I0Q5_9SPIT/1940-2238 | 7.0e-57 | Oxytricha trifallax |
| G1LQB8_AILME/261-523 | 5.2e-57 | Ailuropoda melanoleuca |
| Q7Z479_HUMAN/261-522 | 5.2e-57 | Homo sapiens |
| G1T6R7_RABIT/49-278 | 4.4e-57 | Oryctolagus cuniculus |
| CAN7_HUMAN/261-522 | 5.5e-57 | Homo sapiens |
| H2QM47_PANTR/261-522 | 5.5e-57 | Pan troglodytes |
| G0U9Y5_TRYVY/53-211 | 1.9e-22 | Trypanosoma vivax |
| G0U9Y5_TRYVY/443-662 | 1.5e-28 | Trypanosoma vivax |
| C0S2F0_PARBP/142-432 | 6.0e-57 | Paracoccidioides brasiliensis |
| T2M2K0_HYDVU/188-447 | 6.8e-57 | Hydra vulgaris |
| Q239Z2_TETTS/653-916 | 7.4e-57 | Tetrahymena thermophila |
| H2ML81_ORYLA/274-535 | 7.1e-57 | Oryzias latipes |
| R8BPT7_TOGMI/191-465 | 7.3e-57 | Togninia minima |
| A0A0D1WMC7_9EURO/147-425 | 7.2e-57 | Exophiala mesophila |
| A0A0A0HSB3_PARBD/137-427 | 7.4e-57 | Paracoccidioides brasiliensis Pb18 |
| A0BHR8_PARTE/120-361 | 8.7e-57 | Paramecium tetraurelia |
| A0A016VQX5_9BILA/264-525 | 7.3e-57 | Ancylostoma ceylanicum |
| W5L380_ASTMX/51-253 | 6.3e-57 | Astyanax mexicanus |
| A0A0A1UET8_ENTIV/28-264 | 7.9e-57 | Entamoeba invadens IP1 |
| G3T4W2_LOXAF/236-498 | 9.1e-57 | Loxodonta africana |
| F7ERL1_MONDO/261-522 | 8.4e-57 | Monodelphis domestica |
| E1BY84_CHICK/261-523 | 9.4e-57 | Gallus gallus |
| A0A0D2Y2X3_FUSO4/120-391 | 1.0e-56 | Fusarium oxysporum f. sp. lycopersici |
| A0A091JQ72_9AVES/78-320 | 1.2e-56 | Egretta garzetta |
| A0A0C3QR54_9HOMO/94-375 | 1.0e-56 | Tulasnella calospora MUT 4182 |
| G5C7Y7_HETGA/2-270 | 9.7e-57 | Heterocephalus glaber |
| G1NYI9_MYOLU/261-523 | 1.0e-56 | Myotis lucifugus |
| F9XDQ3_ZYMTI/64-334 | 1.2e-56 | Zymoseptoria tritici |
| W4G3V3_9STRA/314-577 | 1.1e-56 | Aphanomyces astaci |
| Q4CW01_TRYCC/51-276 | 1.4e-26 | Trypanosoma cruzi |
| Q4CW01_TRYCC/668-891 | 3.2e-24 | Trypanosoma cruzi |
| W5M127_LEPOC/261-522 | 1.1e-56 | Lepisosteus oculatus |
| F8MMT0_NEUT8/161-435 | 1.2e-56 | Neurospora tetrasperma |
| F6VHK8_XENTR/75-191 | 5.5e-32 | Xenopus tropicalis |
| F6VHK8_XENTR/248-325 | 8.1e-19 | Xenopus tropicalis |
| G2QGF6_THIHA/159-434 | 1.2e-56 | Thielavia heterothallica |
| B2RAM2_HUMAN/261-522 | 1.2e-56 | Homo sapiens |
| H2YUD5_CIOSA/261-520 | 1.3e-56 | Ciona savignyi |
| E3MHJ1_CAERE/192-431 | 1.5e-56 | Caenorhabditis remanei |
| G2R921_THITE/159-434 | 1.3e-56 | Thielavia terrestris |
| A0BQD1_PARTE/394-647 | 9.9e-57 | Paramecium tetraurelia |
| M4AS68_XIPMA/269-530 | 1.9e-56 | Xiphophorus maculatus |
| Q5GCP6_GECLA/112-312 | 1.5e-56 | Gecarcinus lateralis |
| J9HX68_9SPIT/89-343 | 1.7e-56 | Oxytricha trifallax |
| H2VXF8_CAEJA/270-530 | 1.7e-56 | Caenorhabditis japonica |
| E1FJT3_LOALO/191-420 | 1.8e-56 | Loa loa |
| E9PU30_RAT/261-522 | 1.9e-56 | Rattus norvegicus |
| S3CD37_OPHP1/178-453 | 2.8e-56 | Ophiostoma piceae |
| Q6NVT4_XENTR/217-478 | 2.4e-56 | Xenopus tropicalis |
| T0MJ41_9CETA/75-275 | 2.3e-56 | Camelus ferus |
| F7AXH7_XENTR/264-525 | 2.6e-56 | Xenopus tropicalis |
| H9JX46_BOMMO/237-500 | 2.5e-56 | Bombyx mori |
| G4TER6_PIRID/93-337 | 2.5e-56 | Piriformospora indica |
| A0A0A2F4T5_9PORP/194-467 | 2.3e-56 | Porphyromonas gulae |
| K2RZ25_MACPH/26-301 | 5.1e-56 | Macrophomina phaseolina |
| A6RA87_AJECN/134-431 | 6.4e-56 | Ajellomyces capsulatus |
| T0S7Q2_9STRA/146-402 | 5.0e-56 | Saprolegnia diclina VS20 |
| A0A0B7JZJ5_BIOOC/161-429 | 3.4e-56 | Bionectria ochroleuca |
| W5LHE7_ASTMX/261-522 | 3.5e-56 | Astyanax mexicanus |
| G2XC50_VERDV/167-441 | 3.5e-56 | Verticillium dahliae |
| F2TZL5_SALR5/287-544 | 3.3e-56 | Salpingoeca rosetta |
| A0A084G5T1_9PEZI/153-428 | 3.9e-56 | Scedosporium apiospermum |
| M7BBG8_CHEMY/228-489 | 4.3e-56 | Chelonia mydas |
| C1MTG3_MICPC/259-601 | 4.6e-56 | Micromonas pusilla |
| A0A067BPI6_SAPPC/128-376 | 4.8e-56 | Saprolegnia parasitica CBS 223.65 |
| T1JFC5_STRMM/84-335 | 4.7e-56 | Strigamia maritima |
| W2RW43_9EURO/127-417 | 1.3e-55 | Cyphellophora europaea CBS 101466 |
| U6PDJ0_HAECO/279-540 | 5.0e-56 | Haemonchus contortus |
| B0WDD1_CULQU/168-431 | 1.4e-55 | Culex quinquefasciatus |
| G9N0H7_HYPVG/69-343 | 4.8e-56 | Hypocrea virens |
| W9LCW7_FUSOX/167-437 | 5.2e-56 | Fusarium oxysporum f. sp. lycopersici MN25 |
| A0A061IAA0_CRIGR/67-180 | 1.8e-29 | Cricetulus griseus |
| A0A061IAA0_CRIGR/186-267 | 8.6e-21 | Cricetulus griseus |
| A0A060Z0J7_ONCMY/39-234 | 4.7e-56 | Oncorhynchus mykiss |
| S3C735_OPHP1/268-538 | 1.1e-55 | Ophiostoma piceae |
| I3JI04_ORENI/272-533 | 6.6e-56 | Oreochromis niloticus |
| F6S3F9_MACMU/72-256 | 5.7e-56 | Macaca mulatta |
| A0A067BS78_SAPPC/286-534 | 5.8e-56 | Saprolegnia parasitica CBS 223.65 |
| W9JI54_FUSOX/165-437 | 6.0e-56 | Fusarium oxysporum Fo47 |
| G3NA95_GASAC/270-531 | 8.3e-56 | Gasterosteus aculeatus |
| Q8X0W5_NEUCS/225-452 | 2.3e-55 | Neurospora crassa |
| A0A063BR55_9HYPO/228-500 | 7.3e-56 | Ustilaginoidea virens |
| L8Y6V3_TUPCH/64-309 | 7.1e-56 | Tupaia chinensis |
| A0A0B2W5Z6_TOXCA/262-523 | 9.1e-56 | Toxocara canis |
| W2T575_NECAM/264-516 | 8.3e-56 | Necator americanus |
| Q7RZW0_NEUCR/161-435 | 9.4e-56 | Neurospora crassa |
| A0A090L2H9_STRRB/167-429 | 1.1e-55 | Strongyloides ratti |
| C6KE09_LITVA/1-180 | 7.6e-56 | Litopenaeus vannamei |
| H2SDL0_TAKRU/266-527 | 1.2e-55 | Takifugu rubripes |
| X0GP17_FUSOX/165-437 | 9.1e-56 | Fusarium oxysporum f. sp. conglutinans race 2 54008 |
| H3C9S6_TETNG/266-527 | 1.2e-55 | Tetraodon nigroviridis |
| A0A087YKA6_POEFO/269-531 | 1.3e-55 | Poecilia formosa |
| Q2H809_CHAGB/160-434 | 1.0e-55 | Chaetomium globosum |
| Q23D00_TETTS/414-660 | 9.7e-56 | Tetrahymena thermophila |
| H3D625_TETNG/276-537 | 1.3e-55 | Tetraodon nigroviridis |
| A0A0E9KRT7_9HYPO/228-500 | 1.1e-55 | Ustilaginoidea virens |
| B4DNR4_HUMAN/72-256 | 1.1e-55 | Homo sapiens |
| E7ESS6_HUMAN/72-256 | 1.1e-55 | Homo sapiens |
| Q6PIV8_HUMAN/72-256 | 1.1e-55 | Homo sapiens |
| F7GPP9_CALJA/1-222 | 1.3e-55 | Callithrix jacchus |
| O44903_CAEEL/125-374 | 1.2e-55 | Caenorhabditis elegans |
| A7EPM3_SCLS1/186-471 | 1.3e-55 | Sclerotinia sclerotiorum |
| M4BYT2_HYAAE/274-548 | 1.7e-55 | Hyaloperonospora arabidopsidis |
| J9IKF2_9SPIT/79-324 | 1.8e-55 | Oxytricha trifallax |
| A0A024N5X4_BRUMA/196-395 | 1.2e-55 | Brugia malayi |
| R1BX75_EMIHU/206-516 | 1.3e-55 | Emiliania huxleyi CCMP1516 |
| E9AYV1_LEIMU/47-274 | 1.7e-25 | Leishmania mexicana |
| E9AYV1_LEIMU/668-890 | 4.1e-24 | Leishmania mexicana |
| G0RSR8_HYPJQ/156-431 | 1.5e-55 | Hypocrea jecorina |
| E4YDB9_OIKDI/76-297 | 2.1e-54 | Oikopleura dioica |
| W9MZE3_FUSOX/153-428 | 1.8e-55 | Fusarium oxysporum f. sp. lycopersici MN25 |
| W9KGY8_FUSOX/153-428 | 1.8e-55 | Fusarium oxysporum Fo47 |
| X0HHZ3_FUSOX/153-428 | 1.8e-55 | Fusarium oxysporum f. sp. radicis-lycopersici |
| A0A0C3D7P3_9PEZI/160-436 | 1.8e-55 | Oidiodendron maius Zn |
| W9NIL5_FUSOX/167-437 | 1.8e-55 | Fusarium oxysporum f. sp. pisi HDV247 |
| X0BJP6_FUSOX/167-437 | 1.8e-55 | Fusarium oxysporum f. sp. raphani 54005 |
| F6ZU38_CALJA/72-256 | 2.2e-55 | Callithrix jacchus |
| A0A067PUE5_9HOMO/111-303 | 6.8e-39 | Jaapia argillacea MUCL 33604 |
| A0A067PUE5_9HOMO/309-360 | 1.1e-10 | Jaapia argillacea MUCL 33604 |
| H9GX91_DANRE/263-524 | 2.3e-55 | Danio rerio |
| B4F6P1_DANRE/263-524 | 2.3e-55 | Danio rerio |
| W9LL51_FUSOX/167-437 | 2.6e-55 | Fusarium oxysporum f. sp. lycopersici MN25 |
| A0A0B7ALE4_9EUPU/33-294 | 2.3e-55 | Arion vulgaris |
| U5ESS8_9DIPT/163-426 | 2.4e-55 | Corethrella appendiculata |
| A0A078B2J0_STYLE/82-327 | 2.9e-55 | Stylonychia lemnae |
| A0A084QEW8_9HYPO/39-314 | 2.9e-55 | Stachybotrys chlorohalonata IBT 40285 |
| D2V2Y0_NAEGR/42-282 | 9.9e-55 | Naegleria gruberi |
| A0A084RN48_STACH/48-323 | 2.9e-55 | Stachybotrys chartarum IBT 40288 |
| V4AD60_LOTGI/273-534 | 2.8e-55 | Lottia gigantea |
| A0A024RWV2_HYPJE/156-431 | 3.0e-55 | Trichoderma reesei RUT C-30 |
| N4VGZ7_COLOR/154-429 | 3.1e-55 | Colletotrichum orbiculare |
| W9LBH1_FUSOX/153-428 | 3.2e-55 | Fusarium oxysporum f. sp. lycopersici MN25 |
| X0EVD6_FUSOX/153-428 | 3.2e-55 | Fusarium oxysporum f. sp. radicis-lycopersici 26381 |
| A8XVI8_CAEBR/218-457 | 7.7e-55 | Caenorhabditis briggsae |
| X0FPN6_FUSOX/165-437 | 3.2e-55 | Fusarium oxysporum f. sp. radicis-lycopersici 26381 |
| F1LIM7_CAEEL/86-325 | 3.4e-55 | Caenorhabditis elegans |
| A0A084PDT6_STACH/183-458 | 3.8e-55 | Stachybotrys chartarum IBT 40293 |
| A0A084B4L7_STACH/183-458 | 3.8e-55 | Stachybotrys chartarum IBT 7711 |
| A0A060XRV2_ONCMY/270-531 | 3.8e-55 | Oncorhynchus mykiss |
| C0H9M1_SALSA/270-531 | 4.0e-55 | Salmo salar |
| W3WTG9_9PEZI/217-492 | 5.0e-55 | Pestalotiopsis fici W106-1 |
| G3VKK8_SARHA/261-523 | 4.7e-55 | Sarcophilus harrisii |
| G5EEZ6_CAEEL/188-427 | 4.8e-55 | Caenorhabditis elegans |
| U1NCE0_ASCSU/259-520 | 4.9e-55 | Ascaris suum |
| A7EU64_SCLS1/168-444 | 6.2e-55 | Sclerotinia sclerotiorum |
| X0A2D5_FUSOX/165-437 | 5.1e-55 | Fusarium oxysporum f. sp. melonis 26406 |
| E9GL65_DAPPU/249-511 | 5.6e-55 | Daphnia pulex |
| L5KEJ3_PTEAL/75-274 | 5.3e-55 | Pteropus alecto |
| B9GY59_POPTR/1710-1906 | 7.2e-55 | Populus trichocarpa |
| M2LN67_BAUCO/22-294 | 6.9e-55 | Baudoinia compniacensis |
| L8H2X5_ACACA/66-303 | 9.9e-55 | Acanthamoeba castellanii |
| T0RFR2_9STRA/298-549 | 6.8e-55 | Saprolegnia diclina VS20 |
| H2TH84_TAKRU/7-248 | 9.2e-55 | Takifugu rubripes |
| X0MVP9_FUSOX/153-428 | 8.4e-55 | Fusarium oxysporum f. sp. vasinfectum 25433 |
| E1GED5_LOALO/229-490 | 9.1e-55 | Loa loa |
| W9IY22_FUSOX/153-428 | 8.4e-55 | Fusarium oxysporum FOSC 3-a |
| X0C1Y7_FUSOX/153-428 | 8.4e-55 | Fusarium oxysporum f. sp. raphani 54005 |
| F9FKX4_FUSOF/153-428 | 8.4e-55 | Fusarium oxysporum |
| W9Q109_FUSOX/153-428 | 8.4e-55 | Fusarium oxysporum f. sp. pisi HDV247 |
| V8NMN4_OPHHA/526-747 | 1.3e-54 | Ophiophagus hannah |
| X0JQX0_FUSOX/153-428 | 8.4e-55 | Fusarium oxysporum f. sp. cubense |
| A0A0D2XEW5_FUSO4/153-428 | 8.4e-55 | Fusarium oxysporum f. sp. lycopersici |
| S7PV25_GLOTA/102-366 | 7.8e-55 | Gloeophyllum trabeum |
| A0A0C3HUA9_9PEZI/159-442 | 7.9e-55 | Oidiodendron maius Zn |
| F1KU58_ASCSU/259-520 | 8.8e-55 | Ascaris suum |
| F6SGI6_CIOIN/186-445 | 1.1e-54 | Ciona intestinalis |
| F6SGG4_CIOIN/177-436 | 1.1e-54 | Ciona intestinalis |
| X0BAI4_FUSOX/165-438 | 9.9e-55 | Fusarium oxysporum f. sp. raphani 54005 |
| G3QSZ1_GORGO/75-330 | 4.6e-54 | Gorilla gorilla gorilla |
| A0A016PG23_GIBZA/154-429 | 1.2e-54 | Gibberella zeae |
| K3W2V0_FUSPC/154-429 | 1.2e-54 | Fusarium pseudograminearum |
| E3QYP5_COLGM/154-429 | 1.1e-54 | Colletotrichum graminicola |
| S0DYH9_GIBF5/153-428 | 1.3e-54 | Gibberella fujikuroi |
| A0A0D2YD17_FUSO4/155-429 | 1.3e-54 | Fusarium oxysporum f. sp. lycopersici |
| W9LLC3_FUSOX/155-429 | 1.3e-54 | Fusarium oxysporum f. sp. lycopersici MN25 |
| X1WIQ5_ACYPI/189-423 | 1.2e-54 | Acyrthosiphon pisum |
| F0W2B6_9STRA/144-397 | 1.7e-54 | Albugo laibachii Nc14 |
| H0X735_OTOGA/75-325 | 1.8e-54 | Otolemur garnettii |
| V9KT80_CALMI/265-526 | 1.4e-54 | Callorhinchus milii |
| J9K8Z9_ACYPI/248-511 | 1.6e-54 | Acyrthosiphon pisum |
| A0A086T8A6_ACRCH/156-430 | 1.0e-54 | Acremonium chrysogenum ATCC 11550 |
| W9Z0T8_FUSOX/151-426 | 1.8e-54 | Fusarium oxysporum f. sp. melonis 26406 |
| A0A044VCY1_ONCVO/259-520 | 2.1e-54 | Onchocerca volvulus |
| Q86GS7_OXYTR/54-307 | 3.1e-54 | Oxytricha trifallax |
| W7X994_TETTS/140-399 | 2.0e-54 | Tetrahymena thermophila |
| A0A0B7KJV0_BIOOC/191-465 | 2.1e-54 | Bionectria ochroleuca |
| W5L2P6_ASTMX/75-273 | 1.7e-54 | Astyanax mexicanus |
| B7PKJ8_IXOSC/31-259 | 1.9e-54 | Ixodes scapularis |
| W9LGL4_FUSOX/154-428 | 2.5e-54 | Fusarium oxysporum f. sp. lycopersici MN25 |
| X0BPS4_FUSOX/49-324 | 2.4e-54 | Fusarium oxysporum f. sp. raphani 54005 |
| Q179Z0_AEDAE/164-427 | 2.3e-54 | Aedes aegypti |
| U7PJP0_SPOS1/223-510 | 3.9e-54 | Sporothrix schenckii |
| E2AKL1_CAMFO/272-535 | 2.7e-54 | Camponotus floridanus |
| A0A068YCI9_ECHMU/239-501 | 3.0e-54 | Echinococcus multilocularis |
| C7Z820_NECH7/154-429 | 3.4e-54 | Nectria haematococca |
| A0A0D1Y954_9EURO/4-259 | 3.2e-54 | Exophiala sideris |
| A0A066WXX7_COLSU/154-429 | 3.2e-54 | Colletotrichum sublineola |
| G0QUA3_ICHMG/114-373 | 3.5e-54 | Ichthyophthirius multifiliis |
| A0A068WC42_ECHGR/241-501 | 3.6e-54 | Echinococcus granulosus |
| Q179Z1_AEDAE/164-427 | 4.6e-54 | Aedes aegypti |
| A0A0D8YC55_DICVI/237-482 | 3.6e-54 | Dictyocaulus viviparus |
| M7TKR4_EUTLA/176-451 | 2.8e-54 | Eutypa lata |
| H2YUD9_CIOSA/261-558 | 4.3e-53 | Ciona savignyi |
| I3LAM4_PIG/72-285 | 5.0e-54 | Sus scrofa |
| G2YRJ0_BOTF4/163-449 | 5.6e-54 | Botryotinia fuckeliana |
| F8MF78_NEUT8/224-449 | 5.3e-54 | Neurospora tetrasperma |
| A0A0C2J9A9_9PEZI/276-563 | 1.2e-53 | Sporothrix brasiliensis 5110 |
| X0B9H7_FUSOX/154-428 | 5.5e-54 | Fusarium oxysporum f. sp. raphani 54005 |
| M7TIT4_BOTF1/163-449 | 6.0e-54 | Botryotinia fuckeliana |
| X0KTJ0_FUSOX/153-427 | 7.2e-54 | Fusarium oxysporum f. sp. vasinfectum 25433 |
| A0A0F4ZAG2_9PEZI/154-433 | 7.8e-54 | Thielaviopsis punctulata |
| R7V270_CAPTE/227-487 | 7.9e-54 | Capitella teleta |
| J9I4D1_9SPIT/44-291 | 9.7e-54 | Oxytricha trifallax |
| A0A067CWM0_SAPPC/112-373 | 1.7e-53 | Saprolegnia parasitica CBS 223.65 |
| A0EF65_PARTE/304-528 | 9.6e-54 | Paramecium tetraurelia |
| D6WYV7_TRICA/236-499 | 9.1e-54 | Tribolium castaneum |
| A0CDL2_PARTE/312-540 | 1.1e-53 | Paramecium tetraurelia |
| U9U7A2_RHIID/63-319 | 9.5e-54 | Rhizophagus irregularis |
| A0A078ABM1_STYLE/1460-1703 | 1.2e-53 | Stylonychia lemnae |
| G9P7A2_HYPAI/155-431 | 1.1e-53 | Hypocrea atroviridis |
| F9F8A8_FUSOF/47-313 | 9.9e-54 | Fusarium oxysporum |
| T2NA06_PORGN/194-459 | 9.4e-54 | Porphyromonas gingivalis JCVI SC001 |
| C9SDL1_VERA1/155-430 | 1.1e-53 | Verticillium alfalfae |
| G3RUW4_GORGO/99-373 | 2.1e-53 | Gorilla gorilla gorilla |
| T1JH95_STRMM/402-663 | 1.6e-53 | Strigamia maritima |
| W6UST1_ECHGR/1425-1685 | 1.7e-53 | Echinococcus granulosus |
| J4KLP7_BEAB2/141-399 | 1.0e-52 | Beauveria bassiana |
| J9IMH9_9SPIT/1477-1720 | 2.0e-53 | Oxytricha trifallax |
| U6H7U1_HYMMI/1298-1559 | 2.1e-53 | Hymenolepis microstoma |
| R7UBC6_CAPTE/55-307 | 1.8e-53 | Capitella teleta |
| U6H7U4_HYMMI/1363-1624 | 2.2e-53 | Hymenolepis microstoma |
| L7MEJ7_9ACAR/327-571 | 2.3e-53 | Rhipicephalus pulchellus |
| F4WIK8_ACREC/272-535 | 2.8e-53 | Acromyrmex echinatior |
| A0A0B1SWB5_OESDE/3-238 | 2.4e-53 | Oesophagostomum dentatum |
| I3LFZ3_PIG/72-286 | 2.9e-53 | Sus scrofa |
| G9NC34_HYPVG/157-431 | 2.5e-53 | Hypocrea virens |
| A0BHZ1_PARTE/356-608 | 3.1e-53 | Paramecium tetraurelia |
| W9Z6M8_FUSOX/148-414 | 3.0e-53 | Fusarium oxysporum f. sp. melonis 26406 |
| T0L5L9_COLGC/155-429 | 3.3e-53 | Colletotrichum gloeosporioides |
| A0DFE5_PARTE/299-528 | 3.3e-53 | Paramecium tetraurelia |
| G1PPB0_MYOLU/1-185 | 4.9e-53 | Myotis lucifugus |
| W5JFV7_ANODA/177-440 | 4.1e-53 | Anopheles darlingi |
| H1UXU6_COLHI/154-429 | 3.4e-53 | Colletotrichum higginsianum |
| Q7QGN5_ANOGA/167-430 | 4.5e-53 | Anopheles gambiae |
| L2FRJ0_COLGN/155-429 | 3.8e-53 | Colletotrichum gloeosporioides |
| S3D690_GLAL2/155-430 | 4.5e-53 | Glarea lozoyensis |
| E2BKM8_HARSA/267-530 | 4.5e-53 | Harpegnathos saltator |
| G6CQK6_DANPL/259-456 | 4.5e-53 | Danaus plexippus |
| L5KRL8_PTEAL/32-237 | 4.7e-53 | Pteropus alecto |
| M7C532_CHEMY/93-263 | 1.2e-50 | Chelonia mydas |
| W9L705_FUSOX/147-414 | 5.3e-53 | Fusarium oxysporum Fo47 |
| A0A010QIZ7_9PEZI/154-429 | 5.5e-53 | Colletotrichum fioriniae PJ7 |
| A0A069DX43_9HEMI/255-518 | 3.4e-52 | Panstrongylus megistus |
| B3RQK1_TRIAD/274-520 | 6.3e-53 | Trichoplax adhaerens |
| F9FBI6_FUSOF/447-702 | 8.2e-53 | Fusarium oxysporum |
| X0F195_FUSOX/148-414 | 7.4e-53 | Fusarium oxysporum f. sp. radicis-lycopersici 26381 |
| G2XHI2_VERDV/155-430 | 7.3e-53 | Verticillium dahliae |
| H2YK55_CIOSA/4-211 | 6.8e-53 | Ciona savignyi |
| A0A0A2VBE3_BEABA/127-301 | 1.3e-29 | Beauveria bassiana D1-5 |
| A0A0A2VBE3_BEABA/312-378 | 3.5e-17 | Beauveria bassiana D1-5 |
| A0A091N5C6_9PASS/4-187 | 7.4e-53 | Acanthisitta chloris |
| A0A099ZPK4_TINGU/94-263 | 7.8e-50 | Tinamus guttatus |
| W9IR99_FUSOX/148-414 | 9.9e-53 | Fusarium oxysporum FOSC 3-a |
| A0A024GRZ9_9STRA/271-534 | 1.1e-52 | Albugo candida |
| A0A084W670_9DIPT/170-433 | 2.3e-52 | Anopheles sinensis |
| N6T9J8_DENPD/278-538 | 8.9e-52 | Dendroctonus ponderosae |
| A0A0A9YIL2_LYGHE/176-440 | 1.3e-52 | Lygus hesperus |
| A0CF54_PARTE/399-624 | 1.1e-52 | Paramecium tetraurelia |
| A0A0A9YLM8_LYGHE/274-538 | 1.7e-52 | Lygus hesperus |
| H2RUW5_TAKRU/48-231 | 1.9e-52 | Takifugu rubripes |
| T1IDJ8_RHOPR/128-391 | 2.0e-52 | Rhodnius prolixus |
| B2AS04_PODAN/189-458 | 2.0e-52 | Podospora anserina |
| S9WVW1_9TRYP/90-359 | 2.6e-52 | Angomonas deanei |
| E0VVS6_PEDHC/257-520 | 5.1e-52 | Pediculus humanus subsp. corporis |
| T5ABG8_OPHSC/161-435 | 2.0e-52 | Ophiocordyceps sinensis |
| I7MJR3_TETTS/731-976 | 1.4e-52 | Tetrahymena thermophila |
| V8NJL9_OPHHA/130-174 | 5.2e-07 | Ophiophagus hannah |
| V8NJL9_OPHHA/179-332 | 1.4e-39 | Ophiophagus hannah |
| A0A0B7KHY0_BIOOC/134-409 | 3.9e-52 | Bionectria ochroleuca |
| F7AMH7_CIOIN/1-171 | 2.5e-52 | Ciona intestinalis |
| D8Q2S1_SCHCM/146-430 | 2.8e-52 | Schizophyllum commune |
| A0A087UP98_9ARAC/5-207 | 2.2e-52 | Stegodyphus mimosarum |
| S9UTL0_9TRYP/209-478 | 3.8e-52 | Angomonas deanei |
| S9UYK4_9TRYP/209-478 | 3.9e-52 | Angomonas deanei |
| G1NV09_MYOLU/57-314 | 1.3e-51 | Myotis lucifugus |
| A0DKW2_PARTE/382-610 | 6.8e-52 | Paramecium tetraurelia |
| C5LIR9_PERM5/103-347 | 4.3e-52 | Perkinsus marinus |
| K7IT27_NASVI/235-498 | 4.2e-52 | Nasonia vitripennis |
| D2V3V2_NAEGR/617-863 | 1.6e-51 | Naegleria gruberi |
| K1PHW0_CRAGI/38-228 | 5.0e-52 | Crassostrea gigas |
| G1RSG0_NOMLE/2-186 | 4.8e-52 | Nomascus leucogenys |
| W9CTL6_9HELO/169-445 | 5.4e-52 | Sclerotinia borealis F-4157 |
| Q4T7L4_TETNG/100-270 | 4.3e-52 | Tetraodon nigroviridis |
| A0A0C4E984_MAGP6/168-444 | 5.7e-52 | Magnaporthiopsis poae |
| H2VAL7_TAKRU/104-291 | 8.1e-52 | Takifugu rubripes |
| L9KYG4_TUPCH/503-727 | 1.4e-51 | Tupaia chinensis |
| A0A0C9WSW0_9AGAR/102-372 | 6.8e-52 | Laccaria amethystina LaAM-08-1 |
| A0A090MW73_STRRB/265-497 | 1.2e-51 | Strongyloides ratti |
| A0A099ZVS6_TINGU/44-304 | 7.0e-52 | Tinamus guttatus |
| L8WTW8_THACA/104-353 | 1.5e-49 | Thanatephorus cucumeris |
| L1J1Q4_GUITH/301-563 | 8.1e-52 | Guillardia theta CCMP2712 |
| A0A024TLA8_9STRA/12-281 | 7.7e-52 | Aphanomyces invadans |
| G0QP91_ICHMG/116-371 | 1.2e-51 | Ichthyophthirius multifiliis |
| I3JUZ3_ORENI/44-308 | 8.9e-52 | Oreochromis niloticus |
| A0A026WLR7_CERBI/302-537 | 1.0e-51 | Cerapachys biroi |
| A0A0D2VK63_CAPO3/273-532 | 1.0e-51 | Capsaspora owczarzaki ATCC 30864 |
| L1I9H5_GUITH/286-535 | 9.8e-52 | Guillardia theta CCMP2712 |
| G0PIF6_CAEBE/138-371 | 1.2e-51 | Caenorhabditis brenneri |
| A0A023EZW8_TRIIF/257-520 | 6.8e-51 | Triatoma infestans |
| A0A0C2CJ88_9BILA/286-492 | 1.3e-51 | Ancylostoma duodenale |
| J9J0E8_9SPIT/1135-1417 | 2.5e-51 | Oxytricha trifallax |
| L7IJ27_MAGOY/181-456 | 1.4e-51 | Magnaporthe oryzae |
| L7JRB1_MAGOP/181-456 | 1.4e-51 | Magnaporthe oryzae |
| G4N265_MAGO7/181-456 | 1.4e-51 | Magnaporthe oryzae |
| A0BQB7_PARTE/259-516 | 1.5e-51 | Paramecium tetraurelia |
| A0A093F323_TYTAL/44-304 | 1.3e-51 | Tyto alba |
| A0A0D2XSI8_FUSO4/32-296 | 1.7e-51 | Fusarium oxysporum f. sp. lycopersici |
| C5KNY5_PERM5/183-427 | 1.4e-51 | Perkinsus marinus |
| A0A024TNA2_9STRA/293-562 | 1.5e-51 | Aphanomyces invadans |
| W5JZS8_ASTMX/3-210 | 2.1e-51 | Astyanax mexicanus |
| Q8R486_MOUSE/1-184 | 1.7e-51 | Mus musculus |
| A0A091QSH0_9GRUI/3-187 | 1.4e-51 | Mesitornis unicolor |
| T1FU62_HELRO/242-468 | 3.9e-51 | Helobdella robusta |
| G3RA43_GORGO/99-211 | 1.2e-32 | Gorilla gorilla gorilla |
| G3RA43_GORGO/217-286 | 5.9e-13 | Gorilla gorilla gorilla |
| S9TRL9_9TRYP/209-478 | 5.7e-51 | Strigomonas culicis |
| S7RNQ0_GLOTA/86-388 | 5.1e-50 | Gloeophyllum trabeum |
| A0A091QM50_9GRUI/44-304 | 1.8e-51 | Mesitornis unicolor |
| Q32NI7_XENLA/50-301 | 1.6e-51 | Xenopus laevis |
| R1DN34_EMIHU/101-361 | 2.2e-51 | Emiliania huxleyi CCMP1516 |
| A0A0C9QQ76_9HYME/290-554 | 2.4e-51 | Fopius arisanus |
| W9CED8_9HELO/154-439 | 2.2e-51 | Sclerotinia borealis F-4157 |
| A0CK52_PARTE/129-406 | 1.7e-51 | Paramecium tetraurelia |
| J9IC43_9SPIT/98-353 | 4.1e-51 | Oxytricha trifallax |
| A0A068RM53_9FUNG/339-574 | 2.5e-50 | Lichtheimia corymbifera JMRC:FSU:9682 |
| A0A093HQV3_STRCA/44-304 | 2.5e-51 | Struthio camelus australis |
| A0A0D1Z823_9EURO/147-443 | 1.0e-50 | Exophiala mesophila |
| H2UK18_TAKRU/38-302 | 3.1e-51 | Takifugu rubripes |
| H2UK20_TAKRU/31-280 | 3.1e-51 | Takifugu rubripes |
| C1KEW2_DICLA/1-173 | 2.7e-51 | Dicentrarchus labrax |
| H2UK19_TAKRU/44-308 | 3.4e-51 | Takifugu rubripes |
| A0A087UJG4_9ARAC/284-529 | 3.8e-51 | Stegodyphus mimosarum |
| A0A091HW47_CALAN/44-304 | 3.6e-51 | Calypte anna |
| A0A094KDZ0_ANTCR/44-304 | 3.4e-51 | Antrostomus carolinensis |
| A0A091JYN9_COLST/44-304 | 4.3e-51 | Colius striatus |
| F7C284_XENTR/50-301 | 5.0e-51 | Xenopus tropicalis |
| J9IN00_9SPIT/295-538 | 6.7e-51 | Oxytricha trifallax |
| W9ZBU8_FUSOX/59-323 | 6.1e-51 | Fusarium oxysporum f. sp. melonis 26406 |
| Q174B8_AEDAE/4-200 | 5.6e-51 | Aedes aegypti |
| A0A0D2NDE2_9AGAR/134-407 | 5.9e-51 | Hypholoma sublateritium FD-334 SS-4 |
| L9KZE0_TUPCH/207-274 | 9.7e-10 | Tupaia chinensis |
| L9KZE0_TUPCH/296-436 | 2.4e-35 | Tupaia chinensis |
| W9NEC3_FUSOX/161-425 | 8.0e-51 | Fusarium oxysporum f. sp. pisi HDV247 |
| W9NE41_FUSOX/182-446 | 8.3e-51 | Fusarium oxysporum f. sp. pisi HDV247 |
| A0A0C3C447_HEBCY/137-419 | 7.3e-51 | Hebeloma cylindrosporum h7 |
| A0CN91_PARTE/129-406 | 5.2e-51 | Paramecium tetraurelia |
| A0A091S9C6_NESNO/44-304 | 7.4e-51 | Nestor notabilis |
| V9KSJ8_CALMI/1-179 | 8.2e-51 | Callorhinchus milii |
| D2VKN2_NAEGR/51-330 | 6.0e-49 | Naegleria gruberi |
| E4YD44_OIKDI/45-246 | 9.4e-51 | Oikopleura dioica |
| H3DCW3_TETNG/44-309 | 9.7e-51 | Tetraodon nigroviridis |
| S9UUY1_9TRYP/47-273 | 1.5e-24 | Angomonas deanei |
| S9UUY1_9TRYP/662-809 | 3.4e-20 | Angomonas deanei |
| A0A093BUF9_9AVES/44-304 | 1.0e-50 | Pterocles gutturalis |
| E4XHF7_OIKDI/123-337 | 1.1e-50 | Oikopleura dioica |
| A0A078A3Q8_STYLE/63-308 | 3.9e-50 | Stylonychia lemnae |
| H2KR25_CLOSI/229-478 | 1.2e-50 | Clonorchis sinensis |
| J9F196_9SPIT/193-444 | 1.2e-50 | Oxytricha trifallax |
| J3PGM8_GAGT3/172-447 | 1.4e-50 | Gaeumannomyces graminis var. tritici |
| S9V2C2_9TRYP/215-479 | 2.2e-50 | Strigomonas culicis |
| A0A091VRP4_OPIHO/44-304 | 1.4e-50 | Opisthocomus hoazin |
| Q4S4V6_TETNG/836-1031 | 6.0e-48 | Tetraodon nigroviridis |
| A0A093F128_TAUER/44-304 | 1.7e-50 | Tauraco erythrolophus |
| A0A078AES7_STYLE/244-480 | 4.0e-50 | Stylonychia lemnae |
| A0A091M3C9_CARIC/44-304 | 1.8e-50 | Cariama cristata |
| A0A091U1N1_PHORB/44-304 | 1.7e-50 | Phoenicopterus ruber ruber |
| E4WYV1_OIKDI/93-342 | 2.0e-50 | Oikopleura dioica |
| A0A0A9WA58_LYGHE/5-215 | 2.0e-50 | Lygus hesperus |
| G3HZH6_CRIGR/317-554 | 2.2e-50 | Cricetulus griseus |
| W9NMJ6_FUSOX/32-296 | 2.6e-50 | Fusarium oxysporum f. sp. pisi HDV247 |
| A0A091SV35_9AVES/44-304 | 1.9e-50 | Pelecanus crispus |
| A0A091TQ81_PHALP/44-304 | 1.9e-50 | Phaethon lepturus |
| A0A0B1S4E9_OESDE/85-256 | 1.9e-50 | Oesophagostomum dentatum |
| B7PCL2_IXOSC/1-202 | 2.1e-50 | Ixodes scapularis |
| G1N9E1_MELGA/44-304 | 2.1e-50 | Meleagris gallopavo |
| A0A093GH99_PICPB/44-304 | 2.2e-50 | Picoides pubescens |
| A0A091Q8Y8_LEPDC/44-304 | 2.3e-50 | Leptosomus discolor |
| X0L2S0_FUSOX/8-264 | 3.6e-50 | Fusarium oxysporum f. sp. vasinfectum 25433 |
| S9XEE4_9CETA/60-281 | 3.1e-50 | Camelus ferus |
| A0A067SIW4_9AGAR/137-410 | 2.9e-50 | Galerina marginata CBS 339.88 |
| A0A091LKB7_9GRUI/44-304 | 2.9e-50 | Chlamydotis macqueenii |
| A9V9P6_MONBE/805-1063 | 3.7e-50 | Monosiga brevicollis |
| A0A091LDM0_CATAU/547-773 | 2.2e-49 | Cathartes aura |
| T0QVU2_9STRA/117-383 | 3.7e-50 | Saprolegnia diclina VS20 |
| A0A091H5Z9_BUCRH/44-304 | 3.8e-50 | Buceros rhinoceros silvestris |
| J9I895_9SPIT/71-317 | 4.5e-50 | Oxytricha trifallax |
| S7MRP7_MYOBR/349-573 | 4.7e-50 | Myotis brandtii |
| A0A077ZXJ0_STYLE/189-438 | 4.6e-50 | Stylonychia lemnae |
| G1NW84_MYOLU/85-334 | 4.9e-50 | Myotis lucifugus |
| A0A091WG54_NIPNI/44-304 | 4.1e-50 | Nipponia nippon |
| A0A0F5CRJ7_PRIPA/1-236 | 3.5e-50 | Pristionchus pacificus |
| R1DTX9_EMIHU/233-435 | 5.8e-43 | Emiliania huxleyi CCMP1516 |
| E9BHQ8_LEIDB/215-477 | 5.1e-50 | Leishmania donovani |
| A4I1J4_LEIIN/215-477 | 5.1e-50 | Leishmania infantum |
| F7FGE5_ORNAN/2-260 | 3.8e-50 | Ornithorhynchus anatinus |
| A0A077ZU42_STYLE/100-351 | 6.5e-50 | Stylonychia lemnae |
| A0A093QF25_PHACA/44-304 | 4.6e-50 | Phalacrocorax carbo |
| A0A087VF24_BALRE/44-304 | 4.8e-50 | Balearica regulorum gibbericeps |
| H0YEJ4_HUMAN/5-172 | 4.6e-50 | Homo sapiens |
| A0A091PKE7_APAVI/44-304 | 5.6e-50 | Apaloderma vittatum |
| A0A095BTM4_SCHHA/179-430 | 5.9e-50 | Schistosoma haematobium |
| A4HJ24_LEIBR/225-493 | 9.6e-50 | Leishmania braziliensis |
| A0A093PJ51_PYGAD/44-304 | 6.0e-50 | Pygoscelis adeliae |
| A0A087R832_APTFO/44-304 | 6.0e-50 | Aptenodytes forsteri |
| H0ZIC8_TAEGU/44-304 | 6.2e-50 | Taeniopygia guttata |
| G1QBH9_MYOLU/104-354 | 1.5e-49 | Myotis lucifugus |
| A0A091IXQ4_9AVES/44-304 | 6.7e-50 | Egretta garzetta |
| U3JZH2_FICAL/44-304 | 6.8e-50 | Ficedula albicollis |
| A0A0A0AEP5_CHAVO/44-304 | 7.1e-50 | Charadrius vociferus |
| X0LY78_FUSOX/162-425 | 1.0e-49 | Fusarium oxysporum f. sp. vasinfectum 25433 |
| X0KIG8_FUSOX/183-446 | 1.1e-49 | Fusarium oxysporum f. sp. vasinfectum 25433 |
| J0M4A9_LOALO/1-160 | 9.9e-50 | Loa loa |
| A8P1B5_BRUMA/260-544 | 1.6e-47 | Brugia malayi |
| A0A091G0S4_9AVES/44-304 | 8.4e-50 | Cuculus canorus |
| W4G238_9STRA/2089-2344 | 1.2e-49 | Aphanomyces astaci |
| L8Y3S1_TUPCH/62-283 | 1.2e-49 | Tupaia chinensis |
| W7MG96_GIBM7/119-386 | 1.3e-49 | Gibberella moniliformis |
| A0A095A0Z0_SCHHA/172-375 | 1.1e-49 | Schistosoma haematobium |
| A0A088RSA8_9TRYP/216-477 | 1.5e-49 | Leishmania panamensis |
| A4HE81_LEIBR/216-477 | 1.5e-49 | Leishmania braziliensis |
| A0A088RWX8_9TRYP/225-493 | 2.3e-49 | Leishmania panamensis |
| A0A091EDV4_CORBR/44-304 | 1.4e-49 | Corvus brachyrhynchos |
| A0A091MG50_9PASS/44-304 | 1.4e-49 | Acanthisitta chloris |
| S9USB9_9TRYP/213-477 | 2.6e-49 | Strigomonas culicis |
| S7Q9D1_GLOTA/47-309 | 2.1e-48 | Gloeophyllum trabeum |
| A0A067BV24_SAPPC/116-381 | 1.7e-49 | Saprolegnia parasitica CBS 223.65 |
| W9QS19_FUSOX/119-386 | 2.3e-49 | Fusarium oxysporum f. sp. pisi HDV247 |
| X0FC67_FUSOX/119-386 | 2.4e-49 | Fusarium oxysporum f. sp. radicis-lycopersici 26381 |
| A0DTQ9_PARTE/639-933 | 3.1e-49 | Paramecium tetraurelia |
| Q22B94_TETTS/1294-1572 | 1.7e-49 | Tetrahymena thermophila |
| A0A093JIH8_FULGA/44-304 | 2.2e-49 | Fulmarus glacialis |
| E9AXM9_LEIMU/214-476 | 3.4e-49 | Leishmania mexicana |
| A0A0F5D1S5_PRIPA/2-174 | 2.6e-49 | Pristionchus pacificus |
| Q4Q9U3_LEIMA/215-476 | 3.2e-49 | Leishmania major |
| U6DS10_NEOVI/9-222 | 2.9e-49 | Neovison vison |
| V4AN77_LOTGI/15-265 | 3.2e-49 | Lottia gigantea |
| X0IMB0_FUSOX/49-299 | 3.0e-49 | Fusarium oxysporum f. sp. cubense |
| B2AUZ5_PODAN/154-420 | 5.5e-49 | Podospora anserina |
| H7C1B7_HUMAN/3-242 | 1.6e-48 | Homo sapiens |
| A0A066UZG4_9HOMO/105-263 | 4.1e-33 | Rhizoctonia solani AG-8 WAC10335 |
| A0A066UZG4_9HOMO/266-314 | 2.4e-10 | Rhizoctonia solani AG-8 WAC10335 |
| A0A066VD11_9HOMO/13-228 | 5.6e-49 | Rhizoctonia solani AG-8 WAC10335 |
| A0A024U6Q9_9STRA/2093-2348 | 5.0e-49 | Aphanomyces invadans |
| A4I6E6_LEIIN/289-557 | 8.7e-49 | Leishmania infantum |
| B0DRD8_LACBS/97-390 | 4.5e-49 | Laccaria bicolor) |
| H3B8J1_LATCH/43-303 | 5.7e-49 | Latimeria chalumnae |
| E9BMH1_LEIDB/290-557 | 1.1e-48 | Leishmania donovani |
| E9PL37_HUMAN/8-191 | 6.3e-49 | Homo sapiens |
| A0A016P9Q4_GIBZA/202-460 | 1.4e-48 | Gibberella zeae |
| I7M2X8_TETTS/696-996 | 7.4e-49 | Tetrahymena thermophila |
| T1IAM9_RHOPR/70-326 | 1.2e-48 | Rhodnius prolixus |
| B3RX80_TRIAD/2-193 | 1.2e-48 | Trichoplax adhaerens |
| H2MLU5_ORYLA/44-309 | 1.3e-48 | Oryzias latipes |
| R7S0W7_STEHR/106-319 | 3.8e-29 | Stereum hirsutum |
| R7S0W7_STEHR/362-439 | 7.5e-14 | Stereum hirsutum |
| U3I710_ANAPL/396-632 | 7.1e-48 | Anas platyrhynchos |
| U3I712_ANAPL/398-634 | 7.1e-48 | Anas platyrhynchos |
| E9IMH4_SOLIN/272-530 | 1.8e-48 | Solenopsis invicta |
| F8W4K1_DANRE/40-303 | 1.9e-48 | Danio rerio |
| W4W0N6_ATTCE/291-560 | 2.7e-48 | Atta cephalotes |
| F1QTM5_DANRE/43-306 | 2.0e-48 | Danio rerio |
| Q4Q6L7_LEIMA/245-512 | 3.7e-48 | Leishmania major |
| T0Q6U9_9STRA/1989-2243 | 2.7e-48 | Saprolegnia diclina VS20 |
| A0A067CQU0_SAPPC/1942-2196 | 2.6e-48 | Saprolegnia parasitica CBS 223.65 |
| H2N319_ORYLA/80-327 | 2.8e-48 | Oryzias latipes |
| V9L3X8_CALMI/1-237 | 2.1e-48 | Callorhinchus milii |
| Q4S467_TETNG/264-599 | 2.8e-45 | Tetraodon nigroviridis |
| R1G7V6_EMIHU/92-358 | 3.5e-48 | Emiliania huxleyi CCMP1516 |
| A0A093SFC0_9PASS/44-279 | 2.7e-48 | Manacus vitellinus |
| A0A078AB47_STYLE/188-449 | 2.1e-47 | Stylonychia lemnae |
| H9GRW6_ANOCA/3-184 | 2.2e-48 | Anolis carolinensis |
| N4WXQ1_COCH4/162-392 | 3.2e-48 | Cochliobolus heterostrophus |
| H2N3L7_PONAB/75-142 | 2.0e-11 | Pongo abelii |
| H2N3L7_PONAB/144-281 | 3.8e-31 | Pongo abelii |
| G3PQN9_GASAC/44-308 | 3.0e-48 | Gasterosteus aculeatus |
| F7BYJ0_MONDO/109-278 | 4.6e-48 | Monodelphis domestica |
| H0WB47_CAVPO/2-224 | 2.8e-48 | Cavia porcellus |
| Q9U2B2_CAEEL/240-493 | 4.5e-48 | Caenorhabditis elegans |
| Q4RV78_TETNG/44-315 | 4.2e-48 | Tetraodon nigroviridis |
| K7FD57_PELSI/1-183 | 5.0e-48 | Pelodiscus sinensis |
| K7G1K9_PELSI/44-304 | 5.0e-48 | Pelodiscus sinensis |
| G0QWB9_ICHMG/120-332 | 5.6e-48 | Ichthyophthirius multifiliis |
| G2Y5J4_BOTF4/173-438 | 6.3e-48 | Botryotinia fuckeliana |
| M7U4D5_BOTF1/173-438 | 6.3e-48 | Botryotinia fuckeliana |
| A0E1K3_PARTE/638-931 | 8.0e-48 | Paramecium tetraurelia |
| G1P2U1_MYOLU/43-303 | 8.1e-48 | Myotis lucifugus |
| A0A023FLK8_9ACAR/2-161 | 6.7e-48 | Amblyomma cajennense |
| S9UQH9_9TRYP/39-306 | 1.4e-47 | Angomonas deanei |
| F7CY16_MONDO/44-305 | 9.0e-48 | Monodelphis domestica |
| G0U2G4_TRYVY/326-593 | 1.1e-47 | Trypanosoma vivax |
| M3WDP6_FELCA/1-167 | 3.8e-25 | Felis catus |
| M3WDP6_FELCA/221-293 | 2.4e-16 | Felis catus |
| F7BS99_ORNAN/1-186 | 1.2e-47 | Ornithorhynchus anatinus |
| A0BHX6_PARTE/261-516 | 1.3e-47 | Paramecium tetraurelia |
| K1Y7V6_MARBU/154-419 | 1.4e-47 | Marssonina brunnea f. sp. multigermtubi |
| J3PIN8_GAGT3/130-413 | 1.2e-47 | Gaeumannomyces graminis var. tritici |
| A0A0B7AB94_9EUPU/42-249 | 1.0e-47 | Arion vulgaris |
| A0BNI6_PARTE/303-445 | 8.1e-31 | Paramecium tetraurelia |
| A0BNI6_PARTE/474-524 | 9.1e-12 | Paramecium tetraurelia |
| F0V794_NEOCL/786-1091 | 2.0e-47 | Neospora caninum |
| A0A060Z6A6_ONCMY/18-179 | 1.6e-47 | Oncorhynchus mykiss |
| E8NHF1_LEIMU/19-287 | 3.2e-47 | Leishmania mexicana |
| S9V3E5_9TRYP/212-479 | 2.7e-47 | Angomonas deanei |
| K0TN45_THAOC/338-616 | 2.7e-47 | Thalassiosira oceanica |
| D2W146_NAEGR/356-713 | 1.9e-46 | Naegleria gruberi |
| W5MF25_LEPOC/43-302 | 2.2e-47 | Lepisosteus oculatus |
| A9UYT4_MONBE/152-387 | 4.1e-47 | Monosiga brevicollis |
| A0A074TQW1_9APIC/1361-1673 | 4.4e-47 | Hammondia hammondi |
| E8NHM8_LEIMU/92-360 | 4.3e-47 | Leishmania mexicana |
| Q1JT76_TOXGO/1264-1576 | 4.0e-47 | Toxoplasma gondii |
| A0A086JIF7_TOXGO/1360-1672 | 4.4e-47 | Toxoplasma gondii GAB2-2007-GAL-DOM2 |
| A0A086KRS5_TOXGO/1360-1672 | 4.4e-47 | Toxoplasma gondii FOU |
| A0A086LS53_TOXGO/1360-1672 | 4.4e-47 | Toxoplasma gondii RUB |
| A0A086PSC3_TOXGO/1359-1671 | 4.4e-47 | Toxoplasma gondii VAND |
| V4Z5M2_TOXGO/1359-1671 | 4.4e-47 | Toxoplasma gondii |
| S8F9K1_TOXGO/1359-1671 | 4.4e-47 | Toxoplasma gondii ME49 |
| A0A086JU79_TOXGO/1359-1671 | 4.4e-47 | Toxoplasma gondii p89 |
| S7VX33_TOXGO/1359-1671 | 4.4e-47 | Toxoplasma gondii GT1 |
| A0A086QP37_TOXGO/1359-1671 | 4.4e-47 | Toxoplasma gondii MAS |
| E8NHG8_LEIMU/297-565 | 5.9e-47 | Leishmania mexicana |
| G1MEK2_AILME/43-303 | 3.8e-47 | Ailuropoda melanoleuca |
| D2HLE0_AILME/48-308 | 3.9e-47 | Ailuropoda melanoleuca |
| E9B1J6_LEIMU/219-487 | 6.8e-47 | Leishmania mexicana |
| S9TW66_9TRYP/204-471 | 6.0e-47 | Strigomonas culicis |
| A0A067CSQ5_SAPPC/7-245 | 5.5e-47 | Saprolegnia parasitica CBS 223.65 |
| M3WH08_FELCA/42-302 | 5.5e-47 | Felis catus |
| L5KTY5_PTEAL/38-298 | 7.0e-47 | Pteropus alecto |
| F1PNG4_CANFA/44-304 | 6.7e-47 | Canis familiaris |
| G4V623_SCHMA/125-366 | 7.5e-47 | Schistosoma mansoni |
| D2VKM8_NAEGR/196-460 | 1.7e-45 | Naegleria gruberi |
| M3XM36_MUSPF/38-298 | 8.7e-47 | Mustela putorius furo |
| A0A0C2GGF9_9BILA/2-160 | 8.8e-47 | Ancylostoma duodenale |
| G1TV22_RABIT/491-568 | 8.3e-16 | Oryctolagus cuniculus |
| G1TV22_RABIT/568-703 | 2.8e-25 | Oryctolagus cuniculus |
| R1EN66_EMIHU/464-706 | 1.7e-45 | Emiliania huxleyi CCMP1516 |
| A0A087W282_ECHMU/160-407 | 1.1e-46 | Echinococcus multilocularis |
| L8X2F6_THACA/151-427 | 2.2e-46 | Thanatephorus cucumeris |
| S9VYD8_9TRYP/204-471 | 1.3e-46 | Strigomonas culicis |
| G0NC26_CAEBE/115-343 | 1.3e-46 | Caenorhabditis brenneri |
| F6Q7V0_XENTR/64-334 | 1.5e-45 | Xenopus tropicalis |
| A0A060Y2E6_ONCMY/75-339 | 1.5e-46 | Oncorhynchus mykiss |
| D8LK54_ECTSI/71-262 | 7.4e-32 | Ectocarpus siliculosus |
| D8LK54_ECTSI/257-322 | 6.8e-09 | Ectocarpus siliculosus |
| R7T901_CAPTE/2-220 | 1.3e-46 | Capitella teleta |
| F7VY97_SORMK/160-423 | 2.2e-46 | Sordaria macrospora |
| A0A088RPK1_9TRYP/351-610 | 2.0e-46 | Leishmania panamensis |
| U7PRP9_SPOS1/202-416 | 2.2e-46 | Sporothrix schenckii |
| A0A088S8S0_9TRYP/385-644 | 2.2e-46 | Leishmania panamensis |
| S9X3L3_9TRYP/205-471 | 4.1e-46 | Angomonas deanei |
| E8NHM4_LEIMU/219-487 | 3.7e-46 | Leishmania mexicana) |
| J9F8P9_WUCBA/192-402 | 5.3e-44 | Wuchereria bancrofti |
| G3HC86_CRIGR/72-245 | 1.3e-44 | Cricetulus griseus |
| S9WH82_9TRYP/205-471 | 3.9e-46 | Angomonas deanei |
| A0A087UT38_9ARAC/56-321 | 1.1e-45 | Stegodyphus mimosarum |
| E9AIH1_LEIBR/352-611 | 2.9e-46 | Leishmania braziliensis |
| A0A0F5D272_PRIPA/58-246 | 2.6e-46 | Pristionchus pacificus |
| A0A074YTB3_9PEZI/168-443 | 3.3e-46 | Aureobasidium subglaciale EXF-2481 |
| W9IEY4_FUSOX/1-241 | 3.4e-46 | Fusarium oxysporum FOSC 3-a |
| W9K2J6_FUSOX/1-241 | 3.4e-46 | Fusarium oxysporum Fo47 |
| X0A929_FUSOX/1-241 | 3.4e-46 | Fusarium oxysporum f. sp. melonis 26406 |
| W9LUQ9_FUSOX/1-241 | 3.4e-46 | Fusarium oxysporum f. sp. lycopersici MN25 |
| Q152V0_PIG/38-305 | 3.5e-46 | Sus scrofa domesticus |
| U1NT67_ASCSU/50-246 | 9.5e-45 | Ascaris suum |
| Q152V1_PIG/38-305 | 3.2e-46 | Sus scrofa domesticus |
| R1CAW8_EMIHU/101-349 | 5.6e-46 | Emiliania huxleyi CCMP1516 |
| E8NHM2_LEIMU/219-487 | 7.0e-46 | Leishmania mexicana) |
| J9IF14_9SPIT/230-478 | 8.4e-46 | Oxytricha trifallax |
| I7M2Y1_TETTS/723-1028 | 6.2e-46 | Tetrahymena thermophila |
| F1MBN8_BOVIN/38-298 | 3.9e-46 | Bos taurus |
| Q5U345_RAT/43-303 | 4.2e-46 | Rattus norvegicus |
| X0KQI5_FUSOX/1-241 | 5.6e-46 | Fusarium oxysporum f. sp. cubense tropical race 4 54006 |
| E4YD63_OIKDI/43-288 | 6.4e-46 | Oikopleura dioica |
| L1JZN7_GUITH/230-493 | 7.1e-46 | Guillardia theta CCMP2712 |
| W2TF79_NECAM/150-344 | 2.2e-45 | Necator americanus |
| E9BIY5_LEIDB/45-269 | 4.1e-25 | Leishmania donovani |
| E9BIY5_LEIDB/4965-5169 | 9.1e-15 | Leishmania donovani) |
| B0FGU0_PIG/38-305 | 8.2e-46 | Sus scrofa ussuricus |
| W2SVL0_NECAM/2-209 | 6.4e-46 | Necator americanus |
| B0FGU1_PIG/38-305 | 7.5e-46 | Sus scrofa ussuricus |
| S9X4M4_9CETA/72-258 | 9.7e-46 | Camelus ferus |
| A0A0C2IUI7_9PEZI/305-538 | 1.1e-45 | Sporothrix brasiliensis 5110 |
| L8WMC1_THACA/370-643 | 1.1e-45 | Thanatephorus cucumeris |
| D8TYQ1_VOLCA/87-243 | 2.5e-25 | Volvox carteri |
| D8TYQ1_VOLCA/330-533 | 1.5e-14 | Volvox carteri |
| A0A074Y6T0_AURPU/174-446 | 1.3e-45 | Aureobasidium pullulans EXF-150 |
| D6RGR0_MOUSE/43-303 | 1.0e-45 | Mus musculus |
| W6KU77_9TRYP/358-620 | 1.6e-45 | Phytomonas sp. isolate EM1 |
| K9ITM4_DESRO/43-303 | 2.1e-45 | Desmodus rotundus |
| W7TQ64_9STRA/317-601 | 1.7e-45 | Nannochloropsis gaditana |
| A0A074W8T3_9PEZI/65-340 | 2.0e-45 | Aureobasidium melanogenum CBS 110374 |
| J9IVT8_9SPIT/129-371 | 4.2e-45 | Oxytricha trifallax |
| J9I0B6_9SPIT/216-448 | 2.0e-45 | Oxytricha trifallax |
| Q767K2_RAT/43-303 | 2.0e-45 | Rattus norvegicus |
| CAN10_MOUSE/43-303 | 2.0e-45 | Mus musculus |
| Q7TQ41_RAT/43-303 | 2.2e-45 | Rattus norvegicus |
| A0A087XH24_POEFO/44-309 | 2.6e-45 | Poecilia formosa |
| U6DA02_NEOVI/5-260 | 1.9e-45 | Neovison vison |
| X1WYP2_ACYPI/42-301 | 2.6e-45 | Acyrthosiphon pisum |
| K3W7R2_PYTUL/2066-2323 | 2.7e-45 | Pythium ultimum DAOM BR144 |
| U3I716_ANAPL/82-324 | 4.6e-45 | Anas platyrhynchos |
| X6P8Y3_RETFI/7-246 | 2.7e-45 | Reticulomyxa filosa |
| CAN10_RAT/43-303 | 3.7e-45 | Rattus norvegicus |
| C9SJS5_VERA1/167-314 | 3.4e-27 | Verticillium alfalfae |
| C9SJS5_VERA1/334-412 | 2.2e-12 | Verticillium alfalfae |
| H0VLK9_CAVPO/43-304 | 5.0e-45 | Cavia porcellus |
| A0A075A0T6_9TREM/84-364 | 7.7e-45 | Opisthorchis viverrini |
| K0RHU2_THAOC/257-334 | 1.3e-11 | Thalassiosira oceanica |
| K0RHU2_THAOC/469-623 | 2.8e-27 | Thalassiosira oceanica |
| G3QGL4_GORGO/43-303 | 6.4e-45 | Gorilla gorilla gorilla |
| G3S9K1_GORGO/44-304 | 6.4e-45 | Gorilla gorilla gorilla |
| T1KNX4_TETUR/344-605 | 4.4e-44 | Tetranychus urticae |
| S9UFG0_9TRYP/321-587 | 1.9e-44 | Angomonas deanei |
| Q4E0D8_TRYCC/368-632 | 1.1e-44 | Trypanosoma cruzi |
| K4DTF4_TRYCR/368-632 | 1.1e-44 | Trypanosoma cruzi |
| H3GMV4_PHYRM/2072-2343 | 1.3e-44 | Phytophthora ramorum |
| H2QJQ9_PANTR/43-302 | 9.4e-45 | Pan troglodytes |
| S9UPN6_9TRYP/312-583 | 2.3e-44 | Strigomonas culicis |
| J9I9T5_9SPIT/116-358 | 2.1e-44 | Oxytricha trifallax |
| G3WJK1_SARHA/47-307 | 1.0e-44 | Sarcophilus harrisii |
| V5B5I4_TRYCR/368-632 | 1.2e-44 | Trypanosoma cruzi Dm28c |
| M1EE84_MUSPF/1-171 | 3.6e-44 | Mustela putorius furo |
| D0MZ44_PHYIT/1964-2230 | 1.7e-44 | Phytophthora infestans |
| E4WXJ2_OIKDI/43-281 | 1.4e-44 | Oikopleura dioica |
| A0A024R4A1_HUMAN/43-303 | 1.5e-44 | Homo sapiens |
| N6U8F3_DENPD/12-199 | 1.9e-44 | Dendroctonus ponderosae |
| R1E7P0_EMIHU/261-526 | 1.6e-43 | Emiliania huxleyi CCMP1516 |
| G3HJB2_CRIGR/75-272 | 1.7e-44 | Cricetulus griseus |
| E0V9U5_PEDHC/42-301 | 1.8e-44 | Pediculus humanus subsp. corporis |
| U4UBL4_DENPD/12-199 | 2.2e-44 | Dendroctonus ponderosae |
| E9AUQ7_LEIMU/468-729 | 2.1e-44 | Leishmania mexicana |
| C9ZIE7_TRYB9/607-873 | 2.2e-44 | Trypanosoma brucei gambiense |
| M0R3D7_HUMAN/2-174 | 2.6e-44 | Homo sapiens |
| I7MF26_TETTS/143-392 | 1.5e-44 | Tetrahymena thermophila |
| Q4E0D6_TRYCC/243-497 | 2.5e-44 | Trypanosoma cruzi) |
| W5K7P1_ASTMX/1-161 | 2.3e-44 | Astyanax mexicanus |
| Q4GZ11_TRYB2/675-941 | 2.4e-44 | Trypanosoma brucei brucei |
| M4A2C6_XIPMA/40-305 | 2.3e-44 | Xiphophorus maculatus |
| K2M5K4_TRYCR/243-497 | 2.9e-44 | Trypanosoma cruzi marinkellei |
| H2P955_PONAB/43-303 | 2.3e-44 | Pongo abelii |
| H3FC58_PRIPA/126-210 | 0.00011 | Pristionchus pacificus |
| H3FC58_PRIPA/209-363 | 3.1e-34 | Pristionchus pacificus |
| K2N6A2_TRYCR/369-634 | 3.1e-44 | Trypanosoma cruzi marinkellei |
| H0W0U4_CAVPO/32-177 | 4.2e-30 | Cavia porcellus |
| H0W0U4_CAVPO/223-301 | 2.2e-08 | Cavia porcellus |
| K7CKA1_PANTR/43-303 | 2.7e-44 | Pan troglodytes |
| H0ZL02_TAEGU/76-328 | 2.7e-43 | Taeniopygia guttata |
| J9EW04_9SPIT/62-319 | 3.4e-44 | Oxytricha trifallax |
| A0A067RT07_ZOONE/42-300 | 3.2e-44 | Zootermopsis nevadensis |
| W7MAK8_GIBM7/1-245 | 3.5e-44 | Gibberella moniliformis |
| G0M9K3_CAEBE/92-323 | 4.2e-44 | Caenorhabditis brenneri |
| A0A024MCW8_BRUMA/51-233 | 3.0e-44 | Brugia malayi |
| F6RMG8_MACMU/43-303 | 3.2e-44 | Macaca mulatta |
| D7FQ17_ECTSI/3289-3552 | 4.5e-44 | Ectocarpus siliculosus |
| G1RTJ4_NOMLE/43-303 | 3.5e-44 | Nomascus leucogenys |
| A4HYN0_LEIIN/355-616 | 4.0e-44 | Leishmania infantum |
| F7HT69_CALJA/43-303 | 3.5e-44 | Callithrix jacchus |
| T1EP16_HELRO/56-292 | 3.3e-44 | Helobdella robusta |
| U3DBD4_CALJA/43-303 | 3.8e-44 | Callithrix jacchus |
| W5NEF3_LEPOC/78-233 | 4.4e-44 | Lepisosteus oculatus |
| B7WPF5_HUMAN/43-303 | 3.9e-44 | Homo sapiens |
| S9XJQ7_9CETA/22-266 | 4.5e-44 | Camelus ferus |
| R4FL13_RHOPR/68-321 | 9.8e-44 | Rhodnius prolixus |
| CAN10_HUMAN/43-303 | 4.2e-44 | Homo sapiens |
| Q3UF24_MOUSE/2-166 | 1.1e-43 | Mus musculus |
| B8BYL0_THAPS/370-649 | 5.3e-44 | Thalassiosira pseudonana |
| Q4DQN6_TRYCC/242-497 | 5.6e-44 | Trypanosoma cruzi |
| U6GU82_EIMAC/729-962 | 5.7e-36 | Eimeria acervulina |
| F6RTZ4_MACMU/43-303 | 4.8e-44 | Macaca mulatta |
| F6RU11_MACMU/43-303 | 4.7e-44 | Macaca mulatta |
| A4HYW1_LEIIN/548-809 | 6.0e-44 | Leishmania infantum |
| F7FIP7_CALJA/42-302 | 5.2e-44 | Callithrix jacchus |
| F7EIU4_CALJA/43-303 | 5.2e-44 | Callithrix jacchus |
| H0XDW4_OTOGA/45-306 | 5.6e-44 | Otolemur garnettii |
| G0R4Q2_ICHMG/1-226 | 1.0e-43 | Ichthyophthirius multifiliis |
| H9FPA8_MACMU/43-303 | 7.0e-44 | Macaca mulatta |
| U3BMM2_CALJA/43-303 | 7.0e-44 | Callithrix jacchus |
| U3EA24_CALJA/43-303 | 7.0e-44 | Callithrix jacchus |
| U3EB14_CALJA/43-303 | 7.0e-44 | Callithrix jacchus |
| CAN10_MACFA/43-303 | 7.5e-44 | Macaca fascicularis |
| Q4GZ09_TRYB2/262-516 | 8.5e-44 | Trypanosoma brucei brucei |
| G4YH14_PHYSP/1973-2244 | 9.2e-44 | Phytophthora sojae |
| I2CTV9_MACMU/43-303 | 7.9e-44 | Macaca mulatta |
| G7PKG1_MACFA/43-303 | 8.0e-44 | Macaca fascicularis |
| G7N9C5_MACMU/43-303 | 8.0e-44 | Macaca mulatta |
| G1TPK4_RABIT/43-303 | 8.6e-44 | Oryctolagus cuniculus |
| M0RD70_RAT/2-257 | 9.2e-44 | Rattus norvegicus |
| A0CC52_PARTE/537-772 | 9.7e-44 | Paramecium tetraurelia |
| F6RU22_MACMU/43-303 | 9.0e-44 | Macaca mulatta |
| F6RMD5_MACMU/43-303 | 9.0e-44 | Macaca mulatta |
| G3RQI7_GORGO/236-494 | 1.4e-43 | Gorilla gorilla gorilla |
| C9ZIE9_TRYB9/262-516 | 9.9e-44 | Trypanosoma brucei gambiense |
| F7E6N1_CALJA/42-302 | 9.7e-44 | Callithrix jacchus |
| F7E6Z4_CALJA/43-303 | 9.7e-44 | Callithrix jacchus |
| A0A0D9R0W2_CHLSB/43-303 | 1.0e-43 | Chlorocebus sabaeus |
| A0A096NA77_PAPAN/43-303 | 1.5e-43 | Papio anubis |
| V5BA05_TRYCR/243-497 | 1.8e-43 | Trypanosoma cruzi Dm28c |
| S9UIG5_9TRYP/312-566 | 5.4e-43 | Strigomonas culicis |
| S7ME99_MYOBR/105-149 | 7.3e-07 | Myotis brandtii |
| S7ME99_MYOBR/150-247 | 1.1e-23 | Myotis brandtii |
| K4DUN8_TRYCR/243-497 | 2.2e-43 | Trypanosoma cruzi |
| A0A091LBZ8_CATAU/44-213 | 1.8e-43 | Cathartes aura |
| L9KKQ4_TUPCH/114-374 | 2.4e-43 | Tupaia chinensis |
| U6MPY6_9EIME/518-833 | 3.3e-43 | Eimeria necatrix |
| Q4QCS8_LEIMA/357-618 | 2.5e-43 | Leishmania major |
| D6WGY6_TRICA/42-298 | 2.6e-43 | Tribolium castaneum |
| T1JH14_STRMM/42-297 | 2.6e-43 | Strigamia maritima |
| H2TH85_TAKRU/11-193 | 3.5e-43 | Takifugu rubripes |
| A0A0D2YDT5_FUSO4/9-232 | 2.4e-43 | Fusarium oxysporum f. sp. lycopersici |
| I3PVB1_SOLSE/1-154 | 2.1e-43 | Solea senegalensis |
| S3DBL0_GLAL2/158-442 | 8.2e-43 | Glarea lozoyensis |
| A0A061J510_TRYRA/78-323 | 3.0e-43 | Trypanosoma rangeli SC58 |
| D2VPM6_NAEGR/249-537 | 1.4e-40 | Naegleria gruberi |
| W4Y195_STRPU/67-266 | 8.6e-43 | Strongylocentrotus purpuratus |
| Q4QCS9_LEIMA/511-772 | 3.4e-43 | Leishmania major |
| A0A023EXU3_TRIIF/47-281 | 6.0e-42 | Triatoma infestans |
| V9EXQ9_PHYPR/2031-2293 | 3.7e-43 | Phytophthora parasitica P1569 |
| W2N5W4_PHYPR/2031-2293 | 3.7e-43 | Phytophthora parasitica |
| W2WUI9_PHYPR/2038-2300 | 3.7e-43 | Phytophthora parasitica CJ01A1 |
| W2GP04_PHYPR/2038-2300 | 3.7e-43 | Phytophthora parasitica |
| A0A081A1F5_PHYPR/2038-2300 | 3.7e-43 | Phytophthora parasitica P1976 |
| W2Q5A3_PHYPN/2038-2300 | 3.7e-43 | Phytophthora parasitica |
| W2Z4L0_PHYPR/2038-2300 | 3.7e-43 | Phytophthora parasitica P10297 |
| U6KVD7_EIMTE/516-834 | 6.3e-43 | Eimeria tenella |
| A0A024W1P8_PLAFA/925-1095 | 2.0e-21 | Plasmodium falciparum Tanzania (2000708) |
| A0A024W1P8_PLAFA/1237-1355 | 1.2e-15 | Plasmodium falciparum Tanzania (2000708) |
| W7JPF5_PLAFO/988-1158 | 2.1e-21 | Plasmodium falciparum |
| W7JPF5_PLAFO/1300-1418 | 1.3e-15 | Plasmodium falciparum |
| W7FTB6_PLAFA/940-1109 | 2.1e-21 | Plasmodium falciparum Santa Lucia |
| W7FTB6_PLAFA/1251-1369 | 1.2e-15 | Plasmodium falciparum Santa Lucia |
| A0A024VM74_PLAFA/925-1094 | 2.0e-21 | Plasmodium falciparum FCH/4 |
| A0A024VM74_PLAFA/1236-1354 | 1.2e-15 | Plasmodium falciparum FCH/4 |
| W4J032_PLAFP/1008-1178 | 2.1e-21 | Plasmodium falciparum |
| W4J032_PLAFP/1320-1438 | 1.2e-15 | Plasmodium falciparum |
| A0A024WJP6_PLAFA/936-1106 | 2.0e-21 | Plasmodium falciparum MaliPS096_E11 |
| A0A024WJP6_PLAFA/1248-1366 | 1.2e-15 | Plasmodium falciparum MaliPS096_E11 |
| W7JRV8_PLAFA/1035-1204 | 2.2e-21 | Plasmodium falciparum UGT5.1 |
| W7JRV8_PLAFA/1346-1464 | 1.3e-15 | Plasmodium falciparum UGT5.1 |
| W4YNU9_STRPU/40-211 | 3.1e-43 | Strongylocentrotus purpuratus |
| C0H5K1_PLAF7/1023-1193 | 2.1e-21 | Plasmodium falciparum |
| C0H5K1_PLAF7/1335-1453 | 1.3e-15 | Plasmodium falciparum |
| B9P4K9_PLAFA/1023-1193 | 2.1e-21 | Plasmodium falciparum |
| B9P4K9_PLAFA/1335-1453 | 1.3e-15 | Plasmodium falciparum |
| A0A024UWD9_PLAFA/1016-1185 | 2.2e-21 | Plasmodium falciparum Vietnam Oak-Knoll (FVO) |
| A0A024UWD9_PLAFA/1327-1445 | 1.3e-15 | Plasmodium falciparum Vietnam Oak-Knoll (FVO) |
| Q4D4P0_TRYCC/113-356 | 4.1e-43 | Trypanosoma cruzi |
| W4ICN2_PLAFA/967-1137 | 2.0e-21 | Plasmodium falciparum NF135/5.C10 |
| W4ICN2_PLAFA/1279-1397 | 1.3e-15 | Plasmodium falciparum NF135/5.C10 |
| W7F2D1_PLAF8/1024-1193 | 2.1e-21 | Plasmodium falciparum (isolate 7G8) |
| W7F2D1_PLAF8/1336-1454 | 1.3e-15 | Plasmodium falciparum (isolate 7G8) |
| G3TCV3_LOXAF/2-257 | 3.7e-43 | Loxodonta africana |
| Q57WJ7_TRYB2/273-518 | 4.4e-43 | Trypanosoma brucei brucei |
| C9ZWY4_TRYB9/190-435 | 4.4e-43 | Trypanosoma brucei gambiense |
| F6Z096_XENTR/1-288 | 2.3e-41 | Xenopus tropicalis |
| I3MXM6_SPETR/19-275 | 5.2e-43 | Spermophilus tridecemlineatus |
| X6MSI7_RETFI/3-203 | 4.4e-43 | Reticulomyxa filosa |
| A0A068WJR9_ECHGR/154-426 | 2.3e-42 | Echinococcus granulosus |
| B2W960_PYRTR/184-390 | 2.7e-42 | Pyrenophora tritici-repentis |
| V5D9W4_TRYCR/305-548 | 5.9e-43 | Trypanosoma cruzi Dm28c |
| K4E888_TRYCR/305-548 | 5.9e-43 | Trypanosoma cruzi |
| Q4DSV7_TRYCC/305-548 | 6.1e-43 | Trypanosoma cruzi |
| C9ZN53_TRYB9/256-501 | 5.4e-43 | Trypanosoma brucei gambiense |
| J9I2J4_9SPIT/119-361 | 6.8e-43 | Oxytricha trifallax |
| Q57XZ9_TRYB2/258-501 | 7.4e-43 | Trypanosoma brucei brucei |
| C5LGQ1_PERM5/282-603 | 9.4e-43 | Perkinsus marinus |
| L8WV44_THACA/110-367 | 1.7e-41 | Thanatephorus cucumeris |
| G7YMA6_CLOSI/312-520 | 7.5e-43 | Clonorchis sinensis |
| G4YGQ3_PHYSP/136-404 | 6.7e-43 | Phytophthora sojae |
| F0UUB3_AJEC8/156-369 | 5.6e-43 | Ajellomyces capsulatus |
| A0A088S1L0_9TRYP/330-593 | 1.4e-42 | Leishmania panamensis |
| A4H3W4_LEIBR/330-593 | 1.6e-42 | Leishmania braziliensis |
| B7Z6G3_HUMAN/43-303 | 8.2e-43 | Homo sapiens |
| A0A091DIB6_FUKDA/43-304 | 1.0e-42 | Fukomys damarensis |
| W9YWI4_FUSOX/9-232 | 9.7e-43 | Fusarium oxysporum f. sp. melonis 26406 |
| V5D9Y2_TRYCR/327-599 | 5.2e-42 | Trypanosoma cruzi Dm28c |
| I1GER3_AMPQE/350-546 | 2.7e-42 | Amphimedon queenslandica |
| F2Z3X0_MOUSE/261-470 | 1.7e-42 | Mus musculus |
| F0WE76_9STRA/2010-2270 | 1.9e-42 | Albugo laibachii Nc14 |
| E9GFW8_DAPPU/24-231 | 2.0e-42 | Daphnia pulex |
| E5LAD0_PLAFA/1005-1175 | 2.1e-21 | Plasmodium falciparum |
| E5LAD0_PLAFA/1317-1435 | 5.7e-15 | Plasmodium falciparum |
| K4DU84_TRYCR/327-599 | 7.5e-42 | Trypanosoma cruzi |
| E3LIL5_CAERE/96-325 | 1.8e-42 | Caenorhabditis remanei |
| Q9U0T9_LEIMA/329-593 | 4.3e-42 | Leishmania major |
| M7AWS9_CHEMY/58-206 | 1.9e-31 | Chelonia mydas |
| M7AWS9_CHEMY/206-284 | 1.5e-05 | Chelonia mydas |
| W7AJ59_PLAVN/98-268 | 6.2e-21 | Plasmodium vinckei petteri |
| W7AJ59_PLAVN/514-634 | 7.5e-16 | Plasmodium vinckei petteri |
| Q4CXJ8_TRYCC/327-599 | 1.1e-41 | Trypanosoma cruzi |
| F9WCD1_TRYCI/429-695 | 3.3e-42 | Trypanosoma congolense |
| G5C3Q6_HETGA/43-304 | 3.1e-42 | Heterocephalus glaber |
| F7IJU4_CALJA/3-182 | 3.9e-42 | Callithrix jacchus |
| A0A078A847_STYLE/55-298 | 3.5e-42 | Stylonychia lemnae |
| A0A078B5K1_STYLE/258-528 | 4.9e-42 | Stylonychia lemnae |
| K7E8B2_ORNAN/2-180 | 3.2e-42 | Ornithorhynchus anatinus |
| B3L6Z7_PLAKH/908-1088 | 5.4e-22 | Plasmodium knowlesi |
| B3L6Z7_PLAKH/1352-1467 | 4.2e-14 | Plasmodium knowlesi |
| D7FTW8_ECTSI/273-437 | 3.3e-35 | Ectocarpus siliculosus |
| F9GEB3_FUSOF/2-242 | 5.7e-42 | Fusarium oxysporum |
| F9GGE3_FUSOF/2-242 | 5.9e-42 | Fusarium oxysporum |
| K2NTR4_TRYCR/307-550 | 7.8e-42 | Trypanosoma cruzi marinkellei |
| Q4DBH4_TRYCC/327-599 | 3.4e-41 | Trypanosoma cruzi |
| H2SDL1_TAKRU/266-476 | 8.2e-42 | Takifugu rubripes |
| F6ZDM9_ORNAN/58-230 | 5.8e-16 | Ornithorhynchus anatinus |
| F6ZDM9_ORNAN/274-351 | 1.3e-19 | Ornithorhynchus anatinus |
| E9AK26_LEIMU/272-534 | 1.9e-41 | Leishmania mexicana |
| A0A0D9QM95_PLAFR/944-1125 | 6.7e-22 | Plasmodium fragile |
| A0A0D9QM95_PLAFR/1386-1501 | 4.7e-14 | Plasmodium fragile |
| W5JM84_ANODA/43-305 | 9.3e-42 | Anopheles darlingi |
| J9IA87_9SPIT/122-366 | 1.1e-41 | Oxytricha trifallax |
| W7A6F5_9APIC/967-1132 | 1.8e-21 | Plasmodium inui San Antonio 1 |
| W7A6F5_9APIC/1415-1530 | 2.3e-14 | Plasmodium inui San Antonio 1 |
| Q4Z3U4_PLABA/698-879 | 3.1e-20 | Plasmodium berghei |
| Q4Z3U4_PLABA/986-1101 | 2.3e-15 | Plasmodium berghei |
| K6UDT5_9APIC/1030-1198 | 1.3e-21 | Plasmodium cynomolgi |
| K6UDT5_9APIC/1477-1592 | 4.8e-14 | Plasmodium cynomolgi |
| M2NLP6_BAUCO/192-415 | 1.5e-41 | Baudoinia compniacensis |
| A0A084W6U6_9DIPT/43-306 | 1.3e-41 | Anopheles sinensis |
| H2YUD8_CIOSA/260-463 | 1.7e-41 | Ciona savignyi |
| A0A061JCE2_TRYRA/206-468 | 1.6e-41 | Trypanosoma rangeli SC58 |
| Q5TUE4_ANOGA/43-305 | 1.6e-41 | Anopheles gambiae |
| A0A024GJ80_9STRA/1986-2247 | 2.0e-41 | Albugo candida |
| G0UJ02_TRYCI/271-526 | 1.7e-41 | Trypanosoma congolense |
| G0UYZ8_TRYCI/286-524 | 1.7e-41 | Trypanosoma congolense |
| G3H4T8_CRIGR/96-337 | 2.0e-41 | Cricetulus griseus |
| A0A0C4BKW1_FUSO4/8-231 | 1.9e-41 | Fusarium oxysporum f. sp. lycopersici |
| A0A077TQP6_PLACH/855-1022 | 1.2e-20 | Plasmodium chabaudi chabaudi |
| A0A077TQP6_PLACH/1276-1391 | 1.4e-15 | Plasmodium chabaudi chabaudi |
| A0A024FYE2_9STRA/527-786 | 4.3e-41 | Albugo candida |
| A0A077XDX4_PLABA/859-1048 | 3.7e-20 | Plasmodium berghei |
| A0A077XDX4_PLABA/1278-1393 | 2.8e-15 | Plasmodium berghei |
| A4HS39_LEIIN/330-594 | 8.5e-41 | Leishmania infantum |
| C9ZY36_TRYB9/333-595 | 2.8e-40 | Trypanosoma brucei gambiense |
| E2AP24_CAMFO/85-351 | 4.3e-41 | Camponotus floridanus |
| W6KWF9_9TRYP/295-566 | 1.3e-40 | Phytomonas sp. isolate EM1 |
| E2BQT3_HARSA/42-309 | 5.0e-41 | Harpegnathos saltator |
| A0A0E9NDM9_9ASCO/25-184 | 8.4e-41 | Saitoella complicata NRRL Y-17804 |
| G0QQ58_ICHMG/147-381 | 7.0e-41 | Ichthyophthirius multifiliis |
| A0A078AKF8_STYLE/97-354 | 6.9e-41 | Stylonychia lemnae |
| A5K2M9_PLAVS/1041-1204 | 3.2e-21 | Plasmodium vivax |
| A5K2M9_PLAVS/1475-1590 | 9.8e-14 | Plasmodium vivax |
| A0A026WHH9_CERBI/42-309 | 6.5e-41 | Cerapachys biroi |
| A0A084WIZ8_9DIPT/79-215 | 7.2e-41 | Anopheles sinensis |
| A4HLR3_LEIBR/390-635 | 9.2e-41 | Leishmania braziliensis |
| Q38EJ3_TRYB2/333-595 | 5.8e-40 | Trypanosoma brucei brucei |
| G2RBL5_THITE/165-399 | 9.1e-41 | Thielavia terrestris |
| A0A061IU18_TRYRA/266-501 | 9.6e-41 | Trypanosoma rangeli SC58 |
| A0A061IXD7_TRYRA/266-501 | 9.7e-41 | Trypanosoma rangeli SC58 |
| F4WBI5_ACREC/42-309 | 9.6e-41 | Acromyrmex echinatior |
| A0A088RZC2_9TRYP/390-618 | 1.2e-40 | Leishmania panamensis |
| V7PPZ6_9APIC/852-1085 | 9.6e-22 | Plasmodium yoelii 17X |
| V7PPZ6_9APIC/1271-1391 | 2.1e-15 | Plasmodium yoelii 17X |
| A0A077Y7L3_9APIC/852-1085 | 9.6e-22 | Plasmodium yoelii |
| A0A077Y7L3_9APIC/1271-1391 | 2.1e-15 | Plasmodium yoelii |
| C9JWY7_HUMAN/2-131 | 9.3e-41 | Homo sapiens |
| K4HR98_9EUKA/1-193 | 9.9e-41 | Collodictyon triciliatum |
| Q7RQW4_PLAYO/852-1085 | 1.0e-21 | Plasmodium yoelii yoelii |
| Q7RQW4_PLAYO/1271-1391 | 2.2e-15 | Plasmodium yoelii yoelii |
| E9AUR0_LEIMU/228-490 | 1.6e-40 | Leishmania mexicana |
| E9BES3_LEIDB/228-490 | 1.6e-40 | Leishmania donovani |
| S9WWG6_9TRYP/205-476 | 1.6e-39 | Angomonas deanei |
| S9WMD1_9TRYP/205-476 | 1.6e-39 | Angomonas deanei |
| Q4QCS6_LEIMA/228-490 | 1.7e-40 | Leishmania major |
| A0A061IVH4_TRYRA/243-496 | 1.7e-40 | Trypanosoma rangeli SC58 |
| T1K732_TETUR/44-301 | 1.8e-40 | Tetranychus urticae |
| G0S747_CHATD/186-414 | 1.8e-40 | Chaetomium thermophilum |
| Q4CP00_TRYCC/270-510 | 1.7e-40 | Trypanosoma cruzi |
| B2W417_PYRTR/122-363 | 7.3e-40 | Pyrenophora tritici-repentis |
| D0NS69_PHYIT/545-806 | 3.2e-40 | Phytophthora infestans |
| E9BQE3_LEIDB/385-630 | 2.1e-40 | Leishmania donovani |
| Q4D609_TRYCC/270-510 | 2.1e-40 | Trypanosoma cruzi |
| A7AP43_BABBO/106-399 | 3.1e-40 | Babesia bovis |
| A0A0F4Z4R6_TALEM/191-423 | 2.3e-40 | Rasamsonia emersonii CBS 393.64 |
| Q17FB6_AEDAE/43-305 | 2.3e-40 | Aedes aegypti |
| E9B809_LEIDB/330-594 | 5.9e-40 | Leishmania donovani |
| A4I963_LEIIN/385-630 | 2.7e-40 | Leishmania infantum |
| B0WDU6_CULQU/43-316 | 2.5e-40 | Culex quinquefasciatus |
| D8LUY8_BLAHO/54-294 | 2.6e-40 | Blastocystis hominis |
| K4E5Y1_TRYCR/266-506 | 2.7e-40 | Trypanosoma cruzi |
| V5DES7_TRYCR/266-506 | 2.7e-40 | Trypanosoma cruzi Dm28c |
| V8P6B0_OPHHA/146-309 | 4.8e-40 | Ophiophagus hannah |
| W6KW41_9TRYP/369-626 | 4.0e-40 | Phytomonas sp. isolate Hart1 |
| A0A088A0L3_APIME/271-517 | 2.2e-38 | Apis mellifera |
| E0VPQ6_PEDHC/56-328 | 3.0e-40 | Pediculus humanus subsp. corporis |
| G6DAQ8_DANPL/2-180 | 2.8e-40 | Danaus plexippus |
| K2MTB2_TRYCR/327-598 | 1.9e-39 | Trypanosoma cruzi marinkellei |
| K7IW90_NASVI/42-313 | 3.5e-40 | Nasonia vitripennis |
| D6NLA6_9TELE/75-211 | 2.8e-40 | Hypomesus transpacificus |
| C5NTJ8_BABBI/107-400 | 5.8e-40 | Babesia bigemina |
| E9B439_LEIMU/385-630 | 3.8e-40 | Leishmania mexicana |
| W9LFW3_FUSOX/32-256 | 2.9e-40 | Fusarium oxysporum f. sp. lycopersici MN25 |
| Q4WK75_ASPFU/134-373 | 4.3e-40 | Neosartorya fumigata |
| B0XMZ7_ASPFC/134-373 | 4.3e-40 | Neosartorya fumigata |
| A0A074XSZ3_AURPU/147-394 | 4.8e-40 | Aureobasidium pullulans EXF-150 |
| S9TQL1_9TRYP/170-441 | 8.7e-40 | Strigomonas culicis |
| B6AAW7_CRYMR/154-450 | 1.2e-39 | Cryptosporidium muris |
| T1HUB2_RHOPR/1-232 | 5.4e-40 | Rhodnius prolixus |
| K3W870_PYTUL/156-412 | 2.5e-39 | Pythium ultimum DAOM BR144 |
| Q4Q3Y6_LEIMA/385-613 | 6.8e-40 | Leishmania major |
| A0A060RYM9_PLARE/1022-1190 | 2.4e-21 | Plasmodium reichenowi |
| A0A060RYM9_PLARE/1342-1464 | 8.1e-16 | Plasmodium reichenowi |
| G2QEM4_THIHA/168-388 | 7.5e-40 | Thielavia heterothallica |
| X0KXL2_FUSOX/13-234 | 9.6e-40 | Fusarium oxysporum f. sp. vasinfectum 25433 |
| W2KZ48_PHYPR/593-856 | 1.3e-39 | Phytophthora parasitica |
| A0A080ZZ07_PHYPR/609-872 | 1.3e-39 | Phytophthora parasitica P1976 |
| W2Q2G9_PHYPN/609-872 | 1.3e-39 | Phytophthora parasitica |
| W2Z1Z1_PHYPR/609-872 | 1.3e-39 | Phytophthora parasitica P10297 |
| W2WRM6_PHYPR/609-872 | 1.3e-39 | Phytophthora parasitica CJ01A1 |
| W2IRH0_PHYPR/609-872 | 1.3e-39 | Phytophthora parasitica |
| W2N3L0_PHYPR/609-872 | 1.3e-39 | Phytophthora parasitica |
| V9EVB8_PHYPR/609-872 | 1.3e-39 | Phytophthora parasitica P1569 |
| F8W4L4_DANRE/4-147 | 1.2e-39 | Danio rerio |
| A0A084BHS6_ASPFM/134-373 | 1.0e-39 | Aspergillus fumigatus var. RP-2014 |
| V9L4Y7_CALMI/10-208 | 1.1e-39 | Callorhinchus milii |
| W5MRF9_LEPOC/335-578 | 4.6e-39 | Lepisosteus oculatus |
| W4YMU4_STRPU/410-574 | 1.3e-39 | Strongylocentrotus purpuratus |
| V5FL43_BYSSN/185-420 | 1.3e-39 | Byssochlamys spectabilis |
| M3CZF4_SPHMS/296-518 | 1.6e-39 | Sphaerulina musiva |
| S9W556_9TRYP/301-572 | 2.9e-39 | Strigomonas culicis |
| W9Z1R8_FUSOX/13-234 | 1.9e-39 | Fusarium oxysporum f. sp. melonis 26406 |
| W9NDT6_FUSOX/13-234 | 1.9e-39 | Fusarium oxysporum f. sp. pisi HDV247 |
| A1D4R9_NEOFI/134-373 | 1.5e-39 | Neosartorya fischeri |
| D5GH66_TUBMM/178-416 | 1.8e-39 | Tuber melanosporum |
| A4HYW3_LEIIN/228-490 | 1.9e-39 | Leishmania infantum |
| H9JIZ1_BOMMO/114-280 | 2.1e-39 | Bombyx mori |
| S9VU83_9TRYP/303-572 | 4.0e-39 | Strigomonas culicis |
| G4ZC15_PHYSP/546-808 | 3.8e-39 | Phytophthora sojae |
| G3QXX3_GORGO/479-673 | 2.5e-39 | Gorilla gorilla gorilla |
| M5C6X5_THACB/116-310 | 2.2e-39 | Thanatephorus cucumeris |
| A0A074W7L4_9PEZI/147-394 | 2.5e-39 | Aureobasidium melanogenum CBS 110374 |
| A0A074W876_9PEZI/147-394 | 3.0e-39 | Aureobasidium namibiae CBS 147.97 |
| C6K3U9_9TRYP/162-426 | 6.3e-39 | Crithidia sp. ATCC 30255 |
| R1DHV2_EMIHU/15-236 | 3.3e-39 | Emiliania huxleyi CCMP1516 |
| A0A078B333_STYLE/102-361 | 3.5e-39 | Stylonychia lemnae |
| F9XM17_ZYMTI/126-393 | 4.1e-39 | Zymoseptoria tritici |
| W4X8U5_ATTCE/98-359 | 4.3e-39 | Atta cephalotes |
| S9VER0_9TRYP/294-567 | 8.0e-39 | Angomonas deanei |
| Q4DJS7_TRYCC/270-510 | 4.6e-39 | Trypanosoma cruzi |
| W6L2F2_9TRYP/292-554 | 2.2e-38 | Phytomonas sp. isolate Hart1 |
| H0ELZ3_GLAL7/12-236 | 5.6e-39 | Glarea lozoyensis |
| I7I9P8_BABMI/72-354 | 6.3e-39 | Babesia microti |
| Q4Q6L9_LEIMA/266-518 | 9.6e-39 | Leishmania major |
| A0A0F4G8H7_9PEZI/8-262 | 6.4e-39 | Zymoseptoria brevis |
| A0A0A2KH62_PENIT/167-390 | 5.0e-39 | Penicillium italicum |
| S9VM07_9TRYP/294-567 | 7.0e-39 | Angomonas deanei |
| A0A061AL06_RHOTO/158-380 | 5.9e-39 | Rhodosporidium toruloides |
| A0A0A2K778_PENEN/177-429 | 5.9e-39 | Penicillium expansum |
| A0A074Y584_9PEZI/188-435 | 7.2e-39 | Aureobasidium subglaciale EXF-2481 |
| Q9GPI9_9TRYP/256-501 | 6.9e-39 | Trypanosoma brucei |
| L8H507_ACACA/248-516 | 7.7e-39 | Acanthamoeba castellanii |
| A0A024UDH4_9STRA/99-350 | 8.1e-39 | Aphanomyces invadans |
| K1ZP83_9BACT/153-373 | 2.1e-38 | uncultured bacterium |
| M2Z3X7_PSEFD/170-330 | 5.5e-29 | Pseudocercospora fijiensis |
| M2Z3X7_PSEFD/333-380 | 0.00012 | Pseudocercospora fijiensis |
| L7JJ24_MAGOP/202-340 | 3.1e-17 | Magnaporthe oryzae |
| L7JJ24_MAGOP/355-423 | 8.2e-17 | Magnaporthe oryzae |
| L7IAL0_MAGOY/202-340 | 3.1e-17 | Magnaporthe oryzae |
| L7IAL0_MAGOY/355-423 | 8.2e-17 | Magnaporthe oryzae |
| V9DIA9_9EURO/156-426 | 8.3e-39 | Cladophialophora carrionii CBS 160.54 |
| K7QT42_PENDI/163-419 | 8.4e-39 | Penicillium digitatum |
| K9F739_PEND1/165-421 | 8.4e-39 | Penicillium digitatum |
| H3GEW3_PHYRM/846-1104 | 1.5e-38 | Phytophthora ramorum |
| Q2H9W2_CHAGB/164-384 | 1.0e-38 | Chaetomium globosum |
| A0A0C2H0P0_9BILA/228-381 | 1.2e-29 | Ancylostoma duodenale |
| A4I6E4_LEIIN/266-519 | 1.6e-38 | Leishmania infantum |
| E9G357_DAPPU/43-315 | 1.0e-38 | Daphnia pulex |
| A0A023B009_GRENI/221-535 | 1.9e-38 | Gregarina niphandrodes |
| F9GDD8_FUSOF/165-293 | 2.4e-19 | Fusarium oxysporum |
| F9GDD8_FUSOF/303-410 | 9.5e-14 | Fusarium oxysporum |
| Q5C3K7_SCHJA/2-169 | 1.3e-38 | Schistosoma japonicum |
| W6UNK2_ECHGR/1222-1375 | 9.6e-28 | Echinococcus granulosus |
| W6UNK2_ECHGR/1462-1541 | 6.1e-05 | Echinococcus granulosus |
| A0A0D9MEP0_9EURO/163-390 | 1.2e-38 | Penicillium solitum |
| F0W3B3_9STRA/532-791 | 5.0e-38 | Albugo laibachii Nc14 |
| S9UTS0_9TRYP/142-413 | 5.1e-38 | Strigomonas culicis |
| I7MM41_TETTS/99-328 | 1.5e-38 | Tetrahymena thermophila |
| D2VN65_NAEGR/75-330 | 2.9e-38 | Naegleria gruberi |
| N1J770_BLUG1/163-413 | 1.5e-38 | Blumeria graminis f. sp. hordei |
| A0A088RS13_9TRYP/228-490 | 1.9e-38 | Leishmania panamensis |
| E9AIH4_LEIBR/228-490 | 1.9e-38 | Leishmania braziliensis |
| S9UK10_9TRYP/206-477 | 6.4e-38 | Strigomonas culicis |
| A0A088RPY0_9TRYP/210-482 | 1.3e-37 | Leishmania panamensis |
| E3NGV9_CAERE/126-342 | 2.1e-38 | Caenorhabditis remanei |
| R7YTI3_CONA1/160-412 | 1.8e-38 | Coniosporium apollinis |
| Q4R721_MACFA/1-158 | 2.6e-38 | Macaca fascicularis |
| W6PTA0_PENRO/175-389 | 1.9e-38 | Penicillium roqueforti FM164 |
| Q4CLS9_TRYCC/81-354 | 8.1e-38 | Trypanosoma cruzi |
| E9AIH3_LEIBR/208-480 | 1.5e-37 | Leishmania braziliensis |
| L0B128_BABEQ/120-425 | 3.0e-38 | Babesia equi |
| C9ZIE8_TRYB9/213-467 | 2.1e-38 | Trypanosoma brucei gambiense |
| Q4GZ10_TRYB2/213-467 | 2.1e-38 | Trypanosoma brucei brucei |
| D2VZZ5_NAEGR/300-560 | 2.4e-38 | Naegleria gruberi |
| A0A0C1DFU1_9NOCA/6534-6647 | 1.5e-07 | Nocardia vulneris |
| K2NFL2_TRYCR/270-506 | 2.6e-38 | Trypanosoma cruzi marinkellei |
| E3MBK9_CAERE/102-300 | 3.0e-38 | Caenorhabditis remanei |
| K2MS18_TRYCR/289-564 | 1.0e-37 | Trypanosoma cruzi marinkellei |
| E5A3M6_LEPMJ/324-575 | 2.8e-38 | Leptosphaeria maculans |
| A0A0F0I4L1_ASPPA/182-396 | 2.6e-38 | Aspergillus parasiticus SU-1 |
| A0A078B2D4_STYLE/102-361 | 3.2e-38 | Stylonychia lemnae |
| F6QRQ7_CIOIN/101-238 | 2.8e-38 | Ciona intestinalis |
| A0A0C3HSW7_9PEZI/129-382 | 3.1e-38 | Oidiodendron maius Zn |
| E9AUQ9_LEIMU/208-480 | 1.3e-37 | Leishmania mexicana |
| PALB_EMENI/185-389 | 3.9e-38 | Emericella nidulans |
| H3E5Z7_PRIPA/29-176 | 2.7e-38 | Pristionchus pacificus |
| M2UV59_COCH5/264-504 | 4.0e-38 | Cochliobolus heterostrophus |
| N1PD42_DOTSN/200-435 | 7.9e-38 | Dothistroma septosporum |
| E9BES2_LEIDB/208-480 | 1.5e-37 | Leishmania donovani |
| D7FSU2_ECTSI/57-223 | 1.8e-30 | Ectocarpus siliculosus |
| D8UEL0_VOLCA/117-264 | 2.3e-26 | Volvox carteri |
| D8UEL0_VOLCA/300-369 | 6.8e-06 | Volvox carteri |
| A4HYW2_LEIIN/208-480 | 1.6e-37 | Leishmania infantum |
| G1PVC2_MYOLU/75-325 | 2.4e-37 | Myotis lucifugus |
| F7VYG4_SORMK/162-397 | 4.5e-38 | Sordaria macrospora |
| PALB_ASPOR/184-391 | 4.3e-38 | Aspergillus oryzae |
| I8TK56_ASPO3/184-391 | 4.3e-38 | Aspergillus oryzae |
| A0A064B913_ASPOZ/184-391 | 4.3e-38 | Aspergillus oryzae 100-8 |
| B8MY71_ASPFN/184-391 | 4.3e-38 | Aspergillus flavus |
| C0NS99_AJECG/163-374 | 1.7e-37 | Ajellomyces capsulatus |
| M2RQH4_COCSN/263-503 | 5.3e-38 | Cochliobolus sativus |
| K4E5I8_TRYCR/291-564 | 2.4e-37 | Trypanosoma cruzi |
| W9XD27_9EURO/173-426 | 6.1e-38 | Capronia epimyces CBS 606.96 |
| W9VWI3_9EURO/170-426 | 4.8e-38 | Cladophialophora yegresii CBS 114405 |
| V5BN20_TRYCR/291-564 | 2.5e-37 | Trypanosoma cruzi Dm28c |
| A0A059JF20_9EURO/184-424 | 5.0e-38 | Trichophyton interdigitale MR816 |
| A0A022UJU4_9EURO/184-424 | 5.0e-38 | Trichophyton interdigitale H6 |
| A0A017S3X0_9EURO/182-417 | 5.3e-38 | Aspergillus ruber CBS 135680 |
| F2PTH7_TRIEC/184-424 | 5.4e-38 | Trichophyton equinum |
| M7U162_BOTF1/159-419 | 5.7e-38 | Botryotinia fuckeliana |
| F8MMZ9_NEUT8/212-444 | 6.1e-38 | Neurospora tetrasperma |
| A0A0D6LD07_9BILA/156-309 | 5.5e-30 | Ancylostoma ceylanicum |
| G0NC27_CAEBE/123-346 | 6.7e-38 | Caenorhabditis brenneri |
| Q9GPI8_9TRYP/298-573 | 5.2e-37 | Trypanosoma brucei |
| C9ZN52_TRYB9/298-573 | 5.3e-37 | Trypanosoma brucei gambiense |
| Q57XZ8_TRYB2/298-573 | 5.3e-37 | Trypanosoma brucei brucei |
| G0NC28_CAEBE/124-341 | 4.6e-38 | Caenorhabditis brenneri |
| B6H1Y4_PENCW/175-388 | 6.2e-38 | Penicillium chrysogenum |
| PALB_NEUCR/159-390 | 7.3e-38 | Neurospora crassa |
| B8MH71_TALSN/187-419 | 9.8e-38 | Talaromyces stipitatus |
| A0A0A9XHA3_LYGHE/54-347 | 1.1e-37 | Lygus hesperus |
| A0A077ZUN7_STYLE/48-285 | 7.7e-38 | Stylonychia lemnae |
| F7HTR3_CALJA/73-164 | 2.3e-15 | Callithrix jacchus |
| F7HTR3_CALJA/232-320 | 9.5e-17 | Callithrix jacchus |
| Q4QCS7_LEIMA/208-480 | 3.1e-37 | Leishmania major |
| W7E4P7_COCVI/264-504 | 8.6e-38 | Bipolaris victoriae FI3 |
| W6YC69_COCCA/264-504 | 8.6e-38 | Bipolaris zeicola 26-R-13 |
| U9WGW4_NEUCR/10-240 | 9.3e-38 | Neurospora crassa |
| G0TR85_TRYVY/202-458 | 8.8e-38 | Trypanosoma vivax |
| G0TZZ7_TRYVY/275-513 | 8.4e-38 | Trypanosoma vivax |
| A0A0D2DSC6_9EURO/181-426 | 1.0e-37 | Capronia semiimmersa |
| F2S9V6_TRIT1/184-424 | 9.4e-38 | Trichophyton tonsurans |
| D0A6V9_TRYB9/326-569 | 1.1e-37 | Trypanosoma brucei gambiense |
| B2AW31_PODAN/139-363 | 1.2e-37 | Podospora anserina |
| S3DCE7_GLAL2/172-421 | 1.1e-37 | Glarea lozoyensis |
| O02260_CAEEL/119-334 | 1.4e-37 | Caenorhabditis elegans |
| A8NAE6_COPC7/156-436 | 1.2e-36 | Coprinopsis cinerea |
| C6K3U5_9TRYP/300-574 | 1.3e-37 | Crithidia sp. ATCC 30255 |
| G0R675_ICHMG/226-460 | 8.1e-38 | Ichthyophthirius multifiliis |
| D8TN33_VOLCA/277-478 | 2.7e-36 | Volvox carteri |
| A0A090DBF2_PODAN/140-363 | 1.4e-37 | Podospora anserina |
| W9CLC6_9HELO/169-422 | 1.5e-37 | Sclerotinia borealis F-4157 |
| A0A074ZM92_9TREM/236-378 | 1.6e-37 | Opisthorchis viverrini |
| A4HJ14_LEIBR/300-574 | 3.5e-37 | Leishmania braziliensis |
| A0A088RWU9_9TRYP/300-574 | 3.7e-37 | Leishmania panamensis |
| W6KEE2_9TRYP/390-637 | 1.7e-37 | Phytomonas sp. isolate EM1 |
| A0A0B7A7L9_9EUPU/1-177 | 1.4e-37 | Arion vulgaris |
| A0A0B1NX46_UNCNE/187-395 | 2.4e-37 | Erysiphe necator |
| A0A067C4C6_SAPPC/66-345 | 1.6e-37 | Saprolegnia parasitica CBS 223.65 |
| M4BRQ6_HYAAE/592-848 | 2.3e-37 | Hyaloperonospora arabidopsidis |
| E4V096_ARTGP/184-425 | 1.6e-37 | Arthroderma gypseum |
| A0A077ZUS6_STYLE/79-313 | 2.6e-37 | Stylonychia lemnae |
| C1E1D1_MICSR/250-543 | 3.5e-37 | Micromonas sp. |
| E9BMG9_LEIDB/266-519 | 3.2e-37 | Leishmania donovani |
| D4AJS8_ARTBC/216-456 | 2.2e-37 | Arthroderma benhamiae |
| A0A078B2H8_STYLE/98-355 | 3.2e-37 | Stylonychia lemnae |
| D2V3U1_NAEGR/290-552 | 3.4e-37 | Naegleria gruberi |
| A4I6K4_LEIIN/300-573 | 4.3e-37 | Leishmania infantum |
| B6QNP8_PENMQ/174-422 | 3.0e-37 | Penicillium marneffei |
| E9BMG5_LEIDB/300-573 | 4.3e-37 | Leishmania donovani |
| F7HTY5_CALJA/109-194 | 1.5e-14 | Callithrix jacchus |
| F7HTY5_CALJA/274-363 | 6.3e-17 | Callithrix jacchus |
| K1WWK2_MARBU/146-409 | 2.9e-37 | Marssonina brunnea f. sp. multigermtubi |
| W6YKX6_COCMI/263-502 | 3.3e-37 | Bipolaris oryzae ATCC 44560 |
| E9B1J0_LEIMU/300-573 | 5.7e-37 | Leishmania mexicana |
| W4FGZ3_9STRA/97-346 | 3.4e-37 | Aphanomyces astaci |
| E9AUQ8_LEIMU/245-446 | 3.2e-37 | Leishmania mexicana |
| D4D8S9_TRIVH/184-424 | 3.1e-37 | Trichophyton verrucosum) |
| T0QIA7_9STRA/65-345 | 3.2e-37 | Saprolegnia diclina VS20 |
| M4A2X0_XIPMA/24-232 | 1.0e-36 | Xiphophorus maculatus |
| A0A078ABZ3_STYLE/98-355 | 3.9e-37 | Stylonychia lemnae |
| C5P0Z2_COCP7/160-426 | 3.3e-37 | Coccidioides posadasii |
| A0A0E1RZ13_COCIM/160-426 | 3.3e-37 | Coccidioides immitis RS |
| E9DDN7_COCPS/122-388 | 3.5e-37 | Coccidioides posadasii |
| G3WJK0_SARHA/71-305 | 3.8e-37 | Sarcophilus harrisii |
| C3YB16_BRAFL/29-175 | 3.1e-37 | Branchiostoma floridae |
| A0A077ZWW9_STYLE/104-349 | 4.4e-37 | Stylonychia lemnae |
| G0TUR5_TRYVY/294-569 | 1.4e-36 | Trypanosoma vivax |
| A0A078AGF2_STYLE/98-355 | 7.8e-37 | Stylonychia lemnae |
| G0V096_TRYCI/306-549 | 7.2e-37 | Trypanosoma congolense |
| Q4Q6M4_LEIMA/371-644 | 9.9e-37 | Leishmania major |
| B2W8I0_PYRTR/306-517 | 6.7e-37 | Pyrenophora tritici-repentis |
| Q0CUT6_ASPTN/181-383 | 5.9e-37 | Aspergillus terreus |
| A0A0D9RU15_CHLSB/1-146 | 6.9e-37 | Chlorocebus sabaeus |
| F6ZVH3_CIOIN/54-300 | 7.3e-37 | Ciona intestinalis |
| A4HJ23_LEIBR/21-279 | 1.3e-36 | Leishmania braziliensis |
| W9Y7K2_9EURO/173-426 | 7.8e-37 | Capronia coronata CBS 617.96 |
| H3HC70_PHYRM/161-262 | 5.3e-19 | Phytophthora ramorum |
| H3HC70_PHYRM/270-332 | 1.2e-11 | Phytophthora ramorum |
| A0A0A1TZL5_ENTIV/35-239 | 8.2e-37 | Entamoeba invadens IP1 |
| A0A061A6U1_ONCMY/15-145 | 7.2e-37 | Oncorhynchus mykiss |
| W2RL15_9EURO/180-429 | 8.8e-37 | Cyphellophora europaea CBS 101466 |
| L8G0S6_PSED2/180-421 | 9.0e-37 | Pseudogymnoascus destructans |
| H6C735_EXODN/182-430 | 8.6e-37 | Exophiala dermatitidis |
| C1E045_MICSR/189-485 | 3.4e-36 | Micromonas sp. |
| A0A0D2HG80_9EURO/187-426 | 1.1e-36 | Cladophialophora bantiana CBS 173.52 |
| C5FTB2_ARTOC/166-426 | 1.4e-36 | Arthroderma otae |
| R0IZ85_SETT2/338-550 | 1.7e-36 | Setosphaeria turcica |
| G7XP79_ASPKW/189-402 | 1.8e-36 | Aspergillus kawachii |
| C5GB86_AJEDR/191-405 | 2.0e-36 | Ajellomyces dermatitidis |
| T5BE47_AJEDE/191-405 | 2.0e-36 | Blastomyces dermatitidis ATCC 26199 |
| F2TM96_AJEDA/191-405 | 2.0e-36 | Ajellomyces dermatitidis |
| J9FNR8_9SPIT/7-199 | 1.6e-36 | Oxytricha trifallax |
| E3N2T4_CAERE/88-324 | 1.3e-36 | Caenorhabditis remanei |
| Q385V3_TRYB2/265-508 | 2.0e-36 | Trypanosoma brucei brucei |
| A0A0D1ZLS2_9EURO/152-420 | 2.5e-36 | Exophiala mesophila |
| S9X067_9TRYP/304-544 | 2.4e-36 | Angomonas deanei |
| A0A077ZUH0_STYLE/99-352 | 2.3e-36 | Stylonychia lemnae |
| E3MBF4_CAERE/54-256 | 2.4e-36 | Caenorhabditis remanei |
| E3N2T3_CAERE/136-370 | 5.1e-36 | Caenorhabditis remanei |
| A0A078B8X6_STYLE/96-353 | 3.3e-36 | Stylonychia lemnae |
| A0A0D2I4C1_9EURO/190-429 | 2.9e-36 | Fonsecaea multimorphosa CBS 102226 |
| A0A094H5D5_9PEZI/176-421 | 2.9e-36 | Pseudogymnoascus pannorum VKM F-4519 (FW-2642) |
| Q4DQN5_TRYCC/220-486 | 3.1e-36 | Trypanosoma cruzi |
| X0IMZ7_FUSOX/23-187 | 4.5e-36 | Fusarium oxysporum f. sp. cubense |
| R1C981_EMIHU/85-214 | 3.4e-35 | Emiliania huxleyi CCMP1516 |
| G0TR82_TRYVY/480-745 | 4.1e-36 | Trypanosoma vivax |
| T1IAM6_RHOPR/8-190 | 1.3e-35 | Rhodnius prolixus |
| C1H490_PARBA/207-420 | 5.7e-36 | Paracoccidioides lutzii) |
| A0A016WAQ4_9BILA/13-174 | 6.1e-36 | Ancylostoma ceylanicum |
| A2Q8K6_ASPNC/187-403 | 7.5e-36 | Aspergillus niger |
| G3XS48_ASPNA/187-403 | 7.5e-36 | Aspergillus niger |
| S4R9N5_PETMA/46-117 | 6.4e-12 | Petromyzon marinus |
| S4R9N5_PETMA/213-290 | 2.8e-18 | Petromyzon marinus |
| K2MSX8_TRYCR/220-487 | 7.5e-36 | Trypanosoma cruzi marinkellei |
| E3MBL2_CAERE/78-290 | 8.7e-36 | Caenorhabditis remanei |
| M3A060_PSEFD/168-401 | 9.0e-36 | Pseudocercospora fijiensis |
| C1GKM3_PARBD/201-414 | 8.2e-36 | Paracoccidioides brasiliensis |
| W9X2N8_9EURO/190-429 | 8.6e-36 | Cladophialophora psammophila CBS 110553 |
| S9VT81_9TRYP/213-457 | 9.4e-36 | Angomonas deanei |
| U4L9L5_PYROM/234-474 | 9.9e-36 | Pyronema omphalodes |
| A0A072PFP0_9EURO/185-425 | 1.0e-35 | Exophiala aquamarina CBS 119918 |
| S9URE4_9TRYP/213-457 | 9.4e-36 | Angomonas deanei |
| E8NHG6_LEIMU/267-519 | 1.7e-35 | Leishmania mexicana |
| R1CA79_EMIHU/154-283 | 1.1e-35 | Emiliania huxleyi CCMP1516 |
| G0NNN4_CAEBE/81-324 | 1.1e-35 | Caenorhabditis brenneri |
| F0XQK2_GROCL/184-406 | 1.3e-35 | Grosmannia clavigera |
| A0A088SFS7_9TRYP/263-521 | 2.3e-35 | Leishmania panamensis |
| U5GXT6_USTV1/242-456 | 1.5e-35 | Microbotryum violaceum |
| U5GXT5_USTV1/243-457 | 1.5e-35 | Microbotryum violaceum |
| A0A094GL78_9PEZI/176-421 | 1.4e-35 | Pseudogymnoascus pannorum VKM F-103 |
| A0A094FAI8_9PEZI/180-421 | 1.4e-35 | Pseudogymnoascus pannorum VKM F-4518 (FW-2643) |
| E6ZZD6_SPORE/189-421 | 1.9e-35 | Sporisorium reilianum |
| A7ENU8_SCLS1/141-389 | 1.6e-35 | Sclerotinia sclerotiorum |
| K2PAG9_TRYCR/281-556 | 7.1e-35 | Trypanosoma cruzi marinkellei |
| K1PHF5_CRAGI/114-273 | 4.2e-35 | Crassostrea gigas |
| A0A0D2HFH1_9EURO/187-426 | 1.8e-35 | Fonsecaea pedrosoi CBS 271.37 |
| L7ITR2_MAGOP/166-417 | 2.0e-35 | Magnaporthe oryzae |
| L7INR1_MAGOY/166-417 | 2.0e-35 | Magnaporthe oryzae |
| G0Z5J3_MAGOR/166-417 | 2.0e-35 | Magnaporthe oryzae |
| G4N7X4_MAGO7/167-418 | 2.0e-35 | Magnaporthe oryzae |
| A0A0D1Z562_9EURO/187-426 | 2.0e-35 | Cladophialophora immunda |
| A0A016WAT6_9BILA/13-172 | 2.5e-35 | Ancylostoma ceylanicum |
| C4JVW0_UNCRE/174-418 | 3.1e-35 | Uncinocarpus reesii |
| A0A078B8I1_STYLE/99-352 | 3.2e-35 | Stylonychia lemnae |
| V5BEL3_TRYCR/220-486 | 2.9e-35 | Trypanosoma cruzi Dm28c |
| S4RWU0_PETMA/3-167 | 2.8e-35 | Petromyzon marinus |
| G2X8X8_VERDV/160-400 | 3.0e-35 | Verticillium dahliae |
| W6LDQ2_9TRYP/331-571 | 3.4e-35 | Phytomonas sp. isolate Hart1 |
| L2GBQ8_COLGN/1-116 | 4.7e-17 | Colletotrichum gloeosporioides |
| L2GBQ8_COLGN/113-191 | 2.5e-14 | Colletotrichum gloeosporioides |
| F2TVF3_SALR5/362-530 | 3.5e-35 | Salpingoeca rosetta |
| K4DTH5_TRYCR/220-486 | 3.7e-35 | Trypanosoma cruzi |
| W2L1D7_PHYPR/2041-2293 | 7.1e-35 | Phytophthora parasitica |
| A0A094GK53_9PEZI/180-421 | 3.8e-35 | Pseudogymnoascus pannorum VKM F-4520 (FW-2644) |
| K1PA48_CRAGI/110-270 | 5.1e-35 | Crassostrea gigas |
| A0A0C9RBV4_9HYME/47-284 | 3.9e-35 | Fopius arisanus |
| E3MBF3_CAERE/70-269 | 4.5e-35 | Caenorhabditis remanei |
| G3JH95_CORMM/151-365 | 4.8e-35 | Cordyceps militaris |
| Q4D610_TRYCC/226-501 | 1.6e-34 | Trypanosoma cruzi |
| A0A0C4DRQ2_MAGP6/192-412 | 5.3e-35 | Magnaporthiopsis poae |
| R8BFP0_TOGMI/138-358 | 6.6e-35 | Togninia minima |
| Q4E0D7_TRYCC/220-486 | 6.4e-35 | Trypanosoma cruzi |
| A0A0D2IZ07_9EURO/178-426 | 7.5e-35 | Rhinocladiella mackenziei CBS 650.93 |
| Q5CW80_CRYPI/164-363 | 2.2e-17 | Cryptosporidium parvum |
| Q5CW80_CRYPI/408-487 | 2.2e-11 | Cryptosporidium parvum |
| L5LH31_MYODS/261-522 | 8.4e-35 | Myotis davidii |
| E3RW47_PYRTT/330-537 | 8.0e-35 | Pyrenophora teres f. teres |
| Q99MT5_MOUSE/1-139 | 5.5e-35 | Mus musculus |
| A0A078B6R7_STYLE/104-363 | 7.7e-35 | Stylonychia lemnae |
| Q4DJS8_TRYCC/226-501 | 2.3e-34 | Trypanosoma cruzi |
| E4XMI4_OIKDI/92-292 | 8.0e-35 | Oikopleura dioica |
| U1HYD0_ENDPU/186-440 | 7.8e-35 | Endocarpon pusillum |
| W8BUB8_CERCA/49-310 | 8.7e-35 | Ceratitis capitata |
| G9MX91_HYPVG/173-393 | 1.1e-34 | Hypocrea virens |
| A0A0D2ENC4_9EURO/171-424 | 1.1e-34 | Exophiala xenobiotica |
| A0A094C0S8_9PEZI/180-421 | 1.1e-34 | Pseudogymnoascus pannorum VKM F-4515 (FW-2607) |
| A0A093EUL6_TAUER/1-130 | 8.7e-35 | Tauraco erythrolophus |
| S8AQ32_PENO1/173-386 | 1.1e-34 | Penicillium oxalicum |
| A0A034V743_BACDO/52-310 | 1.2e-34 | Bactrocera dorsalis |
| J9EDC6_WUCBA/195-403 | 1.8e-34 | Wuchereria bancrofti |
| A0A078ABC0_STYLE/102-353 | 1.8e-34 | Stylonychia lemnae |
| K9MPU0_EIMTE/20-221 | 1.8e-34 | Eimeria tenella |
| Q9CPY2_MOUSE/43-193 | 1.5e-34 | Mus musculus |
| G0RWU9_HYPJQ/137-384 | 1.9e-34 | Hypocrea jecorina |
| A0A024RV47_HYPJE/140-387 | 1.9e-34 | Trichoderma reesei RUT C-30 |
| A0A093R133_PHACA/1-131 | 1.5e-34 | Phalacrocorax carbo |
| A0A067MWR6_9HOMO/148-362 | 2.2e-34 | Botryobasidium botryosum FD-172 SS1 |
| A0A0A1WKJ6_BACCU/52-310 | 2.1e-34 | Bactrocera cucurbitae |
| A0A091MFH5_CATAU/1-131 | 1.7e-34 | Cathartes aura |
| A0A093EVY1_GAVST/1-130 | 1.8e-34 | Gavia stellata |
| A0A016WBP9_9BILA/13-179 | 2.4e-34 | Ancylostoma ceylanicum |
| J3P7N4_GAGT3/185-411 | 2.4e-34 | Gaeumannomyces graminis var. tritici |
| A0A094F0N8_9PEZI/213-454 | 2.7e-34 | Pseudogymnoascus pannorum VKM F-4517 (FW-2822) |
| A0A0D2A7W4_9PEZI/218-428 | 3.1e-34 | Verruconis gallopava |
| M7WVF4_RHOT1/187-420 | 3.7e-34 | Rhodosporidium toruloides |
| H1A1G1_TAEGU/1-131 | 2.2e-34 | Taeniopygia guttata |
| B4M289_DROVI/43-302 | 3.4e-34 | Drosophila virilis |
| C7YZ68_NECH7/139-381 | 3.5e-34 | Nectria haematococca |
| A0A0E9NFR7_9ASCO/193-438 | 3.9e-34 | Saitoella complicata NRRL Y-17804 |
| M7SY07_EUTLA/163-420 | 3.7e-34 | Eutypa lata |
| A0A078B1A5_STYLE/204-422 | 5.0e-34 | Stylonychia lemnae |
| K4E5J3_TRYCR/288-563 | 1.2e-33 | Trypanosoma cruzi |
| A0A060T1Q7_BLAAD/143-366 | 4.4e-34 | Blastobotrys adeninivorans |
| U6CZ50_NEOVI/4-146 | 3.2e-34 | Neovison vison |
| I2FZN3_USTH4/184-427 | 9.0e-34 | Ustilago hordei |
| K3VDG0_FUSPC/146-385 | 3.8e-34 | Fusarium pseudograminearum |
| C3ZHX2_BRAFL/115-230 | 2.1e-30 | Branchiostoma floridae |
| E3N2T5_CAERE/116-353 | 5.2e-34 | Caenorhabditis remanei |
| V5AYJ1_TRYCR/228-501 | 1.7e-33 | Trypanosoma cruzi Dm28c |
| J9JBN8_9SPIT/120-356 | 6.4e-34 | Oxytricha trifallax |
| S9TWF1_9TRYP/217-456 | 5.2e-34 | Strigomonas culicis |
| X0AA76_FUSOX/107-355 | 4.9e-34 | Fusarium oxysporum f. sp. melonis 26406 |
| E7KGT5_YEASA/86-350 | 9.0e-34 | Saccharomyces cerevisiae |
| A0A0D3WZD9_YEASX/86-350 | 9.0e-34 | Saccharomyces cerevisiae YJM1244 |
| X0AXP2_FUSOX/138-386 | 5.3e-34 | Fusarium oxysporum f. sp. melonis 26406 |
| A0A0D3X320_YEASX/86-350 | 5.5e-34 | Saccharomyces cerevisiae YJM1252 |
| Q29GJ4_DROPS/44-302 | 6.2e-34 | Drosophila pseudoobscura pseudoobscura |
| B4H111_DROPE/44-302 | 6.2e-34 | Drosophila persimilis |
| A0A093XG22_9PEZI/175-414 | 7.1e-34 | Pseudogymnoascus pannorum VKM F-3557 |
| K2QQJ0_MACPH/187-401 | 7.9e-34 | Macrophomina phaseolina |
| A0A0A9ZB15_LYGHE/9-205 | 6.6e-34 | Lygus hesperus |
| A0A093Z2Q4_9PEZI/176-414 | 7.0e-34 | Pseudogymnoascus pannorum VKM F-4246 |
| X0HX96_FUSOX/56-262 | 8.0e-34 | Fusarium oxysporum f. sp. conglutinans race 2 54008 |
| A0A010S495_9PEZI/167-388 | 7.5e-34 | Colletotrichum fioriniae PJ7 |
| A0A0D3Y909_YEASX/86-350 | 6.9e-34 | Saccharomyces cerevisiae YJM1447 |
| A0A0B7KC82_BIOOC/153-369 | 1.0e-33 | Bionectria ochroleuca |
| A0A0F4ZFF6_9PEZI/168-372 | 1.1e-33 | Thielaviopsis punctulata |
| A0A0C7CEG1_9FUNG/321-465 | 1.0e-33 | Rhizopus microsporus |
| B5VPS6_YEAS6/86-350 | 8.1e-34 | Saccharomyces cerevisiae |
| G2WKK5_YEASK/86-350 | 9.0e-34 | Saccharomyces cerevisiae |
| A6ZML2_YEAS7/86-350 | 9.0e-34 | Saccharomyces cerevisiae |
| A0A0D3XLU5_YEASX/86-350 | 9.0e-34 | Saccharomyces cerevisiae YJM1385 |
| A0A0D3VQZ2_YEASX/86-350 | 9.0e-34 | Saccharomyces cerevisiae YJM271 |
| A0A024Y0R2_YEASX/86-350 | 9.0e-34 | Saccharomyces cerevisiae R103 |
| A0A0D3VPN9_YEASX/86-350 | 9.0e-34 | Saccharomyces cerevisiae YJM270 |
| B3LM41_YEAS1/86-350 | 9.0e-34 | Saccharomyces cerevisiae |
| A0A0D3VJJ1_YEASX/86-350 | 9.0e-34 | Saccharomyces cerevisiae YJM193 |
| A0A0D3XZ86_YEASX/86-350 | 9.0e-34 | Saccharomyces cerevisiae YJM1417 |
| A0A0D3VQ68_YEASX/86-350 | 9.0e-34 | Saccharomyces cerevisiae YJM248 |
| N1NYV6_YEASC/86-350 | 9.0e-34 | Saccharomyces cerevisiae |
| A0A0D3XI94_YEASX/86-350 | 9.0e-34 | Saccharomyces cerevisiae YJM1356 |
| A0A0D3WKL1_YEASX/86-350 | 9.0e-34 | Saccharomyces cerevisiae YJM987 |
| A0A0D3VK97_YEASX/86-350 | 9.0e-34 | Saccharomyces cerevisiae YJM189 |
| A0A0D3W857_YEASX/86-350 | 9.0e-34 | Saccharomyces cerevisiae YJM627 |
| A0A023ZEY9_YEASX/86-350 | 9.0e-34 | Saccharomyces cerevisiae YJM993 |
| C8ZF12_YEAS8/86-350 | 9.0e-34 | Saccharomyces cerevisiae |
| A0A0D3WEE4_YEASX/86-350 | 9.0e-34 | Saccharomyces cerevisiae YJM972 |
| A0A0D3XE47_YEASX/86-350 | 9.0e-34 | Saccharomyces cerevisiae YJM1336 |
| A0A0D3WPY6_YEASX/86-350 | 9.0e-34 | Saccharomyces cerevisiae YJM996 |
| A0A0D3VP10_YEASX/86-350 | 9.0e-34 | Saccharomyces cerevisiae YJM244 |
| A0A0D3WJJ9_YEASX/86-350 | 9.0e-34 | Saccharomyces cerevisiae YJM984 |
| A0A0D3VY60_YEASX/86-350 | 9.0e-34 | Saccharomyces cerevisiae YJM453 |
| A0A0D3WI61_YEASX/86-350 | 9.0e-34 | Saccharomyces cerevisiae YJM981 |
| W7PBZ1_YEASX/86-350 | 9.0e-34 | Saccharomyces cerevisiae R008 |
| W7R777_YEASX/86-350 | 9.0e-34 | Saccharomyces cerevisiae P283 |
| A0A0D3XPH5_YEASX/86-350 | 9.0e-34 | Saccharomyces cerevisiae YJM1387 |
| A0A0D3WJN1_YEASX/86-350 | 9.0e-34 | Saccharomyces cerevisiae YJM978 |
| A0A0D3XIZ5_YEASX/86-350 | 9.0e-34 | Saccharomyces cerevisiae YJM1355 |
| A0A0D3XAL3_YEASX/86-350 | 9.0e-34 | Saccharomyces cerevisiae YJM1332 |
| A0A0D3VUE1_YEASX/86-350 | 9.0e-34 | Saccharomyces cerevisiae YJM428 |
| A0A0D3WPG0_YEASX/86-350 | 9.0e-34 | Saccharomyces cerevisiae YJM990 |
| A0A0D3WD45_YEASX/86-350 | 9.0e-34 | Saccharomyces cerevisiae YJM969 |
| A0A024XJ19_YEASX/86-350 | 9.0e-34 | Saccharomyces cerevisiae P301 |
| A0A0D3WY02_YEASX/86-350 | 9.0e-34 | Saccharomyces cerevisiae YJM1242 |
| A0A0D3WIB9_YEASX/86-350 | 9.0e-34 | Saccharomyces cerevisiae YJM975 |
| A0A0D3WBP2_YEASX/86-350 | 9.0e-34 | Saccharomyces cerevisiae YJM693 |
| A0A0D3YEB1_YEASX/86-350 | 9.0e-34 | Saccharomyces cerevisiae YJM1477 |
| A0A0D3WCL1_YEASX/86-350 | 9.0e-34 | Saccharomyces cerevisiae YJM689 |
| A0A0D3WSH0_YEASX/86-350 | 9.0e-34 | Saccharomyces cerevisiae YJM1129 |
| A0A0D3Y0E0_YEASX/86-350 | 9.0e-34 | Saccharomyces cerevisiae YJM1415 |
| A0A0D3YQ98_YEASX/86-350 | 9.0e-34 | Saccharomyces cerevisiae YJM1574 |
| H0GL68_SACCK/86-350 | 9.0e-34 | Saccharomyces cerevisiae x Saccharomyces kudriavzevii |
| A0A0D3YK92_YEASX/86-350 | 9.0e-34 | Saccharomyces cerevisiae YJM1549 |
| A0A0D3YHR7_YEASX/86-350 | 9.0e-34 | Saccharomyces cerevisiae YJM1526 |
| A0A0D3YA89_YEASX/86-350 | 9.0e-34 | Saccharomyces cerevisiae YJM1444 |
| A0A0D3Y2W4_YEASX/86-350 | 9.0e-34 | Saccharomyces cerevisiae YJM1433 |
| A0A0D3XG67_YEASX/86-350 | 9.0e-34 | Saccharomyces cerevisiae YJM1341 |
| RIM13_YEAST/86-350 | 9.0e-34 | Saccharomyces cerevisiae |
| A0A0D3VV56_YEASX/86-350 | 9.0e-34 | Saccharomyces cerevisiae YJM326 |
| A0A0D3WWQ3_YEASX/86-350 | 9.0e-34 | Saccharomyces cerevisiae YJM1208 |
| A0A0D3VRW1_YEASX/86-350 | 9.0e-34 | Saccharomyces cerevisiae YJM320 |
| A0A0D3W6B3_YEASX/86-350 | 9.0e-34 | Saccharomyces cerevisiae YJM555 |
| A0A0D3W829_YEASX/86-350 | 9.0e-34 | Saccharomyces cerevisiae YJM682 |
| A0A0D3W328_YEASX/86-350 | 9.0e-34 | Saccharomyces cerevisiae YJM554 |
| A0A0D3W9G0_YEASX/86-350 | 9.0e-34 | Saccharomyces cerevisiae YJM681 |
| A0A0D3WPE0_YEASX/86-350 | 9.0e-34 | Saccharomyces cerevisiae YJM1083 |
| A0A0D3YGL8_YEASX/86-350 | 9.0e-34 | Saccharomyces cerevisiae YJM1479 |
| A0A0D3XU61_YEASX/86-350 | 9.0e-34 | Saccharomyces cerevisiae YJM1400 |
| A0A0D3W981_YEASX/86-350 | 9.0e-34 | Saccharomyces cerevisiae YJM683 |
| A0A0D3VMS6_YEASX/86-350 | 9.0e-34 | Saccharomyces cerevisiae YJM195 |
| E7LYS7_YEASV/86-350 | 9.0e-34 | Saccharomyces cerevisiae |
| E7KSR3_YEASL/86-350 | 9.0e-34 | Saccharomyces cerevisiae |
| A0A0D3W476_YEASX/86-350 | 9.0e-34 | Saccharomyces cerevisiae YJM541 |
| A0A0D3XMW5_YEASX/86-350 | 9.0e-34 | Saccharomyces cerevisiae YJM1386 |
| A0A0D3X0M2_YEASX/86-350 | 9.0e-34 | Saccharomyces cerevisiae YJM1248 |
| A0A0D3Y5K1_YEASX/86-350 | 9.0e-34 | Saccharomyces cerevisiae YJM1439 |
| A0A0D3X3S5_YEASX/86-350 | 9.0e-34 | Saccharomyces cerevisiae YJM1250 |
| A0A0D3XSD0_YEASX/86-350 | 9.0e-34 | Saccharomyces cerevisiae YJM1388 |
| A0A0D3YQP2_YEASX/86-350 | 9.0e-34 | Saccharomyces cerevisiae YJM1615 |
| A0A0D3X5I2_YEASX/86-350 | 9.0e-34 | Saccharomyces cerevisiae YJM1304 |
| A0A0D3WUD2_YEASX/86-350 | 9.0e-34 | Saccharomyces cerevisiae YJM1199 |
| A0A0D3X989_YEASX/86-350 | 9.0e-34 | Saccharomyces cerevisiae YJM1307 |
| A0A0D3X9A2_YEASX/86-350 | 9.0e-34 | Saccharomyces cerevisiae YJM1326 |
| A0A0D3WS58_YEASX/86-350 | 9.0e-34 | Saccharomyces cerevisiae YJM1133 |
| A0A0D3WT21_YEASX/86-350 | 9.0e-34 | Saccharomyces cerevisiae YJM1190 |
| A0A0D3Y938_YEASX/86-350 | 9.0e-34 | Saccharomyces cerevisiae YJM1443 |
| A0A0D3WVL0_YEASX/86-350 | 9.0e-34 | Saccharomyces cerevisiae YJM1202 |
| A0A0D3YJ93_YEASX/86-350 | 9.0e-34 | Saccharomyces cerevisiae YJM1527 |
| A0A0D3W0J2_YEASX/86-350 | 9.0e-34 | Saccharomyces cerevisiae YJM470 |
| A0A0D3Y456_YEASX/86-350 | 9.0e-34 | Saccharomyces cerevisiae YJM1419 |
| A0A0D3VZ36_YEASX/86-350 | 9.0e-34 | Saccharomyces cerevisiae YJM451 |
| A0A0D3YCE9_YEASX/86-350 | 9.0e-34 | Saccharomyces cerevisiae YJM1450 |
| A0A0D3YBM9_YEASX/86-350 | 9.0e-34 | Saccharomyces cerevisiae YJM1460 |
| A0A0C5J199_YEASX/86-350 | 9.0e-34 | Saccharomyces cerevisiae YJM1078 |
| A0A0D3Y0D1_YEASX/86-350 | 9.0e-34 | Saccharomyces cerevisiae YJM1418 |
| A0A0D3VXS6_YEASX/86-350 | 9.0e-34 | Saccharomyces cerevisiae YJM450 |
| A0A0D3XJK3_YEASX/86-350 | 9.0e-34 | Saccharomyces cerevisiae YJM1381 |
| A0A0D3YFA0_YEASX/86-350 | 9.2e-34 | Saccharomyces cerevisiae YJM1463 |
| S4RRL7_PETMA/2-140 | 1.1e-33 | Petromyzon marinus |
| J4CDW7_THEOR/103-410 | 1.7e-33 | Theileria orientalis |
| K7HDE8_CAEJA/314-427 | 1.1e-33 | Caenorhabditis japonica |
| A0A086T4C3_ACRCH/165-398 | 1.2e-33 | Acremonium chrysogenum ATCC 11550 |
| C3ZKN9_BRAFL/53-292 | 1.1e-33 | Branchiostoma floridae |
| H1VW68_COLHI/141-388 | 1.1e-33 | Colletotrichum higginsianum |
| A0A0D3XVJ6_YEASX/86-350 | 1.1e-33 | Saccharomyces cerevisiae YJM1401 |
| I1FIB2_AMPQE/140-263 | 1.2e-33 | Amphimedon queenslandica |
| S7MF81_MYOBR/63-263 | 1.4e-33 | Myotis brandtii |
| Q4SWZ8_TETNG/31-146 | 1.1e-33 | Tetraodon nigroviridis |
| C7GQ93_YEAS2/86-350 | 1.3e-33 | Saccharomyces cerevisiae |
| S3C658_OPHP1/200-433 | 1.5e-33 | Ophiostoma piceae |
| V5GFB5_PSEBG/95-336 | 2.8e-33 | Pseudozyma brasiliensis |
| A0A093YG47_9PEZI/179-414 | 1.6e-33 | Pseudogymnoascus pannorum VKM F-3808 |
| W9HW28_FUSOX/107-355 | 1.4e-33 | Fusarium oxysporum FOSC 3-a |
| W9HZV6_FUSOX/138-386 | 1.5e-33 | Fusarium oxysporum FOSC 3-a |
| A0A0D3XFQ8_YEASX/86-350 | 1.6e-33 | Saccharomyces cerevisiae YJM1342 |
| B4L863_DROMO/43-302 | 1.8e-33 | Drosophila mojavensis |
| B4NCM1_DROWI/63-317 | 2.2e-33 | Drosophila willistoni |
| J4VQY5_BEAB2/153-366 | 2.1e-33 | Beauveria bassiana |
| X0JIP6_FUSOX/108-355 | 1.8e-33 | Fusarium oxysporum f. sp. cubense |
| X0A2C7_FUSOX/8-246 | 1.9e-33 | Fusarium oxysporum f. sp. melonis 26406 |
| G7EA97_MIXOS/158-346 | 2.1e-33 | Mixia osmundae |
| X0JIP1_FUSOX/139-386 | 1.9e-33 | Fusarium oxysporum f. sp. cubense |
| A0A077ZNK0_STYLE/124-358 | 2.6e-33 | Stylonychia lemnae |
| W7MGX0_GIBM7/138-355 | 2.1e-33 | Gibberella moniliformis |
| A0A077ZW20_STYLE/98-355 | 2.4e-33 | Stylonychia lemnae |
| A0A094CXW1_9PEZI/176-414 | 2.7e-33 | Pseudogymnoascus pannorum VKM F-4516 (FW-969) |
| A0A0D3XMH8_YEASX/86-350 | 2.5e-33 | Saccharomyces cerevisiae YJM1383 |
| S0EC86_GIBF5/148-363 | 2.4e-33 | Gibberella fujikuroi |
| U7PYN8_SPOS1/225-442 | 3.2e-33 | Sporothrix schenckii |
| X0NDW8_FUSOX/107-355 | 2.6e-33 | Fusarium oxysporum f. sp. vasinfectum 25433 |
| A0A078AGL1_STYLE/98-354 | 2.9e-33 | Stylonychia lemnae |
| W9JSZ5_FUSOX/107-355 | 2.6e-33 | Fusarium oxysporum Fo47 |
| X0FMN0_FUSOX/107-355 | 2.6e-33 | Fusarium oxysporum f. sp. radicis-lycopersici 26381 |
| W9MKW4_FUSOX/107-355 | 2.6e-33 | Fusarium oxysporum f. sp. lycopersici MN25 |
| X0JMW4_FUSOX/107-355 | 2.6e-33 | Fusarium oxysporum f. sp. conglutinans race 2 54008 |
| W9P1T3_FUSOX/107-355 | 2.6e-33 | Fusarium oxysporum f. sp. pisi HDV247 |
| X0CTV4_FUSOX/107-355 | 2.6e-33 | Fusarium oxysporum f. sp. raphani 54005 |
| A0A0D3XVA0_YEASX/86-350 | 2.7e-33 | Saccharomyces cerevisiae YJM1399 |
| A0A0D3XEX6_YEASX/86-350 | 2.8e-33 | Saccharomyces cerevisiae YJM1338 |
| A0A0D3VZA7_YEASX/86-350 | 2.8e-33 | Saccharomyces cerevisiae YJM456 |
| X0MBU4_FUSOX/138-386 | 2.8e-33 | Fusarium oxysporum f. sp. vasinfectum 25433 |
| A0A0D3XAI2_YEASX/86-350 | 2.9e-33 | Saccharomyces cerevisiae YJM1311 |
| W9JX84_FUSOX/138-386 | 2.8e-33 | Fusarium oxysporum Fo47 |
| X0GGG3_FUSOX/138-386 | 2.8e-33 | Fusarium oxysporum f. sp. radicis-lycopersici 26381 |
| W9MRW7_FUSOX/138-386 | 2.8e-33 | Fusarium oxysporum f. sp. lycopersici MN25 |
| S9VTF5_9TRYP/206-453 | 1.5e-32 | Strigomonas culicis |
| X0IBM0_FUSOX/138-386 | 2.8e-33 | Fusarium oxysporum f. sp. conglutinans race 2 54008 |
| W9P7Y3_FUSOX/138-386 | 2.8e-33 | Fusarium oxysporum f. sp. pisi HDV247 |
| X0CTU9_FUSOX/138-386 | 2.8e-33 | Fusarium oxysporum f. sp. raphani 54005 |
| E7Q7W2_YEASB/86-350 | 2.9e-33 | Saccharomyces cerevisiae |
| F9GBL1_FUSOF/138-386 | 2.8e-33 | Fusarium oxysporum |
| A0A0D3YNL6_YEASX/86-350 | 2.9e-33 | Saccharomyces cerevisiae YJM1573 |
| A0A0D3XZ70_YEASX/86-350 | 2.9e-33 | Saccharomyces cerevisiae YJM1402 |
| A0A0D3X4J8_YEASX/86-350 | 2.9e-33 | Saccharomyces cerevisiae YJM1273 |
| A0A0D3Y473_YEASX/86-350 | 2.9e-33 | Saccharomyces cerevisiae YJM1434 |
| A0A0C2ISZ5_9PEZI/215-433 | 4.2e-33 | Sporothrix brasiliensis 5110 |
| Q22BU2_TETTS/293-491 | 4.2e-33 | Tetrahymena thermophila |
| N4USH9_FUSC1/124-374 | 3.1e-33 | Fusarium oxysporum f. sp. cubense |
| B4JJC7_DROGR/44-302 | 4.1e-33 | Drosophila grimshawi |
| A0A077ZX20_STYLE/41-282 | 6.3e-33 | Stylonychia lemnae |
| A0A016PWD1_GIBZA/146-385 | 3.5e-33 | Gibberella zeae |
| A0A094BDQ4_9PEZI/262-500 | 4.4e-33 | Pseudogymnoascus pannorum VKM F-4513 (FW-928) |
| L5M315_MYODS/47-248 | 3.8e-33 | Myotis davidii |
| G0P351_CAEBE/81-323 | 4.8e-33 | Caenorhabditis brenneri |
| A2FAE4_TRIVA/946-1149 | 5.6e-33 | Trichomonas vaginalis |
| A0A0D3YPD1_YEASX/86-350 | 4.4e-33 | Saccharomyces cerevisiae YJM1592 |
| A0A0D3XS25_YEASX/86-350 | 4.4e-33 | Saccharomyces cerevisiae YJM1389 |
| C0SEQ6_PARBP/178-417 | 1.4e-32 | Paracoccidioides brasiliensis |
| A9V2B3_MONBE/93-316 | 4.5e-24 | Monosiga brevicollis |
| E3MBK8_CAERE/75-307 | 1.0e-32 | Caenorhabditis remanei |
| E3MBL4_CAERE/46-273 | 1.2e-32 | Caenorhabditis remanei |
| H6L010_SAPGL/295-494 | 6.7e-33 | Saprospira grandis |
| W9I0M0_FUSOX/8-246 | 5.6e-33 | Fusarium oxysporum FOSC 3-a |
| X0JX81_FUSOX/8-246 | 5.7e-33 | Fusarium oxysporum f. sp. cubense |
| A0A091RWD7_NESNO/230-395 | 6.0e-33 | Nestor notabilis |
| E3QQW2_COLGM/154-389 | 5.6e-33 | Colletotrichum graminicola |
| R7VUB4_COLLI/231-396 | 6.1e-33 | Columba livia |
| A0A093P4L8_PYGAD/191-356 | 5.6e-33 | Pygoscelis adeliae |
| H0ZVM1_TAEGU/231-396 | 6.8e-33 | Taeniopygia guttata |
| A0A091TF92_PHALP/230-395 | 6.3e-33 | Phaethon lepturus |
| A0A091MJB9_9PASS/231-396 | 6.8e-33 | Acanthisitta chloris |
| A0A091JBM9_9AVES/231-396 | 6.8e-33 | Egretta garzetta |
| A0A091PBH3_LEPDC/227-392 | 6.6e-33 | Leptosomus discolor |
| A0A091JYC1_COLST/229-394 | 6.7e-33 | Colius striatus |
| A0A091ERW9_CORBR/231-396 | 6.8e-33 | Corvus brachyrhynchos |
| A0A091WDC6_OPIHO/230-395 | 6.8e-33 | Opisthocomus hoazin |
| A0A091RFX8_9GRUI/231-396 | 6.8e-33 | Mesitornis unicolor |
| A0A091ISA7_CALAN/227-392 | 6.6e-33 | Calypte anna |
| A0A093HLL7_GAVST/231-396 | 6.8e-33 | Gavia stellata |
| A0A091U1G6_PHORB/231-396 | 6.8e-33 | Phoenicopterus ruber ruber |
| A0A093CGM3_TAUER/228-393 | 6.7e-33 | Tauraco erythrolophus |
| A0A093PV80_9PASS/231-396 | 6.8e-33 | Manacus vitellinus |
| A0A091VMK4_NIPNI/231-396 | 6.8e-33 | Nipponia nippon |
| A0A091TLS0_9AVES/231-396 | 6.8e-33 | Pelecanus crispus |
| A0A091LS20_CARIC/227-392 | 6.6e-33 | Cariama cristata |
| A0A091P976_HALAL/231-396 | 6.8e-33 | Haliaeetus albicilla |
| A0A091KHL2_9GRUI/227-392 | 6.6e-33 | Chlamydotis macqueenii |
| A0A093F6Z9_TYTAL/231-396 | 6.8e-33 | Tyto alba |
| A0A0A0A2I6_CHAVO/231-396 | 6.8e-33 | Charadrius vociferus |
| A0A093H4Y1_PICPB/231-396 | 6.8e-33 | Picoides pubescens |
| A0A091QSS3_MERNU/232-397 | 6.9e-33 | Merops nubicus |
| A0A0D3YFG8_YEASX/86-350 | 7.1e-33 | Saccharomyces cerevisiae YJM1478 |
| A0A087RAC3_APTFO/231-396 | 7.2e-33 | Aptenodytes forsteri |
| E3N2V1_CAERE/46-253 | 8.0e-33 | Caenorhabditis remanei |
| A0A0D2C0N6_9EURO/178-422 | 1.0e-32 | Exophiala spinifera |
| A0A063C9J0_9HYPO/154-364 | 1.0e-32 | Ustilaginoidea virens |
| A0A099ZMF3_TINGU/229-394 | 9.5e-33 | Tinamus guttatus |
| A1CRW2_ASPCL/208-404 | 9.8e-33 | Aspergillus clavatus |
| X0MBC6_FUSOX/8-246 | 1.0e-32 | Fusarium oxysporum f. sp. vasinfectum 25433 |
| W9JSW3_FUSOX/8-246 | 1.0e-32 | Fusarium oxysporum Fo47 |
| X0FMN5_FUSOX/8-246 | 1.0e-32 | Fusarium oxysporum f. sp. radicis-lycopersici 26381 |
| W9MZ04_FUSOX/8-246 | 1.0e-32 | Fusarium oxysporum f. sp. lycopersici MN25 |
| U3JZQ5_FICAL/2-69 | 2.1e-09 | Ficedula albicollis |
| U3JZQ5_FICAL/215-292 | 1.4e-17 | Ficedula albicollis |
| W9P1T8_FUSOX/8-246 | 1.0e-32 | Fusarium oxysporum f. sp. pisi HDV247 |
| X0JMW8_FUSOX/8-246 | 1.0e-32 | Fusarium oxysporum f. sp. conglutinans race 2 54008 |
| X0CJZ3_FUSOX/8-246 | 1.0e-32 | Fusarium oxysporum f. sp. raphani 54005 |
| A0A091GC97_9AVES/227-392 | 1.0e-32 | Cuculus canorus |
| I7MHC5_TETTS/684-909 | 1.3e-32 | Tetrahymena thermophila |
| F9WCD2_TRYCI/213-437 | 1.2e-32 | Trypanosoma congolense |
| A0A093R046_PHACA/231-396 | 1.2e-32 | Phalacrocorax carbo |
| A8WYG0_CAEBR/87-293 | 1.2e-32 | Caenorhabditis briggsae |
| K7HDE9_CAEJA/314-425 | 1.2e-32 | Caenorhabditis japonica |
| A0A0F2MDM7_SPOSC/225-442 | 1.6e-32 | Sporothrix schenckii 1099-18 |
| A0A066X4T6_COLSU/171-387 | 1.5e-32 | Colletotrichum sublineola |
| F0WU21_9STRA/86-399 | 1.4e-31 | Albugo laibachii Nc14 |
| G0TR86_TRYVY/247-501 | 1.4e-32 | Trypanosoma vivax |
| A0A084APP0_STACH/157-376 | 1.7e-32 | Stachybotrys chartarum IBT 7711 |
| A0A084PRF7_STACH/157-376 | 1.7e-32 | Stachybotrys chartarum IBT 40293 |
| A0A084RGE8_STACH/214-433 | 1.9e-32 | Stachybotrys chartarum IBT 40288 |
| A0A0C2FQX7_9BILA/7-114 | 1.5e-32 | Ancylostoma duodenale |
| W3XBJ2_9PEZI/162-385 | 2.3e-32 | Pestalotiopsis fici W106-1 |
| N4V4D6_COLOR/178-398 | 3.4e-32 | Colletotrichum orbiculare |
| A0A016WBP1_9BILA/54-170 | 1.9e-32 | Ancylostoma ceylanicum |
| E0UGX6_CYAP2/1305-1509 | 5.2e-32 | Cyanothece sp. |
| A0A0C2H461_9BILA/50-166 | 2.2e-32 | Ancylostoma duodenale |
| A0A091MT66_9PASS/3-190 | 1.8e-30 | Acanthisitta chloris |
| W6KTP1_9TRYP/208-480 | 1.1e-31 | Phytomonas sp. isolate EM1 |
| A0A016WA47_9BILA/75-191 | 2.7e-32 | Ancylostoma ceylanicum |
| A2E4X2_TRIVA/74-301 | 6.5e-32 | Trichomonas vaginalis |
| CANC_DROME/67-313 | 3.8e-32 | Drosophila melanogaster |
| A0A016WCI1_9BILA/54-174 | 2.9e-32 | Ancylostoma ceylanicum |
| A0A087VPE3_BALRE/236-401 | 4.0e-32 | Balearica regulorum gibbericeps |
| A0A093I221_STRCA/227-392 | 4.0e-32 | Struthio camelus australis |
| F6VSF2_HORSE/85-197 | 3.4e-32 | Equus caballus |
| B4IF60_DROSE/65-312 | 4.7e-32 | Drosophila sechellia |
| L2FNS7_COLGN/170-417 | 4.4e-32 | Colletotrichum gloeosporioides |
| A0A078AZL5_STYLE/105-358 | 4.3e-32 | Stylonychia lemnae |
| A0A016WAP9_9BILA/75-195 | 3.9e-32 | Ancylostoma ceylanicum |
| B3NX55_DROER/66-312 | 7.0e-32 | Drosophila erecta |
| A0A084QND0_9HYPO/157-377 | 6.7e-32 | Stachybotrys chlorohalonata IBT 40285 |
| R1DIU9_EMIHU/118-255 | 6.5e-32 | Emiliania huxleyi CCMP1516 |
| T0L1F7_COLGC/158-406 | 7.5e-32 | Colletotrichum gloeosporioides |
| W2HVM1_PHYPR/97-418 | 2.5e-31 | Phytophthora parasitica |
| W2M5K4_PHYPR/97-418 | 2.5e-31 | Phytophthora parasitica |
| V9DXJ0_PHYPR/97-418 | 2.5e-31 | Phytophthora parasitica P1569 |
| W2VV20_PHYPR/97-418 | 2.5e-31 | Phytophthora parasitica CJ01A1 |
| W2FNV6_PHYPR/97-418 | 2.5e-31 | Phytophthora parasitica |
| W2QSK6_PHYPN/97-418 | 2.5e-31 | Phytophthora parasitica |
| A0A080Z0B2_PHYPR/97-418 | 2.5e-31 | Phytophthora parasitica P1976 |
| W2K1S9_PHYPR/97-418 | 2.5e-31 | Phytophthora parasitica |
| W2Y582_PHYPR/97-418 | 2.5e-31 | Phytophthora parasitica P10297 |
| A0A077R2J7_9BASI/206-437 | 1.1e-31 | Melanopsichium pennsylvanicum 4 |
| T1FV80_HELRO/44-335 | 4.5e-31 | Helobdella robusta |
| G9NEE6_HYPAI/123-324 | 8.3e-32 | Hypocrea atroviridis |
| A0A0A2V2N2_PARBA/130-369 | 2.5e-31 | Paracoccidioides lutzii |
| U6D6G6_NEOVI/3-161 | 1.1e-31 | Neovison vison |
| E9BES4_LEIDB/544-807 | 1.9e-31 | Leishmania donovani |
| A0A068XGW5_HYMMI/780-946 | 1.2e-31 | Hymenolepis microstoma |
| G0TX01_TRYVY/339-579 | 1.6e-30 | Trypanosoma vivax |
| E9PRM1_HUMAN/85-197 | 8.8e-32 | Homo sapiens |
| A4HYW4_LEIIN/439-701 | 3.3e-31 | Leishmania infantum |
| B4PXR7_DROYA/68-312 | 1.1e-31 | Drosophila yakuba |
| S9W854_9TRYP/2-168 | 9.0e-32 | Angomonas deanei |
| D0ND88_PHYIT/76-395 | 3.5e-31 | Phytophthora infestans |
| A0A067CCC1_SAPPC/483-731 | 1.1e-30 | Saprolegnia parasitica CBS 223.65 |
| A0A078A0X8_STYLE/103-359 | 1.0e-31 | Stylonychia lemnae |
| G0UB02_TRYVY/1-232 | 1.1e-31 | Trypanosoma vivax |
| S4R839_PETMA/1-128 | 8.4e-32 | Petromyzon marinus |
| G0UYZ9_TRYCI/293-568 | 4.1e-31 | Trypanosoma congolense |
| A4HJ22_LEIBR/456-702 | 1.4e-31 | Leishmania braziliensis |
| T0Q289_9STRA/498-746 | 1.8e-30 | Saprolegnia diclina VS20 |
| A0A0D2DY08_9EURO/169-422 | 1.5e-31 | Exophiala oligosperma |
| G1XGM8_ARTOA/214-445 | 1.6e-31 | Arthrobotrys oligospora |
| F7C4C1_ORNAN/20-286 | 1.4e-31 | Ornithorhynchus anatinus |
| G0U9Y3_TRYVY/49-273 | 2.6e-27 | Trypanosoma vivax |
| X8JHG9_9HOMO/176-384 | 1.7e-31 | Rhizoctonia solani AG-3 Rhs1AP |
| A0A074RRM4_9HOMO/176-384 | 1.7e-31 | Rhizoctonia solani 123E |
| A0A0A1SLZ8_9HYPO/152-361 | 2.0e-31 | Torrubiella hemipterigena |
| A0A078AEE6_STYLE/69-236 | 2.7e-30 | Stylonychia lemnae |
| F4NTM9_BATDJ/42-312 | 2.1e-31 | Batrachochytrium dendrobatidis |
| F9W3F0_TRYCI/293-568 | 1.4e-30 | Trypanosoma congolense |
| R7VK39_CAPTE/29-254 | 2.8e-31 | Capitella teleta |
| K3WI10_PYTUL/7-165 | 6.1e-31 | Pythium ultimum DAOM BR144 |
| G6CXD5_DANPL/42-245 | 2.5e-31 | Danaus plexippus |
| G3MK58_9ACAR/3-78 | 9.4e-14 | Amblyomma maculatum |
| G3MK58_9ACAR/83-133 | 2.6e-13 | Amblyomma maculatum |
| A0A0D1C7R4_USTMA/200-445 | 5.1e-31 | Ustilago maydis |
| S4P701_9NEOP/1-128 | 2.6e-31 | Pararge aegeria |
| A0A0D9Q1E8_METAN/164-370 | 4.0e-31 | Metarhizium anisopliae BRIP 53284 |
| A0A0D9NNA8_METAN/164-370 | 4.0e-31 | Metarhizium anisopliae BRIP 53293 |
| A0A095AHJ3_SCHHA/54-352 | 4.6e-31 | Schistosoma haematobium |
| H3ALS7_LATCH/261-525 | 5.0e-31 | Latimeria chalumnae |
| A0A093C6D4_9AVES/1-140 | 4.2e-31 | Pterocles gutturalis |
| E8NHQ2_LEIMU/2-240 | 8.9e-31 | Leishmania mexicana |
| A0A088RXG6_9TRYP/458-704 | 5.8e-31 | Leishmania panamensis |
| A0A0B4I574_9HYPO/164-370 | 5.5e-31 | Metarhizium majus ARSEF 297 |
| A0A0B4HGY1_9HYPO/164-370 | 6.2e-31 | Metarhizium guizhouense ARSEF 977 |
| A0A0A1V099_9HYPO/125-331 | 6.2e-31 | Metarhizium robertsii |
| M1EH24_MUSPF/12-198 | 5.2e-31 | Mustela putorius furo |
| A0A0B4FPU5_METAN/164-370 | 6.7e-31 | Metarhizium anisopliae ARSEF 549 |
| E9EUD4_METRA/164-370 | 6.7e-31 | Metarhizium robertsii |
| A0A067QZR6_ZOONE/1-128 | 6.0e-31 | Zootermopsis nevadensis |
| Q17E40_AEDAE/60-257 | 5.6e-31 | Aedes aegypti |
| A0A0C2BYX6_9BILA/2-122 | 4.9e-31 | Ancylostoma duodenale |
| G3UET3_LOXAF/85-123 | 1.0e-05 | Loxodonta africana |
| G3UET3_LOXAF/222-323 | 1.2e-18 | Loxodonta africana |
| B4R5S7_DROSI/65-302 | 9.7e-31 | Drosophila simulans |
| G4V693_SCHMA/54-358 | 5.3e-30 | Schistosoma mansoni |
| A0A060XPP0_ONCMY/270-435 | 1.4e-30 | Oncorhynchus mykiss |
| K7WKF4_9NOST/531-741 | 2.1e-30 | Anabaena sp. 90 |
| J9ENM3_WUCBA/51-169 | 1.4e-30 | Wuchereria bancrofti |
| A0A0B4FS33_9HYPO/164-370 | 2.0e-30 | Metarhizium brunneum ARSEF 3297 |
| I3NCA7_SPETR/37-186 | 1.4e-30 | Spermophilus tridecemlineatus |
| G5A1P8_PHYSP/79-404 | 5.0e-30 | Phytophthora sojae |
| H3GS02_PHYRM/76-401 | 6.3e-30 | Phytophthora ramorum |
| F0ULF4_AJEC8/187-419 | 2.3e-30 | Ajellomyces capsulatus |
| Q4U920_THEAN/74-353 | 3.0e-30 | Theileria annulata |
| F6QFR4_HORSE/75-187 | 1.9e-30 | Equus caballus |
| A0A0B2X5Z6_9HYPO/163-372 | 2.6e-30 | Metarhizium album ARSEF 1941 |
| W9Z9N1_FUSOX/31-264 | 2.3e-30 | Fusarium oxysporum f. sp. melonis 26406 |
| X0BPQ5_FUSOX/31-264 | 2.3e-30 | Fusarium oxysporum f. sp. raphani 54005 |
| C6HAF4_AJECH/187-419 | 2.8e-30 | Ajellomyces capsulatus |
| C0NC49_AJECG/187-419 | 2.9e-30 | Ajellomyces capsulatus |
| R1CUW7_EMIHU/62-226 | 3.0e-30 | Emiliania huxleyi CCMP1516 |
| A0A0A0CXU1_PHOLU/117-348 | 1.2e-29 | Photorhabdus luminescens |
| E8Z737_KARVE/158-436 | 6.8e-30 | Karlodinium veneficum |
| S9U2N2_9TRYP/81-310 | 8.4e-30 | Strigomonas culicis |
| U6NVC2_HAECO/12-165 | 3.5e-30 | Haemonchus contortus |
| H2NPK1_PONAB/667-725 | 3.3e-09 | Pongo abelii |
| H2NPK1_PONAB/727-811 | 3.0e-15 | Pongo abelii |
| E0UGY5_CYAP2/320-519 | 6.4e-30 | Cyanothece sp. |
| L1IPC0_GUITH/171-364 | 1.5e-24 | Guillardia theta CCMP2712 |
| V9IEX8_APICE/2-201 | 4.3e-30 | Apis cerana |
| Q4N1R5_THEPA/75-353 | 6.7e-30 | Theileria parva |
| D8LU43_ECTSI/308-505 | 7.2e-30 | Ectocarpus siliculosus |
| A0A093EXT5_GAVST/1-124 | 4.6e-30 | Gavia stellata |
| M2ZS76_PSEFD/293-436 | 7.1e-23 | Pseudocercospora fijiensis |
| A0A0A2V5L4_BEABA/139-307 | 7.5e-30 | Beauveria bassiana D1-5 |
| I4YBK3_WALSC/140-349 | 1.6e-29 | Wallemia sebi |
| S9URF8_9TRYP/253-482 | 1.5e-29 | Strigomonas culicis |
| S9V110_9TRYP/253-482 | 1.6e-29 | Strigomonas culicis |
| S8BTV6_DACHA/195-422 | 1.2e-29 | Dactylellina haptotyla |
| W4FY06_9STRA/485-747 | 1.2e-29 | Aphanomyces astaci |
| A0A022TV29_TRIRU/15-204 | 9.1e-30 | Trichophyton rubrum MR850 |
| A0A023ALN6_TRIRU/15-204 | 9.1e-30 | Trichophyton rubrum CBS 202.88 |
| A0A022W9V3_TRIRU/15-204 | 9.1e-30 | Trichophyton rubrum CBS 288.86 |
| A0A022X4X6_TRIRU/15-204 | 9.1e-30 | Trichophyton rubrum CBS 289.86 |
| A0A022YW23_TRIRU/15-204 | 9.1e-30 | Trichophyton rubrum MR1448 |
| A0A059K8H8_TRIRU/15-204 | 9.1e-30 | Trichophyton rubrum D6 |
| A0A022Y0W8_TRISD/15-204 | 9.1e-30 | Trichophyton soudanense CBS 452.61 |
| F2SUN0_TRIRC/15-204 | 9.1e-30 | Trichophyton rubrum |
| A0A022ZQR8_TRIRU/15-204 | 9.1e-30 | Trichophyton rubrum MR1459 |
| A0A022VD93_TRIRU/15-204 | 9.1e-30 | Trichophyton rubrum CBS 100081 |
| A0A028JWE1_TRIRU/15-204 | 9.1e-30 | Trichophyton rubrum CBS 735.88 |
| R9NX25_PSEHS/195-437 | 2.5e-29 | Pseudozyma hubeiensis |
| W4FYC5_9STRA/485-747 | 1.6e-29 | Aphanomyces astaci |
| S9W0N8_9TRYP/10-184 | 1.9e-29 | Angomonas deanei |
| M1EH31_MUSPF/2-117 | 1.5e-29 | Mustela putorius furo |
| V9KHJ0_CALMI/1-124 | 2.2e-29 | Callorhinchus milii |
| K4DSS3_TRYCR/51-277 | 2.0e-29 | Trypanosoma cruzi |
| N1S7J7_FUSC4/76-226 | 3.0e-29 | Fusarium oxysporum f. sp. cubense |
| X1XCE6_ACYPI/2-126 | 2.1e-29 | Acyrthosiphon pisum |
| D2VGR9_NAEGR/381-582 | 2.8e-29 | Naegleria gruberi |
| K2MU52_TRYCR/50-277 | 3.0e-29 | Trypanosoma cruzi marinkellei |
| V5BE45_TRYCR/30-256 | 3.1e-29 | Trypanosoma cruzi Dm28c |
| E3NM44_CAERE/101-317 | 7.3e-29 | Caenorhabditis remanei |
| Q16WJ3_AEDAE/26-219 | 9.3e-29 | Aedes aegypti |
| G0N111_CAEBE/96-325 | 3.8e-29 | Caenorhabditis brenneri |
| X0EK22_FUSOX/13-194 | 2.8e-29 | Fusarium oxysporum f. sp. radicis-lycopersici 26381 |
| Q4Q6M0_LEIMA/445-695 | 4.0e-29 | Leishmania major |
| F9WFU6_TRYCI/501-720 | 6.1e-26 | Trypanosoma congolense |
| W3VDD9_9BASI/158-380 | 6.3e-29 | Pseudozyma aphidis DSM 70725 |
| A4HFH7_LEIBR/50-276 | 5.8e-29 | Leishmania braziliensis |
| A0A024U7D8_9STRA/501-754 | 8.9e-29 | Aphanomyces invadans |
| I7M6I1_TETTS/208-451 | 8.5e-29 | Tetrahymena thermophila |
| Q4TD92_TETNG/46-157 | 4.9e-29 | Tetraodon nigroviridis |
| K0EQG1_9NOCA/5892-6004 | 1.8e-07 | Nocardia brasiliensis ATCC 700358 |
| N1S725_FUSC4/24-220 | 7.7e-29 | Fusarium oxysporum f. sp. cubense |
| D2UYF2_NAEGR/1216-1477 | 1.2e-27 | Naegleria gruberi |
| K2N576_TRYCR/50-277 | 9.0e-29 | Trypanosoma cruzi marinkellei |
| A0A091SK86_9AVES/2-131 | 6.3e-29 | Pelecanus crispus |
| L8Y4B7_TUPCH/85-227 | 1.1e-28 | Tupaia chinensis |
| A0A0C3KWH5_PISTI/188-369 | 1.1e-28 | Pisolithus tinctorius Marx 270 |
| A0A061ISC0_TRYRA/50-275 | 1.5e-28 | Trypanosoma rangeli SC58 |
| N4UYV3_FUSC1/76-222 | 1.4e-28 | Fusarium oxysporum f. sp. cubense |
| K1REZ8_CRAGI/28-267 | 1.6e-28 | Crassostrea gigas |
| R1CLM0_EMIHU/7-155 | 2.0e-28 | Emiliania huxleyi CCMP1516 |
| A8PR76_MALGO/137-340 | 1.6e-28 | Malassezia globosa |
| A0A0A1NP63_9FUNG/11-137 | 1.2e-28 | Rhizopus microsporus |
| E3NL90_CAERE/300-440 | 1.6e-28 | Caenorhabditis remanei |
| C7ZBJ4_NECH7/199-370 | 2.0e-28 | Nectria haematococca |
| A8WMS0_CAEBR/484-708 | 2.4e-28 | Caenorhabditis briggsae |
| A0A087YL75_POEFO/112-285 | 2.7e-28 | Poecilia formosa |
| M9MD10_PSEA3/158-365 | 3.4e-28 | Pseudozyma antarctica |
| L1JSP6_GUITH/34-98 | 1.8e-05 | Guillardia theta CCMP2712 |
| L1JSP6_GUITH/95-198 | 1.6e-17 | Guillardia theta CCMP2712 |
| D5MF42_9BACT/131-366 | 3.0e-28 | Candidatus Methylomirabilis oxyfera |
| R1F078_EMIHU/294-487 | 2.8e-28 | Emiliania huxleyi CCMP1516 |
| U6NTN6_HAECO/19-145 | 2.9e-28 | Haemonchus contortus |
| A0A081CFE8_CANAR/158-363 | 3.8e-28 | Candida antarctica |
| E3MBL3_CAERE/77-270 | 9.6e-28 | Caenorhabditis remanei |
| A0A066W7T1_9BASI/120-356 | 5.1e-28 | Tilletiaria anomala UBC 951 |
| B7Q1K6_IXOSC/20-160 | 3.9e-28 | Ixodes scapularis |
| D8LKI3_ECTSI/144-275 | 7.0e-22 | Ectocarpus siliculosus |
| Q7N497_PHOLL/116-348 | 1.5e-27 | Photorhabdus luminescens subsp. laumondii |
| K7EGF7_ORNAN/2-97 | 4.1e-28 | Ornithorhynchus anatinus |
| Q4CMV9_TRYCC/51-276 | 6.7e-28 | Trypanosoma cruzi |
| Q4DB11_TRYCC/356-418 | 5.1e-09 | Trypanosoma cruzi |
| Q4DB11_TRYCC/471-593 | 1.2e-12 | Trypanosoma cruzi |
| A0A0C3C834_HEBCY/185-415 | 8.3e-28 | Hebeloma cylindrosporum h7 |
| Q7Q053_ANOGA/7-206 | 7.7e-28 | Anopheles gambiae |
| A0A0A0MV80_PAPAN/186-323 | 1.0e-27 | Papio anubis |
| Q584X4_TRYB2/339-410 | 3.0e-12 | Trypanosoma brucei brucei |
| Q584X4_TRYB2/454-574 | 9.8e-10 | Trypanosoma brucei brucei |
| C9ZR02_TRYB9/339-410 | 3.0e-12 | Trypanosoma brucei gambiense |
| C9ZR02_TRYB9/454-574 | 9.8e-10 | Trypanosoma brucei gambiense |
| K2P9S2_TRYCR/355-418 | 6.5e-09 | Trypanosoma cruzi marinkellei |
| K2P9S2_TRYCR/466-593 | 9.7e-13 | Trypanosoma cruzi marinkellei |
| A0A023EW44_AEDAL/84-278 | 8.7e-28 | Aedes albopictus |
| Q4DCK2_TRYCC/356-418 | 8.0e-09 | Trypanosoma cruzi |
| Q4DCK2_TRYCC/471-593 | 8.6e-13 | Trypanosoma cruzi |
| A0A0C9U840_PAXIN/141-331 | 1.7e-27 | Paxillus involutus ATCC 200175 |
| A0A068XGV4_HYMMI/867-1042 | 1.2e-21 | Hymenolepis microstoma |
| A0A024GDW9_9STRA/61-365 | 3.9e-27 | Albugo candida |
| A0A091U3H2_PHORB/1-103 | 9.0e-28 | Phoenicopterus ruber ruber |
| W5K5I1_ASTMX/22-140 | 1.1e-27 | Astyanax mexicanus |
| S9WLC0_9CETA/117-251 | 1.6e-27 | Camelus ferus |
| G6CW66_DANPL/103-364 | 1.6e-27 | Danaus plexippus |
| W6KXT8_9TRYP/209-480 | 9.6e-27 | Phytomonas sp. isolate Hart1 |
| R1EAI1_EMIHU/294-487 | 1.8e-27 | Emiliania huxleyi CCMP1516 |
| E3J3F6_FRASU/440-642 | 2.2e-27 | Frankia sp. EuI1c |
| F4RUY7_MELLP/133-362 | 2.1e-27 | Melampsora larici-populina |
| A0A088S8S5_9TRYP/1-142 | 6.9e-17 | Leishmania panamensis |
| A0A088S8S5_9TRYP/181-248 | 0.0001 | Leishmania panamensis |
| E9AUR1_LEIMU/1-249 | 4.1e-27 | Leishmania mexicana |
| S9UTA3_9TRYP/241-482 | 3.1e-27 | Angomonas deanei |
| Q05QW4_9SYNE/491-710 | 4.8e-27 | Synechococcus sp. RS9916 |
| W7M774_GIBM7/138-345 | 5.0e-27 | Gibberella moniliformis |
| V5B8W5_TRYCR/356-418 | 7.9e-09 | Trypanosoma cruzi Dm28c |
| V5B8W5_TRYCR/471-593 | 6.3e-12 | Trypanosoma cruzi Dm28c |
| A0A0B6Y3C6_9EUPU/5-141 | 4.7e-27 | Arion vulgaris |
| F0XY34_AURAN/11-126 | 4.4e-27 | Aureococcus anophagefferens |
| B3N0T2_DROAN/44-200 | 5.1e-27 | Drosophila ananassae |
| Q0PXR0_PIG/1-117 | 5.0e-27 | Sus scrofa |
| A0A091GNU2_BUCRH/1-103 | 5.3e-27 | Buceros rhinoceros silvestris |
| K4E680_TRYCR/356-418 | 7.9e-09 | Trypanosoma cruzi |
| K4E680_TRYCR/471-593 | 9.3e-12 | Trypanosoma cruzi |
| A0A016WA52_9BILA/34-153 | 7.6e-27 | Ancylostoma ceylanicum |
| E9AIH6_LEIBR/1-243 | 1.0e-25 | Leishmania braziliensis |
| V9KQR4_CALMI/3-128 | 7.7e-27 | Callorhinchus milii |
| A0A091TVM8_PHALP/1-103 | 6.1e-27 | Phaethon lepturus |
| A0A061J112_TRYRA/353-415 | 1.1e-10 | Trypanosoma rangeli SC58 |
| A0A061J112_TRYRA/468-575 | 4.1e-10 | Trypanosoma rangeli SC58 |
| E9PQB3_HUMAN/85-191 | 7.9e-27 | Homo sapiens |
| A0A087TUF3_9ARAC/312-415 | 1.1e-26 | Stegodyphus mimosarum |
| C1E8N2_MICSR/46-153 | 8.4e-27 | Micromonas sp. |
| U4PMH7_CAEEL/2-101 | 1.3e-26 | Caenorhabditis elegans |
| Q236J8_TETTS/88-284 | 2.5e-26 | Tetrahymena thermophila |
| C6K3U8_9TRYP/503-752 | 1.3e-26 | Crithidia sp. ATCC 30255 |
| A0DEK8_PARTE/35-211 | 3.1e-26 | Paramecium tetraurelia |
| A0A0C2D995_9BILA/17-135 | 1.5e-26 | Ancylostoma duodenale |
| A0A0C3NGZ8_PHLGI/153-357 | 2.0e-26 | Phlebiopsis gigantea 11061_1 CR5-6 |
| S9TK21_9TRYP/226-370 | 1.8e-26 | Strigomonas culicis |
| G1MAM9_AILME/106-184 | 4.9e-19 | Ailuropoda melanoleuca |
| W7M5L3_GIBM7/8-205 | 1.8e-26 | Gibberella moniliformis) |
| G3TGT3_LOXAF/1-182 | 2.2e-26 | Loxodonta africana |
| F0Y5M7_AURAN/34-173 | 1.7e-26 | Aureococcus anophagefferens |
| D8TN36_VOLCA/60-165 | 1.7e-26 | Volvox carteri |
| A0A084G6J9_9PEZI/145-337 | 2.7e-26 | Scedosporium apiospermum |
| Q22UD8_TETTS/167-389 | 6.7e-26 | Tetrahymena thermophila |
| A6R587_AJECN/9-188 | 3.2e-26 | Ajellomyces capsulatus |
| A8HZK8_CHLRE/20-176 | 8.1e-26 | Chlamydomonas reinhardtii |
| F0YB06_AURAN/10-122 | 3.5e-26 | Aureococcus anophagefferens |
| U6M034_EIMMA/758-866 | 1.1e-18 | Eimeria maxima |
| W6LCX3_9TRYP/3-119 | 1.8e-06 | Phytomonas sp. isolate Hart1 |
| W6LCX3_9TRYP/3300-3474 | 3.3e-13 | Phytomonas sp. isolate Hart1 |
| X5DJY4_9BACT/480-686 | 6.3e-26 | Draconibacterium orientale |
| S9V0G9_9TRYP/487-704 | 2.3e-25 | Angomonas deanei |
| S9VRV2_9TRYP/270-515 | 6.5e-26 | Angomonas deanei |
| A0A0C3MI31_9HOMO/177-383 | 7.0e-26 | Tulasnella calospora MUT 4182 |
| S9VJ42_9TRYP/44-251 | 5.5e-26 | Angomonas deanei |
| A0A060ZED8_ONCMY/29-141 | 5.1e-26 | Oncorhynchus mykiss |
| A0A084WHZ5_9DIPT/3-197 | 1.1e-25 | Anopheles sinensis |
| A0A0A9XV97_LYGHE/53-145 | 1.6e-07 | Lygus hesperus |
| A0A0A9XV97_LYGHE/368-523 | 1.7e-12 | Lygus hesperus |
| A0A087W120_ECHMU/51-366 | 5.7e-24 | Echinococcus multilocularis |
| S9WPK3_9TRYP/270-515 | 9.5e-26 | Angomonas deanei |
| A0A084YG06_9CYAN/352-551 | 1.0e-25 | Calothrix sp. 336/3 |
| Q6QDT9_BOVIN/1-100 | 5.9e-26 | Bos taurus |
| W6V4M4_ECHGR/51-366 | 7.1e-24 | Echinococcus granulosus |
| A0A068WQF2_ECHGR/51-366 | 7.1e-24 | Echinococcus granulosus |
| K7GM94_PIG/31-149 | 6.6e-26 | Sus scrofa |
| Q4RQ29_TETNG/75-145 | 1.5e-10 | Tetraodon nigroviridis |
| Q4RQ29_TETNG/147-238 | 1.0e-09 | Tetraodon nigroviridis |
| S9W5T6_9TRYP/386-622 | 1.2e-25 | Strigomonas culicis |
| A0A0D2PT84_9AGAR/160-367 | 1.5e-25 | Hypholoma sublateritium FD-334 SS-4 |
| A4I6F0_LEIIN/447-696 | 1.2e-25 | Leishmania infantum |
| H2KSB0_CLOSI/72-239 | 6.6e-20 | Clonorchis sinensis |
| S9VD46_9TRYP/4-165 | 1.3e-25 | Strigomonas culicis |
| S9VVM8_9TRYP/392-626 | 1.8e-25 | Angomonas deanei |
| S9WN98_9TRYP/392-626 | 1.8e-25 | Angomonas deanei |
| A0A0A9WPX4_LYGHE/48-213 | 1.8e-25 | Lygus hesperus |
| B0XDG9_CULQU/6-191 | 4.2e-25 | Culex quinquefasciatus |
| C5K406_AJEDS/12-152 | 1.5e-25 | Ajellomyces dermatitidis |
| F2QVU6_PICP7/128-317 | 1.8e-25 | Komagataella pastoris |
| A4HFX3_LEIBR/376-595 | 2.0e-25 | Leishmania braziliensis |
| S9TDP0_9TRYP/228-468 | 1.9e-25 | Strigomonas culicis |
| A0A023ETY8_AEDAL/2-192 | 1.8e-25 | Aedes albopictus |
| A0A081S1G4_PHOTE/114-346 | 8.6e-25 | Photorhabdus temperata subsp. temperata Meg1 |
| T0QHA9_PHOTE/114-346 | 8.6e-25 | Photorhabdus temperata subsp. temperata M1021 |
| U7QW08_PHOTE/114-346 | 8.6e-25 | Photorhabdus temperata J3 |
| E2LEI0_MONPE/2-121 | 2.0e-25 | Moniliophthora perniciosa |
| F8NRR1_SERL9/142-354 | 2.7e-25 | Serpula lacrymans var. lacrymans |
| F8PSK4_SERL3/142-354 | 2.7e-25 | Serpula lacrymans var. lacrymans |
| S9U5J1_9TRYP/4-174 | 2.1e-25 | Strigomonas culicis |
| C4R548_PICPG/128-317 | 2.9e-25 | Komagataella pastoris |
| E9BMG8_LEIDB/447-696 | 2.6e-25 | Leishmania donovani |
| A0A0D0AXQ4_9HOMO/145-322 | 3.2e-25 | Suillus luteus UH-Slu-Lm8-n1 |
| K9PHG5_9CYAN/254-454 | 3.2e-25 | Calothrix sp. PCC 7507 |
| M5EDP9_MALS4/116-314 | 2.9e-25 | Malassezia sympodialis |
| A0A0A9WZ44_LYGHE/72-281 | 2.2e-25 | Lygus hesperus |
| W6L2T2_9TRYP/766-996 | 4.0e-25 | Phytomonas sp. isolate EM1 |
| A9B3U8_HERA2/146-337 | 3.9e-25 | Herpetosiphon aurantiacus |
| V2XWH8_MONRO/167-378 | 4.0e-25 | Moniliophthora roreri |
| F9WEU5_TRYCI/57-220 | 3.1e-25 | Trypanosoma congolense |
| E8NHG7_LEIMU/449-698 | 4.5e-25 | Leishmania mexicana |
| A9B2P0_HERA2/144-338 | 5.2e-25 | Herpetosiphon aurantiacus |
| G3ASR4_SPAPN/143-333 | 5.9e-25 | Spathaspora passalidarum |
| A0A061IY53_TRYRA/747-975 | 7.1e-25 | Trypanosoma rangeli SC58 |
| A8N4X4_COPC7/239-461 | 9.5e-25 | Coprinopsis cinerea |
| Q4QCS5_LEIMA/1-251 | 8.9e-25 | Leishmania major |
| C5K6D8_PERM5/300-526 | 1.0e-24 | Perkinsus marinus |
| S8E6W4_FOMPI/137-334 | 9.4e-25 | Fomitopsis pinicola |
| E2L3M3_MONPE/4-110 | 6.6e-25 | Moniliophthora perniciosa |
| F7AJW1_ORNAN/6-93 | 1.7e-24 | Ornithorhynchus anatinus |
| S9TV03_9TRYP/517-699 | 5.3e-22 | Angomonas deanei |
| T1FSF8_HELRO/264-414 | 4.5e-24 | Helobdella robusta |
| F0Y3E3_AURAN/3-105 | 1.3e-24 | Aureococcus anophagefferens |
| A0A084QI99_9HYPO/259-471 | 1.8e-24 | Stachybotrys chlorohalonata IBT 40285 |
| A0A0D6EG25_SPOSA/676-856 | 2.9e-24 | Sporobolomyces salmonicolor |
| C1MP30_MICPC/97-384 | 2.6e-24 | Micromonas pusilla |
| Q07SP7_RHOP5/287-497 | 3.5e-24 | Rhodopseudomonas palustris BisA53 |
| W7MQG0_GIBM7/138-334 | 2.5e-22 | Gibberella moniliformis |
| F0XWR7_AURAN/2-129 | 2.3e-24 | Aureococcus anophagefferens |
| A0A084PRZ3_STACH/259-472 | 3.4e-24 | Stachybotrys chartarum IBT 40293 |
| W5LJM1_ASTMX/40-153 | 3.2e-24 | Astyanax mexicanus |
| W5JMS3_ANODA/25-213 | 1.3e-23 | Anopheles darlingi |
| A0A0B5JKJ3_PANHP/3-118 | 3.1e-24 | Pangasianodon hypophthalmus |
| T1KIH4_TETUR/88-256 | 4.7e-24 | Tetranychus urticae |
| K9VRC2_9CYAN/1103-1315 | 6.2e-24 | Oscillatoria nigro-viridis PCC 7112 |
| R4V0E4_COPFO/40-217 | 4.2e-24 | Coptotermes formosanus |
| R9AIP0_WALI9/153-373 | 4.4e-23 | Wallemia ichthyophaga |
| K2N279_TRYCR/279-503 | 6.4e-24 | Trypanosoma cruzi marinkellei |
| A0A066W1Q0_9HOMO/180-387 | 5.9e-24 | Rhizoctonia solani AG-8 WAC10335 |
| A8LCE5_FRASN/379-564 | 7.4e-24 | Frankia sp. EAN1pec |
| R7TFM3_CAPTE/115-260 | 9.6e-24 | Capitella teleta |
| A0A084RQK5_STACH/259-471 | 5.7e-24 | Stachybotrys chartarum IBT 40288 |
| A0A084B9Y5_STACH/259-471 | 5.7e-24 | Stachybotrys chartarum IBT 7711 |
| K7EHZ9_ORNAN/2-130 | 4.3e-24 | Ornithorhynchus anatinus |
| F0XY19_AURAN/93-206 | 5.0e-24 | Aureococcus anophagefferens |
| A0A0D2FQD6_9EURO/181-398 | 5.7e-24 | Cladophialophora bantiana CBS 173.52 |
| A0A087T1E2_9ARAC/2-111 | 5.5e-24 | Stegodyphus mimosarum |
| R1DUJ7_EMIHU/15-126 | 6.4e-24 | Emiliania huxleyi CCMP1516 |
| F0J9N6_AMBVA/61-171 | 6.3e-24 | Amblyomma variegatum |
| A0A0C3E7M1_9HOMO/151-347 | 1.1e-23 | Scleroderma citrinum Foug A |
| A5EMK2_BRASB/245-471 | 1.1e-23 | Bradyrhizobium sp. |
| A3LNN9_PICST/144-335 | 1.0e-23 | Scheffersomyces stipitis) |
| W9LBI1_FUSOX/198-350 | 2.1e-23 | Fusarium oxysporum f. sp. lycopersici MN25 |
| D8PPJ9_SCHCM/158-347 | 1.8e-23 | Schizophyllum commune |
| W7X4E3_TETTS/134-344 | 8.4e-24 | Tetrahymena thermophila |
| F9FLX1_FUSOF/102-229 | 2.3e-23 | Fusarium oxysporum |
| C1N3N4_MICPC/101-388 | 2.3e-23 | Micromonas pusilla |
| C3Y6P2_BRAFL/12-98 | 1.9e-23 | Branchiostoma floridae |
| G3B031_CANTC/137-321 | 2.0e-23 | Candida tenuis |
| G0UNS9_TRYCI/348-407 | 3.6e-07 | Trypanosoma congolense |
| G0UNS9_TRYCI/450-562 | 1.9e-10 | Trypanosoma congolense |
| F9GDE8_FUSOF/34-190 | 2.4e-23 | Fusarium oxysporum |
| F0XXC4_AURAN/3-113 | 1.7e-23 | Aureococcus anophagefferens |
| H0SNL4_9BRAD/15-241 | 2.4e-23 | Bradyrhizobium sp. ORS 375 |
| Q17E42_AEDAE/113-287 | 2.5e-23 | Aedes aegypti |
| C1N3N6_MICPC/195-489 | 3.0e-23 | Micromonas pusilla |
| S9R3Z3_9DELT/96-302 | 7.3e-23 | Cystobacter fuscus DSM 2262 |
| S4RHV9_PETMA/3-112 | 2.9e-23 | Petromyzon marinus |
| K4DTR4_TRYCR/247-487 | 3.5e-23 | Trypanosoma cruzi |
| A0A066XIU5_COLSU/258-472 | 3.3e-23 | Colletotrichum sublineola |
| V5D5V8_TRYCR/362-602 | 3.5e-23 | Trypanosoma cruzi Dm28c |
| K5WYB9_AGABU/205-414 | 6.3e-23 | Agaricus bisporus var. burnettii |
| W5QAQ3_SHEEP/62-173 | 3.2e-20 | Ovis aries |
| I1FIB4_AMPQE/32-165 | 6.7e-23 | Amphimedon queenslandica |
| D8LU45_ECTSI/318-466 | 5.3e-23 | Ectocarpus siliculosus |
| K9HEC0_AGABB/189-370 | 6.1e-23 | Agaricus bisporus var. bisporus |
| A0A0C9Z8E4_9HOMO/171-356 | 5.8e-23 | Pisolithus microcarpus 441 |
| E3Q4I4_COLGM/258-470 | 5.5e-23 | Colletotrichum graminicola |
| A0A091ND36_9PASS/3-101 | 3.9e-23 | Acanthisitta chloris |
| W9HA60_FUSOX/59-199 | 5.1e-23 | Fusarium oxysporum FOSC 3-a |
| K2M6K4_TRYCR/746-975 | 7.6e-23 | Trypanosoma cruzi marinkellei |
| G4TJ48_PIRID/204-412 | 1.1e-22 | Piriformospora indica |
| Q4H2T1_CIOIN/3-112 | 1.2e-22 | Ciona intestinalis |
| M3HKH3_CANMX/141-330 | 1.5e-22 | Candida maltosa |
| Q4DQ12_TRYCC/746-975 | 9.8e-23 | Trypanosoma cruzi |
| A0A067SZN1_9AGAR/187-423 | 9.0e-23 | Galerina marginata CBS 339.88 |
| A0A078A0N5_STYLE/104-346 | 7.3e-23 | Stylonychia lemnae |
| A0A094KEM7_ANTCR/5-83 | 1.1e-22 | Antrostomus carolinensis |
| A0A067PYN1_9HOMO/171-370 | 1.2e-22 | Jaapia argillacea MUCL 33604 |
| M4ZBJ4_9BRAD/253-478 | 1.4e-22 | Bradyrhizobium oligotrophicum S58 |
| Q4DE63_TRYCC/746-975 | 1.5e-22 | Trypanosoma cruzi |
| G0UTZ5_TRYCI/337-508 | 1.4e-22 | Trypanosoma congolense |
| Q4DQN7_TRYCC/362-602 | 1.5e-22 | Trypanosoma cruzi |
| I4VYX7_9GAMM/174-402 | 1.6e-22 | Rhodanobacter spathiphylli B39 |
| A4YRP8_BRASO/249-471 | 2.2e-22 | Bradyrhizobium sp. ORS278 |
| S9UD26_9TRYP/5-211 | 2.1e-22 | Strigomonas culicis |
| R4X6S2_TAPDE/192-394 | 3.8e-22 | Taphrina deformans |
| F0YAK9_AURAN/1-98 | 1.5e-22 | Aureococcus anophagefferens |
| S9VPM5_9TRYP/1-208 | 1.7e-22 | Strigomonas culicis |
| K4DUR1_TRYCR/746-975 | 2.6e-22 | Trypanosoma cruzi |
| A4HBK9_LEIBR/754-971 | 2.6e-22 | Leishmania braziliensis |
| A0A0D6YGD1_MASLA/10-210 | 2.0e-22 | Mastigocladus laminosus UU774 |
| U6H689_9EIME/190-343 | 8.3e-20 | Eimeria praecox |
| V5BQ28_TRYCR/746-975 | 2.8e-22 | Trypanosoma cruzi Dm28c |
| W9JBW5_FUSOX/300-508 | 2.5e-22 | Fusarium oxysporum Fo47 |
| H0TAL7_9BRAD/252-473 | 3.2e-22 | Bradyrhizobium sp. STM 3809 |
| A0A0B2UUE7_TOXCA/1-75 | 2.2e-22 | Toxocara canis |
| B5DKM0_DROPS/6-109 | 2.4e-22 | Drosophila pseudoobscura pseudoobscura |
| W9NHM8_FUSOX/270-478 | 3.0e-22 | Fusarium oxysporum f. sp. pisi HDV247 |
| A0A0A6MHV0_CANAL/145-327 | 3.7e-22 | Candida albicans |
| C5MJ68_CANTT/178-362 | 3.9e-22 | Candida tropicalis |
| C3Y6N4_BRAFL/8-80 | 3.4e-22 | Branchiostoma floridae |
| A0A0A6LPV4_CANAL/146-327 | 4.1e-22 | Candida albicans |
| A0A088S8Y6_9TRYP/754-971 | 4.0e-22 | Leishmania panamensis |
| E3NW07_CAERE/23-148 | 3.5e-22 | Caenorhabditis remanei |
| A0A061IUP7_TRYRA/260-428 | 4.5e-22 | Trypanosoma rangeli SC58 |
| A0A0C1R747_9CYAN/352-551 | 6.3e-22 | Tolypothrix bouteillei VB521301 |
| H8X996_CANO9/141-331 | 5.0e-22 | Candida orthopsilosis |
| A0A0A3X219_CANAX/145-327 | 5.4e-22 | Candida albicans 12C |
| A0A0A6JPE2_CANAX/145-327 | 5.5e-22 | Candida albicans P37039 |
| A0A0A3DJT2_CANAX/145-327 | 5.5e-22 | Candida albicans P78048 |
| A0A0A3DUJ6_CANAX/145-327 | 5.5e-22 | Candida albicans P37037 |
| G3TW01_LOXAF/3-108 | 4.8e-22 | Loxodonta africana |
| A0A0A6KBT2_CANAX/146-328 | 5.6e-22 | Candida albicans P75010 |
| C4YS11_CANAW/146-327 | 5.9e-22 | Candida albicans) |
| A0A0A6LII6_CANAX/146-327 | 5.9e-22 | Candida albicans P78042 |
| A0A0A4BYW2_CANAX/146-327 | 5.9e-22 | Candida albicans P57055 |
| A0A0A4BHS3_CANAX/146-327 | 5.9e-22 | Candida albicans P34048 |
| A0A0A6LWQ0_CANAX/145-327 | 5.7e-22 | Candida albicans P60002 |
| A0A0A4BUJ7_CANAX/145-327 | 5.7e-22 | Candida albicans P75063 |
| A0A0A6IEB5_CANAX/146-327 | 6.0e-22 | Candida albicans Ca6 |
| A0A0A3DEB3_CANAX/146-327 | 6.0e-22 | Candida albicans P57072 |
| A0A0A6KKT9_CANAX/146-327 | 6.0e-22 | Candida albicans P76067 |
| A0A0A3BSM0_CANAX/146-327 | 6.0e-22 | Candida albicans P94015 |
| A0A0A4A1Y3_CANAX/145-327 | 5.9e-22 | Candida albicans P87 |
| PALB_CANAL/146-327 | 6.0e-22 | Candida albicans |
| A0A0A4A9K4_CANAX/146-327 | 6.0e-22 | Candida albicans 19F |
| A0A0A3BY56_CANAX/146-327 | 6.0e-22 | Candida albicans P37005 |
| A0A0A4A5H0_CANAX/146-327 | 6.0e-22 | Candida albicans L26 |
| A0A0A3CFZ0_CANAX/146-327 | 6.0e-22 | Candida albicans GC75 |
| A0A0A6I7K3_CANAX/146-327 | 6.0e-22 | Candida albicans P76055 |
| A0A0A6KUN5_CANAX/146-327 | 6.0e-22 | Candida albicans P75016 |
| G6FUE0_9CYAN/351-553 | 6.5e-22 | Fischerella sp. JSC-11 |
| Q4XM66_PLACH/155-328 | 8.4e-22 | Plasmodium chabaudi |
| E3MW78_CAERE/130-282 | 7.7e-22 | Caenorhabditis remanei |
| G8BGN7_CANPC/142-330 | 6.7e-22 | Candida parapsilosis |
| B4DEU4_HUMAN/75-152 | 1.0e-20 | Homo sapiens |
| H3DGU1_TETNG/1-89 | 6.1e-22 | Tetraodon nigroviridis |
| B0DXY2_LACBS/187-409 | 5.0e-21 | Laccaria bicolor |
| W5JYC6_ASTMX/1-125 | 6.5e-22 | Astyanax mexicanus |
| H9GHD2_ANOCA/40-154 | 7.6e-22 | Anolis carolinensis |
| K2MWN5_TRYCR/14-254 | 7.1e-22 | Trypanosoma cruzi marinkellei |
| W9Z384_FUSOX/33-242 | 6.5e-22 | Fusarium oxysporum f. sp. melonis 26406 |
| A0A0C9XMZ7_9AGAR/188-406 | 1.2e-20 | Laccaria amethystina LaAM-08-1 |
| H3FJX1_PRIPA/67-128 | 0.00037 | Pristionchus pacificus |
| H3FJX1_PRIPA/133-179 | 1.1e-07 | Pristionchus pacificus |
| B9WI45_CANDC/145-327 | 1.2e-21 | Candida dubliniensis |
| A0A0C3B6Y0_9HOMO/186-333 | 2.1e-21 | Serendipita vermifera MAFF 305830 |
| M5BZ36_THACB/3-90 | 1.0e-21 | Thanatephorus cucumeris |
| C6K3U6_9TRYP/1089-1338 | 3.1e-21 | Crithidia sp. ATCC 30255 |
| G0U5I2_TRYVY/759-978 | 1.4e-21 | Trypanosoma vivax |
| PALB_DEBHA/154-339 | 1.4e-21 | Debaryomyces hansenii |
| X0KP85_FUSOX/300-508 | 1.4e-21 | Fusarium oxysporum f. sp. vasinfectum 25433 |
| H0S4P6_9BRAD/248-474 | 2.3e-21 | Bradyrhizobium sp. ORS 285 |
| L7FM96_ENTIV/7-110 | 2.1e-21 | Entamoeba invadens IP1 |
| U6MH64_EIMMA/116-329 | 1.3e-10 | Eimeria maxima |
| U6MH64_EIMMA/341-420 | 0.00014 | Eimeria maxima |
| H1VAR9_COLHI/260-473 | 1.9e-21 | Colletotrichum higginsianum |
| Q8YXX3_NOSS1/367-581 | 4.1e-21 | Nostoc sp. |
| A0A0B7C0E5_9EUPU/3-115 | 1.4e-21 | Arion vulgaris |
| C3ZZL6_BRAFL/50-169 | 2.4e-21 | Branchiostoma floridae |
| B7PEU5_IXOSC/4-71 | 1.3e-10 | Ixodes scapularis |
| B7PEU5_IXOSC/79-170 | 5.7e-05 | Ixodes scapularis |
| F4F232_VERMA/4067-4302 | 3.4e-08 | Verrucosispora maris |
| F4F232_VERMA/4791-5034 | 7.5e-07 | Verrucosispora maris |
| W9II83_FUSOX/270-477 | 2.7e-21 | Fusarium oxysporum FOSC 3-a |
| A0A060YEC2_ONCMY/203-298 | 5.7e-19 | Oncorhynchus mykiss |
| X0F1G5_FUSOX/270-477 | 2.7e-21 | Fusarium oxysporum f. sp. radicis-lycopersici 26381 |
| W5L8J6_ASTMX/26-102 | 2.3e-21 | Astyanax mexicanus |
| Q2J4J6_FRASC/37-250 | 9.1e-21 | Frankia sp. CcI3 |
| W6LBN7_9TRYP/767-996 | 4.2e-21 | Phytomonas sp. isolate Hart1 |
| J9FWJ6_9SPIT/104-349 | 4.8e-21 | Oxytricha trifallax |
| E3MBK7_CAERE/101-231 | 7.5e-21 | Caenorhabditis remanei |
| A0A0C2WGV8_AMAMU/200-388 | 3.9e-21 | Amanita muscaria Koide BX008 |
| A0A0C1RMX3_9CYAN/260-459 | 4.4e-21 | Tolypothrix bouteillei VB521301 |
| W4YV93_STRPU/3-131 | 3.0e-21 | Strongylocentrotus purpuratus |
| A0A0C2L0I7_9CYAN/358-563 | 4.8e-21 | Tolypothrix campylonemoides VB511288 |
| A0A010R2D7_9PEZI/260-473 | 3.9e-21 | Colletotrichum fioriniae PJ7 |
| E3MBL1_CAERE/116-246 | 9.9e-21 | Caenorhabditis remanei |
| F0Y7C1_AURAN/51-158 | 3.1e-21 | Aureococcus anophagefferens |
| C7ZF18_NECH7/267-473 | 5.9e-21 | Nectria haematococca |
| N4VJ92_COLOR/258-471 | 5.0e-21 | Colletotrichum orbiculare |
| X0B9I2_FUSOX/270-477 | 5.0e-21 | Fusarium oxysporum f. sp. melonis 26406 |
| J4U426_SACK1/88-348 | 5.1e-21 | Saccharomyces kudriavzevii) |
| W9M398_FUSOX/270-477 | 5.0e-21 | Fusarium oxysporum f. sp. lycopersici MN25 |
| W9L6X8_FUSOX/270-477 | 5.0e-21 | Fusarium oxysporum Fo47 |
| D2VGX6_NAEGR/380-593 | 6.0e-21 | Naegleria gruberi |
| X0CGQ5_FUSOX/269-476 | 5.5e-21 | Fusarium oxysporum f. sp. raphani 54005 |
| N4UKJ6_FUSC1/269-476 | 5.5e-21 | Fusarium oxysporum f. sp. cubense |
| A0A077X1B5_9FUNG/356-475 | 8.7e-21 | Absidia idahoensis var. thermophila |
| B0JUQ7_MICAN/457-595 | 5.3e-19 | Microcystis aeruginosa |
| G3WY47_SARHA/135-277 | 5.0e-21 | Sarcophilus harrisii |
| T0K161_COLGC/260-473 | 7.4e-21 | Colletotrichum gloeosporioides |
| L2FFI6_COLGN/260-473 | 7.4e-21 | Colletotrichum gloeosporioides |
| A0A034U6P0_9NOCA/3906-4018 | 1.8e-08 | Nocardia brasiliensis NBRC 14402 |
| B3DJE9_DANRE/1-118 | 9.8e-21 | Danio rerio |
| R7UXL9_CAPTE/3-111 | 7.1e-21 | Capitella teleta |
| W9NXZ4_FUSOX/269-476 | 1.1e-20 | Fusarium oxysporum f. sp. pisi HDV247 |
| A0A0D7BH39_9HOMO/159-354 | 1.2e-20 | Cylindrobasidium torrendii FP15055 ss-10 |
| F9G4N6_FUSOF/1-154 | 8.3e-21 | Fusarium oxysporum |
| K9TN70_9CYAN/1678-1906 | 1.6e-20 | Oscillatoria acuminata PCC 6304 |
| E5T2W1_TRISP/8-84 | 1.4e-20 | Trichinella spiralis |
| X0IWW6_FUSOX/270-477 | 1.5e-20 | Fusarium oxysporum f. sp. cubense |
| K9WYB7_9NOST/237-392 | 7.7e-12 | Cylindrospermum stagnale PCC 7417 |
| S9VMY0_9TRYP/270-405 | 1.7e-20 | Angomonas deanei |
| Q05QW3_9SYNE/489-599 | 9.6e-16 | Synechococcus sp. RS9916 |
| F9F8A0_FUSOF/269-476 | 1.7e-20 | Fusarium oxysporum |
| X0H2Z7_FUSOX/269-476 | 1.7e-20 | Fusarium oxysporum f. sp. conglutinans |
| A2GDY5_TRIVA/1147-1250 | 2.0e-20 | Trichomonas vaginalis |
| D2VVA0_NAEGR/505-612 | 2.6e-20 | Naegleria gruberi |
| E9B1J1_LEIMU/383-621 | 3.6e-19 | Leishmania mexicana) |
| K2NK14_TRYCR/718-953 | 5.7e-20 | Trypanosoma cruzi marinkellei |
| K8GKJ1_9CYAN/592-821 | 4.5e-20 | Oscillatoriales cyanobacterium JSC-12 |
| M3A826_PSEFD/62-226 | 3.1e-20 | Pseudocercospora fijiensis |
| R1ETD8_EMIHU/179-413 | 3.1e-20 | Emiliania huxleyi CCMP1516 |
| A0A0D2YDR5_FUSO4/270-478 | 3.4e-20 | Fusarium oxysporum f. sp. lycopersici |
| R1G0N6_EMIHU/177-410 | 4.0e-20 | Emiliania huxleyi CCMP1516 |
| A0A062X6D4_9ACTN/407-620 | 8.7e-20 | Frankia sp. BMG5.23 |
| W9D518_9ACTN/407-620 | 8.7e-20 | Frankia sp. CcI6 |
| H9FM62_MACMU/5-74 | 2.5e-20 | Macaca mulatta |
| D3CT47_9ACTN/352-544 | 4.8e-20 | Frankia sp. EUN1f |
| W1QG42_OGAPD/117-304 | 4.4e-20 | Ogataea parapolymorpha |
| H9KVY5_CALJA/56-133 | 5.3e-20 | Callithrix jacchus |
| H9KVY9_CALJA/55-132 | 5.3e-20 | Callithrix jacchus |
| H9KVY7_CALJA/55-132 | 5.3e-20 | Callithrix jacchus |
| G3UGM0_LOXAF/1-111 | 6.5e-20 | Loxodonta africana |
| K2NQP4_TRYCR/260-424 | 6.4e-20 | Trypanosoma cruzi marinkellei |
| A0A067RCC1_ZOONE/77-214 | 7.7e-20 | Zootermopsis nevadensis |
| E2LW83_MONPE/158-307 | 7.9e-20 | Moniliophthora perniciosa |
| Q4QCK4_LEIMA/780-997 | 9.8e-20 | Leishmania major |
| A0A093XD05_PENMA/229-352 | 9.1e-20 | Talaromyces marneffei PM1 |
| PALB_YARLI/148-351 | 8.7e-20 | Yarrowia lipolytica |
| O18165_CAEEL/27-187 | 8.9e-20 | Caenorhabditis elegans |
| Q4DJS6_TRYCC/227-399 | 2.5e-16 | Trypanosoma cruzi |
| A0A060X6A1_ONCMY/8-91 | 1.4e-19 | Oncorhynchus mykiss |
| F6HVW5_VITVI/9-90 | 8.9e-20 | Vitis vinifera |
| F9WI93_TRYCI/337-466 | 1.4e-19 | Trypanosoma congolense |
| Q4CQB4_TRYCC/260-438 | 1.6e-19 | Trypanosoma cruzi |
| F0Y3I5_AURAN/2-110 | 1.1e-19 | Aureococcus anophagefferens |
| X0BAU3_FUSOX/300-508 | 1.6e-19 | Fusarium oxysporum f. sp. raphani 54005 |
| E3NM47_CAERE/104-238 | 3.0e-19 | Caenorhabditis remanei |
| A0A061B3K9_CYBFA/145-327 | 2.8e-19 | Cyberlindnera fabianii |
| A2E1X1_TRIVA/77-262 | 9.5e-19 | Trichomonas vaginalis |
| J3PI43_GAGT3/303-544 | 3.3e-19 | Gaeumannomyces graminis var. tritici |
| K4E6V7_TRYCR/715-970 | 2.1e-18 | Trypanosoma cruzi |
| Q38PN9_PIG/7-83 | 1.7e-19 | Sus scrofa |
| E9BLU7_LEIDB/426-489 | 6.6e-07 | Leishmania donovani |
| E9BLU7_LEIDB/645-700 | 1.7e-06 | Leishmania donovani |
| Q4Q6M3_LEIMA/383-621 | 3.8e-18 | Leishmania major |
| A0A078AWP9_STYLE/111-318 | 3.8e-19 | Stylonychia lemnae |
| Q4D4Z7_TRYCC/278-455 | 3.3e-19 | Trypanosoma cruzi |
| K4DWE7_TRYCR/260-438 | 3.6e-19 | Trypanosoma cruzi |
| A4I6K5_LEIIN/383-620 | 3.8e-18 | Leishmania infantum |
| K9R8R5_9CYAN/871-1071 | 5.0e-19 | Rivularia sp. PCC 7116 |
| Q4D608_TRYCC/714-969 | 3.9e-18 | Trypanosoma cruzi |
| A0A093HFK7_TYTAL/10-86 | 4.3e-19 | Tyto alba |
| S9TUV0_9TRYP/327-565 | 1.7e-18 | Strigomonas culicis |
| S9V5J5_9TRYP/327-565 | 1.8e-18 | Strigomonas culicis |
| L7LVA0_9ACAR/5-79 | 5.2e-19 | Rhipicephalus pulchellus |
| A4I5N1_LEIIN/426-489 | 1.2e-06 | Leishmania infantum |
| A4I5N1_LEIIN/645-700 | 1.7e-06 | Leishmania infantum |
| E9BMG6_LEIDB/383-620 | 2.5e-18 | Leishmania donovani |
| A0A0D8BDY2_9ACTN/388-588 | 7.0e-19 | Frankia sp. CpI1-S |
| K1PTS6_CRAGI/94-239 | 8.0e-19 | Crassostrea gigas |
| A0A0D2XRG0_FUSO4/270-477 | 7.1e-19 | Fusarium oxysporum f. sp. lycopersici |
| K0KJJ1_WICCF/141-318 | 9.3e-19 | Wickerhamomyces ciferrii |
| A0A066V4Z3_9HOMO/1-123 | 6.1e-16 | Rhizoctonia solani AG-8 WAC10335 |
| V5BII7_TRYCR/715-973 | 7.3e-18 | Trypanosoma cruzi Dm28c |
| M0QZ20_HUMAN/75-179 | 8.1e-19 | Homo sapiens |
| A0A061GX80_THECC/1743-1817 | 1.2e-18 | theobroma_cacao |
| Q0RAZ2_FRAAA/402-602 | 1.1e-18 | Frankia alni |
| X0B9F4_FUSOX/6-196 | 8.0e-19 | Fusarium oxysporum f. sp. raphani 54005 |
| A2DTE8_TRIVA/1027-1255 | 1.3e-18 | Trichomonas vaginalis |
| G3SU16_LOXAF/7-83 | 8.5e-18 | Loxodonta africana |
| E9AUX8_LEIMU/778-995 | 1.5e-18 | Leishmania mexicana |
| E9B0X6_LEIMU/426-488 | 1.5e-06 | Leishmania mexicana |
| E9B0X6_LEIMU/655-710 | 3.5e-06 | Leishmania mexicana |
| A9V7E5_MONBE/21-88 | 1.3e-18 | Monosiga brevicollis |
| Q4CS86_TRYCC/51-158 | 1.1e-18 | Trypanosoma cruzi |
| S9TQD4_PHAFV/219-422 | 2.1e-18 | Phaeospirillum fulvum MGU-K5 |
| E3LMG6_CAERE/62-229 | 1.7e-18 | Caenorhabditis remanei |
| A0A091UG85_PHORB/6-83 | 1.9e-18 | Phoenicopterus ruber ruber |
| A0A0C4E8D6_MAGP6/255-410 | 2.2e-18 | Magnaporthiopsis poae |
| D3HJL4_LEGLN/140-290 | 5.3e-08 | Legionella longbeachae serogroup 1 |
| D3HJL4_LEGLN/455-530 | 0.00017 | Legionella longbeachae serogroup 1 |
| S9V6X0_9TRYP/338-543 | 2.4e-18 | Strigomonas culicis |
| PALB_CRYNB/176-372 | 3.1e-18 | Cryptococcus neoformans var. neoformans |
| Q38C53_TRYB2/748-977 | 2.8e-18 | Trypanosoma brucei brucei |
| D0A1K3_TRYB9/748-977 | 2.8e-18 | Trypanosoma brucei gambiense |
| M5C792_THACB/139-249 | 1.0e-15 | Thanatephorus cucumeris |
| A0A095CG77_CRYGA/174-365 | 3.9e-18 | Cryptococcus gattii R265 |
| A0A0D0WF33_CRYGA/174-365 | 3.9e-18 | Cryptococcus gattii CA1014 |
| Q4Q790_LEIMA/426-489 | 1.1e-06 | Leishmania major |
| Q4Q790_LEIMA/649-699 | 1.8e-05 | Leishmania major |
| A0A088RXA9_9TRYP/462-710 | 1.9e-17 | Leishmania panamensis |
| E2LXH8_MONPE/99-186 | 6.7e-18 | Moniliophthora perniciosa |
| G0NNN5_CAEBE/3-155 | 6.4e-18 | Caenorhabditis brenneri |
| A4HJ15_LEIBR/944-1185 | 1.7e-17 | Leishmania braziliensis |
| X0BEX0_FUSOX/8-140 | 7.0e-18 | Fusarium oxysporum f. sp. raphani 54005 |
| F9GC11_FUSOF/14-175 | 6.2e-18 | Fusarium oxysporum |
| A0A0D0T8N4_CRYGA/174-365 | 9.9e-18 | Cryptococcus gattii MMRL2647 |
| A0A0D0YCE1_CRYGA/174-365 | 9.9e-18 | Cryptococcus gattii 2001/935-1 |
| A0A0D2IET5_CRYGA/174-365 | 9.9e-18 | Cryptococcus gattii 99/473 |
| A0A0D0V0Z6_CRYGA/174-365 | 9.9e-18 | Cryptococcus gattii Ram5 |
| B7QCA9_IXOSC/1-81 | 3.0e-14 | Ixodes scapularis |
| K3WI09_PYTUL/159-258 | 8.2e-18 | Pythium ultimum DAOM BR144 |
| A0A0D0YL62_CRYGA/173-365 | 9.9e-18 | Cryptococcus gattii IND107 |
| V4B3Z9_LOTGI/98-243 | 1.4e-17 | Lottia gigantea |
| W4JPH0_9HOMO/184-339 | 1.1e-17 | Heterobasidion irregulare TC 32-1 |
| A4HID5_LEIBR/425-491 | 7.8e-07 | Leishmania braziliensis |
| A4HID5_LEIBR/651-710 | 8.0e-05 | Leishmania braziliensis |
| G0U463_TRYVY/269-396 | 1.6e-17 | Trypanosoma vivax |
| G8YT52_PICSO/172-358 | 1.9e-17 | Pichia sorbitophila |
| W4XK54_STRPU/9-75 | 1.6e-17 | Strongylocentrotus purpuratus |
| A0A088SF39_9TRYP/425-491 | 7.8e-07 | Leishmania panamensis |
| A0A088SF39_9TRYP/652-709 | 0.00011 | Leishmania panamensis |
| G8YUK7_PICSO/164-349 | 2.9e-17 | Pichia sorbitophila |
| A0A0D0Z7X6_CRYGA/174-362 | 2.3e-17 | Cryptococcus gattii CBS 10090 |
| A0A0D0TX07_CRYGA/174-362 | 2.3e-17 | Cryptococcus gattii LA55 |
| A0A0D0WHC8_CRYGA/174-365 | 2.1e-17 | Cryptococcus gattii CA1873 |
| A0A0D0V9J3_CRYGA/174-365 | 2.1e-17 | Cryptococcus gattii CA1280 |
| J9W1Z0_CRYNH/176-372 | 2.3e-17 | Cryptococcus neoformans var. grubii |
| U3I6E1_ANAPL/44-96 | 5.8e-05 | Anas platyrhynchos |
| U3I6E1_ANAPL/195-264 | 1.7e-06 | Anas platyrhynchos |
| G0PIH4_CAEBE/3-155 | 2.5e-17 | Caenorhabditis brenneri |
| K9WJ87_9CYAN/381-490 | 2.9e-15 | Microcoleus sp. PCC 7113 |
| A0A061R4Y8_9CHLO/197-309 | 3.4e-17 | Tetraselmis sp. GSL018 |
| A0A061R6S0_9CHLO/4-94 | 2.7e-17 | Tetraselmis sp. GSL018 |
| A0A0A9Y2J4_LYGHE/80-217 | 3.3e-17 | Lygus hesperus |
| W5JY59_ASTMX/18-84 | 2.9e-17 | Astyanax mexicanus |
| K4QVY3_9ACTN/266-471 | 4.6e-17 | Streptomyces davawensis JCM 4913 |
| A0A088RU36_9TRYP/445-654 | 5.0e-17 | Leishmania panamensis |
| D4PBR8_CAPHI/7-81 | 3.1e-17 | Capra hircus |
| F0Y5U5_AURAN/376-532 | 5.4e-17 | Aureococcus anophagefferens |
| Q0BRV8_GRABC/56-250 | 9.0e-17 | Granulibacter bethesdensis |
| Q388U3_TRYB2/191-323 | 6.4e-17 | Trypanosoma brucei brucei |
| W6L6R0_9TRYP/816-1044 | 8.7e-17 | Phytomonas sp. isolate EM1 |
| S9VGK0_9TRYP/469-674 | 9.0e-17 | Angomonas deanei |
| A0A061QPI1_9CHLO/4-94 | 8.3e-17 | Tetraselmis sp. GSL018 |
| D0A5C8_TRYB9/259-391 | 1.2e-16 | Trypanosoma brucei gambiense |
| F9GET4_FUSOF/18-171 | 9.7e-17 | Fusarium oxysporum |
| A0A0A8EHD2_9ACTN/201-394 | 1.8e-16 | Streptomyces sp. 769 |
| U6GG17_EIMAC/168-461 | 1.3e-15 | Eimeria acervulina |
| X0UY67_9ZZZZ/97-236 | 1.7e-16 | marine sediment metagenome |
| M1EE89_MUSPF/2-76 | 1.3e-16 | Mustela putorius furo |
| S9VKA0_9TRYP/7-77 | 2.0e-16 | Strigomonas culicis |
| A0A059KL99_9BURK/910-1123 | 2.4e-16 | Sphaerotilus natans subsp. natans DSM 6575 |
| S9UCC6_9TRYP/284-520 | 5.0e-16 | Angomonas deanei |
| W3WJL4_9PEZI/277-499 | 2.3e-16 | Pestalotiopsis fici W106-1 |
| U6LDW2_9EIME/814-868 | 2.2e-09 | Eimeria brunetti |
| H3DGU0_TETNG/75-153 | 2.1e-16 | Tetraodon nigroviridis |
| K5W904_PHACS/2-122 | 3.1e-16 | Phanerochaete carnosa |
| S9TV13_9TRYP/875-1048 | 2.8e-16 | Angomonas deanei |
| Q05QW2_9SYNE/499-705 | 7.1e-16 | Synechococcus sp. RS9916 |
| A0A0B7A9L2_9EUPU/6-84 | 1.8e-16 | Arion vulgaris |
| A0A091QXG2_MERNU/2-68 | 2.0e-16 | Merops nubicus |
| G7XAP5_ASPKW/42-153 | 2.2e-16 | Aspergillus kawachii |
| I3LQD3_PIG/3-77 | 3.2e-16 | Sus scrofa |
| E3NRK6_CAERE/57-244 | 3.1e-16 | Caenorhabditis remanei |
| A9B2A3_HERA2/141-361 | 5.2e-16 | Herpetosiphon aurantiacus |
| I9KLP1_9ACTN/372-572 | 1.0e-15 | Frankia sp. QA3 |
| E9PSA6_HUMAN/85-163 | 3.2e-16 | Homo sapiens |
| S9UIU6_9TRYP/633-869 | 9.7e-16 | Angomonas deanei |
| A0A0D2WA88_CRYGA/173-364 | 5.2e-16 | Cryptococcus gattii NT-10 |
| A0A0D0XZU8_CRYGA/173-364 | 5.2e-16 | Cryptococcus gattii EJB2 |
| A0A0D2KIB6_CRYGA/173-364 | 5.2e-16 | Cryptococcus gattii E566 |
| E6RE93_CRYGW/173-364 | 5.2e-16 | Cryptococcus gattii serotype B |
| S4RXN0_PETMA/2-68 | 5.4e-16 | Petromyzon marinus |
| F9WFU7_TRYCI/24-226 | 5.5e-16 | Trypanosoma congolense |
| D7G3H8_ECTSI/262-340 | 0.00012 | Ectocarpus siliculosus |
| D7G3H8_ECTSI/367-500 | 1.9e-05 | Ectocarpus siliculosus |
| Q8YQK8_NOSS1/609-797 | 6.8e-16 | Nostoc sp. |
| A0A0D0UVV2_CRYGA/173-364 | 6.8e-16 | Cryptococcus gattii Ru294 |
| W6I3S0_9PROT/58-250 | 1.1e-15 | Granulibacter bethesdensis CGDNIH3 |
| A0A060YCV4_ONCMY/18-103 | 6.9e-16 | Oncorhynchus mykiss |
| A0A078AGW7_STYLE/4-244 | 9.2e-16 | Stylonychia lemnae |
| G5DZK0_9PIPI/1-110 | 5.3e-16 | Hymenochirus curtipes |
| K1UZ01_9ACTN/54-259 | 8.5e-16 | Streptomyces sp. SM8 |
| A0A0F0HXM4_9PSEU/212-408 | 9.6e-16 | Saccharothrix sp. ST-888 |
| K2MYC3_TRYCR/57-219 | 8.3e-16 | Trypanosoma cruzi marinkellei |
| S9TSL6_9TRYP/2-135 | 9.6e-16 | Strigomonas culicis |
| V4INQ4_9ACTN/266-471 | 1.2e-15 | Streptomyces sp. PVA 94-07 |
| E9PJA6_HUMAN/85-162 | 1.1e-15 | Homo sapiens |
| K2MS01_TRYCR/904-1065 | 1.8e-15 | Trypanosoma cruzi marinkellei |
| D9X719_STRVR/316-521 | 1.8e-15 | Streptomyces viridochromogenes DSM 40736 |
| E9BTE3_LEIDB/496-711 | 2.0e-15 | Leishmania donovani |
| A4ICQ6_LEIIN/496-711 | 2.0e-15 | Leishmania infantum |
| S9VBW7_9TRYP/265-421 | 2.2e-15 | Strigomonas culicis |
| A0A0C3FU90_9HOMO/27-169 | 6.5e-15 | Piloderma croceum F 1598 |
| G0UZA2_TRYCI/741-943 | 2.1e-15 | Trypanosoma congolense |
| X0MTA9_STRA9/211-409 | 1.9e-15 | Streptomyces albulus PD-1 |
| A0A059VUX9_STRA9/211-409 | 1.9e-15 | Streptomyces albulus |
| L7IJA2_MAGOY/361-527 | 5.0e-15 | Magnaporthe oryzae |
| L7IR25_MAGOP/361-527 | 5.0e-15 | Magnaporthe oryzae |
| G4N5T4_MAGO7/361-527 | 5.0e-15 | Magnaporthe oryzae |
| A0A0D7CHD0_9ACTN/212-410 | 2.1e-15 | Streptomyces natalensis ATCC 27448 |
| A0A098GDL4_TATMI/119-262 | 8.5e-05 | Tatlockia micdadei |
| A0A098GDL4_TATMI/429-500 | 0.00013 | Tatlockia micdadei |
| E3M575_CAERE/57-244 | 2.3e-15 | Caenorhabditis remanei |
| D0A5Q2_TRYB9/656-855 | 3.2e-15 | Trypanosoma brucei gambiense |
| J9IF86_9SPIT/62-121 | 4.7e-05 | Oxytricha trifallax |
| J9IF86_9SPIT/128-216 | 0.00018 | Oxytricha trifallax |
| I7M1I6_TETTS/284-462 | 5.3e-15 | Tetrahymena thermophila |
| Q387E0_TRYB2/834-1033 | 3.9e-15 | Trypanosoma brucei brucei |
| Q3MBX8_ANAVT/604-796 | 4.9e-15 | Anabaena variabilis) |
| Q6F6K4_CAEEL/3-114 | 8.2e-15 | Caenorhabditis elegans |
| A0A078B0E7_STYLE/498-767 | 5.9e-15 | Stylonychia lemnae |
| M9SJR4_9ACTN/266-471 | 4.0e-15 | Streptomyces albus J1074 |
| Q4Q1Z8_LEIMA/447-662 | 4.8e-15 | Leishmania major |
| Q4R6Y9_MACFA/108-188 | 3.8e-15 | Macaca fascicularis |
| F9F180_FUSOF/36-171 | 4.3e-15 | Fusarium oxysporum |
| D0A5Q4_TRYB9/1080-1278 | 5.8e-15 | Trypanosoma brucei gambiense |
| Q4CW02_TRYCC/763-929 | 6.6e-15 | Trypanosoma cruzi |
| Q387D8_TRYB2/1076-1274 | 6.4e-15 | Trypanosoma brucei brucei |
| U1HFQ4_9BRAD/151-356 | 7.5e-15 | Bradyrhizobium sp. DFCI-1 |
| K4DTC7_TRYCR/405-566 | 7.0e-15 | Trypanosoma cruzi |
| A0A0B7C315_9EUPU/1-66 | 4.8e-15 | Arion vulgaris |
| H9FDP0_MACMU/59-121 | 4.7e-15 | Macaca mulatta |
| D6B0I6_9ACTN/431-636 | 7.4e-15 | Streptomyces albus J1074 |
| A0A0D4DWW2_9ACTN/2291-2531 | 8.6e-15 | Streptomyces lydicus A02 |
| A0A078KUY5_9GAMM/141-285 | 6.1e-06 | Legionella massiliensis |
| W6KFJ7_9TRYP/1501-1690 | 9.9e-15 | Phytomonas sp. isolate EM1 |
| S9TMG5_9TRYP/1878-2072 | 1.1e-14 | Strigomonas culicis |
| A0BM88_PARTE/77-213 | 1.4e-14 | Paramecium tetraurelia |
| L1IK23_GUITH/97-217 | 8.8e-15 | Guillardia theta CCMP2712 |
| Q4CS87_TRYCC/697-858 | 1.2e-14 | Trypanosoma cruzi |
| K2NSY2_TRYCR/509-671 | 1.1e-14 | Trypanosoma cruzi marinkellei |
| G0UZA4_TRYCI/266-463 | 1.2e-14 | Trypanosoma congolense |
| G0U9Y4_TRYVY/314-514 | 1.2e-14 | Trypanosoma vivax |
| K2MAC8_TRYCR/500-658 | 1.4e-14 | Trypanosoma cruzi marinkellei |
| S9V3X2_9TRYP/204-347 | 1.5e-14 | Angomonas deanei |
| A0A0D6L8T9_9BILA/1-99 | 1.1e-14 | Ancylostoma ceylanicum |
| A5DIB3_PICGU/144-327 | 1.4e-14 | Meyerozyma guilliermondii |
| G0UY67_TRYCI/264-396 | 1.4e-14 | Trypanosoma congolense |
| G0MIL5_CAEBE/25-192 | 1.2e-14 | Caenorhabditis brenneri |
| Q9XU14_CAEEL/39-195 | 1.5e-14 | Caenorhabditis elegans |
| V5APL8_TRYCR/1864-2025 | 1.7e-14 | Trypanosoma cruzi Dm28c |
| A0A0D6N892_9PROT/133-327 | 2.9e-14 | Acetobacter cibinongensis 4H-1 |
| Q5DDE4_SCHJA/124-201 | 1.4e-14 | Schistosoma japonicum |
| V5B9J9_TRYCR/337-498 | 2.0e-14 | Trypanosoma cruzi Dm28c |
| A0A067NW38_PLEOS/177-427 | 3.7e-14 | Pleurotus ostreatus PC15 |
| A0A0A1TDP9_9HYPO/289-501 | 2.5e-14 | Torrubiella hemipterigena |
| D6WGT0_TRICA/23-82 | 5.2e-09 | Tribolium castaneum |
| K4DTQ1_TRYCR/513-668 | 3.2e-14 | Trypanosoma cruzi |
| S9TW61_9TRYP/1501-1707 | 3.4e-14 | Angomonas deanei |
| Q8YZT8_NOSS1/277-470 | 3.9e-14 | Nostoc sp. |
| A0A091MZT6_9PASS/9-57 | 4.0e-14 | Acanthisitta chloris |
| A0A077X0I7_9FUNG/3-79 | 3.0e-14 | Absidia idahoensis var. thermophila |
| A4HNT8_LEIBR/503-658 | 3.5e-14 | Leishmania braziliensis |
| U6LVU4_9EIME/320-386 | 4.9e-06 | Eimeria brunetti |
| A0A021VXY7_9GAMM/21-247 | 4.0e-14 | Lysobacter capsici AZ78 |
| A0A088S1H1_9TRYP/506-722 | 4.4e-14 | Leishmania panamensis |
| Q4DIZ1_TRYCC/619-797 | 5.5e-14 | Trypanosoma cruzi |
| R1BXY7_EMIHU/418-598 | 7.1e-14 | Emiliania huxleyi CCMP1516 |
| R1C991_EMIHU/3-64 | 5.0e-14 | Emiliania huxleyi CCMP1516 |
| F9GBV4_FUSOF/37-171 | 8.5e-14 | Fusarium oxysporum |
| J4GUY7_FIBRA/192-236 | 0.00019 | Fibroporia radiculosa |
| A0A024K6X6_9MYCO/152-354 | 1.0e-13 | Mycobacterium triplex |
| Q95V12_ANOGA/2-52 | 8.6e-14 | Anopheles gambiae |
| G0U9Y6_TRYVY/988-1185 | 1.3e-13 | Trypanosoma vivax) |
| S9VQ57_9TRYP/186-327 | 1.3e-13 | Strigomonas culicis |
| V8N445_OPHHA/2-58 | 8.1e-14 | Ophiophagus hannah |
| G9KQL5_MUSPF/2-61 | 1.6e-13 | Mustela putorius furo |
| W9ZW74_FUSOX/40-158 | 2.0e-13 | Fusarium oxysporum f. sp. melonis 26406 |
| T1EIJ8_HELRO/1-78 | 9.5e-14 | Helobdella robusta |
| X0CE33_FUSOX/40-158 | 2.2e-13 | Fusarium oxysporum f. sp. raphani 54005 |
| A0A081HTZ4_9MYCO/152-353 | 1.7e-13 | Mycobacterium sp. TKK-01-0059 |
| X0GL90_FUSOX/23-93 | 1.9e-13 | Fusarium oxysporum f. sp. conglutinans race 2 54008 |
| A0A060W7F3_ONCMY/94-164 | 1.3e-13 | Oncorhynchus mykiss |
| R5SIG1_9GAMM/237-428 | 2.3e-13 | Acinetobacter sp. CAG:196 |
| E9ASJ8_LEIMU/447-662 | 2.1e-13 | Leishmania mexicana |
| S9UW00_9TRYP/325-466 | 2.7e-13 | Strigomonas culicis |
| I7MCV6_TETTS/341-480 | 3.8e-13 | Tetrahymena thermophila |
| A0A016WCI5_9BILA/14-63 | 3.0e-13 | Ancylostoma ceylanicum |
| H2YUD7_CIOSA/6-79 | 3.9e-13 | Ciona savignyi |
| A0A087ZUG9_APIME/78-210 | 5.8e-13 | Apis mellifera |
| A0A087ZUH1_APIME/4-148 | 7.0e-13 | Apis mellifera |
| E9IBY9_SOLIN/1-95 | 6.7e-13 | Solenopsis invicta |
| B7Q089_IXOSC/8-56 | 9.9e-13 | Ixodes scapularis |
| K9UIG2_9CHRO/916-1120 | 1.4e-12 | Chamaesiphon minutus PCC 6605 |
| A0A091MSH3_9PASS/2-86 | 9.0e-13 | Acanthisitta chloris |
| G2R1E5_THITE/193-422 | 1.5e-12 | Thielavia terrestris |
| N4UPM5_FUSC1/4-134 | 1.3e-12 | Fusarium oxysporum f. sp. cubense |
| W9ZAA6_FUSOX/4-134 | 1.3e-12 | Fusarium oxysporum f. sp. melonis 26406 |
| A0A060ZA22_ONCMY/9-92 | 1.1e-12 | Oncorhynchus mykiss |
| C5KJ09_PERM5/7-93 | 1.3e-12 | Perkinsus marinus |
| A0A0A9W6H5_LYGHE/72-197 | 5.1e-12 | Lygus hesperus |
| K7EC16_ORNAN/51-105 | 2.7e-12 | Ornithorhynchus anatinus |
| A0A0C9Y2S1_9AGAR/18-84 | 2.6e-12 | Laccaria amethystina LaAM-08-1 |
| R1DC85_EMIHU/38-153 | 5.9e-12 | Emiliania huxleyi CCMP1516 |
| R1CM45_EMIHU/27-142 | 5.9e-12 | Emiliania huxleyi CCMP1516 |
| W8BL67_CERCA/2-49 | 4.1e-12 | Ceratitis capitata |
| A0A0D6LPR0_9BILA/30-118 | 4.1e-12 | Ancylostoma ceylanicum |
| J0NQ88_9ACTO/123-356 | 1.8e-10 | Actinomyces massiliensis F0489 |
| G3VXN5_SARHA/75-149 | 6.8e-12 | Sarcophilus harrisii |
| F1KXD5_ASCSU/2-48 | 6.2e-12 | Ascaris suum |
| G3TTC4_LOXAF/77-146 | 5.8e-12 | Loxodonta africana |
| J3PJS9_GAGT3/13-75 | 1.8e-11 | Gaeumannomyces graminis var. tritici |
| S9WAE2_9CETA/97-155 | 7.3e-08 | Camelus ferus |
| A0A0C1VPW9_9ACTN/95-320 | 1.2e-11 | Streptomyces sp. RSD-27 |
| A0A0B7B3P9_9EUPU/53-123 | 9.6e-12 | Arion vulgaris |
| W8AVQ7_CERCA/2-49 | 1.2e-11 | Ceratitis capitata |
| A8XRN0_CAEBR/18-133 | 9.9e-12 | Caenorhabditis briggsae |
| A0A069SWY4_9MICO/43-226 | 1.2e-11 | Dermabacter hominis 1368 |
| S3XXB2_9MICO/43-226 | 1.2e-11 | Dermabacter sp. HFH0086 |
| T1IAN4_RHOPR/1-91 | 1.4e-11 | Rhodnius prolixus |
| W9N8Y5_FUSOX/32-101 | 1.1e-11 | Fusarium oxysporum f. sp. pisi HDV247 |
| A4X268_SALTO/3389-3630 | 2.0e-11 | Salinispora tropica |
| A0A0B1S1L9_OESDE/1-47 | 1.3e-11 | Oesophagostomum dentatum |
| A0A0D6LWK2_9BILA/1-47 | 2.0e-11 | Ancylostoma ceylanicum |
| J9AGP8_WUCBA/1-45 | 1.5e-11 | Wuchereria bancrofti |
| Q5C763_SCHJA/7-63 | 2.0e-11 | Schistosoma japonicum |
| A0A060YG45_ONCMY/12-59 | 2.8e-11 | Oncorhynchus mykiss |
| F9FKH2_FUSOF/153-231 | 3.1e-11 | Fusarium oxysporum |
| A0A0D6LIX0_9BILA/50-108 | 2.7e-11 | Ancylostoma ceylanicum |
| Z9JNI9_9MICO/64-236 | 3.7e-11 | Brachybacterium phenoliresistens |
| A0A022KQ68_9MICO/74-246 | 3.4e-11 | Brachybacterium muris UCD-AY4 |
| G8BVV5_TETPH/117-291 | 3.9e-11 | Tetrapisispora phaffii |
| A0A0B1RX60_OESDE/85-144 | 3.2e-11 | Oesophagostomum dentatum |
| F2TVF2_SALR5/5-57 | 3.3e-11 | Salpingoeca rosetta |
| S4PES3_9NEOP/2-152 | 4.6e-11 | Pararge aegeria |
| A0A0F0LS58_9MICO/216-401 | 1.7e-10 | Microbacterium azadirachtae |
| A0A087VWJ6_ECHMU/3-52 | 3.3e-11 | Echinococcus multilocularis |
| F9GAS2_FUSOF/110-177 | 4.2e-11 | Fusarium oxysporum |
| A0A060XHC2_ONCMY/48-100 | 4.4e-11 | Oncorhynchus mykiss |
| W8ASH9_CERCA/3-51 | 5.5e-11 | Ceratitis capitata |
| K0SSX4_THAOC/194-372 | 2.8e-10 | Thalassiosira oceanica |
| A0A060STP6_PYCCI/426-505 | 8.7e-11 | Trametes cinnabarina |
| T1F3L9_HELRO/245-298 | 8.1e-11 | Helobdella robusta |
| F2EA39_HORVD/60-124 | 5.0e-11 | Hordeum vulgare var. distichum |
| W7TAM7_9STRA/1-147 | 1.1e-10 | Nannochloropsis gaditana |
| E9PMC6_HUMAN/85-151 | 6.0e-11 | Homo sapiens |
| S9WFZ3_9CETA/51-99 | 7.6e-11 | Camelus ferus |
| F2E574_HORVD/71-135 | 6.1e-11 | Hordeum vulgare var. distichum |
| Q4F6Y1_9CETA/5-52 | 7.3e-11 | Bos taurus x Bos indicus |
| R6CRU8_9BACE/250-438 | 1.1e-10 | Bacteroides sp. CAG:530 |
| A0CGL4_PARTE/434-560 | 8.6e-10 | Paramecium tetraurelia |
| B0C8L1_ACAM1/259-357 | 0.00038 | Acaryochloris marina |
| Q4SWM1_TETNG/48-105 | 8.3e-11 | Tetraodon nigroviridis |
| B4DTS3_HUMAN/21-70 | 1.6e-10 | Homo sapiens |
| G0ZCG5_CAPHI/3-62 | 1.0e-10 | Capra hircus |
| S9W7J3_9CETA/13-83 | 1.5e-10 | Camelus ferus |
| J6F4Z9_TRIAS/118-256 | 3.1e-07 | Trichosporon asahii var. asahii |
| A0BPF7_PARTE/97-252 | 2.4e-10 | Paramecium tetraurelia |
| E1GN38_LOALO/243-305 | 1.8e-10 | Loa loa |
| S9VDC0_9TRYP/80-154 | 3.4e-10 | Strigomonas culicis |
| E3MW75_CAERE/95-160 | 1.9e-10 | Caenorhabditis remanei |
| B4E1R7_HUMAN/92-149 | 2.6e-10 | Homo sapiens |
| L9KQQ4_TUPCH/66-114 | 2.6e-10 | Tupaia chinensis |
| F6W2B7_CALJA/21-70 | 2.7e-10 | Callithrix jacchus |
| A0A0B7C1V8_9EUPU/1-57 | 2.1e-10 | Arion vulgaris |
| F7GHE2_CALJA/97-149 | 4.5e-10 | Callithrix jacchus |
| S9WCC2_9TRYP/180-444 | 3.8e-10 | Angomonas deanei |
| H0ENZ1_GLAL7/3-54 | 6.1e-10 | Glarea lozoyensis |
| E9PS73_HUMAN/51-96 | 3.5e-10 | Homo sapiens |
| H2AQZ2_KAZAF/110-311 | 5.2e-10 | Kazachstania africana |
| E5XTT9_9ACTN/68-303 | 2.5e-08 | Segniliparus rugosus ATCC BAA-974 |
| S9VD17_9TRYP/11-59 | 4.3e-10 | Angomonas deanei |
| M2R423_CERS8/165-286 | 1.0e-08 | Ceriporiopsis subvermispora |
| K2E4M6_9BACT/296-520 | 1.4e-09 | uncultured bacterium |
| S9U7A3_9TRYP/298-561 | 7.2e-10 | Angomonas deanei |
| E5XTU0_9ACTN/162-406 | 5.1e-09 | Segniliparus rugosus ATCC BAA-974 |
| A0DVI7_PARTE/391-560 | 8.1e-09 | Paramecium tetraurelia |
| A0A0B2VTU4_TOXCA/249-308 | 7.0e-10 | Toxocara canis |
| A0A0C1VZ57_9ACTN/73-318 | 1.4e-08 | Streptomyces sp. RSD-27 |
| A0A095B3V1_SCHHA/84-219 | 1.0e-09 | Schistosoma haematobium |
| E9PLC9_HUMAN/85-144 | 9.2e-10 | Homo sapiens |
| F9WB74_TRYCI/1338-1446 | 1.8e-09 | Trypanosoma congolense |
| S9V419_9TRYP/68-199 | 1.4e-09 | Angomonas deanei |
| A0A078Q043_BACFG/277-465 | 1.7e-09 | Bacteroides fragilis |
| I9SHI3_BACOV/277-465 | 1.7e-09 | Bacteroides ovatus CL02T12C04 |
| A0A078PQ82_BACOV/277-465 | 1.7e-09 | Bacteroides ovatus |
| I8Z374_BACOV/277-465 | 1.7e-09 | Bacteroides ovatus CL03T12C18 |
| A7LTS0_BACO1/277-465 | 1.7e-09 | Bacteroides ovatus |
| A0E0J2_PARTE/386-587 | 1.1e-09 | Paramecium tetraurelia |
| F1LLU4_HUMAN/71-137 | 1.5e-09 | Homo sapiens |
| U3J4D6_ANAPL/160-265 | 2.1e-09 | Anas platyrhynchos |
| A0A060Y878_ONCMY/186-300 | 2.2e-09 | Oncorhynchus mykiss |
| A0DBL1_PARTE/414-553 | 2.4e-08 | Paramecium tetraurelia |
| G7XAP6_ASPKW/1-51 | 2.7e-09 | Aspergillus kawachii |
| R7V915_CAPTE/19-69 | 2.0e-09 | Capitella teleta |
| A0A076FI00_9PHYC/107-314 | 2.1e-09 | Aureococcus anophagefferens virus |
| U3KF62_FICAL/174-277 | 4.5e-09 | Ficedula albicollis |
| I7M4H0_TETTS/193-406 | 6.4e-09 | Tetrahymena thermophila |
| E9ARP3_LEIMU/258-430 | 3.4e-09 | Leishmania mexicana |
| H0ZKF9_TAEGU/143-243 | 4.6e-09 | Taeniopygia guttata |
| W6L0Z2_9TRYP/507-636 | 3.6e-09 | Phytomonas sp. isolate Hart1 |
| K3WE11_PYTUL/114-169 | 4.0e-09 | Pythium ultimum DAOM BR144 |
| A0A0D7AGW8_9AGAR/180-348 | 7.8e-09 | Fistulina hepatica ATCC 64428 |
| R0LHN9_ANAPL/142-246 | 6.3e-09 | Anas platyrhynchos |
| A0A0D8JB70_9BACT/320-516 | 1.6e-08 | Draconibacterium sp. JN14CK-3 |
| A7RWT3_NEMVE/216-337 | 7.0e-09 | Nematostella vectensis |
| W2EP22_9ACTN/257-466 | 9.1e-09 | Microbispora sp. ATCC PTA-5024 |
| C4Y0U3_CLAL4/257-421 | 2.6e-06 | Clavispora lusitaniae |
| D4YQ94_9MICO/104-303 | 1.3e-08 | Brevibacterium mcbrellneri ATCC 49030 |
| E4XMI3_OIKDI/1-48 | 8.2e-09 | Oikopleura dioica |
| A0A0D0D1C6_9HOMO/1-91 | 1.8e-08 | Paxillus rubicundulus Ve08.2h10 |
| K7G3G5_PELSI/54-160 | 1.4e-08 | Pelodiscus sinensis |
| W5NGY4_LEPOC/165-265 | 1.5e-08 | Lepisosteus oculatus |
| W5NGY5_LEPOC/169-269 | 1.4e-08 | Lepisosteus oculatus |
| R7VUY7_COLLI/19-63 | 8.8e-09 | Columba livia |
| D0WL64_9ACTO/185-415 | 6.4e-08 | Actinomyces sp. oral taxon 848 |
| X0L4I8_FUSOX/7-80 | 1.8e-08 | Fusarium oxysporum f. sp. vasinfectum 25433 |
| W5LJL4_ASTMX/69-137 | 1.2e-08 | Astyanax mexicanus |
| K1VGN3_TRIAC/122-256 | 1.5e-06 | Trichosporon asahii var. asahii |
| F1QQ89_DANRE/180-284 | 2.5e-08 | Danio rerio |
| CAN2_RABIT/2-48 | 1.4e-08 | Oryctolagus cuniculus |
| G0VDP6_NAUCC/122-284 | 2.5e-08 | Naumovozyma castellii |
| B3RXT0_TRIAD/189-305 | 3.9e-08 | Trichoplax adhaerens |
| R7TIL3_CAPTE/77-181 | 2.4e-08 | Capitella teleta |
| K9WTN8_9NOST/250-390 | 2.9e-08 | Cylindrospermum stagnale PCC 7417 |
| E8NHK8_LEIMU/10-58 | 2.8e-08 | Leishmania mexicana |
| E9PJJ3_HUMAN/85-135 | 2.5e-08 | Homo sapiens |
| Q4QDT6_LEIMA/269-443 | 3.4e-08 | Leishmania major |
| E9BDT7_LEIDB/258-434 | 3.8e-08 | Leishmania donovani |
| A4HJ21_LEIBR/373-516 | 3.8e-08 | Leishmania braziliensis |
| Q38PP0_PIG/31-75 | 2.6e-08 | Sus scrofa |
| W6LH56_9TRYP/486-563 | 5.1e-08 | Phytomonas sp. isolate Hart1 |
| G0QPP2_ICHMG/9-90 | 3.4e-06 | Ichthyophthirius multifiliis |
| M1EFU3_MUSPF/10-56 | 4.7e-08 | Mustela putorius furo |
| A0A088RZC5_9TRYP/373-522 | 5.5e-08 | Leishmania panamensis |
| A0A0C1V9C7_9ACTN/513-773 | 1.2e-06 | Streptomyces sp. RSD-27 |
| F1NBD8_CHICK/213-315 | 1.0e-07 | Gallus gallus |
| A4HXY0_LEIIN/258-434 | 6.5e-08 | Leishmania infantum |
| T1HF49_RHOPR/208-312 | 5.9e-08 | Rhodnius prolixus |
| H2TD54_TAKRU/137-236 | 7.8e-08 | Takifugu rubripes |
| F4P4B4_BATDJ/350-443 | 1.1e-07 | Batrachochytrium dendrobatidis |
| H3D3Y2_TETNG/131-267 | 7.6e-08 | Tetraodon nigroviridis |
| A0A060YAB6_ONCMY/25-85 | 1.4e-07 | Oncorhynchus mykiss |
| H2KV66_CLOSI/152-264 | 9.8e-08 | Clonorchis sinensis |
| F7CM89_HORSE/57-164 | 9.8e-08 | Equus caballus |
| G8ZXP7_TORDC/118-299 | 1.7e-07 | Torulaspora delbrueckii |
| A2DNH6_TRIVA/58-281 | 1.4e-07 | Trichomonas vaginalis |
| A7TH25_VANPO/117-291 | 1.3e-07 | Vanderwaltozyma polyspora |
| W4YTH0_STRPU/2-81 | 9.3e-08 | Strongylocentrotus purpuratus |
| G7P503_MACFA/193-297 | 1.7e-07 | Macaca fascicularis |
| Q0Q4H3_PIG/60-102 | 1.1e-07 | Sus scrofa |
| V4A3A1_LOTGI/282-390 | 2.7e-07 | Lottia gigantea |
| E6R7X0_CRYGW/48-240 | 2.1e-07 | Cryptococcus gattii serotype B |
| A0A0D0YN09_CRYGA/49-241 | 2.0e-07 | Cryptococcus gattii IND107 |
| D0WLL2_9ACTO/195-414 | 3.9e-07 | Actinomyces sp. oral taxon 848 |
| A5PMP0_DANRE/1-41 | 1.6e-07 | Danio rerio |
| C7MDV9_BRAFD/58-230 | 1.9e-07 | Brachybacterium faecium |
| A0A0D0UBM6_CRYGA/50-241 | 2.3e-07 | Cryptococcus gattii CA1280 |
| K1QU20_CRAGI/282-436 | 3.0e-07 | Crassostrea gigas |
| W0IVT3_9BACT/151-364 | 2.8e-07 | Opitutaceae bacterium TAV5 |
| I6AT95_9BACT/151-364 | 2.8e-07 | Opitutaceae bacterium TAV1 |
| H2ZX85_LATCH/179-286 | 2.1e-07 | Latimeria chalumnae |
| A4I6K6_LEIIN/260-394 | 4.0e-07 | Leishmania infantum |
| E9BMG7_LEIDB/260-394 | 4.0e-07 | Leishmania donovani |
| A0A0D2L9N7_CRYGA/48-240 | 3.1e-07 | Cryptococcus gattii E566 |
| A0A0D0WI91_CRYGA/48-240 | 3.1e-07 | Cryptococcus gattii EJB2 |
| A0A0D2TVE2_CRYGA/48-240 | 3.1e-07 | Cryptococcus gattii NT-10 |
| A0A0D0W4B6_CRYGA/48-240 | 3.1e-07 | Cryptococcus gattii Ru294 |
| A0A0B7A934_9EUPU/1-41 | 3.5e-07 | Arion vulgaris |
| F6TEA5_MACMU/193-297 | 3.1e-07 | Macaca mulatta |
| E2L7L8_MONPE/110-160 | 2.4e-07 | Moniliophthora perniciosa |
| G3PCQ6_GASAC/185-285 | 4.0e-07 | Gasterosteus aculeatus |
| C7MGJ8_BRAFD/51-183 | 3.9e-07 | Brachybacterium faecium |
| A8J614_CHLRE/125-236 | 3.5e-07 | Chlamydomonas reinhardtii |
| A0A0B7A8H6_9EUPU/1-41 | 4.2e-07 | Arion vulgaris |
| H2MGL6_ORYLA/123-236 | 3.7e-07 | Oryzias latipes |
| PALB_CANGA/127-298 | 3.6e-07 | Candida glabrata |
| A0A0D0WFV7_CRYGA/50-241 | 4.5e-07 | Cryptococcus gattii CA1873 |
| G7MQ65_MACMU/193-297 | 9.5e-07 | Macaca mulatta |
| D5SQZ8_PLAL2/133-353 | 5.2e-07 | Planctopirus limnophilus |
| K9ESX5_9CYAN/285-455 | 5.1e-07 | Leptolyngbya sp. PCC 7375 |
| U6GQB4_9EIME/375-488 | 6.0e-07 | Eimeria praecox |
| A0A0D0WBH9_CRYGA/49-241 | 6.2e-07 | Cryptococcus gattii CA1014 |
| A0A095EF78_CRYGA/49-241 | 6.2e-07 | Cryptococcus gattii R265 |
| M5RCT1_9PLAN/383-594 | 5.7e-07 | Rhodopirellula maiorica SM1 |
| S9TNC6_9TRYP/383-539 | 6.9e-07 | Angomonas deanei |
| S3X0F9_9ACTN/193-380 | 5.9e-07 | Propionibacterium sp. oral taxon 192 |
| A0A095A4V2_SCHHA/90-153 | 5.3e-07 | Schistosoma haematobium |
| Z9JR51_9MICO/155-343 | 8.9e-07 | Brachybacterium phenoliresistens |
| A0A0D0UUM8_CRYGA/49-241 | 8.1e-07 | Cryptococcus gattii Ram5 |
| A0A0D0S8H0_CRYGA/49-241 | 8.1e-07 | Cryptococcus gattii LA55 |
| A0A0D0ZI08_CRYGA/49-241 | 8.1e-07 | Cryptococcus gattii CBS 10090 |
| A0A0D0XWA2_CRYGA/49-241 | 8.1e-07 | Cryptococcus gattii 2001/935-1 |
| A0A0D0ULY6_CRYGA/49-241 | 8.1e-07 | Cryptococcus gattii MMRL2647 |
| A0A0D2LVZ0_CRYGA/49-241 | 8.1e-07 | Cryptococcus gattii 99/473 |
| W6MJ04_9ASCO/180-285 | 1.1e-06 | Kuraishia capsulata CBS 1993 |
| H0UYD0_CAVPO/195-297 | 1.1e-06 | Cavia porcellus |
| A0A0D9RX50_CHLSB/168-271 | 2.1e-06 | Chlorocebus sabaeus |
| J7S2I1_KAZNA/142-299 | 1.0e-06 | Kazachstania naganishii |
| Q4Q6M2_LEIMA/260-367 | 9.9e-07 | Leishmania major |
| A0A084WIZ9_9DIPT/3-80 | 4.5e-05 | Anopheles sinensis |
| F6ZV88_HORSE/184-289 | 1.3e-06 | Equus caballus |
| A7T9G8_NEMVE/58-126 | 1.3e-06 | Nematostella vectensis |
| H2PKJ4_PONAB/193-296 | 1.7e-06 | Pongo abelii |
| A0A0B7A6R8_9EUPU/56-114 | 1.1e-06 | Arion vulgaris |
| Q55R52_CRYNB/48-240 | 1.7e-06 | Cryptococcus neoformans var. neoformans |
| V8PE73_OPHHA/187-290 | 1.8e-06 | Ophiophagus hannah |
| A0A093I2E1_FULGA/2-83 | 1.3e-06 | Fulmarus glacialis |
| A0A096N5B5_PAPAN/193-296 | 3.2e-06 | Papio anubis |
| F1P8C4_CANFA/172-279 | 4.9e-06 | Canis familiaris |
| F7HE19_CALJA/193-296 | 3.4e-06 | Callithrix jacchus |
| A0A0B7F6Y0_THACB/2-104 | 1.7e-06 | Thanatephorus cucumeris |
| C5DQ17_ZYGRC/115-277 | 1.8e-06 | Zygosaccharomyces rouxii |
| I1E773_AMPQE/3-71 | 1.8e-06 | Amphimedon queenslandica |
| G1KGC3_ANOCA/194-297 | 4.7e-06 | Anolis carolinensis |
| E9B1J2_LEIMU/261-368 | 2.0e-06 | Leishmania mexicana |
| W6KUK8_9TRYP/4658-4813 | 2.6e-06 | Phytomonas sp. isolate EM1 |
| E9J2A0_SOLIN/6-86 | 1.6e-06 | Solenopsis invicta |
| U1RF94_9ACTO/199-396 | 3.4e-06 | Actinomyces sp. oral taxon 172 |
| W5L4R7_ASTMX/190-288 | 3.5e-06 | Astyanax mexicanus |
| A0A091E4C4_FUKDA/193-295 | 3.1e-06 | Fukomys damarensis |
| A0A087YNX6_POEFO/174-276 | 3.3e-06 | Poecilia formosa |
| W4YCA8_STRPU/194-297 | 3.0e-06 | Strongylocentrotus purpuratus |
| D8UHG2_VOLCA/162-254 | 2.7e-06 | Volvox carteri |
| G1P2N2_MYOLU/169-278 | 3.4e-06 | Myotis lucifugus |
| B3E5A8_GEOLS/126-326 | 5.0e-06 | Geobacter lovleyi |
| Q5DDD0_SCHJA/57-161 | 2.3e-06 | Schistosoma japonicum |
| F1S738_PIG/164-269 | 5.1e-06 | Sus scrofa |
| G1U3K5_RABIT/195-299 | 5.0e-06 | Oryctolagus cuniculus |
| A0A088RML9_9TRYP/277-394 | 3.6e-06 | Leishmania panamensis |
| G3QFI1_GORGO/193-295 | 9.2e-06 | Gorilla gorilla gorilla |
| G1LHD6_AILME/164-271 | 4.7e-06 | Ailuropoda melanoleuca |
| ADGB_HUMAN/193-295 | 9.2e-06 | Homo sapiens |
| K1W5U8_TRIAC/238-335 | 7.1e-06 | Trichosporon asahii var. asahii |
| Q7NDU2_GLOVI/395-588 | 2.5e-05 | Gloeobacter violaceus |
| H2RDH8_PANTR/193-295 | 9.2e-06 | Pan troglodytes |
| H3HDN3_PHYRM/280-341 | 7.1e-06 | Phytophthora ramorum |
| D3YZ77_MOUSE/193-295 | 5.7e-06 | Mus musculus |
| L7N048_CANFA/188-295 | 6.7e-06 | Canis familiaris |
| J4U728_TRIAS/239-335 | 7.9e-06 | Trichosporon asahii var. asahii |
| G5AEU5_PHYSP/314-371 | 5.8e-06 | Phytophthora sojae |
| A0A0C1WY78_9CYAN/50-192 | 5.2e-06 | Scytonema millei VB511283 |
| I3NC29_SPETR/168-270 | 7.5e-06 | Spermophilus tridecemlineatus |
| M3Z2Y8_MUSPF/192-296 | 8.4e-06 | Mustela putorius furo |
| D4U224_9ACTO/169-370 | 7.5e-06 | Actinomyces odontolyticus F0309 |
| G3MXR8_BOVIN/191-298 | 1.1e-05 | Bos taurus |
| A2DDC2_TRIVA/512-623 | 6.9e-06 | Trichomonas vaginalis |
| L8IPS4_9CETA/191-298 | 1.1e-05 | Bos mutus |
| G1RZ48_NOMLE/193-295 | 1.3e-05 | Nomascus leucogenys |
| G1RZ50_NOMLE/193-295 | 1.3e-05 | Nomascus leucogenys |
| D2HKF2_AILME/167-274 | 7.6e-06 | Ailuropoda melanoleuca |
| D3ZNZ6_RAT/168-271 | 1.1e-05 | Rattus norvegicus |
| F1B3B2_PIG/2-37 | 4.7e-06 | Sus scrofa |
| M3ZL87_XIPMA/175-276 | 2.1e-05 | Xiphophorus maculatus |
| A0A0C1WZA7_9CYAN/42-115 | 5.4e-06 | Scytonema millei VB511283 |
| G1LHD2_AILME/193-300 | 8.6e-06 | Ailuropoda melanoleuca |
| J3J6J0_9ACTN/199-397 | 1.5e-05 | Atopobium sp. ICM58 |
| ADGB_MOUSE/193-295 | 1.7e-05 | Mus musculus |
| M1WH15_CLAP2/111-181 | 8.0e-06 | Claviceps purpurea |
| F1LYE2_RAT/168-271 | 1.1e-05 | Rattus norvegicus |
| J3QMP3_MOUSE/193-295 | 1.8e-05 | Mus musculus |
| L1I7V8_GUITH/179-235 | 1.3e-05 | Guillardia theta CCMP2712 |
| H2ZJY1_CIOSA/141-196 | 1.7e-05 | Ciona savignyi |
| D0WP73_9ACTO/185-423 | 2.2e-05 | Actinomyces sp. oral taxon 848 |
| G6DC51_DANPL/242-329 | 1.2e-05 | Danaus plexippus |
| J9ED26_WUCBA/140-188 | 9.1e-06 | Wuchereria bancrofti |
| W2R551_PHYPN/250-307 | 1.6e-05 | Phytophthora parasitica |
| W2KJ42_PHYPR/250-307 | 1.6e-05 | Phytophthora parasitica |
| H1VA18_COLHI/10-53 | 8.2e-06 | Colletotrichum higginsianum |
| H0YC38_HUMAN/6-61 | 9.1e-06 | Homo sapiens |
| F6RUJ9_MONDO/188-303 | 1.6e-05 | Monodelphis domestica |
| E9PQZ4_HUMAN/139-179 | 1.1e-05 | Homo sapiens |
| W2R4J6_PHYPN/348-405 | 1.7e-05 | Phytophthora parasitica |
| W2ICK6_PHYPR/348-405 | 1.7e-05 | Phytophthora parasitica |
| W2KJ93_PHYPR/348-405 | 1.7e-05 | Phytophthora parasitica |
| W2R2Q3_PHYPN/393-450 | 1.8e-05 | Phytophthora parasitica |
| W2R2I1_PHYPN/398-455 | 1.8e-05 | Phytophthora parasitica |
| A4I9J8_LEIIN/564-691 | 1.5e-05 | Leishmania infantum |
| V5HW68_IXORI/23-71 | 1.3e-05 | Ixodes ricinus |
| E9BQI4_LEIDB/564-691 | 1.5e-05 | Leishmania donovani |
| G7YII1_CLOSI/7-70 | 3.0e-05 | Clonorchis sinensis |
| E9PLX0_HUMAN/85-122 | 1.2e-05 | Homo sapiens |
| H2VCU5_TAKRU/130-227 | 1.7e-05 | Takifugu rubripes |
| W5NW73_SHEEP/191-294 | 2.2e-05 | Ovis aries |
| W5NW72_SHEEP/191-294 | 2.3e-05 | Ovis aries |
| D0MYY1_PHYIT/336-392 | 2.0e-05 | Phytophthora infestans |
| A4I7R3_LEIIN/335-398 | 2.0e-05 | Leishmania infantum |
| Q4Q5I5_LEIMA/334-397 | 2.1e-05 | Leishmania major |
| E2C2Q3_HARSA/1-54 | 1.2e-05 | Harpegnathos saltator |
| E9BNK5_LEIDB/335-398 | 2.1e-05 | Leishmania donovani |
| E9B2M1_LEIMU/334-397 | 2.2e-05 | Leishmania mexicana |
| A4H9L6_LEIBR/276-394 | 1.9e-05 | Leishmania braziliensis |
| G0WFC4_NAUDC/140-356 | 2.4e-05 | Naumovozyma dairenensis |
| I2H065_TETBL/126-296 | 2.1e-05 | Tetrapisispora blattae |
| Q4Q3I0_LEIMA/567-694 | 2.5e-05 | Leishmania major |
| A0A0D2JYV0_9CHLO/162-288 | 2.2e-05 | Monoraphidium neglectum |
| J9EF37_9SPIT/2-167 | 2.0e-05 | Oxytricha trifallax |
| A9UVD6_MONBE/159-219 | 2.6e-05 | Monosiga brevicollis |
| F0VPI3_NEOCL/285-422 | 2.8e-05 | Neospora caninum |
| R7KK21_9BACE/2-189 | 2.1e-05 | Bacteroides thetaiotaomicron CAG:40 |
| K3WRR6_PYTUL/380-436 | 3.0e-05 | Pythium ultimum DAOM BR144 |
| T1FQ38_HELRO/309-417 | 3.9e-05 | Helobdella robusta |
| Q4SPK4_TETNG/1-46 | 1.9e-05 | Tetraodon nigroviridis |
| W4GWQ1_9STRA/342-426 | 3.2e-05 | Aphanomyces astaci |
| D0WP79_9ACTO/188-406 | 8.0e-05 | Actinomyces sp. oral taxon 848 |
| I1G9S5_AMPQE/174-257 | 2.9e-05 | Amphimedon queenslandica |
| W2WCS5_PHYPR/250-304 | 4.8e-05 | Phytophthora parasitica CJ01A1 |
| Z4X6H7_9ACTO/203-404 | 4.8e-05 | Actinomyces sp. ICM54 |
| J9AIH0_WUCBA/1-66 | 2.6e-05 | Wuchereria bancrofti |
| W2YMQ7_PHYPR/398-452 | 5.1e-05 | Phytophthora parasitica P10297 |
| E4XLY5_OIKDI/117-219 | 6.3e-05 | Oikopleura dioica |
| W2G6M2_PHYPR/348-402 | 5.3e-05 | Phytophthora parasitica |
| W2WFB9_PHYPR/348-402 | 5.3e-05 | Phytophthora parasitica CJ01A1 |
| K2EF73_9BACT/305-514 | 8.2e-05 | uncultured bacterium |
| S6ENT7_ZYGB2/115-280 | 4.4e-05 | Zygosaccharomyces bailii |
| W0VYU5_ZYGBA/115-280 | 4.4e-05 | Zygosaccharomyces bailii ISA1307 |
| A0A0A9XXK1_LYGHE/328-429 | 4.7e-05 | Lygus hesperus |
| A0A080ZKI5_PHYPR/398-452 | 5.6e-05 | Phytophthora parasitica P1976 |
| V9EK15_PHYPR/398-452 | 5.6e-05 | Phytophthora parasitica P1569 |
| U1SPX1_9ACTO/197-416 | 6.8e-05 | Actinomyces sp. oral taxon 172 |
| A0A087WSM6_MOUSE/17-88 | 3.3e-05 | Mus musculus |
| C3XWK7_BRAFL/1-41 | 5.6e-05 | Branchiostoma floridae |
| J3J645_9ACTN/197-416 | 8.5e-05 | Atopobium sp. ICM58 |
| A0A093JHI3_EURHL/231-315 | 5.1e-05 | Eurypyga helias |
| B0DRE3_LACBS/39-117 | 3.9e-05 | Laccaria bicolor |
| A0A0C2DPP1_9BILA/41-99 | 4.0e-05 | Ancylostoma duodenale |
| A0CVP1_PARTE/123-238 | 7.7e-05 | Paramecium tetraurelia |
| R6UXV3_9BACE/265-453 | 6.6e-05 | Bacteroides faecis CAG:32 |
| A0D938_PARTE/157-268 | 7.9e-05 | Paramecium tetraurelia |
| C6ILT8_9BACE/265-453 | 6.6e-05 | Bacteroides sp. 1_1_6 |
| G1NIV2_MELGA/142-200 | 0.00014 | Meleagris gallopavo |
| Q8A0R1_BACTN/275-463 | 6.9e-05 | Bacteroides thetaiotaomicron |
| R9H254_BACT4/275-463 | 6.9e-05 | Bacteroides thetaiotaomicron dnLKV9 |
| D7IBA4_9BACE/280-468 | 7.0e-05 | Bacteroides sp. 1_1_14 |
| W4FF75_9STRA/210-320 | 7.7e-05 | Aphanomyces astaci |
| A0A074SLJ6_9APIC/288-422 | 6.5e-05 | Hammondia hammondi |
| W4FH65_9STRA/210-320 | 8.1e-05 | Aphanomyces astaci |
| W4FGV5_9STRA/210-320 | 7.9e-05 | Aphanomyces astaci |
| G0U8U1_TRYVY/345-461 | 7.5e-05 | Trypanosoma vivax |
| E9B4J6_LEIMU/563-690 | 8.2e-05 | Leishmania mexicana |
| W4FF15_9STRA/210-320 | 9.1e-05 | Aphanomyces astaci |
| W4FF71_9STRA/210-320 | 9.4e-05 | Aphanomyces astaci |
| A0A086NQC6_METAN/126-190 | 9.0e-05 | Metarhizium anisopliae |
| V5IDE7_IXORI/1-47 | 7.7e-05 | Ixodes ricinus |
| I7LVL5_TETTS/277-395 | 0.00012 | Tetrahymena thermophila |
| W4FGZ1_9STRA/210-320 | 9.3e-05 | Aphanomyces astaci |
| W4FGV1_9STRA/210-320 | 9.5e-05 | Aphanomyces astaci |
| A0A0C9T8Z5_9HOMO/68-122 | 6.8e-05 | Sphaerobolus stellatus SS14 |
| E9E9C4_METAQ/126-190 | 0.00011 | Metarhizium acridum |
| S9Y9L0_9CETA/135-192 | 0.00011 | Camelus ferus |
| U1RY82_9ACTO/198-351 | 0.00015 | Actinomyces sp. oral taxon 172 |
| U6LNI4_9EIME/1616-1728 | 0.00019 | Eimeria brunetti |
| A0A088RN60_9TRYP/529-649 | 0.00015 | Leishmania panamensis |
| E5C759_9BACE/275-463 | 0.00015 | Bacteroides sp. D2 |
| F0VZJ4_9STRA/110-167 | 0.0002 | Albugo laibachii Nc14 |
| A4HAE6_LEIBR/573-693 | 0.00016 | Leishmania braziliensis |
| A0A074Z6G0_9TREM/79-176 | 0.00015 | Opisthorchis viverrini |
| F7CA68_ORNAN/83-140 | 0.00014 | Ornithorhynchus anatinus |
| A0A0A8LBW2_9SACH/136-308 | 0.00023 | Kluyveromyces dobzhanskii CBS 2104 |
| A0A0F5D4R9_PRIPA/2-47 | 0.00022 | Pristionchus pacificus |
| I7M6I8_TETTS/196-336 | 0.00024 | Tetrahymena thermophila |
| A0A088S0B4_9TRYP/335-394 | 0.00024 | Leishmania panamensis |
| A4HK78_LEIBR/335-394 | 0.00024 | Leishmania braziliensis |
| R5SV90_9GAMM/21-252 | 0.0003 | Acinetobacter sp. CAG:196 |
| F7ZZH9_CELGA/168-377 | 0.00045 | Cellvibrio gilvus |
| E3NM48_CAERE/2-71 | 0.00018 | Caenorhabditis remanei |
| S8G8F8_TOXGO/288-423 | 0.00025 | Toxoplasma gondii ME49 |
| A0A086PNT3_TOXGO/288-423 | 0.00025 | Toxoplasma gondii MAS |
| B9Q3H4_TOXGO/288-423 | 0.00025 | Toxoplasma gondii |
| A0A086KTY9_TOXGO/288-423 | 0.00025 | Toxoplasma gondii GAB2-2007-GAL-DOM2 |
| S7V1W1_TOXGO/288-423 | 0.00025 | Toxoplasma gondii GT1 |
| A0A086J994_TOXGO/288-423 | 0.00025 | Toxoplasma gondii p89 |
| A0A086JLE5_TOXGO/288-423 | 0.00025 | Toxoplasma gondii FOU |
| A0A086PXA3_TOXGO/288-423 | 0.00025 | Toxoplasma gondii VAND |
| A0A086LJL9_TOXGO/288-423 | 0.00025 | Toxoplasma gondii RUB |
| K2A763_9BACT/439-666 | 0.00039 | uncultured bacterium |
| G4VSD9_SCHMA/279-380 | 0.00038 | Schistosoma mansoni |
| T0QRC4_9STRA/336-397 | 0.00029 | Saprolegnia diclina VS20 |
| A0A0B6YXU4_9EUPU/221-275 | 0.00032 | Arion vulgaris |
| R6S676_9BACE/261-449 | 0.00032 | Bacteroides finegoldii CAG:203 |
| A0A0B6YYG8_9EUPU/215-269 | 0.00031 | Arion vulgaris |
| A0A024U8D7_9STRA/347-412 | 0.00035 | Aphanomyces invadans |
| H7C2P1_HUMAN/1-41 | 0.00025 | Homo sapiens |
| C1EGZ2_MICSR/183-280 | 0.00045 | Micromonas sp.) |
| V5B134_TRYCR/280-336 | 0.00038 | Trypanosoma cruzi Dm28c |
| W0VUG6_ZYGBA/114-278 | 0.00046 | Zygosaccharomyces bailii ISA1307 |
| T1J9C4_STRMM/126-183 | 0.00028 | Strigamia maritima |
| G3I7B4_CRIGR/113-169 | 0.00057 | Cricetulus griseus |
| E0VX93_PEDHC/202-292 | 0.00094 | Pediculus humanus subsp. corporis |
| H6QU27_PUCGT/160-239 | 0.0005 | Puccinia graminis f. sp. tritici |
| A0A067CJK5_SAPPC/343-405 | 0.00068 | Saprolegnia parasitica CBS 223.65 |
| U6KXH6_EIMTE/60-163 | 0.00052 | Eimeria tenella |
| F2TY84_SALR5/186-294 | 0.00079 | Salpingoeca rosetta |
| A0A0C9WTL3_9AGAR/122-170 | 0.00052 | Laccaria amethystina LaAM-08-1 |
| G0QKC0_ICHMG/387-479 | 0.00099 | Ichthyophthirius multifiliis |
| A0A067BSQ0_SAPPC/93-150 | 0.00075 | Saprolegnia parasitica CBS 223.65 |
| E9K046_ANOGA/3-54 | 0.00072 | Anopheles gambiae S |
| E9JZY0_ANOGA/3-54 | 0.00098 | Anopheles gambiae M |
| F0X073_9STRA/41-105 | 0.00079 | Albugo laibachii Nc14 |
