# Supplementary material for: Bacterial calpains and the evolution of the calpain (C2) family of peptidases
Source: Biol Direct. 2015 Nov 2;10:66. doi: 10.1186/s13062-015-0095-0 (PMC4631099; doi:10.1186/s13062-015-0095-0)
Supplement: Additional file 1: Figure S1. — Protein sequence alignment used for HMMER3 search. The alignment generated by ClustalW is shown. This alignment consists of a representative sequence from every phylum with a known calpain homologue. (DOCX 3054 kb) [file 13062_2015_95_MOESM1_ESM.docx]

|  |  |  |  |  |  |  |  |  |  |  |  |  |  |  |  |  |  |  |  |  |  |  |  |  |  |  |  |  |  |  |  |  |  |  |  | + |  |  |  | + |  |  |  |  | + |  |  | * |  |  |  |  |  |  | * |  |  |  |  |  |  |  |
| --- | --- | --- | --- | --- | --- | --- | --- | --- | --- | --- | --- | --- | --- | --- | --- | --- | --- | --- | --- | --- | --- | --- | --- | --- | --- | --- | --- | --- | --- | --- | --- | --- | --- | --- | --- | --- | --- | --- | --- | --- | --- | --- | --- | --- | --- | --- | --- | --- | --- | --- | --- | --- | --- | --- | --- | --- | --- | --- | --- | --- | --- | --- |
| Q9N4B1_CAEEL | - | - | - | - | - | W | K | R | P | - | - | G | E | I | Y | E | N | P | - | - | - | - | - | - | - | - | - | - | - | - | - | - | Q | L | I | V | - | - | G | E | K | S | R | F | D | V | K | Q | G | - | A | L | G | D | C | W | L | L | A | A | V | 37 |
| Q8IAA8_CAEEL | - | - | - | - | - | W | K | R | P | - | - | G | E | I | I | A | N | P | - | - | - | - | - | - | - | - | - | - | - | - | - | - | Q | L | I | T | - | - | Q | G | E | S | R | F | D | V | K | Q | G | - | A | L | G | D | C | W | F | L | A | A | L | -37 |
| CAN_CAEEL | - | - | - | - | - | W | L | R | P | - | - | G | E | I | T | R | E | P | - | - | - | - | - | - | - | - | - | - | - | - | - | - | Q | L | I | T | - | - | E | G | H | S | R | F | D | V | I | Q | G | - | E | L | G | D | C | W | L | L | A | A | A | -37 |
| Q9U2B2_CAEEL | - | - | - | - | - | W | K | R | P | - | - | G | E | L | F | A | N | P | - | - | - | - | - | - | - | - | - | - | - | - | - | - | R | L | I | G | - | - | S | D | K | T | H | F | D | V | K | L | G | - | R | L | E | N | R | W | F | L | S | G | A | -37 |
| Q9N4B2_CAEEL | - | - | - | - | - | W | L | R | P | - | - | A | K | I | F | A | N | P | - | - | - | - | - | - | - | - | - | - | - | - | - | - | R | L | I | V | - | - | N | G | K | S | R | F | D | V | R | Q | G | - | S | L | G | N | C | W | F | L | A | A | A | -37 |
| C3Y6P6_BRAFL | - | - | - | - | - | W | M | R | P | - | - | Q | D | I | C | D | N | P | - | - | - | - | - | - | - | - | - | - | - | - | - | - | L | F | I | - | - | S | D | G | V | S | R | F | D | V | V | Q | G | - | V | V | G | D | C | W | M | L | A | A | T | -37 |
| C3Y6N1_BRAFL | - | - | - | - | - | W | M | R | P | - | - | G | E | I | V | G | N | P | - | - | - | - | - | - | - | - | - | - | - | - | - | - | E | F | I | D | A | S | E | G | A | S | R | F | D | V | C | Q | G | - | C | V | G | D | C | W | M | L | A | A | T | -39 |
| CANA_DROME | - | - | - | - | - | W | L | R | P | - | - | H | E | I | A | E | N | P | - | - | - | - | - | - | - | - | - | - | - | - | - | - | Q | F | F | V | - | - | E | G | Y | S | R | F | D | V | Q | Q | G | - | E | L | G | D | C | W | L | L | A | A | T | -37 |
| CANB_DROME | - | - | - | - | - | W | L | R | P | - | - | G | D | I | A | D | D | P | - | - | - | - | - | - | - | - | - | - | - | - | - | - | Q | F | F | V | - | - | E | G | Y | S | R | F | D | V | Q | Q | G | - | E | L | G | D | C | W | L | L | A | A | A | -37 |
| X1Y3H71_ANODA | - | - | - | - | - | W | L | R | P | - | - | H | E | F | C | D | A | P | - | - | - | - | - | - | - | - | - | - | - | - | - | - | E | F | F | V | - | - | E | G | F | S | R | F | D | V | Q | Q | G | - | E | L | G | D | C | W | L | L | A | A | C | -37 |
| Q5IW12_GECLA | - | - | - | - | - | W | K | R | P | - | - | H | E | I | T | D | E | P | - | - | - | - | - | - | - | - | - | - | - | - | - | - | Q | L | F | I | - | - | D | G | A | T | R | F | D | V | K | Q | G | - | E | L | G | D | C | W | L | L | A | A | V | -37 |
| K1Q056_CRAGI | - | - | - | - | - | W | K | R | P | - | - | H | E | I | C | D | N | P | - | - | - | - | - | - | - | - | - | - | - | - | - | - | V | F | I | A | - | - | D | G | A | S | R | F | D | V | Q | Q | G | - | E | L | G | D | C | W | L | L | A | A | I | -37 |
| G4V6C0_SCHMA | - | - | - | - | - | W | L | R | P | - | - | V | E | L | S | R | Y | P | - | - | - | - | - | - | - | - | - | - | - | - | - | - | R | F | V | A | - | - | D | G | V | S | R | F | D | I | K | Q | G | - | E | L | G | N | C | W | V | I | A | A | L | -37 |
| R7ULX9_CAPTE | - | - | - | - | - | W | L | R | A | - | - | S | E | I | H | D | D | P | - | - | - | - | - | - | - | - | - | - | - | - | - | - | Q | M | F | V | - | - | D | G | A | C | R | L | D | I | N | Q | G | - | V | L | G | D | C | W | L | L | A | A | V | -37 |
| W4Z5Q7_STRPU | - | - | - | - | - | W | K | R | P | - | - | S | E | I | V | Q | N | P | - | - | - | - | - | - | - | - | - | - | - | - | - | - | Q | L | F | V | - | - | G | G | A | S | R | F | D | V | A | Q | G | - | Q | L | G | D | C | W | F | L | A | A | T | -37 |
| R7VIB6_CAPTE | - | - | - | - | - | W | K | R | P | - | - | S | E | I | C | D | D | P | - | - | - | - | - | - | - | - | - | - | - | - | - | - | Q | M | F | V | - | - | G | G | A | T | R | F | D | V | V | Q | G | - | E | L | G | N | C | W | F | L | A | A | V | -37 |
| T2MJ45_HYDVU | - | - | - | - | - | W | K | R | P | - | - | H | E | I | V | S | D | P | - | - | - | - | - | - | - | - | - | - | - | - | - | - | K | M | F | V | - | - | G | G | A | S | R | F | D | I | T | Q | G | - | M | L | G | D | C | W | L | L | A | A | V | -37 |
| R7UAB6_CAPTE | - | - | - | - | - | W | K | R | P | - | - | T | E | I | T | D | N | P | - | - | - | - | - | - | - | - | - | - | - | - | - | - | Q | W | F | V | - | - | G | G | A | S | R | F | D | V | K | Q | G | - | E | L | G | D | C | W | L | L | A | A | V | -37 |
| C3ZKN8_BRAFL | - | - | - | - | - | W | K | R | P | - | - | H | E | I | T | D | N | P | - | - | - | - | - | - | - | - | - | - | - | - | - | - | E | L | F | V | - | - | G | G | A | S | R | F | D | I | C | Q | G | - | E | L | G | D | C | W | F | L | A | A | I | -37 |
| B3RJS5_TRIAD | - | - | - | - | - | W | K | R | P | - | - | G | E | L | S | K | N | P | - | - | - | - | - | - | - | - | - | - | - | - | - | - | K | L | F | V | - | - | D | G | A | S | R | L | D | I | C | Q | G | - | M | L | G | D | C | W | F | L | A | A | I | -37 |
| H2YVF0_CIOSA | - | - | - | - | - | W | K | R | P | - | - | H | D | I | S | N | D | P | - | - | - | - | - | - | - | - | - | - | - | - | - | - | Q | L | F | V | - | - | G | G | A | S | R | F | D | I | M | Q | G | - | E | L | G | D | C | W | L | L | A | A | I | -37 |
| G4VAG2_SCHMA | - | - | - | - | - | W | K | R | P | - | - | H | E | I | N | P | N | A | - | - | - | - | - | - | - | - | - | - | - | - | - | - | K | F | F | A | - | - | G | G | A | S | R | F | D | I | E | Q | G | - | A | L | G | D | C | W | L | L | A | V | V | -37 |
| Smp_157500 | - | - | - | - | - | W | K | R | P | - | - | K | D | I | N | P | L | A | - | - | - | - | - | - | - | - | - | - | - | - | - | - | A | F | C | T | - | - | K | S | Y | S | R | F | D | V | D | Q | G | - | A | L | G | D | C | W | F | A | S | V | M | -37 |
| Q7SYA9_DANRE | - | - | - | - | - | W | M | R | P | - | - | T | E | L | C | D | D | P | - | - | - | - | - | - | - | - | - | - | - | - | - | - | Q | F | I | V | - | - | G | G | A | T | R | T | D | I | C | Q | G | - | A | L | G | D | C | W | L | L | A | A | I | -37 |
| A5PMP1_DANRE | - | - | - | - | - | W | I | R | P | - | - | T | E | L | S | E | N | P | - | - | - | - | - | - | - | - | - | - | - | - | - | - | Q | F | I | V | - | - | G | G | A | T | R | T | D | I | C | Q | G | - | A | L | G | D | C | W | L | L | A | A | I | -37 |
| CANX_CHICK | - | - | - | - | - | W | K | R | P | - | - | S | E | L | V | D | D | P | - | - | - | - | - | - | - | - | - | - | - | - | - | - | Q | F | I | V | - | - | G | G | A | T | R | T | D | I | C | Q | G | - | A | L | G | D | C | W | L | L | A | A | I | -37 |
| CAN1_HUMAN | - | - | - | - | - | W | K | R | P | - | - | T | E | L | L | S | N | P | - | - | - | - | - | - | - | - | - | - | - | - | - | - | Q | F | I | V | - | - | D | G | A | T | R | T | D | I | C | Q | G | - | A | L | G | D | C | W | L | L | A | A | I | -37 |
| CAN1_MOUSE | - | - | - | - | - | W | K | R | P | - | - | T | E | L | M | S | N | P | - | - | - | - | - | - | - | - | - | - | - | - | - | - | Q | F | I | V | - | - | D | G | A | T | R | T | D | I | C | Q | G | - | A | L | G | D | C | W | L | L | A | A | I | -37 |
| O42133_CHICK | - | - | - | - | - | W | K | R | P | - | - | T | E | L | C | R | H | P | - | - | - | - | - | - | - | - | - | - | - | - | - | - | Q | F | I | V | - | - | D | G | A | T | R | T | D | I | C | Q | G | - | A | L | G | D | C | W | L | L | A | A | I | -37 |
| Q7ZUR1_DANRE | - | - | - | - | - | W | M | R | P | - | - | K | E | M | C | A | R | P | - | - | - | - | - | - | - | - | - | - | - | - | - | - | Q | F | I | V | - | - | D | G | A | T | R | T | D | I | C | Q | G | - | A | L | G | D | C | W | L | L | A | A | I | -37 |
| E9QFN0_DANRE | - | - | - | - | - | W | M | R | P | - | - | T | E | F | C | S | D | P | - | - | - | - | - | - | - | - | - | - | - | - | - | - | H | F | I | V | - | - | D | G | A | T | R | T | D | I | C | Q | G | - | A | L | G | D | C | W | L | L | A | A | I | -37 |
| CAN2_HUMAN | - | - | - | - | - | W | K | R | P | - | - | T | E | I | C | A | D | P | - | - | - | - | - | - | - | - | - | - | - | - | - | - | Q | F | I | I | - | - | G | G | A | T | R | T | D | I | C | Q | G | - | A | L | G | D | C | W | L | L | A | A | I | -37 |
| CAN2_MOUSE | - | - | - | - | - | W | K | R | P | - | - | T | E | I | C | A | D | P | - | - | - | - | - | - | - | - | - | - | - | - | - | - | Q | F | I | I | - | - | G | G | A | T | R | T | D | I | C | Q | G | - | A | L | G | D | C | W | L | L | A | A | I | -37 |
| CAN2_CHICK | - | - | - | - | - | W | C | R | P | - | - | T | E | L | C | S | C | P | - | - | - | - | - | - | - | - | - | - | - | - | - | - | R | F | I | A | - | - | G | G | A | T | R | T | D | I | C | Q | G | - | A | L | G | D | C | W | L | L | A | A | I | -37 |
| Q6DFZ8_DANRE | - | - | - | - | - | W | L | R | P | - | - | K | Q | L | T | S | N | P | - | - | - | - | - | - | - | - | - | - | - | - | - | - | T | F | I | S | - | - | G | G | A | T | R | T | D | I | C | Q | G | - | A | L | G | D | C | W | L | L | A | A | I | -37 |
| Q504F5_DANRE | - | - | - | - | - | W | K | R | P | - | - | G | E | L | C | Q | K | P | - | - | - | - | - | - | - | - | - | - | - | - | - | - | R | F | I | V | - | - | G | G | A | T | K | T | D | I | C | Q | G | - | A | L | G | D | C | W | L | L | A | A | I | -37 |
| Q5BLH5_DANRE | - | - | - | - | - | W | K | R | P | - | - | G | Q | L | N | S | S | P | - | - | - | - | - | - | - | - | - | - | - | - | - | - | E | F | I | V | - | - | G | G | A | T | R | T | D | I | C | Q | G | - | A | L | G | D | C | W | L | L | A | A | I | -37 |
| Q4VBH3_DANRE | - | - | - | - | - | W | K | R | P | - | - | K | E | L | C | S | N | P | - | - | - | - | - | - | - | - | - | - | - | - | - | - | E | F | I | M | - | - | D | G | A | N | R | T | D | I | C | Q | G | - | A | L | G | D | C | W | L | L | A | A | I | -37 |
| CAN8_RAT | - | - | - | - | - | W | K | R | P | - | - | T | E | L | C | P | N | P | - | - | - | - | - | - | - | - | - | - | - | - | - | - | Q | F | I | V | - | - | G | G | A | T | R | T | D | I | R | Q | G | - | G | L | G | D | C | W | L | L | A | A | I | -37 |
| CAN8_MOUSE | - | - | - | - | - | W | K | R | P | - | - | T | E | L | C | S | N | P | - | - | - | - | - | - | - | - | - | - | - | - | - | - | Q | F | I | V | - | - | G | G | A | T | R | T | D | I | R | Q | G | - | G | L | G | D | C | W | L | L | A | A | I | -37 |
| CAN8_HUMAN | - | - | - | - | - | W | K | R | P | - | - | T | E | L | C | P | S | P | - | - | - | - | - | - | - | - | - | - | - | - | - | - | Q | F | I | V | - | - | G | G | A | T | R | T | D | I | C | Q | G | - | G | L | G | D | C | W | L | L | A | A | I | -37 |
| XP_426117 | - | - | - | - | - | W | K | R | P | - | - | T | E | L | C | D | N | P | - | - | - | - | - | - | - | - | - | - | - | - | - | - | Q | F | I | V | - | - | G | G | A | T | R | T | D | V | C | Q | G | - | E | L | G | D | C | W | L | L | A | A | I | -37 |
| CAN9_MOUSE | - | - | - | F | V | W | K | R | P | - | - | G | E | I | V | E | K | P | - | - | - | - | - | - | - | - | - | - | - | - | - | - | E | F | I | L | - | - | G | G | A | T | R | T | D | I | C | Q | G | - | E | L | G | D | C | W | L | L | A | A | I | -39 |
| CAN9_HUMAN | - | - | - | - | - | W | K | R | P | - | - | G | E | I | V | K | N | P | - | - | - | - | - | - | - | - | - | - | - | - | - | - | E | F | I | L | - | - | G | G | A | T | R | T | D | I | C | Q | G | - | E | L | G | D | C | W | L | L | A | A | I | -37 |
| E1C6M3_CHICK | - | - | - | - | - | W | K | R | P | - | - | G | D | I | V | K | D | P | - | - | - | - | - | - | - | - | - | - | - | - | - | - | K | F | I | L | - | - | E | G | A | T | R | T | D | I | C | Q | G | - | D | L | G | D | C | W | L | L | A | A | I | -37 |
| Q6DG44_DANRE | - | - | - | - | - | W | K | R | P | - | - | G | E | I | C | K | E | P | - | - | - | - | - | - | - | - | - | - | - | - | - | - | Q | F | I | T | - | - | G | G | A | T | R | T | D | I | C | Q | G | - | Q | L | G | D | C | W | L | L | A | A | I | -37 |
| CAN3_HUMAN | - | - | - | - | - | W | K | R | P | - | - | P | E | I | C | E | N | P | - | - | - | - | - | - | - | - | - | - | - | - | - | - | R | F | I | I | - | - | D | G | A | N | R | T | D | I | C | Q | G | - | E | L | G | D | C | W | F | L | A | A | I | -37 |
| CAN3_MOUSE | - | - | - | - | - | W | K | R | P | - | - | P | E | I | C | E | N | P | - | - | - | - | - | - | - | - | - | - | - | - | - | - | R | F | I | I | - | - | G | G | A | N | R | T | D | I | C | Q | G | - | D | L | G | D | C | W | F | L | A | A | I | -37 |
| CAN3_CHICK | - | - | - | - | - | W | K | R | P | - | - | R | E | I | C | E | N | P | - | - | - | - | - | - | - | - | - | - | - | - | - | - | R | F | I | I | - | - | G | G | A | N | R | T | D | I | C | Q | G | - | E | L | G | D | C | W | F | L | A | A | I | -37 |
| Q66HU4_DANRE | - | - | - | - | - | W | K | R | P | - | - | K | E | I | C | D | N | P | - | - | - | - | - | - | - | - | - | - | - | - | - | - | Q | F | I | V | - | - | G | G | A | S | R | T | D | I | C | Q | G | - | D | L | G | D | C | W | L | L | A | A | I | -37 |
| F1R632_DANRE | - | - | - | - | - | W | K | R | P | - | - | M | E | I | C | K | N | P | - | - | - | - | - | - | - | - | - | - | - | - | - | - | Q | F | I | L | - | - | G | G | A | N | R | T | D | I | C | Q | G | - | D | L | G | D | C | W | L | L | A | A | I | -37 |
| CAN11_HUMAN | - | - | - | - | - | W | Q | R | P | - | - | K | D | I | I | N | N | P | - | - | - | - | - | - | - | - | - | - | - | - | - | - | L | F | I | M | - | - | D | G | I | S | P | T | D | I | C | Q | G | - | I | L | G | D | C | W | L | L | A | A | I | -37 |
| CAN11_MOUSE | - | - | - | - | - | W | Q | R | P | - | - | K | D | I | I | H | N | P | - | - | - | - | - | - | - | - | - | - | - | - | - | - | Q | F | I | T | - | - | N | D | F | S | P | T | D | I | C | Q | G | - | I | L | G | D | C | W | L | L | A | A | I | -37 |
| CAN12_MOUSE | - | - | - | - | - | W | K | R | P | - | - | H | E | F | C | A | E | P | - | - | - | - | - | - | - | - | - | - | - | - | - | - | Q | F | I | C | - | - | E | D | M | S | R | T | D | V | C | Q | G | - | S | L | G | N | C | W | L | L | A | A | A | -37 |
| CAN12_HUMAN | - | - | - | - | - | W | M | R | P | - | - | H | E | F | C | A | E | P | - | - | - | - | - | - | - | - | - | - | - | - | - | - | K | F | I | C | - | - | E | D | M | S | R | T | D | V | C | Q | G | - | S | L | G | N | C | W | F | L | A | A | A | -37 |
| ENSSARP00000008135 | - | - | - | - | - | W | K | R | P | - | - | H | E | F | C | A | E | P | - | - | - | - | - | - | - | - | - | - | - | - | - | - | H | F | I | C | - | - | E | D | V | S | R | T | D | V | C | Q | G | - | R | L | G | N | C | W | L | L | A | A | A | -37 |
| ENSSARP00000010553 | - | - | - | - | - | W | V | R | P | - | - | E | D | L | S | N | N | P | - | - | - | - | - | - | - | - | - | - | - | - | - | - | T | M | I | Y | - | - | T | - | V | S | S | F | S | I | K | Q | T | - | I | V | S | D | C | S | F | V | A | S | L | -36 |
| A7E2G3_DANRE | - | - | - | - | - | W | L | R | P | - | - | K | E | I | T | S | N | A | - | - | - | - | - | - | - | - | - | - | - | - | - | - | V | F | V | E | - | - | G | T | M | G | T | T | D | I | C | Q | G | - | Q | L | G | N | C | W | L | L | A | A | L | -37 |
| Q6NW69_DANRE | - | - | - | - | - | W | R | R | P | - | - | K | E | I | C | L | F | P | - | - | - | - | - | - | - | - | - | - | - | - | - | - | Q | F | I | V | - | - | D | G | A | T | R | M | D | I | C | Q | V | - | V | L | G | D | C | W | F | L | S | A | V | -37 |
| CAN13_HUMAN | - | - | - | - | - | W | K | R | P | - | - | Q | D | L | P | G | G | P | P | - | - | - | - | - | - | - | - | - | - | - | - | - | H | F | I | L | - | - | D | D | I | S | R | F | D | I | Q | Q | G | - | G | A | A | D | C | W | F | L | A | A | L | -38 |
| CAN13_MOUSE | - | - | - | - | - | W | K | R | P | T | S | K | D | L | P | F | Y | P | P | - | - | - | - | - | - | - | - | - | - | - | - | - | H | F | I | L | - | - | E | G | A | S | R | F | D | I | H | Q | G | - | I | A | G | D | C | W | F | L | A | A | L | -40 |
| E1BRR5_CHICK | - | - | - | - | - | W | K | R | P | - | - | R | E | L | L | R | N | - | P | - | - | - | - | - | - | - | - | - | - | - | - | - | C | L | I | M | - | - | D | G | V | S | R | F | D | I | I | Q | G | - | E | I | G | D | C | W | M | L | A | A | L | -37 |
| E7F0H3_DANRE | - | - | - | - | - | W | K | R | P | - | - | S | E | L | V | S | D | P | - | - | - | - | - | - | - | - | - | - | - | - | - | - | N | L | I | V | - | - | D | D | V | S | R | F | D | Y | A | Q | G | S | V | L | G | N | C | W | F | L | A | S | I | -38 |
| Q1RLS9_DANRE | - | - | - | - | - | W | K | R | P | - | - | Y | E | L | V | P | N | P | - | - | - | - | - | - | - | - | - | - | - | - | - | - | V | Y | I | A | - | - | Q | G | T | S | R | F | D | F | I | Q | G | - | R | L | G | N | C | W | F | L | A | S | V | -37 |
| F1Q9Z1_DANRE | - | - | - | - | - | W | I | R | P | - | - | T | K | L | Y | P | E | A | A | - | - | - | - | - | - | - | - | - | - | - | - | - | E | F | V | V | - | - | D | T | V | S | R | F | D | Y | A | Q | G | - | F | I | G | N | C | W | F | L | A | S | V | -38 |
| A4QNV6_DANRE | - | - | - | - | - | W | L | R | P | - | - | S | K | I | V | A | D | P | - | - | - | - | - | - | - | - | - | - | - | - | - | - | Q | F | I | V | - | - | K | G | V | S | R | F | D | Y | S | Q | G | S | F | L | G | N | C | W | F | L | A | S | V | -38 |
| D6W573_HUMAN | - | - | - | - | - | W | K | R | P | - | - | P | E | L | H | S | N | P | - | - | - | - | - | - | - | - | - | - | - | - | - | - | Q | F | Y | F | - | - | A | K | A | K | R | L | D | L | C | Q | G | - | I | V | G | D | C | W | F | L | A | A | L | -37 |
| R7UBC6_CAPTE | - | - | - | - | - | W | K | R | P | - | - | W | E | L | L | G | N | R | G | L | - | - | - | - | - | - | - | - | - | - | Q | P | M | L | F | A | - | - | D | E | A | T | H | F | R | V | K | Q | G | - | E | L | G | D | L | W | V | V | I | A | A | -41 |
| K1RCZ2_CRAGI | - | - | - | - | - | W | K | R | P | - | - | T | E | I | A | A | Q | H | Q | L | - | - | - | - | - | - | - | - | - | - | K | P | A | F | F | L | - | - | D | N | A | T | K | F | D | V | I | Q | G | - | D | L | G | D | C | W | V | V | S | A | I | -41 |
| Q5IW13_GECLA | - | - | - | - | - | W | K | R | P | - | - | L | E | M | V | Q | N | - | - | - | - | - | - | - | - | - | - | - | - | - | - | P | Q | F | F | I | - | - | D | G | A | T | R | F | D | I | Q | Q | G | - | E | L | G | D | C | W | L | L | A | A | V | -37 |
| R7UUX7_CAPTE | - | - | - | - | - | W | K | R | P | - | - | K | E | I | S | S | D | - | - | - | - | - | - | - | - | - | - | - | - | - | - | P | K | F | I | D | - | - | K | K | S | T | R | F | D | L | D | Q | G | - | D | L | G | D | C | W | F | I | A | G | A | -37 |
| K1Q505_CRAGI | - | - | - | - | - | W | K | R | P | - | - | T | E | L | S | R | N | - | - | - | - | - | - | - | - | - | - | - | - | - | - | P | K | F | I | V | - | - | R | N | A | T | R | F | D | L | D | Q | G | - | Y | L | G | N | C | W | F | I | A | G | A | -37 |
| R7UD02_CAPTE | - | - | - | - | - | W | K | R | P | - | - | A | E | I | C | E | N | - | - | - | - | - | - | - | - | - | - | - | - | - | - | P | M | F | A | Q | - | - | D | G | A | T | I | F | D | L | D | Q | G | - | D | L | G | N | C | W | F | L | S | A | A | -37 |
| CAN6_HUMAN | - | - | - | - | - | W | K | R | P | - | - | Q | D | I | C | D | D | P | - | - | - | - | - | - | - | - | - | - | - | - | - | - | H | L | I | V | - | - | G | N | I | S | N | H | Q | L | T | Q | G | - | R | L | G | H | K | P | M | V | S | A | F | -37 |
| CAN6_MOUSE | - | - | - | - | - | W | K | R | P | - | - | Q | D | I | S | D | D | P | - | - | - | - | - | - | - | - | - | - | - | - | - | - | H | L | I | V | - | - | G | N | I | S | N | H | Q | L | I | Q | G | - | R | L | G | N | K | A | M | I | S | A | F | -37 |
| E1C312_CHICK | - | - | - | - | - | W | K | R | P | - | - | K | D | L | C | Q | D | P | - | - | - | - | - | - | - | - | - | - | - | - | - | - | Y | L | F | V | - | - | N | G | I | S | S | H | D | L | H | Q | G | - | S | L | G | N | C | W | F | V | A | A | C | -37 |
| E7FDN3_DANRE | - | - | - | - | - | W | K | R | P | - | - | S | E | L | C | K | D | P | - | - | - | - | - | - | - | - | - | - | - | - | - | - | R | L | F | V | - | - | D | G | I | S | T | R | D | L | H | Q | G | - | S | L | G | N | C | W | M | V | A | A | T | -37 |
| CAN5_HUMAN | - | - | - | - | - | - | - | - | - | - | - | K | G | I | C | E | D | P | - | - | - | - | - | - | - | - | - | - | - | - | - | - | R | L | F | V | - | - | D | G | I | S | S | H | D | L | H | Q | G | - | Q | V | G | N | C | W | F | V | A | A | C | -33 |
| CAN5_MOUSE | - | - | - | - | - | W | K | R | P | - | - | K | D | I | C | D | D | P | - | - | - | - | - | - | - | - | - | - | - | - | - | - | R | L | F | V | - | - | D | G | I | S | S | H | D | L | H | Q | G | - | Q | V | G | N | C | W | F | V | A | A | C | -37 |
| E1C292_CHICK | - | - | - | - | - | W | K | R | P | - | - | K | E | I | C | S | D | P | - | - | - | - | - | - | - | - | - | - | - | - | - | - | H | L | F | V | - | - | D | G | I | S | S | H | D | L | H | Q | G | - | Q | V | G | N | C | W | F | V | A | A | C | -37 |
| A0JMG3_DANRE | - | - | - | - | - | W | K | R | P | - | - | K | D | L | C | E | D | P | - | - | - | - | - | - | - | - | - | - | - | - | - | - | H | L | F | V | - | - | N | G | I | S | A | H | D | L | N | Q | G | - | Q | L | G | N | C | W | F | V | A | A | C | -37 |
| E7F7F1_DANRE | - | - | - | - | - | W | K | R | P | - | - | E | E | L | C | D | D | P | - | - | - | - | - | - | - | - | - | - | - | - | - | - | H | L | F | V | - | - | D | G | I | S | A | H | D | L | H | Q | G | - | Q | L | G | N | C | W | F | V | A | A | C | -37 |
| XP_006824412 | - | - | - | - | - | - | - | - | - | - | - | - | - | I | S | N | D | P | - | - | - | - | - | - | - | - | - | - | - | - | - | - | K | L | F | V | - | - | E | G | A | S | S | G | D | V | S | Q | G | - | S | L | G | N | C | W | F | V | A | A | C | -31 |
| W4YJN2_STRPU | - | - | - | - | - | W | K | R | P | - | - | G | E | L | T | S | D | P | - | - | - | - | - | - | - | - | - | - | - | - | - | - | H | L | V | V | - | - | D | G | T | K | S | G | D | V | S | Q | G | - | Q | L | G | N | C | W | F | V | A | A | T | -37 |
| C3YJZ3_BRAFL | - | - | - | - | - | W | K | R | P | - | - | R | E | L | C | D | N | P | - | - | - | - | - | - | - | - | - | - | - | - | - | - | R | L | F | V | - | - | E | G | V | S | S | N | D | L | N | Q | G | - | Q | L | G | N | C | W | F | V | A | A | V | -37 |
| T2MIT4_HYDVU | - | - | - | - | - | W | K | R | P | - | - | G | E | I | C | D | D | P | - | - | - | - | - | - | - | - | - | - | - | - | - | - | Q | M | F | V | - | - | D | G | A | S | S | A | D | V | T | Q | G | - | R | V | G | N | C | W | F | V | A | A | A | -37 |
| XP_002154722 | - | - | - | - | - | - | - | - | - | - | - | - | E | I | C | D | D | P | - | - | - | - | - | - | - | - | - | - | - | - | - | - | Q | M | F | V | - | - | D | G | A | S | S | A | D | V | T | Q | G | - | R | V | G | N | C | W | F | V | A | A | A | -32 |
| R7VLA7_CAPTE | - | - | - | - | - | W | K | R | P | - | - | K | D | I | A | E | N | P | - | - | - | - | - | - | - | - | - | - | - | - | - | - | S | L | F | V | - | - | D | G | V | T | C | S | D | L | N | Q | G | - | E | L | G | N | C | W | F | V | A | A | C | -37 |
| K1R1C6_CRAGI | - | - | - | - | - | W | K | R | P | - | - | K | Q | I | C | E | N | P | - | - | - | - | - | - | - | - | - | - | - | - | - | - | K | F | F | V | - | - | E | G | T | S | S | G | D | V | K | Q | G | - | R | L | G | N | C | W | F | V | A | A | S | -37 |
| B3RIE5_TRIAD | - | - | - | - | - | W | K | R | P | - | - | T | E | I | T | D | N | P | - | - | - | - | - | - | - | - | - | - | - | - | - | - | K | F | F | V | - | - | D | G | T | S | P | S | D | V | T | Q | G | - | H | L | G | N | C | W | F | V | A | A | C | -37 |
| K1PW75_CRAGI | - | - | - | - | - | W | K | R | P | - | - | G | E | I | A | S | D | P | - | - | - | - | - | - | - | - | - | - | - | - | - | - | K | F | F | D | - | - | E | G | A | S | A | D | D | F | A | Q | G | - | S | L | G | N | C | W | F | V | A | A | C | -37 |
| K1RSB8_CRAGI | - | - | - | - | - | W | K | R | P | - | - | G | D | I | V | S | N | P | - | - | - | - | - | - | - | - | - | - | - | - | - | - | R | F | F | T | - | - | E | G | R | G | A | D | D | F | S | Q | G | - | S | L | G | N | C | W | F | V | A | A | F | -37 |
| K1RWI3_CRAGI | - | - | - | - | - | W | K | R | P | - | - | G | E | I | A | A | E | P | - | - | - | - | - | - | - | - | - | - | - | - | - | - | K | F | F | V | - | - | D | G | A | S | T | D | D | F | H | Q | G | - | S | L | G | N | C | W | F | V | A | A | A | -37 |
| R7VGC2_CAPTE | - | - | - | - | - | W | K | R | P | - | - | K | E | L | C | R | V | P | - | - | - | - | - | - | - | - | - | - | - | - | - | - | K | L | I | T | - | - | D | G | V | S | C | D | D | L | V | E | G | - | E | L | G | N | A | W | F | V | T | A | C | -37 |
| K1QU26_CRAGI | - | - | - | - | - | W | K | R | P | - | - | K | E | L | C | K | V | P | - | - | - | - | - | - | - | - | - | - | - | - | - | - | K | L | V | V | - | - | E | G | V | T | C | D | D | L | N | Q | G | - | E | L | G | N | T | W | F | V | T | A | C | -37 |
| CAN5_CAEEL | - | - | - | - | - | W | K | R | P | - | - | G | E | L | H | P | D | P | - | - | - | - | - | - | - | - | - | - | - | - | - | - | H | L | F | V | - | - | E | G | A | S | P | N | D | V | T | Q | G | - | I | L | G | N | C | W | F | V | S | A | C | -37 |
| I1FGV4_AMPQE | - | - | - | - | - | W | K | R | P | - | - | K | E | I | V | S | D | P | - | - | - | - | - | - | - | - | - | - | - | - | - | - | Q | M | F | T | - | - | G | G | S | S | R | F | D | V | V | Q | G | - | M | L | G | D | C | W | L | V | A | A | I | -37 |
| K1PHX3_CRAGI | - | - | - | - | - | W | K | R | P | - | - | Y | E | I | A | A | H | P | - | - | - | - | - | - | - | - | - | - | - | - | - | - | K | F | T | A | - | - | D | G | F | S | R | F | D | L | L | Q | G | - | K | L | G | N | C | W | L | V | A | A | F | -37 |
| B3RNL8_TRIAD | - | - | - | - | - | W | L | R | P | - | - | K | E | I | T | D | D | P | - | - | - | - | - | - | - | - | - | - | - | - | - | - | K | L | Y | V | - | - | D | G | G | D | R | L | D | M | R | Q | G | - | R | L | G | D | C | W | F | V | A | S | L | -37 |
| X1Y3H7_ANODA | - | - | - | - | - | W | K | R | P | - | - | K | D | I | V | D | N | P | - | - | - | - | - | - | - | - | - | - | - | - | - | - | Q | F | I | T | - | - | E | G | Y | S | R | F | D | I | N | Q | G | - | G | L | G | D | C | W | F | L | S | S | C | -37 |
| R7TWH9_CAPTE | - | - | - | - | - | W | K | R | P | - | - | S | Q | L | V | P | E | G | K | - | - | - | - | - | - | - | - | - | - | - | S | P | H | M | L | V | - | - | D | G | I | S | R | D | D | I | K | Q | G | - | V | L | G | D | C | W | F | L | S | S | C | -40 |
| K1R5E8_CRAGI | - | - | - | - | - | W | K | R | P | - | - | V | D | L | V | E | E | W | Q | - | - | - | - | - | - | - | - | - | - | - | T | P | Q | L | M | V | - | - | D | G | V | T | R | D | D | I | K | Q | G | - | T | L | G | D | C | W | F | L | S | A | C | -40 |
| XP_006811804 | - | - | - | - | - | W | K | R | P | - | - | K | E | I | S | D | - | - | - | - | - | - | - | - | - | - | - | - | - | - | S | P | S | L | L | V | - | - | D | G | I | S | R | D | D | V | V | Q | G | - | I | L | G | D | C | W | F | L | S | S | C | -37 |
| C3YB23_BRAFL | - | - | - | - | - | W | K | R | P | - | - | Q | E | I | M | G | - | V | T | - | - | - | - | - | - | - | - | - | - | - | Q | P | R | L | L | V | - | - | D | G | I | S | R | D | D | V | I | Q | G | - | M | L | G | D | C | W | F | L | V | S | L | -39 |
| H2ZIF1_CIOSA | - | - | - | - | - | W | K | R | P | - | - | M | D | L | S | P | - | - | - | - | - | - | - | - | - | - | - | - | - | - | N | P | R | L | L | K | - | - | N | G | Q | S | S | E | D | I | I | Q | G | - | M | L | G | N | C | W | W | L | A | S | S | -37 |
| W4YV93_STRPU | - | - | - | - | - | W | K | R | P | - | - | F | E | L | V | F | - | - | - | - | - | - | - | - | - | - | - | - | - | - | N | P | R | L | G | V | - | - | D | G | F | S | R | Q | D | G | V | Q | G | - | N | L | G | D | C | W | F | L | A | S | L | -37 |
| CAN10_HUMAN | - | - | - | - | - | W | R | R | P | - | - | Q | E | I | C | A | - | - | - | - | - | - | - | - | - | - | - | - | - | - | - | - | T | P | R | L | F | P | D | D | P | R | E | G | Q | V | - | K | Q | G | L | L | G | D | C | W | F | L | C | A | C | -37 |
| CAN10_MOUSE | - | - | - | - | - | W | R | R | P | - | - | Q | E | I | C | A | - | - | - | - | - | - | - | - | - | - | - | - | - | - | - | - | T | P | Q | L | F | P | D | N | P | W | E | G | Q | V | - | K | Q | G | L | L | G | D | C | W | F | L | C | A | C | -37 |
| XP_004943443 | - | - | - | - | - | W | L | R | P | - | - | Q | D | I | C | S | - | - | - | - | - | - | - | - | - | - | - | - | - | - | - | - | T | P | R | L | F | S | N | S | L | Q | D | V | Q | V | - | K | Q | G | I | L | G | D | C | W | F | L | C | A | C | -37 |
| F8W4K1_DANRE | - | - | - | - | - | W | L | R | P | - | - | Q | E | I | C | Q | - | - | - | - | - | - | - | - | - | - | - | - | - | - | - | - | Q | P | Q | L | F | P | D | D | P | L | E | A | H | P | - | K | Q | G | I | L | G | D | C | W | L | L | C | A | C | -37 |
| CANC_DROME | - | - | - | - | - | - | - | - | - | - | - | - | - | - | - | - | - | - | - | - | - | - | - | - | - | - | - | - | - | - | - | - | - | - | P | V | F | L | N | E | S | A | E | F | D | V | - | V | P | G | K | M | G | D | R | W | L | V | S | C | L | -26 |
| C3ZKN9_BRAFL | - | - | - | - | - | W | R | R | P | - | - | K | E | L | T | N | - | - | - | - | - | - | - | - | - | - | - | - | - | - | - | - | S | P | Q | L | G | - | S | C | T | D | S | F | D | V | - | D | P | G | E | L | G | S | A | R | I | V | A | S | M | -36 |
| G4V623_SCHMA | - | - | - | - | - | W | M | R | P | - | - | S | E | I | V | A | A | N | S | G | G | G | S | M | G | I | T | P | R | K | I | S | T | P | E | F | I | G | E | G | G | I | K | L | G | E | L | R | Q | G | E | L | G | D | C | W | V | V | A | A | L | -54 |
| R7VK39_CAPTE | - | - | - | - | - | W | K | R | P | - | - | H | E | I | S | - | - | - | - | - | - | - | - | - | - | - | - | - | - | - | - | T | T | P | E | L | V | S | D | K | Y | P | N | M | L | L | N | R | G | S | D | D | K | N | S | H | I | T | D | A | M | -38 |
| G4V693_SCHMA | - | - | - | - | - | Y | L | R | P | - | - | S | E | I | V | S | D | P | - | - | - | - | - | - | - | - | - | - | - | - | - | - | - | C | F | S | - | - | R | N | E | S | Y | F | S | L | K | R | G | - | F | V | K | N | F | N | I | L | L | A | F | -36 |
| R7UPU6_CAPTE | - | - | - | - | - | W | M | R | P | - | - | V | E | F | L | D | H | P | - | - | - | - | - | - | - | - | - | - | - | - | - | - | V | L | F | A | - | - | D | G | T | T | R | F | D | I | E | Q | G | - | G | A | G | T | C | W | F | L | S | I | L | -37 |
| R7V817_CAPTE | - | - | - | - | - | - | - | R | V | - | - | Q | E | F | L | D | H | P | - | - | - | - | - | - | - | - | - | - | - | - | - | - | V | L | F | A | - | - | G | G | T | T | R | F | D | I | E | Q | G | - | G | A | G | T | C | W | F | L | S | I | L | -35 |
| R7UNL8_CAPTE | - | - | - | - | - | - | - | - | - | - | - | - | E | F | C | Q | Q | P | - | - | - | - | - | - | - | - | - | - | - | - | - | - | T | L | F | S | - | - | D | G | T | T | R | F | D | V | E | Q | G | - | G | A | G | T | C | W | F | L | S | I | L | -32 |
| K1PJ80_CRAGI | - | - | - | - | - | W | K | R | P | - | - | P | E | F | A | S | S | P | - | - | - | - | - | - | - | - | - | - | - | - | - | - | C | L | F | S | - | - | E | G | T | T | R | Y | D | I | G | Q | G | - | S | I | G | T | C | W | F | L | S | S | V | -37 |
| K1PCX8_CRAGI | - | - | - | - | - | W | K | R | P | - | - | K | E | F | A | E | K | P | - | - | - | - | - | - | - | - | - | - | - | - | - | - | C | L | F | S | - | - | E | G | T | S | R | Y | D | I | G | Q | G | - | S | I | G | T | C | W | F | L | A | S | V | -37 |
| CAND_DROME | - | - | - | - | - | W | R | R | P | H | E | I | N | C | D | G | - | - | G | - | - | - | - | - | - | - | - | - | - | - | A | Y | P | P | W | A | V | F | R | T | P | L | P | S | D | I | C | Q | G | - | V | L | G | N | C | W | L | L | S | A | L | -42 |
| XP_002735765 | - | - | - | - | - | W | L | R | P | T | E | I | S | C | S | T | R | S | E | - | - | - | - | - | - | - | - | - | - | - | M | K | T | P | W | A | V | F | R | T | P | F | P | S | D | I | S | Q | G | - | I | L | G | N | C | W | F | L | S | A | L | -44 |
| CAN15_HUMAN | - | - | - | - | - | W | L | R | P | Q | E | I | N | C | S | V | F | R | D | - | - | - | - | - | - | - | - | - | - | - | H | R | A | T | W | S | V | F | H | T | L | R | P | S | D | I | L | Q | G | - | L | L | G | N | C | W | F | L | S | A | L | -44 |
| CAN15_MOUSE | - | - | - | - | - | W | L | R | P | H | E | I | N | C | S | V | F | R | D | - | - | - | - | - | - | - | - | - | - | - | H | G | T | P | W | S | V | F | H | T | L | R | P | S | D | I | L | Q | G | - | L | L | G | N | C | W | F | L | S | A | L | -44 |
| E1BTA8_CHICK | - | - | - | - | - | W | L | R | P | H | E | I | N | C | N | I | F | K | D | - | - | - | - | - | - | - | - | - | - | - | R | S | V | K | W | S | V | F | R | T | P | R | P | S | D | I | L | Q | G | - | L | L | G | N | C | W | F | L | S | A | L | -44 |
| E7FAX5_DANRE | - | - | - | - | - | W | L | R | P | H | E | I | N | C | N | N | F | K | D | - | - | - | - | - | - | - | - | - | - | - | R | G | V | K | W | S | V | F | R | T | P | R | P | S | D | I | L | Q | G | - | L | L | G | N | C | W | F | L | S | A | L | -44 |
| C3XWK8_BRAFL | - | - | - | - | - | W | L | R | P | H | Q | I | T | - | D | L | S | Q | D | - | - | - | - | - | - | - | - | - | - | - | R | G | T | K | W | V | V | F | R | T | P | R | P | S | D | I | S | Q | G | - | I | L | G | N | C | W | F | L | S | A | L | -43 |
| W4YMU4_STRPU | - | - | - | - | - | W | L | R | P | S | A | V | Q | L | K | A | R | D | D | - | - | - | - | - | - | - | - | - | - | - | S | P | L | A | W | E | V | F | R | N | P | K | P | S | D | I | S | Q | G | - | L | L | G | N | C | W | F | L | S | A | L | -44 |
| K1PV74_CRAGI | - | - | - | - | - | W | L | R | P | T | E | I | A | A | P | R | H | G | D | - | - | - | - | - | - | - | - | - | - | - | E | K | V | K | W | A | V | C | R | T | P | L | P | D | D | I | S | Q | G | - | L | L | G | N | C | W | F | L | S | A | L | -44 |
| R7TJF2_CAPTE | - | - | - | - | - | W | L | R | P | E | K | I | S | S | M | D | R | S | E | - | - | - | - | - | - | - | - | - | - | - | G | S | I | R | W | T | V | S | R | N | P | A | P | S | D | I | T | Q | G | - | I | L | G | N | C | W | F | L | S | A | L | -44 |
| T2MI17_HYDVU | - | - | - | - | - | W | L | R | P | K | E | I | F | R | S | N | - | - | - | - | - | - | - | - | - | - | - | - | - | - | T | Q | T | K | W | T | M | F | R | N | P | R | P | S | D | I | L | Q | G | - | T | I | G | N | C | W | F | L | S | A | L | -41 |
| Q1RL55_CIOIN | - | - | - | - | - | W | R | - | - | - | - | - | - | - | - | - | - | - | - | - | - | - | - | - | - | - | - | - | - | - | - | N | R | E | W | S | V | F | S | N | P | S | P | T | D | I | K | Q | G | - | V | L | G | N | C | W | F | L | S | A | L | -31 |
| I1EA30_AMPQE | - | - | - | - | - | W | S | R | P | A | D | I | R | L | D | R | - | - | - | - | - | - | - | - | - | - | - | - | - | - | R | H | G | T | W | C | V | F | R | D | P | K | P | D | D | I | A | Q | G | - | M | L | G | D | C | W | F | L | S | A | L | -41 |
| G5EEZ6_CAEEL | - | - | - | - | - | W | L | R | P | S | Q | M | F | T | K | D | - | - | G | - | - | - | - | - | - | - | - | - | - | - | R | S | S | P | W | S | V | L | N | N | P | R | P | S | D | I | E | Q | G | T | L | V | G | D | C | W | L | M | S | A | M | -43 |
| O44903_CAEEL | - | - | - | - | - | W | L | R | P | S | Q | I | R | T | K | D | - | - | G | - | - | - | - | - | - | - | - | - | - | - | T | S | C | A | W | S | V | F | N | N | P | Q | P | S | D | I | E | Q | G | N | L | V | G | D | C | W | L | L | S | A | M | -43 |
| O02260_CAEEL | - | - | - | - | - | W | R | R | I | T | P | P | S | T | D | V | S | - | - | - | - | - | - | - | - | - | - | - | - | - | S | S | L | S | V | Y | N | P | L | T | F | T | A | Y | E | V | F | Q | R | - | K | V | G | D | C | G | L | V | A | A | L | -42 |
| O02259_CAEEL | - | - | - | - | - | - | - | - | - | - | - | - | - | - | - | - | - | - | - | - | - | - | - | - | - | - | - | - | - | - | - | - | - | - | - | - | - | - | - | - | - | T | A | Y | E | V | F | Q | R | - | K | V | G | D | C | G | L | V | A | A | L | -19 |
| C1MTG3_MICPC | - | - | - | - | - | - | - | - | - | - | - | - | - | - | - | - | - | - | - | - | - | - | - | - | - | - | - | - | - | - | - | - | - | - | - | - | - | - | - | - | - | - | - | A | H | V | R | Q | G | - | A | V | G | D | C | W | L | L | S | A | L | -17 |
| C1E493_MICSR | - | - | - | - | - | - | - | - | - | - | - | - | - | - | - | - | - | - | - | - | - | - | - | - | - | - | - | - | - | - | - | - | - | - | - | - | - | - | - | - | - | - | - | S | D | V | R | Q | G | - | A | V | G | D | C | W | L | L | S | A | L | -17 |
| Q012Z6_OSTTA | - | - | - | - | - | - | - | - | - | - | - | - | - | - | - | - | - | - | - | - | - | - | - | - | - | - | - | - | - | - | - | - | - | - | - | - | F | A | R | R | T | S | A | A | D | I | R | Q | G | - | S | V | G | D | C | W | F | L | S | S | L | -24 |
| L1IGB6_GUITH | - | - | - | - | - | - | - | - | - | - | - | - | - | - | - | - | - | - | - | - | - | - | - | - | - | - | - | - | - | - | P | S | F | V | L | V | T | D | F | G | F | S | A | Q | D | L | Q | Q | G | - | G | I | G | D | C | W | F | L | S | A | L | -30 |
| B7FWX6_PHATC | - | - | - | - | - | - | - | - | - | - | - | - | - | - | - | - | - | - | - | - | - | - | - | - | - | - | - | - | - | - | - | - | - | - | - | - | - | - | - | - | - | - | - | S | Q | V | R | Q | G | - | A | V | G | N | C | W | F | L | S | A | L | -17 |
| N9TPR9_ENTHI | - | - | - | - | - | F | L | R | P | N | E | Y | M | E | H | P | - | - | - | - | - | - | - | - | - | - | - | - | - | - | - | - | Q | L | Y | E | N | - | - | G | I | E | P | L | D | I | Q | Q | G | - | C | L | G | D | C | Y | F | L | A | A | L | -37 |
| G0QS43_ICHMG | - | - | - | - | - | W | L | H | P | S | K | I | A | K | Y | E | N | - | - | - | - | - | - | - | - | - | - | - | - | - | - | C | Q | L | F | K | D | - | - | K | I | E | P | N | D | I | R | Q | G | - | F | L | G | N | C | Y | F | L | S | T | L | -39 |
| D8LB20_ECTSI | - | - | - | - | - | W | K | R | A | D | L | I | K | A | R | D | D | D | E | - | - | - | - | - | - | - | - | - | - | - | P | F | H | L | F | N | K | - | - | G | V | K | A | E | D | V | K | Q | G | - | A | L | G | D | C | W | L | V | A | A | M | -42 |
| Q9U0T9_LEIMA | - | - | - | - | - | - | - | - | - | - | P | R | M | Y | V | K | P | E | L | - | - | - | - | - | - | - | - | - | - | - | H | D | Q | I | R | L | F | R | N | R | I | C | P | G | E | V | E | Q | G | - | E | L | G | D | C | W | L | M | C | A | V | -39 |
| Q4CXJ8_TRYCC | - | - | - | - | - | - | - | - | - | - | P | R | M | Y | L | P | E | G | H | - | - | - | - | - | - | - | - | - | - | - | V | E | Q | V | R | L | F | R | R | P | I | F | P | G | D | V | E | Q | G | - | E | L | G | D | C | W | L | M | C | A | I | -39 |
| F4I0A4_ARATH | - | - | - | - | - | W | M | R | P | D | S | I | V | K | E | N | G | S | - | - | - | - | - | - | - | - | - | - | - | - | D | S | R | P | C | L | F | S | G | D | A | N | P | S | D | V | C | Q | G | - | R | L | G | D | C | W | F | L | S | A | V | -43 |
| Q8RUQ1_MAIZE | - | - | - | - | - | W | M | R | P | S | D | I | A | K | D | I | S | I | - | - | - | - | - | - | - | - | - | - | - | - | S | C | Q | P | C | L | F | S | G | S | V | N | S | S | D | V | C | Q | G | - | R | L | G | D | C | W | F | L | S | A | V | -43 |
| D7FQ17_ECTSI | - | - | - | - | - | W | K | V | S | L | A | V | N | P | D | I | R | - | - | - | - | - | - | - | - | - | - | - | - | - | - | - | - | - | - | L | F | D | D | G | T | D | P | D | D | V | R | R | G | - | A | F | A | D | S | W | L | L | S | A | L | -37 |
| D0MZ44_PHYIT | - | - | - | - | - | W | K | V | S | S | A | I | N | I | A | S | G | - | - | - | - | - | - | - | - | - | - | - | - | - | - | - | - | - | - | L | F | D | G | G | T | D | P | D | D | V | R | V | G | - | R | L | D | D | A | W | L | L | S | A | L | -37 |
| L1JBM0_GUITH | - | - | - | - | - | - | - | - | - | - | - | - | - | - | - | - | - | - | - | - | - | - | - | - | - | - | - | - | - | - | - | - | - | - | - | - | - | - | - | - | - | - | - | - | - | - | - | Q | G | - | R | L | R | D | C | W | Y | L | G | A | L | -13 |
| L1J739_GUITH | - | - | - | - | - | W | L | R | P | S | E | F | V | P | K | L | A | H | - | - | - | - | - | - | - | - | - | - | - | - | G | R | Q | P | Q | L | F | V | D | G | A | N | P | S | D | V | V | Q | G | - | A | L | G | D | C | W | F | L | S | A | L | -43 |
| L1J0U9_GUITH | - | - | - | - | - | W | I | R | P | H | Q | I | V | S | S | G | G | - | - | - | - | - | - | - | - | - | - | - | - | - | - | - | - | A | V | V | F | G | E | A | A | P | - | - | - | L | V | E | G | - | S | L | G | N | G | W | F | V | G | A | L | -36 |
| L1J1Q4_GUITH | - | - | - | - | - | W | F | R | P | E | E | F | V | P | A | D | Q | - | - | - | - | - | - | - | - | - | - | - | - | - | - | - | K | P | I | L | F | A | A | S | G | A | M | S | N | V | K | Q | G | - | M | L | K | N | C | H | F | L | G | A | L | -40 |
| D0ND88_PHYIT | - | - | - | - | - | W | Y | R | P | T | E | F | A | A | D | P | D | - | - | - | - | - | - | - | - | - | - | - | - | - | - | - | - | - | - | Y | F | K | S | S | A | G | C | G | D | L | R | E | G | - | L | L | N | D | A | W | L | I | G | V | F | -37 |
| D2VVS6_NAEGR | - | - | - | - | - | W | L | R | P | S | Q | I | T | G | V | T | K | P | - | - | - | - | - | - | - | - | - | - | - | - | - | - | - | - | A | L | F | V | D | G | S | N | Y | K | D | V | I | Q | G | - | S | L | G | N | C | W | M | L | S | A | M | -39 |
| D2V3V2_NAEGR | - | - | - | - | - | W | Y | R | T | W | D | L | C | F | L | R | E | E | S | - | - | - | - | - | - | - | - | - | - | - | - | - | - | - | Q | L | F | V | D | G | T | S | N | T | D | I | M | Q | G | - | G | I | G | D | C | W | L | L | S | A | F | -40 |
| Q5B5J7_EMENI | - | - | - | - | - | - | - | - | - | - | - | - | - | - | I | F | E | K | P | - | - | - | - | - | - | - | - | - | - | - | Q | F | Y | V | N | G | - | - | - | - | P | T | A | S | D | V | R | Q | G | - | R | D | G | D | C | W | F | M | A | A | L | -31 |
| E4ZY10_LEPMJ | - | - | - | - | - | - | - | - | - | K | R | V | E | D | I | F | L | Q | P | - | - | - | - | - | - | - | - | - | - | - | Q | F | Y | M | D | G | - | - | - | - | P | S | P | N | D | I | R | Q | G | - | N | D | G | D | C | W | F | M | S | A | L | -36 |
| A8PIE5_COPC7 | - | - | - | - | - | - | - | - | - | - | - | - | - | - | L | F | D | K | P | - | - | - | - | - | - | - | - | - | - | - | L | F | F | P | P | G | - | - | G | A | V | S | S | N | S | I | R | Q | G | - | G | L | G | D | C | W | F | L | S | A | L | -33 |
| A8NBA6_COPC7 | - | - | - | - | - | - | - | - | - | - | - | - | - | - | I | F | D | K | P | - | - | - | - | - | - | - | - | - | - | - | V | F | F | N | A | A | E | L | G | K | P | K | S | E | D | I | I | Q | G | - | A | L | G | D | C | W | F | L | S | A | L | -35 |
| A8NBA1_COPC7 | - | - | - | - | - | - | - | - | - | Q | R | I | S | E | I | F | D | R | P | - | - | - | - | - | - | - | - | - | - | - | K | F | F | N | E | K | - | - | G | A | A | E | S | S | A | I | V | Q | G | - | G | L | G | D | C | Y | F | L | A | A | L | -38 |
| A8NBA2_COPC7 | - | - | - | - | - | - | - | - | - | R | R | V | T | E | I | F | D | K | P | - | - | - | - | - | - | - | - | - | - | - | K | F | F | N | D | K | - | - | G | A | A | E | S | S | A | I | H | Q | G | - | C | I | G | D | C | Y | F | L | A | G | L | -38 |
| A8NAE6_COPC7 | - | - | - | - | - | - | - | - | - | R | R | V | T | E | L | F | D | K | P | - | - | - | - | - | - | - | - | - | - | - | I | T | F | F | S | G | - | H | G | G | P | G | A | A | E | I | V | Q | G | - | A | V | E | D | C | Y | F | V | S | A | L | -39 |
| A8NAI9_COPC7 | - | - | - | - | - | - | - | - | - | - | R | I | T | D | L | W | E | H | P | - | - | - | - | - | - | - | - | - | - | - | K | F | F | A | D | H | - | - | - | - | A | N | P | N | D | I | R | Q | G | - | T | L | G | D | C | W | F | L | S | A | L | -35 |
| G4TER7_PIRID | - | - | - | - | - | - | - | - | - | - | R | V | S | E | I | W | D | E | P | - | - | - | - | - | - | - | - | - | - | - | Q | F | T | I | D | G | - | - | - | - | F | S | S | S | D | V | A | Q | G | - | N | L | G | D | C | F | F | I | S | A | L | -35 |
| G4TER6_PIRID | - | - | - | - | - | - | - | - | - | - | - | - | - | - | - | - | - | D | P | - | - | - | - | - | - | - | - | - | - | - | E | F | D | L | E | - | - | - | - | - | N | D | E | R | L | C | L | Q | G | - | A | L | G | D | C | Y | F | I | S | A | L | -27 |
| G4TNA1_PIRID | - | - | - | - | - | - | - | - | - | - | R | V | T | E | I | F | D | N | P | - | - | - | - | - | - | - | - | - | - | - | V | L | F | K | D | G | - | - | - | - | A | N | A | S | D | I | A | Q | G | - | V | V | G | D | C | W | F | L | S | A | L | -35 |
| D0MWB9_PHYIT | - | - | - | - | - | - | - | - | - | - | - | - | - | - | - | A | S | N | A | - | - | - | - | - | - | - | - | - | - | - | K | L | F | A | D | G | - | - | - | - | V | S | S | G | D | V | I | Q | G | - | N | L | G | D | C | W | F | L | S | A | L | -30 |
| L1I9H5_GUITH | - | - | - | - | - | W | K | R | P | - | - | N | E | I | G | D | P | S | - | - | - | - | - | - | - | - | - | - | - | - | - | L | K | P | K | V | W | S | G | G | I | D | P | D | D | V | A | Q | G | - | R | L | G | N | C | Y | Y | L | A | A | I | -40 |
| Q7N497_PHOLL | - | - | - | - | - | - | - | - | - | - | - | - | - | - | - | - | - | - | - | - | - | - | - | - | - | - | - | - | - | - | - | - | - | P | G | M | F | I | K | G | I | E | M | I | D | M | G | Q | L | - | Y | L | G | N | C | G | F | V | S | T | I | -27 |
| ZP_11152369 | - | - | - | - | - | - | - | - | - | - | - | - | - | - | - | - | - | - | - | - | - | - | - | - | - | - | - | - | - | - | - | - | - | - | - | - | - | L | N | S | P | T | I | T | D | M | G | Q | K | - | Q | L | A | N | C | G | F | V | A | V | I | -23 |
| A2FEL7_TRIVA | - | - | - | - | - | W | V | R | A | E | Q | L | Y | E | K | Q | K | F | I | D | - | - | - | - | - | - | - | - | - | - | - | - | - | - | - | - | - | - | - | T | C | R | P | N | D | L | V | Q | G | - | Y | F | G | D | C | Y | V | V | T | A | M | -36 |
| A2D9A8_TRIVA | - | - | - | - | - | W | E | R | A | E | K | K | F | K | G | R | E | F | E | K | - | - | - | - | - | - | - | - | - | - | - | - | - | - | - | - | - | - | - | T | C | Q | P | N | E | I | V | Q | G | - | Y | F | G | D | C | Y | L | V | T | S | L | -36 |
| A2FHB3_TRIVA | - | - | - | - | - | W | K | R | A | E | D | E | F | K | - | E | A | P | T | G | - | - | - | - | - | - | - | - | - | - | - | - | - | - | - | - | - | - | - | H | V | N | A | N | D | L | C | Q | G | - | A | L | G | D | C | Y | L | V | S | A | M | -35 |
| A2DTE8_TRIVA | - | - | - | - | - | W | K | R | A | E | H | F | F | S | - | G | P | F | I | G | - | - | - | - | - | - | - | - | - | - | - | - | - | - | - | - | - | - | - | E | F | K | T | D | D | I | I | C | G | - | D | V | D | D | S | Y | L | M | A | V | L | -35 |
| A2E4X2_TRIVA | - | - | - | - | - | W | K | R | I | E | E | V | Y | G | - | T | N | I | F | D | - | - | - | - | - | - | - | - | - | - | - | - | - | - | - | - | - | - | - | D | I | N | E | Y | G | I | C | Q | G | - | E | L | S | D | C | Y | L | L | S | T | L | -35 |
| A2E1X1_TRIVA | - | - | - | - | - | - | - | - | - | - | - | - | - | N | - | P | D | F | L | S | - | - | - | - | - | - | - | - | - | - | - | - | - | - | - | - | - | - | - | N | I | S | S | D | S | I | F | Q | G | - | N | Y | Q | D | C | F | L | I | S | I | L | -27 |
| TPR_PORGI | - | - | - | - | - | - | - | - | - | - | - | - | - | - | P | T | K | N | L | R | D | D | N | N | T | V | H | Q | Y | R | E | V | G | F | Q | S | N | G | A | H | N | L | W | D | T | V | V | Q | G | - | I | A | G | D | C | Y | M | L | A | A | L | -46 |
| D5MF42_9BACT | - | - | - | - | - | - | - | - | - | - | - | - | - | - | - | - | - | - | - | - | - | - | - | - | - | - | - | - | - | - | - | - | - | - | - | - | - | - | - | - | - | - | - | - | - | - | V | Q | G | - | G | L | G | D | C | Y | F | I | A | A | L | -14 |
| R9H254_BACT4 | - | - | - | - | - | - | - | - | - | - | - | - | - | - | - | - | - | - | - | - | - | - | - | - | - | - | - | - | - | - | - | - | - | - | - | - | - | - | - | - | - | - | M | S | D | V | N | Q | R | - | W | V | G | D | C | C | A | C | A | V | I | -18 |
| ZP_04847694 | - | - | - | - | - | - | - | - | - | - | - | - | - | - | - | - | - | - | - | - | - | - | - | - | - | - | - | - | - | - | - | - | - | - | - | - | - | - | - | - | - | - | M | S | D | V | N | Q | R | - | W | V | G | D | C | C | A | C | A | V | I | -18 |
| D7IBA4_9BACE | - | - | - | - | - | - | - | - | - | - | - | - | - | - | - | - | - | - | - | - | - | - | - | - | - | - | - | - | - | - | - | - | - | - | - | - | - | - | - | - | - | - | - | - | D | V | N | Q | R | - | W | V | G | D | C | C | A | C | A | V | I | -16 |
| ZP_09858577 | - | - | - | - | - | - | - | - | - | - | - | - | - | - | - | - | - | - | - | - | - | - | - | - | - | - | - | - | - | - | - | - | - | - | - | - | - | - | - | - | - | - | - | - | D | V | N | Q | R | - | W | V | G | D | C | C | A | C | A | V | I | -16 |
| E5C759_9BACE | - | - | - | - | - | - | - | - | - | - | - | - | - | - | - | - | - | - | - | - | - | - | - | - | - | - | - | - | - | - | - | - | - | - | - | - | - | - | - | - | - | - | L | S | D | V | N | Q | R | - | W | V | G | D | C | C | A | C | A | V | F | -18 |
| A7LTS0_BACO1 | - | - | - | - | - | - | - | - | - | - | - | - | - | - | - | - | - | - | - | - | - | - | - | - | - | - | - | - | - | - | - | - | - | - | - | - | - | - | - | - | - | - | - | A | D | V | N | Q | H | - | S | I | G | D | C | C | L | C | A | V | M | -17 |
| Q8YQK8_NOSS1 | - | - | - | - | - | - | - | - | - | - | - | - | - | - | - | - | - | - | - | - | - | - | - | - | - | - | - | - | - | - | - | - | - | L | F | G | S | A | S | E | A | R | I | G | D | I | D | Q | G | - | G | L | G | D | C | A | F | L | A | A | L | -27 |
| Q3MBX8_ANAVT | - | - | - | - | - | - | - | - | - | - | - | - | - | - | - | - | - | - | - | - | - | - | - | - | - | - | - | - | - | - | - | - | - | L | F | G | S | A | N | E | A | R | I | G | D | I | D | Q | G | - | Q | L | G | D | C | A | F | L | A | A | L | -27 |
| Q8YZT8_NOSS1 | - | - | - | - | - | - | - | - | - | - | - | - | - | - | - | - | - | - | - | - | - | - | - | - | - | - | - | - | - | - | - | - | - | - | - | - | - | - | - | - | - | - | - | - | - | I | H | Q | G | - | I | I | N | D | C | T | F | L | A | A | L | -15 |
| K9UIG2_9CHRO | - | - | - | - | - | - | - | - | - | - | - | - | - | - | - | - | - | - | - | - | - | - | - | - | - | - | - | - | - | - | - | - | - | - | - | - | - | - | - | - | - | - | I | G | G | I | D | Q | R | - | S | F | G | N | C | V | L | L | A | A | L | -18 |
| A4YRP8_BRASO | - | - | - | - | - | - | - | - | - | - | - | - | - | - | - | - | - | - | - | - | - | - | - | - | - | - | - | - | - | - | - | - | - | - | - | - | - | - | - | G | A | S | M | S | D | I | N | Q | G | - | Y | L | G | D | C | Y | L | L | A | G | L | -21 |
| H0TAL7_9BRAD | - | - | - | - | - | - | - | - | - | - | - | - | - | - | - | - | - | - | - | - | - | - | - | - | - | - | - | - | - | - | - | - | - | - | - | F | G | A | S | G | A | S | I | S | D | I | N | Q | G | - | Y | L | G | D | C | Y | L | L | A | G | L | -25 |
| H0SNL4_9BRAD | - | - | - | - | - | - | - | - | - | - | - | - | - | - | - | - | - | - | - | - | - | - | - | - | - | - | - | - | - | - | - | - | - | P | L | F | S | A | A | G | A | S | M | N | D | I | N | Q | G | - | Y | L | G | D | C | Y | L | L | A | G | L | -27 |
| H0S4P6_9BRAD | - | - | - | - | - | - | - | - | - | - | - | - | - | - | - | - | - | - | - | - | - | - | - | - | - | - | - | - | - | - | - | - | - | P | L | F | S | A | A | G | A | S | M | S | D | I | N | Q | G | - | Y | L | G | D | C | Y | L | L | A | G | L | -27 |
| A5EMK2_BRASB | - | - | - | - | - | - | - | - | - | - | - | - | - | - | - | - | - | - | - | - | - | - | - | - | - | - | - | - | - | - | - | - | - | - | - | - | - | - | - | G | A | S | M | N | D | I | N | Q | G | - | Y | L | G | D | C | Y | L | L | A | G | L | -21 |
| M4ZBJ4_9BRAD | - | - | - | - | - | - | - | - | - | - | - | - | - | - | - | - | - | - | - | - | - | - | - | - | - | - | - | - | - | - | - | - | - | P | L | F | A | A | A | G | A | G | I | N | D | I | N | Q | G | - | Y | L | G | D | C | Y | L | L | S | G | L | -27 |
| Q07SP7_RHOP5 | - | - | - | - | - | - | - | - | - | - | - | - | - | - | - | - | - | - | - | - | - | - | - | - | - | - | - | - | - | - | - | - | - | - | - | - | - | - | D | G | Y | G | M | D | D | V | N | Q | G | - | Y | L | G | N | C | Y | F | L | A | P | L | -22 |
| B0JUQ7_MICAN | - | - | - | - | - | - | - | - | - | - | - | - | - | - | - | - | - | - | - | - | - | - | - | - | - | - | - | - | - | - | - | - | - | - | - | L | L | Q | S | G | V | S | Y | Q | D | I | N | Q | G | - | N | L | G | D | C | Y | L | L | A | A | L | -25 |
| K9WJ87_9CYAN | - | - | - | - | - | - | - | - | - | - | - | - | - | - | - | - | - | - | - | - | - | - | - | - | - | - | - | - | - | - | - | - | - | - | - | - | - | - | - | G | I | S | Y | Q | D | I | D | Q | G | - | G | V | G | D | C | Y | F | V | A | S | L | -21 |
| G6FUE0_9CYAN | - | - | - | - | - | - | - | - | - | - | - | - | - | - | - | - | - | - | - | - | - | - | - | - | - | - | - | - | - | - | - | - | - | - | - | - | - | - | - | G | I | T | Y | Q | D | I | K | Q | G | - | S | I | N | D | C | F | F | L | T | G | L | -21 |
| K9PHG5_9CYAN | - | - | - | - | - | - | - | - | - | - | - | - | - | - | - | - | - | - | - | - | - | - | - | - | - | - | - | - | - | - | - | - | - | - | - | - | - | - | - | G | V | S | Y | Q | D | I | S | Q | G | - | N | V | G | D | C | Y | F | L | A | G | L | -21 |
| Q8YXX3_NOSS1 | - | - | - | - | - | - | - | - | - | - | - | - | - | - | - | - | - | - | - | - | - | - | - | - | - | - | - | - | - | - | - | - | - | - | - | L | F | Q | N | G | I | S | Y | Q | D | V | D | Q | G | - | A | I | G | N | C | Y | F | L | A | G | L | -25 |
| K9TN70_9CYAN | - | - | - | - | - | - | - | - | - | - | - | - | - | - | - | - | - | - | - | - | - | - | - | - | - | - | - | - | - | - | - | - | - | - | - | - | - | - | - | G | I | D | Y | T | D | V | R | Q | G | - | Y | L | G | D | C | Y | Y | L | A | A | L | -21 |
| Q05QW3_9SYNE | - | - | - | - | - | - | - | - | - | - | - | - | - | - | - | - | - | - | - | - | - | - | - | - | - | - | - | - | - | - | - | - | - | - | - | L | F | M | D | G | V | S | F | T | D | I | D | Q | G | - | Q | T | G | D | C | Y | L | L | A | P | L | -25 |
| Q05QW2_9SYNE | - | - | - | - | - | - | - | - | - | - | - | - | - | - | - | - | - | - | - | - | - | - | - | - | - | - | - | - | - | - | - | - | - | - | - | L | F | V | D | G | I | D | A | G | D | I | D | Q | G | - | A | T | G | S | C | Y | F | L | S | A | L | -25 |
| Q05QW4_9SYNE | - | - | - | - | - | - | - | - | - | - | - | - | - | - | - | - | - | - | - | - | - | - | - | - | - | - | - | - | - | - | - | - | - | - | - | L | F | V | D | G | V | S | A | D | D | I | D | Q | G | - | G | V | G | S | C | Y | F | L | S | A | L | -25 |
| E0UGX6_CYAP2 | - | - | - | - | - | - | - | - | - | - | - | - | - | - | - | - | - | - | - | - | - | - | - | - | - | - | - | - | - | - | - | - | - | - | - | L | F | Q | N | G | V | S | A | D | D | I | K | Q | G | - | A | L | G | D | C | Y | Y | L | A | T | L | -25 |
| K7WKF4_9NOST | - | - | - | - | - | - | - | - | - | - | - | - | - | - | - | - | - | - | - | - | - | - | - | - | - | - | - | - | - | - | - | - | - | - | - | - | - | - | - | G | A | S | A | D | D | I | Y | Q | G | - | A | V | G | D | C | Y | Y | V | A | T | L | -21 |
| E0UGY5_CYAP2 | - | - | - | - | - | - | - | - | - | - | - | - | - | - | - | - | - | - | - | - | - | - | - | - | - | - | - | - | - | - | - | - | - | - | - | L | F | Q | N | N | I | N | A | N | D | I | R | Q | G | - | L | V | G | D | C | Y | Y | L | S | S | L | -25 |
| A8LCE5_FRASN | - | - | - | - | - | - | - | - | - | - | - | - | - | - | - | - | - | - | - | - | - | - | - | - | - | - | - | - | - | - | H | G | R | D | P | V | W | A | S | G | V | T | P | S | D | V | G | Q | G | - | A | V | G | D | C | Y | L | L | A | A | L | -30 |
| D3CT47_9ACTO | - | - | - | - | - | - | - | - | - | - | - | - | - | - | - | - | - | - | - | - | - | - | - | - | - | - | - | - | - | - | H | T | G | E | P | V | W | V | R | G | A | T | P | S | D | V | R | Q | G | - | R | V | G | D | C | Y | L | L | A | A | L | -30 |
| Q2J4J6_FRASC | - | - | - | - | - | - | - | - | - | - | - | - | - | - | - | - | - | - | - | - | - | - | - | - | - | - | - | - | - | - | - | - | - | D | P | T | V | R | D | G | I | S | P | S | D | V | G | Q | G | - | G | V | A | D | C | Y | L | V | A | A | L | -27 |
| Q0RAZ2_FRAAA | - | - | - | - | - | - | - | - | - | - | - | - | - | - | - | - | - | - | - | - | - | - | - | - | - | - | - | - | - | - | - | - | - | D | A | I | V | R | D | G | A | D | P | G | D | V | G | Q | G | - | A | V | A | D | C | Y | L | A | A | A | L | -27 |
| E3J3F6_FRASU | - | - | - | - | - | - | - | - | - | - | - | - | - | - | - | - | - | - | - | - | - | - | - | - | - | - | - | - | - | - | - | - | - | D | A | V | V | R | D | G | I | S | S | S | D | V | G | Q | G | - | G | V | D | D | C | Y | L | A | A | A | L | -27 |
| ZP_09168240 | - | - | - | - | - | - | - | - | - | - | - | - | - | - | - | - | - | - | - | - | - | - | - | - | - | - | - | - | - | - | - | - | A | D | P | V | V | R | D | G | V | S | T | S | D | V | G | Q | G | - | G | L | G | D | C | Y | L | A | A | T | L | -28 |
| C7MDV9_BRAFD | - | - | - | - | - | - | - | - | - | - | - | - | - | - | - | - | - | - | - | - | - | - | - | - | - | - | - | - | - | - | - | - | - | - | - | - | - | - | - | - | - | - | - | - | - | - | - | Q | G | - | R | L | G | D | C | W | V | I | A | P | M | -13 |
| ZP_10094989 | - | - | - | - | - | - | - | - | - | - | - | - | - | - | - | - | - | - | - | - | - | - | - | - | - | - | - | - | - | - | - | - | - | - | - | - | - | - | - | - | - | - | - | - | - | - | - | Q | G | - | R | L | G | D | C | W | L | M | A | P | M | -13 |
| A4X268_SALTO | - | - | - | - | - | - | - | - | - | - | - | - | - | - | - | - | - | - | - | - | - | - | - | - | - | - | - | - | - | - | - | - | - | - | - | - | - | - | - | - | - | - | - | - | - | V | Q | Q | G | - | K | L | G | D | C | G | M | L | A | S | M | -15 |
| WP_043162213 | - | - | - | - | - | - | - | - | - | - | - | - | - | - | - | - | - | - | - | - | - | - | - | - | - | - | - | - | - | - | - | - | - | - | - | - | - | - | - | - | - | - | - | - | - | V | Q | Q | G | - | K | L | G | D | C | G | M | L | A | S | M | -15 |
| Q0BRV8_GRABC | - | - | - | - | - | - | - | - | - | - | - | - | - | - | - | - | - | - | - | - | - | - | - | - | - | - | - | - | - | - | - | - | - | E | P | G | D | S | T | A | I | S | Y | T | D | I | H | Q | G | - | Q | I | G | D | C | Y | V | L | S | S | I | -27 |
| A9B2P0_HERA2 | - | - | - | - | - | - | - | - | - | - | - | - | - | - | - | - | - | - | - | - | - | - | - | - | - | - | - | - | - | - | - | - | - | - | - | - | - | - | - | - | I | A | I | D | D | V | K | Q | G | - | Q | L | G | D | C | Y | L | M | A | G | T | -20 |
| A9B3U8_HERA2 | - | - | - | - | - | - | - | - | - | - | - | - | - | - | - | - | - | - | - | - | - | - | - | - | - | - | - | - | - | - | - | - | - | - | - | - | - | - | - | - | I | A | M | D | D | V | K | Q | G | - | L | L | G | D | C | Y | L | M | A | G | L | -20 |
| A9B2A3_HERA2 | - | - | - | - | - | - | - | - | - | - | - | - | - | - | - | - | - | - | - | - | - | - | - | - | - | - | - | - | - | - | - | - | - | - | - | - | - | - | - | G | I | H | P | S | D | A | D | Q | G | - | Q | I | G | N | C | F | V | V | A | S | L | -21 |
| D6ZC85_SEGRD | - | - | - | - | - | - | - | - | - | - | - | - | - | - | - | - | - | - | - | - | - | - | - | - | - | - | - | - | - | - | - | - | - | - | - | - | - | S | A | G | P | S | P | E | D | I | R | Q | K | - | N | I | G | D | C | Y | F | D | S | T | L | -23 |
| E5XTT9_9ACTO | - | - | - | - | - | - | - | - | - | - | - | - | - | - | - | - | - | - | - | - | - | - | - | - | - | - | - | - | - | - | - | - | - | - | - | - | - | S | S | G | P | G | A | E | D | V | R | Q | S | - | S | V | G | D | C | Y | F | D | A | A | M | -23 |
| E5XTU0_9ACTO | - | - | - | - | - | - | - | - | - | - | - | - | - | - | - | - | - | - | - | - | - | - | - | - | - | - | - | - | - | - | - | - | - | - | - | - | - | T | T | G | P | N | A | N | D | V | H | Q | S | - | G | I | G | D | C | Y | F | D | A | S | M | -23 |
| D6B0I6_9ACTO | - | - | - | - | - | - | - | - | - | - | - | - | - | - | - | - | - | - | - | - | - | - | - | - | - | - | - | - | - | - | - | - | - | D | P | L | F | R | D | G | V | S | A | D | D | V | S | Q | N | - | Q | F | G | D | C | W | Y | V | A | S | L | -27 |
| D9X719_STRVR | - | - | - | - | - | - | - | - | - | - | - | - | - | - | - | - | - | - | - | - | - | - | - | - | - | - | - | - | - | - | - | - | - | D | P | L | F | Q | D | G | V | S | A | D | D | V | N | Q | R | - | Q | F | G | D | C | W | Y | V | A | S | L | -27 |
| H6L010_SAPGL | - | - | - | - | - | - | - | - | - | - | - | - | - | - | - | - | - | - | - | - | - | - | - | - | - | - | - | - | - | - | - | - | - | - | - | - | - | - | - | - | - | - | - | N | D | V | Q | Q | G | - | Y | L | G | D | C | Y | F | L | A | A | V | -17 |
| K1ZP83_9BACT | - | - | - | - | - | - | - | - | - | - | - | - | - | - | - | - | - | - | - | - | - | - | - | - | - | - | - | - | - | - | - | - | - | - | - | L | I | L | N | G | I | E | G | D | D | V | H | Q | G | - | Q | V | G | D | C | Y | F | L | A | A | L | -25 |
| ADGB_HUMAN | - | - | - | - | - | W | K | R | P | - | - | Q | D | I | L | F | S | - | - | - | Q | T | P | V | V | V | K | N | E | I | T | F | D | L | F | S | A | N | E | H | L | L | C | S | E | L | M | R | W | - | I | I | S | E | I | Y | A | V | W | K | I | -50 |
| J3QMP3_MOUSE | - | - | - | - | - | W | K | R | P | - | - | Q | D | F | I | F | S | - | - | - | R | T | P | V | V | V | K | N | E | I | T | F | D | L | F | S | P | N | E | H | L | L | C | S | E | L | M | R | W | - | I | I | S | E | I | Y | A | V | W | K | I | -50 |
| H2XR46_CIOIN | - | - | - | - | - | W | K | R | P | - | - | Q | D | F | M | L | G | - | - | - | K | I | P | V | V | V | E | N | E | S | E | F | N | L | L | Q | Y | N | E | H | I | C | H | S | Q | L | M | R | S | - | I | I | T | Q | V | T | A | L | W | E | M | -50 |
| CAN7_MOUSE | - | - | - | - | - | W | V | R | P | - | - | E | D | L | T | N | - | - | - | - | - | N | P | T | M | I | Y | T | - | - | - | - | - | - | V | S | S | F | S | - | - | - | - | - | - | I | K | Q | T | - | I | V | S | D | C | S | F | V | A | S | L | -36 |
| CAN7_HUMAN | - | - | - | - | - | W | V | R | P | - | - | E | D | L | T | N | - | - | - | - | - | N | P | T | M | I | Y | T | - | - | - | - | - | - | V | S | S | F | S | - | - | - | - | - | - | I | K | Q | T | - | I | V | S | D | C | S | F | V | A | S | L | -36 |
| E1BY84_CHICK | - | - | - | - | - | W | V | R | P | - | - | D | D | I | T | N | - | - | - | - | - | N | P | T | M | I | Y | T | - | - | - | - | - | - | V | S | S | F | S | - | - | - | - | - | - | I | K | Q | T | - | I | V | S | D | C | S | F | V | A | S | L | -36 |
| H9GX91_DANRE | - | - | - | - | - | W | V | R | P | - | - | D | D | I | C | N | - | - | - | - | - | N | P | T | M | I | F | T | - | - | - | - | - | - | V | S | S | F | S | - | - | - | - | - | - | I | K | Q | T | - | V | V | S | D | C | S | F | V | A | S | L | -36 |
| F6SGI6_CIOIN | - | - | - | - | - | W | K | R | P | - | - | D | E | F | M | N | - | - | - | - | - | E | P | Q | M | I | L | T | - | - | - | - | - | - | V | S | S | L | S | - | - | - | - | - | - | I | M | Q | T | - | V | V | S | D | C | S | F | V | A | S | L | -36 |
| W4Y195_STRPU | - | - | - | - | - | W | V | R | P | - | - | S | D | I | C | S | - | - | - | - | - | D | P | K | I | I | Y | A | - | - | - | - | - | - | V | S | S | F | S | - | - | - | - | - | - | V | K | Q | - | - | - | - | - | - | - | - | - | - | - | - | - | -24 |
| R7V270_CAPTE | - | - | - | - | - | W | A | R | P | - | - | D | E | Y | M | S | - | - | - | - | - | D | P | S | M | V | Y | A | - | - | - | - | - | - | V | S | P | L | A | - | - | - | - | - | - | I | K | Q | S | - | I | V | S | D | C | S | F | V | A | S | L | -36 |
| G4LXK3_SCHMA | - | - | - | - | - | W | V | R | P | - | - | S | D | Y | I | S | - | - | - | - | - | G | P | K | M | I | L | A | - | - | - | - | - | - | I | S | C | F | S | - | - | - | - | - | - | I | C | Q | T | - | V | V | T | D | C | S | F | V | A | S | M | -36 |
| T2M2K0_HYDVU | - | - | - | - | - | W | V | R | P | - | - | S | D | I | C | E | - | - | - | - | - | N | P | Q | M | I | F | A | - | - | - | - | - | - | V | S | S | L | S | - | - | - | - | - | - | I | K | Q | T | - | I | V | S | D | C | S | F | V | A | S | L | -36 |
| B3RQK1_TRIAD | - | - | - | - | - | W | V | R | P | - | - | Y | D | L | S | Q | - | - | - | - | - | D | P | K | I | I | H | V | - | - | - | - | - | - | I | S | S | F | S | - | - | - | - | - | - | I | R | Q | T | - | I | V | S | D | C | S | F | V | A | S | L | -36 |
| I1CS59_RHIO9 | - | - | - | - | - | W | K | R | P | - | - | S | D | I | M | R | - | - | - | - | - | D | P | K | L | I | C | C | - | - | - | - | - | - | I | S | S | T | S | - | - | - | - | - | - | I | I | Q | D | - | I | V | T | D | C | S | F | V | A | S | L | -36 |
| I1GER3_AMPQE | - | - | - | - | - | W | Q | R | P | - | - | G | E | I | C | D | - | - | - | - | - | N | P | K | M | C | H | L | - | - | - | - | - | - | I | S | S | L | S | - | - | - | - | - | - | I | K | Q | T | - | M | I | G | D | C | S | F | V | A | S | L | -36 |
| Q22143_CAEEL | - | - | - | - | - | W | K | R | V | - | - | S | E | L | F | E | - | - | - | - | - | N | P | T | I | I | F | S | - | - | - | - | - | - | I | D | C | H | T | - | - | - | - | - | - | I | K | Q | T | - | V | I | S | D | C | S | F | I | S | S | L | -36 |
| L1JZN7_GUITH | - | - | - | - | - | W | A | R | I | - | - | S | Q | I | Y | S | - | - | - | - | - | N | P | V | V | I | K | Q | - | - | - | - | - | - | F | T | Y | E | N | - | - | - | - | - | - | I | T | Q | T | - | L | V | G | D | C | S | F | V | S | S | L | -36 |
| D0NT34_PHYIT | - | - | - | - | - | W | M | R | P | - | - | S | D | C | A | A | K | C | G | Y | E | A | P | V | M | I | A | D | G | G | - | - | - | - | V | N | P | L | V | - | - | - | - | - | - | V | K | Q | D | - | I | V | T | D | C | S | F | V | A | S | L | -43 |
| A0A064B913_ASPOZ | - | - | - | - | - | W | K | R | P | - | - | S | E | L | L | A | K | - | - | - | - | D | T | E | D | V | Y | T | K | V | - | - | - | - | V | P | V | M | S | V | P | G | K | T | D | L | V | Q | D | - | M | L | T | D | C | S | V | V | A | S | L | -45 |
| A8N4X4_COPC7 | - | - | - | - | - | W | K | R | P | - | - | M | H | E | F | M | S | - | - | - | - | - | E | E | M | G | S | T | C | D | - | - | - | - | W | E | E | T | P | R | I | L | P | Q | D | I | R | Q | H | - | V | V | S | D | C | S | V | C | A | S | I | -44 |
| C0H5K1_PLAF7 | - | - | - | - | - | W | K | R | L | - | - | S | E | L | Y | E | N | - | - | - | - | - | - | P | I | V | M | S | P | Y | - | - | - | - | L | Y | N | N | C | - | - | - | - | - | - | I | R | Q | G | - | F | V | A | D | C | S | F | L | S | S | L | -37 |
| K1REZ8_CRAGI | - | - | - | - | - | - | - | - | - | - | - | - | - | - | - | - | - | - | - | - | - | - | - | - | - | - | - | - | - | - | - | - | - | - | - | - | L | C | K | D | P | E | N | P | D | F | N | Q | G | - | E | L | D | T | S | Y | F | L | T | V | I | -24 |
| F4NTM9_BATDJ | - | - | - | - | - | W | V | R | A | T | E | M | I | H | N | S | - | - | - | - | - | - | - | - | - | - | - | - | - | - | - | - | - | - | H | L | I | T | Q | Y | E | V | C | N | N | V | I | Q | G | - | - | A | F | G | G | W | T | V | A | A | A | -36 |
| Q6F6K5_CAEEL | - | - | - | - | - | - | - | - | - | - | - | - | - | - | - | - | - | - | - | - | - | - | - | - | - | - | - | - | - | - | - | - | - | - | - | - | - | - | - | - | - | - | - | - | V | F | Q | L | G | - | - | V | G | D | C | W | L | I | A | A | L | -15 |
| A8HZK8_CHLRE | - | - | - | - | - | - | - | - | - | - | - | - | - | - | - | - | - | - | - | - | - | - | - | - | - | - | - | - | - | - | - | - | - | - | - | - | - | - | - | - | - | - | - | - | - | - | - | Q | G | - | V | L | E | S | G | P | F | L | G | A | L | -13 |
| A8J6T6_CHLRE | - | - | - | - | - | W | M | R | L | K | D | I | A | A | P | E | Q | F | R | K | G | - | - | - | - | - | - | - | - | - | T | R | G | L | K | L | F | T | G | G | G | T | H | P | D | D | I | D | G | - | - | - | S | S | S | G | L | M | V | A | V | -44 |
| Z9JR51_9MICO | - | - | - | - | - | - | - | - | - | - | - | - | - | - | - | - | - | - | - | - | - | - | - | - | - | - | - | - | - | D | P | E | D | I | G | I | T | E | E | D | I | T | A | A | E | M | Q | Q | G | - | T | L | G | D | C | W | Y | L | A | A | I | -31 |
| F7ZZH9_CELGA | - | - | - | - | - | - | - | - | - | - | - | - | - | - | - | - | - | - | - | - | - | - | - | - | - | - | - | - | - | - | - | - | - | - | - | - | - | - | - | - | - | - | - | - | D | V | A | Q | G | - | S | I | G | D | C | F | M | L | S | S | V | -16 |
| Q7NDU2_GLOVI | - | - | - | - | - | - | - | - | - | - | - | - | - | - | - | - | - | - | - | - | - | - | - | - | - | - | - | - | - | - | - | - | - | - | - | - | - | - | - | - | I | E | A | A | D | V | K | Q | G | - | N | A | G | T | C | N | P | L | S | A | L | -20 |
| I6AT95_9BACT | - | - | - | - | - | - | - | - | - | - | - | - | - | - | - | - | - | - | - | - | - | - | - | - | - | - | - | - | - | - | - | - | - | - | - | - | - | - | - | - | - | S | L | E | T | L | H | Q | G | - | R | L | G | D | C | F | C | L | A | P | L | -19 |
| C7MGJ8_BRAFD | - | - | - | - | - | - | - | - | - | - | - | - | - | - | - | - | - | - | - | - | - | - | - | - | - | - | - | - | - | - | - | - | - | - | - | - | - | - | - | - | - | - | - | - | D | V | N | Q | Q | - | G | Y | G | D | C | W | M | L | A | N | L | -16 |
| D0WP73_9ACTO | - | - | - | - | - | - | - | - | - | - | - | - | - | - | - | - | - | - | - | - | - | - | - | - | - | - | - | - | - | - | - | - | - | - | - | - | - | - | - | - | M | S | Y | E | D | I | Q | Q | H | - | G | I | G | D | C | Y | Y | L | A | A | I | -20 |
| B0C8L1_ACAM1 | - | - | - | - | - | - | - | - | - | - | - | - | - | - | - | - | - | - | - | - | - | - | - | - | - | - | - | - | - | - | - | - | - | - | - | - | - | - | - | - | - | - | P | E | D | I | K | Q | G | - | P | I | G | D | C | F | L | L | A | V | A | -18 |
| B3E5A8_GEOLS | - | - | - | - | - | - | - | - | - | - | - | - | - | - | - | - | - | - | - | - | - | - | - | - | - | - | - | - | - | - | - | - | - | - | - | - | - | - | - | - | - | - | T | F | E | L | L | Q | Q | G | P | V | G | D | C | Y | F | F | S | A | S | -19 |
| D4YQ94_9MICO | - | - | - | - | - | - | - | - | - | - | - | - | - | - | - | - | - | - | - | - | - | - | - | - | - | - | - | - | - | - | - | - | - | - | - | - | - | - | - | - | - | - | - | T | Q | V | R | Q | G | - | Q | L | G | D | C | W | L | I | A | T | L | -17 |
| PAPA_CARPA | - | - | - | - | - | - | - | - | - | - | - | - | - | - | - | - | - | - | - | - | - | - | - | - | - | - | - | - | - | - | I | P | E | Y | V | D | W | R | Q | K | G | A | V | T | P | V | K | N | Q | G | S | C | G | S | C | W | A | F | S | A | V | -31 |
| RIM13_YEAST | - | S | P | V | S | W | N | K | T | E | Y | C | A | P | G | T | E | E | L | Q | R | R | Y | P | G | - | - | - | - | - | - | - | R | A | K | L | Q | N | E | E | D | Y | S | G | G | I | E | Q | C | R | D | V | P | D | C | S | L | V | A | S | L | -53 |

| I |  |  |  |  |  |  |  |  |  |  |  |  |  |  |  |  |  |  |  |  |  |  |  |  |  |  |  |  |  |  |  |  |  |  |  |  |  |  |  |  |  |  |  |  |  |  |  |  |  |  |  |  |  |  |  |  |  |  |  |  |  |  |
| --- | --- | --- | --- | --- | --- | --- | --- | --- | --- | --- | --- | --- | --- | --- | --- | --- | --- | --- | --- | --- | --- | --- | --- | --- | --- | --- | --- | --- | --- | --- | --- | --- | --- | --- | --- | --- | --- | --- | --- | --- | --- | --- | --- | --- | --- | --- | --- | --- | --- | --- | --- | --- | --- | --- | --- | --- | --- | --- | --- | --- | --- | --- |
| Q9N4B1_CAEEL | - | A | N | L | T | L | R | D | - | - | - | - | - | - | - | - | - | E | L | F | Y | R | V | V | - | - | - | - | - | - | - | - | - | - | - | - | P | P | D | Q | S | F | T | E | N | - | - | - | - | - | - | - | - | - | - | - | - | - | Y | A | G | -63 |
| Q8IAA8_CAEEL | - | A | N | I | T | L | Y | D | - | - | - | - | - | - | - | - | - | A | L | F | Y | R | I | V | - | - | - | - | - | - | - | - | - | - | - | - | P | P | N | Q | S | F | T | E | N | - | - | - | - | - | - | - | - | - | - | - | - | - | Y | A | G | -63 |
| CAN_CAEEL | - | A | N | L | T | L | K | D | - | - | - | - | - | - | - | - | - | E | L | F | Y | R | V | V | - | - | - | - | - | - | - | - | - | - | - | - | P | P | D | Q | S | F | T | E | N | - | - | - | - | - | - | - | - | - | - | - | - | - | Y | A | G | -63 |
| Q9U2B2_CAEEL | - | A | N | L | S | L | R | D | - | - | - | - | - | - | - | - | - | E | L | F | Y | R | V | V | - | - | - | - | - | - | - | - | - | - | - | - | P | P | D | Q | S | F | T | E | K | - | - | - | - | - | - | - | - | - | - | - | - | - | Y | A | G | -63 |
| Q9N4B2_CAEEL | - | A | T | L | A | Q | R | D | - | - | - | - | - | - | - | - | - | E | L | F | Y | R | V | V | - | - | - | - | - | - | - | - | - | - | - | - | P | P | D | Q | S | F | T | E | N | - | - | - | - | - | - | - | - | - | - | - | - | - | Y | A | G | -63 |
| C3Y6P6_BRAFL | - | A | A | L | T | T | L | P | - | - | - | - | - | - | - | - | - | G | L | F | K | R | V | V | - | - | - | - | - | - | - | - | - | - | - | - | P | S | G | Q | G | F | Q | D | N | - | - | - | - | - | - | - | - | - | - | - | - | - | Y | A | G | -63 |
| C3Y6N1_BRAFL | - | A | T | L | A | Q | K | P | - | - | - | - | - | - | - | - | - | Y | L | F | K | R | V | C | - | - | - | - | - | - | - | - | - | - | - | - | C | E | D | N | S | Y | S | D | D | - | - | - | - | - | - | - | - | - | - | - | - | - | Y | C | G | -65 |
| CANA_DROME | - | A | N | L | T | Q | E | S | - | - | - | - | - | - | - | - | - | N | L | F | F | R | V | I | - | - | - | - | - | - | - | - | - | - | - | - | P | A | E | Q | S | F | E | E | N | - | - | - | - | - | - | - | - | - | - | - | - | - | Y | A | G | -63 |
| CANB_DROME | - | A | N | L | T | Q | D | S | - | - | - | - | - | - | - | - | - | T | L | F | F | R | V | I | - | - | - | - | - | - | - | - | - | - | - | - | P | P | D | Q | D | F | Q | E | N | - | - | - | - | - | - | - | - | - | - | - | - | - | Y | A | G | -63 |
| X1Y3H71_ANODA | - | A | N | L | T | Q | D | Q | - | - | - | - | - | - | - | - | - | K | M | F | L | R | V | V | - | - | - | - | - | - | - | - | - | - | - | - | P | E | D | N | S | F | E | E | N | - | - | - | - | - | - | - | - | - | - | - | - | - | Y | A | G | -63 |
| Q5IW12_GECLA | - | A | N | L | T | L | N | R | - | - | - | - | - | - | - | - | - | R | L | F | Y | Q | I | V | - | - | - | - | - | - | - | - | - | - | - | - | P | S | D | Q | G | F | G | D | N | - | - | - | - | - | - | - | - | - | - | - | - | - | Y | A | G | -63 |
| K1Q056_CRAGI | - | A | S | L | T | V | N | Q | - | - | - | - | - | - | - | - | - | R | L | F | A | K | V | V | - | - | - | - | - | - | - | - | - | - | - | - | P | P | D | Q | S | F | E | D | G | - | - | - | - | - | - | - | - | - | - | - | - | - | Y | C | G | -63 |
| G4V6C0_SCHMA | - | A | S | L | S | M | Y | P | - | - | - | - | - | - | - | - | - | E | L | F | N | Q | V | V | - | - | - | - | - | - | - | - | - | - | - | - | P | P | D | Q | S | F | E | K | S | S | K | - | - | - | - | - | - | - | - | - | Y | P | Y | V | G | -67 |
| R7ULX9_CAPTE | - | S | C | L | A | T | H | P | - | - | - | - | - | - | - | - | - | R | L | L | K | R | V | V | - | - | - | - | - | - | - | - | - | - | - | - | P | E | D | Q | S | F | Q | E | D | - | - | - | - | - | - | - | - | - | - | - | - | - | Y | A | G | -63 |
| W4Z5Q7_STRPU | - | A | G | L | S | L | D | D | - | - | - | - | - | - | - | - | - | H | L | L | K | K | V | I | - | - | - | - | - | - | - | - | - | - | - | - | P | P | D | Q | N | F | D | L | G | - | - | - | - | - | - | - | - | - | - | - | - | - | - | - | - | -60 |
| R7VIB6_CAPTE | - | G | S | L | C | A | T | K | - | - | - | - | - | - | - | S | K | E | L | L | H | R | V | I | - | - | - | - | - | - | - | - | - | - | - | - | P | L | D | N | S | F | A | K | G | - | - | - | - | - | - | - | - | - | - | - | - | Q | Y | A | G | -66 |
| T2MJ45_HYDVU | - | A | A | L | C | Q | Y | E | - | - | - | - | - | - | - | - | - | G | L | L | Y | Q | V | V | - | - | - | - | - | - | - | - | - | - | - | - | P | P | D | Q | D | F | K | T | - | - | - | - | - | - | - | - | - | - | - | - | - | N | Y | C | G | -63 |
| R7UAB6_CAPTE | - | S | G | L | C | G | C | K | - | - | - | - | - | - | - | - | - | E | L | L | Y | R | V | V | - | - | - | - | - | - | - | - | - | - | - | - | P | P | D | Q | D | F | T | T | - | - | - | - | - | - | - | - | - | - | - | - | - | K | Y | C | G | -63 |
| C3ZKN8_BRAFL | - | A | C | L | S | M | N | E | - | - | - | - | - | - | - | - | - | S | L | L | F | G | V | V | - | - | - | - | - | - | - | - | - | - | - | - | P | P | D | Q | S | F | D | K | G | - | - | - | - | - | - | - | - | - | - | - | - | D | Y | V | G | -64 |
| B3RJS5_TRIAD | - | S | S | L | A | G | E | K | - | - | - | - | - | - | - | - | - | S | L | L | Y | R | V | V | - | - | - | - | - | - | - | - | - | - | - | - | P | P | D | Q | S | F | S | R | - | - | - | - | - | - | - | - | - | - | - | - | - | N | Y | A | G | -63 |
| H2YVF0_CIOSA | - | A | S | L | S | L | N | D | - | - | - | - | - | - | - | - | - | D | L | L | Y | R | I | I | - | - | - | - | - | - | - | - | - | - | - | - | P | P | D | Q | S | F | S | Q | - | - | - | - | - | - | - | - | - | - | - | - | - | D | Y | C | G | -63 |
| G4VAG2_SCHMA | - | A | S | I | S | G | Y | P | - | - | - | - | - | - | - | - | - | Q | L | F | D | Q | V | V | - | - | - | - | - | - | - | - | - | - | - | - | P | K | D | Q | E | L | K | G | P | - | - | - | - | - | - | - | - | - | - | - | - | E | Y | V | G | -64 |
| Smp_157500 | - | A | T | V | T | K | Y | P | - | - | - | - | - | - | - | - | - | S | L | M | S | N | I | I | - | - | - | - | - | - | - | - | - | - | - | - | P | P | N | Q | T | F | K | G | S | - | - | - | - | - | - | - | - | - | - | - | - | G | Y | T | G | -64 |
| Q7SYA9_DANRE | - | G | S | L | T | L | - | - | - | - | - | - | - | - | - | N | E | R | L | L | H | R | V | V | - | - | - | - | - | - | - | - | - | - | - | - | P | H | G | Q | S | F | Q | D | - | - | - | - | - | - | - | - | - | - | - | - | - | D | Y | A | G | -63 |
| A5PMP1_DANRE | - | A | S | L | T | L | - | - | - | - | - | - | - | - | - | N | D | K | L | L | H | R | V | V | - | - | - | - | - | - | - | - | - | - | - | - | P | H | G | Q | S | F | T | N | - | - | - | - | - | - | - | - | - | - | - | - | - | E | Y | A | G | -63 |
| CANX_CHICK | - | G | S | L | T | L | - | - | - | - | - | - | - | - | - | N | E | E | L | L | H | R | V | V | - | - | - | - | - | - | - | - | - | - | - | - | P | H | G | Q | S | F | Q | E | - | - | - | - | - | - | - | - | - | - | - | - | - | D | Y | A | G | -63 |
| CAN1_HUMAN | - | A | S | L | T | L | - | - | - | - | - | - | - | - | - | N | D | T | L | L | H | R | V | V | - | - | - | - | - | - | - | - | - | - | - | - | P | H | G | Q | S | F | Q | N | - | - | - | - | - | - | - | - | - | - | - | - | - | G | Y | A | G | -63 |
| CAN1_MOUSE | - | A | S | L | T | L | - | - | - | - | - | - | - | - | - | N | E | T | I | L | H | R | V | V | - | - | - | - | - | - | - | - | - | - | - | - | P | Y | G | Q | S | F | Q | D | - | - | - | - | - | - | - | - | - | - | - | - | - | G | Y | A | G | -63 |
| O42133_CHICK | - | A | S | L | T | L | - | - | - | - | - | - | - | - | - | N | E | T | I | L | H | R | V | V | - | - | - | - | - | - | - | - | - | - | - | - | P | H | G | Q | S | F | Q | N | - | - | - | - | - | - | - | - | - | - | - | - | - | G | Y | A | G | -63 |
| Q7ZUR1_DANRE | - | A | S | L | T | L | - | - | - | - | - | - | - | - | - | N | D | N | L | L | H | R | V | V | - | - | - | - | - | - | - | - | - | - | - | - | P | H | G | Q | D | F | D | S | - | - | - | - | - | - | - | - | - | - | - | - | - | R | Y | A | G | -63 |
| E9QFN0_DANRE | - | A | C | L | T | L | - | - | - | - | - | - | - | - | - | N | E | P | L | L | R | R | V | V | - | - | - | - | - | - | - | - | - | - | - | - | P | H | G | Q | S | F | H | Q | - | - | - | - | - | - | - | - | - | - | - | - | - | Q | Y | A | G | -63 |
| CAN2_HUMAN | - | A | S | L | T | L | - | - | - | - | - | - | - | - | - | N | E | E | I | L | A | R | V | V | - | - | - | - | - | - | - | - | - | - | - | - | P | L | N | Q | S | F | Q | E | - | - | - | - | - | - | - | - | - | - | - | - | - | N | Y | A | G | -63 |
| CAN2_MOUSE | - | A | S | L | T | L | - | - | - | - | - | - | - | - | - | N | E | E | I | L | A | R | V | V | - | - | - | - | - | - | - | - | - | - | - | - | P | P | D | Q | S | F | Q | E | - | - | - | - | - | - | - | - | - | - | - | - | - | N | Y | A | G | -63 |
| CAN2_CHICK | - | A | S | L | T | L | - | - | - | - | - | - | - | - | - | N | E | E | I | L | A | R | V | V | - | - | - | - | - | - | - | - | - | - | - | - | P | R | D | Q | S | F | Q | D | - | - | - | - | - | - | - | - | - | - | - | - | - | E | Y | A | G | -63 |
| Q6DFZ8_DANRE | - | A | S | L | T | L | - | - | - | - | - | - | - | - | - | N | Q | D | V | F | A | R | V | V | - | - | - | - | - | - | - | - | - | - | - | - | P | A | G | Q | S | F | D | G | - | - | - | - | - | - | - | - | - | - | - | - | - | D | Y | A | G | -63 |
| Q504F5_DANRE | - | A | S | L | T | Q | - | - | - | - | - | - | - | - | - | N | E | D | V | L | A | R | V | V | - | - | - | - | - | - | - | - | - | - | - | - | P | N | G | Q | E | F | D | G | - | - | - | - | - | - | - | - | - | - | - | - | - | T | Y | A | G | -63 |
| Q5BLH5_DANRE | - | A | S | L | T | L | - | - | - | - | - | - | - | - | - | N | E | D | V | L | A | R | V | V | - | - | - | - | - | - | - | - | - | - | - | - | P | S | D | Q | G | F | G | Q | - | - | - | - | - | - | - | - | - | - | - | - | - | D | Y | A | G | -63 |
| Q4VBH3_DANRE | - | A | S | L | T | L | - | - | - | - | - | - | - | - | - | D | K | E | I | L | E | R | V | V | - | - | - | - | - | - | - | - | - | - | - | - | P | S | G | Q | S | F | T | E | - | - | - | - | - | - | - | - | - | - | - | - | - | D | Y | A | G | -63 |
| CAN8_RAT | - | A | S | L | T | L | - | - | - | - | - | - | - | - | - | N | E | K | L | L | Y | R | V | L | - | - | - | - | - | - | - | - | - | - | - | - | P | R | D | Q | S | F | Q | K | - | - | - | - | - | - | - | - | - | - | - | - | - | D | Y | A | G | -63 |
| CAN8_MOUSE | - | A | S | L | T | L | - | - | - | - | - | - | - | - | - | N | E | K | L | L | Y | R | V | V | - | - | - | - | - | - | - | - | - | - | - | - | P | R | D | Q | S | F | Q | K | - | - | - | - | - | - | - | - | - | - | - | - | - | N | Y | A | G | -63 |
| CAN8_HUMAN | - | A | S | L | T | L | - | - | - | - | - | - | - | - | - | N | E | E | L | L | Y | R | V | V | - | - | - | - | - | - | - | - | - | - | - | - | P | R | D | Q | D | F | Q | E | - | - | - | - | - | - | - | - | - | - | - | - | - | N | Y | A | G | -63 |
| XP_426117 | - | A | S | L | T | L | - | - | - | - | - | - | - | - | - | N | P | D | V | L | H | Q | V | V | - | - | - | - | - | - | - | - | - | - | - | - | P | K | A | Q | S | F | Q | E | - | - | - | - | - | - | - | - | - | - | - | - | - | N | Y | A | G | -63 |
| CAN9_MOUSE | - | A | S | L | T | L | - | - | - | - | - | - | - | - | - | N | Q | K | A | L | T | R | V | V | - | - | - | - | - | - | - | - | - | - | - | - | P | Q | D | Q | G | F | G | S | - | - | - | - | - | - | - | - | - | - | - | - | - | G | Y | A | G | -65 |
| CAN9_HUMAN | - | A | S | L | T | L | - | - | - | - | - | - | - | - | - | N | Q | K | A | L | A | R | V | I | - | - | - | - | - | - | - | - | - | - | - | - | P | Q | D | Q | S | F | G | P | - | - | - | - | - | - | - | - | - | - | - | - | - | G | Y | A | G | -63 |
| E1C6M3_CHICK | - | A | S | L | T | L | - | - | - | - | - | - | - | - | - | N | E | K | T | L | A | R | V | V | - | - | - | - | - | - | - | - | - | - | - | - | P | L | D | Q | N | F | G | P | - | - | - | - | - | - | - | - | - | - | - | - | - | G | Y | A | G | -63 |
| Q6DG44_DANRE | - | A | S | L | T | L | - | - | - | - | - | - | - | - | - | K | E | E | T | L | N | R | V | V | - | - | - | - | - | - | - | - | - | - | - | - | P | N | D | Q | T | F | D | R | - | - | - | - | - | - | - | - | - | - | - | - | - | G | Y | A | G | -63 |
| CAN3_HUMAN | - | A | C | L | T | L | - | - | - | - | - | - | - | - | - | N | Q | H | L | L | F | R | V | I | - | - | - | - | - | - | - | - | - | - | - | - | P | H | D | Q | S | F | I | E | - | - | - | - | - | - | - | - | - | - | - | - | - | N | Y | A | G | -63 |
| CAN3_MOUSE | - | A | C | L | T | L | - | - | - | - | - | - | - | - | - | N | E | R | L | L | F | R | V | I | - | - | - | - | - | - | - | - | - | - | - | - | P | H | D | Q | S | F | T | E | - | - | - | - | - | - | - | - | - | - | - | - | - | N | Y | A | G | -63 |
| CAN3_CHICK | - | A | C | L | T | L | - | - | - | - | - | - | - | - | - | N | K | K | L | L | C | R | V | I | - | - | - | - | - | - | - | - | - | - | - | - | P | H | D | Q | S | F | I | Q | - | - | - | - | - | - | - | - | - | - | - | - | - | N | Y | A | G | -63 |
| Q66HU4_DANRE | - | A | C | L | T | L | - | - | - | - | - | - | - | - | - | N | D | K | L | L | Y | R | V | I | - | - | - | - | - | - | - | - | - | - | - | - | P | Q | E | Q | S | F | S | E | - | - | - | - | - | - | - | - | - | - | - | - | - | Q | Y | A | G | -63 |
| F1R632_DANRE | - | A | C | L | T | L | - | - | - | - | - | - | - | - | - | N | D | V | I | L | K | R | V | V | - | - | - | - | - | - | - | - | - | - | - | - | P | H | D | Q | S | F | T | E | - | - | - | - | - | - | - | - | - | - | - | - | - | N | Y | A | G | -63 |
| CAN11_HUMAN | - | G | S | L | T | T | - | - | - | - | - | - | - | - | - | C | P | K | L | L | Y | R | V | V | - | - | - | - | - | - | - | - | - | - | - | - | P | R | G | Q | S | F | K | K | - | - | - | - | - | - | - | - | - | - | - | - | - | N | Y | A | G | -63 |
| CAN11_MOUSE | - | G | S | L | T | T | - | - | - | - | - | - | - | - | - | C | P | K | L | L | Y | R | V | V | - | - | - | - | - | - | - | - | - | - | - | - | P | R | N | Q | S | F | K | K | - | - | - | - | - | - | - | - | - | - | - | - | - | N | Y | A | G | -63 |
| CAN12_MOUSE | - | A | S | L | T | L | - | - | - | - | - | - | - | - | - | Y | P | R | L | L | Y | R | V | V | - | - | - | - | - | - | - | - | - | - | - | - | P | P | G | Q | G | F | Q | D | - | - | - | - | - | - | - | - | - | - | - | - | - | G | Y | A | G | -63 |
| CAN12_HUMAN | - | A | S | L | T | L | - | - | - | - | - | - | - | - | - | Y | P | R | L | L | R | R | V | V | - | - | - | - | - | - | - | - | - | - | - | - | P | P | G | Q | D | F | Q | H | - | - | - | - | - | - | - | - | - | - | - | - | - | G | Y | A | G | -63 |
| ENSSARP00000008135 | - | A | S | L | T | L | - | - | - | - | - | - | - | - | - | Y | P | R | L | L | R | R | V | V | - | - | - | - | - | - | - | - | - | - | - | - | P | L | G | Q | G | F | R | N | - | - | - | - | - | - | - | - | - | - | - | - | - | G | Y | A | G | -63 |
| ENSSARP00000010553 | - | A | I | S | A | A | - | - | - | - | - | - | - | - | - | Y | E | R | V | N | K | K | I | T | - | - | - | - | - | - | - | - | - | - | - | - | X | X | X | X | X | X | X | X | - | - | - | - | - | - | - | - | - | - | - | - | - | X | X | X | X | -62 |
| A7E2G3_DANRE | - | S | C | L | T | M | - | - | - | - | - | - | - | - | - | H | P | T | L | F | V | K | V | V | - | - | - | - | - | - | - | - | - | - | - | - | P | A | G | Q | S | L | S | E | - | - | - | - | - | - | - | - | - | - | - | - | - | S | Y | A | G | -63 |
| Q6NW69_DANRE | - | A | S | L | S | L | - | - | - | - | - | - | - | - | - | Y | P | A | L | L | D | R | V | V | - | - | - | - | - | - | - | - | - | - | - | - | P | A | G | Q | G | F | Q | Q | - | - | - | - | - | - | - | - | - | - | - | - | - | G | Y | N | G | -63 |
| CAN13_HUMAN | - | G | S | L | T | Q | - | - | - | - | - | - | - | - | - | N | P | Q | Y | R | Q | K | I | L | - | - | - | - | - | - | - | - | - | - | - | - | - | M | V | Q | S | F | S | H | - | - | - | - | - | - | - | - | - | - | - | - | - | Q | Y | A | G | -63 |
| CAN13_MOUSE | - | G | S | L | T | Q | - | - | - | - | - | - | - | - | - | N | P | Q | C | L | Q | K | I | L | - | - | - | - | - | - | - | - | - | - | - | - | - | M | D | Q | S | Y | S | H | - | - | - | - | - | - | - | - | - | - | - | - | - | Q | Y | A | G | -65 |
| E1BRR5_CHICK | - | G | S | L | T | M | - | - | - | - | - | - | - | - | - | Q | K | Q | F | L | E | K | V | L | - | - | - | - | - | - | - | - | - | - | - | - | P | K | D | Q | G | F | Q | S | - | - | - | - | - | - | - | - | - | - | - | - | - | D | Y | A | G | -63 |
| E7F0H3_DANRE | - | G | A | L | T | F | - | - | - | - | - | - | - | - | - | E | K | D | I | M | K | Q | V | L | - | - | - | - | - | - | - | - | - | - | - | - | P | A | E | Q | S | F | T | K | - | - | - | - | - | - | - | - | - | - | - | - | - | D | Y | A | G | -64 |
| Q1RLS9_DANRE | - | G | A | L | T | F | - | - | - | - | - | - | - | - | - | Q | K | R | I | M | K | Q | V | I | - | - | - | - | - | - | - | - | - | - | - | - | Q | D | D | Q | T | F | S | V | - | - | - | - | - | - | - | - | - | - | - | - | - | D | Y | A | G | -63 |
| F1Q9Z1_DANRE | - | G | A | L | T | F | - | - | - | - | - | - | - | - | - | Q | S | K | L | L | Q | K | V | V | - | - | - | - | - | - | - | - | - | - | - | - | P | D | G | Q | S | L | R | H | - | - | - | - | - | - | - | - | - | - | - | - | - | N | Y | T | G | -64 |
| A4QNV6_DANRE | - | G | T | L | T | F | - | - | - | - | - | - | - | - | - | Q | K | D | V | L | Y | Q | V | M | - | - | - | - | - | - | - | - | - | - | - | - | P | E | G | Q | S | F | G | K | - | - | - | - | - | - | - | - | - | - | - | - | - | D | Y | A | G | -64 |
| D6W573_HUMAN | - | Q | A | L | A | L | - | - | - | - | - | - | - | - | - | H | Q | D | I | L | S | R | V | V | - | - | - | - | - | - | - | - | - | - | - | - | P | L | N | Q | S | F | T | E | - | - | - | - | - | - | - | - | - | - | - | - | - | K | Y | A | G | -63 |
| R7UBC6_CAPTE | - | A | C | L | F | - | - | E | - | - | - | - | - | - | - | N | I | P | L | L | Q | H | V | V | - | - | - | - | - | - | - | - | - | - | - | - | P | F | T | Q | N | F | R | K | D | - | - | - | - | - | - | - | - | - | - | - | - | S | Y | A | G | -68 |
| K1RCZ2_CRAGI | - | A | C | L | T | S | P | D | - | - | - | - | - | - | - | H | R | E | L | F | R | R | V | V | - | - | - | - | - | - | - | - | - | - | - | - | P | A | D | Q | G | F | Q | D | G | - | - | - | - | - | - | - | - | - | - | - | - | W | Y | A | G | -70 |
| Q5IW13_GECLA | - | S | N | L | T | - | - | L | - | - | - | - | - | - | - | H | P | H | L | F | H | V | V | V | - | - | - | - | - | - | - | - | - | - | - | - | P | R | D | Q | G | F | T | H | - | - | - | - | - | - | - | - | - | - | - | - | - | L | Y | A | G | -63 |
| R7UUX7_CAPTE | - | A | S | L | A | V | - | S | - | - | - | - | - | - | - | N | R | K | L | F | E | R | C | V | - | - | - | - | - | - | - | - | - | - | - | - | P | H | N | Q | T | F | D | D | E | - | - | - | - | - | - | - | - | - | - | - | - | D | Y | A | G | -65 |
| K1Q505_CRAGI | - | A | - | L | V | A | - | T | - | - | - | - | - | - | - | N | K | K | L | L | E | R | V | V | - | - | - | - | - | - | - | - | - | - | - | - | P | Q | D | Q | N | F | D | R | - | - | - | - | - | - | - | - | - | - | - | - | - | N | Y | A | G | -63 |
| R7UD02_CAPTE | - | G | - | T | I | A | - | L | - | - | - | - | - | - | - | N | P | D | L | F | A | R | V | V | - | - | - | - | - | - | - | - | - | - | - | - | P | I | D | Q | T | F | T | E | - | - | - | - | - | - | - | - | - | - | - | - | - | G | Y | A | G | -63 |
| CAN6_HUMAN | - | S | C | L | A | V | - | - | - | - | - | - | - | - | - | Q | E | S | H | W | T | K | T | I | - | - | - | - | - | - | - | - | - | - | - | - | P | N | H | K | E | Q | E | W | D | P | Q | - | K | T | - | - | - | - | - | - | E | K | Y | A | G | -69 |
| CAN6_MOUSE | - | S | C | L | A | V | - | - | - | - | - | - | - | - | - | Q | E | S | H | W | T | K | A | I | - | - | - | - | - | - | - | - | - | - | - | - | P | N | H | K | D | Q | E | W | D | P | R | - | K | P | - | - | - | - | - | - | E | K | Y | A | G | -69 |
| E1C312_CHICK | - | S | C | L | A | L | - | - | - | - | - | - | - | - | - | R | K | K | L | W | Q | Q | V | I | - | - | - | - | - | - | - | - | - | - | - | - | P | D | F | Q | E | Q | E | W | D | P | K | - | T | P | - | - | - | - | - | - | E | K | Y | A | G | -69 |
| E7FDN3_DANRE | - | S | C | L | A | A | - | - | - | - | - | - | - | - | - | E | S | S | L | W | K | K | V | I | - | - | - | - | - | - | - | - | - | - | - | - | P | D | H | T | E | Q | E | W | H | P | K | - | R | P | - | - | - | - | - | - | D | L | Y | A | G | -69 |
| CAN5_HUMAN | - | S | S | L | A | S | - | - | - | - | - | - | - | - | - | R | E | S | L | W | Q | K | V | I | - | - | - | - | - | - | - | - | - | - | - | - | P | D | W | K | E | Q | E | W | D | P | E | - | K | P | - | - | - | - | - | - | N | A | Y | A | G | -65 |
| CAN5_MOUSE | - | S | S | L | A | S | - | - | - | - | - | - | - | - | - | R | E | S | L | W | Q | K | V | I | - | - | - | - | - | - | - | - | - | - | - | - | P | D | W | K | E | Q | E | W | N | P | E | - | K | P | - | - | - | - | - | - | D | S | Y | A | G | -69 |
| E1C292_CHICK | - | S | S | L | A | S | - | - | - | - | - | - | - | - | - | R | E | A | L | W | Q | K | V | I | - | - | - | - | - | - | - | - | - | - | - | - | P | D | W | K | E | Q | E | W | N | A | E | - | K | P | - | - | - | - | - | - | E | S | Y | A | G | -69 |
| A0JMG3_DANRE | - | S | S | L | A | S | - | - | - | - | - | - | - | - | - | R | E | A | L | W | Q | K | V | I | - | - | - | - | - | - | - | - | - | - | - | - | P | D | W | K | D | Q | E | W | D | K | N | - | K | P | - | - | - | - | - | - | E | S | Y | A | G | -69 |
| E7F7F1_DANRE | - | S | S | L | A | S | - | - | - | - | - | - | - | - | - | R | E | S | L | W | Q | K | V | I | - | - | - | - | - | - | - | - | - | - | - | - | P | D | W | K | E | Q | E | W | D | P | E | - | K | A | - | - | - | - | - | - | D | S | Y | A | G | -69 |
| XP_006824412 | - | S | S | L | A | Q | - | - | - | - | - | - | - | - | - | Q | Q | E | L | W | T | K | V | V | - | - | - | - | - | - | - | - | - | - | - | - | P | T | H | E | E | Q | E | W | D | S | E | - | N | P | - | - | - | - | - | - | Q | K | Y | A | G | -63 |
| W4YJN2_STRPU | - | A | N | L | A | Q | - | - | - | - | - | - | - | - | - | E | K | E | L | W | E | T | V | V | - | - | - | - | - | - | - | - | - | - | - | - | P | N | A | K | D | Q | D | W | D | A | D | - | N | K | - | - | - | - | - | - | E | K | Y | A | G | -69 |
| C3YJZ3_BRAFL | - | A | S | L | T | L | - | - | - | - | - | - | - | - | - | E | K | D | L | W | K | E | V | I | - | - | - | - | - | - | - | - | - | - | - | - | P | D | Y | K | E | Q | E | W | D | T | E | - | H | P | - | - | - | - | - | - | E | N | Y | Q | G | -69 |
| T2MIT4_HYDVU | - | A | S | L | A | L | - | - | - | - | - | - | - | - | - | E | K | D | L | Y | T | K | V | I | - | - | - | - | - | - | - | - | - | - | - | - | P | N | H | K | T | L | T | F | K | S | V | - | K | P | - | - | - | - | - | - | E | D | Y | L | G | -69 |
| XP_002154722 | - | A | S | L | A | L | - | - | - | - | - | - | - | - | - | E | K | D | L | Y | T | K | N | F | - | - | - | - | - | - | - | - | - | - | - | - | I | L | S | K | - | - | - | - | - | - | - | - | - | - | - | - | - | - | - | - | E | Y | Y | H | G | -55 |
| R7VLA7_CAPTE | - | S | C | L | A | T | - | - | - | - | - | - | - | - | - | N | K | E | I | W | R | K | V | I | - | - | - | - | - | - | - | - | - | - | - | - | P | N | H | K | D | Q | E | W | D | P | E | - | K | P | - | - | - | - | - | - | E | K | Y | A | G | -69 |
| K1R1C6_CRAGI | - | S | C | L | A | I | - | - | - | - | - | - | - | - | - | H | K | D | I | W | H | K | V | I | - | - | - | - | - | - | - | - | - | - | - | - | P | D | A | K | D | Q | E | W | D | E | N | - | N | P | - | - | - | - | - | - | N | N | Y | Q | G | -69 |
| B3RIE5_TRIAD | - | A | S | L | S | Q | - | - | - | - | - | - | - | - | - | E | K | S | L | V | N | K | V | I | - | - | - | - | - | - | - | - | - | - | - | - | P | N | V | G | E | Q | D | Y | D | T | S | - | D | G | - | - | - | - | - | - | E | - | H | S | G | -68 |
| K1PW75_CRAGI | - | A | C | I | A | E | - | - | - | - | - | - | - | - | - | D | K | S | L | L | K | K | I | V | - | - | - | - | - | - | - | - | - | - | - | - | P | D | Y | E | E | Q | E | W | S | - | - | - | K | D | - | - | - | - | - | - | N | K | Y | A | G | -67 |
| K1RSB8_CRAGI | - | A | C | I | A | E | - | - | - | - | - | - | - | - | - | R | K | N | L | L | R | K | I | V | - | - | - | - | - | - | - | - | - | - | - | - | P | D | I | E | K | Q | D | W | A | - | - | - | K | G | - | - | - | - | - | - | R | K | Y | A | G | -67 |
| K1RWI3_CRAGI | - | A | C | I | A | E | - | - | - | - | - | - | - | - | - | K | P | K | L | L | K | K | I | I | - | - | - | - | - | - | - | - | - | - | - | - | P | D | I | E | K | Q | E | W | L | P | E | - | N | K | - | - | - | - | - | - | N | K | Y | A | G | -69 |
| R7VGC2_CAPTE | - | S | S | L | V | L | - | - | - | - | - | - | - | - | - | E | P | K | M | W | Q | K | V | I | - | - | - | - | - | - | - | - | - | - | - | - | P | N | Y | K | A | Q | D | F | P | T | - | - | D | E | - | - | - | - | - | - | T | Q | Y | A | G | -68 |
| K1QU26_CRAGI | - | A | S | L | A | L | - | - | - | - | - | - | - | - | - | E | K | K | L | F | E | K | V | L | - | - | - | - | - | - | - | - | - | - | - | - | P | N | L | K | E | Q | E | W | D | E | R | A | E | K | - | - | - | - | - | - | N | K | Y | A | G | -70 |
| CAN5_CAEEL | - | S | A | L | T | H | - | - | - | - | - | - | - | - | - | N | F | K | L | L | A | Q | V | I | - | - | - | - | - | - | - | - | - | - | - | - | P | D | A | D | D | Q | E | W | S | T | - | - | - | K | - | - | - | - | - | - | H | A | Y | A | G | -67 |
| I1FGV4_AMPQE | - | A | S | L | T | Q | - | - | - | - | - | - | - | - | - | D | K | K | L | I | N | K | V | I | - | - | - | - | - | - | - | - | - | - | - | - | P | Q | N | Q | G | W | G | K | G | - | - | - | - | - | - | - | - | - | - | - | - | - | Y | A | G | -63 |
| K1PHX3_CRAGI | - | S | C | L | T | L | - | - | - | - | - | - | - | - | - | T | P | K | L | L | G | R | C | I | - | - | - | - | - | - | - | - | - | - | - | - | P | E | D | Q | G | F | T | - | - | - | - | - | - | - | - | - | - | - | - | - | H | K | Y | A | G | -63 |
| B3RNL8_TRIAD | - | S | S | L | A | T | - | - | - | - | - | - | - | - | - | E | K | N | L | L | Y | R | V | I | - | - | - | - | - | - | - | - | - | - | - | - | P | N | D | Q | T | F | D | - | - | - | - | - | - | - | - | - | - | - | - | - | K | D | Y | A | G | -63 |
| X1Y3H7_ANODA | - | A | S | L | A | S | - | - | - | - | - | - | - | - | - | K | P | H | L | F | E | R | V | V | - | - | - | - | - | - | - | - | - | - | - | - | P | M | D | N | G | N | F | S | A | K | G | - | - | - | - | - | - | - | - | - | - | Q | Y | A | G | -66 |
| R7TWH9_CAPTE | - | A | A | V | S | Q | - | - | - | - | - | - | - | - | - | Q | E | K | F | M | R | K | I | I | - | - | - | - | - | - | - | - | - | - | - | - | P | D | D | Q | P | L | C | G | S | A | - | - | - | - | - | - | - | - | - | - | - | - | Y | K | G | -67 |
| K1R5E8_CRAGI | - | A | A | V | S | Q | - | - | - | - | - | - | - | - | - | K | K | E | F | M | K | K | I | V | - | - | - | - | - | - | - | - | - | - | - | - | P | S | D | Q | P | L | C | G | P | G | - | - | - | - | - | - | - | - | - | - | - | - | Y | K | G | -67 |
| XP_006811804 | - | A | S | I | A | Q | - | - | - | - | - | - | - | - | - | H | K | R | F | M | E | K | V | V | - | - | - | - | - | - | - | - | - | - | - | - | P | T | D | Q | Y | L | W | S | S | G | K | - | - | - | - | - | - | - | - | - | - | - | Y | C | G | -65 |
| C3YB23_BRAFL | - | A | A | I | C | R | - | - | - | - | - | - | - | - | - | D | S | A | L | M | E | K | V | V | - | - | - | - | - | - | - | - | - | - | - | - | P | R | D | Q | Y | M | W | Q | E | G | E | - | G | E | - | - | - | - | - | - | G | S | Y | V | G | -71 |
| H2ZIF1_CIOSA | - | A | A | I | C | R | - | - | - | - | - | - | - | - | - | S | S | K | H | I | Q | K | V | V | - | - | - | - | - | - | - | - | - | - | - | - | P | G | D | Q | V | M | T | G | P | D | - | - | - | - | - | - | - | - | - | - | - | - | Y | V | G | -64 |
| W4YV93_STRPU | - | S | A | I | A | K | - | - | - | - | - | - | - | - | - | M | P | A | L | I | Q | K | I | I | - | - | - | - | - | - | - | - | - | - | - | - | P | S | D | Q | - | S | F | T | D | G | - | - | - | - | - | - | - | - | - | - | - | - | Y | S | G | -63 |
| CAN10_HUMAN | - | A | A | L | Q | K | - | - | - | - | - | - | - | - | - | S | R | H | L | L | D | Q | V | I | - | - | - | - | - | - | - | - | - | - | - | - | P | P | G | Q | P | S | W | A | D | Q | E | - | - | - | - | - | - | - | - | - | - | - | Y | R | G | -65 |
| CAN10_MOUSE | - | A | A | L | Q | K | - | - | - | - | - | - | - | - | - | S | Q | H | L | L | D | Q | V | F | - | - | - | - | - | - | - | - | - | - | - | - | P | P | G | Q | P | G | W | S | D | Q | K | - | - | - | - | - | - | - | - | - | - | - | Y | Q | G | -65 |
| XP_004943443 | - | V | A | L | Q | K | - | - | - | - | - | - | - | - | - | S | K | Y | L | L | N | K | V | F | - | - | - | - | - | - | - | - | - | - | - | - | P | P | G | Q | P | S | W | A | D | E | L | - | - | - | - | - | - | - | - | - | - | - | Y | Q | G | -65 |
| F8W4K1_DANRE | - | S | M | L | L | K | - | - | - | - | - | - | - | - | - | N | Q | H | L | L | D | K | V | F | - | - | - | - | - | - | - | - | - | - | - | - | P | P | E | Q | P | L | W | G | D | S | A | - | - | - | - | - | - | - | - | - | - | - | Y | R | G | -65 |
| CANC_DROME | - | G | L | L | S | S | - | - | - | - | - | - | - | - | - | L | R | N | L | F | Y | R | V | V | - | - | - | - | - | - | - | - | - | - | - | - | P | A | D | Q | T | L | A | S | - | - | - | - | - | - | - | - | - | - | - | - | - | - | A | H | G | -51 |
| C3ZKN9_BRAFL | - | S | S | V | C | Q | - | - | - | - | - | - | - | - | - | N | S | K | L | F | R | H | A | V | - | - | - | - | - | - | - | - | - | - | - | - | Q | P | E | L | Q | G | F | S | E | G | - | - | - | - | - | - | - | - | - | - | - | - | Y | C | G | -63 |
| G4V623_SCHMA | - | A | A | I | A | S | - | - | - | - | - | - | - | - | - | Q | P | S | L | L | S | R | T | I | - | - | - | - | - | - | - | - | - | - | - | - | P | T | G | - | Q | S | F | R | V | E | W | - | - | - | - | - | - | - | - | - | - | - | Y | A | G | -81 |
| R7VK39_CAPTE | - | F | I | L | A | S | - | - | - | - | - | - | - | - | - | S | K | N | S | F | D | Q | V | V | - | - | - | - | - | - | - | - | - | - | - | - | H | D | S | - | E | T | F | E | H | N | - | - | - | - | - | - | - | - | - | - | - | - | Y | T | G | -64 |
| G4V693_SCHMA | - | T | G | L | K | Y | - | - | - | - | - | - | - | - | - | K | A | K | Y | W | A | K | V | L | - | - | - | - | - | - | - | - | - | - | - | - | V | D | I | K | S | Q | E | W | D | V | K | H | V | N | - | - | - | - | - | - | - | E | H | P | G | -68 |
| R7UPU6_CAPTE | - | S | S | L | A | D | - | - | - | - | - | - | - | - | - | K | P | E | V | L | K | Q | I | I | - | - | - | - | - | - | - | - | - | - | - | - | P | E | D | S | Y | N | I | G | R | E | - | - | - | - | - | - | - | - | - | - | - | D | Y | D | G | -65 |
| R7V817_CAPTE | - | S | S | L | A | D | - | - | - | - | - | - | - | - | - | K | P | E | V | L | K | Q | V | C | - | - | - | - | - | - | - | - | - | - | - | - | N | - | H | S | Y | N | I | G | R | E | - | - | - | - | - | - | - | - | - | - | - | D | Y | D | G | -62 |
| R7UNL8_CAPTE | - | S | S | L | A | D | - | - | - | - | - | - | - | - | - | N | G | E | A | I | K | Q | I | I | - | - | - | - | - | - | - | - | - | - | - | - | P | D | D | A | Y | P | I | G | S | D | - | - | - | - | - | - | - | - | - | - | - | K | Y | S | G | -60 |
| K1PJ80_CRAGI | - | A | N | V | A | D | - | - | - | - | - | - | - | - | - | K | P | K | L | L | R | R | I | I | - | - | - | - | - | - | - | - | - | - | - | - | P | S | D | S | Y | P | V | G | S | P | - | - | - | - | - | - | - | - | - | - | - | K | Y | D | G | -65 |
| K1PCX8_CRAGI | - | A | N | I | A | D | - | - | - | - | - | - | - | - | - | K | P | K | L | L | R | R | I | I | - | - | - | - | - | - | - | - | - | - | - | - | P | R | D | S | Y | P | V | E | P | Q | - | - | - | - | - | - | - | - | - | - | - | N | Y | D | G | -65 |
| CAND_DROME | - | A | V | L | A | - | E | R | - | - | - | - | - | - | - | E | D | L | - | - | - | - | - | - | - | - | - | - | - | - | - | - | - | - | - | - | - | V | K | E | V | L | V | T | K | E | I | C | G | - | - | - | - | - | - | - | - | - | - | Q | G | -65 |
| XP_002735765 | - | S | V | L | A | - | E | R | - | - | - | - | - | - | - | P | D | L | - | - | - | - | - | - | - | - | - | - | - | - | - | - | - | - | - | - | - | I | E | K | V | L | I | T | K | E | Y | T | P | - | - | - | - | - | - | - | - | - | - | E | G | -67 |
| CAN15_HUMAN | - | A | V | L | A | - | E | R | - | - | - | - | - | - | - | P | D | L | - | - | - | - | - | - | - | - | - | - | - | - | - | - | - | - | - | - | - | V | E | R | V | M | V | T | R | S | L | C | A | - | - | - | - | - | - | - | - | - | - | E | G | -67 |
| CAN15_MOUSE | - | A | V | L | A | - | E | R | - | - | - | - | - | - | - | P | D | L | - | - | - | - | - | - | - | - | - | - | - | - | - | - | - | - | - | - | - | V | E | R | V | M | V | T | R | S | L | C | A | - | - | - | - | - | - | - | - | - | - | E | G | -67 |
| E1BTA8_CHICK | - | A | V | L | A | - | E | R | - | - | - | - | - | - | - | P | E | L | - | - | - | - | - | - | - | - | - | - | - | - | - | - | - | - | - | - | - | V | E | R | V | M | I | T | R | T | L | C | P | - | - | - | - | - | - | - | - | - | - | E | G | -67 |
| E7FAX5_DANRE | - | A | V | L | A | - | E | R | - | - | - | - | - | - | - | P | E | L | - | - | - | - | - | - | - | - | - | - | - | - | - | - | - | - | - | - | - | V | E | R | V | M | I | T | R | S | I | C | Q | - | - | - | - | - | - | - | - | - | - | E | G | -67 |
| C3XWK8_BRAFL | - | A | V | L | A | - | E | R | - | - | - | - | - | - | - | P | E | L | - | - | - | - | - | - | - | - | - | - | - | - | - | - | - | - | - | - | - | V | E | R | V | M | I | T | R | E | I | C | P | - | - | - | - | - | - | - | - | - | - | E | G | -66 |
| W4YMU4_STRPU | - | S | V | L | A | - | G | R | - | - | - | - | - | - | - | A | D | L | - | - | - | - | - | - | - | - | - | - | - | - | - | - | - | - | - | - | - | L | K | R | I | M | I | T | H | E | V | N | P | - | - | - | - | - | - | - | - | - | - | E | G | -67 |
| K1PV74_CRAGI | - | A | V | L | A | - | E | R | - | - | - | - | - | - | - | P | E | L | - | - | - | - | - | - | - | - | - | - | - | - | - | - | - | - | - | - | - | V | E | S | I | I | L | T | K | D | Y | C | P | - | - | - | - | - | - | - | - | - | - | Q | G | -67 |
| R7TJF2_CAPTE | - | A | V | L | A | - | E | R | - | - | - | - | - | - | - | P | E | L | - | - | - | - | - | - | - | - | - | - | - | - | - | - | - | - | - | - | - | V | E | K | V | L | L | T | K | K | V | N | R | - | - | - | - | - | - | - | - | - | - | E | G | -67 |
| T2MI17_HYDVU | - | A | V | V | C | - | E | R | - | - | - | - | - | - | - | P | E | L | - | - | - | - | - | - | - | - | - | - | - | - | - | - | - | - | - | - | - | L | E | K | I | M | V | T | R | E | I | C | D | - | - | - | - | - | - | - | - | - | - | E | G | -64 |
| Q1RL55_CIOIN | - | A | V | L | A | - | E | R | - | - | - | - | - | - | - | P | D | L | - | - | - | - | - | - | - | - | - | - | - | - | - | - | - | - | - | - | - | L | K | R | V | M | V | T | K | N | Y | N | K | - | - | - | - | - | - | - | - | - | - | E | G | -54 |
| I1EA30_AMPQE | - | A | V | I | A | - | E | V | - | - | - | - | - | - | - | P | E | L | - | - | - | - | - | - | - | - | - | - | - | - | - | - | - | - | - | - | - | L | Q | K | I | L | I | T | K | E | F | C | P | - | - | - | - | - | - | - | - | - | - | Q | G | -64 |
| G5EEZ6_CAEEL | - | A | L | I | A | - | E | R | - | - | - | - | - | - | - | P | D | V | - | - | - | - | - | - | - | - | - | - | - | - | - | - | - | - | - | - | - | L | D | D | I | I | P | K | K | Q | Y | S | H | - | - | - | - | - | - | - | - | - | - | Y | G | -66 |
| O44903_CAEEL | - | A | L | I | A | - | E | K | - | - | - | - | - | - | - | P | E | V | - | - | - | - | - | - | - | - | - | - | - | - | - | - | - | - | - | - | - | L | D | A | I | I | P | K | K | Q | Y | S | K | - | - | - | - | - | - | - | - | - | - | H | G | -66 |
| O02260_CAEEL | - | S | A | I | S | - | T | K | - | - | - | - | - | - | - | P | E | V | - | - | - | - | - | - | - | - | - | - | - | - | - | - | - | - | - | - | - | I | M | N | I | F | D | S | L | Q | L | S | K | - | - | - | - | - | - | - | - | - | - | Y | G | -65 |
| O02259_CAEEL | - | S | A | I | S | - | T | K | - | - | - | - | - | - | - | P | E | V | - | - | - | - | - | - | - | - | - | - | - | - | - | - | - | - | - | - | - | I | M | N | I | F | D | S | L | Q | L | S | K | - | - | - | - | - | - | - | - | - | - | Y | G | -42 |
| C1MTG3_MICPC | - | A | V | I | A | - | E | R | - | - | - | - | - | - | - | Q | D | L | - | - | - | - | - | - | - | - | - | - | - | - | - | - | - | - | - | - | - | A | R | R | V | V | G | P | S | A | A | S | P | A | - | - | A | A | A | A | A | A | A | S | G | -48 |
| C1E493_MICSR | - | A | V | V | A | - | E | R | - | - | - | - | - | - | - | Q | D | L | - | - | - | - | - | - | - | - | - | - | - | - | - | - | - | - | - | - | - | V | A | R | V | V | G | P | A | A | K | C | P | A | - | - | A | T | R | - | - | - | - | R | G | -44 |
| Q012Z6_OSTTA | - | A | V | I | A | - | E | R | - | - | - | - | - | - | - | E | D | L | - | - | - | - | - | - | - | - | - | - | - | - | - | - | - | - | - | - | - | L | E | Q | V | V | G | V | S | R | R | H | A | G | - | - | V | F | E | K | - | - | - | H | G | -52 |
| L1IGB6_GUITH | - | A | V | V | A | - | E | R | - | - | - | - | - | - | - | H | D | L | - | - | - | - | - | - | - | - | - | - | - | - | - | - | - | - | - | - | - | I | A | K | L | F | Y | D | T | A | P | N | A | - | - | - | - | - | - | - | - | - | - | N | G | -53 |
| B7FWX6_PHATC | - | A | V | V | A | - | E | K | - | - | - | - | - | - | - | S | Y | L | - | - | - | - | - | - | - | - | - | - | - | - | - | - | - | - | - | - | - | V | R | Q | L | L | P | H | D | K | L | N | P | - | - | - | - | - | - | - | - | - | - | Q | G | -40 |
| N9TPR9_ENTHI | - | A | S | L | S | - | E | F | - | - | - | - | - | - | - | P | E | R | - | - | - | - | - | - | - | - | - | - | - | - | - | - | - | - | - | - | - | I | K | T | M | I | K | S | - | - | - | C | G | - | - | - | - | - | - | - | - | - | - | N | G | -57 |
| G0QS43_ICHMG | - | S | S | L | A | - | E | T | - | - | - | - | - | - | - | P | S | R | - | - | - | - | - | - | - | - | - | - | - | - | - | - | - | - | - | - | - | I | E | R | L | F | S | H | H | I | E | N | S | - | - | - | - | - | - | - | - | - | - | H | G | -62 |
| D8LB20_ECTSI | - | A | T | L | A | G | T | M | - | - | - | - | - | - | - | P | G | A | - | - | - | - | - | - | - | - | - | - | - | - | - | - | - | - | - | - | - | I | K | K | L | F | G | N | S | E | R | S | Y | - | - | - | - | - | - | - | - | - | - | R | G | -66 |
| Q9U0T9_LEIMA | - | A | T | L | A | - | E | D | - | - | - | - | - | - | - | P | T | V | - | - | - | - | - | - | - | - | - | - | - | - | - | - | - | - | - | - | - | V | M | Q | V | F | R | H | P | K | G | A | D | S | - | - | A | R | C | E | R | - | A | I | G | -69 |
| Q4CXJ8_TRYCC | - | A | T | L | E | - | E | D | - | - | - | - | - | - | - | P | G | D | - | - | - | - | - | - | - | - | - | - | - | - | - | - | - | - | - | - | - | V | M | R | M | F | R | N | P | F | G | A | E | T | - | - | A | R | R | E | R | - | A | V | G | -69 |
| F4I0A4_ARATH | - | A | V | L | T | E | V | S | - | - | - | - | - | - | - | - | - | - | - | - | - | - | - | - | - | - | - | - | - | - | - | - | - | - | - | - | R | I | S | E | V | I | I | T | P | - | - | - | - | - | - | - | - | - | - | E | Y | N | E | E | G | -65 |
| Q8RUQ1_MAIZE | - | A | V | L | T | E | M | S | - | - | - | - | - | - | - | - | - | - | - | - | - | - | - | - | - | - | - | - | - | - | - | - | - | - | - | - | R | I | S | E | V | I | I | T | P | - | - | - | - | - | - | - | - | - | - | E | Y | N | D | E | G | -65 |
| D7FQ17_ECTSI | - | S | I | I | S | A | A | S | - | - | - | - | - | - | - | - | V | G | D | G | G | V | D | E | - | - | - | - | - | - | - | - | - | - | - | - | Q | I | A | Q | L | F | V | S | P | I | G | G | D | G | - | - | R | P | V | L | E | S | Y | T | G | -75 |
| D0MZ44_PHYIT | - | S | I | M | A | A | S | G | - | - | - | - | - | - | - | G | V | D | D | G | K | V | D | E | - | - | - | - | - | - | - | - | - | - | - | - | L | I | D | R | L | F | V | T | - | - | - | - | - | - | - | - | - | - | K | Q | T | S | L | T | G | -68 |
| L1JBM0_GUITH | - | S | I | M | A | G | R | R | - | - | - | - | - | - | - | R | E | R | - | - | - | - | - | - | - | - | - | - | - | - | - | - | - | - | - | - | - | L | E | A | L | V | C | F | G | - | - | - | - | - | - | - | - | - | - | - | W | V | A | R | G | -36 |
| L1J739_GUITH | - | S | C | V | A | S | R | R | - | - | - | - | - | - | - | - | D | L | - | - | - | - | - | - | - | - | - | - | - | - | - | - | - | - | - | - | - | L | R | N | L | F | A | - | - | - | - | - | - | - | - | - | S | T | E | Y | G | - | S | Q | G | -66 |
| L1J0U9_GUITH | - | G | C | L | V | G | R | R | - | - | - | - | - | - | - | - | D | L | - | - | - | - | - | - | - | - | - | - | - | - | - | - | - | - | - | - | - | L | E | V | L | I | P | - | - | - | - | - | - | - | - | - | S | T | E | N | G | R | A | Y | G | -60 |
| L1J1Q4_GUITH | - | S | V | I | A | C | R | P | - | - | - | - | - | - | - | - | D | M | - | - | - | - | - | - | - | - | - | - | - | - | - | - | - | - | - | - | - | V | E | Q | I | F | G | P | L | K | Q | V | D | P | N | V | S | H | S | Y | L | L | D | R | G | -73 |
| D0ND88_PHYIT | - | A | A | V | A | L | H | P | - | - | - | - | - | - | - | D | N | L | - | - | - | - | - | - | - | - | - | - | - | - | - | - | - | - | - | - | - | I | E | N | L | F | V | S | - | - | - | - | - | - | - | E | S | L | Q | N | F | K | Q | F | G | -64 |
| D2VVS6_NAEGR | - | A | I | V | A | S | H | D | - | - | - | - | - | - | - | F | V | M | - | - | - | - | - | - | - | - | - | - | - | - | - | - | - | - | - | - | - | - | K | K | V | V | V | E | S | K | T | N | S | A | - | - | - | - | - | - | - | - | - | I | G | -63 |
| D2V3V2_NAEGR | - | S | I | V | A | D | Y | P | - | - | - | - | - | - | - | D | I | M | - | - | - | - | - | - | - | - | - | - | - | - | - | - | - | - | - | - | - | - | S | K | I | I | L | E | S | K | S | D | P | D | - | - | - | - | - | - | - | - | - | N | G | -64 |
| Q5B5J7_EMENI | - | C | T | M | G | - | N | K | - | - | - | - | - | - | - | A | G | L | - | - | - | - | - | - | - | - | - | - | - | - | - | - | - | - | - | - | - | I | E | K | I | C | V | H | R | D | E | E | - | - | - | - | - | - | - | - | - | - | - | V | G | -53 |
| E4ZY10_LEPMJ | - | A | T | I | S | - | N | K | - | - | - | - | - | - | - | E | D | L | - | - | - | - | - | - | - | - | - | - | - | - | - | - | - | - | - | - | - | I | Q | R | V | C | V | A | R | D | E | R | - | - | - | - | - | - | - | - | - | - | - | V | G | -58 |
| A8PIE5_COPC7 | - | A | T | V | S | - | T | M | - | - | - | - | - | - | - | P | G | L | - | - | - | - | - | - | - | - | - | - | - | - | - | - | - | - | - | - | - | I | E | K | V | C | V | A | R | D | E | E | - | - | - | - | - | - | - | - | - | - | - | V | G | -55 |
| A8NBA6_COPC7 | - | A | T | I | A | - | S | V | - | - | - | - | - | - | - | P | G | L | - | - | - | - | - | - | - | - | - | - | - | - | - | - | - | - | - | - | - | I | E | E | I | C | V | A | R | D | E | E | - | - | - | - | - | - | - | - | - | - | - | V | G | -57 |
| A8NBA1_COPC7 | - | A | T | V | C | - | C | L | - | - | - | - | - | - | - | P | G | L | - | - | - | - | - | - | - | - | - | - | - | - | - | - | - | - | - | - | - | I | E | K | I | C | V | A | R | D | E | E | - | - | - | - | - | - | - | - | - | - | - | V | G | -60 |
| A8NBA2_COPC7 | - | G | T | V | C | - | C | I | - | - | - | - | - | - | - | P | G | L | - | - | - | - | - | - | - | - | - | - | - | - | - | - | - | - | - | - | - | I | E | K | I | C | V | A | R | D | E | E | - | - | - | - | - | - | - | - | - | - | - | V | G | -60 |
| A8NAE6_COPC7 | - | S | A | M | T | - | C | M | - | - | - | - | - | - | - | P | E | L | - | - | - | - | - | - | - | - | - | - | - | - | - | - | - | - | - | - | - | V | E | N | V | C | V | A | R | D | E | E | - | - | - | - | - | - | - | - | - | - | - | V | G | -61 |
| A8NAI9_COPC7 | - | S | T | I | S | - | G | L | - | - | - | - | - | - | - | K | G | L | - | - | - | - | - | - | - | - | - | - | - | - | - | - | - | - | - | - | - | V | S | K | L | C | V | A | R | D | E | E | - | - | - | - | - | - | - | - | - | - | - | V | G | -57 |
| G4TER7_PIRID | - | A | N | V | A | - | T | I | - | - | - | - | - | - | - | P | G | L | - | - | - | - | - | - | - | - | - | - | - | - | - | - | - | - | - | - | - | L | E | K | I | C | V | E | R | D | E | Q | - | - | - | - | - | - | - | - | - | - | - | V | G | -57 |
| G4TER6_PIRID | - | A | N | V | V | - | T | I | - | - | - | - | - | - | - | T | G | L | - | - | - | - | - | - | - | - | - | - | - | - | - | - | - | - | - | - | - | L | E | Q | I | C | V | A | Q | E | P | V | - | - | - | - | - | - | - | - | - | - | - | V | G | -49 |
| G4TNA1_PIRID | - | G | A | I | A | - | A | I | - | - | - | - | - | - | - | P | G | L | - | - | - | - | - | - | - | - | - | - | - | - | - | - | - | - | - | - | - | I | E | K | I | C | V | A | H | D | V | Q | - | - | - | - | - | - | - | - | - | - | - | V | G | -57 |
| D0MWB9_PHYIT | - | S | V | V | A | - | T | R | - | - | - | - | - | - | - | S | D | L | - | - | - | - | - | - | - | - | - | - | - | - | - | - | - | - | - | - | - | L | E | Q | T | F | W | R | R | D | E | H | K | S | - | - | - | - | - | - | - | - | - | K | G | -54 |
| L1I9H5_GUITH | - | S | A | C | S | I | G | S | - | - | - | - | - | - | - | S | D | I | L | L | H | D | L | C | - | - | - | - | - | - | - | - | - | - | - | - | I | E | D | G | M | D | Q | G | - | - | - | - | - | - | - | - | - | - | - | - | - | - | - | - | - | -64 |
| Q7N497_PHOLL | - | G | A | I | A | S | H | P | - | - | - | - | - | - | - | E | G | H | N | L | L | F | S | A | - | - | - | - | - | - | - | - | - | - | - | - | I | Y | P | P | V | Y | N | P | - | - | - | - | - | - | - | - | - | - | - | - | - | - | - | T | G | -53 |
| ZP_11152369 | - | G | S | L | T | L | H | P | - | - | - | - | - | - | - | Q | A | H | N | L | L | F | S | M | - | - | - | - | - | - | - | - | - | - | - | - | I | Y | P | A | V | Y | N | P | - | - | - | - | - | - | - | - | - | - | - | - | - | - | - | L | G | -49 |
| A2FEL7_TRIVA | - | A | S | I | C | R | - | - | - | - | - | - | - | - | - | - | - | - | - | - | - | S | I | S | - | - | - | - | - | - | - | - | - | - | - | - | N | I | K | N | L | F | Y | I | P | D | I | - | - | - | - | - | - | - | - | - | A | V | K | N | G | -60 |
| A2D9A8_TRIVA | - | S | S | I | C | Q | - | - | - | - | - | - | - | - | - | - | - | - | - | - | - | N | S | R | - | - | - | - | - | - | - | - | - | - | - | - | I | I | K | N | L | F | Y | K | S | E | L | - | - | - | - | - | - | - | - | - | A | V | S | N | G | -60 |
| A2FHB3_TRIVA | - | A | A | I | C | A | - | - | - | - | - | - | - | - | - | - | - | - | - | - | - | A | P | H | - | - | - | - | - | - | - | - | - | - | - | - | D | V | V | D | I | F | E | D | H | T | K | - | - | - | - | - | - | - | - | - | I | Q | K | T | G | -59 |
| A2DTE8_TRIVA | - | S | C | L | S | R | - | - | - | - | - | - | - | - | - | - | - | - | - | - | - | D | K | N | - | - | - | - | - | - | - | - | - | - | - | - | V | I | N | D | L | F | K | Q | P | V | K | - | - | - | - | - | - | - | - | - | L | E | R | - | G | -58 |
| A2E4X2_TRIVA | - | I | Q | M | T | N | - | - | - | - | - | - | - | - | - | - | - | - | - | - | - | K | P | A | - | - | - | - | - | - | - | - | - | - | - | - | N | V | R | A | L | F | Q | D | P | I | - | - | - | - | - | - | - | - | - | - | D | I | N | T | G | -58 |
| A2E1X1_TRIVA | - | L | H | L | S | K | - | - | - | - | - | - | - | - | - | - | - | - | - | - | - | H | P | D | - | - | - | - | - | - | - | - | - | - | - | - | M | I | K | S | I | F | V | D | P | - | - | - | - | - | - | - | - | - | - | - | D | I | S | T | G | -49 |
| TPR_PORGI | - | S | A | I | A | W | V | W | - | - | - | - | - | - | - | - | P | A | L | L | N | M | D | V | - | - | - | - | - | - | - | - | - | - | - | - | D | I | M | S | N | Q | D | E | W | R | L | Y | R | Y | - | - | - | - | - | - | - | - | - | F | I | -77 |
| D5MF42_9BACT | - | S | A | V | A | W | S | R | - | - | - | - | - | - | - | - | P | Y | V | I | A | L | R | Q | - | - | - | - | - | - | - | - | - | - | - | - | R | A | T | G | T | N | Q | - | - | - | - | - | Q | D | - | - | - | - | - | - | - | - | - | F | L | -40 |
| R9H254_BACT4 | - | A | S | M | A | Y | L | Y | - | - | - | - | - | - | - | - | P | R | F | V | K | H | I | I | - | - | - | - | - | - | - | - | - | - | - | - | K | - | D | N | M | D | K | - | - | - | - | - | - | - | - | - | - | - | - | - | - | - | - | - | - | -39 |
| ZP_04847694 | - | A | S | M | A | Y | L | Y | - | - | - | - | - | - | - | - | P | R | F | V | K | H | I | I | - | - | - | - | - | - | - | - | - | - | - | - | K | - | D | N | M | D | K | - | - | - | - | - | - | - | - | - | - | - | - | - | - | - | - | - | - | -39 |
| D7IBA4_9BACE | - | A | S | M | A | Y | L | Y | - | - | - | - | - | - | - | - | P | R | F | V | K | H | I | I | - | - | - | - | - | - | - | - | - | - | - | - | K | - | D | N | M | D | K | - | - | - | - | - | - | - | - | - | - | - | - | - | - | - | - | - | - | -37 |
| ZP_09858577 | - | A | S | M | A | Y | L | Y | - | - | - | - | - | - | - | - | P | R | F | V | K | H | I | I | - | - | - | - | - | - | - | - | - | - | - | - | K | - | D | N | M | D | K | - | - | - | - | - | - | - | - | - | - | - | - | - | - | - | - | - | - | -37 |
| E5C759_9BACE | - | A | S | M | A | Y | L | Y | - | - | - | - | - | - | - | - | P | R | F | I | K | H | I | I | - | - | - | - | - | - | - | - | - | - | - | - | K | - | D | N | K | D | K | - | - | - | - | - | - | - | - | - | - | - | - | - | - | - | - | - | - | -39 |
| A7LTS0_BACO1 | - | G | S | L | S | Y | V | Y | - | - | - | - | - | - | - | - | P | G | F | I | K | N | I | I | - | - | - | - | - | - | - | - | - | - | - | - | K | - | N | N | G | N | Q | - | - | - | - | - | - | - | - | - | - | - | - | - | - | - | - | - | - | -38 |
| Q8YQK8_NOSS1 | - | G | A | T | F | G | R | Q | F | N | D | A | G | N | T | S | S | S | V | I | N | S | M | I | - | - | - | - | - | - | - | - | - | - | - | - | T | - | D | N | G | D | N | - | - | - | - | - | - | - | - | - | - | - | - | - | - | - | - | - | - | -56 |
| Q3MBX8_ANAVT | - | G | A | T | F | G | R | Q | S | N | D | A | G | N | A | S | S | S | V | I | N | S | M | I | - | - | - | - | - | - | - | - | - | - | - | - | T | - | D | N | G | D | N | - | - | - | - | - | - | - | - | - | - | - | - | - | - | - | - | - | - | -56 |
| Q8YZT8_NOSS1 | - | G | A | T | F | S | R | Q | S | D | D | S | G | N | A | S | S | S | V | I | K | N | M | I | - | - | - | - | - | - | - | - | - | - | - | - | K | - | D | N | G | D | N | - | - | - | - | - | - | - | - | - | - | - | - | - | - | - | - | - | - | -44 |
| K9UIG2_9CHRO | - | G | A | T | F | A | P | Q | S | N | D | A | G | N | S | I | S | K | T | I | N | D | M | L | - | - | - | - | - | - | - | - | - | - | - | - | L | - | D | N | G | D | N | - | - | - | - | - | - | - | - | - | - | - | - | - | - | - | - | - | - | -47 |
| A4YRP8_BRASO | - | A | E | V | A | Y | K | E | - | - | - | - | - | - | - | - | Q | S | V | I | S | S | M | F | - | - | - | - | - | - | - | - | - | - | - | - | T | - | A | N | G | N | N | - | - | - | - | - | - | - | - | - | - | - | - | - | - | - | - | - | - | -42 |
| H0TAL7_9BRAD | - | A | E | V | A | Y | K | E | - | - | - | - | - | - | - | - | Q | S | V | I | S | S | M | F | - | - | - | - | - | - | - | - | - | - | - | - | T | - | A | N | G | N | N | - | - | - | - | - | - | - | - | - | - | - | - | - | - | - | - | - | - | -46 |
| H0SNL4_9BRAD | - | A | E | V | A | Y | K | E | - | - | - | - | - | - | - | - | Q | S | V | I | S | S | M | F | - | - | - | - | - | - | - | - | - | - | - | - | T | - | V | N | G | N | N | - | - | - | - | - | - | - | - | - | - | - | - | - | - | - | - | - | - | -48 |
| H0S4P6_9BRAD | - | A | E | V | A | Y | K | E | - | - | - | - | - | - | - | - | Q | S | V | I | S | S | M | F | - | - | - | - | - | - | - | - | - | - | - | - | T | - | A | N | G | N | N | - | - | - | - | - | - | - | - | - | - | - | - | - | - | - | - | - | - | -48 |
| A5EMK2_BRASB | - | A | E | V | A | Y | K | E | - | - | - | - | - | - | - | - | Q | S | V | I | S | S | M | F | - | - | - | - | - | - | - | - | - | - | - | - | T | - | A | N | G | N | G | - | - | - | - | - | - | - | - | - | - | - | - | - | - | - | - | - | - | -42 |
| M4ZBJ4_9BRAD | - | A | E | V | A | Y | K | E | - | - | - | - | - | - | - | - | Q | S | V | I | S | S | M | F | - | - | - | - | - | - | - | - | - | - | - | - | T | - | T | N | G | N | G | - | - | - | - | - | - | - | - | - | - | - | - | - | - | - | - | - | - | -48 |
| Q07SP7_RHOP5 | - | A | E | V | A | D | Q | N | - | - | - | - | - | - | - | - | P | D | L | I | S | S | M | F | - | - | - | - | - | - | - | - | - | - | - | - | T | - | S | N | G | N | G | - | - | - | - | - | - | - | - | - | - | - | - | - | - | - | - | - | - | -43 |
| B0JUQ7_MICAN | - | A | G | T | A | Y | R | T | - | - | - | - | - | - | - | - | P | S | T | I | Q | N | M | F | - | - | - | - | - | - | - | - | - | - | - | - | T | - | D | N | G | D | G | - | - | - | - | - | - | - | - | - | - | - | - | - | - | - | - | - | - | -46 |
| K9WJ87_9CYAN | - | A | G | T | A | F | R | S | - | - | - | - | - | - | - | - | P | D | T | I | R | D | M | F | - | - | - | - | - | - | - | - | - | - | - | - | I | - | D | N | N | D | G | - | - | - | - | - | - | - | - | - | - | - | - | - | - | - | - | - | - | -42 |
| G6FUE0_9CYAN | - | A | A | T | A | F | R | S | - | - | - | - | - | - | - | - | S | S | M | I | E | N | M | F | - | - | - | - | - | - | - | - | - | - | - | - | I | - | D | N | G | D | Q | - | - | - | - | - | - | - | - | - | - | - | - | - | - | - | - | - | - | -42 |
| K9PHG5_9CYAN | - | A | E | V | A | I | H | S | - | - | - | - | - | - | - | - | P | S | T | I | E | S | M | F | - | - | - | - | - | - | - | - | - | - | - | - | I | - | D | N | G | D | S | - | - | - | - | - | - | - | - | - | - | - | - | - | - | - | - | - | - | -42 |
| Q8YXX3_NOSS1 | - | A | N | T | A | L | R | T | - | - | - | - | - | - | - | - | P | N | V | I | Q | S | M | F | - | - | - | - | - | - | - | - | - | - | - | - | I | - | D | N | G | D | N | - | - | - | - | - | - | - | - | - | - | - | - | - | - | - | - | - | - | -46 |
| K9TN70_9CYAN | - | A | G | I | A | M | Q | D | - | - | - | - | - | - | - | - | S | Q | K | I | E | E | M | F | - | - | - | - | - | - | - | - | - | - | - | - | I | - | D | N | G | D | G | - | - | - | - | - | - | - | - | - | - | - | - | - | - | - | - | - | - | -42 |
| Q05QW3_9SYNE | - | A | S | T | A | H | E | K | - | - | - | - | - | - | - | - | P | S | I | I | R | D | M | F | - | - | - | - | - | - | - | - | - | - | - | - | E | - | D | N | G | D | G | - | - | - | - | - | - | - | - | - | - | - | - | - | - | - | - | - | - | -46 |
| Q05QW2_9SYNE | - | A | G | T | A | D | K | K | - | - | - | - | - | - | - | - | E | S | I | I | E | D | M | F | - | - | - | - | - | - | - | - | - | - | - | - | T | - | D | N | G | D | G | - | - | - | - | - | - | - | - | - | - | - | - | - | - | - | - | - | - | -46 |
| Q05QW4_9SYNE | - | A | G | T | A | N | D | K | - | - | - | - | - | - | - | - | A | E | M | I | Q | D | M | F | - | - | - | - | - | - | - | - | - | - | - | - | T | - | D | N | G | D | G | - | - | - | - | - | - | - | - | - | - | - | - | - | - | - | - | - | - | -46 |
| E0UGX6_CYAP2 | - | S | S | I | A | Q | E | K | - | - | - | - | - | - | - | - | P | S | Y | I | Q | N | M | F | - | - | - | - | - | - | - | - | - | - | - | - | T | - | D | N | G | D | N | - | - | - | - | - | - | - | - | - | - | - | - | - | - | - | - | - | - | -46 |
| K7WKF4_9NOST | - | A | S | I | A | Q | E | K | - | - | - | - | - | - | - | - | P | E | Y | I | Q | N | M | F | - | - | - | - | - | - | - | - | - | - | - | - | T | - | D | N | G | D | N | - | - | - | - | - | - | - | - | - | - | - | - | - | - | - | - | - | - | -42 |
| E0UGY5_CYAP2 | - | S | S | I | A | Q | E | K | - | - | - | - | - | - | - | - | P | S | N | I | Q | D | M | F | - | - | - | - | - | - | - | - | - | - | - | - | I | - | D | N | G | D | N | - | - | - | - | - | - | - | - | - | - | - | - | - | - | - | - | - | - | -46 |
| A8LCE5_FRASN | - | I | G | I | A | Q | A | D | - | - | - | - | - | - | - | - | P | G | L | L | R | R | N | L | - | - | - | - | - | - | - | - | - | - | - | - | R | - | E | N | P | N | G | - | - | - | - | - | - | - | - | - | - | - | - | - | - | - | - | - | - | -51 |
| D3CT47_9ACTO | - | I | G | I | V | R | A | D | - | - | - | - | - | - | - | - | P | G | L | L | R | R | N | L | - | - | - | - | - | - | - | - | - | - | - | - | R | - | E | N | P | N | G | - | - | - | - | - | - | - | - | - | - | - | - | - | - | - | - | - | - | -51 |
| Q2J4J6_FRASC | - | I | G | I | A | R | Q | D | - | - | - | - | - | - | - | - | P | G | L | L | A | E | R | L | - | - | - | - | - | - | - | - | - | - | - | - | R | - | A | N | P | N | G | - | - | - | - | - | - | - | - | - | - | - | - | - | - | - | - | - | - | -48 |
| Q0RAZ2_FRAAA | - | I | G | I | A | R | Q | D | - | - | - | - | - | - | - | - | P | A | L | L | T | E | R | L | - | - | - | - | - | - | - | - | - | - | - | - | R | - | R | N | P | N | G | - | - | - | - | - | - | - | - | - | - | - | - | - | - | - | - | - | - | -48 |
| E3J3F6_FRASU | - | V | G | L | A | R | Q | R | - | - | - | - | - | - | - | - | P | A | L | L | A | D | G | I | - | - | - | - | - | - | - | - | - | - | - | - | R | - | E | N | A | N | G | - | - | - | - | - | - | - | - | - | - | - | - | - | - | - | - | - | - | -48 |
| ZP_09168240 | - | I | G | L | A | R | Q | R | - | - | - | - | - | - | - | - | P | D | L | L | V | E | G | I | - | - | - | - | - | - | - | - | - | - | - | - | R | - | E | N | P | N | G | - | - | - | - | - | - | - | - | - | - | - | - | - | - | - | - | - | - | -49 |
| C7MDV9_BRAFD | - | L | A | I | H | E | T | A | - | - | - | - | - | - | - | - | P | E | R | L | R | G | L | L | - | - | - | - | - | - | - | - | - | - | - | - | S | - | A | E | E | G | G | - | - | - | - | - | - | - | - | - | - | - | - | - | - | - | - | - | - | -34 |
| ZP_10094989 | - | L | A | L | H | E | T | A | - | - | - | - | - | - | - | - | P | E | R | L | R | A | L | I | - | - | - | - | - | - | - | - | - | - | - | - | G | - | E | E | T | D | G | - | - | - | - | - | - | - | - | - | - | - | - | - | - | - | - | - | - | -34 |
| A4X268_SALTO | - | A | A | V | A | G | H | R | - | - | - | - | - | - | - | - | P | E | A | I | A | Q | L | F | - | - | - | - | - | - | - | - | - | - | - | - | Q | - | P | N | P | D | G | - | - | - | - | - | - | - | - | - | - | - | - | - | - | - | - | - | - | -36 |
| WP_043162213 | - | A | A | V | A | G | H | R | - | - | - | - | - | - | - | - | P | E | A | I | A | Q | L | F | - | - | - | - | - | - | - | - | - | - | - | - | Q | - | P | N | P | D | G | - | - | - | - | - | - | - | - | - | - | - | - | - | - | - | - | - | - | -36 |
| Q0BRV8_GRABC | - | G | E | L | A | M | W | Y | - | - | - | - | - | - | - | - | P | G | A | I | E | R | M | I | - | - | - | - | - | - | - | - | - | - | - | - | T | - | R | N | A | D | G | - | - | - | - | - | - | - | - | - | - | - | - | - | - | - | - | - | - | -48 |
| A9B2P0_HERA2 | - | A | A | I | A | N | T | R | - | - | - | - | - | - | - | - | P | D | I | I | R | N | A | I | - | - | - | - | - | - | - | - | - | - | - | - | R | - | D | N | G | D | G | - | - | - | - | - | - | - | - | - | - | - | - | - | - | - | - | - | - | -41 |
| A9B3U8_HERA2 | - | A | A | I | A | K | T | N | - | - | - | - | - | - | - | - | P | D | V | I | R | K | A | I | - | - | - | - | - | - | - | - | - | - | - | - | R | - | D | N | G | D | G | - | - | - | - | - | - | - | - | - | - | - | - | - | - | - | - | - | - | -41 |
| A9B2A3_HERA2 | - | A | A | V | A | Q | N | N | - | - | - | - | - | - | - | - | P | D | V | I | R | N | A | I | - | - | - | - | - | - | - | - | - | - | - | - | E | - | D | N | G | D | G | - | - | - | - | - | - | - | - | - | - | - | - | - | - | - | - | - | - | -42 |
| D6ZC85_SEGRD | - | A | A | V | A | F | A | Q | - | - | - | - | - | - | - | - | P | E | R | I | V | R | A | I | - | - | - | - | - | - | - | - | - | - | - | - | S | F | D | P | D | I | D | - | - | - | - | - | - | - | - | - | - | - | - | - | - | - | - | - | - | -45 |
| E5XTT9_9ACTO | - | A | A | V | A | H | T | Q | - | - | - | - | - | - | - | - | P | E | R | I | M | N | A | I | - | - | - | - | - | - | - | - | - | - | - | - | N | F | D | Q | A | T | G | - | - | - | - | - | - | - | - | - | - | - | - | - | - | - | - | - | - | -45 |
| E5XTU0_9ACTO | - | A | A | V | A | Y | A | Q | - | - | - | - | - | - | - | - | P | D | L | I | K | K | N | I | - | - | - | - | - | - | - | - | - | - | - | - | S | Y | D | P | K | T | G | - | - | - | - | - | - | - | - | - | - | - | - | - | - | - | - | - | - | -45 |
| D6B0I6_9ACTO | - | A | G | L | S | Q | Q | N | - | - | - | - | - | - | - | - | P | R | F | I | E | E | G | I | - | - | - | - | - | - | - | - | - | - | - | - | K | - | E | N | P | N | G | - | - | - | - | - | - | - | - | - | - | - | - | - | - | - | - | - | - | -48 |
| D9X719_STRVR | - | A | G | L | A | Q | K | D | - | - | - | - | - | - | - | - | P | A | F | V | Q | E | G | I | - | - | - | - | - | - | - | - | - | - | - | - | K | - | Q | N | P | N | G | - | - | - | - | - | - | - | - | - | - | - | - | - | - | - | - | - | - | -48 |
| H6L010_SAPGL | - | A | S | L | A | K | T | D | - | - | - | - | - | - | - | - | P | G | A | I | K | K | L | I | - | - | - | - | - | - | - | - | - | - | - | - | K | - | D | N | G | D | G | - | - | - | - | - | - | - | - | - | - | - | - | - | - | - | - | - | - | -38 |
| K1ZP83_9BACT | - | S | S | L | A | E | T | H | - | - | - | - | - | - | - | - | P | E | F | L | E | K | N | I | - | - | - | - | - | - | - | - | - | - | - | - | Q | - | K | N | C | D | G | - | - | - | - | - | - | - | - | - | - | - | - | - | - | - | - | - | - | -46 |
| ADGB_HUMAN | - | F | N | G | G | I | L | S | N | Y | F | K | G | T | S | G | E | P | P | L | L | P | W | K | P | W | E | H | I | Y | S | L | C | K | A | V | K | G | H | M | P | L | F | N | - | - | - | - | - | - | - | - | - | - | - | - | - | - | S | Y | G | -96 |
| J3QMP3_MOUSE | - | F | N | G | G | I | L | S | N | Y | H | K | G | N | L | G | E | L | P | I | L | P | W | K | P | W | E | H | I | Y | S | L | C | K | A | V | K | G | H | V | P | L | F | N | - | - | - | - | - | - | - | - | - | - | - | - | - | - | S | Y | G | -96 |
| H2XR46_CIOIN | - | N | N | N | N | V | P | D | S | H | H | S | S | V | D | G | D | P | - | - | - | G | W | S | P | W | E | H | I | Y | S | L | C | K | A | T | K | G | H | I | P | L | Y | N | - | - | - | - | - | - | - | - | - | - | - | - | - | - | Q | Y | G | -93 |
| CAN7_MOUSE | - | A | I | S | A | A | Y | E | R | R | F | N | K | K | L | I | T | S | I | I | Y | P | Q | N | - | - | - | - | - | - | - | - | - | - | - | - | K | D | G | E | P | E | Y | N | - | - | - | - | - | - | - | - | - | - | - | - | - | - | P | C | G | -70 |
| CAN7_HUMAN | - | A | I | S | A | A | Y | E | R | R | F | N | K | K | L | I | T | G | I | I | Y | P | Q | N | - | - | - | - | - | - | - | - | - | - | - | - | K | D | G | E | P | E | Y | N | - | - | - | - | - | - | - | - | - | - | - | - | - | - | P | C | G | -70 |
| E1BY84_CHICK | - | A | I | S | A | A | Y | E | R | R | Y | N | K | K | L | I | T | S | I | I | Y | P | Q | N | - | - | - | - | - | - | - | - | - | - | - | - | K | K | G | E | P | E | Y | N | - | - | - | - | - | - | - | - | - | - | - | - | - | - | P | C | G | -70 |
| H9GX91_DANRE | - | A | I | S | A | A | Y | E | R | R | Y | S | K | K | L | I | T | S | I | I | Y | P | Q | N | - | - | - | - | - | - | - | - | - | - | - | - | R | K | G | E | P | E | Y | N | - | - | - | - | - | - | - | - | - | - | - | - | - | - | P | C | G | -70 |
| F6SGI6_CIOIN | - | A | I | S | A | A | Y | E | R | K | Y | N | K | K | L | I | T | S | I | I | Y | P | Q | N | - | - | - | - | - | - | - | - | - | - | - | - | R | K | G | D | P | I | Y | N | - | - | - | - | - | - | - | - | - | - | - | - | - | - | P | C | G | -70 |
| W4Y195_STRPU | - | - | - | - | - | - | - | - | - | - | - | - | - | - | - | V | G | D | I | I | Y | P | Q | D | - | - | - | - | - | - | - | - | - | - | - | - | R | S | H | K | P | I | Y | N | - | - | - | - | - | - | - | - | - | - | - | - | - | - | P | C | G | -44 |
| R7V270_CAPTE | - | A | I | A | A | Q | Y | E | R | R | F | N | T | K | L | I | T | S | I | I | Y | P | Q | N | - | - | - | - | - | - | - | - | - | - | - | - | R | K | G | D | P | Q | Y | N | - | - | - | - | - | - | - | - | - | - | - | - | - | - | P | C | G | -70 |
| G4LXK3_SCHMA | - | A | I | A | A | Q | Y | E | R | R | F | K | K | K | L | I | T | N | V | I | Y | P | Q | G | - | - | - | - | - | - | - | - | - | - | - | - | I | N | G | E | A | I | Y | N | - | - | - | - | - | - | - | - | - | - | - | - | - | - | P | C | G | -70 |
| T2M2K0_HYDVU | - | A | I | S | A | A | Y | E | R | Q | F | Q | R | K | L | I | T | S | I | I | F | P | Q | Q | - | - | - | - | - | - | - | - | - | - | - | - | K | D | G | T | P | F | I | N | - | - | - | - | - | - | - | - | - | - | - | - | - | - | P | C | G | -70 |
| B3RQK1_TRIAD | - | A | V | S | A | A | Y | E | R | K | F | R | K | Q | I | I | T | S | C | I | F | P | Q | D | - | - | - | - | - | - | - | - | - | - | - | - | K | Q | K | R | P | I | V | N | - | - | - | - | - | - | - | - | - | - | - | - | - | - | P | C | G | -70 |
| I1CS59_RHIO9 | - | C | V | A | A | A | Y | E | Q | K | F | K | K | Q | L | I | T | S | C | V | Y | P | Q | N | - | - | - | - | - | - | - | - | - | - | - | - | K | Q | G | Q | P | C | Y | N | - | - | - | - | - | - | - | - | - | - | - | - | - | - | P | N | G | -70 |
| I1GER3_AMPQE | - | G | V | C | A | D | Y | E | R | R | F | K | R | K | L | I | T | S | C | I | F | P | Q | N | - | - | - | - | - | - | - | - | - | - | - | - | R | H | K | E | P | I | I | N | - | - | - | - | - | - | - | - | - | - | - | - | - | - | P | N | G | -70 |
| Q22143_CAEEL | - | S | I | A | A | L | Y | E | K | R | F | K | K | Q | L | V | T | S | I | I | F | P | Q | D | - | - | - | - | - | - | - | - | - | - | - | - | A | N | G | K | P | I | Y | N | - | - | - | - | - | - | - | - | - | - | - | - | - | - | P | A | G | -70 |
| L1JZN7_GUITH | - | A | V | C | A | S | W | E | R | R | Y | G | R | T | L | I | S | R | N | I | Y | P | Q | - | - | - | - | - | - | - | - | - | - | - | - | - | K | N | N | V | P | V | I | S | - | - | - | - | - | - | - | - | - | - | - | - | - | - | P | S | G | -69 |
| D0NT34_PHYIT | - | C | I | A | A | A | Y | E | Q | R | F | Q | K | H | L | I | T | N | I | I | F | P | A | D | P | - | - | - | - | - | - | - | - | - | - | - | R | T | K | Q | P | V | Y | N | - | - | - | - | - | - | - | - | - | - | - | - | - | - | P | F | G | -78 |
| A0A064B913_ASPOZ | - | C | A | T | T | S | M | L | E | R | G | Q | C | T | H | F | L | P | M | I | Y | P | S | R | - | - | - | - | - | - | - | - | - | - | - | - | - | - | G | S | S | Q | P | S | - | - | - | - | - | - | - | - | - | - | - | - | - | - | P | S | G | -77 |
| A8N4X4_COPC7 | - | S | V | C | L | E | H | G | R | R | F | G | S | Y | L | A | E | S | P | L | H | H | Y | C | D | - | - | - | - | - | - | - | - | - | - | - | A | E | K | W | V | M | R | I | G | M | G | S | K | L | - | - | - | - | F | F | S | Q | K | S | G | -89 |
| C0H5K1_PLAF7 | - | T | V | L | I | E | Y | E | K | K | H | K | I | P | V | I | S | S | I | I | T | P | C | S | W | N | Y | L | C | I | - | - | - | - | - | - | N | K | T | W | P | I | F | N | - | - | - | - | - | - | - | - | - | - | - | - | - | - | P | C | G | -77 |
| K1REZ8_CRAGI | - | E | K | L | T | S | C | T | - | - | - | - | - | - | - | - | - | D | I | I | K | H | L | I | P | N | - | - | - | - | - | - | - | - | - | D | T | L | R | T | V | Y | K | K | A | E | V | - | - | - | - | - | - | - | - | - | - | - | Y | V | G | -55 |
| F4NTM9_BATDJ | - | R | L | F | A | L | H | P | - | - | - | - | - | - | - | - | - | Q | V | F | K | Q | V | V | P | - | - | - | - | - | - | - | - | - | - | I | T | K | K | N | A | Y | K | T | S | G | S | S | C | V | E | - | - | - | - | E | S | N | H | P | G | -73 |
| Q6F6K5_CAEEL | - | Q | T | I | A | R | R | K | - | - | - | - | - | - | - | - | - | T | L | L | D | Q | I | V | P | - | - | - | - | - | - | - | - | - | - | - | - | - | K | R | W | F | T | M | K | - | - | - | - | - | - | - | - | - | - | - | - | - | - | H | G | -39 |
| A8HZK8_CHLRE | - | A | A | V | A | A | A | R | - | - | - | - | - | - | - | G | G | E | L | L | L | D | L | I | V | S | - | - | - | - | - | - | - | - | - | - | - | - | D | D | S | A | G | S | - | - | - | - | - | - | - | - | - | - | - | - | - | - | - | - | G | -38 |
| A8J6T6_CHLRE | - | A | C | L | A | N | K | P | - | - | - | - | - | - | - | - | - | G | A | I | E | E | M | F | - | - | - | - | - | - | - | - | - | - | - | - | L | A | G | K | V | Y | R | P | - | - | - | - | - | - | - | - | - | - | - | - | - | - | - | D | G | -68 |
| Z9JR51_9MICO | - | M | A | T | Q | Q | T | D | - | - | - | - | - | - | - | - | P | G | L | L | A | E | N | I | A | G | - | - | - | - | - | - | - | - | - | - | - | L | G | S | P | P | G | A | D | G | - | - | - | - | - | - | - | - | - | - | - | - | - | - | - | -57 |
| F7ZZH9_CELGA | - | V | A | L | L | N | T | D | A | G | - | - | - | - | - | - | D | A | F | I | R | D | H | V | R | - | - | - | - | - | - | - | - | - | - | - | - | W | D | A | E | R | E | G | - | - | - | - | - | - | - | - | - | - | - | - | - | - | - | - | - | -41 |
| Q7NDU2_GLOVI | - | I | G | E | L | S | Q | D | - | - | - | - | - | - | - | - | P | E | A | V | Q | E | R | F | G | - | - | - | - | - | - | - | - | - | - | - | - | - | Q | N | - | - | - | - | - | - | - | - | - | - | - | - | - | - | - | - | - | - | A | D | G | -41 |
| I6AT95_9BACT | - | G | S | M | V | C | R | N | - | - | - | - | - | - | - | P | K | D | V | V | Q | M | I | R | - | - | - | - | - | - | - | - | - | - | - | - | - | - | K | Q | G | D | G | S | - | - | - | - | - | - | - | - | - | - | - | - | - | - | - | - | - | -41 |
| C7MGJ8_BRAFD | - | L | A | V | V | H | E | D | - | - | - | - | - | - | - | - | P | S | - | - | - | - | - | - | - | - | - | - | - | - | - | - | - | - | - | W | P | A | E | H | V | V | D | N | G | - | - | - | - | - | - | - | - | - | - | - | - | - | - | D | G | -37 |
| D0WP73_9ACTO | - | M | A | L | T | K | S | P | - | - | - | - | - | - | - | - | - | - | - | - | - | - | - | - | - | - | - | - | - | - | - | - | - | - | - | - | E | G | R | Q | T | I | Q | D | - | - | - | - | - | - | - | - | - | - | - | - | - | - | - | - | - | -35 |
| B0C8L1_ACAM1 | - | G | S | I | A | H | T | N | - | - | - | - | - | - | - | - | P | E | F | I | K | N | M | M | K | - | - | - | - | - | - | - | - | - | - | - | D | K | G | E | T | V | S | V | R | - | - | - | - | - | - | - | - | - | - | - | - | - | - | - | - | -43 |
| B3E5A8_GEOLS | - | G | W | M | A | R | N | R | - | - | - | - | - | - | - | P | A | E | V | M | N | A | I | T | - | - | - | - | - | - | - | - | - | - | - | - | R | L | D | D | - | - | - | - | - | - | - | - | - | - | - | - | - | - | - | - | - | - | - | - | G | -40 |
| D4YQ94_9MICO | - | A | A | C | E | N | A | K | P | G | - | - | - | - | F | T | A | S | L | I | E | H | S | P | - | - | - | - | - | - | - | - | - | - | - | - | H | I | N | S | P | H | T | V | H | - | - | - | - | - | - | - | - | - | - | - | - | - | - | - | - | -45 |
| PAPA_CARPA | - | V | T | I | E | G | - | - | - | - | - | - | - | - | - | - | - | - | - | - | - | - | - | - | - | - | - | - | - | - | - | - | - | - | - | - | - | - | - | - | - | - | - | - | - | - | - | - | - | - | - | - | - | - | - | - | - | - | I | I | K | -39 |
| RIM13_YEAST | - | I | N | L | R | S | K | N | - | - | - | - | - | - | L | N | L | P | L | I | K | Q | I | S | - | - | - | - | - | - | - | - | - | - | - | - | - | - | - | - | - | - | - | - | - | - | - | - | - | - | - | - | - | - | - | - | - | - | - | S | T | -72 |

| I |  |  |  |  |  |  |  |  |  |  |  |  |  |  |  |  |  |  |  |  |  |  |  |  |  |  |  |  |  |  |  |  |  |  |  |  |  |  |  |  |  |  |  |  |  |  |  |  |  |  |  |  |  |  |  |  |  |  |  |  |  |  |
| --- | --- | --- | --- | --- | --- | --- | --- | --- | --- | --- | --- | --- | --- | --- | --- | --- | --- | --- | --- | --- | --- | --- | --- | --- | --- | --- | --- | --- | --- | --- | --- | --- | --- | --- | --- | --- | --- | --- | --- | --- | --- | --- | --- | --- | --- | --- | --- | --- | --- | --- | --- | --- | --- | --- | --- | --- | --- | --- | --- | --- | --- | --- |
| Q9N4B1_CAEEL | - | I | F | H | F | Q | F | W | R | Y | G | Q | - | - | - | - | - | - | - | - | - | - | - | - | - | - | - | - | - | - | - | - | - | - | - | W | V | D | V | V | I | D | D | R | L | P | T | V | - | - | - | - | - | - | - | - | - | - | - | - | - | -87 |
| Q8IAA8_CAEEL | - | I | F | H | F | Q | F | W | H | Y | G | K | - | - | - | - | - | - | - | - | - | - | - | - | - | - | - | - | - | - | - | - | - | - | - | W | V | D | V | V | V | D | D | R | L | P | T | V | - | - | - | - | - | - | - | - | - | - | - | - | - | -87 |
| CAN_CAEEL | - | I | F | H | F | Q | F | W | Q | Y | G | K | - | - | - | - | - | - | - | - | - | - | - | - | - | - | - | - | - | - | - | - | - | - | - | W | V | D | V | V | I | D | D | R | L | P | T | S | - | - | - | - | - | - | - | - | - | - | - | - | - | -87 |
| Q9U2B2_CAEEL | - | I | F | H | F | Q | F | W | R | Y | G | E | - | - | - | - | - | - | - | - | - | - | - | - | - | - | - | - | - | - | - | - | - | - | - | W | V | D | V | V | I | D | D | R | L | P | T | V | - | - | - | - | - | - | - | - | - | - | - | - | - | -87 |
| Q9N4B2_CAEEL | - | I | F | H | F | Q | F | W | R | Y | G | E | - | - | - | - | - | - | - | - | - | - | - | - | - | - | - | - | - | - | - | - | - | - | - | W | V | D | V | V | I | D | D | R | L | P | T | V | - | - | - | - | - | - | - | - | - | - | - | - | - | -87 |
| C3Y6P6_BRAFL | - | I | F | H | F | K | F | W | R | Y | G | E | - | - | - | - | - | - | - | - | - | - | - | - | - | - | - | - | - | - | - | - | - | - | - | W | I | D | V | V | V | D | D | R | I | P | M | V | M | D | P | V | Q | G | - | - | - | - | - | - | - | -93 |
| C3Y6N1_BRAFL | - | A | F | H | F | R | F | W | Q | Y | G | E | - | - | - | - | - | - | - | - | - | - | - | - | - | - | - | - | - | - | - | - | - | - | - | W | V | D | V | V | I | D | D | R | I | P | M | V | V | F | D | W | - | G | - | - | - | - | - | - | - | -94 |
| CANA_DROME | - | I | F | H | F | R | F | W | Q | Y | G | K | - | - | - | - | - | - | - | - | - | - | - | - | - | - | - | - | - | - | - | - | - | - | - | W | V | D | V | I | I | D | D | R | L | P | T | Y | N | G | - | - | - | - | - | - | - | - | - | - | - | -89 |
| CANB_DROME | - | I | F | H | F | K | F | W | Q | Y | G | K | - | - | - | - | - | - | - | - | - | - | - | - | - | - | - | - | - | - | - | - | - | - | - | W | V | E | V | V | I | D | D | R | L | P | T | Y | N | G | - | - | - | - | - | - | - | - | - | - | - | -89 |
| X1Y3H71_ANODA | - | V | F | H | F | R | F | W | Q | Y | G | K | - | - | - | - | - | - | - | - | - | - | - | - | - | - | - | - | - | - | - | - | - | - | - | W | V | D | V | V | I | D | D | R | L | P | T | Y | R | G | - | - | - | - | - | - | - | - | - | - | - | -89 |
| Q5IW12_GECLA | - | I | F | H | F | R | F | W | Q | Y | G | R | - | - | - | - | - | - | - | - | - | - | - | - | - | - | - | - | - | - | - | - | - | - | - | W | V | D | V | V | V | D | D | R | L | P | T | F | Y | G | - | - | - | - | - | - | - | - | - | - | - | -89 |
| K1Q056_CRAGI | - | M | F | R | F | H | F | W | R | Q | G | E | - | - | - | - | - | - | - | - | - | - | - | - | - | - | - | - | - | - | - | - | - | - | - | W | V | E | V | V | V | D | D | R | L | P | T | Y | Y | G | - | - | - | - | - | - | - | - | - | - | - | -89 |
| G4V6C0_SCHMA | - | M | F | W | F | R | F | W | R | F | G | D | - | - | - | - | - | - | - | - | - | - | - | - | - | - | - | - | - | - | - | - | - | - | - | W | Y | D | V | V | V | D | D | R | L | P | T | R | N | G | - | - | - | - | - | - | - | - | - | - | - | -93 |
| R7ULX9_CAPTE | - | V | F | R | F | E | I | W | Q | F | G | R | - | - | - | - | - | - | - | - | - | - | - | - | - | - | - | - | - | - | - | - | - | - | - | W | V | E | V | C | I | D | D | R | L | P | T | C | N | G | - | - | - | - | - | - | - | - | - | - | - | -89 |
| W4Z5Q7_STRPU | - | V | V | K | F | N | F | W | Q | Y | G | K | - | - | - | - | - | - | - | - | - | - | - | - | - | - | - | - | - | - | - | - | - | - | - | W | V | T | V | V | I | D | D | Q | L | P | T | Y | N | N | - | - | - | - | - | - | - | - | - | - | - | -86 |
| R7VIB6_CAPTE | - | V | F | H | F | K | I | W | Q | Y | G | K | - | - | - | - | - | - | - | - | - | - | - | - | - | - | - | - | - | - | - | - | - | - | - | W | M | D | V | V | V | D | D | R | L | P | T | L | N | G | - | - | - | - | - | - | - | - | - | - | - | -92 |
| T2MJ45_HYDVU | - | L | I | R | F | R | F | W | Q | Y | G | D | - | - | - | - | - | - | - | - | - | - | - | - | - | - | - | - | - | - | - | - | - | - | - | W | N | E | I | V | I | D | D | R | L | P | T | Y | N | N | - | - | - | - | - | - | - | - | - | - | - | -89 |
| R7UAB6_CAPTE | - | I | F | R | F | Q | F | W | Q | Y | G | K | - | - | - | - | - | - | - | - | - | - | - | - | - | - | - | - | - | - | - | - | - | - | - | W | T | E | I | L | V | D | D | R | L | P | T | Y | N | N | - | - | - | - | - | - | - | - | - | - | - | -89 |
| C3ZKN8_BRAFL | - | C | F | R | F | N | F | W | H | Y | G | T | - | - | - | - | - | - | - | - | - | - | - | - | - | - | - | - | - | - | - | - | - | - | - | W | T | E | V | V | V | D | D | Q | L | P | T | F | N | N | - | - | - | - | - | - | - | - | - | - | - | -90 |
| B3RJS5_TRIAD | - | I | F | H | F | Q | F | W | R | F | G | Q | - | - | - | - | - | - | - | - | - | - | - | - | - | - | - | - | - | - | - | - | - | - | - | W | T | D | V | I | I | D | D | L | L | P | T | Y | N | G | - | - | - | - | - | - | - | - | - | - | - | -89 |
| H2YVF0_CIOSA | - | M | F | H | F | Q | F | W | Q | Y | G | E | - | - | - | - | - | - | - | - | - | - | - | - | - | - | - | - | - | - | - | - | - | - | - | W | I | D | V | M | V | D | D | L | L | P | T | V | R | G | - | - | - | - | - | - | - | - | - | - | - | -89 |
| G4VAG2_SCHMA | - | V | V | R | F | R | F | W | R | F | G | H | - | - | - | - | - | - | - | - | - | - | - | - | - | - | - | - | - | - | - | - | - | - | - | W | V | E | V | L | I | D | D | R | L | P | V | R | Q | G | R | N | - | - | - | - | - | - | - | - | - | -92 |
| Smp_157500 | - | A | F | V | F | N | F | W | R | F | G | E | - | - | - | - | - | - | - | - | - | - | - | - | - | - | - | - | - | - | - | - | - | - | - | W | V | E | V | V | V | D | D | R | I | P | V | V | K | G | T | C | - | - | - | - | - | - | - | - | - | -92 |
| Q7SYA9_DANRE | - | I | F | H | F | Q | F | W | Q | F | G | E | - | - | - | - | - | - | - | - | - | - | - | - | - | - | - | - | - | - | - | - | - | - | - | W | V | D | I | V | I | D | D | R | L | P | V | K | D | G | - | - | - | - | - | - | - | - | - | - | - | -89 |
| A5PMP1_DANRE | - | I | F | H | F | Q | F | W | Q | F | G | E | - | - | - | - | - | - | - | - | - | - | - | - | - | - | - | - | - | - | - | - | - | - | - | W | V | D | I | V | I | D | D | R | L | P | V | K | D | K | - | - | - | - | - | - | - | - | - | - | - | -89 |
| CANX_CHICK | - | I | F | H | F | Q | I | W | Q | F | G | E | - | - | - | - | - | - | - | - | - | - | - | - | - | - | - | - | - | - | - | - | - | - | - | W | V | D | V | V | V | D | D | L | L | P | T | K | D | G | - | - | - | - | - | - | - | - | - | - | - | -89 |
| CAN1_HUMAN | - | I | F | H | F | Q | L | W | Q | F | G | E | - | - | - | - | - | - | - | - | - | - | - | - | - | - | - | - | - | - | - | - | - | - | - | W | V | D | V | V | V | D | D | L | L | P | I | K | D | G | - | - | - | - | - | - | - | - | - | - | - | -89 |
| CAN1_MOUSE | - | I | F | H | F | Q | L | W | Q | F | G | E | - | - | - | - | - | - | - | - | - | - | - | - | - | - | - | - | - | - | - | - | - | - | - | W | V | D | V | V | I | D | D | L | L | P | T | K | D | G | - | - | - | - | - | - | - | - | - | - | - | -89 |
| O42133_CHICK | - | I | F | H | F | Q | I | W | Q | F | G | E | - | - | - | - | - | - | - | - | - | - | - | - | - | - | - | - | - | - | - | - | - | - | - | W | Q | D | V | V | V | D | D | Y | L | P | T | K | D | G | - | - | - | - | - | - | - | - | - | - | - | -89 |
| Q7ZUR1_DANRE | - | I | F | H | F | Q | F | W | Q | F | G | E | - | - | - | - | - | - | - | - | - | - | - | - | - | - | - | - | - | - | - | - | - | - | - | W | V | D | V | V | I | D | D | R | L | P | T | K | D | G | - | - | - | - | - | - | - | - | - | - | - | -89 |
| E9QFN0_DANRE | - | I | F | H | F | Q | F | W | Q | F | G | D | - | - | - | - | - | - | - | - | - | - | - | - | - | - | - | - | - | - | - | - | - | - | - | W | V | D | V | V | I | D | D | R | L | P | V | R | D | G | - | - | - | - | - | - | - | - | - | - | - | -89 |
| CAN2_HUMAN | - | I | F | H | F | Q | F | W | Q | Y | G | E | - | - | - | - | - | - | - | - | - | - | - | - | - | - | - | - | - | - | - | - | - | - | - | W | V | E | V | V | V | D | D | R | L | P | T | K | D | G | - | - | - | - | - | - | - | - | - | - | - | -89 |
| CAN2_MOUSE | - | I | F | H | F | Q | F | W | Q | Y | G | E | - | - | - | - | - | - | - | - | - | - | - | - | - | - | - | - | - | - | - | - | - | - | - | W | V | E | V | V | V | D | D | R | L | P | T | K | D | G | - | - | - | - | - | - | - | - | - | - | - | -89 |
| CAN2_CHICK | - | I | F | H | F | Q | F | W | Q | Y | G | E | - | - | - | - | - | - | - | - | - | - | - | - | - | - | - | - | - | - | - | - | - | - | - | W | V | D | V | V | V | D | D | R | L | P | T | K | N | G | - | - | - | - | - | - | - | - | - | - | - | -89 |
| Q6DFZ8_DANRE | - | I | F | H | F | Q | F | W | Q | F | G | E | - | - | - | - | - | - | - | - | - | - | - | - | - | - | - | - | - | - | - | - | - | - | - | W | V | D | V | V | I | D | D | R | L | P | A | R | N | G | - | - | - | - | - | - | - | - | - | - | - | -89 |
| Q504F5_DANRE | - | I | F | H | F | Q | F | W | Q | F | G | E | - | - | - | - | - | - | - | - | - | - | - | - | - | - | - | - | - | - | - | - | - | - | - | W | V | D | V | V | I | D | D | R | L | P | V | K | D | G | - | - | - | - | - | - | - | - | - | - | - | -89 |
| Q5BLH5_DANRE | - | I | F | H | F | Q | F | W | Q | F | G | E | - | - | - | - | - | - | - | - | - | - | - | - | - | - | - | - | - | - | - | - | - | - | - | W | V | D | V | V | V | D | D | R | L | P | T | R | D | G | - | - | - | - | - | - | - | - | - | - | - | -89 |
| Q4VBH3_DANRE | - | I | F | H | F | Q | F | W | Q | Y | G | E | - | - | - | - | - | - | - | - | - | - | - | - | - | - | - | - | - | - | - | - | - | - | - | W | V | D | V | V | I | D | D | R | L | P | T | R | D | G | - | - | - | - | - | - | - | - | - | - | - | -89 |
| CAN8_RAT | - | I | F | H | F | Q | F | W | Q | Y | G | E | - | - | - | - | - | - | - | - | - | - | - | - | - | - | - | - | - | - | - | - | - | - | - | W | V | E | V | V | I | D | D | R | L | P | T | K | N | G | - | - | - | - | - | - | - | - | - | - | - | -89 |
| CAN8_MOUSE | - | I | F | H | F | Q | F | W | Q | Y | G | E | - | - | - | - | - | - | - | - | - | - | - | - | - | - | - | - | - | - | - | - | - | - | - | W | V | E | V | V | I | D | D | R | L | P | T | K | N | G | - | - | - | - | - | - | - | - | - | - | - | -89 |
| CAN8_HUMAN | - | I | F | H | F | Q | F | W | Q | Y | G | E | - | - | - | - | - | - | - | - | - | - | - | - | - | - | - | - | - | - | - | - | - | - | - | W | V | E | V | V | I | D | D | R | L | P | T | K | N | G | - | - | - | - | - | - | - | - | - | - | - | -89 |
| XP_426117 | - | I | F | H | F | Q | F | W | Q | Y | G | E | - | - | - | - | - | - | - | - | - | - | - | - | - | - | - | - | - | - | - | - | - | - | - | W | V | D | V | V | V | D | D | R | L | P | T | K | N | G | - | - | - | - | - | - | - | - | - | - | - | -89 |
| CAN9_MOUSE | - | I | F | H | F | Q | F | W | Q | H | S | E | - | - | - | - | - | - | - | - | - | - | - | - | - | - | - | - | - | - | - | - | - | - | - | W | L | D | V | V | I | D | D | R | L | P | T | F | K | D | - | - | - | - | - | - | - | - | - | - | - | -91 |
| CAN9_HUMAN | - | I | F | H | F | Q | F | W | Q | H | S | E | - | - | - | - | - | - | - | - | - | - | - | - | - | - | - | - | - | - | - | - | - | - | - | W | L | D | V | V | I | D | D | R | L | P | T | F | R | D | - | - | - | - | - | - | - | - | - | - | - | -89 |
| E1C6M3_CHICK | - | I | F | H | F | Q | F | W | Q | H | N | E | - | - | - | - | - | - | - | - | - | - | - | - | - | - | - | - | - | - | - | - | - | - | - | W | L | D | V | V | I | D | D | R | L | P | T | F | K | D | - | - | - | - | - | - | - | - | - | - | - | -89 |
| Q6DG44_DANRE | - | I | F | H | F | Q | F | W | Q | H | N | K | - | - | - | - | - | - | - | - | - | - | - | - | - | - | - | - | - | - | - | - | - | - | - | W | M | D | V | V | V | D | D | R | L | P | C | V | R | D | - | - | - | - | - | - | - | - | - | - | - | -89 |
| CAN3_HUMAN | - | I | F | H | F | Q | F | W | R | Y | G | E | - | - | - | - | - | - | - | - | - | - | - | - | - | - | - | - | - | - | - | - | - | - | - | W | V | D | V | V | I | D | D | C | L | P | T | Y | N | N | - | - | - | - | - | - | - | - | - | - | - | -89 |
| CAN3_MOUSE | - | I | F | H | F | Q | F | W | R | Y | G | D | - | - | - | - | - | - | - | - | - | - | - | - | - | - | - | - | - | - | - | - | - | - | - | W | V | D | V | V | I | D | D | C | L | P | T | Y | N | N | - | - | - | - | - | - | - | - | - | - | - | -89 |
| CAN3_CHICK | - | I | F | H | F | Q | F | W | R | Y | G | D | - | - | - | - | - | - | - | - | - | - | - | - | - | - | - | - | - | - | - | - | - | - | - | W | V | D | V | I | I | D | D | C | L | P | T | Y | N | N | - | - | - | - | - | - | - | - | - | - | - | -89 |
| Q66HU4_DANRE | - | I | F | H | F | Q | F | W | R | Y | G | D | - | - | - | - | - | - | - | - | - | - | - | - | - | - | - | - | - | - | - | - | - | - | - | W | V | D | V | V | V | D | D | R | I | P | T | F | N | N | - | - | - | - | - | - | - | - | - | - | - | -89 |
| F1R632_DANRE | - | I | F | H | F | Q | F | W | R | Y | G | E | - | - | - | - | - | - | - | - | - | - | - | - | - | - | - | - | - | - | - | - | - | - | - | W | V | D | V | V | I | D | D | R | L | P | T | Y | K | N | - | - | - | - | - | - | - | - | - | - | - | -89 |
| CAN11_HUMAN | - | I | F | H | F | Q | I | W | Q | F | G | Q | - | - | - | - | - | - | - | - | - | - | - | - | - | - | - | - | - | - | - | - | - | - | - | W | V | N | V | V | V | D | D | R | L | P | T | K | N | D | - | - | - | - | - | - | - | - | - | - | - | -89 |
| CAN11_MOUSE | - | I | F | H | F | Q | L | W | Q | F | G | H | - | - | - | - | - | - | - | - | - | - | - | - | - | - | - | - | - | - | - | - | - | - | - | W | L | N | V | V | V | D | D | R | L | P | T | R | N | N | - | - | - | - | - | - | - | - | - | - | - | -89 |
| CAN12_MOUSE | - | V | F | H | F | Q | L | W | Q | F | G | R | - | - | - | - | - | - | - | - | - | - | - | - | - | - | - | - | - | - | - | - | - | - | - | W | V | D | V | V | V | D | D | K | L | P | V | R | E | G | - | - | - | - | - | - | - | - | - | - | - | -89 |
| CAN12_HUMAN | - | V | F | H | F | Q | L | W | Q | F | G | R | - | - | - | - | - | - | - | - | - | - | - | - | - | - | - | - | - | - | - | - | - | - | - | W | M | D | V | V | V | D | D | R | L | P | V | R | E | G | - | - | - | - | - | - | - | - | - | - | - | -89 |
| ENSSARP00000008135 | - | V | F | H | F | Q | L | W | Q | F | G | R | - | - | - | - | - | - | - | - | - | - | - | - | - | - | - | - | - | - | - | - | - | - | - | W | V | D | V | V | V | D | D | R | L | P | V | R | N | G | - | - | - | - | - | - | - | - | - | - | - | -89 |
| ENSSARP00000010553 | - | X | X | X | X | X | X | X | X | X | X | X | - | - | - | - | - | - | - | - | - | - | - | - | - | - | - | - | - | - | - | - | - | - | - | X | X | X | X | X | X | X | X | X | X | X | X | X | X | X | - | - | - | - | - | - | - | - | - | - | - | -88 |
| A7E2G3_DANRE | - | I | F | R | F | K | F | W | Q | Y | G | E | - | - | - | - | - | - | - | - | - | - | - | - | - | - | - | - | - | - | - | - | - | - | - | W | V | E | V | V | V | D | D | R | L | P | V | R | E | G | - | - | - | - | - | - | - | - | - | - | - | -89 |
| Q6NW69_DANRE | - | C | F | C | F | Q | F | W | Q | Y | G | E | - | - | - | - | - | - | - | - | - | - | - | - | - | - | - | - | - | - | - | - | - | - | - | W | I | S | V | K | I | D | D | L | L | P | T | C | N | N | - | - | - | - | - | - | - | - | - | - | - | -89 |
| CAN13_HUMAN | - | I | F | R | F | R | F | W | Q | C | G | Q | - | - | - | - | - | - | - | - | - | - | - | - | - | - | - | - | - | - | - | - | - | - | - | W | V | E | V | V | I | D | D | R | L | P | V | Q | G | D | - | - | - | - | - | - | - | - | - | - | - | -89 |
| CAN13_MOUSE | - | I | F | Q | F | R | F | W | Q | C | G | Q | - | - | - | - | - | - | - | - | - | - | - | - | - | - | - | - | - | - | - | - | - | - | - | W | V | E | V | V | I | D | D | Q | L | P | V | I | G | D | - | - | - | - | - | - | - | - | - | - | - | -91 |
| E1BRR5_CHICK | - | I | F | H | F | R | F | W | Q | F | G | D | - | - | - | - | - | - | - | - | - | - | - | - | - | - | - | - | - | - | - | - | - | - | - | W | V | D | V | V | I | D | D | R | L | P | F | L | N | G | - | - | - | - | - | - | - | - | - | - | - | -89 |
| E7F0H3_DANRE | - | I | F | H | F | R | F | W | R | F | E | K | - | - | - | - | - | - | - | - | - | - | - | - | - | - | - | - | - | - | - | - | - | - | - | W | I | D | V | V | I | D | D | Q | L | P | T | I | D | D | - | - | - | - | - | - | - | - | - | - | - | -90 |
| Q1RLS9_DANRE | - | I | F | H | F | R | F | W | R | F | G | S | - | - | - | - | - | - | - | - | - | - | - | - | - | - | - | - | - | - | - | - | - | - | - | W | V | D | V | V | I | D | D | K | L | P | T | I | D | N | - | - | - | - | - | - | - | - | - | - | - | -89 |
| F1Q9Z1_DANRE | - | L | F | H | F | R | F | W | R | F | G | K | - | - | - | - | - | - | - | - | - | - | - | - | - | - | - | - | - | - | - | - | - | - | - | W | F | D | V | V | I | D | D | K | L | P | T | I | N | R | - | - | - | - | - | - | - | - | - | - | - | -90 |
| A4QNV6_DANRE | - | I | F | H | F | R | F | W | R | F | G | K | - | - | - | - | - | - | - | - | - | - | - | - | - | - | - | - | - | - | - | - | - | - | - | W | I | D | V | V | I | D | D | K | L | P | T | I | K | G | - | - | - | - | - | - | - | - | - | - | - | -90 |
| D6W573_HUMAN | - | I | F | R | F | W | F | W | H | Y | G | N | - | - | - | - | - | - | - | - | - | - | - | - | - | - | - | - | - | - | - | - | - | - | - | W | V | P | V | V | I | D | D | R | L | P | V | N | E | A | G | - | - | - | - | - | - | - | - | - | - | -90 |
| R7UBC6_CAPTE | - | V | F | C | F | R | F | W | R | Y | G | Q | - | - | - | - | - | - | - | - | - | - | - | - | - | - | - | - | - | - | - | - | - | - | - | W | Q | E | V | V | I | D | D | R | L | P | T | I | N | G | - | - | - | - | - | - | - | - | - | - | - | -94 |
| K1RCZ2_CRAGI | - | I | F | R | F | N | F | W | H | F | G | R | - | - | - | - | - | - | - | - | - | - | - | - | - | - | - | - | - | - | - | - | - | - | - | W | V | E | V | V | V | D | D | R | L | P | T | Y | R | G | - | - | - | - | - | - | - | - | - | - | - | -96 |
| Q5IW13_GECLA | - | I | F | H | F | K | F | W | Q | Y | G | R | - | - | - | - | - | - | - | - | - | - | - | - | - | - | - | - | - | - | - | - | - | - | - | W | Q | E | V | V | I | D | D | L | L | P | T | H | N | G | - | - | - | - | - | - | - | - | - | - | - | -89 |
| R7UUX7_CAPTE | - | I | F | M | F | R | F | W | H | F | S | A | - | - | - | - | - | - | - | - | - | - | - | - | - | - | - | - | - | - | - | - | - | - | - | W | T | E | V | V | I | D | D | L | L | P | T | V | N | G | - | - | - | - | - | - | - | - | - | - | - | -91 |
| K1Q505_CRAGI | - | I | F | H | F | N | F | W | W | Y | G | R | - | - | - | - | - | - | - | - | - | - | - | - | - | - | - | - | - | - | - | - | - | - | - | W | V | E | V | I | V | D | D | F | L | P | T | D | G | Y | - | - | - | - | - | - | - | - | - | - | - | -89 |
| R7UD02_CAPTE | - | I | F | R | F | N | F | W | R | Y | G | V | - | - | - | - | - | - | - | - | - | - | - | - | - | - | - | - | - | - | - | - | - | - | - | W | T | E | V | V | V | D | D | R | L | P | T | K | N | N | - | - | - | - | - | - | - | - | - | - | - | -89 |
| CAN6_HUMAN | - | I | F | H | F | R | F | W | H | F | G | E | - | - | - | - | - | - | - | - | - | - | - | - | - | - | - | - | - | - | - | - | - | - | - | W | T | E | V | V | I | D | D | L | L | P | T | I | N | - | - | - | - | - | - | - | - | - | - | - | - | -94 |
| CAN6_MOUSE | - | I | F | H | F | R | F | W | H | F | G | E | - | - | - | - | - | - | - | - | - | - | - | - | - | - | - | - | - | - | - | - | - | - | - | W | T | E | V | V | I | D | D | L | L | P | T | I | N | - | - | - | - | - | - | - | - | - | - | - | - | -94 |
| E1C312_CHICK | - | I | F | R | F | R | F | W | C | F | G | I | - | - | - | - | - | - | - | - | - | - | - | - | - | - | - | - | - | - | - | - | - | - | - | W | T | E | V | V | I | D | D | L | L | P | T | I | D | - | - | - | - | - | - | - | - | - | - | - | - | -94 |
| E7FDN3_DANRE | - | I | F | H | F | R | F | W | R | L | G | Q | - | - | - | - | - | - | - | - | - | - | - | - | - | - | - | - | - | - | - | - | - | - | - | W | T | D | V | V | V | D | D | R | L | P | V | S | G | D | - | - | - | - | - | - | - | - | - | - | - | -95 |
| CAN5_HUMAN | - | I | F | H | F | H | F | W | R | F | G | E | - | - | - | - | - | - | - | - | - | - | - | - | - | - | - | - | - | - | - | - | - | - | - | W | V | D | V | V | I | D | D | R | L | P | T | V | N | - | - | - | - | - | - | - | - | - | - | - | - | -90 |
| CAN5_MOUSE | - | I | F | H | F | N | F | W | R | F | G | E | - | - | - | - | - | - | - | - | - | - | - | - | - | - | - | - | - | - | - | - | - | - | - | W | V | D | V | I | V | D | D | R | L | P | T | V | N | - | - | - | - | - | - | - | - | - | - | - | - | -94 |
| E1C292_CHICK | - | I | F | H | F | Q | F | W | R | F | G | Q | - | - | - | - | - | - | - | - | - | - | - | - | - | - | - | - | - | - | - | - | - | - | - | W | L | D | V | V | I | D | D | R | L | P | T | L | H | - | - | - | - | - | - | - | - | - | - | - | - | -94 |
| A0JMG3_DANRE | - | I | F | H | F | R | F | W | R | F | G | D | - | - | - | - | - | - | - | - | - | - | - | - | - | - | - | - | - | - | - | - | - | - | - | W | V | G | V | V | I | D | D | R | L | P | T | A | N | - | - | - | - | - | - | - | - | - | - | - | - | -94 |
| E7F7F1_DANRE | - | I | F | H | F | R | F | W | R | F | G | E | - | - | - | - | - | - | - | - | - | - | - | - | - | - | - | - | - | - | - | - | - | - | - | W | V | D | V | V | I | D | D | R | L | P | T | V | D | - | - | - | - | - | - | - | - | - | - | - | - | -94 |
| XP_006824412 | - | I | F | H | F | R | F | W | R | F | G | E | - | - | - | - | - | - | - | - | - | - | - | - | - | - | - | - | - | - | - | - | - | - | - | W | L | D | V | V | V | D | D | R | L | P | T | I | N | - | - | - | - | - | - | - | - | - | - | - | - | -88 |
| W4YJN2_STRPU | - | I | F | H | F | R | F | W | R | F | G | K | - | - | - | - | - | - | - | - | - | - | - | - | - | - | - | - | - | - | - | - | - | - | - | W | T | D | V | V | I | D | D | R | L | P | T | H | G | - | - | - | - | - | - | - | - | - | - | - | - | -94 |
| C3YJZ3_BRAFL | - | I | F | R | F | R | F | W | R | F | G | T | - | - | - | - | - | - | - | - | - | - | - | - | - | - | - | - | - | - | - | - | - | - | - | W | T | E | V | V | V | D | D | L | L | P | T | I | N | - | - | - | - | - | - | - | - | - | - | - | - | -94 |
| T2MIT4_HYDVU | - | I | F | R | F | R | F | W | R | F | G | D | - | - | - | - | - | - | - | - | - | - | - | - | - | - | - | - | - | - | - | - | - | - | - | W | I | E | V | V | I | D | D | R | L | P | T | I | N | - | - | - | - | - | - | - | - | - | - | - | - | -94 |
| XP_002154722 | - | I | F | R | F | R | F | W | R | F | G | E | - | - | - | - | - | - | - | - | - | - | - | - | - | - | - | - | - | - | - | - | - | - | - | W | I | E | V | V | I | D | D | R | L | P | T | I | N | - | - | - | - | - | - | - | - | - | - | - | - | -80 |
| R7VLA7_CAPTE | - | I | Y | Y | F | R | F | W | R | F | G | E | - | - | - | - | - | - | - | - | - | - | - | - | - | - | - | - | - | - | - | - | - | - | - | W | T | D | V | V | I | D | D | L | L | P | T | R | N | - | - | - | - | - | - | - | - | - | - | - | - | -94 |
| K1R1C6_CRAGI | - | I | F | H | F | H | F | W | R | F | G | E | - | - | - | - | - | - | - | - | - | - | - | - | - | - | - | - | - | - | - | - | - | - | - | W | I | D | V | V | I | D | D | F | L | P | T | Q | N | - | - | - | - | - | - | - | - | - | - | - | - | -94 |
| B3RIE5_TRIAD | - | I | V | H | F | R | F | W | R | F | G | E | - | - | - | - | - | - | - | - | - | - | - | - | - | - | - | - | - | - | - | - | - | - | - | W | V | D | V | V | I | D | D | R | L | P | M | V | D | - | - | - | - | - | - | - | - | - | - | - | - | -93 |
| K1PW75_CRAGI | - | I | F | H | F | R | F | W | Q | M | G | E | - | - | - | - | - | - | - | - | - | - | - | - | - | - | - | - | - | - | - | - | - | - | - | W | I | D | V | V | I | D | D | R | L | P | T | R | H | - | - | - | - | - | - | - | - | - | - | - | - | -92 |
| K1RSB8_CRAGI | - | I | F | H | F | K | F | W | Q | C | G | E | - | - | - | - | - | - | - | - | - | - | - | - | - | - | - | - | - | - | - | - | - | - | - | W | V | D | V | V | I | D | D | F | L | P | T | R | Q | - | - | - | - | - | - | - | - | - | - | - | - | -92 |
| K1RWI3_CRAGI | - | I | F | H | F | R | F | W | I | C | G | E | - | - | - | - | - | - | - | - | - | - | - | - | - | - | - | - | - | - | - | - | - | - | - | W | S | D | V | V | V | D | D | Y | L | P | T | R | N | - | - | - | - | - | - | - | - | - | - | - | - | -94 |
| R7VGC2_CAPTE | - | I | F | H | F | Q | F | W | R | Y | G | K | - | - | - | - | - | - | - | - | - | - | - | - | - | - | - | - | - | - | - | - | - | - | - | W | V | D | I | V | I | D | D | Q | L | P | T | K | N | - | - | - | - | - | - | - | - | - | - | - | - | -93 |
| K1QU26_CRAGI | - | V | F | H | F | H | F | W | R | F | G | E | - | - | - | - | - | - | - | - | - | - | - | - | - | - | - | - | - | - | - | - | - | - | - | W | V | D | I | V | I | D | D | R | L | P | T | K | N | - | - | - | - | - | - | - | - | - | - | - | - | -95 |
| CAN5_CAEEL | - | I | F | R | F | R | F | W | R | F | G | K | - | - | - | - | - | - | - | - | - | - | - | - | - | - | - | - | - | - | - | - | - | - | - | W | V | E | V | V | I | D | D | L | L | P | T | R | D | - | - | - | - | - | - | - | - | - | - | - | - | -92 |
| I1FGV4_AMPQE | - | I | F | R | F | H | F | W | H | F | G | E | - | - | - | - | - | - | - | - | - | - | - | - | - | - | - | - | - | - | - | - | - | - | - | W | I | D | I | V | I | D | D | R | L | P | T | Y | N | - | - | - | - | - | - | - | - | - | - | - | - | -88 |
| K1PHX3_CRAGI | - | I | F | H | F | R | F | W | R | Y | G | E | - | - | - | - | - | - | - | - | - | - | - | - | - | - | - | - | - | - | - | - | - | - | - | W | I | D | A | V | I | D | D | R | L | P | T | I | N | G | - | - | - | - | - | - | - | - | - | - | - | -89 |
| B3RNL8_TRIAD | - | I | L | H | V | R | F | W | R | M | G | Q | - | - | - | - | - | - | - | - | - | - | - | - | - | - | - | - | - | - | - | - | - | - | - | W | A | D | I | V | I | D | D | R | L | P | C | F | E | K | S | - | - | - | - | - | - | - | - | - | - | -90 |
| X1Y3H7_ANODA | - | V | F | H | F | R | F | W | H | Y | G | R | - | - | - | - | - | - | - | - | - | - | - | - | - | - | - | - | - | - | - | - | - | - | - | W | V | D | V | V | V | D | D | Q | L | P | V | C | R | G | - | - | - | - | - | - | - | - | - | - | - | -92 |
| R7TWH9_CAPTE | - | C | V | H | F | C | F | W | R | F | G | S | - | - | - | - | - | - | - | - | - | - | - | - | - | - | - | - | - | - | - | - | - | - | - | W | V | D | V | V | I | D | D | R | L | P | V | K | E | G | E | - | - | - | - | - | - | - | - | - | - | -94 |
| K1R5E8_CRAGI | - | V | V | H | F | R | F | W | R | F | G | D | - | - | - | - | - | - | - | - | - | - | - | - | - | - | - | - | - | - | - | - | - | - | - | W | V | D | V | Y | I | D | D | L | L | P | T | V | N | G | K | - | - | - | - | - | - | - | - | - | - | -94 |
| XP_006811804 | - | L | F | H | F | R | F | W | R | F | G | K | - | - | - | - | - | - | - | - | - | - | - | - | - | - | - | - | - | - | - | - | - | - | - | W | V | D | V | I | I | D | D | R | I | P | V | R | R | D | Q | - | - | - | - | - | - | - | - | - | - | -92 |
| C3YB23_BRAFL | - | M | F | H | F | K | F | W | R | Y | G | K | - | - | - | - | - | - | - | - | - | - | - | - | - | - | - | - | - | - | - | - | - | - | - | W | V | D | T | I | V | D | D | M | L | P | T | M | A | G | Q | Q | - | - | - | - | - | - | - | - | - | -99 |
| H2ZIF1_CIOSA | - | M | F | H | F | R | F | W | R | F | G | Q | - | - | - | - | - | - | - | - | - | - | - | - | - | - | - | - | - | - | - | - | - | - | - | W | V | D | V | I | V | D | D | N | L | P | T | L | R | G | S | - | - | - | - | - | - | - | - | - | - | -91 |
| W4YV93_STRPU | - | M | F | R | F | R | F | W | R | F | G | R | - | - | - | - | - | - | - | - | - | - | - | - | - | - | - | - | - | - | - | - | - | - | - | W | I | T | V | V | I | D | D | R | L | P | V | S | H | Q | G | R | - | - | - | - | - | - | - | - | - | -91 |
| CAN10_HUMAN | - | S | F | T | C | R | I | W | Q | F | G | R | - | - | - | - | - | - | - | - | - | - | - | - | - | - | - | - | - | - | - | - | - | - | - | W | V | E | V | T | T | D | D | R | L | P | C | - | L | A | G | - | - | - | - | - | - | - | - | - | - | -91 |
| CAN10_MOUSE | - | F | F | T | C | R | I | W | Q | F | G | H | - | - | - | - | - | - | - | - | - | - | - | - | - | - | - | - | - | - | - | - | - | - | - | W | E | E | V | T | I | D | D | R | L | P | C | - | L | A | G | - | - | - | - | - | - | - | - | - | - | -91 |
| XP_004943443 | - | R | F | T | C | H | V | W | Q | F | G | H | - | - | - | - | - | - | - | - | - | - | - | - | - | - | - | - | - | - | - | - | - | - | - | W | V | E | V | T | I | D | D | R | L | P | C | - | L | G | G | - | - | - | - | - | - | - | - | - | - | -91 |
| F8W4K1_DANRE | - | E | F | L | F | R | F | W | Q | N | G | H | - | - | - | - | - | - | - | - | - | - | - | - | - | - | - | - | - | - | - | - | - | - | - | W | I | E | V | R | V | D | D | R | L | P | C | - | I | N | D | - | - | - | - | - | - | - | - | - | - | -91 |
| CANC_DROME | - | V | F | R | F | R | L | W | W | C | G | E | - | - | - | - | - | - | - | - | - | - | - | - | - | - | - | - | - | - | - | - | - | - | - | W | V | E | V | L | V | D | D | R | L | P | T | - | I | N | G | - | - | - | - | - | - | - | - | - | - | -77 |
| C3ZKN9_BRAFL | - | L | F | S | F | R | L | W | G | P | D | G | - | - | - | - | - | - | - | - | - | - | - | - | - | - | - | - | - | - | - | - | - | - | - | W | T | E | V | A | V | D | D | R | L | P | T | - | I | D | G | - | - | - | - | - | - | - | - | - | - | -89 |
| G4V623_SCHMA | - | I | F | A | F | R | F | W | R | F | G | R | - | - | - | - | - | - | - | - | - | - | - | - | - | - | - | - | - | - | - | - | - | - | - | W | E | E | V | Y | I | D | D | R | L | P | V | K | S | G | G | - | - | - | - | - | - | - | - | - | - | -108 |
| R7VK39_CAPTE | - | E | F | S | F | N | F | W | K | W | G | E | - | - | - | - | - | - | - | - | - | - | - | - | - | - | - | - | - | - | - | - | - | - | - | W | T | T | V | S | V | D | D | R | L | P | V | A | L | D | G | - | - | - | - | - | - | - | - | - | - | -91 |
| G4V693_SCHMA | - | I | F | R | F | R | F | W | Q | E | G | V | - | - | - | - | - | - | - | - | - | - | - | - | - | - | - | - | - | - | - | - | - | - | - | Y | F | E | V | T | I | D | D | M | L | P | C | Y | E | G | - | - | - | - | - | - | - | - | - | - | - | -94 |
| R7UPU6_CAPTE | - | V | F | H | C | R | F | W | K | F | G | Q | - | - | - | - | - | - | - | - | - | - | - | - | - | - | - | - | - | - | - | - | - | - | - | W | I | D | V | Y | C | D | D | Y | L | P | V | K | Q | W | G | - | - | - | - | - | - | - | - | - | - | -92 |
| R7V817_CAPTE | - | V | F | H | C | R | F | W | K | F | G | Q | - | - | - | - | - | - | - | - | - | - | - | - | - | - | - | - | - | - | - | - | - | - | - | W | I | D | V | Y | C | D | D | Y | L | P | V | K | Q | W | G | - | - | - | - | - | - | - | - | - | - | -89 |
| R7UNL8_CAPTE | - | L | F | H | C | K | L | W | R | F | G | Q | - | - | - | - | - | - | - | - | - | - | - | - | - | - | - | - | - | - | - | - | - | - | - | W | V | D | V | Y | V | D | D | L | L | P | V | K | L | Y | G | - | - | - | - | - | - | - | - | - | - | -87 |
| K1PJ80_CRAGI | - | I | F | H | C | R | F | W | R | F | G | I | - | - | - | - | - | - | - | - | - | - | - | - | - | - | - | - | - | - | - | - | - | - | - | W | D | D | V | F | I | D | D | C | L | P | I | I | Y | G | N | - | - | - | - | - | - | - | - | - | - | -92 |
| K1PCX8_CRAGI | - | I | F | H | C | R | F | W | R | F | G | I | - | - | - | - | - | - | - | - | - | - | - | - | - | - | - | - | - | - | - | - | - | - | - | W | D | D | V | F | I | D | D | Y | L | P | I | I | Y | K | K | - | - | - | - | - | - | - | - | - | - | -92 |
| CAND_DROME | - | A | Y | Q | V | R | L | C | K | D | G | K | - | - | - | - | - | - | - | - | - | - | - | - | - | - | - | - | - | - | - | - | - | - | - | W | T | T | V | L | V | D | D | L | L | P | C | D | K | - | - | - | - | - | - | - | - | - | - | - | - | -90 |
| XP_002735765 | - | A | Y | Q | L | R | L | C | K | D | G | I | - | - | - | - | - | - | - | - | - | - | - | - | - | - | - | - | - | - | - | - | - | - | - | W | Q | T | V | L | I | D | D | L | F | P | C | D | Q | - | - | - | - | - | - | - | - | - | - | - | - | -92 |
| CAN15_HUMAN | - | A | Y | Q | V | R | L | C | K | D | G | T | - | - | - | - | - | - | - | - | - | - | - | - | - | - | - | - | - | - | - | - | - | - | - | W | T | T | V | L | V | D | D | M | L | P | C | D | E | - | - | - | - | - | - | - | - | - | - | - | - | -92 |
| CAN15_MOUSE | - | A | Y | Q | V | R | L | C | K | D | G | T | - | - | - | - | - | - | - | - | - | - | - | - | - | - | - | - | - | - | - | - | - | - | - | W | T | T | V | L | V | D | D | M | L | P | C | D | E | - | - | - | - | - | - | - | - | - | - | - | - | -92 |
| E1BTA8_CHICK | - | A | Y | Q | V | R | L | C | K | D | G | T | - | - | - | - | - | - | - | - | - | - | - | - | - | - | - | - | - | - | - | - | - | - | - | W | T | T | V | L | V | D | D | M | L | P | C | D | E | - | - | - | - | - | - | - | - | - | - | - | - | -92 |
| E7FAX5_DANRE | - | A | Y | Q | V | R | L | C | K | D | G | M | - | - | - | - | - | - | - | - | - | - | - | - | - | - | - | - | - | - | - | - | - | - | - | W | T | T | V | L | V | D | D | M | L | P | C | D | E | - | - | - | - | - | - | - | - | - | - | - | - | -92 |
| C3XWK8_BRAFL | - | A | Y | Q | V | R | L | C | K | D | G | E | - | - | - | - | - | - | - | - | - | - | - | - | - | - | - | - | - | - | - | - | - | - | - | W | M | T | V | L | V | D | D | L | L | P | C | D | H | - | - | - | - | - | - | - | - | - | - | - | - | -91 |
| W4YMU4_STRPU | - | A | Y | Q | V | R | L | C | R | D | G | I | - | - | - | - | - | - | - | - | - | - | - | - | - | - | - | - | - | - | - | - | - | - | - | W | D | M | V | L | V | D | D | L | L | P | C | Q | E | - | - | - | - | - | - | - | - | - | - | - | - | -92 |
| K1PV74_CRAGI | - | A | Y | Q | V | R | L | C | K | D | G | K | - | - | - | - | - | - | - | - | - | - | - | - | - | - | - | - | - | - | - | - | - | - | - | W | Q | I | V | L | I | D | D | L | L | P | C | D | H | - | - | - | - | - | - | - | - | - | - | - | - | -92 |
| R7TJF2_CAPTE | - | I | Y | Q | V | R | L | C | K | D | G | Q | - | - | - | - | - | - | - | - | - | - | - | - | - | - | - | - | - | - | - | - | - | - | - | W | Q | T | V | L | V | D | D | L | L | P | C | N | S | - | - | - | - | - | - | - | - | - | - | - | - | -92 |
| T2MI17_HYDVU | - | A | Y | Q | I | R | I | C | K | N | G | K | - | - | - | - | - | - | - | - | - | - | - | - | - | - | - | - | - | - | - | - | - | - | - | W | I | V | V | L | V | D | D | L | L | P | C | D | A | - | - | - | - | - | - | - | - | - | - | - | - | -89 |
| Q1RL55_CIOIN | - | V | Y | V | V | R | L | C | K | A | G | V | - | - | - | - | - | - | - | - | - | - | - | - | - | - | - | - | - | - | - | - | - | - | - | W | Q | T | V | F | V | D | D | L | L | P | C | D | E | - | - | - | - | - | - | - | - | - | - | - | - | -79 |
| I1EA30_AMPQE | - | V | Y | L | V | K | L | C | R | D | G | L | - | - | - | - | - | - | - | - | - | - | - | - | - | - | - | - | - | - | - | - | - | - | - | W | Q | T | I | L | V | D | D | Y | F | P | V | T | A | D | - | - | - | - | - | - | - | - | - | - | - | -90 |
| G5EEZ6_CAEEL | - | V | Y | Q | I | K | L | C | V | E | G | K | - | - | - | - | - | - | - | - | - | - | - | - | - | - | - | - | - | - | - | - | - | - | - | W | E | V | I | I | V | D | D | F | F | P | C | Y | S | - | - | - | - | - | - | - | - | - | - | - | - | -91 |
| O44903_CAEEL | - | V | Y | Q | V | K | V | C | V | E | G | K | - | - | - | - | - | - | - | - | - | - | - | - | - | - | - | - | - | - | - | - | - | - | - | W | E | V | I | I | V | D | D | F | F | P | C | Y | R | - | - | - | - | - | - | - | - | - | - | - | - | -91 |
| O02260_CAEEL | - | V | Y | K | V | K | L | F | V | D | G | Q | - | - | - | - | - | - | - | - | - | - | - | - | - | - | - | - | - | - | - | - | - | - | - | W | K | T | I | I | I | D | D | Y | F | P | Y | T | T | - | - | - | - | - | - | - | - | - | - | - | - | -90 |
| O02259_CAEEL | - | V | Y | K | V | K | L | F | V | D | G | Q | - | - | - | - | - | - | - | - | - | - | - | - | - | - | - | - | - | - | - | - | - | - | - | W | K | T | I | I | I | D | D | Y | F | P | Y | T | T | - | - | - | - | - | - | - | - | - | - | - | - | -67 |
| C1MTG3_MICPC | - | A | Y | L | V | R | L | F | L | G | G | R | - | - | - | - | - | - | - | - | - | - | - | - | - | - | - | - | - | - | - | - | - | - | - | W | R | G | V | V | V | D | P | C | L | P | V | A | G | R | S | V | E | V | S | R | G | A | N | G | P | -85 |
| C1E493_MICSR | - | A | F | L | M | R | L | F | L | A | G | R | - | - | - | - | - | - | - | - | - | - | - | - | - | - | - | - | - | - | - | - | - | - | - | W | R | G | V | A | V | D | P | C | L | P | V | S | G | R | S | T | K | V | L | A | G | A | N | - | - | -79 |
| Q012Z6_OSTTA | - | A | W | F | V | R | L | F | L | G | G | R | - | - | - | - | - | - | - | - | - | - | - | - | - | - | - | - | - | - | - | - | - | - | - | W | R | A | I | V | V | D | D | M | L | P | V | K | T | P | K | T | K | K | - | - | - | - | - | - | - | -82 |
| L1IGB6_GUITH | - | C | Y | M | I | R | L | F | L | D | G | S | - | - | - | - | - | - | - | - | - | - | - | - | - | - | - | - | - | - | - | - | - | - | - | W | Q | T | I | L | V | D | D | Q | L | P | C | T | R | S | P | R | R | P | - | - | - | - | - | - | - | -83 |
| B7FWX6_PHATC | - | C | Y | E | V | N | L | C | L | D | G | A | - | - | - | - | - | - | - | - | - | - | - | - | - | - | - | - | - | - | - | - | - | - | - | W | T | P | V | R | V | D | S | T | L | P | V | V | L | Q | D | V | N | K | T | T | G | G | S | L | L | -77 |
| N9TPR9_ENTHI | - | K | Y | E | I | T | L | F | Y | M | G | K | - | - | - | - | - | - | - | - | - | - | - | - | - | - | - | - | - | - | - | - | - | - | - | E | R | H | I | V | I | D | D | L | I | P | C | N | N | G | - | - | - | - | - | - | - | - | - | - | - | -83 |
| G0QS43_ICHMG | - | V | Y | C | V | K | I | C | H | K | G | Y | - | - | - | - | - | - | - | - | - | - | - | - | - | - | - | - | - | - | - | - | - | - | - | W | K | E | V | I | V | D | Q | F | I | P | C | L | N | S | T | - | - | - | - | - | - | - | - | - | - | -89 |
| D8LB20_ECTSI | - | K | Y | K | V | R | L | F | D | I | T | Q | D | - | - | - | - | - | - | - | - | - | - | - | - | - | - | - | - | - | - | - | - | - | R | W | R | T | I | T | V | D | D | N | I | P | T | R | H | G | - | - | - | - | - | - | - | - | - | - | - | -94 |
| Q9U0T9_LEIMA | - | A | Y | R | V | S | F | N | K | N | G | L | - | - | - | - | - | - | - | - | - | - | - | - | - | - | - | - | - | - | - | - | - | - | - | W | R | S | I | L | V | D | D | Y | F | P | V | I | A | G | - | - | - | - | - | - | - | - | - | - | - | -95 |
| Q4CXJ8_TRYCC | - | A | Y | R | V | T | L | N | T | S | G | L | - | - | - | - | - | - | - | - | - | - | - | - | - | - | - | - | - | - | - | - | - | - | - | W | R | S | V | I | V | D | D | Y | F | P | G | Y | A | G | - | - | - | - | - | - | - | - | - | - | - | -95 |
| F4I0A4_ARATH | - | I | Y | T | V | R | F | C | I | Q | G | E | - | - | - | - | - | - | - | - | - | - | - | - | - | - | - | - | - | - | - | - | - | - | - | W | V | P | V | V | I | D | D | W | I | P | C | E | S | P | G | K | - | - | - | - | - | - | - | - | - | -93 |
| Q8RUQ1_MAIZE | - | I | Y | T | V | R | F | C | I | Q | G | E | - | - | - | - | - | - | - | - | - | - | - | - | - | - | - | - | - | - | - | - | - | - | - | W | V | A | V | V | V | D | D | W | I | P | C | E | S | P | G | K | - | - | - | - | - | - | - | - | - | -93 |
| D7FQ17_ECTSI | - | A | Y | A | V | K | I | T | V | G | G | K | - | - | - | - | - | - | - | - | - | - | - | - | - | - | - | - | - | - | - | - | - | - | - | W | Q | V | V | I | V | D | D | Y | F | P | A | L | E | T | S | Q | A | D | D | D | - | - | - | - | - | -107 |
| D0MZ44_PHYIT | - | A | Y | A | L | R | L | Y | Q | N | C | Q | - | - | - | - | - | - | - | - | - | - | - | - | - | - | - | - | - | - | - | - | - | - | - | W | E | T | V | I | V | D | D | Y | F | P | V | L | Y | D | S | A | G | L | D | A | G | G | D | A | - | -104 |
| L1JBM0_GUITH | - | V | H | A | V | R | F | F | V | E | G | R | - | - | - | - | - | - | - | - | - | - | - | - | - | - | - | - | - | - | - | - | - | - | - | W | V | V | V | V | V | D | D | L | I | P | C | - | - | D | R | F | G | - | - | - | - | - | - | - | - | -63 |
| L1J739_GUITH | - | I | Y | T | I | R | F | Y | K | N | G | R | - | - | - | - | - | - | - | - | - | - | - | - | - | - | - | - | - | - | - | - | - | - | - | W | K | Y | V | S | I | D | D | R | I | P | C | - | - | S | P | S | G | - | - | - | - | - | - | - | - | -93 |
| L1J0U9_GUITH | - | I | Y | T | V | R | Y | Y | K | Y | G | Q | - | - | - | - | - | - | - | - | - | - | - | - | - | - | - | - | - | - | - | - | - | - | - | W | H | N | V | V | I | D | D | R | I | P | C | - | - | D | Q | V | N | - | - | - | - | - | - | - | - | -87 |
| L1J1Q4_GUITH | - | I | I | T | V | Q | L | Y | K | D | C | A | - | - | - | - | - | - | - | - | - | - | - | - | - | - | - | - | - | - | - | - | - | - | - | W | H | D | V | T | V | D | T | R | I | P | C | V | I | D | P | S | S | G | G | - | - | - | - | - | - | -104 |
| D0ND88_PHYIT | - | V | F | T | C | R | F | F | K | D | D | N | - | - | - | - | - | - | - | - | - | - | - | - | - | - | - | - | - | - | - | - | - | - | - | W | L | P | V | T | T | D | T | R | I | P | Y | S | M | E | L | Q | S | E | D | K | V | S | N | G | G | -101 |
| D2VVS6_NAEGR | - | K | Y | T | F | C | L | F | K | E | G | R | - | - | - | - | - | - | - | - | - | - | - | - | - | - | - | - | - | - | - | - | - | - | - | N | K | E | I | T | I | D | D | L | I | P | C | D | V | T | T | - | - | - | - | - | - | - | - | - | - | -90 |
| D2V3V2_NAEGR | - | K | Y | V | F | S | I | F | G | K | E | G | - | - | - | - | - | - | - | - | - | - | - | - | - | - | - | - | - | - | - | - | - | - | - | P | T | E | V | V | I | D | N | R | L | P | C | F | S | D | S | - | - | - | - | - | - | - | - | - | - | -91 |
| Q5B5J7_EMENI | - | V | Y | G | F | V | F | Y | R | D | G | E | - | - | - | - | - | - | - | - | - | - | - | - | - | - | - | - | - | - | - | - | - | - | - | W | Q | Q | C | I | V | D | D | K | L | Y | L | R | A | A | D | Y | D | E | S | I | D | E | R | P | V | -90 |
| E4ZY10_LEPMJ | - | V | Y | G | F | V | F | H | R | D | G | E | - | - | - | - | - | - | - | - | - | - | - | - | - | - | - | - | - | - | - | - | - | - | - | W | I | S | E | V | I | D | D | K | L | Y | L | I | K | E | D | F | D | E | A | K | I | E | R | Y | H | -95 |
| A8PIE5_COPC7 | - | V | Y | G | F | I | F | Y | G | D | N | G | - | - | - | - | - | - | - | - | - | - | - | - | - | - | - | - | - | - | - | - | - | - | - | W | D | S | V | I | I | D | D | M | L | F | T | N | I | P | - | K | Y | E | E | L | T | Q | - | - | - | -88 |
| A8NBA6_COPC7 | - | V | Y | G | F | V | F | H | R | D | G | L | - | - | - | - | - | - | - | - | - | - | - | - | - | - | - | - | - | - | - | - | - | - | - | W | K | P | V | I | I | D | D | L | L | F | T | N | I | P | - | K | Y | E | E | L | K | E | - | - | - | -90 |
| A8NBA1_COPC7 | - | V | Y | G | F | I | F | F | Q | D | D | G | - | - | - | - | - | - | - | - | - | - | - | - | - | - | - | - | - | - | - | - | - | - | - | W | A | K | V | I | V | D | D | F | V | F | T | T | Q | P | P | S | F | D | S | L | G | E | - | - | - | -94 |
| A8NBA2_COPC7 | - | V | Y | G | F | I | F | F | Q | D | D | G | - | - | - | - | - | - | - | - | - | - | - | - | - | - | - | - | - | - | - | - | - | - | - | W | C | R | V | I | V | D | D | L | L | P | I | Q | N | P | R | H | F | D | S | L | D | D | - | - | - | -94 |
| A8NAE6_COPC7 | - | V | Y | G | F | I | F | Y | R | D | C | Y | - | - | - | - | - | - | - | - | - | - | - | - | - | - | - | - | - | - | - | - | - | - | - | W | V | P | V | V | I | D | D | L | L | F | T | R | V | P | - | K | Y | E | Q | L | T | S | - | - | - | -94 |
| A8NAI9_COPC7 | - | V | Y | G | F | I | F | C | K | H | G | I | - | - | - | - | - | - | - | - | - | - | - | - | - | - | - | - | - | - | - | - | - | - | - | W | T | P | V | I | V | D | D | L | L | Y | S | Q | M | P | - | I | Y | E | N | L | R | T | - | - | - | -90 |
| G4TER7_PIRID | - | V | Y | G | F | L | F | F | R | D | H | T | - | - | - | - | - | - | - | - | - | - | - | - | - | - | - | - | - | - | - | - | - | - | - | W | S | P | V | I | I | D | D | Q | L | F | V | Q | V | P | - | K | Y | E | S | L | T | P | - | - | - | -90 |
| G4TER6_PIRID | - | V | Y | G | F | I | F | F | R | D | G | A | - | - | - | - | - | - | - | - | - | - | - | - | - | - | - | - | - | - | - | - | - | - | - | W | V | D | V | I | I | D | D | Q | L | F | V | S | F | P | - | E | Y | E | S | L | S | Q | - | - | - | -82 |
| G4TNA1_PIRID | - | V | Y | A | F | I | F | F | R | D | G | L | - | - | - | - | - | - | - | - | - | - | - | - | - | - | - | - | - | - | - | - | - | - | - | W | Q | E | V | I | I | D | D | Q | L | F | V | S | T | A | - | S | Y | D | E | L | S | F | - | - | - | -90 |
| D0MWB9_PHYIT | - | L | F | V | C | K | F | M | K | N | F | V | - | - | - | - | - | - | - | - | - | - | - | - | - | - | - | - | - | - | - | - | - | - | - | W | N | Y | V | L | I | D | D | R | L | P | - | - | - | - | - | - | - | - | - | - | - | - | - | - | - | -76 |
| L1I9H5_GUITH | - | M | F | G | V | K | F | F | L | N | G | K | - | - | - | - | - | - | - | - | - | - | - | - | - | - | - | - | - | - | - | - | - | - | - | W | V | T | V | L | I | D | D | R | F | P | C | V | R | R | G | S | - | - | - | - | - | - | - | - | - | -92 |
| Q7N497_PHOLL | - | I | Y | S | I | R | I | I | N | Q | S | T | - | - | - | - | - | - | - | - | - | - | - | - | - | - | - | - | - | - | - | - | - | - | - | V | G | Y | L | L | I | D | D | D | L | P | R | V | S | - | - | - | - | - | - | - | - | - | - | - | - | -78 |
| ZP_11152369 | - | I | Y | S | I | R | L | I | V | D | P | E | - | - | - | - | - | - | - | - | - | - | - | - | - | - | - | - | - | - | - | - | - | - | - | V | R | Y | L | L | I | D | D | Q | I | P | H | E | N | - | - | - | - | - | - | - | - | - | - | - | - | -74 |
| A2FEL7_TRIVA | - | L | C | C | V | V | F | K | A | M | N | K | - | - | - | - | - | - | - | - | - | - | - | - | - | - | - | - | - | - | - | - | - | - | - | E | M | S | V | N | V | D | T | L | I | P | L | L | N | - | - | - | - | - | - | - | - | - | - | - | - | -85 |
| A2D9A8_TRIVA | - | V | S | C | V | S | F | N | A | M | D | T | - | - | - | - | - | - | - | - | - | - | - | - | - | - | - | - | - | - | - | - | - | - | - | N | L | Y | V | L | V | D | T | M | I | P | V | S | K | E | - | - | - | - | - | - | - | - | - | - | - | -86 |
| A2FHB3_TRIVA | - | A | A | C | I | S | F | Y | S | M | G | K | - | - | - | - | - | - | - | - | - | - | - | - | - | - | - | - | - | - | - | - | - | - | - | R | G | H | V | I | V | D | T | Q | L | P | F | S | T | K | S | - | - | - | - | - | - | - | - | - | - | -86 |
| A2DTE8_TRIVA | - | I | F | A | V | H | F | N | Y | L | G | K | - | - | - | - | - | - | - | - | - | - | - | - | - | - | - | - | - | - | - | - | - | - | - | D | I | Y | S | V | V | D | S | M | I | P | I | E | G | - | - | - | - | - | - | - | - | - | - | - | - | -83 |
| A2E4X2_TRIVA | - | C | V | C | V | K | F | Q | I | F | E | K | - | - | - | - | - | - | - | - | - | - | - | - | - | - | - | - | - | - | - | - | - | - | - | P | A | Y | V | I | I | D | T | L | I | P | F | T | Q | S | K | D | D | Q | P | - | - | - | - | - | - | -89 |
| A2E1X1_TRIVA | - | C | C | C | L | K | F | S | F | I | G | K | - | - | - | - | - | - | - | - | - | - | - | - | - | - | - | - | - | - | - | - | - | - | - | P | Y | Y | V | I | I | D | T | T | V | P | F | V | D | N | - | - | - | - | - | - | - | - | - | - | - | -75 |
| TPR_PORGI | - | G | R | S | K | Q | T | Y | A | R | - | - | - | - | - | - | - | - | - | - | - | - | - | - | - | - | - | - | - | - | - | - | - | P | S | G | S | G | T | S | T | N | E | I | L | Q | E | G | - | - | - | - | - | - | - | - | - | - | - | - | - | -101 |
| D5MF42_9BACT | - | D | R | - | I | D | F | Y | S | N | - | - | - | - | - | - | - | - | - | - | - | - | - | - | - | - | - | - | - | - | - | - | - | G | T | K | S | M | E | V | T | E | R | V | P | L | V | K | - | - | - | - | - | - | - | - | - | - | - | - | - | -63 |
| R9H254_BACT4 | - | T | Y | T | V | T | M | Y | D | P | K | - | - | - | - | - | - | - | - | - | - | - | - | - | - | - | - | - | - | - | - | - | - | G | K | Q | I | S | V | G | V | G | G | D | Y | F | I | G | - | - | - | - | - | - | - | - | - | - | - | - | - | -64 |
| ZP_04847694 | - | T | Y | T | V | T | M | Y | D | P | K | - | - | - | - | - | - | - | - | - | - | - | - | - | - | - | - | - | - | - | - | - | - | G | K | Q | I | S | V | G | V | G | G | D | Y | F | I | G | - | - | - | - | - | - | - | - | - | - | - | - | - | -64 |
| D7IBA4_9BACE | - | T | Y | T | V | T | M | Y | D | P | K | - | - | - | - | - | - | - | - | - | - | - | - | - | - | - | - | - | - | - | - | - | - | G | K | Q | I | S | V | G | V | G | G | D | Y | F | I | G | - | - | - | - | - | - | - | - | - | - | - | - | - | -62 |
| ZP_09858577 | - | T | Y | T | V | T | M | Y | D | P | K | - | - | - | - | - | - | - | - | - | - | - | - | - | - | - | - | - | - | - | - | - | - | G | K | Q | I | S | V | G | V | G | G | D | Y | F | I | G | - | - | - | - | - | - | - | - | - | - | - | - | - | -62 |
| E5C759_9BACE | - | T | Y | T | V | T | M | Y | D | P | T | - | - | - | - | - | - | - | - | - | - | - | - | - | - | - | - | - | - | - | - | - | - | G | K | Q | I | P | V | S | V | G | G | D | Y | F | I | G | - | - | - | - | - | - | - | - | - | - | - | - | - | -64 |
| A7LTS0_BACO1 | - | T | F | T | V | K | L | Y | D | P | K | - | - | - | - | - | - | - | - | - | - | - | - | - | - | - | - | - | - | - | - | - | - | G | Q | Q | I | E | V | G | V | S | - | N | L | F | V | G | - | - | - | - | - | - | - | - | - | - | - | - | - | -62 |
| Q8YQK8_NOSS1 | - | T | Y | T | M | R | F | Y | Q | N | - | - | - | - | - | - | - | - | - | - | - | - | - | - | - | - | - | - | - | - | - | - | - | G | V | A | E | Y | V | T | V | D | R | R | I | A | T | S | - | - | - | - | - | - | - | - | - | - | - | - | - | -80 |
| Q3MBX8_ANAVT | - | T | Y | T | V | R | F | Y | S | T | T | - | - | - | - | - | - | - | - | - | - | - | - | - | - | - | - | - | - | I | F | D | P | G | E | A | Q | Y | V | T | I | D | R | R | I | A | T | S | - | - | - | - | - | - | - | - | - | - | - | - | - | -85 |
| Q8YZT8_NOSS1 | - | T | Y | T | V | R | F | Y | S | G | K | - | - | - | - | - | - | - | - | - | - | - | - | - | - | - | - | - | - | Y | Y | A | P | G | E | A | Q | Y | V | T | V | D | R | R | I | A | T | N | - | - | - | - | - | - | - | - | - | - | - | - | - | -73 |
| K9UIG2_9CHRO | - | T | Y | T | V | R | F | F | T | Q | D | - | - | - | - | - | - | - | - | - | - | - | - | - | - | - | - | - | - | - | - | - | - | L | K | A | E | W | V | T | V | D | N | R | L | A | T | T | D | G | K | - | - | - | - | - | - | - | - | - | - | -75 |
| A4YRP8_BRASO | - | T | Y | G | V | R | F | Y | V | N | - | - | - | - | - | - | - | - | - | - | - | - | - | - | - | - | - | - | - | - | - | - | - | G | T | A | R | Y | V | T | V | N | T | S | L | A | N | G | - | - | - | - | - | - | - | - | - | - | - | - | - | -66 |
| H0TAL7_9BRAD | - | T | Y | G | V | R | F | Y | V | N | - | - | - | - | - | - | - | - | - | - | - | - | - | - | - | - | - | - | - | - | - | - | - | G | T | A | R | Y | V | T | V | N | T | A | L | A | N | G | - | - | - | - | - | - | - | - | - | - | - | - | - | -70 |
| H0SNL4_9BRAD | - | T | Y | G | V | R | F | Y | V | N | - | - | - | - | - | - | - | - | - | - | - | - | - | - | - | - | - | - | - | - | - | - | - | G | T | A | R | Y | V | T | V | N | T | A | L | A | N | G | - | - | - | - | - | - | - | - | - | - | - | - | - | -72 |
| H0S4P6_9BRAD | - | T | Y | G | V | R | F | Y | V | N | - | - | - | - | - | - | - | - | - | - | - | - | - | - | - | - | - | - | - | - | - | - | - | G | T | A | R | Y | V | T | V | N | T | A | L | A | N | G | - | - | - | - | - | - | - | - | - | - | - | - | - | -72 |
| A5EMK2_BRASB | - | T | Y | G | V | R | F | Y | V | N | - | - | - | - | - | - | - | - | - | - | - | - | - | - | - | - | - | - | - | - | - | - | - | G | A | A | R | Y | V | T | V | N | S | A | L | A | N | G | - | - | - | - | - | - | - | - | - | - | - | - | - | -66 |
| M4ZBJ4_9BRAD | - | T | Y | G | V | R | F | Y | V | N | - | - | - | - | - | - | - | - | - | - | - | - | - | - | - | - | - | - | - | - | - | - | - | G | A | A | R | Y | V | T | V | N | T | A | L | A | N | G | - | - | - | - | - | - | - | - | - | - | - | - | - | -72 |
| Q07SP7_RHOP5 | - | T | Y | G | V | R | L | F | V | N | - | - | - | - | - | - | - | - | - | - | - | - | - | - | - | - | - | - | - | - | - | - | - | G | V | A | Q | Y | V | T | V | N | S | S | L | A | N | G | - | - | - | - | - | - | - | - | - | - | - | - | - | -67 |
| B0JUQ7_MICAN | - | T | F | T | V | R | F | F | R | N | - | - | - | - | - | - | - | - | - | - | - | - | - | - | - | - | - | - | - | - | - | - | - | G | V | A | N | Y | V | T | V | D | R | Y | L | P | T | T | - | - | - | - | - | - | - | - | - | - | - | - | - | -70 |
| K9WJ87_9CYAN | - | T | F | T | V | R | F | F | N | G | - | - | - | - | - | - | - | - | - | - | - | - | - | - | - | - | - | - | - | - | - | - | - | D | K | A | D | Y | V | T | V | D | K | Y | L | P | T | T | - | - | - | - | - | - | - | - | - | - | - | - | - | -66 |
| G6FUE0_9CYAN | - | T | F | T | V | R | F | Y | N | N | - | - | - | - | - | - | - | - | - | - | - | - | - | - | - | - | - | - | - | - | - | - | - | S | I | A | D | Y | V | T | V | D | R | Y | L | P | T | N | - | - | - | - | - | - | - | - | - | - | - | - | - | -66 |
| K9PHG5_9CYAN | - | T | Y | T | V | R | F | Y | N | R | - | - | - | - | - | - | - | - | - | - | - | - | - | - | - | - | - | - | - | - | - | - | - | G | V | A | D | Y | V | T | V | D | K | Y | L | P | V | D | - | - | - | - | - | - | - | - | - | - | - | - | - | -66 |
| Q8YXX3_NOSS1 | - | T | F | T | V | R | F | Y | N | N | - | - | - | - | - | - | - | - | - | - | - | - | - | - | - | - | - | - | - | - | - | - | - | K | V | A | D | F | V | T | V | D | K | F | L | A | T | L | N | N | L | N | S | N | Y | V | - | - | - | - | - | -78 |
| K9TN70_9CYAN | - | T | Y | T | V | R | F | F | N | N | - | - | - | - | - | - | - | - | - | - | - | - | - | - | - | - | - | - | - | - | - | - | - | G | T | A | D | Y | V | T | V | N | R | L | L | P | T | L | - | - | - | - | - | - | - | - | - | - | - | - | - | -66 |
| Q05QW3_9SYNE | - | T | Y | S | V | R | F | Y | T | N | - | - | - | - | - | - | - | - | - | - | - | - | - | - | - | - | - | - | - | - | - | - | - | G | Q | A | D | Y | V | T | V | D | N | M | M | A | T | D | - | - | - | - | - | - | - | - | - | - | - | - | - | -70 |
| Q05QW2_9SYNE | - | T | Y | S | V | R | F | Y | T | N | - | - | - | - | - | - | - | - | - | - | - | - | - | - | - | - | - | - | - | - | - | - | - | G | Q | T | D | Y | V | T | V | D | S | M | M | A | T | R | - | - | - | - | - | - | - | - | - | - | - | - | - | -70 |
| Q05QW4_9SYNE | - | T | Y | S | V | R | F | Y | T | N | - | - | - | - | - | - | - | - | - | - | - | - | - | - | - | - | - | - | - | - | - | - | - | G | K | V | D | Y | V | T | V | D | S | M | M | A | T | S | - | - | - | - | - | - | - | - | - | - | - | - | - | -70 |
| E0UGX6_CYAP2 | - | T | F | T | V | R | F | Y | N | N | - | - | - | - | - | - | - | - | - | - | - | - | - | - | - | - | - | - | - | - | - | - | - | G | V | A | D | F | V | T | V | D | N | Y | L | P | T | N | - | - | - | - | - | - | - | - | - | - | - | - | - | -70 |
| K7WKF4_9NOST | - | T | F | T | V | R | F | Y | N | N | - | - | - | - | - | - | - | - | - | - | - | - | - | - | - | - | - | - | - | - | - | - | - | G | V | A | D | Y | V | T | V | D | R | Y | L | P | T | Y | - | - | - | - | - | - | - | - | - | - | - | - | - | -66 |
| E0UGY5_CYAP2 | - | T | F | T | V | R | F | F | N | N | - | - | - | - | - | - | - | - | - | - | - | - | - | - | - | - | - | - | - | - | - | - | - | G | V | A | D | Y | V | T | V | D | R | Y | L | P | T | N | - | - | - | - | - | - | - | - | - | - | - | - | - | -70 |
| A8LCE5_FRASN | - | T | V | S | V | T | V | H | L | P | - | - | T | G | A | V | P | - | - | - | - | - | - | - | - | - | - | - | - | - | - | - | - | - | - | - | - | - | V | T | V | T | R | S | L | P | A | - | - | - | - | - | - | - | - | - | - | - | - | - | - | -74 |
| D3CT47_9ACTO | - | T | V | T | V | T | F | P | G | G | - | - | A | G | A | T | P | G | P | I | - | - | - | - | - | - | - | - | - | - | - | - | - | - | - | - | - | A | V | T | I | T | R | S | L | P | E | - | - | - | - | - | - | - | - | - | - | - | - | - | - | -78 |
| Q2J4J6_FRASC | - | T | V | T | I | I | M | H | P | R | G | P | A | G | A | G | V | G | - | - | - | - | - | - | - | - | - | - | - | - | - | - | - | - | - | - | - | - | V | T | V | T | A | S | L | P | A | R | R | V | Q | P | P | Y | G | - | - | - | - | - | - | -82 |
| Q0RAZ2_FRAAA | - | T | V | T | I | T | F | H | P | G | - | - | S | G | A | P | V | G | - | - | - | - | - | - | - | - | - | - | - | - | - | - | - | - | - | - | - | - | V | T | V | T | A | M | L | P | A | R | R | L | S | - | - | - | - | - | - | - | - | - | - | -76 |
| E3J3F6_FRASU | - | T | F | T | V | T | L | Y | R | D | - | - | G | R | A | F | P | - | - | - | - | - | - | - | - | - | - | - | - | - | - | - | - | - | - | - | - | - | V | T | V | T | R | D | L | P | G | L | A | S | A | D | - | - | - | - | - | - | - | - | - | -76 |
| ZP_09168240 | - | T | F | T | V | T | F | Y | R | D | - | - | G | R | P | F | P | - | - | - | - | - | - | - | - | - | - | - | - | - | - | - | - | - | - | - | - | - | V | T | V | T | R | A | L | P | G | L | P | T | G | D | - | - | - | - | - | - | - | - | - | -77 |
| C7MDV9_BRAFD | - | V | V | A | V | H | L | P | G | M | - | - | - | - | T | R | P | - | - | - | - | - | - | - | - | - | - | - | - | - | - | - | - | - | - | - | - | - | V | R | V | D | R | R | L | P | V | D | - | - | - | - | - | - | - | - | - | - | - | - | - | -56 |
| ZP_10094989 | - | I | V | T | V | R | L | P | G | L | - | - | - | - | E | E | P | - | - | - | - | - | - | - | - | - | - | - | - | - | - | - | - | - | - | - | - | - | L | R | V | D | R | R | F | P | V | D | - | - | - | - | - | - | - | - | - | - | - | - | - | -56 |
| A4X268_SALTO | - | T | V | D | V | L | L | H | E | S | T | L | L | G | D | S | I | G | P | T | - | - | - | - | - | - | - | - | - | - | - | - | - | G | R | R | L | R | I | T | V | H | P | D | V | P | I | W | - | - | - | - | - | - | - | - | - | - | - | - | - | -70 |
| WP_043162213 | - | T | V | D | V | L | L | H | E | S | T | L | L | G | D | S | I | G | P | T | - | - | - | - | - | - | - | - | - | - | - | - | - | G | R | R | L | R | I | T | V | H | P | D | V | P | I | W | - | - | - | - | - | - | - | - | - | - | - | - | - | -70 |
| Q0BRV8_GRABC | - | T | E | S | V | T | L | Y | M | S | A | S | G | - | - | T | I | P | H | Y | - | - | - | - | - | - | - | - | - | - | - | - | - | G | E | T | S | F | V | A | T | T | V | T | V | T | N | T | - | - | - | - | - | - | - | - | - | - | - | - | - | -80 |
| A9B2P0_HERA2 | - | T | Y | T | V | T | L | Y | P | E | - | - | - | - | - | - | E | G | V | S | G | F | F | G | - | - | - | - | - | - | - | - | M | R | S | K | V | E | V | T | V | T | N | E | F | V | H | S | - | - | - | - | - | - | - | - | - | - | - | - | - | -74 |
| A9B3U8_HERA2 | - | T | Y | T | V | T | F | H | P | E | A | E | - | - | - | - | S | G | I | K | K | I | F | G | - | - | - | - | - | - | - | - | M | R | S | T | V | E | V | T | V | T | N | E | F | P | H | T | - | - | - | - | - | - | - | - | - | - | - | - | - | -76 |
| A9B2A3_HERA2 | - | T | Y | T | V | T | F | Y | Q | R | E | A | D | T | R | F | N | R | L | N | N | W | F | D | N | - | - | - | - | - | - | - | G | F | D | P | V | K | I | T | V | T | A | E | F | P | V | L | - | - | - | - | - | - | - | - | - | - | - | - | - | -82 |
| D6ZC85_SEGRD | - | V | F | T | V | R | L | Y | D | Y | A | E | G | R | D | Y | I | P | G | T | H | G | D | G | N | P | W | P | A | G | - | T | P | V | T | P | T | T | I | A | V | T | Q | H | D | I | Q | E | - | - | - | - | - | - | - | - | - | - | - | - | - | -91 |
| E5XTT9_9ACTO | - | N | F | I | V | R | L | Y | N | Y | A | E | G | R | D | Y | I | P | G | T | H | G | D | G | N | P | W | P | A | G | - | V | A | A | V | P | V | S | V | T | V | T | Q | N | D | I | K | D | - | - | - | - | - | - | - | - | - | - | - | - | - | -91 |
| E5XTU0_9ACTO | - | N | F | T | V | Q | V | Y | D | Y | P | D | G | R | N | W | T | G | A | Q | H | G | D | D | N | P | W | P | T | T | N | Q | H | I | T | P | K | V | V | T | V | T | Q | D | D | I | R | Q | - | - | - | - | - | - | - | - | - | - | - | - | - | -92 |
| D6B0I6_9ACTO | - | T | V | S | V | R | V | W | D | K | E | - | - | - | - | - | - | - | - | - | - | - | - | - | - | - | - | - | - | - | - | - | - | G | N | H | H | W | V | T | M | T | A | D | L | P | T | D | - | - | - | - | - | - | - | - | - | - | - | - | - | -73 |
| D9X719_STRVR | - | T | V | S | V | R | V | W | D | K | A | - | - | - | - | - | - | - | - | - | - | - | - | - | - | - | - | - | - | - | - | - | - | G | N | Y | H | W | V | T | M | T | A | D | L | P | S | D | - | - | - | - | - | - | - | - | - | - | - | - | - | -73 |
| H6L010_SAPGL | - | T | Y | D | V | T | L | H | V | Y | K | Y | - | - | - | W | I | S | W | D | - | - | - | - | - | - | - | - | - | - | - | - | - | R | S | P | V | T | V | K | V | K | P | E | F | P | V | D | - | - | - | - | - | - | - | - | - | - | - | - | - | -69 |
| K1ZP83_9BACT | - | T | Y | T | V | G | F | H | K | I | T | H | P | D | G | C | I | K | Y | D | E | D | G | A | Y | - | - | - | - | T | L | G | I | R | T | T | K | K | I | T | V | D | T | E | L | P | K | Y | - | - | - | - | - | - | - | - | - | - | - | - | - | -89 |
| ADGB_HUMAN | - | K | Y | V | V | K | L | Y | W | M | G | C | - | - | - | - | - | - | - | - | - | - | - | - | - | - | - | - | - | - | - | - | - | - | - | W | R | K | I | T | I | D | D | F | L | P | F | D | E | D | N | - | - | - | - | - | - | - | - | - | - | -123 |
| J3QMP3_MOUSE | - | K | Y | V | V | K | L | Y | W | M | G | C | - | - | - | - | - | - | - | - | - | - | - | - | - | - | - | - | - | - | - | - | - | - | - | W | R | K | I | T | V | D | D | F | L | P | F | D | E | E | N | - | - | - | - | - | - | - | - | - | - | -123 |
| H2XR46_CIOIN | - | K | Y | V | V | R | L | F | W | M | G | H | - | - | - | - | - | - | - | - | - | - | - | - | - | - | - | - | - | - | - | - | - | - | - | W | R | K | I | V | V | D | D | Q | M | P | F | D | A | N | D | - | - | - | - | - | - | - | - | - | - | -120 |
| CAN7_MOUSE | - | K | Y | M | V | K | L | H | L | N | G | V | - | - | - | - | - | - | - | - | - | - | - | - | - | - | - | - | - | - | - | - | - | - | - | P | R | K | V | I | I | D | D | Q | L | P | V | D | H | K | G | - | - | - | - | - | - | - | - | - | - | -97 |
| CAN7_HUMAN | - | K | Y | M | V | K | L | H | L | N | G | V | - | - | - | - | - | - | - | - | - | - | - | - | - | - | - | - | - | - | - | - | - | - | - | P | R | K | V | I | I | D | D | Q | L | P | V | D | H | K | G | - | - | - | - | - | - | - | - | - | - | -97 |
| E1BY84_CHICK | - | K | Y | M | V | K | L | H | I | N | G | V | - | - | - | - | - | - | - | - | - | - | - | - | - | - | - | - | - | - | - | - | - | - | - | P | R | K | V | I | I | D | D | Q | L | P | V | D | H | S | G | - | - | - | - | - | - | - | - | - | - | -97 |
| H9GX91_DANRE | - | K | Y | M | V | K | L | H | I | N | G | V | - | - | - | - | - | - | - | - | - | - | - | - | - | - | - | - | - | - | - | - | - | - | - | P | R | K | V | I | I | D | D | F | L | P | V | D | R | N | G | - | - | - | - | - | - | - | - | - | - | -97 |
| F6SGI6_CIOIN | - | K | Y | M | V | K | L | H | L | N | G | V | - | - | - | - | - | - | - | - | - | - | - | - | - | - | - | - | - | - | - | - | - | - | - | F | R | K | V | V | I | D | D | R | L | P | V | D | R | Q | G | - | - | - | - | - | - | - | - | - | - | -97 |
| W4Y195_STRPU | - | K | Y | M | A | I | L | W | I | N | G | V | - | - | - | - | - | - | - | - | - | - | - | - | - | - | - | - | - | - | - | - | - | - | - | K | R | K | V | I | F | D | D | Y | L | P | V | D | K | H | G | - | - | - | - | - | - | - | - | - | - | -71 |
| R7V270_CAPTE | - | K | Y | M | V | K | L | N | L | N | G | V | - | - | - | - | - | - | - | - | - | - | - | - | - | - | - | - | - | - | - | - | - | - | - | P | R | K | V | V | I | D | D | L | L | P | I | G | H | H | G | - | - | - | - | - | - | - | - | - | - | -97 |
| G4LXK3_SCHMA | - | K | Y | M | V | R | L | N | F | N | G | V | - | - | - | - | - | - | - | - | - | - | - | - | - | - | - | - | - | - | - | - | - | - | - | P | R | K | V | I | I | D | D | F | L | P | M | G | K | D | G | - | - | - | - | - | - | - | - | - | - | -97 |
| T2M2K0_HYDVU | - | K | Y | M | V | K | L | H | F | N | G | V | - | - | - | - | - | - | - | - | - | - | - | - | - | - | - | - | - | - | - | - | - | - | - | A | R | K | V | I | I | D | D | L | L | P | M | G | K | D | G | - | - | - | - | - | - | - | - | - | - | -97 |
| B3RQK1_TRIAD | - | K | Y | L | V | R | L | N | I | N | G | I | - | - | - | - | - | - | - | - | - | - | - | - | - | - | - | - | - | - | - | - | - | - | - | Q | R | K | I | A | I | D | D | T | L | P | L | G | N | D | G | - | - | - | - | - | - | - | - | - | - | -97 |
| I1CS59_RHIO9 | - | K | Y | V | V | K | L | V | Y | N | G | I | - | - | - | - | - | - | - | - | - | - | - | - | - | - | - | - | - | - | - | - | - | - | - | T | R | K | V | V | V | D | D | L | L | P | V | S | R | E | G | - | - | - | - | - | - | - | - | - | - | -97 |
| I1GER3_AMPQE | - | K | Y | L | I | K | L | F | L | N | G | C | - | - | - | - | - | - | - | - | - | - | - | - | - | - | - | - | - | - | - | - | - | - | - | R | R | K | V | V | I | D | D | L | L | P | V | D | G | Y | S | - | - | - | - | - | - | - | - | - | - | -97 |
| Q22143_CAEEL | - | K | Y | M | F | K | F | H | L | N | G | A | - | - | - | - | - | - | - | - | - | - | - | - | - | - | - | - | - | - | - | - | - | - | - | W | R | K | V | L | I | D | D | Y | F | P | V | D | E | N | N | - | - | - | - | - | - | - | - | - | - | -97 |
| L1JZN7_GUITH | - | K | Y | M | I | K | M | Y | L | N | G | A | - | - | - | - | - | - | - | - | - | - | - | - | - | - | - | - | - | - | - | - | - | - | - | V | R | K | I | V | I | D | D | Y | L | P | V | V | Q | S | M | P | P | - | - | - | - | - | - | - | - | -98 |
| D0NT34_PHYIT | - | K | Y | V | V | K | L | W | A | N | G | V | - | - | - | - | - | - | - | - | - | - | - | - | - | - | - | - | - | - | - | - | - | - | - | P | R | K | V | V | I | D | D | L | L | P | V | S | A | T | S | G | - | - | - | - | - | - | - | - | - | -106 |
| A0A064B913_ASPOZ | - | K | Y | I | F | R | F | Y | F | N | G | C | - | - | - | - | - | - | - | - | - | - | - | - | - | - | - | - | - | - | - | - | - | - | - | F | R | K | V | I | I | D | D | R | L | P | S | S | K | T | S | R | - | - | - | - | - | - | - | - | - | -105 |
| A8N4X4_COPC7 | - | R | F | D | F | R | M | L | V | N | G | A | - | - | - | - | - | - | - | - | - | - | - | - | - | - | - | - | - | - | - | - | - | - | - | W | R | R | I | V | I | D | D | H | L | P | C | N | P | A | D | N | S | - | - | - | - | - | - | - | - | -118 |
| C0H5K1_PLAF7 | - | M | Y | I | C | R | L | H | C | N | G | I | - | - | - | - | - | - | - | - | - | - | - | - | - | - | - | - | - | - | - | - | - | - | - | Q | R | K | I | I | I | D | D | Y | V | P | V | K | N | N | N | - | - | - | - | - | - | - | - | - | - | -104 |
| K1REZ8_CRAGI | - | V | V | H | C | R | V | W | R | A | G | R | - | - | - | - | - | - | - | - | - | - | - | - | - | - | - | - | - | - | - | - | - | - | - | W | V | D | I | Y | V | D | D | S | V | P | S | V | A | N | S | G | - | - | - | - | - | - | - | - | - | -83 |
| F4NTM9_BATDJ | - | I | F | R | F | R | F | F | R | F | G | T | - | - | - | - | - | - | - | - | - | - | - | - | - | - | - | - | - | - | - | - | - | - | - | W | V | E | V | V | V | D | D | F | L | P | C | T | T | Q | G | - | - | - | - | - | - | - | - | - | - | -100 |
| Q6F6K5_CAEEL | - | I | A | Q | I | R | L | L | I | N | G | E | - | - | - | - | - | - | - | - | - | - | - | - | - | - | - | - | - | - | - | - | - | - | - | W | K | V | I | K | I | D | F | H | V | P | S | S | S | A | S | - | - | - | - | - | - | - | - | - | - | -66 |
| A8HZK8_CHLRE | - | A | Y | T | F | Q | F | F | K | H | G | C | - | - | - | - | - | - | - | - | - | - | - | - | - | - | - | - | - | - | - | - | - | - | - | W | Q | A | V | V | V | D | N | Y | L | P | C | L | A | D | E | E | - | - | - | - | - | - | - | - | - | -66 |
| A8J6T6_CHLRE | - | S | Y | Q | V | R | L | Y | D | P | K | S | - | - | - | - | - | - | - | - | - | - | - | - | - | - | - | - | - | - | - | - | - | G | G | A | T | I | S | P | I | D | D | W | I | P | C | F | N | G | - | - | - | - | - | - | - | - | - | - | - | -96 |
| Z9JR51_9MICO | - | - | W | E | V | E | L | F | I | D | G | E | - | - | - | - | - | - | - | - | - | - | - | - | - | - | - | - | - | - | - | - | - | - | - | W | Q | S | V | P | V | S | P | D | E | I | T | V | N | G | - | - | - | - | - | - | - | - | - | - | - | -82 |
| F7ZZH9_CELGA | - | - | Y | W | V | T | L | Y | A | N | G | E | - | - | - | - | - | - | - | - | - | - | - | - | - | - | - | - | - | - | - | - | - | - | - | P | E | E | V | F | V | E | Y | V | F | D | N | G | A | K | Q | S | - | - | - | - | - | - | - | - | - | -68 |
| Q7NDU2_GLOVI | - | S | F | S | V | E | L | G | S | G | E | Q | - | - | - | - | - | - | - | - | - | - | - | - | - | - | - | - | - | - | - | - | - | - | - | K | V | A | P | L | T | D | A | E | R | A | Y | F | G | R | A | E | - | - | - | - | - | - | - | - | - | -69 |
| I6AT95_9BACT | - | - | Y | L | V | R | L | G | A | R | D | V | - | - | - | - | - | - | - | - | - | - | - | - | - | - | - | - | - | - | - | - | - | - | - | A | V | P | A | L | T | D | A | E | L | A | L | T | A | T | T | G | - | - | - | - | - | - | - | - | - | -68 |
| C7MGJ8_BRAFD | - | T | V | T | V | T | L | Y | D | H | A | G | - | - | - | - | - | - | - | - | - | - | - | - | - | - | - | - | - | - | - | - | - | - | N | P | R | D | V | T | V | T | D | D | L | P | M | R | S | D | G | S | - | - | - | - | - | - | - | - | - | -66 |
| D0WP73_9ACTO | - | S | I | K | V | H | Y | G | P | D | G | K | P | - | - | - | - | - | - | - | - | - | - | - | - | - | - | - | - | - | - | - | - | D | G | F | M | V | T | V | Y | D | D | P | L | H | P | D | A | K | A | S | - | - | - | - | - | - | - | - | - | -66 |
| B0C8L1_ACAM1 | - | F | Y | Q | V | E | E | H | K | G | D | K | D | - | - | - | - | - | - | - | - | - | - | - | - | - | - | - | - | - | K | R | N | F | T | P | F | E | V | T | I | N | K | S | V | V | K | T | D | S | G | - | - | - | - | - | - | - | - | - | - | -76 |
| B3E5A8_GEOLS | - | R | F | R | V | R | F | A | N | G | D | Q | - | - | - | - | - | - | - | - | - | - | - | - | - | - | - | - | - | - | - | - | - | - | A | T | V | T | A | P | T | D | A | E | L | A | I | N | D | S | - | - | - | - | - | - | - | - | - | - | - | -67 |
| D4YQ94_9MICO | - | T | A | G | V | R | L | H | S | P | W | P | - | - | - | - | - | - | - | - | - | - | - | - | - | - | - | - | - | - | - | - | - | - | - | - | R | T | I | A | V | T | R | W | V | P | R | R | H | R | - | - | - | - | - | - | - | - | - | - | - | -70 |
| PAPA_CARPA | - | I | R | T | G | N | L | N | E | Y | S | E | - | - | - | - | - | - | - | - | - | - | - | - | - | - | - | - | - | - | - | - | - | - | - | Q | E | L | L | D | C | D | R | R | S | Y | G | C | N | G | G | - | - | - | - | - | - | - | - | - | - | -66 |
| RIM13_YEAST | - | K | Y | H | V | N | L | S | F | N | G | S | N | - | - | - | - | - | - | - | - | - | - | - | - | - | - | - | - | - | - | - | - | - | K | R | L | V | T | V | D | I | S | Q | I | P | T | S | V | D | G | - | - | - | - | - | - | - | - | - | - | -101 |

| I |  |  |  |  |  |  |  |  |  |  |  |  |  |  |  |  |  |  |  |  |  |  |  |  |  |  |  |  |  |  |  |  |  |  |  |  |  |  |  |  |  |  |  |  |  |  |  |  |  |  |  |  |  |  |  |  |  |  |  |  |  |  |
| --- | --- | --- | --- | --- | --- | --- | --- | --- | --- | --- | --- | --- | --- | --- | --- | --- | --- | --- | --- | --- | --- | --- | --- | --- | --- | --- | --- | --- | --- | --- | --- | --- | --- | --- | --- | --- | --- | --- | --- | --- | --- | --- | --- | --- | --- | --- | --- | --- | --- | --- | --- | --- | --- | --- | --- | --- | --- | --- | --- | --- | --- | --- |
| Q9N4B1_CAEEL | - | - | - | - | - | - | - | - | - | - | - | - | - | - | - | - | - | - | - | - | - | - | - | - | - | D | G | R | L | C | Y | M | R | S | Q | E | N | N | - | - | - | - | - | - | - | - | - | - | - | - | - | - | - | - | - | - | - | - | - | - | - | -100 |
| Q8IAA8_CAEEL | - | - | - | - | - | - | - | - | - | - | - | - | - | - | - | - | - | - | - | - | - | - | - | - | - | N | N | Q | L | Y | Y | L | H | S | A | D | N | T | - | - | - | - | - | - | - | - | - | - | - | - | - | - | - | - | - | - | - | - | - | - | - | -100 |
| CAN_CAEEL | - | - | - | - | - | - | - | - | - | - | - | - | - | - | - | - | - | - | - | - | - | - | - | - | - | N | G | E | L | L | Y | M | H | S | A | S | N | N | - | - | - | - | - | - | - | - | - | - | - | - | - | - | - | - | - | - | - | - | - | - | - | -100 |
| Q9U2B2_CAEEL | - | - | - | - | - | - | - | - | - | - | - | - | - | - | - | - | - | - | - | - | - | - | - | - | - | N | G | T | L | C | Y | M | S | S | P | G | G | S | - | - | - | - | - | - | - | - | - | - | - | - | - | - | - | - | - | - | - | - | - | - | - | -100 |
| Q9N4B2_CAEEL | - | - | - | - | - | - | - | - | - | - | - | - | - | - | - | - | - | - | - | - | - | - | - | - | - | N | G | K | L | I | Y | M | S | S | Q | D | G | D | - | - | - | - | - | - | - | - | - | - | - | - | - | - | - | - | - | - | - | - | - | - | - | -100 |
| C3Y6P6_BRAFL | - | - | - | - | - | - | - | - | - | - | - | - | - | - | - | - | - | - | - | - | - | - | - | - | - | R | P | A | F | F | S | L | H | S | D | Q | Q | N | - | - | - | - | - | - | - | - | - | - | - | - | - | - | - | - | - | - | - | - | - | - | - | -106 |
| C3Y6N1_BRAFL | - | - | - | - | - | - | - | - | - | - | - | - | - | - | - | - | - | - | - | - | - | - | - | - | - | D | P | Q | Y | F | S | L | H | S | N | D | K | N | - | - | - | - | - | - | - | - | - | - | - | - | - | - | - | - | - | - | - | - | - | - | - | -107 |
| CANA_DROME | - | - | - | - | - | - | - | - | - | - | - | - | - | - | - | - | - | - | - | - | - | - | - | - | - | - | - | E | L | M | Y | M | H | S | T | E | K | N | - | - | - | - | - | - | - | - | - | - | - | - | - | - | - | - | - | - | - | - | - | - | - | -100 |
| CANB_DROME | - | - | - | - | - | - | - | - | - | - | - | - | - | - | - | - | - | - | - | - | - | - | - | - | - | - | - | E | L | I | Y | M | H | S | T | E | K | N | - | - | - | - | - | - | - | - | - | - | - | - | - | - | - | - | - | - | - | - | - | - | - | -100 |
| X1Y3H71_ANODA | - | - | - | - | - | - | - | - | - | - | - | - | - | - | - | - | - | - | - | - | - | - | - | - | - | - | - | Q | L | I | Y | M | R | S | S | E | Q | N | - | - | - | - | - | - | - | - | - | - | - | - | - | - | - | - | - | - | - | - | - | - | - | -100 |
| Q5IW12_GECLA | - | - | - | - | - | - | - | - | - | - | - | - | - | - | - | - | - | - | - | - | - | - | - | - | - | - | - | R | L | V | F | M | H | S | E | E | K | N | - | - | - | - | - | - | - | - | - | - | - | - | - | - | - | - | - | - | - | - | - | - | - | -100 |
| K1Q056_CRAGI | - | - | - | - | - | - | - | - | - | - | - | - | - | - | - | - | - | - | - | - | - | - | - | - | - | - | - | Q | L | T | F | M | H | S | V | D | K | N | - | - | - | - | - | - | - | - | - | - | - | - | - | - | - | - | - | - | - | - | - | - | - | -100 |
| G4V6C0_SCHMA | - | - | - | - | - | - | - | - | - | - | - | - | - | - | - | - | - | - | - | - | - | - | - | - | - | - | - | H | L | V | F | M | H | S | A | D | S | N | - | - | - | - | - | - | - | - | - | - | - | - | - | - | - | - | - | - | - | - | - | - | - | -104 |
| R7ULX9_CAPTE | - | - | - | - | - | - | - | - | - | - | - | - | - | - | - | - | - | - | - | - | - | - | - | - | - | - | - | K | L | I | Y | M | H | S | D | N | T | N | - | - | - | - | - | - | - | - | - | - | - | - | - | - | - | - | - | - | - | - | - | - | - | -100 |
| W4Z5Q7_STRPU | - | - | - | - | - | - | - | - | - | - | - | - | - | - | - | - | - | - | - | - | - | - | - | - | - | - | - | E | L | V | F | V | H | S | K | E | K | N | - | - | - | - | - | - | - | - | - | - | - | - | - | - | - | - | - | - | - | - | - | - | - | -97 |
| R7VIB6_CAPTE | - | - | - | - | - | - | - | - | - | - | - | - | - | - | - | - | - | - | - | - | - | - | - | - | - | - | - | R | L | Q | Y | L | H | S | P | S | N | N | - | - | - | - | - | - | - | - | - | - | - | - | - | - | - | - | - | - | - | - | - | - | - | -103 |
| T2MJ45_HYDVU | - | - | - | - | - | - | - | - | - | - | - | - | - | - | - | - | - | - | - | - | - | - | - | - | - | - | - | K | L | V | F | L | H | S | A | Q | N | N | - | - | - | - | - | - | - | - | - | - | - | - | - | - | - | - | - | - | - | - | - | - | - | -100 |
| R7UAB6_CAPTE | - | - | - | - | - | - | - | - | - | - | - | - | - | - | - | - | - | - | - | - | - | - | - | - | - | - | - | K | L | V | F | M | H | S | Q | D | M | N | - | - | - | - | - | - | - | - | - | - | - | - | - | - | - | - | - | - | - | - | - | - | - | -100 |
| C3ZKN8_BRAFL | - | - | - | - | - | - | - | - | - | - | - | - | - | - | - | - | - | - | - | - | - | - | - | - | - | - | - | R | L | T | F | V | H | S | A | E | E | N | - | - | - | - | - | - | - | - | - | - | - | - | - | - | - | - | - | - | - | - | - | - | - | -101 |
| B3RJS5_TRIAD | - | - | - | - | - | - | - | - | - | - | - | - | - | - | - | - | - | - | - | - | - | - | - | - | - | - | - | R | L | I | F | V | H | S | A | D | T | N | - | - | - | - | - | - | - | - | - | - | - | - | - | - | - | - | - | - | - | - | - | - | - | -100 |
| H2YVF0_CIOSA | - | - | - | - | - | - | - | - | - | - | - | - | - | - | - | - | - | - | - | - | - | - | - | - | - | - | - | K | L | V | Y | L | H | S | A | Q | N | N | - | - | - | - | - | - | - | - | - | - | - | - | - | - | - | - | - | - | - | - | - | - | - | -100 |
| G4VAG2_SCHMA | - | - | - | - | - | - | - | - | - | - | - | - | - | - | - | - | - | - | - | - | - | - | - | - | - | - | - | T | L | V | F | M | H | S | N | D | P | T | - | - | - | - | - | - | - | - | - | - | - | - | - | - | - | - | - | - | - | - | - | - | - | -103 |
| Smp_157500 | - | - | - | - | - | - | - | - | - | - | - | - | - | - | - | - | - | - | - | - | - | - | - | - | - | - | - | N | P | V | F | I | H | S | T | D | N | S | - | - | - | - | - | - | - | - | - | - | - | - | - | - | - | - | - | - | - | - | - | - | - | -103 |
| Q7SYA9_DANRE | - | - | - | - | - | - | - | - | - | - | - | - | - | - | - | - | - | - | - | - | - | - | - | - | - | - | - | E | L | M | F | V | H | S | A | E | G | N | - | - | - | - | - | - | - | - | - | - | - | - | - | - | - | - | - | - | - | - | - | - | - | -100 |
| A5PMP1_DANRE | - | - | - | - | - | - | - | - | - | - | - | - | - | - | - | - | - | - | - | - | - | - | - | - | - | - | - | E | L | M | F | V | H | S | A | E | G | N | - | - | - | - | - | - | - | - | - | - | - | - | - | - | - | - | - | - | - | - | - | - | - | -100 |
| CANX_CHICK | - | - | - | - | - | - | - | - | - | - | - | - | - | - | - | - | - | - | - | - | - | - | - | - | - | - | - | E | L | L | F | V | H | S | A | E | C | T | - | - | - | - | - | - | - | - | - | - | - | - | - | - | - | - | - | - | - | - | - | - | - | -100 |
| CAN1_HUMAN | - | - | - | - | - | - | - | - | - | - | - | - | - | - | - | - | - | - | - | - | - | - | - | - | - | - | - | K | L | V | F | V | H | S | A | E | G | N | - | - | - | - | - | - | - | - | - | - | - | - | - | - | - | - | - | - | - | - | - | - | - | -100 |
| CAN1_MOUSE | - | - | - | - | - | - | - | - | - | - | - | - | - | - | - | - | - | - | - | - | - | - | - | - | - | - | - | K | L | V | F | V | H | S | A | Q | G | N | - | - | - | - | - | - | - | - | - | - | - | - | - | - | - | - | - | - | - | - | - | - | - | -100 |
| O42133_CHICK | - | - | - | - | - | - | - | - | - | - | - | - | - | - | - | - | - | - | - | - | - | - | - | - | - | - | - | K | L | L | F | V | H | S | A | E | G | T | - | - | - | - | - | - | - | - | - | - | - | - | - | - | - | - | - | - | - | - | - | - | - | -100 |
| Q7ZUR1_DANRE | - | - | - | - | - | - | - | - | - | - | - | - | - | - | - | - | - | - | - | - | - | - | - | - | - | - | - | K | L | L | F | V | H | S | A | E | G | G | - | - | - | - | - | - | - | - | - | - | - | - | - | - | - | - | - | - | - | - | - | - | - | -100 |
| E9QFN0_DANRE | - | - | - | - | - | - | - | - | - | - | - | - | - | - | - | - | - | - | - | - | - | - | - | - | - | - | - | K | L | L | F | V | H | S | A | E | G | S | - | - | - | - | - | - | - | - | - | - | - | - | - | - | - | - | - | - | - | - | - | - | - | -100 |
| CAN2_HUMAN | - | - | - | - | - | - | - | - | - | - | - | - | - | - | - | - | - | - | - | - | - | - | - | - | - | - | - | E | L | L | F | V | H | S | A | E | G | S | - | - | - | - | - | - | - | - | - | - | - | - | - | - | - | - | - | - | - | - | - | - | - | -100 |
| CAN2_MOUSE | - | - | - | - | - | - | - | - | - | - | - | - | - | - | - | - | - | - | - | - | - | - | - | - | - | - | - | E | L | L | F | V | H | S | A | E | G | S | - | - | - | - | - | - | - | - | - | - | - | - | - | - | - | - | - | - | - | - | - | - | - | -100 |
| CAN2_CHICK | - | - | - | - | - | - | - | - | - | - | - | - | - | - | - | - | - | - | - | - | - | - | - | - | - | - | - | E | L | L | F | V | H | S | A | E | G | S | - | - | - | - | - | - | - | - | - | - | - | - | - | - | - | - | - | - | - | - | - | - | - | -100 |
| Q6DFZ8_DANRE | - | - | - | - | - | - | - | - | - | - | - | - | - | - | - | - | - | - | - | - | - | - | - | - | - | - | - | E | L | L | F | V | H | S | A | E | G | S | - | - | - | - | - | - | - | - | - | - | - | - | - | - | - | - | - | - | - | - | - | - | - | -100 |
| Q504F5_DANRE | - | - | - | - | - | - | - | - | - | - | - | - | - | - | - | - | - | - | - | - | - | - | - | - | - | - | - | E | L | L | F | V | Y | S | A | E | K | N | - | - | - | - | - | - | - | - | - | - | - | - | - | - | - | - | - | - | - | - | - | - | - | -100 |
| Q5BLH5_DANRE | - | - | - | - | - | - | - | - | - | - | - | - | - | - | - | - | - | - | - | - | - | - | - | - | - | - | - | E | L | L | F | V | H | S | V | T | G | S | - | - | - | - | - | - | - | - | - | - | - | - | - | - | - | - | - | - | - | - | - | - | - | -100 |
| Q4VBH3_DANRE | - | - | - | - | - | - | - | - | - | - | - | - | - | - | - | - | - | - | - | - | - | - | - | - | - | - | - | K | L | L | F | V | H | S | A | E | G | S | - | - | - | - | - | - | - | - | - | - | - | - | - | - | - | - | - | - | - | - | - | - | - | -100 |
| CAN8_RAT | - | - | - | - | - | - | - | - | - | - | - | - | - | - | - | - | - | - | - | - | - | - | - | - | - | - | - | Q | L | L | F | L | H | S | E | E | G | N | - | - | - | - | - | - | - | - | - | - | - | - | - | - | - | - | - | - | - | - | - | - | - | -100 |
| CAN8_MOUSE | - | - | - | - | - | - | - | - | - | - | - | - | - | - | - | - | - | - | - | - | - | - | - | - | - | - | - | Q | L | L | F | L | H | S | E | E | G | N | - | - | - | - | - | - | - | - | - | - | - | - | - | - | - | - | - | - | - | - | - | - | - | -100 |
| CAN8_HUMAN | - | - | - | - | - | - | - | - | - | - | - | - | - | - | - | - | - | - | - | - | - | - | - | - | - | - | - | Q | L | L | F | L | H | S | E | Q | G | N | - | - | - | - | - | - | - | - | - | - | - | - | - | - | - | - | - | - | - | - | - | - | - | -100 |
| XP_426117 | - | - | - | - | - | - | - | - | - | - | - | - | - | - | - | - | - | - | - | - | - | - | - | - | - | - | - | E | L | L | F | V | H | S | E | E | G | N | - | - | - | - | - | - | - | - | - | - | - | - | - | - | - | - | - | - | - | - | - | - | - | -100 |
| CAN9_MOUSE | - | - | - | - | - | - | - | - | - | - | - | - | - | - | - | - | - | - | - | - | - | - | - | - | - | - | - | R | L | V | F | L | H | S | A | D | H | N | - | - | - | - | - | - | - | - | - | - | - | - | - | - | - | - | - | - | - | - | - | - | - | -102 |
| CAN9_HUMAN | - | - | - | - | - | - | - | - | - | - | - | - | - | - | - | - | - | - | - | - | - | - | - | - | - | - | - | R | L | V | F | L | H | S | A | D | H | N | - | - | - | - | - | - | - | - | - | - | - | - | - | - | - | - | - | - | - | - | - | - | - | -100 |
| E1C6M3_CHICK | - | - | - | - | - | - | - | - | - | - | - | - | - | - | - | - | - | - | - | - | - | - | - | - | - | - | - | R | L | V | F | V | H | S | A | E | H | N | - | - | - | - | - | - | - | - | - | - | - | - | - | - | - | - | - | - | - | - | - | - | - | -100 |
| Q6DG44_DANRE | - | - | - | - | - | - | - | - | - | - | - | - | - | - | - | - | - | - | - | - | - | - | - | - | - | - | - | K | L | V | F | L | H | S | A | D | N | N | - | - | - | - | - | - | - | - | - | - | - | - | - | - | - | - | - | - | - | - | - | - | - | -100 |
| CAN3_HUMAN | - | - | - | - | - | - | - | - | - | - | - | - | - | - | - | - | - | - | - | - | - | - | - | - | - | - | - | Q | L | V | F | T | K | S | N | H | R | N | - | - | - | - | - | - | - | - | - | - | - | - | - | - | - | - | - | - | - | - | - | - | - | -100 |
| CAN3_MOUSE | - | - | - | - | - | - | - | - | - | - | - | - | - | - | - | - | - | - | - | - | - | - | - | - | - | - | - | Q | L | V | F | T | K | S | N | H | R | N | - | - | - | - | - | - | - | - | - | - | - | - | - | - | - | - | - | - | - | - | - | - | - | -100 |
| CAN3_CHICK | - | - | - | - | - | - | - | - | - | - | - | - | - | - | - | - | - | - | - | - | - | - | - | - | - | - | - | Q | L | V | F | T | K | S | S | Q | R | N | - | - | - | - | - | - | - | - | - | - | - | - | - | - | - | - | - | - | - | - | - | - | - | -100 |
| Q66HU4_DANRE | - | - | - | - | - | - | - | - | - | - | - | - | - | - | - | - | - | - | - | - | - | - | - | - | - | - | - | Q | L | V | F | T | K | S | A | E | R | N | - | - | - | - | - | - | - | - | - | - | - | - | - | - | - | - | - | - | - | - | - | - | - | -100 |
| F1R632_DANRE | - | - | - | - | - | - | - | - | - | - | - | - | - | - | - | - | - | - | - | - | - | - | - | - | - | - | - | K | L | V | F | T | R | S | G | Q | N | N | - | - | - | - | - | - | - | - | - | - | - | - | - | - | - | - | - | - | - | - | - | - | - | -100 |
| CAN11_HUMAN | - | - | - | - | - | - | - | - | - | - | - | - | - | - | - | - | - | - | - | - | - | - | - | - | - | - | - | K | L | V | F | V | H | S | T | E | R | S | - | - | - | - | - | - | - | - | - | - | - | - | - | - | - | - | - | - | - | - | - | - | - | -100 |
| CAN11_MOUSE | - | - | - | - | - | - | - | - | - | - | - | - | - | - | - | - | - | - | - | - | - | - | - | - | - | - | - | K | L | V | F | V | H | A | S | H | R | Q | - | - | - | - | - | - | - | - | - | - | - | - | - | - | - | - | - | - | - | - | - | - | - | -100 |
| CAN12_MOUSE | - | - | - | - | - | - | - | - | - | - | - | - | - | - | - | - | - | - | - | - | - | - | - | - | - | - | - | K | L | M | F | V | R | S | E | Q | R | N | - | - | - | - | - | - | - | - | - | - | - | - | - | - | - | - | - | - | - | - | - | - | - | -100 |
| CAN12_HUMAN | - | - | - | - | - | - | - | - | - | - | - | - | - | - | - | - | - | - | - | - | - | - | - | - | - | - | - | K | L | M | F | V | R | S | E | Q | R | N | - | - | - | - | - | - | - | - | - | - | - | - | - | - | - | - | - | - | - | - | - | - | - | -100 |
| ENSSARP00000008135 | - | - | - | - | - | - | - | - | - | - | - | - | - | - | - | - | - | - | - | - | - | - | - | - | - | - | - | K | L | M | F | V | R | S | E | Q | R | N | - | - | - | - | - | - | - | - | - | - | - | - | - | - | - | - | - | - | - | - | - | - | - | -100 |
| ENSSARP00000010553 | - | - | - | - | - | - | - | - | - | - | - | - | - | - | - | - | - | - | - | - | - | - | - | - | - | - | - | X | X | X | X | X | X | X | X | X | X | X | - | - | - | - | - | - | - | - | - | - | - | - | - | - | - | - | - | - | - | - | - | - | - | -99 |
| A7E2G3_DANRE | - | - | - | - | - | - | - | - | - | - | - | - | - | - | - | - | - | - | - | - | - | - | - | - | - | - | - | R | L | L | F | S | Y | S | R | T | S | N | - | - | - | - | - | - | - | - | - | - | - | - | - | - | - | - | - | - | - | - | - | - | - | -100 |
| Q6NW69_DANRE | - | - | - | - | - | - | - | - | - | - | - | - | - | - | - | - | - | - | - | - | - | - | - | - | - | - | - | Q | L | I | Y | L | H | S | A | S | R | D | - | - | - | - | - | - | - | - | - | - | - | - | - | - | - | - | - | - | - | - | - | - | - | -100 |
| CAN13_HUMAN | - | - | - | - | - | - | - | - | - | - | - | - | - | - | - | - | - | - | - | - | - | - | - | - | - | - | - | K | C | L | F | V | R | P | R | H | Q | N | Q | - | - | - | - | - | - | - | - | - | - | - | - | - | - | - | - | - | - | - | - | - | - | -101 |
| CAN13_MOUSE | - | - | - | - | - | - | - | - | - | - | - | - | - | - | - | - | - | - | - | - | - | - | - | - | - | - | - | N | F | L | F | V | H | P | R | G | G | N | K | - | - | - | - | - | - | - | - | - | - | - | - | - | - | - | - | - | - | - | - | - | - | -103 |
| E1BRR5_CHICK | - | - | - | - | - | - | - | - | - | - | - | - | - | - | - | - | - | - | - | - | - | - | - | - | - | - | - | N | Y | L | S | V | H | A | R | T | S | N | - | - | - | - | - | - | - | - | - | - | - | - | - | - | - | - | - | - | - | - | - | - | - | -100 |
| E7F0H3_DANRE | - | - | - | - | - | - | - | - | - | - | - | - | - | - | - | - | - | - | - | - | - | - | - | - | - | - | - | E | L | L | F | V | C | S | K | T | S | N | - | - | - | - | - | - | - | - | - | - | - | - | - | - | - | - | - | - | - | - | - | - | - | -101 |
| Q1RLS9_DANRE | - | - | - | - | - | - | - | - | - | - | - | - | - | - | - | - | - | - | - | - | - | - | - | - | - | - | - | Q | L | V | F | V | Q | S | K | T | P | N | - | - | - | - | - | - | - | - | - | - | - | - | - | - | - | - | - | - | - | - | - | - | - | -100 |
| F1Q9Z1_DANRE | - | - | - | - | - | - | - | - | - | - | - | - | - | - | - | - | - | - | - | - | - | - | - | - | - | - | - | Q | L | I | F | V | K | S | K | T | Y | N | - | - | - | - | - | - | - | - | - | - | - | - | - | - | - | - | - | - | - | - | - | - | - | -101 |
| A4QNV6_DANRE | - | - | - | - | - | - | - | - | - | - | - | - | - | - | - | - | - | - | - | - | - | - | - | - | - | - | - | D | L | I | F | V | H | S | K | T | S | N | - | - | - | - | - | - | - | - | - | - | - | - | - | - | - | - | - | - | - | - | - | - | - | -101 |
| D6W573_HUMAN | - | - | - | - | - | - | - | - | - | - | - | - | - | - | - | - | - | - | - | - | - | - | - | - | - | - | - | Q | L | V | F | V | S | S | T | Y | K | N | - | - | - | - | - | - | - | - | - | - | - | - | - | - | - | - | - | - | - | - | - | - | - | -101 |
| R7UBC6_CAPTE | - | - | - | - | - | - | - | - | - | - | - | - | - | - | - | - | - | - | - | - | - | - | - | - | - | - | - | E | L | V | F | V | Q | S | S | - | S | P | V | - | - | - | - | - | - | - | - | - | - | - | - | - | - | - | - | - | - | - | - | - | - | -105 |
| K1RCZ2_CRAGI | - | - | - | - | - | - | - | - | - | - | - | - | - | - | - | - | - | - | - | - | - | - | - | - | - | - | - | Q | L | M | F | V | H | S | W | - | H | K | N | - | - | - | - | - | - | - | - | - | - | - | - | - | - | - | - | - | - | - | - | - | - | -107 |
| Q5IW13_GECLA | - | - | - | - | - | - | - | - | - | - | - | - | - | - | - | - | - | - | - | - | - | - | - | - | - | - | - | R | L | V | F | M | H | S | R | - | N | S | S | - | - | - | - | - | - | - | - | - | - | - | - | - | - | - | - | - | - | - | - | - | - | -100 |
| R7UUX7_CAPTE | - | - | - | - | - | - | - | - | - | - | - | - | - | - | - | - | - | - | - | - | - | - | - | - | - | - | - | R | L | L | Y | A | H | N | R | D | Q | P | N | - | - | - | - | - | - | - | - | - | - | - | - | - | - | - | - | - | - | - | - | - | - | -103 |
| K1Q505_CRAGI | - | - | - | - | - | - | - | - | - | - | - | - | - | - | - | - | - | - | - | - | - | - | - | - | - | - | - | R | L | V | Y | A | S | N | R | E | E | P | N | - | - | - | - | - | - | - | - | - | - | - | - | - | - | - | - | - | - | - | - | - | - | -101 |
| R7UD02_CAPTE | - | - | - | - | - | - | - | - | - | - | - | - | - | - | - | - | - | - | - | - | - | - | - | - | - | - | - | R | L | V | F | C | R | N | R | E | S | L | N | - | - | - | - | - | - | - | - | - | - | - | - | - | - | - | - | - | - | - | - | - | - | -101 |
| CAN6_HUMAN | - | - | - | - | - | - | - | - | - | - | - | - | - | - | - | - | - | - | - | - | - | - | - | - | - | - | G | D | L | V | F | S | F | S | T | S | M | N | - | - | - | - | - | - | - | - | - | - | - | - | - | - | - | - | - | - | - | - | - | - | - | -106 |
| CAN6_MOUSE | - | - | - | - | - | - | - | - | - | - | - | - | - | - | - | - | - | - | - | - | - | - | - | - | - | - | G | D | L | V | F | S | F | S | T | S | M | N | - | - | - | - | - | - | - | - | - | - | - | - | - | - | - | - | - | - | - | - | - | - | - | -106 |
| E1C312_CHICK | - | - | - | - | - | - | - | - | - | - | - | - | - | - | - | - | - | - | - | - | - | - | - | - | - | - | G | T | L | I | Y | C | H | S | N | V | E | N | - | - | - | - | - | - | - | - | - | - | - | - | - | - | - | - | - | - | - | - | - | - | - | -106 |
| E7FDN3_DANRE | - | - | - | - | - | - | - | - | - | - | - | - | - | - | - | - | - | - | - | - | - | - | - | - | - | - | G | T | L | L | F | C | R | S | A | T | P | R | - | - | - | - | - | - | - | - | - | - | - | - | - | - | - | - | - | - | - | - | - | - | - | -107 |
| CAN5_HUMAN | - | - | - | - | - | - | - | - | - | - | - | - | - | - | - | - | - | - | - | - | - | - | - | - | - | - | N | Q | L | I | Y | C | H | S | N | S | R | N | - | - | - | - | - | - | - | - | - | - | - | - | - | - | - | - | - | - | - | - | - | - | - | -102 |
| CAN5_MOUSE | - | - | - | - | - | - | - | - | - | - | - | - | - | - | - | - | - | - | - | - | - | - | - | - | - | - | N | Q | L | I | Y | C | H | S | N | S | K | N | - | - | - | - | - | - | - | - | - | - | - | - | - | - | - | - | - | - | - | - | - | - | - | -106 |
| E1C292_CHICK | - | - | - | - | - | - | - | - | - | - | - | - | - | - | - | - | - | - | - | - | - | - | - | - | - | - | N | Q | L | I | Y | C | H | S | N | S | K | N | - | - | - | - | - | - | - | - | - | - | - | - | - | - | - | - | - | - | - | - | - | - | - | -106 |
| A0JMG3_DANRE | - | - | - | - | - | - | - | - | - | - | - | - | - | - | - | - | - | - | - | - | - | - | - | - | - | - | G | K | L | I | Y | C | H | S | N | D | S | N | - | - | - | - | - | - | - | - | - | - | - | - | - | - | - | - | - | - | - | - | - | - | - | -106 |
| E7F7F1_DANRE | - | - | - | - | - | - | - | - | - | - | - | - | - | - | - | - | - | - | - | - | - | - | - | - | - | - | N | Q | L | V | Y | C | H | S | N | D | S | N | - | - | - | - | - | - | - | - | - | - | - | - | - | - | - | - | - | - | - | - | - | - | - | -106 |
| XP_006824412 | - | - | - | - | - | - | - | - | - | - | - | - | - | - | - | - | - | - | - | - | - | - | - | - | - | - | N | Q | L | I | F | V | H | S | Q | T | K | N | - | - | - | - | - | - | - | - | - | - | - | - | - | - | - | - | - | - | - | - | - | - | - | -100 |
| W4YJN2_STRPU | - | - | - | - | - | - | - | - | - | - | - | - | - | - | - | - | - | - | - | - | - | - | - | - | - | - | N | K | L | I | Y | T | H | S | E | E | G | N | - | - | - | - | - | - | - | - | - | - | - | - | - | - | - | - | - | - | - | - | - | - | - | -106 |
| C3YJZ3_BRAFL | - | - | - | - | - | - | - | - | - | - | - | - | - | - | - | - | - | - | - | - | - | - | - | - | - | - | G | Q | L | V | Y | N | R | S | K | D | Q | N | - | - | - | - | - | - | - | - | - | - | - | - | - | - | - | - | - | - | - | - | - | - | - | -106 |
| T2MIT4_HYDVU | - | - | - | - | - | - | - | - | - | - | - | - | - | - | - | - | - | - | - | - | - | - | - | - | - | - | G | E | L | I | Y | V | K | S | K | S | K | N | - | - | - | - | - | - | - | - | - | - | - | - | - | - | - | - | - | - | - | - | - | - | - | -106 |
| XP_002154722 | - | - | - | - | - | - | - | - | - | - | - | - | - | - | - | - | - | - | - | - | - | - | - | - | - | - | G | E | L | - | - | - | - | - | - | - | - | - | - | - | - | - | - | - | - | - | - | - | - | - | - | - | - | - | - | - | - | - | - | - | - | -83 |
| R7VLA7_CAPTE | - | - | - | - | - | - | - | - | - | - | - | - | - | - | - | - | - | - | - | - | - | - | - | - | - | - | G | D | L | V | Y | A | H | S | K | D | R | N | - | - | - | - | - | - | - | - | - | - | - | - | - | - | - | - | - | - | - | - | - | - | - | -106 |
| K1R1C6_CRAGI | - | - | - | - | - | - | - | - | - | - | - | - | - | - | - | - | - | - | - | - | - | - | - | - | - | - | N | Q | L | I | Y | I | H | S | Q | T | K | N | - | - | - | - | - | - | - | - | - | - | - | - | - | - | - | - | - | - | - | - | - | - | - | -106 |
| B3RIE5_TRIAD | - | - | - | - | - | - | - | - | - | - | - | - | - | - | - | - | - | - | - | - | - | - | - | - | - | - | G | K | L | I | Y | V | H | S | K | Q | K | N | - | - | - | - | - | - | - | - | - | - | - | - | - | - | - | - | - | - | - | - | - | - | - | -105 |
| K1PW75_CRAGI | - | - | - | - | - | - | - | - | - | - | - | - | - | - | - | - | - | - | - | - | - | - | - | - | - | - | G | E | L | V | Y | V | Q | S | R | S | K | N | - | - | - | - | - | - | - | - | - | - | - | - | - | - | - | - | - | - | - | - | - | - | - | -104 |
| K1RSB8_CRAGI | - | - | - | - | - | - | - | - | - | - | - | - | - | - | - | - | - | - | - | - | - | - | - | - | - | - | G | R | L | I | F | M | H | S | K | S | R | N | - | - | - | - | - | - | - | - | - | - | - | - | - | - | - | - | - | - | - | - | - | - | - | -104 |
| K1RWI3_CRAGI | - | - | - | - | - | - | - | - | - | - | - | - | - | - | - | - | - | - | - | - | - | - | - | - | - | - | G | N | L | I | Y | I | H | S | K | T | R | N | - | - | - | - | - | - | - | - | - | - | - | - | - | - | - | - | - | - | - | - | - | - | - | -106 |
| R7VGC2_CAPTE | - | - | - | - | - | - | - | - | - | - | - | - | - | - | - | - | - | - | - | - | - | - | - | - | - | - | N | K | L | V | F | C | H | S | N | S | K | N | - | - | - | - | - | - | - | - | - | - | - | - | - | - | - | - | - | - | - | - | - | - | - | -105 |
| K1QU26_CRAGI | - | - | - | - | - | - | - | - | - | - | - | - | - | - | - | - | - | - | - | - | - | - | - | - | - | - | G | K | L | I | F | C | H | S | K | S | R | N | - | - | - | - | - | - | - | - | - | - | - | - | - | - | - | - | - | - | - | - | - | - | - | -107 |
| CAN5_CAEEL | - | - | - | - | - | - | - | - | - | - | - | - | - | - | - | - | - | - | - | - | - | - | - | - | - | - | G | K | L | L | F | A | R | S | K | T | P | N | - | - | - | - | - | - | - | - | - | - | - | - | - | - | - | - | - | - | - | - | - | - | - | -104 |
| I1FGV4_AMPQE | - | - | - | - | - | - | - | - | - | - | - | - | - | - | - | - | - | - | - | - | - | - | - | - | - | - | G | K | L | I | F | I | H | S | T | D | E | N | - | - | - | - | - | - | - | - | - | - | - | - | - | - | - | - | - | - | - | - | - | - | - | -100 |
| K1PHX3_CRAGI | - | - | - | - | - | - | - | - | - | - | - | - | - | - | - | - | - | - | - | - | - | - | - | - | - | - | - | E | L | I | Y | L | R | S | S | D | R | Q | - | - | - | - | - | - | - | - | - | - | - | - | - | - | - | - | - | - | - | - | - | - | - | -100 |
| B3RNL8_TRIAD | - | - | - | - | - | - | - | - | - | - | - | - | - | - | - | - | - | - | - | - | - | - | - | - | - | - | K | R | P | C | Y | V | H | P | S | D | K | H | - | - | - | - | - | - | - | - | - | - | - | - | - | - | - | - | - | - | - | - | - | - | - | -102 |
| X1Y3H7_ANODA | - | - | - | - | - | - | - | - | - | - | - | - | - | - | - | - | - | - | - | - | - | - | - | - | - | - | D | E | L | I | Y | G | R | S | S | V | E | N | - | - | - | - | - | - | - | - | - | - | - | - | - | - | - | - | - | - | - | - | - | - | - | -104 |
| R7TWH9_CAPTE | - | - | - | - | - | - | - | - | - | - | - | - | - | - | - | - | - | - | - | - | - | - | - | - | - | - | - | - | L | I | Y | G | R | C | E | D | P | T | - | - | - | - | - | - | - | - | - | - | - | - | - | - | - | - | - | - | - | - | - | - | - | -104 |
| K1R5E8_CRAGI | - | - | - | - | - | - | - | - | - | - | - | - | - | - | - | - | - | - | - | - | - | - | - | - | - | - | - | - | L | V | Y | G | R | C | T | E | P | Q | - | - | - | - | - | - | - | - | - | - | - | - | - | - | - | - | - | - | - | - | - | - | - | -104 |
| XP_006811804 | - | - | - | - | - | - | - | - | - | - | - | - | - | - | - | - | - | - | - | - | - | - | - | - | - | - | - | - | V | I | F | A | K | S | S | N | S | K | - | - | - | - | - | - | - | - | - | - | - | - | - | - | - | - | - | - | - | - | - | - | - | -102 |
| C3YB23_BRAFL | - | - | - | - | - | - | - | - | - | - | - | - | - | - | - | - | - | - | - | - | - | - | - | - | - | - | - | - | P | M | Y | C | R | S | S | D | P | N | - | - | - | - | - | - | - | - | - | - | - | - | - | - | - | - | - | - | - | - | - | - | - | -109 |
| H2ZIF1_CIOSA | - | - | - | - | - | - | - | - | - | - | - | - | - | - | - | - | - | - | - | - | - | - | - | - | - | - | - | - | L | C | F | G | R | S | A | N | P | D | - | - | - | - | - | - | - | - | - | - | - | - | - | - | - | - | - | - | - | - | - | - | - | -101 |
| W4YV93_STRPU | - | - | - | - | - | - | - | - | - | - | - | - | - | - | - | - | - | - | - | - | - | - | - | - | - | - | - | - | R | V | F | A | S | S | S | D | P | N | - | - | - | - | - | - | - | - | - | - | - | - | - | - | - | - | - | - | - | - | - | - | - | -101 |
| CAN10_HUMAN | - | - | - | - | - | - | - | - | - | - | - | - | - | - | - | - | - | - | - | - | - | - | - | - | - | - | - | R | L | C | F | S | R | C | Q | R | - | E | D | - | - | - | - | - | - | - | - | - | - | - | - | - | - | - | - | - | - | - | - | - | - | -102 |
| CAN10_MOUSE | - | - | - | - | - | - | - | - | - | - | - | - | - | - | - | - | - | - | - | - | - | - | - | - | - | - | - | R | L | C | F | S | R | C | Q | R | - | E | D | - | - | - | - | - | - | - | - | - | - | - | - | - | - | - | - | - | - | - | - | - | - | -102 |
| XP_004943443 | - | - | - | - | - | - | - | - | - | - | - | - | - | - | - | - | - | - | - | - | - | - | - | - | - | - | - | K | L | C | F | S | Q | C | Q | T | - | E | D | - | - | - | - | - | - | - | - | - | - | - | - | - | - | - | - | - | - | - | - | - | - | -102 |
| F8W4K1_DANRE | - | - | - | - | - | - | - | - | - | - | - | - | - | - | - | - | - | - | - | - | - | - | - | - | - | - | - | N | L | C | F | S | R | C | Q | S | - | P | R | - | - | - | - | - | - | - | - | - | - | - | - | - | - | - | - | - | - | - | - | - | - | -102 |
| CANC_DROME | - | - | - | - | - | - | - | - | - | - | - | - | - | - | - | - | - | - | - | - | - | - | - | - | - | - | - | R | L | A | F | M | Q | P | Q | A | - | S | N | - | - | - | - | - | - | - | - | - | - | - | - | - | - | - | - | - | - | - | - | - | - | -88 |
| C3ZKN9_BRAFL | - | - | - | - | - | - | - | - | - | - | - | - | - | - | - | - | - | - | - | - | - | - | - | - | - | - | - | K | L | V | F | A | H | S | V | S | - | K | R | - | - | - | - | - | - | - | - | - | - | - | - | - | - | - | - | - | - | - | - | - | - | -100 |
| G4V623_SCHMA | - | - | - | - | - | - | - | - | - | - | - | - | - | - | - | - | - | - | - | - | - | - | - | - | - | - | - | Q | P | L | F | V | H | S | G | R | - | S | T | - | - | - | - | - | - | - | - | - | - | - | - | - | - | - | - | - | - | - | - | - | - | -119 |
| R7VK39_CAPTE | - | - | - | - | - | - | - | - | - | - | - | - | - | - | - | - | - | - | - | - | - | - | - | - | - | - | - | H | P | A | F | I | Q | N | E | K | N | L | H | - | - | - | - | - | - | - | - | - | - | - | - | - | - | - | - | - | - | - | - | - | - | -103 |
| G4V693_SCHMA | - | - | - | - | - | - | - | - | - | - | - | - | - | - | - | - | - | - | - | - | - | - | - | - | - | - | - | - | K | C | I | S | L | S | S | S | S | A | K | - | - | - | - | - | - | - | - | - | - | - | - | - | - | - | - | - | - | - | - | - | - | -105 |
| R7UPU6_CAPTE | - | - | - | - | - | - | - | - | - | - | - | - | - | - | - | - | - | - | - | - | - | - | - | - | - | D | G | F | V | P | W | G | A | R | S | T | N | R | N | - | - | - | - | - | - | - | - | - | - | - | - | - | - | - | - | - | - | - | - | - | - | -106 |
| R7V817_CAPTE | - | - | - | - | - | - | - | - | - | - | - | - | - | - | - | - | - | - | - | - | - | - | - | - | - | D | G | F | V | P | W | G | A | R | S | T | N | R | N | - | - | - | - | - | - | - | - | - | - | - | - | - | - | - | - | - | - | - | - | - | - | -103 |
| R7UNL8_CAPTE | - | - | - | - | - | - | - | - | - | - | - | - | - | - | - | - | - | - | - | - | - | - | - | - | - | S | E | F | K | L | W | G | A | K | S | T | D | D | R | - | - | - | - | - | - | - | - | - | - | - | - | - | - | - | - | - | - | - | - | - | - | -101 |
| K1PJ80_CRAGI | - | - | - | - | - | - | - | - | - | - | - | - | - | - | - | - | - | - | - | - | - | - | - | - | - | Q | I | Y | S | - | - | A | H | S | N | T | D | P | N | - | - | - | - | - | - | - | - | - | - | - | - | - | - | - | - | - | - | - | - | - | - | -104 |
| K1PCX8_CRAGI | - | - | - | - | - | - | - | - | - | - | - | - | - | - | - | - | - | - | - | - | - | - | - | - | - | Q | I | Y | S | - | - | A | H | S | V | T | D | P | N | - | - | - | - | - | - | - | - | - | - | - | - | - | - | - | - | - | - | - | - | - | - | -104 |
| CAND_DROME | - | - | - | - | - | - | - | - | - | - | - | - | - | - | - | - | - | - | - | - | - | - | - | - | - | R | G | H | L | V | Y | S | Q | A | - | - | - | - | - | - | - | - | - | - | - | - | - | - | - | - | - | - | - | - | - | - | - | - | - | - | - | -99 |
| XP_002735765 | - | - | - | - | - | - | - | - | - | - | - | - | - | - | - | - | - | - | - | - | - | - | - | - | - | N | G | F | L | V | Y | S | Q | A | - | - | - | - | - | - | - | - | - | - | - | - | - | - | - | - | - | - | - | - | - | - | - | - | - | - | - | -101 |
| CAN15_HUMAN | - | - | - | - | - | - | - | - | - | - | - | - | - | - | - | - | - | - | - | - | - | - | - | - | - | A | G | C | L | L | F | S | Q | A | - | - | - | - | - | - | - | - | - | - | - | - | - | - | - | - | - | - | - | - | - | - | - | - | - | - | - | -101 |
| CAN15_MOUSE | - | - | - | - | - | - | - | - | - | - | - | - | - | - | - | - | - | - | - | - | - | - | - | - | - | A | G | F | L | L | F | S | Q | A | - | - | - | - | - | - | - | - | - | - | - | - | - | - | - | - | - | - | - | - | - | - | - | - | - | - | - | -101 |
| E1BTA8_CHICK | - | - | - | - | - | - | - | - | - | - | - | - | - | - | - | - | - | - | - | - | - | - | - | - | - | C | G | Y | L | L | F | S | Q | A | - | - | - | - | - | - | - | - | - | - | - | - | - | - | - | - | - | - | - | - | - | - | - | - | - | - | - | -101 |
| E7FAX5_DANRE | - | - | - | - | - | - | - | - | - | - | - | - | - | - | - | - | - | - | - | - | - | - | - | - | - | Y | G | F | L | L | F | S | Q | A | - | - | - | - | - | - | - | - | - | - | - | - | - | - | - | - | - | - | - | - | - | - | - | - | - | - | - | -101 |
| C3XWK8_BRAFL | - | - | - | - | - | - | - | - | - | - | - | - | - | - | - | - | - | - | - | - | - | - | - | - | - | R | G | F | L | V | Y | S | Q | A | - | - | - | - | - | - | - | - | - | - | - | - | - | - | - | - | - | - | - | - | - | - | - | - | - | - | - | -100 |
| W4YMU4_STRPU | - | - | - | - | - | - | - | - | - | - | - | - | - | - | - | - | - | - | - | - | - | - | - | - | - | Q | G | F | L | V | Y | S | K | A | - | - | - | - | - | - | - | - | - | - | - | - | - | - | - | - | - | - | - | - | - | - | - | - | - | - | - | -101 |
| K1PV74_CRAGI | - | - | - | - | - | - | - | - | - | - | - | - | - | - | - | - | - | - | - | - | - | - | - | - | - | H | K | R | L | V | F | S | Q | A | - | - | - | - | - | - | - | - | - | - | - | - | - | - | - | - | - | - | - | - | - | - | - | - | - | - | - | -101 |
| R7TJF2_CAPTE | - | - | - | - | - | - | - | - | - | - | - | - | - | - | - | - | - | - | - | - | - | - | - | - | - | H | G | L | L | V | Y | S | Q | A | - | - | - | - | - | - | - | - | - | - | - | - | - | - | - | - | - | - | - | - | - | - | - | - | - | - | - | -101 |
| T2MI17_HYDVU | - | - | - | - | - | - | - | - | - | - | - | - | - | - | - | - | - | - | - | - | - | - | - | - | - | N | G | Q | L | V | F | S | Q | A | - | - | - | - | - | - | - | - | - | - | - | - | - | - | - | - | - | - | - | - | - | - | - | - | - | - | - | -98 |
| Q1RL55_CIOIN | - | - | - | - | - | - | - | - | - | - | - | - | - | - | - | - | - | - | - | - | - | - | - | - | - | K | R | R | L | I | Y | S | H | A | - | - | - | - | - | - | - | - | - | - | - | - | - | - | - | - | - | - | - | - | - | - | - | - | - | - | - | -88 |
| I1EA30_AMPQE | - | - | - | - | - | - | - | - | - | - | - | - | - | - | - | - | - | - | - | - | - | - | - | F | W | S | N | Q | L | V | F | S | K | A | - | - | - | - | - | - | - | - | - | - | - | - | - | - | - | - | - | - | - | - | - | - | - | - | - | - | - | -101 |
| G5EEZ6_CAEEL | - | - | - | - | - | - | - | - | - | - | - | - | - | - | - | - | - | - | - | - | - | - | - | - | K | T | N | S | I | A | M | A | V | G | - | - | - | - | - | - | - | - | - | - | - | - | - | - | - | - | - | - | - | - | - | - | - | - | - | - | - | -101 |
| O44903_CAEEL | - | - | - | - | - | - | - | - | - | - | - | - | - | - | - | - | - | - | - | - | - | - | - | - | N | T | N | S | I | A | I | A | V | G | - | - | - | - | - | - | - | - | - | - | - | - | - | - | - | - | - | - | - | - | - | - | - | - | - | - | - | -101 |
| O02260_CAEEL | - | - | - | - | - | - | - | - | - | - | - | - | - | - | - | - | - | - | - | - | - | - | - | - | - | - | D | G | I | R | I | G | A | T | - | - | - | - | - | - | - | - | - | - | - | - | - | - | - | - | - | - | - | - | - | - | - | - | - | - | - | -98 |
| O02259_CAEEL | - | - | - | - | - | - | - | - | - | - | - | - | - | - | - | - | - | - | - | - | - | - | - | - | - | - | D | D | M | R | I | G | A | T | - | - | - | - | - | - | - | - | - | - | - | - | - | - | - | - | - | - | - | - | - | - | - | - | - | - | - | -75 |
| C1MTG3_MICPC | - | - | - | - | - | - | - | - | - | - | R | P | E | V | N | - | - | - | - | K | R | R | A | L | D | D | F | A | P | A | F | S | R | A | - | - | - | - | - | - | - | - | - | - | - | - | - | - | - | - | - | - | - | - | - | - | - | - | - | - | - | -105 |
| C1E493_MICSR | - | - | - | - | - | - | - | - | - | - | - | - | K | V | G | - | - | - | - | - | A | R | S | N | D | E | Y | A | L | A | Y | S | R | A | - | - | - | - | - | - | - | - | - | - | - | - | - | - | - | - | - | - | - | - | - | - | - | - | - | - | - | -96 |
| Q012Z6_OSTTA | - | - | - | - | - | - | - | - | - | - | - | - | - | - | - | - | - | - | - | - | - | - | - | D | D | E | Y | S | L | A | F | S | R | A | - | - | - | - | - | - | - | - | - | - | - | - | - | - | - | - | - | - | - | - | - | - | - | - | - | - | - | -93 |
| L1IGB6_GUITH | - | - | - | - | - | - | - | - | - | - | - | - | - | - | - | - | - | - | - | - | - | E | H | A | L | E | T | N | L | A | F | S | R | G | - | - | - | - | - | - | - | - | - | - | - | - | - | - | - | - | - | - | - | - | - | - | - | - | - | - | - | -96 |
| B7FWX6_PHATC | - | - | - | - | - | - | - | - | - | - | Q | S | L | R | H | G | V | P | L | N | S | C | K | E | L | V | A | T | P | A | F | C | S | A | - | - | - | - | - | - | - | - | - | - | - | - | - | - | - | - | - | - | - | - | - | - | - | - | - | - | - | -101 |
| N9TPR9_ENTHI | - | - | - | - | - | - | - | - | - | - | - | - | - | - | - | - | - | - | - | - | - | - | - | - | - | - | - | Q | P | F | F | S | H | N | - | - | - | - | - | - | - | - | - | - | - | - | - | - | - | - | - | - | - | - | - | - | - | - | - | - | - | -90 |
| G0QS43_ICHMG | - | - | - | - | - | - | - | - | - | - | - | - | - | - | - | - | - | - | - | - | - | - | - | - | - | - | K | Q | P | A | F | T | R | G | - | - | - | - | - | - | - | - | - | - | - | - | - | - | - | - | - | - | - | - | - | - | - | - | - | - | - | -97 |
| D8LB20_ECTSI | - | - | - | - | - | - | - | - | - | - | - | - | - | - | - | - | - | - | - | - | - | - | - | - | - | - | - | N | P | I | F | A | K | P | - | - | - | - | - | - | - | - | - | - | - | - | - | - | - | - | - | - | - | - | - | - | - | - | - | - | - | -101 |
| Q9U0T9_LEIMA | - | - | - | - | - | - | - | - | - | - | - | - | - | - | - | - | - | - | - | - | - | - | - | - | - | - | A | P | A | F | A | H | S | R | - | - | - | - | - | - | - | - | - | - | - | - | - | - | - | - | - | - | - | - | - | - | - | - | - | - | - | -103 |
| Q4CXJ8_TRYCC | - | - | - | - | - | - | - | - | - | - | - | - | - | - | - | - | - | - | - | - | - | - | - | - | - | - | M | L | R | F | A | H | S | S | - | - | - | - | - | - | - | - | - | - | - | - | - | - | - | - | - | - | - | - | - | - | - | - | - | - | - | -103 |
| F4I0A4_ARATH | - | - | - | - | - | - | - | - | - | - | - | - | - | - | - | - | - | - | - | - | - | - | - | - | - | - | - | P | A | F | A | T | S | R | - | - | - | - | - | - | - | - | - | - | - | - | - | - | - | - | - | - | - | - | - | - | - | - | - | - | - | -100 |
| Q8RUQ1_MAIZE | - | - | - | - | - | - | - | - | - | - | - | - | - | - | - | - | - | - | - | - | - | - | - | - | - | - | - | P | A | F | A | T | S | R | - | - | - | - | - | - | - | - | - | - | - | - | - | - | - | - | - | - | - | - | - | - | - | - | - | - | - | -100 |
| D7FQ17_ECTSI | - | - | - | - | - | - | - | - | - | - | - | - | - | - | - | - | - | - | - | - | - | - | - | - | N | K | G | L | A | V | G | H | S | Y | - | - | - | - | - | - | - | - | - | - | - | - | - | - | - | - | - | - | - | - | - | - | - | - | - | - | - | -117 |
| D0MZ44_PHYIT | - | - | - | - | - | - | - | - | - | - | - | - | - | - | - | - | - | - | - | - | E | L | S | V | S | A | G | A | A | F | A | H | S | R | - | - | - | - | - | - | - | - | - | - | - | - | - | - | - | - | - | - | - | - | - | - | - | - | - | - | - | -118 |
| L1JBM0_GUITH | - | - | - | - | - | - | - | - | - | - | - | - | - | - | - | - | - | - | - | - | - | - | - | - | - | - | R | P | I | F | G | R | C | R | - | - | - | - | - | - | - | - | - | - | - | - | - | - | - | - | - | - | - | - | - | - | - | - | - | - | - | -71 |
| L1J739_GUITH | - | - | - | - | - | - | - | - | - | - | - | - | - | - | - | - | - | - | - | - | - | - | - | - | - | - | E | P | L | Y | A | R | C | K | - | - | - | - | - | - | - | - | - | - | - | - | - | - | - | - | - | - | - | - | - | - | - | - | - | - | - | -101 |
| L1J0U9_GUITH | - | - | - | - | - | - | - | - | - | - | - | - | - | - | - | - | - | - | - | - | - | - | - | - | - | - | V | P | L | Y | T | R | S | S | - | - | - | - | - | - | - | - | - | - | - | - | - | - | - | - | - | - | - | - | - | - | - | - | - | - | - | -95 |
| L1J1Q4_GUITH | - | - | - | - | - | - | - | - | - | - | - | - | - | - | - | - | - | - | - | - | - | - | - | - | - | F | V | P | A | F | G | R | C | V | - | - | - | - | - | - | - | - | - | - | - | - | - | - | - | - | - | - | - | - | - | - | - | - | - | - | - | -113 |
| D0ND88_PHYIT | - | - | - | - | - | - | - | - | - | - | G | S | T | F | S | - | - | - | - | - | - | - | - | - | P | G | S | A | L | Y | G | S | S | V | - | - | - | - | - | - | - | - | - | - | - | - | - | - | - | - | - | - | - | - | - | - | - | - | - | - | - | -116 |
| D2VVS6_NAEGR | - | - | - | - | - | - | - | - | - | - | - | - | - | - | - | - | - | - | - | - | - | - | - | - | - | N | S | Y | V | F | S | R | S | A | - | - | - | - | - | - | - | - | - | - | - | - | - | - | - | - | - | - | - | - | - | - | - | - | - | - | - | -99 |
| D2V3V2_NAEGR | - | - | - | - | - | - | - | - | - | - | - | - | - | - | - | - | - | - | - | - | - | - | - | - | - | G | S | L | A | F | S | R | S | G | - | - | - | - | - | - | - | - | - | - | - | - | - | - | - | - | - | - | - | - | - | - | - | - | - | - | - | -100 |
| Q5B5J7_EMENI | - | W | D | N | I | - | N | R | K | D | T | E | E | E | Y | R | R | A | F | Q | V | G | S | R | - | - | - | A | L | Y | F | A | Q | C | - | - | - | - | - | - | - | - | - | - | - | - | - | - | - | - | - | - | - | - | - | - | - | - | - | - | - | -119 |
| E4ZY10_LEPMJ | - | W | L | E | L | Q | N | R | K | S | P | E | E | E | Y | R | T | V | M | Q | T | G | S | R | - | - | - | A | L | Y | F | A | Q | C | - | - | - | - | - | - | - | - | - | - | - | - | - | - | - | - | - | - | - | - | - | - | - | - | - | - | - | -125 |
| A8PIE5_COPC7 | - | - | Q | A | K | L | T | Y | H | E | D | K | S | R | Y | N | Q | I | A | R | R | G | F | K | G | G | A | H | L | L | Y | A | G | A | - | - | - | - | - | - | - | - | - | - | - | - | - | - | - | - | - | - | - | - | - | - | - | - | - | - | - | -120 |
| A8NBA6_COPC7 | - | - | V | E | K | E | L | Y | H | H | D | K | D | L | Y | T | K | L | A | R | K | G | K | K | - | - | - | I | L | T | Y | A | K | S | - | - | - | - | - | - | - | - | - | - | - | - | - | - | - | - | - | - | - | - | - | - | - | - | - | - | - | -119 |
| A8NBA1_COPC7 | - | - | E | G | K | E | A | F | L | G | D | R | E | L | F | H | N | V | H | K | N | T | G | R | - | - | - | H | L | V | Y | A | R | S | - | - | - | - | - | - | - | - | - | - | - | - | - | - | - | - | - | - | - | - | - | - | - | - | - | - | - | -123 |
| A8NBA2_COPC7 | - | - | G | E | R | Q | L | F | V | E | D | R | D | L | F | H | H | V | Q | K | N | R | G | S | - | - | - | R | L | T | Y | A | K | S | - | - | - | - | - | - | - | - | - | - | - | - | - | - | - | - | - | - | - | - | - | - | - | - | - | - | - | -123 |
| A8NAE6_COPC7 | - | - | G | E | K | E | L | Y | H | F | E | K | S | R | Y | N | S | T | A | R | R | S | G | E | - | - | - | S | L | Y | F | A | K | S | A | G | K | K | K | T | D | V | S | D | S | V | K | P | D | C | D | N | V | D | G | G | H | G | G | S | G | -150 |
| A8NAI9_COPC7 | - | - | R | E | Q | Q | I | C | R | Q | D | K | D | A | Y | N | A | M | A | R | K | G | G | E | - | - | - | G | L | F | F | A | K | S | - | - | - | - | - | - | - | - | - | - | - | - | - | - | - | - | - | - | - | - | - | - | - | - | - | - | - | -119 |
| G4TER7_PIRID | - | - | D | E | Q | K | L | Y | H | N | D | P | D | L | Y | N | D | V | A | R | K | G | S | K | - | - | - | I | L | Y | F | A | K | G | - | - | - | - | - | - | - | - | - | - | - | - | - | - | - | - | - | - | - | - | - | - | - | - | - | - | - | -119 |
| G4TER6_PIRID | - | - | E | A | R | - | - | A | Q | G | T | V | D | E | Y | N | I | R | V | R | K | G | N | K | - | - | - | V | L | Y | F | A | K | S | - | - | - | - | - | - | - | - | - | - | - | - | - | - | - | - | - | - | - | - | - | - | - | - | - | - | - | -109 |
| G4TNA1_PIRID | - | - | E | E | A | K | V | Y | H | F | D | R | D | L | Y | N | S | I | A | R | K | G | R | K | - | - | - | I | L | C | F | G | R | G | - | - | - | - | - | - | - | - | - | - | - | - | - | - | - | - | - | - | - | - | - | - | - | - | - | - | - | -119 |
| D0MWB9_PHYIT | - | - | - | - | - | - | - | - | - | - | - | - | - | V | F | G | F | T | D | K | K | P | G | K | - | - | - | - | P | Y | F | A | R | C | R | - | - | - | - | - | - | - | - | - | - | - | - | - | - | - | - | - | - | - | - | - | - | - | - | - | - | -94 |
| L1I9H5_GUITH | - | - | - | - | - | - | - | - | - | - | - | - | - | - | - | - | - | - | - | - | - | - | - | - | - | L | W | Y | P | I | F | C | G | F | K | D | H | S | G | Q | E | V | N | - | - | - | - | - | - | - | - | - | - | - | - | - | - | - | - | - | - | -110 |
| Q7N497_PHOLL | - | - | - | - | - | - | - | - | - | - | - | - | - | - | - | - | - | - | - | - | - | - | - | - | - | - | - | - | - | - | F | S | A | M | E | D | K | N | - | - | - | - | - | - | - | - | - | - | - | - | - | - | - | - | - | - | - | - | - | - | - | -86 |
| ZP_11152369 | - | - | - | - | - | - | - | - | - | - | - | - | - | - | - | - | - | - | - | - | - | - | - | - | - | - | - | - | - | - | Y | S | A | M | Q | N | T | T | - | - | - | - | - | - | - | - | - | - | - | - | - | - | - | - | - | - | - | - | - | - | - | -82 |
| A2FEL7_TRIVA | - | - | - | - | - | - | - | - | - | - | - | - | - | - | - | - | - | - | - | - | - | - | - | - | - | - | G | K | P | R | M | V | H | P | A | T | - | - | - | - | - | - | - | - | - | - | - | - | - | - | - | - | - | - | - | - | - | - | - | - | - | -95 |
| A2D9A8_TRIVA | - | - | - | - | - | - | - | - | - | - | - | - | - | - | - | - | - | - | - | - | - | - | - | - | - | - | G | K | P | K | M | V | S | P | L | N | - | - | - | - | - | - | - | - | - | - | - | - | - | - | - | - | - | - | - | - | - | - | - | - | - | -96 |
| A2FHB3_TRIVA | - | - | - | - | - | - | - | - | - | - | - | - | - | - | - | - | - | - | - | - | - | - | - | - | - | - | G | K | G | K | F | V | H | P | R | D | P | - | - | - | - | - | - | - | - | - | - | - | - | - | - | - | - | - | - | - | - | - | - | - | - | -97 |
| A2DTE8_TRIVA | - | - | - | - | - | - | - | - | - | - | - | - | - | - | - | - | - | - | - | - | - | - | - | - | - | - | G | F | I | K | L | S | G | P | T | S | A | - | - | - | - | - | - | - | - | - | - | - | - | - | - | - | - | - | - | - | - | - | - | - | - | -94 |
| A2E4X2_TRIVA | - | - | - | - | - | - | - | - | - | - | - | - | - | - | - | - | - | - | - | - | - | - | - | - | - | - | G | Q | P | I | F | S | Y | P | R | T | - | - | - | - | - | - | - | - | - | - | - | - | - | - | - | - | - | - | - | - | - | - | - | - | - | -99 |
| A2E1X1_TRIVA | - | - | - | - | - | - | - | - | - | - | - | - | - | - | - | - | - | - | - | - | - | - | - | - | - | - | - | C | P | K | F | A | H | P | R | S | - | - | - | - | - | - | - | - | - | - | - | - | - | - | - | - | - | - | - | - | - | - | - | - | - | -84 |
| TPR_PORGI | - | - | - | - | - | - | - | - | - | - | - | - | - | - | - | - | - | - | - | - | - | - | - | - | - | - | - | - | - | - | - | - | - | - | - | - | - | - | - | - | - | - | - | - | - | - | - | - | - | Y | Y | K | V | P | I | F | A | R | S | R | Y | -113 |
| D5MF42_9BACT | - | - | - | - | - | - | - | - | - | - | - | - | - | - | - | - | - | - | - | - | - | - | - | - | - | - | - | - | - | - | - | - | - | - | - | - | - | - | - | - | - | - | - | - | - | - | - | - | - | G | T | H | A | W | I | Y | A | R | S | - | - | -73 |
| R9H254_BACT4 | - | - | - | - | - | - | - | - | - | - | - | - | - | - | - | - | - | - | - | - | - | - | - | - | - | - | - | - | - | - | - | - | - | - | - | - | - | - | - | - | - | - | - | - | - | - | - | - | - | - | - | N | S | G | D | L | G | A | L | G | G | -74 |
| ZP_04847694 | - | - | - | - | - | - | - | - | - | - | - | - | - | - | - | - | - | - | - | - | - | - | - | - | - | - | - | - | - | - | - | - | - | - | - | - | - | - | - | - | - | - | - | - | - | - | - | - | - | - | - | N | S | G | D | L | G | A | L | G | G | -74 |
| D7IBA4_9BACE | - | - | - | - | - | - | - | - | - | - | - | - | - | - | - | - | - | - | - | - | - | - | - | - | - | - | - | - | - | - | - | - | - | - | - | - | - | - | - | - | - | - | - | - | - | - | - | - | - | - | - | N | S | G | D | L | G | A | L | G | G | -72 |
| ZP_09858577 | - | - | - | - | - | - | - | - | - | - | - | - | - | - | - | - | - | - | - | - | - | - | - | - | - | - | - | - | - | - | - | - | - | - | - | - | - | - | - | - | - | - | - | - | - | - | - | - | - | - | - | N | S | G | D | L | G | A | L | G | G | -72 |
| E5C759_9BACE | - | - | - | - | - | - | - | - | - | - | - | - | - | - | - | - | - | - | - | - | - | - | - | - | - | - | - | - | - | - | - | - | - | - | - | - | - | - | - | - | - | - | - | - | - | - | - | - | - | - | - | N | S | G | D | L | G | A | L | G | G | -74 |
| A7LTS0_BACO1 | - | - | - | - | - | - | - | - | - | - | - | - | - | - | - | - | - | - | - | - | - | - | - | - | - | - | - | - | - | - | - | - | - | - | - | - | - | - | - | - | - | - | - | - | - | - | - | - | - | - | - | T | S | G | S | L | G | A | S | S | G | -72 |
| Q8YQK8_NOSS1 | - | - | - | - | - | - | - | - | - | - | - | - | - | - | - | - | - | - | - | - | - | - | - | - | - | - | - | - | - | - | - | - | - | - | - | - | - | - | - | - | - | - | - | - | - | - | - | - | - | - | - | - | - | - | - | - | - | I | S | A | K | -84 |
| Q3MBX8_ANAVT | - | - | - | - | - | - | - | - | - | - | - | - | - | - | - | - | - | - | - | - | - | - | - | - | - | - | - | - | - | - | - | - | - | - | - | - | - | - | - | - | - | - | - | - | - | - | - | - | - | - | - | - | - | - | - | - | - | V | A | A | K | -89 |
| Q8YZT8_NOSS1 | - | - | - | - | - | - | - | - | - | - | - | - | - | - | - | - | - | - | - | - | - | - | - | - | - | - | - | - | - | - | - | - | - | - | - | - | - | - | - | - | - | - | - | - | - | - | - | - | - | - | - | - | - | - | - | - | - | I | A | A | Q | -77 |
| K9UIG2_9CHRO | - | - | - | - | - | - | - | - | - | - | - | - | - | - | - | - | - | - | - | - | - | - | - | - | - | - | - | - | - | - | - | - | - | - | - | - | - | - | - | - | - | - | - | - | - | - | - | - | - | - | - | - | - | - | - | - | N | L | F | G | T | -80 |
| A4YRP8_BRASO | - | - | - | - | - | - | - | - | - | - | - | - | - | - | - | - | - | - | - | - | - | - | - | - | - | - | - | - | - | - | - | - | - | - | - | - | - | - | - | - | - | - | - | - | - | - | - | - | - | - | - | - | - | - | - | - | G | T | I | F | N | -71 |
| H0TAL7_9BRAD | - | - | - | - | - | - | - | - | - | - | - | - | - | - | - | - | - | - | - | - | - | - | - | - | - | - | - | - | - | - | - | - | - | - | - | - | - | - | - | - | - | - | - | - | - | - | - | - | - | - | - | - | - | - | - | - | G | T | I | F | N | -75 |
| H0SNL4_9BRAD | - | - | - | - | - | - | - | - | - | - | - | - | - | - | - | - | - | - | - | - | - | - | - | - | - | - | - | - | - | - | - | - | - | - | - | - | - | - | - | - | - | - | - | - | - | - | - | - | - | - | - | - | - | - | - | - | G | N | I | F | N | -77 |
| H0S4P6_9BRAD | - | - | - | - | - | - | - | - | - | - | - | - | - | - | - | - | - | - | - | - | - | - | - | - | - | - | - | - | - | - | - | - | - | - | - | - | - | - | - | - | - | - | - | - | - | - | - | - | - | - | - | - | - | - | - | - | G | N | I | F | N | -77 |
| A5EMK2_BRASB | - | - | - | - | - | - | - | - | - | - | - | - | - | - | - | - | - | - | - | - | - | - | - | - | - | - | - | - | - | - | - | - | - | - | - | - | - | - | - | - | - | - | - | - | - | - | - | - | - | - | - | - | - | - | - | - | G | T | I | F | N | -71 |
| M4ZBJ4_9BRAD | - | - | - | - | - | - | - | - | - | - | - | - | - | - | - | - | - | - | - | - | - | - | - | - | - | - | - | - | - | - | - | - | - | - | - | - | - | - | - | - | - | - | - | - | - | - | - | - | - | - | - | - | - | - | - | - | G | N | I | F | N | -77 |
| Q07SP7_RHOP5 | - | - | - | - | - | - | - | - | - | - | - | - | - | - | - | - | - | - | - | - | - | - | - | - | - | - | - | - | - | - | - | - | - | - | - | - | - | - | - | - | - | - | - | - | - | - | - | - | - | - | - | - | - | - | - | - | G | S | L | F | N | -72 |
| B0JUQ7_MICAN | - | - | - | - | - | - | - | - | - | - | - | - | - | - | - | - | - | - | - | - | - | - | - | - | A | S | G | S | F | Y | Y | A | N | R | D | R | - | - | - | - | - | - | - | - | - | - | - | - | - | - | - | - | - | - | - | G | L | L | Y | N | N | -88 |
| K9WJ87_9CYAN | - | - | - | - | - | - | - | - | - | - | - | - | - | - | - | - | - | - | - | - | - | - | - | - | A | G | G | N | A | V | F | A | G | W | G | - | - | - | - | - | - | - | - | - | - | - | - | - | - | - | - | - | - | - | - | G | G | S | Y | N | E | -83 |
| G6FUE0_9CYAN | - | - | - | - | - | - | - | - | - | - | - | - | - | - | - | - | - | - | - | - | - | - | - | - | Q | E | G | Y | F | V | Y | A | S | K | D | - | - | - | - | - | - | - | - | - | - | - | - | - | - | - | - | - | - | - | - | - | N | Y | Y | G | N | -82 |
| K9PHG5_9CYAN | - | - | - | - | - | - | - | - | - | - | - | - | - | - | - | - | - | - | - | - | - | - | - | - | S | D | G | D | L | V | Y | A | N | Q | A | G | - | - | - | - | - | - | - | - | - | - | - | - | - | - | - | - | - | - | - | A | K | L | G | K | Y | -84 |
| Q8YXX3_NOSS1 | - | - | - | - | - | - | - | - | - | - | - | - | - | - | - | - | - | - | - | - | - | - | - | - | S | G | G | N | I | P | Y | A | K | I | G | - | - | - | - | - | - | - | - | - | - | - | - | - | - | - | - | - | - | - | - | - | G | R | H | D | N | -94 |
| K9TN70_9CYAN | - | - | - | - | - | - | - | - | - | - | - | - | - | - | - | - | - | - | - | - | - | - | - | - | D | S | G | Q | F | I | F | A | S | V | G | G | T | L | Q | Q | - | - | - | - | - | S | W | W | S | A | N | P | Y | Y | V | Q | N | T | Y | N | N | -98 |
| Q05QW3_9SYNE | - | - | - | - | - | - | - | - | - | - | - | - | - | - | - | - | - | - | - | - | - | - | - | - | S | N | G | R | Y | M | Y | A | D | - | - | D | G | - | - | - | - | - | - | - | - | - | - | - | - | - | - | - | - | - | A | A | G | K | Q | Q | V | -88 |
| Q05QW2_9SYNE | - | - | - | - | - | - | - | - | - | - | - | - | - | - | - | - | - | - | - | - | - | - | - | - | A | D | G | T | Y | L | H | A | D | A | G | D | G | - | - | - | - | - | - | - | - | - | - | - | - | - | - | - | - | - | T | A | T | N | A | L | V | -90 |
| Q05QW4_9SYNE | - | - | - | - | - | - | - | - | - | - | - | - | - | - | - | - | - | - | - | - | - | - | - | - | A | T | D | R | Y | L | Y | A | N | S | G | G | D | - | - | - | - | - | - | - | - | - | - | - | - | - | - | - | - | - | N | G | V | Q | N | V | V | -90 |
| E0UGX6_CYAP2 | - | - | - | - | - | - | - | - | - | - | - | - | - | - | - | - | - | - | - | - | - | - | - | - | S | S | G | N | L | V | Y | A | N | S | G | S | - | - | - | - | - | - | - | - | - | - | - | - | - | - | - | - | - | - | S | A | T | N | S | - | - | -87 |
| K7WKF4_9NOST | - | - | - | - | - | - | - | - | - | - | - | - | - | - | - | - | - | - | - | - | - | - | - | - | G | N | - | Y | A | A | Y | A | G | W | G | G | G | - | - | - | - | - | - | - | - | - | - | - | - | - | - | - | - | - | S | V | T | S | T | - | - | -83 |
| E0UGY5_CYAP2 | - | - | - | - | - | - | - | - | - | - | - | - | - | - | - | - | - | - | - | - | - | - | - | - | Q | Y | G | Y | V | V | Y | A | G | L | G | N | - | - | - | - | - | - | - | - | - | - | - | - | - | - | - | - | - | - | L | Y | N | N | P | - | - | -87 |
| A8LCE5_FRASN | - | - | - | - | - | - | - | - | - | - | - | - | - | - | - | - | - | - | - | - | - | - | - | - | - | - | - | - | - | - | - | - | - | - | - | - | - | - | - | - | - | - | - | - | - | - | R | A | G | A | G | Q | E | I | A | A | D | A | D | N | A | -89 |
| D3CT47_9ACTO | - | - | - | - | - | - | - | - | - | - | - | - | - | - | - | - | - | - | - | - | - | - | - | - | - | - | - | - | - | - | - | - | - | - | - | - | - | - | - | - | - | - | - | - | - | - | E | A | E | T | Q | R | E | I | G | A | G | A | E | N | A | -93 |
| Q2J4J6_FRASC | - | - | - | - | - | - | - | - | - | - | - | - | - | - | - | - | - | - | - | - | - | - | - | - | R | S | E | D | E | A | P | G | P | R | A | P | G | T | S | R | - | - | - | - | - | S | R | A | A | G | Y | E | E | V | A | M | D | G | D | D | A | -114 |
| Q0RAZ2_FRAAA | - | - | - | - | - | - | - | - | - | - | - | - | - | - | - | - | - | - | - | - | - | - | - | - | - | - | - | - | - | - | - | A | P | A | S | T | G | R | E | G | - | - | - | - | - | E | R | G | E | G | V | Q | E | I | A | M | D | G | D | N | A | -101 |
| E3J3F6_FRASU | - | - | - | - | - | - | - | - | - | - | - | - | - | - | - | - | - | - | - | - | - | - | - | - | - | - | - | - | G | S | G | - | - | - | - | - | - | - | - | - | - | - | - | - | - | D | A | G | G | R | P | A | R | T | G | M | A | A | Y | D | V | -95 |
| ZP_09168240 | - | - | - | - | - | - | - | - | - | - | - | - | - | - | - | - | - | - | - | - | - | - | - | - | - | - | - | - | A | A | G | G | A | A | G | G | G | G | G | G | - | - | - | - | - | E | A | G | G | A | R | P | V | P | S | I | A | A | F | D | L | -105 |
| C7MDV9_BRAFD | - | - | - | - | - | - | - | - | - | - | - | - | - | - | - | - | - | - | - | - | - | - | - | - | - | - | - | - | - | - | - | - | - | - | - | - | - | - | - | - | - | - | - | - | - | - | - | - | - | - | - | S | R | G | M | F | Q | Y | G | R | R | -66 |
| ZP_10094989 | - | - | - | - | - | - | - | - | - | - | - | - | - | - | - | - | - | - | - | - | - | - | - | - | - | - | - | - | - | - | - | - | - | - | - | - | - | - | - | - | - | - | - | - | - | - | - | - | - | - | - | A | R | G | V | F | Q | Y | G | R | R | -66 |
| A4X268_SALTO | - | - | - | - | - | - | - | - | - | - | - | - | - | - | - | - | - | - | - | - | - | - | - | - | - | - | - | - | - | - | - | - | - | - | - | - | - | - | - | - | - | - | - | - | - | - | D | S | V | S | G | E | S | A | Y | A | D | Q | S | V | I | -85 |
| WP_043162213 | - | - | - | - | - | - | - | - | - | - | - | - | - | - | - | - | - | - | - | - | - | - | - | - | - | - | - | - | - | - | - | - | - | - | - | - | - | - | - | - | - | - | - | - | - | - | D | S | V | S | G | E | S | A | Y | A | D | Q | S | V | I | -85 |
| Q0BRV8_GRABC | - | - | - | - | - | - | - | - | - | - | - | - | - | - | - | - | - | - | - | - | - | - | - | - | - | - | - | - | - | - | - | - | - | - | - | - | - | - | - | - | - | - | - | - | - | - | F | L | N | S | G | V | N | N | G | S | S | Q | N | V | V | -95 |
| A9B2P0_HERA2 | - | - | - | - | - | - | - | - | - | - | - | - | - | - | - | - | - | - | - | - | - | - | - | - | - | - | - | - | - | - | - | - | - | - | - | - | - | - | - | - | - | - | - | - | - | - | K | G | S | G | A | L | G | Y | A | Q | - | - | - | - | L | -85 |
| A9B3U8_HERA2 | - | - | - | - | - | - | - | - | - | - | - | - | - | - | - | - | - | - | - | - | - | - | - | - | - | - | - | - | - | - | - | - | - | - | - | - | - | - | - | - | - | - | - | - | - | - | K | G | A | I | D | P | A | F | A | K | - | - | - | - | L | -87 |
| A9B2A3_HERA2 | - | - | - | - | - | - | - | - | - | - | - | - | - | - | - | - | - | - | - | - | - | - | - | - | - | - | - | - | - | - | - | - | - | - | - | - | - | - | - | - | - | - | - | - | - | - | A | D | G | T | Q | P | Y | I | H | E | N | Q | E | V | L | -97 |
| D6ZC85_SEGRD | - | - | - | - | - | - | - | - | - | - | - | - | - | - | - | - | - | - | - | - | - | - | - | - | - | - | - | - | - | - | - | - | - | - | - | - | - | - | - | - | - | - | - | - | - | - | D | I | H | A | A | G | A | S | S | V | Q | T | - | - | - | -103 |
| E5XTT9_9ACTO | - | - | - | - | - | - | - | - | - | - | - | - | - | - | - | - | - | - | - | - | - | - | - | - | - | - | - | - | - | - | - | - | - | - | - | - | - | - | - | - | - | - | - | - | - | - | D | I | R | A | G | G | A | S | S | L | D | D | - | - | - | -103 |
| E5XTU0_9ACTO | - | - | - | - | - | - | - | - | - | - | - | - | - | - | - | - | - | - | - | - | - | - | - | - | - | - | - | - | - | - | - | - | - | - | - | - | - | - | - | - | - | - | - | - | - | - | N | L | A | T | T | G | A | S | G | L | D | D | S | N | T | -107 |
| D6B0I6_9ACTO | - | - | - | - | - | - | - | - | - | - | - | - | - | - | - | - | - | - | - | - | - | - | - | - | - | - | - | - | - | - | - | - | - | - | - | - | - | - | - | - | - | - | - | - | - | - | Q | N | G | N | P | I | S | T | Y | G | N | G | - | - | - | -85 |
| D9X719_STRVR | - | - | - | - | - | - | - | - | - | - | - | - | - | - | - | - | - | - | - | - | - | - | - | - | - | - | - | - | - | - | - | - | - | - | - | - | - | - | - | - | - | - | - | - | - | - | Q | N | G | D | P | I | G | T | Y | G | N | G | - | - | - | -85 |
| H6L010_SAPGL | - | - | - | - | - | - | - | - | - | - | - | - | - | - | - | - | - | - | - | - | - | - | - | - | - | - | - | - | - | - | - | - | - | - | - | - | - | - | - | - | - | - | - | - | - | - | - | - | - | - | - | E | N | G | N | P | A | Y | A | R | - | -78 |
| K1ZP83_9BACT | - | - | - | - | - | - | - | - | - | - | - | - | - | - | - | - | - | - | - | - | - | - | - | - | - | - | - | - | - | - | - | - | - | - | - | - | - | - | - | - | - | - | - | - | - | - | - | - | - | - | - | G | T | Q | N | - | L | Y | A | R | G | -98 |
| ADGB_HUMAN | - | - | - | - | - | - | - | - | - | - | - | - | - | - | - | - | - | - | - | - | - | - | - | - | - | - | - | N | L | L | L | P | A | T | T | Y | E | F | E | - | - | - | - | - | - | - | - | - | - | - | - | - | - | - | - | - | - | - | - | - | - | -135 |
| J3QMP3_MOUSE | - | - | - | - | - | - | - | - | - | - | - | - | - | - | - | - | - | - | - | - | - | - | - | - | - | - | - | N | L | L | L | P | A | T | S | Y | E | F | E | - | - | - | - | - | - | - | - | - | - | - | - | - | - | - | - | - | - | - | - | - | - | -135 |
| H2XR46_CIOIN | - | - | - | - | - | - | - | - | - | - | - | - | - | - | - | - | - | - | - | - | - | - | - | - | - | - | - | Q | L | L | L | P | I | T | T | S | N | H | E | - | - | - | - | - | - | - | - | - | - | - | - | - | - | - | - | - | - | - | - | - | - | -132 |
| CAN7_MOUSE | - | - | - | - | - | - | - | - | - | - | - | - | - | - | - | - | - | - | - | - | - | - | - | - | - | - | - | E | L | L | C | S | Y | S | N | N | K | S | E | - | - | - | - | - | - | - | - | - | - | - | - | - | - | - | - | - | - | - | - | - | - | -109 |
| CAN7_HUMAN | - | - | - | - | - | - | - | - | - | - | - | - | - | - | - | - | - | - | - | - | - | - | - | - | - | - | - | E | L | L | C | S | Y | S | N | N | K | S | E | - | - | - | - | - | - | - | - | - | - | - | - | - | - | - | - | - | - | - | - | - | - | -109 |
| E1BY84_CHICK | - | - | - | - | - | - | - | - | - | - | - | - | - | - | - | - | - | - | - | - | - | - | - | - | - | - | - | E | L | L | C | S | Y | S | N | N | K | N | E | - | - | - | - | - | - | - | - | - | - | - | - | - | - | - | - | - | - | - | - | - | - | -109 |
| H9GX91_DANRE | - | - | - | - | - | - | - | - | - | - | - | - | - | - | - | - | - | - | - | - | - | - | - | - | - | - | - | E | L | L | C | S | Y | S | S | N | R | N | E | - | - | - | - | - | - | - | - | - | - | - | - | - | - | - | - | - | - | - | - | - | - | -109 |
| F6SGI6_CIOIN | - | - | - | - | - | - | - | - | - | - | - | - | - | - | - | - | - | - | - | - | - | - | - | - | - | - | - | K | L | L | C | S | Y | S | A | N | R | N | E | - | - | - | - | - | - | - | - | - | - | - | - | - | - | - | - | - | - | - | - | - | - | -109 |
| W4Y195_STRPU | - | - | - | - | - | - | - | - | - | - | - | - | - | - | - | - | - | - | - | - | - | - | - | - | - | - | - | D | L | L | C | S | F | S | S | N | R | N | E | - | - | - | - | - | - | - | - | - | - | - | - | - | - | - | - | - | - | - | - | - | - | -83 |
| R7V270_CAPTE | - | - | - | - | - | - | - | - | - | - | - | - | - | - | - | - | - | - | - | - | - | - | - | - | - | - | - | E | L | L | C | S | Y | S | I | N | R | N | E | - | - | - | - | - | - | - | - | - | - | - | - | - | - | - | - | - | - | - | - | - | - | -109 |
| G4LXK3_SCHMA | - | - | - | - | - | - | - | - | - | - | - | - | - | - | - | - | - | - | - | - | - | - | - | - | - | - | - | K | L | L | C | C | Y | S | N | N | Q | N | E | - | - | - | - | - | - | - | - | - | - | - | - | - | - | - | - | - | - | - | - | - | - | -109 |
| T2M2K0_HYDVU | - | - | - | - | - | - | - | - | - | - | - | - | - | - | - | - | - | - | - | - | - | - | - | - | - | - | - | Q | L | L | C | S | F | S | Q | N | C | N | E | - | - | - | - | - | - | - | - | - | - | - | - | - | - | - | - | - | - | - | - | - | - | -109 |
| B3RQK1_TRIAD | - | - | - | - | - | - | - | - | - | - | - | - | - | - | - | - | - | - | - | - | - | - | - | - | - | - | - | Q | L | L | C | S | F | S | H | N | R | D | E | - | - | - | - | - | - | - | - | - | - | - | - | - | - | - | - | - | - | - | - | - | - | -109 |
| I1CS59_RHIO9 | - | - | - | - | - | - | - | - | - | - | - | - | - | - | - | - | - | - | - | - | - | - | - | - | - | - | - | T | L | M | C | T | F | S | T | N | K | G | E | - | - | - | - | - | - | - | - | - | - | - | - | - | - | - | - | - | - | - | - | - | - | -109 |
| I1GER3_AMPQE | - | - | - | - | - | - | - | - | - | - | - | - | - | - | - | - | - | - | - | - | - | - | - | - | - | - | - | N | I | L | G | S | Y | S | N | S | R | D | E | - | - | - | - | - | - | - | - | - | - | - | - | - | - | - | - | - | - | - | - | - | - | -109 |
| Q22143_CAEEL | - | - | - | - | - | - | - | - | - | - | - | - | - | - | - | - | - | - | - | - | - | - | - | - | - | - | - | R | M | M | C | S | Q | T | E | N | K | G | E | - | - | - | - | - | - | - | - | - | - | - | - | - | - | - | - | - | - | - | - | - | - | -109 |
| L1JZN7_GUITH | - | - | - | - | - | - | - | - | - | - | - | - | - | - | - | - | - | - | - | - | - | - | - | - | - | - | - | Q | L | L | C | S | Y | S | R | I | G | E | E | - | - | - | - | - | - | - | - | - | - | - | - | - | - | - | - | - | - | - | - | - | - | -110 |
| D0NT34_PHYIT | - | - | - | - | - | - | - | - | - | - | - | - | - | - | - | - | - | - | - | - | - | - | - | - | - | - | - | Q | L | L | S | S | C | T | T | R | K | N | E | - | - | - | - | - | - | - | - | - | - | - | - | - | - | - | - | - | - | - | - | - | - | -118 |
| A0A064B913_ASPOZ | - | - | - | - | - | - | - | - | - | - | - | - | - | - | - | - | - | - | - | - | - | - | - | - | - | - | - | S | L | H | V | I | D | R | K | N | P | N | F | - | - | - | - | - | - | - | - | - | - | - | - | - | - | - | - | - | - | - | - | - | - | -117 |
| A8N4X4_COPC7 | - | - | - | - | - | - | - | - | - | - | - | - | - | - | - | - | - | - | - | - | - | - | - | - | - | - | - | W | M | C | M | S | I | S | P | Q | T | K | S | P | Y | T | - | - | - | - | - | - | - | - | - | - | - | - | - | - | - | - | - | - | - | -133 |
| C0H5K1_PLAF7 | - | - | - | - | - | - | - | - | - | - | - | - | - | - | - | - | - | - | - | - | - | - | - | - | - | - | - | S | L | L | V | A | Y | S | N | N | Q | K | E | - | - | - | - | - | - | - | - | - | - | - | - | - | - | - | - | - | - | - | - | - | - | -116 |
| K1REZ8_CRAGI | - | - | - | - | - | - | - | - | - | - | - | - | - | - | - | - | - | - | - | - | - | - | - | - | - | - | G | I | R | L | L | S | Y | C | H | D | K | D | - | - | - | - | - | - | - | - | - | - | - | - | - | - | - | - | - | - | - | - | - | - | - | -95 |
| F4NTM9_BATDJ | - | - | - | - | - | - | - | - | - | - | - | - | - | - | - | - | - | - | - | - | - | - | - | - | - | - | - | E | W | L | C | S | L | S | K | N | K | Q | - | - | - | - | - | - | - | - | - | - | - | - | - | - | - | - | - | - | - | - | - | - | - | -111 |
| Q6F6K5_CAEEL | - | - | - | - | - | - | - | - | - | - | - | - | - | - | - | - | - | - | - | - | - | - | - | - | - | - | - | Y | E | I | F | T | P | M | V | R | - | - | - | - | - | - | - | - | - | - | - | - | - | - | - | - | - | - | - | - | - | - | - | - | - | -75 |
| A8HZK8_CHLRE | - | - | - | - | - | - | - | - | - | - | - | - | - | - | - | - | - | - | - | - | - | - | - | - | - | - | - | R | L | A | F | A | C | S | A | V | V | G | - | - | - | - | - | - | - | - | - | - | - | - | - | - | - | - | - | - | - | - | - | - | - | -77 |
| A8J6T6_CHLRE | - | - | - | - | - | - | - | - | - | - | - | - | - | - | - | - | - | - | - | - | - | - | - | - | - | - | - | N | S | I | T | K | T | K | I | L | G | N | - | - | - | - | - | - | - | - | - | - | - | - | - | - | - | - | - | - | - | - | - | - | - | -107 |
| Z9JR51_9MICO | - | - | - | - | - | - | - | - | - | - | - | - | - | - | - | - | - | - | - | - | - | - | - | - | - | - | A | R | Q | V | D | G | S | D | W | A | D | G | E | - | - | - | - | - | - | - | - | - | - | - | - | - | - | - | - | - | - | - | - | - | - | -95 |
| F7ZZH9_CELGA | - | - | - | - | - | - | - | - | - | - | - | - | - | - | - | - | - | - | - | - | - | - | - | - | - | - | - | D | W | D | W | W | I | F | G | G | D | K | - | - | - | - | - | - | - | - | - | - | - | - | - | - | - | - | - | - | - | - | - | - | - | -79 |
| Q7NDU2_GLOVI | - | - | - | - | - | - | - | - | - | - | - | - | - | - | - | - | - | - | - | - | - | - | - | - | - | - | - | - | - | - | - | - | - | - | - | - | - | - | - | - | - | - | - | - | - | - | - | - | - | - | - | - | - | - | - | - | - | - | - | - | - | -69 |
| I6AT95_9BACT | - | - | - | - | - | - | - | - | - | - | - | - | - | - | - | - | - | - | - | - | - | - | - | - | - | - | - | - | - | - | - | - | - | - | - | - | - | - | - | - | - | - | - | - | - | - | - | - | - | - | - | - | - | - | - | - | - | - | - | - | - | -68 |
| C7MGJ8_BRAFD | - | - | - | - | - | - | - | - | - | - | - | - | - | - | - | - | - | - | - | - | - | - | - | - | - | - | Y | Q | G | A | Y | A | G | P | G | G | A | D | F | G | - | - | - | - | - | - | - | - | - | - | - | - | - | - | - | - | - | - | - | - | D | -81 |
| D0WP73_9ACTO | - | - | - | - | - | - | - | - | - | - | - | - | - | - | - | - | - | - | - | - | - | - | - | - | - | - | - | R | T | V | F | V | D | D | V | Y | A | N | G | - | - | - | - | - | - | - | - | - | - | - | - | - | - | - | - | - | - | - | V | T | G | -81 |
| B0C8L1_ACAM1 | - | - | - | - | - | - | - | - | - | - | - | - | - | - | - | - | - | - | - | - | - | - | - | - | - | - | - | E | D | A | Y | G | K | G | - | - | - | - | - | - | - | - | - | - | - | - | - | - | - | - | - | - | - | - | - | - | - | - | - | - | - | -83 |
| B3E5A8_GEOLS | - | - | - | - | - | - | - | - | - | - | - | - | - | - | - | - | - | - | - | - | - | - | - | - | - | - | - | D | S | T | L | Q | D | G | - | - | - | - | - | - | - | - | - | - | - | - | - | - | - | - | - | - | - | - | - | - | - | - | - | - | - | -74 |
| D4YQ94_9MICO | - | - | - | - | - | - | - | - | - | - | - | - | - | - | - | - | - | - | - | - | - | - | - | - | - | - | - | - | - | A | G | D | N | R | L | D | A | N | N | - | - | - | - | - | - | - | - | - | - | - | - | - | - | - | - | - | - | - | - | - | - | -80 |
| PAPA_CARPA | - | - | - | - | - | - | - | - | - | - | - | - | - | - | - | - | - | - | - | - | - | - | - | - | - | - | - | - | - | - | - | - | - | - | - | - | - | - | - | - | - | - | - | - | - | - | - | - | - | - | - | - | - | - | - | - | - | - | - | - | - | -66 |
| RIM13_YEAST | - | - | - | - | - | - | - | - | - | - | - | - | - | - | - | - | - | - | - | - | - | - | - | - | - | - | - | K | Q | L | S | L | K | S | N | - | - | - | - | - | - | - | - | - | - | - | - | - | - | - | - | - | - | - | - | - | - | - | - | - | - | -109 |

| I |  |  |  |  |  |  |  |  |  |  |  |  |  |  |  |  |  |  |  |  |  |  |  |  |  |  |  |  |  |  |  |  |  |  |  |  |  |  |  |  |  |  |  |  |  |  |  |  |  |  |  |  |  |  |  |  |  |  |  |  |  |  |
| --- | --- | --- | --- | --- | --- | --- | --- | --- | --- | --- | --- | --- | --- | --- | --- | --- | --- | --- | --- | --- | --- | --- | --- | --- | --- | --- | --- | --- | --- | --- | --- | --- | --- | --- | --- | --- | --- | --- | --- | --- | --- | --- | --- | --- | --- | --- | --- | --- | --- | --- | --- | --- | --- | --- | --- | --- | --- | --- | --- | --- | --- | --- |
| Q9N4B1_CAEEL | - | - | - | - | - | E | F | W | S | A | L | L | E | K | A | Y | A | K | L | Y | G | - | - | - | - | - | - | - | - | G | Y | E | N | L | D | G | - | - | - | - | - | - | - | - | - | - | - | - | - | - | - | - | - | - | - | - | - | - | - | - | - | -123 |
| Q8IAA8_CAEEL | - | - | - | - | - | E | F | W | S | A | L | V | E | K | A | Y | A | K | L | H | G | - | - | - | - | - | - | - | - | G | Y | E | N | L | D | G | - | - | - | - | - | - | - | - | - | - | - | - | - | - | - | - | - | - | - | - | - | - | - | - | - | -123 |
| CAN_CAEEL | - | - | - | - | - | E | F | W | S | A | L | L | E | K | A | Y | A | K | L | F | G | - | - | - | - | - | - | - | - | S | Y | E | A | L | K | G | - | - | - | - | - | - | - | - | - | - | - | - | - | - | - | - | - | - | - | - | - | - | - | - | - | -123 |
| Q9U2B2_CAEEL | - | - | - | - | - | E | F | W | G | P | L | L | E | K | A | Y | A | K | L | L | G | - | - | - | - | - | - | - | - | T | Y | E | H | L | N | D | - | - | - | - | - | - | - | - | - | - | - | - | - | - | - | - | - | - | - | - | - | - | - | - | - | -123 |
| Q9N4B2_CAEEL | - | - | - | - | - | E | C | W | G | P | L | L | E | K | A | Y | A | K | L | Y | G | - | - | - | - | - | - | - | - | T | Y | E | H | L | D | G | - | - | - | - | - | - | - | - | - | - | - | - | - | - | - | - | - | - | - | - | - | - | - | - | - | -123 |
| C3Y6P6_BRAFL | - | - | - | - | - | E | F | W | S | A | L | L | E | K | A | Y | A | K | V | H | G | - | - | - | - | - | - | - | - | S | Y | E | A | L | E | G | - | - | - | - | - | - | - | - | - | - | - | - | - | - | - | - | - | - | - | - | - | - | - | - | - | -129 |
| C3Y6N1_BRAFL | - | - | - | - | - | E | F | W | S | A | L | L | E | K | A | Y | A | K | L | H | G | - | - | - | - | - | - | - | - | S | Y | E | S | L | E | G | - | - | - | - | - | - | - | - | - | - | - | - | - | - | - | - | - | - | - | - | - | - | - | - | - | -130 |
| CANA_DROME | - | - | - | - | - | E | F | W | S | A | L | L | E | K | A | Y | A | K | L | H | G | - | - | - | - | - | - | - | - | S | Y | E | A | L | K | G | - | - | - | - | - | - | - | - | - | - | - | - | - | - | - | - | - | - | - | - | - | - | - | - | - | -123 |
| CANB_DROME | - | - | - | - | - | E | F | W | S | A | L | L | E | K | A | Y | A | K | L | H | G | - | - | - | - | - | - | - | - | S | Y | E | A | L | K | G | - | - | - | - | - | - | - | - | - | - | - | - | - | - | - | - | - | - | - | - | - | - | - | - | - | -123 |
| X1Y3H71_ANODA | - | - | - | - | - | E | F | W | S | A | L | L | E | K | A | Y | A | K | L | H | G | - | - | - | - | - | - | - | - | S | Y | E | S | L | R | G | - | - | - | - | - | - | - | - | - | - | - | - | - | - | - | - | - | - | - | - | - | - | - | - | - | -123 |
| Q5IW12_GECLA | - | - | - | - | - | E | F | W | S | A | L | A | E | K | A | Y | A | K | L | H | G | - | - | - | - | - | - | - | - | S | Y | E | A | L | K | G | - | - | - | - | - | - | - | - | - | - | - | - | - | - | - | - | - | - | - | - | - | - | - | - | - | -123 |
| K1Q056_CRAGI | - | - | - | - | - | E | F | W | S | A | L | L | E | K | A | Y | A | K | L | E | G | - | - | - | - | - | - | - | - | S | Y | E | S | L | K | G | - | - | - | - | - | - | - | - | - | - | - | - | - | - | - | - | - | - | - | - | - | - | - | - | - | -123 |
| G4V6C0_SCHMA | - | - | - | - | - | E | F | W | S | A | L | L | E | K | A | Y | A | K | L | V | S | - | - | - | - | - | - | - | - | S | Y | D | A | L | R | G | - | - | - | - | - | - | - | - | - | - | - | - | - | - | - | - | - | - | - | - | - | - | - | - | - | -127 |
| R7ULX9_CAPTE | - | - | - | - | - | E | F | W | S | A | M | L | E | K | A | Y | A | K | L | C | G | - | - | - | - | - | - | - | - | C | Y | E | S | L | S | G | - | - | - | - | - | - | - | - | - | - | - | - | - | - | - | - | - | - | - | - | - | - | - | - | - | -123 |
| W4Z5Q7_STRPU | - | - | - | - | - | E | F | W | S | A | L | L | E | K | A | Y | A | K | L | H | G | - | - | - | - | - | - | - | - | S | Y | E | C | L | K | G | - | - | - | - | - | - | - | - | - | - | - | - | - | - | - | - | - | - | - | - | - | - | - | - | - | -120 |
| R7VIB6_CAPTE | - | - | - | - | - | E | F | W | A | A | L | L | E | K | A | Y | A | K | L | C | A | - | - | - | - | - | - | - | - | S | Y | E | A | L | S | G | - | - | - | - | - | - | - | - | - | - | - | - | - | - | - | - | - | - | - | - | - | - | - | - | - | -126 |
| T2MJ45_HYDVU | - | - | - | - | - | E | F | W | S | A | L | L | E | K | A | Y | A | K | L | C | G | - | - | - | - | - | - | - | - | S | Y | E | S | L | K | G | - | - | - | - | - | - | - | - | - | - | - | - | - | - | - | - | - | - | - | - | - | - | - | - | - | -123 |
| R7UAB6_CAPTE | - | - | - | - | - | E | F | W | S | P | L | L | E | K | A | Y | A | K | L | C | G | - | - | - | - | - | - | - | - | S | Y | E | A | L | K | G | - | - | - | - | - | - | - | - | - | - | - | - | - | - | - | - | - | - | - | - | - | - | - | - | - | -123 |
| C3ZKN8_BRAFL | - | - | - | - | - | E | Y | W | S | A | L | L | E | K | A | Y | A | K | L | Y | G | - | - | - | - | - | - | - | - | S | Y | E | A | L | K | G | - | - | - | - | - | - | - | - | - | - | - | - | - | - | - | - | - | - | - | - | - | - | - | - | - | -124 |
| B3RJS5_TRIAD | - | - | - | - | - | E | Y | W | S | A | L | L | E | K | A | Y | A | K | L | A | G | - | - | - | - | - | - | - | - | S | Y | E | A | L | K | G | - | - | - | - | - | - | - | - | - | - | - | - | - | - | - | - | - | - | - | - | - | - | - | - | - | -123 |
| H2YVF0_CIOSA | - | - | - | - | - | E | F | W | S | A | L | L | E | K | A | Y | A | K | L | N | G | - | - | - | - | - | - | - | - | S | Y | E | A | L | K | G | - | - | - | - | - | - | - | - | - | - | - | - | - | - | - | - | - | - | - | - | - | - | - | - | - | -123 |
| G4VAG2_SCHMA | - | - | - | - | - | E | F | W | S | A | L | L | E | K | A | Y | A | K | L | N | G | - | - | - | - | - | - | - | - | C | Y | A | H | L | S | G | - | - | - | - | - | - | - | - | - | - | - | - | - | - | - | - | - | - | - | - | - | - | - | - | - | -126 |
| Smp_157500 | - | - | - | - | - | E | F | W | S | C | L | L | E | K | A | Y | A | K | L | C | G | - | - | - | - | - | - | - | - | S | Y | A | H | L | T | G | - | - | - | - | - | - | - | - | - | - | - | - | - | - | - | - | - | - | - | - | - | - | - | - | - | -126 |
| Q7SYA9_DANRE | - | - | - | - | - | E | F | W | S | A | L | V | E | K | A | Y | A | K | L | N | G | - | - | - | - | - | - | - | - | S | Y | E | A | L | S | G | - | - | - | - | - | - | - | - | - | - | - | - | - | - | - | - | - | - | - | - | - | - | - | - | - | -123 |
| A5PMP1_DANRE | - | - | - | - | - | E | F | W | S | A | L | L | E | K | A | Y | A | K | L | N | G | - | - | - | - | - | - | - | - | S | Y | E | A | L | S | G | - | - | - | - | - | - | - | - | - | - | - | - | - | - | - | - | - | - | - | - | - | - | - | - | - | -123 |
| CANX_CHICK | - | - | - | - | - | E | F | W | S | A | L | L | E | K | A | Y | A | K | L | N | G | - | - | - | - | - | - | - | - | C | Y | E | S | L | S | G | - | - | - | - | - | - | - | - | - | - | - | - | - | - | - | - | - | - | - | - | - | - | - | - | - | -123 |
| CAN1_HUMAN | - | - | - | - | - | E | F | W | S | A | L | L | E | K | A | Y | A | K | V | N | G | - | - | - | - | - | - | - | - | S | Y | E | A | L | S | G | - | - | - | - | - | - | - | - | - | - | - | - | - | - | - | - | - | - | - | - | - | - | - | - | - | -123 |
| CAN1_MOUSE | - | - | - | - | - | E | F | W | S | A | L | L | E | K | A | Y | A | K | V | N | G | - | - | - | - | - | - | - | - | S | Y | E | A | L | S | G | - | - | - | - | - | - | - | - | - | - | - | - | - | - | - | - | - | - | - | - | - | - | - | - | - | -123 |
| O42133_CHICK | - | - | - | - | - | E | F | W | S | A | L | L | E | K | A | Y | A | K | V | N | G | - | - | - | - | - | - | - | - | C | Y | E | A | L | S | G | - | - | - | - | - | - | - | - | - | - | - | - | - | - | - | - | - | - | - | - | - | - | - | - | - | -123 |
| Q7ZUR1_DANRE | - | - | - | - | - | E | F | W | S | A | L | L | E | K | A | Y | A | K | L | N | G | - | - | - | - | - | - | - | - | C | Y | E | A | L | S | G | - | - | - | - | - | - | - | - | - | - | - | - | - | - | - | - | - | - | - | - | - | - | - | - | - | -123 |
| E9QFN0_DANRE | - | - | - | - | - | E | F | W | S | A | L | V | E | K | A | Y | A | K | L | N | G | - | - | - | - | - | - | - | - | C | Y | E | A | L | S | G | - | - | - | - | - | - | - | - | - | - | - | - | - | - | - | - | - | - | - | - | - | - | - | - | - | -123 |
| CAN2_HUMAN | - | - | - | - | - | E | F | W | S | A | L | L | E | K | A | Y | A | K | I | N | G | - | - | - | - | - | - | - | - | C | Y | E | A | L | S | G | - | - | - | - | - | - | - | - | - | - | - | - | - | - | - | - | - | - | - | - | - | - | - | - | - | -123 |
| CAN2_MOUSE | - | - | - | - | - | E | F | W | S | A | L | L | E | K | A | Y | A | K | I | N | G | - | - | - | - | - | - | - | - | C | Y | E | A | L | S | G | - | - | - | - | - | - | - | - | - | - | - | - | - | - | - | - | - | - | - | - | - | - | - | - | - | -123 |
| CAN2_CHICK | - | - | - | - | - | E | F | W | S | A | L | L | E | K | A | Y | A | K | L | N | G | - | - | - | - | - | - | - | - | S | Y | E | A | L | S | G | - | - | - | - | - | - | - | - | - | - | - | - | - | - | - | - | - | - | - | - | - | - | - | - | - | -123 |
| Q6DFZ8_DANRE | - | - | - | - | - | E | F | W | S | A | L | L | E | K | A | Y | A | K | L | N | G | - | - | - | - | - | - | - | - | C | Y | E | A | L | S | G | - | - | - | - | - | - | - | - | - | - | - | - | - | - | - | - | - | - | - | - | - | - | - | - | - | -123 |
| Q504F5_DANRE | - | - | - | - | - | E | F | W | S | A | L | L | E | K | A | Y | A | K | V | N | G | - | - | - | - | - | - | - | - | C | Y | E | A | L | S | G | - | - | - | - | - | - | - | - | - | - | - | - | - | - | - | - | - | - | - | - | - | - | - | - | - | -123 |
| Q5BLH5_DANRE | - | - | - | - | - | E | F | W | S | A | L | L | E | K | A | Y | A | K | V | N | G | - | - | - | - | - | - | - | - | C | Y | E | A | L | S | G | - | - | - | - | - | - | - | - | - | - | - | - | - | - | - | - | - | - | - | - | - | - | - | - | - | -123 |
| Q4VBH3_DANRE | - | - | - | - | - | E | F | W | S | A | L | L | E | K | A | Y | A | K | V | N | G | - | - | - | - | - | - | - | - | S | Y | E | A | L | T | G | - | - | - | - | - | - | - | - | - | - | - | - | - | - | - | - | - | - | - | - | - | - | - | - | - | -123 |
| CAN8_RAT | - | - | - | - | - | E | F | W | S | A | L | L | E | K | A | Y | A | K | L | N | G | - | - | - | - | - | - | - | - | S | Y | E | A | L | V | G | - | - | - | - | - | - | - | - | - | - | - | - | - | - | - | - | - | - | - | - | - | - | - | - | - | -123 |
| CAN8_MOUSE | - | - | - | - | - | E | F | W | S | A | L | L | E | K | A | Y | A | K | L | N | G | - | - | - | - | - | - | - | - | S | Y | E | A | L | A | G | - | - | - | - | - | - | - | - | - | - | - | - | - | - | - | - | - | - | - | - | - | - | - | - | - | -123 |
| CAN8_HUMAN | - | - | - | - | - | E | F | W | S | A | L | L | E | K | A | Y | A | K | L | N | G | - | - | - | - | - | - | - | - | C | Y | E | A | L | A | G | - | - | - | - | - | - | - | - | - | - | - | - | - | - | - | - | - | - | - | - | - | - | - | - | - | -123 |
| XP_426117 | - | - | - | - | - | E | F | W | S | A | L | L | E | K | A | Y | A | K | L | N | G | - | - | - | - | - | - | - | - | S | Y | E | A | L | A | G | - | - | - | - | - | - | - | - | - | - | - | - | - | - | - | - | - | - | - | - | - | - | - | - | - | -123 |
| CAN9_MOUSE | - | - | - | - | - | E | F | W | S | A | L | L | E | K | A | Y | A | K | L | N | G | - | - | - | - | - | - | - | - | S | Y | E | A | L | E | G | - | - | - | - | - | - | - | - | - | - | - | - | - | - | - | - | - | - | - | - | - | - | - | - | - | -125 |
| CAN9_HUMAN | - | - | - | - | - | E | F | W | S | A | L | L | E | K | A | Y | A | K | L | N | G | - | - | - | - | - | - | - | - | S | Y | E | A | L | K | G | - | - | - | - | - | - | - | - | - | - | - | - | - | - | - | - | - | - | - | - | - | - | - | - | - | -123 |
| E1C6M3_CHICK | - | - | - | - | - | E | F | W | S | A | L | L | E | K | A | Y | A | K | L | N | G | - | - | - | - | - | - | - | - | S | Y | E | A | L | K | G | - | - | - | - | - | - | - | - | - | - | - | - | - | - | - | - | - | - | - | - | - | - | - | - | - | -123 |
| Q6DG44_DANRE | - | - | - | - | - | E | F | W | S | A | L | L | E | K | A | Y | A | K | L | N | G | - | - | - | - | - | - | - | - | S | Y | E | A | L | K | G | - | - | - | - | - | - | - | - | - | - | - | - | - | - | - | - | - | - | - | - | - | - | - | - | - | -123 |
| CAN3_HUMAN | - | - | - | - | - | E | F | W | S | A | L | L | E | K | A | Y | A | K | L | H | G | - | - | - | - | - | - | - | - | S | Y | E | A | L | K | G | - | - | - | - | - | - | - | - | - | - | - | - | - | - | - | - | - | - | - | - | - | - | - | - | - | -123 |
| CAN3_MOUSE | - | - | - | - | - | E | F | W | S | A | L | L | E | K | A | Y | A | K | L | H | G | - | - | - | - | - | - | - | - | S | Y | E | A | L | K | G | - | - | - | - | - | - | - | - | - | - | - | - | - | - | - | - | - | - | - | - | - | - | - | - | - | -123 |
| CAN3_CHICK | - | - | - | - | - | E | F | W | S | A | L | L | E | K | A | Y | A | K | L | H | G | - | - | - | - | - | - | - | - | S | Y | E | A | L | K | G | - | - | - | - | - | - | - | - | - | - | - | - | - | - | - | - | - | - | - | - | - | - | - | - | - | -123 |
| Q66HU4_DANRE | - | - | - | - | - | E | F | W | S | A | L | L | E | K | A | Y | A | K | L | H | G | - | - | - | - | - | - | - | - | S | Y | E | A | L | K | G | - | - | - | - | - | - | - | - | - | - | - | - | - | - | - | - | - | - | - | - | - | - | - | - | - | -123 |
| F1R632_DANRE | - | - | - | - | - | E | F | W | S | A | L | M | E | K | A | Y | A | K | L | H | G | - | - | - | - | - | - | - | - | S | Y | E | A | L | K | G | - | - | - | - | - | - | - | - | - | - | - | - | - | - | - | - | - | - | - | - | - | - | - | - | - | -123 |
| CAN11_HUMAN | - | - | - | - | - | E | F | W | S | A | L | L | E | K | A | Y | A | K | L | S | G | - | - | - | - | - | - | - | - | S | Y | E | A | L | S | G | - | - | - | - | - | - | - | - | - | - | - | - | - | - | - | - | - | - | - | - | - | - | - | - | - | -123 |
| CAN11_MOUSE | - | - | - | - | - | D | F | W | S | A | L | L | E | K | A | Y | A | K | L | I | G | - | - | - | - | - | - | - | - | S | Y | G | A | L | S | G | - | - | - | - | - | - | - | - | - | - | - | - | - | - | - | - | - | - | - | - | - | - | - | - | - | -123 |
| CAN12_MOUSE | - | - | - | - | - | E | F | W | A | P | L | L | E | K | A | Y | A | K | L | H | G | - | - | - | - | - | - | - | - | S | Y | E | V | M | R | G | - | - | - | - | - | - | - | - | - | - | - | - | - | - | - | - | - | - | - | - | - | - | - | - | - | -123 |
| CAN12_HUMAN | - | - | - | - | - | E | F | W | A | P | L | L | E | K | A | Y | A | K | L | H | G | - | - | - | - | - | - | - | - | S | Y | E | V | M | R | G | - | - | - | - | - | - | - | - | - | - | - | - | - | - | - | - | - | - | - | - | - | - | - | - | - | -123 |
| ENSSARP00000008135 | - | - | - | - | - | E | F | W | A | P | L | X | X | X | X | X | X | X | X | X | X | - | - | - | - | - | - | - | - | X | X | X | X | X | X | X | - | - | - | - | - | - | - | - | - | - | - | - | - | - | - | - | - | - | - | - | - | - | - | - | - | -123 |
| ENSSARP00000010553 | - | - | - | - | - | X | X | X | X | X | X | X | X | X | X | X | X | X | X | X | X | - | - | - | - | - | - | - | - | X | X | X | X | X | X | X | - | - | - | - | - | - | - | - | - | - | - | - | - | - | - | - | - | - | - | - | - | - | - | - | - | -122 |
| A7E2G3_DANRE | - | - | - | - | - | E | F | W | S | A | L | V | E | K | A | Y | A | K | L | I | G | - | - | - | - | - | - | - | - | S | Y | G | S | L | K | G | - | - | - | - | - | - | - | - | - | - | - | - | - | - | - | - | - | - | - | - | - | - | - | - | - | -123 |
| Q6NW69_DANRE | - | - | - | - | - | E | F | W | S | A | L | L | E | K | A | Y | A | K | L | K | G | - | - | - | - | - | - | - | - | G | Y | S | A | L | N | M | - | - | - | - | - | - | - | - | - | - | - | - | - | - | - | - | - | - | - | - | - | - | - | - | - | -123 |
| CAN13_HUMAN | - | - | - | - | - | E | F | W | P | C | L | L | E | K | A | Y | A | K | L | L | G | - | - | - | - | - | - | - | - | S | Y | S | D | L | H | Y | - | - | - | - | - | - | - | - | - | - | - | - | - | - | - | - | - | - | - | - | - | - | - | - | - | -124 |
| CAN13_MOUSE | - | - | - | - | - | E | F | W | P | C | L | M | E | K | A | Y | A | K | L | L | G | - | - | - | - | - | - | - | - | S | Y | S | Q | L | H | Y | - | - | - | - | - | - | - | - | - | - | - | - | - | - | - | - | - | - | - | - | - | - | - | - | - | -126 |
| E1BRR5_CHICK | - | - | - | - | - | E | F | W | P | S | L | L | E | K | A | Y | A | K | L | Q | G | - | - | - | - | - | - | - | - | S | Y | Q | N | L | H | G | - | - | - | - | - | - | - | - | - | - | - | - | - | - | - | - | - | - | - | - | - | - | - | - | - | -123 |
| E7F0H3_DANRE | - | - | - | - | - | E | F | W | P | A | L | L | E | K | A | Y | A | K | V | C | G | - | - | - | - | - | - | - | - | S | Y | A | D | L | N | A | - | - | - | - | - | - | - | - | - | - | - | - | - | - | - | - | - | - | - | - | - | - | - | - | - | -124 |
| Q1RLS9_DANRE | - | - | - | - | - | E | F | W | P | A | L | L | E | K | A | Y | A | K | V | C | G | - | - | - | - | - | - | - | - | S | Y | A | D | M | D | A | - | - | - | - | - | - | - | - | - | - | - | - | - | - | - | - | - | - | - | - | - | - | - | - | - | -123 |
| F1Q9Z1_DANRE | - | - | - | - | - | E | F | W | P | A | L | L | E | K | A | Y | A | K | V | C | G | - | - | - | - | - | - | - | - | S | Y | A | D | M | H | T | - | - | - | - | - | - | - | - | - | - | - | - | - | - | - | - | - | - | - | - | - | - | - | - | - | -124 |
| A4QNV6_DANRE | - | - | - | - | - | E | F | W | P | A | L | L | E | K | A | Y | A | K | V | C | G | - | - | - | - | - | - | - | - | S | Y | S | D | M | D | A | - | - | - | - | - | - | - | - | - | - | - | - | - | - | - | - | - | - | - | - | - | - | - | - | - | -124 |
| D6W573_HUMAN | - | - | - | - | - | L | F | W | G | A | L | L | E | K | A | Y | A | K | L | S | G | - | - | - | - | - | - | - | - | S | Y | E | D | L | Q | S | - | - | - | - | - | - | - | - | - | - | - | - | - | - | - | - | - | - | - | - | - | - | - | - | - | -124 |
| R7UBC6_CAPTE | - | - | - | - | - | E | F | W | A | A | L | L | E | K | A | Y | A | K | L | L | G | - | - | - | - | - | - | - | - | S | Y | E | A | L | K | S | - | - | - | - | - | - | - | - | - | - | - | - | - | - | - | - | - | - | - | - | - | - | - | - | - | -128 |
| K1RCZ2_CRAGI | - | - | - | - | - | E | F | W | A | A | L | M | E | K | A | Y | A | K | L | Y | G | - | - | - | - | - | - | - | - | S | Y | E | A | L | K | G | - | - | - | - | - | - | - | - | - | - | - | - | - | - | - | - | - | - | - | - | - | - | - | - | - | -130 |
| Q5IW13_GECLA | - | - | - | - | - | E | F | W | C | A | L | L | E | K | A | Y | A | K | L | Y | G | - | - | - | - | - | - | - | - | G | Y | E | A | L | R | G | - | - | - | - | - | - | - | - | - | - | - | - | - | - | - | - | - | - | - | - | - | - | - | - | - | -123 |
| R7UUX7_CAPTE | - | - | - | - | - | E | F | W | A | A | L | L | E | K | A | Y | A | K | L | Y | G | - | - | - | - | - | - | - | - | C | Y | E | N | I | D | G | - | - | - | - | - | - | - | - | - | - | - | - | - | - | - | - | - | - | - | - | - | - | - | - | - | -126 |
| K1Q505_CRAGI | - | - | - | - | - | E | F | W | A | A | L | L | E | K | A | Y | A | K | L | R | G | - | - | - | - | - | - | - | - | C | Y | E | G | V | D | G | - | - | - | - | - | - | - | - | - | - | - | - | - | - | - | - | - | - | - | - | - | - | - | - | - | -124 |
| R7UD02_CAPTE | - | - | - | - | - | E | F | W | S | A | L | L | E | K | A | Y | A | K | I | F | G | - | - | - | - | - | - | - | - | S | Y | Q | A | L | E | T | - | - | - | - | - | - | - | - | - | - | - | - | - | - | - | - | - | - | - | - | - | - | - | - | - | -124 |
| CAN6_HUMAN | - | - | - | - | - | E | F | W | N | A | L | L | E | K | A | Y | A | K | L | L | G | - | - | - | - | - | - | - | - | C | Y | E | A | L | D | G | - | - | - | - | - | - | - | - | - | - | - | - | - | - | - | - | - | - | - | - | - | - | - | - | - | -129 |
| CAN6_MOUSE | - | - | - | - | - | E | F | W | N | A | L | L | E | K | A | Y | A | K | L | L | G | - | - | - | - | - | - | - | - | C | Y | E | A | L | D | G | - | - | - | - | - | - | - | - | - | - | - | - | - | - | - | - | - | - | - | - | - | - | - | - | - | -129 |
| E1C312_CHICK | - | - | - | - | - | E | F | W | S | A | L | L | E | K | A | Y | A | K | L | A | G | - | - | - | - | - | - | - | - | S | Y | E | A | L | D | G | - | - | - | - | - | - | - | - | - | - | - | - | - | - | - | - | - | - | - | - | - | - | - | - | - | -129 |
| E7FDN3_DANRE | - | - | - | - | - | E | F | W | S | A | L | L | E | K | A | Y | A | K | L | N | G | - | - | - | - | - | - | - | - | C | Y | E | A | L | E | G | - | - | - | - | - | - | - | - | - | - | - | - | - | - | - | - | - | - | - | - | - | - | - | - | - | -130 |
| CAN5_HUMAN | - | - | - | - | - | E | F | W | C | A | L | V | E | K | A | Y | A | K | L | A | G | - | - | - | - | - | - | - | - | C | Y | Q | A | L | D | G | - | - | - | - | - | - | - | - | - | - | - | - | - | - | - | - | - | - | - | - | - | - | - | - | - | -125 |
| CAN5_MOUSE | - | - | - | - | - | E | F | W | C | A | L | V | E | K | A | Y | A | K | L | A | G | - | - | - | - | - | - | - | - | C | Y | Q | A | L | D | G | - | - | - | - | - | - | - | - | - | - | - | - | - | - | - | - | - | - | - | - | - | - | - | - | - | -129 |
| E1C292_CHICK | - | - | - | - | - | E | F | W | C | A | L | V | E | K | A | Y | A | K | L | S | G | - | - | - | - | - | - | - | - | C | Y | E | A | L | D | G | - | - | - | - | - | - | - | - | - | - | - | - | - | - | - | - | - | - | - | - | - | - | - | - | - | -129 |
| A0JMG3_DANRE | - | - | - | - | - | E | F | W | S | A | L | V | E | K | A | Y | A | K | M | C | G | - | - | - | - | - | - | - | - | C | Y | E | A | L | D | G | - | - | - | - | - | - | - | - | - | - | - | - | - | - | - | - | - | - | - | - | - | - | - | - | - | -129 |
| E7F7F1_DANRE | - | - | - | - | - | E | F | W | S | A | L | V | E | K | A | Y | A | K | V | Y | G | - | - | - | - | - | - | - | - | C | Y | E | A | L | D | G | - | - | - | - | - | - | - | - | - | - | - | - | - | - | - | - | - | - | - | - | - | - | - | - | - | -129 |
| XP_006824412 | - | - | - | - | - | E | F | W | S | A | L | L | E | K | A | Y | A | K | I | S | G | - | - | - | - | - | - | - | - | C | Y | E | N | L | D | G | - | - | - | - | - | - | - | - | - | - | - | - | - | - | - | - | - | - | - | - | - | - | - | - | - | -123 |
| W4YJN2_STRPU | - | - | - | - | - | E | F | W | S | A | L | L | E | K | A | Y | A | K | I | S | G | - | - | - | - | - | - | - | - | C | Y | E | N | L | D | G | - | - | - | - | - | - | - | - | - | - | - | - | - | - | - | - | - | - | - | - | - | - | - | - | - | -129 |
| C3YJZ3_BRAFL | - | - | - | - | - | E | L | W | S | S | L | L | E | K | A | Y | A | K | L | A | G | - | - | - | - | - | - | - | - | C | Y | E | A | L | Q | G | - | - | - | - | - | - | - | - | - | - | - | - | - | - | - | - | - | - | - | - | - | - | - | - | - | -129 |
| T2MIT4_HYDVU | - | - | - | - | - | E | F | W | S | A | L | L | E | K | A | Y | A | K | L | A | G | - | - | - | - | - | - | - | - | S | Y | E | A | L | E | A | - | - | - | - | - | - | - | - | - | - | - | - | - | - | - | - | - | - | - | - | - | - | - | - | - | -129 |
| XP_002154722 | - | - | - | - | - | - | - | - | - | - | - | - | - | - | - | - | - | - | L | S | G | - | - | - | - | - | - | - | - | S | Y | E | A | L | E | S | - | - | - | - | - | - | - | - | - | - | - | - | - | - | - | - | - | - | - | - | - | - | - | - | - | -93 |
| R7VLA7_CAPTE | - | - | - | - | - | E | F | W | S | A | L | L | E | K | A | Y | A | K | L | Y | G | - | - | - | - | - | - | - | - | S | Y | E | S | L | D | G | - | - | - | - | - | - | - | - | - | - | - | - | - | - | - | - | - | - | - | - | - | - | - | - | - | -129 |
| K1R1C6_CRAGI | - | - | - | - | - | E | F | W | S | A | L | L | E | K | A | Y | A | K | L | F | G | - | - | - | - | - | - | - | - | C | Y | E | A | L | D | G | - | - | - | - | - | - | - | - | - | - | - | - | - | - | - | - | - | - | - | - | - | - | - | - | - | -129 |
| B3RIE5_TRIAD | - | - | - | - | - | E | F | W | S | A | L | L | E | K | A | Y | A | K | L | A | G | - | - | - | - | - | - | - | - | S | Y | E | S | L | E | A | - | - | - | - | - | - | - | - | - | - | - | - | - | - | - | - | - | - | - | - | - | - | - | - | - | -128 |
| K1PW75_CRAGI | - | - | - | - | - | E | F | W | S | A | L | L | E | K | A | Y | A | K | I | F | G | - | - | - | - | - | - | - | - | D | Y | E | S | L | K | G | - | - | - | - | - | - | - | - | - | - | - | - | - | - | - | - | - | - | - | - | - | - | - | - | - | -127 |
| K1RSB8_CRAGI | - | - | - | - | - | E | F | W | S | A | L | L | E | K | A | Y | A | K | L | F | G | - | - | - | - | - | - | - | - | D | Y | E | A | L | V | A | - | - | - | - | - | - | - | - | - | - | - | - | - | - | - | - | - | - | - | - | - | - | - | - | - | -127 |
| K1RWI3_CRAGI | - | - | - | - | - | E | F | W | S | A | L | L | E | K | A | Y | A | K | I | F | G | - | - | - | - | - | - | - | - | S | Y | E | A | L | E | G | - | - | - | - | - | - | - | - | - | - | - | - | - | - | - | - | - | - | - | - | - | - | - | - | - | -129 |
| R7VGC2_CAPTE | - | - | - | - | - | E | F | W | S | A | L | L | E | K | A | Y | A | K | L | Y | G | - | - | - | - | - | - | - | - | D | Y | E | S | L | A | A | - | - | - | - | - | - | - | - | - | - | - | - | - | - | - | - | - | - | - | - | - | - | - | - | - | -128 |
| K1QU26_CRAGI | - | - | - | - | - | E | F | W | S | A | L | L | E | K | A | Y | A | K | L | Y | G | - | - | - | - | - | - | - | - | D | Y | E | S | L | N | D | - | - | - | - | - | - | - | - | - | - | - | - | - | - | - | - | - | - | - | - | - | - | - | - | - | -130 |
| CAN5_CAEEL | - | - | - | - | - | E | F | W | S | A | L | L | E | K | A | F | A | K | L | Y | G | - | - | - | - | - | - | - | - | C | Y | E | N | L | V | G | - | - | - | - | - | - | - | - | - | - | - | - | - | - | - | - | - | - | - | - | - | - | - | - | - | -127 |
| I1FGV4_AMPQE | - | - | - | - | - | E | F | W | S | A | L | L | E | K | A | Y | A | K | L | Y | G | - | - | - | - | - | - | - | - | G | F | E | A | L | K | G | - | - | - | - | - | - | - | - | - | - | - | - | - | - | - | - | - | - | - | - | - | - | - | - | - | -123 |
| K1PHX3_CRAGI | - | - | - | - | - | E | F | W | P | A | L | F | E | K | A | Y | A | K | C | Y | G | - | - | - | - | - | - | - | - | S | Y | E | N | T | S | G | - | - | - | - | - | - | - | - | - | - | - | - | - | - | - | - | - | - | - | - | - | - | - | - | - | -123 |
| B3RNL8_TRIAD | - | - | - | - | - | E | F | W | G | C | F | I | E | K | A | Y | A | K | V | N | N | - | - | - | - | - | - | - | - | S | Y | E | A | L | V | G | - | - | - | - | - | - | - | - | - | - | - | - | - | - | - | - | - | - | - | - | - | - | - | - | - | -125 |
| X1Y3H7_ANODA | - | - | - | - | - | E | F | W | S | A | L | L | E | K | A | Y | A | K | L | H | G | - | - | - | - | - | - | - | - | S | Y | H | A | L | A | G | - | - | - | - | - | - | - | - | - | - | - | - | - | - | - | - | - | - | - | - | - | - | - | - | - | -127 |
| R7TWH9_CAPTE | - | - | - | - | - | E | F | W | V | A | L | V | E | K | A | Y | A | K | L | H | G | - | - | - | - | - | - | - | - | S | Y | E | A | I | E | G | - | - | - | - | - | - | - | - | - | - | - | - | - | - | - | - | - | - | - | - | - | - | - | - | - | -127 |
| K1R5E8_CRAGI | - | - | - | - | - | E | F | W | V | A | L | I | E | K | A | Y | A | K | L | H | G | - | - | - | - | - | - | - | - | S | Y | E | A | I | E | G | - | - | - | - | - | - | - | - | - | - | - | - | - | - | - | - | - | - | - | - | - | - | - | - | - | -127 |
| XP_006811804 | - | - | - | - | - | E | F | W | V | S | L | L | E | K | A | Y | A | K | L | H | G | - | - | - | - | - | - | - | - | S | Y | E | G | L | A | G | - | - | - | - | - | - | - | - | - | - | - | - | - | - | - | - | - | - | - | - | - | - | - | - | - | -125 |
| C3YB23_BRAFL | - | - | - | - | - | E | F | W | S | A | L | V | E | K | A | Y | A | K | L | R | G | - | - | - | - | - | - | - | - | S | Y | E | A | L | N | G | - | - | - | - | - | - | - | - | - | - | - | - | - | - | - | - | - | - | - | - | - | - | - | - | - | -132 |
| H2ZIF1_CIOSA | - | - | - | - | - | E | F | W | L | P | L | M | E | K | A | Y | A | K | V | H | G | - | - | - | - | - | - | - | - | S | Y | Q | A | T | E | G | - | - | - | - | - | - | - | - | - | - | - | - | - | - | - | - | - | - | - | - | - | - | - | - | - | -124 |
| W4YV93_STRPU | - | - | - | - | - | E | F | W | P | S | L | V | E | K | A | Y | A | K | L | H | G | - | - | - | - | - | - | - | - | S | Y | S | G | L | S | G | - | - | - | - | - | - | - | - | - | - | - | - | - | - | - | - | - | - | - | - | - | - | - | - | - | -124 |
| CAN10_HUMAN | - | - | - | - | - | V | F | W | L | P | L | L | E | K | V | Y | A | K | V | H | G | - | - | - | - | - | - | - | - | S | Y | E | H | L | W | A | - | - | - | - | - | - | - | - | - | - | - | - | - | - | - | - | - | - | - | - | - | - | - | - | - | -125 |
| CAN10_MOUSE | - | - | - | - | - | V | F | W | L | P | L | L | E | K | A | Y | A | K | V | H | G | - | - | - | - | - | - | - | - | S | Y | E | H | L | W | A | - | - | - | - | - | - | - | - | - | - | - | - | - | - | - | - | - | - | - | - | - | - | - | - | - | -125 |
| XP_004943443 | - | - | - | - | - | L | F | W | L | P | L | L | E | K | A | Y | A | K | V | H | G | - | - | - | - | - | - | - | - | S | Y | E | Q | L | W | A | - | - | - | - | - | - | - | - | - | - | - | - | - | - | - | - | - | - | - | - | - | - | - | - | - | -125 |
| F8W4K1_DANRE | - | - | - | - | - | A | F | W | V | A | L | L | E | K | A | Y | A | K | L | H | G | - | - | - | - | - | - | - | - | S | Y | E | H | L | W | A | - | - | - | - | - | - | - | - | - | - | - | - | - | - | - | - | - | - | - | - | - | - | - | - | - | -125 |
| CANC_DROME | - | - | - | - | - | C | F | W | A | A | L | L | E | K | A | I | A | K | L | H | G | - | - | - | - | - | - | - | - | S | Y | E | A | L | K | Y | - | - | - | - | - | - | - | - | - | - | - | - | - | - | - | - | - | - | - | - | - | - | - | - | - | -111 |
| C3ZKN9_BRAFL | - | - | - | - | - | H | F | W | A | S | L | L | E | K | A | Y | A | K | L | L | G | - | - | - | - | - | - | - | - | S | Y | A | A | L | R | E | - | - | - | - | - | - | - | - | - | - | - | - | - | - | - | - | - | - | - | - | - | - | - | - | - | -123 |
| G4V623_SCHMA | - | - | - | - | - | E | F | W | P | T | L | L | E | K | A | Y | A | K | L | N | G | - | - | - | - | - | - | - | - | S | Y | E | A | L | N | V | - | - | - | - | - | - | - | - | - | - | - | - | - | - | - | - | - | - | - | - | - | - | - | - | - | -142 |
| R7VK39_CAPTE | - | - | - | - | - | E | Y | W | P | A | L | L | H | K | A | Y | L | K | F | T | D | - | - | - | - | - | - | - | - | R | N | P | P | K | - | - | - | - | - | - | - | - | - | - | - | - | - | - | - | - | - | - | - | - | - | - | - | - | - | - | - | -124 |
| G4V693_SCHMA | - | - | - | - | - | E | F | W | P | A | L | L | E | K | A | Y | A | K | L | L | G | - | - | - | - | - | - | - | - | G | Y | D | K | L | E | Y | - | - | - | - | - | - | - | - | - | - | - | - | - | - | - | - | - | - | - | - | - | - | - | - | - | -128 |
| R7UPU6_CAPTE | - | - | - | - | - | E | V | W | V | S | L | M | E | K | A | F | A | R | F | N | G | - | - | - | - | - | - | - | - | G | Y | P | Q | V | Y | G | - | - | - | - | - | - | - | - | - | - | - | - | - | - | - | - | - | - | - | - | - | - | - | - | - | -129 |
| R7V817_CAPTE | - | - | - | - | - | E | V | W | V | S | L | I | E | K | A | F | A | R | F | N | G | - | - | - | - | - | - | - | - | G | Y | P | Q | V | Y | G | - | - | - | - | - | - | - | - | - | - | - | - | - | - | - | - | - | - | - | - | - | - | - | - | - | -126 |
| R7UNL8_CAPTE | - | - | - | - | - | E | M | W | V | S | L | M | E | K | A | F | A | R | F | K | G | - | - | - | - | - | - | - | - | G | Y | A | N | V | Y | G | - | - | - | - | - | - | - | - | - | - | - | - | - | - | - | - | - | - | - | - | - | - | - | - | - | -124 |
| K1PJ80_CRAGI | - | - | - | - | - | E | M | W | V | A | L | L | E | K | A | F | A | R | M | Y | G | - | - | - | - | - | - | - | - | S | Y | T | D | V | S | G | - | - | - | - | - | - | - | - | - | - | - | - | - | - | - | - | - | - | - | - | - | - | - | - | - | -127 |
| K1PCX8_CRAGI | - | - | - | - | - | E | M | W | V | S | L | L | E | K | A | F | A | R | M | Y | G | - | - | - | - | - | - | - | - | S | Y | T | E | V | S | G | - | - | - | - | - | - | - | - | - | - | - | - | - | - | - | - | - | - | - | - | - | - | - | - | - | -127 |
| CAND_DROME | - | - | K | R | K | Q | L | W | V | P | L | I | E | K | A | V | A | K | I | H | G | - | - | - | - | - | - | - | - | C | Y | E | A | L | V | S | - | - | - | - | - | - | - | - | - | - | - | - | - | - | - | - | - | - | - | - | - | - | - | - | - | -125 |
| XP_002735765 | - | - | K | R | R | Q | L | W | V | P | L | I | E | K | A | M | A | K | M | H | G | - | - | - | - | - | - | - | - | C | Y | E | A | L | V | S | - | - | - | - | - | - | - | - | - | - | - | - | - | - | - | - | - | - | - | - | - | - | - | - | - | -127 |
| CAN15_HUMAN | - | - | Q | R | K | Q | L | W | V | A | L | I | E | K | A | L | A | K | L | H | G | - | - | - | - | - | - | - | - | S | Y | F | A | L | Q | A | - | - | - | - | - | - | - | - | - | - | - | - | - | - | - | - | - | - | - | - | - | - | - | - | - | -127 |
| CAN15_MOUSE | - | - | Q | R | K | Q | L | W | V | A | L | I | E | K | A | L | A | K | L | H | G | - | - | - | - | - | - | - | - | S | Y | F | A | L | Q | A | - | - | - | - | - | - | - | - | - | - | - | - | - | - | - | - | - | - | - | - | - | - | - | - | - | -127 |
| E1BTA8_CHICK | - | - | Q | R | K | Q | L | W | V | A | L | I | E | K | A | L | A | K | L | H | G | - | - | - | - | - | - | - | - | S | Y | F | A | L | Q | A | - | - | - | - | - | - | - | - | - | - | - | - | - | - | - | - | - | - | - | - | - | - | - | - | - | -127 |
| E7FAX5_DANRE | - | - | Q | R | R | Q | L | W | V | A | L | I | E | K | A | L | A | K | L | H | G | - | - | - | - | - | - | - | - | S | Y | F | A | L | Q | A | - | - | - | - | - | - | - | - | - | - | - | - | - | - | - | - | - | - | - | - | - | - | - | - | - | -127 |
| C3XWK8_BRAFL | - | - | K | R | R | Q | L | W | V | P | L | I | E | K | A | L | A | K | L | H | G | - | - | - | - | - | - | - | - | C | Y | E | A | L | I | S | - | - | - | - | - | - | - | - | - | - | - | - | - | - | - | - | - | - | - | - | - | - | - | - | - | -126 |
| W4YMU4_STRPU | - | - | K | R | K | Q | L | W | V | P | L | I | E | K | A | L | A | K | M | S | G | - | - | - | - | - | - | - | - | C | Y | E | A | L | T | A | G | R | C | I | E | G | L | A | T | L | T | G | A | P | C | E | S | V | Q | L | H | A | S | G | K | -152 |
| K1PV74_CRAGI | - | - | K | R | K | Q | L | W | V | P | L | I | E | K | A | M | A | K | L | H | G | - | - | - | - | - | - | - | - | S | Y | E | S | L | V | A | - | - | - | - | - | - | - | - | - | - | - | - | - | - | - | - | - | - | - | - | - | - | - | - | - | -127 |
| R7TJF2_CAPTE | - | - | R | R | R | Q | L | W | V | P | L | I | E | K | A | L | A | K | L | H | G | - | - | - | - | - | - | - | - | C | Y | E | A | L | I | S | - | - | - | - | - | - | - | - | - | - | - | - | - | - | - | - | - | - | - | - | - | - | - | - | - | -127 |
| T2MI17_HYDVU | - | - | A | R | N | Q | L | W | V | P | I | I | E | K | A | V | A | K | S | V | G | - | - | - | - | - | - | - | - | C | Y | E | A | L | V | A | - | - | - | - | - | - | - | - | - | - | - | - | - | - | - | - | - | - | - | - | - | - | - | - | - | -124 |
| Q1RL55_CIOIN | - | - | R | G | K | Q | L | W | V | P | L | V | E | K | A | L | A | K | L | H | G | - | - | - | - | - | - | - | - | C | Y | E | A | L | Q | A | - | - | - | - | - | - | - | - | - | - | - | - | - | - | - | - | - | - | - | - | - | - | - | - | - | -114 |
| I1EA30_AMPQE | - | - | R | R | G | Q | L | W | V | P | L | I | E | K | A | M | A | K | M | Y | G | - | - | - | - | - | - | - | - | S | Y | E | S | L | D | G | - | - | - | - | - | - | - | - | - | - | - | - | - | - | - | - | - | - | - | - | - | - | - | - | - | -127 |
| G5EEZ6_CAEEL | - | - | R | R | N | Q | L | W | V | P | L | I | E | K | A | M | A | K | V | L | G | - | - | - | - | - | - | - | - | S | Y | S | K | L | H | G | - | - | - | - | - | - | - | - | - | - | - | - | - | - | - | - | - | - | - | - | - | - | - | - | - | -127 |
| O44903_CAEEL | - | - | R | R | N | Q | L | W | V | P | L | I | E | K | A | M | A | K | V | L | G | - | - | - | - | - | - | - | - | S | Y | S | K | L | H | G | - | - | - | - | - | - | - | - | - | - | - | - | - | - | - | - | - | - | - | - | - | - | - | - | - | -127 |
| O02260_CAEEL | - | - | S | G | Y | Q | I | W | A | A | L | I | E | K | A | L | V | K | E | C | G | - | - | - | - | - | - | - | - | N | Y | K | G | I | H | G | - | - | - | - | - | - | - | - | - | - | - | - | - | - | - | - | - | - | - | - | - | - | - | - | - | -124 |
| O02259_CAEEL | - | - | S | G | K | Q | I | W | A | A | L | I | E | K | A | L | A | K | E | R | G | - | - | - | - | - | - | - | - | N | Y | K | G | I | T | G | - | - | - | - | - | - | - | - | - | - | - | - | - | - | - | - | - | - | - | - | - | - | - | - | - | -101 |
| C1MTG3_MICPC | - | - | S | G | N | R | L | W | V | P | V | V | E | K | A | Y | A | K | A | H | G | - | - | - | - | - | - | - | - | S | Y | H | A | I | S | G | - | - | - | - | - | - | - | - | - | - | - | - | - | - | - | - | - | - | - | - | - | - | - | - | - | -131 |
| C1E493_MICSR | - | - | A | D | N | Q | L | W | V | P | L | V | E | K | A | Y | A | K | S | H | G | - | - | - | - | - | - | - | - | S | Y | H | A | I | S | G | - | - | - | - | - | - | - | - | - | - | - | - | - | - | - | - | - | - | - | - | - | - | - | - | - | -122 |
| Q012Z6_OSTTA | - | - | A | N | N | Q | L | W | V | S | L | T | E | K | A | Y | A | K | A | H | G | - | - | - | - | - | - | - | - | S | Y | S | A | I | S | G | - | - | - | - | - | - | - | - | - | - | - | - | - | - | - | - | - | - | - | - | - | - | - | - | - | -119 |
| L1IGB6_GUITH | - | - | Q | N | C | Q | L | W | V | S | L | L | E | K | A | Y | A | K | A | H | G | - | - | - | - | - | - | - | - | S | Y | R | A | I | S | G | - | - | - | - | - | - | - | - | - | - | - | - | - | - | - | - | - | - | - | - | - | - | - | - | - | -122 |
| B7FWX6_PHATC | - | - | P | D | L | Q | L | W | P | A | L | V | E | K | A | Y | A | K | A | H | G | - | - | - | - | - | - | - | - | S | Y | A | Q | L | S | G | - | - | - | - | - | - | - | - | - | - | - | - | - | - | - | - | - | - | - | - | - | - | - | - | - | -127 |
| N9TPR9_ENTHI | - | - | N | G | D | E | L | W | V | M | L | L | E | K | A | Y | A | K | V | V | G | - | - | - | - | - | - | - | - | S | Y | G | E | I | E | G | - | - | - | - | - | - | - | - | - | - | - | - | - | - | - | - | - | - | - | - | - | - | - | - | - | -116 |
| G0QS43_ICHMG | - | - | V | D | G | E | I | W | V | M | I | L | E | K | A | W | A | K | L | Y | G | - | - | - | - | - | - | - | - | S | Y | D | N | I | E | S | - | - | - | - | - | - | - | - | - | - | - | - | - | - | - | - | - | - | - | - | - | - | - | - | - | -123 |
| D8LB20_ECTSI | - | - | N | G | K | E | L | W | V | V | L | L | E | K | A | V | A | K | F | C | G | - | - | - | - | - | - | - | - | S | Y | S | N | I | A | G | - | - | - | - | - | - | - | - | - | - | - | - | - | - | - | - | - | - | - | - | - | - | - | - | - | -127 |
| Q9U0T9_LEIMA | - | - | D | L | C | E | L | W | P | A | V | L | E | K | A | F | A | K | M | H | G | - | - | - | - | - | - | - | - | S | Y | A | M | I | Q | S | - | - | - | - | - | - | - | - | - | - | - | - | - | - | - | - | - | - | - | - | - | - | - | - | - | -129 |
| Q4CXJ8_TRYCC | - | - | D | V | C | E | I | W | P | S | V | L | Q | K | A | F | A | K | L | N | G | - | - | - | - | - | - | - | - | S | Y | G | L | V | Q | S | - | - | - | - | - | - | - | - | - | - | - | - | - | - | - | - | - | - | - | - | - | - | - | - | - | -129 |
| F4I0A4_ARATH | - | - | K | L | N | E | L | W | V | S | M | V | E | K | A | Y | A | K | L | H | G | - | - | - | - | - | - | - | - | S | Y | E | A | L | E | G | - | - | - | - | - | - | - | - | - | - | - | - | - | - | - | - | - | - | - | - | - | - | - | - | - | -126 |
| Q8RUQ1_MAIZE | - | - | K | Q | N | E | L | W | V | S | I | L | E | K | A | Y | A | K | L | H | G | - | - | - | - | - | - | - | - | S | Y | E | A | L | E | G | - | - | - | - | - | - | - | - | - | - | - | - | - | - | - | - | - | - | - | - | - | - | - | - | - | -126 |
| D7FQ17_ECTSI | - | - | G | A | R | E | L | W | V | S | L | L | E | K | A | Y | A | K | F | F | G | - | - | - | - | - | - | - | - | S | Y | A | A | L | E | T | - | - | - | - | - | - | - | - | - | - | - | - | - | - | - | - | - | - | - | - | - | - | - | - | - | -143 |
| D0MZ44_PHYIT | - | - | D | F | E | E | L | W | V | P | L | V | E | K | A | F | A | K | Y | C | G | - | - | - | - | - | - | - | - | G | Y | A | A | L | E | G | - | - | - | - | - | - | - | - | - | - | - | - | - | - | - | - | - | - | - | - | - | - | - | - | - | -144 |
| L1JBM0_GUITH | - | - | K | M | E | H | C | W | V | Q | I | F | E | K | A | F | A | K | L | C | G | - | - | - | - | - | - | - | - | S | Y | A | D | I | E | G | - | - | - | - | - | - | - | - | - | - | - | - | - | - | - | - | - | - | - | - | - | - | - | - | - | -97 |
| L1J739_GUITH | - | - | D | V | C | E | I | W | V | M | L | I | E | K | A | Y | A | K | M | H | G | R | - | - | - | - | - | - | - | C | Y | R | N | L | E | T | - | - | - | - | - | - | - | - | - | - | - | - | - | - | - | - | - | - | - | - | - | - | - | - | - | -128 |
| L1J0U9_GUITH | - | - | D | P | Q | Q | I | W | P | M | L | L | E | K | A | Y | A | K | M | H | G | - | - | - | - | - | - | - | - | C | Y | E | A | L | V | T | - | - | - | - | - | - | - | - | - | - | - | - | - | - | - | - | - | - | - | - | - | - | - | - | - | -121 |
| L1J1Q4_GUITH | - | - | S | P | A | Q | V | W | V | Q | L | L | E | K | A | Y | A | K | L | H | G | - | - | - | - | - | - | - | - | S | Y | A | A | I | E | T | - | - | - | - | - | - | - | - | - | - | - | - | - | - | - | - | - | - | - | - | - | - | - | - | - | -139 |
| D0ND88_PHYIT | - | - | N | R | N | E | V | F | V | L | F | L | E | K | A | Y | A | K | L | H | G | - | - | - | - | - | - | - | - | S | Y | Q | I | L | D | E | K | Y | G | G | G | I | G | G | S | A | G | - | - | - | - | - | - | - | - | - | - | - | - | - | - | -153 |
| D2VVS6_NAEGR | - | - | D | P | N | E | L | W | V | P | L | L | E | K | A | Y | A | A | L | Y | G | - | - | - | - | - | - | - | - | N | Y | F | S | L | K | G | - | - | - | - | - | - | - | - | - | - | - | - | - | - | - | - | - | - | - | - | - | - | - | - | - | -125 |
| D2V3V2_NAEGR | - | - | T | S | N | E | M | W | I | S | L | L | E | K | A | Y | A | A | Y | Y | G | - | - | - | - | - | - | - | - | S | Y | Q | A | L | Q | S | - | - | - | - | - | - | - | - | - | - | - | - | - | - | - | - | - | - | - | - | - | - | - | - | - | -126 |
| Q5B5J7_EMENI | - | V | D | E | N | E | T | W | L | P | L | L | E | K | A | Y | A | K | A | H | G | - | - | - | - | - | - | - | - | D | Y | S | A | I | E | G | - | - | - | - | - | - | - | - | - | - | - | - | - | - | - | - | - | - | - | - | - | - | - | - | - | -146 |
| E4ZY10_LEPMJ | - | S | D | P | N | E | T | W | L | P | L | L | E | K | A | F | A | K | A | H | G | - | - | - | - | - | - | - | - | D | Y | A | A | I | D | G | - | - | - | - | - | - | - | - | - | - | - | - | - | - | - | - | - | - | - | - | - | - | - | - | - | -152 |
| A8PIE5_COPC7 | - | G | A | D | G | E | T | W | V | P | L | I | E | K | A | Y | A | K | L | N | G | - | - | - | - | - | - | - | - | N | F | A | H | L | E | G | - | - | - | - | - | - | - | - | - | - | - | - | - | - | - | - | - | - | - | - | - | - | - | - | - | -147 |
| A8NBA6_COPC7 | - | G | A | Q | D | E | T | W | V | P | L | I | E | K | A | Y | A | K | F | Y | C | - | - | - | - | - | - | - | - | N | Y | D | H | L | N | G | - | - | - | - | - | - | - | - | - | - | - | - | - | - | - | - | - | - | - | - | - | - | - | - | - | -146 |
| A8NBA1_COPC7 | - | G | S | E | G | E | T | W | V | P | L | I | E | K | A | Y | A | K | L | Y | G | - | - | - | - | - | - | - | - | N | F | K | H | L | D | G | - | - | - | - | - | - | - | - | - | - | - | - | - | - | - | - | - | - | - | - | - | - | - | - | - | -150 |
| A8NBA2_COPC7 | - | G | S | D | G | E | T | W | V | P | L | I | E | K | A | Y | A | K | L | Y | G | - | - | - | - | - | - | - | - | S | Y | E | H | L | D | R | - | - | - | - | - | - | - | - | - | - | - | - | - | - | - | - | - | - | - | - | - | - | - | - | - | -150 |
| A8NAE6_COPC7 | - | G | G | G | G | D | T | W | V | P | L | I | E | K | A | Y | A | K | V | H | G | - | - | - | - | - | - | - | - | D | Y | A | S | V | M | F | - | - | - | - | - | - | - | - | - | - | - | - | - | - | - | - | - | - | - | - | - | - | - | - | - | -177 |
| A8NAI9_COPC7 | - | G | Y | D | G | E | T | W | V | P | L | I | E | K | A | F | A | K | L | H | G | - | - | - | - | - | - | - | - | T | Y | E | A | L | S | G | - | - | - | - | - | - | - | - | - | - | - | - | - | - | - | - | - | - | - | - | - | - | - | - | - | -146 |
| G4TER7_PIRID | - | K | T | E | N | E | T | W | V | S | L | I | E | K | A | Y | A | K | I | H | G | - | - | - | - | - | - | - | - | N | Y | A | Y | L | S | G | - | - | - | - | - | - | - | - | - | - | - | - | - | - | - | - | - | - | - | - | - | - | - | - | - | -146 |
| G4TER6_PIRID | - | M | T | E | G | E | T | W | V | P | L | I | E | K | A | Y | A | K | L | H | G | - | - | - | - | - | - | - | - | N | Y | H | Y | I | V | G | - | - | - | - | - | - | - | - | - | - | - | - | - | - | - | - | - | - | - | - | - | - | - | - | - | -136 |
| G4TNA1_PIRID | - | L | T | D | D | E | V | W | V | P | L | I | E | K | A | Y | A | K | F | Y | G | - | - | - | - | - | - | - | - | S | Y | A | A | I | S | G | - | - | - | - | - | - | - | - | - | - | - | - | - | - | - | - | - | - | - | - | - | - | - | - | - | -146 |
| D0MWB9_PHYIT | - | - | N | P | D | E | L | W | V | S | L | L | E | K | A | Y | A | K | L | H | G | - | - | - | - | - | - | - | - | S | Y | E | A | L | I | G | - | - | - | - | - | - | - | - | - | - | - | - | - | - | - | - | - | - | - | - | - | - | - | - | - | -120 |
| L1I9H5_GUITH | - | - | - | T | K | E | L | W | P | L | V | F | E | K | A | W | A | K | L | H | L | - | - | - | - | - | - | - | - | S | Y | E | A | T | A | G | - | - | - | - | - | - | - | - | - | - | - | - | - | - | - | - | - | - | - | - | - | - | - | - | - | -135 |
| Q7N497_PHOLL | - | - | - | - | - | E | F | W | Y | Y | L | I | E | K | A | F | A | K | L | H | G | - | - | - | - | - | - | - | - | S | Y | E | N | L | G | G | - | - | - | - | - | - | - | - | - | - | - | - | - | - | - | - | - | - | - | - | - | - | - | - | - | -109 |
| ZP_11152369 | - | - | - | - | - | E | F | W | Y | F | L | I | E | K | A | F | A | K | L | A | G | - | - | - | - | - | - | - | - | G | Y | L | K | L | S | G | - | - | - | - | - | - | - | - | - | - | - | - | - | - | - | - | - | - | - | - | - | - | - | - | - | -105 |
| A2FEL7_TRIVA | - | - | E | R | S | P | Y | W | F | C | I | V | E | K | A | Y | A | K | I | V | G | - | - | - | - | - | - | - | - | S | Y | S | A | A | T | G | - | - | - | - | - | - | - | - | - | - | - | - | - | - | - | - | - | - | - | - | - | - | - | - | - | -121 |
| A2D9A8_TRIVA | - | - | E | N | S | P | W | W | F | C | M | V | E | K | T | F | C | K | L | V | G | - | - | - | - | - | - | - | - | S | L | S | E | S | E | G | - | - | - | - | - | - | - | - | - | - | - | - | - | - | - | - | - | - | - | - | - | - | - | - | - | -122 |
| A2FHB3_TRIVA | - | - | E | T | N | G | W | W | F | T | L | V | E | K | A | Y | A | K | Q | N | G | - | - | - | - | - | - | - | - | S | Y | S | A | I | E | G | - | - | - | - | - | - | - | - | - | - | - | - | - | - | - | - | - | - | - | - | - | - | - | - | - | -123 |
| A2DTE8_TRIVA | - | - | A | S | S | A | W | F | A | S | I | V | E | K | A | F | S | K | I | S | G | - | - | - | - | - | - | - | - | G | F | S | Q | I | A | G | - | - | - | - | - | - | - | - | - | - | - | - | - | - | - | - | - | - | - | - | - | - | - | - | - | -120 |
| A2E4X2_TRIVA | - | - | E | N | D | S | Y | W | F | A | L | I | E | K | A | Y | V | K | M | L | G | - | - | - | - | - | - | - | - | S | C | R | A | V | K | G | - | - | - | - | - | - | - | - | - | - | - | - | - | - | - | - | - | - | - | - | - | - | - | - | - | -125 |
| A2E1X1_TRIVA | - | - | K | S | D | S | I | W | F | C | L | V | E | K | A | Y | A | K | L | A | G | - | - | - | - | - | - | - | - | G | W | K | K | V | G | G | - | - | - | - | - | - | - | - | - | - | - | - | - | - | - | - | - | - | - | - | - | - | - | - | - | -110 |
| TPR_PORGI | - | W | F | N | G | E | Y | W | P | A | L | F | E | Q | A | Y | A | N | W | K | F | P | - | - | - | - | - | - | - | N | D | S | - | - | - | - | - | - | - | - | - | - | - | - | - | - | - | - | - | - | - | - | - | - | - | - | - | - | - | - | - | -137 |
| D5MF42_9BACT | - | A | E | A | G | E | I | W | P | A | V | Y | E | K | A | F | A | K | W | K | T | N | - | - | - | - | - | - | - | D | T | T | - | - | - | - | - | - | - | - | - | - | - | - | - | - | - | - | - | - | - | - | - | - | - | - | - | - | - | - | - | -97 |
| R9H254_BACT4 | - | R | N | K | E | V | T | W | A | T | I | L | E | K | A | M | I | K | W | N | Q | I | - | - | - | - | - | - | - | Y | Q | - | - | - | - | - | - | - | - | - | - | - | - | - | - | - | - | - | - | - | - | - | - | - | - | - | - | - | - | - | - | -97 |
| ZP_04847694 | - | R | N | K | E | V | T | W | A | T | I | L | E | K | A | M | I | K | W | N | Q | I | - | - | - | - | - | - | - | Y | Q | - | - | - | - | - | - | - | - | - | - | - | - | - | - | - | - | - | - | - | - | - | - | - | - | - | - | - | - | - | - | -97 |
| D7IBA4_9BACE | - | R | N | K | E | V | T | W | A | T | I | L | E | K | A | M | I | K | W | N | Q | I | - | - | - | - | - | - | - | Y | Q | - | - | - | - | - | - | - | - | - | - | - | - | - | - | - | - | - | - | - | - | - | - | - | - | - | - | - | - | - | - | -95 |
| ZP_09858577 | - | R | N | K | E | V | T | W | A | T | I | L | E | K | A | M | I | K | W | N | Q | I | - | - | - | - | - | - | - | Y | Q | - | - | - | - | - | - | - | - | - | - | - | - | - | - | - | - | - | - | - | - | - | - | - | - | - | - | - | - | - | - | -95 |
| E5C759_9BACE | - | R | N | K | E | V | T | W | A | T | I | L | E | K | A | M | M | K | W | R | Q | V | - | - | - | - | - | - | - | Y | L | - | - | - | - | - | - | - | - | - | - | - | - | - | - | - | - | - | - | - | - | - | - | - | - | - | - | - | - | - | - | -97 |
| A7LTS0_BACO1 | - | K | S | G | Q | P | T | W | S | T | V | L | E | K | A | V | I | K | W | F | Q | A | - | - | - | - | - | - | - | F | R | - | - | - | - | - | - | - | - | - | - | - | - | - | - | - | - | - | - | - | - | - | - | - | - | - | - | - | - | - | - | -95 |
| Q8YQK8_NOSS1 | - | R | N | D | G | V | I | W | V | A | L | V | E | K | A | Y | A | Q | W | R | E | W | - | - | - | - | - | - | - | - | - | - | - | R | E | N | - | - | - | - | - | - | - | - | - | - | - | - | - | - | - | - | - | - | - | - | - | - | - | - | - | -108 |
| Q3MBX8_ANAVT | - | T | N | G | G | V | L | W | V | A | L | V | E | K | A | Y | A | Q | W | R | E | W | - | - | - | - | - | - | - | - | - | - | - | R | E | G | - | - | - | - | - | - | - | - | - | - | - | - | - | - | - | - | - | - | - | - | - | - | - | - | - | -113 |
| Q8YZT8_NOSS1 | - | R | N | D | G | V | L | W | V | A | L | V | E | K | A | Y | A | Q | W | R | E | W | - | - | - | - | - | - | - | - | - | - | - | T | D | G | - | - | - | - | - | - | - | - | - | - | - | - | - | - | - | - | - | - | - | - | - | - | - | - | - | -101 |
| K9UIG2_9CHRO | - | S | N | K | D | G | L | W | A | P | I | I | E | K | A | C | A | Q | W | R | E | F | - | - | - | - | - | - | - | - | - | - | - | N | E | G | S | S | T | - | - | - | - | - | - | - | - | - | - | - | - | - | - | - | - | - | - | - | - | - | - | -107 |
| A4YRP8_BRASO | - | N | G | G | N | A | I | W | A | S | L | A | E | K | A | Y | A | E | L | Q | A | S | - | - | - | - | - | - | - | G | V | Y | T | G | N | S | I | N | Y | - | - | - | - | - | - | - | - | - | - | - | - | - | - | - | - | - | - | - | - | - | - | -102 |
| H0TAL7_9BRAD | - | N | G | G | N | A | I | W | A | S | L | A | E | K | A | Y | A | E | L | Q | A | S | - | - | - | - | - | - | - | G | V | Y | T | G | N | S | I | N | Y | - | - | - | - | - | - | - | - | - | - | - | - | - | - | - | - | - | - | - | - | - | - | -106 |
| H0SNL4_9BRAD | - | N | G | S | S | A | I | W | A | S | L | A | E | K | A | Y | A | E | L | Q | A | S | - | - | - | - | - | - | - | G | V | Y | T | G | N | S | I | N | Y | - | - | - | - | - | - | - | - | - | - | - | - | - | - | - | - | - | - | - | - | - | - | -108 |
| H0S4P6_9BRAD | - | N | G | G | A | A | I | W | A | S | L | A | E | K | G | Y | A | E | L | Q | A | S | - | - | - | - | - | - | - | G | V | Y | T | G | N | S | I | N | Y | - | - | - | - | - | - | - | - | - | - | - | - | - | - | - | - | - | - | - | - | - | - | -108 |
| A5EMK2_BRASB | - | N | G | G | T | A | I | W | A | S | L | A | E | K | A | Y | A | E | L | Q | A | S | - | - | - | - | - | - | - | G | V | Y | T | G | N | S | I | N | Y | - | - | - | - | - | - | - | - | - | - | - | - | - | - | - | - | - | - | - | - | - | - | -102 |
| M4ZBJ4_9BRAD | - | N | G | G | S | A | I | W | A | S | L | A | E | K | A | Y | A | E | L | Q | A | S | - | - | - | - | - | - | - | G | V | Y | T | G | N | S | I | N | Y | - | - | - | - | - | - | - | - | - | - | - | - | - | - | - | - | - | - | - | - | - | - | -108 |
| Q07SP7_RHOP5 | - | H | D | N | - | E | L | W | A | S | L | A | E | K | A | Y | A | Q | V | G | G | - | - | - | - | - | - | - | - | - | - | - | - | F | N | S | T | - | - | - | - | - | - | - | - | - | - | - | - | - | - | - | - | - | - | - | - | - | - | - | - | -95 |
| B0JUQ7_MICAN | - | T | - | G | N | E | L | W | V | A | L | A | E | R | A | Y | A | Q | I | N | - | E | - | - | - | - | - | - | - | S | G | W | I | G | - | - | - | - | - | - | - | - | - | - | - | - | - | - | - | - | - | - | - | - | - | - | - | - | - | - | - | -112 |
| K9WJ87_9CYAN | - | S | - | N | N | E | L | W | V | A | L | A | E | K | A | Y | A | Q | I | N | - | E | - | - | - | - | - | - | - | S | A | W | I | G | - | - | - | - | - | - | - | - | - | - | - | - | - | - | - | - | - | - | - | - | - | - | - | - | - | - | - | -107 |
| G6FUE0_9CYAN | - | S | - | A | N | E | L | W | V | A | L | A | E | K | A | Y | A | Q | L | N | - | E | - | - | - | - | - | - | - | S | G | W | I | Y | - | - | - | - | - | - | - | - | - | - | - | - | - | - | - | - | - | - | - | - | - | - | - | - | - | - | - | -106 |
| K9PHG5_9CYAN | - | Y | E | N | N | E | L | W | T | A | L | A | E | K | A | Y | A | Q | I | N | - | E | - | - | - | - | - | - | - | S | G | W | I | A | - | - | - | - | - | - | - | - | - | - | - | - | - | - | - | - | - | - | - | - | - | - | - | - | - | - | - | -109 |
| Q8YXX3_NOSS1 | - | P | - | N | T | E | L | W | V | A | L | A | E | K | A | Y | A | Q | I | N | - | E | - | - | - | - | - | - | - | S | G | W | T | R | G | P | A | S | - | - | - | - | - | - | - | - | - | - | - | - | - | - | - | - | - | - | - | - | - | - | - | -122 |
| K9TN70_9CYAN | - | V | - | N | N | E | L | W | V | A | L | A | E | K | A | Y | A | Q | I | N | - | E | - | - | - | - | - | - | - | A | A | W | I | G | - | - | - | - | - | - | - | - | - | - | - | - | - | - | - | - | - | - | - | - | - | - | - | - | - | - | - | -122 |
| Q05QW3_9SYNE | - | A | D | N | N | E | L | W | V | A | L | A | E | K | A | Y | A | Q | L | N | - | E | - | - | - | - | - | - | - | S | G | R | L | G | Q | E | E | S | T | N | - | - | - | - | - | - | - | - | - | - | - | - | - | - | - | - | - | - | - | - | - | -119 |
| Q05QW2_9SYNE | - | A | D | N | N | E | L | W | V | A | L | A | E | K | A | Y | A | Q | L | N | - | E | - | - | - | - | - | - | - | S | G | R | L | N | Q | E | E | A | T | N | - | - | - | - | - | - | - | - | - | - | - | - | - | - | - | - | - | - | - | - | - | -121 |
| Q05QW4_9SYNE | - | A | D | N | N | E | L | W | V | A | L | A | E | K | A | Y | A | Q | V | N | - | E | - | - | - | - | - | - | - | S | G | R | I | S | Q | N | - | - | - | - | - | - | - | - | - | - | - | - | - | - | - | - | - | - | - | - | - | - | - | - | - | -117 |
| E0UGX6_CYAP2 | - | - | - | A | N | E | L | W | V | A | F | A | E | K | A | Y | A | Q | L | A | - | E | - | - | - | - | - | - | - | E | G | W | T | R | S | S | S | - | - | - | - | - | - | - | - | - | - | - | - | - | - | - | - | - | - | - | - | - | - | - | - | -113 |
| K7WKF4_9NOST | - | - | - | S | N | E | L | W | V | A | L | A | E | K | A | Y | A | Q | L | A | - | E | - | - | - | - | - | - | - | S | G | W | S | R | T | Y | S | G | T | - | - | - | - | - | - | - | - | - | - | - | - | - | - | - | - | - | - | - | - | - | - | -111 |
| E0UGY5_CYAP2 | - | - | - | A | N | E | L | W | V | S | L | A | E | K | A | Y | A | Q | L | A | Q | E | - | - | - | - | - | - | - | E | G | W | S | R | P | G | M | A | - | - | - | - | - | - | - | - | - | - | - | - | - | - | - | - | - | - | - | - | - | - | - | -115 |
| A8LCE5_FRASN | - | A | G | E | P | E | L | W | A | A | L | Y | E | K | A | C | A | - | R | M | A | G | - | - | - | - | - | - | - | S | - | - | - | - | - | - | - | - | - | - | - | - | - | - | - | - | - | - | - | - | - | - | - | - | - | - | - | - | - | - | - | -110 |
| D3CT47_9ACTO | - | D | G | D | P | E | L | W | V | A | L | Y | E | K | A | Y | A | - | R | L | A | G | - | - | - | - | - | - | - | S | - | - | - | - | - | - | - | - | - | - | - | - | - | - | - | - | - | - | - | - | - | - | - | - | - | - | - | - | - | - | - | -114 |
| Q2J4J6_FRASC | - | A | G | R | P | E | L | W | P | A | L | Y | E | K | A | Y | A | - | R | L | H | G | - | - | - | - | - | - | - | G | - | - | - | - | - | - | - | - | - | - | - | - | - | - | - | - | - | - | - | - | - | - | - | - | - | - | - | - | - | - | - | -135 |
| Q0RAZ2_FRAAA | - | A | G | Q | P | E | L | W | P | A | L | Y | E | K | A | Y | A | - | R | L | H | G | - | - | - | - | - | - | - | G | - | - | - | - | - | - | - | - | - | - | - | - | - | - | - | - | - | - | - | - | - | - | - | - | - | - | - | - | - | - | - | -122 |
| E3J3F6_FRASU | - | N | G | R | P | E | L | W | A | A | V | Y | E | K | A | Y | A | - | R | A | H | G | - | - | - | - | - | - | - | G | - | - | - | - | - | - | - | - | - | - | - | - | - | - | - | - | - | - | - | - | - | - | - | - | - | - | - | - | - | - | - | -116 |
| ZP_09168240 | - | T | G | R | P | E | L | W | A | A | V | Y | E | K | A | Y | A | - | R | L | H | G | - | - | - | - | - | - | - | G | - | - | - | - | - | - | - | - | - | - | - | - | - | - | - | - | - | - | - | - | - | - | - | - | - | - | - | - | - | - | - | -126 |
| C7MDV9_BRAFD | - | D | G | A | N | P | G | W | V | G | V | L | E | K | A | I | A | G | H | V | A | G | - | - | - | - | - | - | - | D | - | - | - | - | - | - | - | - | - | - | - | - | - | - | - | - | - | - | - | - | - | - | - | - | - | - | - | - | - | - | - | -88 |
| ZP_10094989 | - | D | G | A | N | P | G | W | V | G | V | L | E | K | A | I | A | G | H | I | A | G | - | - | - | - | - | - | - | D | - | - | - | - | - | - | - | - | - | - | - | - | - | - | - | - | - | - | - | - | - | - | - | - | - | - | - | - | - | - | - | -88 |
| A4X268_SALTO | - | G | - | - | - | S | S | W | A | S | L | L | E | K | A | L | A | A | V | D | R | T | - | - | - | - | - | - | - | W | S | S | D | R | H | Q | Q | W | Q | L | T | W | A | A | W | E | - | - | - | - | - | - | - | - | - | - | - | - | - | - | - | -120 |
| WP_043162213 | - | G | - | - | - | S | S | W | A | S | L | L | E | K | A | L | A | A | V | D | R | T | - | - | - | - | - | - | - | W | S | S | D | R | H | Q | Q | W | Q | L | T | W | A | A | W | E | - | - | - | - | - | - | - | - | - | - | - | - | - | - | - | -120 |
| Q0BRV8_GRABC | - | N | G | T | K | E | I | W | P | Q | V | L | E | K | A | I | A | T | L | G | - | - | - | - | - | - | - | - | - | - | - | - | - | - | - | - | - | - | - | - | - | - | - | - | - | - | - | - | - | - | - | - | - | - | - | - | - | - | - | - | - | -114 |
| A9B2P0_HERA2 | - | S | D | E | F | E | I | W | P | M | L | V | E | K | A | Y | A | Q | H | K | G | G | - | - | - | - | - | - | - | - | - | - | - | - | - | - | - | - | - | - | - | - | - | - | - | - | - | - | - | - | - | - | - | - | - | - | - | - | - | - | - | -106 |
| A9B3U8_HERA2 | - | S | N | D | S | E | M | W | P | M | L | I | E | K | A | Y | A | K | Y | K | G | G | - | - | - | - | - | - | - | - | - | - | - | - | - | - | - | - | - | - | - | - | - | - | - | - | - | - | - | - | - | - | - | - | - | - | - | - | - | - | - | -108 |
| A9B2A3_HERA2 | - | D | G | K | R | E | L | W | P | A | I | M | E | K | A | Y | A | Q | F | L | S | Q | - | - | - | - | - | - | - | S | N | N | - | - | - | - | - | - | - | - | - | - | - | - | - | - | - | - | - | - | - | - | - | - | - | - | - | - | - | - | - | -121 |
| D6ZC85_SEGRD | - | G | T | P | G | Y | I | W | P | A | V | M | E | A | A | Y | A | K | L | L | D | G | - | - | - | - | - | - | - | E | Y | A | R | Y | T | D | V | K | K | F | M | N | P | V | - | - | - | - | - | - | - | - | - | - | - | - | - | - | - | - | - | -139 |
| E5XTT9_9ACTO | - | G | I | P | G | Y | I | W | P | A | V | M | E | A | A | Y | A | K | I | L | F | G | - | - | - | - | - | - | - | N | Y | P | T | H | P | N | A | Q | G | A | S | G | A | V | - | - | - | - | - | - | - | - | - | - | - | - | - | - | - | - | - | -139 |
| E5XTU0_9ACTO | - | G | R | K | G | Y | I | W | P | A | V | L | E | S | A | V | A | K | M | N | E | D | - | - | - | - | - | - | - | D | Y | G | S | R | A | D | P | H | H | P | G | H | T | Y | - | - | - | - | - | - | - | - | - | - | - | - | - | - | - | - | - | -143 |
| D6B0I6_9ACTO | - | - | - | - | - | E | T | W | P | A | Y | Y | E | K | A | F | A | M | V | Y | S | E | - | - | - | - | - | - | - | D | G | E | G | - | - | - | - | - | - | - | - | - | - | - | - | - | - | - | - | - | - | - | - | - | - | - | - | - | - | - | - | -106 |
| D9X719_STRVR | - | - | - | - | - | E | S | W | P | A | Y | Y | E | K | A | F | A | M | V | Y | S | D | - | - | - | - | - | - | - | D | G | D | D | - | - | - | - | - | - | - | - | - | - | - | - | - | - | - | - | - | - | - | - | - | - | - | - | - | - | - | - | -106 |
| H6L010_SAPGL | - | L | G | D | Q | E | L | W | V | M | L | L | E | K | A | Y | A | Q | Y | Q | G | - | - | - | - | - | - | - | - | - | - | - | - | - | - | - | - | - | - | - | - | - | - | - | - | - | - | - | - | - | - | - | - | - | - | - | - | - | - | - | - | -98 |
| K1ZP83_9BACT | - | E | S | N | N | E | L | W | V | A | L | Y | E | K | A | Y | A | V | Y | K | G | N | - | - | - | - | - | - | - | - | - | - | - | - | - | - | - | - | - | - | - | - | - | - | - | - | - | - | - | - | - | - | - | - | - | - | - | - | - | - | - | -119 |
| ADGB_HUMAN | - | - | - | - | - | - | L | W | P | M | L | L | S | K | A | I | I | K | L | A | N | I | D | - | I | H | - | - | - | V | A | D | R | R | E | L | - | - | - | - | - | - | - | - | - | - | - | - | - | - | - | - | - | - | - | - | - | - | - | - | - | -161 |
| J3QMP3_MOUSE | - | - | - | - | - | - | L | W | P | M | L | L | S | K | A | I | I | K | L | A | N | V | D | - | V | H | - | - | - | V | A | H | R | R | E | L | - | - | - | - | - | - | - | - | - | - | - | - | - | - | - | - | - | - | - | - | - | - | - | - | - | -161 |
| H2XR46_CIOIN | - | - | - | - | - | - | L | W | P | L | L | L | T | K | A | L | V | K | I | A | A | I | D | Y | V | C | - | - | - | N | N | D | A | S | E | I | - | - | - | - | - | - | - | - | - | - | - | - | - | - | - | - | - | - | - | - | - | - | - | - | - | -159 |
| CAN7_MOUSE | - | - | - | - | - | - | L | W | V | S | L | I | E | K | A | Y | M | K | V | M | G | G | - | - | - | - | - | - | - | - | Y | D | F | P | G | S | - | - | - | - | - | - | - | - | - | - | - | - | - | - | - | - | - | - | - | - | - | - | - | - | - | -131 |
| CAN7_HUMAN | - | - | - | - | - | - | L | W | V | S | L | I | E | K | A | Y | M | K | V | M | G | G | - | - | - | - | - | - | - | - | Y | D | F | P | G | S | - | - | - | - | - | - | - | - | - | - | - | - | - | - | - | - | - | - | - | - | - | - | - | - | - | -131 |
| E1BY84_CHICK | - | - | - | - | - | - | L | W | V | S | L | I | E | K | A | Y | M | K | V | M | G | G | - | - | - | - | - | - | - | - | Y | D | F | P | G | S | - | - | - | - | - | - | - | - | - | - | - | - | - | - | - | - | - | - | - | - | - | - | - | - | - | -131 |
| H9GX91_DANRE | - | - | - | - | - | - | L | W | V | S | L | I | E | K | A | Y | M | K | V | M | G | G | - | - | - | - | - | - | - | - | Y | D | F | P | G | S | - | - | - | - | - | - | - | - | - | - | - | - | - | - | - | - | - | - | - | - | - | - | - | - | - | -131 |
| F6SGI6_CIOIN | - | - | - | - | - | - | L | W | V | S | L | V | E | K | A | Y | M | K | V | M | G | G | - | - | - | - | - | - | - | - | Y | D | F | P | G | S | - | - | - | - | - | - | - | - | - | - | - | - | - | - | - | - | - | - | - | - | - | - | - | - | - | -131 |
| W4Y195_STRPU | - | - | - | - | - | - | F | W | V | S | L | L | E | K | A | Y | M | K | V | M | G | G | - | - | - | - | - | - | - | - | Y | D | F | P | G | S | - | - | - | - | - | - | - | - | - | - | - | - | - | - | - | - | - | - | - | - | - | - | - | - | - | -105 |
| R7V270_CAPTE | - | - | - | - | - | - | L | W | V | S | L | L | E | K | A | Y | L | K | V | M | G | G | - | - | - | - | - | - | - | - | Y | D | F | P | G | S | - | - | - | - | - | - | - | - | - | - | - | - | - | - | - | - | - | - | - | - | - | - | - | - | - | -131 |
| G4LXK3_SCHMA | - | - | - | - | - | - | L | W | V | S | L | L | E | K | A | Y | M | K | V | M | G | G | - | - | - | - | - | - | - | - | Y | D | F | P | G | S | - | - | - | - | - | - | - | - | - | - | - | - | - | - | - | - | - | - | - | - | - | - | - | - | - | -131 |
| T2M2K0_HYDVU | - | - | - | - | - | - | F | W | V | S | L | L | E | K | A | Y | M | K | V | M | G | G | - | - | - | - | - | - | - | - | Y | D | F | P | G | S | - | - | - | - | - | - | - | - | - | - | - | - | - | - | - | - | - | - | - | - | - | - | - | - | - | -131 |
| B3RQK1_TRIAD | - | - | - | - | - | - | F | W | V | S | L | M | E | K | A | Y | M | K | V | M | G | G | - | - | - | - | - | - | - | - | Y | D | F | P | G | S | - | - | - | - | - | - | - | - | - | - | - | - | - | - | - | - | - | - | - | - | - | - | - | - | - | -131 |
| I1CS59_RHIO9 | - | - | - | - | - | - | L | W | A | S | I | I | E | K | A | - | - | - | L | M | G | G | - | - | - | - | - | - | - | - | Y | D | F | P | G | S | - | - | - | - | - | - | - | - | - | - | - | - | - | - | - | - | - | - | - | - | - | - | - | - | - | -128 |
| I1GER3_AMPQE | - | - | - | - | - | - | F | W | V | S | L | L | E | K | A | Y | M | K | V | M | G | G | - | - | - | - | - | - | - | - | Y | D | F | P | G | S | - | - | - | - | - | - | - | - | - | - | - | - | - | - | - | - | - | - | - | - | - | - | - | - | - | -131 |
| Q22143_CAEEL | - | - | - | - | - | - | L | W | V | S | L | L | E | K | A | Y | M | K | V | M | G | G | - | - | - | - | - | - | - | - | Y | D | F | P | G | S | - | - | - | - | - | - | - | - | - | - | - | - | - | - | - | - | - | - | - | - | - | - | - | - | - | -131 |
| L1JZN7_GUITH | - | - | - | - | - | - | A | W | L | P | L | Y | E | K | A | Y | M | K | L | H | G | G | - | - | - | - | - | - | - | - | Y | D | F | P | G | S | - | - | - | - | - | - | - | - | - | - | - | - | - | - | - | - | - | - | - | - | - | - | - | - | - | -132 |
| D0NT34_PHYIT | - | - | - | - | - | - | L | W | V | S | L | I | E | K | A | Y | L | K | L | N | G | G | - | - | - | - | - | - | - | - | Y | D | F | P | G | G | - | - | - | - | - | - | - | - | - | - | - | - | - | - | - | - | - | - | - | - | - | - | - | - | - | -140 |
| A0A064B913_ASPOZ | - | - | - | - | - | - | L | W | P | A | L | V | E | K | A | Y | L | K | L | R | G | G | - | - | - | - | - | - | - | - | Y | D | F | P | G | S | - | - | - | - | - | - | - | - | - | - | - | - | - | - | - | - | - | - | - | - | - | - | - | - | - | -139 |
| A8N4X4_COPC7 | - | - | - | - | - | - | V | W | P | S | L | L | E | K | A | Y | M | K | L | M | G | G | - | - | - | - | - | - | - | - | Y | D | F | P | G | S | - | - | - | - | - | - | - | - | - | - | - | - | - | - | - | - | - | - | - | - | - | - | - | - | - | -155 |
| C0H5K1_PLAF7 | - | - | - | - | - | - | L | W | V | T | L | L | E | K | A | F | V | K | L | M | G | G | S | - | - | - | - | - | - | - | Y | S | M | F | G | S | - | - | - | - | - | - | - | - | - | - | - | - | - | - | - | - | - | - | - | - | - | - | - | - | - | -139 |
| K1REZ8_CRAGI | - | - | - | - | - | E | L | W | L | T | F | I | E | K | A | W | A | R | F | W | G | - | - | - | - | - | - | - | - | G | Y | S | N | I | E | Y | - | - | - | - | - | - | - | - | - | - | - | - | - | - | - | - | - | - | - | - | - | - | - | - | - | -118 |
| F4NTM9_BATDJ | - | - | - | - | - | N | L | W | V | P | L | L | E | K | A | F | S | K | L | N | G | - | - | - | - | - | - | - | - | S | Y | Q | L | I | K | S | - | - | - | - | - | - | - | - | - | - | - | - | - | - | - | - | - | - | - | - | - | - | - | - | - | -134 |
| Q6F6K5_CAEEL | - | - | - | - | K | Q | A | W | A | A | L | I | Q | K | A | F | A | K | L | G | G | - | - | - | - | - | - | - | - | S | Y | A | K | L | H | G | - | - | - | - | - | - | - | - | - | - | - | - | - | - | - | - | - | - | - | - | - | - | - | - | - | -99 |
| A8HZK8_CHLRE | - | - | - | - | - | E | L | W | P | S | L | L | E | K | A | Y | A | K | V | H | G | - | - | - | - | - | - | - | - | S | Y | Y | A | L | A | G | - | - | - | - | - | - | - | - | - | - | - | - | - | - | - | - | - | - | - | - | - | - | - | - | - | -100 |
| A8J6T6_CHLRE | - | - | - | - | - | E | V | W | V | L | L | L | E | K | A | V | A | R | M | V | S | - | - | - | - | - | - | - | - | S | Y | S | K | V | A | M | W | G | - | - | - | - | - | - | - | - | - | - | - | - | - | - | - | - | - | - | - | - | - | - | - | -132 |
| Z9JR51_9MICO | - | - | - | - | - | P | G | F | M | S | I | Y | E | R | A | M | I | N | A | H | D | G | - | - | - | - | - | - | - | R | P | S | A | V | S | - | - | - | - | - | - | - | - | - | - | - | - | - | - | - | - | - | - | - | - | - | - | - | - | - | - | -118 |
| F7ZZH9_CELGA | - | - | - | - | - | P | S | I | A | A | L | Y | E | A | A | I | R | Q | E | Y | G | - | - | - | - | - | - | - | - | - | Y | D | F | I | D | G | - | - | - | - | - | - | - | - | - | - | - | - | - | - | - | - | - | - | - | - | - | - | - | - | - | -101 |
| Q7NDU2_GLOVI | - | - | - | - | N | G | D | W | Q | A | V | Y | E | K | A | V | L | Q | A | G | L | S | - | - | - | - | - | - | - | - | - | - | - | - | - | - | - | - | - | - | - | - | - | - | - | - | - | - | - | - | - | - | - | - | - | - | - | - | - | - | - | -87 |
| I6AT95_9BACT | - | - | - | P | D | G | V | W | V | N | V | Y | E | K | A | V | G | L | L | R | V | E | K | A | K | S | - | - | - | D | G | E | P | A | S | L | I | D | - | - | - | - | - | - | - | - | - | - | - | - | - | - | - | - | - | - | - | - | - | - | - | -100 |
| C7MGJ8_BRAFD | - | G | D | S | S | A | L | W | V | A | Y | V | E | K | A | M | A | A | S | F | Q | D | D | S | A | Y | D | P | G | Q | Y | E | A | I | V | G | - | - | - | - | - | - | - | - | - | - | - | - | - | - | - | - | - | - | - | - | - | - | - | - | - | -116 |
| D0WP73_9ACTO | - | P | D | G | K | P | N | Y | A | S | I | L | E | S | A | Y | G | Q | Q | H | P | G | G | - | - | - | - | - | - | A | L | D | S | G | K | D | - | - | - | - | - | - | - | - | - | - | - | - | - | - | - | - | - | - | - | - | - | - | - | - | - | -110 |
| B0C8L1_ACAM1 | - | - | - | - | - | A | L | W | T | Q | I | L | E | K | A | Y | V | A | S | G | I | D | - | - | - | - | - | - | - | P | T | Q | E | L | T | K | - | - | - | - | - | - | - | - | - | - | - | - | - | - | - | - | - | - | - | - | - | - | - | - | - | -107 |
| B3E5A8_GEOLS | - | - | - | - | - | - | L | W | M | S | V | L | E | K | A | T | G | T | I | Q | A | R | - | - | - | - | - | - | - | S | Y | S | K | S | A | A | I | P | D | P | - | - | - | - | - | - | - | - | - | - | - | - | - | - | - | - | - | - | - | - | - | -101 |
| D4YQ94_9MICO | - | - | - | - | - | - | - | - | A | S | L | V | E | K | A | V | A | S | L | V | H | S | - | - | - | - | - | - | - | - | Y | V | R | I | Q | F | N | - | - | - | - | - | - | - | - | - | - | - | - | - | - | - | - | - | - | - | - | - | - | - | - | -101 |
| PAPA_CARPA | - | - | - | - | - | Y | P | W | S | A | L | Q | L | V | A | Q | Y | G | I | H | Y | R | N | - | - | - | - | T | Y | P | Y | E | G | V | Q | R | - | - | - | - | - | - | - | - | - | - | - | - | - | - | - | - | - | - | - | - | - | - | - | - | - | -93 |
| RIM13_YEAST | - | - | - | - | - | D | I | S | D | K | I | G | E | L | A | L | L | L | V | S | K | G | - | - | - | - | - | - | - | T | Y | S | T | D | G | S | - | - | - | - | - | - | - | - | - | - | - | - | - | - | - | - | - | - | - | - | - | - | - | - | - | -133 |

| I |  |  |  |  |  |  |  |  |  |  |  |  |  |  |  |  |  |  |  |  |  |  |  |  |  |  |  |  |  |  |  |  |  |  |  |  |  |  |  |  |  |  |  |  |  |  |  |  |  |  |  |  |  |  |  |  |  |  |  |  |  |  |
| --- | --- | --- | --- | --- | --- | --- | --- | --- | --- | --- | --- | --- | --- | --- | --- | --- | --- | --- | --- | --- | --- | --- | --- | --- | --- | --- | --- | --- | --- | --- | --- | --- | --- | --- | --- | --- | --- | --- | --- | --- | --- | --- | --- | --- | --- | --- | --- | --- | --- | --- | --- | --- | --- | --- | --- | --- | --- | --- | --- | --- | --- | --- |
| Q9N4B1_CAEEL | - | - | - | - | - | - | - | - | - | - | - | - | - | - | - | - | - | - | - | - | - | - | - | - | - | - | - | - | - | - | - | - | - | - | - | - | - | - | - | - | - | - | - | - | - | - | - | - | - | - | - | - | - | - | - | - | - | - | - | - | - | -123 |
| Q8IAA8_CAEEL | - | - | - | - | - | - | - | - | - | - | - | - | - | - | - | - | - | - | - | - | - | - | - | - | - | - | - | - | - | - | - | - | - | - | - | - | - | - | - | - | - | - | - | - | - | - | - | - | - | - | - | - | - | - | - | - | - | - | - | - | - | -123 |
| CAN_CAEEL | - | - | - | - | - | - | - | - | - | - | - | - | - | - | - | - | - | - | - | - | - | - | - | - | - | - | - | - | - | - | - | - | - | - | - | - | - | - | - | - | - | - | - | - | - | - | - | - | - | - | - | - | - | - | - | - | - | - | - | - | - | -123 |
| Q9U2B2_CAEEL | - | - | - | - | - | - | - | - | - | - | - | - | - | - | - | - | - | - | - | - | - | - | - | - | - | - | - | - | - | - | - | - | - | - | - | - | - | - | - | - | - | - | - | - | - | - | - | - | - | - | - | - | - | - | - | - | - | - | - | - | - | -123 |
| Q9N4B2_CAEEL | - | - | - | - | - | - | - | - | - | - | - | - | - | - | - | - | - | - | - | - | - | - | - | - | - | - | - | - | - | - | - | - | - | - | - | - | - | - | - | - | - | - | - | - | - | - | - | - | - | - | - | - | - | - | - | - | - | - | - | - | - | -123 |
| C3Y6P6_BRAFL | - | - | - | - | - | - | - | - | - | - | - | - | - | - | - | - | - | - | - | - | - | - | - | - | - | - | - | - | - | - | - | - | - | - | - | - | - | - | - | - | - | - | - | - | - | - | - | - | - | - | - | - | - | - | - | - | - | - | - | - | - | -129 |
| C3Y6N1_BRAFL | - | - | - | - | - | - | - | - | - | - | - | - | - | - | - | - | - | - | - | - | - | - | - | - | - | - | - | - | - | - | - | - | - | - | - | - | - | - | - | - | - | - | - | - | - | - | - | - | - | - | - | - | - | - | - | - | - | - | - | - | - | -130 |
| CANA_DROME | - | - | - | - | - | - | - | - | - | - | - | - | - | - | - | - | - | - | - | - | - | - | - | - | - | - | - | - | - | - | - | - | - | - | - | - | - | - | - | - | - | - | - | - | - | - | - | - | - | - | - | - | - | - | - | - | - | - | - | - | - | -123 |
| CANB_DROME | - | - | - | - | - | - | - | - | - | - | - | - | - | - | - | - | - | - | - | - | - | - | - | - | - | - | - | - | - | - | - | - | - | - | - | - | - | - | - | - | - | - | - | - | - | - | - | - | - | - | - | - | - | - | - | - | - | - | - | - | - | -123 |
| X1Y3H71_ANODA | - | - | - | - | - | - | - | - | - | - | - | - | - | - | - | - | - | - | - | - | - | - | - | - | - | - | - | - | - | - | - | - | - | - | - | - | - | - | - | - | - | - | - | - | - | - | - | - | - | - | - | - | - | - | - | - | - | - | - | - | - | -123 |
| Q5IW12_GECLA | - | - | - | - | - | - | - | - | - | - | - | - | - | - | - | - | - | - | - | - | - | - | - | - | - | - | - | - | - | - | - | - | - | - | - | - | - | - | - | - | - | - | - | - | - | - | - | - | - | - | - | - | - | - | - | - | - | - | - | - | - | -123 |
| K1Q056_CRAGI | - | - | - | - | - | - | - | - | - | - | - | - | - | - | - | - | - | - | - | - | - | - | - | - | - | - | - | - | - | - | - | - | - | - | - | - | - | - | - | - | - | - | - | - | - | - | - | - | - | - | - | - | - | - | - | - | - | - | - | - | - | -123 |
| G4V6C0_SCHMA | - | - | - | - | - | - | - | - | - | - | - | - | - | - | - | - | - | - | - | - | - | - | - | - | - | - | - | - | - | - | - | - | - | - | - | - | - | - | - | - | - | - | - | - | - | - | - | - | - | - | - | - | - | - | - | - | - | - | - | - | - | -127 |
| R7ULX9_CAPTE | - | - | - | - | - | - | - | - | - | - | - | - | - | - | - | - | - | - | - | - | - | - | - | - | - | - | - | - | - | - | - | - | - | - | - | - | - | - | - | - | - | - | - | - | - | - | - | - | - | - | - | - | - | - | - | - | - | - | - | - | - | -123 |
| W4Z5Q7_STRPU | - | - | - | - | - | - | - | - | - | - | - | - | - | - | - | - | - | - | - | - | - | - | - | - | - | - | - | - | - | - | - | - | - | - | - | - | - | - | - | - | - | - | - | - | - | - | - | - | - | - | - | - | - | - | - | - | - | - | - | - | - | -120 |
| R7VIB6_CAPTE | - | - | - | - | - | - | - | - | - | - | - | - | - | - | - | - | - | - | - | - | - | - | - | - | - | - | - | - | - | - | - | - | - | - | - | - | - | - | - | - | - | - | - | - | - | - | - | - | - | - | - | - | - | - | - | - | - | - | - | - | - | -126 |
| T2MJ45_HYDVU | - | - | - | - | - | - | - | - | - | - | - | - | - | - | - | - | - | - | - | - | - | - | - | - | - | - | - | - | - | - | - | - | - | - | - | - | - | - | - | - | - | - | - | - | - | - | - | - | - | - | - | - | - | - | - | - | - | - | - | - | - | -123 |
| R7UAB6_CAPTE | - | - | - | - | - | - | - | - | - | - | - | - | - | - | - | - | - | - | - | - | - | - | - | - | - | - | - | - | - | - | - | - | - | - | - | - | - | - | - | - | - | - | - | - | - | - | - | - | - | - | - | - | - | - | - | - | - | - | - | - | - | -123 |
| C3ZKN8_BRAFL | - | - | - | - | - | - | - | - | - | - | - | - | - | - | - | - | - | - | - | - | - | - | - | - | - | - | - | - | - | - | - | - | - | - | - | - | - | - | - | - | - | - | - | - | - | - | - | - | - | - | - | - | - | - | - | - | - | - | - | - | - | -124 |
| B3RJS5_TRIAD | - | - | - | - | - | - | - | - | - | - | - | - | - | - | - | - | - | - | - | - | - | - | - | - | - | - | - | - | - | - | - | - | - | - | - | - | - | - | - | - | - | - | - | - | - | - | - | - | - | - | - | - | - | - | - | - | - | - | - | - | - | -123 |
| H2YVF0_CIOSA | - | - | - | - | - | - | - | - | - | - | - | - | - | - | - | - | - | - | - | - | - | - | - | - | - | - | - | - | - | - | - | - | - | - | - | - | - | - | - | - | - | - | - | - | - | - | - | - | - | - | - | - | - | - | - | - | - | - | - | - | - | -123 |
| G4VAG2_SCHMA | - | - | - | - | - | - | - | - | - | - | - | - | - | - | - | - | - | - | - | - | - | - | - | - | - | - | - | - | - | - | - | - | - | - | - | - | - | - | - | - | - | - | - | - | - | - | - | - | - | - | - | - | - | - | - | - | - | - | - | - | - | -126 |
| Smp_157500 | - | - | - | - | - | - | - | - | - | - | - | - | - | - | - | - | - | - | - | - | - | - | - | - | - | - | - | - | - | - | - | - | - | - | - | - | - | - | - | - | - | - | - | - | - | - | - | - | - | - | - | - | - | - | - | - | - | - | - | - | - | -126 |
| Q7SYA9_DANRE | - | - | - | - | - | - | - | - | - | - | - | - | - | - | - | - | - | - | - | - | - | - | - | - | - | - | - | - | - | - | - | - | - | - | - | - | - | - | - | - | - | - | - | - | - | - | - | - | - | - | - | - | - | - | - | - | - | - | - | - | - | -123 |
| A5PMP1_DANRE | - | - | - | - | - | - | - | - | - | - | - | - | - | - | - | - | - | - | - | - | - | - | - | - | - | - | - | - | - | - | - | - | - | - | - | - | - | - | - | - | - | - | - | - | - | - | - | - | - | - | - | - | - | - | - | - | - | - | - | - | - | -123 |
| CANX_CHICK | - | - | - | - | - | - | - | - | - | - | - | - | - | - | - | - | - | - | - | - | - | - | - | - | - | - | - | - | - | - | - | - | - | - | - | - | - | - | - | - | - | - | - | - | - | - | - | - | - | - | - | - | - | - | - | - | - | - | - | - | - | -123 |
| CAN1_HUMAN | - | - | - | - | - | - | - | - | - | - | - | - | - | - | - | - | - | - | - | - | - | - | - | - | - | - | - | - | - | - | - | - | - | - | - | - | - | - | - | - | - | - | - | - | - | - | - | - | - | - | - | - | - | - | - | - | - | - | - | - | - | -123 |
| CAN1_MOUSE | - | - | - | - | - | - | - | - | - | - | - | - | - | - | - | - | - | - | - | - | - | - | - | - | - | - | - | - | - | - | - | - | - | - | - | - | - | - | - | - | - | - | - | - | - | - | - | - | - | - | - | - | - | - | - | - | - | - | - | - | - | -123 |
| O42133_CHICK | - | - | - | - | - | - | - | - | - | - | - | - | - | - | - | - | - | - | - | - | - | - | - | - | - | - | - | - | - | - | - | - | - | - | - | - | - | - | - | - | - | - | - | - | - | - | - | - | - | - | - | - | - | - | - | - | - | - | - | - | - | -123 |
| Q7ZUR1_DANRE | - | - | - | - | - | - | - | - | - | - | - | - | - | - | - | - | - | - | - | - | - | - | - | - | - | - | - | - | - | - | - | - | - | - | - | - | - | - | - | - | - | - | - | - | - | - | - | - | - | - | - | - | - | - | - | - | - | - | - | - | - | -123 |
| E9QFN0_DANRE | - | - | - | - | - | - | - | - | - | - | - | - | - | - | - | - | - | - | - | - | - | - | - | - | - | - | - | - | - | - | - | - | - | - | - | - | - | - | - | - | - | - | - | - | - | - | - | - | - | - | - | - | - | - | - | - | - | - | - | - | - | -123 |
| CAN2_HUMAN | - | - | - | - | - | - | - | - | - | - | - | - | - | - | - | - | - | - | - | - | - | - | - | - | - | - | - | - | - | - | - | - | - | - | - | - | - | - | - | - | - | - | - | - | - | - | - | - | - | - | - | - | - | - | - | - | - | - | - | - | - | -123 |
| CAN2_MOUSE | - | - | - | - | - | - | - | - | - | - | - | - | - | - | - | - | - | - | - | - | - | - | - | - | - | - | - | - | - | - | - | - | - | - | - | - | - | - | - | - | - | - | - | - | - | - | - | - | - | - | - | - | - | - | - | - | - | - | - | - | - | -123 |
| CAN2_CHICK | - | - | - | - | - | - | - | - | - | - | - | - | - | - | - | - | - | - | - | - | - | - | - | - | - | - | - | - | - | - | - | - | - | - | - | - | - | - | - | - | - | - | - | - | - | - | - | - | - | - | - | - | - | - | - | - | - | - | - | - | - | -123 |
| Q6DFZ8_DANRE | - | - | - | - | - | - | - | - | - | - | - | - | - | - | - | - | - | - | - | - | - | - | - | - | - | - | - | - | - | - | - | - | - | - | - | - | - | - | - | - | - | - | - | - | - | - | - | - | - | - | - | - | - | - | - | - | - | - | - | - | - | -123 |
| Q504F5_DANRE | - | - | - | - | - | - | - | - | - | - | - | - | - | - | - | - | - | - | - | - | - | - | - | - | - | - | - | - | - | - | - | - | - | - | - | - | - | - | - | - | - | - | - | - | - | - | - | - | - | - | - | - | - | - | - | - | - | - | - | - | - | -123 |
| Q5BLH5_DANRE | - | - | - | - | - | - | - | - | - | - | - | - | - | - | - | - | - | - | - | - | - | - | - | - | - | - | - | - | - | - | - | - | - | - | - | - | - | - | - | - | - | - | - | - | - | - | - | - | - | - | - | - | - | - | - | - | - | - | - | - | - | -123 |
| Q4VBH3_DANRE | - | - | - | - | - | - | - | - | - | - | - | - | - | - | - | - | - | - | - | - | - | - | - | - | - | - | - | - | - | - | - | - | - | - | - | - | - | - | - | - | - | - | - | - | - | - | - | - | - | - | - | - | - | - | - | - | - | - | - | - | - | -123 |
| CAN8_RAT | - | - | - | - | - | - | - | - | - | - | - | - | - | - | - | - | - | - | - | - | - | - | - | - | - | - | - | - | - | - | - | - | - | - | - | - | - | - | - | - | - | - | - | - | - | - | - | - | - | - | - | - | - | - | - | - | - | - | - | - | - | -123 |
| CAN8_MOUSE | - | - | - | - | - | - | - | - | - | - | - | - | - | - | - | - | - | - | - | - | - | - | - | - | - | - | - | - | - | - | - | - | - | - | - | - | - | - | - | - | - | - | - | - | - | - | - | - | - | - | - | - | - | - | - | - | - | - | - | - | - | -123 |
| CAN8_HUMAN | - | - | - | - | - | - | - | - | - | - | - | - | - | - | - | - | - | - | - | - | - | - | - | - | - | - | - | - | - | - | - | - | - | - | - | - | - | - | - | - | - | - | - | - | - | - | - | - | - | - | - | - | - | - | - | - | - | - | - | - | - | -123 |
| XP_426117 | - | - | - | - | - | - | - | - | - | - | - | - | - | - | - | - | - | - | - | - | - | - | - | - | - | - | - | - | - | - | - | - | - | - | - | - | - | - | - | - | - | - | - | - | - | - | - | - | - | - | - | - | - | - | - | - | - | - | - | - | - | -123 |
| CAN9_MOUSE | - | - | - | - | - | - | - | - | - | - | - | - | - | - | - | - | - | - | - | - | - | - | - | - | - | - | - | - | - | - | - | - | - | - | - | - | - | - | - | - | - | - | - | - | - | - | - | - | - | - | - | - | - | - | - | - | - | - | - | - | - | -125 |
| CAN9_HUMAN | - | - | - | - | - | - | - | - | - | - | - | - | - | - | - | - | - | - | - | - | - | - | - | - | - | - | - | - | - | - | - | - | - | - | - | - | - | - | - | - | - | - | - | - | - | - | - | - | - | - | - | - | - | - | - | - | - | - | - | - | - | -123 |
| E1C6M3_CHICK | - | - | - | - | - | - | - | - | - | - | - | - | - | - | - | - | - | - | - | - | - | - | - | - | - | - | - | - | - | - | - | - | - | - | - | - | - | - | - | - | - | - | - | - | - | - | - | - | - | - | - | - | - | - | - | - | - | - | - | - | - | -123 |
| Q6DG44_DANRE | - | - | - | - | - | - | - | - | - | - | - | - | - | - | - | - | - | - | - | - | - | - | - | - | - | - | - | - | - | - | - | - | - | - | - | - | - | - | - | - | - | - | - | - | - | - | - | - | - | - | - | - | - | - | - | - | - | - | - | - | - | -123 |
| CAN3_HUMAN | - | - | - | - | - | - | - | - | - | - | - | - | - | - | - | - | - | - | - | - | - | - | - | - | - | - | - | - | - | - | - | - | - | - | - | - | - | - | - | - | - | - | - | - | - | - | - | - | - | - | - | - | - | - | - | - | - | - | - | - | - | -123 |
| CAN3_MOUSE | - | - | - | - | - | - | - | - | - | - | - | - | - | - | - | - | - | - | - | - | - | - | - | - | - | - | - | - | - | - | - | - | - | - | - | - | - | - | - | - | - | - | - | - | - | - | - | - | - | - | - | - | - | - | - | - | - | - | - | - | - | -123 |
| CAN3_CHICK | - | - | - | - | - | - | - | - | - | - | - | - | - | - | - | - | - | - | - | - | - | - | - | - | - | - | - | - | - | - | - | - | - | - | - | - | - | - | - | - | - | - | - | - | - | - | - | - | - | - | - | - | - | - | - | - | - | - | - | - | - | -123 |
| Q66HU4_DANRE | - | - | - | - | - | - | - | - | - | - | - | - | - | - | - | - | - | - | - | - | - | - | - | - | - | - | - | - | - | - | - | - | - | - | - | - | - | - | - | - | - | - | - | - | - | - | - | - | - | - | - | - | - | - | - | - | - | - | - | - | - | -123 |
| F1R632_DANRE | - | - | - | - | - | - | - | - | - | - | - | - | - | - | - | - | - | - | - | - | - | - | - | - | - | - | - | - | - | - | - | - | - | - | - | - | - | - | - | - | - | - | - | - | - | - | - | - | - | - | - | - | - | - | - | - | - | - | - | - | - | -123 |
| CAN11_HUMAN | - | - | - | - | - | - | - | - | - | - | - | - | - | - | - | - | - | - | - | - | - | - | - | - | - | - | - | - | - | - | - | - | - | - | - | - | - | - | - | - | - | - | - | - | - | - | - | - | - | - | - | - | - | - | - | - | - | - | - | - | - | -123 |
| CAN11_MOUSE | - | - | - | - | - | - | - | - | - | - | - | - | - | - | - | - | - | - | - | - | - | - | - | - | - | - | - | - | - | - | - | - | - | - | - | - | - | - | - | - | - | - | - | - | - | - | - | - | - | - | - | - | - | - | - | - | - | - | - | - | - | -123 |
| CAN12_MOUSE | - | - | - | - | - | - | - | - | - | - | - | - | - | - | - | - | - | - | - | - | - | - | - | - | - | - | - | - | - | - | - | - | - | - | - | - | - | - | - | - | - | - | - | - | - | - | - | - | - | - | - | - | - | - | - | - | - | - | - | - | - | -123 |
| CAN12_HUMAN | - | - | - | - | - | - | - | - | - | - | - | - | - | - | - | - | - | - | - | - | - | - | - | - | - | - | - | - | - | - | - | - | - | - | - | - | - | - | - | - | - | - | - | - | - | - | - | - | - | - | - | - | - | - | - | - | - | - | - | - | - | -123 |
| ENSSARP00000008135 | - | - | - | - | - | - | - | - | - | - | - | - | - | - | - | - | - | - | - | - | - | - | - | - | - | - | - | - | - | - | - | - | - | - | - | - | - | - | - | - | - | - | - | - | - | - | - | - | - | - | - | - | - | - | - | - | - | - | - | - | - | -123 |
| ENSSARP00000010553 | - | - | - | - | - | - | - | - | - | - | - | - | - | - | - | - | - | - | - | - | - | - | - | - | - | - | - | - | - | - | - | - | - | - | - | - | - | - | - | - | - | - | - | - | - | - | - | - | - | - | - | - | - | - | - | - | - | - | - | - | - | -122 |
| A7E2G3_DANRE | - | - | - | - | - | - | - | - | - | - | - | - | - | - | - | - | - | - | - | - | - | - | - | - | - | - | - | - | - | - | - | - | - | - | - | - | - | - | - | - | - | - | - | - | - | - | - | - | - | - | - | - | - | - | - | - | - | - | - | - | - | -123 |
| Q6NW69_DANRE | - | - | - | - | - | - | - | - | - | - | - | - | - | - | - | - | - | - | - | - | - | - | - | - | - | - | - | - | - | - | - | - | - | - | - | - | - | - | - | - | - | - | - | - | - | - | - | - | - | - | - | - | - | - | - | - | - | - | - | - | - | -123 |
| CAN13_HUMAN | - | - | - | - | - | - | - | - | - | - | - | - | - | - | - | - | - | - | - | - | - | - | - | - | - | - | - | - | - | - | - | - | - | - | - | - | - | - | - | - | - | - | - | - | - | - | - | - | - | - | - | - | - | - | - | - | - | - | - | - | - | -124 |
| CAN13_MOUSE | - | - | - | - | - | - | - | - | - | - | - | - | - | - | - | - | - | - | - | - | - | - | - | - | - | - | - | - | - | - | - | - | - | - | - | - | - | - | - | - | - | - | - | - | - | - | - | - | - | - | - | - | - | - | - | - | - | - | - | - | - | -126 |
| E1BRR5_CHICK | - | - | - | - | - | - | - | - | - | - | - | - | - | - | - | - | - | - | - | - | - | - | - | - | - | - | - | - | - | - | - | - | - | - | - | - | - | - | - | - | - | - | - | - | - | - | - | - | - | - | - | - | - | - | - | - | - | - | - | - | - | -123 |
| E7F0H3_DANRE | - | - | - | - | - | - | - | - | - | - | - | - | - | - | - | - | - | - | - | - | - | - | - | - | - | - | - | - | - | - | - | - | - | - | - | - | - | - | - | - | - | - | - | - | - | - | - | - | - | - | - | - | - | - | - | - | - | - | - | - | - | -124 |
| Q1RLS9_DANRE | - | - | - | - | - | - | - | - | - | - | - | - | - | - | - | - | - | - | - | - | - | - | - | - | - | - | - | - | - | - | - | - | - | - | - | - | - | - | - | - | - | - | - | - | - | - | - | - | - | - | - | - | - | - | - | - | - | - | - | - | - | -123 |
| F1Q9Z1_DANRE | - | - | - | - | - | - | - | - | - | - | - | - | - | - | - | - | - | - | - | - | - | - | - | - | - | - | - | - | - | - | - | - | - | - | - | - | - | - | - | - | - | - | - | - | - | - | - | - | - | - | - | - | - | - | - | - | - | - | - | - | - | -124 |
| A4QNV6_DANRE | - | - | - | - | - | - | - | - | - | - | - | - | - | - | - | - | - | - | - | - | - | - | - | - | - | - | - | - | - | - | - | - | - | - | - | - | - | - | - | - | - | - | - | - | - | - | - | - | - | - | - | - | - | - | - | - | - | - | - | - | - | -124 |
| D6W573_HUMAN | - | - | - | - | - | - | - | - | - | - | - | - | - | - | - | - | - | - | - | - | - | - | - | - | - | - | - | - | - | - | - | - | - | - | - | - | - | - | - | - | - | - | - | - | - | - | - | - | - | - | - | - | - | - | - | - | - | - | - | - | - | -124 |
| R7UBC6_CAPTE | - | - | - | - | - | - | - | - | - | - | - | - | - | - | - | - | - | - | - | - | - | - | - | - | - | - | - | - | - | - | - | - | - | - | - | - | - | - | - | - | - | - | - | - | - | - | - | - | - | - | - | - | - | - | - | - | - | - | - | - | - | -128 |
| K1RCZ2_CRAGI | - | - | - | - | - | - | - | - | - | - | - | - | - | - | - | - | - | - | - | - | - | - | - | - | - | - | - | - | - | - | - | - | - | - | - | - | - | - | - | - | - | - | - | - | - | - | - | - | - | - | - | - | - | - | - | - | - | - | - | - | - | -130 |
| Q5IW13_GECLA | - | - | - | - | - | - | - | - | - | - | - | - | - | - | - | - | - | - | - | - | - | - | - | - | - | - | - | - | - | - | - | - | - | - | - | - | - | - | - | - | - | - | - | - | - | - | - | - | - | - | - | - | - | - | - | - | - | - | - | - | - | -123 |
| R7UUX7_CAPTE | - | - | - | - | - | - | - | - | - | - | - | - | - | - | - | - | - | - | - | - | - | - | - | - | - | - | - | - | - | - | - | - | - | - | - | - | - | - | - | - | - | - | - | - | - | - | - | - | - | - | - | - | - | - | - | - | - | - | - | - | - | -126 |
| K1Q505_CRAGI | - | - | - | - | - | - | - | - | - | - | - | - | - | - | - | - | - | - | - | - | - | - | - | - | - | - | - | - | - | - | - | - | - | - | - | - | - | - | - | - | - | - | - | - | - | - | - | - | - | - | - | - | - | - | - | - | - | - | - | - | - | -124 |
| R7UD02_CAPTE | - | - | - | - | - | - | - | - | - | - | - | - | - | - | - | - | - | - | - | - | - | - | - | - | - | - | - | - | - | - | - | - | - | - | - | - | - | - | - | - | - | - | - | - | - | - | - | - | - | - | - | - | - | - | - | - | - | - | - | - | - | -124 |
| CAN6_HUMAN | - | - | - | - | - | - | - | - | - | - | - | - | - | - | - | - | - | - | - | - | - | - | - | - | - | - | - | - | - | - | - | - | - | - | - | - | - | - | - | - | - | - | - | - | - | - | - | - | - | - | - | - | - | - | - | - | - | - | - | - | - | -129 |
| CAN6_MOUSE | - | - | - | - | - | - | - | - | - | - | - | - | - | - | - | - | - | - | - | - | - | - | - | - | - | - | - | - | - | - | - | - | - | - | - | - | - | - | - | - | - | - | - | - | - | - | - | - | - | - | - | - | - | - | - | - | - | - | - | - | - | -129 |
| E1C312_CHICK | - | - | - | - | - | - | - | - | - | - | - | - | - | - | - | - | - | - | - | - | - | - | - | - | - | - | - | - | - | - | - | - | - | - | - | - | - | - | - | - | - | - | - | - | - | - | - | - | - | - | - | - | - | - | - | - | - | - | - | - | - | -129 |
| E7FDN3_DANRE | - | - | - | - | - | - | - | - | - | - | - | - | - | - | - | - | - | - | - | - | - | - | - | - | - | - | - | - | - | - | - | - | - | - | - | - | - | - | - | - | - | - | - | - | - | - | - | - | - | - | - | - | - | - | - | - | - | - | - | - | - | -130 |
| CAN5_HUMAN | - | - | - | - | - | - | - | - | - | - | - | - | - | - | - | - | - | - | - | - | - | - | - | - | - | - | - | - | - | - | - | - | - | - | - | - | - | - | - | - | - | - | - | - | - | - | - | - | - | - | - | - | - | - | - | - | - | - | - | - | - | -125 |
| CAN5_MOUSE | - | - | - | - | - | - | - | - | - | - | - | - | - | - | - | - | - | - | - | - | - | - | - | - | - | - | - | - | - | - | - | - | - | - | - | - | - | - | - | - | - | - | - | - | - | - | - | - | - | - | - | - | - | - | - | - | - | - | - | - | - | -129 |
| E1C292_CHICK | - | - | - | - | - | - | - | - | - | - | - | - | - | - | - | - | - | - | - | - | - | - | - | - | - | - | - | - | - | - | - | - | - | - | - | - | - | - | - | - | - | - | - | - | - | - | - | - | - | - | - | - | - | - | - | - | - | - | - | - | - | -129 |
| A0JMG3_DANRE | - | - | - | - | - | - | - | - | - | - | - | - | - | - | - | - | - | - | - | - | - | - | - | - | - | - | - | - | - | - | - | - | - | - | - | - | - | - | - | - | - | - | - | - | - | - | - | - | - | - | - | - | - | - | - | - | - | - | - | - | - | -129 |
| E7F7F1_DANRE | - | - | - | - | - | - | - | - | - | - | - | - | - | - | - | - | - | - | - | - | - | - | - | - | - | - | - | - | - | - | - | - | - | - | - | - | - | - | - | - | - | - | - | - | - | - | - | - | - | - | - | - | - | - | - | - | - | - | - | - | - | -129 |
| XP_006824412 | - | - | - | - | - | - | - | - | - | - | - | - | - | - | - | - | - | - | - | - | - | - | - | - | - | - | - | - | - | - | - | - | - | - | - | - | - | - | - | - | - | - | - | - | - | - | - | - | - | - | - | - | - | - | - | - | - | - | - | - | - | -123 |
| W4YJN2_STRPU | - | - | - | - | - | - | - | - | - | - | - | - | - | - | - | - | - | - | - | - | - | - | - | - | - | - | - | - | - | - | - | - | - | - | - | - | - | - | - | - | - | - | - | - | - | - | - | - | - | - | - | - | - | - | - | - | - | - | - | - | - | -129 |
| C3YJZ3_BRAFL | - | - | - | - | - | - | - | - | - | - | - | - | - | - | - | - | - | - | - | - | - | - | - | - | - | - | - | - | - | - | - | - | - | - | - | - | - | - | - | - | - | - | - | - | - | - | - | - | - | - | - | - | - | - | - | - | - | - | - | - | - | -129 |
| T2MIT4_HYDVU | - | - | - | - | - | - | - | - | - | - | - | - | - | - | - | - | - | - | - | - | - | - | - | - | - | - | - | - | - | - | - | - | - | - | - | - | - | - | - | - | - | - | - | - | - | - | - | - | - | - | - | - | - | - | - | - | - | - | - | - | - | -129 |
| XP_002154722 | - | - | - | - | - | - | - | - | - | - | - | - | - | - | - | - | - | - | - | - | - | - | - | - | - | - | - | - | - | - | - | - | - | - | - | - | - | - | - | - | - | - | - | - | - | - | - | - | - | - | - | - | - | - | - | - | - | - | - | - | - | -93 |
| R7VLA7_CAPTE | - | - | - | - | - | - | - | - | - | - | - | - | - | - | - | - | - | - | - | - | - | - | - | - | - | - | - | - | - | - | - | - | - | - | - | - | - | - | - | - | - | - | - | - | - | - | - | - | - | - | - | - | - | - | - | - | - | - | - | - | - | -129 |
| K1R1C6_CRAGI | - | - | - | - | - | - | - | - | - | - | - | - | - | - | - | - | - | - | - | - | - | - | - | - | - | - | - | - | - | - | - | - | - | - | - | - | - | - | - | - | - | - | - | - | - | - | - | - | - | - | - | - | - | - | - | - | - | - | - | - | - | -129 |
| B3RIE5_TRIAD | - | - | - | - | - | - | - | - | - | - | - | - | - | - | - | - | - | - | - | - | - | - | - | - | - | - | - | - | - | - | - | - | - | - | - | - | - | - | - | - | - | - | - | - | - | - | - | - | - | - | - | - | - | - | - | - | - | - | - | - | - | -128 |
| K1PW75_CRAGI | - | - | - | - | - | - | - | - | - | - | - | - | - | - | - | - | - | - | - | - | - | - | - | - | - | - | - | - | - | - | - | - | - | - | - | - | - | - | - | - | - | - | - | - | - | - | - | - | - | - | - | - | - | - | - | - | - | - | - | - | - | -127 |
| K1RSB8_CRAGI | - | - | - | - | - | - | - | - | - | - | - | - | - | - | - | - | - | - | - | - | - | - | - | - | - | - | - | - | - | - | - | - | - | - | - | - | - | - | - | - | - | - | - | - | - | - | - | - | - | - | - | - | - | - | - | - | - | - | - | - | - | -127 |
| K1RWI3_CRAGI | - | - | - | - | - | - | - | - | - | - | - | - | - | - | - | - | - | - | - | - | - | - | - | - | - | - | - | - | - | - | - | - | - | - | - | - | - | - | - | - | - | - | - | - | - | - | - | - | - | - | - | - | - | - | - | - | - | - | - | - | - | -129 |
| R7VGC2_CAPTE | - | - | - | - | - | - | - | - | - | - | - | - | - | - | - | - | - | - | - | - | - | - | - | - | - | - | - | - | - | - | - | - | - | - | - | - | - | - | - | - | - | - | - | - | - | - | - | - | - | - | - | - | - | - | - | - | - | - | - | - | - | -128 |
| K1QU26_CRAGI | - | - | - | - | - | - | - | - | - | - | - | - | - | - | - | - | - | - | - | - | - | - | - | - | - | - | - | - | - | - | - | - | - | - | - | - | - | - | - | - | - | - | - | - | - | - | - | - | - | - | - | - | - | - | - | - | - | - | - | - | - | -130 |
| CAN5_CAEEL | - | - | - | - | - | - | - | - | - | - | - | - | - | - | - | - | - | - | - | - | - | - | - | - | - | - | - | - | - | - | - | - | - | - | - | - | - | - | - | - | - | - | - | - | - | - | - | - | - | - | - | - | - | - | - | - | - | - | - | - | - | -127 |
| I1FGV4_AMPQE | - | - | - | - | - | - | - | - | - | - | - | - | - | - | - | - | - | - | - | - | - | - | - | - | - | - | - | - | - | - | - | - | - | - | - | - | - | - | - | - | - | - | - | - | - | - | - | - | - | - | - | - | - | - | - | - | - | - | - | - | - | -123 |
| K1PHX3_CRAGI | - | - | - | - | - | - | - | - | - | - | - | - | - | - | - | - | - | - | - | - | - | - | - | - | - | - | - | - | - | - | - | - | - | - | - | - | - | - | - | - | - | - | - | - | - | - | - | - | - | - | - | - | - | - | - | - | - | - | - | - | - | -123 |
| B3RNL8_TRIAD | - | - | - | - | - | - | - | - | - | - | - | - | - | - | - | - | - | - | - | - | - | - | - | - | - | - | - | - | - | - | - | - | - | - | - | - | - | - | - | - | - | - | - | - | - | - | - | - | - | - | - | - | - | - | - | - | - | - | - | - | - | -125 |
| X1Y3H7_ANODA | - | - | - | - | - | - | - | - | - | - | - | - | - | - | - | - | - | - | - | - | - | - | - | - | - | - | - | - | - | - | - | - | - | - | - | - | - | - | - | - | - | - | - | - | - | - | - | - | - | - | - | - | - | - | - | - | - | - | - | - | - | -127 |
| R7TWH9_CAPTE | - | - | - | - | - | - | - | - | - | - | - | - | - | - | - | - | - | - | - | - | - | - | - | - | - | - | - | - | - | - | - | - | - | - | - | - | - | - | - | - | - | - | - | - | - | - | - | - | - | - | - | - | - | - | - | - | - | - | - | - | - | -127 |
| K1R5E8_CRAGI | - | - | - | - | - | - | - | - | - | - | - | - | - | - | - | - | - | - | - | - | - | - | - | - | - | - | - | - | - | - | - | - | - | - | - | - | - | - | - | - | - | - | - | - | - | - | - | - | - | - | - | - | - | - | - | - | - | - | - | - | - | -127 |
| XP_006811804 | - | - | - | - | - | - | - | - | - | - | - | - | - | - | - | - | - | - | - | - | - | - | - | - | - | - | - | - | - | - | - | - | - | - | - | - | - | - | - | - | - | - | - | - | - | - | - | - | - | - | - | - | - | - | - | - | - | - | - | - | - | -125 |
| C3YB23_BRAFL | - | - | - | - | - | - | - | - | - | - | - | - | - | - | - | - | - | - | - | - | - | - | - | - | - | - | - | - | - | - | - | - | - | - | - | - | - | - | - | - | - | - | - | - | - | - | - | - | - | - | - | - | - | - | - | - | - | - | - | - | - | -132 |
| H2ZIF1_CIOSA | - | - | - | - | - | - | - | - | - | - | - | - | - | - | - | - | - | - | - | - | - | - | - | - | - | - | - | - | - | - | - | - | - | - | - | - | - | - | - | - | - | - | - | - | - | - | - | - | - | - | - | - | - | - | - | - | - | - | - | - | - | -124 |
| W4YV93_STRPU | - | - | - | - | - | - | - | - | - | - | - | - | - | - | - | - | - | - | - | - | - | - | - | - | - | - | - | - | - | - | - | - | - | - | - | - | - | - | - | - | - | - | - | - | - | - | - | - | - | - | - | - | - | - | - | - | - | - | - | - | - | -124 |
| CAN10_HUMAN | - | - | - | - | - | - | - | - | - | - | - | - | - | - | - | - | - | - | - | - | - | - | - | - | - | - | - | - | - | - | - | - | - | - | - | - | - | - | - | - | - | - | - | - | - | - | - | - | - | - | - | - | - | - | - | - | - | - | - | - | - | -125 |
| CAN10_MOUSE | - | - | - | - | - | - | - | - | - | - | - | - | - | - | - | - | - | - | - | - | - | - | - | - | - | - | - | - | - | - | - | - | - | - | - | - | - | - | - | - | - | - | - | - | - | - | - | - | - | - | - | - | - | - | - | - | - | - | - | - | - | -125 |
| XP_004943443 | - | - | - | - | - | - | - | - | - | - | - | - | - | - | - | - | - | - | - | - | - | - | - | - | - | - | - | - | - | - | - | - | - | - | - | - | - | - | - | - | - | - | - | - | - | - | - | - | - | - | - | - | - | - | - | - | - | - | - | - | - | -125 |
| F8W4K1_DANRE | - | - | - | - | - | - | - | - | - | - | - | - | - | - | - | - | - | - | - | - | - | - | - | - | - | - | - | - | - | - | - | - | - | - | - | - | - | - | - | - | - | - | - | - | - | - | - | - | - | - | - | - | - | - | - | - | - | - | - | - | - | -125 |
| CANC_DROME | - | - | - | - | - | - | - | - | - | - | - | - | - | - | - | - | - | - | - | - | - | - | - | - | - | - | - | - | - | - | - | - | - | - | - | - | - | - | - | - | - | - | - | - | - | - | - | - | - | - | - | - | - | - | - | - | - | - | - | - | - | -111 |
| C3ZKN9_BRAFL | - | - | - | - | - | - | - | - | - | - | - | - | - | - | - | - | - | - | - | - | - | - | - | - | - | - | - | - | - | - | - | - | - | - | - | - | - | - | - | - | - | - | - | - | - | - | - | - | - | - | - | - | - | - | - | - | - | - | - | - | - | -123 |
| G4V623_SCHMA | - | - | - | - | - | - | - | - | - | - | - | - | - | - | - | - | - | - | - | - | - | - | - | - | - | - | - | - | - | - | - | - | - | - | - | - | - | - | - | - | - | - | - | - | - | - | - | - | - | - | - | - | - | - | - | - | - | - | - | - | - | -142 |
| R7VK39_CAPTE | - | - | - | - | - | - | - | - | - | - | - | - | - | - | - | - | - | - | - | - | - | - | - | - | - | - | - | - | - | - | - | - | - | - | - | - | - | - | - | - | - | - | - | - | - | - | - | - | - | - | - | - | - | - | - | - | - | - | - | - | - | -124 |
| G4V693_SCHMA | - | - | - | - | - | - | - | - | - | - | - | - | - | - | - | - | - | - | - | - | - | - | - | - | - | - | - | - | - | - | - | - | - | - | - | - | - | - | - | - | - | - | - | - | - | - | - | - | - | - | - | - | - | - | - | - | - | - | - | - | - | -128 |
| R7UPU6_CAPTE | - | - | - | - | - | - | - | - | - | - | - | - | - | - | - | - | - | - | - | - | - | - | - | - | - | - | - | - | - | - | - | - | - | - | - | - | - | - | - | - | - | - | - | - | - | - | - | - | - | - | - | - | - | - | - | - | - | - | - | - | - | -129 |
| R7V817_CAPTE | - | - | - | - | - | - | - | - | - | - | - | - | - | - | - | - | - | - | - | - | - | - | - | - | - | - | - | - | - | - | - | - | - | - | - | - | - | - | - | - | - | - | - | - | - | - | - | - | - | - | - | - | - | - | - | - | - | - | - | - | - | -126 |
| R7UNL8_CAPTE | - | - | - | - | - | - | - | - | - | - | - | - | - | - | - | - | - | - | - | - | - | - | - | - | - | - | - | - | - | - | - | - | - | - | - | - | - | - | - | - | - | - | - | - | - | - | - | - | - | - | - | - | - | - | - | - | - | - | - | - | - | -124 |
| K1PJ80_CRAGI | - | - | - | - | - | - | - | - | - | - | - | - | - | - | - | - | - | - | - | - | - | - | - | - | - | - | - | - | - | - | - | - | - | - | - | - | - | - | - | - | - | - | - | - | - | - | - | - | - | - | - | - | - | - | - | - | - | - | - | - | - | -127 |
| K1PCX8_CRAGI | - | - | - | - | - | - | - | - | - | - | - | - | - | - | - | - | - | - | - | - | - | - | - | - | - | - | - | - | - | - | - | - | - | - | - | - | - | - | - | - | - | - | - | - | - | - | - | - | - | - | - | - | - | - | - | - | - | - | - | - | - | -127 |
| CAND_DROME | - | - | - | - | - | - | - | - | - | - | - | - | - | - | - | - | - | - | - | - | - | - | - | - | - | - | - | - | - | - | - | - | - | - | - | - | - | - | - | - | - | - | - | - | - | - | - | - | - | - | - | - | - | - | - | - | - | - | - | - | - | -125 |
| XP_002735765 | - | - | - | - | - | - | - | - | - | - | - | - | - | - | - | - | - | - | - | - | - | - | - | - | - | - | - | - | - | - | - | - | - | - | - | - | - | - | - | - | - | - | - | - | - | - | - | - | - | - | - | - | - | - | - | - | - | - | - | - | - | -127 |
| CAN15_HUMAN | - | - | - | - | - | - | - | - | - | - | - | - | - | - | - | - | - | - | - | - | - | - | - | - | - | - | - | - | - | - | - | - | - | - | - | - | - | - | - | - | - | - | - | - | - | - | - | - | - | - | - | - | - | - | - | - | - | - | - | - | - | -127 |
| CAN15_MOUSE | - | - | - | - | - | - | - | - | - | - | - | - | - | - | - | - | - | - | - | - | - | - | - | - | - | - | - | - | - | - | - | - | - | - | - | - | - | - | - | - | - | - | - | - | - | - | - | - | - | - | - | - | - | - | - | - | - | - | - | - | - | -127 |
| E1BTA8_CHICK | - | - | - | - | - | - | - | - | - | - | - | - | - | - | - | - | - | - | - | - | - | - | - | - | - | - | - | - | - | - | - | - | - | - | - | - | - | - | - | - | - | - | - | - | - | - | - | - | - | - | - | - | - | - | - | - | - | - | - | - | - | -127 |
| E7FAX5_DANRE | - | - | - | - | - | - | - | - | - | - | - | - | - | - | - | - | - | - | - | - | - | - | - | - | - | - | - | - | - | - | - | - | - | - | - | - | - | - | - | - | - | - | - | - | - | - | - | - | - | - | - | - | - | - | - | - | - | - | - | - | - | -127 |
| C3XWK8_BRAFL | - | - | - | - | - | - | - | - | - | - | - | - | - | - | - | - | - | - | - | - | - | - | - | - | - | - | - | - | - | - | - | - | - | - | - | - | - | - | - | - | - | - | - | - | - | - | - | - | - | - | - | - | - | - | - | - | - | - | - | - | - | -126 |
| W4YMU4_STRPU | - | F | L | S | A | L | S | V | L | A | E | R | A | D | L | L | K | R | I | M | I | T | H | E | V | N | P | E | G | A | Y | Q | V | R | L | C | R | D | G | I | W | D | M | V | L | V | D | D | L | L | P | C | Q | E | Q | G | C | L | V | Y | S | -212 |
| K1PV74_CRAGI | - | - | - | - | - | - | - | - | - | - | - | - | - | - | - | - | - | - | - | - | - | - | - | - | - | - | - | - | - | - | - | - | - | - | - | - | - | - | - | - | - | - | - | - | - | - | - | - | - | - | - | - | - | - | - | - | - | - | - | - | - | -127 |
| R7TJF2_CAPTE | - | - | - | - | - | - | - | - | - | - | - | - | - | - | - | - | - | - | - | - | - | - | - | - | - | - | - | - | - | - | - | - | - | - | - | - | - | - | - | - | - | - | - | - | - | - | - | - | - | - | - | - | - | - | - | - | - | - | - | - | - | -127 |
| T2MI17_HYDVU | - | - | - | - | - | - | - | - | - | - | - | - | - | - | - | - | - | - | - | - | - | - | - | - | - | - | - | - | - | - | - | - | - | - | - | - | - | - | - | - | - | - | - | - | - | - | - | - | - | - | - | - | - | - | - | - | - | - | - | - | - | -124 |
| Q1RL55_CIOIN | - | - | - | - | - | - | - | - | - | - | - | - | - | - | - | - | - | - | - | - | - | - | - | - | - | - | - | - | - | - | - | - | - | - | - | - | - | - | - | - | - | - | - | - | - | - | - | - | - | - | - | - | - | - | - | - | - | - | - | - | - | -114 |
| I1EA30_AMPQE | - | - | - | - | - | - | - | - | - | - | - | - | - | - | - | - | - | - | - | - | - | - | - | - | - | - | - | - | - | - | - | - | - | - | - | - | - | - | - | - | - | - | - | - | - | - | - | - | - | - | - | - | - | - | - | - | - | - | - | - | - | -127 |
| G5EEZ6_CAEEL | - | - | - | - | - | - | - | - | - | - | - | - | - | - | - | - | - | - | - | - | - | - | - | - | - | - | - | - | - | - | - | - | - | - | - | - | - | - | - | - | - | - | - | - | - | - | - | - | - | - | - | - | - | - | - | - | - | - | - | - | - | -127 |
| O44903_CAEEL | - | - | - | - | - | - | - | - | - | - | - | - | - | - | - | - | - | - | - | - | - | - | - | - | - | - | - | - | - | - | - | - | - | - | - | - | - | - | - | - | - | - | - | - | - | - | - | - | - | - | - | - | - | - | - | - | - | - | - | - | - | -127 |
| O02260_CAEEL | - | - | - | - | - | - | - | - | - | - | - | - | - | - | - | - | - | - | - | - | - | - | - | - | - | - | - | - | - | - | - | - | - | - | - | - | - | - | - | - | - | - | - | - | - | - | - | - | - | - | - | - | - | - | - | - | - | - | - | - | - | -124 |
| O02259_CAEEL | - | - | - | - | - | - | - | - | - | - | - | - | - | - | - | - | - | - | - | - | - | - | - | - | - | - | - | - | - | - | - | - | - | - | - | - | - | - | - | - | - | - | - | - | - | - | - | - | - | - | - | - | - | - | - | - | - | - | - | - | - | -101 |
| C1MTG3_MICPC | - | - | - | - | - | - | - | - | - | - | - | - | - | - | - | - | - | - | - | - | - | - | - | - | - | - | - | - | - | - | - | - | - | - | - | - | - | - | - | - | - | - | - | - | - | - | - | - | - | - | - | - | - | - | - | - | - | - | - | - | - | -131 |
| C1E493_MICSR | - | - | - | - | - | - | - | - | - | - | - | - | - | - | - | - | - | - | - | - | - | - | - | - | - | - | - | - | - | - | - | - | - | - | - | - | - | - | - | - | - | - | - | - | - | - | - | - | - | - | - | - | - | - | - | - | - | - | - | - | - | -122 |
| Q012Z6_OSTTA | - | - | - | - | - | - | - | - | - | - | - | - | - | - | - | - | - | - | - | - | - | - | - | - | - | - | - | - | - | - | - | - | - | - | - | - | - | - | - | - | - | - | - | - | - | - | - | - | - | - | - | - | - | - | - | - | - | - | - | - | - | -119 |
| L1IGB6_GUITH | - | - | - | - | - | - | - | - | - | - | - | - | - | - | - | - | - | - | - | - | - | - | - | - | - | - | - | - | - | - | - | - | - | - | - | - | - | - | - | - | - | - | - | - | - | - | - | - | - | - | - | - | - | - | - | - | - | - | - | - | - | -122 |
| B7FWX6_PHATC | - | - | - | - | - | - | - | - | - | - | - | - | - | - | - | - | - | - | - | - | - | - | - | - | - | - | - | - | - | - | - | - | - | - | - | - | - | - | - | - | - | - | - | - | - | - | - | - | - | - | - | - | - | - | - | - | - | - | - | - | - | -127 |
| N9TPR9_ENTHI | - | - | - | - | - | - | - | - | - | - | - | - | - | - | - | - | - | - | - | - | - | - | - | - | - | - | - | - | - | - | - | - | - | - | - | - | - | - | - | - | - | - | - | - | - | - | - | - | - | - | - | - | - | - | - | - | - | - | - | - | - | -116 |
| G0QS43_ICHMG | - | - | - | - | - | - | - | - | - | - | - | - | - | - | - | - | - | - | - | - | - | - | - | - | - | - | - | - | - | - | - | - | - | - | - | - | - | - | - | - | - | - | - | - | - | - | - | - | - | - | - | - | - | - | - | - | - | - | - | - | - | -123 |
| D8LB20_ECTSI | - | - | - | - | - | - | - | - | - | - | - | - | - | - | - | - | - | - | - | - | - | - | - | - | - | - | - | - | - | - | - | - | - | - | - | - | - | - | - | - | - | - | - | - | - | - | - | - | - | - | - | - | - | - | - | - | - | - | - | - | - | -127 |
| Q9U0T9_LEIMA | - | - | - | - | - | - | - | - | - | - | - | - | - | - | - | - | - | - | - | - | - | - | - | - | - | - | - | - | - | - | - | - | - | - | - | - | - | - | - | - | - | - | - | - | - | - | - | - | - | - | - | - | - | - | - | - | - | - | - | - | - | -129 |
| Q4CXJ8_TRYCC | - | - | - | - | - | - | - | - | - | - | - | - | - | - | - | - | - | - | - | - | - | - | - | - | - | - | - | - | - | - | - | - | - | - | - | - | - | - | - | - | - | - | - | - | - | - | - | - | - | - | - | - | - | - | - | - | - | - | - | - | - | -129 |
| F4I0A4_ARATH | - | - | - | - | - | - | - | - | - | - | - | - | - | - | - | - | - | - | - | - | - | - | - | - | - | - | - | - | - | - | - | - | - | - | - | - | - | - | - | - | - | - | - | - | - | - | - | - | - | - | - | - | - | - | - | - | - | - | - | - | - | -126 |
| Q8RUQ1_MAIZE | - | - | - | - | - | - | - | - | - | - | - | - | - | - | - | - | - | - | - | - | - | - | - | - | - | - | - | - | - | - | - | - | - | - | - | - | - | - | - | - | - | - | - | - | - | - | - | - | - | - | - | - | - | - | - | - | - | - | - | - | - | -126 |
| D7FQ17_ECTSI | - | - | - | - | - | - | - | - | - | - | - | - | - | - | - | - | - | - | - | - | - | - | - | - | - | - | - | - | - | - | - | - | - | - | - | - | - | - | - | - | - | - | - | - | - | - | - | - | - | - | - | - | - | - | - | - | - | - | - | - | - | -143 |
| D0MZ44_PHYIT | - | - | - | - | - | - | - | - | - | - | - | - | - | - | - | - | - | - | - | - | - | - | - | - | - | - | - | - | - | - | - | - | - | - | - | - | - | - | - | - | - | - | - | - | - | - | - | - | - | - | - | - | - | - | - | - | - | - | - | - | - | -144 |
| L1JBM0_GUITH | - | - | - | - | - | - | - | - | - | - | - | - | - | - | - | - | - | - | - | - | - | - | - | - | - | - | - | - | - | - | - | - | - | - | - | - | - | - | - | - | - | - | - | - | - | - | - | - | - | - | - | - | - | - | - | - | - | - | - | - | - | -97 |
| L1J739_GUITH | - | - | - | - | - | - | - | - | - | - | - | - | - | - | - | - | - | - | - | - | - | - | - | - | - | - | - | - | - | - | - | - | - | - | - | - | - | - | - | - | - | - | - | - | - | - | - | - | - | - | - | - | - | - | - | - | - | - | - | - | - | -128 |
| L1J0U9_GUITH | - | - | - | - | - | - | - | - | - | - | - | - | - | - | - | - | - | - | - | - | - | - | - | - | - | - | - | - | - | - | - | - | - | - | - | - | - | - | - | - | - | - | - | - | - | - | - | - | - | - | - | - | - | - | - | - | - | - | - | - | - | -121 |
| L1J1Q4_GUITH | - | - | - | - | - | - | - | - | - | - | - | - | - | - | - | - | - | - | - | - | - | - | - | - | - | - | - | - | - | - | - | - | - | - | - | - | - | - | - | - | - | - | - | - | - | - | - | - | - | - | - | - | - | - | - | - | - | - | - | - | - | -139 |
| D0ND88_PHYIT | - | - | - | - | - | - | - | - | - | - | - | - | - | - | - | - | - | - | - | - | - | - | - | - | - | - | - | - | - | - | - | - | - | - | - | - | - | - | - | - | - | - | - | - | - | - | - | - | - | - | - | - | - | - | - | - | - | - | - | - | - | -153 |
| D2VVS6_NAEGR | - | - | - | - | - | - | - | - | - | - | - | - | - | - | - | - | - | - | - | - | - | - | - | - | - | - | - | - | - | - | - | - | - | - | - | - | - | - | - | - | - | - | - | - | - | - | - | - | - | - | - | - | - | - | - | - | - | - | - | - | - | -125 |
| D2V3V2_NAEGR | - | - | - | - | - | - | - | - | - | - | - | - | - | - | - | - | - | - | - | - | - | - | - | - | - | - | - | - | - | - | - | - | - | - | - | - | - | - | - | - | - | - | - | - | - | - | - | - | - | - | - | - | - | - | - | - | - | - | - | - | - | -126 |
| Q5B5J7_EMENI | - | - | - | - | - | - | - | - | - | - | - | - | - | - | - | - | - | - | - | - | - | - | - | - | - | - | - | - | - | - | - | - | - | - | - | - | - | - | - | - | - | - | - | - | - | - | - | - | - | - | - | - | - | - | - | - | - | - | - | - | - | -146 |
| E4ZY10_LEPMJ | - | - | - | - | - | - | - | - | - | - | - | - | - | - | - | - | - | - | - | - | - | - | - | - | - | - | - | - | - | - | - | - | - | - | - | - | - | - | - | - | - | - | - | - | - | - | - | - | - | - | - | - | - | - | - | - | - | - | - | - | - | -152 |
| A8PIE5_COPC7 | - | - | - | - | - | - | - | - | - | - | - | - | - | - | - | - | - | - | - | - | - | - | - | - | - | - | - | - | - | - | - | - | - | - | - | - | - | - | - | - | - | - | - | - | - | - | - | - | - | - | - | - | - | - | - | - | - | - | - | - | - | -147 |
| A8NBA6_COPC7 | - | - | - | - | - | - | - | - | - | - | - | - | - | - | - | - | - | - | - | - | - | - | - | - | - | - | - | - | - | - | - | - | - | - | - | - | - | - | - | - | - | - | - | - | - | - | - | - | - | - | - | - | - | - | - | - | - | - | - | - | - | -146 |
| A8NBA1_COPC7 | - | - | - | - | - | - | - | - | - | - | - | - | - | - | - | - | - | - | - | - | - | - | - | - | - | - | - | - | - | - | - | - | - | - | - | - | - | - | - | - | - | - | - | - | - | - | - | - | - | - | - | - | - | - | - | - | - | - | - | - | - | -150 |
| A8NBA2_COPC7 | - | - | - | - | - | - | - | - | - | - | - | - | - | - | - | - | - | - | - | - | - | - | - | - | - | - | - | - | - | - | - | - | - | - | - | - | - | - | - | - | - | - | - | - | - | - | - | - | - | - | - | - | - | - | - | - | - | - | - | - | - | -150 |
| A8NAE6_COPC7 | - | - | - | - | - | - | - | - | - | - | - | - | - | - | - | - | - | - | - | - | - | - | - | - | - | - | - | - | - | - | - | - | - | - | - | - | - | - | - | - | - | - | - | - | - | - | - | - | - | - | - | - | - | - | - | - | - | - | - | - | - | -177 |
| A8NAI9_COPC7 | - | - | - | - | - | - | - | - | - | - | - | - | - | - | - | - | - | - | - | - | - | - | - | - | - | - | - | - | - | - | - | - | - | - | - | - | - | - | - | - | - | - | - | - | - | - | - | - | - | - | - | - | - | - | - | - | - | - | - | - | - | -146 |
| G4TER7_PIRID | - | - | - | - | - | - | - | - | - | - | - | - | - | - | - | - | - | - | - | - | - | - | - | - | - | - | - | - | - | - | - | - | - | - | - | - | - | - | - | - | - | - | - | - | - | - | - | - | - | - | - | - | - | - | - | - | - | - | - | - | - | -146 |
| G4TER6_PIRID | - | - | - | - | - | - | - | - | - | - | - | - | - | - | - | - | - | - | - | - | - | - | - | - | - | - | - | - | - | - | - | - | - | - | - | - | - | - | - | - | - | - | - | - | - | - | - | - | - | - | - | - | - | - | - | - | - | - | - | - | - | -136 |
| G4TNA1_PIRID | - | - | - | - | - | - | - | - | - | - | - | - | - | - | - | - | - | - | - | - | - | - | - | - | - | - | - | - | - | - | - | - | - | - | - | - | - | - | - | - | - | - | - | - | - | - | - | - | - | - | - | - | - | - | - | - | - | - | - | - | - | -146 |
| D0MWB9_PHYIT | - | - | - | - | - | - | - | - | - | - | - | - | - | - | - | - | - | - | - | - | - | - | - | - | - | - | - | - | - | - | - | - | - | - | - | - | - | - | - | - | - | - | - | - | - | - | - | - | - | - | - | - | - | - | - | - | - | - | - | - | - | -120 |
| L1I9H5_GUITH | - | - | - | - | - | - | - | - | - | - | - | - | - | - | - | - | - | - | - | - | - | - | - | - | - | - | - | - | - | - | - | - | - | - | - | - | - | - | - | - | - | - | - | - | - | - | - | - | - | - | - | - | - | - | - | - | - | - | - | - | - | -135 |
| Q7N497_PHOLL | - | - | - | - | - | - | - | - | - | - | - | - | - | - | - | - | - | - | - | - | - | - | - | - | - | - | - | - | - | - | - | - | - | - | - | - | - | - | - | - | - | - | - | - | - | - | - | - | - | - | - | - | - | - | - | - | - | - | - | - | - | -109 |
| ZP_11152369 | - | - | - | - | - | - | - | - | - | - | - | - | - | - | - | - | - | - | - | - | - | - | - | - | - | - | - | - | - | - | - | - | - | - | - | - | - | - | - | - | - | - | - | - | - | - | - | - | - | - | - | - | - | - | - | - | - | - | - | - | - | -105 |
| A2FEL7_TRIVA | - | - | - | - | - | - | - | - | - | - | - | - | - | - | - | - | - | - | - | - | - | - | - | - | - | - | - | - | - | - | - | - | - | - | - | - | - | - | - | - | - | - | - | - | - | - | - | - | - | - | - | - | - | - | - | - | - | - | - | - | - | -121 |
| A2D9A8_TRIVA | - | - | - | - | - | - | - | - | - | - | - | - | - | - | - | - | - | - | - | - | - | - | - | - | - | - | - | - | - | - | - | - | - | - | - | - | - | - | - | - | - | - | - | - | - | - | - | - | - | - | - | - | - | - | - | - | - | - | - | - | - | -122 |
| A2FHB3_TRIVA | - | - | - | - | - | - | - | - | - | - | - | - | - | - | - | - | - | - | - | - | - | - | - | - | - | - | - | - | - | - | - | - | - | - | - | - | - | - | - | - | - | - | - | - | - | - | - | - | - | - | - | - | - | - | - | - | - | - | - | - | - | -123 |
| A2DTE8_TRIVA | - | - | - | - | - | - | - | - | - | - | - | - | - | - | - | - | - | - | - | - | - | - | - | - | - | - | - | - | - | - | - | - | - | - | - | - | - | - | - | - | - | - | - | - | - | - | - | - | - | - | - | - | - | - | - | - | - | - | - | - | - | -120 |
| A2E4X2_TRIVA | - | - | - | - | - | - | - | - | - | - | - | - | - | - | - | - | - | - | - | - | - | - | - | - | - | - | - | - | - | - | - | - | - | - | - | - | - | - | - | - | - | - | - | - | - | - | - | - | - | - | - | - | - | - | - | - | - | - | - | - | - | -125 |
| A2E1X1_TRIVA | - | - | - | - | - | - | - | - | - | - | - | - | - | - | - | - | - | - | - | - | - | - | - | - | - | - | - | - | - | - | - | - | - | - | - | - | - | - | - | - | - | - | - | - | - | - | - | - | - | - | - | - | - | - | - | - | - | - | - | - | - | -110 |
| TPR_PORGI | - | - | - | - | - | - | - | - | - | - | - | - | - | - | - | - | - | - | - | - | - | - | - | - | - | - | - | - | - | - | - | - | - | - | - | - | - | - | - | - | - | - | - | - | - | - | - | - | - | - | - | - | - | - | - | - | - | - | - | - | - | -137 |
| D5MF42_9BACT | - | - | - | - | - | - | - | - | - | - | - | - | - | - | - | - | - | - | - | - | - | - | - | - | - | - | - | - | - | - | - | - | - | - | - | - | - | - | - | - | - | - | - | - | - | - | - | - | - | - | - | - | - | - | - | - | - | - | - | - | - | -97 |
| R9H254_BACT4 | - | - | - | - | - | - | - | - | - | - | - | - | - | - | - | - | - | - | - | - | - | - | - | - | - | - | - | - | - | - | - | - | - | - | - | - | - | - | - | - | - | - | - | - | - | - | - | - | - | - | - | - | - | - | - | - | - | - | - | - | - | -97 |
| ZP_04847694 | - | - | - | - | - | - | - | - | - | - | - | - | - | - | - | - | - | - | - | - | - | - | - | - | - | - | - | - | - | - | - | - | - | - | - | - | - | - | - | - | - | - | - | - | - | - | - | - | - | - | - | - | - | - | - | - | - | - | - | - | - | -97 |
| D7IBA4_9BACE | - | - | - | - | - | - | - | - | - | - | - | - | - | - | - | - | - | - | - | - | - | - | - | - | - | - | - | - | - | - | - | - | - | - | - | - | - | - | - | - | - | - | - | - | - | - | - | - | - | - | - | - | - | - | - | - | - | - | - | - | - | -95 |
| ZP_09858577 | - | - | - | - | - | - | - | - | - | - | - | - | - | - | - | - | - | - | - | - | - | - | - | - | - | - | - | - | - | - | - | - | - | - | - | - | - | - | - | - | - | - | - | - | - | - | - | - | - | - | - | - | - | - | - | - | - | - | - | - | - | -95 |
| E5C759_9BACE | - | - | - | - | - | - | - | - | - | - | - | - | - | - | - | - | - | - | - | - | - | - | - | - | - | - | - | - | - | - | - | - | - | - | - | - | - | - | - | - | - | - | - | - | - | - | - | - | - | - | - | - | - | - | - | - | - | - | - | - | - | -97 |
| A7LTS0_BACO1 | - | - | - | - | - | - | - | - | - | - | - | - | - | - | - | - | - | - | - | - | - | - | - | - | - | - | - | - | - | - | - | - | - | - | - | - | - | - | - | - | - | - | - | - | - | - | - | - | - | - | - | - | - | - | - | - | - | - | - | - | - | -95 |
| Q8YQK8_NOSS1 | - | - | - | - | - | - | - | - | - | - | - | - | - | - | - | - | - | - | - | - | - | - | - | - | - | - | - | - | - | - | - | - | - | - | - | - | - | - | - | - | - | - | - | - | - | - | - | - | - | - | - | - | - | - | - | - | - | - | - | - | - | -108 |
| Q3MBX8_ANAVT | - | - | - | - | - | - | - | - | - | - | - | - | - | - | - | - | - | - | - | - | - | - | - | - | - | - | - | - | - | - | - | - | - | - | - | - | - | - | - | - | - | - | - | - | - | - | - | - | - | - | - | - | - | - | - | - | - | - | - | - | - | -113 |
| Q8YZT8_NOSS1 | - | - | - | - | - | - | - | - | - | - | - | - | - | - | - | - | - | - | - | - | - | - | - | - | - | - | - | - | - | - | - | - | - | - | - | - | - | - | - | - | - | - | - | - | - | - | - | - | - | - | - | - | - | - | - | - | - | - | - | - | - | -101 |
| K9UIG2_9CHRO | - | - | - | - | - | - | - | - | - | - | - | - | - | - | - | - | - | - | - | - | - | - | - | - | - | - | - | - | - | - | - | - | - | - | - | - | - | - | - | - | - | - | - | - | - | - | - | - | - | - | - | - | - | - | - | - | - | - | - | - | - | -107 |
| A4YRP8_BRASO | - | - | - | - | - | - | - | - | - | - | - | - | - | - | - | - | - | - | - | - | - | - | - | - | - | - | - | - | - | - | - | - | - | - | - | - | - | - | - | - | - | - | - | - | - | - | - | - | - | - | - | - | - | - | - | - | - | - | - | - | - | -102 |
| H0TAL7_9BRAD | - | - | - | - | - | - | - | - | - | - | - | - | - | - | - | - | - | - | - | - | - | - | - | - | - | - | - | - | - | - | - | - | - | - | - | - | - | - | - | - | - | - | - | - | - | - | - | - | - | - | - | - | - | - | - | - | - | - | - | - | - | -106 |
| H0SNL4_9BRAD | - | - | - | - | - | - | - | - | - | - | - | - | - | - | - | - | - | - | - | - | - | - | - | - | - | - | - | - | - | - | - | - | - | - | - | - | - | - | - | - | - | - | - | - | - | - | - | - | - | - | - | - | - | - | - | - | - | - | - | - | - | -108 |
| H0S4P6_9BRAD | - | - | - | - | - | - | - | - | - | - | - | - | - | - | - | - | - | - | - | - | - | - | - | - | - | - | - | - | - | - | - | - | - | - | - | - | - | - | - | - | - | - | - | - | - | - | - | - | - | - | - | - | - | - | - | - | - | - | - | - | - | -108 |
| A5EMK2_BRASB | - | - | - | - | - | - | - | - | - | - | - | - | - | - | - | - | - | - | - | - | - | - | - | - | - | - | - | - | - | - | - | - | - | - | - | - | - | - | - | - | - | - | - | - | - | - | - | - | - | - | - | - | - | - | - | - | - | - | - | - | - | -102 |
| M4ZBJ4_9BRAD | - | - | - | - | - | - | - | - | - | - | - | - | - | - | - | - | - | - | - | - | - | - | - | - | - | - | - | - | - | - | - | - | - | - | - | - | - | - | - | - | - | - | - | - | - | - | - | - | - | - | - | - | - | - | - | - | - | - | - | - | - | -108 |
| Q07SP7_RHOP5 | - | - | - | - | - | - | - | - | - | - | - | - | - | - | - | - | - | - | - | - | - | - | - | - | - | - | - | - | - | - | - | - | - | - | - | - | - | - | - | - | - | - | - | - | - | - | - | - | - | - | - | - | - | - | - | - | - | - | - | - | - | -95 |
| B0JUQ7_MICAN | - | - | - | - | - | - | - | - | - | - | - | - | - | - | - | - | - | - | - | - | - | - | - | - | - | - | - | - | - | - | - | - | - | - | - | - | - | - | - | - | - | - | - | - | - | - | - | - | - | - | - | - | - | - | - | - | - | - | - | - | - | -112 |
| K9WJ87_9CYAN | - | - | - | - | - | - | - | - | - | - | - | - | - | - | - | - | - | - | - | - | - | - | - | - | - | - | - | - | - | - | - | - | - | - | - | - | - | - | - | - | - | - | - | - | - | - | - | - | - | - | - | - | - | - | - | - | - | - | - | - | - | -107 |
| G6FUE0_9CYAN | - | - | - | - | - | - | - | - | - | - | - | - | - | - | - | - | - | - | - | - | - | - | - | - | - | - | - | - | - | - | - | - | - | - | - | - | - | - | - | - | - | - | - | - | - | - | - | - | - | - | - | - | - | - | - | - | - | - | - | - | - | -106 |
| K9PHG5_9CYAN | - | - | - | - | - | - | - | - | - | - | - | - | - | - | - | - | - | - | - | - | - | - | - | - | - | - | - | - | - | - | - | - | - | - | - | - | - | - | - | - | - | - | - | - | - | - | - | - | - | - | - | - | - | - | - | - | - | - | - | - | - | -109 |
| Q8YXX3_NOSS1 | - | - | - | - | - | - | - | - | - | - | - | - | - | - | - | - | - | - | - | - | - | - | - | - | - | - | - | - | - | - | - | - | - | - | - | - | - | - | - | - | - | - | - | - | - | - | - | - | - | - | - | - | - | - | - | - | - | - | - | - | - | -122 |
| K9TN70_9CYAN | - | - | - | - | - | - | - | - | - | - | - | - | - | - | - | - | - | - | - | - | - | - | - | - | - | - | - | - | - | - | - | - | - | - | - | - | - | - | - | - | - | - | - | - | - | - | - | - | - | - | - | - | - | - | - | - | - | - | - | - | - | -122 |
| Q05QW3_9SYNE | - | - | - | - | - | - | - | - | - | - | - | - | - | - | - | - | - | - | - | - | - | - | - | - | - | - | - | - | - | - | - | - | - | - | - | - | - | - | - | - | - | - | - | - | - | - | - | - | - | - | - | - | - | - | - | - | - | - | - | - | - | -119 |
| Q05QW2_9SYNE | - | - | - | - | - | - | - | - | - | - | - | - | - | - | - | - | - | - | - | - | - | - | - | - | - | - | - | - | - | - | - | - | - | - | - | - | - | - | - | - | - | - | - | - | - | - | - | - | - | - | - | - | - | - | - | - | - | - | - | - | - | -121 |
| Q05QW4_9SYNE | - | - | - | - | - | - | - | - | - | - | - | - | - | - | - | - | - | - | - | - | - | - | - | - | - | - | - | - | - | - | - | - | - | - | - | - | - | - | - | - | - | - | - | - | - | - | - | - | - | - | - | - | - | - | - | - | - | - | - | - | - | -117 |
| E0UGX6_CYAP2 | - | - | - | - | - | - | - | - | - | - | - | - | - | - | - | - | - | - | - | - | - | - | - | - | - | - | - | - | - | - | - | - | - | - | - | - | - | - | - | - | - | - | - | - | - | - | - | - | - | - | - | - | - | - | - | - | - | - | - | - | - | -113 |
| K7WKF4_9NOST | - | - | - | - | - | - | - | - | - | - | - | - | - | - | - | - | - | - | - | - | - | - | - | - | - | - | - | - | - | - | - | - | - | - | - | - | - | - | - | - | - | - | - | - | - | - | - | - | - | - | - | - | - | - | - | - | - | - | - | - | - | -111 |
| E0UGY5_CYAP2 | - | - | - | - | - | - | - | - | - | - | - | - | - | - | - | - | - | - | - | - | - | - | - | - | - | - | - | - | - | - | - | - | - | - | - | - | - | - | - | - | - | - | - | - | - | - | - | - | - | - | - | - | - | - | - | - | - | - | - | - | - | -115 |
| A8LCE5_FRASN | - | - | - | - | - | - | - | - | - | - | - | - | - | - | - | - | - | - | - | - | - | - | - | - | - | - | - | - | - | - | - | - | - | - | - | - | - | - | - | - | - | - | - | - | - | - | - | - | - | - | - | - | - | - | - | - | - | - | - | - | - | -110 |
| D3CT47_9ACTO | - | - | - | - | - | - | - | - | - | - | - | - | - | - | - | - | - | - | - | - | - | - | - | - | - | - | - | - | - | - | - | - | - | - | - | - | - | - | - | - | - | - | - | - | - | - | - | - | - | - | - | - | - | - | - | - | - | - | - | - | - | -114 |
| Q2J4J6_FRASC | - | - | - | - | - | - | - | - | - | - | - | - | - | - | - | - | - | - | - | - | - | - | - | - | - | - | - | - | - | - | - | - | - | - | - | - | - | - | - | - | - | - | - | - | - | - | - | - | - | - | - | - | - | - | - | - | - | - | - | - | - | -135 |
| Q0RAZ2_FRAAA | - | - | - | - | - | - | - | - | - | - | - | - | - | - | - | - | - | - | - | - | - | - | - | - | - | - | - | - | - | - | - | - | - | - | - | - | - | - | - | - | - | - | - | - | - | - | - | - | - | - | - | - | - | - | - | - | - | - | - | - | - | -122 |
| E3J3F6_FRASU | - | - | - | - | - | - | - | - | - | - | - | - | - | - | - | - | - | - | - | - | - | - | - | - | - | - | - | - | - | - | - | - | - | - | - | - | - | - | - | - | - | - | - | - | - | - | - | - | - | - | - | - | - | - | - | - | - | - | - | - | - | -116 |
| ZP_09168240 | - | - | - | - | - | - | - | - | - | - | - | - | - | - | - | - | - | - | - | - | - | - | - | - | - | - | - | - | - | - | - | - | - | - | - | - | - | - | - | - | - | - | - | - | - | - | - | - | - | - | - | - | - | - | - | - | - | - | - | - | - | -126 |
| C7MDV9_BRAFD | - | - | - | - | - | - | - | - | - | - | - | - | - | - | - | - | - | - | - | - | - | - | - | - | - | - | - | - | - | - | - | - | - | - | - | - | - | - | - | - | - | - | - | - | - | - | - | - | - | - | - | - | - | - | - | - | - | - | - | - | - | -88 |
| ZP_10094989 | - | - | - | - | - | - | - | - | - | - | - | - | - | - | - | - | - | - | - | - | - | - | - | - | - | - | - | - | - | - | - | - | - | - | - | - | - | - | - | - | - | - | - | - | - | - | - | - | - | - | - | - | - | - | - | - | - | - | - | - | - | -88 |
| A4X268_SALTO | - | - | - | - | - | - | - | - | - | - | - | - | - | - | - | - | - | - | - | - | - | - | - | - | - | - | - | - | - | - | - | - | - | - | - | - | - | - | - | - | - | - | - | - | - | - | - | - | - | - | - | - | - | - | - | - | - | - | - | - | - | -120 |
| WP_043162213 | - | - | - | - | - | - | - | - | - | - | - | - | - | - | - | - | - | - | - | - | - | - | - | - | - | - | - | - | - | - | - | - | - | - | - | - | - | - | - | - | - | - | - | - | - | - | - | - | - | - | - | - | - | - | - | - | - | - | - | - | - | -120 |
| Q0BRV8_GRABC | - | - | - | - | - | - | - | - | - | - | - | - | - | - | - | - | - | - | - | - | - | - | - | - | - | - | - | - | - | - | - | - | - | - | - | - | - | - | - | - | - | - | - | - | - | - | - | - | - | - | - | - | - | - | - | - | - | - | - | - | - | -114 |
| A9B2P0_HERA2 | - | - | - | - | - | - | - | - | - | - | - | - | - | - | - | - | - | - | - | - | - | - | - | - | - | - | - | - | - | - | - | - | - | - | - | - | - | - | - | - | - | - | - | - | - | - | - | - | - | - | - | - | - | - | - | - | - | - | - | - | - | -106 |
| A9B3U8_HERA2 | - | - | - | - | - | - | - | - | - | - | - | - | - | - | - | - | - | - | - | - | - | - | - | - | - | - | - | - | - | - | - | - | - | - | - | - | - | - | - | - | - | - | - | - | - | - | - | - | - | - | - | - | - | - | - | - | - | - | - | - | - | -108 |
| A9B2A3_HERA2 | - | - | - | - | - | - | - | - | - | - | - | - | - | - | - | - | - | - | - | - | - | - | - | - | - | - | - | - | - | - | - | - | - | - | - | - | - | - | - | - | - | - | - | - | - | - | - | - | - | - | - | - | - | - | - | - | - | - | - | - | - | -121 |
| D6ZC85_SEGRD | - | - | - | - | - | - | - | - | - | - | - | - | - | - | - | - | - | - | - | - | - | - | - | - | - | - | - | - | - | - | - | - | - | - | - | - | - | - | - | - | - | - | - | - | - | - | - | - | - | - | - | - | - | - | - | - | - | - | - | - | - | -139 |
| E5XTT9_9ACTO | - | - | - | - | - | - | - | - | - | - | - | - | - | - | - | - | - | - | - | - | - | - | - | - | - | - | - | - | - | - | - | - | - | - | - | - | - | - | - | - | - | - | - | - | - | - | - | - | - | - | - | - | - | - | - | - | - | - | - | - | - | -139 |
| E5XTU0_9ACTO | - | - | - | - | - | - | - | - | - | - | - | - | - | - | - | - | - | - | - | - | - | - | - | - | - | - | - | - | - | - | - | - | - | - | - | - | - | - | - | - | - | - | - | - | - | - | - | - | - | - | - | - | - | - | - | - | - | - | - | - | - | -143 |
| D6B0I6_9ACTO | - | - | - | - | - | - | - | - | - | - | - | - | - | - | - | - | - | - | - | - | - | - | - | - | - | - | - | - | - | - | - | - | - | - | - | - | - | - | - | - | - | - | - | - | - | - | - | - | - | - | - | - | - | - | - | - | - | - | - | - | - | -106 |
| D9X719_STRVR | - | - | - | - | - | - | - | - | - | - | - | - | - | - | - | - | - | - | - | - | - | - | - | - | - | - | - | - | - | - | - | - | - | - | - | - | - | - | - | - | - | - | - | - | - | - | - | - | - | - | - | - | - | - | - | - | - | - | - | - | - | -106 |
| H6L010_SAPGL | - | - | - | - | - | - | - | - | - | - | - | - | - | - | - | - | - | - | - | - | - | - | - | - | - | - | - | - | - | - | - | - | - | - | - | - | - | - | - | - | - | - | - | - | - | - | - | - | - | - | - | - | - | - | - | - | - | - | - | - | - | -98 |
| K1ZP83_9BACT | - | - | - | - | - | - | - | - | - | - | - | - | - | - | - | - | - | - | - | - | - | - | - | - | - | - | - | - | - | - | - | - | - | - | - | - | - | - | - | - | - | - | - | - | - | - | - | - | - | - | - | - | - | - | - | - | - | - | - | - | - | -119 |
| ADGB_HUMAN | - | - | - | - | - | - | - | - | - | - | - | - | - | - | - | - | - | - | - | - | - | - | - | - | - | - | - | - | - | - | - | - | - | - | - | - | - | - | - | - | - | - | - | - | - | - | - | - | - | - | - | - | - | - | - | - | - | - | - | - | - | -161 |
| J3QMP3_MOUSE | - | - | - | - | - | - | - | - | - | - | - | - | - | - | - | - | - | - | - | - | - | - | - | - | - | - | - | - | - | - | - | - | - | - | - | - | - | - | - | - | - | - | - | - | - | - | - | - | - | - | - | - | - | - | - | - | - | - | - | - | - | -161 |
| H2XR46_CIOIN | - | - | - | - | - | - | - | - | - | - | - | - | - | - | - | - | - | - | - | - | - | - | - | - | - | - | - | - | - | - | - | - | - | - | - | - | - | - | - | - | - | - | - | - | - | - | - | - | - | - | - | - | - | - | - | - | - | - | - | - | - | -159 |
| CAN7_MOUSE | - | - | - | - | - | - | - | - | - | - | - | - | - | - | - | - | - | - | - | - | - | - | - | - | - | - | - | - | - | - | - | - | - | - | - | - | - | - | - | - | - | - | - | - | - | - | - | - | - | - | - | - | - | - | - | - | - | - | - | - | - | -131 |
| CAN7_HUMAN | - | - | - | - | - | - | - | - | - | - | - | - | - | - | - | - | - | - | - | - | - | - | - | - | - | - | - | - | - | - | - | - | - | - | - | - | - | - | - | - | - | - | - | - | - | - | - | - | - | - | - | - | - | - | - | - | - | - | - | - | - | -131 |
| E1BY84_CHICK | - | - | - | - | - | - | - | - | - | - | - | - | - | - | - | - | - | - | - | - | - | - | - | - | - | - | - | - | - | - | - | - | - | - | - | - | - | - | - | - | - | - | - | - | - | - | - | - | - | - | - | - | - | - | - | - | - | - | - | - | - | -131 |
| H9GX91_DANRE | - | - | - | - | - | - | - | - | - | - | - | - | - | - | - | - | - | - | - | - | - | - | - | - | - | - | - | - | - | - | - | - | - | - | - | - | - | - | - | - | - | - | - | - | - | - | - | - | - | - | - | - | - | - | - | - | - | - | - | - | - | -131 |
| F6SGI6_CIOIN | - | - | - | - | - | - | - | - | - | - | - | - | - | - | - | - | - | - | - | - | - | - | - | - | - | - | - | - | - | - | - | - | - | - | - | - | - | - | - | - | - | - | - | - | - | - | - | - | - | - | - | - | - | - | - | - | - | - | - | - | - | -131 |
| W4Y195_STRPU | - | - | - | - | - | - | - | - | - | - | - | - | - | - | - | - | - | - | - | - | - | - | - | - | - | - | - | - | - | - | - | - | - | - | - | - | - | - | - | - | - | - | - | - | - | - | - | - | - | - | - | - | - | - | - | - | - | - | - | - | - | -105 |
| R7V270_CAPTE | - | - | - | - | - | - | - | - | - | - | - | - | - | - | - | - | - | - | - | - | - | - | - | - | - | - | - | - | - | - | - | - | - | - | - | - | - | - | - | - | - | - | - | - | - | - | - | - | - | - | - | - | - | - | - | - | - | - | - | - | - | -131 |
| G4LXK3_SCHMA | - | - | - | - | - | - | - | - | - | - | - | - | - | - | - | - | - | - | - | - | - | - | - | - | - | - | - | - | - | - | - | - | - | - | - | - | - | - | - | - | - | - | - | - | - | - | - | - | - | - | - | - | - | - | - | - | - | - | - | - | - | -131 |
| T2M2K0_HYDVU | - | - | - | - | - | - | - | - | - | - | - | - | - | - | - | - | - | - | - | - | - | - | - | - | - | - | - | - | - | - | - | - | - | - | - | - | - | - | - | - | - | - | - | - | - | - | - | - | - | - | - | - | - | - | - | - | - | - | - | - | - | -131 |
| B3RQK1_TRIAD | - | - | - | - | - | - | - | - | - | - | - | - | - | - | - | - | - | - | - | - | - | - | - | - | - | - | - | - | - | - | - | - | - | - | - | - | - | - | - | - | - | - | - | - | - | - | - | - | - | - | - | - | - | - | - | - | - | - | - | - | - | -131 |
| I1CS59_RHIO9 | - | - | - | - | - | - | - | - | - | - | - | - | - | - | - | - | - | - | - | - | - | - | - | - | - | - | - | - | - | - | - | - | - | - | - | - | - | - | - | - | - | - | - | - | - | - | - | - | - | - | - | - | - | - | - | - | - | - | - | - | - | -128 |
| I1GER3_AMPQE | - | - | - | - | - | - | - | - | - | - | - | - | - | - | - | - | - | - | - | - | - | - | - | - | - | - | - | - | - | - | - | - | - | - | - | - | - | - | - | - | - | - | - | - | - | - | - | - | - | - | - | - | - | - | - | - | - | - | - | - | - | -131 |
| Q22143_CAEEL | - | - | - | - | - | - | - | - | - | - | - | - | - | - | - | - | - | - | - | - | - | - | - | - | - | - | - | - | - | - | - | - | - | - | - | - | - | - | - | - | - | - | - | - | - | - | - | - | - | - | - | - | - | - | - | - | - | - | - | - | - | -131 |
| L1JZN7_GUITH | - | - | - | - | - | - | - | - | - | - | - | - | - | - | - | - | - | - | - | - | - | - | - | - | - | - | - | - | - | - | - | - | - | - | - | - | - | - | - | - | - | - | - | - | - | - | - | - | - | - | - | - | - | - | - | - | - | - | - | - | - | -132 |
| D0NT34_PHYIT | - | - | - | - | - | - | - | - | - | - | - | - | - | - | - | - | - | - | - | - | - | - | - | - | - | - | - | - | - | - | - | - | - | - | - | - | - | - | - | - | - | - | - | - | - | - | - | - | - | - | - | - | - | - | - | - | - | - | - | - | - | -140 |
| A0A064B913_ASPOZ | - | - | - | - | - | - | - | - | - | - | - | - | - | - | - | - | - | - | - | - | - | - | - | - | - | - | - | - | - | - | - | - | - | - | - | - | - | - | - | - | - | - | - | - | - | - | - | - | - | - | - | - | - | - | - | - | - | - | - | - | - | -139 |
| A8N4X4_COPC7 | - | - | - | - | - | - | - | - | - | - | - | - | - | - | - | - | - | - | - | - | - | - | - | - | - | - | - | - | - | - | - | - | - | - | - | - | - | - | - | - | - | - | - | - | - | - | - | - | - | - | - | - | - | - | - | - | - | - | - | - | - | -155 |
| C0H5K1_PLAF7 | - | - | - | - | - | - | - | - | - | - | - | - | - | - | - | - | - | - | - | - | - | - | - | - | - | - | - | - | - | - | - | - | - | - | - | - | - | - | - | - | - | - | - | - | - | - | - | - | - | - | - | - | - | - | - | - | - | - | - | - | - | -139 |
| K1REZ8_CRAGI | - | - | - | - | - | - | - | - | - | - | - | - | - | - | - | - | - | - | - | - | - | - | - | - | - | - | - | - | - | - | - | - | - | - | - | - | - | - | - | - | - | - | - | - | - | - | - | - | - | - | - | - | - | - | - | - | - | - | - | - | - | -118 |
| F4NTM9_BATDJ | - | - | - | - | - | - | - | - | - | - | - | - | - | - | - | - | - | - | - | - | - | - | - | - | - | - | - | - | - | - | - | - | - | - | - | - | - | - | - | - | - | - | - | - | - | - | - | - | - | - | - | - | - | - | - | - | - | - | - | - | - | -134 |
| Q6F6K5_CAEEL | - | - | - | - | - | - | - | - | - | - | - | - | - | - | - | - | - | - | - | - | - | - | - | - | - | - | - | - | - | - | - | - | - | - | - | - | - | - | - | - | - | - | - | - | - | - | - | - | - | - | - | - | - | - | - | - | - | - | - | - | - | -99 |
| A8HZK8_CHLRE | - | - | - | - | - | - | - | - | - | - | - | - | - | - | - | - | - | - | - | - | - | - | - | - | - | - | - | - | - | - | - | - | - | - | - | - | - | - | - | - | - | - | - | - | - | - | - | - | - | - | - | - | - | - | - | - | - | - | - | - | - | -100 |
| A8J6T6_CHLRE | - | - | - | - | - | - | - | - | - | - | - | - | - | - | - | - | - | - | - | - | - | - | - | - | - | - | - | - | - | - | - | - | - | - | - | - | - | - | - | - | - | - | - | - | - | - | - | - | - | - | - | - | - | - | - | - | - | - | - | - | - | -132 |
| Z9JR51_9MICO | - | - | - | - | - | - | - | - | - | - | - | - | - | - | - | - | - | - | - | - | - | - | - | - | - | - | - | - | - | - | - | - | - | - | - | - | - | - | - | - | - | - | - | - | - | - | - | - | - | - | - | - | - | - | - | - | - | - | - | - | - | -118 |
| F7ZZH9_CELGA | - | - | - | - | - | - | - | - | - | - | - | - | - | - | - | - | - | - | - | - | - | - | - | - | - | - | - | - | - | - | - | - | - | - | - | - | - | - | - | - | - | - | - | - | - | - | - | - | - | - | - | - | - | - | - | - | - | - | - | - | - | -101 |
| Q7NDU2_GLOVI | - | - | - | - | - | - | - | - | - | - | - | - | - | - | - | - | - | - | - | - | - | - | - | - | - | - | - | - | - | - | - | - | - | - | - | - | - | - | - | - | - | - | - | - | - | - | - | - | - | - | - | - | - | - | - | - | - | - | - | - | - | -87 |
| I6AT95_9BACT | - | - | - | - | - | - | - | - | - | - | - | - | - | - | - | - | - | - | - | - | - | - | - | - | - | - | - | - | - | - | - | - | - | - | - | - | - | - | - | - | - | - | - | - | - | - | - | - | - | - | - | - | - | - | - | - | - | - | - | - | - | -100 |
| C7MGJ8_BRAFD | - | - | - | - | - | - | - | - | - | - | - | - | - | - | - | - | - | - | - | - | - | - | - | - | - | - | - | - | - | - | - | - | - | - | - | - | - | - | - | - | - | - | - | - | - | - | - | - | - | - | - | - | - | - | - | - | - | - | - | - | - | -116 |
| D0WP73_9ACTO | - | - | - | - | - | - | - | - | - | - | - | - | - | - | - | - | - | - | - | - | - | - | - | - | - | - | - | - | - | - | - | - | - | - | - | - | - | - | - | - | - | - | - | - | - | - | - | - | - | - | - | - | - | - | - | - | - | - | - | - | - | -110 |
| B0C8L1_ACAM1 | - | - | - | - | - | - | - | - | - | - | - | - | - | - | - | - | - | - | - | - | - | - | - | - | - | - | - | - | - | - | - | - | - | - | - | - | - | - | - | - | - | - | - | - | - | - | - | - | - | - | - | - | - | - | - | - | - | - | - | - | - | -107 |
| B3E5A8_GEOLS | - | - | - | - | - | - | - | - | - | - | - | - | - | - | - | - | - | - | - | - | - | - | - | - | - | - | - | - | - | - | - | - | - | - | - | - | - | - | - | - | - | - | - | - | - | - | - | - | - | - | - | - | - | - | - | - | - | - | - | - | - | -101 |
| D4YQ94_9MICO | - | - | - | - | - | - | - | - | - | - | - | - | - | - | - | - | - | - | - | - | - | - | - | - | - | - | - | - | - | - | - | - | - | - | - | - | - | - | - | - | - | - | - | - | - | - | - | - | - | - | - | - | - | - | - | - | - | - | - | - | - | -101 |
| PAPA_CARPA | - | - | - | - | - | - | - | - | - | - | - | - | - | - | - | - | - | - | - | - | - | - | - | - | - | - | - | - | - | - | - | - | - | - | - | - | - | - | - | - | - | - | - | - | - | - | - | - | - | - | - | - | - | - | - | - | - | - | - | - | - | -93 |
| RIM13_YEAST | - | - | - | - | - | - | - | - | - | - | - | - | - | - | - | - | - | - | - | - | - | - | - | - | - | - | - | - | - | - | - | - | - | - | - | - | - | - | - | - | - | - | - | - | - | - | - | - | - | - | - | - | - | - | - | - | - | - | - | - | - | -133 |

| I |  |  |  |  |  |  |  |  |  |  |  |  |  |  |  |  |  |  |  |  |  |  |  |  |  |  |  |  |  |  |  |  |  |  |  |  |  |  |  |  |  |  |  |  |  |  |  |  |  |  |  |  |  |  |  |  |  |  |  |  |  |  |
| --- | --- | --- | --- | --- | --- | --- | --- | --- | --- | --- | --- | --- | --- | --- | --- | --- | --- | --- | --- | --- | --- | --- | --- | --- | --- | --- | --- | --- | --- | --- | --- | --- | --- | --- | --- | --- | --- | --- | --- | --- | --- | --- | --- | --- | --- | --- | --- | --- | --- | --- | --- | --- | --- | --- | --- | --- | --- | --- | --- | --- | --- | --- |
| Q9N4B1_CAEEL | - | - | - | - | - | - | - | - | - | - | - | - | - | - | - | - | - | - | - | - | - | - | - | - | - | - | - | - | - | G | T | T | A | E | A | L | - | - | - | - | E | D | F | T | G | - | - | - | - | - | - | - | - | - | - | - | - | - | - | - | - | -135 |
| Q8IAA8_CAEEL | - | - | - | - | - | - | - | - | - | - | - | - | - | - | - | - | - | - | - | - | - | - | - | - | - | - | - | - | - | G | T | T | A | E | A | L | - | - | - | - | E | D | F | T | G | - | - | - | - | - | - | - | - | - | - | - | - | - | - | - | - | -135 |
| CAN_CAEEL | - | - | - | - | - | - | - | - | - | - | - | - | - | - | - | - | - | - | - | - | - | - | - | - | - | - | - | - | - | G | T | T | S | E | A | L | - | - | - | - | E | D | M | T | G | - | - | - | - | - | - | - | - | - | - | - | - | - | - | - | - | -135 |
| Q9U2B2_CAEEL | - | - | - | - | - | - | - | - | - | - | - | - | - | - | - | - | - | - | - | - | - | - | - | - | - | - | - | - | - | P | Y | T | E | Q | A | L | W | Q | A | S | A | S | F | T | G | - | - | - | - | - | - | - | - | - | - | - | - | - | - | - | - | -139 |
| Q9N4B2_CAEEL | - | - | - | - | - | - | - | - | - | - | - | - | - | - | - | - | - | - | - | - | - | - | - | - | - | - | - | - | - | G | T | T | T | E | A | L | - | - | - | - | E | D | F | T | G | - | - | - | - | - | - | - | - | - | - | - | - | - | - | - | - | -135 |
| C3Y6P6_BRAFL | - | - | - | - | - | - | - | - | - | - | - | - | - | - | - | - | - | - | - | - | - | - | - | - | - | - | - | - | - | G | H | T | S | D | A | L | - | - | - | - | V | D | F | T | G | - | - | - | - | - | - | - | - | - | - | - | - | - | - | - | - | -141 |
| C3Y6N1_BRAFL | - | - | - | - | - | - | - | - | - | - | - | - | - | - | - | - | - | - | - | - | - | - | - | - | - | - | - | - | - | G | H | T | S | D | A | L | - | - | - | - | V | D | F | T | G | - | - | - | - | - | - | - | - | - | - | - | - | - | - | - | - | -142 |
| CANA_DROME | - | - | - | - | - | - | - | - | - | - | - | - | - | - | - | - | - | - | - | - | - | - | - | - | - | - | - | - | - | G | S | T | C | E | A | M | - | - | - | - | E | D | F | T | G | - | - | - | - | - | - | - | - | - | - | - | - | - | - | - | - | -135 |
| CANB_DROME | - | - | - | - | - | - | - | - | - | - | - | - | - | - | - | - | - | - | - | - | - | - | - | - | - | - | - | - | - | G | T | T | C | E | A | M | - | - | - | - | E | D | F | T | G | - | - | - | - | - | - | - | - | - | - | - | - | - | - | - | - | -135 |
| X1Y3H71_ANODA | - | - | - | - | - | - | - | - | - | - | - | - | - | - | - | - | - | - | - | - | - | - | - | - | - | - | - | - | - | G | T | T | C | E | A | M | - | - | - | - | E | D | F | T | G | - | - | - | - | - | - | - | - | - | - | - | - | - | - | - | - | -135 |
| Q5IW12_GECLA | - | - | - | - | - | - | - | - | - | - | - | - | - | - | - | - | - | - | - | - | - | - | - | - | - | - | - | - | - | G | T | T | C | E | A | M | - | - | - | - | E | D | F | T | G | - | - | - | - | - | - | - | - | - | - | - | - | - | - | - | - | -135 |
| K1Q056_CRAGI | - | - | - | - | - | - | - | - | - | - | - | - | - | - | - | - | - | - | - | - | - | - | - | - | - | - | - | - | - | G | S | T | C | E | A | M | - | - | - | - | V | D | F | T | G | - | - | - | - | - | - | - | - | - | - | - | - | - | - | - | - | -135 |
| G4V6C0_SCHMA | - | - | - | - | - | - | - | - | - | - | - | - | - | - | - | - | - | - | - | - | - | - | - | - | - | - | - | - | - | G | C | T | A | E | A | M | - | - | - | - | E | D | F | T | G | - | - | - | - | - | - | - | - | - | - | - | - | - | - | - | - | -139 |
| R7ULX9_CAPTE | - | - | - | - | - | - | - | - | - | - | - | - | - | - | - | - | - | - | - | - | - | - | - | - | - | - | - | - | - | G | L | T | S | E | A | L | - | - | - | - | T | D | F | T | G | - | - | - | - | - | - | - | - | - | - | - | - | - | - | - | - | -135 |
| W4Z5Q7_STRPU | - | - | - | - | - | - | - | - | - | - | - | - | - | - | - | - | - | - | - | - | - | - | - | - | - | - | - | - | - | G | N | V | I | E | A | M | - | - | - | - | E | D | F | T | G | - | - | - | - | - | - | - | - | - | - | - | - | - | - | - | - | -132 |
| R7VIB6_CAPTE | - | - | - | - | - | - | - | - | - | - | - | - | - | - | - | - | - | - | - | - | - | - | - | - | - | - | - | - | - | G | S | S | S | E | A | M | - | - | - | - | E | D | F | T | G | - | - | - | - | - | - | - | - | - | - | - | - | - | - | - | - | -138 |
| T2MJ45_HYDVU | - | - | - | - | - | - | - | - | - | - | - | - | - | - | - | - | - | - | - | - | - | - | - | - | - | - | - | - | - | G | Q | T | S | E | A | M | - | - | - | - | E | D | F | T | G | - | - | - | - | - | - | - | - | - | - | - | - | - | - | - | - | -135 |
| R7UAB6_CAPTE | - | - | - | - | - | - | - | - | - | - | - | - | - | - | - | - | - | - | - | - | - | - | - | - | - | - | - | - | - | G | S | T | C | E | A | M | - | - | - | - | E | D | F | T | G | - | - | - | - | - | - | - | - | - | - | - | - | - | - | - | - | -135 |
| C3ZKN8_BRAFL | - | - | - | - | - | - | - | - | - | - | - | - | - | - | - | - | - | - | - | - | - | - | - | - | - | - | - | - | - | G | S | V | T | E | A | M | - | - | - | - | E | D | F | T | G | - | - | - | - | - | - | - | - | - | - | - | - | - | - | - | - | -136 |
| B3RJS5_TRIAD | - | - | - | - | - | - | - | - | - | - | - | - | - | - | - | - | - | - | - | - | - | - | - | - | - | - | - | - | - | G | H | S | S | E | A | L | - | - | - | - | E | D | F | T | G | - | - | - | - | - | - | - | - | - | - | - | - | - | - | - | - | -135 |
| H2YVF0_CIOSA | - | - | - | - | - | - | - | - | - | - | - | - | - | - | - | - | - | - | - | - | - | - | - | - | - | - | - | - | - | G | S | T | T | E | A | M | - | - | - | - | E | D | F | T | G | - | - | - | - | - | - | - | - | - | - | - | - | - | - | - | - | -135 |
| G4VAG2_SCHMA | - | - | - | - | - | - | - | - | - | - | - | - | - | - | - | - | - | - | - | - | - | - | - | - | - | - | - | - | - | G | S | Q | S | E | A | M | - | - | - | - | E | D | L | T | G | - | - | - | - | - | - | - | - | - | - | - | - | - | - | - | - | -138 |
| Smp_157500 | - | - | - | - | - | - | - | - | - | - | - | - | - | - | - | - | - | - | - | - | - | - | - | - | - | - | - | - | - | G | F | Q | S | E | A | M | - | - | - | - | E | D | F | T | G | - | - | - | - | - | - | - | - | - | - | - | - | - | - | - | - | -138 |
| Q7SYA9_DANRE | - | - | - | - | - | - | - | - | - | - | - | - | - | - | - | - | - | - | - | - | - | - | - | - | - | - | - | - | - | G | S | T | T | E | G | F | - | - | - | - | E | D | F | T | G | - | - | - | - | - | - | - | - | - | - | - | - | - | - | - | - | -135 |
| A5PMP1_DANRE | - | - | - | - | - | - | - | - | - | - | - | - | - | - | - | - | - | - | - | - | - | - | - | - | - | - | - | - | - | G | S | T | T | E | G | F | - | - | - | - | E | D | F | T | G | - | - | - | - | - | - | - | - | - | - | - | - | - | - | - | - | -135 |
| CANX_CHICK | - | - | - | - | - | - | - | - | - | - | - | - | - | - | - | - | - | - | - | - | - | - | - | - | - | - | - | - | - | G | S | T | T | E | G | F | - | - | - | - | E | D | F | T | G | - | - | - | - | - | - | - | - | - | - | - | - | - | - | - | - | -135 |
| CAN1_HUMAN | - | - | - | - | - | - | - | - | - | - | - | - | - | - | - | - | - | - | - | - | - | - | - | - | - | - | - | - | - | G | S | T | S | E | G | F | - | - | - | - | E | D | F | T | G | - | - | - | - | - | - | - | - | - | - | - | - | - | - | - | - | -135 |
| CAN1_MOUSE | - | - | - | - | - | - | - | - | - | - | - | - | - | - | - | - | - | - | - | - | - | - | - | - | - | - | - | - | - | G | C | T | S | E | A | F | - | - | - | - | E | D | F | T | G | - | - | - | - | - | - | - | - | - | - | - | - | - | - | - | - | -135 |
| O42133_CHICK | - | - | - | - | - | - | - | - | - | - | - | - | - | - | - | - | - | - | - | - | - | - | - | - | - | - | - | - | - | G | S | T | S | E | G | F | - | - | - | - | E | D | F | T | G | - | - | - | - | - | - | - | - | - | - | - | - | - | - | - | - | -135 |
| Q7ZUR1_DANRE | - | - | - | - | - | - | - | - | - | - | - | - | - | - | - | - | - | - | - | - | - | - | - | - | - | - | - | - | - | G | S | T | C | E | G | F | - | - | - | - | E | D | F | T | G | - | - | - | - | - | - | - | - | - | - | - | - | - | - | - | - | -135 |
| E9QFN0_DANRE | - | - | - | - | - | - | - | - | - | - | - | - | - | - | - | - | - | - | - | - | - | - | - | - | - | - | - | - | - | G | S | T | S | E | G | F | - | - | - | - | E | D | F | T | G | - | - | - | - | - | - | - | - | - | - | - | - | - | - | - | - | -135 |
| CAN2_HUMAN | - | - | - | - | - | - | - | - | - | - | - | - | - | - | - | - | - | - | - | - | - | - | - | - | - | - | - | - | - | G | A | T | T | E | G | F | - | - | - | - | E | D | F | T | G | - | - | - | - | - | - | - | - | - | - | - | - | - | - | - | - | -135 |
| CAN2_MOUSE | - | - | - | - | - | - | - | - | - | - | - | - | - | - | - | - | - | - | - | - | - | - | - | - | - | - | - | - | - | G | A | T | T | E | G | F | - | - | - | - | E | D | F | T | G | - | - | - | - | - | - | - | - | - | - | - | - | - | - | - | - | -135 |
| CAN2_CHICK | - | - | - | - | - | - | - | - | - | - | - | - | - | - | - | - | - | - | - | - | - | - | - | - | - | - | - | - | - | G | T | T | T | E | G | F | - | - | - | - | E | D | F | T | G | - | - | - | - | - | - | - | - | - | - | - | - | - | - | - | - | -135 |
| Q6DFZ8_DANRE | - | - | - | - | - | - | - | - | - | - | - | - | - | - | - | - | - | - | - | - | - | - | - | - | - | - | - | - | - | G | T | T | T | E | G | F | - | - | - | - | E | D | F | T | G | - | - | - | - | - | - | - | - | - | - | - | - | - | - | - | - | -135 |
| Q504F5_DANRE | - | - | - | - | - | - | - | - | - | - | - | - | - | - | - | - | - | - | - | - | - | - | - | - | - | - | - | - | - | G | S | T | S | E | G | F | - | - | - | - | E | D | F | T | G | - | - | - | - | - | - | - | - | - | - | - | - | - | - | - | - | -135 |
| Q5BLH5_DANRE | - | - | - | - | - | - | - | - | - | - | - | - | - | - | - | - | - | - | - | - | - | - | - | - | - | - | - | - | - | G | S | T | T | E | G | F | - | - | - | - | E | D | F | T | G | - | - | - | - | - | - | - | - | - | - | - | - | - | - | - | - | -135 |
| Q4VBH3_DANRE | - | - | - | - | - | - | - | - | - | - | - | - | - | - | - | - | - | - | - | - | - | - | - | - | - | - | - | - | - | G | S | T | T | E | G | F | - | - | - | - | E | D | F | T | G | - | - | - | - | - | - | - | - | - | - | - | - | - | - | - | - | -135 |
| CAN8_RAT | - | - | - | - | - | - | - | - | - | - | - | - | - | - | - | - | - | - | - | - | - | - | - | - | - | - | - | - | - | G | S | T | I | E | G | F | - | - | - | - | E | D | F | T | G | - | - | - | - | - | - | - | - | - | - | - | - | - | - | - | - | -135 |
| CAN8_MOUSE | - | - | - | - | - | - | - | - | - | - | - | - | - | - | - | - | - | - | - | - | - | - | - | - | - | - | - | - | - | G | S | T | I | E | G | F | - | - | - | - | E | D | F | T | G | - | - | - | - | - | - | - | - | - | - | - | - | - | - | - | - | -135 |
| CAN8_HUMAN | - | - | - | - | - | - | - | - | - | - | - | - | - | - | - | - | - | - | - | - | - | - | - | - | - | - | - | - | - | G | S | T | V | E | G | F | - | - | - | - | E | D | F | T | G | - | - | - | - | - | - | - | - | - | - | - | - | - | - | - | - | -135 |
| XP_426117 | - | - | - | - | - | - | - | - | - | - | - | - | - | - | - | - | - | - | - | - | - | - | - | - | - | - | - | - | - | G | S | T | I | E | G | F | - | - | - | - | E | D | F | T | G | - | - | - | - | - | - | - | - | - | - | - | - | - | - | - | - | -135 |
| CAN9_MOUSE | - | - | - | - | - | - | - | - | - | - | - | - | - | - | - | - | - | - | - | - | - | - | - | - | - | - | - | - | - | G | S | A | I | E | A | M | - | - | - | - | E | D | F | T | G | - | - | - | - | - | - | - | - | - | - | - | - | - | - | - | - | -137 |
| CAN9_HUMAN | - | - | - | - | - | - | - | - | - | - | - | - | - | - | - | - | - | - | - | - | - | - | - | - | - | - | - | - | - | G | S | A | I | E | A | M | - | - | - | - | E | D | F | T | G | - | - | - | - | - | - | - | - | - | - | - | - | - | - | - | - | -135 |
| E1C6M3_CHICK | - | - | - | - | - | - | - | - | - | - | - | - | - | - | - | - | - | - | - | - | - | - | - | - | - | - | - | - | - | G | S | T | I | E | A | M | - | - | - | - | E | D | F | T | G | - | - | - | - | - | - | - | - | - | - | - | - | - | - | - | - | -135 |
| Q6DG44_DANRE | - | - | - | - | - | - | - | - | - | - | - | - | - | - | - | - | - | - | - | - | - | - | - | - | - | - | - | - | - | G | S | T | M | E | A | M | - | - | - | - | E | D | F | T | G | - | - | - | - | - | - | - | - | - | - | - | - | - | - | - | - | -135 |
| CAN3_HUMAN | - | - | - | - | - | - | - | - | - | - | - | - | - | - | - | - | - | - | - | - | - | - | - | - | - | - | - | - | - | G | N | T | T | E | A | M | - | - | - | - | E | D | F | T | G | - | - | - | - | - | - | - | - | - | - | - | - | - | - | - | - | -135 |
| CAN3_MOUSE | - | - | - | - | - | - | - | - | - | - | - | - | - | - | - | - | - | - | - | - | - | - | - | - | - | - | - | - | - | G | N | T | T | E | A | M | - | - | - | - | E | D | F | T | G | - | - | - | - | - | - | - | - | - | - | - | - | - | - | - | - | -135 |
| CAN3_CHICK | - | - | - | - | - | - | - | - | - | - | - | - | - | - | - | - | - | - | - | - | - | - | - | - | - | - | - | - | - | G | N | T | T | E | A | M | - | - | - | - | E | D | F | T | G | - | - | - | - | - | - | - | - | - | - | - | - | - | - | - | - | -135 |
| Q66HU4_DANRE | - | - | - | - | - | - | - | - | - | - | - | - | - | - | - | - | - | - | - | - | - | - | - | - | - | - | - | - | - | G | N | T | A | E | G | M | - | - | - | - | E | D | F | T | G | - | - | - | - | - | - | - | - | - | - | - | - | - | - | - | - | -135 |
| F1R632_DANRE | - | - | - | - | - | - | - | - | - | - | - | - | - | - | - | - | - | - | - | - | - | - | - | - | - | - | - | - | - | G | N | S | L | E | A | M | - | - | - | - | E | D | F | T | G | - | - | - | - | - | - | - | - | - | - | - | - | - | - | - | - | -135 |
| CAN11_HUMAN | - | - | - | - | - | - | - | - | - | - | - | - | - | - | - | - | - | - | - | - | - | - | - | - | - | - | - | - | - | G | S | T | M | E | G | L | - | - | - | - | E | D | F | T | G | - | - | - | - | - | - | - | - | - | - | - | - | - | - | - | - | -135 |
| CAN11_MOUSE | - | - | - | - | - | - | - | - | - | - | - | - | - | - | - | - | - | - | - | - | - | - | - | - | - | - | - | - | - | G | S | T | L | E | G | L | - | - | - | - | E | D | F | T | G | - | - | - | - | - | - | - | - | - | - | - | - | - | - | - | - | -135 |
| CAN12_MOUSE | - | - | - | - | - | - | - | - | - | - | - | - | - | - | - | - | - | - | - | - | - | - | - | - | - | - | - | - | - | G | H | M | N | E | A | F | - | - | - | - | V | D | F | T | G | - | - | - | - | - | - | - | - | - | - | - | - | - | - | - | - | -135 |
| CAN12_HUMAN | - | - | - | - | - | - | - | - | - | - | - | - | - | - | - | - | - | - | - | - | - | - | - | - | - | - | - | - | - | G | H | M | N | E | A | F | - | - | - | - | V | D | F | T | G | - | - | - | - | - | - | - | - | - | - | - | - | - | - | - | - | -135 |
| ENSSARP00000008135 | - | - | - | - | - | - | - | - | - | - | - | - | - | - | - | - | - | - | - | - | - | - | - | - | - | - | - | - | - | X | X | X | X | X | X | X | - | - | - | - | X | X | X | X | X | - | - | - | - | - | - | - | - | - | - | - | - | - | - | - | - | -135 |
| ENSSARP00000010553 | - | - | - | - | - | - | - | - | - | - | - | - | - | - | - | - | - | - | - | - | - | - | - | - | - | - | - | - | - | X | X | X | X | X | X | X | - | - | - | - | X | X | X | X | X | - | - | - | - | - | - | - | - | - | - | - | - | - | - | - | - | -134 |
| A7E2G3_DANRE | - | - | - | - | - | - | - | - | - | - | - | - | - | - | - | - | - | - | - | - | - | - | - | - | - | - | - | - | - | G | I | I | S | E | G | M | - | - | - | - | E | D | F | T | G | - | - | - | - | - | - | - | - | - | - | - | - | - | - | - | - | -135 |
| Q6NW69_DANRE | - | - | - | - | - | - | - | - | - | - | - | - | - | - | - | - | - | - | - | - | - | - | - | - | - | - | - | - | - | G | F | P | H | E | A | M | - | - | - | - | V | D | M | T | G | - | - | - | - | - | - | - | - | - | - | - | - | - | - | - | - | -135 |
| CAN13_HUMAN | - | - | - | - | - | - | - | - | - | - | - | - | - | - | - | - | - | - | - | - | - | - | - | - | - | - | - | - | - | G | F | L | E | D | A | L | - | - | - | - | V | D | L | T | G | - | - | - | - | - | - | - | - | - | - | - | - | - | - | - | - | -136 |
| CAN13_MOUSE | - | - | - | - | - | - | - | - | - | - | - | - | - | - | - | - | - | - | - | - | - | - | - | - | - | - | - | - | - | G | Y | L | P | D | A | L | - | - | - | - | V | D | L | T | G | - | - | - | - | - | - | - | - | - | - | - | - | - | - | - | - | -138 |
| E1BRR5_CHICK | - | - | - | - | - | - | - | - | - | - | - | - | - | - | - | - | - | - | - | - | - | - | - | - | - | - | - | - | - | G | Y | I | S | D | A | L | - | - | - | - | V | D | F | T | G | - | - | - | - | - | - | - | - | - | - | - | - | - | - | - | - | -135 |
| E7F0H3_DANRE | - | - | - | - | - | - | - | - | - | - | - | - | - | - | - | - | - | - | - | - | - | - | - | - | - | - | - | - | - | G | F | I | S | E | A | L | - | - | - | - | M | D | F | T | G | - | - | - | - | - | - | - | - | - | - | - | - | - | - | - | - | -136 |
| Q1RLS9_DANRE | - | - | - | - | - | - | - | - | - | - | - | - | - | - | - | - | - | - | - | - | - | - | - | - | - | - | - | - | - | G | N | I | S | E | A | L | - | - | - | - | M | D | F | T | G | - | - | - | - | - | - | - | - | - | - | - | - | - | - | - | - | -135 |
| F1Q9Z1_DANRE | - | - | - | - | - | - | - | - | - | - | - | - | - | - | - | - | - | - | - | - | - | - | - | - | - | - | - | - | - | G | R | V | S | E | A | L | - | - | - | - | L | D | F | T | G | - | - | - | - | - | - | - | - | - | - | - | - | - | - | - | - | -136 |
| A4QNV6_DANRE | - | - | - | - | - | - | - | - | - | - | - | - | - | - | - | - | - | - | - | - | - | - | - | - | - | - | - | - | - | G | V | V | S | E | A | L | - | - | - | - | L | D | F | T | G | - | - | - | - | - | - | - | - | - | - | - | - | - | - | - | - | -136 |
| D6W573_HUMAN | - | - | - | - | - | - | - | - | - | - | - | - | - | - | - | - | - | - | - | - | - | - | - | - | - | - | - | - | - | G | Q | V | S | E | A | L | - | - | - | - | V | D | F | T | G | - | - | - | - | - | - | - | - | - | - | - | - | - | - | - | - | -136 |
| R7UBC6_CAPTE | - | - | - | - | - | - | - | - | - | - | - | - | - | - | - | - | - | - | - | - | - | - | - | - | - | - | - | - | - | N | L | L | I | D | A | L | - | - | - | - | H | D | F | T | G | - | - | - | - | - | - | - | - | - | - | - | - | - | - | - | - | -140 |
| K1RCZ2_CRAGI | - | - | - | - | - | - | - | - | - | - | - | - | - | - | - | - | - | - | - | - | - | - | - | - | - | - | - | - | - | G | N | V | A | D | A | L | - | - | - | - | T | D | F | T | G | - | - | - | - | - | - | - | - | - | - | - | - | - | - | - | - | -142 |
| Q5IW13_GECLA | - | - | - | - | - | - | - | - | - | - | - | - | - | - | - | - | - | - | - | - | - | - | - | - | - | - | - | - | - | G | N | I | N | E | S | M | - | - | - | - | V | D | L | T | G | - | - | - | - | - | - | - | - | - | - | - | - | - | - | - | - | -135 |
| R7UUX7_CAPTE | - | - | - | - | - | - | - | - | - | - | - | - | - | - | - | - | - | - | - | - | - | - | - | - | - | - | - | - | - | G | H | I | N | D | S | L | - | - | - | - | V | D | F | T | G | - | - | - | - | - | - | - | - | - | - | - | - | - | - | - | - | -138 |
| K1Q505_CRAGI | - | - | - | - | - | - | - | - | - | - | - | - | - | - | - | - | - | - | - | - | - | - | - | - | - | - | - | - | - | G | K | L | Q | D | S | V | - | - | - | - | V | D | L | T | G | - | - | - | - | - | - | - | - | - | - | - | - | - | - | - | - | -136 |
| R7UD02_CAPTE | - | - | - | - | - | - | - | - | - | - | - | - | - | - | - | - | - | - | - | - | - | - | - | - | - | - | - | - | - | G | F | S | H | E | A | L | - | - | - | - | V | D | M | T | G | - | - | - | - | - | - | - | - | - | - | - | - | - | - | - | - | -136 |
| CAN6_HUMAN | - | - | - | - | - | - | - | - | - | - | - | - | - | - | - | - | - | - | - | - | - | - | - | - | - | - | - | - | - | L | T | I | T | D | I | I | - | - | - | - | V | D | F | T | G | - | - | - | - | - | - | - | - | - | - | - | - | - | - | - | - | -141 |
| CAN6_MOUSE | - | - | - | - | - | - | - | - | - | - | - | - | - | - | - | - | - | - | - | - | - | - | - | - | - | - | - | - | - | L | T | I | T | D | I | I | - | - | - | - | M | D | F | T | G | - | - | - | - | - | - | - | - | - | - | - | - | - | - | - | - | -141 |
| E1C312_CHICK | - | - | - | - | - | - | - | - | - | - | - | - | - | - | - | - | - | - | - | - | - | - | - | - | - | - | - | - | - | G | S | A | A | D | A | I | - | - | - | - | V | D | F | T | G | - | - | - | - | - | - | - | - | - | - | - | - | - | - | - | - | -141 |
| E7FDN3_DANRE | - | - | - | - | - | - | - | - | - | - | - | - | - | - | - | - | - | - | - | - | - | - | - | - | - | - | - | - | - | G | N | T | A | E | A | L | - | - | - | - | V | D | F | T | G | - | - | - | - | - | - | - | - | - | - | - | - | - | - | - | - | -142 |
| CAN5_HUMAN | - | - | - | - | - | - | - | - | - | - | - | - | - | - | - | - | - | - | - | - | - | - | - | - | - | - | - | - | - | G | N | T | A | D | A | L | - | - | - | - | V | D | F | T | G | - | - | - | - | - | - | - | - | - | - | - | - | - | - | - | - | -137 |
| CAN5_MOUSE | - | - | - | - | - | - | - | - | - | - | - | - | - | - | - | - | - | - | - | - | - | - | - | - | - | - | - | - | - | G | N | T | A | D | A | L | - | - | - | - | V | D | F | T | G | - | - | - | - | - | - | - | - | - | - | - | - | - | - | - | - | -141 |
| E1C292_CHICK | - | - | - | - | - | - | - | - | - | - | - | - | - | - | - | - | - | - | - | - | - | - | - | - | - | - | - | - | - | G | N | T | A | D | A | L | - | - | - | - | V | D | F | T | G | - | - | - | - | - | - | - | - | - | - | - | - | - | - | - | - | -141 |
| A0JMG3_DANRE | - | - | - | - | - | - | - | - | - | - | - | - | - | - | - | - | - | - | - | - | - | - | - | - | - | - | - | - | - | G | N | T | A | D | A | L | - | - | - | - | V | D | F | T | G | - | - | - | - | - | - | - | - | - | - | - | - | - | - | - | - | -141 |
| E7F7F1_DANRE | - | - | - | - | - | - | - | - | - | - | - | - | - | - | - | - | - | - | - | - | - | - | - | - | - | - | - | - | - | G | N | T | A | D | A | L | - | - | - | - | V | D | F | T | G | - | - | - | - | - | - | - | - | - | - | - | - | - | - | - | - | -141 |
| XP_006824412 | - | - | - | - | - | - | - | - | - | - | - | - | - | - | - | - | - | - | - | - | - | - | - | - | - | - | - | - | - | G | N | T | A | D | A | L | - | - | - | - | V | D | F | T | G | - | - | - | - | - | - | - | - | - | - | - | - | - | - | - | - | -135 |
| W4YJN2_STRPU | - | - | - | - | - | - | - | - | - | - | - | - | - | - | - | - | - | - | - | - | - | - | - | - | - | - | - | - | - | G | N | T | A | D | A | L | - | - | - | - | V | D | F | T | G | - | - | - | - | - | - | - | - | - | - | - | - | - | - | - | - | -141 |
| C3YJZ3_BRAFL | - | - | - | - | - | - | - | - | - | - | - | - | - | - | - | - | - | - | - | - | - | - | - | - | - | - | - | - | - | G | N | T | L | D | A | L | - | - | - | - | V | D | F | T | G | - | - | - | - | - | - | - | - | - | - | - | - | - | - | - | - | -141 |
| T2MIT4_HYDVU | - | - | - | - | - | - | - | - | - | - | - | - | - | - | - | - | - | - | - | - | - | - | - | - | - | - | - | - | - | G | N | P | A | D | A | L | - | - | - | - | V | D | F | T | G | - | - | - | - | - | - | - | - | - | - | - | - | - | - | - | - | -141 |
| XP_002154722 | - | - | - | - | - | - | - | - | - | - | - | - | - | - | - | - | - | - | - | - | - | - | - | - | - | - | - | - | - | G | N | P | G | D | A | L | - | - | - | - | V | D | F | T | G | - | - | - | - | - | - | - | - | - | - | - | - | - | - | - | - | -105 |
| R7VLA7_CAPTE | - | - | - | - | - | - | - | - | - | - | - | - | - | - | - | - | - | - | - | - | - | - | - | - | - | - | - | - | - | G | E | L | A | E | A | L | - | - | - | - | E | D | F | T | G | - | - | - | - | - | - | - | - | - | - | - | - | - | - | - | - | -141 |
| K1R1C6_CRAGI | - | - | - | - | - | - | - | - | - | - | - | - | - | - | - | - | - | - | - | - | - | - | - | - | - | - | - | - | - | G | E | L | S | E | A | L | - | - | - | - | E | D | F | T | G | - | - | - | - | - | - | - | - | - | - | - | - | - | - | - | - | -141 |
| B3RIE5_TRIAD | - | - | - | - | - | - | - | - | - | - | - | - | - | - | - | - | - | - | - | - | - | - | - | - | - | - | - | - | - | G | N | T | A | D | A | L | - | - | - | - | V | D | F | T | G | - | - | - | - | - | - | - | - | - | - | - | - | - | - | - | - | -140 |
| K1PW75_CRAGI | - | - | - | - | - | - | - | - | - | - | - | - | - | - | - | - | - | - | - | - | - | - | - | - | - | - | - | - | - | G | Q | A | K | D | A | M | - | - | - | - | V | D | M | T | G | - | - | - | - | - | - | - | - | - | - | - | - | - | - | - | - | -139 |
| K1RSB8_CRAGI | - | - | - | - | - | - | - | - | - | - | - | - | - | - | - | - | - | - | - | - | - | - | - | - | - | - | - | - | - | G | K | A | K | D | A | M | - | - | - | - | V | D | M | T | G | - | - | - | - | - | - | - | - | - | - | - | - | - | - | - | - | -139 |
| K1RWI3_CRAGI | - | - | - | - | - | - | - | - | - | - | - | - | - | - | - | - | - | - | - | - | - | - | - | - | - | - | - | - | - | G | H | A | R | D | A | L | - | - | - | - | V | N | M | T | G | - | - | - | - | - | - | - | - | - | - | - | - | - | - | - | - | -141 |
| R7VGC2_CAPTE | - | - | - | - | - | - | - | - | - | - | - | - | - | - | - | - | - | - | - | - | - | - | - | - | - | - | - | - | - | G | H | T | A | D | A | L | - | - | - | - | V | D | F | T | G | - | - | - | - | - | - | - | - | - | - | - | - | - | - | - | - | -140 |
| K1QU26_CRAGI | - | - | - | - | - | - | - | - | - | - | - | - | - | - | - | - | - | - | - | - | - | - | - | - | - | - | - | - | - | G | R | S | A | D | A | L | - | - | - | - | V | D | F | T | G | - | - | - | - | - | - | - | - | - | - | - | - | - | - | - | - | -142 |
| CAN5_CAEEL | - | - | - | - | - | - | - | - | - | - | - | - | - | - | - | - | - | - | - | - | - | - | - | - | - | - | - | - | - | G | H | L | S | D | A | L | - | - | - | - | Q | D | V | S | G | - | - | - | - | - | - | - | - | - | - | - | - | - | - | - | - | -139 |
| I1FGV4_AMPQE | - | - | - | - | - | - | - | - | - | - | - | - | - | - | - | - | - | - | - | - | - | - | - | - | - | - | - | - | - | G | K | T | S | E | A | L | - | - | - | - | T | D | F | T | G | - | - | - | - | - | - | - | - | - | - | - | - | - | - | - | - | -135 |
| K1PHX3_CRAGI | - | - | - | - | - | - | - | - | - | - | - | - | - | - | - | - | - | - | - | - | - | - | - | - | - | - | - | - | - | G | L | T | N | W | A | L | - | - | - | - | Q | D | L | T | G | - | - | - | - | - | - | - | - | - | - | - | - | - | - | - | - | -135 |
| B3RNL8_TRIAD | - | - | - | - | - | - | - | - | - | - | - | - | - | - | - | - | - | - | - | - | - | - | - | - | - | - | - | - | - | G | V | N | W | E | A | L | - | - | - | - | E | T | F | T | G | - | - | - | - | - | - | - | - | - | - | - | - | - | - | - | - | -137 |
| X1Y3H7_ANODA | - | - | - | - | - | - | - | - | - | - | - | - | - | - | - | - | - | - | - | - | - | - | - | - | - | - | - | - | - | G | T | G | R | E | G | M | - | - | - | - | V | N | L | T | G | - | - | - | - | - | - | - | - | - | - | - | - | - | - | - | - | -139 |
| R7TWH9_CAPTE | - | - | - | - | - | - | - | - | - | - | - | - | - | - | - | - | - | - | - | - | - | - | - | - | - | - | - | - | - | G | Q | T | M | D | A | L | - | - | - | - | V | D | L | T | G | - | - | - | - | - | - | - | - | - | - | - | - | - | - | - | - | -139 |
| K1R5E8_CRAGI | - | - | - | - | - | - | - | - | - | - | - | - | - | - | - | - | - | - | - | - | - | - | - | - | - | - | - | - | - | G | Q | S | M | D | A | L | - | - | - | - | V | D | L | T | G | - | - | - | - | - | - | - | - | - | - | - | - | - | - | - | - | -139 |
| XP_006811804 | - | - | - | - | - | - | - | - | - | - | - | - | - | - | - | - | - | - | - | - | - | - | - | - | - | - | - | - | - | G | Q | A | S | D | A | L | - | - | - | - | I | D | M | T | G | - | - | - | - | - | - | - | - | - | - | - | - | - | - | - | - | -137 |
| C3YB23_BRAFL | - | - | - | - | - | - | - | - | - | - | - | - | - | - | - | - | - | - | - | - | - | - | - | - | - | - | - | - | - | G | Q | A | I | D | A | L | - | - | - | - | E | D | L | T | G | - | - | - | - | - | - | - | - | - | - | - | - | - | - | - | - | -144 |
| H2ZIF1_CIOSA | - | - | - | - | - | - | - | - | - | - | - | - | - | - | - | - | - | - | - | - | - | - | - | - | - | - | - | - | - | G | F | T | Q | D | G | M | - | - | - | - | E | D | L | T | G | - | - | - | - | - | - | - | - | - | - | - | - | - | - | - | - | -136 |
| W4YV93_STRPU | - | - | - | - | - | - | - | - | - | - | - | - | - | - | - | - | - | - | - | - | - | - | - | - | - | - | - | - | - | G | M | A | A | D | A | F | - | - | - | - | V | D | L | T | G | - | - | - | - | - | - | - | - | - | - | - | - | - | - | - | - | -136 |
| CAN10_HUMAN | - | - | - | - | - | - | - | - | - | - | - | - | - | - | - | - | - | - | - | - | - | - | - | - | - | - | - | - | - | G | Q | V | A | D | A | L | - | - | - | - | V | D | L | T | G | - | - | - | - | - | - | - | - | - | - | - | - | - | - | - | - | -137 |
| CAN10_MOUSE | - | - | - | - | - | - | - | - | - | - | - | - | - | - | - | - | - | - | - | - | - | - | - | - | - | - | - | - | - | G | Q | V | A | D | A | L | - | - | - | - | V | D | L | T | G | - | - | - | - | - | - | - | - | - | - | - | - | - | - | - | - | -137 |
| XP_004943443 | - | - | - | - | - | - | - | - | - | - | - | - | - | - | - | - | - | - | - | - | - | - | - | - | - | - | - | - | - | G | Q | V | A | D | A | L | - | - | - | - | V | D | L | T | G | - | - | - | - | - | - | - | - | - | - | - | - | - | - | - | - | -137 |
| F8W4K1_DANRE | - | - | - | - | - | - | - | - | - | - | - | - | - | - | - | - | - | - | - | - | - | - | - | - | - | - | - | - | - | G | Q | V | C | E | A | M | - | - | - | - | V | D | L | S | G | - | - | - | - | - | - | - | - | - | - | - | - | - | - | - | - | -137 |
| CANC_DROME | - | - | - | - | - | - | - | - | - | - | - | - | - | - | - | - | - | - | - | - | - | - | - | - | - | - | - | - | - | G | T | R | S | D | G | L | - | - | - | - | T | D | L | L | G | - | - | - | - | - | - | - | - | - | - | - | - | - | - | - | - | -123 |
| C3ZKN9_BRAFL | - | - | - | - | - | - | - | - | - | - | - | - | - | - | - | - | - | - | - | - | - | - | - | - | - | - | - | - | - | L | S | T | T | D | A | V | - | - | - | - | E | D | W | T | G | - | - | - | - | - | - | - | - | - | - | - | - | - | - | - | - | -135 |
| G4V623_SCHMA | - | - | - | - | - | - | - | - | - | - | - | - | - | - | - | - | - | - | - | - | - | - | - | - | - | - | - | - | - | G | L | V | G | D | A | M | - | - | - | - | D | D | L | T | G | - | - | - | - | - | - | - | - | - | - | - | - | - | - | - | - | -154 |
| R7VK39_CAPTE | - | - | - | - | - | - | - | - | - | - | - | - | - | - | - | - | - | - | - | - | - | - | - | - | - | - | - | - | - | - | K | L | I | D | V | V | - | - | - | - | I | D | L | S | G | - | - | - | - | - | - | - | - | - | - | - | - | - | - | - | - | -135 |
| G4V693_SCHMA | - | - | - | - | - | - | - | - | - | - | - | - | - | - | - | - | - | - | - | - | - | - | - | - | - | - | - | - | - | V | R | L | E | E | A | M | - | - | - | - | M | D | L | T | G | - | - | - | - | - | - | - | - | - | - | - | - | - | - | - | - | -140 |
| R7UPU6_CAPTE | - | - | - | - | - | - | - | - | - | - | - | - | - | - | - | - | - | - | - | - | - | - | - | - | - | - | - | - | - | G | W | A | G | D | A | F | - | - | - | - | V | A | M | T | G | - | - | - | - | - | - | - | - | - | - | - | - | - | - | - | - | -141 |
| R7V817_CAPTE | - | - | - | - | - | - | - | - | - | - | - | - | - | - | - | - | - | - | - | - | - | - | - | - | - | - | - | - | - | G | W | A | G | D | A | F | - | - | - | - | A | T | M | T | G | - | - | - | - | - | - | - | - | - | - | - | - | - | - | - | - | -138 |
| R7UNL8_CAPTE | - | - | - | - | - | - | - | - | - | - | - | - | - | - | - | - | - | - | - | - | - | - | - | - | - | - | - | - | - | G | L | P | G | D | A | F | - | - | - | - | T | T | L | T | G | - | - | - | - | - | - | - | - | - | - | - | - | - | - | - | - | -136 |
| K1PJ80_CRAGI | - | - | - | - | - | - | - | - | - | - | - | - | - | - | - | - | - | - | - | - | - | - | - | - | - | - | - | - | - | G | M | A | S | D | S | Y | - | - | - | - | M | A | L | T | G | - | - | - | - | - | - | - | - | - | - | - | - | - | - | - | - | -139 |
| K1PCX8_CRAGI | - | - | - | - | - | - | - | - | - | - | - | - | - | - | - | - | - | - | - | - | - | - | - | - | - | - | - | - | - | G | V | S | S | D | S | Y | - | - | - | - | M | A | L | T | G | - | - | - | - | - | - | - | - | - | - | - | - | - | - | - | - | -139 |
| CAND_DROME | - | - | - | - | - | - | - | - | - | - | - | - | - | - | - | - | - | - | - | - | - | - | - | - | - | - | - | - | - | G | R | A | I | E | G | L | - | - | - | - | A | T | L | T | G | - | - | - | - | - | - | - | - | - | - | - | - | - | - | - | - | -137 |
| XP_002735765 | - | - | - | - | - | - | - | - | - | - | - | - | - | - | - | - | - | - | - | - | - | - | - | - | - | - | - | - | - | G | R | C | I | E | G | L | - | - | - | - | A | T | L | T | G | - | - | - | - | - | - | - | - | - | - | - | - | - | - | - | - | -139 |
| CAN15_HUMAN | - | - | - | - | - | - | - | - | - | - | - | - | - | - | - | - | - | - | - | - | - | - | - | - | - | - | - | - | - | G | R | A | I | E | G | L | - | - | - | - | A | T | L | T | G | - | - | - | - | - | - | - | - | - | - | - | - | - | - | - | - | -139 |
| CAN15_MOUSE | - | - | - | - | - | - | - | - | - | - | - | - | - | - | - | - | - | - | - | - | - | - | - | - | - | - | - | - | - | G | R | A | I | E | G | L | - | - | - | - | A | T | L | T | G | - | - | - | - | - | - | - | - | - | - | - | - | - | - | - | - | -139 |
| E1BTA8_CHICK | - | - | - | - | - | - | - | - | - | - | - | - | - | - | - | - | - | - | - | - | - | - | - | - | - | - | - | - | - | G | R | A | I | E | G | L | - | - | - | - | A | T | L | T | G | - | - | - | - | - | - | - | - | - | - | - | - | - | - | - | - | -139 |
| E7FAX5_DANRE | - | - | - | - | - | - | - | - | - | - | - | - | - | - | - | - | - | - | - | - | - | - | - | - | - | - | - | - | - | G | R | A | I | E | G | L | - | - | - | - | A | T | L | T | G | - | - | - | - | - | - | - | - | - | - | - | - | - | - | - | - | -139 |
| C3XWK8_BRAFL | - | - | - | - | - | - | - | - | - | - | - | - | - | - | - | - | - | - | - | - | - | - | - | - | - | - | - | - | - | G | R | S | I | E | G | L | - | - | - | - | S | T | L | T | G | - | - | - | - | - | - | - | - | - | - | - | - | - | - | - | - | -138 |
| W4YMU4_STRPU | - | K | A | K | R | K | Q | L | W | V | P | L | I | E | K | A | L | A | K | M | S | G | C | Y | E | A | L | T | A | G | R | C | I | E | G | L | - | - | - | - | A | T | L | T | G | - | - | - | - | - | - | - | - | - | - | - | - | - | - | - | - | -252 |
| K1PV74_CRAGI | - | - | - | - | - | - | - | - | - | - | - | - | - | - | - | - | - | - | - | - | - | - | - | - | - | - | - | - | - | G | K | C | L | E | G | L | - | - | - | - | A | T | L | T | G | - | - | - | - | - | - | - | - | - | - | - | - | - | - | - | - | -139 |
| R7TJF2_CAPTE | - | - | - | - | - | - | - | - | - | - | - | - | - | - | - | - | - | - | - | - | - | - | - | - | - | - | - | - | - | G | R | C | I | E | G | L | - | - | - | - | A | T | L | T | G | - | - | - | - | - | - | - | - | - | - | - | - | - | - | - | - | -139 |
| T2MI17_HYDVU | - | - | - | - | - | - | - | - | - | - | - | - | - | - | - | - | - | - | - | - | - | - | - | - | - | - | - | - | - | G | R | C | I | E | G | L | - | - | - | - | Q | L | L | T | G | - | - | - | - | - | - | - | - | - | - | - | - | - | - | - | - | -136 |
| Q1RL55_CIOIN | - | - | - | - | - | - | - | - | - | - | - | - | - | - | - | - | - | - | - | - | - | - | - | - | - | - | - | - | - | G | R | S | I | E | G | L | - | - | - | - | A | T | L | T | G | - | - | - | - | - | - | - | - | - | - | - | - | - | - | - | - | -126 |
| I1EA30_AMPQE | - | - | - | - | - | - | - | - | - | - | - | - | - | - | - | - | - | - | - | - | - | - | - | - | - | - | - | - | - | G | T | I | I | S | G | L | - | - | - | - | S | V | L | T | G | - | - | - | - | - | - | - | - | - | - | - | - | - | - | - | - | -139 |
| G5EEZ6_CAEEL | - | - | - | - | - | - | - | - | - | - | - | - | - | - | - | - | - | - | - | - | - | - | - | - | - | - | - | - | - | A | S | L | A | Q | G | L | - | - | - | - | S | M | L | T | G | - | - | - | - | - | - | - | - | - | - | - | - | - | - | - | - | -139 |
| O44903_CAEEL | - | - | - | - | - | - | - | - | - | - | - | - | - | - | - | - | - | - | - | - | - | - | - | - | - | - | - | - | - | A | S | L | A | R | G | L | - | - | - | - | S | M | L | T | G | - | - | - | - | - | - | - | - | - | - | - | - | - | - | - | - | -139 |
| O02260_CAEEL | - | - | - | - | - | - | - | - | - | - | - | - | - | - | - | - | - | - | - | - | - | - | - | - | - | - | - | - | - | F | Q | S | L | S | A | F | - | - | - | - | S | A | L | T | G | - | - | - | - | - | - | - | - | - | - | - | - | - | - | - | - | -136 |
| O02259_CAEEL | - | - | - | - | - | - | - | - | - | - | - | - | - | - | - | - | - | - | - | - | - | - | - | - | - | - | - | - | - | F | Q | N | Q | S | A | F | - | - | - | - | S | A | L | T | G | - | - | - | - | - | - | - | - | - | - | - | - | - | - | - | - | -113 |
| C1MTG3_MICPC | - | - | - | - | - | - | - | - | - | - | - | - | - | - | - | - | - | - | - | - | - | - | - | - | - | - | - | - | - | G | Y | I | S | E | G | L | - | - | - | - | L | D | L | T | G | - | - | - | - | - | - | - | - | - | - | - | - | - | - | - | - | -143 |
| C1E493_MICSR | - | - | - | - | - | - | - | - | - | - | - | - | - | - | - | - | - | - | - | - | - | - | - | - | - | - | - | - | - | G | W | I | A | E | G | L | - | - | - | - | L | D | L | T | G | - | - | - | - | - | - | - | - | - | - | - | - | - | - | - | - | -134 |
| Q012Z6_OSTTA | - | - | - | - | - | - | - | - | - | - | - | - | - | - | - | - | - | - | - | - | - | - | - | - | - | - | - | - | - | G | Y | V | S | E | G | L | - | - | - | - | H | D | L | T | G | - | - | - | - | - | - | - | - | - | - | - | - | - | - | - | - | -131 |
| L1IGB6_GUITH | - | - | - | - | - | - | - | - | - | - | - | - | - | - | - | - | - | - | - | - | - | - | - | - | - | - | - | - | - | G | E | I | A | E | A | L | - | - | - | - | L | D | L | T | G | - | - | - | - | - | - | - | - | - | - | - | - | - | - | - | - | -134 |
| B7FWX6_PHATC | - | - | - | - | - | - | - | - | - | - | - | - | - | - | - | - | - | - | - | - | - | - | - | - | - | - | - | - | - | G | F | I | A | E | G | L | - | - | - | - | T | D | L | T | G | - | - | - | - | - | - | - | - | - | - | - | - | - | - | - | - | -139 |
| N9TPR9_ENTHI | - | - | - | - | - | - | - | - | - | - | - | - | - | - | - | - | - | - | - | - | - | - | - | - | - | - | - | - | - | G | I | P | F | L | A | L | - | - | - | - | S | D | L | T | G | - | - | - | - | - | - | - | - | - | - | - | - | - | - | - | - | -128 |
| G0QS43_ICHMG | - | - | - | - | - | - | - | - | - | - | - | - | - | - | - | - | - | - | - | - | - | - | - | - | - | - | - | - | - | G | L | T | R | E | C | L | - | - | - | - | H | D | L | T | C | - | - | - | - | - | - | - | - | - | - | - | - | - | - | - | - | -135 |
| D8LB20_ECTSI | - | - | - | - | - | - | - | - | - | - | - | - | - | - | - | - | - | - | - | - | - | - | - | - | - | - | - | - | - | G | F | E | A | W | G | L | - | - | - | - | K | V | L | T | G | N | H | V | W | T | F | R | R | L | K | S | A | R | P | G | K | -155 |
| Q9U0T9_LEIMA | - | - | - | - | - | - | - | - | - | - | - | - | - | - | - | - | - | - | - | - | - | - | - | - | - | - | - | - | - | G | D | P | M | H | A | L | - | - | - | - | T | D | M | T | G | - | - | - | - | - | - | - | - | - | - | - | - | - | - | - | - | -141 |
| Q4CXJ8_TRYCC | - | - | - | - | - | - | - | - | - | - | - | - | - | - | - | - | - | - | - | - | - | - | - | - | - | - | - | - | - | G | D | P | V | H | A | L | - | - | - | - | T | D | M | T | G | - | - | - | - | - | - | - | - | - | - | - | - | - | - | - | - | -141 |
| F4I0A4_ARATH | - | - | - | - | - | - | - | - | - | - | - | - | - | - | - | - | - | - | - | - | - | - | - | - | - | - | - | - | - | G | L | V | Q | D | A | L | - | - | - | - | V | D | L | T | G | - | - | - | - | - | - | - | - | - | - | - | - | - | - | - | - | -138 |
| Q8RUQ1_MAIZE | - | - | - | - | - | - | - | - | - | - | - | - | - | - | - | - | - | - | - | - | - | - | - | - | - | - | - | - | - | G | L | V | Q | D | A | L | - | - | - | - | V | D | L | T | G | - | - | - | - | - | - | - | - | - | - | - | - | - | - | - | - | -138 |
| D7FQ17_ECTSI | - | - | - | - | - | - | - | - | - | - | - | - | - | - | - | - | - | - | - | - | - | - | - | - | - | - | - | - | - | G | H | V | H | H | A | L | - | - | - | - | S | A | F | T | G | - | - | - | - | - | - | - | - | - | - | - | - | - | - | - | - | -155 |
| D0MZ44_PHYIT | - | - | - | - | - | - | - | - | - | - | - | - | - | - | - | - | - | - | - | - | - | - | - | - | - | - | - | - | - | G | Y | V | H | H | A | L | - | - | - | - | R | V | L | S | G | - | - | - | - | - | - | - | - | - | - | - | - | - | - | - | - | -156 |
| L1JBM0_GUITH | - | - | - | - | - | - | - | - | - | - | - | - | - | - | - | - | - | - | - | - | - | - | - | - | - | - | - | - | - | G | S | E | E | E | A | L | - | - | - | - | V | C | L | T | G | - | - | - | - | - | - | - | - | - | - | - | - | - | - | - | - | -109 |
| L1J739_GUITH | - | - | - | - | - | - | - | - | - | - | - | - | - | - | - | - | - | - | - | - | - | - | - | - | - | - | - | - | - | G | A | M | T | Y | A | L | - | - | - | - | K | D | L | T | G | - | - | - | - | - | - | - | - | - | - | - | - | - | - | - | - | -140 |
| L1J0U9_GUITH | - | - | - | - | - | - | - | - | - | - | - | - | - | - | - | - | - | - | - | - | - | - | - | - | - | - | - | - | - | G | S | V | T | Y | A | L | - | - | - | - | R | D | L | T | G | - | - | - | - | - | - | - | - | - | - | - | - | - | - | - | - | -133 |
| L1J1Q4_GUITH | - | - | - | - | - | - | - | - | - | - | - | - | - | - | - | - | - | - | - | - | - | - | - | - | - | - | - | - | - | G | K | I | S | E | A | L | - | - | - | - | A | D | L | T | G | - | - | - | - | - | - | - | - | - | - | - | - | - | - | - | - | -151 |
| D0ND88_PHYIT | - | - | - | - | - | - | - | - | - | - | - | - | - | - | - | - | - | - | - | - | - | - | - | - | - | - | T | S | S | G | R | I | L | E | A | F | - | - | - | - | L | D | C | T | G | - | - | - | - | - | - | - | - | - | - | - | - | - | - | - | - | -168 |
| D2VVS6_NAEGR | - | - | - | - | - | - | - | - | - | - | - | - | - | - | - | - | - | - | - | - | - | - | - | - | - | - | - | - | - | G | H | L | K | D | A | L | - | - | - | - | V | D | L | T | G | - | - | - | - | - | - | - | - | - | - | - | - | - | - | - | - | -137 |
| D2V3V2_NAEGR | - | - | - | - | - | - | - | - | - | - | - | - | - | - | - | - | - | - | - | - | - | - | - | - | - | - | - | - | - | G | Y | M | R | D | A | L | - | - | - | - | V | S | L | T | G | - | - | - | - | - | - | - | - | - | - | - | - | - | - | - | - | -138 |
| Q5B5J7_EMENI | - | - | - | - | - | - | - | - | - | - | - | - | - | - | - | - | - | - | - | - | - | - | - | - | - | - | - | - | - | G | F | V | G | E | A | V | - | - | - | - | E | D | L | T | G | - | - | - | - | - | - | - | - | - | - | - | - | - | - | - | - | -158 |
| E4ZY10_LEPMJ | - | - | - | - | - | - | - | - | - | - | - | - | - | - | - | - | - | - | - | - | - | - | - | - | - | - | - | - | - | G | F | V | G | E | G | I | - | - | - | - | E | D | L | T | G | - | - | - | - | - | - | - | - | - | - | - | - | - | - | - | - | -164 |
| A8PIE5_COPC7 | - | - | - | - | - | - | - | - | - | - | - | - | - | - | - | - | - | - | - | - | - | - | - | - | - | - | - | - | - | G | Y | T | C | E | G | V | - | - | - | - | E | D | L | T | G | - | - | - | - | - | - | - | - | - | - | - | - | - | - | - | - | -159 |
| A8NBA6_COPC7 | - | - | - | - | - | - | - | - | - | - | - | - | - | - | - | - | - | - | - | - | - | - | - | - | - | - | - | - | - | G | W | T | G | E | A | I | - | - | - | - | E | D | L | T | G | - | - | - | - | - | - | - | - | - | - | - | - | - | - | - | - | -158 |
| A8NBA1_COPC7 | - | - | - | - | - | - | - | - | - | - | - | - | - | - | - | - | - | - | - | - | - | - | - | - | - | - | - | - | - | G | W | T | T | E | A | I | - | - | - | - | E | D | L | S | G | - | - | - | - | - | - | - | - | - | - | - | - | - | - | - | - | -162 |
| A8NBA2_COPC7 | - | - | - | - | - | - | - | - | - | - | - | - | - | - | - | - | - | - | - | - | - | - | - | - | - | - | - | - | - | G | W | T | T | E | A | I | - | - | - | - | E | D | L | S | G | - | - | - | - | - | - | - | - | - | - | - | - | - | - | - | - | -162 |
| A8NAE6_COPC7 | - | - | - | - | - | - | - | - | - | - | - | - | - | - | - | - | - | - | - | - | - | - | - | - | - | - | - | - | - | G | R | T | - | - | - | - | - | - | - | - | - | - | - | - | - | - | - | - | - | - | - | - | - | - | - | - | - | - | - | - | - | -180 |
| A8NAI9_COPC7 | - | - | - | - | - | - | - | - | - | - | - | - | - | - | - | - | - | - | - | - | - | - | - | - | - | - | - | - | - | G | Q | T | A | Q | A | L | - | - | - | - | E | D | L | T | G | - | - | - | - | - | - | - | - | - | - | - | - | - | - | - | - | -158 |
| G4TER7_PIRID | - | - | - | - | - | - | - | - | - | - | - | - | - | - | - | - | - | - | - | - | - | - | - | - | - | - | - | - | - | G | R | A | M | E | A | I | - | - | - | - | E | D | L | T | G | - | - | - | - | - | - | - | - | - | - | - | - | - | - | - | - | -158 |
| G4TER6_PIRID | - | - | - | - | - | - | - | - | - | - | - | - | - | - | - | - | - | - | - | - | - | - | - | - | - | - | - | - | - | G | F | A | N | E | A | I | - | - | - | - | E | D | L | T | G | - | - | - | - | - | - | - | - | - | - | - | - | - | - | - | - | -148 |
| G4TNA1_PIRID | - | - | - | - | - | - | - | - | - | - | - | - | - | - | - | - | - | - | - | - | - | - | - | - | - | - | - | - | - | G | W | T | M | E | G | I | - | - | - | - | E | D | L | T | G | - | - | - | - | - | - | - | - | - | - | - | - | - | - | - | - | -158 |
| D0MWB9_PHYIT | - | - | - | - | - | - | - | - | - | - | - | - | - | - | - | - | - | - | - | - | - | - | - | - | - | - | - | - | - | G | F | V | D | C | A | L | - | - | - | - | N | D | L | T | G | - | - | - | - | - | - | - | - | - | - | - | - | - | - | - | - | -132 |
| L1I9H5_GUITH | - | - | - | - | - | - | - | - | - | - | - | - | - | - | - | - | - | - | - | - | - | - | - | - | - | - | - | - | - | G | L | T | A | D | T | T | - | - | - | - | N | Y | L | T | G | - | - | - | - | - | - | - | - | - | - | - | - | - | - | - | - | -147 |
| Q7N497_PHOLL | - | - | - | - | - | - | - | - | - | - | - | - | - | - | - | - | - | - | - | - | - | - | - | - | - | - | - | - | - | - | - | - | - | - | G | N | - | - | - | - | E | I | Y | F | G | - | - | - | - | - | - | - | - | - | - | - | - | - | - | - | - | -116 |
| ZP_11152369 | - | - | - | - | - | - | - | - | - | - | - | - | - | - | - | - | - | - | - | - | - | - | - | - | - | - | - | - | - | - | - | - | - | - | G | I | - | - | - | - | E | D | Y | L | G | - | - | - | - | - | - | - | - | - | - | - | - | - | - | - | - | -112 |
| A2FEL7_TRIVA | - | - | - | - | - | - | - | - | - | - | - | - | - | - | - | - | - | - | - | - | - | - | - | - | - | - | - | - | - | G | T | P | D | E | A | L | Y | - | - | - | - | R | L | L | G | - | - | - | - | - | - | - | - | - | - | - | - | - | - | - | - | -133 |
| A2D9A8_TRIVA | - | - | - | - | - | - | - | - | - | - | - | - | - | - | - | - | - | - | - | - | - | - | - | - | - | - | - | - | - | G | A | P | S | N | A | L | Y | - | - | - | - | R | L | V | G | - | - | - | - | - | - | - | - | - | - | - | - | - | - | - | - | -134 |
| A2FHB3_TRIVA | - | - | - | - | - | - | - | - | - | - | - | - | - | - | - | - | - | - | - | - | - | - | - | - | - | - | - | - | - | G | N | S | H | L | A | L | Y | - | - | - | - | R | L | V | G | - | - | - | - | - | - | - | - | - | - | - | - | - | - | - | - | -135 |
| A2DTE8_TRIVA | - | - | - | - | - | - | - | - | - | - | - | - | - | - | - | - | - | - | - | - | - | - | - | - | - | - | - | - | - | G | F | C | S | S | A | F | N | - | - | - | - | M | L | C | D | - | - | - | - | - | - | - | - | - | - | - | - | - | - | - | - | -132 |
| A2E4X2_TRIVA | - | - | - | - | - | - | - | - | - | - | - | - | - | - | - | - | - | - | - | - | - | - | - | - | - | - | - | - | - | C | A | A | I | T | S | C | Y | - | - | - | - | H | I | L | G | - | - | - | - | - | - | - | - | - | - | - | - | - | - | - | - | -137 |
| A2E1X1_TRIVA | - | - | - | - | - | - | - | - | - | - | - | - | - | - | - | - | - | - | - | - | - | - | - | - | - | - | - | - | - | N | P | A | D | A | M | N | - | - | - | - | - | N | I | F | G | - | - | - | - | - | - | - | - | - | - | - | - | - | - | - | - | -121 |
| TPR_PORGI | - | - | - | - | - | - | - | - | - | - | - | - | - | - | - | - | - | - | - | - | - | - | - | K | Y | N | A | I | L | Q | I | G | G | G | W | P | E | E | A | L | C | E | L | S | G | D | - | - | - | - | - | - | - | - | - | - | - | - | - | - | - | -160 |
| D5MF42_9BACT | - | - | - | - | - | - | - | - | - | - | - | - | - | - | - | - | - | - | - | - | - | - | - | D | E | P | N | Y | G | P | I | S | G | G | W | P | V | Q | A | T | T | E | L | T | N | L | - | - | - | - | - | - | - | - | - | - | - | - | - | - | - | -120 |
| R9H254_BACT4 | - | - | - | - | - | - | - | - | - | - | - | - | - | - | - | - | - | - | - | - | - | - | - | - | - | - | G | S | S | N | V | G | G | I | G | T | E | Y | V | S | A | I | F | T | G | D | - | - | - | - | - | - | - | - | - | - | - | - | - | - | - | -117 |
| ZP_04847694 | - | - | - | - | - | - | - | - | - | - | - | - | - | - | - | - | - | - | - | - | - | - | - | - | - | - | G | S | S | N | V | G | G | I | G | T | E | Y | V | S | A | I | F | T | G | D | - | - | - | - | - | - | - | - | - | - | - | - | - | - | - | -117 |
| D7IBA4_9BACE | - | - | - | - | - | - | - | - | - | - | - | - | - | - | - | - | - | - | - | - | - | - | - | - | - | - | G | S | S | N | V | G | G | I | G | T | E | Y | V | S | A | I | F | T | G | D | - | - | - | - | - | - | - | - | - | - | - | - | - | - | - | -115 |
| ZP_09858577 | - | - | - | - | - | - | - | - | - | - | - | - | - | - | - | - | - | - | - | - | - | - | - | - | - | - | G | S | S | N | V | G | G | I | G | T | E | Y | V | S | A | I | F | T | G | D | - | - | - | - | - | - | - | - | - | - | - | - | - | - | - | -115 |
| E5C759_9BACE | - | - | - | - | - | - | - | - | - | - | - | - | - | - | - | - | - | - | - | - | - | - | - | - | - | - | G | S | S | N | V | G | G | I | G | T | E | Y | V | S | A | I | F | T | G | D | - | - | - | - | - | - | - | - | - | - | - | - | - | - | - | -117 |
| A7LTS0_BACO1 | - | - | - | - | - | - | - | - | - | - | - | - | - | - | - | - | - | - | - | - | - | - | - | - | - | - | N | T | S | D | I | G | G | I | G | S | E | Y | A | A | A | I | I | T | G | N | - | - | - | - | - | - | - | - | - | - | - | - | - | - | - | -115 |
| Q8YQK8_NOSS1 | - | - | - | - | - | - | - | - | - | - | - | - | - | - | - | - | - | - | - | - | - | - | - | T | P | G | Y | N | L | I | G | N | G | D | T | L | N | R | P | L | G | F | I | T | G | Q | - | - | - | - | - | - | - | - | - | - | - | - | - | - | - | -131 |
| Q3MBX8_ANAVT | - | - | - | - | - | - | - | - | - | - | - | - | - | - | - | - | - | - | - | - | - | - | - | K | P | G | Y | N | I | I | G | N | G | D | A | L | S | R | P | L | Q | F | V | T | G | R | - | - | - | - | - | - | - | - | - | - | - | - | - | - | - | -136 |
| Q8YZT8_NOSS1 | - | - | - | - | - | - | - | - | - | - | - | - | - | - | - | - | - | - | - | - | - | - | - | R | P | G | Y | N | I | I | G | N | G | G | A | L | A | P | P | L | S | F | I | T | G | R | - | - | - | - | - | - | - | - | - | - | - | - | - | - | - | -124 |
| K9UIG2_9CHRO | - | - | - | - | - | - | - | - | - | - | - | - | - | - | - | - | - | - | - | - | - | - | - | R | T | G | W | D | I | I | G | N | G | D | Y | L | D | D | G | L | Q | R | V | T | G | R | - | - | - | - | - | - | - | - | - | - | - | - | - | - | - | -130 |
| A4YRP8_BRASO | - | - | - | - | - | - | - | - | - | - | - | - | - | - | - | - | - | - | - | - | - | - | - | G | N | S | Y | T | T | I | G | N | G | G | A | P | E | I | A | L | E | A | I | T | G | A | - | - | - | - | - | - | - | - | - | - | - | - | - | - | - | -125 |
| H0TAL7_9BRAD | - | - | - | - | - | - | - | - | - | - | - | - | - | - | - | - | - | - | - | - | - | - | - | G | N | S | Y | T | T | I | G | N | G | G | A | P | E | I | A | L | E | A | I | T | G | A | - | - | - | - | - | - | - | - | - | - | - | - | - | - | - | -129 |
| H0SNL4_9BRAD | - | - | - | - | - | - | - | - | - | - | - | - | - | - | - | - | - | - | - | - | - | - | - | G | N | S | Y | T | T | I | G | N | G | G | A | P | E | I | A | L | E | A | I | T | G | A | - | - | - | - | - | - | - | - | - | - | - | - | - | - | - | -131 |
| H0S4P6_9BRAD | - | - | - | - | - | - | - | - | - | - | - | - | - | - | - | - | - | - | - | - | - | - | - | G | N | S | Y | T | T | I | G | N | G | G | A | P | E | I | A | L | E | A | I | T | G | A | - | - | - | - | - | - | - | - | - | - | - | - | - | - | - | -131 |
| A5EMK2_BRASB | - | - | - | - | - | - | - | - | - | - | - | - | - | - | - | - | - | - | - | - | - | - | - | G | N | S | Y | T | T | I | G | N | G | G | A | P | Q | I | A | L | E | A | I | T | G | A | - | - | - | - | - | - | - | - | - | - | - | - | - | - | - | -125 |
| M4ZBJ4_9BRAD | - | - | - | - | - | - | - | - | - | - | - | - | - | - | - | - | - | - | - | - | - | - | - | G | N | S | Y | T | T | I | G | N | G | G | A | P | E | I | A | L | E | A | I | T | G | A | - | - | - | - | - | - | - | - | - | - | - | - | - | - | - | -131 |
| Q07SP7_RHOP5 | - | - | - | - | - | - | - | - | - | - | - | - | - | - | - | - | - | - | - | - | - | - | - | S | N | S | W | T | K | I | G | N | G | G | S | V | E | R | A | L | A | A | I | T | G | A | - | - | - | - | - | - | - | - | - | - | - | - | - | - | - | -118 |
| B0JUQ7_MICAN | - | - | - | - | - | - | - | - | - | - | - | - | - | - | - | - | - | - | - | - | Q | D | N | T | N | S | Y | A | G | I | - | N | G | G | W | S | D | T | T | V | Q | Q | I | T | G | R | - | - | - | - | - | - | - | - | - | - | - | - | - | - | - | -137 |
| K9WJ87_9CYAN | - | - | - | - | - | - | - | - | - | - | - | - | - | - | - | - | - | - | - | - | Q | D | N | T | N | S | Y | Q | G | I | - | A | Y | G | - | T | L | K | A | I | S | H | I | T | G | L | - | - | - | - | - | - | - | - | - | - | - | - | - | - | - | -131 |
| G6FUE0_9CYAN | - | - | - | - | - | - | - | - | - | - | - | - | - | - | - | - | - | - | - | - | Q | D | N | T | N | S | Y | N | G | I | G | N | G | G | Y | V | S | D | A | L | T | N | I | T | G | L | - | - | - | - | - | - | - | - | - | - | - | - | - | - | - | -132 |
| K9PHG5_9CYAN | - | - | - | - | - | - | - | - | - | - | - | - | - | - | - | - | - | - | - | - | Q | D | N | T | N | S | Y | A | G | I | - | S | A | G | W | D | M | D | A | I | R | H | I | T | G | K | - | - | - | - | - | - | - | - | - | - | - | - | - | - | - | -134 |
| Q8YXX3_NOSS1 | - | - | - | - | - | - | - | - | - | - | - | - | - | - | - | - | - | - | - | - | G | Q | R | R | N | S | Y | Q | A | I | - | V | S | G | Y | A | G | R | A | I | T | D | I | T | G | - | - | - | - | - | - | - | - | - | - | - | - | - | - | - | - | -146 |
| K9TN70_9CYAN | - | - | - | - | - | - | - | - | - | - | - | - | - | - | - | - | - | - | - | - | Q | D | G | T | N | S | Y | K | G | I | - | E | E | G | R | A | Q | V | A | S | E | H | I | A | E | V | - | - | - | - | - | - | - | - | - | - | - | - | - | - | - | -147 |
| Q05QW3_9SYNE | - | - | - | - | - | - | - | - | - | - | - | - | - | - | - | - | - | - | - | - | R | Y | G | R | Q | W | N | E | G | I | - | S | W | G | W | A | D | A | T | T | T | H | I | T | G | L | - | - | - | - | - | - | - | - | - | - | - | - | - | - | - | -144 |
| Q05QW2_9SYNE | - | - | - | - | - | - | - | - | - | - | - | - | - | - | - | - | - | - | - | - | R | Y | G | I | A | G | N | E | G | I | - | S | W | G | F | T | T | D | A | I | T | H | I | T | G | L | - | - | - | - | - | - | - | - | - | - | - | - | - | - | - | -146 |
| Q05QW4_9SYNE | - | - | - | - | - | - | - | - | - | - | - | - | - | - | - | - | - | - | - | - | - | - | G | R | N | S | Y | A | G | I | - | S | G | G | N | S | A | T | A | I | T | H | I | T | G | L | - | - | - | - | - | - | - | - | - | - | - | - | - | - | - | -140 |
| E0UGX6_CYAP2 | - | - | - | - | - | - | - | - | - | - | - | - | - | - | - | - | - | - | - | - | - | - | - | T | N | S | Y | G | A | I | - | E | G | G | W | M | D | A | V | I | K | Q | V | T | G | L | - | - | - | - | - | - | - | - | - | - | - | - | - | - | - | -135 |
| K7WKF4_9NOST | - | - | - | - | - | - | - | - | - | - | - | - | - | - | - | - | - | - | - | - | - | - | Q | N | N | S | Y | A | A | I | - | E | G | G | W | M | D | T | V | I | R | Q | V | T | G | L | - | - | - | - | - | - | - | - | - | - | - | - | - | - | - | -134 |
| E0UGY5_CYAP2 | - | - | - | - | - | - | - | - | - | - | - | - | - | - | - | - | - | - | - | - | - | - | - | E | N | S | Y | A | A | I | - | E | G | G | W | M | D | A | A | I | Q | E | I | T | G | L | - | - | - | - | - | - | - | - | - | - | - | - | - | - | - | -137 |
| A8LCE5_FRASN | - | - | - | - | - | - | - | - | - | - | - | - | - | - | - | - | - | - | - | - | - | - | - | - | - | - | - | Y | A | R | L | E | G | G | D | P | A | T | A | M | E | Y | L | T | G | T | - | - | - | - | - | - | - | - | - | - | - | - | - | - | - | -129 |
| D3CT47_9ACTO | - | - | - | - | - | - | - | - | - | - | - | - | - | - | - | - | - | - | - | - | - | - | - | - | - | - | - | Y | A | G | T | D | G | G | D | P | A | V | A | M | R | Q | L | T | G | V | - | - | - | - | - | - | - | - | - | - | - | - | - | - | - | -133 |
| Q2J4J6_FRASC | - | - | - | - | - | - | - | - | - | - | - | - | - | - | - | - | - | - | - | - | - | - | - | - | - | - | - | Y | P | A | I | E | F | G | N | A | A | T | A | L | R | T | L | T | G | R | - | - | - | - | - | - | - | - | - | - | - | - | - | - | - | -154 |
| Q0RAZ2_FRAAA | - | - | - | - | - | - | - | - | - | - | - | - | - | - | - | - | - | - | - | - | - | - | - | - | - | - | - | Y | P | A | I | G | F | G | S | A | S | L | A | L | G | T | L | T | G | R | - | - | - | - | - | - | - | - | - | - | - | - | - | - | - | -141 |
| E3J3F6_FRASU | - | - | - | - | - | - | - | - | - | - | - | - | - | - | - | - | - | - | - | - | - | - | - | - | - | - | - | Y | D | S | I | N | G | G | D | P | G | V | A | T | S | D | L | T | G | R | - | - | - | - | - | - | - | - | - | - | - | - | - | - | - | -135 |
| ZP_09168240 | - | - | - | - | - | - | - | - | - | - | - | - | - | - | - | - | - | - | - | - | - | - | - | - | - | - | - | Y | D | A | I | S | G | G | D | P | G | V | A | A | S | D | L | T | G | R | - | - | - | - | - | - | - | - | - | - | - | - | - | - | - | -145 |
| C7MDV9_BRAFD | - | - | - | - | - | - | - | - | - | - | - | - | - | - | - | - | - | - | - | - | - | - | - | - | - | - | - | Y | G | A | L | Q | R | G | V | A | R | F | G | F | E | L | L | L | G | E | - | - | - | - | - | - | - | - | - | - | - | - | - | - | - | -107 |
| ZP_10094989 | - | - | - | - | - | - | - | - | - | - | - | - | - | - | - | - | - | - | - | - | - | - | - | - | - | - | - | Y | A | F | L | Q | R | G | L | A | R | Y | G | F | A | L | L | L | G | E | - | - | - | - | - | - | - | - | - | - | - | - | - | - | - | -107 |
| A4X268_SALTO | - | - | - | - | - | - | - | - | - | - | - | - | - | - | S | P | N | D | P | L | V | A | A | P | T | G | Y | A | R | L | D | V | G | S | H | R | Q | M | Q | A | E | L | L | T | Q | L | - | - | - | - | - | - | - | - | - | - | - | - | - | - | - | -152 |
| WP_043162213 | - | - | - | - | - | - | - | - | - | - | - | - | - | - | S | P | N | D | P | L | V | A | A | P | T | G | Y | A | R | L | D | V | G | S | H | R | Q | M | Q | A | E | L | L | T | Q | L | - | - | - | - | - | - | - | - | - | - | - | - | - | - | - | -152 |
| Q0BRV8_GRABC | - | - | - | - | - | - | - | - | - | - | - | - | - | - | - | - | - | - | - | - | - | - | - | - | G | G | Y | S | S | I | A | Y | G | G | N | - | - | - | P | T | T | T | L | M | M | L | - | - | - | - | - | - | - | - | - | - | - | - | - | - | - | -133 |
| A9B2P0_HERA2 | - | - | - | - | - | - | - | - | - | - | - | - | - | - | - | - | - | - | - | - | - | - | - | - | - | - | Y | A | N | I | V | S | G | - | N | A | G | E | F | M | A | I | L | T | G | N | - | - | - | - | - | - | - | - | - | - | - | - | - | - | - | -125 |
| A9B3U8_HERA2 | - | - | - | - | - | - | - | - | - | - | - | - | - | - | - | - | - | - | - | - | - | - | - | - | - | - | Y | A | N | I | E | L | G | - | N | A | G | E | F | I | E | L | L | T | G | N | - | - | - | - | - | - | - | - | - | - | - | - | - | - | - | -127 |
| A9B2A3_HERA2 | - | - | - | - | - | - | - | - | - | - | - | - | - | - | - | - | - | - | - | - | - | - | P | I | D | M | Y | S | T | L | N | K | G | G | N | P | A | D | V | L | E | A | I | T | G | Q | - | - | - | - | - | - | - | - | - | - | - | - | - | - | - | -145 |
| D6ZC85_SEGRD | - | - | - | - | - | - | - | - | - | - | - | - | - | - | E | L | G | L | K | I | A | E | A | S | N | E | K | N | P | L | W | A | G | G | D | V | D | Q | G | M | A | S | V | T | G | E | - | - | - | - | - | - | - | - | - | - | - | - | - | - | - | -171 |
| E5XTT9_9ACTO | - | - | - | - | - | - | - | - | - | - | - | - | - | - | E | A | G | L | K | V | A | E | A | A | S | P | V | N | P | L | W | S | G | G | D | V | D | Q | G | M | A | T | I | T | G | Q | - | - | - | - | - | - | - | - | - | - | - | - | - | - | - | -171 |
| E5XTU0_9ACTO | - | - | - | - | - | - | - | - | - | - | - | - | - | - | D | S | W | Y | R | A | T | N | P | V | D | A | G | M | H | I | M | Q | D | G | S | S | D | Q | G | M | A | V | V | T | G | H | - | - | - | - | - | - | - | - | - | - | - | - | - | - | - | -175 |
| D6B0I6_9ACTO | - | - | - | - | - | - | - | - | - | - | - | - | - | - | - | - | - | - | - | - | - | - | - | - | E | R | G | Y | G | G | I | E | G | D | D | P | K | K | S | A | P | Y | L | T | G | Q | - | - | - | - | - | - | - | - | - | - | - | - | - | - | - | -128 |
| D9X719_STRVR | - | - | - | - | - | - | - | - | - | - | - | - | - | - | - | - | - | - | - | - | - | - | - | - | E | R | G | Y | G | G | I | E | G | D | D | P | K | K | S | A | P | Y | L | T | G | R | - | - | - | - | - | - | - | - | - | - | - | - | - | - | - | -128 |
| H6L010_SAPGL | - | - | - | - | - | - | - | - | - | - | - | - | - | - | - | - | - | - | - | - | - | - | - | - | - | - | S | Y | Q | D | I | H | G | G | Y | V | E | K | A | M | G | L | L | T | G | E | - | - | - | - | - | - | - | - | - | - | - | - | - | - | - | -118 |
| K1ZP83_9BACT | - | - | - | - | - | - | - | - | - | - | - | - | - | - | - | - | - | - | - | - | - | - | - | - | - | - | D | Y | D | A | I | H | R | G | N | P | A | Q | A | F | Y | E | L | V | G | V | - | - | - | - | - | - | - | - | - | - | - | - | - | - | - | -139 |
| ADGB_HUMAN | - | - | - | - | - | - | - | - | - | - | - | - | - | - | - | - | - | - | - | - | - | - | - | - | - | - | - | - | - | G | E | F | T | V | I | H | - | - | - | - | - | A | L | T | G | - | - | - | - | - | - | - | - | - | - | - | - | - | - | - | - | -172 |
| J3QMP3_MOUSE | - | - | - | - | - | - | - | - | - | - | - | - | - | - | - | - | - | - | - | - | - | - | - | - | - | - | - | - | - | G | E | L | T | V | I | H | - | - | - | - | - | A | L | T | G | - | - | - | - | - | - | - | - | - | - | - | - | - | - | - | - | -172 |
| H2XR46_CIOIN | - | - | - | - | - | - | - | - | - | - | - | - | - | - | - | - | - | - | - | - | - | - | - | - | - | - | - | - | - | G | D | F | Y | V | I | H | - | - | - | - | - | A | L | T | G | - | - | - | - | - | - | - | - | - | - | - | - | - | - | - | - | -170 |
| CAN7_MOUSE | - | - | - | - | - | - | - | - | - | - | - | - | - | - | - | - | - | - | - | - | - | - | - | - | - | - | - | - | - | N | S | N | I | D | L | H | - | - | - | - | - | A | L | T | G | - | - | - | - | - | - | - | - | - | - | - | - | - | - | - | - | -142 |
| CAN7_HUMAN | - | - | - | - | - | - | - | - | - | - | - | - | - | - | - | - | - | - | - | - | - | - | - | - | - | - | - | - | - | N | S | N | I | D | L | H | - | - | - | - | - | A | L | T | G | - | - | - | - | - | - | - | - | - | - | - | - | - | - | - | - | -142 |
| E1BY84_CHICK | - | - | - | - | - | - | - | - | - | - | - | - | - | - | - | - | - | - | - | - | - | - | - | - | - | - | - | - | - | N | S | N | I | D | L | H | - | - | - | - | - | A | L | T | G | - | - | - | - | - | - | - | - | - | - | - | - | - | - | - | - | -142 |
| H9GX91_DANRE | - | - | - | - | - | - | - | - | - | - | - | - | - | - | - | - | - | - | - | - | - | - | - | - | - | - | - | - | - | N | S | N | I | D | L | H | - | - | - | - | - | A | L | T | G | - | - | - | - | - | - | - | - | - | - | - | - | - | - | - | - | -142 |
| F6SGI6_CIOIN | - | - | - | - | - | - | - | - | - | - | - | - | - | - | - | - | - | - | - | - | - | - | - | - | - | - | - | - | - | N | S | N | I | D | L | H | - | - | - | - | - | S | L | T | G | - | - | - | - | - | - | - | - | - | - | - | - | - | - | - | - | -142 |
| W4Y195_STRPU | - | - | - | - | - | - | - | - | - | - | - | - | - | - | - | - | - | - | - | - | - | - | - | - | - | - | - | - | - | N | S | N | I | D | L | H | - | - | - | - | - | A | L | T | G | - | - | - | - | - | - | - | - | - | - | - | - | - | - | - | - | -116 |
| R7V270_CAPTE | - | - | - | - | - | - | - | - | - | - | - | - | - | - | - | - | - | - | - | - | - | - | - | - | - | - | - | - | - | N | S | N | I | D | L | H | - | - | - | - | - | A | L | T | G | - | - | - | - | - | - | - | - | - | - | - | - | - | - | - | - | -142 |
| G4LXK3_SCHMA | - | - | - | - | - | - | - | - | - | - | - | - | - | - | - | - | - | - | - | - | - | - | - | - | - | - | - | - | - | N | S | N | I | D | L | H | - | - | - | - | - | A | L | T | G | - | - | - | - | - | - | - | - | - | - | - | - | - | - | - | - | -142 |
| T2M2K0_HYDVU | - | - | - | - | - | - | - | - | - | - | - | - | - | - | - | - | - | - | - | - | - | - | - | - | - | - | - | - | - | N | S | N | I | D | L | N | - | - | - | - | - | A | L | T | G | - | - | - | - | - | - | - | - | - | - | - | - | - | - | - | - | -142 |
| B3RQK1_TRIAD | - | - | - | - | - | - | - | - | - | - | - | - | - | - | - | - | - | - | - | - | - | - | - | - | - | - | - | - | - | N | S | N | I | D | L | H | - | - | - | - | - | A | L | T | G | - | - | - | - | - | - | - | - | - | - | - | - | - | - | - | - | -142 |
| I1CS59_RHIO9 | - | - | - | - | - | - | - | - | - | - | - | - | - | - | - | - | - | - | - | - | - | - | - | - | - | - | - | - | - | N | S | G | I | D | L | H | - | - | - | - | - | C | L | T | G | - | - | - | - | - | - | - | - | - | - | - | - | - | - | - | - | -139 |
| I1GER3_AMPQE | - | - | - | - | - | - | - | - | - | - | - | - | - | - | - | - | - | - | - | - | - | - | - | - | - | - | - | - | - | S | S | N | N | D | L | H | - | - | - | - | - | A | L | T | G | - | - | - | - | - | - | - | - | - | - | - | - | - | - | - | - | -142 |
| Q22143_CAEEL | - | - | - | - | - | - | - | - | - | - | - | - | - | - | - | - | - | - | - | - | - | - | - | - | - | - | - | - | - | N | S | N | I | D | L | N | - | - | - | - | - | A | L | T | G | - | - | - | - | - | - | - | - | - | - | - | - | - | - | - | - | -142 |
| L1JZN7_GUITH | - | - | - | - | - | - | - | - | - | - | - | - | - | - | - | - | - | - | - | - | - | - | - | - | - | - | - | - | - | T | S | S | I | D | L | H | - | - | - | - | - | A | L | C | G | - | - | - | - | - | - | - | - | - | - | - | - | - | - | - | - | -143 |
| D0NT34_PHYIT | - | - | - | - | - | - | - | - | - | - | - | - | - | - | - | - | - | - | - | - | - | - | - | - | - | - | - | - | - | N | S | G | I | D | L | F | - | - | - | - | - | A | L | T | G | - | - | - | - | - | - | - | - | - | - | - | - | - | - | - | - | -151 |
| A0A064B913_ASPOZ | - | - | - | - | - | - | - | - | - | - | - | - | - | - | - | - | - | - | - | - | - | - | - | - | - | - | - | - | - | N | S | G | T | D | L | W | - | - | - | - | - | V | L | T | G | - | - | - | - | - | - | - | - | - | - | - | - | - | - | - | - | -150 |
| A8N4X4_COPC7 | - | - | - | - | - | - | - | - | - | - | - | - | - | - | - | - | - | - | - | - | - | - | - | - | - | - | - | - | - | N | S | S | I | D | L | H | - | - | - | - | - | A | I | T | G | - | - | - | - | - | - | - | - | - | - | - | - | - | - | - | - | -166 |
| C0H5K1_PLAF7 | - | - | - | - | - | - | - | - | - | - | - | - | - | - | - | - | - | - | - | - | - | - | - | - | - | - | - | - | - | N | P | G | S | D | L | Y | - | - | - | - | - | Y | L | T | G | - | - | - | - | - | - | - | - | - | - | - | - | - | - | - | - | -150 |
| K1REZ8_CRAGI | - | - | - | - | - | - | - | - | - | - | - | - | - | - | - | - | - | - | - | - | - | - | - | - | - | - | - | - | - | G | Y | I | H | D | T | Y | - | - | - | - | S | S | L | T | G | - | - | - | - | - | - | - | - | - | - | - | - | - | - | - | - | -130 |
| F4NTM9_BATDJ | - | - | - | - | - | - | - | - | - | - | - | - | - | - | - | - | - | - | - | - | - | - | - | - | - | - | - | - | I | K | P | C | T | S | V | F | - | - | - | - | A | D | L | T | G | - | - | - | - | - | - | - | - | - | - | - | - | - | - | - | - | -147 |
| Q6F6K5_CAEEL | - | - | - | - | - | - | - | - | - | - | - | - | - | - | - | - | - | - | - | - | - | - | - | - | - | - | - | - | - | G | F | A | D | I | A | F | - | - | - | - | L | Q | L | T | G | - | - | - | - | - | - | - | - | - | - | - | - | - | - | - | - | -111 |
| A8HZK8_CHLRE | - | - | - | - | - | - | - | - | - | - | - | - | - | - | - | - | - | - | - | - | - | - | - | - | - | - | - | - | - | G | S | V | H | E | A | L | - | - | - | - | V | D | L | T | G | - | - | - | - | - | - | - | - | - | - | - | - | - | - | - | - | -112 |
| A8J6T6_CHLRE | - | - | - | - | - | - | - | - | - | - | - | - | - | - | - | - | - | - | - | - | - | - | - | - | - | - | - | - | - | V | Q | V | C | W | A | L | A | A | L | T | G | Q | Y | S | T | M | L | L | W | A | E | D | K | - | - | - | - | - | - | - | - | -156 |
| Z9JR51_9MICO | - | - | - | - | - | - | - | - | - | - | - | - | - | - | - | - | - | - | - | - | - | - | - | - | - | - | - | - | - | A | D | T | P | A | A | G | - | - | - | I | E | M | I | T | G | - | - | - | - | - | - | - | - | - | - | - | - | - | - | - | - | -131 |
| F7ZZH9_CELGA | - | - | - | - | - | - | - | - | - | - | - | - | - | - | - | - | - | - | - | - | - | - | - | - | - | - | - | - | - | G | V | P | A | E | A | M | - | - | - | - | E | I | I | T | G | - | - | - | - | - | - | - | - | - | - | - | - | - | - | - | - | -113 |
| Q7NDU2_GLOVI | - | - | - | - | - | - | - | - | - | - | - | - | - | - | - | - | - | - | - | - | - | - | - | - | - | - | - | - | - | - | G | D | G | N | N | F | E | N | T | I | R | A | V | T | G | - | - | - | - | - | - | - | - | - | - | - | - | - | - | - | - | -102 |
| I6AT95_9BACT | - | - | - | - | - | - | - | - | - | - | - | - | - | - | - | - | - | - | - | - | - | - | - | - | - | - | - | - | L | L | A | K | G | G | S | A | G | S | T | I | E | V | V | T | G | - | - | - | - | - | - | - | - | - | - | - | - | - | - | - | - | -117 |
| C7MGJ8_BRAFD | - | - | - | - | - | - | - | - | - | - | - | - | - | - | - | - | - | - | - | - | - | - | - | - | - | - | - | - | - | G | W | D | Q | A | A | P | - | - | - | - | - | Y | L | T | P | E | - | - | - | - | - | - | - | - | - | - | - | - | - | - | - | -128 |
| D0WP73_9ACTO | - | - | - | - | - | - | - | - | - | - | - | - | - | - | - | - | - | - | - | - | - | - | - | - | - | - | - | - | N | G | I | S | G | G | W | P | N | E | A | T | Q | D | L | T | N | - | - | - | - | - | - | - | - | - | - | - | - | - | - | - | - | -127 |
| B0C8L1_ACAM1 | - | - | - | - | - | - | - | - | - | - | - | - | - | - | - | - | - | - | - | - | - | - | - | - | - | - | - | - | - | R | R | S | S | R | S | Y | N | - | - | - | - | V | F | D | D | - | - | - | - | - | - | - | - | - | - | - | - | - | - | - | - | -119 |
| B3E5A8_GEOLS | - | - | - | - | - | - | - | - | - | - | - | - | - | - | - | - | - | - | - | - | - | - | - | - | - | T | V | A | I | D | T | A | G | I | P | I | G | S | V | V | K | R | W | T | G | - | - | - | - | - | - | - | - | - | - | - | - | - | - | - | - | -121 |
| D4YQ94_9MICO | - | - | - | - | - | - | - | - | - | - | - | - | - | - | - | - | - | - | - | - | - | - | - | - | - | - | - | - | - | - | F | A | G | T | A | L | - | - | - | - | W | L | L | T | G | - | - | - | - | - | - | - | - | - | - | - | - | - | - | - | - | -112 |
| PAPA_CARPA | - | - | - | - | - | - | - | - | - | - | - | - | - | - | - | - | - | - | - | - | - | - | - | - | - | - | - | - | - | Y | C | R | S | R | E | K | G | P | Y | A | A | K | T | D | G | - | - | - | - | - | - | - | - | - | - | - | - | - | - | - | - | -109 |
| RIM13_YEAST | - | - | - | - | - | - | - | - | - | - | - | - | - | - | - | - | - | - | - | - | - | - | - | - | - | - | - | - | - | N | I | S | I | D | T | Y | - | - | - | - | - | R | L | S | G | - | - | - | - | - | - | - | - | - | - | - | - | - | - | - | - | -144 |

| I |  |  |  |  |  |  |  |  |  |  |  |  |  |  |  |  |  |  |  |  |  |  |  |  |  |  |  |  |  |  |  |  |  |  |  |  |  |  |  |  |  |  |  |  |  |  |  |  |  |  |  |  |  |  |  |  |  |  |  |  |  |  |
| --- | --- | --- | --- | --- | --- | --- | --- | --- | --- | --- | --- | --- | --- | --- | --- | --- | --- | --- | --- | --- | --- | --- | --- | --- | --- | --- | --- | --- | --- | --- | --- | --- | --- | --- | --- | --- | --- | --- | --- | --- | --- | --- | --- | --- | --- | --- | --- | --- | --- | --- | --- | --- | --- | --- | --- | --- | --- | --- | --- | --- | --- | --- |
| Q9N4B1_CAEEL | - | - | - | - | - | - | - | - | - | - | - | - | - | - | - | - | - | - | - | G | L | T | E | F | F | D | I | S | K | G | D | K | S | - | - | - | - | - | - | - | - | - | - | - | - | - | - | - | - | - | - | - | - | - | - | - | - | - | - | - | - | -149 |
| Q8IAA8_CAEEL | - | - | - | - | - | - | - | - | - | - | - | - | - | - | - | - | - | - | - | G | L | T | E | Y | F | D | L | R | K | S | E | K | A | - | - | - | - | - | - | - | - | - | - | - | - | - | - | - | - | - | - | - | - | - | - | - | - | - | - | - | - | -149 |
| CAN_CAEEL | - | - | - | - | - | - | - | - | - | - | - | - | - | - | - | - | - | - | - | G | L | T | E | F | I | D | L | K | N | P | P | R | - | - | - | - | - | - | - | - | - | - | - | - | - | - | - | - | - | - | - | - | - | - | - | - | - | - | - | - | - | -148 |
| Q9U2B2_CAEEL | - | - | - | - | - | - | - | - | - | - | - | - | - | - | - | - | - | - | - | G | L | T | Q | W | F | D | I | F | M | A | D | E | M | - | - | - | - | - | - | - | - | - | - | - | - | - | - | - | - | - | - | - | - | - | - | - | - | - | - | - | - | -153 |
| Q9N4B2_CAEEL | - | - | - | - | - | - | - | - | - | - | - | - | - | - | - | - | - | - | - | G | L | T | E | F | Y | D | L | T | S | T | D | K | T | - | - | - | - | - | - | - | - | - | - | - | - | - | - | - | - | - | - | - | - | - | - | - | - | - | - | - | - | -149 |
| C3Y6P6_BRAFL | - | - | - | - | - | - | - | - | - | - | - | - | - | - | - | - | - | - | - | G | V | T | E | T | Y | - | M | L | D | P | N | N | V | P | P | - | - | - | - | - | - | - | - | - | - | - | - | - | - | - | - | - | - | - | - | - | - | - | - | - | - | -156 |
| C3Y6N1_BRAFL | - | - | - | - | - | - | - | - | - | - | - | - | - | - | - | - | - | - | - | G | M | T | E | R | Y | D | T | T | D | P | S | K | L | P | G | - | - | - | - | - | - | - | - | - | - | - | - | - | - | - | - | - | - | - | - | - | - | - | - | - | - | -158 |
| CANA_DROME | - | - | - | - | - | - | - | - | - | - | - | - | - | - | - | - | - | - | - | G | V | S | E | W | Y | D | L | K | - | E | - | A | P | G | - | - | - | - | - | - | - | - | - | - | - | - | - | - | - | - | - | - | - | - | - | - | - | - | - | - | - | -148 |
| CANB_DROME | - | - | - | - | - | - | - | - | - | - | - | - | - | - | - | - | - | - | - | G | V | T | E | W | Y | D | I | K | - | E | - | A | P | P | - | - | - | - | - | - | - | - | - | - | - | - | - | - | - | - | - | - | - | - | - | - | - | - | - | - | - | -148 |
| X1Y3H71_ANODA | - | - | - | - | - | - | - | - | - | - | - | - | - | - | - | - | - | - | - | G | V | A | E | M | Y | D | L | K | D | Q | - | T | P | P | - | - | - | - | - | - | - | - | - | - | - | - | - | - | - | - | - | - | - | - | - | - | - | - | - | - | - | -149 |
| Q5IW12_GECLA | - | - | - | - | - | - | - | - | - | - | - | - | - | - | - | - | - | - | - | G | V | S | E | I | Y | D | L | T | - | K | - | A | P | P | - | - | - | - | - | - | - | - | - | - | - | - | - | - | - | - | - | - | - | - | - | - | - | - | - | - | - | -148 |
| K1Q056_CRAGI | - | - | - | - | - | - | - | - | - | - | - | - | - | - | - | - | - | - | - | G | V | S | E | F | F | D | L | R | - | K | - | A | P | P | - | - | - | - | - | - | - | - | - | - | - | - | - | - | - | - | - | - | - | - | - | - | - | - | - | - | - | -148 |
| G4V6C0_SCHMA | - | - | - | - | - | - | - | - | - | - | - | - | - | - | - | - | - | - | - | G | L | T | E | L | V | D | L | G | A | K | - | A | P | T | - | - | - | - | - | - | - | - | - | - | - | - | - | - | - | - | - | - | - | - | - | - | - | - | - | - | - | -153 |
| R7ULX9_CAPTE | - | - | - | - | - | - | - | - | - | - | - | - | - | - | - | - | - | - | - | G | I | A | E | K | F | E | L | R | E | N | - | A | P | E | - | - | - | - | - | - | - | - | - | - | - | - | - | - | - | - | - | - | - | - | - | - | - | - | - | - | - | -149 |
| W4Z5Q7_STRPU | - | - | - | - | - | - | - | - | - | - | - | - | - | - | - | - | - | - | - | G | V | S | E | S | I | D | L | N | Q | N | - | P | P | K | - | - | - | - | - | - | - | - | - | - | - | - | - | - | - | - | - | - | - | - | - | - | - | - | - | - | - | -146 |
| R7VIB6_CAPTE | - | - | - | - | - | - | - | - | - | - | - | - | - | - | - | - | - | - | - | G | I | V | E | H | Y | E | L | T | K | N | - | V | P | D | - | - | - | - | - | - | - | - | - | - | - | - | - | - | - | - | - | - | - | - | - | - | - | - | - | - | - | -152 |
| T2MJ45_HYDVU | - | - | - | - | - | - | - | - | - | - | - | - | - | - | - | - | - | - | - | G | V | T | E | S | F | N | T | Q | K | - | - | A | P | K | - | - | - | - | - | - | - | - | - | - | - | - | - | - | - | - | - | - | - | - | - | - | - | - | - | - | - | -148 |
| R7UAB6_CAPTE | - | - | - | - | - | - | - | - | - | - | - | - | - | - | - | - | - | - | - | G | V | T | E | M | F | D | L | R | Q | - | - | A | P | P | - | - | - | - | - | - | - | - | - | - | - | - | - | - | - | - | - | - | - | - | - | - | - | - | - | - | - | -148 |
| C3ZKN8_BRAFL | - | - | - | - | - | - | - | - | - | - | - | - | - | - | - | - | - | - | - | G | V | T | E | G | M | D | L | R | N | - | - | A | P | K | - | - | - | - | - | - | - | - | - | - | - | - | - | - | - | - | - | - | - | - | - | - | - | - | - | - | - | -149 |
| B3RJS5_TRIAD | - | - | - | - | - | - | - | - | - | - | - | - | - | - | - | - | - | - | - | G | V | T | E | V | I | D | L | K | K | - | - | P | P | N | - | - | - | - | - | - | - | - | - | - | - | - | - | - | - | - | - | - | - | - | - | - | - | - | - | - | - | -148 |
| H2YVF0_CIOSA | - | - | - | - | - | - | - | - | - | - | - | - | - | - | - | - | - | - | - | G | V | T | E | V | I | D | L | K | E | - | - | A | P | N | - | - | - | - | - | - | - | - | - | - | - | - | - | - | - | - | - | - | - | - | - | - | - | - | - | - | - | -148 |
| G4VAG2_SCHMA | - | - | - | - | - | - | - | - | - | - | - | - | - | - | - | - | - | - | - | G | I | C | L | S | L | E | L | N | Q | K | E | R | P | S | - | - | - | - | - | - | - | - | - | - | - | - | - | - | - | - | - | - | - | - | - | - | - | - | - | - | - | -153 |
| Smp_157500 | - | - | - | - | - | - | - | - | - | - | - | - | - | - | - | - | - | - | - | G | I | C | T | T | L | I | L | N | K | K | E | R | P | P | - | - | - | - | - | - | - | - | - | - | - | - | - | - | - | - | - | - | - | - | - | - | - | - | - | - | - | -153 |
| Q7SYA9_DANRE | - | - | - | - | - | - | - | - | - | - | - | - | - | - | - | - | - | - | - | G | V | S | E | M | Y | E | L | R | K | A | P | R | - | - | - | - | - | - | - | - | - | - | - | - | - | - | - | - | - | - | - | - | - | - | - | - | - | - | - | - | - | -148 |
| A5PMP1_DANRE | - | - | - | - | - | - | - | - | - | - | - | - | - | - | - | - | - | - | - | G | V | A | E | M | Y | E | L | R | S | A | P | K | - | - | - | - | - | - | - | - | - | - | - | - | - | - | - | - | - | - | - | - | - | - | - | - | - | - | - | - | - | -148 |
| CANX_CHICK | - | - | - | - | - | - | - | - | - | - | - | - | - | - | - | - | - | - | - | G | V | A | E | M | Y | D | L | K | R | A | P | R | - | - | - | - | - | - | - | - | - | - | - | - | - | - | - | - | - | - | - | - | - | - | - | - | - | - | - | - | - | -148 |
| CAN1_HUMAN | - | - | - | - | - | - | - | - | - | - | - | - | - | - | - | - | - | - | - | G | V | T | E | W | Y | E | L | R | K | A | P | S | - | - | - | - | - | - | - | - | - | - | - | - | - | - | - | - | - | - | - | - | - | - | - | - | - | - | - | - | - | -148 |
| CAN1_MOUSE | - | - | - | - | - | - | - | - | - | - | - | - | - | - | - | - | - | - | - | G | V | T | E | W | Y | D | L | Q | K | A | P | S | - | - | - | - | - | - | - | - | - | - | - | - | - | - | - | - | - | - | - | - | - | - | - | - | - | - | - | - | - | -148 |
| O42133_CHICK | - | - | - | - | - | - | - | - | - | - | - | - | - | - | - | - | - | - | - | G | V | T | E | W | Y | D | L | R | K | P | P | A | - | - | - | - | - | - | - | - | - | - | - | - | - | - | - | - | - | - | - | - | - | - | - | - | - | - | - | - | - | -148 |
| Q7ZUR1_DANRE | - | - | - | - | - | - | - | - | - | - | - | - | - | - | - | - | - | - | - | G | V | T | E | M | Y | E | L | K | K | A | P | A | - | - | - | - | - | - | - | - | - | - | - | - | - | - | - | - | - | - | - | - | - | - | - | - | - | - | - | - | - | -148 |
| E9QFN0_DANRE | - | - | - | - | - | - | - | - | - | - | - | - | - | - | - | - | - | - | - | G | V | T | E | M | Y | E | L | K | N | A | P | P | - | - | - | - | - | - | - | - | - | - | - | - | - | - | - | - | - | - | - | - | - | - | - | - | - | - | - | - | - | -148 |
| CAN2_HUMAN | - | - | - | - | - | - | - | - | - | - | - | - | - | - | - | - | - | - | - | G | I | A | E | W | Y | E | L | K | K | P | P | P | - | - | - | - | - | - | - | - | - | - | - | - | - | - | - | - | - | - | - | - | - | - | - | - | - | - | - | - | - | -148 |
| CAN2_MOUSE | - | - | - | - | - | - | - | - | - | - | - | - | - | - | - | - | - | - | - | G | I | A | E | W | Y | E | L | R | K | P | P | P | - | - | - | - | - | - | - | - | - | - | - | - | - | - | - | - | - | - | - | - | - | - | - | - | - | - | - | - | - | -148 |
| CAN2_CHICK | - | - | - | - | - | - | - | - | - | - | - | - | - | - | - | - | - | - | - | G | I | A | E | W | Y | E | L | Q | K | A | P | P | - | - | - | - | - | - | - | - | - | - | - | - | - | - | - | - | - | - | - | - | - | - | - | - | - | - | - | - | - | -148 |
| Q6DFZ8_DANRE | - | - | - | - | - | - | - | - | - | - | - | - | - | - | - | - | - | - | - | G | I | A | E | V | H | E | L | A | K | A | G | P | - | - | - | - | - | - | - | - | - | - | - | - | - | - | - | - | - | - | - | - | - | - | - | - | - | - | - | - | - | -148 |
| Q504F5_DANRE | - | - | - | - | - | - | - | - | - | - | - | - | - | - | - | - | - | - | - | G | I | A | E | S | Y | E | I | R | K | A | P | T | - | - | - | - | - | - | - | - | - | - | - | - | - | - | - | - | - | - | - | - | - | - | - | - | - | - | - | - | - | -148 |
| Q5BLH5_DANRE | - | - | - | - | - | - | - | - | - | - | - | - | - | - | - | - | - | - | - | G | I | A | E | M | Y | E | L | K | S | A | P | T | - | - | - | - | - | - | - | - | - | - | - | - | - | - | - | - | - | - | - | - | - | - | - | - | - | - | - | - | - | -148 |
| Q4VBH3_DANRE | - | - | - | - | - | - | - | - | - | - | - | - | - | - | - | - | - | - | - | G | I | T | E | N | Y | E | L | S | K | A | P | P | - | - | - | - | - | - | - | - | - | - | - | - | - | - | - | - | - | - | - | - | - | - | - | - | - | - | - | - | - | -148 |
| CAN8_RAT | - | - | - | - | - | - | - | - | - | - | - | - | - | - | - | - | - | - | - | G | I | S | E | F | Y | D | L | K | K | P | P | E | - | - | - | - | - | - | - | - | - | - | - | - | - | - | - | - | - | - | - | - | - | - | - | - | - | - | - | - | - | -148 |
| CAN8_MOUSE | - | - | - | - | - | - | - | - | - | - | - | - | - | - | - | - | - | - | - | G | I | S | E | F | Y | D | L | R | K | P | P | G | - | - | - | - | - | - | - | - | - | - | - | - | - | - | - | - | - | - | - | - | - | - | - | - | - | - | - | - | - | -148 |
| CAN8_HUMAN | - | - | - | - | - | - | - | - | - | - | - | - | - | - | - | - | - | - | - | G | I | S | E | F | Y | D | L | K | K | P | P | A | - | - | - | - | - | - | - | - | - | - | - | - | - | - | - | - | - | - | - | - | - | - | - | - | - | - | - | - | - | -148 |
| XP_426117 | - | - | - | - | - | - | - | - | - | - | - | - | - | - | - | - | - | - | - | G | I | S | E | S | Y | D | L | R | R | A | P | A | - | - | - | - | - | - | - | - | - | - | - | - | - | - | - | - | - | - | - | - | - | - | - | - | - | - | - | - | - | -148 |
| CAN9_MOUSE | - | - | - | - | - | - | - | - | - | - | - | - | - | - | - | - | - | - | - | G | V | A | E | N | F | Q | I | R | E | A | P | E | - | - | - | - | - | - | - | - | - | - | - | - | - | - | - | - | - | - | - | - | - | - | - | - | - | - | - | - | - | -150 |
| CAN9_HUMAN | - | - | - | - | - | - | - | - | - | - | - | - | - | - | - | - | - | - | - | G | V | A | E | T | F | Q | T | K | E | A | P | E | - | - | - | - | - | - | - | - | - | - | - | - | - | - | - | - | - | - | - | - | - | - | - | - | - | - | - | - | - | -148 |
| E1C6M3_CHICK | - | - | - | - | - | - | - | - | - | - | - | - | - | - | - | - | - | - | - | G | V | G | E | M | Y | E | V | K | K | A | P | D | - | - | - | - | - | - | - | - | - | - | - | - | - | - | - | - | - | - | - | - | - | - | - | - | - | - | - | - | - | -148 |
| Q6DG44_DANRE | - | - | - | - | - | - | - | - | - | - | - | - | - | - | - | - | - | - | - | G | V | G | E | M | Y | E | T | K | N | G | P | S | - | - | - | - | - | - | - | - | - | - | - | - | - | - | - | - | - | - | - | - | - | - | - | - | - | - | - | - | - | -148 |
| CAN3_HUMAN | - | - | - | - | - | - | - | - | - | - | - | - | - | - | - | - | - | - | - | G | V | A | E | F | F | E | I | R | D | A | P | S | - | - | - | - | - | - | - | - | - | - | - | - | - | - | - | - | - | - | - | - | - | - | - | - | - | - | - | - | - | -148 |
| CAN3_MOUSE | - | - | - | - | - | - | - | - | - | - | - | - | - | - | - | - | - | - | - | G | V | T | E | F | F | E | I | K | D | A | P | S | - | - | - | - | - | - | - | - | - | - | - | - | - | - | - | - | - | - | - | - | - | - | - | - | - | - | - | - | - | -148 |
| CAN3_CHICK | - | - | - | - | - | - | - | - | - | - | - | - | - | - | - | - | - | - | - | G | V | I | E | F | Y | E | I | K | D | A | P | K | - | - | - | - | - | - | - | - | - | - | - | - | - | - | - | - | - | - | - | - | - | - | - | - | - | - | - | - | - | -148 |
| Q66HU4_DANRE | - | - | - | - | - | - | - | - | - | - | - | - | - | - | - | - | - | - | - | G | V | T | E | F | Y | E | M | K | E | A | P | K | - | - | - | - | - | - | - | - | - | - | - | - | - | - | - | - | - | - | - | - | - | - | - | - | - | - | - | - | - | -148 |
| F1R632_DANRE | - | - | - | - | - | - | - | - | - | - | - | - | - | - | - | - | - | - | - | G | V | T | E | F | Y | E | I | T | E | A | P | K | - | - | - | - | - | - | - | - | - | - | - | - | - | - | - | - | - | - | - | - | - | - | - | - | - | - | - | - | - | -148 |
| CAN11_HUMAN | - | - | - | - | - | - | - | - | - | - | - | - | - | - | - | - | - | - | - | G | V | A | Q | S | F | Q | L | Q | R | P | P | Q | - | - | - | - | - | - | - | - | - | - | - | - | - | - | - | - | - | - | - | - | - | - | - | - | - | - | - | - | - | -148 |
| CAN11_MOUSE | - | - | - | - | - | - | - | - | - | - | - | - | - | - | - | - | - | - | - | G | V | A | Q | C | I | P | L | Q | K | P | P | G | - | - | - | - | - | - | - | - | - | - | - | - | - | - | - | - | - | - | - | - | - | - | - | - | - | - | - | - | - | -148 |
| CAN12_MOUSE | - | - | - | - | - | - | - | - | - | - | - | - | - | - | - | - | - | - | - | G | V | G | E | V | L | Y | L | R | Q | N | T | P | - | - | - | - | - | - | - | - | - | - | - | - | - | - | - | - | - | - | - | - | - | - | - | - | - | - | - | - | - | -148 |
| CAN12_HUMAN | - | - | - | - | - | - | - | - | - | - | - | - | - | - | - | - | - | - | - | G | V | G | E | V | L | Y | L | R | Q | N | S | M | - | - | - | - | - | - | - | - | - | - | - | - | - | - | - | - | - | - | - | - | - | - | - | - | - | - | - | - | - | -148 |
| ENSSARP00000008135 | - | - | - | - | - | - | - | - | - | - | - | - | - | - | - | - | - | - | - | X | X | X | X | X | X | X | X | X | X | X | X | X | - | - | - | - | - | - | - | - | - | - | - | - | - | - | - | - | - | - | - | - | - | - | - | - | - | - | - | - | - | -148 |
| ENSSARP00000010553 | - | - | - | - | - | - | - | - | - | - | - | - | - | - | - | - | - | - | - | X | X | X | X | X | X | X | X | X | X | X | X | X | - | - | - | - | - | - | - | - | - | - | - | - | - | - | - | - | - | - | - | - | - | - | - | - | - | - | - | - | - | -147 |
| A7E2G3_DANRE | - | - | - | - | - | - | - | - | - | - | - | - | - | - | - | - | - | - | - | G | I | A | Y | S | L | P | V | S | S | R | P | P | R | - | - | - | - | - | - | - | - | - | - | - | - | - | - | - | - | - | - | - | - | - | - | - | - | - | - | - | - | -149 |
| Q6NW69_DANRE | - | - | - | - | - | - | - | - | - | - | - | - | - | - | - | - | - | - | - | G | I | T | E | V | F | M | V | A | S | L | P | R | - | - | - | - | - | - | - | - | - | - | - | - | - | - | - | - | - | - | - | - | - | - | - | - | - | - | - | - | - | -148 |
| CAN13_HUMAN | - | - | - | - | - | - | - | - | - | - | - | - | - | - | - | - | - | - | - | G | V | I | T | N | I | H | L | H | S | S | P | V | - | - | - | - | - | - | - | - | - | - | - | - | - | - | - | - | - | - | - | - | - | - | - | - | - | - | - | - | - | -149 |
| CAN13_MOUSE | - | - | - | - | - | - | - | - | - | - | - | - | - | - | - | - | - | - | - | G | V | V | T | I | I | N | L | H | S | S | P | F | - | - | - | - | - | - | - | - | - | - | - | - | - | - | - | - | - | - | - | - | - | - | - | - | - | - | - | - | - | -151 |
| E1BRR5_CHICK | - | - | - | - | - | - | - | - | - | - | - | - | - | - | - | - | - | - | - | G | V | Q | M | Q | F | S | L | K | D | P | P | P | - | - | - | - | - | - | - | - | - | - | - | - | - | - | - | - | - | - | - | - | - | - | - | - | - | - | - | - | - | -148 |
| E7F0H3_DANRE | - | - | - | - | - | - | - | - | - | - | - | - | - | - | - | - | - | - | - | G | T | H | M | Y | Y | T | L | S | S | A | P | A | - | - | - | - | - | - | - | - | - | - | - | - | - | - | - | - | - | - | - | - | - | - | - | - | - | - | - | - | - | -149 |
| Q1RLS9_DANRE | - | - | - | - | - | - | - | - | - | - | - | - | - | - | - | - | - | - | - | G | P | H | M | T | I | K | L | S | Q | A | S | D | - | - | - | - | - | - | - | - | - | - | - | - | - | - | - | - | - | - | - | - | - | - | - | - | - | - | - | - | - | -148 |
| F1Q9Z1_DANRE | - | - | - | - | - | - | - | - | - | - | - | - | - | - | - | - | - | - | - | G | V | H | M | H | Y | D | L | K | T | A | P | T | - | - | - | - | - | - | - | - | - | - | - | - | - | - | - | - | - | - | - | - | - | - | - | - | - | - | - | - | - | -149 |
| A4QNV6_DANRE | - | - | - | - | - | - | - | - | - | - | - | - | - | - | - | - | - | - | - | G | I | H | V | R | F | E | L | E | K | P | P | L | - | - | - | - | - | - | - | - | - | - | - | - | - | - | - | - | - | - | - | - | - | - | - | - | - | - | - | - | - | -149 |
| D6W573_HUMAN | - | - | - | - | - | - | - | - | - | - | - | - | - | - | - | - | - | - | - | G | V | T | M | T | I | N | L | A | E | A | H | G | - | - | - | - | - | - | - | - | - | - | - | - | - | - | - | - | - | - | - | - | - | - | - | - | - | - | - | - | - | -149 |
| R7UBC6_CAPTE | - | - | - | - | - | - | - | - | - | - | - | - | - | - | - | - | - | - | - | G | L | V | E | V | F | N | F | E | E | E | K | P | - | T | Q | E | - | - | - | - | - | - | - | - | - | - | - | - | - | - | - | - | - | - | - | - | - | - | - | - | - | -156 |
| K1RCZ2_CRAGI | - | - | - | - | - | - | - | - | - | - | - | - | - | - | - | - | - | - | - | G | I | C | E | G | Y | T | L | R | G | V | N | A | N | V | P | R | - | - | - | - | - | - | - | - | - | - | - | - | - | - | - | - | - | - | - | - | - | - | - | - | - | -159 |
| Q5IW13_GECLA | - | - | - | - | - | - | - | - | - | - | - | - | - | - | - | - | - | - | - | G | V | V | E | L | I | D | L | R | S | P | A | S | - | - | - | - | - | - | - | - | - | - | - | - | - | - | - | - | - | - | - | - | - | - | - | - | - | - | - | - | - | -148 |
| R7UUX7_CAPTE | - | - | - | - | - | - | - | - | - | - | - | - | - | - | - | - | - | - | - | G | I | S | E | V | I | H | L | N | - | - | - | E | A | P | A | - | - | - | - | - | - | - | - | - | - | - | - | - | - | - | - | - | - | - | - | - | - | - | - | - | - | -151 |
| K1Q505_CRAGI | - | - | - | - | - | - | - | - | - | - | - | - | - | - | - | - | - | - | - | G | I | S | E | T | I | D | I | K | D | K | A | Q | I | P | V | - | - | - | - | - | - | - | - | - | - | - | - | - | - | - | - | - | - | - | - | - | - | - | - | - | - | -152 |
| R7UD02_CAPTE | - | - | - | - | - | - | - | - | - | - | - | - | - | - | - | - | - | - | - | G | I | G | E | F | I | D | V | K | N | - | I | P | D | R | S | - | - | - | - | - | - | - | - | - | - | - | - | - | - | - | - | - | - | - | - | - | - | - | - | - | - | -151 |
| CAN6_HUMAN | - | - | - | - | - | - | - | - | - | - | - | - | - | - | - | - | - | - | - | T | L | A | E | T | V | D | M | Q | K | G | R | Y | T | E | L | V | E | E | K | - | - | - | - | - | - | - | - | - | - | - | - | - | - | - | - | - | - | - | - | - | - | -161 |
| CAN6_MOUSE | - | - | - | - | - | - | - | - | - | - | - | - | - | - | - | - | - | - | - | T | L | A | E | I | I | D | M | Q | K | G | R | Y | T | D | L | V | E | E | K | - | - | - | - | - | - | - | - | - | - | - | - | - | - | - | - | - | - | - | - | - | - | -161 |
| E1C312_CHICK | - | - | - | - | - | - | - | - | - | - | - | - | - | - | - | - | - | - | - | A | V | A | E | S | I | D | L | V | Q | G | K | Y | S | E | T | I | S | E | Q | - | - | - | - | - | - | - | - | - | - | - | - | - | - | - | - | - | - | - | - | - | - | -161 |
| E7FDN3_DANRE | - | - | - | - | - | - | - | - | - | - | - | - | - | - | - | - | - | - | - | G | V | S | E | P | L | S | L | N | Q | E | E | L | I | Q | H | S | D | H | R | - | - | - | - | - | - | - | - | - | - | - | - | - | - | - | - | - | - | - | - | - | - | -162 |
| CAN5_HUMAN | - | - | - | - | - | - | - | - | - | - | - | - | - | - | - | - | - | - | - | G | V | S | E | P | I | D | L | T | E | G | D | F | A | N | D | E | T | K | R | - | - | - | - | - | - | - | - | - | - | - | - | - | - | - | - | - | - | - | - | - | - | -157 |
| CAN5_MOUSE | - | - | - | - | - | - | - | - | - | - | - | - | - | - | - | - | - | - | - | G | V | S | E | P | I | D | L | T | E | G | D | L | A | T | D | E | A | K | R | - | - | - | - | - | - | - | - | - | - | - | - | - | - | - | - | - | - | - | - | - | - | -161 |
| E1C292_CHICK | - | - | - | - | - | - | - | - | - | - | - | - | - | - | - | - | - | - | - | G | V | S | E | P | I | D | L | T | E | G | D | Y | T | T | D | E | A | K | R | - | - | - | - | - | - | - | - | - | - | - | - | - | - | - | - | - | - | - | - | - | - | -161 |
| A0JMG3_DANRE | - | - | - | - | - | - | - | - | - | - | - | - | - | - | - | - | - | - | - | G | V | S | E | P | M | D | L | L | E | G | K | F | A | Q | D | E | E | A | R | - | - | - | - | - | - | - | - | - | - | - | - | - | - | - | - | - | - | - | - | - | - | -161 |
| E7F7F1_DANRE | - | - | - | - | - | - | - | - | - | - | - | - | - | - | - | - | - | - | - | G | V | S | E | P | M | D | L | L | E | G | Q | F | A | Q | D | E | T | A | R | - | - | - | - | - | - | - | - | - | - | - | - | - | - | - | - | - | - | - | - | - | - | -161 |
| XP_006824412 | - | - | - | - | - | - | - | - | - | - | - | - | - | - | - | - | - | - | - | G | V | A | E | N | I | D | L | M | E | P | - | - | A | V | D | E | E | K | R | - | - | - | - | - | - | - | - | - | - | - | - | - | - | - | - | - | - | - | - | - | - | -153 |
| W4YJN2_STRPU | - | - | - | - | - | - | - | - | - | - | - | - | - | - | - | - | - | - | - | G | V | A | E | Q | I | N | L | A | D | P | E | F | Q | A | D | Q | N | K | K | - | - | - | - | - | - | - | - | - | - | - | - | - | - | - | - | - | - | - | - | - | - | -161 |
| C3YJZ3_BRAFL | - | - | - | - | - | - | - | - | - | - | - | - | - | - | - | - | - | - | - | G | V | A | E | P | I | A | L | D | K | G | G | Y | R | E | D | E | E | K | K | - | - | - | - | - | - | - | - | - | - | - | - | - | - | - | - | - | - | - | - | - | - | -161 |
| T2MIT4_HYDVU | - | - | - | - | - | - | - | - | - | - | - | - | - | - | - | - | - | - | - | G | V | S | E | T | I | D | L | V | K | E | D | Y | H | N | N | F | E | K | K | - | - | - | - | - | - | - | - | - | - | - | - | - | - | - | - | - | - | - | - | - | - | -161 |
| XP_002154722 | - | - | - | - | - | - | - | - | - | - | - | - | - | - | - | - | - | - | - | G | V | S | E | R | I | D | L | V | E | G | D | Y | H | N | N | L | E | K | K | - | - | - | - | - | - | - | - | - | - | - | - | - | - | - | - | - | - | - | - | - | - | -125 |
| R7VLA7_CAPTE | - | - | - | - | - | - | - | - | - | - | - | - | - | - | - | - | - | - | - | G | V | S | E | P | L | N | L | V | D | L | K | V | G | I | E | E | E | A | R | - | - | - | - | - | - | - | - | - | - | - | - | - | - | - | - | - | - | - | - | - | - | -161 |
| K1R1C6_CRAGI | - | - | - | - | - | - | - | - | - | - | - | - | - | - | - | - | - | - | - | G | V | S | V | T | V | N | M | V | K | L | N | L | A | N | D | A | Q | E | R | - | - | - | - | - | - | - | - | - | - | - | - | - | - | - | - | - | - | - | - | - | - | -161 |
| B3RIE5_TRIAD | - | - | - | - | - | - | - | - | - | - | - | - | - | - | - | - | - | - | - | G | I | A | E | T | I | K | M | D | G | E | D | Y | S | - | D | D | N | N | R | - | - | - | - | - | - | - | - | - | - | - | - | - | - | - | - | - | - | - | - | - | - | -159 |
| K1PW75_CRAGI | - | - | - | - | - | - | - | - | - | - | - | - | - | - | - | - | - | - | - | G | V | G | E | A | L | E | L | - | - | R | D | Y | T | K | T | E | E | D | K | - | - | - | - | - | - | - | - | - | - | - | - | - | - | - | - | - | - | - | - | - | - | -157 |
| K1RSB8_CRAGI | - | - | - | - | - | - | - | - | - | - | - | - | - | - | - | - | - | - | - | G | V | A | E | G | I | E | I | - | - | R | D | Y | - | K | N | E | E | E | R | - | - | - | - | - | - | - | - | - | - | - | - | - | - | - | - | - | - | - | - | - | - | -156 |
| K1RWI3_CRAGI | - | - | - | - | - | - | - | - | - | - | - | - | - | - | - | - | - | - | - | G | V | G | E | D | I | T | M | - | - | A | D | Y | T | D | S | G | E | K | K | - | - | - | - | - | - | - | - | - | - | - | - | - | - | - | - | - | - | - | - | - | - | -159 |
| R7VGC2_CAPTE | - | - | - | - | - | - | - | - | - | - | - | - | - | - | - | - | - | - | - | G | V | A | E | K | F | N | L | S | E | I | N | L | Q | V | - | E | D | M | R | - | - | - | - | - | - | - | - | - | - | - | - | - | - | - | - | - | - | - | - | - | - | -159 |
| K1QU26_CRAGI | - | - | - | - | - | - | - | - | - | - | - | - | - | - | - | - | - | - | - | G | V | A | E | K | L | V | L | T | R | L | D | L | N | D | - | T | K | I | V | - | - | - | - | - | - | - | - | - | - | - | - | - | - | - | - | - | - | - | - | - | - | -161 |
| CAN5_CAEEL | - | - | - | - | - | - | - | - | - | - | - | - | - | - | - | - | - | - | - | G | V | A | E | T | L | H | V | R | K | F | L | K | D | D | P | N | D | T | E | - | - | - | - | - | - | - | - | - | - | - | - | - | - | - | - | - | - | - | - | - | - | -159 |
| I1FGV4_AMPQE | - | - | - | - | - | - | - | - | - | - | - | - | - | - | - | - | - | - | - | G | V | V | E | T | Y | D | L | K | K | C | D | - | - | - | - | - | - | - | - | - | - | - | - | - | - | - | - | - | - | - | - | - | - | - | - | - | - | - | - | - | - | -147 |
| K1PHX3_CRAGI | - | - | - | - | - | - | - | - | - | - | - | - | - | - | - | - | - | - | - | G | V | T | E | N | H | Q | V | Y | - | - | - | - | - | - | - | E | N | P | R | - | - | - | - | - | - | - | - | - | - | - | - | - | - | - | - | - | - | - | - | - | - | -148 |
| B3RNL8_TRIAD | - | - | - | - | - | - | - | - | - | - | - | - | - | - | - | - | - | - | - | G | I | H | E | D | Y | H | M | P | - | - | - | - | - | - | - | S | M | P | A | - | - | - | - | - | - | - | - | - | - | - | - | - | - | - | - | - | - | - | - | - | - | -150 |
| X1Y3H7_ANODA | - | - | - | - | - | - | - | - | - | - | - | - | - | - | - | - | - | - | - | G | M | A | E | T | H | L | L | K | G | W | Q | P | - | - | - | - | - | - | - | - | - | - | - | - | - | - | - | - | - | - | - | - | - | - | - | - | - | - | - | - | - | -152 |
| R7TWH9_CAPTE | - | - | - | - | - | - | - | - | - | - | - | - | - | - | - | - | - | - | - | G | L | A | E | R | Y | E | V | K | G | A | D | P | - | - | - | - | - | - | - | - | - | - | - | - | - | - | - | - | - | - | - | - | - | - | - | - | - | - | - | - | - | -152 |
| K1R5E8_CRAGI | - | - | - | - | - | - | - | - | - | - | - | - | - | - | - | - | - | - | - | G | I | P | R | L | Y | E | I | Q | D | K | D | P | - | - | - | - | - | - | - | - | - | - | - | - | - | - | - | - | - | - | - | - | - | - | - | - | - | - | - | - | - | -152 |
| XP_006811804 | - | - | - | - | - | - | - | - | - | - | - | - | - | - | - | - | - | - | - | G | L | T | E | R | C | D | L | K | E | P | R | A | - | - | - | - | - | - | - | - | - | - | - | - | - | - | - | - | - | - | - | - | - | - | - | - | - | - | - | - | - | -150 |
| C3YB23_BRAFL | - | - | - | - | - | - | - | - | - | - | - | - | - | - | - | - | - | - | - | G | M | A | E | S | Y | D | L | K | K | A | P | G | - | - | - | - | - | - | - | - | - | - | - | - | - | - | - | - | - | - | - | - | - | - | - | - | - | - | - | - | - | -157 |
| H2ZIF1_CIOSA | - | - | - | - | - | - | - | - | - | - | - | - | - | - | - | - | - | - | - | G | I | A | V | T | Y | D | L | G | S | K | T | P | S | - | - | - | - | - | - | - | - | - | - | - | - | - | - | - | - | - | - | - | - | - | - | - | - | - | - | - | - | -150 |
| W4YV93_STRPU | - | - | - | - | - | - | - | - | - | - | - | - | - | - | - | - | - | - | - | G | L | A | E | V | Y | D | L | T | T | E | N | P | D | - | - | - | - | - | - | - | - | - | - | - | - | - | - | - | - | - | - | - | - | - | - | - | - | - | - | - | - | -150 |
| CAN10_HUMAN | - | - | - | - | - | - | - | - | - | - | - | - | - | - | - | - | - | - | - | G | L | A | E | R | W | N | L | K | G | V | A | G | S | G | G | Q | Q | D | R | - | - | - | - | - | - | - | - | - | - | - | - | - | - | - | - | - | - | - | - | - | - | -157 |
| CAN10_MOUSE | - | - | - | - | - | - | - | - | - | - | - | - | - | - | - | - | - | - | - | S | L | A | E | R | W | S | L | K | D | V | T | K | A | S | G | Q | Q | D | R | - | - | - | - | - | - | - | - | - | - | - | - | - | - | - | - | - | - | - | - | - | - | -157 |
| XP_004943443 | - | - | - | - | - | - | - | - | - | - | - | - | - | - | - | - | - | - | - | G | I | A | E | R | W | A | L | K | D | P | A | R | N | T | G | K | E | K | S | - | - | - | - | - | - | - | - | - | - | - | - | - | - | - | - | - | - | - | - | - | - | -157 |
| F8W4K1_DANRE | - | - | - | - | - | - | - | - | - | - | - | - | - | - | - | - | - | - | - | A | V | A | V | R | W | S | I | K | D | P | E | S | P | Q | Q | P | N | D | S | - | - | - | - | - | - | - | - | - | - | - | - | - | - | - | - | - | - | - | - | - | - | -157 |
| CANC_DROME | - | - | - | - | - | - | - | - | - | - | - | - | - | - | - | - | - | - | - | G | V | V | R | Q | M | P | I | L | S | D | N | I | R | P | Q | T | L | K | E | - | - | - | - | - | - | - | - | - | - | - | - | - | - | - | - | - | - | - | - | - | - | -143 |
| C3ZKN9_BRAFL | - | - | - | - | - | - | - | - | - | - | - | - | - | - | - | - | - | - | - | G | I | S | E | V | Y | F | L | K | E | K | L | P | G | - | - | - | - | - | - | - | - | - | - | - | - | - | - | - | - | - | - | - | - | - | - | - | - | - | - | - | - | -149 |
| G4V623_SCHMA | - | - | - | - | - | - | - | - | - | - | - | - | - | - | - | - | - | - | - | G | L | T | E | S | Y | T | L | P | G | A | E | D | H | G | L | Q | P | P | A | - | - | - | - | - | - | - | - | - | - | - | - | - | - | - | - | - | - | - | - | - | - | -174 |
| R7VK39_CAPTE | - | - | - | - | - | - | - | - | - | - | - | - | - | - | - | - | - | - | - | G | I | P | E | S | F | D | L | R | N | E | E | - | - | - | - | K | L | P | D | - | - | - | - | - | - | - | - | - | - | - | - | - | - | - | - | - | - | - | - | - | - | -151 |
| G4V693_SCHMA | - | - | - | - | - | - | - | - | - | - | - | - | - | - | - | - | - | - | - | G | V | T | E | S | I | S | L | Q | N | L | Y | S | A | P | A | M | K | Q | V | - | - | - | - | - | - | - | - | - | - | - | - | - | - | - | - | - | - | - | - | - | - | -160 |
| R7UPU6_CAPTE | - | - | - | - | - | - | - | - | - | - | - | - | - | - | - | - | - | - | - | G | V | A | E | R | V | E | S | K | - | - | D | I | T | A | P | - | - | - | - | - | - | - | - | - | - | - | - | - | - | - | - | - | - | - | - | - | - | - | - | - | - | -155 |
| R7V817_CAPTE | - | - | - | - | - | - | - | - | - | - | - | - | - | - | - | - | - | - | - | G | V | A | E | R | V | E | S | K | - | - | D | I | T | A | P | - | - | - | - | - | - | - | - | - | - | - | - | - | - | - | - | - | - | - | - | - | - | - | - | - | - | -152 |
| R7UNL8_CAPTE | - | - | - | - | - | - | - | - | - | - | - | - | - | - | - | - | - | - | - | G | V | A | E | L | V | E | I | Q | - | - | D | T | D | A | A | - | - | - | - | - | - | - | - | - | - | - | - | - | - | - | - | - | - | - | - | - | - | - | - | - | - | -150 |
| K1PJ80_CRAGI | - | - | - | - | - | - | - | - | - | - | - | - | - | - | - | - | - | - | - | G | V | P | E | D | I | N | L | R | E | L | N | M | E | P | D | - | - | - | - | - | - | - | - | - | - | - | - | - | - | - | - | - | - | - | - | - | - | - | - | - | - | -155 |
| K1PCX8_CRAGI | - | - | - | - | - | - | - | - | - | - | - | - | - | - | - | - | - | - | - | G | V | P | E | D | F | D | L | R | K | L | D | M | E | P | D | - | - | - | - | - | - | - | - | - | - | - | - | - | - | - | - | - | - | - | - | - | - | - | - | - | - | -155 |
| CAND_DROME | - | - | - | - | - | - | - | - | - | - | - | - | - | - | - | - | - | - | - | A | P | C | E | S | I | P | L | Q | A | S | S | L | P | M | - | - | - | - | - | - | - | - | - | - | - | - | - | - | - | - | - | - | - | - | - | - | - | - | - | - | - | -152 |
| XP_002735765 | - | - | - | - | - | - | - | - | - | - | - | - | - | - | - | - | - | - | - | S | P | C | E | S | L | P | L | H | S | S | S | L | N | A | - | - | - | - | - | - | - | - | - | - | - | - | - | - | - | - | - | - | - | - | - | - | - | - | - | - | - | -154 |
| CAN15_HUMAN | - | - | - | - | - | - | - | - | - | - | - | - | - | - | - | - | - | - | - | A | P | C | E | S | L | A | L | Q | L | S | S | T | N | - | - | - | - | - | - | - | - | - | - | - | - | - | - | - | - | - | - | - | - | - | - | - | - | - | - | - | - | -153 |
| CAN15_MOUSE | - | - | - | - | - | - | - | - | - | - | - | - | - | - | - | - | - | - | - | A | P | C | E | S | L | A | L | Q | V | S | S | T | N | - | - | - | - | - | - | - | - | - | - | - | - | - | - | - | - | - | - | - | - | - | - | - | - | - | - | - | - | -153 |
| E1BTA8_CHICK | - | - | - | - | - | - | - | - | - | - | - | - | - | - | - | - | - | - | - | A | P | C | E | S | L | M | L | Q | V | S | S | T | N | - | - | - | - | - | - | - | - | - | - | - | - | - | - | - | - | - | - | - | - | - | - | - | - | - | - | - | - | -153 |
| E7FAX5_DANRE | - | - | - | - | - | - | - | - | - | - | - | - | - | - | - | - | - | - | - | A | P | C | E | S | L | M | L | Q | V | S | S | T | N | - | - | - | - | - | - | - | - | - | - | - | - | - | - | - | - | - | - | - | - | - | - | - | - | - | - | - | - | -153 |
| C3XWK8_BRAFL | - | - | - | - | - | - | - | - | - | - | - | - | - | - | - | - | - | - | - | A | P | C | E | S | V | Q | L | H | R | A | Q | T | N | N | - | - | - | - | - | - | - | - | - | - | - | - | - | - | - | - | - | - | - | - | - | - | - | - | - | - | - | -153 |
| W4YMU4_STRPU | - | - | - | - | - | - | - | - | - | - | - | - | - | - | - | - | - | - | - | A | P | C | E | S | V | Q | L | H | A | S | G | K | Y | I | - | - | - | - | - | - | - | - | - | - | - | - | - | - | - | - | - | - | - | - | - | - | - | - | - | - | - | -267 |
| K1PV74_CRAGI | - | - | - | - | - | - | - | - | - | - | - | - | - | - | - | - | - | - | - | A | P | C | D | N | I | E | L | Q | P | G | P | Q | K | - | - | - | - | - | - | - | - | - | - | - | - | - | - | - | - | - | - | - | - | - | - | - | - | - | - | - | - | -153 |
| R7TJF2_CAPTE | - | - | - | - | - | - | - | - | - | - | - | - | - | - | - | - | - | - | - | A | P | C | E | T | I | Q | L | Q | G | K | T | S | - | - | - | - | - | - | - | - | - | - | - | - | - | - | - | - | - | - | - | - | - | - | - | - | - | - | - | - | - | -152 |
| T2MI17_HYDVU | - | - | - | - | - | - | - | - | - | - | - | - | - | - | - | - | - | - | - | C | P | C | E | C | I | L | L | S | - | N | H | M | D | D | - | - | - | - | - | - | - | - | - | - | - | - | - | - | - | - | - | - | - | - | - | - | - | - | - | - | - | -150 |
| Q1RL55_CIOIN | - | - | - | - | - | - | - | - | - | - | - | - | - | - | - | - | - | - | - | Y | P | C | E | S | L | A | L | Q | Q | S | C | P | - | - | - | - | - | - | - | - | - | - | - | - | - | - | - | - | - | - | - | - | - | - | - | - | - | - | - | - | - | -139 |
| I1EA30_AMPQE | - | - | - | - | - | - | - | - | - | - | - | - | - | - | - | - | - | - | - | Y | P | C | D | V | I | H | L | R | A | H | H | A | D | - | - | - | - | - | - | - | - | - | - | - | - | - | - | - | - | - | - | - | - | - | - | - | - | - | - | - | - | -153 |
| G5EEZ6_CAEEL | - | - | - | - | - | - | - | - | - | - | - | - | - | - | - | - | - | - | - | A | S | C | V | N | Y | N | C | P | P | I | P | S | S | - | - | - | - | - | - | - | - | - | - | - | - | - | - | - | - | - | - | - | - | - | - | - | - | - | - | - | - | -153 |
| O44903_CAEEL | - | - | - | - | - | - | - | - | - | - | - | - | - | - | - | - | - | - | - | A | S | C | V | R | Y | K | C | P | Q | T | L | K | I | - | - | - | - | - | - | - | - | - | - | - | - | - | - | - | - | - | - | - | - | - | - | - | - | - | - | - | - | -153 |
| O02260_CAEEL | - | - | - | - | - | - | - | - | - | - | - | - | - | - | - | - | - | - | - | S | P | V | L | L | I | P | V | A | K | M | F | N | N | L | - | - | - | - | - | - | - | - | - | - | - | - | - | - | - | - | - | - | - | - | - | - | - | - | - | - | - | -151 |
| O02259_CAEEL | - | - | - | - | - | - | - | - | - | - | - | - | - | - | - | - | - | - | - | S | P | V | L | S | I | L | T | T | R | I | L | K | E | L | - | - | - | - | - | - | - | - | - | - | - | - | - | - | - | - | - | - | - | - | - | - | - | - | - | - | - | -128 |
| C1MTG3_MICPC | - | - | - | - | - | - | - | - | - | - | - | - | - | - | - | - | - | - | - | C | P | T | E | T | I | A | L | L | G | A | G | S | E | H | Y | G | G | E | T | A | S | H | T | T | P | F | A | W | C | T | P | F | L | K | D | F | S | R | R | H | S | -185 |
| C1E493_MICSR | - | - | - | - | - | - | - | - | - | - | - | - | - | - | - | - | - | - | - | C | P | T | E | T | I | R | L | D | A | S | G | - | - | - | - | - | - | - | - | - | - | - | - | - | - | - | - | - | - | - | - | - | - | - | - | - | - | - | - | - | - | -146 |
| Q012Z6_OSTTA | - | - | - | - | - | - | - | - | - | - | - | - | - | - | - | - | - | - | - | S | A | T | E | Q | I | D | F | H | A | N | D | - | - | - | - | - | - | - | - | - | - | - | - | - | - | - | - | - | - | - | - | - | - | - | - | - | - | - | - | - | - | -143 |
| L1IGB6_GUITH | - | - | - | - | - | - | - | - | - | - | - | - | - | - | - | - | - | - | - | A | P | T | L | T | I | N | F | D | S | P | D | - | - | - | - | - | - | - | - | - | - | - | - | - | - | - | - | - | - | - | - | - | - | - | - | - | - | - | - | - | - | -146 |
| B7FWX6_PHATC | - | - | - | - | - | - | - | - | - | - | - | - | - | - | - | - | - | - | - | A | P | T | E | T | I | I | F | S | D | L | I | - | - | - | - | - | - | - | - | - | - | - | - | - | - | - | - | - | - | - | - | - | - | - | - | - | - | - | - | - | - | -151 |
| N9TPR9_ENTHI | - | - | - | - | - | - | - | - | - | - | - | - | - | - | - | - | - | - | - | M | P | V | K | R | I | S | T | R | - | - | - | - | - | - | - | - | - | - | - | - | - | - | - | - | - | - | - | - | - | - | - | - | - | - | - | - | - | - | - | - | - | -137 |
| G0QS43_ICHMG | - | - | - | - | - | - | - | - | - | - | - | - | - | - | - | - | - | - | - | A | P | T | Q | T | I | W | L | D | G | - | - | - | - | - | - | - | - | - | - | - | - | - | - | - | - | - | - | - | - | - | - | - | - | - | - | - | - | - | - | - | - | -145 |
| D8LB20_ECTSI | - | P | G | Q | W | R | R | Y | E | F | L | F | K | P | S | A | D | N | K | R | D | A | S | S | V | G | T | E | E | - | - | - | - | - | - | - | - | - | - | - | - | - | - | - | - | - | - | - | - | - | - | - | - | - | - | - | - | - | - | - | - | -183 |
| Q9U0T9_LEIMA | - | - | - | - | - | - | - | - | - | - | - | - | - | - | - | - | - | - | - | F | P | A | M | R | I | D | E | M | V | S | K | A | T | V | - | - | - | - | - | - | - | - | - | - | - | - | - | - | - | - | - | - | - | - | - | - | - | - | - | - | - | -156 |
| Q4CXJ8_TRYCC | - | - | - | - | - | - | - | - | - | - | - | - | - | - | - | - | - | - | - | C | P | A | S | R | F | D | E | V | F | A | V | A | Q | E | - | - | - | - | - | - | - | - | - | - | - | - | - | - | - | - | - | - | - | - | - | - | - | - | - | - | - | -156 |
| F4I0A4_ARATH | - | - | - | - | - | - | - | - | - | - | - | - | - | - | - | - | - | - | - | G | A | G | E | E | I | D | L | R | S | A | Q | A | Q | I | D | - | - | - | - | - | - | - | - | - | - | - | - | - | - | - | - | - | - | - | - | - | - | - | - | - | - | -154 |
| Q8RUQ1_MAIZE | - | - | - | - | - | - | - | - | - | - | - | - | - | - | - | - | - | - | - | G | A | G | E | E | I | D | M | R | S | P | Q | A | Q | L | D | - | - | - | - | - | - | - | - | - | - | - | - | - | - | - | - | - | - | - | - | - | - | - | - | - | - | -154 |
| D7FQ17_ECTSI | - | - | - | - | - | - | - | - | - | - | - | - | - | - | - | - | - | - | - | A | Q | S | E | E | I | F | L | A | G | A | G | R | G | V | G | - | - | - | - | - | - | - | - | - | - | - | - | - | - | - | - | - | - | - | - | - | - | - | - | - | - | -171 |
| D0MZ44_PHYIT | - | - | - | - | - | - | - | - | - | - | - | - | - | - | - | - | - | - | - | C | E | C | D | E | V | Y | L | A | P | A | A | R | G | A | L | - | - | - | - | - | - | - | - | - | - | - | - | - | - | - | - | - | - | - | - | - | - | - | - | - | - | -172 |
| L1JBM0_GUITH | - | - | - | - | - | - | - | - | - | - | - | - | - | - | - | - | - | - | - | G | M | P | K | I | V | D | L | R | E | R | E | - | - | E | L | L | L | D | G | - | - | - | - | - | - | - | - | - | - | - | - | - | - | - | - | - | - | - | - | - | - | -127 |
| L1J739_GUITH | - | - | - | - | - | - | - | - | - | - | - | - | - | - | - | - | - | - | - | G | D | P | H | L | V | N | L | M | D | P | Q | T | R | D | E | C | E | N | G | - | - | - | - | - | - | - | - | - | - | - | - | - | - | - | - | - | - | - | - | - | - | -160 |
| L1J0U9_GUITH | - | - | - | - | - | - | - | - | - | - | - | - | - | - | - | - | - | - | - | G | A | A | Q | A | I | N | L | R | E | E | N | V | Q | D | Q | I | M | N | G | - | - | - | - | - | - | - | - | - | - | - | - | - | - | - | - | - | - | - | - | - | - | -153 |
| L1J1Q4_GUITH | - | - | - | - | - | - | - | - | - | - | - | - | - | - | - | - | - | - | - | G | V | T | E | T | I | D | T | K | G | R | E | G | L | E | R | I | K | T | G | - | - | - | - | - | - | - | - | - | - | - | - | - | - | - | - | - | - | - | - | - | - | -171 |
| D0ND88_PHYIT | - | - | - | - | - | - | - | - | - | - | - | - | - | - | - | - | - | - | - | G | S | A | H | R | V | D | L | Q | H | E | R | V | K | Q | L | Q | F | D | Q | A | T | A | - | - | - | - | - | - | - | - | - | - | - | - | - | - | - | - | - | - | - | -191 |
| D2VVS6_NAEGR | - | - | - | - | - | - | - | - | - | - | - | - | - | - | - | - | - | - | - | G | I | S | F | S | Y | D | I | D | P | K | K | I | E | E | - | - | - | - | - | - | - | - | - | - | - | - | - | - | - | - | - | - | - | - | - | - | - | - | - | - | - | -152 |
| D2V3V2_NAEGR | - | - | - | - | - | - | - | - | - | - | - | - | - | - | - | - | - | - | - | G | V | P | F | T | L | D | I | S | V | E | N | K | E | E | - | - | - | - | - | - | - | - | - | - | - | - | - | - | - | - | - | - | - | - | - | - | - | - | - | - | - | -153 |
| Q5B5J7_EMENI | - | - | - | - | - | - | - | - | - | - | - | - | - | - | - | - | - | - | - | G | V | T | S | D | L | L | T | S | N | I | L | N | - | - | - | - | - | - | - | - | - | - | - | - | - | - | - | - | - | - | - | - | - | - | - | - | - | - | - | - | - | -171 |
| E4ZY10_LEPMJ | - | - | - | - | - | - | - | - | - | - | - | - | - | - | - | - | - | - | - | G | V | T | S | E | V | F | A | T | D | I | L | D | - | - | - | - | - | - | - | - | - | - | - | - | - | - | - | - | - | - | - | - | - | - | - | - | - | - | - | - | - | -177 |
| A8PIE5_COPC7 | - | - | - | - | - | - | - | - | - | - | - | - | - | - | - | - | - | - | - | G | V | S | T | V | F | L | I | K | D | I | L | D | - | - | - | - | - | - | - | - | - | - | - | - | - | - | - | - | - | - | - | - | - | - | - | - | - | - | - | - | - | -172 |
| A8NBA6_COPC7 | - | - | - | - | - | - | - | - | - | - | - | - | - | - | - | - | - | - | - | G | I | A | K | V | I | E | V | K | D | I | L | N | - | - | - | - | - | - | - | - | - | - | - | - | - | - | - | - | - | - | - | - | - | - | - | - | - | - | - | - | - | -171 |
| A8NBA1_COPC7 | - | - | - | - | - | - | - | - | - | - | - | - | - | - | - | - | - | - | - | G | V | S | T | T | F | K | T | K | D | I | L | D | - | - | - | - | - | - | - | - | - | - | - | - | - | - | - | - | - | - | - | - | - | - | - | - | - | - | - | - | - | -175 |
| A8NBA2_COPC7 | - | - | - | - | - | - | - | - | - | - | - | - | - | - | - | - | - | - | - | G | V | S | T | T | F | Y | T | Q | D | I | L | D | - | - | - | - | - | - | - | - | - | - | - | - | - | - | - | - | - | - | - | - | - | - | - | - | - | - | - | - | - | -175 |
| A8NAE6_COPC7 | - | - | - | - | - | - | - | - | - | - | - | - | - | - | - | - | - | - | - | G | V | S | S | L | I | L | S | K | D | I | L | D | - | - | - | - | - | - | - | - | - | - | - | - | - | - | - | - | - | - | - | - | - | - | - | - | - | - | - | - | - | -193 |
| A8NAI9_COPC7 | - | - | - | - | - | - | - | - | - | - | - | - | - | - | - | - | - | - | - | G | V | N | E | S | W | K | I | M | D | I | L | D | - | - | - | - | - | - | - | - | - | - | - | - | - | - | - | - | - | - | - | - | - | - | - | - | - | - | - | - | - | -171 |
| G4TER7_PIRID | - | - | - | - | - | - | - | - | - | - | - | - | - | - | - | - | - | - | - | G | I | C | T | Q | I | P | L | T | D | V | L | D | - | - | - | - | - | - | - | - | - | - | - | - | - | - | - | - | - | - | - | - | - | - | - | - | - | - | - | - | - | -171 |
| G4TER6_PIRID | - | - | - | - | - | - | - | - | - | - | - | - | - | - | - | - | - | - | - | A | V | C | S | R | T | S | I | N | Y | I | L | D | - | - | - | - | - | - | - | - | - | - | - | - | - | - | - | - | - | - | - | - | - | - | - | - | - | - | - | - | - | -161 |
| G4TNA1_PIRID | - | - | - | - | - | - | - | - | - | - | - | - | - | - | - | - | - | - | - | G | V | S | Y | A | R | E | M | H | D | V | L | D | - | - | - | - | - | - | - | - | - | - | - | - | - | - | - | - | - | - | - | - | - | - | - | - | - | - | - | - | - | -171 |
| D0MWB9_PHYIT | - | - | - | - | - | - | - | - | - | - | - | - | - | - | - | - | - | - | - | M | C | A | E | Q | I | I | L | K | E | G | F | P | G | F | G | E | N | P | Y | S | P | A | K | P | L | Q | K | H | - | - | - | - | - | - | - | - | - | - | - | - | - | -161 |
| L1I9H5_GUITH | - | - | - | - | - | - | - | - | - | - | - | - | - | - | - | - | - | - | - | G | T | V | Q | S | I | S | L | D | E | P | D | K | A | W | S | - | - | - | - | - | - | - | - | - | - | - | - | - | - | - | - | - | - | - | - | - | - | - | - | - | - | -163 |
| Q7N497_PHOLL | - | - | - | - | - | - | - | - | - | - | - | - | - | - | - | - | - | - | I | E | S | T | A | N | T | S | I | N | Q | G | N | Q | D | R | - | - | - | - | - | - | - | - | - | - | - | - | - | - | - | - | - | - | - | - | - | - | - | - | - | - | - | -132 |
| ZP_11152369 | - | - | - | - | - | - | - | - | - | - | - | - | - | - | - | - | - | - | I | E | S | S | S | N | L | N | I | N | E | K | N | K | N | C | - | - | - | - | - | - | - | - | - | - | - | - | - | - | - | - | - | - | - | - | - | - | - | - | - | - | - | -128 |
| A2FEL7_TRIVA | - | - | - | - | - | - | - | - | - | - | - | - | - | - | - | - | - | - | - | G | F | P | I | N | I | D | L | T | D | L | D | V | R | E | S | I | - | - | - | - | - | - | - | - | - | - | - | - | - | - | - | - | - | - | - | - | - | - | - | - | - | -150 |
| A2D9A8_TRIVA | - | - | - | - | - | - | - | - | - | - | - | - | - | - | - | - | - | - | - | G | Y | P | M | V | I | E | L | N | T | L | D | V | R | E | K | F | - | - | - | - | - | - | - | - | - | - | - | - | - | - | - | - | - | - | - | - | - | - | - | - | - | -151 |
| A2FHB3_TRIVA | - | - | - | - | - | - | - | - | - | - | - | - | - | - | - | - | - | - | - | G | W | P | E | I | F | N | L | D | S | L | E | M | K | E | R | I | - | - | - | - | - | - | - | - | - | - | - | - | - | - | - | - | - | - | - | - | - | - | - | - | - | -152 |
| A2DTE8_TRIVA | - | - | - | - | - | - | - | - | - | - | - | - | - | - | - | - | - | - | - | G | Y | S | N | L | I | D | L | D | S | D | N | G | I | E | M | R | - | - | - | - | - | - | - | - | - | - | - | - | - | - | - | - | - | - | - | - | - | - | - | - | - | -149 |
| A2E4X2_TRIVA | - | - | - | - | - | - | - | - | - | - | - | - | - | - | - | - | - | - | - | Y | S | P | T | S | I | K | L | Q | D | L | K | E | T | P | S | E | - | - | - | - | - | - | - | - | - | - | - | - | - | - | - | - | - | - | - | - | - | - | - | - | - | -154 |
| A2E1X1_TRIVA | - | - | - | - | - | - | - | - | - | - | - | - | - | - | - | - | - | - | - | I | Y | N | Q | F | Q | K | L | S | E | V | K | N | I | I | P | - | - | - | - | - | - | - | - | - | - | - | - | - | - | - | - | - | - | - | - | - | - | - | - | - | - | -137 |
| TPR_PORGI | - | - | - | - | - | - | - | - | - | - | - | - | - | - | - | - | - | - | - | S | W | F | T | S | S | G | K | L | M | - | - | - | - | - | - | - | - | - | - | - | - | - | - | - | - | - | - | - | - | - | - | - | - | - | - | - | - | - | - | - | - | -170 |
| D5MF42_9BACT | - | - | - | - | - | - | - | - | - | - | - | - | - | - | - | - | - | - | - | A | G | V | T | K | T | - | - | - | - | - | - | - | - | - | - | - | - | - | - | - | - | - | - | - | - | - | - | - | - | - | - | - | - | - | - | - | - | - | - | - | - | -126 |
| R9H254_BACT4 | - | - | - | - | - | - | - | - | - | - | - | - | - | - | - | - | - | - | - | G | E | S | V | G | F | G | A | N | - | - | - | - | - | - | - | - | - | - | - | - | - | - | - | - | - | - | - | - | - | - | - | - | - | - | - | - | - | - | - | - | - | -126 |
| ZP_04847694 | - | - | - | - | - | - | - | - | - | - | - | - | - | - | - | - | - | - | - | G | E | S | V | G | F | G | A | N | - | - | - | - | - | - | - | - | - | - | - | - | - | - | - | - | - | - | - | - | - | - | - | - | - | - | - | - | - | - | - | - | - | -126 |
| D7IBA4_9BACE | - | - | - | - | - | - | - | - | - | - | - | - | - | - | - | - | - | - | - | G | E | S | V | G | F | G | A | N | - | - | - | - | - | - | - | - | - | - | - | - | - | - | - | - | - | - | - | - | - | - | - | - | - | - | - | - | - | - | - | - | - | -124 |
| ZP_09858577 | - | - | - | - | - | - | - | - | - | - | - | - | - | - | - | - | - | - | - | G | E | S | V | G | F | G | A | N | - | - | - | - | - | - | - | - | - | - | - | - | - | - | - | - | - | - | - | - | - | - | - | - | - | - | - | - | - | - | - | - | - | -124 |
| E5C759_9BACE | - | - | - | - | - | - | - | - | - | - | - | - | - | - | - | - | - | - | - | G | E | S | V | G | F | G | A | G | - | - | - | - | - | - | - | - | - | - | - | - | - | - | - | - | - | - | - | - | - | - | - | - | - | - | - | - | - | - | - | - | - | -126 |
| A7LTS0_BACO1 | - | - | - | - | - | - | - | - | - | - | - | - | - | - | - | - | - | - | - | G | S | S | F | A | F | A | P | E | - | - | - | - | - | - | - | - | - | - | - | - | - | - | - | - | - | - | - | - | - | - | - | - | - | - | - | - | - | - | - | - | - | -124 |
| Q8YQK8_NOSS1 | - | - | - | - | - | - | - | - | - | - | - | - | - | - | - | - | - | - | - | A | S | T | Y | V | S | S | - | - | - | - | - | - | - | - | - | - | - | - | - | - | - | - | - | - | - | - | - | - | - | - | - | - | - | - | - | - | - | - | - | - | - | -138 |
| Q3MBX8_ANAVT | - | - | - | - | - | - | - | - | - | - | - | - | - | - | - | - | - | - | - | D | F | T | P | A | D | P | - | - | - | - | - | - | - | - | - | - | - | - | - | - | - | - | - | - | - | - | - | - | - | - | - | - | - | - | - | - | - | - | - | - | - | -143 |
| Q8YZT8_NOSS1 | - | - | - | - | - | - | - | - | - | - | - | - | - | - | - | - | - | - | - | K | T | N | N | Y | S | K | - | - | - | - | - | - | - | - | - | - | - | - | - | - | - | - | - | - | - | - | - | - | - | - | - | - | - | - | - | - | - | - | - | - | - | -131 |
| K9UIG2_9CHRO | - | - | - | - | - | - | - | - | - | - | - | - | - | - | - | - | - | - | - | A | A | R | N | Y | Y | T | G | - | - | - | - | - | - | - | - | - | - | - | - | - | - | - | - | - | - | - | - | - | - | - | - | - | - | - | - | - | - | - | - | - | - | -138 |
| A4YRP8_BRASO | - | - | - | - | - | - | - | - | - | - | - | - | - | - | - | - | - | - | - | S | Q | I | T | D | F | R | A | M | G | G | S | W | G | K | V | V | Y | N | S | S | F | G | V | T | S | Y | - | - | - | - | - | - | - | - | - | - | - | - | - | - | - | -152 |
| H0TAL7_9BRAD | - | - | - | - | - | - | - | - | - | - | - | - | - | - | - | - | - | - | - | S | Q | I | T | D | F | R | A | M | G | G | S | W | G | K | V | V | Y | N | S | S | F | G | V | T | S | Y | - | - | - | - | - | - | - | - | - | - | - | - | - | - | - | -156 |
| H0SNL4_9BRAD | - | - | - | - | - | - | - | - | - | - | - | - | - | - | - | - | - | - | - | T | Q | I | T | D | F | R | A | M | G | G | S | W | G | K | V | V | Y | N | S | S | F | G | V | T | N | Y | - | - | - | - | - | - | - | - | - | - | - | - | - | - | - | -158 |
| H0S4P6_9BRAD | - | - | - | - | - | - | - | - | - | - | - | - | - | - | - | - | - | - | - | T | Q | I | T | D | F | R | A | M | G | G | S | W | G | K | V | V | Y | N | S | S | F | G | V | T | N | Y | - | - | - | - | - | - | - | - | - | - | - | - | - | - | - | -158 |
| A5EMK2_BRASB | - | - | - | - | - | - | - | - | - | - | - | - | - | - | - | - | - | - | - | T | Q | I | T | D | F | R | A | M | G | G | S | W | G | K | V | V | Y | N | S | S | F | G | V | T | G | Y | - | - | - | - | - | - | - | - | - | - | - | - | - | - | - | -152 |
| M4ZBJ4_9BRAD | - | - | - | - | - | - | - | - | - | - | - | - | - | - | - | - | - | - | - | S | Q | I | T | D | F | R | A | M | G | G | S | W | G | K | V | V | Y | N | S | S | F | G | V | N | A | Y | - | - | - | - | - | - | - | - | - | - | - | - | - | - | - | -158 |
| Q07SP7_RHOP5 | - | - | - | - | - | - | - | - | - | - | - | - | - | - | - | - | - | - | - | S | T | I | T | D | Y | F | A | S | G | G | S | W | G | K | V | T | Y | N | S | S | L | S | M | T | G | Y | - | - | - | - | - | - | - | - | - | - | - | - | - | - | - | -145 |
| B0JUQ7_MICAN | - | - | - | - | - | - | - | - | - | - | - | - | - | - | - | - | - | - | - | N | T | V | R | D | Y | T | L | D | N | - | - | - | - | - | - | - | - | - | - | - | - | - | - | - | - | - | - | - | - | - | - | - | - | - | - | - | - | - | - | - | - | -147 |
| K9WJ87_9CYAN | - | - | - | - | - | - | - | - | - | - | - | - | - | - | - | - | - | - | - | N | N | T | G | Y | K | D | L | E | A | - | - | - | - | - | - | - | - | - | - | - | - | - | - | - | - | - | - | - | - | - | - | - | - | - | - | - | - | - | - | - | - | -141 |
| G6FUE0_9CYAN | - | - | - | - | - | - | - | - | - | - | - | - | - | - | - | - | - | - | - | N | T | S | L | A | N | L | L | N | - | - | - | - | - | - | - | - | - | - | - | - | - | - | - | - | - | - | - | - | - | - | - | - | - | - | - | - | - | - | - | - | - | -141 |
| K9PHG5_9CYAN | - | - | - | - | - | - | - | - | - | - | - | - | - | - | - | - | - | - | - | N | S | G | W | S | D | K | F | D | - | - | - | - | - | - | - | - | - | - | - | - | - | - | - | - | - | - | - | - | - | - | - | - | - | - | - | - | - | - | - | - | - | -143 |
| Q8YXX3_NOSS1 | - | - | - | - | - | - | - | - | - | - | - | - | - | - | - | - | - | - | - | S | S | T | Q | Y | D | N | L | D | S | - | - | - | - | - | - | - | - | - | - | - | - | - | - | - | - | - | - | - | - | - | - | - | - | - | - | - | - | - | - | - | - | -156 |
| K9TN70_9CYAN | - | - | - | - | - | - | - | - | - | - | - | - | - | - | - | - | - | - | - | E | G | - | K | Y | H | D | F | S | H | - | - | - | - | - | - | - | - | - | - | - | - | - | - | - | - | - | - | - | - | - | - | - | - | - | - | - | - | - | - | - | - | -156 |
| Q05QW3_9SYNE | - | - | - | - | - | - | - | - | - | - | - | - | - | - | - | - | - | - | - | N | T | T | Q | E | R | L | V | E | S | T | W | W | N | P | F | S | W | - | - | - | - | - | - | - | - | - | - | - | - | - | - | - | - | - | - | - | - | - | - | - | - | -162 |
| Q05QW2_9SYNE | - | - | - | - | - | - | - | - | - | - | - | - | - | - | - | - | - | - | - | N | S | S | W | G | N | A | S | A | S | G | V | G | - | - | - | - | - | - | - | - | - | - | - | - | - | - | - | - | - | - | - | - | - | - | - | - | - | - | - | - | - | -159 |
| Q05QW4_9SYNE | - | - | - | - | - | - | - | - | - | - | - | - | - | - | - | - | - | - | - | S | A | T | S | E | A | V | N | L | T | G | I | N | - | - | - | - | - | - | - | - | - | - | - | - | - | - | - | - | - | - | - | - | - | - | - | - | - | - | - | - | - | -153 |
| E0UGX6_CYAP2 | - | - | - | - | - | - | - | - | - | - | - | - | - | - | - | - | - | - | - | S | T | T | S | Q | S | V | S | S | - | - | - | - | - | - | - | - | - | - | - | - | - | - | - | - | - | - | - | - | - | - | - | - | - | - | - | - | - | - | - | - | - | -144 |
| K7WKF4_9NOST | - | - | - | - | - | - | - | - | - | - | - | - | - | - | - | - | - | - | - | S | A | T | S | Q | S | V | G | N | - | - | - | - | - | - | - | - | - | - | - | - | - | - | - | - | - | - | - | - | - | - | - | - | - | - | - | - | - | - | - | - | - | -143 |
| E0UGY5_CYAP2 | - | - | - | - | - | - | - | - | - | - | - | - | - | - | - | - | - | - | - | T | T | T | S | T | D | V | N | S | - | - | - | - | - | - | - | - | - | - | - | - | - | - | - | - | - | - | - | - | - | - | - | - | - | - | - | - | - | - | - | - | - | -146 |
| A8LCE5_FRASN | - | - | - | - | - | - | - | - | - | - | - | - | - | - | - | - | - | - | - | A | A | V | R | R | S | P | S | - | - | - | - | - | - | - | - | - | - | - | - | - | - | - | - | - | - | - | - | - | - | - | - | - | - | - | - | - | - | - | - | - | - | -137 |
| D3CT47_9ACTO | - | - | - | - | - | - | - | - | - | - | - | - | - | - | - | - | - | - | - | E | P | D | R | V | D | P | D | - | - | - | - | - | - | - | - | - | - | - | - | - | - | - | - | - | - | - | - | - | - | - | - | - | - | - | - | - | - | - | - | - | - | -141 |
| Q2J4J6_FRASC | - | - | - | - | - | - | - | - | - | - | - | - | - | - | - | - | - | - | - | S | A | H | V | F | A | A | H | - | - | - | - | - | - | - | - | - | - | - | - | - | - | - | - | - | - | - | - | - | - | - | - | - | - | - | - | - | - | - | - | - | - | -162 |
| Q0RAZ2_FRAAA | - | - | - | - | - | - | - | - | - | - | - | - | - | - | - | - | - | - | - | P | A | S | R | R | A | A | R | - | - | - | - | - | - | - | - | - | - | - | - | - | - | - | - | - | - | - | - | - | - | - | - | - | - | - | - | - | - | - | - | - | - | -149 |
| E3J3F6_FRASU | - | - | - | - | - | - | - | - | - | - | - | - | - | - | - | - | - | - | - | P | H | R | T | L | R | P | G | - | - | - | - | - | - | - | - | - | - | - | - | - | - | - | - | - | - | - | - | - | - | - | - | - | - | - | - | - | - | - | - | - | - | -143 |
| ZP_09168240 | - | - | - | - | - | - | - | - | - | - | - | - | - | - | - | - | - | - | - | P | H | H | A | V | P | P | G | - | - | - | - | - | - | - | - | - | - | - | - | - | - | - | - | - | - | - | - | - | - | - | - | - | - | - | - | - | - | - | - | - | - | -153 |
| C7MDV9_BRAFD | - | - | - | - | - | - | - | - | - | - | - | - | - | - | - | - | - | - | - | R | V | R | T | L | L | R | R | - | - | - | - | - | - | - | - | - | - | - | - | - | - | - | - | - | - | - | - | - | - | - | - | - | - | - | - | - | - | - | - | - | - | -115 |
| ZP_10094989 | - | - | - | - | - | - | - | - | - | - | - | - | - | - | - | - | - | - | - | R | V | R | T | N | L | R | L | - | - | - | - | - | - | - | - | - | - | - | - | - | - | - | - | - | - | - | - | - | - | - | - | - | - | - | - | - | - | - | - | - | - | -115 |
| A4X268_SALTO | - | - | - | - | - | - | - | - | - | - | - | - | - | - | - | - | - | - | - | T | G | L | P | S | R | A | A | S | F | D | Q | T | - | - | - | - | - | - | - | - | - | - | - | - | - | - | - | - | - | - | - | - | - | - | - | - | - | - | - | - | - | -165 |
| WP_043162213 | - | - | - | - | - | - | - | - | - | - | - | - | - | - | - | - | - | - | - | T | G | L | P | S | R | A | A | S | F | D | Q | T | - | - | - | - | - | - | - | - | - | - | - | - | - | - | - | - | - | - | - | - | - | - | - | - | - | - | - | - | - | -165 |
| Q0BRV8_GRABC | - | - | - | - | - | - | - | - | - | - | - | - | - | - | - | - | - | - | - | T | G | Q | T | A | Q | A | Y | - | - | - | - | - | - | - | - | - | - | - | - | - | - | - | - | - | - | - | - | - | - | - | - | - | - | - | - | - | - | - | - | - | - | -141 |
| A9B2P0_HERA2 | - | - | - | - | - | - | - | - | - | - | - | - | - | - | - | - | - | - | - | D | S | S | H | T | D | V | E | - | - | - | - | - | - | - | - | - | - | - | - | - | - | - | - | - | - | - | - | - | - | - | - | - | - | - | - | - | - | - | - | - | - | -133 |
| A9B3U8_HERA2 | - | - | - | - | - | - | - | - | - | - | - | - | - | - | - | - | - | - | - | D | S | V | H | T | N | V | A | - | - | - | - | - | - | - | - | - | - | - | - | - | - | - | - | - | - | - | - | - | - | - | - | - | - | - | - | - | - | - | - | - | - | -135 |
| A9B2A3_HERA2 | - | - | - | - | - | - | - | - | - | - | - | - | - | - | - | - | - | - | - | R | S | A | I | N | E | P | Q | - | - | - | - | - | - | - | - | - | - | - | - | - | - | - | - | - | - | - | - | - | - | - | - | - | - | - | - | - | - | - | - | - | - | -153 |
| D6ZC85_SEGRD | - | - | - | - | - | - | - | - | - | - | - | - | - | - | - | - | - | - | - | T | G | S | Y | V | P | A | A | - | - | - | - | - | - | - | - | - | - | - | - | - | - | - | - | - | - | - | - | - | - | - | - | - | - | - | - | - | - | - | - | - | - | -179 |
| E5XTT9_9ACTO | - | - | - | - | - | - | - | - | - | - | - | - | - | - | - | - | - | - | - | T | G | T | Y | T | P | A | A | - | - | - | - | - | - | - | - | - | - | - | - | - | - | - | - | - | - | - | - | - | - | - | - | - | - | - | - | - | - | - | - | - | - | -179 |
| E5XTU0_9ACTO | - | - | - | - | - | - | - | - | - | - | - | - | - | - | - | - | - | - | - | T | G | T | Y | T | A | P | S | - | - | - | - | - | - | - | - | - | - | - | - | - | - | - | - | - | - | - | - | - | - | - | - | - | - | - | - | - | - | - | - | - | - | -183 |
| D6B0I6_9ACTO | - | - | - | - | - | - | - | - | - | - | - | - | - | - | - | - | - | - | - | E | G | E | D | L | E | T | G | G | F | L | G | I | - | - | - | - | - | - | - | - | - | - | - | - | - | - | - | - | - | - | - | - | - | - | - | - | - | - | - | - | - | -141 |
| D9X719_STRVR | - | - | - | - | - | - | - | - | - | - | - | - | - | - | - | - | - | - | - | E | G | E | D | L | T | T | G | G | F | L | G | I | - | - | - | - | - | - | - | - | - | - | - | - | - | - | - | - | - | - | - | - | - | - | - | - | - | - | - | - | - | -141 |
| H6L010_SAPGL | - | - | - | - | - | - | - | - | - | - | - | - | - | - | - | - | - | - | - | D | G | D | V | Y | K | T | K | S | - | - | - | - | - | - | - | - | - | - | - | - | - | - | - | - | - | - | - | - | - | - | - | - | - | - | - | - | - | - | - | - | - | -127 |
| K1ZP83_9BACT | - | - | - | - | - | - | - | - | - | - | - | - | - | - | - | - | - | - | - | T | S | K | T | T | T | T | S | S | - | - | - | - | - | - | - | - | - | - | - | - | - | - | - | - | - | - | - | - | - | - | - | - | - | - | - | - | - | - | - | - | - | -148 |
| ADGB_HUMAN | - | - | - | - | - | - | - | - | - | - | - | - | - | - | - | - | - | - | - | W | L | P | E | V | I | S | L | H | P | G | Y | M | D | - | - | - | - | - | - | - | - | - | - | - | - | - | - | - | - | - | - | - | - | - | - | - | - | - | - | - | - | -186 |
| J3QMP3_MOUSE | - | - | - | - | - | - | - | - | - | - | - | - | - | - | - | - | - | - | - | W | L | P | E | V | I | P | L | H | P | A | Y | V | D | - | - | - | - | - | - | - | - | - | - | - | - | - | - | - | - | - | - | - | - | - | - | - | - | - | - | - | - | -186 |
| H2XR46_CIOIN | - | - | - | - | - | - | - | - | - | - | - | - | - | - | - | - | - | - | - | W | I | P | E | V | I | P | I | K | Q | S | P | S | E | - | - | - | - | - | - | - | - | - | - | - | - | - | - | - | - | - | - | - | - | - | - | - | - | - | - | - | - | -184 |
| CAN7_MOUSE | - | - | - | - | - | - | - | - | - | - | - | - | - | - | - | - | - | - | - | W | I | P | E | R | I | A | M | H | S | D | S | Q | T | - | - | - | - | - | - | - | - | - | - | - | - | - | - | - | - | - | - | - | - | - | - | - | - | - | - | - | - | -156 |
| CAN7_HUMAN | - | - | - | - | - | - | - | - | - | - | - | - | - | - | - | - | - | - | - | W | I | P | E | R | I | A | M | H | S | D | S | Q | T | - | - | - | - | - | - | - | - | - | - | - | - | - | - | - | - | - | - | - | - | - | - | - | - | - | - | - | - | -156 |
| E1BY84_CHICK | - | - | - | - | - | - | - | - | - | - | - | - | - | - | - | - | - | - | - | W | I | P | E | R | I | A | M | H | S | D | N | Q | A | - | - | - | - | - | - | - | - | - | - | - | - | - | - | - | - | - | - | - | - | - | - | - | - | - | - | - | - | -156 |
| H9GX91_DANRE | - | - | - | - | - | - | - | - | - | - | - | - | - | - | - | - | - | - | - | W | I | P | E | R | I | A | M | H | S | D | N | Q | S | - | - | - | - | - | - | - | - | - | - | - | - | - | - | - | - | - | - | - | - | - | - | - | - | - | - | - | - | -156 |
| F6SGI6_CIOIN | - | - | - | - | - | - | - | - | - | - | - | - | - | - | - | - | - | - | - | W | I | P | E | R | Q | S | L | R | D | K | N | - | - | - | - | - | - | - | - | - | - | - | - | - | - | - | - | - | - | - | - | - | - | - | - | - | - | - | - | - | - | -154 |
| W4Y195_STRPU | - | - | - | - | - | - | - | - | - | - | - | - | - | - | - | - | - | - | - | W | I | P | E | R | V | A | I | R | Q | N | T | P | G | - | - | - | - | - | - | - | - | - | - | - | - | - | - | - | - | - | - | - | - | - | - | - | - | - | - | - | - | -130 |
| R7V270_CAPTE | - | - | - | - | - | - | - | - | - | - | - | - | - | - | - | - | - | - | - | W | I | P | E | R | V | D | L | Q | D | P | E | E | - | - | - | - | - | - | - | - | - | - | - | - | - | - | - | - | - | - | - | - | - | - | - | - | - | - | - | - | - | -155 |
| G4LXK3_SCHMA | - | - | - | - | - | - | - | - | - | - | - | - | - | - | - | - | - | - | - | W | I | P | E | R | I | S | I | R | S | S | S | S | S | - | - | - | - | - | - | - | - | - | - | - | - | - | - | - | - | - | - | - | - | - | - | - | - | - | - | - | - | -156 |
| T2M2K0_HYDVU | - | - | - | - | - | - | - | - | - | - | - | - | - | - | - | - | - | - | - | W | I | P | E | R | A | S | L | K | S | K | D | I | S | - | - | - | - | - | - | - | - | - | - | - | - | - | - | - | - | - | - | - | - | - | - | - | - | - | - | - | - | -156 |
| B3RQK1_TRIAD | - | - | - | - | - | - | - | - | - | - | - | - | - | - | - | - | - | - | - | W | I | P | E | R | I | A | T | K | N | E | E | S | - | - | - | - | - | - | - | - | - | - | - | - | - | - | - | - | - | - | - | - | - | - | - | - | - | - | - | - | - | -155 |
| I1CS59_RHIO9 | - | - | - | - | - | - | - | - | - | - | - | - | - | - | - | - | - | - | - | W | I | P | E | H | I | F | I | H | D | K | Y | - | - | - | - | - | - | - | - | - | - | - | - | - | - | - | - | - | - | - | - | - | - | - | - | - | - | - | - | - | - | -151 |
| I1GER3_AMPQE | - | - | - | - | - | - | - | - | - | - | - | - | - | - | - | - | - | - | - | W | I | P | E | R | I | D | I | K | E | S | N | - | - | - | - | - | - | - | - | - | - | - | - | - | - | - | - | - | - | - | - | - | - | - | - | - | - | - | - | - | - | -154 |
| Q22143_CAEEL | - | - | - | - | - | - | - | - | - | - | - | - | - | - | - | - | - | - | - | W | I | P | E | R | I | E | L | S | D | T | S | K | - | - | - | - | - | - | - | - | - | - | - | - | - | - | - | - | - | - | - | - | - | - | - | - | - | - | - | - | - | -155 |
| L1JZN7_GUITH | - | - | - | - | - | - | - | - | - | - | - | - | - | - | - | - | - | - | - | W | I | P | E | T | F | H | L | K | E | D | S | K | S | R | - | - | - | - | - | - | - | - | - | - | - | - | - | - | - | - | - | - | - | - | - | - | - | - | - | - | - | -158 |
| D0NT34_PHYIT | - | - | - | - | - | - | - | - | - | - | - | - | - | - | - | - | - | - | - | W | V | P | E | R | V | P | V | S | E | L | I | D | A | P | - | - | - | - | - | - | - | - | - | - | - | - | - | - | - | - | - | - | - | - | - | - | - | - | - | - | - | -166 |
| A0A064B913_ASPOZ | - | - | - | - | - | - | - | - | - | - | - | - | - | - | - | - | - | - | - | W | I | P | E | Q | V | F | L | H | N | D | D | V | T | - | - | - | - | - | - | - | - | - | - | - | - | - | - | - | - | - | - | - | - | - | - | - | - | - | - | - | - | -164 |
| A8N4X4_COPC7 | - | - | - | - | - | - | - | - | - | - | - | - | - | - | - | - | - | - | - | W | I | P | E | H | V | E | I | H | R | S | D | F | E | - | - | - | - | - | - | - | - | - | - | - | - | - | - | - | - | - | - | - | - | - | - | - | - | - | - | - | - | -180 |
| C0H5K1_PLAF7 | - | - | - | - | - | - | - | - | - | - | - | - | - | - | - | - | - | - | - | W | I | P | V | T | I | S | L | K | S | K | I | S | S | S | P | P | S | F | D | K | H | S | T | H | S | L | I | R | D | E | R | I | D | E | L | V | Y | E | T | N | E | -192 |
| K1REZ8_CRAGI | - | - | - | - | - | - | - | - | - | - | - | - | - | - | - | - | - | - | - | G | I | Y | D | A | I | N | L | E | K | T | R | L | K | P | - | - | - | - | - | - | - | - | - | - | - | - | - | - | - | - | - | - | - | - | - | - | - | - | - | - | - | -145 |
| F4NTM9_BATDJ | - | - | - | - | - | - | - | - | - | - | - | - | - | - | - | - | - | - | - | N | I | P | E | Q | I | D | L | S | V | F | S | Q | D | A | L | S | E | D | S | - | - | - | - | - | - | - | - | - | - | - | - | - | - | - | - | - | - | - | - | - | - | -167 |
| Q6F6K5_CAEEL | - | - | - | - | - | - | - | - | - | - | - | - | - | - | - | - | - | - | - | S | F | T | S | T | Y | Y | L | N | K | L | S | S | D | N | - | - | - | - | - | - | - | - | - | - | - | - | - | - | - | - | - | - | - | - | - | - | - | - | - | - | - | -126 |
| A8HZK8_CHLRE | - | - | - | - | - | - | - | - | - | - | - | - | - | - | - | - | - | - | - | G | V | G | F | K | V | K | T | D | T | P | E | G | Q | A | A | A | A | D | G | - | - | - | - | - | - | - | - | - | - | - | - | - | - | - | - | - | - | - | - | - | - | -132 |
| A8J6T6_CHLRE | - | - | - | - | - | - | - | - | - | - | - | - | - | - | - | - | - | - | - | Q | K | W | R | R | L | E | L | Q | Y | I | E | K | E | P | G | K | Y | R | A | E | Y | A | A | D | D | V | S | - | - | - | - | - | - | - | - | - | - | - | - | - | - | -184 |
| Z9JR51_9MICO | - | - | - | - | - | - | - | - | - | - | - | - | - | - | - | - | - | - | - | G | S | T | E | T | S | T | L | G | G | Q | P | S | - | - | - | - | - | - | - | - | - | - | - | - | - | - | - | - | - | - | - | - | - | - | - | - | - | - | - | - | - | -144 |
| F7ZZH9_CELGA | - | - | - | - | - | - | - | - | - | - | - | - | - | - | - | - | - | - | - | H | P | V | D | V | I | E | N | G | N | Y | A | G | - | - | - | - | - | - | - | - | - | - | - | - | - | - | - | - | - | - | - | - | - | - | - | - | - | - | - | - | - | -126 |
| Q7NDU2_GLOVI | - | - | - | - | - | - | - | - | - | - | - | - | - | - | - | - | - | - | - | N | D | S | T | A | I | T | L | D | G | E | H | R | - | - | - | - | - | - | - | - | - | - | - | - | - | - | - | - | - | - | - | - | - | - | - | - | - | - | - | - | - | -115 |
| I6AT95_9BACT | - | - | - | - | - | - | - | - | - | - | - | - | - | - | - | - | - | - | - | H | P | I | E | R | F | S | C | K | F | A | K | E | E | K | L | S | E | K | E | F | G | - | - | - | - | - | - | - | - | - | - | - | - | - | - | - | - | - | - | - | - | -139 |
| C7MGJ8_BRAFD | - | - | - | - | - | - | - | - | - | - | - | - | - | - | - | - | - | - | - | G | S | V | D | S | V | D | V | Q | D | L | A | A | T | - | - | - | - | - | - | - | - | - | - | - | - | - | - | - | - | - | - | - | - | - | - | - | - | - | - | - | - | -142 |
| D0WP73_9ACTO | - | - | - | - | - | - | - | - | - | - | - | - | - | - | - | - | - | - | - | N | P | A | S | D | V | S | G | Q | G | G | Y | S | S | - | - | - | - | - | - | - | - | - | - | - | - | - | - | - | - | - | - | - | - | - | - | - | - | - | - | - | - | -141 |
| B0C8L1_ACAM1 | - | - | - | - | - | - | - | - | - | - | - | - | - | - | - | - | - | - | - | G | G | T | A | S | L | T | F | Q | L | L | L | G | - | - | - | - | - | - | - | - | - | - | - | - | - | - | - | - | - | - | - | - | - | - | - | - | - | - | - | - | - | -132 |
| B3E5A8_GEOLS | - | - | - | - | - | - | - | - | - | - | - | - | - | - | - | - | - | - | - | R | E | V | K | M | Y | H | L | G | A | R | V | Q | R | - | - | - | - | - | - | - | - | - | - | - | - | - | - | - | - | - | - | - | - | - | - | - | - | - | - | - | - | -135 |
| D4YQ94_9MICO | - | - | - | - | - | - | - | - | - | - | - | - | - | - | - | - | - | - | - | T | W | C | P | A | R | P | V | P | R | D | L | G | - | - | - | - | - | - | - | - | - | - | - | - | - | - | - | - | - | - | - | - | - | - | - | - | - | - | - | - | - | -125 |
| PAPA_CARPA | - | - | - | - | - | - | - | - | - | - | - | - | - | - | - | - | - | - | - | - | V | R | Q | V | Q | P | Y | N | E | G | - | - | - | - | - | - | - | - | - | - | - | - | - | - | - | - | - | - | - | - | - | - | - | - | - | - | - | - | - | - | - | -119 |
| RIM13_YEAST | - | - | - | - | - | - | - | - | - | - | - | - | - | - | - | - | - | - | - | F | L | P | E | I | T | Q | V | N | S | - | - | - | - | - | - | - | - | - | - | - | - | - | - | - | - | - | - | - | - | - | - | - | - | - | - | - | - | - | - | - | - | -154 |

| I |  |  |  |  |  |  |  |  |  |  |  |  |  |  |  |  |  |  |  |  |  |  |  |  |  |  |  |  |  |  |  |  |  |  |  |  |  |  |  |  |  |  |  |  |  |  |  |  |  |  |  |  |  |  |  |  |  |  |  |  |  |  |
| --- | --- | --- | --- | --- | --- | --- | --- | --- | --- | --- | --- | --- | --- | --- | --- | --- | --- | --- | --- | --- | --- | --- | --- | --- | --- | --- | --- | --- | --- | --- | --- | --- | --- | --- | --- | --- | --- | --- | --- | --- | --- | --- | --- | --- | --- | --- | --- | --- | --- | --- | --- | --- | --- | --- | --- | --- | --- | --- | --- | --- | --- | --- |
| Q9N4B1_CAEEL | - | - | - | - | - | - | - | - | - | - | - | - | - | - | - | - | - | - | - | - | - | - | - | - | - | - | - | - | - | - | - | - | - | - | - | - | - | - | - | - | - | - | - | - | - | - | - | - | - | - | - | - | - | - | - | - | - | - | - | - | - | -149 |
| Q8IAA8_CAEEL | - | - | - | - | - | - | - | - | - | - | - | - | - | - | - | - | - | - | - | - | - | - | - | - | - | - | - | - | - | - | - | - | - | - | - | - | - | - | - | - | - | - | - | - | - | - | - | - | - | - | - | - | - | - | - | - | - | - | - | - | - | -149 |
| CAN_CAEEL | - | - | - | - | - | - | - | - | - | - | - | - | - | - | - | - | - | - | - | - | - | - | - | - | - | - | - | - | - | - | - | - | - | - | - | - | - | - | - | - | - | - | - | - | - | - | - | - | - | - | - | - | - | - | - | - | - | - | - | - | - | -148 |
| Q9U2B2_CAEEL | - | - | - | - | - | - | - | - | - | - | - | - | - | - | - | - | - | - | - | - | - | - | - | - | - | - | - | - | - | - | - | - | - | - | - | - | - | - | - | - | - | - | - | - | - | - | - | - | - | - | - | - | - | - | - | - | - | - | - | - | - | -153 |
| Q9N4B2_CAEEL | - | - | - | - | - | - | - | - | - | - | - | - | - | - | - | - | - | - | - | - | - | - | - | - | - | - | - | - | - | - | - | - | - | - | - | - | - | - | - | - | - | - | - | - | - | - | - | - | - | - | - | - | - | - | - | - | - | - | - | - | - | -149 |
| C3Y6P6_BRAFL | - | - | - | - | - | - | - | - | - | - | - | - | - | - | - | - | - | - | - | - | - | - | - | - | - | - | - | - | - | - | - | - | - | - | - | - | - | - | - | - | - | - | - | - | - | - | - | - | - | - | - | - | - | - | - | - | - | - | - | - | - | -156 |
| C3Y6N1_BRAFL | - | - | - | - | - | - | - | - | - | - | - | - | - | - | - | - | - | - | - | - | - | - | - | - | - | - | - | - | - | - | - | - | - | - | - | - | - | - | - | - | - | - | - | - | - | - | - | - | - | - | - | - | - | - | - | - | - | - | - | - | - | -158 |
| CANA_DROME | - | - | - | - | - | - | - | - | - | - | - | - | - | - | - | - | - | - | - | - | - | - | - | - | - | - | - | - | - | - | - | - | - | - | - | - | - | - | - | - | - | - | - | - | - | - | - | - | - | - | - | - | - | - | - | - | - | - | - | - | - | -148 |
| CANB_DROME | - | - | - | - | - | - | - | - | - | - | - | - | - | - | - | - | - | - | - | - | - | - | - | - | - | - | - | - | - | - | - | - | - | - | - | - | - | - | - | - | - | - | - | - | - | - | - | - | - | - | - | - | - | - | - | - | - | - | - | - | - | -148 |
| X1Y3H71_ANODA | - | - | - | - | - | - | - | - | - | - | - | - | - | - | - | - | - | - | - | - | - | - | - | - | - | - | - | - | - | - | - | - | - | - | - | - | - | - | - | - | - | - | - | - | - | - | - | - | - | - | - | - | - | - | - | - | - | - | - | - | - | -149 |
| Q5IW12_GECLA | - | - | - | - | - | - | - | - | - | - | - | - | - | - | - | - | - | - | - | - | - | - | - | - | - | - | - | - | - | - | - | - | - | - | - | - | - | - | - | - | - | - | - | - | - | - | - | - | - | - | - | - | - | - | - | - | - | - | - | - | - | -148 |
| K1Q056_CRAGI | - | - | - | - | - | - | - | - | - | - | - | - | - | - | - | - | - | - | - | - | - | - | - | - | - | - | - | - | - | - | - | - | - | - | - | - | - | - | - | - | - | - | - | - | - | - | - | - | - | - | - | - | - | - | - | - | - | - | - | - | - | -148 |
| G4V6C0_SCHMA | - | - | - | - | - | - | - | - | - | - | - | - | - | - | - | - | - | - | - | - | - | - | - | - | - | - | - | - | - | - | - | - | - | - | - | - | - | - | - | - | - | - | - | - | - | - | - | - | - | - | - | - | - | - | - | - | - | - | - | - | - | -153 |
| R7ULX9_CAPTE | - | - | - | - | - | - | - | - | - | - | - | - | - | - | - | - | - | - | - | - | - | - | - | - | - | - | - | - | - | - | - | - | - | - | - | - | - | - | - | - | - | - | - | - | - | - | - | - | - | - | - | - | - | - | - | - | - | - | - | - | - | -149 |
| W4Z5Q7_STRPU | - | - | - | - | - | - | - | - | - | - | - | - | - | - | - | - | - | - | - | - | - | - | - | - | - | - | - | - | - | - | - | - | - | - | - | - | - | - | - | - | - | - | - | - | - | - | - | - | - | - | - | - | - | - | - | - | - | - | - | - | - | -146 |
| R7VIB6_CAPTE | - | - | - | - | - | - | - | - | - | - | - | - | - | - | - | - | - | - | - | - | - | - | - | - | - | - | - | - | - | - | - | - | - | - | - | - | - | - | - | - | - | - | - | - | - | - | - | - | - | - | - | - | - | - | - | - | - | - | - | - | - | -152 |
| T2MJ45_HYDVU | - | - | - | - | - | - | - | - | - | - | - | - | - | - | - | - | - | - | - | - | - | - | - | - | - | - | - | - | - | - | - | - | - | - | - | - | - | - | - | - | - | - | - | - | - | - | - | - | - | - | - | - | - | - | - | - | - | - | - | - | - | -148 |
| R7UAB6_CAPTE | - | - | - | - | - | - | - | - | - | - | - | - | - | - | - | - | - | - | - | - | - | - | - | - | - | - | - | - | - | - | - | - | - | - | - | - | - | - | - | - | - | - | - | - | - | - | - | - | - | - | - | - | - | - | - | - | - | - | - | - | - | -148 |
| C3ZKN8_BRAFL | - | - | - | - | - | - | - | - | - | - | - | - | - | - | - | - | - | - | - | - | - | - | - | - | - | - | - | - | - | - | - | - | - | - | - | - | - | - | - | - | - | - | - | - | - | - | - | - | - | - | - | - | - | - | - | - | - | - | - | - | - | -149 |
| B3RJS5_TRIAD | - | - | - | - | - | - | - | - | - | - | - | - | - | - | - | - | - | - | - | - | - | - | - | - | - | - | - | - | - | - | - | - | - | - | - | - | - | - | - | - | - | - | - | - | - | - | - | - | - | - | - | - | - | - | - | - | - | - | - | - | - | -148 |
| H2YVF0_CIOSA | - | - | - | - | - | - | - | - | - | - | - | - | - | - | - | - | - | - | - | - | - | - | - | - | - | - | - | - | - | - | - | - | - | - | - | - | - | - | - | - | - | - | - | - | - | - | - | - | - | - | - | - | - | - | - | - | - | - | - | - | - | -148 |
| G4VAG2_SCHMA | - | - | - | - | - | - | - | - | - | - | - | - | - | - | - | - | - | - | - | - | - | - | - | - | - | - | - | - | - | - | - | - | - | - | - | - | - | - | - | - | - | - | - | - | - | - | - | - | - | - | - | - | - | - | - | - | - | - | - | - | - | -153 |
| Smp_157500 | - | - | - | - | - | - | - | - | - | - | - | - | - | - | - | - | - | - | - | - | - | - | - | - | - | - | - | - | - | - | - | - | - | - | - | - | - | - | - | - | - | - | - | - | - | - | - | - | - | - | - | - | - | - | - | - | - | - | - | - | - | -153 |
| Q7SYA9_DANRE | - | - | - | - | - | - | - | - | - | - | - | - | - | - | - | - | - | - | - | - | - | - | - | - | - | - | - | - | - | - | - | - | - | - | - | - | - | - | - | - | - | - | - | - | - | - | - | - | - | - | - | - | - | - | - | - | - | - | - | - | - | -148 |
| A5PMP1_DANRE | - | - | - | - | - | - | - | - | - | - | - | - | - | - | - | - | - | - | - | - | - | - | - | - | - | - | - | - | - | - | - | - | - | - | - | - | - | - | - | - | - | - | - | - | - | - | - | - | - | - | - | - | - | - | - | - | - | - | - | - | - | -148 |
| CANX_CHICK | - | - | - | - | - | - | - | - | - | - | - | - | - | - | - | - | - | - | - | - | - | - | - | - | - | - | - | - | - | - | - | - | - | - | - | - | - | - | - | - | - | - | - | - | - | - | - | - | - | - | - | - | - | - | - | - | - | - | - | - | - | -148 |
| CAN1_HUMAN | - | - | - | - | - | - | - | - | - | - | - | - | - | - | - | - | - | - | - | - | - | - | - | - | - | - | - | - | - | - | - | - | - | - | - | - | - | - | - | - | - | - | - | - | - | - | - | - | - | - | - | - | - | - | - | - | - | - | - | - | - | -148 |
| CAN1_MOUSE | - | - | - | - | - | - | - | - | - | - | - | - | - | - | - | - | - | - | - | - | - | - | - | - | - | - | - | - | - | - | - | - | - | - | - | - | - | - | - | - | - | - | - | - | - | - | - | - | - | - | - | - | - | - | - | - | - | - | - | - | - | -148 |
| O42133_CHICK | - | - | - | - | - | - | - | - | - | - | - | - | - | - | - | - | - | - | - | - | - | - | - | - | - | - | - | - | - | - | - | - | - | - | - | - | - | - | - | - | - | - | - | - | - | - | - | - | - | - | - | - | - | - | - | - | - | - | - | - | - | -148 |
| Q7ZUR1_DANRE | - | - | - | - | - | - | - | - | - | - | - | - | - | - | - | - | - | - | - | - | - | - | - | - | - | - | - | - | - | - | - | - | - | - | - | - | - | - | - | - | - | - | - | - | - | - | - | - | - | - | - | - | - | - | - | - | - | - | - | - | - | -148 |
| E9QFN0_DANRE | - | - | - | - | - | - | - | - | - | - | - | - | - | - | - | - | - | - | - | - | - | - | - | - | - | - | - | - | - | - | - | - | - | - | - | - | - | - | - | - | - | - | - | - | - | - | - | - | - | - | - | - | - | - | - | - | - | - | - | - | - | -148 |
| CAN2_HUMAN | - | - | - | - | - | - | - | - | - | - | - | - | - | - | - | - | - | - | - | - | - | - | - | - | - | - | - | - | - | - | - | - | - | - | - | - | - | - | - | - | - | - | - | - | - | - | - | - | - | - | - | - | - | - | - | - | - | - | - | - | - | -148 |
| CAN2_MOUSE | - | - | - | - | - | - | - | - | - | - | - | - | - | - | - | - | - | - | - | - | - | - | - | - | - | - | - | - | - | - | - | - | - | - | - | - | - | - | - | - | - | - | - | - | - | - | - | - | - | - | - | - | - | - | - | - | - | - | - | - | - | -148 |
| CAN2_CHICK | - | - | - | - | - | - | - | - | - | - | - | - | - | - | - | - | - | - | - | - | - | - | - | - | - | - | - | - | - | - | - | - | - | - | - | - | - | - | - | - | - | - | - | - | - | - | - | - | - | - | - | - | - | - | - | - | - | - | - | - | - | -148 |
| Q6DFZ8_DANRE | - | - | - | - | - | - | - | - | - | - | - | - | - | - | - | - | - | - | - | - | - | - | - | - | - | - | - | - | - | - | - | - | - | - | - | - | - | - | - | - | - | - | - | - | - | - | - | - | - | - | - | - | - | - | - | - | - | - | - | - | - | -148 |
| Q504F5_DANRE | - | - | - | - | - | - | - | - | - | - | - | - | - | - | - | - | - | - | - | - | - | - | - | - | - | - | - | - | - | - | - | - | - | - | - | - | - | - | - | - | - | - | - | - | - | - | - | - | - | - | - | - | - | - | - | - | - | - | - | - | - | -148 |
| Q5BLH5_DANRE | - | - | - | - | - | - | - | - | - | - | - | - | - | - | - | - | - | - | - | - | - | - | - | - | - | - | - | - | - | - | - | - | - | - | - | - | - | - | - | - | - | - | - | - | - | - | - | - | - | - | - | - | - | - | - | - | - | - | - | - | - | -148 |
| Q4VBH3_DANRE | - | - | - | - | - | - | - | - | - | - | - | - | - | - | - | - | - | - | - | - | - | - | - | - | - | - | - | - | - | - | - | - | - | - | - | - | - | - | - | - | - | - | - | - | - | - | - | - | - | - | - | - | - | - | - | - | - | - | - | - | - | -148 |
| CAN8_RAT | - | - | - | - | - | - | - | - | - | - | - | - | - | - | - | - | - | - | - | - | - | - | - | - | - | - | - | - | - | - | - | - | - | - | - | - | - | - | - | - | - | - | - | - | - | - | - | - | - | - | - | - | - | - | - | - | - | - | - | - | - | -148 |
| CAN8_MOUSE | - | - | - | - | - | - | - | - | - | - | - | - | - | - | - | - | - | - | - | - | - | - | - | - | - | - | - | - | - | - | - | - | - | - | - | - | - | - | - | - | - | - | - | - | - | - | - | - | - | - | - | - | - | - | - | - | - | - | - | - | - | -148 |
| CAN8_HUMAN | - | - | - | - | - | - | - | - | - | - | - | - | - | - | - | - | - | - | - | - | - | - | - | - | - | - | - | - | - | - | - | - | - | - | - | - | - | - | - | - | - | - | - | - | - | - | - | - | - | - | - | - | - | - | - | - | - | - | - | - | - | -148 |
| XP_426117 | - | - | - | - | - | - | - | - | - | - | - | - | - | - | - | - | - | - | - | - | - | - | - | - | - | - | - | - | - | - | - | - | - | - | - | - | - | - | - | - | - | - | - | - | - | - | - | - | - | - | - | - | - | - | - | - | - | - | - | - | - | -148 |
| CAN9_MOUSE | - | - | - | - | - | - | - | - | - | - | - | - | - | - | - | - | - | - | - | - | - | - | - | - | - | - | - | - | - | - | - | - | - | - | - | - | - | - | - | - | - | - | - | - | - | - | - | - | - | - | - | - | - | - | - | - | - | - | - | - | - | -150 |
| CAN9_HUMAN | - | - | - | - | - | - | - | - | - | - | - | - | - | - | - | - | - | - | - | - | - | - | - | - | - | - | - | - | - | - | - | - | - | - | - | - | - | - | - | - | - | - | - | - | - | - | - | - | - | - | - | - | - | - | - | - | - | - | - | - | - | -148 |
| E1C6M3_CHICK | - | - | - | - | - | - | - | - | - | - | - | - | - | - | - | - | - | - | - | - | - | - | - | - | - | - | - | - | - | - | - | - | - | - | - | - | - | - | - | - | - | - | - | - | - | - | - | - | - | - | - | - | - | - | - | - | - | - | - | - | - | -148 |
| Q6DG44_DANRE | - | - | - | - | - | - | - | - | - | - | - | - | - | - | - | - | - | - | - | - | - | - | - | - | - | - | - | - | - | - | - | - | - | - | - | - | - | - | - | - | - | - | - | - | - | - | - | - | - | - | - | - | - | - | - | - | - | - | - | - | - | -148 |
| CAN3_HUMAN | - | - | - | - | - | - | - | - | - | - | - | - | - | - | - | - | - | - | - | - | - | - | - | - | - | - | - | - | - | - | - | - | - | - | - | - | - | - | - | - | - | - | - | - | - | - | - | - | - | - | - | - | - | - | - | - | - | - | - | - | - | -148 |
| CAN3_MOUSE | - | - | - | - | - | - | - | - | - | - | - | - | - | - | - | - | - | - | - | - | - | - | - | - | - | - | - | - | - | - | - | - | - | - | - | - | - | - | - | - | - | - | - | - | - | - | - | - | - | - | - | - | - | - | - | - | - | - | - | - | - | -148 |
| CAN3_CHICK | - | - | - | - | - | - | - | - | - | - | - | - | - | - | - | - | - | - | - | - | - | - | - | - | - | - | - | - | - | - | - | - | - | - | - | - | - | - | - | - | - | - | - | - | - | - | - | - | - | - | - | - | - | - | - | - | - | - | - | - | - | -148 |
| Q66HU4_DANRE | - | - | - | - | - | - | - | - | - | - | - | - | - | - | - | - | - | - | - | - | - | - | - | - | - | - | - | - | - | - | - | - | - | - | - | - | - | - | - | - | - | - | - | - | - | - | - | - | - | - | - | - | - | - | - | - | - | - | - | - | - | -148 |
| F1R632_DANRE | - | - | - | - | - | - | - | - | - | - | - | - | - | - | - | - | - | - | - | - | - | - | - | - | - | - | - | - | - | - | - | - | - | - | - | - | - | - | - | - | - | - | - | - | - | - | - | - | - | - | - | - | - | - | - | - | - | - | - | - | - | -148 |
| CAN11_HUMAN | - | - | - | - | - | - | - | - | - | - | - | - | - | - | - | - | - | - | - | - | - | - | - | - | - | - | - | - | - | - | - | - | - | - | - | - | - | - | - | - | - | - | - | - | - | - | - | - | - | - | - | - | - | - | - | - | - | - | - | - | - | -148 |
| CAN11_MOUSE | - | - | - | - | - | - | - | - | - | - | - | - | - | - | - | - | - | - | - | - | - | - | - | - | - | - | - | - | - | - | - | - | - | - | - | - | - | - | - | - | - | - | - | - | - | - | - | - | - | - | - | - | - | - | - | - | - | - | - | - | - | -148 |
| CAN12_MOUSE | - | - | - | - | - | - | - | - | - | - | - | - | - | - | - | - | - | - | - | - | - | - | - | - | - | - | - | - | - | - | - | - | - | - | - | - | - | - | - | - | - | - | - | - | - | - | - | - | - | - | - | - | - | - | - | - | - | - | - | - | - | -148 |
| CAN12_HUMAN | - | - | - | - | - | - | - | - | - | - | - | - | - | - | - | - | - | - | - | - | - | - | - | - | - | - | - | - | - | - | - | - | - | - | - | - | - | - | - | - | - | - | - | - | - | - | - | - | - | - | - | - | - | - | - | - | - | - | - | - | - | -148 |
| ENSSARP00000008135 | - | - | - | - | - | - | - | - | - | - | - | - | - | - | - | - | - | - | - | - | - | - | - | - | - | - | - | - | - | - | - | - | - | - | - | - | - | - | - | - | - | - | - | - | - | - | - | - | - | - | - | - | - | - | - | - | - | - | - | - | - | -148 |
| ENSSARP00000010553 | - | - | - | - | - | - | - | - | - | - | - | - | - | - | - | - | - | - | - | - | - | - | - | - | - | - | - | - | - | - | - | - | - | - | - | - | - | - | - | - | - | - | - | - | - | - | - | - | - | - | - | - | - | - | - | - | - | - | - | - | - | -147 |
| A7E2G3_DANRE | - | - | - | - | - | - | - | - | - | - | - | - | - | - | - | - | - | - | - | - | - | - | - | - | - | - | - | - | - | - | - | - | - | - | - | - | - | - | - | - | - | - | - | - | - | - | - | - | - | - | - | - | - | - | - | - | - | - | - | - | - | -149 |
| Q6NW69_DANRE | - | - | - | - | - | - | - | - | - | - | - | - | - | - | - | - | - | - | - | - | - | - | - | - | - | - | - | - | - | - | - | - | - | - | - | - | - | - | - | - | - | - | - | - | - | - | - | - | - | - | - | - | - | - | - | - | - | - | - | - | - | -148 |
| CAN13_HUMAN | - | - | - | - | - | - | - | - | - | - | - | - | - | - | - | - | - | - | - | - | - | - | - | - | - | - | - | - | - | - | - | - | - | - | - | - | - | - | - | - | - | - | - | - | - | - | - | - | - | - | - | - | - | - | - | - | - | - | - | - | - | -149 |
| CAN13_MOUSE | - | - | - | - | - | - | - | - | - | - | - | - | - | - | - | - | - | - | - | - | - | - | - | - | - | - | - | - | - | - | - | - | - | - | - | - | - | - | - | - | - | - | - | - | - | - | - | - | - | - | - | - | - | - | - | - | - | - | - | - | - | -151 |
| E1BRR5_CHICK | - | - | - | - | - | - | - | - | - | - | - | - | - | - | - | - | - | - | - | - | - | - | - | - | - | - | - | - | - | - | - | - | - | - | - | - | - | - | - | - | - | - | - | - | - | - | - | - | - | - | - | - | - | - | - | - | - | - | - | - | - | -148 |
| E7F0H3_DANRE | - | - | - | - | - | - | - | - | - | - | - | - | - | - | - | - | - | - | - | - | - | - | - | - | - | - | - | - | - | - | - | - | - | - | - | - | - | - | - | - | - | - | - | - | - | - | - | - | - | - | - | - | - | - | - | - | - | - | - | - | - | -149 |
| Q1RLS9_DANRE | - | - | - | - | - | - | - | - | - | - | - | - | - | - | - | - | - | - | - | - | - | - | - | - | - | - | - | - | - | - | - | - | - | - | - | - | - | - | - | - | - | - | - | - | - | - | - | - | - | - | - | - | - | - | - | - | - | - | - | - | - | -148 |
| F1Q9Z1_DANRE | - | - | - | - | - | - | - | - | - | - | - | - | - | - | - | - | - | - | - | - | - | - | - | - | - | - | - | - | - | - | - | - | - | - | - | - | - | - | - | - | - | - | - | - | - | - | - | - | - | - | - | - | - | - | - | - | - | - | - | - | - | -149 |
| A4QNV6_DANRE | - | - | - | - | - | - | - | - | - | - | - | - | - | - | - | - | - | - | - | - | - | - | - | - | - | - | - | - | - | - | - | - | - | - | - | - | - | - | - | - | - | - | - | - | - | - | - | - | - | - | - | - | - | - | - | - | - | - | - | - | - | -149 |
| D6W573_HUMAN | - | - | - | - | - | - | - | - | - | - | - | - | - | - | - | - | - | - | - | - | - | - | - | - | - | - | - | - | - | - | - | - | - | - | - | - | - | - | - | - | - | - | - | - | - | - | - | - | - | - | - | - | - | - | - | - | - | - | - | - | - | -149 |
| R7UBC6_CAPTE | - | - | - | - | - | - | - | - | - | - | - | - | - | - | - | - | - | - | - | - | - | - | - | - | - | - | - | - | - | - | - | - | - | - | - | - | - | - | - | - | - | - | - | - | - | - | - | - | - | - | - | - | - | - | - | - | - | - | - | - | - | -156 |
| K1RCZ2_CRAGI | - | - | - | - | - | - | - | - | - | - | - | - | - | - | - | - | - | - | - | - | - | - | - | - | - | - | - | - | - | - | - | - | - | - | - | - | - | - | - | - | - | - | - | - | - | - | - | - | - | - | - | - | - | - | - | - | - | - | - | - | - | -159 |
| Q5IW13_GECLA | - | - | - | - | - | - | - | - | - | - | - | - | - | - | - | - | - | - | - | - | - | - | - | - | - | - | - | - | - | - | - | - | - | - | - | - | - | - | - | - | - | - | - | - | - | - | - | - | - | - | - | - | - | - | - | - | - | - | - | - | - | -148 |
| R7UUX7_CAPTE | - | - | - | - | - | - | - | - | - | - | - | - | - | - | - | - | - | - | - | - | - | - | - | - | - | - | - | - | - | - | - | - | - | - | - | - | - | - | - | - | - | - | - | - | - | - | - | - | - | - | - | - | - | - | - | - | - | - | - | - | - | -151 |
| K1Q505_CRAGI | - | - | - | - | - | - | - | - | - | - | - | - | - | - | - | - | - | - | - | - | - | - | - | - | - | - | - | - | - | - | - | - | - | - | - | - | - | - | - | - | - | - | - | - | - | - | - | - | - | - | - | - | - | - | - | - | - | - | - | - | - | -152 |
| R7UD02_CAPTE | - | - | - | - | - | - | - | - | - | - | - | - | - | - | - | - | - | - | - | - | - | - | - | - | - | - | - | - | - | - | - | - | - | - | - | - | - | - | - | - | - | - | - | - | - | - | - | - | - | - | - | - | - | - | - | - | - | - | - | - | - | -151 |
| CAN6_HUMAN | - | - | - | - | - | - | - | - | - | - | - | - | - | - | - | - | - | - | - | - | - | - | - | - | - | - | - | - | - | - | - | - | - | - | - | - | - | - | - | - | - | - | - | - | - | - | - | - | - | - | - | - | - | - | - | - | - | - | - | - | - | -161 |
| CAN6_MOUSE | - | - | - | - | - | - | - | - | - | - | - | - | - | - | - | - | - | - | - | - | - | - | - | - | - | - | - | - | - | - | - | - | - | - | - | - | - | - | - | - | - | - | - | - | - | - | - | - | - | - | - | - | - | - | - | - | - | - | - | - | - | -161 |
| E1C312_CHICK | - | - | - | - | - | - | - | - | - | - | - | - | - | - | - | - | - | - | - | - | - | - | - | - | - | - | - | - | - | - | - | - | - | - | - | - | - | - | - | - | - | - | - | - | - | - | - | - | - | - | - | - | - | - | - | - | - | - | - | - | - | -161 |
| E7FDN3_DANRE | - | - | - | - | - | - | - | - | - | - | - | - | - | - | - | - | - | - | - | - | - | - | - | - | - | - | - | - | - | - | - | - | - | - | - | - | - | - | - | - | - | - | - | - | - | - | - | - | - | - | - | - | - | - | - | - | - | - | - | - | - | -162 |
| CAN5_HUMAN | - | - | - | - | - | - | - | - | - | - | - | - | - | - | - | - | - | - | - | - | - | - | - | - | - | - | - | - | - | - | - | - | - | - | - | - | - | - | - | - | - | - | - | - | - | - | - | - | - | - | - | - | - | - | - | - | - | - | - | - | - | -157 |
| CAN5_MOUSE | - | - | - | - | - | - | - | - | - | - | - | - | - | - | - | - | - | - | - | - | - | - | - | - | - | - | - | - | - | - | - | - | - | - | - | - | - | - | - | - | - | - | - | - | - | - | - | - | - | - | - | - | - | - | - | - | - | - | - | - | - | -161 |
| E1C292_CHICK | - | - | - | - | - | - | - | - | - | - | - | - | - | - | - | - | - | - | - | - | - | - | - | - | - | - | - | - | - | - | - | - | - | - | - | - | - | - | - | - | - | - | - | - | - | - | - | - | - | - | - | - | - | - | - | - | - | - | - | - | - | -161 |
| A0JMG3_DANRE | - | - | - | - | - | - | - | - | - | - | - | - | - | - | - | - | - | - | - | - | - | - | - | - | - | - | - | - | - | - | - | - | - | - | - | - | - | - | - | - | - | - | - | - | - | - | - | - | - | - | - | - | - | - | - | - | - | - | - | - | - | -161 |
| E7F7F1_DANRE | - | - | - | - | - | - | - | - | - | - | - | - | - | - | - | - | - | - | - | - | - | - | - | - | - | - | - | - | - | - | - | - | - | - | - | - | - | - | - | - | - | - | - | - | - | - | - | - | - | - | - | - | - | - | - | - | - | - | - | - | - | -161 |
| XP_006824412 | - | - | - | - | - | - | - | - | - | - | - | - | - | - | - | - | - | - | - | - | - | - | - | - | - | - | - | - | - | - | - | - | - | - | - | - | - | - | - | - | - | - | - | - | - | - | - | - | - | - | - | - | - | - | - | - | - | - | - | - | - | -153 |
| W4YJN2_STRPU | - | - | - | - | - | - | - | - | - | - | - | - | - | - | - | - | - | - | - | - | - | - | - | - | - | - | - | - | - | - | - | - | - | - | - | - | - | - | - | - | - | - | - | - | - | - | - | - | - | - | - | - | - | - | - | - | - | - | - | - | - | -161 |
| C3YJZ3_BRAFL | - | - | - | - | - | - | - | - | - | - | - | - | - | - | - | - | - | - | - | - | - | - | - | - | - | - | - | - | - | - | - | - | - | - | - | - | - | - | - | - | - | - | - | - | - | - | - | - | - | - | - | - | - | - | - | - | - | - | - | - | - | -161 |
| T2MIT4_HYDVU | - | - | - | - | - | - | - | - | - | - | - | - | - | - | - | - | - | - | - | - | - | - | - | - | - | - | - | - | - | - | - | - | - | - | - | - | - | - | - | - | - | - | - | - | - | - | - | - | - | - | - | - | - | - | - | - | - | - | - | - | - | -161 |
| XP_002154722 | - | - | - | - | - | - | - | - | - | - | - | - | - | - | - | - | - | - | - | - | - | - | - | - | - | - | - | - | - | - | - | - | - | - | - | - | - | - | - | - | - | - | - | - | - | - | - | - | - | - | - | - | - | - | - | - | - | - | - | - | - | -125 |
| R7VLA7_CAPTE | - | - | - | - | - | - | - | - | - | - | - | - | - | - | - | - | - | - | - | - | - | - | - | - | - | - | - | - | - | - | - | - | - | - | - | - | - | - | - | - | - | - | - | - | - | - | - | - | - | - | - | - | - | - | - | - | - | - | - | - | - | -161 |
| K1R1C6_CRAGI | - | - | - | - | - | - | - | - | - | - | - | - | - | - | - | - | - | - | - | - | - | - | - | - | - | - | - | - | - | - | - | - | - | - | - | - | - | - | - | - | - | - | - | - | - | - | - | - | - | - | - | - | - | - | - | - | - | - | - | - | - | -161 |
| B3RIE5_TRIAD | - | - | - | - | - | - | - | - | - | - | - | - | - | - | - | - | - | - | - | - | - | - | - | - | - | - | - | - | - | - | - | - | - | - | - | - | - | - | - | - | - | - | - | - | - | - | - | - | - | - | - | - | - | - | - | - | - | - | - | - | - | -159 |
| K1PW75_CRAGI | - | - | - | - | - | - | - | - | - | - | - | - | - | - | - | - | - | - | - | - | - | - | - | - | - | - | - | - | - | - | - | - | - | - | - | - | - | - | - | - | - | - | - | - | - | - | - | - | - | - | - | - | - | - | - | - | - | - | - | - | - | -157 |
| K1RSB8_CRAGI | - | - | - | - | - | - | - | - | - | - | - | - | - | - | - | - | - | - | - | - | - | - | - | - | - | - | - | - | - | - | - | - | - | - | - | - | - | - | - | - | - | - | - | - | - | - | - | - | - | - | - | - | - | - | - | - | - | - | - | - | - | -156 |
| K1RWI3_CRAGI | - | - | - | - | - | - | - | - | - | - | - | - | - | - | - | - | - | - | - | - | - | - | - | - | - | - | - | - | - | - | - | - | - | - | - | - | - | - | - | - | - | - | - | - | - | - | - | - | - | - | - | - | - | - | - | - | - | - | - | - | - | -159 |
| R7VGC2_CAPTE | - | - | - | - | - | - | - | - | - | - | - | - | - | - | - | - | - | - | - | - | - | - | - | - | - | - | - | - | - | - | - | - | - | - | - | - | - | - | - | - | - | - | - | - | - | - | - | - | - | - | - | - | - | - | - | - | - | - | - | - | - | -159 |
| K1QU26_CRAGI | - | - | - | - | - | - | - | - | - | - | - | - | - | - | - | - | - | - | - | - | - | - | - | - | - | - | - | - | - | - | - | - | - | - | - | - | - | - | - | - | - | - | - | - | - | - | - | - | - | - | - | - | - | - | - | - | - | - | - | - | - | -161 |
| CAN5_CAEEL | - | - | - | - | - | - | - | - | - | - | - | - | - | - | - | - | - | - | - | - | - | - | - | - | - | - | - | - | - | - | - | - | - | - | - | - | - | - | - | - | - | - | - | - | - | - | - | - | - | - | - | - | - | - | - | - | - | - | - | - | - | -159 |
| I1FGV4_AMPQE | - | - | - | - | - | - | - | - | - | - | - | - | - | - | - | - | - | - | - | - | - | - | - | - | - | - | - | - | - | - | - | - | - | - | - | - | - | - | - | - | - | - | - | - | - | - | - | - | - | - | - | - | - | - | - | - | - | - | - | - | - | -147 |
| K1PHX3_CRAGI | - | - | - | - | - | - | - | - | - | - | - | - | - | - | - | - | - | - | - | - | - | - | - | - | - | - | - | - | - | - | - | - | - | - | - | - | - | - | - | - | - | - | - | - | - | - | - | - | - | - | - | - | - | - | - | - | - | - | - | - | - | -148 |
| B3RNL8_TRIAD | - | - | - | - | - | - | - | - | - | - | - | - | - | - | - | - | - | - | - | - | - | - | - | - | - | - | - | - | - | - | - | - | - | - | - | - | - | - | - | - | - | - | - | - | - | - | - | - | - | - | - | - | - | - | - | - | - | - | - | - | - | -150 |
| X1Y3H7_ANODA | - | - | - | - | - | - | - | - | - | - | - | - | - | - | - | - | - | - | - | - | - | - | - | - | - | - | - | - | - | - | - | - | - | - | - | - | - | - | - | - | - | - | - | - | - | - | - | - | - | - | - | - | - | - | - | - | - | - | - | - | - | -152 |
| R7TWH9_CAPTE | - | - | - | - | - | - | - | - | - | - | - | - | - | - | - | - | - | - | - | - | - | - | - | - | - | - | - | - | - | - | - | - | - | - | - | - | - | - | - | - | - | - | - | - | - | - | - | - | - | - | - | - | - | - | - | - | - | - | - | - | - | -152 |
| K1R5E8_CRAGI | - | - | - | - | - | - | - | - | - | - | - | - | - | - | - | - | - | - | - | - | - | - | - | - | - | - | - | - | - | - | - | - | - | - | - | - | - | - | - | - | - | - | - | - | - | - | - | - | - | - | - | - | - | - | - | - | - | - | - | - | - | -152 |
| XP_006811804 | - | - | - | - | - | - | - | - | - | - | - | - | - | - | - | - | - | - | - | - | - | - | - | - | - | - | - | - | - | - | - | - | - | - | - | - | - | - | - | - | - | - | - | - | - | - | - | - | - | - | - | - | - | - | - | - | - | - | - | - | - | -150 |
| C3YB23_BRAFL | - | - | - | - | - | - | - | - | - | - | - | - | - | - | - | - | - | - | - | - | - | - | - | - | - | - | - | - | - | - | - | - | - | - | - | - | - | - | - | - | - | - | - | - | - | - | - | - | - | - | - | - | - | - | - | - | - | - | - | - | - | -157 |
| H2ZIF1_CIOSA | - | - | - | - | - | - | - | - | - | - | - | - | - | - | - | - | - | - | - | - | - | - | - | - | - | - | - | - | - | - | - | - | - | - | - | - | - | - | - | - | - | - | - | - | - | - | - | - | - | - | - | - | - | - | - | - | - | - | - | - | - | -150 |
| W4YV93_STRPU | - | - | - | - | - | - | - | - | - | - | - | - | - | - | - | - | - | - | - | - | - | - | - | - | - | - | - | - | - | - | - | - | - | - | - | - | - | - | - | - | - | - | - | - | - | - | - | - | - | - | - | - | - | - | - | - | - | - | - | - | - | -150 |
| CAN10_HUMAN | - | - | - | - | - | - | - | - | - | - | - | - | - | - | - | - | - | - | - | - | - | - | - | - | - | - | - | - | - | - | - | - | - | - | - | - | - | - | - | - | - | - | - | - | - | - | - | - | - | - | - | - | - | - | - | - | - | - | - | - | - | -157 |
| CAN10_MOUSE | - | - | - | - | - | - | - | - | - | - | - | - | - | - | - | - | - | - | - | - | - | - | - | - | - | - | - | - | - | - | - | - | - | - | - | - | - | - | - | - | - | - | - | - | - | - | - | - | - | - | - | - | - | - | - | - | - | - | - | - | - | -157 |
| XP_004943443 | - | - | - | - | - | - | - | - | - | - | - | - | - | - | - | - | - | - | - | - | - | - | - | - | - | - | - | - | - | - | - | - | - | - | - | - | - | - | - | - | - | - | - | - | - | - | - | - | - | - | - | - | - | - | - | - | - | - | - | - | - | -157 |
| F8W4K1_DANRE | - | - | - | - | - | - | - | - | - | - | - | - | - | - | - | - | - | - | - | - | - | - | - | - | - | - | - | - | - | - | - | - | - | - | - | - | - | - | - | - | - | - | - | - | - | - | - | - | - | - | - | - | - | - | - | - | - | - | - | - | - | -157 |
| CANC_DROME | - | - | - | - | - | - | - | - | - | - | - | - | - | - | - | - | - | - | - | - | - | - | - | - | - | - | - | - | - | - | - | - | - | - | - | - | - | - | - | - | - | - | - | - | - | - | - | - | - | - | - | - | - | - | - | - | - | - | - | - | - | -143 |
| C3ZKN9_BRAFL | - | - | - | - | - | - | - | - | - | - | - | - | - | - | - | - | - | - | - | - | - | - | - | - | - | - | - | - | - | - | - | - | - | - | - | - | - | - | - | - | - | - | - | - | - | - | - | - | - | - | - | - | - | - | - | - | - | - | - | - | - | -149 |
| G4V623_SCHMA | - | - | - | - | - | - | - | - | - | - | - | - | - | - | - | - | - | - | - | - | - | - | - | - | - | - | - | - | - | - | - | - | - | - | - | - | - | - | - | - | - | - | - | - | - | - | - | - | - | - | - | - | - | - | - | - | - | - | - | - | - | -174 |
| R7VK39_CAPTE | - | - | - | - | - | - | - | - | - | - | - | - | - | - | - | - | - | - | - | - | - | - | - | - | - | - | - | - | - | - | - | - | - | - | - | - | - | - | - | - | - | - | - | - | - | - | - | - | - | - | - | - | - | - | - | - | - | - | - | - | - | -151 |
| G4V693_SCHMA | - | - | - | - | - | - | - | - | - | - | - | - | - | - | - | - | - | - | - | - | - | - | - | - | - | - | - | - | - | - | - | - | - | - | - | - | - | - | - | - | - | - | - | - | - | - | - | - | - | - | - | - | - | - | - | - | - | - | - | - | - | -160 |
| R7UPU6_CAPTE | - | - | - | - | - | - | - | - | - | - | - | - | - | - | - | - | - | - | - | - | - | - | - | - | - | - | - | - | - | - | - | - | - | - | - | - | - | - | - | - | - | - | - | - | - | - | - | - | - | - | - | - | - | - | - | - | - | - | - | - | - | -155 |
| R7V817_CAPTE | - | - | - | - | - | - | - | - | - | - | - | - | - | - | - | - | - | - | - | - | - | - | - | - | - | - | - | - | - | - | - | - | - | - | - | - | - | - | - | - | - | - | - | - | - | - | - | - | - | - | - | - | - | - | - | - | - | - | - | - | - | -152 |
| R7UNL8_CAPTE | - | - | - | - | - | - | - | - | - | - | - | - | - | - | - | - | - | - | - | - | - | - | - | - | - | - | - | - | - | - | - | - | - | - | - | - | - | - | - | - | - | - | - | - | - | - | - | - | - | - | - | - | - | - | - | - | - | - | - | - | - | -150 |
| K1PJ80_CRAGI | - | - | - | - | - | - | - | - | - | - | - | - | - | - | - | - | - | - | - | - | - | - | - | - | - | - | - | - | - | - | - | - | - | - | - | - | - | - | - | - | - | - | - | - | - | - | - | - | - | - | - | - | - | - | - | - | - | - | - | - | - | -155 |
| K1PCX8_CRAGI | - | - | - | - | - | - | - | - | - | - | - | - | - | - | - | - | - | - | - | - | - | - | - | - | - | - | - | - | - | - | - | - | - | - | - | - | - | - | - | - | - | - | - | - | - | - | - | - | - | - | - | - | - | - | - | - | - | - | - | - | - | -155 |
| CAND_DROME | - | - | - | - | - | - | - | - | - | - | - | - | - | - | - | - | - | - | - | - | - | - | - | - | - | - | - | - | - | - | - | - | - | - | - | - | - | - | - | - | - | - | - | - | - | - | - | - | - | - | - | - | - | - | - | - | - | - | - | - | - | -152 |
| XP_002735765 | - | - | - | - | - | - | - | - | - | - | - | - | - | - | - | - | - | - | - | - | - | - | - | - | - | - | - | - | - | - | - | - | - | - | - | - | - | - | - | - | - | - | - | - | - | - | - | - | - | - | - | - | - | - | - | - | - | - | - | - | - | -154 |
| CAN15_HUMAN | - | - | - | - | - | - | - | - | - | - | - | - | - | - | - | - | - | - | - | - | - | - | - | - | - | - | - | - | - | - | - | - | - | - | - | - | - | - | - | - | - | - | - | - | - | - | - | - | - | - | - | - | - | - | - | - | - | - | - | - | - | -153 |
| CAN15_MOUSE | - | - | - | - | - | - | - | - | - | - | - | - | - | - | - | - | - | - | - | - | - | - | - | - | - | - | - | - | - | - | - | - | - | - | - | - | - | - | - | - | - | - | - | - | - | - | - | - | - | - | - | - | - | - | - | - | - | - | - | - | - | -153 |
| E1BTA8_CHICK | - | - | - | - | - | - | - | - | - | - | - | - | - | - | - | - | - | - | - | - | - | - | - | - | - | - | - | - | - | - | - | - | - | - | - | - | - | - | - | - | - | - | - | - | - | - | - | - | - | - | - | - | - | - | - | - | - | - | - | - | - | -153 |
| E7FAX5_DANRE | - | - | - | - | - | - | - | - | - | - | - | - | - | - | - | - | - | - | - | - | - | - | - | - | - | - | - | - | - | - | - | - | - | - | - | - | - | - | - | - | - | - | - | - | - | - | - | - | - | - | - | - | - | - | - | - | - | - | - | - | - | -153 |
| C3XWK8_BRAFL | - | - | - | - | - | - | - | - | - | - | - | - | - | - | - | - | - | - | - | - | - | - | - | - | - | - | - | - | - | - | - | - | - | - | - | - | - | - | - | - | - | - | - | - | - | - | - | - | - | - | - | - | - | - | - | - | - | - | - | - | - | -153 |
| W4YMU4_STRPU | - | - | - | - | - | - | - | - | - | - | - | - | - | - | - | - | - | - | - | - | - | - | - | - | - | - | - | - | - | - | - | - | - | - | - | - | - | - | - | - | - | - | - | - | - | - | - | - | - | - | - | - | - | - | - | - | - | - | - | - | - | -267 |
| K1PV74_CRAGI | - | - | - | - | - | - | - | - | - | - | - | - | - | - | - | - | - | - | - | - | - | - | - | - | - | - | - | - | - | - | - | - | - | - | - | - | - | - | - | - | - | - | - | - | - | - | - | - | - | - | - | - | - | - | - | - | - | - | - | - | - | -153 |
| R7TJF2_CAPTE | - | - | - | - | - | - | - | - | - | - | - | - | - | - | - | - | - | - | - | - | - | - | - | - | - | - | - | - | - | - | - | - | - | - | - | - | - | - | - | - | - | - | - | - | - | - | - | - | - | - | - | - | - | - | - | - | - | - | - | - | - | -152 |
| T2MI17_HYDVU | - | - | - | - | - | - | - | - | - | - | - | - | - | - | - | - | - | - | - | - | - | - | - | - | - | - | - | - | - | - | - | - | - | - | - | - | - | - | - | - | - | - | - | - | - | - | - | - | - | - | - | - | - | - | - | - | - | - | - | - | - | -150 |
| Q1RL55_CIOIN | - | - | - | - | - | - | - | - | - | - | - | - | - | - | - | - | - | - | - | - | - | - | - | - | - | - | - | - | - | - | - | - | - | - | - | - | - | - | - | - | - | - | - | - | - | - | - | - | - | - | - | - | - | - | - | - | - | - | - | - | - | -139 |
| I1EA30_AMPQE | - | - | - | - | - | - | - | - | - | - | - | - | - | - | - | - | - | - | - | - | - | - | - | - | - | - | - | - | - | - | - | - | - | - | - | - | - | - | - | - | - | - | - | - | - | - | - | - | - | - | - | - | - | - | - | - | - | - | - | - | - | -153 |
| G5EEZ6_CAEEL | - | - | - | - | - | - | - | - | - | - | - | - | - | - | - | - | - | - | - | - | - | - | - | - | - | - | - | - | - | - | - | - | - | - | - | - | - | - | - | - | - | - | - | - | - | - | - | - | - | - | - | - | - | - | - | - | - | - | - | - | - | -153 |
| O44903_CAEEL | - | - | - | - | - | - | - | - | - | - | - | - | - | - | - | - | - | - | - | - | - | - | - | - | - | - | - | - | - | - | - | - | - | - | - | - | - | - | - | - | - | - | - | - | - | - | - | - | - | - | - | - | - | - | - | - | - | - | - | - | - | -153 |
| O02260_CAEEL | - | - | - | - | - | - | - | - | - | - | - | - | - | - | - | - | - | - | - | - | - | - | - | - | - | - | - | - | - | - | - | - | - | - | - | - | - | - | - | - | - | - | - | - | - | - | - | - | - | - | - | - | - | - | - | - | - | - | - | - | - | -151 |
| O02259_CAEEL | - | - | - | - | - | - | - | - | - | - | - | - | - | - | - | - | - | - | - | - | - | - | - | - | - | - | - | - | - | - | - | - | - | - | - | - | - | - | - | - | - | - | - | - | - | - | - | - | - | - | - | - | - | - | - | - | - | - | - | - | - | -128 |
| C1MTG3_MICPC | - | S | P | A | L | P | F | Q | R | L | T | - | - | - | - | - | - | - | - | - | - | - | - | - | - | - | - | - | - | - | - | - | - | - | - | - | - | - | - | - | - | - | - | - | - | - | - | - | - | - | - | - | - | - | - | - | - | - | - | - | - | -195 |
| C1E493_MICSR | - | - | - | - | - | - | - | - | - | - | - | - | - | - | - | - | - | - | - | - | - | - | - | - | - | - | - | - | - | - | - | - | - | - | - | - | - | - | - | - | - | - | - | - | - | - | - | - | - | - | - | - | - | - | - | - | - | - | - | - | - | -146 |
| Q012Z6_OSTTA | - | - | - | - | - | - | - | - | - | - | - | - | - | - | - | - | - | - | - | - | - | - | - | - | - | - | - | - | - | - | - | - | - | - | - | - | - | - | - | - | - | - | - | - | - | - | - | - | - | - | - | - | - | - | - | - | - | - | - | - | - | -143 |
| L1IGB6_GUITH | - | - | - | - | - | - | - | - | - | - | - | - | - | - | - | - | - | - | - | - | - | - | - | - | - | - | - | - | - | - | - | - | - | - | - | - | - | - | - | - | - | - | - | - | - | - | - | - | - | - | - | - | - | - | - | - | - | - | - | - | - | -146 |
| B7FWX6_PHATC | - | - | - | - | - | - | - | - | - | - | - | - | - | - | - | - | - | - | - | - | - | - | - | - | - | - | - | - | - | - | - | - | - | - | - | - | - | - | - | - | - | - | - | - | - | - | - | - | - | - | - | - | - | - | - | - | - | - | - | - | - | -151 |
| N9TPR9_ENTHI | - | - | - | - | - | - | - | - | - | - | - | - | - | - | - | - | - | - | - | - | - | - | - | - | - | - | - | - | - | - | - | - | - | - | - | - | - | - | - | - | - | - | - | - | - | - | - | - | - | - | - | - | - | - | - | - | - | - | - | - | - | -137 |
| G0QS43_ICHMG | - | - | - | - | - | - | - | - | - | - | - | - | - | - | - | - | - | - | - | - | - | - | - | - | - | - | - | - | - | - | - | - | - | - | - | - | - | - | - | - | - | - | - | - | - | - | - | - | - | - | - | - | - | - | - | - | - | - | - | - | - | -145 |
| D8LB20_ECTSI | - | - | - | - | - | - | - | - | - | - | - | - | - | - | - | - | - | - | - | - | - | - | - | - | - | - | - | - | - | - | - | - | - | - | - | - | - | - | - | - | - | - | - | - | - | - | - | - | - | - | - | - | - | - | - | - | - | - | - | - | - | -183 |
| Q9U0T9_LEIMA | - | - | - | - | - | - | - | - | - | - | - | - | - | - | - | - | - | - | - | - | - | - | - | - | - | - | - | - | - | - | - | - | - | - | - | - | - | - | - | - | - | - | - | - | - | - | - | - | - | - | - | - | - | - | - | - | - | - | - | - | - | -156 |
| Q4CXJ8_TRYCC | - | - | - | - | - | - | - | - | - | - | - | - | - | - | - | - | - | - | - | - | - | - | - | - | - | - | - | - | - | - | - | - | - | - | - | - | - | - | - | - | - | - | - | - | - | - | - | - | - | - | - | - | - | - | - | - | - | - | - | - | - | -156 |
| F4I0A4_ARATH | - | - | - | - | - | - | - | - | - | - | - | - | - | - | - | - | - | - | - | - | - | - | - | - | - | - | - | - | - | - | - | - | - | - | - | - | - | - | - | - | - | - | - | - | - | - | - | - | - | - | - | - | - | - | - | - | - | - | - | - | - | -154 |
| Q8RUQ1_MAIZE | - | - | - | - | - | - | - | - | - | - | - | - | - | - | - | - | - | - | - | - | - | - | - | - | - | - | - | - | - | - | - | - | - | - | - | - | - | - | - | - | - | - | - | - | - | - | - | - | - | - | - | - | - | - | - | - | - | - | - | - | - | -154 |
| D7FQ17_ECTSI | - | - | - | - | - | - | - | - | - | - | - | - | - | - | - | - | - | - | - | - | - | - | - | - | - | - | - | - | - | - | - | - | - | - | - | - | - | - | - | - | - | - | - | - | - | - | - | - | - | - | - | - | - | - | - | - | - | - | - | - | - | -171 |
| D0MZ44_PHYIT | - | - | - | - | - | - | - | - | - | - | - | - | - | - | - | - | - | - | - | - | - | - | - | - | - | - | - | - | - | - | - | - | - | - | - | - | - | - | - | - | - | - | - | - | - | - | - | - | - | - | - | - | - | - | - | - | - | - | - | - | - | -172 |
| L1JBM0_GUITH | - | - | - | - | - | - | - | - | - | - | - | - | - | - | - | - | - | - | - | - | - | - | - | - | - | - | - | - | - | - | - | - | - | - | - | - | - | - | - | - | - | - | - | - | - | - | - | - | - | - | - | - | - | - | - | - | - | - | - | - | - | -127 |
| L1J739_GUITH | - | - | - | - | - | - | - | - | - | - | - | - | - | - | - | - | - | - | - | - | - | - | - | - | - | - | - | - | - | - | - | - | - | - | - | - | - | - | - | - | - | - | - | - | - | - | - | - | - | - | - | - | - | - | - | - | - | - | - | - | - | -160 |
| L1J0U9_GUITH | - | - | - | - | - | - | - | - | - | - | - | - | - | - | - | - | - | - | - | - | - | - | - | - | - | - | - | - | - | - | - | - | - | - | - | - | - | - | - | - | - | - | - | - | - | - | - | - | - | - | - | - | - | - | - | - | - | - | - | - | - | -153 |
| L1J1Q4_GUITH | - | - | - | - | - | - | - | - | - | - | - | - | - | - | - | - | - | - | - | - | - | - | - | - | - | - | - | - | - | - | - | - | - | - | - | - | - | - | - | - | - | - | - | - | - | - | - | - | - | - | - | - | - | - | - | - | - | - | - | - | - | -171 |
| D0ND88_PHYIT | - | - | - | - | - | - | - | - | - | - | - | - | - | - | - | - | - | - | - | - | - | - | - | - | - | - | - | - | - | - | - | - | - | - | - | - | - | - | - | - | - | - | - | - | - | - | - | - | - | - | - | - | - | - | - | - | - | - | - | - | - | -191 |
| D2VVS6_NAEGR | - | - | - | - | - | - | - | - | - | - | - | - | - | - | - | - | - | - | - | - | - | - | - | - | - | - | - | - | - | - | - | - | - | - | - | - | - | - | - | - | - | - | - | - | - | - | - | - | - | - | - | - | - | - | - | - | - | - | - | - | - | -152 |
| D2V3V2_NAEGR | - | - | - | - | - | - | - | - | - | - | - | - | - | - | - | - | - | - | - | - | - | - | - | - | - | - | - | - | - | - | - | - | - | - | - | - | - | - | - | - | - | - | - | - | - | - | - | - | - | - | - | - | - | - | - | - | - | - | - | - | - | -153 |
| Q5B5J7_EMENI | - | - | - | - | - | - | - | - | - | - | - | - | - | - | - | - | - | - | - | - | - | - | - | - | - | - | - | - | - | - | - | - | - | - | - | - | - | - | - | - | - | - | - | - | - | - | - | - | - | - | - | - | - | - | - | - | - | - | - | - | - | -171 |
| E4ZY10_LEPMJ | - | - | - | - | - | - | - | - | - | - | - | - | - | - | - | - | - | - | - | - | - | - | - | - | - | - | - | - | - | - | - | - | - | - | - | - | - | - | - | - | - | - | - | - | - | - | - | - | - | - | - | - | - | - | - | - | - | - | - | - | - | -177 |
| A8PIE5_COPC7 | - | - | - | - | - | - | - | - | - | - | - | - | - | - | - | - | - | - | - | - | - | - | - | - | - | - | - | - | - | - | - | - | - | - | - | - | - | - | - | - | - | - | - | - | - | - | - | - | - | - | - | - | - | - | - | - | - | - | - | - | - | -172 |
| A8NBA6_COPC7 | - | - | - | - | - | - | - | - | - | - | - | - | - | - | - | - | - | - | - | - | - | - | - | - | - | - | - | - | - | - | - | - | - | - | - | - | - | - | - | - | - | - | - | - | - | - | - | - | - | - | - | - | - | - | - | - | - | - | - | - | - | -171 |
| A8NBA1_COPC7 | - | - | - | - | - | - | - | - | - | - | - | - | - | - | - | - | - | - | - | - | - | - | - | - | - | - | - | - | - | - | - | - | - | - | - | - | - | - | - | - | - | - | - | - | - | - | - | - | - | - | - | - | - | - | - | - | - | - | - | - | - | -175 |
| A8NBA2_COPC7 | - | - | - | - | - | - | - | - | - | - | - | - | - | - | - | - | - | - | - | - | - | - | - | - | - | - | - | - | - | - | - | - | - | - | - | - | - | - | - | - | - | - | - | - | - | - | - | - | - | - | - | - | - | - | - | - | - | - | - | - | - | -175 |
| A8NAE6_COPC7 | - | - | - | - | - | - | - | - | - | - | - | - | - | - | - | - | - | - | - | - | - | - | - | - | - | - | - | - | - | - | - | - | - | - | - | - | - | - | - | - | - | - | - | - | - | - | - | - | - | - | - | - | - | - | - | - | - | - | - | - | - | -193 |
| A8NAI9_COPC7 | - | - | - | - | - | - | - | - | - | - | - | - | - | - | - | - | - | - | - | - | - | - | - | - | - | - | - | - | - | - | - | - | - | - | - | - | - | - | - | - | - | - | - | - | - | - | - | - | - | - | - | - | - | - | - | - | - | - | - | - | - | -171 |
| G4TER7_PIRID | - | - | - | - | - | - | - | - | - | - | - | - | - | - | - | - | - | - | - | - | - | - | - | - | - | - | - | - | - | - | - | - | - | - | - | - | - | - | - | - | - | - | - | - | - | - | - | - | - | - | - | - | - | - | - | - | - | - | - | - | - | -171 |
| G4TER6_PIRID | - | - | - | - | - | - | - | - | - | - | - | - | - | - | - | - | - | - | - | - | - | - | - | - | - | - | - | - | - | - | - | - | - | - | - | - | - | - | - | - | - | - | - | - | - | - | - | - | - | - | - | - | - | - | - | - | - | - | - | - | - | -161 |
| G4TNA1_PIRID | - | - | - | - | - | - | - | - | - | - | - | - | - | - | - | - | - | - | - | - | - | - | - | - | - | - | - | - | - | - | - | - | - | - | - | - | - | - | - | - | - | - | - | - | - | - | - | - | - | - | - | - | - | - | - | - | - | - | - | - | - | -171 |
| D0MWB9_PHYIT | - | - | - | - | - | - | - | - | - | - | - | - | - | - | - | - | - | - | - | - | - | - | - | - | - | - | - | - | - | - | - | - | - | - | - | - | - | - | - | - | - | - | - | - | - | - | - | - | - | - | - | - | - | - | - | - | - | - | - | - | - | -161 |
| L1I9H5_GUITH | - | - | - | - | - | - | - | - | - | - | - | - | - | - | - | - | - | - | - | - | - | - | - | - | - | - | - | - | - | - | - | - | - | - | - | - | - | - | - | - | - | - | - | - | - | - | - | - | - | - | - | - | - | - | - | - | - | - | - | - | - | -163 |
| Q7N497_PHOLL | - | - | - | - | - | - | - | - | - | - | - | - | - | - | - | - | - | - | - | - | - | - | - | - | - | - | - | - | - | - | - | - | - | - | - | - | - | - | - | - | - | - | - | - | - | - | - | - | - | - | - | - | - | - | - | - | - | - | - | - | - | -132 |
| ZP_11152369 | - | - | - | - | - | - | - | - | - | - | - | - | - | - | - | - | - | - | - | - | - | - | - | - | - | - | - | - | - | - | - | - | - | - | - | - | - | - | - | - | - | - | - | - | - | - | - | - | - | - | - | - | - | - | - | - | - | - | - | - | - | -128 |
| A2FEL7_TRIVA | - | - | - | - | - | - | - | - | - | - | - | - | - | - | - | - | - | - | - | - | - | - | - | - | - | - | - | - | - | - | - | - | - | - | - | - | - | - | - | - | - | - | - | - | - | - | - | - | - | - | - | - | - | - | - | - | - | - | - | - | - | -150 |
| A2D9A8_TRIVA | - | - | - | - | - | - | - | - | - | - | - | - | - | - | - | - | - | - | - | - | - | - | - | - | - | - | - | - | - | - | - | - | - | - | - | - | - | - | - | - | - | - | - | - | - | - | - | - | - | - | - | - | - | - | - | - | - | - | - | - | - | -151 |
| A2FHB3_TRIVA | - | - | - | - | - | - | - | - | - | - | - | - | - | - | - | - | - | - | - | - | - | - | - | - | - | - | - | - | - | - | - | - | - | - | - | - | - | - | - | - | - | - | - | - | - | - | - | - | - | - | - | - | - | - | - | - | - | - | - | - | - | -152 |
| A2DTE8_TRIVA | - | - | - | - | - | - | - | - | - | - | - | - | - | - | - | - | - | - | - | - | - | - | - | - | - | - | - | - | - | - | - | - | - | - | - | - | - | - | - | - | - | - | - | - | - | - | - | - | - | - | - | - | - | - | - | - | - | - | - | - | - | -149 |
| A2E4X2_TRIVA | - | - | - | - | - | - | - | - | - | - | - | - | - | - | - | - | - | - | - | - | - | - | - | - | - | - | - | - | - | - | - | - | - | - | - | - | - | - | - | - | - | - | - | - | - | - | - | - | - | - | - | - | - | - | - | - | - | - | - | - | - | -154 |
| A2E1X1_TRIVA | - | - | - | - | - | - | - | - | - | - | - | - | - | - | - | - | - | - | - | - | - | - | - | - | - | - | - | - | - | - | - | - | - | - | - | - | - | - | - | - | - | - | - | - | - | - | - | - | - | - | - | - | - | - | - | - | - | - | - | - | - | -137 |
| TPR_PORGI | - | - | - | - | - | - | - | - | - | - | - | - | - | - | - | - | - | - | - | - | - | - | - | - | - | - | - | - | - | - | - | - | - | - | - | - | - | - | - | - | - | - | - | - | - | - | - | - | - | - | - | - | - | - | - | - | - | - | - | - | - | -170 |
| D5MF42_9BACT | - | - | - | - | - | - | - | - | - | - | - | - | - | - | - | - | - | - | - | - | - | - | - | - | - | - | - | - | - | - | - | - | - | - | - | - | - | - | - | - | - | - | - | - | - | - | - | - | - | - | - | - | - | - | - | - | - | - | - | - | - | -126 |
| R9H254_BACT4 | - | - | - | - | - | - | - | - | - | - | - | - | - | - | - | - | - | - | - | - | - | - | - | - | - | - | - | - | - | - | - | - | - | - | - | - | - | - | - | - | - | - | - | - | - | - | - | - | - | - | - | - | - | - | - | - | - | - | - | - | - | -126 |
| ZP_04847694 | - | - | - | - | - | - | - | - | - | - | - | - | - | - | - | - | - | - | - | - | - | - | - | - | - | - | - | - | - | - | - | - | - | - | - | - | - | - | - | - | - | - | - | - | - | - | - | - | - | - | - | - | - | - | - | - | - | - | - | - | - | -126 |
| D7IBA4_9BACE | - | - | - | - | - | - | - | - | - | - | - | - | - | - | - | - | - | - | - | - | - | - | - | - | - | - | - | - | - | - | - | - | - | - | - | - | - | - | - | - | - | - | - | - | - | - | - | - | - | - | - | - | - | - | - | - | - | - | - | - | - | -124 |
| ZP_09858577 | - | - | - | - | - | - | - | - | - | - | - | - | - | - | - | - | - | - | - | - | - | - | - | - | - | - | - | - | - | - | - | - | - | - | - | - | - | - | - | - | - | - | - | - | - | - | - | - | - | - | - | - | - | - | - | - | - | - | - | - | - | -124 |
| E5C759_9BACE | - | - | - | - | - | - | - | - | - | - | - | - | - | - | - | - | - | - | - | - | - | - | - | - | - | - | - | - | - | - | - | - | - | - | - | - | - | - | - | - | - | - | - | - | - | - | - | - | - | - | - | - | - | - | - | - | - | - | - | - | - | -126 |
| A7LTS0_BACO1 | - | - | - | - | - | - | - | - | - | - | - | - | - | - | - | - | - | - | - | - | - | - | - | - | - | - | - | - | - | - | - | - | - | - | - | - | - | - | - | - | - | - | - | - | - | - | - | - | - | - | - | - | - | - | - | - | - | - | - | - | - | -124 |
| Q8YQK8_NOSS1 | - | - | - | - | - | - | - | - | - | - | - | - | - | - | - | - | - | - | - | - | - | - | - | - | - | - | - | - | - | - | - | - | - | - | - | - | - | - | - | - | - | - | - | - | - | - | - | - | - | - | - | - | - | - | - | - | - | - | - | - | - | -138 |
| Q3MBX8_ANAVT | - | - | - | - | - | - | - | - | - | - | - | - | - | - | - | - | - | - | - | - | - | - | - | - | - | - | - | - | - | - | - | - | - | - | - | - | - | - | - | - | - | - | - | - | - | - | - | - | - | - | - | - | - | - | - | - | - | - | - | - | - | -143 |
| Q8YZT8_NOSS1 | - | - | - | - | - | - | - | - | - | - | - | - | - | - | - | - | - | - | - | - | - | - | - | - | - | - | - | - | - | - | - | - | - | - | - | - | - | - | - | - | - | - | - | - | - | - | - | - | - | - | - | - | - | - | - | - | - | - | - | - | - | -131 |
| K9UIG2_9CHRO | - | - | - | - | - | - | - | - | - | - | - | - | - | - | - | - | - | - | - | - | - | - | - | - | - | - | - | - | - | - | - | - | - | - | - | - | - | - | - | - | - | - | - | - | - | - | - | - | - | - | - | - | - | - | - | - | - | - | - | - | - | -138 |
| A4YRP8_BRASO | - | - | - | - | - | - | - | - | - | - | - | - | - | - | - | - | - | - | - | - | - | - | - | - | - | - | - | - | - | - | - | - | - | - | - | - | - | - | - | - | - | - | - | - | - | - | - | - | - | - | - | - | - | - | - | - | - | - | - | - | - | -152 |
| H0TAL7_9BRAD | - | - | - | - | - | - | - | - | - | - | - | - | - | - | - | - | - | - | - | - | - | - | - | - | - | - | - | - | - | - | - | - | - | - | - | - | - | - | - | - | - | - | - | - | - | - | - | - | - | - | - | - | - | - | - | - | - | - | - | - | - | -156 |
| H0SNL4_9BRAD | - | - | - | - | - | - | - | - | - | - | - | - | - | - | - | - | - | - | - | - | - | - | - | - | - | - | - | - | - | - | - | - | - | - | - | - | - | - | - | - | - | - | - | - | - | - | - | - | - | - | - | - | - | - | - | - | - | - | - | - | - | -158 |
| H0S4P6_9BRAD | - | - | - | - | - | - | - | - | - | - | - | - | - | - | - | - | - | - | - | - | - | - | - | - | - | - | - | - | - | - | - | - | - | - | - | - | - | - | - | - | - | - | - | - | - | - | - | - | - | - | - | - | - | - | - | - | - | - | - | - | - | -158 |
| A5EMK2_BRASB | - | - | - | - | - | - | - | - | - | - | - | - | - | - | - | - | - | - | - | - | - | - | - | - | - | - | - | - | - | - | - | - | - | - | - | - | - | - | - | - | - | - | - | - | - | - | - | - | - | - | - | - | - | - | - | - | - | - | - | - | - | -152 |
| M4ZBJ4_9BRAD | - | - | - | - | - | - | - | - | - | - | - | - | - | - | - | - | - | - | - | - | - | - | - | - | - | - | - | - | - | - | - | - | - | - | - | - | - | - | - | - | - | - | - | - | - | - | - | - | - | - | - | - | - | - | - | - | - | - | - | - | - | -158 |
| Q07SP7_RHOP5 | - | - | - | - | - | - | - | - | - | - | - | - | - | - | - | - | - | - | - | - | - | - | - | - | - | - | - | - | - | - | - | - | - | - | - | - | - | - | - | - | - | - | - | - | - | - | - | - | - | - | - | - | - | - | - | - | - | - | - | - | - | -145 |
| B0JUQ7_MICAN | - | - | - | - | - | - | - | - | - | - | - | - | - | - | - | - | - | - | - | - | - | - | - | - | - | - | - | - | - | - | - | - | - | - | - | - | - | - | - | - | - | - | - | - | - | - | - | - | - | - | - | - | - | - | - | - | - | - | - | - | - | -147 |
| K9WJ87_9CYAN | - | - | - | - | - | - | - | - | - | - | - | - | - | - | - | - | - | - | - | - | - | - | - | - | - | - | - | - | - | - | - | - | - | - | - | - | - | - | - | - | - | - | - | - | - | - | - | - | - | - | - | - | - | - | - | - | - | - | - | - | - | -141 |
| G6FUE0_9CYAN | - | - | - | - | - | - | - | - | - | - | - | - | - | - | - | - | - | - | - | - | - | - | - | - | - | - | - | - | - | - | - | - | - | - | - | - | - | - | - | - | - | - | - | - | - | - | - | - | - | - | - | - | - | - | - | - | - | - | - | - | - | -141 |
| K9PHG5_9CYAN | - | - | - | - | - | - | - | - | - | - | - | - | - | - | - | - | - | - | - | - | - | - | - | - | - | - | - | - | - | - | - | - | - | - | - | - | - | - | - | - | - | - | - | - | - | - | - | - | - | - | - | - | - | - | - | - | - | - | - | - | - | -143 |
| Q8YXX3_NOSS1 | - | - | - | - | - | - | - | - | - | - | - | - | - | - | - | - | - | - | - | - | - | - | - | - | - | - | - | - | - | - | - | - | - | - | - | - | - | - | - | - | - | - | - | - | - | - | - | - | - | - | - | - | - | - | - | - | - | - | - | - | - | -156 |
| K9TN70_9CYAN | - | - | - | - | - | - | - | - | - | - | - | - | - | - | - | - | - | - | - | - | - | - | - | - | - | - | - | - | - | - | - | - | - | - | - | - | - | - | - | - | - | - | - | - | - | - | - | - | - | - | - | - | - | - | - | - | - | - | - | - | - | -156 |
| Q05QW3_9SYNE | - | - | - | - | - | - | - | - | - | - | - | - | - | - | - | - | - | - | - | - | - | - | - | - | - | - | - | - | - | - | - | - | - | - | - | - | - | - | - | - | - | - | - | - | - | - | - | - | - | - | - | - | - | - | - | - | - | - | - | - | - | -162 |
| Q05QW2_9SYNE | - | - | - | - | - | - | - | - | - | - | - | - | - | - | - | - | - | - | - | - | - | - | - | - | - | - | - | - | - | - | - | - | - | - | - | - | - | - | - | - | - | - | - | - | - | - | - | - | - | - | - | - | - | - | - | - | - | - | - | - | - | -159 |
| Q05QW4_9SYNE | - | - | - | - | - | - | - | - | - | - | - | - | - | - | - | - | - | - | - | - | - | - | - | - | - | - | - | - | - | - | - | - | - | - | - | - | - | - | - | - | - | - | - | - | - | - | - | - | - | - | - | - | - | - | - | - | - | - | - | - | - | -153 |
| E0UGX6_CYAP2 | - | - | - | - | - | - | - | - | - | - | - | - | - | - | - | - | - | - | - | - | - | - | - | - | - | - | - | - | - | - | - | - | - | - | - | - | - | - | - | - | - | - | - | - | - | - | - | - | - | - | - | - | - | - | - | - | - | - | - | - | - | -144 |
| K7WKF4_9NOST | - | - | - | - | - | - | - | - | - | - | - | - | - | - | - | - | - | - | - | - | - | - | - | - | - | - | - | - | - | - | - | - | - | - | - | - | - | - | - | - | - | - | - | - | - | - | - | - | - | - | - | - | - | - | - | - | - | - | - | - | - | -143 |
| E0UGY5_CYAP2 | - | - | - | - | - | - | - | - | - | - | - | - | - | - | - | - | - | - | - | - | - | - | - | - | - | - | - | - | - | - | - | - | - | - | - | - | - | - | - | - | - | - | - | - | - | - | - | - | - | - | - | - | - | - | - | - | - | - | - | - | - | -146 |
| A8LCE5_FRASN | - | - | - | - | - | - | - | - | - | - | - | - | - | - | - | - | - | - | - | - | - | - | - | - | - | - | - | - | - | - | - | - | - | - | - | - | - | - | - | - | - | - | - | - | - | - | - | - | - | - | - | - | - | - | - | - | - | - | - | - | - | -137 |
| D3CT47_9ACTO | - | - | - | - | - | - | - | - | - | - | - | - | - | - | - | - | - | - | - | - | - | - | - | - | - | - | - | - | - | - | - | - | - | - | - | - | - | - | - | - | - | - | - | - | - | - | - | - | - | - | - | - | - | - | - | - | - | - | - | - | - | -141 |
| Q2J4J6_FRASC | - | - | - | - | - | - | - | - | - | - | - | - | - | - | - | - | - | - | - | - | - | - | - | - | - | - | - | - | - | - | - | - | - | - | - | - | - | - | - | - | - | - | - | - | - | - | - | - | - | - | - | - | - | - | - | - | - | - | - | - | - | -162 |
| Q0RAZ2_FRAAA | - | - | - | - | - | - | - | - | - | - | - | - | - | - | - | - | - | - | - | - | - | - | - | - | - | - | - | - | - | - | - | - | - | - | - | - | - | - | - | - | - | - | - | - | - | - | - | - | - | - | - | - | - | - | - | - | - | - | - | - | - | -149 |
| E3J3F6_FRASU | - | - | - | - | - | - | - | - | - | - | - | - | - | - | - | - | - | - | - | - | - | - | - | - | - | - | - | - | - | - | - | - | - | - | - | - | - | - | - | - | - | - | - | - | - | - | - | - | - | - | - | - | - | - | - | - | - | - | - | - | - | -143 |
| ZP_09168240 | - | - | - | - | - | - | - | - | - | - | - | - | - | - | - | - | - | - | - | - | - | - | - | - | - | - | - | - | - | - | - | - | - | - | - | - | - | - | - | - | - | - | - | - | - | - | - | - | - | - | - | - | - | - | - | - | - | - | - | - | - | -153 |
| C7MDV9_BRAFD | - | - | - | - | - | - | - | - | - | - | - | - | - | - | - | - | - | - | - | - | - | - | - | - | - | - | - | - | - | - | - | - | - | - | - | - | - | - | - | - | - | - | - | - | - | - | - | - | - | - | - | - | - | - | - | - | - | - | - | - | - | -115 |
| ZP_10094989 | - | - | - | - | - | - | - | - | - | - | - | - | - | - | - | - | - | - | - | - | - | - | - | - | - | - | - | - | - | - | - | - | - | - | - | - | - | - | - | - | - | - | - | - | - | - | - | - | - | - | - | - | - | - | - | - | - | - | - | - | - | -115 |
| A4X268_SALTO | - | - | - | - | - | - | - | - | - | - | - | - | - | - | - | - | - | - | - | - | - | - | - | - | - | - | - | - | - | - | - | - | - | - | - | - | - | - | - | - | - | - | - | - | - | - | - | - | - | - | - | - | - | - | - | - | - | - | - | - | - | -165 |
| WP_043162213 | - | - | - | - | - | - | - | - | - | - | - | - | - | - | - | - | - | - | - | - | - | - | - | - | - | - | - | - | - | - | - | - | - | - | - | - | - | - | - | - | - | - | - | - | - | - | - | - | - | - | - | - | - | - | - | - | - | - | - | - | - | -165 |
| Q0BRV8_GRABC | - | - | - | - | - | - | - | - | - | - | - | - | - | - | - | - | - | - | - | - | - | - | - | - | - | - | - | - | - | - | - | - | - | - | - | - | - | - | - | - | - | - | - | - | - | - | - | - | - | - | - | - | - | - | - | - | - | - | - | - | - | -141 |
| A9B2P0_HERA2 | - | - | - | - | - | - | - | - | - | - | - | - | - | - | - | - | - | - | - | - | - | - | - | - | - | - | - | - | - | - | - | - | - | - | - | - | - | - | - | - | - | - | - | - | - | - | - | - | - | - | - | - | - | - | - | - | - | - | - | - | - | -133 |
| A9B3U8_HERA2 | - | - | - | - | - | - | - | - | - | - | - | - | - | - | - | - | - | - | - | - | - | - | - | - | - | - | - | - | - | - | - | - | - | - | - | - | - | - | - | - | - | - | - | - | - | - | - | - | - | - | - | - | - | - | - | - | - | - | - | - | - | -135 |
| A9B2A3_HERA2 | - | - | - | - | - | - | - | - | - | - | - | - | - | - | - | - | - | - | - | - | - | - | - | - | - | - | - | - | - | - | - | - | - | - | - | - | - | - | - | - | - | - | - | - | - | - | - | - | - | - | - | - | - | - | - | - | - | - | - | - | - | -153 |
| D6ZC85_SEGRD | - | - | - | - | - | - | - | - | - | - | - | - | - | - | - | - | - | - | - | - | - | - | - | - | - | - | - | - | - | - | - | - | - | - | - | - | - | - | - | - | - | - | - | - | - | - | - | - | - | - | - | - | - | - | - | - | - | - | - | - | - | -179 |
| E5XTT9_9ACTO | - | - | - | - | - | - | - | - | - | - | - | - | - | - | - | - | - | - | - | - | - | - | - | - | - | - | - | - | - | - | - | - | - | - | - | - | - | - | - | - | - | - | - | - | - | - | - | - | - | - | - | - | - | - | - | - | - | - | - | - | - | -179 |
| E5XTU0_9ACTO | - | - | - | - | - | - | - | - | - | - | - | - | - | - | - | - | - | - | - | - | - | - | - | - | - | - | - | - | - | - | - | - | - | - | - | - | - | - | - | - | - | - | - | - | - | - | - | - | - | - | - | - | - | - | - | - | - | - | - | - | - | -183 |
| D6B0I6_9ACTO | - | - | - | - | - | - | - | - | - | - | - | - | - | - | - | - | - | - | - | - | - | - | - | - | - | - | - | - | - | - | - | - | - | - | - | - | - | - | - | - | - | - | - | - | - | - | - | - | - | - | - | - | - | - | - | - | - | - | - | - | - | -141 |
| D9X719_STRVR | - | - | - | - | - | - | - | - | - | - | - | - | - | - | - | - | - | - | - | - | - | - | - | - | - | - | - | - | - | - | - | - | - | - | - | - | - | - | - | - | - | - | - | - | - | - | - | - | - | - | - | - | - | - | - | - | - | - | - | - | - | -141 |
| H6L010_SAPGL | - | - | - | - | - | - | - | - | - | - | - | - | - | - | - | - | - | - | - | - | - | - | - | - | - | - | - | - | - | - | - | - | - | - | - | - | - | - | - | - | - | - | - | - | - | - | - | - | - | - | - | - | - | - | - | - | - | - | - | - | - | -127 |
| K1ZP83_9BACT | - | - | - | - | - | - | - | - | - | - | - | - | - | - | - | - | - | - | - | - | - | - | - | - | - | - | - | - | - | - | - | - | - | - | - | - | - | - | - | - | - | - | - | - | - | - | - | - | - | - | - | - | - | - | - | - | - | - | - | - | - | -148 |
| ADGB_HUMAN | - | - | - | - | - | - | - | - | - | - | - | - | - | - | - | - | - | - | - | - | - | - | - | - | - | - | - | - | - | - | - | - | - | - | - | - | - | - | - | - | - | - | - | - | - | - | - | - | - | - | - | - | - | - | - | - | - | - | - | - | - | -186 |
| J3QMP3_MOUSE | - | - | - | - | - | - | - | - | - | - | - | - | - | - | - | - | - | - | - | - | - | - | - | - | - | - | - | - | - | - | - | - | - | - | - | - | - | - | - | - | - | - | - | - | - | - | - | - | - | - | - | - | - | - | - | - | - | - | - | - | - | -186 |
| H2XR46_CIOIN | - | - | - | - | - | - | - | - | - | - | - | - | - | - | - | - | - | - | - | - | - | - | - | - | - | - | - | - | - | - | - | - | - | - | - | - | - | - | - | - | - | - | - | - | - | - | - | - | - | - | - | - | - | - | - | - | - | - | - | - | - | -184 |
| CAN7_MOUSE | - | - | - | - | - | - | - | - | - | - | - | - | - | - | - | - | - | - | - | - | - | - | - | - | - | - | - | - | - | - | - | - | - | - | - | - | - | - | - | - | - | - | - | - | - | - | - | - | - | - | - | - | - | - | - | - | - | - | - | - | - | -156 |
| CAN7_HUMAN | - | - | - | - | - | - | - | - | - | - | - | - | - | - | - | - | - | - | - | - | - | - | - | - | - | - | - | - | - | - | - | - | - | - | - | - | - | - | - | - | - | - | - | - | - | - | - | - | - | - | - | - | - | - | - | - | - | - | - | - | - | -156 |
| E1BY84_CHICK | - | - | - | - | - | - | - | - | - | - | - | - | - | - | - | - | - | - | - | - | - | - | - | - | - | - | - | - | - | - | - | - | - | - | - | - | - | - | - | - | - | - | - | - | - | - | - | - | - | - | - | - | - | - | - | - | - | - | - | - | - | -156 |
| H9GX91_DANRE | - | - | - | - | - | - | - | - | - | - | - | - | - | - | - | - | - | - | - | - | - | - | - | - | - | - | - | - | - | - | - | - | - | - | - | - | - | - | - | - | - | - | - | - | - | - | - | - | - | - | - | - | - | - | - | - | - | - | - | - | - | -156 |
| F6SGI6_CIOIN | - | - | - | - | - | - | - | - | - | - | - | - | - | - | - | - | - | - | - | - | - | - | - | - | - | - | - | - | - | - | - | - | - | - | - | - | - | - | - | - | - | - | - | - | - | - | - | - | - | - | - | - | - | - | - | - | - | - | - | - | - | -154 |
| W4Y195_STRPU | - | - | - | - | - | - | - | - | - | - | - | - | - | - | - | - | - | - | - | - | - | - | - | - | - | - | - | - | - | - | - | - | - | - | - | - | - | - | - | - | - | - | - | - | - | - | - | - | - | - | - | - | - | - | - | - | - | - | - | - | - | -130 |
| R7V270_CAPTE | - | - | - | - | - | - | - | - | - | - | - | - | - | - | - | - | - | - | - | - | - | - | - | - | - | - | - | - | - | - | - | - | - | - | - | - | - | - | - | - | - | - | - | - | - | - | - | - | - | - | - | - | - | - | - | - | - | - | - | - | - | -155 |
| G4LXK3_SCHMA | - | - | - | - | - | - | - | - | - | - | - | - | - | - | - | - | - | - | - | - | - | - | - | - | - | - | - | - | - | - | - | - | - | - | - | - | - | - | - | - | - | - | - | - | - | - | - | - | - | - | - | - | - | - | - | - | - | - | - | - | - | -156 |
| T2M2K0_HYDVU | - | - | - | - | - | - | - | - | - | - | - | - | - | - | - | - | - | - | - | - | - | - | - | - | - | - | - | - | - | - | - | - | - | - | - | - | - | - | - | - | - | - | - | - | - | - | - | - | - | - | - | - | - | - | - | - | - | - | - | - | - | -156 |
| B3RQK1_TRIAD | - | - | - | - | - | - | - | - | - | - | - | - | - | - | - | - | - | - | - | - | - | - | - | - | - | - | - | - | - | - | - | - | - | - | - | - | - | - | - | - | - | - | - | - | - | - | - | - | - | - | - | - | - | - | - | - | - | - | - | - | - | -155 |
| I1CS59_RHIO9 | - | - | - | - | - | - | - | - | - | - | - | - | - | - | - | - | - | - | - | - | - | - | - | - | - | - | - | - | - | - | - | - | - | - | - | - | - | - | - | - | - | - | - | - | - | - | - | - | - | - | - | - | - | - | - | - | - | - | - | - | - | -151 |
| I1GER3_AMPQE | - | - | - | - | - | - | - | - | - | - | - | - | - | - | - | - | - | - | - | - | - | - | - | - | - | - | - | - | - | - | - | - | - | - | - | - | - | - | - | - | - | - | - | - | - | - | - | - | - | - | - | - | - | - | - | - | - | - | - | - | - | -154 |
| Q22143_CAEEL | - | - | - | - | - | - | - | - | - | - | - | - | - | - | - | - | - | - | - | - | - | - | - | - | - | - | - | - | - | - | - | - | - | - | - | - | - | - | - | - | - | - | - | - | - | - | - | - | - | - | - | - | - | - | - | - | - | - | - | - | - | -155 |
| L1JZN7_GUITH | - | - | - | - | - | - | - | - | - | - | - | - | - | - | - | - | - | - | - | - | - | - | - | - | - | - | - | - | - | - | - | - | - | - | - | - | - | - | - | - | - | - | - | - | - | - | - | - | - | - | - | - | - | - | - | - | - | - | - | - | - | -158 |
| D0NT34_PHYIT | - | - | - | - | - | - | - | - | - | - | - | - | - | - | - | - | - | - | - | - | - | - | - | - | - | - | - | - | - | - | - | - | - | - | - | - | - | - | - | - | - | - | - | - | - | - | - | - | - | - | - | - | - | - | - | - | - | - | - | - | - | -166 |
| A0A064B913_ASPOZ | - | - | - | - | - | - | - | - | - | - | - | - | - | - | - | - | - | - | - | - | - | - | - | - | - | - | - | - | - | - | - | - | - | - | - | - | - | - | - | - | - | - | - | - | - | - | - | - | - | - | - | - | - | - | - | - | - | - | - | - | - | -164 |
| A8N4X4_COPC7 | - | - | - | - | - | - | - | - | - | - | - | - | - | - | - | - | - | - | - | - | - | - | - | - | - | - | - | - | - | - | - | - | - | - | - | - | - | - | - | - | - | - | - | - | - | - | - | - | - | - | - | - | - | - | - | - | - | - | - | - | - | -180 |
| C0H5K1_PLAF7 | - | V | M | I | N | K | K | H | G | Y | V | N | S | V | E | F | V | E | D | T | K | Y | N | H | N | N | N | N | N | N | N | K | N | N | T | D | H | D | Q | Y | M | C | D | D | N | I | C | F | N | N | Y | N | F | C | Y | K | D | D | Y | E | V | -252 |
| K1REZ8_CRAGI | - | - | - | - | - | - | - | - | - | - | - | - | - | - | - | - | - | - | - | - | - | - | - | - | - | - | - | - | - | - | - | - | - | - | - | - | - | - | - | - | - | - | - | - | - | - | - | - | - | - | - | - | - | - | - | - | - | - | - | - | - | -145 |
| F4NTM9_BATDJ | - | - | - | - | - | - | - | - | - | - | - | - | - | - | - | - | - | - | - | - | - | - | - | - | - | - | - | - | - | - | - | - | - | - | - | - | - | - | - | - | - | - | - | - | - | - | - | - | - | - | - | - | - | - | - | - | - | - | - | - | - | -167 |
| Q6F6K5_CAEEL | - | - | - | - | - | - | - | - | - | - | - | - | - | - | - | - | - | - | - | - | - | - | - | - | - | - | - | - | - | - | - | - | - | - | - | - | - | - | - | - | - | - | - | - | - | - | - | - | - | - | - | - | - | - | - | - | - | - | - | - | - | -126 |
| A8HZK8_CHLRE | - | - | - | - | - | - | - | - | - | - | - | - | - | - | - | - | - | - | - | - | - | - | - | - | - | - | - | - | - | - | - | - | - | - | - | - | - | - | - | - | - | - | - | - | - | - | - | - | - | - | - | - | - | - | - | - | - | - | - | - | - | -132 |
| A8J6T6_CHLRE | - | - | - | - | - | - | - | - | - | - | - | - | - | - | - | - | - | - | - | - | - | - | - | - | - | - | - | - | - | - | - | - | - | - | - | - | - | - | - | - | - | - | - | - | - | - | - | - | - | - | - | - | - | - | - | - | - | - | - | - | - | -184 |
| Z9JR51_9MICO | - | - | - | - | - | - | - | - | - | - | - | - | - | - | - | - | - | - | - | - | - | - | - | - | - | - | - | - | - | - | - | - | - | - | - | - | - | - | - | - | - | - | - | - | - | - | - | - | - | - | - | - | - | - | - | - | - | - | - | - | - | -144 |
| F7ZZH9_CELGA | - | - | - | - | - | - | - | - | - | - | - | - | - | - | - | - | - | - | - | - | - | - | - | - | - | - | - | - | - | - | - | - | - | - | - | - | - | - | - | - | - | - | - | - | - | - | - | - | - | - | - | - | - | - | - | - | - | - | - | - | - | -126 |
| Q7NDU2_GLOVI | - | - | - | - | - | - | - | - | - | - | - | - | - | - | - | - | - | - | - | - | - | - | - | - | - | - | - | - | - | - | - | - | - | - | - | - | - | - | - | - | - | - | - | - | - | - | - | - | - | - | - | - | - | - | - | - | - | - | - | - | - | -115 |
| I6AT95_9BACT | - | - | - | - | - | - | - | - | - | - | - | - | - | - | - | - | - | - | - | - | - | - | - | - | - | - | - | - | - | - | - | - | - | - | - | - | - | - | - | - | - | - | - | - | - | - | - | - | - | - | - | - | - | - | - | - | - | - | - | - | - | -139 |
| C7MGJ8_BRAFD | - | - | - | - | - | - | - | - | - | - | - | - | - | - | - | - | - | - | - | - | - | - | - | - | - | - | - | - | - | - | - | - | - | - | - | - | - | - | - | - | - | - | - | - | - | - | - | - | - | - | - | - | - | - | - | - | - | - | - | - | - | -142 |
| D0WP73_9ACTO | - | - | - | - | - | - | - | - | - | - | - | - | - | - | - | - | - | - | - | - | - | - | - | - | - | - | - | - | - | - | - | - | - | - | - | - | - | - | - | - | - | - | - | - | - | - | - | - | - | - | - | - | - | - | - | - | - | - | - | - | - | -141 |
| B0C8L1_ACAM1 | - | - | - | - | - | - | - | - | - | - | - | - | - | - | - | - | - | - | - | - | - | - | - | - | - | - | - | - | - | - | - | - | - | - | - | - | - | - | - | - | - | - | - | - | - | - | - | - | - | - | - | - | - | - | - | - | - | - | - | - | - | -132 |
| B3E5A8_GEOLS | - | - | - | - | - | - | - | - | - | - | - | - | - | - | - | - | - | - | - | - | - | - | - | - | - | - | - | - | - | - | - | - | - | - | - | - | - | - | - | - | - | - | - | - | - | - | - | - | - | - | - | - | - | - | - | - | - | - | - | - | - | -135 |
| D4YQ94_9MICO | - | - | - | - | - | - | - | - | - | - | - | - | - | - | - | - | - | - | - | - | - | - | - | - | - | - | - | - | - | - | - | - | - | - | - | - | - | - | - | - | - | - | - | - | - | - | - | - | - | - | - | - | - | - | - | - | - | - | - | - | - | -125 |
| PAPA_CARPA | - | - | - | - | - | - | - | - | - | - | - | - | - | - | - | - | - | - | - | - | - | - | - | - | - | - | - | - | - | - | - | - | - | - | - | - | - | - | - | - | - | - | - | - | - | - | - | - | - | - | - | - | - | - | - | - | - | - | - | - | - | -119 |
| RIM13_YEAST | - | - | - | - | - | - | - | - | - | - | - | - | - | - | - | - | - | - | - | - | - | - | - | - | - | - | - | - | - | - | - | - | - | - | - | - | - | - | - | - | - | - | - | - | - | - | - | - | - | - | - | - | - | - | - | - | - | - | - | - | - | -154 |

| I |  |  |  |  |  |  |  |  |  |  |  |  |  |  |  |  |  |  |  |  |  |  |  |  |  |  |  |  |  |  |  |  |  |  |  |  |  |  |  |  |  |  |  |  |  |  |  |  |  |  |  |  |  |  |  |  |  |  |  |  |  |  |
| --- | --- | --- | --- | --- | --- | --- | --- | --- | --- | --- | --- | --- | --- | --- | --- | --- | --- | --- | --- | --- | --- | --- | --- | --- | --- | --- | --- | --- | --- | --- | --- | --- | --- | --- | --- | --- | --- | --- | --- | --- | --- | --- | --- | --- | --- | --- | --- | --- | --- | --- | --- | --- | --- | --- | --- | --- | --- | --- | --- | --- | --- | --- |
| Q9N4B1_CAEEL | - | - | - | - | - | - | - | - | - | - | - | - | - | - | - | - | - | - | - | - | - | - | - | - | - | - | - | - | - | - | - | - | - | - | - | - | - | - | - | - | - | - | - | - | - | - | - | - | - | - | - | - | - | - | - | - | - | - | - | - | - | -149 |
| Q8IAA8_CAEEL | - | - | - | - | - | - | - | - | - | - | - | - | - | - | - | - | - | - | - | - | - | - | - | - | - | - | - | - | - | - | - | - | - | - | - | - | - | - | - | - | - | - | - | - | - | - | - | - | - | - | - | - | - | - | - | - | - | - | - | - | - | -149 |
| CAN_CAEEL | - | - | - | - | - | - | - | - | - | - | - | - | - | - | - | - | - | - | - | - | - | - | - | - | - | - | - | - | - | - | - | - | - | - | - | - | - | - | - | - | - | - | - | - | - | - | - | - | - | - | - | - | - | - | - | - | - | - | - | - | - | -148 |
| Q9U2B2_CAEEL | - | - | - | - | - | - | - | - | - | - | - | - | - | - | - | - | - | - | - | - | - | - | - | - | - | - | - | - | - | - | - | - | - | - | - | - | - | - | - | - | - | - | - | - | - | - | - | - | - | - | - | - | - | - | - | - | - | - | - | - | - | -153 |
| Q9N4B2_CAEEL | - | - | - | - | - | - | - | - | - | - | - | - | - | - | - | - | - | - | - | - | - | - | - | - | - | - | - | - | - | - | - | - | - | - | - | - | - | - | - | - | - | - | - | - | - | - | - | - | - | - | - | - | - | - | - | - | - | - | - | - | - | -149 |
| C3Y6P6_BRAFL | - | - | - | - | - | - | - | - | - | - | - | - | - | - | - | - | - | - | - | - | - | - | - | - | - | - | - | - | - | - | - | - | - | - | - | - | - | - | - | - | - | - | - | - | - | - | - | - | - | - | - | - | - | - | - | - | - | - | - | - | - | -156 |
| C3Y6N1_BRAFL | - | - | - | - | - | - | - | - | - | - | - | - | - | - | - | - | - | - | - | - | - | - | - | - | - | - | - | - | - | - | - | - | - | - | - | - | - | - | - | - | - | - | - | - | - | - | - | - | - | - | - | - | - | - | - | - | - | - | - | - | - | -158 |
| CANA_DROME | - | - | - | - | - | - | - | - | - | - | - | - | - | - | - | - | - | - | - | - | - | - | - | - | - | - | - | - | - | - | - | - | - | - | - | - | - | - | - | - | - | - | - | - | - | - | - | - | - | - | - | - | - | - | - | - | - | - | - | - | - | -148 |
| CANB_DROME | - | - | - | - | - | - | - | - | - | - | - | - | - | - | - | - | - | - | - | - | - | - | - | - | - | - | - | - | - | - | - | - | - | - | - | - | - | - | - | - | - | - | - | - | - | - | - | - | - | - | - | - | - | - | - | - | - | - | - | - | - | -148 |
| X1Y3H71_ANODA | - | - | - | - | - | - | - | - | - | - | - | - | - | - | - | - | - | - | - | - | - | - | - | - | - | - | - | - | - | - | - | - | - | - | - | - | - | - | - | - | - | - | - | - | - | - | - | - | - | - | - | - | - | - | - | - | - | - | - | - | - | -149 |
| Q5IW12_GECLA | - | - | - | - | - | - | - | - | - | - | - | - | - | - | - | - | - | - | - | - | - | - | - | - | - | - | - | - | - | - | - | - | - | - | - | - | - | - | - | - | - | - | - | - | - | - | - | - | - | - | - | - | - | - | - | - | - | - | - | - | - | -148 |
| K1Q056_CRAGI | - | - | - | - | - | - | - | - | - | - | - | - | - | - | - | - | - | - | - | - | - | - | - | - | - | - | - | - | - | - | - | - | - | - | - | - | - | - | - | - | - | - | - | - | - | - | - | - | - | - | - | - | - | - | - | - | - | - | - | - | - | -148 |
| G4V6C0_SCHMA | - | - | - | - | - | - | - | - | - | - | - | - | - | - | - | - | - | - | - | - | - | - | - | - | - | - | - | - | - | - | - | - | - | - | - | - | - | - | - | - | - | - | - | - | - | - | - | - | - | - | - | - | - | - | - | - | - | - | - | - | - | -153 |
| R7ULX9_CAPTE | - | - | - | - | - | - | - | - | - | - | - | - | - | - | - | - | - | - | - | - | - | - | - | - | - | - | - | - | - | - | - | - | - | - | - | - | - | - | - | - | - | - | - | - | - | - | - | - | - | - | - | - | - | - | - | - | - | - | - | - | - | -149 |
| W4Z5Q7_STRPU | - | - | - | - | - | - | - | - | - | - | - | - | - | - | - | - | - | - | - | - | - | - | - | - | - | - | - | - | - | - | - | - | - | - | - | - | - | - | - | - | - | - | - | - | - | - | - | - | - | - | - | - | - | - | - | - | - | - | - | - | - | -146 |
| R7VIB6_CAPTE | - | - | - | - | - | - | - | - | - | - | - | - | - | - | - | - | - | - | - | - | - | - | - | - | - | - | - | - | - | - | - | - | - | - | - | - | - | - | - | - | - | - | - | - | - | - | - | - | - | - | - | - | - | - | - | - | - | - | - | - | - | -152 |
| T2MJ45_HYDVU | - | - | - | - | - | - | - | - | - | - | - | - | - | - | - | - | - | - | - | - | - | - | - | - | - | - | - | - | - | - | - | - | - | - | - | - | - | - | - | - | - | - | - | - | - | - | - | - | - | - | - | - | - | - | - | - | - | - | - | - | - | -148 |
| R7UAB6_CAPTE | - | - | - | - | - | - | - | - | - | - | - | - | - | - | - | - | - | - | - | - | - | - | - | - | - | - | - | - | - | - | - | - | - | - | - | - | - | - | - | - | - | - | - | - | - | - | - | - | - | - | - | - | - | - | - | - | - | - | - | - | - | -148 |
| C3ZKN8_BRAFL | - | - | - | - | - | - | - | - | - | - | - | - | - | - | - | - | - | - | - | - | - | - | - | - | - | - | - | - | - | - | - | - | - | - | - | - | - | - | - | - | - | - | - | - | - | - | - | - | - | - | - | - | - | - | - | - | - | - | - | - | - | -149 |
| B3RJS5_TRIAD | - | - | - | - | - | - | - | - | - | - | - | - | - | - | - | - | - | - | - | - | - | - | - | - | - | - | - | - | - | - | - | - | - | - | - | - | - | - | - | - | - | - | - | - | - | - | - | - | - | - | - | - | - | - | - | - | - | - | - | - | - | -148 |
| H2YVF0_CIOSA | - | - | - | - | - | - | - | - | - | - | - | - | - | - | - | - | - | - | - | - | - | - | - | - | - | - | - | - | - | - | - | - | - | - | - | - | - | - | - | - | - | - | - | - | - | - | - | - | - | - | - | - | - | - | - | - | - | - | - | - | - | -148 |
| G4VAG2_SCHMA | - | - | - | - | - | - | - | - | - | - | - | - | - | - | - | - | - | - | - | - | - | - | - | - | - | - | - | - | - | - | - | - | - | - | - | - | - | - | - | - | - | - | - | - | - | - | - | - | - | - | - | - | - | - | - | - | - | - | - | - | - | -153 |
| Smp_157500 | - | - | - | - | - | - | - | - | - | - | - | - | - | - | - | - | - | - | - | - | - | - | - | - | - | - | - | - | - | - | - | - | - | - | - | - | - | - | - | - | - | - | - | - | - | - | - | - | - | - | - | - | - | - | - | - | - | - | - | - | - | -153 |
| Q7SYA9_DANRE | - | - | - | - | - | - | - | - | - | - | - | - | - | - | - | - | - | - | - | - | - | - | - | - | - | - | - | - | - | - | - | - | - | - | - | - | - | - | - | - | - | - | - | - | - | - | - | - | - | - | - | - | - | - | - | - | - | - | - | - | - | -148 |
| A5PMP1_DANRE | - | - | - | - | - | - | - | - | - | - | - | - | - | - | - | - | - | - | - | - | - | - | - | - | - | - | - | - | - | - | - | - | - | - | - | - | - | - | - | - | - | - | - | - | - | - | - | - | - | - | - | - | - | - | - | - | - | - | - | - | - | -148 |
| CANX_CHICK | - | - | - | - | - | - | - | - | - | - | - | - | - | - | - | - | - | - | - | - | - | - | - | - | - | - | - | - | - | - | - | - | - | - | - | - | - | - | - | - | - | - | - | - | - | - | - | - | - | - | - | - | - | - | - | - | - | - | - | - | - | -148 |
| CAN1_HUMAN | - | - | - | - | - | - | - | - | - | - | - | - | - | - | - | - | - | - | - | - | - | - | - | - | - | - | - | - | - | - | - | - | - | - | - | - | - | - | - | - | - | - | - | - | - | - | - | - | - | - | - | - | - | - | - | - | - | - | - | - | - | -148 |
| CAN1_MOUSE | - | - | - | - | - | - | - | - | - | - | - | - | - | - | - | - | - | - | - | - | - | - | - | - | - | - | - | - | - | - | - | - | - | - | - | - | - | - | - | - | - | - | - | - | - | - | - | - | - | - | - | - | - | - | - | - | - | - | - | - | - | -148 |
| O42133_CHICK | - | - | - | - | - | - | - | - | - | - | - | - | - | - | - | - | - | - | - | - | - | - | - | - | - | - | - | - | - | - | - | - | - | - | - | - | - | - | - | - | - | - | - | - | - | - | - | - | - | - | - | - | - | - | - | - | - | - | - | - | - | -148 |
| Q7ZUR1_DANRE | - | - | - | - | - | - | - | - | - | - | - | - | - | - | - | - | - | - | - | - | - | - | - | - | - | - | - | - | - | - | - | - | - | - | - | - | - | - | - | - | - | - | - | - | - | - | - | - | - | - | - | - | - | - | - | - | - | - | - | - | - | -148 |
| E9QFN0_DANRE | - | - | - | - | - | - | - | - | - | - | - | - | - | - | - | - | - | - | - | - | - | - | - | - | - | - | - | - | - | - | - | - | - | - | - | - | - | - | - | - | - | - | - | - | - | - | - | - | - | - | - | - | - | - | - | - | - | - | - | - | - | -148 |
| CAN2_HUMAN | - | - | - | - | - | - | - | - | - | - | - | - | - | - | - | - | - | - | - | - | - | - | - | - | - | - | - | - | - | - | - | - | - | - | - | - | - | - | - | - | - | - | - | - | - | - | - | - | - | - | - | - | - | - | - | - | - | - | - | - | - | -148 |
| CAN2_MOUSE | - | - | - | - | - | - | - | - | - | - | - | - | - | - | - | - | - | - | - | - | - | - | - | - | - | - | - | - | - | - | - | - | - | - | - | - | - | - | - | - | - | - | - | - | - | - | - | - | - | - | - | - | - | - | - | - | - | - | - | - | - | -148 |
| CAN2_CHICK | - | - | - | - | - | - | - | - | - | - | - | - | - | - | - | - | - | - | - | - | - | - | - | - | - | - | - | - | - | - | - | - | - | - | - | - | - | - | - | - | - | - | - | - | - | - | - | - | - | - | - | - | - | - | - | - | - | - | - | - | - | -148 |
| Q6DFZ8_DANRE | - | - | - | - | - | - | - | - | - | - | - | - | - | - | - | - | - | - | - | - | - | - | - | - | - | - | - | - | - | - | - | - | - | - | - | - | - | - | - | - | - | - | - | - | - | - | - | - | - | - | - | - | - | - | - | - | - | - | - | - | - | -148 |
| Q504F5_DANRE | - | - | - | - | - | - | - | - | - | - | - | - | - | - | - | - | - | - | - | - | - | - | - | - | - | - | - | - | - | - | - | - | - | - | - | - | - | - | - | - | - | - | - | - | - | - | - | - | - | - | - | - | - | - | - | - | - | - | - | - | - | -148 |
| Q5BLH5_DANRE | - | - | - | - | - | - | - | - | - | - | - | - | - | - | - | - | - | - | - | - | - | - | - | - | - | - | - | - | - | - | - | - | - | - | - | - | - | - | - | - | - | - | - | - | - | - | - | - | - | - | - | - | - | - | - | - | - | - | - | - | - | -148 |
| Q4VBH3_DANRE | - | - | - | - | - | - | - | - | - | - | - | - | - | - | - | - | - | - | - | - | - | - | - | - | - | - | - | - | - | - | - | - | - | - | - | - | - | - | - | - | - | - | - | - | - | - | - | - | - | - | - | - | - | - | - | - | - | - | - | - | - | -148 |
| CAN8_RAT | - | - | - | - | - | - | - | - | - | - | - | - | - | - | - | - | - | - | - | - | - | - | - | - | - | - | - | - | - | - | - | - | - | - | - | - | - | - | - | - | - | - | - | - | - | - | - | - | - | - | - | - | - | - | - | - | - | - | - | - | - | -148 |
| CAN8_MOUSE | - | - | - | - | - | - | - | - | - | - | - | - | - | - | - | - | - | - | - | - | - | - | - | - | - | - | - | - | - | - | - | - | - | - | - | - | - | - | - | - | - | - | - | - | - | - | - | - | - | - | - | - | - | - | - | - | - | - | - | - | - | -148 |
| CAN8_HUMAN | - | - | - | - | - | - | - | - | - | - | - | - | - | - | - | - | - | - | - | - | - | - | - | - | - | - | - | - | - | - | - | - | - | - | - | - | - | - | - | - | - | - | - | - | - | - | - | - | - | - | - | - | - | - | - | - | - | - | - | - | - | -148 |
| XP_426117 | - | - | - | - | - | - | - | - | - | - | - | - | - | - | - | - | - | - | - | - | - | - | - | - | - | - | - | - | - | - | - | - | - | - | - | - | - | - | - | - | - | - | - | - | - | - | - | - | - | - | - | - | - | - | - | - | - | - | - | - | - | -148 |
| CAN9_MOUSE | - | - | - | - | - | - | - | - | - | - | - | - | - | - | - | - | - | - | - | - | - | - | - | - | - | - | - | - | - | - | - | - | - | - | - | - | - | - | - | - | - | - | - | - | - | - | - | - | - | - | - | - | - | - | - | - | - | - | - | - | - | -150 |
| CAN9_HUMAN | - | - | - | - | - | - | - | - | - | - | - | - | - | - | - | - | - | - | - | - | - | - | - | - | - | - | - | - | - | - | - | - | - | - | - | - | - | - | - | - | - | - | - | - | - | - | - | - | - | - | - | - | - | - | - | - | - | - | - | - | - | -148 |
| E1C6M3_CHICK | - | - | - | - | - | - | - | - | - | - | - | - | - | - | - | - | - | - | - | - | - | - | - | - | - | - | - | - | - | - | - | - | - | - | - | - | - | - | - | - | - | - | - | - | - | - | - | - | - | - | - | - | - | - | - | - | - | - | - | - | - | -148 |
| Q6DG44_DANRE | - | - | - | - | - | - | - | - | - | - | - | - | - | - | - | - | - | - | - | - | - | - | - | - | - | - | - | - | - | - | - | - | - | - | - | - | - | - | - | - | - | - | - | - | - | - | - | - | - | - | - | - | - | - | - | - | - | - | - | - | - | -148 |
| CAN3_HUMAN | - | - | - | - | - | - | - | - | - | - | - | - | - | - | - | - | - | - | - | - | - | - | - | - | - | - | - | - | - | - | - | - | - | - | - | - | - | - | - | - | - | - | - | - | - | - | - | - | - | - | - | - | - | - | - | - | - | - | - | - | - | -148 |
| CAN3_MOUSE | - | - | - | - | - | - | - | - | - | - | - | - | - | - | - | - | - | - | - | - | - | - | - | - | - | - | - | - | - | - | - | - | - | - | - | - | - | - | - | - | - | - | - | - | - | - | - | - | - | - | - | - | - | - | - | - | - | - | - | - | - | -148 |
| CAN3_CHICK | - | - | - | - | - | - | - | - | - | - | - | - | - | - | - | - | - | - | - | - | - | - | - | - | - | - | - | - | - | - | - | - | - | - | - | - | - | - | - | - | - | - | - | - | - | - | - | - | - | - | - | - | - | - | - | - | - | - | - | - | - | -148 |
| Q66HU4_DANRE | - | - | - | - | - | - | - | - | - | - | - | - | - | - | - | - | - | - | - | - | - | - | - | - | - | - | - | - | - | - | - | - | - | - | - | - | - | - | - | - | - | - | - | - | - | - | - | - | - | - | - | - | - | - | - | - | - | - | - | - | - | -148 |
| F1R632_DANRE | - | - | - | - | - | - | - | - | - | - | - | - | - | - | - | - | - | - | - | - | - | - | - | - | - | - | - | - | - | - | - | - | - | - | - | - | - | - | - | - | - | - | - | - | - | - | - | - | - | - | - | - | - | - | - | - | - | - | - | - | - | -148 |
| CAN11_HUMAN | - | - | - | - | - | - | - | - | - | - | - | - | - | - | - | - | - | - | - | - | - | - | - | - | - | - | - | - | - | - | - | - | - | - | - | - | - | - | - | - | - | - | - | - | - | - | - | - | - | - | - | - | - | - | - | - | - | - | - | - | - | -148 |
| CAN11_MOUSE | - | - | - | - | - | - | - | - | - | - | - | - | - | - | - | - | - | - | - | - | - | - | - | - | - | - | - | - | - | - | - | - | - | - | - | - | - | - | - | - | - | - | - | - | - | - | - | - | - | - | - | - | - | - | - | - | - | - | - | - | - | -148 |
| CAN12_MOUSE | - | - | - | - | - | - | - | - | - | - | - | - | - | - | - | - | - | - | - | - | - | - | - | - | - | - | - | - | - | - | - | - | - | - | - | - | - | - | - | - | - | - | - | - | - | - | - | - | - | - | - | - | - | - | - | - | - | - | - | - | - | -148 |
| CAN12_HUMAN | - | - | - | - | - | - | - | - | - | - | - | - | - | - | - | - | - | - | - | - | - | - | - | - | - | - | - | - | - | - | - | - | - | - | - | - | - | - | - | - | - | - | - | - | - | - | - | - | - | - | - | - | - | - | - | - | - | - | - | - | - | -148 |
| ENSSARP00000008135 | - | - | - | - | - | - | - | - | - | - | - | - | - | - | - | - | - | - | - | - | - | - | - | - | - | - | - | - | - | - | - | - | - | - | - | - | - | - | - | - | - | - | - | - | - | - | - | - | - | - | - | - | - | - | - | - | - | - | - | - | - | -148 |
| ENSSARP00000010553 | - | - | - | - | - | - | - | - | - | - | - | - | - | - | - | - | - | - | - | - | - | - | - | - | - | - | - | - | - | - | - | - | - | - | - | - | - | - | - | - | - | - | - | - | - | - | - | - | - | - | - | - | - | - | - | - | - | - | - | - | - | -147 |
| A7E2G3_DANRE | - | - | - | - | - | - | - | - | - | - | - | - | - | - | - | - | - | - | - | - | - | - | - | - | - | - | - | - | - | - | - | - | - | - | - | - | - | - | - | - | - | - | - | - | - | - | - | - | - | - | - | - | - | - | - | - | - | - | - | - | - | -149 |
| Q6NW69_DANRE | - | - | - | - | - | - | - | - | - | - | - | - | - | - | - | - | - | - | - | - | - | - | - | - | - | - | - | - | - | - | - | - | - | - | - | - | - | - | - | - | - | - | - | - | - | - | - | - | - | - | - | - | - | - | - | - | - | - | - | - | - | -148 |
| CAN13_HUMAN | - | - | - | - | - | - | - | - | - | - | - | - | - | - | - | - | - | - | - | - | - | - | - | - | - | - | - | - | - | - | - | - | - | - | - | - | - | - | - | - | - | - | - | - | - | - | - | - | - | - | - | - | - | - | - | - | - | - | - | - | - | -149 |
| CAN13_MOUSE | - | - | - | - | - | - | - | - | - | - | - | - | - | - | - | - | - | - | - | - | - | - | - | - | - | - | - | - | - | - | - | - | - | - | - | - | - | - | - | - | - | - | - | - | - | - | - | - | - | - | - | - | - | - | - | - | - | - | - | - | - | -151 |
| E1BRR5_CHICK | - | - | - | - | - | - | - | - | - | - | - | - | - | - | - | - | - | - | - | - | - | - | - | - | - | - | - | - | - | - | - | - | - | - | - | - | - | - | - | - | - | - | - | - | - | - | - | - | - | - | - | - | - | - | - | - | - | - | - | - | - | -148 |
| E7F0H3_DANRE | - | - | - | - | - | - | - | - | - | - | - | - | - | - | - | - | - | - | - | - | - | - | - | - | - | - | - | - | - | - | - | - | - | - | - | - | - | - | - | - | - | - | - | - | - | - | - | - | - | - | - | - | - | - | - | - | - | - | - | - | - | -149 |
| Q1RLS9_DANRE | - | - | - | - | - | - | - | - | - | - | - | - | - | - | - | - | - | - | - | - | - | - | - | - | - | - | - | - | - | - | - | - | - | - | - | - | - | - | - | - | - | - | - | - | - | - | - | - | - | - | - | - | - | - | - | - | - | - | - | - | - | -148 |
| F1Q9Z1_DANRE | - | - | - | - | - | - | - | - | - | - | - | - | - | - | - | - | - | - | - | - | - | - | - | - | - | - | - | - | - | - | - | - | - | - | - | - | - | - | - | - | - | - | - | - | - | - | - | - | - | - | - | - | - | - | - | - | - | - | - | - | - | -149 |
| A4QNV6_DANRE | - | - | - | - | - | - | - | - | - | - | - | - | - | - | - | - | - | - | - | - | - | - | - | - | - | - | - | - | - | - | - | - | - | - | - | - | - | - | - | - | - | - | - | - | - | - | - | - | - | - | - | - | - | - | - | - | - | - | - | - | - | -149 |
| D6W573_HUMAN | - | - | - | - | - | - | - | - | - | - | - | - | - | - | - | - | - | - | - | - | - | - | - | - | - | - | - | - | - | - | - | - | - | - | - | - | - | - | - | - | - | - | - | - | - | - | - | - | - | - | - | - | - | - | - | - | - | - | - | - | - | -149 |
| R7UBC6_CAPTE | - | - | - | - | - | - | - | - | - | - | - | - | - | - | - | - | - | - | - | - | - | - | - | - | - | - | - | - | - | - | - | - | - | - | - | - | - | - | - | - | - | - | - | - | - | - | - | - | - | - | - | - | - | - | - | - | - | - | - | - | - | -156 |
| K1RCZ2_CRAGI | - | - | - | - | - | - | - | - | - | - | - | - | - | - | - | - | - | - | - | - | - | - | - | - | - | - | - | - | - | - | - | - | - | - | - | - | - | - | - | - | - | - | - | - | - | - | - | - | - | - | - | - | - | - | - | - | - | - | - | - | - | -159 |
| Q5IW13_GECLA | - | - | - | - | - | - | - | - | - | - | - | - | - | - | - | - | - | - | - | - | - | - | - | - | - | - | - | - | - | - | - | - | - | - | - | - | - | - | - | - | - | - | - | - | - | - | - | - | - | - | - | - | - | - | - | - | - | - | - | - | - | -148 |
| R7UUX7_CAPTE | - | - | - | - | - | - | - | - | - | - | - | - | - | - | - | - | - | - | - | - | - | - | - | - | - | - | - | - | - | - | - | - | - | - | - | - | - | - | - | - | - | - | - | - | - | - | - | - | - | - | - | - | - | - | - | - | - | - | - | - | - | -151 |
| K1Q505_CRAGI | - | - | - | - | - | - | - | - | - | - | - | - | - | - | - | - | - | - | - | - | - | - | - | - | - | - | - | - | - | - | - | - | - | - | - | - | - | - | - | - | - | - | - | - | - | - | - | - | - | - | - | - | - | - | - | - | - | - | - | - | - | -152 |
| R7UD02_CAPTE | - | - | - | - | - | - | - | - | - | - | - | - | - | - | - | - | - | - | - | - | - | - | - | - | - | - | - | - | - | - | - | - | - | - | - | - | - | - | - | - | - | - | - | - | - | - | - | - | - | - | - | - | - | - | - | - | - | - | - | - | - | -151 |
| CAN6_HUMAN | - | - | - | - | - | - | - | - | - | - | - | - | - | - | - | - | - | - | - | - | - | - | - | - | - | - | - | - | - | - | - | - | - | - | - | - | - | - | - | - | - | - | - | - | - | - | - | - | - | - | - | - | - | - | - | - | - | - | - | - | - | -161 |
| CAN6_MOUSE | - | - | - | - | - | - | - | - | - | - | - | - | - | - | - | - | - | - | - | - | - | - | - | - | - | - | - | - | - | - | - | - | - | - | - | - | - | - | - | - | - | - | - | - | - | - | - | - | - | - | - | - | - | - | - | - | - | - | - | - | - | -161 |
| E1C312_CHICK | - | - | - | - | - | - | - | - | - | - | - | - | - | - | - | - | - | - | - | - | - | - | - | - | - | - | - | - | - | - | - | - | - | - | - | - | - | - | - | - | - | - | - | - | - | - | - | - | - | - | - | - | - | - | - | - | - | - | - | - | - | -161 |
| E7FDN3_DANRE | - | - | - | - | - | - | - | - | - | - | - | - | - | - | - | - | - | - | - | - | - | - | - | - | - | - | - | - | - | - | - | - | - | - | - | - | - | - | - | - | - | - | - | - | - | - | - | - | - | - | - | - | - | - | - | - | - | - | - | - | - | -162 |
| CAN5_HUMAN | - | - | - | - | - | - | - | - | - | - | - | - | - | - | - | - | - | - | - | - | - | - | - | - | - | - | - | - | - | - | - | - | - | - | - | - | - | - | - | - | - | - | - | - | - | - | - | - | - | - | - | - | - | - | - | - | - | - | - | - | - | -157 |
| CAN5_MOUSE | - | - | - | - | - | - | - | - | - | - | - | - | - | - | - | - | - | - | - | - | - | - | - | - | - | - | - | - | - | - | - | - | - | - | - | - | - | - | - | - | - | - | - | - | - | - | - | - | - | - | - | - | - | - | - | - | - | - | - | - | - | -161 |
| E1C292_CHICK | - | - | - | - | - | - | - | - | - | - | - | - | - | - | - | - | - | - | - | - | - | - | - | - | - | - | - | - | - | - | - | - | - | - | - | - | - | - | - | - | - | - | - | - | - | - | - | - | - | - | - | - | - | - | - | - | - | - | - | - | - | -161 |
| A0JMG3_DANRE | - | - | - | - | - | - | - | - | - | - | - | - | - | - | - | - | - | - | - | - | - | - | - | - | - | - | - | - | - | - | - | - | - | - | - | - | - | - | - | - | - | - | - | - | - | - | - | - | - | - | - | - | - | - | - | - | - | - | - | - | - | -161 |
| E7F7F1_DANRE | - | - | - | - | - | - | - | - | - | - | - | - | - | - | - | - | - | - | - | - | - | - | - | - | - | - | - | - | - | - | - | - | - | - | - | - | - | - | - | - | - | - | - | - | - | - | - | - | - | - | - | - | - | - | - | - | - | - | - | - | - | -161 |
| XP_006824412 | - | - | - | - | - | - | - | - | - | - | - | - | - | - | - | - | - | - | - | - | - | - | - | - | - | - | - | - | - | - | - | - | - | - | - | - | - | - | - | - | - | - | - | - | - | - | - | - | - | - | - | - | - | - | - | - | - | - | - | - | - | -153 |
| W4YJN2_STRPU | - | - | - | - | - | - | - | - | - | - | - | - | - | - | - | - | - | - | - | - | - | - | - | - | - | - | - | - | - | - | - | - | - | - | - | - | - | - | - | - | - | - | - | - | - | - | - | - | - | - | - | - | - | - | - | - | - | - | - | - | - | -161 |
| C3YJZ3_BRAFL | - | - | - | - | - | - | - | - | - | - | - | - | - | - | - | - | - | - | - | - | - | - | - | - | - | - | - | - | - | - | - | - | - | - | - | - | - | - | - | - | - | - | - | - | - | - | - | - | - | - | - | - | - | - | - | - | - | - | - | - | - | -161 |
| T2MIT4_HYDVU | - | - | - | - | - | - | - | - | - | - | - | - | - | - | - | - | - | - | - | - | - | - | - | - | - | - | - | - | - | - | - | - | - | - | - | - | - | - | - | - | - | - | - | - | - | - | - | - | - | - | - | - | - | - | - | - | - | - | - | - | - | -161 |
| XP_002154722 | - | - | - | - | - | - | - | - | - | - | - | - | - | - | - | - | - | - | - | - | - | - | - | - | - | - | - | - | - | - | - | - | - | - | - | - | - | - | - | - | - | - | - | - | - | - | - | - | - | - | - | - | - | - | - | - | - | - | - | - | - | -125 |
| R7VLA7_CAPTE | - | - | - | - | - | - | - | - | - | - | - | - | - | - | - | - | - | - | - | - | - | - | - | - | - | - | - | - | - | - | - | - | - | - | - | - | - | - | - | - | - | - | - | - | - | - | - | - | - | - | - | - | - | - | - | - | - | - | - | - | - | -161 |
| K1R1C6_CRAGI | - | - | - | - | - | - | - | - | - | - | - | - | - | - | - | - | - | - | - | - | - | - | - | - | - | - | - | - | - | - | - | - | - | - | - | - | - | - | - | - | - | - | - | - | - | - | - | - | - | - | - | - | - | - | - | - | - | - | - | - | - | -161 |
| B3RIE5_TRIAD | - | - | - | - | - | - | - | - | - | - | - | - | - | - | - | - | - | - | - | - | - | - | - | - | - | - | - | - | - | - | - | - | - | - | - | - | - | - | - | - | - | - | - | - | - | - | - | - | - | - | - | - | - | - | - | - | - | - | - | - | - | -159 |
| K1PW75_CRAGI | - | - | - | - | - | - | - | - | - | - | - | - | - | - | - | - | - | - | - | - | - | - | - | - | - | - | - | - | - | - | - | - | - | - | - | - | - | - | - | - | - | - | - | - | - | - | - | - | - | - | - | - | - | - | - | - | - | - | - | - | - | -157 |
| K1RSB8_CRAGI | - | - | - | - | - | - | - | - | - | - | - | - | - | - | - | - | - | - | - | - | - | - | - | - | - | - | - | - | - | - | - | - | - | - | - | - | - | - | - | - | - | - | - | - | - | - | - | - | - | - | - | - | - | - | - | - | - | - | - | - | - | -156 |
| K1RWI3_CRAGI | - | - | - | - | - | - | - | - | - | - | - | - | - | - | - | - | - | - | - | - | - | - | - | - | - | - | - | - | - | - | - | - | - | - | - | - | - | - | - | - | - | - | - | - | - | - | - | - | - | - | - | - | - | - | - | - | - | - | - | - | - | -159 |
| R7VGC2_CAPTE | - | - | - | - | - | - | - | - | - | - | - | - | - | - | - | - | - | - | - | - | - | - | - | - | - | - | - | - | - | - | - | - | - | - | - | - | - | - | - | - | - | - | - | - | - | - | - | - | - | - | - | - | - | - | - | - | - | - | - | - | - | -159 |
| K1QU26_CRAGI | - | - | - | - | - | - | - | - | - | - | - | - | - | - | - | - | - | - | - | - | - | - | - | - | - | - | - | - | - | - | - | - | - | - | - | - | - | - | - | - | - | - | - | - | - | - | - | - | - | - | - | - | - | - | - | - | - | - | - | - | - | -161 |
| CAN5_CAEEL | - | - | - | - | - | - | - | - | - | - | - | - | - | - | - | - | - | - | - | - | - | - | - | - | - | - | - | - | - | - | - | - | - | - | - | - | - | - | - | - | - | - | - | - | - | - | - | - | - | - | - | - | - | - | - | - | - | - | - | - | - | -159 |
| I1FGV4_AMPQE | - | - | - | - | - | - | - | - | - | - | - | - | - | - | - | - | - | - | - | - | - | - | - | - | - | - | - | - | - | - | - | - | - | - | - | - | - | - | - | - | - | - | - | - | - | - | - | - | - | - | - | - | - | - | - | - | - | - | - | - | - | -147 |
| K1PHX3_CRAGI | - | - | - | - | - | - | - | - | - | - | - | - | - | - | - | - | - | - | - | - | - | - | - | - | - | - | - | - | - | - | - | - | - | - | - | - | - | - | - | - | - | - | - | - | - | - | - | - | - | - | - | - | - | - | - | - | - | - | - | - | - | -148 |
| B3RNL8_TRIAD | - | - | - | - | - | - | - | - | - | - | - | - | - | - | - | - | - | - | - | - | - | - | - | - | - | - | - | - | - | - | - | - | - | - | - | - | - | - | - | - | - | - | - | - | - | - | - | - | - | - | - | - | - | - | - | - | - | - | - | - | - | -150 |
| X1Y3H7_ANODA | - | - | - | - | - | - | - | - | - | - | - | - | - | - | - | - | - | - | - | - | - | - | - | - | - | - | - | - | - | - | - | - | - | - | - | - | - | - | - | - | - | - | - | - | - | - | - | - | - | - | - | - | - | - | - | - | - | - | - | - | - | -152 |
| R7TWH9_CAPTE | - | - | - | - | - | - | - | - | - | - | - | - | - | - | - | - | - | - | - | - | - | - | - | - | - | - | - | - | - | - | - | - | - | - | - | - | - | - | - | - | - | - | - | - | - | - | - | - | - | - | - | - | - | - | - | - | - | - | - | - | - | -152 |
| K1R5E8_CRAGI | - | - | - | - | - | - | - | - | - | - | - | - | - | - | - | - | - | - | - | - | - | - | - | - | - | - | - | - | - | - | - | - | - | - | - | - | - | - | - | - | - | - | - | - | - | - | - | - | - | - | - | - | - | - | - | - | - | - | - | - | - | -152 |
| XP_006811804 | - | - | - | - | - | - | - | - | - | - | - | - | - | - | - | - | - | - | - | - | - | - | - | - | - | - | - | - | - | - | - | - | - | - | - | - | - | - | - | - | - | - | - | - | - | - | - | - | - | - | - | - | - | - | - | - | - | - | - | - | - | -150 |
| C3YB23_BRAFL | - | - | - | - | - | - | - | - | - | - | - | - | - | - | - | - | - | - | - | - | - | - | - | - | - | - | - | - | - | - | - | - | - | - | - | - | - | - | - | - | - | - | - | - | - | - | - | - | - | - | - | - | - | - | - | - | - | - | - | - | - | -157 |
| H2ZIF1_CIOSA | - | - | - | - | - | - | - | - | - | - | - | - | - | - | - | - | - | - | - | - | - | - | - | - | - | - | - | - | - | - | - | - | - | - | - | - | - | - | - | - | - | - | - | - | - | - | - | - | - | - | - | - | - | - | - | - | - | - | - | - | - | -150 |
| W4YV93_STRPU | - | - | - | - | - | - | - | - | - | - | - | - | - | - | - | - | - | - | - | - | - | - | - | - | - | - | - | - | - | - | - | - | - | - | - | - | - | - | - | - | - | - | - | - | - | - | - | - | - | - | - | - | - | - | - | - | - | - | - | - | - | -150 |
| CAN10_HUMAN | - | - | - | - | - | - | - | - | - | - | - | - | - | - | - | - | - | - | - | - | - | - | - | - | - | - | - | - | - | - | - | - | - | - | - | - | - | - | - | - | - | - | - | - | - | - | - | - | - | - | - | - | - | - | - | - | - | - | - | - | - | -157 |
| CAN10_MOUSE | - | - | - | - | - | - | - | - | - | - | - | - | - | - | - | - | - | - | - | - | - | - | - | - | - | - | - | - | - | - | - | - | - | - | - | - | - | - | - | - | - | - | - | - | - | - | - | - | - | - | - | - | - | - | - | - | - | - | - | - | - | -157 |
| XP_004943443 | - | - | - | - | - | - | - | - | - | - | - | - | - | - | - | - | - | - | - | - | - | - | - | - | - | - | - | - | - | - | - | - | - | - | - | - | - | - | - | - | - | - | - | - | - | - | - | - | - | - | - | - | - | - | - | - | - | - | - | - | - | -157 |
| F8W4K1_DANRE | - | - | - | - | - | - | - | - | - | - | - | - | - | - | - | - | - | - | - | - | - | - | - | - | - | - | - | - | - | - | - | - | - | - | - | - | - | - | - | - | - | - | - | - | - | - | - | - | - | - | - | - | - | - | - | - | - | - | - | - | - | -157 |
| CANC_DROME | - | - | - | - | - | - | - | - | - | - | - | - | - | - | - | - | - | - | - | - | - | - | - | - | - | - | - | - | - | - | - | - | - | - | - | - | - | - | - | - | - | - | - | - | - | - | - | - | - | - | - | - | - | - | - | - | - | - | - | - | - | -143 |
| C3ZKN9_BRAFL | - | - | - | - | - | - | - | - | - | - | - | - | - | - | - | - | - | - | - | - | - | - | - | - | - | - | - | - | - | - | - | - | - | - | - | - | - | - | - | - | - | - | - | - | - | - | - | - | - | - | - | - | - | - | - | - | - | - | - | - | - | -149 |
| G4V623_SCHMA | - | - | - | - | - | - | - | - | - | - | - | - | - | - | - | - | - | - | - | - | - | - | - | - | - | - | - | - | - | - | - | - | - | - | - | - | - | - | - | - | - | - | - | - | - | - | - | - | - | - | - | - | - | - | - | - | - | - | - | - | - | -174 |
| R7VK39_CAPTE | - | - | - | - | - | - | - | - | - | - | - | - | - | - | - | - | - | - | - | - | - | - | - | - | - | - | - | - | - | - | - | - | - | - | - | - | - | - | - | - | - | - | - | - | - | - | - | - | - | - | - | - | - | - | - | - | - | - | - | - | - | -151 |
| G4V693_SCHMA | - | - | - | - | - | - | - | - | - | - | - | - | - | - | - | - | - | - | - | - | - | - | - | - | - | - | - | - | - | - | - | - | - | - | - | - | - | - | - | - | - | - | - | - | - | - | - | - | - | - | - | - | - | - | - | - | - | - | - | - | - | -160 |
| R7UPU6_CAPTE | - | - | - | - | - | - | - | - | - | - | - | - | - | - | - | - | - | - | - | - | - | - | - | - | - | - | - | - | - | - | - | - | - | - | - | - | - | - | - | - | - | - | - | - | - | - | - | - | - | - | - | - | - | - | - | - | - | - | - | - | - | -155 |
| R7V817_CAPTE | - | - | - | - | - | - | - | - | - | - | - | - | - | - | - | - | - | - | - | - | - | - | - | - | - | - | - | - | - | - | - | - | - | - | - | - | - | - | - | - | - | - | - | - | - | - | - | - | - | - | - | - | - | - | - | - | - | - | - | - | - | -152 |
| R7UNL8_CAPTE | - | - | - | - | - | - | - | - | - | - | - | - | - | - | - | - | - | - | - | - | - | - | - | - | - | - | - | - | - | - | - | - | - | - | - | - | - | - | - | - | - | - | - | - | - | - | - | - | - | - | - | - | - | - | - | - | - | - | - | - | - | -150 |
| K1PJ80_CRAGI | - | - | - | - | - | - | - | - | - | - | - | - | - | - | - | - | - | - | - | - | - | - | - | - | - | - | - | - | - | - | - | - | - | - | - | - | - | - | - | - | - | - | - | - | - | - | - | - | - | - | - | - | - | - | - | - | - | - | - | - | - | -155 |
| K1PCX8_CRAGI | - | - | - | - | - | - | - | - | - | - | - | - | - | - | - | - | - | - | - | - | - | - | - | - | - | - | - | - | - | - | - | - | - | - | - | - | - | - | - | - | - | - | - | - | - | - | - | - | - | - | - | - | - | - | - | - | - | - | - | - | - | -155 |
| CAND_DROME | - | - | - | - | - | - | - | - | - | - | - | - | - | - | - | - | - | - | - | - | - | - | - | - | - | - | - | - | - | - | - | - | - | - | - | - | - | - | - | - | - | - | - | - | - | - | - | - | - | - | - | - | - | - | - | - | - | - | - | P | S | -154 |
| XP_002735765 | - | - | - | - | - | - | - | - | - | - | - | - | - | - | - | - | - | - | - | - | - | - | - | - | - | - | - | - | - | - | - | - | - | - | - | - | - | - | - | - | - | - | - | - | - | - | - | - | - | - | - | - | - | - | - | - | - | - | - | N | - | -155 |
| CAN15_HUMAN | - | - | - | - | - | - | - | - | - | - | - | - | - | - | - | - | - | - | - | - | - | - | - | - | - | - | - | - | - | - | - | - | - | - | - | - | - | - | - | - | - | - | - | - | - | - | - | - | - | - | - | - | - | - | - | - | - | - | - | P | R | -155 |
| CAN15_MOUSE | - | - | - | - | - | - | - | - | - | - | - | - | - | - | - | - | - | - | - | - | - | - | - | - | - | - | - | - | - | - | - | - | - | - | - | - | - | - | - | - | - | - | - | - | - | - | - | - | - | - | - | - | - | - | - | - | - | - | - | P | R | -155 |
| E1BTA8_CHICK | - | - | - | - | - | - | - | - | - | - | - | - | - | - | - | - | - | - | - | - | - | - | - | - | - | - | - | - | - | - | - | - | - | - | - | - | - | - | - | - | - | - | - | - | - | - | - | - | - | - | - | - | - | - | - | - | - | - | - | P | R | -155 |
| E7FAX5_DANRE | - | - | - | - | - | - | - | - | - | - | - | - | - | - | - | - | - | - | - | - | - | - | - | - | - | - | - | - | - | - | - | - | - | - | - | - | - | - | - | - | - | - | - | - | - | - | - | - | - | - | - | - | - | - | - | - | - | - | - | P | R | -155 |
| C3XWK8_BRAFL | - | - | - | - | - | - | - | - | - | - | - | - | - | - | - | - | - | - | - | - | - | - | - | - | - | - | - | - | - | - | - | - | - | - | - | - | - | - | - | - | - | - | - | - | - | - | - | - | - | - | - | - | - | - | - | - | - | - | - | P | D | -155 |
| W4YMU4_STRPU | - | - | - | - | - | - | - | - | - | - | - | - | - | - | - | - | - | - | - | - | - | - | - | - | - | - | - | - | - | - | - | - | - | - | - | - | - | - | - | - | - | - | - | - | - | - | - | - | - | - | - | - | - | - | - | - | - | - | - | V | G | -269 |
| K1PV74_CRAGI | - | - | - | - | - | - | - | - | - | - | - | - | - | - | - | - | - | - | - | - | - | - | - | - | - | - | - | - | - | - | - | - | - | - | - | - | - | - | - | - | - | - | - | - | - | - | - | - | - | - | - | - | - | - | - | - | - | - | - | - | - | -153 |
| R7TJF2_CAPTE | - | - | - | - | - | - | - | - | - | - | - | - | - | - | - | - | - | - | - | - | - | - | - | - | - | - | - | - | - | - | - | - | - | - | - | - | - | - | - | - | - | - | - | - | - | - | - | - | - | - | - | - | - | - | - | - | - | - | - | - | - | -152 |
| T2MI17_HYDVU | - | - | - | - | - | - | - | - | - | - | - | - | - | - | - | - | - | - | - | - | - | - | - | - | - | - | - | - | - | - | - | - | - | - | - | - | - | - | - | - | - | - | - | - | - | - | - | - | - | - | - | - | - | - | - | - | - | - | - | A | E | -152 |
| Q1RL55_CIOIN | - | - | - | - | - | - | - | - | - | - | - | - | - | - | - | - | - | - | - | - | - | - | - | - | - | - | - | - | - | - | - | - | - | - | - | - | - | - | - | - | - | - | - | - | - | - | - | - | - | - | - | - | - | - | - | - | - | - | - | - | - | -139 |
| I1EA30_AMPQE | - | - | - | - | - | - | - | - | - | - | - | - | - | - | - | - | - | - | - | - | - | - | - | - | - | - | - | - | - | - | - | - | - | - | - | - | - | - | - | - | - | - | - | - | - | - | - | - | - | - | - | - | - | - | - | - | - | - | - | - | - | -153 |
| G5EEZ6_CAEEL | - | - | - | - | - | - | - | - | - | - | - | - | - | - | - | - | - | - | - | - | - | - | - | - | - | - | - | - | - | - | - | - | - | - | - | - | - | - | - | - | - | - | - | - | - | - | - | - | - | - | - | - | - | - | - | - | - | - | - | - | - | -153 |
| O44903_CAEEL | - | - | - | - | - | - | - | - | - | - | - | - | - | - | - | - | - | - | - | - | - | - | - | - | - | - | - | - | - | - | - | - | - | - | - | - | - | - | - | - | - | - | - | - | - | - | - | - | - | - | - | - | - | - | - | - | - | - | - | - | - | -153 |
| O02260_CAEEL | - | - | - | - | - | - | - | - | - | - | - | - | - | - | - | - | - | - | - | - | - | - | - | - | - | - | - | - | - | - | - | - | - | - | - | - | - | - | - | - | - | - | - | - | - | - | - | - | - | - | - | - | - | - | - | - | - | - | - | R | K | -153 |
| O02259_CAEEL | - | - | - | - | - | - | - | - | - | - | - | - | - | - | - | - | - | - | - | - | - | - | - | - | - | - | - | - | - | - | - | - | - | - | - | - | - | - | - | - | - | - | - | - | - | - | - | - | - | - | - | - | - | - | - | - | - | - | - | R | K | -130 |
| C1MTG3_MICPC | - | - | - | - | - | - | - | - | - | - | - | - | - | - | - | - | - | - | - | - | - | - | - | - | - | - | - | - | - | - | - | - | - | - | - | - | - | - | - | - | - | - | - | - | - | - | - | - | - | - | - | - | - | - | - | - | - | - | - | G | G | -197 |
| C1E493_MICSR | - | - | - | - | - | - | - | - | - | - | - | - | - | - | - | - | - | - | - | - | - | - | - | - | - | - | - | - | - | - | - | - | - | - | - | - | - | - | - | - | - | - | - | - | - | - | - | - | - | - | - | - | - | - | - | - | - | - | - | - | - | -146 |
| Q012Z6_OSTTA | - | - | - | - | - | - | - | - | - | - | - | - | - | - | - | - | - | - | - | - | - | - | - | - | - | - | - | - | - | - | - | - | - | - | - | - | - | - | - | - | - | - | - | - | - | - | - | - | - | - | - | - | - | - | - | - | - | - | - | - | - | -143 |
| L1IGB6_GUITH | - | - | - | - | - | - | - | - | - | - | - | - | - | - | - | - | - | - | - | - | - | - | - | - | - | - | - | - | - | - | - | - | - | - | - | - | - | - | - | - | - | - | - | - | - | - | - | - | - | - | - | - | - | - | - | - | - | - | - | - | - | -146 |
| B7FWX6_PHATC | - | - | - | - | - | - | - | - | - | - | - | - | - | - | - | - | - | - | - | - | - | - | - | - | - | - | - | - | - | - | - | - | - | - | - | - | - | - | - | - | - | - | - | - | - | - | - | - | - | - | - | - | - | - | - | - | - | - | - | - | - | -151 |
| N9TPR9_ENTHI | - | - | - | - | - | - | - | - | - | - | - | - | - | - | - | - | - | - | - | - | - | - | - | - | - | - | - | - | - | - | - | - | - | - | - | - | - | - | - | - | - | - | - | - | - | - | - | - | - | - | - | - | - | - | - | - | - | - | - | - | - | -137 |
| G0QS43_ICHMG | - | - | - | - | - | - | - | - | - | - | - | - | - | - | - | - | - | - | - | - | - | - | - | - | - | - | - | - | - | - | - | - | - | - | - | - | - | - | - | - | - | - | - | - | - | - | - | - | - | - | - | - | - | - | - | - | - | - | - | - | - | -145 |
| D8LB20_ECTSI | - | - | - | - | - | - | - | - | - | - | - | - | - | - | - | - | - | - | - | - | - | - | - | - | - | - | - | - | - | - | - | - | - | - | - | - | - | - | - | - | - | - | - | - | - | - | - | - | - | - | - | - | - | - | - | - | - | - | - | - | - | -183 |
| Q9U0T9_LEIMA | - | - | - | - | - | - | - | - | - | - | - | - | - | - | - | - | - | - | - | - | - | - | - | - | - | - | - | - | - | - | - | - | - | - | - | - | - | - | - | - | - | - | - | - | - | - | - | - | - | - | - | - | - | - | - | - | - | - | - | - | - | -156 |
| Q4CXJ8_TRYCC | - | - | - | - | - | - | - | - | - | - | - | - | - | - | - | - | - | - | - | - | - | - | - | - | - | - | - | - | - | - | - | - | - | - | - | - | - | - | - | - | - | - | - | - | - | - | - | - | - | - | - | - | - | - | - | - | - | - | - | - | - | -156 |
| F4I0A4_ARATH | - | - | - | - | - | - | - | - | - | - | - | - | - | - | - | - | - | - | - | - | - | - | - | - | - | - | - | - | - | - | - | - | - | - | - | - | - | - | - | - | - | - | - | - | - | - | - | - | - | - | - | - | - | - | - | - | - | - | - | - | - | -154 |
| Q8RUQ1_MAIZE | - | - | - | - | - | - | - | - | - | - | - | - | - | - | - | - | - | - | - | - | - | - | - | - | - | - | - | - | - | - | - | - | - | - | - | - | - | - | - | - | - | - | - | - | - | - | - | - | - | - | - | - | - | - | - | - | - | - | - | - | - | -154 |
| D7FQ17_ECTSI | - | - | - | - | - | - | - | - | - | - | - | - | - | - | - | - | - | - | - | - | - | - | - | - | - | - | - | - | - | - | - | - | - | - | - | - | - | - | - | - | - | - | - | - | - | - | - | - | - | - | - | - | - | - | - | - | - | - | - | - | - | -171 |
| D0MZ44_PHYIT | - | - | - | - | - | - | - | - | - | - | - | - | - | - | - | - | - | - | - | - | - | - | - | - | - | - | - | - | - | - | - | - | - | - | - | - | - | - | - | - | - | - | - | - | - | - | - | - | - | - | - | - | - | - | - | - | - | - | - | - | - | -172 |
| L1JBM0_GUITH | - | - | - | - | - | - | - | - | - | - | - | - | - | - | - | - | - | - | - | - | - | - | - | - | - | - | - | - | - | - | - | - | - | - | - | - | - | - | - | - | - | - | - | - | - | - | - | - | - | - | - | - | - | - | - | - | - | - | - | - | - | -127 |
| L1J739_GUITH | - | - | - | - | - | - | - | - | - | - | - | - | - | - | - | - | - | - | - | - | - | - | - | - | - | - | - | - | - | - | - | - | - | - | - | - | - | - | - | - | - | - | - | - | - | - | - | - | - | - | - | - | - | - | - | - | - | - | - | - | - | -160 |
| L1J0U9_GUITH | - | - | - | - | - | - | - | - | - | - | - | - | - | - | - | - | - | - | - | - | - | - | - | - | - | - | - | - | - | - | - | - | - | - | - | - | - | - | - | - | - | - | - | - | - | - | - | - | - | - | - | - | - | - | - | - | - | - | - | - | - | -153 |
| L1J1Q4_GUITH | - | - | - | - | - | - | - | - | - | - | - | - | - | - | - | - | - | - | - | - | - | - | - | - | - | - | - | - | - | - | - | - | - | - | - | - | - | - | - | - | - | - | - | - | - | - | - | - | - | - | - | - | - | - | - | - | - | - | - | - | - | -171 |
| D0ND88_PHYIT | - | - | - | - | - | - | - | - | - | - | - | - | - | - | - | - | - | - | - | - | - | - | - | - | - | - | - | - | - | - | - | - | - | - | - | - | - | - | - | - | - | - | - | - | - | - | - | - | - | - | - | - | - | - | - | - | - | - | - | G | N | -193 |
| D2VVS6_NAEGR | - | - | - | - | - | - | - | - | - | - | - | - | - | - | - | - | - | - | - | - | - | - | - | - | - | - | - | - | - | - | - | - | - | - | - | - | - | - | - | - | - | - | - | - | - | - | - | - | - | - | - | - | - | - | - | - | - | - | - | - | - | -152 |
| D2V3V2_NAEGR | - | - | - | - | - | - | - | - | - | - | - | - | - | - | - | - | - | - | - | - | - | - | - | - | - | - | - | - | - | - | - | - | - | - | - | - | - | - | - | - | - | - | - | - | - | - | - | - | - | - | - | - | - | - | - | - | - | - | - | - | - | -153 |
| Q5B5J7_EMENI | - | - | - | - | - | - | - | - | - | - | - | - | - | - | - | - | - | - | - | - | - | - | - | - | - | - | - | - | - | - | - | - | - | - | - | - | - | - | - | - | - | - | - | - | - | - | - | - | - | - | - | - | - | - | - | - | - | - | - | - | - | -171 |
| E4ZY10_LEPMJ | - | - | - | - | - | - | - | - | - | - | - | - | - | - | - | - | - | - | - | - | - | - | - | - | - | - | - | - | - | - | - | - | - | - | - | - | - | - | - | - | - | - | - | - | - | - | - | - | - | - | - | - | - | - | - | - | - | - | - | - | - | -177 |
| A8PIE5_COPC7 | - | - | - | - | - | - | - | - | - | - | - | - | - | - | - | - | - | - | - | - | - | - | - | - | - | - | - | - | - | - | - | - | - | - | - | - | - | - | - | - | - | - | - | - | - | - | - | - | - | - | - | - | - | - | - | - | - | - | - | - | - | -172 |
| A8NBA6_COPC7 | - | - | - | - | - | - | - | - | - | - | - | - | - | - | - | - | - | - | - | - | - | - | - | - | - | - | - | - | - | - | - | - | - | - | - | - | - | - | - | - | - | - | - | - | - | - | - | - | - | - | - | - | - | - | - | - | - | - | - | - | - | -171 |
| A8NBA1_COPC7 | - | - | - | - | - | - | - | - | - | - | - | - | - | - | - | - | - | - | - | - | - | - | - | - | - | - | - | - | - | - | - | - | - | - | - | - | - | - | - | - | - | - | - | - | - | - | - | - | - | - | - | - | - | - | - | - | - | - | - | - | - | -175 |
| A8NBA2_COPC7 | - | - | - | - | - | - | - | - | - | - | - | - | - | - | - | - | - | - | - | - | - | - | - | - | - | - | - | - | - | - | - | - | - | - | - | - | - | - | - | - | - | - | - | - | - | - | - | - | - | - | - | - | - | - | - | - | - | - | - | - | - | -175 |
| A8NAE6_COPC7 | - | - | - | - | - | - | - | - | - | - | - | - | - | - | - | - | - | - | - | - | - | - | - | - | - | - | - | - | - | - | - | - | - | - | - | - | - | - | - | - | - | - | - | - | - | - | - | - | - | - | - | - | - | - | - | - | - | - | - | - | - | -193 |
| A8NAI9_COPC7 | - | - | - | - | - | - | - | - | - | - | - | - | - | - | - | - | - | - | - | - | - | - | - | - | - | - | - | - | - | - | - | - | - | - | - | - | - | - | - | - | - | - | - | - | - | - | - | - | - | - | - | - | - | - | - | - | - | - | - | - | - | -171 |
| G4TER7_PIRID | - | - | - | - | - | - | - | - | - | - | - | - | - | - | - | - | - | - | - | - | - | - | - | - | - | - | - | - | - | - | - | - | - | - | - | - | - | - | - | - | - | - | - | - | - | - | - | - | - | - | - | - | - | - | - | - | - | - | - | - | - | -171 |
| G4TER6_PIRID | - | - | - | - | - | - | - | - | - | - | - | - | - | - | - | - | - | - | - | - | - | - | - | - | - | - | - | - | - | - | - | - | - | - | - | - | - | - | - | - | - | - | - | - | - | - | - | - | - | - | - | - | - | - | - | - | - | - | - | - | - | -161 |
| G4TNA1_PIRID | - | - | - | - | - | - | - | - | - | - | - | - | - | - | - | - | - | - | - | - | - | - | - | - | - | - | - | - | - | - | - | - | - | - | - | - | - | - | - | - | - | - | - | - | - | - | - | - | - | - | - | - | - | - | - | - | - | - | - | - | - | -171 |
| D0MWB9_PHYIT | - | - | - | - | - | - | - | - | - | - | - | - | - | - | - | - | - | - | - | - | - | - | - | - | - | - | - | - | - | - | - | - | - | - | - | - | - | - | - | - | - | - | - | - | - | - | - | - | - | - | - | - | - | - | - | - | - | - | - | - | - | -161 |
| L1I9H5_GUITH | - | - | - | - | - | - | - | - | - | - | - | - | - | - | - | - | - | - | - | - | - | - | - | - | - | - | - | - | - | - | - | - | - | - | - | - | - | - | - | - | - | - | - | - | - | - | - | - | - | - | - | - | - | - | - | - | - | - | - | - | - | -163 |
| Q7N497_PHOLL | - | - | - | - | - | - | - | - | - | - | - | - | - | - | - | - | - | - | - | - | - | - | - | - | - | - | - | - | - | - | - | - | - | - | - | - | - | - | - | - | - | - | - | - | - | - | - | - | - | - | - | - | - | - | - | - | - | - | - | - | - | -132 |
| ZP_11152369 | - | - | - | - | - | - | - | - | - | - | - | - | - | - | - | - | - | - | - | - | - | - | - | - | - | - | - | - | - | - | - | - | - | - | - | - | - | - | - | - | - | - | - | - | - | - | - | - | - | - | - | - | - | - | - | - | - | - | - | - | - | -128 |
| A2FEL7_TRIVA | - | - | - | - | - | - | - | - | - | - | - | - | - | - | - | - | - | - | - | - | - | - | - | - | - | - | - | - | - | - | - | - | - | - | - | - | - | - | - | - | - | - | - | - | - | - | - | - | - | - | - | - | - | - | - | - | - | - | - | - | - | -150 |
| A2D9A8_TRIVA | - | - | - | - | - | - | - | - | - | - | - | - | - | - | - | - | - | - | - | - | - | - | - | - | - | - | - | - | - | - | - | - | - | - | - | - | - | - | - | - | - | - | - | - | - | - | - | - | - | - | - | - | - | - | - | - | - | - | - | - | - | -151 |
| A2FHB3_TRIVA | - | - | - | - | - | - | - | - | - | - | - | - | - | - | - | - | - | - | - | - | - | - | - | - | - | - | - | - | - | - | - | - | - | - | - | - | - | - | - | - | - | - | - | - | - | - | - | - | - | - | - | - | - | - | - | - | - | - | - | - | - | -152 |
| A2DTE8_TRIVA | - | - | - | - | - | - | - | - | - | - | - | - | - | - | - | - | - | - | - | - | - | - | - | - | - | - | - | - | - | - | - | - | - | - | - | - | - | - | - | - | - | - | - | - | - | - | - | - | - | - | - | - | - | - | - | - | - | - | - | - | - | -149 |
| A2E4X2_TRIVA | - | - | - | - | - | - | - | - | - | - | - | - | - | - | - | - | - | - | - | - | - | - | - | - | - | - | - | - | - | - | - | - | - | - | - | - | - | - | - | - | - | - | - | - | - | - | - | - | - | - | - | - | - | - | - | - | - | - | - | - | - | -154 |
| A2E1X1_TRIVA | - | - | - | - | - | - | - | - | - | - | - | - | - | - | - | - | - | - | - | - | - | - | - | - | - | - | - | - | - | - | - | - | - | - | - | - | - | - | - | - | - | - | - | - | - | - | - | - | - | - | - | - | - | - | - | - | - | - | - | - | - | -137 |
| TPR_PORGI | - | - | - | - | - | - | - | - | - | - | - | - | - | - | - | - | - | - | - | - | - | - | - | - | - | - | - | - | - | - | - | - | - | - | - | - | - | - | - | - | - | - | - | - | - | - | - | - | - | - | - | - | - | - | - | - | - | - | - | - | - | -170 |
| D5MF42_9BACT | - | - | - | - | - | - | - | - | - | - | - | - | - | - | - | - | - | - | - | - | - | - | - | - | - | - | - | - | - | - | - | - | - | - | - | - | - | - | - | - | - | - | - | - | - | - | - | - | - | - | - | - | - | - | - | - | - | - | - | - | - | -126 |
| R9H254_BACT4 | - | - | - | - | - | - | - | - | - | - | - | - | - | - | - | - | - | - | - | - | - | - | - | - | - | - | - | - | - | - | - | - | - | - | - | - | - | - | - | - | - | - | - | - | - | - | - | - | - | - | - | - | - | - | - | - | - | - | - | - | - | -126 |
| ZP_04847694 | - | - | - | - | - | - | - | - | - | - | - | - | - | - | - | - | - | - | - | - | - | - | - | - | - | - | - | - | - | - | - | - | - | - | - | - | - | - | - | - | - | - | - | - | - | - | - | - | - | - | - | - | - | - | - | - | - | - | - | - | - | -126 |
| D7IBA4_9BACE | - | - | - | - | - | - | - | - | - | - | - | - | - | - | - | - | - | - | - | - | - | - | - | - | - | - | - | - | - | - | - | - | - | - | - | - | - | - | - | - | - | - | - | - | - | - | - | - | - | - | - | - | - | - | - | - | - | - | - | - | - | -124 |
| ZP_09858577 | - | - | - | - | - | - | - | - | - | - | - | - | - | - | - | - | - | - | - | - | - | - | - | - | - | - | - | - | - | - | - | - | - | - | - | - | - | - | - | - | - | - | - | - | - | - | - | - | - | - | - | - | - | - | - | - | - | - | - | - | - | -124 |
| E5C759_9BACE | - | - | - | - | - | - | - | - | - | - | - | - | - | - | - | - | - | - | - | - | - | - | - | - | - | - | - | - | - | - | - | - | - | - | - | - | - | - | - | - | - | - | - | - | - | - | - | - | - | - | - | - | - | - | - | - | - | - | - | - | - | -126 |
| A7LTS0_BACO1 | - | - | - | - | - | - | - | - | - | - | - | - | - | - | - | - | - | - | - | - | - | - | - | - | - | - | - | - | - | - | - | - | - | - | - | - | - | - | - | - | - | - | - | - | - | - | - | - | - | - | - | - | - | - | - | - | - | - | - | - | - | -124 |
| Q8YQK8_NOSS1 | - | - | - | - | - | - | - | - | - | - | - | - | - | - | - | - | - | - | - | - | - | - | - | - | - | - | - | - | - | - | - | - | - | - | - | - | - | - | - | - | - | - | - | - | - | - | - | - | - | - | - | - | - | - | - | - | - | - | - | - | - | -138 |
| Q3MBX8_ANAVT | - | - | - | - | - | - | - | - | - | - | - | - | - | - | - | - | - | - | - | - | - | - | - | - | - | - | - | - | - | - | - | - | - | - | - | - | - | - | - | - | - | - | - | - | - | - | - | - | - | - | - | - | - | - | - | - | - | - | - | - | - | -143 |
| Q8YZT8_NOSS1 | - | - | - | - | - | - | - | - | - | - | - | - | - | - | - | - | - | - | - | - | - | - | - | - | - | - | - | - | - | - | - | - | - | - | - | - | - | - | - | - | - | - | - | - | - | - | - | - | - | - | - | - | - | - | - | - | - | - | - | - | - | -131 |
| K9UIG2_9CHRO | - | - | - | - | - | - | - | - | - | - | - | - | - | - | - | - | - | - | - | - | - | - | - | - | - | - | - | - | - | - | - | - | - | - | - | - | - | - | - | - | - | - | - | - | - | - | - | - | - | - | - | - | - | - | - | - | - | - | - | - | - | -138 |
| A4YRP8_BRASO | - | - | - | - | - | - | - | - | - | - | - | - | - | - | - | - | - | - | - | - | - | - | - | - | - | - | - | - | - | - | - | - | - | - | - | - | - | - | - | - | - | - | - | - | - | - | - | - | - | - | - | - | - | - | - | - | - | - | - | - | - | -152 |
| H0TAL7_9BRAD | - | - | - | - | - | - | - | - | - | - | - | - | - | - | - | - | - | - | - | - | - | - | - | - | - | - | - | - | - | - | - | - | - | - | - | - | - | - | - | - | - | - | - | - | - | - | - | - | - | - | - | - | - | - | - | - | - | - | - | - | - | -156 |
| H0SNL4_9BRAD | - | - | - | - | - | - | - | - | - | - | - | - | - | - | - | - | - | - | - | - | - | - | - | - | - | - | - | - | - | - | - | - | - | - | - | - | - | - | - | - | - | - | - | - | - | - | - | - | - | - | - | - | - | - | - | - | - | - | - | - | - | -158 |
| H0S4P6_9BRAD | - | - | - | - | - | - | - | - | - | - | - | - | - | - | - | - | - | - | - | - | - | - | - | - | - | - | - | - | - | - | - | - | - | - | - | - | - | - | - | - | - | - | - | - | - | - | - | - | - | - | - | - | - | - | - | - | - | - | - | - | - | -158 |
| A5EMK2_BRASB | - | - | - | - | - | - | - | - | - | - | - | - | - | - | - | - | - | - | - | - | - | - | - | - | - | - | - | - | - | - | - | - | - | - | - | - | - | - | - | - | - | - | - | - | - | - | - | - | - | - | - | - | - | - | - | - | - | - | - | - | - | -152 |
| M4ZBJ4_9BRAD | - | - | - | - | - | - | - | - | - | - | - | - | - | - | - | - | - | - | - | - | - | - | - | - | - | - | - | - | - | - | - | - | - | - | - | - | - | - | - | - | - | - | - | - | - | - | - | - | - | - | - | - | - | - | - | - | - | - | - | - | - | -158 |
| Q07SP7_RHOP5 | - | - | - | - | - | - | - | - | - | - | - | - | - | - | - | - | - | - | - | - | - | - | - | - | - | - | - | - | - | - | - | - | - | - | - | - | - | - | - | - | - | - | - | - | - | - | - | - | - | - | - | - | - | - | - | - | - | - | - | - | - | -145 |
| B0JUQ7_MICAN | - | - | - | - | - | - | - | - | - | - | - | - | - | - | - | - | - | - | - | - | - | - | - | - | - | - | - | - | - | - | - | - | - | - | - | - | - | - | - | - | - | - | - | - | - | - | - | - | - | - | - | - | - | - | - | - | - | - | - | - | - | -147 |
| K9WJ87_9CYAN | - | - | - | - | - | - | - | - | - | - | - | - | - | - | - | - | - | - | - | - | - | - | - | - | - | - | - | - | - | - | - | - | - | - | - | - | - | - | - | - | - | - | - | - | - | - | - | - | - | - | - | - | - | - | - | - | - | - | - | - | - | -141 |
| G6FUE0_9CYAN | - | - | - | - | - | - | - | - | - | - | - | - | - | - | - | - | - | - | - | - | - | - | - | - | - | - | - | - | - | - | - | - | - | - | - | - | - | - | - | - | - | - | - | - | - | - | - | - | - | - | - | - | - | - | - | - | - | - | - | - | - | -141 |
| K9PHG5_9CYAN | - | - | - | - | - | - | - | - | - | - | - | - | - | - | - | - | - | - | - | - | - | - | - | - | - | - | - | - | - | - | - | - | - | - | - | - | - | - | - | - | - | - | - | - | - | - | - | - | - | - | - | - | - | - | - | - | - | - | - | - | - | -143 |
| Q8YXX3_NOSS1 | - | - | - | - | - | - | - | - | - | - | - | - | - | - | - | - | - | - | - | - | - | - | - | - | - | - | - | - | - | - | - | - | - | - | - | - | - | - | - | - | - | - | - | - | - | - | - | - | - | - | - | - | - | - | - | - | - | - | - | - | - | -156 |
| K9TN70_9CYAN | - | - | - | - | - | - | - | - | - | - | - | - | - | - | - | - | - | - | - | - | - | - | - | - | - | - | - | - | - | - | - | - | - | - | - | - | - | - | - | - | - | - | - | - | - | - | - | - | - | - | - | - | - | - | - | - | - | - | - | - | - | -156 |
| Q05QW3_9SYNE | - | - | - | - | - | - | - | - | - | - | - | - | - | - | - | - | - | - | - | - | - | - | - | - | - | - | - | - | - | - | - | - | - | - | - | - | - | - | - | - | - | - | - | - | - | - | - | - | - | - | - | - | - | - | - | - | - | - | - | - | - | -162 |
| Q05QW2_9SYNE | - | - | - | - | - | - | - | - | - | - | - | - | - | - | - | - | - | - | - | - | - | - | - | - | - | - | - | - | - | - | - | - | - | - | - | - | - | - | - | - | - | - | - | - | - | - | - | - | - | - | - | - | - | - | - | - | - | - | - | - | - | -159 |
| Q05QW4_9SYNE | - | - | - | - | - | - | - | - | - | - | - | - | - | - | - | - | - | - | - | - | - | - | - | - | - | - | - | - | - | - | - | - | - | - | - | - | - | - | - | - | - | - | - | - | - | - | - | - | - | - | - | - | - | - | - | - | - | - | - | - | - | -153 |
| E0UGX6_CYAP2 | - | - | - | - | - | - | - | - | - | - | - | - | - | - | - | - | - | - | - | - | - | - | - | - | - | - | - | - | - | - | - | - | - | - | - | - | - | - | - | - | - | - | - | - | - | - | - | - | - | - | - | - | - | - | - | - | - | - | - | - | - | -144 |
| K7WKF4_9NOST | - | - | - | - | - | - | - | - | - | - | - | - | - | - | - | - | - | - | - | - | - | - | - | - | - | - | - | - | - | - | - | - | - | - | - | - | - | - | - | - | - | - | - | - | - | - | - | - | - | - | - | - | - | - | - | - | - | - | - | - | - | -143 |
| E0UGY5_CYAP2 | - | - | - | - | - | - | - | - | - | - | - | - | - | - | - | - | - | - | - | - | - | - | - | - | - | - | - | - | - | - | - | - | - | - | - | - | - | - | - | - | - | - | - | - | - | - | - | - | - | - | - | - | - | - | - | - | - | - | - | - | - | -146 |
| A8LCE5_FRASN | - | - | - | - | - | - | - | - | - | - | - | - | - | - | - | - | - | - | - | - | - | - | - | - | - | - | - | - | - | - | - | - | - | - | - | - | - | - | - | - | - | - | - | - | - | - | - | - | - | - | - | - | - | - | - | - | - | - | - | - | - | -137 |
| D3CT47_9ACTO | - | - | - | - | - | - | - | - | - | - | - | - | - | - | - | - | - | - | - | - | - | - | - | - | - | - | - | - | - | - | - | - | - | - | - | - | - | - | - | - | - | - | - | - | - | - | - | - | - | - | - | - | - | - | - | - | - | - | - | - | - | -141 |
| Q2J4J6_FRASC | - | - | - | - | - | - | - | - | - | - | - | - | - | - | - | - | - | - | - | - | - | - | - | - | - | - | - | - | - | - | - | - | - | - | - | - | - | - | - | - | - | - | - | - | - | - | - | - | - | - | - | - | - | - | - | - | - | - | - | - | - | -162 |
| Q0RAZ2_FRAAA | - | - | - | - | - | - | - | - | - | - | - | - | - | - | - | - | - | - | - | - | - | - | - | - | - | - | - | - | - | - | - | - | - | - | - | - | - | - | - | - | - | - | - | - | - | - | - | - | - | - | - | - | - | - | - | - | - | - | - | - | - | -149 |
| E3J3F6_FRASU | - | - | - | - | - | - | - | - | - | - | - | - | - | - | - | - | - | - | - | - | - | - | - | - | - | - | - | - | - | - | - | - | - | - | - | - | - | - | - | - | - | - | - | - | - | - | - | - | - | - | - | - | - | - | - | - | - | - | - | - | - | -143 |
| ZP_09168240 | - | - | - | - | - | - | - | - | - | - | - | - | - | - | - | - | - | - | - | - | - | - | - | - | - | - | - | - | - | - | - | - | - | - | - | - | - | - | - | - | - | - | - | - | - | - | - | - | - | - | - | - | - | - | - | - | - | - | - | - | - | -153 |
| C7MDV9_BRAFD | - | - | - | - | - | - | - | - | - | - | - | - | - | - | - | - | - | - | - | - | - | - | - | - | - | - | - | - | - | - | - | - | - | - | - | - | - | - | - | - | - | - | - | - | - | - | - | - | - | - | - | - | - | - | - | - | - | - | - | - | - | -115 |
| ZP_10094989 | - | - | - | - | - | - | - | - | - | - | - | - | - | - | - | - | - | - | - | - | - | - | - | - | - | - | - | - | - | - | - | - | - | - | - | - | - | - | - | - | - | - | - | - | - | - | - | - | - | - | - | - | - | - | - | - | - | - | - | - | - | -115 |
| A4X268_SALTO | - | - | - | - | - | - | - | - | - | - | - | - | - | - | - | - | - | - | - | - | - | - | - | - | - | - | - | - | - | - | - | - | - | - | - | - | - | - | - | - | - | - | - | - | - | - | - | - | - | - | - | - | - | - | - | - | - | - | - | - | - | -165 |
| WP_043162213 | - | - | - | - | - | - | - | - | - | - | - | - | - | - | - | - | - | - | - | - | - | - | - | - | - | - | - | - | - | - | - | - | - | - | - | - | - | - | - | - | - | - | - | - | - | - | - | - | - | - | - | - | - | - | - | - | - | - | - | - | - | -165 |
| Q0BRV8_GRABC | - | - | - | - | - | - | - | - | - | - | - | - | - | - | - | - | - | - | - | - | - | - | - | - | - | - | - | - | - | - | - | - | - | - | - | - | - | - | - | - | - | - | - | - | - | - | - | - | - | - | - | - | - | - | - | - | - | - | - | - | - | -141 |
| A9B2P0_HERA2 | - | - | - | - | - | - | - | - | - | - | - | - | - | - | - | - | - | - | - | - | - | - | - | - | - | - | - | - | - | - | - | - | - | - | - | - | - | - | - | - | - | - | - | - | - | - | - | - | - | - | - | - | - | - | - | - | - | - | - | - | - | -133 |
| A9B3U8_HERA2 | - | - | - | - | - | - | - | - | - | - | - | - | - | - | - | - | - | - | - | - | - | - | - | - | - | - | - | - | - | - | - | - | - | - | - | - | - | - | - | - | - | - | - | - | - | - | - | - | - | - | - | - | - | - | - | - | - | - | - | - | - | -135 |
| A9B2A3_HERA2 | - | - | - | - | - | - | - | - | - | - | - | - | - | - | - | - | - | - | - | - | - | - | - | - | - | - | - | - | - | - | - | - | - | - | - | - | - | - | - | - | - | - | - | - | - | - | - | - | - | - | - | - | - | - | - | - | - | - | - | - | - | -153 |
| D6ZC85_SEGRD | - | - | - | - | - | - | - | - | - | - | - | - | - | - | - | - | - | - | - | - | - | - | - | - | - | - | - | - | - | - | - | - | - | - | - | - | - | - | - | - | - | - | - | - | - | - | - | - | - | - | - | - | - | - | - | - | - | - | - | - | - | -179 |
| E5XTT9_9ACTO | - | - | - | - | - | - | - | - | - | - | - | - | - | - | - | - | - | - | - | - | - | - | - | - | - | - | - | - | - | - | - | - | - | - | - | - | - | - | - | - | - | - | - | - | - | - | - | - | - | - | - | - | - | - | - | - | - | - | - | - | - | -179 |
| E5XTU0_9ACTO | - | - | - | - | - | - | - | - | - | - | - | - | - | - | - | - | - | - | - | - | - | - | - | - | - | - | - | - | - | - | - | - | - | - | - | - | - | - | - | - | - | - | - | - | - | - | - | - | - | - | - | - | - | - | - | - | - | - | - | - | - | -183 |
| D6B0I6_9ACTO | - | - | - | - | - | - | - | - | - | - | - | - | - | - | - | - | - | - | - | - | - | - | - | - | - | - | - | - | - | - | - | - | - | - | - | - | - | - | - | - | - | - | - | - | - | - | - | - | - | - | - | - | - | - | - | - | - | - | - | - | - | -141 |
| D9X719_STRVR | - | - | - | - | - | - | - | - | - | - | - | - | - | - | - | - | - | - | - | - | - | - | - | - | - | - | - | - | - | - | - | - | - | - | - | - | - | - | - | - | - | - | - | - | - | - | - | - | - | - | - | - | - | - | - | - | - | - | - | - | - | -141 |
| H6L010_SAPGL | - | - | - | - | - | - | - | - | - | - | - | - | - | - | - | - | - | - | - | - | - | - | - | - | - | - | - | - | - | - | - | - | - | - | - | - | - | - | - | - | - | - | - | - | - | - | - | - | - | - | - | - | - | - | - | - | - | - | - | - | - | -127 |
| K1ZP83_9BACT | - | - | - | - | - | - | - | - | - | - | - | - | - | - | - | - | - | - | - | - | - | - | - | - | - | - | - | - | - | - | - | - | - | - | - | - | - | - | - | - | - | - | - | - | - | - | - | - | - | - | - | - | - | - | - | - | - | - | - | - | - | -148 |
| ADGB_HUMAN | - | - | - | - | - | - | - | - | - | - | - | - | - | - | - | - | - | - | - | - | - | - | - | - | - | - | - | - | - | - | - | - | - | - | - | - | - | - | - | - | - | - | - | - | - | - | - | - | - | - | - | - | - | - | - | - | - | - | - | - | - | -186 |
| J3QMP3_MOUSE | - | - | - | - | - | - | - | - | - | - | - | - | - | - | - | - | - | - | - | - | - | - | - | - | - | - | - | - | - | - | - | - | - | - | - | - | - | - | - | - | - | - | - | - | - | - | - | - | - | - | - | - | - | - | - | - | - | - | - | - | - | -186 |
| H2XR46_CIOIN | - | - | - | - | - | - | - | - | - | - | - | - | - | - | - | - | - | - | - | - | - | - | - | - | - | - | - | - | - | - | - | - | - | - | - | - | - | - | - | - | - | - | - | - | - | - | - | - | - | - | - | - | - | - | - | - | - | - | - | - | - | -184 |
| CAN7_MOUSE | - | - | - | - | - | - | - | - | - | - | - | - | - | - | - | - | - | - | - | - | - | - | - | - | - | - | - | - | - | - | - | - | - | - | - | - | - | - | - | - | - | - | - | - | - | - | - | - | - | - | - | - | - | - | - | - | - | - | - | - | - | -156 |
| CAN7_HUMAN | - | - | - | - | - | - | - | - | - | - | - | - | - | - | - | - | - | - | - | - | - | - | - | - | - | - | - | - | - | - | - | - | - | - | - | - | - | - | - | - | - | - | - | - | - | - | - | - | - | - | - | - | - | - | - | - | - | - | - | - | - | -156 |
| E1BY84_CHICK | - | - | - | - | - | - | - | - | - | - | - | - | - | - | - | - | - | - | - | - | - | - | - | - | - | - | - | - | - | - | - | - | - | - | - | - | - | - | - | - | - | - | - | - | - | - | - | - | - | - | - | - | - | - | - | - | - | - | - | - | - | -156 |
| H9GX91_DANRE | - | - | - | - | - | - | - | - | - | - | - | - | - | - | - | - | - | - | - | - | - | - | - | - | - | - | - | - | - | - | - | - | - | - | - | - | - | - | - | - | - | - | - | - | - | - | - | - | - | - | - | - | - | - | - | - | - | - | - | - | - | -156 |
| F6SGI6_CIOIN | - | - | - | - | - | - | - | - | - | - | - | - | - | - | - | - | - | - | - | - | - | - | - | - | - | - | - | - | - | - | - | - | - | - | - | - | - | - | - | - | - | - | - | - | - | - | - | - | - | - | - | - | - | - | - | - | - | - | - | - | - | -154 |
| W4Y195_STRPU | - | - | - | - | - | - | - | - | - | - | - | - | - | - | - | - | - | - | - | - | - | - | - | - | - | - | - | - | - | - | - | - | - | - | - | - | - | - | - | - | - | - | - | - | - | - | - | - | - | - | - | - | - | - | - | - | - | - | - | - | - | -130 |
| R7V270_CAPTE | - | - | - | - | - | - | - | - | - | - | - | - | - | - | - | - | - | - | - | - | - | - | - | - | - | - | - | - | - | - | - | - | - | - | - | - | - | - | - | - | - | - | - | - | - | - | - | - | - | - | - | - | - | - | - | - | - | - | - | - | - | -155 |
| G4LXK3_SCHMA | - | - | - | - | - | - | - | - | - | - | - | - | - | - | - | - | - | - | - | - | - | - | - | - | - | - | - | - | - | - | - | - | - | - | - | - | - | - | - | - | - | - | - | - | - | - | - | - | - | - | - | - | - | - | - | - | - | - | - | - | - | -156 |
| T2M2K0_HYDVU | - | - | - | - | - | - | - | - | - | - | - | - | - | - | - | - | - | - | - | - | - | - | - | - | - | - | - | - | - | - | - | - | - | - | - | - | - | - | - | - | - | - | - | - | - | - | - | - | - | - | - | - | - | - | - | - | - | - | - | - | - | -156 |
| B3RQK1_TRIAD | - | - | - | - | - | - | - | - | - | - | - | - | - | - | - | - | - | - | - | - | - | - | - | - | - | - | - | - | - | - | - | - | - | - | - | - | - | - | - | - | - | - | - | - | - | - | - | - | - | - | - | - | - | - | - | - | - | - | - | - | - | -155 |
| I1CS59_RHIO9 | - | - | - | - | - | - | - | - | - | - | - | - | - | - | - | - | - | - | - | - | - | - | - | - | - | - | - | - | - | - | - | - | - | - | - | - | - | - | - | - | - | - | - | - | - | - | - | - | - | - | - | - | - | - | - | - | - | - | - | - | - | -151 |
| I1GER3_AMPQE | - | - | - | - | - | - | - | - | - | - | - | - | - | - | - | - | - | - | - | - | - | - | - | - | - | - | - | - | - | - | - | - | - | - | - | - | - | - | - | - | - | - | - | - | - | - | - | - | - | - | - | - | - | - | - | - | - | - | - | - | - | -154 |
| Q22143_CAEEL | - | - | - | - | - | - | - | - | - | - | - | - | - | - | - | - | - | - | - | - | - | - | - | - | - | - | - | - | - | - | - | - | - | - | - | - | - | - | - | - | - | - | - | - | - | - | - | - | - | - | - | - | - | - | - | - | - | - | - | - | - | -155 |
| L1JZN7_GUITH | - | - | - | - | - | - | - | - | - | - | - | - | - | - | - | - | - | - | - | - | - | - | - | - | - | - | - | - | - | - | - | - | - | - | - | - | - | - | - | - | - | - | - | - | - | - | - | - | - | - | - | - | - | - | - | - | - | - | - | - | - | -158 |
| D0NT34_PHYIT | - | - | - | - | - | - | - | - | - | - | - | - | - | - | - | - | - | - | - | - | - | - | - | - | - | - | - | - | - | - | - | - | - | - | - | - | - | - | - | - | - | - | - | - | - | - | - | - | - | - | - | - | - | - | - | - | - | - | - | - | - | -166 |
| A0A064B913_ASPOZ | - | - | - | - | - | - | - | - | - | - | - | - | - | - | - | - | - | - | - | - | - | - | - | - | - | - | - | - | - | - | - | - | - | - | - | - | - | - | - | - | - | - | - | - | - | - | - | - | - | - | - | - | - | - | - | - | - | - | - | - | - | -164 |
| A8N4X4_COPC7 | - | - | - | - | - | - | - | - | - | - | - | - | - | - | - | - | - | - | - | - | - | - | - | - | - | - | - | - | - | - | - | - | - | - | - | - | - | - | - | - | - | - | - | - | - | - | - | - | - | - | - | - | - | - | - | - | - | - | - | - | - | -180 |
| C0H5K1_PLAF7 | - | R | N | K | L | Q | K | Q | F | L | K | E | E | L | A | T | M | D | F | A | K | Y | M | N | G | S | V | D | N | Y | S | D | L | I | K | R | G | G | H | K | T | L | G | I | N | C | E | E | G | K | Y | E | P | Y | E | Q | Y | E | Q | Y | E | -312 |
| K1REZ8_CRAGI | - | - | - | - | - | - | - | - | - | - | - | - | - | - | - | - | - | - | - | - | - | - | - | - | - | - | - | - | - | - | - | - | - | - | - | - | - | - | - | - | - | - | - | - | - | - | - | - | - | - | - | - | - | - | - | - | - | - | - | - | - | -145 |
| F4NTM9_BATDJ | - | - | - | - | - | - | - | - | - | - | - | - | - | - | - | - | - | - | - | - | - | - | - | - | - | - | - | - | - | - | - | - | - | - | - | - | - | - | - | - | - | - | - | - | - | - | - | - | - | - | - | - | - | - | - | - | - | - | - | - | - | -167 |
| Q6F6K5_CAEEL | - | - | - | - | - | - | - | - | - | - | - | - | - | - | - | - | - | - | - | - | - | - | - | - | - | - | - | - | - | - | - | - | - | - | - | - | - | - | - | - | - | - | - | - | - | - | - | - | - | - | - | - | - | - | - | - | - | - | - | - | - | -126 |
| A8HZK8_CHLRE | - | - | - | - | - | - | - | - | - | - | - | - | - | - | - | - | - | - | - | - | - | - | - | - | - | - | - | - | - | - | - | - | - | - | - | - | - | - | - | - | - | - | - | - | - | - | - | - | - | - | - | - | - | - | - | - | - | - | - | - | - | -132 |
| A8J6T6_CHLRE | - | - | - | - | - | - | - | - | - | - | - | - | - | - | - | - | - | - | - | - | - | - | - | - | - | - | - | - | - | - | - | - | - | - | - | - | - | - | - | - | - | - | - | - | - | - | - | - | - | - | - | - | - | - | - | - | - | - | - | - | - | -184 |
| Z9JR51_9MICO | - | - | - | - | - | - | - | - | - | - | - | - | - | - | - | - | - | - | - | - | - | - | - | - | - | - | - | - | - | - | - | - | - | - | - | - | - | - | - | - | - | - | - | - | - | - | - | - | - | - | - | - | - | - | - | - | - | - | - | - | - | -144 |
| F7ZZH9_CELGA | - | - | - | - | - | - | - | - | - | - | - | - | - | - | - | - | - | - | - | - | - | - | - | - | - | - | - | - | - | - | - | - | - | - | - | - | - | - | - | - | - | - | - | - | - | - | - | - | - | - | - | - | - | - | - | - | - | - | - | - | - | -126 |
| Q7NDU2_GLOVI | - | - | - | - | - | - | - | - | - | - | - | - | - | - | - | - | - | - | - | - | - | - | - | - | - | - | - | - | - | - | - | - | - | - | - | - | - | - | - | - | - | - | - | - | - | - | - | - | - | - | - | - | - | - | - | - | - | - | - | - | - | -115 |
| I6AT95_9BACT | - | - | - | - | - | - | - | - | - | - | - | - | - | - | - | - | - | - | - | - | - | - | - | - | - | - | - | - | - | - | - | - | - | - | - | - | - | - | - | - | - | - | - | - | - | - | - | - | - | - | - | - | - | - | - | - | - | - | - | - | - | -139 |
| C7MGJ8_BRAFD | - | - | - | - | - | - | - | - | - | - | - | - | - | - | - | - | - | - | - | - | - | - | - | - | - | - | - | - | - | - | - | - | - | - | - | - | - | - | - | - | - | - | - | - | - | - | - | - | - | - | - | - | - | - | - | - | - | - | - | - | - | -142 |
| D0WP73_9ACTO | - | - | - | - | - | - | - | - | - | - | - | - | - | - | - | - | - | - | - | - | - | - | - | - | - | - | - | - | - | - | - | - | - | - | - | - | - | - | - | - | - | - | - | - | - | - | - | - | - | - | - | - | - | - | - | - | - | - | - | - | - | -141 |
| B0C8L1_ACAM1 | - | - | - | - | - | - | - | - | - | - | - | - | - | - | - | - | - | - | - | - | - | - | - | - | - | - | - | - | - | - | - | - | - | - | - | - | - | - | - | - | - | - | - | - | - | - | - | - | - | - | - | - | - | - | - | - | - | - | - | - | - | -132 |
| B3E5A8_GEOLS | - | - | - | - | - | - | - | - | - | - | - | - | - | - | - | - | - | - | - | - | - | - | - | - | - | - | - | - | - | - | - | - | - | - | - | - | - | - | - | - | - | - | - | - | - | - | - | - | - | - | - | - | - | - | - | - | - | - | - | - | - | -135 |
| D4YQ94_9MICO | - | - | - | - | - | - | - | - | - | - | - | - | - | - | - | - | - | - | - | - | - | - | - | - | - | - | - | - | - | - | - | - | - | - | - | - | - | - | - | - | - | - | - | - | - | - | - | - | - | - | - | - | - | - | - | - | - | - | - | - | - | -125 |
| PAPA_CARPA | - | - | - | - | - | - | - | - | - | - | - | - | - | - | - | - | - | - | - | - | - | - | - | - | - | - | - | - | - | - | - | - | - | - | - | - | - | - | - | - | - | - | - | - | - | - | - | - | - | - | - | - | - | - | - | - | - | - | - | - | - | -119 |
| RIM13_YEAST | - | - | - | - | - | - | - | - | - | - | - | - | - | - | - | - | - | - | - | - | - | - | - | - | - | - | - | - | - | - | - | - | - | - | - | - | - | - | - | - | - | - | - | - | - | - | - | - | - | - | - | - | - | - | - | - | - | - | - | - | - | -154 |

| I |  |  |  |  |  |  |  |  |  |  |  |  |  |  |  |  |  |  |  |  |  |  |  |  |  |  |  |  |  |  |  |  |  |  |  |  |  |  |  |  |  |  |  |  |  |  |  |  |  |  |  |  |  |  |  |  |  |  |  |  |  |  |
| --- | --- | --- | --- | --- | --- | --- | --- | --- | --- | --- | --- | --- | --- | --- | --- | --- | --- | --- | --- | --- | --- | --- | --- | --- | --- | --- | --- | --- | --- | --- | --- | --- | --- | --- | --- | --- | --- | --- | --- | --- | --- | --- | --- | --- | --- | --- | --- | --- | --- | --- | --- | --- | --- | --- | --- | --- | --- | --- | --- | --- | --- | --- |
| Q9N4B1_CAEEL | - | - | - | - | - | - | - | - | T | T | L | A | M | L | V | R | G | M | Q | M | G | S | L | F | G | C | S | - | I | D | A | D | E | - | - | - | - | - | - | - | - | - | - | - | - | - | - | - | - | - | - | - | - | - | - | - | - | - | - | - | - | -173 |
| Q8IAA8_CAEEL | - | - | - | - | - | - | - | - | A | V | L | A | A | L | V | K | G | M | E | M | G | S | L | F | G | C | S | - | I | D | A | D | A | - | - | - | - | - | - | - | - | - | - | - | - | - | - | - | - | - | - | - | - | - | - | - | - | - | - | - | - | -173 |
| CAN_CAEEL | - | - | - | - | - | - | - | - | N | L | M | Q | M | M | M | R | G | F | E | M | G | S | L | F | G | C | S | - | I | E | A | D | P | - | - | - | - | - | - | - | - | - | - | - | - | - | - | - | - | - | - | - | - | - | - | - | - | - | - | - | - | -172 |
| Q9U2B2_CAEEL | - | - | - | - | - | - | - | - | A | V | L | A | M | I | M | R | G | V | Q | M | G | S | L | I | V | C | - | - | L | N | I | D | V | - | - | - | - | - | - | - | - | - | - | - | - | - | - | - | - | - | - | - | - | - | - | - | - | - | - | - | - | -176 |
| Q9N4B2_CAEEL | - | - | - | - | - | - | - | - | M | I | L | A | M | I | M | K | G | M | Q | M | G | S | L | F | A | C | S | - | I | D | P | D | P | - | - | - | - | - | - | - | - | - | - | - | - | - | - | - | - | - | - | - | - | - | - | - | - | - | - | - | - | -173 |
| C3Y6P6_BRAFL | - | - | - | - | - | - | - | - | K | F | Y | Q | I | L | K | K | A | H | D | R | G | S | L | M | G | V | S | G | I | K | G | G | T | - | - | - | - | - | - | - | - | - | - | - | - | - | - | - | - | - | - | - | - | - | - | - | - | - | - | - | - | -181 |
| C3Y6N1_BRAFL | - | - | - | - | - | - | - | - | D | L | Y | K | I | L | Q | K | A | F | E | R | G | S | T | M | G | C | S | G | I | K | G | G | S | - | - | - | - | - | - | - | - | - | - | - | - | - | - | - | - | - | - | - | - | - | - | - | - | - | - | - | - | -183 |
| CANA_DROME | - | - | - | - | - | - | - | - | N | L | F | T | I | L | Q | K | A | A | E | R | N | S | M | M | G | C | S | - | I | E | P | D | P | - | - | - | - | - | - | - | - | - | - | - | - | - | - | - | - | - | - | - | - | - | - | - | - | - | - | - | - | -172 |
| CANB_DROME | - | - | - | - | - | - | - | - | N | L | F | S | I | M | M | K | A | A | E | R | G | S | M | M | G | C | S | - | L | E | P | D | P | - | - | - | - | - | - | - | - | - | - | - | - | - | - | - | - | - | - | - | - | - | - | - | - | - | - | - | - | -172 |
| X1Y3H71_ANODA | - | - | - | - | - | - | - | - | N | L | F | E | I | I | E | K | G | F | R | R | N | S | M | F | G | C | S | - | I | E | A | D | P | - | - | - | - | - | - | - | - | - | - | - | - | - | - | - | - | - | - | - | - | - | - | - | - | - | - | - | - | -173 |
| Q5IW12_GECLA | - | - | - | - | - | - | - | - | N | L | F | N | I | M | L | K | A | Y | Q | R | G | S | L | M | G | C | S | - | I | D | P | D | P | - | - | - | - | - | - | - | - | - | - | - | - | - | - | - | - | - | - | - | - | - | - | - | - | - | - | - | - | -172 |
| K1Q056_CRAGI | - | - | - | - | - | - | - | - | N | L | F | S | I | M | L | K | A | S | Q | R | G | S | L | M | G | C | S | - | I | D | A | D | P | - | - | - | - | - | - | - | - | - | - | - | - | - | - | - | - | - | - | - | - | - | - | - | - | - | - | - | - | -172 |
| G4V6C0_SCHMA | - | - | - | - | - | - | - | - | N | L | F | G | I | M | E | H | A | L | N | R | A | S | L | M | A | C | S | - | I | D | A | D | P | - | - | - | - | - | - | - | - | - | - | - | - | - | - | - | - | - | - | - | - | - | - | - | - | - | - | - | - | -177 |
| R7ULX9_CAPTE | - | - | - | - | - | - | - | - | D | L | F | K | I | M | A | K | A | H | S | K | R | N | L | L | G | C | S | - | I | D | A | T | P | - | - | - | - | - | - | - | - | - | - | - | - | - | - | - | - | - | - | - | - | - | - | - | - | - | - | - | - | -173 |
| W4Z5Q7_STRPU | - | - | - | - | - | - | - | - | G | L | F | K | I | L | K | S | S | Y | D | R | D | S | Q | L | G | C | S | - | I | N | A | K | P | - | - | - | - | - | - | - | - | - | - | - | - | - | - | - | - | - | - | - | - | - | - | - | - | - | - | - | - | -170 |
| R7VIB6_CAPTE | - | - | - | - | - | - | - | - | D | L | F | N | I | M | K | K | C | Y | E | R | Q | S | L | L | C | T | S | - | I | D | V | S | R | - | - | - | - | - | - | - | - | - | - | - | - | - | - | - | - | - | - | - | - | - | - | - | - | - | - | - | - | -176 |
| T2MJ45_HYDVU | - | - | - | - | - | - | - | - | R | F | F | K | L | L | Q | K | A | Q | E | R | Q | S | L | M | C | C | S | - | I | E | A | A | P | - | - | - | - | - | - | - | - | - | - | - | - | - | - | - | - | - | - | - | - | - | - | - | - | - | - | - | - | -172 |
| R7UAB6_CAPTE | - | - | - | - | - | - | - | - | N | L | F | Q | I | M | L | K | A | Y | E | R | N | S | Q | M | G | C | S | - | I | D | A | D | P | - | - | - | - | - | - | - | - | - | - | - | - | - | - | - | - | - | - | - | - | - | - | - | - | - | - | - | - | -172 |
| C3ZKN8_BRAFL | - | - | - | - | - | - | - | - | D | L | L | K | I | V | E | K | A | M | M | R | N | S | L | M | G | C | S | - | V | E | A | D | P | - | - | - | - | - | - | - | - | - | - | - | - | - | - | - | - | - | - | - | - | - | - | - | - | - | - | - | - | -173 |
| B3RJS5_TRIAD | - | - | - | - | - | - | - | - | N | L | F | D | Q | I | L | K | S | F | E | R | H | S | L | M | G | C | S | - | I | E | A | S | P | - | - | - | - | - | - | - | - | - | - | - | - | - | - | - | - | - | - | - | - | - | - | - | - | - | - | - | - | -172 |
| H2YVF0_CIOSA | - | - | - | - | - | - | - | - | N | L | Y | S | V | M | H | K | S | L | K | K | G | A | L | M | G | C | A | - | V | E | T | T | S | K | - | - | - | - | - | - | - | - | - | - | - | - | - | - | - | - | - | - | - | - | - | - | - | - | - | - | - | -173 |
| G4VAG2_SCHMA | - | - | - | - | - | - | - | - | D | L | I | D | Q | L | K | I | Y | A | Q | R | C | C | L | M | G | C | S | - | I | D | S | S | V | - | - | - | - | - | - | - | - | - | - | - | - | - | - | - | - | - | - | - | - | - | - | - | - | - | - | - | - | -177 |
| Smp_157500 | - | - | - | - | - | - | - | - | N | L | L | K | M | M | E | H | Y | T | K | T | C | C | V | M | G | A | D | - | V | G | G | D | K | - | - | - | - | - | - | - | - | - | - | - | - | - | - | - | - | - | - | - | - | - | - | - | - | - | - | - | - | -177 |
| Q7SYA9_DANRE | - | - | - | - | - | - | - | - | D | L | Y | R | I | I | S | K | A | L | D | R | G | S | L | L | G | C | S | I | D | I | T | S | - | - | - | - | - | - | - | - | - | - | - | - | - | - | - | - | - | - | - | - | - | - | - | - | - | - | - | - | - | -172 |
| A5PMP1_DANRE | - | - | - | - | - | - | - | - | D | L | H | R | I | I | A | K | A | L | E | R | G | S | L | L | G | C | S | I | D | I | T | S | - | - | - | - | - | - | - | - | - | - | - | - | - | - | - | - | - | - | - | - | - | - | - | - | - | - | - | - | - | -172 |
| CANX_CHICK | - | - | - | - | - | - | - | - | N | M | G | H | I | I | R | K | A | L | E | R | G | S | L | L | G | C | S | I | D | I | T | S | - | - | - | - | - | - | - | - | - | - | - | - | - | - | - | - | - | - | - | - | - | - | - | - | - | - | - | - | - | -172 |
| CAN1_HUMAN | - | - | - | - | - | - | - | - | D | L | Y | Q | I | I | L | K | A | L | E | R | G | S | L | L | G | C | S | I | D | I | S | S | - | - | - | - | - | - | - | - | - | - | - | - | - | - | - | - | - | - | - | - | - | - | - | - | - | - | - | - | - | -172 |
| CAN1_MOUSE | - | - | - | - | - | - | - | - | D | L | Y | Q | I | I | L | K | A | L | E | R | G | S | L | L | G | C | S | I | N | I | S | D | - | - | - | - | - | - | - | - | - | - | - | - | - | - | - | - | - | - | - | - | - | - | - | - | - | - | - | - | - | -172 |
| O42133_CHICK | - | - | - | - | - | - | - | - | D | L | Y | Q | I | I | L | K | A | L | E | R | G | S | L | L | G | C | S | I | D | I | T | S | - | - | - | - | - | - | - | - | - | - | - | - | - | - | - | - | - | - | - | - | - | - | - | - | - | - | - | - | - | -172 |
| Q7ZUR1_DANRE | - | - | - | - | - | - | - | - | D | L | F | S | I | I | G | R | A | I | E | R | G | S | L | L | G | C | S | I | D | I | T | S | - | - | - | - | - | - | - | - | - | - | - | - | - | - | - | - | - | - | - | - | - | - | - | - | - | - | - | - | - | -172 |
| E9QFN0_DANRE | - | - | - | - | - | - | - | - | N | L | F | S | I | I | T | R | A | V | E | R | G | S | L | M | G | C | S | I | D | I | T | S | - | - | - | - | - | - | - | - | - | - | - | - | - | - | - | - | - | - | - | - | - | - | - | - | - | - | - | - | - | -172 |
| CAN2_HUMAN | - | - | - | - | - | - | - | - | N | L | F | K | I | I | Q | K | A | L | Q | K | G | S | L | L | G | C | S | I | D | I | T | S | - | - | - | - | - | - | - | - | - | - | - | - | - | - | - | - | - | - | - | - | - | - | - | - | - | - | - | - | - | -172 |
| CAN2_MOUSE | - | - | - | - | - | - | - | - | N | L | F | K | I | I | Q | K | A | L | E | K | G | S | L | L | G | C | S | I | D | I | T | S | - | - | - | - | - | - | - | - | - | - | - | - | - | - | - | - | - | - | - | - | - | - | - | - | - | - | - | - | - | -172 |
| CAN2_CHICK | - | - | - | - | - | - | - | - | N | L | F | K | I | I | Q | K | A | L | Q | K | G | S | L | L | G | C | S | I | D | I | T | S | - | - | - | - | - | - | - | - | - | - | - | - | - | - | - | - | - | - | - | - | - | - | - | - | - | - | - | - | - | -172 |
| Q6DFZ8_DANRE | - | - | - | - | - | - | - | - | N | L | F | K | T | I | Q | K | A | L | S | W | G | S | L | L | G | C | S | I | D | I | T | S | - | - | - | - | - | - | - | - | - | - | - | - | - | - | - | - | - | - | - | - | - | - | - | - | - | - | - | - | - | -172 |
| Q504F5_DANRE | - | - | - | - | - | - | - | - | N | L | F | Q | I | I | Q | K | A | L | E | A | G | A | L | L | G | C | S | I | D | I | T | S | - | - | - | - | - | - | - | - | - | - | - | - | - | - | - | - | - | - | - | - | - | - | - | - | - | - | - | - | - | -172 |
| Q5BLH5_DANRE | - | - | - | - | - | - | - | - | N | L | F | Q | I | I | K | K | A | L | D | S | G | A | L | L | G | C | S | I | D | I | T | S | - | - | - | - | - | - | - | - | - | - | - | - | - | - | - | - | - | - | - | - | - | - | - | - | - | - | - | - | - | -172 |
| Q4VBH3_DANRE | - | - | - | - | - | - | - | - | N | L | F | K | L | V | Q | K | A | L | A | L | G | S | L | L | G | C | S | I | D | I | T | S | - | - | - | - | - | - | - | - | - | - | - | - | - | - | - | - | - | - | - | - | - | - | - | - | - | - | - | - | - | -172 |
| CAN8_RAT | - | - | - | - | - | - | - | - | N | L | Y | Y | I | I | Q | K | A | L | R | K | G | S | L | L | G | C | S | I | D | V | S | T | - | - | - | - | - | - | - | - | - | - | - | - | - | - | - | - | - | - | - | - | - | - | - | - | - | - | - | - | - | -172 |
| CAN8_MOUSE | - | - | - | - | - | - | - | - | N | L | Y | Y | T | I | Q | K | A | L | R | K | G | S | L | L | G | C | S | I | D | V | S | N | - | - | - | - | - | - | - | - | - | - | - | - | - | - | - | - | - | - | - | - | - | - | - | - | - | - | - | - | - | -172 |
| CAN8_HUMAN | - | - | - | - | - | - | - | - | N | L | Y | Q | I | I | R | K | A | L | C | A | G | S | L | L | G | C | S | I | D | V | S | S | - | - | - | - | - | - | - | - | - | - | - | - | - | - | - | - | - | - | - | - | - | - | - | - | - | - | - | - | - | -172 |
| XP_426117 | - | - | - | - | - | - | - | - | N | L | F | Q | I | V | Q | K | A | L | R | A | G | S | L | L | G | C | S | I | D | I | T | R | - | - | - | - | - | - | - | - | - | - | - | - | - | - | - | - | - | - | - | - | - | - | - | - | - | - | - | - | - | -172 |
| CAN9_MOUSE | - | - | - | - | - | - | - | - | D | F | F | E | I | L | E | K | A | L | K | R | G | S | L | L | G | C | S | I | D | T | L | N | - | - | - | - | - | - | - | - | - | - | - | - | - | - | - | - | - | - | - | - | - | - | - | - | - | - | - | - | - | -174 |
| CAN9_HUMAN | - | - | - | - | - | - | - | - | N | F | Y | E | I | L | E | K | A | L | K | R | G | S | L | L | G | C | F | I | D | T | R | S | - | - | - | - | - | - | - | - | - | - | - | - | - | - | - | - | - | - | - | - | - | - | - | - | - | - | - | - | - | -172 |
| E1C6M3_CHICK | - | - | - | - | - | - | - | - | N | F | Y | E | I | L | E | K | A | L | K | R | S | S | M | V | G | C | S | I | D | T | S | S | - | - | - | - | - | - | - | - | - | - | - | - | - | - | - | - | - | - | - | - | - | - | - | - | - | - | - | - | - | -172 |
| Q6DG44_DANRE | - | - | - | - | - | - | - | - | N | L | F | L | I | L | K | K | A | I | E | R | G | S | M | L | G | C | S | I | D | I | T | S | - | - | - | - | - | - | - | - | - | - | - | - | - | - | - | - | - | - | - | - | - | - | - | - | - | - | - | - | - | -172 |
| CAN3_HUMAN | - | - | - | - | - | - | - | - | D | M | Y | K | I | M | K | K | A | I | E | R | G | S | L | M | G | C | S | I | D | D | G | T | N | M | T | Y | G | T | S | P | S | G | L | N | M | G | E | L | I | A | R | M | V | R | N | M | D | N | S | L | L | -201 |
| CAN3_MOUSE | - | - | - | - | - | - | - | - | D | M | Y | K | I | M | R | K | A | I | E | R | G | S | L | M | G | C | S | I | D | D | G | T | N | M | T | Y | G | T | S | P | S | G | L | N | M | G | E | L | I | A | R | M | V | R | N | M | D | N | S | L | L | -201 |
| CAN3_CHICK | - | - | - | - | - | - | - | - | D | I | Y | K | I | M | K | H | A | I | A | R | G | S | L | M | A | S | S | I | D | D | N | L | G | F | H | Y | G | A | A | P | R | - | S | D | I | G | E | L | I | A | R | M | V | K | N | L | E | N | A | Q | M | -200 |
| Q66HU4_DANRE | - | - | - | - | - | - | - | - | E | L | Y | K | I | M | Q | K | A | L | E | R | G | S | L | M | G | C | S | I | D | S | - | - | - | - | - | - | - | - | - | - | - | - | - | - | - | - | - | - | - | - | - | - | - | - | - | - | - | - | - | - | - | -170 |
| F1R632_DANRE | - | - | - | - | - | - | - | - | E | L | Y | N | I | M | R | K | A | L | K | R | G | S | L | M | G | C | A | I | D | T | - | - | - | - | - | - | - | - | - | - | - | - | - | - | - | - | - | - | - | - | - | - | - | - | - | - | - | - | - | - | - | -170 |
| CAN11_HUMAN | - | - | - | - | - | - | - | - | N | L | L | R | L | L | R | K | A | V | E | R | S | S | L | M | G | C | S | I | E | V | T | S | - | - | - | - | - | - | - | - | - | - | - | - | - | - | - | - | - | - | - | - | - | - | - | - | - | - | - | - | - | -172 |
| CAN11_MOUSE | - | - | - | - | - | - | - | - | N | M | L | R | L | L | K | K | A | L | E | K | S | S | L | M | G | C | S | I | E | V | T | D | - | - | - | - | - | - | - | - | - | - | - | - | - | - | - | - | - | - | - | - | - | - | - | - | - | - | - | - | - | -172 |
| CAN12_MOUSE | - | - | - | - | - | - | - | - | G | V | F | A | A | L | R | H | A | L | A | K | E | S | L | V | G | A | T | - | - | - | - | - | - | - | - | - | - | - | - | - | - | - | - | - | - | - | - | - | - | - | - | - | - | - | - | - | - | - | - | - | - | -167 |
| CAN12_HUMAN | - | - | - | - | - | - | - | - | G | L | F | S | A | L | R | H | A | L | A | K | E | S | L | V | G | A | T | - | - | - | - | - | - | - | - | - | - | - | - | - | - | - | - | - | - | - | - | - | - | - | - | - | - | - | - | - | - | - | - | - | - | -167 |
| ENSSARP00000008135 | - | - | - | - | - | - | - | - | X | X | X | X | X | X | X | X | X | X | X | X | X | X | X | X | X | X | X | - | - | - | - | - | - | - | - | - | - | - | - | - | - | - | - | - | - | - | - | - | - | - | - | - | - | - | - | - | - | - | - | - | - | -167 |
| ENSSARP00000010553 | - | - | - | - | - | - | - | - | X | X | X | X | X | X | X | X | X | X | X | X | X | X | X | X | X | X | X | X | F | H | K | G | D | V | L | - | - | - | - | - | - | - | - | - | - | - | - | - | - | - | - | - | - | - | - | - | - | - | - | - | - | -174 |
| A7E2G3_DANRE | - | - | - | - | - | - | - | - | M | L | W | K | A | I | T | A | A | L | A | R | N | S | P | L | S | C | F | I | H | A | T | - | - | - | - | - | - | - | - | - | - | - | - | - | - | - | - | - | - | - | - | - | - | - | - | - | - | - | - | - | - | -172 |
| Q6NW69_DANRE | - | - | - | - | - | - | - | - | A | L | G | S | F | L | R | S | L | L | E | K | G | A | L | I | N | C | A | N | S | Q | G | P | - | - | - | - | - | - | - | - | - | - | - | - | - | - | - | - | - | - | - | - | - | - | - | - | - | - | - | - | - | -172 |
| CAN13_HUMAN | - | - | - | - | - | - | - | - | D | L | V | K | A | V | K | T | A | T | K | A | G | S | L | I | T | C | A | T | - | - | P | S | - | - | - | - | - | - | - | - | - | - | - | - | - | - | - | - | - | - | - | - | - | - | - | - | - | - | - | - | - | -171 |
| CAN13_MOUSE | - | - | - | - | - | - | - | - | D | L | L | T | T | V | K | T | A | I | Q | A | G | S | M | V | A | C | A | T | - | - | E | N | - | - | - | - | - | - | - | - | - | - | - | - | - | - | - | - | - | - | - | - | - | - | - | - | - | - | - | - | - | -173 |
| E1BRR5_CHICK | - | - | - | - | - | - | - | - | D | L | E | D | V | L | K | A | A | S | K | S | R | C | L | M | G | C | S | T | - | - | S | G | - | - | - | - | - | - | - | - | - | - | - | - | - | - | - | - | - | - | - | - | - | - | - | - | - | - | - | - | - | -170 |
| E7F0H3_DANRE | - | - | - | - | - | - | - | - | D | L | W | K | I | M | E | C | A | F | I | S | K | T | L | I | G | C | G | S | - | - | N | S | - | - | - | - | - | - | - | - | - | - | - | - | - | - | - | - | - | - | - | - | - | - | - | - | - | - | - | - | - | -171 |
| Q1RLS9_DANRE | - | - | - | - | - | - | - | - | K | L | W | D | I | M | R | R | A | G | Q | S | E | S | L | M | G | C | G | T | P | G | G | Q | - | - | - | - | - | - | - | - | - | - | - | - | - | - | - | - | - | - | - | - | - | - | - | - | - | - | - | - | - | -172 |
| F1Q9Z1_DANRE | - | - | - | - | - | - | - | - | D | L | W | E | I | M | Y | R | A | S | Q | S | E | V | L | M | G | C | E | S | - | - | - | P | - | - | - | - | - | - | - | - | - | - | - | - | - | - | - | - | - | - | - | - | - | - | - | - | - | - | - | - | - | -170 |
| A4QNV6_DANRE | - | - | - | - | - | - | - | - | D | L | W | S | L | M | D | R | A | A | K | C | K | A | L | M | A | C | G | T | - | - | Y | P | - | - | - | - | - | - | - | - | - | - | - | - | - | - | - | - | - | - | - | - | - | - | - | - | - | - | - | - | - | -171 |
| D6W573_HUMAN | - | - | - | - | - | - | - | - | N | L | W | D | I | L | I | E | A | T | Y | N | R | T | L | I | G | C | Q | T | - | - | - | - | - | - | - | - | - | - | - | - | - | - | - | - | - | - | - | - | - | - | - | - | - | - | - | - | - | - | - | - | - | -169 |
| R7UBC6_CAPTE | - | - | - | - | - | - | - | - | E | F | T | N | V | M | F | K | A | L | E | R | Q | S | L | V | G | C | I | I | N | S | - | - | - | - | - | - | - | - | - | - | - | - | - | - | - | - | - | - | - | - | - | - | - | - | - | - | - | - | - | - | - | -178 |
| K1RCZ2_CRAGI | - | - | - | - | - | - | - | - | N | I | V | N | I | L | F | K | A | L | D | R | Q | S | L | I | G | C | G | I | N | - | - | - | - | - | - | - | - | - | - | - | - | - | - | - | - | - | - | - | - | - | - | - | - | - | - | - | - | - | - | - | - | -180 |
| Q5IW13_GECLA | - | - | - | - | - | - | - | - | N | L | F | P | R | L | L | K | A | C | R | R | G | S | L | I | G | C | A | I | E | S | - | - | - | - | - | - | - | - | - | - | - | - | - | - | - | - | - | - | - | - | - | - | - | - | - | - | - | - | - | - | - | -170 |
| R7UUX7_CAPTE | - | - | - | - | - | - | - | - | N | L | F | Q | L | M | Y | R | T | L | R | M | N | S | M | V | G | C | A | I | N | - | - | - | - | - | - | - | - | - | - | - | - | - | - | - | - | - | - | - | - | - | - | - | - | - | - | - | - | - | - | - | - | -172 |
| K1Q505_CRAGI | - | - | - | - | - | - | - | - | D | I | Y | E | I | L | W | R | S | W | K | M | N | S | F | L | G | C | S | I | S | L | P | Q | - | - | - | - | - | - | - | - | - | - | - | - | - | - | - | - | - | - | - | - | - | - | - | - | - | - | - | - | - | -176 |
| R7UD02_CAPTE | - | - | - | - | - | - | - | - | K | F | F | K | L | I | K | R | L | H | K | R | G | S | L | I | C | S | A | I | E | T | G | - | - | - | - | - | - | - | - | - | - | - | - | - | - | - | - | - | - | - | - | - | - | - | - | - | - | - | - | - | - | -174 |
| CAN6_HUMAN | - | - | - | - | - | - | - | Y | K | L | F | G | E | L | Y | K | T | F | T | K | G | G | L | I | C | C | S | I | E | S | P | N | Q | - | - | - | - | - | - | - | - | - | - | - | - | - | - | - | - | - | - | - | - | - | - | - | - | - | - | - | - | -187 |
| CAN6_MOUSE | - | - | - | - | - | - | - | Y | K | L | F | G | E | L | Y | K | T | F | T | K | G | G | L | I | C | C | S | I | E | S | P | S | Q | - | - | - | - | - | - | - | - | - | - | - | - | - | - | - | - | - | - | - | - | - | - | - | - | - | - | - | - | -187 |
| E1C312_CHICK | - | - | - | - | - | - | - | M | K | L | F | E | D | L | L | K | V | H | K | R | G | G | L | I | S | C | F | I | A | S | S | S | G | - | - | - | - | - | - | - | - | - | - | - | - | - | - | - | - | - | - | - | - | - | - | - | - | - | - | - | - | -187 |
| E7FDN3_DANRE | - | - | - | - | - | - | - | K | I | L | F | Q | T | L | A | H | A | H | S | H | K | A | L | I | T | C | S | I | R | P | A | E | G | - | - | - | - | - | - | - | - | - | - | - | - | - | - | - | - | - | - | - | - | - | - | - | - | - | - | - | - | -188 |
| CAN5_HUMAN | - | - | - | - | - | - | - | N | Q | L | F | E | R | M | L | K | V | H | S | R | G | G | L | I | S | A | S | I | K | A | V | T | A | - | - | - | - | - | - | - | - | - | - | - | - | - | - | - | - | - | - | - | - | - | - | - | - | - | - | - | - | -183 |
| CAN5_MOUSE | - | - | - | - | - | - | - | N | Q | L | F | E | R | V | L | K | V | H | S | R | G | G | L | I | S | A | S | I | K | A | V | T | A | - | - | - | - | - | - | - | - | - | - | - | - | - | - | - | - | - | - | - | - | - | - | - | - | - | - | - | - | -187 |
| E1C292_CHICK | - | - | - | - | - | - | - | N | L | L | F | E | R | V | L | K | V | H | N | R | G | G | L | I | S | C | S | I | K | A | M | S | A | - | - | - | - | - | - | - | - | - | - | - | - | - | - | - | - | - | - | - | - | - | - | - | - | - | - | - | - | -187 |
| A0JMG3_DANRE | - | - | - | - | - | - | - | H | Q | L | F | D | R | V | L | K | V | H | N | R | G | G | L | I | S | C | S | I | R | A | T | T | Q | - | - | - | - | - | - | - | - | - | - | - | - | - | - | - | - | - | - | - | - | - | - | - | - | - | - | - | - | -187 |
| E7F7F1_DANRE | - | - | - | - | - | - | - | N | Q | L | F | E | R | V | L | K | V | H | N | R | D | G | L | I | S | C | S | I | R | A | T | T | V | - | - | - | - | - | - | - | - | - | - | - | - | - | - | - | - | - | - | - | - | - | - | - | - | - | - | - | - | -187 |
| XP_006824412 | - | - | - | - | - | - | - | N | V | L | F | K | E | M | Q | K | C | L | D | R | N | S | L | M | S | S | S | I | K | V | T | S | A | - | - | - | - | - | - | - | - | - | - | - | - | - | - | - | - | - | - | - | - | - | - | - | - | - | - | - | - | -179 |
| W4YJN2_STRPU | - | - | - | - | - | - | - | K | E | L | H | S | Q | M | K | K | Y | S | E | R | R | F | Q | M | S | A | S | I | K | V | T | N | A | - | - | - | - | - | - | - | - | - | - | - | - | - | - | - | - | - | - | - | - | - | - | - | - | - | - | - | - | -187 |
| C3YJZ3_BRAFL | - | - | - | - | - | - | - | E | K | L | F | K | V | M | H | K | A | A | E | R | G | S | L | L | T | C | S | I | R | V | T | S | R | - | - | - | - | - | - | - | - | - | - | - | - | - | - | - | - | - | - | - | - | - | - | - | - | - | - | - | - | -187 |
| T2MIT4_HYDVU | - | - | - | - | - | - | - | M | A | L | H | A | K | L | R | K | L | M | E | R | K | S | M | S | S | A | S | I | K | V | T | N | R | - | - | - | - | - | - | - | - | - | - | - | - | - | - | - | - | - | - | - | - | - | - | - | - | - | - | - | - | -187 |
| XP_002154722 | - | - | - | - | - | - | - | K | L | L | H | V | K | L | S | K | L | M | S | R | K | S | M | I | S | A | S | I | Q | V | Q | N | R | - | - | - | - | - | - | - | - | - | - | - | - | - | - | - | - | - | - | - | - | - | - | - | - | - | - | - | - | -151 |
| R7VLA7_CAPTE | - | - | - | - | - | - | - | N | D | L | Y | K | V | M | R | K | A | M | D | N | H | A | M | M | A | A | A | I | P | S | A | T | K | - | - | - | - | - | - | - | - | - | - | - | - | - | - | - | - | - | - | - | - | - | - | - | - | - | - | - | - | -187 |
| K1R1C6_CRAGI | - | - | - | - | - | - | - | A | A | L | F | T | R | M | Q | K | E | A | G | R | K | A | L | M | A | A | S | I | P | A | E | S | S | - | - | - | - | - | - | - | - | - | - | - | - | - | - | - | - | - | - | - | - | - | - | - | - | - | - | - | - | -187 |
| B3RIE5_TRIAD | - | - | - | - | - | - | - | D | Q | L | F | K | L | M | D | K | S | M | D | R | H | A | L | I | C | C | S | I | R | T | A | D | Q | - | - | - | - | - | - | - | - | - | - | - | - | - | - | - | - | - | - | - | - | - | - | - | - | - | - | - | - | -185 |
| K1PW75_CRAGI | - | - | - | - | - | - | - | L | K | L | F | K | I | L | R | K | C | K | E | H | H | S | L | I | S | A | A | I | P | V | T | S | A | - | - | - | - | - | - | - | - | - | - | - | - | - | - | - | - | - | - | - | - | - | - | - | - | - | - | - | - | -183 |
| K1RSB8_CRAGI | - | - | - | - | - | - | - | K | K | L | F | K | I | L | R | K | S | K | E | N | M | S | L | I | S | T | S | I | A | A | V | G | - | - | - | - | - | - | - | - | - | - | - | - | - | - | - | - | - | - | - | - | - | - | - | - | - | - | - | - | - | -181 |
| K1RWI3_CRAGI | - | - | - | - | - | - | - | E | K | L | F | K | I | L | K | S | S | I | K | D | G | S | L | I | S | A | S | I | S | A | K | G | - | - | - | - | - | - | - | - | - | - | - | - | - | - | - | - | - | - | - | - | - | - | - | - | - | - | - | - | - | -184 |
| R7VGC2_CAPTE | - | - | - | - | - | - | - | E | G | L | Y | E | D | V | H | F | A | S | N | N | K | A | L | V | T | A | S | I | L | C | S | N | P | - | - | - | - | - | - | - | - | - | - | - | - | - | - | - | - | - | - | - | - | - | - | - | - | - | - | - | - | -185 |
| K1QU26_CRAGI | - | - | - | - | - | - | - | D | Q | L | F | Y | K | L | K | E | S | C | D | N | S | A | L | M | N | C | N | I | E | C | Q | K | T | - | - | - | - | - | - | - | - | - | - | - | - | - | - | - | - | - | - | - | - | - | - | - | - | - | - | - | - | -187 |
| CAN5_CAEEL | - | - | - | - | - | - | - | L | K | L | F | N | D | L | K | T | A | F | D | K | G | A | L | V | V | A | A | I | A | A | R | T | K | - | - | - | - | - | - | - | - | - | - | - | - | - | - | - | - | - | - | - | - | - | - | - | - | - | - | - | - | -185 |
| I1FGV4_AMPQE | - | - | - | - | - | - | - | A | T | L | F | D | T | I | T | R | N | A | T | R | S | C | L | M | S | C | S | I | K | I | E | G | R | - | - | - | - | - | - | - | - | - | - | - | - | - | - | - | - | - | - | - | - | - | - | - | - | - | - | - | - | -173 |
| K1PHX3_CRAGI | - | - | - | - | - | - | - | - | L | I | Q | R | L | L | D | V | S | M | T | N | A | S | L | V | G | T | Y | I | S | T | N | M | T | - | - | - | - | - | - | - | - | - | - | - | - | - | - | - | - | - | - | - | - | - | - | - | - | - | - | - | - | -173 |
| B3RNL8_TRIAD | - | - | - | - | - | - | - | D | G | F | Q | R | I | F | N | - | A | L | A | R | H | S | L | L | G | C | F | G | V | T | Q | L | R | - | - | - | - | - | - | - | - | - | - | - | - | - | - | - | - | - | - | - | - | - | - | - | - | - | - | - | - | -175 |
| X1Y3H7_ANODA | - | - | - | - | - | - | - | L | D | L | F | D | R | L | E | Q | C | M | A | R | N | T | L | V | T | A | G | I | N | D | V | E | T | - | - | - | - | - | - | - | - | - | - | - | - | - | - | - | - | - | - | - | - | - | - | - | - | - | - | - | - | -178 |
| R7TWH9_CAPTE | - | - | - | - | - | - | - | - | N | M | Y | R | Y | L | L | R | A | H | K | A | G | S | F | V | A | C | S | R | K | G | D | W | R | - | - | - | - | - | - | - | - | - | - | - | - | - | - | - | - | - | - | - | - | - | - | - | - | - | - | - | - | -177 |
| K1R5E8_CRAGI | - | - | - | - | - | - | - | - | F | L | Y | R | M | I | E | K | A | Q | Q | K | G | A | F | I | T | C | S | R | K | G | N | W | K | - | - | - | - | - | - | - | - | - | - | - | - | - | - | - | - | - | - | - | - | - | - | - | - | - | - | - | - | -177 |
| XP_006811804 | - | - | - | - | - | - | - | - | G | L | F | R | Q | L | L | R | N | F | K | Y | G | S | F | L | T | C | N | K | K | G | D | W | R | - | - | - | - | - | - | - | - | - | - | - | - | - | - | - | - | - | - | - | - | - | - | - | - | - | - | - | - | -175 |
| C3YB23_BRAFL | - | - | - | - | - | - | - | - | N | L | Y | Q | I | M | R | R | A | F | K | K | R | A | F | F | T | T | S | K | K | T | T | Q | A | - | - | - | - | - | - | - | - | - | - | - | - | - | - | - | - | - | - | - | - | - | - | - | - | - | - | - | - | -182 |
| H2ZIF1_CIOSA | - | - | - | - | - | - | - | - | N | L | F | R | I | M | T | K | A | L | R | N | D | S | F | V | C | G | G | I | Q | G | T | T | E | - | - | - | - | - | - | - | - | - | - | - | - | - | - | - | - | - | - | - | - | - | - | - | - | - | - | - | - | -175 |
| W4YV93_STRPU | - | - | - | - | - | - | - | - | D | V | Y | G | I | L | Y | R | G | T | R | S | G | A | F | M | C | C | S | R | K | G | N | W | R | - | - | - | - | - | - | - | - | - | - | - | - | - | - | - | - | - | - | - | - | - | - | - | - | - | - | - | - | -175 |
| CAN10_HUMAN | - | - | - | - | - | - | - | P | G | R | W | E | H | R | - | - | - | T | C | R | Q | L | L | H | L | K | D | Q | C | L | I | S | C | C | - | - | - | - | - | - | - | - | - | - | - | - | - | - | - | - | - | - | - | - | - | - | - | - | - | - | - | -181 |
| CAN10_MOUSE | - | - | - | - | - | - | - | P | S | G | G | E | H | R | - | - | - | T | C | R | Q | L | L | H | L | K | D | R | C | L | I | S | C | S | - | - | - | - | - | - | - | - | - | - | - | - | - | - | - | - | - | - | - | - | - | - | - | - | - | - | - | -181 |
| XP_004943443 | - | - | - | - | - | - | - | G | M | V | L | K | K | E | - | - | - | V | F | R | R | L | M | V | L | K | E | Q | C | V | I | S | C | S | - | - | - | - | - | - | - | - | - | - | - | - | - | - | - | - | - | - | - | - | - | - | - | - | - | - | - | -181 |
| F8W4K1_DANRE | - | - | - | - | - | - | - | Q | I | Q | H | A | L | H | G | L | S | V | S | D | L | P | L | E | L | K | E | R | C | A | I | S | C | C | - | - | - | - | - | - | - | - | - | - | - | - | - | - | - | - | - | - | - | - | - | - | - | - | - | - | - | -184 |
| CANC_DROME | - | - | - | - | - | - | - | L | L | T | - | - | - | - | - | - | - | T | T | C | I | V | T | C | L | A | D | K | S | A | T | V | A | K | - | - | - | - | - | - | - | - | - | - | - | - | - | - | - | - | - | - | - | - | - | - | - | - | - | - | - | -163 |
| C3ZKN9_BRAFL | - | - | - | - | - | - | - | - | - | - | - | - | - | - | - | - | - | - | - | - | H | F | F | S | L | I | Q | K | A | F | E | R | Y | S | - | - | - | - | - | - | - | - | - | - | - | - | - | - | - | - | - | - | - | - | - | - | - | - | - | - | - | -163 |
| G4V623_SCHMA | - | - | - | - | - | - | - | D | L | D | D | I | L | I | K | - | - | - | - | S | F | D | R | R | S | L | I | T | A | R | F | V | I | P | - | - | - | - | - | - | - | - | - | - | - | - | - | - | - | - | - | - | - | - | - | - | - | - | - | - | - | -197 |
| R7VK39_CAPTE | - | - | - | - | - | - | - | Y | L | W | D | I | I | L | K | - | - | - | - | S | H | E | M | E | S | L | M | M | C | Q | I | T | A | D | - | - | - | - | - | - | - | - | - | - | - | - | - | - | - | - | - | - | - | - | - | - | - | - | - | - | - | -174 |
| G4V693_SCHMA | - | - | - | - | - | - | - | L | F | F | E | Q | M | E | K | D | L | I | D | E | C | I | V | I | F | C | T | K | E | D | S | E | Q | Q | D | G | D | S | E | T | S | P | H | I | F | G | - | - | - | - | - | - | - | - | - | - | - | - | - | - | - | -199 |
| R7UPU6_CAPTE | - | - | - | - | - | - | - | - | A | L | F | A | R | V | K | N | A | L | N | S | G | A | V | I | A | C | S | C | P | - | - | - | - | - | - | - | - | - | - | - | - | - | - | - | - | - | - | - | - | - | - | - | - | - | - | - | - | - | - | - | - | -176 |
| R7V817_CAPTE | - | - | - | - | - | - | - | - | A | L | F | A | R | M | K | N | A | L | N | S | G | A | V | I | A | C | S | C | P | V | G | - | - | - | - | - | - | - | - | - | - | - | - | - | - | - | - | - | - | - | - | - | - | - | - | - | - | - | - | - | - | -175 |
| R7UNL8_CAPTE | - | - | - | - | - | - | - | - | T | L | Y | S | R | M | K | N | A | L | S | C | G | S | I | I | T | C | S | C | P | - | - | - | - | - | - | - | - | - | - | - | - | - | - | - | - | - | - | - | - | - | - | - | - | - | - | - | - | - | - | - | - | -171 |
| K1PJ80_CRAGI | - | - | - | - | - | - | - | - | Q | L | H | T | R | V | R | N | A | L | S | S | G | A | A | V | T | C | S | V | P | - | - | - | - | - | - | - | - | - | - | - | - | - | - | - | - | - | - | - | - | - | - | - | - | - | - | - | - | - | - | - | - | -176 |
| K1PCX8_CRAGI | - | - | - | - | - | - | - | - | Q | L | H | T | R | V | R | N | A | L | Y | S | N | A | A | V | T | C | G | T | P | - | - | - | - | - | - | - | - | - | - | - | - | - | - | - | - | - | - | - | - | - | - | - | - | - | - | - | - | - | - | - | - | -176 |
| CAND_DROME | - | E | D | E | L | D | K | D | L | I | W | A | Q | L | L | S | S | R | C | V | R | F | L | M | G | A | S | C | G | G | G | N | M | K | V | D | E | E | - | - | - | - | - | - | - | - | - | - | - | - | - | - | - | - | - | - | - | - | - | - | - | -191 |
| XP_002735765 | - | D | P | V | V | D | K | D | L | I | W | A | K | L | L | S | S | K | E | A | G | F | L | M | G | A | S | C | G | G | G | N | M | K | V | N | N | K | - | - | - | - | - | - | - | - | - | - | - | - | - | - | - | - | - | - | - | - | - | - | - | -192 |
| CAN15_HUMAN | - | E | E | P | V | D | T | D | L | I | W | A | K | M | L | S | S | K | E | A | G | F | L | M | G | A | S | C | G | G | G | N | M | K | V | D | D | S | - | - | - | - | - | - | - | - | - | - | - | - | - | - | - | - | - | - | - | - | - | - | - | -192 |
| CAN15_MOUSE | - | E | E | P | V | D | T | D | L | I | W | A | K | M | L | S | S | K | E | A | G | F | L | M | G | A | S | C | G | G | G | N | M | K | V | D | D | A | - | - | - | - | - | - | - | - | - | - | - | - | - | - | - | - | - | - | - | - | - | - | - | -192 |
| E1BTA8_CHICK | - | E | E | P | I | D | T | D | L | I | W | A | K | M | L | S | S | K | E | A | G | F | L | M | G | A | S | C | G | G | G | N | M | K | V | D | D | V | - | - | - | - | - | - | - | - | - | - | - | - | - | - | - | - | - | - | - | - | - | - | - | -192 |
| E7FAX5_DANRE | - | E | E | P | I | D | T | D | L | I | W | A | K | M | L | S | S | K | E | A | G | F | L | M | G | A | S | C | G | G | G | N | M | K | V | D | D | A | - | - | - | - | - | - | - | - | - | - | - | - | - | - | - | - | - | - | - | - | - | - | - | -192 |
| C3XWK8_BRAFL | - | E | D | P | V | E | P | D | L | I | W | A | R | L | L | S | S | K | E | A | G | F | L | M | G | A | S | C | G | G | G | N | M | K | V | D | D | A | - | - | - | - | - | - | - | - | - | - | - | - | - | - | - | - | - | - | - | - | - | - | - | -192 |
| W4YMU4_STRPU | - | R | K | P | A | D | P | D | L | V | W | A | K | L | L | S | A | K | E | A | G | Y | L | M | G | A | S | C | G | S | G | N | M | K | I | D | P | Q | - | - | - | - | - | - | - | - | - | - | - | - | - | - | - | - | - | - | - | - | - | - | - | -306 |
| K1PV74_CRAGI | - | - | E | E | I | D | P | N | L | I | W | A | K | L | L | S | C | R | E | S | R | F | L | M | G | A | S | C | G | G | G | N | M | K | T | E | D | K | - | - | - | - | - | - | - | - | - | - | - | - | - | - | - | - | - | - | - | - | - | - | - | -189 |
| R7TJF2_CAPTE | - | - | E | R | L | D | A | D | L | I | W | A | R | M | L | S | S | R | E | S | K | Y | L | M | G | A | S | C | G | G | G | N | M | Q | T | D | D | D | - | - | - | - | - | - | - | - | - | - | - | - | - | - | - | - | - | - | - | - | - | - | - | -188 |
| T2MI17_HYDVU | - | E | D | I | V | D | K | D | L | V | W | A | K | L | L | S | S | R | N | A | G | Y | L | M | G | A | S | C | G | G | D | I | T | N | Q | E | A | I | D | - | - | - | - | - | - | - | - | - | - | - | - | - | - | - | - | - | - | - | - | - | - | -190 |
| Q1RL55_CIOIN | - | E | T | E | V | E | E | D | M | V | W | V | K | L | L | S | S | M | E | A | G | Y | L | M | G | A | S | C | G | G | G | N | M | D | T | N | D | E | - | - | - | - | - | - | - | - | - | - | - | - | - | - | - | - | - | - | - | - | - | - | - | -176 |
| I1EA30_AMPQE | - | - | E | E | V | E | Q | E | L | V | W | A | K | L | L | S | F | K | E | S | K | F | P | M | A | A | S | S | - | - | S | P | V | D | P | T | E | S | - | - | - | - | - | - | - | - | - | - | - | - | - | - | - | - | - | - | - | - | - | - | - | -187 |
| G5EEZ6_CAEEL | - | - | - | V | D | D | V | D | T | F | W | A | Q | L | V | S | S | K | E | S | G | F | L | M | C | C | H | C | G | A | F | E | N | V | V | A | E | A | - | - | - | - | - | - | - | - | - | - | - | - | - | - | - | - | - | - | - | - | - | - | - | -188 |
| O44903_CAEEL | - | - | - | D | E | E | V | D | T | F | W | A | Q | L | V | S | S | Q | E | S | G | F | L | T | C | C | H | C | G | P | F | E | S | E | E | A | E | A | - | - | - | - | - | - | - | - | - | - | - | - | - | - | - | - | - | - | - | - | - | - | - | -188 |
| O02260_CAEEL | - | Y | W | K | T | L | M | E | F | R | - | - | - | - | - | - | - | - | - | - | - | - | - | - | N | N | Q | Y | P | M | A | C | G | T | L | N | R | E | - | - | - | - | - | - | - | - | - | - | - | - | - | - | - | - | - | - | - | - | - | - | - | -176 |
| O02259_CAEEL | - | H | W | K | I | L | M | D | Y | K | Y | E | F | L | K | I | L | I | G | V | V | I | F | Q | K | N | Q | Y | P | M | A | C | E | T | T | I | Q | R | - | - | - | - | - | - | - | - | - | - | - | - | - | - | - | - | - | - | - | - | - | - | - | -167 |
| C1MTG3_MICPC | - | G | G | A | F | D | E | A | R | A | W | E | K | V | L | S | Y | C | E | A | G | F | P | M | G | C | A | T | - | - | - | - | S | S | G | G | R | G | - | - | - | - | - | - | - | - | - | - | - | - | - | - | - | - | - | - | - | - | - | - | - | -230 |
| C1E493_MICSR | - | - | - | - | F | K | S | E | E | A | W | R | K | L | L | S | Y | A | A | A | K | F | P | M | G | C | A | T | Q | G | D | P | S | H | R | E | V | G | - | - | - | - | - | - | - | - | - | - | - | - | - | - | - | - | - | - | - | - | - | - | - | -180 |
| Q012Z6_OSTTA | - | - | - | - | F | D | S | E | E | C | W | A | R | L | M | S | F | S | A | A | G | F | P | L | G | C | A | T | - | - | - | - | S | F | S | A | E | G | - | - | - | - | - | - | - | - | - | - | - | - | - | - | - | - | - | - | - | - | - | - | - | -173 |
| L1IGB6_GUITH | - | - | - | - | F | S | M | E | H | L | W | D | Q | L | L | Y | F | K | S | Q | G | F | P | M | G | C | A | T | G | G | D | A | E | L | K | E | V | G | - | - | - | - | - | - | - | - | - | - | - | - | - | - | - | - | - | - | - | - | - | - | - | -180 |
| B7FWX6_PHATC | - | - | - | - | - | D | L | D | E | L | W | A | R | L | L | S | F | H | Q | A | G | F | L | V | G | V | A | T | - | - | - | - | S | R | G | G | E | G | - | - | - | - | - | - | - | - | - | - | - | - | - | - | - | - | - | - | - | - | - | - | - | -180 |
| N9TPR9_ENTHI | - | - | - | E | T | D | V | N | R | L | F | K | K | I | A | D | Y | D | K | K | K | Y | C | M | V | A | N | V | P | D | T | P | G | I | D | I | E | K | - | - | - | - | - | - | - | - | - | - | - | - | - | - | - | - | - | - | - | - | - | - | - | -172 |
| G0QS43_ICHMG | - | - | - | S | D | D | L | E | L | V | W | K | K | L | L | Y | A | E | K | S | N | F | A | M | T | C | S | S | K | A | E | I | N | D | D | D | L | Q | - | - | - | - | - | - | - | - | - | - | - | - | - | - | - | - | - | - | - | - | - | - | - | -180 |
| D8LB20_ECTSI | - | - | - | V | H | E | S | D | K | F | W | G | V | L | L | T | Y | A | Q | G | R | K | G | A | M | C | C | S | I | T | G | P | V | S | E | R | V | R | - | - | - | - | - | - | - | - | - | - | - | - | - | - | - | - | - | - | - | - | - | - | - | -218 |
| Q9U0T9_LEIMA | - | - | - | - | H | S | G | R | D | L | E | S | R | M | I | Q | W | Q | R | K | G | Y | Q | A | I | L | T | T | P | G | K | A | P | A | - | I | P | A | E | A | Q | S | S | P | D | F | S | D | Q | P | E | M | - | - | - | - | - | - | - | - | - | -203 |
| Q4CXJ8_TRYCC | - | - | - | - | N | G | G | K | E | L | F | Q | Q | L | L | H | Y | Q | D | S | G | F | Q | I | I | L | T | T | A | G | K | A | P | A | F | I | S | G | T | N | K | I | A | S | L | F | D | E | E | P | E | L | - | - | - | - | - | - | - | - | - | -204 |
| F4I0A4_ARATH | - | - | - | - | L | A | S | G | R | L | W | S | Q | L | L | R | F | K | Q | E | G | F | L | L | G | A | G | S | P | S | G | - | - | S | D | V | H | V | S | - | - | - | - | - | - | - | - | - | - | - | - | - | - | - | - | - | - | - | - | - | - | -187 |
| Q8RUQ1_MAIZE | - | - | - | - | L | A | S | G | R | L | W | S | Q | L | L | H | F | K | Q | E | G | F | L | L | G | A | G | S | P | S | G | - | - | S | D | A | H | I | S | - | - | - | - | - | - | - | - | - | - | - | - | - | - | - | - | - | - | - | - | - | - | -187 |
| D7FQ17_ECTSI | - | - | - | - | K | K | S | - | - | L | W | K | K | M | T | K | Y | K | R | N | G | Y | L | L | G | A | G | T | I | T | G | N | L | V | E | K | Q | V | Q | - | - | - | - | - | - | - | - | - | - | - | - | - | - | - | - | - | - | - | - | - | - | -204 |
| D0MZ44_PHYIT | - | - | - | - | K | K | T | - | - | L | W | Q | Q | L | K | L | F | R | K | N | R | F | L | L | G | A | A | S | L | P | S | E | H | A | D | P | A | L | R | - | - | - | - | - | - | - | - | - | - | - | - | - | - | - | - | - | - | - | - | - | - | -205 |
| L1JBM0_GUITH | - | - | - | - | - | - | - | - | Q | M | W | V | K | L | V | K | Y | S | D | N | G | H | L | M | G | C | A | N | - | - | - | - | - | - | - | H | V | A | A | - | - | - | - | - | - | - | - | - | - | - | - | - | - | - | - | - | - | - | - | - | - | -151 |
| L1J739_GUITH | - | - | - | - | - | - | - | - | F | L | W | Q | K | L | K | L | W | M | K | Q | N | S | L | C | G | C | S | Y | S | V | S | K | G | E | A | R | H | Q | E | - | - | - | - | - | - | - | - | - | - | - | - | - | - | - | - | - | - | - | - | - | - | -191 |
| L1J0U9_GUITH | - | - | - | - | - | - | - | - | Y | L | W | Q | Q | L | L | L | W | C | K | Q | G | - | L | L | G | C | W | Q | E | V | E | R | - | E | G | R | T | T | D | - | - | - | - | - | - | - | - | - | - | - | - | - | - | - | - | - | - | - | - | - | - | -182 |
| L1J1Q4_GUITH | - | - | - | - | - | - | - | - | S | L | W | R | Q | L | Q | E | A | K | S | E | G | H | L | L | A | C | S | L | S | I | R | N | A | Q | P | E | A | Q | G | - | - | - | - | - | - | - | - | - | - | - | - | - | - | - | - | - | - | - | - | - | - | -202 |
| D0ND88_PHYIT | - | P | A | S | S | P | S | M | L | L | W | K | Q | L | L | R | Y | K | K | K | K | C | I | L | T | T | Q | L | R | Q | L | S | F | N | A | Q | D | V | T | - | - | - | - | - | - | - | - | - | - | - | - | - | - | - | - | - | - | - | - | - | - | -231 |
| D2VVS6_NAEGR | - | - | - | V | W | Q | Y | L | S | C | L | P | K | Q | N | S | S | S | S | S | F | K | L | M | G | A | R | S | E | T | S | A | S | D | Y | T | - | - | - | - | - | - | - | - | - | - | - | - | - | - | - | - | - | - | - | - | - | - | - | - | - | -185 |
| D2V3V2_NAEGR | - | - | - | V | W | E | I | L | K | T | I | K | K | K | S | T | D | P | S | S | Y | C | L | M | G | C | R | S | D | K | - | A | P | A | R | T | - | - | - | - | - | - | - | - | - | - | - | - | - | - | - | - | - | - | - | - | - | - | - | - | - | -185 |
| Q5B5J7_EMENI | - | - | - | - | - | - | K | D | K | F | W | N | D | E | L | M | Q | V | N | K | E | F | L | F | G | C | G | T | G | L | Y | S | N | W | L | T | P | N | H | R | - | - | - | - | - | - | - | - | - | - | - | - | - | - | - | - | - | - | - | - | - | -205 |
| E4ZY10_LEPMJ | - | - | - | - | - | - | K | D | K | F | W | R | E | E | L | M | N | V | N | K | T | F | L | F | G | C | G | - | - | - | - | - | - | - | - | - | - | Q | M | G | - | - | - | - | - | - | - | - | - | - | - | - | - | - | - | - | - | - | - | - | - | -201 |
| A8PIE5_COPC7 | - | - | - | - | - | - | I | D | K | F | W | K | E | E | L | L | M | A | N | K | D | R | V | L | A | C | S | F | Q | R | L | R | A | P | E | D | D | W | W | G | - | - | - | - | - | - | - | - | - | - | - | - | - | - | - | - | - | - | - | - | - | -206 |
| A8NBA6_COPC7 | - | - | - | - | - | - | I | D | Q | F | W | T | E | E | L | L | N | A | N | R | D | R | V | F | A | C | A | F | H | Q | L | D | S | S | R | N | G | V | G | - | - | - | - | - | - | - | - | - | - | - | - | - | - | - | - | - | - | - | - | - | - | -204 |
| A8NBA1_COPC7 | - | - | - | - | - | - | R | D | R | F | W | N | E | E | V | L | K | V | N | H | D | R | L | L | S | A | S | F | S | - | - | - | - | - | - | - | - | - | - | - | - | - | - | - | - | - | - | - | - | - | - | - | - | - | - | - | - | - | - | - | - | -198 |
| A8NBA2_COPC7 | - | - | - | - | - | - | R | D | R | F | W | K | E | E | M | S | K | A | N | T | D | R | L | L | S | A | S | F | F | - | - | - | - | - | - | - | - | - | - | - | - | - | - | - | - | - | - | - | - | - | - | - | - | - | - | - | - | - | - | - | - | -198 |
| A8NAE6_COPC7 | - | - | - | - | - | - | I | D | K | F | W | N | E | E | L | V | K | A | N | K | D | R | L | F | G | C | W | F | K | A | L | D | G | S | R | N | T | L | K | - | - | - | - | - | - | - | - | - | - | - | - | - | - | - | - | - | - | - | - | - | - | -226 |
| A8NAI9_COPC7 | - | - | - | - | - | - | T | D | R | F | W | E | E | E | L | S | K | A | G | V | D | R | F | F | D | V | S | L | A | R | L | T | K | N | A | S | - | - | - | - | - | - | - | - | - | - | - | - | - | - | - | - | - | - | - | - | - | - | - | - | - | -201 |
| G4TER7_PIRID | - | - | - | - | - | - | P | K | Q | L | W | N | E | L | V | H | N | A | N | R | T | R | L | Y | G | C | A | I | H | G | I | D | N | E | R | G | L | N | T | - | - | - | - | - | - | - | - | - | - | - | - | - | - | - | - | - | - | - | - | - | - | -204 |
| G4TER6_PIRID | - | - | - | - | - | - | P | N | K | F | W | N | E | L | V | A | T | A | G | R | T | R | L | Y | G | C | F | L | R | G | - | D | D | Q | S | A | S | D | V | - | - | - | - | - | - | - | - | - | - | - | - | - | - | - | - | - | - | - | - | - | - | -193 |
| G4TNA1_PIRID | - | - | - | - | - | - | L | D | A | L | W | N | E | L | V | E | H | T | S | R | E | N | K | S | K | T | Y | A | C | G | S | N | R | P | E | N | A | G | G | - | - | - | - | - | - | - | - | - | - | - | - | - | - | - | - | - | - | - | - | - | - | -204 |
| D0MWB9_PHYIT | - | - | - | - | - | - | G | D | P | F | W | E | K | I | S | R | Y | K | N | S | G | T | L | M | G | C | S | I | Q | P | P | V | T | S | S | K | E | V | A | - | - | - | - | - | - | - | - | - | - | - | - | - | - | - | - | - | - | - | - | - | - | -194 |
| L1I9H5_GUITH | - | - | - | - | - | I | A | F | A | A | L | Y | P | D | E | S | G | K | D | N | S | C | F | L | S | C | S | V | R | S | D | A | P | - | - | - | - | - | - | - | - | - | - | - | - | - | - | - | - | - | - | - | - | - | - | - | - | - | - | - | - | -191 |
| Q7N497_PHOLL | - | - | - | - | - | I | W | D | E | K | F | I | D | I | F | K | I | K | H | H | V | T | Y | Q | G | T | G | S | S | T | D | - | - | - | - | - | - | - | - | - | - | - | - | - | - | - | - | - | - | - | - | - | - | - | - | - | - | - | - | - | - | -158 |
| ZP_11152369 | - | - | - | - | - | V | W | K | D | Y | F | L | Y | I | F | Q | N | R | K | S | T | T | Y | Q | G | T | G | P | I | T | H | L | - | - | - | - | - | - | - | - | - | - | - | - | - | - | - | - | - | - | - | - | - | - | - | - | - | - | - | - | - | -155 |
| A2FEL7_TRIVA | - | - | - | - | - | T | S | G | E | L | F | K | Q | M | V | E | W | N | K | R | G | S | H | L | I | A | G | S | P | E | G | S | H | F | - | - | - | - | - | - | - | - | - | - | - | - | - | - | - | - | - | - | - | - | - | - | - | - | - | - | - | -179 |
| A2D9A8_TRIVA | - | - | - | - | - | K | S | G | A | L | F | D | Q | I | N | E | I | F | M | N | G | G | Y | L | V | S | G | S | M | E | G | N | N | S | - | - | - | - | - | - | - | - | - | - | - | - | - | - | - | - | - | - | - | - | - | - | - | - | - | - | - | -180 |
| A2FHB3_TRIVA | - | - | - | - | - | N | N | G | T | L | W | K | R | I | L | Q | W | H | Q | D | K | D | Y | I | C | C | G | S | H | P | G | S | D | T | - | - | - | - | - | - | - | - | - | - | - | - | - | - | - | - | - | - | - | - | - | - | - | - | - | - | - | -181 |
| A2DTE8_TRIVA | - | - | - | - | - | E | N | G | E | M | F | K | L | I | K | N | T | L | E | N | G | G | Y | V | C | A | S | K | F | N | - | - | - | - | - | - | - | - | - | - | - | - | - | - | - | - | - | - | - | - | - | - | - | - | - | - | - | - | - | - | - | -174 |
| A2E4X2_TRIVA | - | - | - | - | - | Q | I | Q | K | L | L | D | I | N | G | D | V | Y | A | S | I | D | K | N | E | E | - | - | - | - | - | - | - | - | - | - | - | - | - | - | - | - | - | - | - | - | - | - | - | - | - | - | - | - | - | - | - | - | - | - | - | -175 |
| A2E1X1_TRIVA | - | - | - | - | - | H | L | D | S | I | L | K | E | T | E | F | V | F | L | S | T | F | K | T | Q | V | - | - | - | - | - | - | - | - | - | - | - | - | - | - | - | - | - | - | - | - | - | - | - | - | - | - | - | - | - | - | - | - | - | - | - | -158 |
| TPR_PORGI | - | L | S | S | F | T | D | L | S | L | L | N | F | M | K | S | M | C | Y | S | W | K | T | I | K | P | M | V | I | V | T | P | C | W | E | P | - | - | - | - | - | - | - | - | - | - | - | - | - | - | - | - | - | - | - | - | - | - | - | - | - | -205 |
| D5MF42_9BACT | - | C | S | A | L | T | A | D | Q | V | W | S | S | V | R | S | N | C | L | S | Y | R | T | I | N | P | M | T | A | W | T | F | C | V | T | P | - | - | - | - | - | - | - | - | - | - | - | - | - | - | - | - | - | - | - | - | - | - | - | - | - | -161 |
| R9H254_BACT4 | - | - | - | A | L | I | A | E | D | L | Q | R | A | V | E | V | S | L | K | Q | G | R | L | V | I | G | G | F | T | R | S | G | E | - | - | - | - | - | - | - | - | - | - | - | - | - | - | - | - | - | - | - | - | - | - | - | - | - | - | - | - | -156 |
| ZP_04847694 | - | - | - | A | L | I | A | E | D | L | Q | R | A | V | E | V | S | L | K | Q | G | R | L | V | I | G | G | F | T | R | S | G | E | - | - | - | - | - | - | - | - | - | - | - | - | - | - | - | - | - | - | - | - | - | - | - | - | - | - | - | - | -156 |
| D7IBA4_9BACE | - | - | - | A | L | I | A | E | D | L | Q | R | A | V | E | V | S | L | K | Q | G | R | L | V | I | G | G | F | T | R | S | G | E | - | - | - | - | - | - | - | - | - | - | - | - | - | - | - | - | - | - | - | - | - | - | - | - | - | - | - | - | -154 |
| ZP_09858577 | - | - | - | A | L | I | A | E | D | L | Q | R | A | V | E | V | S | L | K | Q | G | R | L | V | I | G | G | F | T | R | S | G | E | - | - | - | - | - | - | - | - | - | - | - | - | - | - | - | - | - | - | - | - | - | - | - | - | - | - | - | - | -154 |
| E5C759_9BACE | - | - | - | A | L | I | A | E | D | L | Q | R | A | V | E | V | S | L | K | Q | G | R | L | V | I | G | G | F | T | R | S | G | E | - | - | - | - | - | - | - | - | - | - | - | - | - | - | - | - | - | - | - | - | - | - | - | - | - | - | - | - | -156 |
| A7LTS0_BACO1 | - | - | - | K | L | T | P | A | E | L | Q | R | A | V | S | V | S | L | K | R | G | K | L | V | V | G | G | F | T | K | S | D | V | - | - | - | - | - | - | - | - | - | - | - | - | - | - | - | - | - | - | - | - | - | - | - | - | - | - | - | - | -154 |
| Q8YQK8_NOSS1 | - | - | - | - | - | S | S | T | N | L | Y | S | A | I | E | T | A | L | A | N | G | - | R | A | V | A | T | A | R | I | S | S | T | - | - | - | - | - | - | - | - | - | - | - | - | - | - | - | - | - | - | - | - | - | - | - | - | - | - | - | - | -165 |
| Q3MBX8_ANAVT | - | - | - | - | - | T | N | I | N | C | F | S | T | I | E | T | A | L | A | N | G | - | K | A | V | T | A | A | R | M | G | D | S | - | - | - | - | - | - | - | - | - | - | - | - | - | - | - | - | - | - | - | - | - | - | - | - | - | - | - | - | -170 |
| Q8YZT8_NOSS1 | - | - | - | - | - | S | R | I | S | - | F | S | L | L | E | D | A | L | K | A | G | - | K | A | I | T | T | S | R | S | G | K | D | - | - | - | - | - | - | - | - | - | - | - | - | - | - | - | - | - | - | - | - | - | - | - | - | - | - | - | - | -157 |
| K9UIG2_9CHRO | - | - | - | G | G | S | W | D | F | S | F | N | L | I | K | D | S | L | S | A | G | - | K | A | I | L | S | A | G | V | P | S | V | - | - | - | - | - | - | - | - | - | - | - | - | - | - | - | - | - | - | - | - | - | - | - | - | - | - | - | - | -167 |
| A4YRP8_BRASO | - | T | T | G | N | A | T | S | G | V | L | A | T | I | A | A | S | L | A | A | G | D | D | V | I | L | S | S | Y | T | N | A | K | - | - | - | - | - | - | - | - | - | - | - | - | - | - | - | - | - | - | - | - | - | - | - | - | - | - | - | - | -184 |
| H0TAL7_9BRAD | - | T | T | G | N | A | T | S | G | V | L | A | T | I | A | A | S | L | A | A | G | D | D | V | I | L | S | S | Y | T | N | A | K | - | - | - | - | - | - | - | - | - | - | - | - | - | - | - | - | - | - | - | - | - | - | - | - | - | - | - | - | -188 |
| H0SNL4_9BRAD | - | T | T | G | N | A | T | S | S | V | L | A | T | I | A | A | S | L | A | A | G | D | D | V | I | L | S | S | Y | T | N | A | K | - | - | - | - | - | - | - | - | - | - | - | - | - | - | - | - | - | - | - | - | - | - | - | - | - | - | - | - | -190 |
| H0S4P6_9BRAD | - | T | T | G | N | S | T | S | G | V | L | S | T | I | A | A | S | L | A | A | G | D | D | V | I | L | S | S | Y | T | N | A | K | - | - | - | - | - | - | - | - | - | - | - | - | - | - | - | - | - | - | - | - | - | - | - | - | - | - | - | - | -190 |
| A5EMK2_BRASB | - | T | T | G | N | S | T | S | S | V | L | A | T | I | A | A | S | L | A | A | G | D | D | V | I | L | S | S | Y | T | N | A | K | - | - | - | - | - | - | - | - | - | - | - | - | - | - | - | - | - | - | - | - | - | - | - | - | - | - | - | - | -184 |
| M4ZBJ4_9BRAD | - | T | T | G | N | S | T | S | G | V | L | A | T | I | A | A | S | L | A | A | G | D | D | V | I | L | S | S | Y | T | N | A | K | - | - | - | - | - | - | - | - | - | - | - | - | - | - | - | - | - | - | - | - | - | - | - | - | - | - | - | - | -190 |
| Q07SP7_RHOP5 | - | S | A | G | Q | S | T | A | A | V | L | S | S | I | V | A | A | L | D | A | G | N | D | V | V | L | A | S | Y | T | N | A | T | - | - | - | - | - | - | - | - | - | - | - | - | - | - | - | - | - | - | - | - | - | - | - | - | - | - | - | - | -177 |
| B0JUQ7_MICAN | - | - | - | - | - | - | - | - | S | D | R | T | A | V | I | N | A | F | N | A | G | R | I | V | F | L | N | W | - | - | - | - | - | - | - | - | - | - | - | - | - | - | - | - | - | - | - | - | - | - | - | - | - | - | - | - | - | - | - | - | - | -167 |
| K9WJ87_9CYAN | - | - | - | - | - | - | - | - | G | D | F | N | S | V | V | N | A | F | N | A | D | R | I | I | Y | L | S | D | - | - | - | - | - | - | - | - | - | - | - | - | - | - | - | - | - | - | - | - | - | - | - | - | - | - | - | - | - | - | - | - | - | -161 |
| G6FUE0_9CYAN | - | - | - | - | - | - | - | - | - | - | F | N | S | I | V | N | A | F | N | F | G | Q | M | I | G | L | T | T | K | - | - | - | - | - | - | - | - | - | - | - | - | - | - | - | - | - | - | - | - | - | - | - | - | - | - | - | - | - | - | - | - | -160 |
| K9PHG5_9CYAN | - | - | - | - | - | - | - | - | - | - | L | N | S | L | V | S | A | Y | N | A | G | T | L | M | G | I | S | S | K | - | - | - | - | - | - | - | - | - | - | - | - | - | - | - | - | - | - | - | - | - | - | - | - | - | - | - | - | - | - | - | - | -162 |
| Q8YXX3_NOSS1 | - | - | - | - | - | - | - | - | L | D | M | N | A | I | V | T | A | F | N | Q | G | Q | M | I | C | F | A | S | R | - | - | - | - | - | - | - | - | - | - | - | - | - | - | - | - | - | - | - | - | - | - | - | - | - | - | - | - | - | - | - | - | -177 |
| K9TN70_9CYAN | - | - | - | - | - | - | - | - | S | S | V | A | A | A | I | A | A | F | T | A | G | S | A | V | F | F | S | S | D | K | N | Q | H | - | - | - | - | - | - | - | - | - | - | - | - | - | - | - | - | - | - | - | - | - | - | - | - | - | - | - | - | -181 |
| Q05QW3_9SYNE | - | - | - | - | - | D | A | G | L | T | K | N | E | L | I | N | L | V | N | G | D | N | I | V | T | I | D | G | F | N | G | N | A | S | T | D | D | D | G | D | G | - | - | - | - | - | - | - | - | - | - | - | - | - | - | - | - | - | - | - | - | -198 |
| Q05QW2_9SYNE | - | - | - | - | - | A | T | N | V | S | S | S | E | L | Q | T | L | V | N | S | N | R | V | V | A | A | G | - | - | - | - | - | - | - | - | - | - | - | - | - | - | - | - | - | - | - | - | - | - | - | - | - | - | - | - | - | - | - | - | - | - | -181 |
| Q05QW4_9SYNE | - | - | - | - | - | - | - | G | V | S | N | A | E | L | I | N | Y | V | N | S | D | R | V | V | T | V | R | G | F | N | G | L | A | T | G | S | G | S | G | P | T | - | - | - | - | - | - | - | - | - | - | - | - | - | - | - | - | - | - | - | - | -187 |
| E0UGX6_CYAP2 | - | - | - | - | - | - | - | - | M | S | K | T | Q | L | I | N | L | V | N | S | N | Q | I | L | T | A | G | F | V | S | G | A | N | - | - | - | - | - | - | - | - | - | - | - | - | - | - | - | - | - | - | - | - | - | - | - | - | - | - | - | - | -169 |
| K7WKF4_9NOST | - | - | - | - | - | - | - | - | M | T | Q | T | Q | L | I | N | L | V | N | S | N | Q | V | L | T | A | G | F | V | Y | G | A | G | - | - | - | - | - | - | - | - | - | - | - | - | - | - | - | - | - | - | - | - | - | - | - | - | - | - | - | - | -168 |
| E0UGY5_CYAP2 | - | - | - | - | - | - | - | - | M | T | Q | S | D | L | I | N | L | V | N | S | N | Q | I | L | T | A | G | F | I | Y | G | S | G | - | - | - | - | - | - | - | - | - | - | - | - | - | - | - | - | - | - | - | - | - | - | - | - | - | - | - | - | -171 |
| A8LCE5_FRASN | - | - | - | - | - | - | - | A | V | G | V | D | E | L | A | A | R | L | A | A | G | G | V | V | T | V | V | T | R | - | - | - | - | - | - | - | - | - | - | - | - | - | - | - | - | - | - | - | - | - | - | - | - | - | - | - | - | - | - | - | - | -159 |
| D3CT47_9ACTO | - | - | - | - | - | - | - | Q | T | S | V | E | G | I | A | A | R | L | A | A | G | D | V | V | T | L | S | T | R | - | - | - | - | - | - | - | - | - | - | - | - | - | - | - | - | - | - | - | - | - | - | - | - | - | - | - | - | - | - | - | - | -163 |
| Q2J4J6_FRASC | - | - | - | - | - | - | - | E | A | T | V | P | A | L | G | E | L | L | A | D | G | E | V | V | I | V | T | T | R | - | - | - | - | - | - | - | - | - | - | - | - | - | - | - | - | - | - | - | - | - | - | - | - | - | - | - | - | - | - | - | - | -184 |
| Q0RAZ2_FRAAA | - | - | - | - | - | - | - | A | T | S | V | A | D | L | A | A | L | L | D | R | G | E | V | V | V | L | A | T | R | - | - | - | - | - | - | - | - | - | - | - | - | - | - | - | - | - | - | - | - | - | - | - | - | - | - | - | - | - | - | - | - | -171 |
| E3J3F6_FRASU | - | - | - | - | - | - | - | E | V | S | T | G | E | L | A | A | R | L | A | A | G | D | V | V | I | V | S | T | G | - | G | - | - | - | - | - | - | - | - | - | - | - | - | - | - | - | - | - | - | - | - | - | - | - | - | - | - | - | - | - | - | -166 |
| ZP_09168240 | - | - | - | - | - | - | - | S | L | S | V | A | D | I | A | S | R | L | A | T | G | D | V | I | T | V | S | T | R | P | G | - | - | - | - | - | - | - | - | - | - | - | - | - | - | - | - | - | - | - | - | - | - | - | - | - | - | - | - | - | - | -177 |
| C7MDV9_BRAFD | - | - | - | - | - | - | - | P | G | A | A | Q | I | L | A | W | R | D | Q | G | R | A | I | A | A | S | T | H | P | L | S | - | - | - | - | - | - | - | - | - | - | - | - | - | - | - | - | - | - | - | - | - | - | - | - | - | - | - | - | - | - | -139 |
| ZP_10094989 | - | - | - | - | - | - | - | P | D | A | G | Q | V | L | A | W | R | A | E | G | R | A | L | C | A | S | T | H | P | L | S | - | - | - | - | - | - | - | - | - | - | - | - | - | - | - | - | - | - | - | - | - | - | - | - | - | - | - | - | - | - | -139 |
| A4X268_SALTO | - | - | - | P | G | R | E | T | A | S | E | A | R | L | A | A | L | L | A | A | G | S | P | V | I | T | A | T | R | G | E | D | Q | Y | - | - | - | - | - | - | - | - | - | - | - | - | - | - | - | - | - | - | - | - | - | - | - | - | - | - | - | -196 |
| WP_043162213 | - | - | - | P | G | R | E | T | T | S | E | A | R | L | A | A | L | L | A | A | G | S | P | V | I | T | A | T | R | G | E | D | Q | Y | - | - | - | - | - | - | - | - | - | - | - | - | - | - | - | - | - | - | - | - | - | - | - | - | - | - | - | -196 |
| Q0BRV8_GRABC | - | - | - | - | - | Y | P | Y | M | S | A | E | M | L | Q | G | L | L | A | T | K | S | L | V | V | M | D | T | G | - | - | - | - | - | - | - | - | - | - | - | - | - | - | - | - | - | - | - | - | - | - | - | - | - | - | - | - | - | - | - | - | -165 |
| A9B2P0_HERA2 | - | - | - | - | - | - | - | D | V | D | F | A | D | L | K | S | R | L | D | N | G | A | A | I | T | A | G | T | P | D | S | L | T | - | - | - | - | - | - | - | - | - | - | - | - | - | - | - | - | - | - | - | - | - | - | - | - | - | - | - | - | -159 |
| A9B3U8_HERA2 | - | - | - | - | - | - | - | D | V | D | F | A | T | L | K | R | Q | V | D | Q | G | D | A | V | T | T | G | T | P | D | K | L | N | - | - | - | - | - | - | - | - | - | - | - | - | - | - | - | - | - | - | - | - | - | - | - | - | - | - | - | - | -161 |
| A9B2A3_HERA2 | - | - | - | - | - | - | - | S | Y | S | I | H | Q | L | A | T | M | H | N | N | Q | Q | A | I | I | F | G | T | P | D | P | S | D | P | - | - | - | - | - | - | - | - | - | - | - | - | - | - | - | - | - | - | - | - | - | - | - | - | - | - | - | -180 |
| D6ZC85_SEGRD | - | - | - | - | - | - | - | L | S | S | Y | D | A | I | A | S | A | I | A | E | R | R | P | V | T | M | G | T | R | P | S | T | - | - | - | - | - | - | - | - | - | - | - | - | - | - | - | - | - | - | - | - | - | - | - | - | - | - | - | - | - | -204 |
| E5XTT9_9ACTO | - | - | - | - | - | - | - | L | S | S | Y | D | V | L | A | S | A | L | A | E | R | R | P | V | T | M | G | T | T | P | S | R | - | - | - | - | - | - | - | - | - | - | - | - | - | - | - | - | - | - | - | - | - | - | - | - | - | - | - | - | - | -204 |
| E5XTU0_9ACTO | - | - | - | - | - | - | - | L | S | S | Y | D | A | I | A | A | A | L | A | N | H | Q | P | V | T | M | G | T | T | A | A | P | G | - | - | - | - | - | - | - | - | - | - | - | - | - | - | - | - | - | - | - | - | - | - | - | - | - | - | - | - | -209 |
| D6B0I6_9ACTO | - | - | - | G | Q | S | Q | D | K | D | I | D | R | L | R | E | A | Y | E | S | G | Q | V | V | T | L | S | T | P | D | D | E | S | L | - | - | - | - | - | - | - | - | - | - | - | - | - | - | - | - | - | - | - | - | - | - | - | - | - | - | - | -172 |
| D9X719_STRVR | - | - | - | G | E | H | E | D | K | S | L | E | S | L | K | E | A | F | G | S | G | K | V | V | T | V | S | T | P | A | D | E | E | V | - | - | - | - | - | - | - | - | - | - | - | - | - | - | - | - | - | - | - | - | - | - | - | - | - | - | - | -172 |
| H6L010_SAPGL | - | - | - | - | Y | S | A | K | E | I | E | E | M | I | T | E | A | L | E | D | K | R | M | V | A | A | D | T | K | G | N | D | D | S | Q | Q | - | - | - | - | - | - | - | - | - | - | - | - | - | - | - | - | - | - | - | - | - | - | - | - | - | -159 |
| K1ZP83_9BACT | - | - | - | - | C | D | A | D | E | L | Y | E | C | L | A | T | A | V | K | E | G | N | P | I | V | A | S | S | - | - | - | N | D | S | A | D | - | - | - | - | - | - | - | - | - | - | - | - | - | - | - | - | - | - | - | - | - | - | - | - | - | -177 |
| ADGB_HUMAN | - | - | - | - | - | K | V | W | E | L | L | K | E | I | L | P | E | F | K | L | S | D | E | - | - | A | S | S | E | S | K | T | A | V | L | D | S | K | L | K | - | - | - | - | - | - | - | - | - | - | - | - | - | - | - | - | - | - | - | - | - | -219 |
| J3QMP3_MOUSE | - | - | - | - | - | R | V | W | E | L | L | K | E | I | L | P | E | F | K | L | T | E | E | - | - | P | S | S | E | S | K | I | T | T | I | D | N | K | L | K | - | - | - | - | - | - | - | - | - | - | - | - | - | - | - | - | - | - | - | - | - | -219 |
| H2XR46_CIOIN | - | - | - | - | - | N | I | W | E | F | L | K | Q | S | L | P | Q | W | T | L | P | E | T | N | P | A | E | D | E | N | K | T | S | S | A | S | S | K | R | E | N | V | P | A | P | - | - | - | - | - | - | - | - | - | - | - | - | - | - | - | - | -224 |
| CAN7_MOUSE | - | - | - | - | F | S | K | D | N | S | F | R | M | L | Y | Q | R | F | H | K | G | D | V | L | I | T | A | S | T | G | V | M | T | E | A | E | G | E | - | - | - | - | - | - | - | - | - | - | - | - | - | - | - | - | - | - | - | - | - | - | - | -190 |
| CAN7_HUMAN | - | - | - | - | F | S | K | D | N | S | F | R | M | L | Y | Q | R | F | H | K | G | D | V | L | I | T | A | S | T | G | M | M | T | E | A | E | G | E | - | - | - | - | - | - | - | - | - | - | - | - | - | - | - | - | - | - | - | - | - | - | - | -190 |
| E1BY84_CHICK | - | - | - | - | F | D | R | D | S | T | F | R | M | L | Y | Q | R | F | H | K | G | D | V | L | I | T | T | A | T | G | V | M | S | E | E | E | G | E | - | - | - | - | - | - | - | - | - | - | - | - | - | - | - | - | - | - | - | - | - | - | - | -190 |
| H9GX91_DANRE | - | - | - | - | F | N | K | D | D | T | F | R | M | L | Y | Q | R | F | H | R | G | D | V | L | I | T | T | A | T | G | V | M | T | E | E | E | G | E | - | - | - | - | - | - | - | - | - | - | - | - | - | - | - | - | - | - | - | - | - | - | - | -190 |
| F6SGI6_CIOIN | - | - | - | - | F | D | Y | D | G | F | F | Q | K | V | R | D | R | F | H | K | G | H | C | L | A | T | I | S | T | G | M | I | S | E | A | E | A | D | - | - | - | - | - | - | - | - | - | - | - | - | - | - | - | - | - | - | - | - | - | - | - | -188 |
| W4Y195_STRPU | - | - | - | - | F | D | A | D | K | V | F | K | K | L | Y | E | R | F | H | R | G | E | V | L | A | T | V | A | T | G | M | L | P | E | A | D | A | D | - | - | - | - | - | - | - | - | - | - | - | - | - | - | - | - | - | - | - | - | - | - | - | -164 |
| R7V270_CAPTE | - | - | - | - | F | D | P | D | K | E | F | L | K | I | Q | S | R | F | H | K | G | D | C | L | V | T | T | G | T | G | E | L | S | D | D | E | A | D | - | - | - | - | - | - | - | - | - | - | - | - | - | - | - | - | - | - | - | - | - | - | - | -189 |
| G4LXK3_SCHMA | - | - | - | - | F | N | K | D | R | E | F | R | R | L | L | S | R | F | H | K | G | H | C | L | V | T | I | A | T | G | Q | L | S | D | D | E | S | E | - | - | - | - | - | - | - | - | - | - | - | - | - | - | - | - | - | - | - | - | - | - | - | -190 |
| T2M2K0_HYDVU | - | - | - | - | F | D | V | L | - | - | F | D | R | M | L | N | G | L | E | K | G | D | V | L | I | T | A | A | T | G | V | M | S | D | A | E | A | D | - | - | - | - | - | - | - | - | - | - | - | - | - | - | - | - | - | - | - | - | - | - | - | -188 |
| B3RQK1_TRIAD | - | - | - | - | F | K | A | N | S | V | F | A | R | I | K | D | G | L | E | R | G | D | V | L | V | T | V | A | T | G | A | L | S | N | T | D | T | E | - | - | - | - | - | - | - | - | - | - | - | - | - | - | - | - | - | - | - | - | - | - | - | -189 |
| I1CS59_RHIO9 | - | - | - | - | F | V | P | D | N | V | W | D | R | I | F | D | G | M | K | Y | G | D | A | L | V | T | I | A | T | G | E | M | T | E | E | E | A | T | - | - | - | - | - | - | - | - | - | - | - | - | - | - | - | - | - | - | - | - | - | - | - | -185 |
| I1GER3_AMPQE | - | - | - | - | - | - | K | D | K | T | F | K | M | L | E | A | K | L | K | A | G | S | A | L | V | T | M | G | T | G | P | L | T | K | E | E | S | E | - | - | - | - | - | - | - | - | - | - | - | - | - | - | - | - | - | - | - | - | - | - | - | -186 |
| Q22143_CAEEL | - | - | - | - | A | D | P | D | E | V | F | R | K | L | F | D | R | F | H | R | G | D | C | L | I | T | L | A | T | G | K | M | T | E | D | M | Q | K | - | - | - | - | - | - | - | - | - | - | - | - | - | - | - | - | - | - | - | - | - | - | - | -189 |
| L1JZN7_GUITH | - | - | - | - | S | D | P | E | E | A | W | R | R | L | E | Q | G | Q | K | K | G | T | A | L | F | T | M | G | T | K | S | L | S | A | D | E | E | E | - | - | - | - | - | - | - | - | - | - | - | - | - | - | - | - | - | - | - | - | - | - | - | -192 |
| D0NT34_PHYIT | - | - | - | - | S | K | E | E | R | L | W | E | Q | L | K | S | A | F | H | Y | G | D | C | I | I | T | M | S | T | G | D | I | T | K | Q | E | A | K | - | - | - | - | - | - | - | - | - | - | - | - | - | - | - | - | - | - | - | - | - | - | - | -200 |
| A0A064B913_ASPOZ | - | - | - | - | - | - | G | D | Q | L | W | K | R | L | Y | R | S | F | H | Q | G | D | V | L | L | T | I | G | T | G | E | L | T | E | R | E | Q | R | - | - | - | - | - | - | - | - | - | - | - | - | - | - | - | - | - | - | - | - | - | - | - | -196 |
| A8N4X4_COPC7 | - | - | - | - | - | - | R | E | R | T | W | Q | R | I | S | T | G | F | L | N | G | K | C | M | V | T | L | G | T | G | T | R | P | I | S | F | D | S | - | - | - | - | - | - | - | - | - | - | - | - | - | - | - | - | - | - | - | - | - | - | - | -212 |
| C0H5K1_PLAF7 | - | E | Y | D | E | K | W | D | S | V | W | N | N | I | Y | D | G | I | K | K | G | K | C | V | V | C | L | G | T | L | E | L | K | D | A | A | P | S | G | L | D | Y | P | E | G | V | S | I | - | - | - | - | - | - | - | - | - | - | - | - | - | -359 |
| K1REZ8_CRAGI | - | - | - | - | - | - | - | G | D | I | Y | F | R | I | R | R | A | I | S | Q | G | A | I | V | G | C | A | V | S | R | K | H | D | G | - | - | - | - | - | - | - | - | - | - | - | - | - | - | - | - | - | - | - | - | - | - | - | - | - | - | - | -172 |
| F4NTM9_BATDJ | - | - | - | - | - | - | - | M | Q | L | F | N | A | M | D | S | L | M | R | G | G | A | L | I | S | C | F | F | Q | K | S | N | P | - | - | - | - | - | - | - | - | - | - | - | - | - | - | - | - | - | - | - | - | - | - | - | - | - | - | - | - | -193 |
| Q6F6K5_CAEEL | - | - | - | - | - | - | - | - | D | I | W | D | F | I | L | S | M | Q | K | S | K | F | L | V | T | A | C | S | T | Y | C | E | E | - | - | - | - | - | - | - | - | - | - | - | - | - | - | - | - | - | - | - | - | - | - | - | - | - | - | - | - | -151 |
| A8HZK8_CHLRE | - | - | - | - | - | - | - | - | G | L | W | T | D | L | L | A | W | L | G | S | K | S | I | I | G | A | V | A | K | R | A | A | P | - | - | - | - | - | - | - | - | - | - | - | - | - | - | - | - | - | - | - | - | - | - | - | - | - | - | - | - | -157 |
| A8J6T6_CHLRE | - | - | - | L | Y | V | S | D | D | V | F | K | A | I | T | T | Q | L | A | L | G | C | L | M | A | A | E | T | D | K | G | E | - | - | - | - | - | - | - | - | - | - | - | - | - | - | - | - | - | - | - | - | - | - | - | - | - | - | - | - | - | -213 |
| Z9JR51_9MICO | - | - | - | - | - | - | - | - | - | - | F | E | E | Y | R | E | A | I | D | A | G | R | P | I | T | V | M | T | D | P | I | A | P | - | - | - | - | - | - | - | - | - | - | - | - | - | - | - | - | - | - | - | - | - | - | - | - | - | - | - | - | -167 |
| F7ZZH9_CELGA | - | - | - | - | - | L | D | A | D | Q | V | D | E | L | R | D | V | L | D | D | G | G | Q | V | V | I | S | S | P | R | S | G | D | H | Q | I | T | V | T | D | - | - | - | - | - | - | - | - | - | - | - | - | - | - | - | - | - | - | - | - | - | -161 |
| Q7NDU2_GLOVI | - | - | - | - | - | - | - | A | E | A | L | S | A | L | E | S | A | L | E | D | D | R | L | A | L | A | S | L | S | D | N | E | D | - | - | - | - | - | - | - | - | - | - | - | - | - | - | - | - | - | - | - | - | - | - | - | - | - | - | - | - | -141 |
| I6AT95_9BACT | - | - | - | - | A | K | L | D | D | L | R | K | K | L | T | A | A | F | A | E | K | R | L | V | T | C | G | T | A | S | G | G | T | R | - | - | - | - | - | - | - | - | - | - | - | - | - | - | - | - | - | - | - | - | - | - | - | - | - | - | - | -169 |
| C7MGJ8_BRAFD | - | - | - | - | - | - | - | Y | D | R | D | G | A | P | V | A | V | L | T | P | N | S | D | S | A | T | D | T | A | K | G | D | P | - | - | - | - | - | - | - | - | - | - | - | - | - | - | - | - | - | - | - | - | - | - | - | - | - | - | - | - | -168 |
| D0WP73_9ACTO | - | - | - | - | - | - | - | - | N | E | R | R | E | I | I | N | A | A | N | S | S | N | P | V | T | A | E | T | A | S | A | P | R | E | - | - | - | - | - | - | - | - | - | - | - | - | - | - | - | - | - | - | - | - | - | - | - | - | - | - | - | -167 |
| B0C8L1_ACAM1 | - | - | - | - | - | - | - | - | - | - | - | K | Q | A | K | G | Y | L | G | S | H | Y | Y | S | V | L | D | C | V | T | G | - | - | - | - | - | - | - | - | - | - | - | - | - | - | - | - | - | - | - | - | - | - | - | - | - | - | - | - | - | - | -152 |
| B3E5A8_GEOLS | - | - | - | - | - | - | - | D | Q | V | R | R | A | L | I | R | M | Q | E | R | K | S | L | A | T | S | L | L | L | H | R | P | P | - | - | - | - | - | - | - | - | - | - | - | - | - | - | - | - | - | - | - | - | - | - | - | - | - | - | - | - | -161 |
| D4YQ94_9MICO | - | - | - | - | - | - | V | V | R | E | W | L | A | S | G | R | P | V | V | A | S | T | L | V | H | R | R | G | A | R | R | L | P | R | - | - | - | - | - | - | - | - | - | - | - | - | - | - | - | - | - | - | - | - | - | - | - | - | - | - | - | -153 |
| PAPA_CARPA | - | - | - | - | - | - | - | - | A | L | L | Y | S | I | A | N | Q | P | V | S | V | V | L | E | A | A | G | K | D | F | Q | L | Y | R | G | - | - | - | - | - | - | - | - | - | - | - | - | - | - | - | - | - | - | - | - | - | - | - | - | - | - | -146 |
| RIM13_YEAST | - | - | - | - | - | - | - | - | Y | P | F | E | K | L | W | K | F | H | K | S | N | L | C | L | M | G | A | G | T | G | N | R | S | - | - | - | - | - | - | - | - | - | - | - | - | - | - | - | - | - | - | - | - | - | - | - | - | - | - | - | - | -179 |

| I |  |  |  |  |  |  |  |  |  |  |  |  |  |  |  |  |  |  |  |  |  |  |  |  |  |  |  |  |  |  |  |  |  |  |  |  |  |  |  | * |  |  |  |  |  |  |  |  |  |  |  |  |  |  |  |  |  |  |  |  |  |  |
| --- | --- | --- | --- | --- | --- | --- | --- | --- | --- | --- | --- | --- | --- | --- | --- | --- | --- | --- | --- | --- | --- | --- | --- | --- | --- | --- | --- | --- | --- | --- | --- | --- | --- | --- | --- | --- | --- | --- | --- | --- | --- | --- | --- | --- | --- | --- | --- | --- | --- | --- | --- | --- | --- | --- | --- | --- | --- | --- | --- | --- | --- | --- |
| Q9N4B1_CAEEL | - | - | - | - | - | - | - | - | - | - | - | - | - | - | - | - | - | - | - | - | - | N | V | K | E | A | Q | L | T | N | G | - | - | - | - | L | V | R | G | H | A | Y | S | I | T | A | I | Q | T | V | N | - | - | - | - | - | - | - | - | - | T | -200 |
| Q8IAA8_CAEEL | - | - | - | - | - | - | - | - | - | - | - | - | - | - | - | - | - | - | - | - | - | N | I | K | E | A | Q | L | R | N | G | - | - | - | - | L | V | C | G | H | A | Y | S | I | T | A | I | H | S | I | T | - | - | - | - | - | - | - | - | - | Y | -200 |
| CAN_CAEEL | - | - | - | - | - | - | - | - | - | - | - | - | - | - | - | - | - | - | - | - | - | N | V | W | E | A | K | M | S | N | G | - | - | - | - | L | V | K | G | H | A | Y | S | I | T | G | C | R | I | V | D | - | - | - | - | - | - | - | - | - | G | -199 |
| Q9U2B2_CAEEL | - | - | - | - | - | - | - | - | - | - | - | - | - | - | - | - | - | - | - | - | - | N | E | K | G | V | R | Q | K | N | G | - | - | - | - | L | I | K | G | Y | G | Y | C | I | T | G | V | N | L | M | E | - | - | - | - | - | - | - | - | - | T | -203 |
| Q9N4B2_CAEEL | - | - | - | - | - | - | - | - | - | - | - | - | - | - | - | - | - | - | - | - | - | R | E | K | E | A | Q | L | A | N | G | - | - | - | - | L | V | R | G | H | A | Y | S | V | T | G | V | H | T | V | E | - | - | - | - | - | - | - | - | - | T | -200 |
| C3Y6P6_BRAFL | - | - | - | - | - | - | - | - | - | - | - | - | - | - | - | - | - | - | - | - | - | A | G | L | E | A | V | Q | S | N | G | - | - | - | - | L | V | Q | Q | H | A | Y | S | V | T | G | F | A | T | V | S | L | V | L | N | R | T | Y | T | H | I | -217 |
| C3Y6N1_BRAFL | - | - | - | - | - | - | - | - | - | - | - | - | - | - | - | - | - | - | - | - | - | A | G | L | E | A | I | Q | D | N | G | - | - | - | - | L | V | Q | Q | H | A | Y | S | I | T | G | F | A | E | V | - | - | - | - | - | - | - | - | - | - | - | -208 |
| CANA_DROME | - | - | - | - | - | - | - | - | - | - | - | - | - | - | - | - | - | - | - | - | - | N | V | T | E | A | E | T | P | Q | G | - | - | - | - | L | I | R | G | H | A | Y | S | I | T | K | V | C | L | I | D | I | V | T | P | - | - | - | - | - | N | -203 |
| CANB_DROME | - | - | - | - | - | - | - | - | - | - | - | - | - | - | - | - | - | - | - | - | - | H | V | L | E | A | E | T | P | Q | G | - | - | - | - | L | I | R | G | H | A | Y | S | I | T | K | V | C | L | M | D | I | S | T | P | - | - | - | - | - | N | -203 |
| X1Y3H71_ANODA | - | - | - | - | - | - | - | - | - | - | - | - | - | - | - | - | - | - | - | - | - | H | V | L | E | A | E | T | P | E | G | - | - | - | - | L | I | R | G | H | A | Y | S | I | T | K | I | Q | L | V | D | I | E | T | P | - | - | - | - | - | G | -204 |
| Q5IW12_GECLA | - | - | - | - | - | - | - | - | - | - | - | - | - | - | - | - | - | - | - | - | - | N | V | V | E | A | R | C | D | N | G | - | - | - | - | L | I | R | G | H | A | Y | S | I | T | R | I | K | Y | C | D | I | E | T | P | - | - | - | - | - | R | -203 |
| K1Q056_CRAGI | - | - | - | - | - | - | - | - | - | - | - | - | - | - | - | - | - | - | - | - | - | N | Q | L | E | A | R | L | A | N | G | - | - | - | - | L | V | M | G | H | A | Y | S | I | T | S | V | I | L | M | D | I | E | T | P | - | - | - | - | - | T | -203 |
| G4V6C0_SCHMA | - | - | - | - | - | - | - | - | - | - | - | - | - | - | - | - | - | - | - | - | - | H | E | I | E | A | N | G | P | L | G | - | - | - | - | L | I | L | G | H | A | Y | S | V | T | D | I | R | Q | V | H | T | N | Y | G | - | - | - | - | - | S | -208 |
| R7ULX9_CAPTE | - | - | - | - | - | - | - | - | - | - | - | - | - | - | - | - | - | - | - | - | - | D | S | M | E | A | Q | L | D | N | G | - | - | - | - | L | I | I | G | H | A | Y | S | V | T | D | V | R | Y | V | E | V | E | T | P | - | - | - | - | - | R | -204 |
| W4Z5Q7_STRPU | - | - | - | - | - | - | - | - | - | - | - | - | - | - | - | - | - | - | - | - | - | G | V | T | E | A | K | L | P | S | G | - | - | - | - | L | I | A | G | H | A | Y | T | I | T | D | V | K | K | V | N | V | K | G | R | - | - | - | - | - | D | -201 |
| R7VIB6_CAPTE | - | - | - | - | - | - | - | - | - | - | - | - | - | - | - | - | - | - | - | - | - | M | A | L | E | S | E | L | P | T | G | - | - | - | - | L | V | V | G | H | A | Y | S | V | T | A | V | K | S | L | K | V | R | G | H | - | - | - | - | - | D | -207 |
| T2MJ45_HYDVU | - | - | - | - | - | - | - | - | - | - | - | - | - | - | - | - | - | - | - | - | - | N | E | V | E | A | K | L | D | N | G | - | - | - | - | L | I | K | G | H | A | Y | T | I | T | - | - | - | - | - | D | I | K | K | L | - | - | - | - | - | H | -198 |
| R7UAB6_CAPTE | - | - | - | - | - | - | - | - | - | - | - | - | - | - | - | - | - | - | - | - | - | N | Q | T | E | A | K | L | T | N | G | - | - | - | - | L | I | M | G | H | A | Y | T | I | T | R | V | A | F | V | D | I | K | T | S | - | - | - | - | - | R | -203 |
| C3ZKN8_BRAFL | - | - | - | - | - | - | - | - | - | - | - | - | - | - | - | - | - | - | - | - | - | N | A | I | D | S | K | M | D | N | G | - | - | - | - | L | V | M | G | H | A | Y | S | I | T | G | L | Q | K | V | N | V | Q | G | Q | - | - | - | - | - | E | -204 |
| B3RJS5_TRIAD | - | - | - | - | - | - | - | - | - | - | - | - | - | - | - | - | - | - | - | - | - | N | Q | I | E | H | K | L | D | N | G | - | - | - | - | L | V | M | G | H | A | Y | S | V | S | K | A | V | R | V | K | V | R | G | R | - | - | - | - | - | I | -203 |
| H2YVF0_CIOSA | - | - | - | - | - | - | - | - | - | - | - | - | - | - | - | - | - | - | - | - | - | S | Q | R | E | A | K | L | S | N | G | - | - | - | - | L | L | M | G | H | A | Y | S | I | T | G | - | - | - | L | D | I | L | R | N | - | - | - | - | - | G | -201 |
| G4VAG2_SCHMA | - | - | - | - | - | - | - | - | - | - | - | - | - | - | - | - | - | - | - | - | - | - | - | M | E | Q | K | M | D | N | G | - | - | - | - | L | I | G | S | H | A | Y | S | L | T | G | V | Y | P | V | N | Y | R | G | R | - | - | - | - | - | T | -206 |
| Smp_157500 | - | - | - | - | - | - | - | - | - | - | - | - | - | - | - | - | - | - | - | - | - | - | - | N | K | R | R | E | E | L | G | - | - | - | - | L | I | G | S | H | A | Y | S | V | T | G | V | G | K | V | N | Y | M | G | K | - | - | - | - | - | D | -206 |
| Q7SYA9_DANRE | - | - | - | - | - | - | - | - | - | - | - | - | - | - | - | - | - | - | - | - | A | F | D | M | E | S | V | T | F | K | K | - | - | - | - | L | V | K | G | H | A | Y | S | V | T | A | L | K | Q | V | E | Y | R | G | R | M | - | - | - | - | - | -204 |
| A5PMP1_DANRE | - | - | - | - | - | - | - | - | - | - | - | - | - | - | - | - | - | - | - | - | A | F | D | M | E | A | V | T | F | K | K | - | - | - | - | L | V | K | G | H | A | Y | S | V | T | A | L | R | E | V | N | F | R | G | N | R | - | - | - | - | - | -204 |
| CANX_CHICK | - | - | - | - | - | - | - | - | - | - | - | - | - | - | - | - | - | - | - | - | A | F | D | M | E | A | V | T | F | K | K | - | - | - | - | L | V | K | G | H | A | Y | S | V | T | A | F | K | D | V | N | Y | R | G | Q | Q | - | - | - | - | - | -204 |
| CAN1_HUMAN | - | - | - | - | - | - | - | - | - | - | - | - | - | - | - | - | - | - | - | - | V | L | D | M | E | A | I | T | F | K | K | - | - | - | - | L | V | K | G | H | A | Y | S | V | T | G | A | K | Q | V | N | Y | R | G | Q | V | - | - | - | - | - | -204 |
| CAN1_MOUSE | - | - | - | - | - | - | - | - | - | - | - | - | - | - | - | - | - | - | - | - | I | R | D | L | E | A | I | T | F | K | N | - | - | - | - | L | V | R | G | H | A | Y | S | V | T | G | A | K | Q | V | T | Y | Q | G | Q | R | - | - | - | - | - | -204 |
| O42133_CHICK | - | - | - | - | - | - | - | - | - | - | - | - | - | - | - | - | - | - | - | - | A | F | D | M | E | A | V | T | F | K | K | - | - | - | - | L | V | K | G | H | A | Y | S | V | T | G | A | K | Q | I | S | Y | R | G | Q | S | - | - | - | - | - | -204 |
| Q7ZUR1_DANRE | - | - | - | - | - | - | - | - | - | - | - | - | - | - | - | - | - | - | - | - | K | F | D | M | E | A | V | T | F | K | K | - | - | - | - | L | V | K | G | H | A | Y | S | V | T | G | A | E | E | V | V | Y | R | G | N | M | - | - | - | - | - | -204 |
| E9QFN0_DANRE | - | - | - | - | - | - | - | - | - | - | - | - | - | - | - | - | - | - | - | - | F | F | D | M | E | A | I | T | F | K | K | - | - | - | - | L | V | K | G | H | A | Y | S | I | T | G | V | D | E | V | E | Y | R | R | S | Q | - | - | - | - | - | -204 |
| CAN2_HUMAN | - | - | - | - | - | - | - | - | - | - | - | - | - | - | - | - | - | - | - | - | A | A | D | S | E | A | I | T | F | Q | K | - | - | - | - | L | V | K | G | H | A | Y | S | V | T | G | A | E | E | V | E | S | N | G | S | L | - | - | - | - | - | -204 |
| CAN2_MOUSE | - | - | - | - | - | - | - | - | - | - | - | - | - | - | - | - | - | - | - | - | A | A | D | S | E | A | V | T | Y | Q | K | - | - | - | - | L | V | K | G | H | A | Y | S | V | T | G | A | E | E | V | E | S | S | G | S | L | - | - | - | - | - | -204 |
| CAN2_CHICK | - | - | - | - | - | - | - | - | - | - | - | - | - | - | - | - | - | - | - | - | A | A | E | T | E | A | V | T | S | Q | K | - | - | - | - | L | V | K | G | H | A | Y | S | V | T | G | A | E | E | V | N | F | R | G | S | I | - | - | - | - | - | -204 |
| Q6DFZ8_DANRE | - | - | - | - | - | - | - | - | - | - | - | - | - | - | - | - | - | - | - | - | S | S | D | S | E | A | I | T | S | Q | K | - | - | - | - | L | V | K | G | H | A | Y | S | V | T | G | A | E | E | V | E | Y | R | G | D | L | - | - | - | - | - | -204 |
| Q504F5_DANRE | - | - | - | - | - | - | - | - | - | - | - | - | - | - | - | - | - | - | - | - | A | A | D | S | E | A | I | T | R | Q | K | - | - | - | - | L | V | K | G | H | A | Y | S | L | T | G | A | T | E | V | N | Y | R | G | R | K | - | - | - | - | - | -204 |
| Q5BLH5_DANRE | - | - | - | - | - | - | - | - | - | - | - | - | - | - | - | - | - | - | - | - | A | A | D | S | E | A | V | T | Y | Q | K | - | - | - | - | L | V | K | G | H | A | Y | S | L | T | G | A | T | E | V | N | Y | R | S | R | K | - | - | - | - | - | -204 |
| Q4VBH3_DANRE | - | - | - | - | - | - | - | - | - | - | - | - | - | - | - | - | - | - | - | - | S | Y | E | T | E | A | V | T | S | L | K | - | - | - | - | L | V | K | G | H | A | Y | S | V | T | A | A | E | E | V | H | F | R | G | S | L | - | - | - | - | - | -204 |
| CAN8_RAT | - | - | - | - | - | - | - | - | - | - | - | - | - | - | - | - | - | - | - | - | A | A | E | A | E | A | T | T | R | Q | K | - | - | - | - | L | V | K | G | H | A | Y | S | V | T | G | V | E | E | V | N | F | H | G | R | P | - | - | - | - | - | -204 |
| CAN8_MOUSE | - | - | - | - | - | - | - | - | - | - | - | - | - | - | - | - | - | - | - | - | A | A | E | A | E | A | T | T | R | Q | K | - | - | - | - | L | V | K | G | H | A | Y | S | V | T | G | V | E | E | V | D | F | R | G | L | P | - | - | - | - | - | -204 |
| CAN8_HUMAN | - | - | - | - | - | - | - | - | - | - | - | - | - | - | - | - | - | - | - | - | A | A | E | A | E | A | I | T | S | Q | K | - | - | - | - | L | V | K | S | H | A | Y | S | V | T | G | V | E | E | V | N | F | Q | G | H | P | - | - | - | - | - | -204 |
| XP_426117 | - | - | - | - | - | - | - | - | - | - | - | - | - | - | - | - | - | - | - | - | A | S | E | T | E | A | I | T | S | L | K | - | - | - | - | L | V | K | G | H | A | Y | S | V | T | G | A | E | E | V | Y | Y | R | G | R | P | - | - | - | - | - | -204 |
| CAN9_MOUSE | - | - | - | - | - | - | - | - | - | - | - | - | - | - | - | - | - | - | - | - | A | S | E | S | E | A | R | T | S | L | G | - | - | - | - | L | I | K | G | H | A | Y | T | V | T | G | L | D | Q | V | N | F | H | G | Q | R | - | - | - | - | - | -206 |
| CAN9_HUMAN | - | - | - | - | - | - | - | - | - | - | - | - | - | - | - | - | - | - | - | - | A | A | E | S | E | A | R | T | P | F | G | - | - | - | - | L | I | K | G | H | A | Y | S | V | T | G | I | D | Q | V | S | F | R | G | Q | R | - | - | - | - | - | -204 |
| E1C6M3_CHICK | - | - | - | - | - | - | - | - | - | - | - | - | - | - | - | - | - | - | - | - | A | A | E | S | E | A | R | T | P | F | G | - | - | - | - | L | I | K | G | H | A | Y | S | V | T | G | I | D | E | V | S | Y | Q | G | Q | K | - | - | - | - | - | -204 |
| Q6DG44_DANRE | - | - | - | - | - | - | - | - | - | - | - | - | - | - | - | - | - | - | - | - | S | A | E | S | E | A | Q | T | S | T | G | - | - | - | - | L | V | K | G | H | A | Y | S | V | T | G | V | E | E | V | N | Y | R | G | A | K | - | - | - | - | - | -204 |
| CAN3_HUMAN | - | Q | D | S | D | L | D | P | R | G | S | D | E | R | P | T | R | T | I | I | P | V | Q | Y | E | T | R | M | A | C | G | - | - | - | - | L | V | R | G | H | A | Y | S | V | T | G | L | D | E | V | P | F | K | G | E | K | - | - | - | - | - | -252 |
| CAN3_MOUSE | - | R | D | S | D | L | D | P | R | G | S | D | D | R | P | S | R | T | I | V | P | V | Q | Y | E | T | R | M | A | C | G | - | - | - | - | L | V | K | G | H | A | Y | S | V | T | G | L | E | E | A | L | F | K | G | E | K | - | - | - | - | - | -252 |
| CAN3_CHICK | - | T | Y | S | T | V | D | Y | Q | G | T | D | E | R | P | A | W | T | I | M | P | M | Q | Y | E | T | R | M | S | C | G | - | - | - | - | L | V | K | G | H | A | Y | S | V | T | A | V | E | E | T | T | Y | K | G | E | K | - | - | - | - | - | -251 |
| Q66HU4_DANRE | - | - | - | - | - | - | - | - | - | - | - | - | - | - | - | - | - | - | L | V | P | A | R | F | E | T | R | T | A | T | G | - | - | - | - | L | V | K | G | H | A | Y | S | V | T | A | V | E | E | C | K | Q | S | Q | Q | K | E | S | - | - | - | -206 |
| F1R632_DANRE | - | - | - | - | - | - | - | - | - | - | - | - | - | - | - | - | - | - | L | V | P | T | A | Q | R | T | K | T | A | S | G | - | - | - | - | L | V | R | G | H | A | Y | S | V | T | G | V | E | Q | G | K | R | K | D | G | K | D | L | - | - | - | -206 |
| CAN11_HUMAN | - | - | - | - | - | - | - | - | - | - | - | - | - | - | - | - | - | - | - | - | D | S | E | L | E | S | M | T | D | K | M | - | - | - | - | L | V | R | G | H | A | Y | S | V | T | G | L | Q | D | V | H | Y | R | G | K | M | - | - | - | - | - | -204 |
| CAN11_MOUSE | - | - | - | - | - | - | - | - | - | - | - | - | - | - | - | - | - | - | - | - | N | S | E | V | E | T | M | T | H | N | M | - | - | - | - | L | V | R | G | H | A | Y | A | V | T | G | L | E | D | V | Y | Y | R | D | K | L | - | - | - | - | - | -204 |
| CAN12_MOUSE | - | - | - | - | - | - | - | - | - | - | - | - | - | - | - | - | - | - | A | L | S | D | R | G | E | I | R | T | D | E | G | - | - | - | - | L | V | K | G | H | A | Y | S | V | T | G | T | H | K | M | S | L | G | F | T | K | - | - | - | - | - | -201 |
| CAN12_HUMAN | - | - | - | - | - | - | - | - | - | - | - | - | - | - | - | - | - | - | A | L | S | D | R | G | E | Y | R | T | E | E | G | - | - | - | - | L | V | K | G | H | A | Y | S | I | T | G | T | H | K | V | F | L | G | F | T | K | - | - | - | - | - | -201 |
| ENSSARP00000008135 | - | - | - | - | - | - | - | - | - | - | - | - | - | - | - | - | - | - | X | X | S | N | R | G | E | Y | R | T | D | E | G | - | - | - | - | L | V | K | G | H | A | Y | S | V | T | G | T | H | K | V | T | L | G | F | T | K | - | - | - | - | - | -201 |
| ENSSARP00000010553 | - | - | - | - | - | - | - | - | - | - | - | - | - | - | - | - | - | - | I | T | A | S | T | G | M | M | T | E | A | E | G | E | K | W | G | L | V | P | T | H | A | Y | A | V | L | D | I | R | - | - | - | - | E | F | K | G | - | - | - | - | - | -208 |
| A7E2G3_DANRE | - | - | - | - | - | - | - | - | - | - | - | - | - | - | - | - | - | - | T | V | K | D | I | G | - | T | V | T | P | E | G | - | - | - | - | L | I | K | G | H | A | Y | A | I | T | E | T | G | K | V | K | K | A | S | E | E | - | - | - | - | - | -205 |
| Q6NW69_DANRE | - | - | - | - | - | - | - | - | - | - | - | - | - | - | - | - | - | - | - | - | - | - | - | L | E | K | S | N | E | F | G | - | - | - | - | I | L | F | R | H | A | Y | S | V | T | A | L | E | T | V | K | S | T | I | G | P | - | - | - | - | - | -201 |
| CAN13_HUMAN | - | - | - | - | - | - | - | - | - | - | - | - | - | - | - | - | - | - | - | G | P | T | D | T | A | Q | A | M | E | N | G | - | - | - | - | L | V | S | L | H | A | Y | T | V | T | G | A | E | Q | I | Q | Y | R | R | G | W | - | - | - | - | - | -204 |
| CAN13_MOUSE | - | - | - | - | - | - | - | - | - | - | - | - | - | - | - | - | - | - | - | G | L | T | E | E | A | K | V | M | E | N | G | - | - | - | - | L | V | S | Q | H | A | Y | T | V | T | G | A | E | K | I | Q | Y | Q | G | R | W | - | - | - | - | - | -206 |
| E1BRR5_CHICK | - | - | - | - | - | - | - | - | - | - | - | - | - | - | - | - | - | - | - | W | M | R | N | - | - | T | V | L | R | N | G | - | - | - | - | I | V | Q | G | H | A | Y | T | V | T | G | A | V | K | I | R | F | K | N | Q | W | - | - | - | - | - | -201 |
| E7F0H3_DANRE | - | - | - | - | - | - | - | - | - | - | - | - | - | - | - | - | - | - | - | E | G | S | P | E | E | N | E | L | L | H | G | - | - | - | - | I | I | A | D | H | A | Y | T | V | T | G | V | F | E | V | I | S | E | E | N | P | - | - | - | - | - | -204 |
| Q1RLS9_DANRE | - | - | - | - | - | - | - | - | - | - | - | - | - | - | - | - | - | - | - | A | G | V | L | Q | N | T | V | L | P | N | G | - | - | - | - | L | V | E | M | H | A | Y | T | V | T | G | V | T | E | V | V | C | K | G | R | P | - | - | - | - | - | -205 |
| F1Q9Z1_DANRE | - | - | - | - | - | - | - | - | - | - | - | - | - | - | - | - | - | - | - | A | G | - | - | N | E | E | R | L | P | N | G | - | - | - | - | I | V | L | G | H | A | Y | T | V | T | K | V | Y | Q | V | M | S | G | R | N | P | - | - | - | - | - | -201 |
| A4QNV6_DANRE | - | - | - | - | - | - | - | - | - | - | - | - | - | - | - | - | - | - | - | G | K | T | S | A | N | T | E | L | P | N | G | - | - | - | - | I | V | Q | G | H | A | Y | S | V | T | G | I | F | K | V | T | W | Q | G | A | P | - | - | - | - | - | -204 |
| D6W573_HUMAN | - | - | - | - | - | - | - | - | - | - | - | - | - | - | - | - | - | - | - | - | - | H | S | G | E | K | I | L | E | N | G | - | - | - | - | L | V | E | G | H | A | Y | T | L | T | G | I | R | K | V | T | C | K | H | R | P | - | - | - | - | - | -200 |
| R7UBC6_CAPTE | - | - | - | - | - | - | - | - | - | - | - | - | - | - | - | - | - | H | T | H | P | N | R | S | A | P | I | L | S | N | G | - | - | - | - | L | V | T | G | R | T | Y | N | V | T | D | L | R | E | I | R | V | V | S | S | - | K | D | E | - | - | -215 |
| K1RCZ2_CRAGI | - | - | - | - | - | - | - | - | - | - | - | - | - | - | - | - | - | P | V | K | G | N | Q | G | E | Q | K | L | P | S | G | - | - | - | - | L | V | A | G | H | A | Y | S | V | T | D | L | R | E | I | L | L | M | S | D | - | N | R | E | - | - | -217 |
| Q5IW13_GECLA | - | - | - | - | - | - | - | - | - | - | - | - | - | - | - | - | - | D | H | L | G | V | R | P | E | S | I | M | S | N | G | - | - | - | - | L | I | V | R | H | A | Y | S | I | T | R | V | T | A | I | S | L | A | T | A | P | K | L | K | - | - | -208 |
| R7UUX7_CAPTE | - | - | - | - | - | - | - | - | - | - | - | - | - | - | - | - | - | - | - | A | T | S | H | R | E | E | D | L | G | N | G | - | - | - | - | L | Y | A | G | H | A | Y | S | V | T | N | I | I | Q | A | T | V | D | G | D | - | - | - | - | - | - | -204 |
| K1Q505_CRAGI | - | - | - | - | - | - | - | - | - | - | - | - | - | - | - | - | - | N | V | A | V | S | S | R | E | V | R | R | P | N | G | - | - | - | - | L | Y | M | G | H | A | Y | S | I | T | A | L | A | V | I | P | Y | Q | G | S | - | - | - | - | - | - | -210 |
| R7UD02_CAPTE | - | - | - | - | - | - | - | - | - | - | - | - | - | - | - | - | - | - | - | D | D | G | T | Y | E | S | R | L | D | N | G | - | - | - | - | L | V | T | G | H | A | Y | S | L | T | N | I | S | E | V | T | V | N | E | A | - | - | - | - | - | - | -206 |
| CAN6_HUMAN | - | - | - | - | - | - | - | - | - | - | - | - | - | - | - | - | - | - | - | - | - | E | E | Q | E | V | E | T | D | W | G | - | - | - | - | L | L | K | G | H | T | Y | T | M | T | D | I | R | K | I | R | L | G | - | E | R | L | V | E | V | F | -222 |
| CAN6_MOUSE | - | - | - | - | - | - | - | - | - | - | - | - | - | - | - | - | - | - | - | - | - | E | E | Q | E | V | E | T | D | W | G | - | - | - | - | L | L | K | G | Y | T | Y | T | M | T | D | I | R | K | L | R | L | G | - | E | R | L | V | E | V | F | -222 |
| E1C312_CHICK | - | - | - | - | - | - | - | - | - | - | - | - | - | - | - | - | - | - | - | - | - | R | A | N | E | V | E | A | K | T | G | - | - | - | - | L | V | T | G | H | A | Y | S | V | T | A | I | R | K | V | H | L | G | - | E | R | L | T | F | S | F | -222 |
| E7FDN3_DANRE | - | - | - | - | - | - | - | - | - | - | - | - | - | - | - | - | - | - | - | - | - | E | A | L | E | S | V | L | D | C | G | - | - | - | - | L | I | K | G | H | A | Y | G | V | T | A | V | K | K | L | R | M | S | - | E | T | L | H | S | M | C | -223 |
| CAN5_HUMAN | - | - | - | - | - | - | - | - | - | - | - | - | - | - | - | - | - | - | - | - | - | A | D | M | E | A | R | L | A | C | G | - | - | - | - | L | V | K | G | H | A | Y | A | V | T | D | V | R | K | V | R | L | G | - | H | G | L | L | A | F | F | -218 |
| CAN5_MOUSE | - | - | - | - | - | - | - | - | - | - | - | - | - | - | - | - | - | - | - | - | - | A | D | M | E | A | R | L | A | C | G | - | - | - | - | L | V | K | G | H | A | Y | A | V | T | D | V | R | K | V | R | L | G | - | H | G | L | L | A | F | F | -222 |
| E1C292_CHICK | - | - | - | - | - | - | - | - | - | - | - | - | - | - | - | - | - | - | - | - | - | A | D | M | E | A | R | L | A | C | G | - | - | - | - | L | V | K | G | H | A | Y | A | V | T | D | V | R | K | V | R | L | G | - | H | G | L | L | S | F | F | -222 |
| A0JMG3_DANRE | - | - | - | - | - | - | - | - | - | - | - | - | - | - | - | - | - | - | - | - | - | A | D | M | E | A | R | L | D | C | G | - | - | - | - | L | V | K | G | H | A | Y | A | V | T | D | V | R | K | V | R | L | G | - | S | G | L | L | A | F | F | -222 |
| E7F7F1_DANRE | - | - | - | - | - | - | - | - | - | - | - | - | - | - | - | - | - | - | - | - | - | E | D | M | E | A | R | L | D | C | G | - | - | - | - | L | V | K | G | H | A | Y | A | V | T | D | V | R | K | V | R | L | G | - | H | G | L | L | A | Y | F | -222 |
| XP_006824412 | - | - | - | - | - | - | - | - | - | - | - | - | - | - | - | - | - | - | - | - | - | S | E | M | E | A | K | T | E | V | G | - | - | - | - | L | I | K | G | H | A | Y | G | V | T | D | V | R | N | V | K | I | G | D | T | G | L | L | S | Y | F | -215 |
| W4YJN2_STRPU | - | - | - | - | - | - | - | - | - | - | - | - | - | - | - | - | - | - | - | - | - | E | E | M | E | A | R | T | D | V | G | - | - | - | - | L | V | K | G | H | A | Y | G | V | T | A | V | K | S | V | K | L | G | - | - | - | S | S | G | F | F | -220 |
| C3YJZ3_BRAFL | - | - | - | - | - | - | - | - | - | - | - | - | - | - | - | - | - | - | - | - | - | D | E | M | E | A | S | T | E | S | G | - | - | - | - | L | V | K | G | H | A | Y | S | V | T | A | V | K | K | V | K | V | G | E | S | G | M | L | S | G | I | -223 |
| T2MIT4_HYDVU | - | - | - | - | - | - | - | - | - | - | - | - | - | - | - | - | - | - | - | - | - | N | E | M | E | L | Q | L | D | T | G | - | - | - | - | L | I | K | G | H | A | Y | G | I | S | A | V | K | K | I | T | L | G | - | Q | N | F | F | S | S | F | -222 |
| XP_002154722 | - | - | - | - | - | - | - | - | - | - | - | - | - | - | - | - | - | - | - | - | - | D | E | M | E | L | Q | M | D | H | G | - | - | - | - | L | V | K | G | H | A | Y | S | I | L | A | V | K | K | I | K | I | G | - | N | D | H | I | S | I | F | -186 |
| R7VLA7_CAPTE | - | - | - | - | - | - | - | - | - | - | - | - | - | - | - | - | - | - | - | - | - | E | E | Q | E | E | V | M | D | M | G | - | - | - | - | L | V | K | G | H | A | Y | G | I | T | A | V | K | R | I | A | L | E | G | T | G | I | F | N | M | F | -223 |
| K1R1C6_CRAGI | - | - | - | - | - | - | - | - | - | - | - | - | - | - | - | - | - | - | - | - | - | E | E | M | E | A | S | T | E | T | G | - | - | - | - | L | V | K | G | H | A | Y | G | V | T | A | V | K | N | V | A | L | E | G | S | G | L | F | S | F | F | -223 |
| B3RIE5_TRIAD | - | - | - | - | - | - | - | - | - | - | - | - | - | - | - | - | - | - | - | - | - | T | K | R | E | E | R | T | D | M | G | - | - | - | - | L | I | L | L | H | A | Y | G | V | T | K | V | T | N | F | K | L | - | G | E | G | L | R | S | F | F | -220 |
| K1PW75_CRAGI | - | - | - | - | - | - | - | - | - | - | - | - | - | - | - | - | - | - | - | - | - | N | D | M | E | A | Q | L | S | C | G | - | - | - | - | L | V | K | G | H | A | Y | S | V | N | A | V | K | K | V | R | L | G | - | K | G | L | M | S | H | F | -218 |
| K1RSB8_CRAGI | - | - | - | - | - | - | - | - | - | - | - | - | - | - | - | - | - | - | - | - | - | K | E | M | E | E | K | L | S | C | G | - | - | - | - | L | V | K | G | H | A | Y | S | V | T | N | V | R | K | L | K | L | G | - | T | G | L | K | S | I | F | -216 |
| K1RWI3_CRAGI | - | - | - | - | - | - | - | - | - | - | - | - | - | - | - | - | - | - | - | - | - | D | E | M | E | A | K | T | N | T | G | - | - | - | - | L | V | K | G | H | A | Y | S | V | T | A | V | R | N | I | K | L | G | - | Q | G | L | L | S | F | F | -219 |
| R7VGC2_CAPTE | - | - | - | - | - | - | - | - | - | - | - | - | - | - | - | - | - | - | - | - | - | D | D | I | G | T | E | A | G | H | G | - | - | - | - | L | I | K | G | H | G | Y | A | I | T | A | V | R | T | V | S | I | A | - | K | A | L | K | G | - | - | -218 |
| K1QU26_CRAGI | - | - | - | - | - | - | - | - | - | - | - | - | - | - | - | - | - | - | - | - | - | - | E | V | G | K | E | L | P | N | G | - | - | - | - | L | I | L | G | H | G | Y | N | I | T | K | V | L | E | S | K | V | E | - | K | K | L | Q | G | - | - | -219 |
| CAN5_CAEEL | - | - | - | - | - | - | - | - | - | - | - | - | - | - | - | - | - | - | - | - | - | E | E | I | E | E | S | L | D | C | G | - | - | - | - | L | V | K | G | H | A | Y | A | V | S | A | V | C | T | I | D | V | T | - | N | P | N | E | R | S | F | -220 |
| I1FGV4_AMPQE | - | - | - | - | - | - | - | - | - | - | - | - | - | - | - | - | - | - | - | - | - | D | E | V | E | A | K | L | D | S | G | - | - | - | - | L | I | K | G | H | A | Y | S | V | T | G | A | K | T | L | N | M | K | - | - | - | - | - | - | - | - | -201 |
| K1PHX3_CRAGI | - | - | - | - | - | - | - | - | - | - | - | - | - | - | - | - | - | - | - | - | - | Q | G | Q | R | R | R | L | S | N | G | - | - | - | - | L | V | T | G | H | A | Y | S | V | T | G | Y | A | Q | I | P | V | K | - | - | - | - | - | - | - | - | -201 |
| B3RNL8_TRIAD | - | - | - | - | - | - | - | - | - | - | - | - | - | - | - | - | - | - | - | - | - | - | V | K | G | H | V | L | P | N | G | - | - | - | - | L | V | W | G | H | A | Y | S | V | D | K | A | A | K | I | E | G | N | - | - | - | - | - | - | - | - | -202 |
| X1Y3H7_ANODA | - | - | - | - | - | - | - | - | - | - | - | - | - | - | - | - | - | - | - | - | - | - | - | D | D | L | L | Q | L | V | N | - | - | - | - | L | V | K | G | H | E | Y | S | I | T | R | V | E | R | L | N | S | H | Q | H | M | I | G | M | E | D | -212 |
| R7TWH9_CAPTE | - | - | - | - | - | - | - | - | - | - | - | - | - | - | - | - | - | - | - | - | - | K | A | T | K | - | A | E | A | N | G | - | - | - | - | L | V | S | G | H | A | Y | T | I | T | R | V | M | R | I | S | H | R | M | G | E | E | R | - | - | - | -209 |
| K1R5E8_CRAGI | - | - | - | - | - | - | - | - | - | - | - | - | - | - | - | - | - | - | - | - | - | L | S | T | K | - | A | D | P | N | G | - | - | - | - | L | V | S | G | H | A | Y | T | V | T | E | V | R | K | I | K | H | V | R | G | E | D | K | - | - | - | -209 |
| XP_006811804 | - | - | - | - | - | - | - | - | - | - | - | - | - | - | - | - | - | - | - | - | - | A | A | D | E | - | A | D | E | H | G | - | - | - | - | L | V | S | G | H | A | Y | T | V | T | S | V | R | R | V | Q | T | R | H | G | D | Y | V | N | - | - | -208 |
| C3YB23_BRAFL | - | - | - | - | - | - | - | - | - | - | - | - | - | - | - | - | - | - | - | - | - | L | T | Q | H | G | V | T | A | Q | G | - | - | - | - | L | V | G | G | H | A | Y | S | I | T | S | V | H | I | V | H | S | N | F | G | D | F | P | - | - | - | -215 |
| H2ZIF1_CIOSA | - | - | - | - | - | - | - | - | - | - | - | - | - | - | - | - | - | - | - | - | - | S | V | D | S | - | - | - | T | T | G | - | - | - | - | L | V | S | A | H | A | Y | S | V | L | K | V | A | K | V | N | Y | R | - | G | D | T | V | K | - | - | -205 |
| W4YV93_STRPU | - | - | - | - | - | - | - | - | - | - | - | - | - | - | - | - | - | - | - | - | - | A | N | E | T | - | - | N | N | L | G | - | - | - | - | I | V | T | G | H | S | Y | A | L | N | R | V | Q | T | G | V | L | K | D | G | S | K | V | R | - | - | -207 |
| CAN10_HUMAN | - | - | - | - | - | - | - | - | - | - | - | - | - | - | - | - | - | - | - | - | - | V | L | S | P | R | A | G | A | R | E | - | - | - | - | L | G | E | F | H | A | F | I | V | S | D | L | R | E | L | Q | G | Q | A | G | Q | C | - | - | - | - | -213 |
| CAN10_MOUSE | - | - | - | - | - | - | - | - | - | - | - | - | - | - | - | - | - | - | - | - | - | V | L | S | P | R | A | G | A | R | E | - | - | - | - | L | G | E | F | H | A | F | I | I | S | D | L | Q | E | L | R | S | Q | T | G | Q | G | - | - | - | - | -213 |
| XP_004943443 | - | - | - | - | - | - | - | - | - | - | - | - | - | - | - | - | - | - | - | - | - | V | L | N | S | R | Q | G | A | S | E | - | - | - | - | L | G | E | F | H | A | F | I | V | T | D | M | L | N | L | S | E | V | S | G | K | E | - | - | - | - | -213 |
| F8W4K1_DANRE | - | - | - | - | - | - | - | - | - | - | - | - | - | - | - | - | - | - | - | - | - | V | H | S | A | T | T | G | F | P | E | - | - | - | - | L | G | Q | F | H | A | M | C | V | M | E | W | T | S | V | T | T | I | T | G | E | G | - | - | - | - | -216 |
| CANC_DROME | - | - | - | - | - | - | - | - | - | - | - | - | - | - | - | - | - | - | - | - | - | K | N | L | A | E | R | M | P | N | G | - | - | - | - | I | L | V | N | V | N | Y | R | L | S | S | L | D | K | V | K | T | L | M | G | D | S | - | - | - | - | -195 |
| C3ZKN9_BRAFL | - | - | - | - | - | - | - | - | - | - | - | - | - | - | - | - | - | - | - | - | - | L | L | S | C | Y | I | S | S | P | E | - | - | - | - | K | P | P | Q | R | K | L | T | A | S | G | L | Y | A | E | Q | A | Y | A | I | S | A | - | - | - | - | -195 |
| G4V623_SCHMA | - | - | - | - | - | - | - | - | - | - | - | - | - | - | - | - | - | - | - | - | - | V | G | F | V | P | G | Q | A | F | G | - | - | - | - | L | T | D | C | R | K | L | R | L | T | D | A | S | G | S | R | L | L | - | - | - | - | - | - | - | - | -225 |
| R7VK39_CAPTE | - | - | - | - | - | - | - | - | - | - | - | - | - | - | - | - | - | - | - | - | - | D | D | D | E | E | E | M | W | N | G | - | - | - | - | L | V | R | G | H | V | Y | I | V | T | A | V | I | E | S | R | I | E | G | W | T | - | - | - | - | - | -205 |
| G4V693_SCHMA | - | - | - | - | - | - | - | - | - | - | - | - | - | - | - | - | - | A | P | N | L | H | D | H | V | L | N | K | E | S | G | - | - | - | - | L | Y | L | N | Y | G | Y | L | L | T | R | V | C | A | V | P | K | D | I | S | V | F | G | A | F | K | -239 |
| R7UPU6_CAPTE | - | - | - | - | - | - | - | - | - | - | - | - | - | - | - | - | - | - | - | - | - | - | - | D | A | M | D | G | Q | M | G | - | - | - | - | L | V | G | G | H | A | Y | T | I | T | G | A | I | K | I | E | Y | Q | G | S | A | - | - | - | - | - | -205 |
| R7V817_CAPTE | - | - | - | - | - | - | - | - | - | - | - | - | - | - | - | - | - | - | - | - | - | - | - | D | A | I | D | G | Q | M | G | - | - | - | - | L | V | G | D | H | A | Y | T | I | T | G | A | I | M | I | E | Y | R | G | S | V | - | - | - | - | - | -204 |
| R7UNL8_CAPTE | - | - | - | - | - | - | - | - | - | - | - | - | - | - | - | - | - | - | - | - | - | - | - | - | - | V | N | G | I | K | G | - | - | - | - | L | V | G | N | H | A | Y | T | I | T | G | T | F | R | I | N | H | R | A | Q | S | - | - | - | - | - | -198 |
| K1PJ80_CRAGI | - | - | - | - | - | - | - | - | - | - | - | - | - | - | - | - | - | - | - | - | - | - | - | G | E | F | D | G | Q | H | G | - | - | - | - | L | V | G | G | H | E | Y | T | L | T | G | A | V | T | A | N | G | - | - | - | - | - | - | - | - | - | -201 |
| K1PCX8_CRAGI | - | - | - | - | - | - | - | - | - | - | - | - | - | - | - | - | - | - | - | - | - | - | - | G | T | F | V | K | K | Y | G | - | - | - | - | L | L | G | S | H | E | Y | T | I | N | G | A | V | T | A | K | G | - | - | - | - | - | - | - | - | - | -201 |
| CAND_DROME | - | - | - | - | - | - | - | - | - | - | - | - | - | - | - | - | - | - | - | - | - | - | - | - | - | - | - | - | - | E | Y | Q | Q | K | G | L | R | P | R | H | A | Y | S | V | L | D | V | K | D | I | Q | - | - | - | - | - | - | - | - | - | - | -213 |
| XP_002735765 | - | - | - | - | - | - | - | - | - | - | - | - | - | - | - | - | - | - | - | - | - | - | - | - | - | - | - | - | - | D | Y | H | D | I | G | L | R | P | R | H | A | Y | S | V | L | D | V | Q | D | I | L | V | C | - | - | - | - | - | - | - | - | -216 |
| CAN15_HUMAN | - | - | - | - | - | - | - | - | - | - | - | - | - | - | - | - | - | - | - | - | - | - | - | - | - | - | - | - | - | A | Y | E | S | L | G | L | R | P | R | H | A | Y | S | I | L | D | V | R | D | V | Q | - | - | - | - | - | - | - | - | - | - | -214 |
| CAN15_MOUSE | - | - | - | - | - | - | - | - | - | - | - | - | - | - | - | - | - | - | - | - | - | - | - | - | - | - | - | - | - | A | Y | E | S | L | G | L | R | P | R | H | A | Y | S | V | L | D | V | R | D | V | Q | - | - | - | - | - | - | - | - | - | - | -214 |
| E1BTA8_CHICK | - | - | - | - | - | - | - | - | - | - | - | - | - | - | - | - | - | - | - | - | - | - | - | - | - | - | - | - | - | A | Y | E | S | M | G | L | R | P | R | H | A | Y | S | I | L | D | V | R | D | V | Q | - | - | - | - | - | - | - | - | - | - | -214 |
| E7FAX5_DANRE | - | - | - | - | - | - | - | - | - | - | - | - | - | - | - | - | - | - | - | - | - | - | - | - | - | - | - | - | - | V | Y | E | S | L | G | L | R | P | R | H | A | Y | S | I | L | D | V | R | D | V | Q | - | - | - | - | - | - | - | - | - | - | -214 |
| C3XWK8_BRAFL | - | - | - | - | - | - | - | - | - | - | - | - | - | - | - | - | - | - | - | - | - | - | - | - | - | - | - | - | - | E | Y | E | S | K | G | L | R | P | R | H | A | Y | S | V | L | D | V | Q | D | V | N | - | - | - | - | - | - | - | - | - | - | -214 |
| W4YMU4_STRPU | - | - | - | - | - | - | - | - | - | - | - | - | - | - | - | - | - | - | - | - | - | - | - | - | - | - | - | - | - | L | Y | Q | D | V | G | L | R | P | R | H | S | Y | S | V | L | D | I | Q | D | I | C | - | - | - | - | - | - | - | - | - | - | -328 |
| K1PV74_CRAGI | - | - | - | - | - | - | - | - | - | - | - | - | - | - | - | - | - | - | - | - | - | - | - | - | - | - | - | - | - | V | Y | E | E | K | G | L | R | P | R | H | A | Y | S | I | L | D | V | K | D | I | D | - | - | - | - | - | - | - | - | - | - | -211 |
| R7TJF2_CAPTE | - | - | - | - | - | - | - | - | - | - | - | - | - | - | - | - | - | - | - | - | - | - | - | - | - | - | - | - | - | V | Y | N | E | I | G | L | K | A | R | H | A | Y | S | V | L | D | I | R | S | V | R | A | L | S | L | - | - | - | - | - | - | -214 |
| T2MI17_HYDVU | - | - | - | - | - | - | - | - | - | - | - | - | - | - | - | - | - | - | - | - | - | - | - | - | - | - | - | - | - | A | Y | T | K | V | G | L | H | A | Q | H | A | Y | S | V | L | D | I | Q | Q | F | G | - | - | - | - | - | - | - | - | - | - | -212 |
| Q1RL55_CIOIN | - | - | - | - | - | - | - | - | - | - | - | - | - | - | - | - | - | - | - | - | - | - | - | - | - | - | - | - | - | E | Y | N | Q | I | G | L | R | P | R | H | A | Y | S | V | L | R | V | T | S | E | Y | N | - | - | I | - | - | - | - | - | - | -200 |
| I1EA30_AMPQE | - | - | - | - | - | - | - | - | - | - | - | - | - | - | - | - | - | - | - | - | - | - | - | - | - | - | - | - | - | I | D | T | E | L | G | I | Q | P | F | H | A | Y | S | I | L | D | I | K | Q | I | G | - | - | - | - | - | - | - | - | - | - | -209 |
| G5EEZ6_CAEEL | - | - | - | - | - | - | - | - | - | - | - | - | - | - | - | - | - | - | - | - | - | - | - | - | - | - | - | - | - | E | F | K | A | M | G | L | L | T | N | H | A | Y | S | I | L | D | V | I | Y | E | Q | - | - | - | - | - | - | - | - | - | - | -210 |
| O44903_CAEEL | - | - | - | - | - | - | - | - | - | - | - | - | - | - | - | - | - | - | - | - | - | - | - | - | - | - | - | - | - | E | F | K | A | L | G | L | V | T | S | H | A | Y | S | I | L | D | V | V | H | E | Q | - | - | - | - | - | - | - | - | - | - | -210 |
| O02260_CAEEL | - | - | - | - | - | - | - | - | - | - | - | - | - | - | - | - | - | - | - | - | - | - | - | - | - | - | - | - | - | - | - | - | V | N | G | I | L | P | Q | H | A | Y | T | I | M | D | I | V | E | R | D | - | - | - | - | - | - | - | - | - | - | -195 |
| O02259_CAEEL | - | - | - | - | - | - | - | - | - | - | - | - | - | - | - | - | - | - | - | - | - | - | - | - | - | - | - | - | - | - | - | - | R | N | G | I | L | S | K | H | V | Y | T | I | M | D | I | V | E | R | D | - | - | - | - | - | - | - | - | - | - | -186 |
| C1MTG3_MICPC | - | - | - | - | - | - | - | - | - | - | - | - | - | - | - | - | - | - | - | - | - | - | - | - | - | - | - | - | - | - | - | - | - | - | - | L | V | G | G | H | A | Y | S | V | L | D | V | R | Q | L | H | G | V | A | V | G | R | Q | T | K | L | -256 |
| C1E493_MICSR | - | - | - | - | - | - | - | - | - | - | - | - | - | - | - | - | - | - | - | - | - | - | - | - | - | - | - | - | - | - | - | - | - | - | - | L | V | G | N | H | A | Y | S | V | M | E | V | R | E | L | H | G | A | S | V | G | R | Q | T | K | L | -206 |
| Q012Z6_OSTTA | - | - | - | - | - | - | - | - | - | - | - | - | - | - | - | - | - | - | - | - | - | - | - | - | - | - | - | - | - | - | - | - | - | - | - | I | V | G | H | H | A | Y | S | V | L | E | V | R | E | V | V | G | V | K | K | G | V | Q | T | K | L | -199 |
| L1IGB6_GUITH | - | - | - | - | - | - | - | - | - | - | - | - | - | - | - | - | - | - | - | - | - | - | - | - | - | - | - | - | - | - | - | - | - | - | - | L | C | G | N | H | A | Y | S | I | L | E | C | R | E | V | P | - | D | R | R | G | R | D | G | - | - | -203 |
| B7FWX6_PHATC | - | - | - | - | - | - | - | - | - | - | - | - | - | - | - | - | - | - | - | - | - | - | - | - | - | - | - | - | - | - | - | - | - | - | - | L | V | G | G | H | A | Y | S | L | L | D | V | I | E | I | N | N | S | L | I | G | E | Q | K | K | V | -206 |
| N9TPR9_ENTHI | - | - | - | - | - | - | - | - | - | - | - | - | - | - | - | - | - | - | - | - | - | - | - | - | - | - | - | - | - | - | - | - | E | Y | G | L | V | E | N | H | A | Y | T | L | I | G | A | Y | E | V | D | G | - | - | - | - | - | - | - | - | - | -192 |
| G0QS43_ICHMG | - | - | - | - | - | - | - | - | - | - | - | - | - | - | - | - | - | - | - | - | - | - | - | - | - | - | - | - | - | - | - | - | K | M | G | I | V | K | A | H | A | Y | S | L | L | S | A | Y | D | L | D | I | G | G | N | - | - | - | - | - | - | -203 |
| D8LB20_ECTSI | - | - | - | - | - | - | - | - | - | - | - | - | - | - | - | - | - | - | - | - | - | - | - | - | - | - | - | - | - | - | - | - | K | D | G | L | I | E | N | H | A | Y | S | I | M | R | A | I | D | M | H | G | - | - | - | - | - | - | - | - | - | -238 |
| Q9U0T9_LEIMA | - | - | - | - | - | - | - | - | - | - | - | - | - | - | - | - | - | - | - | - | - | - | - | - | - | - | - | E | E | A | F | A | G | T | G | F | L | P | G | H | A | Y | S | V | L | D | V | K | E | F | Q | K | - | - | - | - | - | - | - | - | - | -228 |
| Q4CXJ8_TRYCC | - | - | - | - | - | - | - | - | - | - | - | - | - | - | - | - | - | - | - | - | - | - | - | - | - | - | - | E | R | I | L | G | G | T | G | I | L | P | G | H | A | Y | T | I | T | D | V | R | Y | F | P | K | E | - | - | - | - | - | - | - | - | -230 |
| F4I0A4_ARATH | - | - | - | - | - | - | - | - | - | - | - | - | - | - | - | - | - | - | - | - | - | - | - | - | - | - | - | - | - | - | - | - | S | S | G | I | V | Q | G | H | A | Y | S | V | L | Q | V | R | E | V | D | - | - | - | - | - | - | - | - | - | - | -206 |
| Q8RUQ1_MAIZE | - | - | - | - | - | - | - | - | - | - | - | - | - | - | - | - | - | - | - | - | - | - | - | - | - | - | - | - | - | - | - | - | S | S | G | I | V | Q | G | H | A | Y | S | I | L | Q | V | R | E | V | D | - | - | - | - | - | - | - | - | - | - | -206 |
| D7FQ17_ECTSI | - | - | - | - | - | - | - | - | - | - | - | - | - | - | - | - | - | - | - | - | - | - | - | - | - | - | - | - | - | - | - | - | D | I | G | L | V | F | G | A | A | Y | T | I | L | D | V | R | D | S | D | - | - | - | - | - | - | - | - | - | - | -223 |
| D0MZ44_PHYIT | - | - | - | - | - | - | - | - | - | - | - | - | - | - | - | - | - | - | - | - | - | - | - | - | - | - | - | - | - | - | - | - | A | S | G | L | V | F | D | A | C | Y | V | I | Y | D | V | R | D | V | D | - | - | - | - | - | - | - | - | - | - | -224 |
| L1JBM0_GUITH | - | - | - | - | - | - | - | - | - | - | - | - | - | - | - | - | - | - | - | - | - | - | - | - | - | - | - | - | - | - | - | T | S | G | N | I | I | R | D | H | A | Y | S | I | L | S | V | Y | S | V | P | H | V | P | - | - | - | - | - | - | - | -174 |
| L1J739_GUITH | - | - | - | - | - | - | - | - | - | - | - | - | - | - | - | - | - | - | - | - | - | - | - | - | - | - | - | - | - | - | - | S | D | G | G | I | L | K | G | H | A | Y | S | I | V | D | L | K | E | Y | R | - | - | - | - | - | - | - | - | - | - | -211 |
| L1J0U9_GUITH | - | - | - | - | - | - | - | - | - | - | - | - | - | - | - | - | - | - | - | - | - | - | - | - | - | - | - | - | - | - | - | F | D | G | D | F | K | S | H | C | V | Y | S | I | L | D | V | K | E | P | A | V | - | - | - | - | - | - | - | - | - | -203 |
| L1J1Q4_GUITH | - | - | - | - | - | - | - | - | - | - | - | - | - | - | - | - | - | - | - | - | - | - | - | - | - | - | - | - | - | - | - | P | C | G | - | L | L | V | N | H | G | Y | A | I | L | D | V | E | T | I | P | D | N | - | - | - | - | - | - | - | - | -223 |
| D0ND88_PHYIT | - | - | - | - | - | - | - | - | - | - | - | - | - | - | - | - | - | - | - | - | - | - | - | - | - | - | - | - | - | - | - | A | M | G | - | I | V | K | N | R | Q | Y | V | I | L | H | V | T | E | V | G | L | P | S | G | Q | G | G | P | N | - | -259 |
| D2VVS6_NAEGR | - | - | - | - | - | - | - | - | - | - | - | - | - | - | - | - | - | - | - | - | - | - | - | - | - | - | - | - | - | - | - | - | - | - | G | I | I | N | F | H | A | Y | A | I | L | D | A | I | E | F | P | P | H | L | T | N | - | - | - | - | - | -207 |
| D2V3V2_NAEGR | - | - | - | - | - | - | - | - | - | - | - | - | - | - | - | - | - | - | - | - | - | - | - | - | - | - | - | - | - | - | - | - | - | - | G | I | Q | T | H | H | A | Y | A | I | L | D | C | F | D | C | P | L | E | M | T | N | - | - | - | - | - | -207 |
| Q5B5J7_EMENI | - | - | - | - | - | - | - | - | - | - | - | - | - | - | - | - | - | - | - | - | - | - | - | - | - | - | - | G | P | P | R | D | R | K | G | I | S | E | N | H | S | Y | S | I | M | D | V | K | E | I | D | G | V | R | - | - | - | - | - | - | - | -232 |
| E4ZY10_LEPMJ | - | - | - | - | - | - | - | - | - | - | - | - | - | - | - | - | - | - | - | - | - | - | - | - | - | - | - | G | I | Y | G | A | R | K | G | I | Q | E | K | H | A | Y | S | V | M | E | A | R | E | I | D | G | E | R | - | - | - | - | - | - | - | -228 |
| A8PIE5_COPC7 | - | - | - | - | - | - | - | - | - | - | - | - | - | - | - | - | - | - | - | - | - | - | - | - | - | - | - | Q | - | S | P | D | V | E | G | L | Y | G | N | H | A | Y | S | I | M | R | A | V | E | T | R | G | K | R | - | - | - | - | - | - | - | -232 |
| A8NBA6_COPC7 | - | - | - | - | - | - | - | - | - | - | - | - | - | - | - | - | - | - | - | - | - | - | - | - | - | - | - | - | - | N | A | R | V | Q | G | L | V | G | S | H | A | Y | S | V | L | R | A | V | E | C | K | G | R | R | - | - | - | - | - | - | - | -229 |
| A8NBA1_COPC7 | - | - | - | - | - | - | - | - | - | - | - | - | - | - | - | - | - | - | - | - | - | - | - | - | - | - | - | - | - | G | T | V | T | Q | G | L | I | P | N | H | A | Y | S | V | L | R | A | I | E | W | K | G | K | R | - | - | - | - | - | - | - | -223 |
| A8NBA2_COPC7 | - | - | - | - | - | - | - | - | - | - | - | - | - | - | - | - | - | - | - | - | - | - | - | - | - | - | - | - | - | G | D | E | T | Q | G | L | I | S | E | H | A | Y | S | I | L | K | A | I | E | W | N | G | K | R | - | - | - | - | - | - | - | -223 |
| A8NAE6_COPC7 | - | - | - | - | - | - | - | - | - | - | - | - | - | - | - | - | - | - | - | - | - | - | - | - | - | - | - | - | - | N | A | T | V | D | G | L | V | G | N | L | S | H | S | V | I | K | A | V | E | C | R | G | K | R | - | - | - | - | - | - | - | -251 |
| A8NAI9_COPC7 | - | - | - | - | - | - | - | - | - | - | - | - | - | - | - | - | - | - | - | - | - | - | - | - | - | - | - | Q | - | T | Y | N | T | E | N | L | V | F | R | H | A | Y | S | V | L | R | A | R | Q | V | C | G | R | R | - | - | - | - | - | - | - | -227 |
| G4TER7_PIRID | - | - | - | - | - | - | - | - | - | - | - | - | - | - | - | - | - | - | - | - | - | - | - | - | - | - | - | - | - | I | T | K | T | H | G | L | I | A | S | H | A | Y | S | V | I | R | A | V | E | Y | N | G | K | K | - | - | - | - | - | - | - | -229 |
| G4TER6_PIRID | - | - | - | - | - | - | - | - | - | - | - | - | - | - | - | - | - | - | - | - | - | - | - | - | - | - | - | - | - | H | D | S | E | Y | G | L | I | A | S | H | A | Y | T | V | L | K | A | V | E | Y | K | G | K | R | - | - | - | - | - | - | - | -218 |
| G4TNA1_PIRID | - | - | - | - | - | - | - | - | - | - | - | - | - | - | - | - | - | - | - | - | - | - | - | - | - | - | - | - | - | V | K | Q | E | N | - | L | I | Y | N | H | A | Y | A | V | L | K | A | A | E | Y | N | G | K | R | - | - | - | - | - | - | - | -228 |
| D0MWB9_PHYIT | - | - | - | - | - | - | - | - | - | - | - | - | - | - | - | - | - | - | - | - | - | - | - | - | - | - | - | V | E | S | S | A | G | N | G | L | Y | F | K | H | A | Y | A | L | V | D | A | A | E | I | K | T | A | K | G | E | I | V | R | - | - | -226 |
| L1I9H5_GUITH | - | - | - | - | - | - | - | - | - | - | - | - | - | - | - | - | - | - | - | - | - | - | - | - | G | N | P | Q | S | M | G | - | - | - | - | L | V | D | G | H | A | Y | S | V | L | K | M | A | E | V | A | A | G | - | - | - | - | - | - | - | - | -216 |
| Q7N497_PHOLL | - | - | - | - | - | - | - | - | - | - | - | - | - | - | - | - | - | - | - | - | - | - | - | - | - | - | - | - | - | - | - | - | - | - | - | L | V | S | G | H | A | Y | L | I | L | D | A | Q | Q | W | N | D | - | - | - | - | - | - | - | - | - | -175 |
| ZP_11152369 | - | - | - | - | - | - | - | - | - | - | - | - | - | - | - | - | - | - | - | - | - | - | - | - | - | - | - | - | - | - | - | - | - | - | - | L | V | K | S | H | A | Y | S | L | I | D | A | A | E | W | S | G | - | - | - | - | - | - | - | - | - | -172 |
| A2FEL7_TRIVA | - | - | - | - | - | - | - | - | - | - | - | - | - | - | - | - | - | - | - | - | - | - | - | - | - | - | - | - | - | K | K | N | K | D | G | I | V | Q | G | H | A | Y | N | I | L | D | V | V | E | F | H | G | V | - | - | - | - | - | - | - | - | -203 |
| A2D9A8_TRIVA | - | - | - | - | - | - | - | - | - | - | - | - | - | - | - | - | - | - | - | - | - | - | - | - | - | - | - | - | - | S | - | V | K | H | G | I | V | Q | G | H | A | Y | N | I | L | D | V | V | D | Y | R | G | I | - | - | - | - | - | - | - | - | -203 |
| A2FHB3_TRIVA | - | - | - | - | - | - | - | - | - | - | - | - | - | - | - | - | - | - | - | - | - | - | - | - | - | - | - | - | - | T | K | S | K | T | N | I | V | Q | G | H | A | Y | S | V | L | Q | A | V | E | V | K | G | F | - | - | - | - | - | - | - | - | -205 |
| A2DTE8_TRIVA | - | - | - | - | - | - | - | - | - | - | - | - | - | - | - | - | - | - | - | - | - | - | - | - | - | - | - | - | - | - | - | - | - | P | E | L | K | K | Q | S | S | Y | A | I | Y | N | Y | V | E | W | K | K | F | - | - | - | - | - | - | - | - | -194 |
| A2E4X2_TRIVA | - | - | - | - | - | - | - | - | - | - | - | - | - | - | - | - | - | - | - | - | - | - | - | - | - | - | - | - | - | - | A | V | K | L | G | L | I | E | D | H | T | Y | A | V | V | G | L | N | Q | V | G | G | Y | - | - | - | - | - | - | - | - | -198 |
| A2E1X1_TRIVA | - | - | - | - | - | - | - | - | - | - | - | - | - | - | - | - | - | - | - | - | - | - | - | - | - | - | - | - | - | - | P | E | K | H | Q | L | F | S | N | H | S | Y | A | L | L | S | I | E | V | V | N | G | S | - | - | - | - | - | - | - | - | -181 |
| TPR_PORGI | - | - | - | - | - | - | - | - | - | - | - | - | - | - | - | - | - | - | - | - | - | - | - | - | - | - | L | P | P | M | M | P | - | - | G | I | A | A | Y | H | A | Y | T | V | L | G | Y | T | V | S | N | G | - | A | Y | - | - | - | - | - | - | -231 |
| D5MF42_9BACT | - | - | - | - | - | - | - | - | - | - | - | - | - | - | - | - | - | - | - | - | - | - | - | - | - | - | A | P | V | D | Y | T | G | T | G | I | V | G | Y | H | A | Y | T | I | L | G | W | A | F | V | N | N | - | I | R | - | - | - | - | - | - | -189 |
| R9H254_BACT4 | - | - | - | - | - | - | - | - | - | - | - | - | - | - | - | - | - | - | - | - | - | - | - | - | - | - | - | - | Q | V | D | Q | N | W | Q | T | T | S | G | H | A | F | T | F | I | L | P | D | D | N | T | H | - | - | - | - | - | - | - | - | - | -180 |
| ZP_04847694 | - | - | - | - | - | - | - | - | - | - | - | - | - | - | - | - | - | - | - | - | - | - | - | - | - | - | - | - | Q | V | D | Q | N | W | Q | T | T | S | G | H | A | F | T | F | I | L | P | D | D | N | T | H | - | - | - | - | - | - | - | - | - | -180 |
| D7IBA4_9BACE | - | - | - | - | - | - | - | - | - | - | - | - | - | - | - | - | - | - | - | - | - | - | - | - | - | - | - | - | Q | V | D | Q | N | W | Q | T | T | S | G | H | A | F | T | F | I | L | P | D | D | N | T | H | - | - | - | - | - | - | - | - | - | -178 |
| ZP_09858577 | - | - | - | - | - | - | - | - | - | - | - | - | - | - | - | - | - | - | - | - | - | - | - | - | - | - | - | - | Q | V | D | Q | N | W | Q | T | T | S | G | H | A | F | T | F | I | L | P | D | D | N | T | H | - | - | - | - | - | - | - | - | - | -178 |
| E5C759_9BACE | - | - | - | - | - | - | - | - | - | - | - | - | - | - | - | - | - | - | - | - | - | - | - | - | - | - | - | - | Q | V | D | E | N | W | Q | T | T | S | G | H | A | F | T | F | I | L | P | D | D | D | S | H | - | - | - | - | - | - | - | - | - | -180 |
| A7LTS0_BACO1 | - | - | - | - | - | - | - | - | - | - | - | - | - | - | - | - | - | - | - | - | - | - | - | - | - | - | - | - | P | V | D | G | K | Y | K | T | V | S | A | H | A | Y | T | V | S | L | P | V | D | D | T | M | - | - | - | - | - | - | - | - | - | -178 |
| Q8YQK8_NOSS1 | - | - | - | - | - | - | - | - | - | - | - | - | - | - | - | - | - | - | - | - | - | - | - | - | - | - | - | - | D | S | L | - | A | I | G | G | H | A | Y | S | V | T | N | V | Y | T | N | S | S | G | E | K | - | - | - | - | - | - | - | - | - | -188 |
| Q3MBX8_ANAVT | - | - | - | - | - | - | - | - | - | - | - | - | - | - | - | - | - | - | - | - | - | - | - | - | - | - | - | - | T | S | Y | - | I | V | G | N | H | A | Y | S | V | T | N | V | Y | T | N | T | S | G | E | K | - | - | - | - | - | - | - | - | - | -193 |
| Q8YZT8_NOSS1 | - | - | - | - | - | - | - | - | - | - | - | - | - | - | - | - | - | - | - | - | - | - | - | - | - | - | - | - | T | S | F | - | I | L | G | S | H | A | Y | S | V | S | N | A | Y | I | S | A | S | G | E | Q | - | - | - | - | - | - | - | - | - | -180 |
| K9UIG2_9CHRO | - | - | - | - | - | - | - | - | - | - | - | - | - | - | - | - | - | - | - | - | - | - | - | - | - | - | - | - | N | G | L | N | L | I | S | G | H | A | Y | T | V | T | N | A | Y | I | S | N | T | G | E | Q | - | - | - | - | - | - | - | - | - | -191 |
| A4YRP8_BRASO | - | - | - | - | - | - | - | - | - | - | - | - | - | - | - | - | - | - | - | - | - | - | - | - | - | - | - | - | D | A | T | G | K | T | T | L | V | A | S | H | A | M | S | I | Y | G | Y | N | S | A | N | G | - | - | - | - | - | - | - | - | - | -208 |
| H0TAL7_9BRAD | - | - | - | - | - | - | - | - | - | - | - | - | - | - | - | - | - | - | - | - | - | - | - | - | - | - | - | - | D | A | T | G | K | T | T | L | V | A | S | H | A | M | S | I | Y | G | Y | N | S | A | N | G | - | - | - | - | - | - | - | - | - | -212 |
| H0SNL4_9BRAD | - | - | - | - | - | - | - | - | - | - | - | - | - | - | - | - | - | - | - | - | - | - | - | - | - | - | - | - | D | A | T | G | K | T | T | L | V | A | N | H | A | M | S | I | Y | G | Y | N | S | A | T | G | - | - | - | - | - | - | - | - | - | -214 |
| H0S4P6_9BRAD | - | - | - | - | - | - | - | - | - | - | - | - | - | - | - | - | - | - | - | - | - | - | - | - | - | - | - | - | D | S | T | G | K | T | T | L | V | A | N | H | A | M | S | I | Y | G | Y | N | S | S | T | G | - | - | - | - | - | - | - | - | - | -214 |
| A5EMK2_BRASB | - | - | - | - | - | - | - | - | - | - | - | - | - | - | - | - | - | - | - | - | - | - | - | - | - | - | - | - | D | S | T | G | K | T | T | L | V | A | S | H | A | M | S | I | Y | G | Y | N | S | A | N | G | - | - | - | - | - | - | - | - | - | -208 |
| M4ZBJ4_9BRAD | - | - | - | - | - | - | - | - | - | - | - | - | - | - | - | - | - | - | - | - | - | - | - | - | - | - | - | - | D | S | T | G | K | T | T | L | V | A | N | H | A | M | S | I | Y | G | Y | N | S | S | N | G | - | - | - | - | - | - | - | - | - | -214 |
| Q07SP7_RHOP5 | - | - | - | - | - | - | - | - | - | - | - | - | - | - | - | - | - | - | - | - | - | - | - | - | - | - | - | - | N | S | A | G | K | Q | T | L | V | K | N | H | A | M | S | I | Y | G | Y | N | S | S | T | G | - | - | - | - | - | - | - | - | - | -201 |
| B0JUQ7_MICAN | - | - | - | - | - | - | - | - | - | - | - | - | - | - | - | - | - | - | - | - | - | - | - | - | - | - | - | - | - | - | - | - | - | - | - | - | - | A | G | H | A | L | T | L | V | G | Y | N | P | T | T | Q | - | - | - | - | - | - | - | - | - | -182 |
| K9WJ87_9CYAN | - | - | - | - | - | - | - | - | - | - | - | - | - | - | - | - | - | - | - | - | - | - | - | - | - | - | - | - | - | - | - | - | - | - | - | - | - | G | G | H | A | Y | T | L | V | G | Y | N | A | T | T | Q | - | - | - | - | - | - | - | - | - | -176 |
| G6FUE0_9CYAN | - | - | - | - | - | - | - | - | - | - | - | - | - | - | - | - | - | - | - | - | - | - | - | - | - | - | - | S | T | - | V | V | D | A | N | I | I | A | S | H | A | Y | A | L | I | G | Y | N | S | A | T | Q | - | - | - | - | - | - | - | - | - | -184 |
| K9PHG5_9CYAN | - | - | - | - | - | - | - | - | - | - | - | - | - | - | - | - | - | - | - | - | - | - | - | - | - | - | - | A | D | G | V | T | D | S | R | I | V | P | N | H | V | Y | V | V | V | D | Y | N | S | S | T | Q | - | - | - | - | - | - | - | - | - | -187 |
| Q8YXX3_NOSS1 | - | - | - | - | - | - | - | - | - | - | - | - | - | - | - | - | - | - | - | - | - | - | - | - | - | - | - | S | S | T | Q | V | A | T | N | V | V | S | S | H | A | Y | V | L | V | S | Y | S | S | S | T | Q | - | - | - | - | - | - | - | - | - | -202 |
| K9TN70_9CYAN | - | - | - | - | - | - | - | - | - | - | - | - | - | - | - | - | - | - | - | - | - | - | - | - | - | - | - | G | T | L | I | G | S | D | K | L | V | G | G | H | A | Y | L | L | T | S | Y | D | S | V | N | K | - | - | - | - | - | - | - | - | - | -206 |
| Q05QW3_9SYNE | - | - | - | - | - | - | - | - | - | - | - | - | - | - | - | - | - | - | - | - | - | S | I | D | W | K | S | T | S | S | T | N | I | G | S | A | V | Q | G | H | V | Y | A | I | V | G | Y | N | A | A | T | Q | - | - | - | - | - | - | - | - | - | -229 |
| Q05QW2_9SYNE | - | - | - | - | - | - | - | - | - | - | - | - | - | - | - | - | - | - | - | - | - | - | - | - | W | G | - | - | - | - | - | - | - | - | - | - | - | - | G | H | A | R | T | I | T | G | Y | N | A | T | T | G | - | - | - | - | - | - | - | - | - | -197 |
| Q05QW4_9SYNE | - | - | - | - | - | - | - | - | - | - | - | - | - | - | - | - | - | - | - | - | - | N | I | S | S | G | - | - | - | - | - | - | - | - | - | - | V | Q | G | H | A | Y | S | I | V | G | Y | D | A | E | T | G | - | - | - | - | - | - | - | - | - | -208 |
| E0UGX6_CYAP2 | - | - | - | - | - | - | - | - | - | - | - | - | - | - | - | - | - | - | - | - | - | - | - | - | Y | G | - | - | - | - | - | - | - | - | - | V | V | N | S | H | A | Y | T | I | T | S | Y | D | S | T | N | Q | - | - | - | - | - | - | - | - | - | -188 |
| K7WKF4_9NOST | - | - | - | - | - | - | - | - | - | - | - | - | - | - | - | - | - | - | - | - | - | - | - | - | Y | G | - | - | - | - | - | - | - | - | - | V | V | D | G | H | A | Y | T | I | T | S | Y | N | A | T | N | Q | - | - | - | - | - | - | - | - | - | -187 |
| E0UGY5_CYAP2 | - | - | - | - | - | - | - | - | - | - | - | - | - | - | - | - | - | - | - | - | - | - | - | - | Y | G | - | - | - | - | - | - | - | - | - | V | I | N | D | H | A | Y | T | I | T | G | Y | D | A | L | T | G | - | - | - | - | - | - | - | - | - | -190 |
| A8LCE5_FRASN | - | - | - | - | - | - | - | - | - | - | - | - | - | - | - | - | - | - | - | - | - | - | - | - | - | - | S | D | L | P | P | G | C | G | L | V | P | N | - | H | A | Y | A | V | L | K | A | D | A | R | T | G | - | - | - | - | - | - | - | - | - | -184 |
| D3CT47_9ACTO | - | - | - | - | - | - | - | - | - | - | - | - | - | - | - | - | - | - | - | - | - | - | - | - | - | - | A | R | V | T | G | S | S | G | L | V | P | G | - | H | A | Y | A | I | L | A | A | D | P | P | T | G | - | - | - | - | - | - | - | - | - | -188 |
| Q2J4J6_FRASC | - | - | - | - | - | - | - | - | - | - | - | - | - | - | - | - | - | - | - | - | - | - | - | - | - | - | R | R | T | R | P | G | - | G | L | V | A | A | - | H | A | Y | T | L | L | A | V | D | V | A | R | R | - | - | - | - | - | - | - | - | - | -208 |
| Q0RAZ2_FRAAA | - | - | - | - | - | - | - | - | - | - | - | - | - | - | - | - | - | - | - | - | - | - | - | - | - | - | S | R | T | R | S | G | - | G | L | V | A | A | - | H | A | Y | P | A | L | A | V | D | A | A | R | G | - | - | - | - | - | - | - | - | - | -195 |
| E3J3F6_FRASU | - | - | - | - | - | - | - | - | - | - | - | - | - | - | - | - | - | - | - | - | - | - | - | - | - | - | E | A | P | R | V | D | S | G | L | V | R | R | - | H | A | Y | T | V | L | A | V | D | V | T | G | G | - | - | - | - | - | - | - | - | - | -191 |
| ZP_09168240 | - | - | - | - | - | - | - | - | - | - | - | - | - | - | - | - | - | - | - | - | - | - | - | - | - | - | A | A | D | G | P | S | G | G | L | V | E | R | - | H | A | Y | A | V | L | A | A | D | A | A | G | G | - | - | - | - | - | - | - | - | - | -202 |
| C7MDV9_BRAFD | - | - | - | - | - | - | - | - | - | - | - | - | - | - | - | - | - | - | - | - | - | - | - | - | - | - | P | R | V | P | T | S | A | G | P | L | P | R | N | H | V | F | A | V | V | G | A | D | A | R | S | G | - | - | - | - | - | - | - | - | - | -165 |
| ZP_10094989 | - | - | - | - | - | - | - | - | - | - | - | - | - | - | - | - | - | - | - | - | - | - | - | - | - | - | L | R | V | P | S | A | A | G | P | L | P | P | N | H | V | F | A | V | V | G | A | D | A | T | S | G | - | - | - | - | - | - | - | - | - | -165 |
| A4X268_SALTO | - | - | - | - | - | - | - | - | - | - | - | - | - | - | - | - | - | - | - | - | - | - | - | - | P | A | A | I | R | N | Q | L | P | H | G | L | V | A | G | H | A | Y | E | V | V | A | V | - | - | - | - | - | G | N | G | - | - | - | - | - | - | -222 |
| WP_043162213 | - | - | - | - | - | - | - | - | - | - | - | - | - | - | - | - | - | - | - | - | - | - | - | - | P | A | A | I | R | N | Q | L | P | Y | G | L | V | A | G | H | A | Y | E | V | V | A | V | - | - | - | - | - | G | N | G | - | - | - | - | - | - | -222 |
| Q0BRV8_GRABC | - | - | - | - | - | - | - | - | - | - | - | - | - | - | - | - | - | - | - | - | - | - | - | - | - | - | - | - | T | S | S | L | S | Y | G | L | V | G | S | H | A | Y | M | L | T | G | L | S | - | Y | I | N | G | E | A | - | - | - | - | - | - | -191 |
| A9B2P0_HERA2 | - | - | - | - | - | - | - | - | - | - | - | - | - | - | - | - | - | - | - | - | - | - | - | - | - | - | N | K | P | A | G | V | H | - | - | - | - | A | D | H | A | Y | V | I | K | S | I | D | P | T | N | K | - | - | - | - | - | - | - | - | - | -181 |
| A9B3U8_HERA2 | - | - | - | - | - | - | - | - | - | - | - | - | - | - | - | - | - | - | - | - | - | - | - | - | - | - | N | P | P | Q | F | V | S | - | - | - | - | E | D | H | A | Y | V | V | Q | H | V | D | L | N | N | K | - | - | - | - | - | - | - | - | - | -183 |
| A9B2A3_HERA2 | - | - | - | - | - | - | - | - | - | - | - | - | - | - | - | - | - | - | - | - | - | - | - | - | S | V | N | Q | P | A | F | I | N | K | Q | L | Q | P | K | H | A | Y | Y | V | S | H | I | D | Q | Q | R | N | - | - | - | - | - | - | - | - | - | -208 |
| D6ZC85_SEGRD | - | - | - | - | - | - | - | - | - | - | - | - | - | - | - | - | - | - | - | - | - | - | - | - | - | - | - | - | - | Q | T | F | Q | D | R | L | V | F | N | H | Q | Y | T | V | L | G | I | A | K | N | A | N | G | E | V | - | - | - | - | - | - | -230 |
| E5XTT9_9ACTO | - | - | - | - | - | - | - | - | - | - | - | - | - | - | - | - | - | - | - | - | - | - | - | - | - | - | - | - | - | R | L | S | Q | D | R | L | V | L | N | H | Q | Y | T | V | M | A | I | S | K | D | A | S | G | A | I | - | - | - | - | - | - | -230 |
| E5XTU0_9ACTO | - | - | - | - | - | - | - | - | - | - | - | - | - | - | - | - | - | - | - | - | - | - | - | - | - | - | - | G | D | R | E | F | Q | D | K | L | A | H | D | H | E | Y | T | V | E | G | I | A | K | D | A | N | G | N | V | - | - | - | - | - | - | -237 |
| D6B0I6_9ACTO | - | - | - | - | - | - | - | - | - | - | - | - | - | - | - | - | - | - | - | - | - | - | - | - | D | K | D | H | P | K | E | W | G | S | T | Y | H | T | N | H | A | Y | Y | V | R | G | F | T | D | D | G | K | - | - | - | - | - | - | - | - | - | -200 |
| D9X719_STRVR | - | - | - | - | - | - | - | - | - | - | - | - | - | - | - | - | - | - | - | - | - | - | - | - | D | K | N | H | P | A | E | W | G | N | A | Y | H | S | N | H | A | Y | Y | V | R | G | F | T | D | D | G | K | - | - | - | - | - | - | - | - | - | -200 |
| H6L010_SAPGL | - | - | - | - | - | - | - | - | - | - | - | - | - | - | - | - | - | - | - | - | - | - | - | - | - | - | - | T | K | L | K | D | G | Q | R | V | V | H | G | H | S | Y | A | V | M | G | V | - | - | - | N | G | - | G | K | - | - | - | - | - | - | -183 |
| K1ZP83_9BACT | - | - | - | - | - | - | - | - | - | - | - | - | - | - | - | - | - | - | - | - | - | - | - | - | - | - | - | G | T | L | V | T | A | Q | N | I | L | C | D | H | A | Y | S | V | L | G | V | K | E | R | N | G | - | T | K | - | - | - | - | - | - | -204 |
| ADGB_HUMAN | - | - | - | - | - | - | - | - | - | - | - | - | - | - | - | - | - | - | - | - | - | - | - | - | - | - | - | - | - | - | - | - | E | P | G | K | E | G | K | E | G | K | E | I | K | D | G | K | E | V | K | - | - | - | - | - | - | - | - | - | - | -238 |
| J3QMP3_MOUSE | - | - | - | - | - | - | - | - | - | - | - | - | - | - | - | - | - | - | - | - | - | - | - | - | - | - | - | - | - | - | - | - | E | D | G | K | D | G | K | D | G | K | D | G | K | D | G | K | D | G | K | - | - | - | - | - | - | - | - | - | - | -238 |
| H2XR46_CIOIN | - | - | - | - | - | - | - | - | - | - | - | - | - | - | - | - | - | - | - | - | - | - | - | - | - | - | - | - | - | - | - | - | T | N | S | A | S | H | K | E | K | G | S | G | K | D | S | K | S | R | E | - | - | - | - | - | - | - | - | - | - | -243 |
| CAN7_MOUSE | - | - | - | - | - | - | - | - | - | - | - | - | - | - | - | - | - | - | - | - | - | - | - | - | - | - | - | - | - | - | - | - | K | W | G | L | V | P | T | H | A | Y | A | V | L | D | I | R | E | F | K | - | - | - | - | - | - | - | - | - | - | -209 |
| CAN7_HUMAN | - | - | - | - | - | - | - | - | - | - | - | - | - | - | - | - | - | - | - | - | - | - | - | - | - | - | - | - | - | - | - | - | K | W | G | L | V | P | T | H | A | Y | A | V | L | D | I | R | E | F | K | - | - | - | - | - | - | - | - | - | - | -209 |
| E1BY84_CHICK | - | - | - | - | - | - | - | - | - | - | - | - | - | - | - | - | - | - | - | - | - | - | - | - | - | - | - | - | - | - | - | - | K | W | G | L | V | P | T | H | A | Y | A | V | L | D | I | R | E | H | K | - | - | - | - | - | - | - | - | - | - | -209 |
| H9GX91_DANRE | - | - | - | - | - | - | - | - | - | - | - | - | - | - | - | - | - | - | - | - | - | - | - | - | - | - | - | - | - | - | - | - | R | W | G | L | V | P | T | H | A | Y | A | V | L | D | I | R | Q | Y | K | - | - | - | - | - | - | - | - | - | - | -209 |
| F6SGI6_CIOIN | - | - | - | - | - | - | - | - | - | - | - | - | - | - | - | - | - | - | - | - | - | - | - | - | - | - | - | - | - | - | - | - | R | A | G | L | V | P | T | H | A | Y | A | V | L | D | V | Q | V | V | Y | - | - | - | - | - | - | - | - | - | - | -207 |
| W4Y195_STRPU | - | - | - | - | - | - | - | - | - | - | - | - | - | - | - | - | - | - | - | - | - | - | - | - | - | - | - | - | - | - | - | - | R | A | G | L | V | P | T | H | A | Y | A | M | L | D | I | R | E | I | Q | - | - | - | - | - | - | - | - | - | - | -183 |
| R7V270_CAPTE | - | - | - | - | - | - | - | - | - | - | - | - | - | - | - | - | - | - | - | - | - | - | - | - | - | - | - | - | - | - | - | - | R | A | G | L | V | S | T | H | A | Y | A | M | L | N | I | M | E | V | K | - | - | - | - | - | - | - | - | - | - | -208 |
| G4LXK3_SCHMA | - | - | - | - | - | - | - | - | - | - | - | - | - | - | - | - | - | - | - | - | - | - | - | - | - | - | - | - | - | - | - | - | R | S | G | L | V | P | T | H | A | Y | A | M | L | D | I | R | E | V | E | - | - | - | - | - | - | - | - | - | - | -209 |
| T2M2K0_HYDVU | - | - | - | - | - | - | - | - | - | - | - | - | - | - | - | - | - | - | - | - | - | - | - | - | - | - | - | - | - | - | - | - | R | A | G | L | V | P | T | H | A | Y | A | V | L | N | L | K | K | V | Q | - | - | - | - | - | - | - | - | - | - | -207 |
| B3RQK1_TRIAD | - | - | - | - | - | - | - | - | - | - | - | - | - | - | - | - | - | - | - | - | - | - | - | - | - | - | - | - | - | - | - | - | R | T | G | L | V | S | S | H | A | Y | A | L | L | D | A | K | Q | I | K | - | - | - | - | - | - | - | - | - | - | -208 |
| I1CS59_RHIO9 | - | - | - | - | - | - | - | - | - | - | - | - | - | - | - | - | - | - | - | - | - | - | - | - | - | - | - | - | - | - | - | - | Q | L | G | L | V | P | T | H | A | Y | A | V | I | D | I | K | M | T | P | D | - | - | - | - | - | - | - | - | - | -205 |
| I1GER3_AMPQE | - | - | - | - | - | - | - | - | - | - | - | - | - | - | - | - | - | - | - | - | - | - | - | - | - | - | - | - | - | - | - | - | R | T | G | L | V | P | T | H | A | Y | A | V | L | R | V | T | E | T | C | - | - | - | - | - | - | - | - | - | - | -205 |
| Q22143_CAEEL | - | - | - | - | - | - | - | - | - | - | - | - | - | - | - | - | - | - | - | - | - | - | - | - | - | - | - | - | - | - | - | - | R | S | G | L | V | E | T | H | A | Y | A | V | I | D | I | R | C | V | E | - | - | - | - | - | - | - | - | - | - | -208 |
| L1JZN7_GUITH | - | - | - | - | - | - | - | - | - | - | - | - | - | - | - | - | - | - | - | - | - | - | - | - | - | - | - | - | - | - | - | - | R | S | G | L | A | S | Q | H | A | Y | A | V | L | E | V | V | M | A | G | - | - | - | - | - | - | - | - | - | - | -211 |
| D0NT34_PHYIT | - | - | - | - | - | - | - | - | - | - | - | - | - | - | - | - | - | - | - | - | - | - | - | - | - | - | - | - | - | - | - | - | A | I | G | L | V | P | M | H | V | Y | A | V | L | N | V | Y | E | L | A | N | N | S | V | A | S | K | E | - | - | -227 |
| A0A064B913_ASPOZ | - | - | - | - | - | - | - | - | - | - | - | - | - | - | - | - | - | - | - | - | - | - | - | - | - | - | - | - | - | - | - | - | E | L | G | L | V | S | E | H | D | Y | A | I | L | D | M | K | E | S | K | - | - | - | - | - | - | - | - | - | - | -215 |
| A8N4X4_COPC7 | - | - | - | - | - | - | - | - | - | - | - | - | - | - | - | - | - | - | - | - | - | - | - | - | - | - | - | - | - | - | - | - | R | I | Q | L | L | P | S | H | S | Y | A | V | T | D | V | F | E | A | D | - | - | - | - | - | - | - | - | - | - | -231 |
| C0H5K1_PLAF7 | - | - | - | - | - | - | - | - | - | - | - | - | - | - | - | - | - | - | - | - | - | - | - | - | - | - | - | - | - | - | - | - | S | T | G | I | V | T | R | H | A | Y | S | I | L | N | I | E | N | Y | N | - | - | - | - | - | - | - | - | - | - | -378 |
| K1REZ8_CRAGI | - | - | - | - | - | - | - | - | - | - | - | - | - | - | - | - | - | - | - | - | - | - | - | - | - | - | - | - | - | - | - | - | V | Y | G | L | T | A | G | D | T | F | T | I | E | D | T | Y | Q | V | H | T | Q | R | G | K | - | - | - | - | - | -196 |
| F4NTM9_BATDJ | - | - | - | - | - | - | - | - | - | - | - | - | - | - | - | - | - | - | - | - | - | - | - | - | - | - | - | - | F | G | N | A | C | T | D | D | L | C | S | D | M | Y | A | V | T | K | V | F | Y | T | H | I | K | T | S | L | F | H | K | - | - | -224 |
| Q6F6K5_CAEEL | - | - | - | - | - | - | - | - | - | - | - | - | - | - | - | - | - | - | - | - | - | - | - | - | - | - | - | - | - | - | - | - | - | - | - | - | - | G | S | E | E | W | D | I | F | L | K | N | Q | I | S | P | N | H | G | - | - | - | - | - | - | -169 |
| A8HZK8_CHLRE | - | - | - | - | - | - | - | - | - | - | - | - | - | - | - | - | - | - | - | - | - | - | - | - | - | - | - | A | R | Q | R | F | R | P | D | V | I | P | E | S | E | E | G | A | T | T | S | E | A | L | A | Q | E | F | A | E | G | - | - | - | - | -187 |
| A8J6T6_CHLRE | - | - | - | - | - | - | - | - | - | - | - | - | - | - | - | - | - | - | - | - | - | - | - | - | - | - | - | A | V | D | T | E | A | T | G | L | A | R | A | R | S | Y | S | V | M | D | A | R | F | V | A | I | N | G | T | G | G | V | V | S | V | -247 |
| Z9JR51_9MICO | - | - | - | - | - | - | - | - | - | - | - | - | - | - | - | - | - | - | - | - | - | - | - | - | - | - | - | - | M | G | S | S | A | D | D | L | V | A | A | H | V | Y | Q | V | S | G | Y | D | E | S | T | G | - | - | - | - | - | - | - | - | - | -191 |
| F7ZZH9_CELGA | - | - | - | - | - | - | - | - | - | - | - | - | - | - | - | - | - | - | - | - | - | - | - | - | - | - | P | D | G | N | S | R | E | V | D | V | V | T | S | H | S | Y | V | I | T | R | I | D | P | D | G | S | - | - | - | - | - | - | - | - | - | -187 |
| Q7NDU2_GLOVI | - | - | - | - | - | - | - | - | - | - | - | - | - | - | - | - | - | - | - | - | - | - | - | - | - | - | - | E | K | Q | N | G | G | I | V | L | P | D | N | H | A | Y | A | V | V | G | Y | E | D | G | Q | - | - | - | - | - | - | - | - | - | - | -165 |
| I6AT95_9BACT | - | - | - | - | - | - | - | - | - | - | - | - | - | - | - | - | - | - | - | - | - | - | - | - | - | - | - | - | - | - | - | - | V | P | N | V | D | G | N | H | A | Y | A | V | I | G | F | D | P | K | T | D | - | - | - | - | - | - | - | - | - | -189 |
| C7MGJ8_BRAFD | - | - | - | - | - | - | - | - | - | - | - | - | - | - | - | - | - | - | - | - | - | - | - | - | - | - | - | - | - | - | - | - | - | - | A | Y | V | T | N | H | V | F | V | L | T | D | S | Y | T | A | D | G | - | - | - | - | - | - | - | - | - | -186 |
| D0WP73_9ACTO | - | - | - | - | - | - | - | - | - | - | - | - | - | - | - | - | - | - | - | - | - | - | - | - | - | - | - | - | - | - | - | - | - | - | - | N | F | P | D | D | G | K | A | E | V | G | V | T | L | P | N | G | E | - | - | - | - | - | - | - | - | -185 |
| B0C8L1_ACAM1 | - | - | - | - | - | - | - | - | - | - | - | - | - | - | - | - | - | - | - | - | - | - | - | - | - | - | - | - | - | - | - | - | - | - | - | - | V | T | D | K | D | D | K | T | T | T | V | P | G | V | N | - | - | - | - | - | - | - | - | - | - | -167 |
| B3E5A8_GEOLS | - | - | - | - | - | - | - | - | - | - | - | - | - | - | - | - | - | - | - | - | - | - | - | - | - | - | - | - | - | - | - | - | - | A | K | L | A | Y | D | H | V | Y | A | I | M | G | F | D | P | E | T | D | - | - | - | - | - | - | - | - | - | -180 |
| D4YQ94_9MICO | - | - | - | - | - | - | - | - | - | - | - | - | - | - | - | - | - | - | - | - | - | - | - | - | - | - | E | D | H | P | D | R | W | V | G | V | M | A | G | H | V | Y | V | V | C | E | V | L | R | C | D | E | D | G | R | T | D | D | D | - | - | -186 |
| PAPA_CARPA | - | - | - | - | - | - | - | - | - | - | - | - | - | - | - | - | - | - | - | - | - | - | - | - | - | - | - | - | - | - | - | - | - | G | I | F | V | G | P | C | G | N | K | V | D | H | A | V | A | A | V | G | Y | G | - | - | - | - | - | - | - | -167 |
| RIM13_YEAST | - | - | - | - | - | - | - | - | - | - | - | - | - | - | - | - | - | - | - | - | - | - | - | - | - | - | - | - | - | N | D | M | I | K | P | L | V | E | N | H | D | Y | S | I | I | D | I | T | Y | D | S | R | - | - | - | - | - | - | - | - | - | -202 |

| I |  |  |  |  |  |  |  |  |  |  |  |  |  |  |  |  |  |  |  |  |  |  |  |  |  |  |  |  |  |  |  |  |  |  |  |  |  |  |  |  |  |  |  |  |  |  |  |  | * |  |  |  |  |  |  |  |  |  |  |  |  |  |
| --- | --- | --- | --- | --- | --- | --- | --- | --- | --- | --- | --- | --- | --- | --- | --- | --- | --- | --- | --- | --- | --- | --- | --- | --- | --- | --- | --- | --- | --- | --- | --- | --- | --- | --- | --- | --- | --- | --- | --- | --- | --- | --- | --- | --- | --- | --- | --- | --- | --- | --- | --- | --- | --- | --- | --- | --- | --- | --- | --- | --- | --- | --- |
| Q9N4B1_CAEEL | - | Y | G | - | - | - | - | - | - | - | - | - | - | - | - | - | - | - | - | - | - | - | - | - | - | - | - | - | - | - | - | - | - | - | - | - | - | - | - | G | Q | V | P | L | L | R | I | R | N | - | - | P | W | G | N | - | - | - | - | - | - | -216 |
| Q8IAA8_CAEEL | - | Y | G | - | - | - | - | - | - | - | - | - | - | - | - | - | - | - | - | - | - | - | - | - | - | - | - | - | - | - | - | - | - | - | - | - | - | - | - | E | D | T | T | L | L | R | L | R | N | - | - | P | W | G | N | - | - | - | - | - | - | -216 |
| CAN_CAEEL | - | P | N | - | - | - | - | - | - | - | - | - | - | - | - | - | - | - | - | - | - | - | - | - | - | - | - | - | - | - | - | - | - | - | - | - | - | - | - | G | Q | T | C | I | L | R | I | R | N | - | - | P | W | G | N | - | - | - | - | - | - | -215 |
| Q9U2B2_CAEEL | - | E | W | - | - | - | - | - | - | - | - | - | - | - | - | - | - | - | - | - | - | - | - | - | - | - | - | - | - | - | - | - | - | - | - | - | - | - | - | E | K | A | P | L | I | R | I | R | S | - | - | P | W | G | - | - | - | - | - | - | - | -218 |
| Q9N4B2_CAEEL | - | D | K | - | - | - | - | - | - | - | - | - | - | - | - | - | - | - | - | - | - | - | - | - | - | - | - | - | - | - | - | - | - | - | - | - | - | - | - | E | K | V | A | L | L | R | I | R | N | - | - | P | W | G | - | - | - | - | - | - | - | -215 |
| C3Y6P6_BRAFL | - | R | S | - | - | - | - | - | - | - | - | - | - | - | - | - | - | - | - | - | - | - | - | - | - | - | - | - | - | - | - | - | - | - | - | - | - | - | - | S | R | T | Q | I | V | R | V | R | N | - | - | P | W | G | - | - | - | - | - | - | - | -232 |
| C3Y6N1_BRAFL | - | - | - | - | - | - | - | - | - | - | - | - | - | - | - | - | - | - | - | - | - | - | - | - | - | - | - | - | - | - | - | - | - | - | - | - | - | - | - | E | G | T | Q | L | V | R | I | R | N | - | - | P | W | G | - | - | - | - | - | - | - | -221 |
| CANA_DROME | - | R | Q | - | - | - | - | - | - | - | - | - | - | - | - | - | - | - | - | - | - | - | - | - | - | - | - | - | - | - | - | - | - | - | - | - | - | - | - | G | K | I | P | M | I | R | M | R | N | - | - | P | W | G | N | - | - | - | - | - | - | -219 |
| CANB_DROME | - | R | Q | - | - | - | - | - | - | - | - | - | - | - | - | - | - | - | - | - | - | - | - | - | - | - | - | - | - | - | - | - | - | - | - | - | - | - | - | G | K | L | P | M | I | R | M | R | N | - | - | P | W | G | N | - | - | - | - | - | - | -219 |
| X1Y3H71_ANODA | - | R | S | - | - | - | - | - | - | - | - | - | - | - | - | - | - | - | - | - | - | - | - | - | - | - | - | - | - | - | - | - | - | - | - | - | - | - | - | G | K | I | P | L | I | R | L | R | N | - | - | P | W | G | N | - | - | - | - | - | - | -220 |
| Q5IW12_GECLA | - | V | S | - | - | - | - | - | - | - | - | - | - | - | - | - | - | - | - | - | - | - | - | - | - | - | - | - | - | - | - | - | - | - | - | - | - | - | - | G | K | I | P | L | V | R | I | R | N | - | - | P | W | G | N | - | - | - | - | - | - | -219 |
| K1Q056_CRAGI | - | M | S | - | - | - | - | - | - | - | - | - | - | - | - | - | - | - | - | - | - | - | - | - | - | - | - | - | - | - | - | - | - | - | - | - | - | - | - | G | K | I | P | M | V | R | I | R | N | - | - | P | W | G | N | - | - | - | - | - | - | -219 |
| G4V6C0_SCHMA | - | Q | S | - | - | - | - | - | - | - | - | - | - | - | - | - | - | - | - | - | - | - | - | - | - | - | - | - | - | - | - | - | - | - | - | - | - | - | - | - | - | I | R | L | I | R | L | R | N | - | - | P | W | G | N | - | - | - | - | - | - | -222 |
| R7ULX9_CAPTE | - | V | S | - | - | - | - | - | - | - | - | - | - | - | - | - | - | - | - | - | - | - | - | - | - | - | - | - | - | - | - | - | - | - | - | - | - | - | - | G | R | I | P | M | V | R | V | R | N | - | - | P | W | G | D | - | - | - | - | - | - | -220 |
| W4Z5Q7_STRPU | - | V | R | - | - | - | - | - | - | - | - | - | - | - | - | - | - | - | - | - | - | - | - | - | - | - | - | - | - | - | - | - | - | - | - | - | - | - | - | - | - | - | - | L | I | R | I | R | N | - | - | P | W | G | - | - | - | - | - | - | - | -212 |
| R7VIB6_CAPTE | - | V | Q | - | - | - | - | - | - | - | - | - | - | - | - | - | - | - | - | - | - | - | - | - | - | - | - | - | - | - | - | - | - | - | - | - | - | - | - | - | - | - | - | L | V | R | I | R | N | - | - | P | W | G | N | - | - | - | - | - | - | -219 |
| T2MJ45_HYDVU | - | E | H | - | - | - | - | - | - | - | - | - | - | - | - | - | - | - | - | - | - | - | - | - | - | - | - | - | - | - | - | - | - | - | - | - | - | - | - | - | - | - | K | L | I | R | V | R | N | - | - | P | W | G | N | - | - | - | - | - | - | -211 |
| R7UAB6_CAPTE | - | T | E | - | - | - | - | - | - | - | - | - | - | - | - | - | - | - | - | - | - | - | - | - | - | - | - | - | - | - | - | - | - | - | - | - | - | - | - | G | R | L | P | M | I | R | V | R | N | - | - | P | W | G | D | - | - | - | - | - | - | -219 |
| C3ZKN8_BRAFL | - | I | N | - | - | - | - | - | - | - | - | - | - | - | - | - | - | - | - | - | - | - | - | - | - | - | - | - | - | - | - | - | - | - | - | - | - | - | - | - | - | - | - | L | I | R | V | R | N | - | - | P | W | G | - | - | - | - | - | - | - | -215 |
| B3RJS5_TRIAD | - | E | E | - | - | - | - | - | - | - | - | - | - | - | - | - | - | - | - | - | - | - | - | - | - | - | - | - | - | - | - | - | - | - | - | - | - | - | - | D | - | - | - | L | I | R | I | R | N | - | - | P | W | G | N | - | - | - | - | - | - | -216 |
| H2YVF0_CIOSA | - | V | K | - | - | - | - | - | - | - | - | - | - | - | - | - | - | - | - | - | - | - | - | - | - | - | - | - | - | - | - | - | - | - | - | - | - | - | - | - | - | - | - | L | V | R | V | R | N | - | - | P | W | G | - | - | - | - | - | - | - | -212 |
| G4VAG2_SCHMA | - | Q | W | - | - | - | - | - | - | - | - | - | - | - | - | - | - | - | - | - | - | - | - | - | - | - | - | - | - | - | - | - | - | - | - | - | - | - | - | - | - | - | - | L | M | R | L | R | N | - | - | P | W | G | D | - | - | - | - | - | - | -218 |
| Smp_157500 | - | V | Y | - | - | - | - | - | - | - | - | - | - | - | - | - | - | - | - | - | - | - | - | - | - | - | - | - | - | - | - | - | - | - | - | - | - | - | - | - | - | - | - | L | V | R | C | R | N | - | - | P | W | G | E | - | - | - | - | - | - | -218 |
| Q7SYA9_DANRE | - | - | - | - | - | - | - | - | - | - | - | - | - | - | - | - | - | - | - | - | - | - | - | - | - | - | - | - | - | - | - | - | - | - | - | - | - | - | - | - | - | E | R | L | I | R | I | R | N | - | - | P | W | G | - | - | - | - | - | - | - | -215 |
| A5PMP1_DANRE | - | - | - | - | - | - | - | - | - | - | - | - | - | - | - | - | - | - | - | - | - | - | - | - | - | - | - | - | - | - | - | - | - | - | - | - | - | - | - | - | - | E | R | L | I | R | I | R | N | - | - | P | W | G | - | - | - | - | - | - | - | -215 |
| CANX_CHICK | - | - | - | - | - | - | - | - | - | - | - | - | - | - | - | - | - | - | - | - | - | - | - | - | - | - | - | - | - | - | - | - | - | - | - | - | - | - | - | - | - | E | Q | L | I | R | I | R | N | - | - | P | W | G | - | - | - | - | - | - | - | -215 |
| CAN1_HUMAN | - | - | - | - | - | - | - | - | - | - | - | - | - | - | - | - | - | - | - | - | - | - | - | - | - | - | - | - | - | - | - | - | - | - | - | - | - | - | - | - | - | V | S | L | I | R | M | R | N | - | - | P | W | G | - | - | - | - | - | - | - | -215 |
| CAN1_MOUSE | - | - | - | - | - | - | - | - | - | - | - | - | - | - | - | - | - | - | - | - | - | - | - | - | - | - | - | - | - | - | - | - | - | - | - | - | - | - | - | - | - | V | N | L | I | R | M | R | N | - | - | P | W | G | - | - | - | - | - | - | - | -215 |
| O42133_CHICK | - | - | - | - | - | - | - | - | - | - | - | - | - | - | - | - | - | - | - | - | - | - | - | - | - | - | - | - | - | - | - | - | - | - | - | - | - | - | - | - | - | L | G | L | I | R | M | R | N | - | - | P | W | G | - | - | - | - | - | - | - | -215 |
| Q7ZUR1_DANRE | - | - | - | - | - | - | - | - | - | - | - | - | - | - | - | - | - | - | - | - | - | - | - | - | - | - | - | - | - | - | - | - | - | - | - | - | - | - | - | - | - | T | K | L | V | R | I | R | N | - | - | P | W | G | - | - | - | - | - | - | - | -215 |
| E9QFN0_DANRE | - | - | - | - | - | - | - | - | - | - | - | - | - | - | - | - | - | - | - | - | - | - | - | - | - | - | - | - | - | - | - | - | - | - | - | - | - | - | - | - | - | T | K | L | L | R | I | R | N | - | - | P | W | G | - | - | - | - | - | - | - | -215 |
| CAN2_HUMAN | - | - | - | - | - | - | - | - | - | - | - | - | - | - | - | - | - | - | - | - | - | - | - | - | - | - | - | - | - | - | - | - | - | - | - | - | - | - | - | - | - | Q | K | L | I | R | I | R | N | - | - | P | W | G | - | - | - | - | - | - | - | -215 |
| CAN2_MOUSE | - | - | - | - | - | - | - | - | - | - | - | - | - | - | - | - | - | - | - | - | - | - | - | - | - | - | - | - | - | - | - | - | - | - | - | - | - | - | - | - | - | Q | K | L | I | R | I | R | N | - | - | P | W | G | - | - | - | - | - | - | - | -215 |
| CAN2_CHICK | - | - | - | - | - | - | - | - | - | - | - | - | - | - | - | - | - | - | - | - | - | - | - | - | - | - | - | - | - | - | - | - | - | - | - | - | - | - | - | - | - | Q | K | L | I | R | I | R | N | - | - | P | W | G | - | - | - | - | - | - | - | -215 |
| Q6DFZ8_DANRE | - | - | - | - | - | - | - | - | - | - | - | - | - | - | - | - | - | - | - | - | - | - | - | - | - | - | - | - | - | - | - | - | - | - | - | - | - | - | - | - | - | T | K | L | I | R | I | R | N | - | - | P | W | G | - | - | - | - | - | - | - | -215 |
| Q504F5_DANRE | - | - | - | - | - | - | - | - | - | - | - | - | - | - | - | - | - | - | - | - | - | - | - | - | - | - | - | - | - | - | - | - | - | - | - | - | - | - | - | - | - | E | K | L | V | R | M | R | N | - | - | P | W | G | - | - | - | - | - | - | - | -215 |
| Q5BLH5_DANRE | - | - | - | - | - | - | - | - | - | - | - | - | - | - | - | - | - | - | - | - | - | - | - | - | - | - | - | - | - | - | - | - | - | - | - | - | - | - | - | - | - | E | K | L | V | R | V | R | N | - | - | P | W | G | - | - | - | - | - | - | - | -215 |
| Q4VBH3_DANRE | - | - | - | - | - | - | - | - | - | - | - | - | - | - | - | - | - | - | - | - | - | - | - | - | - | - | - | - | - | - | - | - | - | - | - | - | - | - | - | - | - | V | Q | L | V | R | I | R | N | - | - | P | W | G | - | - | - | - | - | - | - | -215 |
| CAN8_RAT | - | - | - | - | - | - | - | - | - | - | - | - | - | - | - | - | - | - | - | - | - | - | - | - | - | - | - | - | - | - | - | - | - | - | - | - | - | - | - | - | - | E | K | L | I | R | L | R | N | - | - | P | W | G | - | - | - | - | - | - | - | -215 |
| CAN8_MOUSE | - | - | - | - | - | - | - | - | - | - | - | - | - | - | - | - | - | - | - | - | - | - | - | - | - | - | - | - | - | - | - | - | - | - | - | - | - | - | - | - | - | E | K | L | I | R | L | R | N | - | - | P | W | G | - | - | - | - | - | - | - | -215 |
| CAN8_HUMAN | - | - | - | - | - | - | - | - | - | - | - | - | - | - | - | - | - | - | - | - | - | - | - | - | - | - | - | - | - | - | - | - | - | - | - | - | - | - | - | - | - | E | K | L | I | R | L | R | N | - | - | P | W | G | - | - | - | - | - | - | - | -215 |
| XP_426117 | - | - | - | - | - | - | - | - | - | - | - | - | - | - | - | - | - | - | - | - | - | - | - | - | - | - | - | - | - | - | - | - | - | - | - | - | - | - | - | - | - | E | K | L | V | R | L | R | N | - | - | P | W | G | - | - | - | - | - | - | - | -215 |
| CAN9_MOUSE | - | - | - | - | - | - | - | - | - | - | - | - | - | - | - | - | - | - | - | - | - | - | - | - | - | - | - | - | - | - | - | - | - | - | - | - | - | - | - | - | - | I | K | L | I | R | V | R | N | - | - | P | W | G | - | - | - | - | - | - | - | -217 |
| CAN9_HUMAN | - | - | - | - | - | - | - | - | - | - | - | - | - | - | - | - | - | - | - | - | - | - | - | - | - | - | - | - | - | - | - | - | - | - | - | - | - | - | - | - | - | I | E | L | I | R | I | R | N | - | - | P | W | G | - | - | - | - | - | - | - | -215 |
| E1C6M3_CHICK | - | - | - | - | - | - | - | - | - | - | - | - | - | - | - | - | - | - | - | - | - | - | - | - | - | - | - | - | - | - | - | - | - | - | - | - | - | - | - | - | - | V | Q | L | I | R | I | R | N | - | - | P | W | G | - | - | - | - | - | - | - | -215 |
| Q6DG44_DANRE | - | - | - | - | - | - | - | - | - | - | - | - | - | - | - | - | - | - | - | - | - | - | - | - | - | - | - | - | - | - | - | - | - | - | - | - | - | - | - | - | - | V | Q | L | I | R | V | R | N | - | - | P | W | G | - | - | - | - | - | - | - | -215 |
| CAN3_HUMAN | - | - | - | - | - | - | - | - | - | - | - | - | - | - | - | - | - | - | - | - | - | - | - | - | - | - | - | - | - | - | - | - | - | - | - | - | - | - | - | - | - | V | K | L | V | R | L | R | N | - | - | P | W | G | - | - | - | - | - | - | - | -263 |
| CAN3_MOUSE | - | - | - | - | - | - | - | - | - | - | - | - | - | - | - | - | - | - | - | - | - | - | - | - | - | - | - | - | - | - | - | - | - | - | - | - | - | - | - | - | - | V | K | L | V | R | L | R | N | - | - | P | W | G | - | - | - | - | - | - | - | -263 |
| CAN3_CHICK | - | - | - | - | - | - | - | - | - | - | - | - | - | - | - | - | - | - | - | - | - | - | - | - | - | - | - | - | - | - | - | - | - | - | - | - | - | - | - | - | - | M | R | L | V | R | L | R | N | - | - | P | W | G | - | - | - | - | - | - | - | -262 |
| Q66HU4_DANRE | - | - | - | - | - | - | - | - | - | - | - | - | - | - | - | - | - | - | - | - | - | - | - | - | - | - | - | - | - | - | - | - | - | - | - | - | - | - | - | - | R | V | R | L | V | R | L | R | N | - | - | P | W | G | - | - | - | - | - | - | - | -218 |
| F1R632_DANRE | - | - | - | - | - | - | - | - | - | - | - | - | - | - | - | - | - | - | - | - | - | - | - | - | - | - | - | - | - | - | - | - | - | - | - | - | - | - | - | - | M | I | R | L | V | R | V | R | D | - | - | P | W | G | - | - | - | - | - | - | - | -218 |
| CAN11_HUMAN | - | - | - | - | - | - | - | - | - | - | - | - | - | - | - | - | - | - | - | - | - | - | - | - | - | - | - | - | - | - | - | - | - | - | - | - | - | - | - | - | - | E | T | L | I | R | V | R | N | - | - | P | W | G | - | - | - | - | - | - | - | -215 |
| CAN11_MOUSE | - | - | - | - | - | - | - | - | - | - | - | - | - | - | - | - | - | - | - | - | - | - | - | - | - | - | - | - | - | - | - | - | - | - | - | - | - | - | - | - | - | E | T | L | I | R | I | Q | N | - | - | P | W | G | - | - | - | - | - | - | - | -215 |
| CAN12_MOUSE | - | - | - | - | - | - | - | - | - | - | - | - | - | - | - | - | - | - | - | - | - | - | - | - | - | - | - | - | - | - | - | - | - | - | - | - | - | - | - | - | - | V | R | L | L | R | L | R | N | - | - | P | W | G | - | - | - | - | - | - | - | -212 |
| CAN12_HUMAN | - | - | - | - | - | - | - | - | - | - | - | - | - | - | - | - | - | - | - | - | - | - | - | - | - | - | - | - | - | - | - | - | - | - | - | - | - | - | - | - | - | V | R | L | L | R | L | R | N | - | - | P | W | G | - | - | - | - | - | - | - | -212 |
| ENSSARP00000008135 | - | - | - | - | - | - | - | - | - | - | - | - | - | - | - | - | - | - | - | - | - | - | - | - | - | - | - | - | - | - | - | - | - | - | - | - | - | - | - | - | - | L | Q | L | L | R | L | R | N | - | - | P | W | G | - | - | - | - | - | - | - | -212 |
| ENSSARP00000010553 | - | - | - | - | - | - | - | - | - | - | - | - | - | - | - | - | - | - | - | - | - | - | - | - | - | - | - | - | - | - | - | - | - | - | - | - | - | - | - | - | - | L | R | F | I | Q | L | K | N | - | - | P | W | S | - | - | - | - | - | - | - | -219 |
| A7E2G3_DANRE | - | - | - | - | - | - | - | - | - | - | - | - | - | - | - | - | - | - | - | - | - | - | - | - | - | - | - | - | - | - | - | - | - | - | - | - | - | - | - | - | - | V | F | L | L | R | L | R | N | - | - | P | W | G | - | - | - | - | - | - | - | -216 |
| Q6NW69_DANRE | - | - | - | - | - | - | - | - | - | - | - | - | - | - | - | - | - | - | - | - | - | - | - | - | - | - | - | - | - | - | - | - | - | - | - | - | - | - | - | - | - | V | Q | L | V | R | I | R | N | - | - | P | W | G | - | - | - | - | - | - | - | -212 |
| CAN13_HUMAN | - | - | - | - | - | - | - | - | - | - | - | - | - | - | - | - | - | - | - | - | - | - | - | - | - | - | - | - | - | - | - | - | - | - | - | - | - | - | - | - | - | E | E | I | I | S | L | W | N | - | - | P | W | G | W | G | - | - | - | - | - | -217 |
| CAN13_MOUSE | - | - | - | - | - | - | - | - | - | - | - | - | - | - | - | - | - | - | - | - | - | - | - | - | - | - | - | - | - | - | - | - | - | - | - | - | - | - | - | - | - | E | D | I | I | R | L | W | N | - | - | P | W | G | - | - | - | - | - | - | - | -217 |
| E1BRR5_CHICK | - | - | - | - | - | - | - | - | - | - | - | - | - | - | - | - | - | - | - | - | - | - | - | - | - | - | - | - | - | - | - | - | - | - | - | - | - | - | - | - | - | E | H | I | I | R | V | W | N | - | - | P | W | G | - | - | - | - | - | - | - | -212 |
| E7F0H3_DANRE | - | - | - | - | - | - | - | - | - | - | - | - | - | - | - | - | - | - | - | - | - | - | - | - | - | - | - | - | - | - | - | - | - | - | - | - | - | - | - | - | - | V | Q | L | V | R | L | L | N | - | - | P | W | G | - | - | - | - | - | - | - | -215 |
| Q1RLS9_DANRE | - | - | - | - | - | - | - | - | - | - | - | - | - | - | - | - | - | - | - | - | - | - | - | - | - | - | - | - | - | - | - | - | - | - | - | - | - | - | - | - | - | V | K | L | V | R | I | F | N | - | - | P | W | G | - | - | - | - | - | - | - | -216 |
| F1Q9Z1_DANRE | - | - | - | - | - | - | - | - | - | - | - | - | - | - | - | - | - | - | - | - | - | - | - | - | - | - | - | - | - | - | - | - | - | - | - | - | - | - | - | - | - | V | Q | L | V | R | L | F | N | - | - | P | W | G | - | - | - | - | - | - | - | -212 |
| A4QNV6_DANRE | - | - | - | - | - | - | - | - | - | - | - | - | - | - | - | - | - | - | - | - | - | - | - | - | - | - | - | - | - | - | - | - | - | - | - | - | - | - | - | - | - | V | R | L | V | R | V | L | N | - | - | P | W | G | - | - | - | - | - | - | - | -215 |
| D6W573_HUMAN | - | - | - | - | - | - | - | - | - | - | - | - | - | - | - | - | - | - | - | - | - | - | - | - | - | - | - | - | - | - | - | - | - | - | - | - | - | - | - | - | - | E | Y | L | V | K | L | R | N | - | - | P | W | G | - | - | - | - | - | - | - | -211 |
| R7UBC6_CAPTE | - | - | - | - | - | - | - | - | - | - | - | - | - | - | - | - | - | - | - | - | - | - | - | - | - | - | - | - | - | - | - | - | - | - | - | - | - | - | - | E | S | I | S | L | I | R | L | R | D | - | - | P | F | G | A | - | - | - | - | - | - | -229 |
| K1RCZ2_CRAGI | - | - | - | - | - | - | - | - | - | - | - | - | - | - | - | - | - | - | - | - | - | - | - | - | - | - | - | - | - | - | - | - | - | - | - | - | - | - | - | T | P | I | T | L | I | R | V | R | N | - | - | P | W | G | N | - | - | - | - | - | - | -231 |
| Q5IW13_GECLA | - | - | - | - | - | - | - | - | - | - | - | - | - | - | - | - | - | - | - | - | - | - | - | - | - | - | - | - | - | - | - | - | - | - | - | - | - | - | - | G | E | A | H | L | V | R | L | H | N | - | - | P | W | G | N | - | - | - | - | - | - | -222 |
| R7UUX7_CAPTE | - | - | - | - | - | - | - | - | - | - | - | - | - | - | - | - | - | - | - | - | - | - | - | - | - | - | - | - | - | - | - | - | - | - | - | - | - | - | - | - | V | I | Q | L | I | R | L | R | N | - | - | P | W | G | - | - | - | - | - | - | - | -216 |
| K1Q505_CRAGI | - | - | - | - | - | - | - | - | - | - | - | - | - | - | - | - | - | - | - | - | - | - | - | - | - | - | - | - | - | - | - | - | - | - | - | - | - | - | - | - | N | V | R | L | C | R | L | R | N | - | - | P | W | G | - | - | - | - | - | - | - | -222 |
| R7UD02_CAPTE | - | - | - | - | - | - | - | - | - | - | - | - | - | - | - | - | - | - | - | - | - | - | - | - | - | - | - | - | - | - | - | - | - | - | - | - | - | - | - | - | K | V | Q | L | V | R | L | R | N | - | - | P | W | G | - | - | - | - | - | - | - | -218 |
| CAN6_HUMAN | - | S | - | - | - | - | - | - | - | - | - | - | - | - | - | - | - | - | - | - | - | - | - | - | - | - | - | - | - | - | - | - | - | - | - | - | - | - | A | E | K | V | Y | M | V | R | L | R | N | - | - | P | L | G | - | - | - | - | - | - | - | -237 |
| CAN6_MOUSE | - | S | - | - | - | - | - | - | - | - | - | - | - | - | - | - | - | - | - | - | - | - | - | - | - | - | - | - | - | - | - | - | - | - | - | - | - | - | T | E | K | L | Y | M | V | R | L | R | N | - | - | P | L | G | - | - | - | - | - | - | - | -237 |
| E1C312_CHICK | - | K | - | - | - | - | - | - | - | - | - | - | - | - | - | - | - | - | - | - | - | - | - | - | - | - | - | - | - | - | - | - | - | - | - | - | - | - | A | E | K | L | F | M | V | R | L | R | N | - | - | P | W | G | - | - | - | - | - | - | - | -237 |
| E7FDN3_DANRE | - | N | - | - | - | - | - | - | - | - | - | - | - | - | - | - | - | - | - | - | - | - | - | - | - | - | - | - | - | - | - | - | - | - | - | - | - | - | A | S | R | L | Y | M | V | R | I | R | N | - | - | P | W | G | - | - | - | - | - | - | - | -238 |
| CAN5_HUMAN | - | K | - | - | - | - | - | - | - | - | - | - | - | - | - | - | - | - | - | - | - | - | - | - | - | - | - | - | - | - | - | - | - | - | - | - | - | - | S | E | K | L | D | M | I | R | L | R | N | - | - | P | W | G | - | - | - | - | - | - | - | -233 |
| CAN5_MOUSE | - | K | - | - | - | - | - | - | - | - | - | - | - | - | - | - | - | - | - | - | - | - | - | - | - | - | - | - | - | - | - | - | - | - | - | - | - | - | S | E | K | L | D | M | I | R | L | R | N | - | - | P | W | G | - | - | - | - | - | - | - | -237 |
| E1C292_CHICK | - | K | - | - | - | - | - | - | - | - | - | - | - | - | - | - | - | - | - | - | - | - | - | - | - | - | - | - | - | - | - | - | - | - | - | - | - | - | S | E | K | L | D | M | I | R | M | R | N | - | - | P | W | G | - | - | - | - | - | - | - | -237 |
| A0JMG3_DANRE | - | K | - | - | - | - | - | - | - | - | - | - | - | - | - | - | - | - | - | - | - | - | - | - | - | - | - | - | - | - | - | - | - | - | - | - | - | - | S | E | K | L | S | M | I | R | M | R | N | - | - | P | W | G | - | - | - | - | - | - | - | -237 |
| E7F7F1_DANRE | - | K | - | - | - | - | - | - | - | - | - | - | - | - | - | - | - | - | - | - | - | - | - | - | - | - | - | - | - | - | - | - | - | - | - | - | - | - | S | E | K | L | H | M | I | R | M | R | N | - | - | P | W | G | - | - | - | - | - | - | - | -237 |
| XP_006824412 | - | K | G | - | - | - | - | - | - | - | - | - | - | - | - | - | - | - | - | - | - | - | - | - | - | - | - | - | - | - | - | - | - | - | - | - | - | - | I | E | K | I | A | M | I | R | L | R | N | - | - | P | W | G | - | - | - | - | - | - | - | -231 |
| W4YJN2_STRPU | - | K | - | - | - | - | - | - | - | - | - | - | - | - | - | - | - | - | - | - | - | - | - | - | - | - | - | - | - | - | - | - | - | - | - | - | - | - | K | E | K | E | Y | L | V | R | L | R | N | - | - | P | W | G | - | - | - | - | - | - | - | -235 |
| C3YJZ3_BRAFL | - | L | G | - | - | - | - | - | - | - | - | - | - | - | - | - | - | - | - | - | - | - | - | - | - | - | - | - | - | - | - | - | - | - | - | - | - | N | Q | E | K | I | Y | M | I | R | M | R | N | - | - | P | W | G | - | - | - | - | - | - | - | -240 |
| T2MIT4_HYDVU | - | N | - | - | - | - | - | - | - | - | - | - | - | - | - | - | - | - | - | - | - | - | - | - | - | - | - | - | - | - | - | - | - | - | - | - | - | - | T | E | T | L | Y | L | V | K | L | R | N | - | - | P | W | G | - | - | - | - | - | - | - | -237 |
| XP_002154722 | - | N | - | - | - | - | - | - | - | - | - | - | - | - | - | - | - | - | - | - | - | - | - | - | - | - | - | - | - | - | - | - | - | - | - | - | - | - | A | N | R | L | F | M | V | K | L | R | N | - | - | P | W | G | - | - | - | - | - | - | - | -201 |
| R7VLA7_CAPTE | - | N | - | - | - | - | - | - | - | - | - | - | - | - | - | - | - | - | - | - | - | - | - | - | - | - | - | - | - | - | - | - | - | - | - | - | - | - | K | E | K | I | A | M | I | R | L | R | N | - | - | P | W | G | - | - | - | - | - | - | - | -238 |
| K1R1C6_CRAGI | - | K | - | - | - | - | - | - | - | - | - | - | - | - | - | - | - | - | - | - | - | - | - | - | - | - | - | - | - | - | - | - | - | - | - | - | - | - | T | E | K | I | A | M | V | R | L | R | N | - | - | P | W | G | - | - | - | - | - | - | - | -238 |
| B3RIE5_TRIAD | - | R | - | - | - | - | - | - | - | - | - | - | - | - | - | - | - | - | - | - | - | - | - | - | - | - | - | - | - | - | - | - | - | - | - | - | - | - | S | E | K | V | P | M | V | R | M | R | N | - | - | P | W | G | - | - | - | - | - | - | - | -235 |
| K1PW75_CRAGI | - | N | - | - | - | - | - | - | - | - | - | - | - | - | - | - | - | - | - | - | - | - | - | - | - | - | - | - | - | - | - | - | - | - | - | - | - | - | K | E | K | I | L | M | I | R | C | R | N | - | - | P | W | G | - | - | - | - | - | - | - | -233 |
| K1RSB8_CRAGI | - | S | - | - | - | - | - | - | - | - | - | - | - | - | - | - | - | - | - | - | - | - | - | - | - | - | - | - | - | - | - | - | - | - | - | - | - | - | R | E | K | I | H | M | I | R | C | R | N | - | - | P | W | G | - | - | - | - | - | - | - | -231 |
| K1RWI3_CRAGI | - | K | - | - | - | - | - | - | - | - | - | - | - | - | - | - | - | - | - | - | - | - | - | - | - | - | - | - | - | - | - | - | - | - | - | - | - | - | R | E | H | I | E | M | V | R | C | R | N | - | - | P | W | G | - | - | - | - | - | - | - | -234 |
| R7VGC2_CAPTE | - | - | - | - | R | L | G | - | - | - | - | - | - | - | - | - | - | - | - | - | - | - | - | - | - | - | - | - | - | - | - | - | - | - | - | - | - | G | D | - | K | V | Q | L | V | R | M | S | N | - | - | P | W | G | - | - | - | - | - | - | - | -235 |
| K1QU26_CRAGI | - | - | - | - | A | V | G | - | - | - | - | - | - | - | - | - | - | - | - | - | - | - | - | - | - | - | - | - | - | - | - | - | - | - | - | - | - | N | N | - | T | L | Q | M | V | R | L | A | N | - | - | P | W | G | - | - | - | - | - | - | - | -236 |
| CAN5_CAEEL | - | T | S | F | I | M | G | - | - | - | - | - | - | - | - | - | - | - | - | - | - | - | - | - | - | - | - | - | - | - | - | - | - | - | - | - | - | S | K | R | K | Q | N | L | I | R | L | Q | N | - | - | P | W | G | - | - | - | - | - | - | - | -241 |
| I1FGV4_AMPQE | - | - | - | - | - | - | - | - | - | - | - | - | - | - | - | - | - | - | - | - | - | - | - | - | - | - | - | - | - | - | - | - | - | - | - | - | - | - | G | K | V | I | K | L | L | R | I | R | N | - | - | P | W | G | - | - | - | - | - | - | - | -215 |
| K1PHX3_CRAGI | - | - | - | - | - | - | - | - | - | - | - | - | - | - | - | - | - | - | - | - | - | - | - | - | - | - | - | - | - | - | - | - | - | - | - | - | - | - | N | G | M | I | I | L | I | R | L | R | N | - | - | P | W | G | - | - | - | - | - | - | - | -215 |
| B3RNL8_TRIAD | - | - | - | - | - | - | - | - | - | - | - | - | - | - | - | - | - | - | - | - | - | - | - | - | - | - | - | - | - | - | - | - | - | - | - | - | - | - | D | E | E | V | P | L | V | R | I | R | N | - | - | P | W | G | - | - | - | - | - | - | - | -216 |
| X1Y3H7_ANODA | - | Q | E | - | - | - | - | - | - | - | - | - | - | - | - | - | - | - | - | - | - | - | - | - | - | - | - | - | - | - | - | - | - | - | - | - | - | - | P | R | E | I | R | L | I | C | L | R | N | - | - | P | W | G | - | - | - | - | - | - | - | -228 |
| R7TWH9_CAPTE | - | - | - | - | - | - | - | - | - | - | - | - | - | - | - | - | - | - | - | - | - | - | - | - | - | - | - | - | - | - | - | - | - | - | - | - | - | - | - | - | - | - | - | L | L | R | I | R | N | - | - | P | W | G | N | - | - | - | - | - | - | -219 |
| K1R5E8_CRAGI | - | - | - | - | - | - | - | - | - | - | - | - | - | - | - | - | - | - | - | - | - | - | - | - | - | - | - | - | - | - | - | - | - | - | - | - | - | - | - | - | - | - | - | L | V | R | I | R | N | - | - | P | W | G | D | - | - | - | - | - | - | -219 |
| XP_006811804 | - | - | - | - | - | - | - | - | - | - | - | - | - | - | - | - | - | - | - | - | - | - | - | - | - | - | - | - | - | - | - | - | - | - | - | - | - | - | - | - | - | - | - | L | L | R | V | R | N | - | - | P | W | G | N | - | - | - | - | - | - | -218 |
| C3YB23_BRAFL | - | - | - | - | - | - | - | - | - | - | - | - | - | - | - | - | - | - | - | - | - | - | - | - | - | - | - | - | - | - | - | - | - | - | - | - | - | - | - | - | - | - | - | L | V | R | I | R | N | - | - | P | W | G | D | - | - | - | - | - | - | -225 |
| H2ZIF1_CIOSA | - | - | - | - | - | - | - | - | - | - | - | - | - | - | - | - | - | - | - | - | - | - | - | - | - | - | - | - | - | - | - | - | - | - | - | - | - | - | - | - | - | - | - | L | V | K | L | R | N | - | - | P | W | G | G | - | - | - | - | - | - | -215 |
| W4YV93_STRPU | - | - | - | - | - | - | - | - | - | - | - | - | - | - | - | - | - | - | - | - | - | - | - | - | - | - | - | - | - | - | - | - | - | - | - | - | - | - | - | - | - | - | - | L | V | K | V | R | N | - | - | P | W | A | N | - | - | - | - | - | - | -217 |
| CAN10_HUMAN | - | - | - | - | - | - | - | - | - | - | - | - | - | - | - | - | - | - | - | - | - | - | - | - | - | - | - | - | - | - | - | - | - | - | - | - | - | - | - | - | - | I | L | L | L | R | I | Q | N | - | - | P | W | G | R | R | - | - | - | - | - | -226 |
| CAN10_MOUSE | - | - | - | - | - | - | - | - | - | - | - | - | - | - | - | - | - | - | - | - | - | - | - | - | - | - | - | - | - | - | - | - | - | - | - | - | - | - | - | - | - | I | L | L | L | R | I | H | N | - | - | P | W | G | R | R | - | - | - | - | - | -226 |
| XP_004943443 | - | - | - | - | - | - | - | - | - | - | - | - | - | - | - | - | - | - | - | - | - | - | - | - | - | - | - | - | - | - | - | - | - | - | - | - | - | - | - | - | - | I | I | L | L | R | I | R | N | - | - | P | W | G | R | R | - | - | - | - | - | -226 |
| F8W4K1_DANRE | - | - | - | - | - | - | - | - | - | - | - | - | - | - | - | - | - | - | - | - | - | - | - | - | - | - | - | - | - | - | - | - | - | - | - | - | - | - | - | - | - | V | Q | L | I | R | I | R | N | - | - | P | W | G | R | R | - | - | - | - | - | -229 |
| CANC_DROME | - | - | - | - | - | - | - | - | - | - | - | - | - | - | - | - | - | - | - | - | - | - | - | - | - | - | - | - | - | - | - | - | - | - | - | - | - | - | - | - | - | V | Q | L | V | C | L | K | D | - | - | T | F | S | S | K | P | F | G | E | - | -212 |
| C3ZKN9_BRAFL | - | - | - | - | - | - | - | - | - | - | - | - | - | - | - | - | - | - | - | - | - | - | - | - | - | - | - | - | - | - | - | - | - | - | - | - | - | - | - | - | - | L | A | K | V | R | V | R | T | - | - | P | R | G | D | D | - | - | - | - | - | -208 |
| G4V623_SCHMA | - | - | - | - | - | - | - | - | - | - | - | - | - | - | - | - | - | - | - | - | - | - | - | - | - | - | - | - | - | - | - | - | - | - | - | - | - | - | - | - | - | - | R | L | V | R | L | R | N | - | - | L | W | P | S | V | - | - | - | - | - | -237 |
| R7VK39_CAPTE | - | - | - | - | - | - | - | - | - | - | - | - | - | - | - | - | - | - | - | - | - | - | - | - | - | - | - | - | - | - | - | - | - | - | - | - | - | - | - | - | - | T | K | L | L | R | L | K | N | - | - | T | R | S | - | - | - | - | - | - | - | -216 |
| G4V693_SCHMA | - | D | M | F | R | R | A | G | - | - | - | - | - | - | - | - | - | - | - | - | - | - | - | - | - | - | - | - | - | - | - | - | - | - | - | - | D | C | P | I | Q | A | R | L | M | R | L | R | C | - | - | P | L | S | V | I | D | S | G | S | G | -269 |
| R7UPU6_CAPTE | - | - | - | - | - | - | - | - | - | - | - | - | - | - | - | - | - | - | - | - | - | - | - | - | - | - | - | - | - | - | - | - | - | - | - | - | - | - | - | - | - | K | A | M | I | R | V | R | N | - | - | P | W | G | V | G | - | - | - | - | - | -218 |
| R7V817_CAPTE | - | - | - | - | - | - | - | - | - | - | - | - | - | - | - | - | - | - | - | - | - | - | - | - | - | - | - | - | - | - | - | - | - | - | - | - | - | - | - | - | - | K | A | M | I | R | I | R | N | - | - | P | W | G | S | R | - | - | - | - | - | -217 |
| R7UNL8_CAPTE | - | - | - | - | - | - | - | - | - | - | - | - | - | - | - | - | - | - | - | - | - | - | - | - | - | - | - | - | - | - | - | - | - | - | - | - | - | - | - | - | - | K | M | L | L | R | L | R | N | - | - | P | W | G | T | - | - | - | - | - | - | -210 |
| K1PJ80_CRAGI | - | - | - | - | - | - | - | - | - | - | - | - | - | - | - | - | - | - | - | - | - | - | - | - | - | - | - | - | - | - | - | - | - | - | - | - | - | - | - | - | - | V | R | L | F | R | V | R | N | - | - | P | W | G | N | - | - | - | - | - | - | -213 |
| K1PCX8_CRAGI | - | - | - | - | - | - | - | - | - | - | - | - | - | - | - | - | - | - | - | - | - | - | - | - | - | - | - | - | - | - | - | - | - | - | - | - | - | - | - | - | - | V | R | L | F | R | L | R | N | - | - | P | W | G | K | - | - | - | - | - | - | -213 |
| CAND_DROME | - | - | - | - | - | - | - | - | - | - | - | - | - | - | - | - | - | - | - | - | - | - | - | - | - | - | - | - | - | - | - | - | - | - | - | - | - | - | - | - | G | H | R | L | L | K | L | R | N | - | - | P | W | G | H | - | - | - | - | - | - | -226 |
| XP_002735765 | - | - | - | - | - | - | - | - | - | - | - | - | - | - | - | - | - | - | - | - | - | - | - | - | - | - | - | - | - | - | - | - | - | - | - | - | - | - | - | - | G | H | R | L | I | R | L | R | N | - | - | P | W | G | T | - | - | - | - | - | - | -229 |
| CAN15_HUMAN | - | - | - | - | - | - | - | - | - | - | - | - | - | - | - | - | - | - | - | - | - | - | - | - | - | - | - | - | - | - | - | - | - | - | - | - | - | - | - | - | G | T | R | L | L | R | L | R | N | - | - | P | W | G | R | - | - | - | - | - | - | -227 |
| CAN15_MOUSE | - | - | - | - | - | - | - | - | - | - | - | - | - | - | - | - | - | - | - | - | - | - | - | - | - | - | - | - | - | - | - | - | - | - | - | - | - | - | - | - | G | S | R | L | L | R | L | R | N | - | - | P | W | G | R | - | - | - | - | - | - | -227 |
| E1BTA8_CHICK | - | - | - | - | - | - | - | - | - | - | - | - | - | - | - | - | - | - | - | - | - | - | - | - | - | - | - | - | - | - | - | - | - | - | - | - | - | - | - | - | G | F | R | L | L | R | L | R | N | - | - | P | W | G | R | - | - | - | - | - | - | -227 |
| E7FAX5_DANRE | - | - | - | - | - | - | - | - | - | - | - | - | - | - | - | - | - | - | - | - | - | - | - | - | - | - | - | - | - | - | - | - | - | - | - | - | - | - | - | - | G | Y | R | L | L | R | L | R | N | - | - | P | W | G | R | - | - | - | - | - | - | -227 |
| C3XWK8_BRAFL | - | - | - | - | - | - | - | - | - | - | - | - | - | - | - | - | - | - | - | - | - | - | - | - | - | - | - | - | - | - | - | - | - | - | - | - | - | - | - | - | G | S | R | L | L | R | M | R | N | - | - | P | W | G | R | - | - | - | - | - | - | -227 |
| W4YMU4_STRPU | - | - | - | - | - | - | - | - | - | - | - | - | - | - | - | - | - | - | - | - | - | - | - | - | - | - | - | - | - | - | - | - | - | - | - | - | - | - | - | - | G | K | R | L | I | Q | L | R | N | - | - | P | W | G | H | - | - | - | - | - | - | -341 |
| K1PV74_CRAGI | - | - | - | - | - | - | - | - | - | - | - | - | - | - | - | - | - | - | - | - | - | - | - | - | - | - | - | - | - | - | - | - | - | - | - | - | - | - | - | - | G | N | R | L | L | R | L | R | N | - | - | P | W | G | R | - | - | - | - | - | - | -224 |
| R7TJF2_CAPTE | - | - | - | - | - | - | - | - | - | - | - | - | - | - | - | - | - | - | - | - | - | - | - | - | - | - | - | - | - | - | - | - | - | - | - | - | - | - | T | L | F | P | R | L | V | R | L | R | N | - | - | P | W | G | R | - | - | - | - | - | - | -229 |
| T2MI17_HYDVU | - | - | - | - | - | - | - | - | - | - | - | - | - | - | - | - | - | - | - | - | - | - | - | - | - | - | - | - | - | - | - | - | - | - | - | - | - | - | - | - | D | N | R | L | I | R | L | R | N | - | - | P | W | G | R | - | - | - | - | - | - | -225 |
| Q1RL55_CIOIN | - | - | - | - | - | - | - | - | - | - | - | - | - | - | - | - | - | - | - | - | - | - | - | - | - | - | - | - | - | - | - | - | - | - | - | - | - | - | K | F | I | F | R | L | V | Q | L | R | N | - | - | P | W | G | H | - | - | - | - | - | - | -215 |
| I1EA30_AMPQE | - | - | - | - | - | - | - | - | - | - | - | - | - | - | - | - | - | - | - | - | - | - | - | - | - | - | - | - | - | - | - | - | - | - | - | - | - | - | - | - | T | E | S | V | V | V | L | R | D | - | - | P | W | G | H | - | - | - | - | - | - | -222 |
| G5EEZ6_CAEEL | - | - | - | - | - | - | - | - | - | - | - | - | - | - | - | - | - | - | - | - | - | - | - | - | - | - | - | - | - | - | - | - | - | - | - | - | - | - | - | - | G | Y | R | L | L | R | I | R | N | - | - | P | W | G | Q | - | - | - | - | - | - | -223 |
| O44903_CAEEL | - | - | - | - | - | - | - | - | - | - | - | - | - | - | - | - | - | - | - | - | - | - | - | - | - | - | - | - | - | - | - | - | - | - | - | - | - | - | - | - | G | H | R | L | L | R | I | R | N | - | - | P | W | G | Q | - | - | - | - | - | - | -223 |
| O02260_CAEEL | - | - | - | - | - | - | - | - | - | - | - | - | - | - | - | - | - | - | - | - | - | - | - | - | - | - | - | - | - | - | - | - | - | - | - | - | - | - | - | - | G | H | K | L | L | L | L | R | N | - | - | P | S | G | G | - | - | - | - | - | - | -208 |
| O02259_CAEEL | - | - | - | - | - | - | - | - | - | - | - | - | - | - | - | - | - | - | - | - | - | - | - | - | - | - | - | - | - | - | - | - | - | - | - | - | - | - | - | - | G | H | K | L | L | L | L | R | N | - | - | P | S | G | G | - | - | - | - | - | - | -199 |
| C1MTG3_MICPC | - | D | G | F | V | E | R | E | - | V | E | I | V | D | P | A | P | A | L | V | S | A | S | A | S | K | Y | A | S | S | A | A | A | A | D | A | S | S | P | P | P | L | R | L | V | R | V | R | N | - | - | P | W | G | R | - | - | - | - | - | - | -307 |
| C1E493_MICSR | - | T | S | F | V | A | K | E | R | V | E | D | V | D | - | - | - | - | - | - | - | - | - | - | - | - | - | - | - | - | - | - | A | A | A | G | G | G | G | P | P | L | R | L | L | R | I | R | N | - | - | P | W | G | R | - | - | - | - | - | - | -240 |
| Q012Z6_OSTTA | - | T | E | V | Y | A | R | G | - | K | D | A | L | D | - | - | - | - | - | - | - | - | - | - | - | - | - | - | - | - | - | - | - | V | E | V | E | N | - | - | - | L | R | L | L | K | I | R | N | - | - | P | W | G | K | - | - | - | - | - | - | -228 |
| L1IGB6_GUITH | - | - | - | - | - | - | - | - | - | - | - | - | - | - | - | - | - | - | - | - | - | - | - | - | - | - | - | - | - | - | - | - | - | - | - | - | - | - | - | - | M | V | R | L | V | R | I | R | N | - | - | P | H | G | V | - | - | - | - | - | - | -216 |
| B7FWX6_PHATC | - | T | D | Y | F | S | S | P | S | K | K | H | R | K | L | T | G | - | - | - | - | - | - | - | - | - | - | - | - | - | - | - | - | P | N | Y | D | T | C | A | P | I | R | L | V | R | I | R | N | - | - | P | W | G | K | - | - | - | - | - | - | -242 |
| N9TPR9_ENTHI | - | - | - | - | - | - | - | - | - | - | - | - | - | - | - | - | - | - | - | - | - | - | - | - | - | - | - | - | - | - | - | - | - | - | - | - | - | - | - | - | - | I | K | L | L | K | I | R | N | - | - | P | W | G | C | - | - | - | - | - | - | -204 |
| G0QS43_ICHMG | - | - | - | - | - | - | - | - | - | - | - | - | - | - | - | - | - | - | - | - | - | - | - | - | - | - | - | - | - | - | - | - | - | - | - | - | - | - | - | - | D | V | K | L | L | K | L | R | N | - | - | P | W | G | D | - | - | - | - | - | - | -216 |
| D8LB20_ECTSI | - | - | - | - | - | - | - | - | - | - | - | - | - | - | - | - | - | - | - | - | - | - | - | - | - | - | - | - | - | - | - | - | - | - | - | - | - | - | - | - | - | E | Q | L | I | Q | I | R | N | - | - | P | W | G | K | K | - | - | - | - | - | -251 |
| Q9U0T9_LEIMA | - | - | - | - | - | - | - | - | - | - | - | - | - | - | - | - | - | - | - | - | - | - | - | - | - | - | - | - | - | - | - | - | - | - | - | - | - | - | - | G | Q | I | R | L | I | C | L | R | N | - | - | P | W | V | H | G | - | - | - | - | - | -243 |
| Q4CXJ8_TRYCC | - | - | - | - | - | - | - | - | - | - | - | - | - | - | - | - | - | - | - | - | - | - | - | - | - | - | - | - | - | - | - | - | - | - | - | - | - | - | - | G | D | L | Q | L | L | Q | I | R | N | - | - | V | W | G | S | C | - | - | - | - | - | -245 |
| F4I0A4_ARATH | - | - | - | - | - | - | - | - | - | - | - | - | - | - | - | - | - | - | - | - | - | - | - | - | - | - | - | - | - | - | - | - | - | - | - | - | - | - | - | - | G | H | R | L | V | Q | I | R | N | - | - | P | W | A | N | E | - | - | - | - | - | -220 |
| Q8RUQ1_MAIZE | - | - | - | - | - | - | - | - | - | - | - | - | - | - | - | - | - | - | - | - | - | - | - | - | - | - | - | - | - | - | - | - | - | - | - | - | - | - | - | - | G | H | K | L | I | Q | I | R | N | - | - | P | W | A | N | E | - | - | - | - | - | -220 |
| D7FQ17_ECTSI | - | - | - | - | - | - | - | - | - | - | - | - | - | - | - | - | - | - | - | - | - | - | - | - | - | - | - | - | - | - | - | - | - | - | - | - | - | - | - | - | G | E | Q | L | I | K | L | R | N | - | - | P | P | G | D | H | - | - | - | - | - | -237 |
| D0MZ44_PHYIT | - | - | - | - | - | - | - | - | - | - | - | - | - | - | - | - | - | - | - | - | - | - | - | - | - | - | - | - | - | - | - | - | - | - | - | - | - | - | - | - | G | A | Q | L | L | Q | L | R | N | - | - | P | P | G | D | H | - | - | - | - | - | -238 |
| L1JBM0_GUITH | - | - | - | - | - | - | - | - | - | - | - | - | - | - | - | - | - | - | - | - | - | - | - | - | - | - | - | - | - | - | - | - | - | - | - | - | - | - | - | - | G | D | G | L | L | L | L | R | N | - | - | P | W | G | K | - | - | - | - | - | - | -187 |
| L1J739_GUITH | - | - | - | - | - | - | - | - | - | - | - | - | - | - | - | - | - | - | - | - | - | - | - | - | - | - | - | - | - | - | - | - | - | - | - | - | - | - | - | - | D | L | R | F | L | Q | V | R | N | - | - | P | W | G | R | - | - | - | - | - | - | -224 |
| L1J0U9_GUITH | - | - | - | - | - | - | - | - | - | - | - | - | - | - | - | - | - | - | - | - | - | - | - | - | - | - | - | - | - | - | - | - | - | - | - | - | - | - | - | - | G | M | R | F | M | K | L | K | P | N | V | Q | T | N | S | - | - | - | - | - | - | -218 |
| L1J1Q4_GUITH | - | - | - | - | - | - | - | - | - | - | - | - | - | - | - | - | - | - | - | - | - | - | - | - | - | - | - | - | - | - | - | - | - | - | - | - | - | - | - | - | G | F | R | L | V | K | V | R | N | P | W | G | T | N | S | - | - | - | - | - | - | -238 |
| D0ND88_PHYIT | - | - | - | - | - | - | - | - | - | - | - | - | - | - | - | - | - | - | - | - | - | - | - | - | - | - | - | - | - | - | - | - | - | - | - | A | S | R | E | E | V | L | R | F | V | K | L | K | T | - | - | V | W | G | R | - | - | - | - | - | - | -277 |
| D2VVS6_NAEGR | - | - | - | - | - | - | - | - | - | - | - | - | - | - | - | - | - | - | - | - | - | - | - | - | - | - | - | - | - | - | - | - | - | - | - | - | - | - | S | Q | R | V | R | L | I | K | M | K | N | - | - | P | W | A | N | G | - | - | - | - | - | -223 |
| D2V3V2_NAEGR | - | - | - | - | - | - | - | - | - | - | - | - | - | - | - | - | - | - | - | - | - | - | - | - | - | - | - | - | - | - | - | - | - | - | - | - | - | - | G | K | R | T | R | L | V | K | L | K | N | - | - | P | W | G | Y | G | - | - | - | - | - | -223 |
| Q5B5J7_EMENI | - | - | - | - | - | - | - | - | - | - | - | - | - | - | - | - | - | - | - | - | - | - | - | - | - | - | - | - | - | - | - | - | - | - | - | - | - | - | - | - | - | - | - | L | V | K | L | R | N | - | - | P | W | G | K | - | - | - | - | - | - | -242 |
| E4ZY10_LEPMJ | - | - | - | - | - | - | - | - | - | - | - | - | - | - | - | - | - | - | - | - | - | - | - | - | - | - | - | - | - | - | - | - | - | - | - | - | - | - | - | - | - | - | - | L | L | K | L | R | N | - | - | P | W | G | R | - | - | - | - | - | - | -238 |
| A8PIE5_COPC7 | - | - | - | - | - | - | - | - | - | - | - | - | - | - | - | - | - | - | - | - | - | - | - | - | - | - | - | - | - | - | - | - | - | - | - | - | - | - | - | - | - | - | - | F | V | I | V | R | N | - | - | P | W | G | T | - | - | - | - | - | - | -242 |
| A8NBA6_COPC7 | - | - | - | - | - | - | - | - | - | - | - | - | - | - | - | - | - | - | - | - | - | - | - | - | - | - | - | - | - | - | - | - | - | - | - | - | - | - | - | - | - | - | - | F | L | V | V | R | N | - | - | P | W | G | D | - | - | - | - | - | - | -239 |
| A8NBA1_COPC7 | - | - | - | - | - | - | - | - | - | - | - | - | - | - | - | - | - | - | - | - | - | - | - | - | - | - | - | - | - | - | - | - | - | - | - | - | - | - | - | - | - | - | - | F | L | V | L | R | N | - | - | P | W | G | Q | - | - | - | - | - | - | -233 |
| A8NBA2_COPC7 | - | - | - | - | - | - | - | - | - | - | - | - | - | - | - | - | - | - | - | - | - | - | - | - | - | - | - | - | - | - | - | - | - | - | - | - | - | - | - | - | - | - | - | F | V | L | V | R | N | - | - | P | W | G | D | - | - | - | - | - | - | -233 |
| A8NAE6_COPC7 | - | - | - | - | - | - | - | - | - | - | - | - | - | - | - | - | - | - | - | - | - | - | - | - | - | - | - | - | - | - | - | - | - | - | - | - | - | - | - | - | - | - | - | F | V | V | L | R | D | - | - | P | W | G | E | - | - | - | - | - | - | -261 |
| A8NAI9_COPC7 | - | - | - | - | - | - | - | - | - | - | - | - | - | - | - | - | - | - | - | - | - | - | - | - | - | - | - | - | - | - | - | - | - | - | - | - | - | - | - | - | - | - | - | F | V | V | I | R | N | - | - | P | W | G | K | T | - | - | - | - | - | -238 |
| G4TER7_PIRID | - | - | - | - | - | - | - | - | - | - | - | - | - | - | - | - | - | - | - | - | - | - | - | - | - | - | - | - | - | - | - | - | - | - | - | - | - | - | - | - | - | - | - | F | V | V | I | R | N | - | - | P | W | G | T | - | - | - | - | - | - | -239 |
| G4TER6_PIRID | - | - | - | - | - | - | - | - | - | - | - | - | - | - | - | - | - | - | - | - | - | - | - | - | - | - | - | - | - | - | - | - | - | - | - | - | - | - | - | - | - | - | - | F | V | L | V | Q | N | - | - | P | W | G | S | - | - | - | - | - | - | -228 |
| G4TNA1_PIRID | - | - | - | - | - | - | - | - | - | - | - | - | - | - | - | - | - | - | - | - | - | - | - | - | - | - | - | - | - | - | - | - | - | - | - | - | - | - | - | - | - | - | - | F | V | V | I | R | N | - | - | P | W | G | N | - | - | - | - | - | - | -238 |
| D0MWB9_PHYIT | - | - | - | - | - | - | - | - | - | - | - | - | - | - | - | - | - | - | - | - | - | - | - | - | - | - | - | - | - | - | - | - | - | - | - | - | - | - | - | - | - | - | - | L | V | K | L | R | N | - | - | P | W | G | M | G | - | - | - | - | - | -237 |
| L1I9H5_GUITH | - | - | - | - | - | - | - | - | - | - | - | - | - | - | - | - | - | - | - | - | - | - | - | - | - | - | - | - | - | - | - | - | - | - | - | - | - | - | - | - | - | - | R | F | V | Q | I | R | N | - | - | P | W | G | - | - | - | - | - | - | - | -226 |
| Q7N497_PHOLL | - | - | - | - | - | - | - | - | - | - | - | - | - | - | - | - | - | - | - | - | - | - | - | - | - | - | - | - | - | - | - | - | - | - | - | - | - | - | - | - | - | I | K | L | V | R | L | H | N | - | - | P | W | N | - | - | - | - | - | - | - | -186 |
| ZP_11152369 | - | - | - | - | - | - | - | - | - | - | - | - | - | - | - | - | - | - | - | - | - | - | - | - | - | - | - | - | - | - | - | - | - | - | - | - | - | - | - | - | - | F | K | L | V | R | L | H | N | - | - | P | W | N | - | - | - | - | - | - | - | -183 |
| A2FEL7_TRIVA | - | - | - | - | - | - | - | - | - | - | - | - | - | - | - | - | - | - | - | - | - | - | - | - | - | - | - | - | - | - | - | - | - | - | - | - | - | - | - | - | - | - | Q | L | L | K | L | R | N | - | - | P | W | G | - | - | - | - | - | - | - | -213 |
| A2D9A8_TRIVA | - | - | - | - | - | - | - | - | - | - | - | - | - | - | - | - | - | - | - | - | - | - | - | - | - | - | - | - | - | - | - | - | - | - | - | - | - | - | - | - | - | - | K | L | L | K | L | R | N | - | - | P | W | G | - | - | - | - | - | - | - | -213 |
| A2FHB3_TRIVA | - | - | - | - | - | - | - | - | - | - | - | - | - | - | - | - | - | - | - | - | - | - | - | - | - | - | - | - | - | - | - | - | - | - | - | - | - | - | - | - | - | - | Q | L | L | Q | L | R | N | - | - | P | W | G | - | - | - | - | - | - | - | -215 |
| A2DTE8_TRIVA | - | - | - | - | - | - | - | - | - | - | - | - | - | - | - | - | - | - | - | - | - | - | - | - | - | - | - | - | - | - | - | - | - | - | - | - | - | - | - | - | - | - | Q | M | L | R | L | R | N | - | - | P | Y | N | - | - | - | - | - | - | - | -204 |
| A2E4X2_TRIVA | - | - | - | - | - | - | - | - | - | - | - | - | - | - | - | - | - | - | - | - | - | - | - | - | - | - | - | - | - | - | - | - | - | - | - | - | - | - | - | - | - | - | K | L | I | H | L | R | N | - | - | P | W | G | R | - | - | - | - | - | - | -209 |
| A2E1X1_TRIVA | - | - | - | - | - | - | - | - | - | - | - | - | - | - | - | - | - | - | - | - | - | - | - | - | - | - | - | - | - | - | - | - | - | - | - | - | - | - | - | - | - | - | K | Y | I | H | L | Q | D | - | - | P | H | G | - | - | - | - | - | - | - | -191 |
| TPR_PORGI | - | - | - | - | - | - | - | - | - | - | - | - | - | - | - | - | - | - | - | - | - | - | - | - | - | - | - | - | - | - | - | - | - | - | - | - | - | - | - | - | - | - | - | Y | L | I | I | R | N | - | - | P | W | G | V | T | E | P | T | G | D | -247 |
| D5MF42_9BACT | - | - | - | - | - | - | - | - | - | - | - | - | - | - | - | - | - | - | - | - | - | - | - | - | - | - | - | - | - | - | - | - | - | - | - | - | - | - | - | - | - | - | - | Y | I | V | L | R | N | - | - | P | W | G | H | N | G | P | - | - | - | -202 |
| R9H254_BACT4 | - | - | - | - | - | - | - | - | - | - | - | - | - | - | - | - | - | - | - | - | - | - | - | - | - | - | - | - | - | - | - | - | - | - | - | - | - | - | - | - | - | - | - | L | F | K | M | R | N | - | - | P | W | G | - | - | - | - | - | - | - | -189 |
| ZP_04847694 | - | - | - | - | - | - | - | - | - | - | - | - | - | - | - | - | - | - | - | - | - | - | - | - | - | - | - | - | - | - | - | - | - | - | - | - | - | - | - | - | - | - | - | L | F | K | M | R | N | - | - | P | W | G | - | - | - | - | - | - | - | -189 |
| D7IBA4_9BACE | - | - | - | - | - | - | - | - | - | - | - | - | - | - | - | - | - | - | - | - | - | - | - | - | - | - | - | - | - | - | - | - | - | - | - | - | - | - | - | - | - | - | - | L | F | K | M | R | N | - | - | P | W | G | - | - | - | - | - | - | - | -187 |
| ZP_09858577 | - | - | - | - | - | - | - | - | - | - | - | - | - | - | - | - | - | - | - | - | - | - | - | - | - | - | - | - | - | - | - | - | - | - | - | - | - | - | - | - | - | - | - | L | F | K | M | R | N | - | - | P | W | G | - | - | - | - | - | - | - | -187 |
| E5C759_9BACE | - | - | - | - | - | - | - | - | - | - | - | - | - | - | - | - | - | - | - | - | - | - | - | - | - | - | - | - | - | - | - | - | - | - | - | - | - | - | - | - | - | - | - | L | F | K | M | R | N | - | - | P | W | G | - | - | - | - | - | - | - | -189 |
| A7LTS0_BACO1 | - | - | - | - | - | - | - | - | - | - | - | - | - | - | - | - | - | - | - | - | - | - | - | - | - | - | - | - | - | - | - | - | - | - | - | - | - | - | - | - | - | - | - | L | F | V | M | R | N | - | - | P | W | G | - | - | - | - | - | - | - | -187 |
| Q8YQK8_NOSS1 | - | - | - | - | - | - | - | - | - | - | - | - | - | - | - | - | - | - | - | - | - | - | - | - | - | - | - | - | - | - | - | - | - | - | - | - | - | - | - | - | - | - | - | R | F | V | V | R | N | - | - | P | W | G | - | - | - | - | - | - | - | -197 |
| Q3MBX8_ANAVT | - | - | - | - | - | - | - | - | - | - | - | - | - | - | - | - | - | - | - | - | - | - | - | - | - | - | - | - | - | - | - | - | - | - | - | - | - | - | - | - | - | - | - | R | F | V | V | R | N | - | - | P | W | G | - | - | - | - | - | - | - | -202 |
| Q8YZT8_NOSS1 | - | - | - | - | - | - | - | - | - | - | - | - | - | - | - | - | - | - | - | - | - | - | - | - | - | - | - | - | - | - | - | - | - | - | - | - | - | - | - | - | - | - | - | R | V | V | V | R | N | - | - | P | W | G | V | D | G | K | A | K | S | -196 |
| K9UIG2_9CHRO | - | - | - | - | - | - | - | - | - | - | - | - | - | - | - | - | - | - | - | - | - | - | - | - | - | - | - | - | - | - | - | - | - | - | - | - | - | - | - | - | - | - | - | R | V | V | V | R | N | - | - | P | W | G | I | D | Y | A | W | S | G | -207 |
| A4YRP8_BRASO | - | - | - | - | - | - | - | - | - | - | - | - | - | - | - | - | - | - | - | - | - | - | - | - | - | - | - | - | - | - | - | - | - | - | - | - | - | - | - | - | - | - | - | M | L | Q | V | R | N | - | - | P | W | G | - | - | - | - | - | - | - | -217 |
| H0TAL7_9BRAD | - | - | - | - | - | - | - | - | - | - | - | - | - | - | - | - | - | - | - | - | - | - | - | - | - | - | - | - | - | - | - | - | - | - | - | - | - | - | - | - | - | - | - | M | L | Q | V | R | N | - | - | P | W | G | S | - | - | - | - | - | - | -222 |
| H0SNL4_9BRAD | - | - | - | - | - | - | - | - | - | - | - | - | - | - | - | - | - | - | - | - | - | - | - | - | - | - | - | - | - | - | - | - | - | - | - | - | - | - | - | - | - | - | - | M | L | Q | V | R | N | - | - | P | W | G | S | - | - | - | - | - | - | -224 |
| H0S4P6_9BRAD | - | - | - | - | - | - | - | - | - | - | - | - | - | - | - | - | - | - | - | - | - | - | - | - | - | - | - | - | - | - | - | - | - | - | - | - | - | - | - | - | - | - | - | M | L | Q | V | R | N | - | - | P | W | G | S | - | - | - | - | - | - | -224 |
| A5EMK2_BRASB | - | - | - | - | - | - | - | - | - | - | - | - | - | - | - | - | - | - | - | - | - | - | - | - | - | - | - | - | - | - | - | - | - | - | - | - | - | - | - | - | - | - | - | M | L | Q | V | R | N | - | - | P | W | G | - | - | - | - | - | - | - | -217 |
| M4ZBJ4_9BRAD | - | - | - | - | - | - | - | - | - | - | - | - | - | - | - | - | - | - | - | - | - | - | - | - | - | - | - | - | - | - | - | - | - | - | - | - | - | - | - | - | - | - | - | M | L | Q | V | R | N | - | - | P | W | G | S | A | S | - | - | - | - | -226 |
| Q07SP7_RHOP5 | - | - | - | - | - | - | - | - | - | - | - | - | - | - | - | - | - | - | - | - | - | - | - | - | - | - | - | - | - | - | - | - | - | - | - | - | - | - | - | - | - | - | - | M | L | Q | V | R | N | - | - | P | W | G | - | - | - | - | - | - | - | -210 |
| B0JUQ7_MICAN | - | - | - | - | - | - | - | - | - | - | - | - | - | - | - | - | - | - | - | - | - | - | - | - | - | - | - | - | - | - | - | - | - | - | - | - | - | - | - | - | - | - | - | L | F | T | I | Y | N | - | - | P | W | G | - | - | - | - | - | - | - | -191 |
| K9WJ87_9CYAN | - | - | - | - | - | - | - | - | - | - | - | - | - | - | - | - | - | - | - | - | - | - | - | - | - | - | - | - | - | - | - | - | - | - | - | - | - | - | - | - | - | - | - | M | F | K | V | Y | N | - | - | P | Y | G | S | T | Q | - | - | - | - | -188 |
| G6FUE0_9CYAN | - | - | - | - | - | - | - | - | - | - | - | - | - | - | - | - | - | - | - | - | - | - | - | - | - | - | - | - | - | - | - | - | - | - | - | - | - | - | - | - | - | - | - | M | F | T | L | F | N | - | - | P | W | G | V | E | N | G | T | S | K | -200 |
| K9PHG5_9CYAN | - | - | - | - | - | - | - | - | - | - | - | - | - | - | - | - | - | - | - | - | - | - | - | - | - | - | - | - | - | - | - | - | - | - | - | - | - | - | - | - | - | - | - | Q | F | T | L | F | N | - | - | P | W | G | - | - | - | - | - | - | - | -196 |
| Q8YXX3_NOSS1 | - | - | - | - | - | - | - | - | - | - | - | - | - | - | - | - | - | - | - | - | - | - | - | - | - | - | - | - | - | - | - | - | - | - | - | - | - | - | - | - | - | - | - | R | F | R | L | Y | N | - | - | P | W | G | - | - | - | - | - | - | - | -211 |
| K9TN70_9CYAN | - | - | - | - | - | - | - | - | - | - | - | - | - | - | - | - | - | - | - | - | - | - | - | - | - | - | - | - | - | - | - | - | - | - | - | - | - | - | - | - | - | - | - | T | V | T | L | F | N | - | - | P | W | - | - | - | - | - | - | - | - | -214 |
| Q05QW3_9SYNE | - | - | - | - | - | - | - | - | - | - | - | - | - | - | - | - | - | - | - | - | - | - | - | - | - | - | - | - | - | - | - | - | - | - | - | - | - | - | - | - | - | - | - | R | F | N | I | Q | N | - | - | P | W | G | - | - | - | - | - | - | - | -238 |
| Q05QW2_9SYNE | - | - | - | - | - | - | - | - | - | - | - | - | - | - | - | - | - | - | - | - | - | - | - | - | - | - | - | - | - | - | - | - | - | - | - | - | - | - | - | - | - | - | - | Q | Y | T | I | R | N | - | - | P | Y | - | - | - | - | - | - | - | - | -205 |
| Q05QW4_9SYNE | - | - | - | - | - | - | - | - | - | - | - | - | - | - | - | - | - | - | - | - | - | - | - | - | - | - | - | - | - | - | - | - | - | - | - | - | - | - | - | - | - | - | - | R | F | D | I | R | N | - | - | P | W | - | - | - | - | - | - | - | - | -216 |
| E0UGX6_CYAP2 | - | - | - | - | - | - | - | - | - | - | - | - | - | - | - | - | - | - | - | - | - | - | - | - | - | - | - | - | - | - | - | - | - | - | - | - | - | - | - | - | - | - | - | T | F | H | L | R | N | - | - | P | W | G | - | - | - | - | - | - | - | -197 |
| K7WKF4_9NOST | - | - | - | - | - | - | - | - | - | - | - | - | - | - | - | - | - | - | - | - | - | - | - | - | - | - | - | - | - | - | - | - | - | - | - | - | - | - | - | - | - | - | - | T | F | H | L | R | N | - | - | P | W | G | S | T | H | A | D | V | T | -203 |
| E0UGY5_CYAP2 | - | - | - | - | - | - | - | - | - | - | - | - | - | - | - | - | - | - | - | - | - | - | - | - | - | - | - | - | - | - | - | - | - | - | - | - | - | - | - | - | - | - | - | R | F | H | L | R | N | - | - | P | W | G | - | - | - | - | - | - | - | -199 |
| A8LCE5_FRASN | - | - | - | - | - | - | - | - | - | - | - | - | - | - | - | - | - | - | - | - | - | - | - | - | - | - | - | - | - | - | - | - | - | - | - | - | - | - | - | - | - | - | - | Q | A | L | L | R | N | - | - | P | W | D | Q | A | G | T | D | D | R | -200 |
| D3CT47_9ACTO | - | - | - | - | - | - | - | - | - | - | - | - | - | - | - | - | - | - | - | - | - | - | - | - | - | - | - | - | - | - | - | - | - | - | - | - | - | - | - | - | - | - | - | R | V | L | V | R | S | - | - | P | W | D | Q | T | A | S | D | D | R | -204 |
| Q2J4J6_FRASC | - | - | - | - | - | - | - | - | - | - | - | - | - | - | - | - | - | - | - | - | - | - | - | - | - | - | - | - | - | - | - | - | - | - | - | - | - | - | - | - | - | - | - | R | V | L | L | R | N | - | - | P | W | D | P | A | P | G | Q | N | N | -224 |
| Q0RAZ2_FRAAA | - | - | - | - | - | - | - | - | - | - | - | - | - | - | - | - | - | - | - | - | - | - | - | - | - | - | - | - | - | - | - | - | - | - | - | - | - | - | - | - | - | - | - | R | V | L | L | R | N | - | - | P | W | D | P | A | P | G | A | D | N | -211 |
| E3J3F6_FRASU | - | - | - | - | - | - | - | - | - | - | - | - | - | - | - | - | - | - | - | - | - | - | - | - | - | - | - | - | - | - | - | - | - | - | - | - | - | - | - | - | - | - | - | R | V | L | L | R | N | - | - | P | W | E | H | V | - | G | G | E | L | -206 |
| ZP_09168240 | - | - | - | - | - | - | - | - | - | - | - | - | - | - | - | - | - | - | - | - | - | - | - | - | - | - | - | - | - | - | - | - | - | - | - | - | - | - | - | - | - | - | - | R | L | L | L | R | N | - | - | P | W | G | S | P | - | D | E | E | L | -217 |
| C7MDV9_BRAFD | - | - | - | - | - | - | - | - | - | - | - | - | - | - | - | - | - | - | - | - | - | - | - | - | - | - | - | - | - | - | - | - | - | - | - | - | - | - | - | - | - | - | - | H | V | L | L | R | N | - | - | P | - | - | - | - | - | - | - | - | - | -172 |
| ZP_10094989 | - | - | - | - | - | - | - | - | - | - | - | - | - | - | - | - | - | - | - | - | - | - | - | - | - | - | - | - | - | - | - | - | - | - | - | - | - | - | - | - | - | - | - | H | L | H | L | R | N | - | - | P | - | - | - | - | - | - | - | - | - | -172 |
| A4X268_SALTO | - | - | - | - | - | - | - | - | - | - | - | - | - | - | - | - | - | - | - | - | - | - | - | - | - | - | - | - | - | - | - | - | - | - | - | - | - | - | - | - | - | - | - | Q | V | Q | L | H | N | - | - | P | W | N | - | - | - | - | - | - | - | -231 |
| WP_043162213 | - | - | - | - | - | - | - | - | - | - | - | - | - | - | - | - | - | - | - | - | - | - | - | - | - | - | - | - | - | - | - | - | - | - | - | - | - | - | - | - | - | - | - | Q | V | Q | L | H | N | - | - | P | W | N | - | - | - | - | - | - | - | -231 |
| Q0BRV8_GRABC | - | - | - | - | - | - | - | - | - | - | - | - | - | - | - | - | - | - | - | - | - | - | - | - | - | - | - | - | - | - | - | - | - | - | - | - | - | - | - | - | - | - | - | Y | V | N | L | A | N | - | - | P | W | - | - | - | - | - | - | - | - | -199 |
| A9B2P0_HERA2 | - | - | - | - | - | - | - | - | - | - | - | - | - | - | - | - | - | - | - | - | - | - | - | - | - | - | - | - | - | - | - | - | - | - | - | - | - | - | - | - | - | - | - | T | V | T | L | Y | N | - | - | P | W | G | - | - | - | - | - | - | - | -190 |
| A9B3U8_HERA2 | - | - | - | - | - | - | - | - | - | - | - | - | - | - | - | - | - | - | - | - | - | - | - | - | - | - | - | - | - | - | - | - | - | - | - | - | - | - | - | - | - | - | - | T | V | T | L | Y | N | - | - | P | W | G | - | - | - | - | - | - | - | -192 |
| A9B2A3_HERA2 | - | - | - | - | - | - | - | - | - | - | - | - | - | - | - | - | - | - | - | - | - | - | - | - | - | - | - | - | - | - | - | - | - | - | - | - | - | - | - | - | - | - | - | W | V | T | L | R | N | - | - | P | W | - | - | - | - | - | - | - | - | -216 |
| D6ZC85_SEGRD | - | - | - | - | - | - | - | - | - | - | - | - | - | - | - | - | - | - | - | - | - | - | - | - | - | - | - | - | - | - | - | - | - | - | - | - | - | - | - | - | - | - | - | T | L | T | V | R | N | - | - | P | W | - | - | - | - | - | - | - | - | -238 |
| E5XTT9_9ACTO | - | - | - | - | - | - | - | - | - | - | - | - | - | - | - | - | - | - | - | - | - | - | - | - | - | - | - | - | - | - | - | - | - | - | - | - | - | - | - | - | - | - | - | Q | L | T | V | R | N | - | - | P | W | - | - | - | - | - | - | - | - | -238 |
| E5XTU0_9ACTO | - | - | - | - | - | - | - | - | - | - | - | - | - | - | - | - | - | - | - | - | - | - | - | - | - | - | - | - | - | - | - | - | - | - | - | - | - | - | - | - | - | - | - | A | L | T | V | R | N | - | - | P | W | - | - | - | - | - | - | - | - | -245 |
| D6B0I6_9ACTO | - | - | - | - | - | - | - | - | - | - | - | - | - | - | - | - | - | - | - | - | - | - | - | - | - | - | - | - | - | - | - | - | - | - | - | - | - | - | - | - | - | - | - | - | V | I | L | G | N | - | - | P | W | - | - | - | - | - | - | - | - | -207 |
| D9X719_STRVR | - | - | - | - | - | - | - | - | - | - | - | - | - | - | - | - | - | - | - | - | - | - | - | - | - | - | - | - | - | - | - | - | - | - | - | - | - | - | - | - | - | - | - | - | V | I | L | G | N | - | - | P | W | G | V | S | G | - | - | - | - | -211 |
| H6L010_SAPGL | - | - | - | - | - | - | - | - | - | - | - | - | - | - | - | - | - | - | - | - | - | - | - | - | - | - | - | - | - | - | - | - | - | - | - | - | - | - | - | - | - | - | - | - | I | K | L | R | N | - | - | P | W | G | - | - | - | - | - | - | - | -191 |
| K1ZP83_9BACT | - | - | - | - | - | - | - | - | - | - | - | - | - | - | - | - | - | - | - | - | - | - | - | - | - | - | - | - | - | - | - | - | - | - | - | - | - | - | - | - | - | - | - | Y | V | Q | L | R | N | - | - | P | W | G | Y | K | E | W | T | G | D | -220 |
| ADGB_HUMAN | - | - | - | - | - | - | - | - | - | - | - | - | - | - | - | - | - | - | - | - | - | - | - | - | - | - | - | - | - | - | - | - | - | - | - | - | - | - | - | - | D | V | K | E | F | K | P | E | S | - | - | S | L | T | - | - | - | - | - | - | - | -250 |
| J3QMP3_MOUSE | - | - | - | - | - | - | - | - | - | - | - | - | - | - | - | - | - | - | - | - | - | - | - | - | - | - | - | - | - | - | - | - | - | - | - | - | - | - | - | - | D | S | K | D | F | K | L | E | V | - | - | S | L | T | - | - | - | - | - | - | - | -250 |
| H2XR46_CIOIN | - | - | - | - | - | - | - | - | - | - | - | - | - | - | - | - | - | - | - | - | - | - | - | - | - | - | - | - | - | - | - | - | - | - | - | - | - | - | - | - | G | K | K | R | D | D | K | E | K | - | - | S | K | S | G | S | - | - | - | - | - | -257 |
| CAN7_MOUSE | - | - | - | - | - | - | - | - | - | - | - | - | - | - | - | - | - | - | - | - | - | - | - | - | - | - | - | - | - | - | - | - | - | - | - | - | - | - | - | - | G | L | R | F | I | Q | L | K | N | - | - | P | W | S | H | L | - | - | - | - | - | -223 |
| CAN7_HUMAN | - | - | - | - | - | - | - | - | - | - | - | - | - | - | - | - | - | - | - | - | - | - | - | - | - | - | - | - | - | - | - | - | - | - | - | - | - | - | - | - | G | L | R | F | I | Q | L | K | N | - | - | P | W | S | H | L | - | - | - | - | - | -223 |
| E1BY84_CHICK | - | - | - | - | - | - | - | - | - | - | - | - | - | - | - | - | - | - | - | - | - | - | - | - | - | - | - | - | - | - | - | - | - | - | - | - | - | - | - | - | G | L | R | F | L | Q | L | K | N | - | - | P | W | S | H | L | - | - | - | - | - | -223 |
| H9GX91_DANRE | - | - | - | - | - | - | - | - | - | - | - | - | - | - | - | - | - | - | - | - | - | - | - | - | - | - | - | - | - | - | - | - | - | - | - | - | - | - | - | - | D | K | R | F | L | Q | L | K | N | - | - | P | W | S | H | L | - | - | - | - | - | -223 |
| F6SGI6_CIOIN | - | - | - | - | - | - | - | - | - | - | - | - | - | - | - | - | - | - | - | - | - | - | - | - | - | - | - | - | - | - | - | - | - | - | - | - | - | - | - | - | G | I | K | L | L | Q | L | K | N | - | - | P | W | S | H | L | - | - | - | - | - | -221 |
| W4Y195_STRPU | - | - | - | - | - | - | - | - | - | - | - | - | - | - | - | - | - | - | - | - | - | - | - | - | - | - | - | - | - | - | - | - | - | - | - | - | - | - | - | - | G | K | K | L | L | Q | L | K | N | - | - | P | W | S | H | L | - | - | - | - | - | -197 |
| R7V270_CAPTE | - | - | - | - | - | - | - | - | - | - | - | - | - | - | - | - | - | - | - | - | - | - | - | - | - | - | - | - | - | - | - | - | - | - | - | - | - | - | - | - | G | H | R | L | F | Q | L | K | N | - | - | P | W | N | V | K | - | - | - | - | - | -222 |
| G4LXK3_SCHMA | - | - | - | - | - | - | - | - | - | - | - | - | - | - | - | - | - | - | - | - | - | - | - | - | - | - | - | - | - | - | - | - | - | - | - | - | - | - | - | - | G | Y | R | L | F | L | L | K | N | - | - | P | W | S | - | - | - | - | - | - | - | -221 |
| T2M2K0_HYDVU | - | - | - | - | - | - | - | - | - | - | - | - | - | - | - | - | - | - | - | - | - | - | - | - | - | - | - | - | - | - | - | - | - | - | - | - | - | - | - | - | G | K | R | L | C | L | L | K | N | - | - | P | W | S | H | L | - | - | - | - | - | -221 |
| B3RQK1_TRIAD | - | - | - | - | - | - | - | - | - | - | - | - | - | - | - | - | - | - | - | - | - | - | - | - | - | - | - | - | - | - | - | - | - | - | - | - | - | - | - | - | D | I | K | L | F | Q | L | K | N | - | - | P | W | S | H | L | - | - | - | - | - | -222 |
| I1CS59_RHIO9 | - | - | - | - | - | - | - | - | - | - | - | - | - | - | - | - | - | - | - | - | - | - | - | - | - | - | - | - | - | - | - | - | - | - | - | - | - | - | - | - | G | K | R | L | L | Q | V | K | N | - | - | P | W | S | H | K | - | - | - | - | - | -219 |
| I1GER3_AMPQE | - | - | - | - | - | - | - | - | - | - | - | - | - | - | - | - | - | - | - | - | - | - | - | - | - | - | - | - | - | - | - | - | - | - | - | - | - | - | - | - | G | V | R | L | L | Q | L | K | N | - | - | P | W | S | E | K | - | - | - | - | - | -219 |
| Q22143_CAEEL | - | - | - | - | - | - | - | - | - | - | - | - | - | - | - | - | - | - | - | - | - | - | - | - | - | - | - | - | - | - | - | - | - | - | - | - | - | - | - | - | T | K | R | L | L | K | V | K | N | - | - | P | W | T | H | S | - | - | - | - | - | -222 |
| L1JZN7_GUITH | - | - | - | - | - | - | - | - | - | - | - | - | - | - | - | - | - | - | - | - | - | - | - | - | - | - | - | - | - | - | - | - | - | - | - | - | - | - | - | - | G | R | R | L | V | K | L | M | N | - | - | P | W | R | R | M | - | - | - | - | - | -225 |
| D0NT34_PHYIT | - | - | - | - | - | - | - | - | - | - | - | - | - | - | - | - | - | - | - | - | - | - | - | - | - | - | - | - | - | - | - | - | - | - | - | - | - | - | G | K | K | I | R | L | L | Q | V | K | N | - | - | P | W | R | K | M | - | - | - | - | - | -243 |
| A0A064B913_ASPOZ | - | - | - | - | - | - | - | - | - | - | - | - | - | - | - | - | - | - | - | - | - | - | - | - | - | - | - | - | - | - | - | - | - | - | - | - | - | - | - | - | G | R | R | Q | L | L | V | K | N | - | - | P | W | A | G | - | - | - | - | - | - | -228 |
| A8N4X4_COPC7 | - | - | - | - | - | - | - | - | - | - | - | - | - | - | - | - | - | - | - | - | - | - | - | - | - | - | - | - | - | - | - | - | - | - | - | - | - | - | - | - | E | G | R | M | F | T | V | L | D | - | - | S | W | A | P | E | - | - | - | - | - | -245 |
| C0H5K1_PLAF7 | - | - | - | - | - | - | - | - | - | - | - | - | - | - | - | - | - | - | - | - | - | - | - | - | - | - | - | - | - | - | - | - | - | - | - | - | - | - | - | - | G | D | K | L | L | Y | I | K | N | - | - | P | W | G | C | I | - | - | - | - | - | -392 |
| K1REZ8_CRAGI | - | - | - | - | - | - | - | - | - | - | - | - | - | - | - | - | - | - | - | - | - | - | - | - | - | - | - | - | - | - | - | - | - | - | - | - | - | - | - | - | E | I | H | L | L | R | M | R | D | - | - | P | L | G | - | - | - | - | - | - | - | -208 |
| F4NTM9_BATDJ | - | - | - | - | - | - | - | - | - | - | - | - | - | - | - | - | - | - | - | - | - | - | - | - | - | - | - | - | - | - | - | - | - | - | - | - | - | - | - | K | T | V | P | V | V | Q | I | Y | K | - | - | P | F | L | N | - | - | - | - | - | - | -238 |
| Q6F6K5_CAEEL | - | - | - | - | - | - | - | - | - | - | - | - | - | - | - | - | - | - | - | - | - | - | - | - | - | - | - | - | - | - | - | - | - | - | - | - | - | - | - | - | - | H | G | Y | T | E | M | G | S | - | - | - | - | - | - | - | - | - | - | F | T | -179 |
| A8HZK8_CHLRE | - | - | - | - | - | - | - | - | - | - | - | - | - | - | - | - | - | - | - | - | - | - | - | - | - | - | - | - | - | - | - | - | - | - | - | - | - | - | - | F | E | G | S | G | E | E | G | E | Q | Q | G | P | Y | G | L | M | Y | G | - | - | Q | -207 |
| A8J6T6_CHLRE | - | S | Y | A | A | T | V | P | P | A | G | - | - | - | - | - | - | - | - | - | - | - | - | - | - | - | - | - | - | - | - | - | - | - | - | - | - | - | G | R | N | L | R | L | I | K | L | R | D | - | - | P | A | R | T | N | - | - | - | - | - | -273 |
| Z9JR51_9MICO | - | - | - | - | - | - | - | - | - | - | - | - | - | - | - | - | - | - | - | - | - | - | - | - | - | - | - | - | - | - | - | - | - | - | - | - | - | - | - | - | - | - | - | E | I | I | L | T | N | - | - | P | H | G | V | N | - | - | - | - | - | -202 |
| F7ZZH9_CELGA | - | - | - | - | - | - | - | - | - | - | - | - | - | - | - | - | - | - | - | - | - | - | - | - | - | - | - | - | - | - | - | - | - | - | - | - | - | - | - | - | - | - | - | - | V | W | M | R | N | - | - | P | W | G | P | - | - | - | - | - | - | -196 |
| Q7NDU2_GLOVI | - | - | - | - | - | - | - | - | - | - | - | - | - | - | - | - | - | - | - | - | - | - | - | - | - | - | - | - | - | - | - | - | - | - | - | - | - | - | - | - | - | - | - | - | V | E | M | Q | N | - | - | P | W | - | - | - | - | - | - | - | - | -172 |
| I6AT95_9BACT | - | - | - | - | - | - | - | - | - | - | - | - | - | - | - | - | - | - | - | - | - | - | - | - | - | - | - | - | - | - | - | - | - | - | - | - | - | - | - | - | - | - | - | E | I | V | L | W | N | - | - | P | H | G | - | - | - | - | - | - | - | -198 |
| C7MGJ8_BRAFD | - | - | - | - | - | - | - | - | - | - | - | - | - | - | - | - | - | - | - | - | - | - | - | - | - | - | - | - | - | - | - | - | - | - | - | - | - | - | - | - | - | T | T | Y | Y | E | F | S | N | - | - | P | H | G | - | - | - | - | - | - | - | -197 |
| D0WP73_9ACTO | - | - | - | - | - | - | - | - | - | - | - | - | - | - | - | - | - | - | - | - | - | - | - | - | - | - | - | - | - | - | - | - | - | - | - | - | - | - | - | - | - | K | T | N | V | V | L | Y | G | - | - | - | - | - | - | - | - | - | - | - | - | -193 |
| B0C8L1_ACAM1 | - | - | - | - | - | - | - | - | - | - | - | - | - | - | - | - | - | - | - | - | - | - | - | - | - | - | - | - | - | - | - | - | - | - | - | - | - | - | - | - | - | L | H | W | I | K | V | R | N | - | - | P | W | G | S | Y | G | V | A | Y | D | -185 |
| B3E5A8_GEOLS | - | - | - | - | - | - | - | - | - | - | - | - | - | - | - | - | - | - | - | - | - | - | - | - | - | - | - | - | - | - | - | - | - | - | - | - | - | - | - | - | - | - | - | A | V | T | I | W | N | - | - | P | W | G | - | - | - | - | - | - | - | -189 |
| D4YQ94_9MICO | - | - | - | - | - | - | - | - | - | - | - | - | - | - | - | - | - | - | - | - | - | - | - | - | - | - | - | - | - | - | - | - | - | - | - | - | - | - | - | - | A | P | W | R | V | H | V | H | N | - | - | P | L | G | - | - | - | - | - | - | - | -198 |
| PAPA_CARPA | - | - | - | - | - | - | - | - | - | - | - | - | - | - | - | - | - | - | - | - | - | - | - | - | - | - | - | - | - | - | - | - | - | - | - | - | - | - | - | - | - | P | N | Y | I | L | I | K | N | - | - | S | W | G | - | - | - | - | - | - | - | -178 |
| RIM13_YEAST | - | - | - | - | - | - | - | - | - | - | - | - | - | - | - | - | - | - | - | - | - | - | - | - | - | - | - | - | - | - | - | - | - | - | - | - | - | - | - | - | - | - | - | L | V | K | L | R | D | - | - | P | R | N | S | A | L | N | V | E | I | -218 |

| I |  |  |  |  |  |  |  |  |  |  |  |  |  |  |  |  |  |  |  |  |  |  |  |  |  |  |  |  |  |  |  |  |  |  |  |  |  |  |  |  |  |  |  |  |  |  |  |  |  |  |  |  |  |  |  |  |  |  |  |  |  |  |
| --- | --- | --- | --- | --- | --- | --- | --- | --- | --- | --- | --- | --- | --- | --- | --- | --- | --- | --- | --- | --- | --- | --- | --- | --- | --- | --- | --- | --- | --- | --- | --- | --- | --- | --- | --- | --- | --- | --- | --- | --- | --- | --- | --- | --- | --- | --- | --- | --- | --- | --- | --- | --- | --- | --- | --- | --- | --- | --- | --- | --- | --- | --- |
| Q9N4B1_CAEEL | - | S | K | E | W | N | G | A | W | S | D | G | S | S | E | - | W | S | Q | V | D | P | Q | Q | R | E | Q | M | G | - | - | - | - | - | - | - | - | - | - | - | V | Q | F | A | K | D | G | E | F | W | M | - | - | - | - | - | - | - | - | - | - | -254 |
| Q8IAA8_CAEEL | - | E | K | E | W | N | G | A | W | S | D | G | S | S | E | - | W | S | K | I | D | E | A | T | K | K | Q | I | D | - | - | - | - | - | - | - | - | - | - | - | V | Q | F | A | R | D | G | E | F | W | M | - | - | - | - | - | - | - | - | - | - | -254 |
| CAN_CAEEL | - | E | Q | E | W | N | G | P | W | S | D | N | S | R | E | - | W | R | S | V | P | D | S | V | K | Q | D | M | G | - | - | - | - | - | - | - | - | - | - | - | L | K | F | D | H | D | G | E | F | W | M | S | - | - | - | - | - | - | - | - | - | -254 |
| Q9U2B2_CAEEL | - | K | V | K | W | K | G | D | F | C | Y | R | S | H | R | - | W | F | G | L | D | K | E | K | R | E | - | - | S | - | - | - | - | - | - | - | - | - | - | - | F | R | I | K | E | D | G | E | F | W | M | S | - | - | - | - | - | - | - | - | - | -255 |
| Q9N4B2_CAEEL | - | D | T | E | W | N | G | D | W | S | D | K | S | S | L | - | W | E | Q | V | D | Q | E | Q | R | E | K | M | E | - | - | - | - | - | - | - | - | - | - | - | F | R | I | K | E | D | G | E | F | W | M | - | - | - | - | - | - | - | - | - | - | -253 |
| C3Y6P6_BRAFL | - | K | I | E | W | K | G | P | W | S | D | E | S | P | Q | - | W | E | G | V | T | E | E | R | K | E | E | L | G | - | - | - | - | - | - | - | - | - | - | - | V | V | K | R | D | D | G | E | F | W | M | S | - | - | - | - | - | - | - | - | - | -271 |
| C3Y6N1_BRAFL | - | K | T | E | W | K | G | P | W | G | D | - | - | - | - | - | - | - | - | - | - | - | - | - | - | - | - | - | - | - | - | - | - | - | - | - | - | - | - | - | - | - | - | - | - | - | - | - | - | - | - | - | - | - | - | - | - | - | - | - | - | -231 |
| CANA_DROME | - | E | A | E | W | N | G | P | W | S | D | S | S | P | E | - | W | R | Y | I | P | E | E | Q | K | A | E | I | G | - | - | - | - | - | - | - | - | - | - | - | L | T | F | D | R | D | G | E | F | W | M | S | - | - | - | - | - | - | - | - | - | -258 |
| CANB_DROME | - | D | A | E | W | S | G | P | W | S | D | S | S | P | E | - | W | R | F | I | P | E | H | T | K | E | E | I | G | - | - | - | - | - | - | - | - | - | - | - | L | N | F | D | R | D | G | E | F | W | M | S | - | - | - | - | - | - | - | - | - | -258 |
| X1Y3H71_ANODA | - | E | A | E | W | N | G | P | W | S | D | K | S | A | E | - | W | R | Y | I | P | D | E | Q | K | Q | E | L | G | - | - | - | - | - | - | - | - | - | - | - | L | N | F | D | H | D | G | E | F | W | M | S | - | - | - | - | - | - | - | - | - | -259 |
| Q5IW12_GECLA | - | E | A | E | W | V | G | S | W | S | D | K | S | P | E | - | W | Q | F | I | P | P | E | E | K | E | E | M | G | - | - | - | - | - | - | - | - | - | - | - | L | T | F | E | H | D | G | E | F | W | M | S | - | - | - | - | - | - | - | - | - | -258 |
| K1Q056_CRAGI | - | E | A | E | W | K | G | R | W | S | D | Q | S | R | E | - | W | S | L | I | P | D | S | Q | K | D | S | I | G | - | - | - | - | - | - | - | - | - | - | - | L | T | F | D | D | D | G | E | F | W | M | S | - | - | - | - | - | - | - | - | - | -258 |
| G4V6C0_SCHMA | - | E | S | E | W | S | G | P | W | S | D | Q | S | - | - | - | - | - | - | - | - | - | - | - | - | - | - | - | - | - | - | - | - | - | - | - | - | - | - | - | - | - | - | - | - | - | - | - | - | - | - | - | - | - | - | - | - | - | - | - | - | -234 |
| R7ULX9_CAPTE | - | S | H | E | W | K | G | A | W | S | D | D | S | E | E | - | W | T | L | V | P | E | E | E | K | E | E | M | G | - | - | - | - | - | - | - | - | - | - | - | L | T | Y | S | H | D | G | E | F | W | M | A | - | - | - | - | - | - | - | - | - | -259 |
| W4Z5Q7_STRPU | - | A | V | E | W | N | G | R | W | S | D | N | A | R | E | - | W | D | D | I | N | Q | R | E | R | K | N | L | G | - | - | - | - | - | - | - | - | - | - | - | L | V | S | G | D | D | G | E | F | W | M | E | F | S | D | F | K | R | N | F | E | -260 |
| R7VIB6_CAPTE | - | K | V | E | W | N | G | A | W | S | D | K | S | S | E | - | W | N | M | L | G | E | N | E | R | Q | E | L | G | - | - | - | - | - | - | - | - | - | - | - | L | S | F | E | D | D | G | E | F | W | M | S | Y | P | D | F | T | R | N | F | H | -267 |
| T2MJ45_HYDVU | - | D | R | E | W | T | G | A | W | S | D | K | S | N | E | - | W | H | R | V | S | Q | A | D | K | E | S | L | G | - | - | - | - | - | - | - | - | - | - | - | I | T | Y | D | D | D | G | E | F | W | M | S | F | D | D | F | R | K | N | F | T | -259 |
| R7UAB6_CAPTE | - | D | H | E | W | N | G | A | W | S | D | K | S | R | E | - | W | S | L | I | S | E | E | E | R | E | E | H | G | - | - | - | - | - | - | - | - | - | - | - | L | N | F | S | D | D | G | E | F | W | M | A | F | K | D | F | Q | A | N | F | Q | -267 |
| C3ZKN8_BRAFL | - | S | V | E | W | M | G | D | W | S | D | E | S | P | K | - | W | D | L | L | S | S | E | Q | K | A | K | L | N | T | - | - | - | - | - | D | D | D | G | D | L | N | F | G | D | D | G | E | F | W | M | D | F | E | D | W | R | T | N | F | T | -269 |
| B3RJS5_TRIAD | - | E | K | E | W | R | G | A | W | G | D | G | S | P | E | - | W | K | T | I | S | E | A | E | K | A | K | M | E | - | - | - | - | - | - | - | - | - | - | - | L | S | F | D | D | D | G | E | F | W | M | A | F | K | D | F | C | A | E | F | S | -264 |
| H2YVF0_CIOSA | - | Q | V | E | W | N | G | A | W | S | D | G | S | S | S | - | W | N | R | V | P | D | - | E | K | Q | R | L | D | - | - | - | - | - | - | - | - | - | - | - | V | E | F | G | E | D | G | E | F | W | M | S | - | - | - | - | - | - | - | - | - | -250 |
| G4VAG2_SCHMA | - | S | H | E | W | K | G | A | W | C | D | G | S | P | Q | - | W | R | E | I | S | E | Q | E | K | K | N | I | N | - | - | - | - | - | - | - | - | - | - | - | L | S | F | T | A | D | G | E | F | W | M | S | - | - | - | - | - | - | - | - | - | -257 |
| Smp_157500 | - | R | H | E | W | K | G | P | W | S | D | N | S | P | E | - | W | K | D | V | K | S | K | D | K | R | A | L | Q | - | - | - | - | - | - | - | - | - | - | - | Y | K | P | K | E | D | G | E | F | - | - | - | - | - | - | - | - | - | - | - | - | -254 |
| Q7SYA9_DANRE | - | Q | V | E | W | T | G | A | W | S | D | N | S | P | E | - | W | D | E | I | D | P | S | E | K | - | - | D | D | - | - | - | - | - | - | - | - | - | L | H | L | Q | - | M | E | D | G | E | F | W | M | S | - | - | - | - | - | - | - | - | - | -253 |
| A5PMP1_DANRE | - | Q | V | E | W | T | G | A | W | S | D | N | S | S | E | - | W | N | G | I | D | P | S | E | R | - | - | E | E | - | - | - | - | - | - | - | - | - | L | N | N | H | - | M | E | D | G | E | F | W | M | S | - | - | - | - | - | - | - | - | - | -253 |
| CANX_CHICK | - | Q | V | E | W | T | G | A | W | S | D | G | S | S | E | - | W | D | N | I | D | P | S | D | R | - | - | E | E | - | - | - | - | - | - | - | - | - | L | Q | L | K | - | M | E | D | G | E | F | W | M | S | - | - | - | - | - | - | - | - | - | -253 |
| CAN1_HUMAN | - | E | V | E | W | T | G | A | W | S | D | S | S | S | E | - | W | N | N | V | D | P | Y | E | R | - | - | D | Q | - | - | - | - | - | - | - | - | - | L | R | V | K | - | M | E | D | G | E | F | W | M | S | - | - | - | - | - | - | - | - | - | -253 |
| CAN1_MOUSE | - | E | V | E | W | K | G | P | W | S | D | S | S | Y | E | - | W | N | K | V | D | P | Y | E | R | - | - | E | Q | - | - | - | - | - | - | - | - | - | L | R | V | K | - | M | E | D | G | E | F | W | M | S | - | - | - | - | - | - | - | - | - | -253 |
| O42133_CHICK | - | E | V | E | W | T | G | A | W | S | D | N | S | S | E | - | W | N | A | V | E | P | A | L | R | - | - | Q | Q | - | - | - | - | - | - | - | - | - | L | M | V | R | - | M | E | D | G | E | F | W | M | S | - | - | - | - | - | - | - | - | - | -253 |
| Q7ZUR1_DANRE | - | E | V | E | W | T | G | A | W | S | D | N | S | R | E | - | W | D | N | V | D | R | S | V | R | - | - | G | R | - | - | - | - | - | - | - | - | - | L | Q | N | R | - | S | E | D | G | E | F | W | M | S | - | - | - | - | - | - | - | - | - | -253 |
| E9QFN0_DANRE | - | E | V | E | W | T | G | P | W | S | D | E | S | K | E | - | W | R | E | I | D | A | S | V | R | - | - | S | R | - | - | - | - | - | - | - | - | - | L | H | N | C | - | Q | E | D | G | E | F | W | M | A | - | - | - | - | - | - | - | - | - | -253 |
| CAN2_HUMAN | - | E | V | E | W | T | G | R | W | N | D | N | C | P | S | - | W | N | T | I | D | P | E | E | R | - | - | E | R | - | - | - | - | - | - | - | - | - | L | T | R | R | - | H | E | D | G | E | F | W | M | S | - | - | - | - | - | - | - | - | - | -253 |
| CAN2_MOUSE | - | Q | V | E | W | T | G | K | W | N | D | N | C | P | S | - | W | N | T | V | D | P | E | V | R | - | - | A | N | - | - | - | - | - | - | - | - | - | L | T | E | R | - | Q | E | D | G | E | F | W | M | S | - | - | - | - | - | - | - | - | - | -253 |
| CAN2_CHICK | - | E | V | E | W | T | G | K | W | N | D | N | C | P | N | - | W | S | G | V | D | P | E | V | R | - | - | E | R | - | - | - | - | - | - | - | - | - | L | T | R | R | - | H | E | D | G | E | F | W | M | A | - | - | - | - | - | - | - | - | - | -253 |
| Q6DFZ8_DANRE | - | Q | V | E | W | T | G | P | W | S | D | G | S | S | E | - | W | R | Q | I | S | D | N | D | R | - | - | E | R | - | - | - | - | - | - | - | - | - | L | S | S | K | - | A | E | D | G | E | F | W | M | S | - | - | - | - | - | - | - | - | - | -253 |
| Q504F5_DANRE | - | Q | V | E | W | T | G | A | W | S | D | N | S | P | E | - | W | N | S | V | A | A | S | E | R | - | - | P | D | - | - | - | - | - | - | - | - | - | A | S | A | - | - | - | E | D | G | E | F | W | M | A | - | - | - | - | - | - | - | - | - | -251 |
| Q5BLH5_DANRE | - | Q | V | E | W | T | G | A | W | S | D | N | S | S | E | - | W | N | A | V | D | P | S | E | R | - | - | E | N | - | - | - | - | - | - | - | - | - | V | K | S | - | - | - | E | D | G | E | F | W | M | S | - | - | - | - | - | - | - | - | - | -251 |
| Q4VBH3_DANRE | - | E | V | E | W | T | G | A | W | S | D | N | S | K | E | - | W | D | S | V | P | P | E | E | K | - | - | A | K | - | - | - | - | - | - | - | - | - | L | D | Y | S | - | A | E | D | G | E | F | W | M | A | - | - | - | - | - | - | - | - | - | -253 |
| CAN8_RAT | - | E | V | E | W | S | G | A | W | S | D | N | A | P | E | - | W | N | Y | I | D | P | R | R | K | - | - | E | E | - | - | - | - | - | - | - | - | - | L | D | K | K | - | A | E | D | G | E | F | W | M | S | - | - | - | - | - | - | - | - | - | -253 |
| CAN8_MOUSE | - | E | V | E | W | T | G | A | W | S | D | S | A | P | E | - | W | N | Y | I | D | P | Q | K | K | - | - | G | E | - | - | - | - | - | - | - | - | - | L | D | K | R | - | A | E | D | G | E | F | W | M | S | - | - | - | - | - | - | - | - | - | -253 |
| CAN8_HUMAN | - | E | V | E | W | S | G | A | W | S | D | D | A | P | E | - | W | N | H | I | D | P | R | R | K | - | - | E | E | - | - | - | - | - | - | - | - | - | L | D | K | K | - | V | E | D | G | E | F | W | M | S | - | - | - | - | - | - | - | - | - | -253 |
| XP_426117 | - | E | I | E | W | T | G | A | W | S | D | N | A | P | E | - | W | N | Y | I | D | P | K | Q | K | - | - | Q | A | - | - | - | - | - | - | - | - | - | L | D | K | Q | - | V | D | D | G | E | F | W | M | A | - | - | - | - | - | - | - | - | - | -253 |
| CAN9_MOUSE | - | Q | V | E | W | N | G | P | W | S | D | S | S | P | E | - | W | R | S | V | D | L | E | E | Q | - | - | K | R | - | - | - | - | - | - | - | - | - | L | G | H | T | A | L | D | D | G | E | F | W | - | - | - | - | - | - | - | - | - | - | - | -254 |
| CAN9_HUMAN | - | Q | V | E | W | N | G | S | W | S | D | S | S | P | E | - | W | R | S | V | G | P | A | E | Q | - | - | K | R | - | - | - | - | - | - | - | - | - | L | C | H | T | A | L | D | D | G | E | F | W | M | A | - | - | - | - | - | - | - | - | - | -254 |
| E1C6M3_CHICK | - | Q | V | E | W | N | G | P | W | S | D | N | S | L | E | - | W | R | L | V | S | P | S | E | Q | - | - | K | R | - | - | - | - | - | - | - | - | - | L | A | Q | T | A | L | D | D | G | E | F | W | M | - | - | - | - | - | - | - | - | - | - | -253 |
| Q6DG44_DANRE | - | Q | V | E | W | N | G | P | W | S | D | N | S | R | E | - | W | T | V | I | D | S | S | E | K | - | - | K | R | - | - | - | - | - | - | - | - | - | L | L | Q | N | S | L | D | D | G | E | F | W | M | - | - | - | - | - | - | - | - | - | - | -253 |
| CAN3_HUMAN | - | Q | V | E | W | N | G | S | W | S | D | R | W | K | D | - | W | S | F | V | D | K | D | E | K | - | - | A | R | - | - | - | - | - | - | - | - | - | L | Q | H | Q | V | T | E | D | G | E | F | W | M | S | - | - | - | - | - | - | - | - | - | -302 |
| CAN3_MOUSE | - | Q | V | E | W | N | G | S | W | S | D | G | W | K | D | - | W | S | F | V | D | K | D | E | K | - | - | A | R | - | - | - | - | - | - | - | - | - | L | Q | H | Q | V | T | E | D | G | E | F | W | M | S | - | - | - | - | - | - | - | - | - | -302 |
| CAN3_CHICK | - | Q | V | E | W | N | G | P | W | S | D | K | S | E | E | - | W | N | F | I | D | E | E | E | K | - | - | I | R | - | - | - | - | - | - | - | - | - | L | Q | H | K | I | A | E | D | G | E | F | W | I | S | - | - | - | - | - | - | - | - | - | -301 |
| Q66HU4_DANRE | - | Q | V | E | W | N | G | P | W | S | D | N | S | K | E | - | W | E | S | L | S | K | A | E | K | - | - | E | K | - | - | - | - | - | - | - | - | - | L | Q | Q | Q | N | A | E | D | G | E | F | W | M | S | F | E | D | F | K | K | N | Y | T | -266 |
| F1R632_DANRE | - | V | A | P | - | - | - | P | P | A | C | K | S | N | D | - | W | V | E | L | A | K | T | E | Q | - | - | D | K | - | - | - | - | - | - | - | - | - | Q | R | L | Q | P | G | E | Q | G | E | F | W | M | - | - | - | - | - | - | - | - | - | - | -253 |
| CAN11_HUMAN | - | R | I | E | W | N | G | A | W | S | D | S | A | R | E | - | W | E | E | V | A | S | D | I | Q | - | - | M | Q | - | - | - | - | - | - | - | - | - | L | L | H | K | - | T | E | D | G | E | F | W | M | S | - | - | - | - | - | - | - | - | - | -253 |
| CAN11_MOUSE | - | R | V | E | W | N | G | A | W | S | D | K | A | T | E | - | W | E | E | V | S | P | D | V | R | - | - | V | Q | - | - | - | - | - | - | - | - | - | L | L | H | K | - | K | D | D | G | D | F | W | M | S | - | - | - | - | - | - | - | - | - | -253 |
| CAN12_MOUSE | - | R | V | E | W | S | G | P | W | S | D | S | C | P | R | - | W | D | M | L | P | S | E | W | R | - | - | D | A | - | - | - | - | - | - | - | - | - | L | L | V | K | K | E | D | - | G | E | F | W | M | - | - | - | - | - | - | - | - | - | - | -249 |
| CAN12_HUMAN | - | C | V | E | W | T | G | A | W | S | D | S | C | P | R | - | W | D | T | L | P | T | E | C | R | - | - | D | A | - | - | - | - | - | - | - | - | - | L | L | V | K | K | E | D | - | G | E | F | W | M | - | - | - | - | - | - | - | - | - | - | -249 |
| ENSSARP00000008135 | - | H | V | E | W | N | G | A | W | - | - | - | - | - | - | - | - | - | - | - | - | - | - | - | - | - | - | - | - | - | - | - | - | - | - | - | - | - | - | - | - | - | - | - | - | - | - | - | - | - | - | - | - | - | - | - | - | - | - | - | - | -220 |
| ENSSARP00000010553 | - | H | L | R | W | K | G | R | Y | S | E | N | D | V | K | N | W | T | P | E | L | Q | K | Y | L | N | F | D | P | - | - | - | - | - | - | - | - | - | R | T | A | Q | K | I | D | N | G | I | F | W | I | S | - | - | - | - | - | - | - | - | - | -261 |
| A7E2G3_DANRE | - | F | V | E | Y | C | G | P | W | S | D | K | C | K | D | - | W | D | F | V | D | N | T | E | K | - | - | T | K | - | - | - | - | - | - | - | - | - | L | E | L | A | K | V | E | D | G | E | F | W | I | S | I | E | D | F | C | R | F | Y | N | -264 |
| Q6NW69_DANRE | - | K | A | E | W | E | G | P | W | S | D | K | K | G | I | E | W | H | M | V | T | A | E | E | Q | - | - | R | R | - | - | - | - | - | - | - | - | - | V | Q | R | I | Q | Q | E | D | G | E | F | W | M | S | L | A | D | F | R | Q | N | F | E | -261 |
| CAN13_HUMAN | - | E | A | E | W | R | G | R | W | S | D | G | S | Q | E | - | W | E | E | T | C | D | P | R | K | - | - | - | S | - | - | - | - | - | - | - | - | - | Q | L | H | K | K | R | E | D | G | E | F | W | M | S | - | - | - | - | - | - | - | - | - | -255 |
| CAN13_MOUSE | - | K | T | E | W | K | G | R | W | K | D | G | S | K | E | - | W | - | - | - | - | - | - | - | - | - | - | - | - | - | - | - | - | - | - | - | - | - | - | - | - | - | - | - | - | - | - | - | - | - | - | - | - | - | - | - | - | - | - | - | - | -232 |
| E1BRR5_CHICK | - | H | G | E | W | R | G | P | W | S | D | G | S | P | E | - | W | D | Y | V | E | P | E | I | K | - | - | - | E | - | - | - | - | - | - | - | - | - | E | L | Y | R | K | K | N | D | G | E | F | W | M | S | - | - | - | - | - | - | - | - | - | -250 |
| E7F0H3_DANRE | - | E | E | E | W | Q | G | D | W | S | D | E | S | P | L | - | W | E | T | V | C | E | E | A | R | - | - | - | K | - | - | - | - | - | - | - | - | - | C | C | R | E | V | L | N | D | G | E | F | W | M | S | M | E | D | F | T | R | L | F | E | -262 |
| Q1RLS9_DANRE | - | S | G | E | W | N | R | D | W | S | D | R | S | P | L | - | W | E | L | V | R | P | E | D | Q | - | - | - | K | - | - | - | - | - | - | - | - | - | Y | N | - | D | V | L | D | N | G | E | F | W | M | S | M | E | D | F | C | R | N | F | S | -262 |
| F1Q9Z1_DANRE | - | D | S | E | W | N | G | D | W | S | D | N | S | P | L | - | W | N | T | V | D | D | E | D | R | - | - | - | K | - | - | - | - | - | - | - | - | - | Q | L | - | L | V | S | D | N | G | E | F | W | M | S | M | K | D | F | L | R | T | F | D | -258 |
| A4QNV6_DANRE | - | K | R | E | W | T | G | A | W | C | D | K | S | S | M | - | W | N | K | V | S | E | S | E | R | - | - | - | T | - | - | - | - | - | - | - | - | - | K | C | W | S | L | A | N | D | G | E | F | W | M | S | M | E | D | F | S | K | N | F | E | -262 |
| D6W573_HUMAN | - | K | V | E | W | K | G | D | W | S | D | S | S | S | K | - | W | E | L | L | S | P | K | E | K | - | - | - | I | - | - | - | - | - | - | - | - | - | L | L | L | R | K | D | N | D | G | E | F | W | M | T | L | Q | D | F | K | T | H | F | V | -258 |
| R7UBC6_CAPTE | - | K | V | N | W | N | G | S | W | N | E | S | S | A | E | - | W | T | A | V | P | L | Q | D | R | - | - | Q | K | - | - | - | - | - | - | - | - | - | M | G | L | V | C | R | D | H | A | E | F | W | M | D | M | K | Q | F | L | H | H | F | D | -277 |
| K1RCZ2_CRAGI | - | K | V | E | W | K | G | S | W | G | D | R | S | R | E | - | W | N | S | I | P | A | D | D | R | - | - | E | K | - | - | - | - | - | - | - | - | - | M | G | L | I | F | R | D | D | G | E | F | W | M | - | - | - | - | - | - | - | - | - | - | -269 |
| Q5IW13_GECLA | - | E | T | E | W | K | G | S | W | S | D | K | S | P | E | - | W | N | A | V | P | P | G | E | R | - | - | Q | R | - | - | - | - | - | - | - | - | - | L | G | L | T | F | N | D | D | G | E | F | W | M | S | - | - | - | - | - | - | - | - | - | -261 |
| R7UUX7_CAPTE | - | R | D | E | W | K | G | A | W | S | D | Y | S | P | E | - | W | R | K | M | T | E | T | M | R | - | - | K | K | - | - | - | - | - | - | - | - | - | I | G | L | V | K | D | D | D | G | E | F | W | M | A | F | K | D | F | V | K | E | F | D | -264 |
| K1Q505_CRAGI | - | R | S | E | W | N | G | D | W | S | D | N | S | Y | Q | - | L | R | N | L | S | Q | D | A | R | - | - | Q | R | - | - | - | - | - | - | - | - | - | L | G | I | V | V | Q | D | E | G | E | F | W | I | - | - | - | - | - | - | - | - | - | - | -260 |
| R7UD02_CAPTE | - | F | K | E | W | N | G | A | W | S | D | S | S | E | E | - | W | T | S | L | S | E | E | Q | R | - | - | Q | T | - | - | - | - | - | - | - | - | - | F | D | L | T | V | D | N | D | G | E | F | W | I | T | L | G | D | W | L | D | N | F | H | -266 |
| CAN6_HUMAN | - | R | Q | E | W | S | G | P | W | S | E | I | S | E | E | - | W | Q | Q | L | T | A | S | D | R | K | N | L | - | - | - | - | - | - | - | G | L | V | M | S | D | - | - | - | - | D | G | E | F | W | M | S | - | - | - | - | - | - | - | - | - | -276 |
| CAN6_MOUSE | - | R | Q | E | W | S | G | P | W | S | E | I | S | E | E | - | W | Q | Q | L | T | V | T | D | R | K | N | L | - | - | - | - | - | - | - | G | L | V | M | S | D | - | - | - | - | D | G | E | F | W | M | S | - | - | - | - | - | - | - | - | - | -276 |
| E1C312_CHICK | - | N | R | E | W | N | G | A | W | S | D | N | S | E | E | - | W | K | K | V | S | N | A | E | R | K | S | L | - | - | - | - | - | - | - | G | L | T | V | E | N | - | - | - | - | D | G | E | F | W | M | T | - | - | - | - | - | - | - | - | - | -276 |
| E7FDN3_DANRE | - | T | T | D | W | T | G | A | W | G | L | G | S | P | E | - | W | Q | Q | F | N | R | K | E | K | E | K | M | - | - | - | - | - | - | - | G | L | I | V | R | D | - | - | - | - | V | G | E | F | W | M | - | - | - | - | - | - | - | - | - | - | -276 |
| CAN5_HUMAN | - | E | R | E | W | N | G | P | W | S | D | T | S | E | E | - | W | Q | K | V | S | K | S | E | R | E | K | M | - | - | - | - | - | - | - | G | V | T | V | Q | D | - | - | - | - | D | G | E | F | W | M | T | - | - | - | - | - | - | - | - | - | -272 |
| CAN5_MOUSE | - | E | R | E | W | T | G | P | W | S | D | T | S | E | E | - | W | Q | K | V | S | K | S | E | R | E | K | M | - | - | - | - | - | - | - | G | V | T | V | Q | D | - | - | - | - | D | G | E | F | W | M | T | - | - | - | - | - | - | - | - | - | -276 |
| E1C292_CHICK | - | E | R | E | W | N | G | P | W | S | D | T | S | E | E | - | W | Q | K | V | S | K | S | E | R | E | K | M | - | - | - | - | - | - | - | G | M | T | V | E | D | - | - | - | - | D | G | E | F | W | M | T | - | - | - | - | - | - | - | - | - | -276 |
| A0JMG3_DANRE | - | Q | R | G | W | N | G | A | W | S | D | S | S | E | E | - | W | K | R | V | S | K | G | E | R | E | R | L | - | - | - | - | - | - | - | G | V | T | V | E | D | - | - | - | - | D | G | E | F | W | M | - | - | - | - | - | - | - | - | - | - | -275 |
| E7F7F1_DANRE | - | E | K | E | W | S | G | P | W | S | D | S | S | E | E | - | W | K | K | V | S | K | S | E | R | E | K | L | - | - | - | - | - | - | - | G | V | T | V | Q | D | - | - | - | - | D | G | E | F | W | M | - | - | - | - | - | - | - | - | - | - | -275 |
| XP_006824412 | - | E | K | E | W | N | G | A | W | S | D | Q | S | P | E | - | W | S | K | V | G | K | S | E | R | E | K | M | - | - | - | - | - | - | - | G | L | T | F | D | D | - | - | - | - | D | G | E | F | W | M | T | - | - | - | - | - | - | - | - | - | -270 |
| W4YJN2_STRPU | - | E | K | E | W | N | G | P | W | S | D | G | S | P | E | - | W | Q | N | I | S | E | S | Q | R | K | S | I | - | - | - | - | - | - | - | G | I | V | N | E | D | - | - | - | - | D | G | E | F | W | M | - | - | - | - | - | - | - | - | - | - | -273 |
| C3YJZ3_BRAFL | - | Q | K | E | W | R | G | P | W | S | D | D | S | P | E | - | W | Q | Q | V | S | S | S | E | K | E | K | L | - | - | - | - | - | - | - | G | L | V | K | E | D | - | - | - | - | D | G | E | F | W | M | - | - | - | - | - | - | - | - | - | - | -278 |
| T2MIT4_HYDVU | - | E | K | E | W | N | G | A | W | S | D | G | S | A | E | - | W | K | K | L | S | V | K | E | R | E | K | L | - | - | - | - | - | - | - | G | I | K | N | E | N | - | - | - | - | D | G | E | F | W | M | S | - | - | - | - | - | - | - | - | - | -276 |
| XP_002154722 | - | D | T | E | W | N | G | P | W | S | D | G | S | P | Q | - | W | N | L | L | S | A | Q | E | R | E | K | Y | - | - | - | - | - | - | - | G | L | V | N | E | N | - | - | - | - | S | G | E | F | W | M | S | - | - | - | - | - | - | - | - | - | -240 |
| R7VLA7_CAPTE | - | G | T | E | W | K | G | A | F | S | D | E | S | E | E | - | W | K | K | I | D | K | E | S | R | E | K | I | - | - | - | - | - | - | - | G | L | T | F | D | D | - | - | - | - | D | G | E | F | W | M | - | - | - | - | - | - | - | - | - | - | -276 |
| K1R1C6_CRAGI | - | Q | G | E | W | N | G | P | F | S | D | G | S | P | E | - | W | Q | K | I | S | K | D | E | R | E | K | I | - | - | - | - | - | - | - | G | L | T | F | E | E | - | - | - | - | D | G | E | F | W | M | - | - | - | - | - | - | - | - | - | - | -276 |
| B3RIE5_TRIAD | - | K | G | E | W | K | G | A | W | S | D | G | S | E | E | - | W | N | R | V | K | K | S | E | R | E | K | V | - | - | - | - | - | - | - | G | L | T | F | E | E | - | - | - | - | D | G | E | F | W | M | S | - | - | - | - | - | - | - | - | - | -274 |
| K1PW75_CRAGI | - | G | T | E | W | K | G | A | W | S | D | G | S | P | E | - | W | E | K | I | S | S | S | E | K | K | D | M | - | - | - | - | - | - | - | G | L | T | F | D | E | - | - | - | - | D | G | D | F | W | M | T | - | - | - | - | - | - | - | - | - | -272 |
| K1RSB8_CRAGI | - | G | S | D | W | K | G | A | W | S | N | G | S | P | E | - | W | K | K | V | S | D | S | E | K | K | E | L | - | - | - | - | - | - | - | G | L | T | F | A | D | - | - | - | - | N | G | E | F | W | M | S | - | - | - | - | - | - | - | - | - | -270 |
| K1RWI3_CRAGI | - | G | T | E | W | K | G | A | W | S | D | G | S | E | E | - | W | S | K | V | S | D | G | E | K | K | E | L | - | - | - | - | - | - | - | G | I | T | I | D | D | - | - | - | - | N | G | E | F | W | M | S | - | - | - | - | - | - | - | - | - | -273 |
| R7VGC2_CAPTE | - | T | K | E | W | T | G | A | W | S | D | E | S | S | E | - | W | A | A | M | S | T | N | D | R | E | Q | M | - | - | - | - | - | - | - | G | I | K | F | E | S | - | - | - | - | E | G | E | F | W | M | - | - | - | - | - | - | - | - | - | - | -273 |
| K1QU26_CRAGI | - | I | K | E | W | N | G | P | W | S | D | D | S | P | E | - | W | S | K | I | N | K | S | E | W | E | K | M | - | - | - | - | - | - | - | G | L | K | F | E | Q | - | - | - | - | E | G | E | F | W | M | S | - | - | - | - | - | - | - | - | - | -275 |
| CAN5_CAEEL | - | E | K | E | W | N | G | A | W | S | D | D | S | P | E | - | W | Q | N | V | S | A | S | Q | L | S | T | M | - | - | - | - | - | - | - | G | V | Q | P | A | N | S | D | S | D | D | G | D | F | W | M | P | - | - | - | - | - | - | - | - | - | -284 |
| I1FGV4_AMPQE | - | Q | K | E | W | N | G | D | W | S | D | N | S | P | S | - | W | N | Q | I | S | A | D | K | R | K | E | I | L | E | - | - | - | - | - | G | E | S | A | K | E | - | - | - | - | D | G | E | F | Y | M | - | - | - | - | - | - | - | - | - | - | -255 |
| K1PHX3_CRAGI | - | H | F | E | W | N | G | A | W | S | D | G | S | P | H | - | W | E | A | L | S | E | D | L | R | K | R | L | - | - | - | - | - | - | - | R | P | D | K | A | D | - | - | - | - | D | G | E | F | W | M | S | - | - | - | - | - | - | - | - | - | -254 |
| B3RNL8_TRIAD | - | C | G | E | W | K | G | A | W | S | D | G | S | A | E | - | W | D | T | V | S | E | E | T | K | K | A | I | - | - | - | - | - | - | - | G | M | A | V | A | N | - | - | - | - | N | G | E | F | W | M | S | - | - | - | - | - | - | - | - | - | -255 |
| X1Y3H7_ANODA | - | S | G | E | W | N | R | A | W | S | D | Q | S | S | L | - | W | D | E | V | T | S | E | K | R | N | Q | L | S | - | - | - | - | - | - | - | - | - | - | - | - | S | V | R | Q | D | G | E | F | W | M | - | - | - | - | - | - | - | - | - | - | -265 |
| R7TWH9_CAPTE | - | E | T | E | W | K | G | S | W | S | D | D | D | S | N | - | W | Q | W | V | D | D | D | T | K | R | Y | M | E | M | - | - | - | - | - | T | - | - | - | - | - | - | H | R | D | D | G | E | F | W | M | S | F | K | D | F | C | R | H | F | Q | -267 |
| K1R5E8_CRAGI | - | N | N | E | W | K | G | S | W | S | D | S | D | V | N | - | W | T | W | V | D | D | Q | T | K | K | E | I | G | L | - | - | - | - | - | Q | - | - | - | - | - | - | A | K | D | D | G | E | F | W | M | S | - | - | - | - | - | - | - | - | - | -258 |
| XP_006811804 | - | A | T | E | W | N | G | N | W | S | D | E | S | A | T | - | W | D | L | V | S | D | E | V | K | G | Q | L | E | L | - | - | - | - | - | K | - | - | - | - | - | - | D | K | D | D | G | E | F | - | - | - | - | - | - | - | - | - | - | - | - | -254 |
| C3YB23_BRAFL | - | G | T | E | W | T | G | D | W | S | D | S | S | P | L | - | W | N | R | V | P | Q | H | E | R | D | R | I | G | F | - | - | - | - | - | T | - | - | - | - | - | - | D | K | D | D | G | E | F | W | M | - | - | - | - | - | - | - | - | - | - | -263 |
| H2ZIF1_CIOSA | - | K | N | E | W | K | G | D | W | S | D | D | S | F | T | - | W | H | Y | I | P | D | D | V | K | Y | S | M | G | Y | - | - | - | - | - | E | - | - | - | - | - | - | N | R | D | N | G | E | W | W | M | - | - | - | - | - | - | - | - | - | - | -253 |
| W4YV93_STRPU | - | S | T | E | W | K | G | K | W | S | D | S | S | K | L | - | W | N | N | L | K | N | K | A | E | F | E | E | D | E | - | - | - | - | - | E | R | - | - | - | - | - | S | L | S | D | G | E | F | W | M | S | - | - | - | - | - | - | - | - | - | -257 |
| CAN10_HUMAN | - | - | - | C | W | Q | G | L | W | R | E | G | G | E | G | - | W | S | Q | V | D | A | A | V | A | S | E | L | L | - | - | - | - | - | - | - | - | - | - | - | - | S | Q | L | Q | E | G | E | F | W | V | E | - | - | - | - | - | - | - | - | - | -262 |
| CAN10_MOUSE | - | - | - | C | W | Q | G | L | W | R | E | G | G | E | G | - | W | N | Q | V | E | P | A | K | E | S | E | L | L | - | - | - | - | - | - | - | - | - | - | - | - | A | Q | L | Q | E | G | E | F | W | V | E | - | - | - | - | - | - | - | - | - | -262 |
| XP_004943443 | - | - | - | C | W | R | G | P | W | C | E | G | G | E | G | - | W | S | Q | L | D | P | V | V | A | S | E | L | L | - | - | - | - | - | - | - | - | - | - | - | - | S | Q | I | Q | E | G | E | F | W | V | D | - | - | - | - | - | - | - | - | - | -262 |
| F8W4K1_DANRE | - | - | - | S | W | A | G | V | W | R | E | R | G | S | G | - | W | A | S | L | E | A | S | C | A | Q | T | L | L | - | - | - | - | - | - | - | - | - | - | - | - | S | R | T | A | K | G | E | F | W | M | D | - | - | - | - | - | - | - | - | - | -265 |
| CANC_DROME | - | K | T | H | F | L | G | D | W | S | P | M | S | K | T | - | W | E | R | V | S | Q | V | E | R | A | R | L | I | - | - | - | - | - | - | - | - | - | - | - | - | R | Q | L | G | P | G | E | F | W | L | S | - | - | - | - | - | - | - | - | - | -250 |
| C3ZKN9_BRAFL | - | - | - | - | - | - | - | - | - | R | E | I | P | L | L | - | R | L | R | S | P | H | G | E | T | V | D | W | K | - | - | - | - | - | - | - | - | - | - | - | - | G | S | W | S | E | E | - | - | - | - | - | - | - | - | - | - | - | - | - | - | -233 |
| G4V623_SCHMA | - | R | V | D | W | T | G | A | W | S | E | G | S | T | E | - | W | L | S | L | P | P | Q | D | R | L | K | V | G | - | - | - | - | - | - | - | - | - | - | - | - | L | V | K | A | Q | E | E | F | W | M | S | - | - | - | - | - | - | - | - | - | -275 |
| R7VK39_CAPTE | - | R | N | G | W | T | G | K | W | S | E | - | - | - | - | - | - | - | - | - | - | - | - | - | - | - | - | - | - | - | - | - | - | - | - | - | - | - | - | - | - | - | - | - | - | - | - | - | - | - | - | - | - | - | - | - | - | - | - | - | - | -226 |
| G4V693_SCHMA | - | Q | G | E | W | K | G | A | Y | S | S | S | S | S | E | - | W | E | E | I | N | L | E | A | R | K | R | L | G | - | - | - | - | - | - | - | - | - | - | - | L | T | F | D | S | E | T | E | F | W | I | - | - | - | - | - | - | - | - | - | - | -307 |
| R7UPU6_CAPTE | - | K | G | E | W | L | G | D | W | S | D | S | S | A | L | - | W | D | E | V | T | D | D | V | K | T | K | M | K | - | - | - | - | - | - | - | - | - | - | - | V | S | E | R | Q | D | G | E | F | W | M | T | L | E | D | Y | M | A | Y | F | S | -266 |
| R7V817_CAPTE | - | K | G | E | W | L | G | D | W | S | D | S | S | V | L | - | W | D | E | V | T | D | D | V | K | T | K | M | K | - | - | - | - | - | - | - | - | - | - | - | V | S | E | R | Q | D | G | E | F | W | M | A | - | - | - | - | - | - | - | - | - | -256 |
| R7UNL8_CAPTE | - | K | G | E | W | K | G | D | W | S | D | K | S | Y | L | - | W | N | D | V | T | D | S | V | K | E | K | M | K | - | - | - | - | - | - | - | - | - | - | - | V | N | K | K | M | D | G | E | F | W | M | T | M | A | D | F | M | S | H | F | C | -258 |
| K1PJ80_CRAGI | - | - | T | E | W | T | G | P | Y | S | D | G | S | P | E | - | W | Q | S | I | Q | S | I | I | E | G | - | - | - | - | - | - | - | - | - | - | - | - | - | - | - | P | N | K | D | D | G | E | F | Y | I | S | - | - | - | - | - | - | - | - | - | -247 |
| K1PCX8_CRAGI | - | - | T | E | W | I | G | P | Y | S | D | G | S | R | E | - | W | Q | S | I | Q | G | I | I | K | G | - | - | - | - | - | - | - | - | - | - | - | - | - | - | - | P | N | K | D | D | G | E | F | Y | I | S | - | - | - | - | - | - | - | - | - | -247 |
| CAND_DROME | - | Y | S | W | R | - | G | D | W | S | D | D | S | S | L | W | T | - | - | - | D | D | L | R | D | A | L | M | P | H | - | - | - | - | - | G | - | - | - | A | S | E | - | - | - | - | G | V | F | W | I | S | - | - | - | - | - | - | - | - | - | -261 |
| XP_002735765 | - | Y | S | W | K | - | G | D | W | S | D | E | S | P | L | W | T | - | - | - | P | D | L | R | D | A | L | M | V | H | - | - | - | - | - | G | - | - | - | A | A | E | - | - | - | - | G | V | F | W | M | S | - | - | - | - | - | - | - | - | - | -264 |
| CAN15_HUMAN | - | F | S | W | N | - | G | S | W | S | D | E | W | P | H | W | P | - | - | - | G | H | L | R | G | E | L | M | P | H | - | - | - | - | - | G | - | - | - | S | S | E | - | - | - | - | G | V | F | W | M | - | - | - | - | - | - | - | - | - | - | -261 |
| CAN15_MOUSE | - | F | S | W | N | - | G | S | W | S | D | E | W | P | H | W | P | - | - | - | G | H | L | R | A | E | L | M | P | H | - | - | - | - | - | G | - | - | - | S | S | E | - | - | - | - | G | V | F | W | M | E | - | - | - | - | - | - | - | - | - | -262 |
| E1BTA8_CHICK | - | F | S | W | N | - | G | S | W | S | D | E | W | P | H | W | P | - | - | - | A | P | L | R | H | D | L | M | P | H | - | - | - | - | - | G | - | - | - | S | S | E | - | - | - | - | G | V | F | W | M | E | - | - | - | - | - | - | - | - | - | -262 |
| E7FAX5_DANRE | - | F | S | W | N | - | G | S | W | S | D | E | W | S | D | W | P | - | - | - | Q | H | L | R | H | E | L | M | A | H | - | - | - | - | - | G | - | - | - | S | S | E | - | - | - | - | G | V | F | W | M | E | - | - | - | - | - | - | - | - | - | -262 |
| C3XWK8_BRAFL | - | F | S | W | K | - | G | D | W | S | D | S | S | P | L | W | T | - | - | - | P | E | M | R | D | R | L | L | A | H | - | - | - | - | - | G | - | - | - | A | S | E | - | - | - | - | G | V | F | W | M | - | - | - | - | - | - | - | - | - | - | -261 |
| W4YMU4_STRPU | - | F | S | W | N | - | G | A | W | S | D | G | S | A | L | W | T | - | - | - | D | I | M | K | E | A | L | M | A | H | - | - | - | - | - | G | - | - | - | A | S | E | - | - | - | - | G | I | F | W | M | S | - | - | - | - | - | - | - | - | - | -376 |
| K1PV74_CRAGI | - | F | S | W | N | - | G | D | W | S | D | D | S | A | R | W | A | N | I | S | T | R | A | K | Q | D | L | M | L | Y | - | - | - | - | - | G | - | - | - | H | T | S | - | - | - | - | G | V | F | W | M | A | - | - | - | - | - | - | - | - | - | -262 |
| R7TJF2_CAPTE | - | F | S | W | K | - | R | A | W | S | D | G | S | S | Q | W | M | E | I | S | Q | K | E | R | D | S | L | M | P | H | - | - | - | - | - | G | - | - | - | A | E | E | - | - | - | - | G | V | F | W | I | S | - | - | - | - | - | - | - | - | - | -267 |
| T2MI17_HYDVU | - | G | S | W | T | - | G | R | W | S | D | S | S | F | L | W | T | - | - | - | N | E | L | R | E | M | L | N | A | H | - | - | - | - | - | G | - | - | - | S | E | E | - | - | - | - | G | V | F | W | I | S | - | - | - | - | - | - | - | - | - | -260 |
| Q1RL55_CIOIN | - | F | S | W | K | - | G | D | W | S | N | E | S | V | L | W | Q | Q | - | N | P | Q | L | A | N | Q | L | F | Q | R | - | - | - | - | - | N | - | - | - | A | D | N | - | - | - | - | G | T | F | W | M | - | - | - | - | - | - | - | - | - | - | -251 |
| I1EA30_AMPQE | - | T | K | P | G | - | R | E | W | R | E | S | E | P | - | - | - | - | - | - | - | - | - | - | - | - | - | - | - | - | - | - | - | - | - | - | - | - | - | - | - | - | - | - | - | - | - | - | - | - | - | - | - | - | - | - | - | - | - | - | - | -234 |
| G5EEZ6_CAEEL | - | F | V | W | N | - | G | K | W | S | D | G | W | P | G | W | P | - | - | - | I | G | M | K | Q | K | F | L | N | Q | - | - | - | - | - | R | - | - | - | K | D | E | - | - | - | T | G | A | F | W | M | - | - | - | - | - | - | - | - | - | - | -258 |
| O44903_CAEEL | - | F | V | W | N | - | G | K | W | S | D | G | W | L | E | W | P | - | - | - | I | D | M | K | R | A | L | L | D | K | - | - | - | - | - | R | - | - | - | S | D | E | - | - | - | T | G | A | F | W | M | - | - | - | - | - | - | - | - | - | - | -258 |
| O02260_CAEEL | - | S | V | W | T | - | R | N | W | S | K | E | W | E | W | W | P | E | N | M | K | D | L | L | E | G | M | I | R | - | - | - | - | - | - | - | - | - | - | - | - | - | - | - | - | - | G | S | F | W | I | S | - | - | - | - | - | - | - | - | - | -241 |
| O02259_CAEEL | - | S | V | W | T | - | G | N | W | S | K | E | W | E | W | W | P | E | N | M | K | D | L | L | E | G | M | I | R | - | - | - | - | - | - | - | - | - | - | - | - | - | - | - | - | - | G | S | F | W | I | S | - | - | - | - | - | - | - | - | - | -232 |
| C1MTG3_MICPC | - | K | E | W | N | - | G | E | W | G | A | K | S | E | T | W | T | - | - | - | S | R | L | G | A | E | L | G | N | T | - | - | - | - | - | R | - | - | - | A | D | - | - | - | D | - | G | T | F | W | M | S | - | - | - | - | - | - | - | - | - | -342 |
| C1E493_MICSR | - | K | E | W | S | - | G | E | W | G | A | G | S | E | V | W | T | - | - | - | S | K | L | G | A | E | L | G | H | T | - | - | - | - | - | R | - | - | - | V | D | - | - | - | D | - | G | T | F | W | M | S | - | - | - | - | - | - | - | - | - | -275 |
| Q012Z6_OSTTA | - | R | E | W | R | - | G | E | F | S | S | N | S | D | A | W | T | - | - | - | K | R | L | G | D | V | L | S | R | T | - | - | - | - | - | R | - | - | - | A | D | - | - | - | D | - | G | E | F | W | M | S | - | - | - | - | - | - | - | - | - | -263 |
| L1IGB6_GUITH | - | G | E | W | N | - | G | D | W | S | D | A | S | E | K | W | S | - | - | - | G | M | I | C | E | G | L | E | R | T | - | - | - | - | - | G | - | - | - | V | N | - | - | - | D | - | G | T | F | W | M | - | - | - | - | - | - | - | - | - | - | -250 |
| B7FWX6_PHATC | - | R | E | W | K | - | G | D | W | S | V | D | S | E | R | W | T | - | - | - | R | A | L | R | K | K | I | G | S | D | - | - | - | - | - | A | - | - | - | F | A | R | G | - | D | - | G | T | F | F | M | S | - | - | - | - | - | - | - | - | - | -279 |
| N9TPR9_ENTHI | - | C | E | W | K | - | G | K | W | R | D | D | D | P | - | - | - | A | W | T | E | S | M | K | K | K | L | E | V | V | - | - | - | - | - | E | - | - | - | V | N | - | - | - | - | D | G | I | Y | F | M | - | - | - | - | - | - | - | - | - | - | -238 |
| G0QS43_ICHMG | - | G | E | W | K | - | G | K | W | N | D | N | D | E | D | L | M | K | L | N | E | E | N | Q | R | K | I | G | F | V | - | - | - | - | - | Q | - | - | - | N | S | R | - | - | - | D | G | T | F | F | I | - | - | - | - | - | - | - | - | - | - | -254 |
| D8LB20_ECTSI | - | G | E | W | K | - | G | R | W | A | D | G | T | S | - | E | W | K | T | S | P | L | I | S | V | A | V | G | H | E | - | - | - | - | - | N | - | - | - | N | D | N | - | - | - | D | G | T | F | W | M | - | - | - | - | - | - | - | - | - | - | -288 |
| Q9U0T9_LEIMA | - | S | G | W | T | - | R | A | W | A | W | D | A | L | E | W | Q | Q | - | H | R | D | I | A | T | A | C | G | F | S | - | - | - | - | - | K | - | - | - | H | K | G | - | - | D | T | S | I | V | W | M | T | - | - | - | - | - | - | - | - | - | -282 |
| Q4CXJ8_TRYCC | - | E | E | W | S | - | G | P | W | S | S | E | S | S | E | W | D | S | - | H | P | E | V | R | D | A | C | G | - | S | - | - | - | - | - | S | - | - | - | H | R | - | - | - | D | D | K | V | I | W | M | - | - | - | - | - | - | - | - | - | - | -281 |
| F4I0A4_ARATH | - | V | E | W | N | - | G | P | W | S | D | S | S | P | E | W | T | - | - | - | D | R | M | K | H | K | L | K | H | V | - | - | - | - | - | P | - | - | - | Q | S | - | - | - | K | E | G | I | F | W | M | S | - | - | - | - | - | - | - | - | - | -256 |
| Q8RUQ1_MAIZE | - | V | E | W | N | - | G | P | W | S | D | S | S | P | E | W | T | - | - | - | E | R | M | K | H | K | L | M | H | V | - | - | - | - | - | P | - | - | - | Q | S | - | - | - | K | N | G | V | F | W | M | S | - | - | - | - | - | - | - | - | - | -256 |
| D7FQ17_ECTSI | - | E | E | W | R | - | G | D | W | G | D | N | S | M | L | W | T | - | - | - | R | R | R | R | A | R | L | G | L | V | - | - | - | - | - | K | - | - | - | N | D | - | - | - | K | D | N | A | F | W | M | S | - | - | - | - | - | - | - | - | - | -273 |
| D0MZ44_PHYIT | - | Q | E | W | K | - | G | D | W | S | D | S | S | R | L | W | T | - | - | - | R | R | L | R | K | R | L | G | V | R | - | - | - | - | - | K | - | - | - | G | D | D | A | G | D | D | N | T | F | W | M | S | - | - | - | - | - | - | - | - | - | -277 |
| L1JBM0_GUITH | - | H | E | W | T | - | G | R | Y | S | R | E | S | P | S | W | E | D | V | D | D | N | I | L | S | M | L | G | L | T | - | - | - | - | - | R | E | A | L | R | R | E | V | F | E | D | G | S | F | F | M | - | - | - | - | - | - | - | - | - | - | -231 |
| L1J739_GUITH | - | K | E | W | T | - | G | R | W | A | D | F | A | P | E | W | E | K | L | R | E | R | - | - | - | - | - | - | - | - | - | - | - | - | - | - | - | - | - | Y | P | D | A | P | D | - | - | - | - | W | M | - | - | - | - | - | - | - | - | - | - | -252 |
| L1J0U9_GUITH | - | S | P | W | S | - | G | M | W | S | N | D | G | P | E | W | V | A | N | P | E | I | A | K | A | L | D | - | - | - | - | - | - | - | - | - | - | - | - | Y | D | K | A | S | M | G | G | S | F | W | M | T | - | - | - | - | - | - | - | - | - | -256 |
| L1J1Q4_GUITH | - | G | V | W | D | - | G | D | W | G | E | D | S | A | L | W | S | I | Y | P | E | V | S | K | H | L | G | - | - | - | - | - | - | - | - | - | - | - | - | R | M | Q | E | P | G | - | - | V | F | W | M | S | - | - | - | - | - | - | - | - | - | -274 |
| D0ND88_PHYIT | - | G | M | W | K | - | G | E | W | A | N | D | D | S | K | W | E | E | H | T | Q | V | E | Q | A | M | H | S | D | P | - | - | - | - | - | R | - | C | E | F | S | R | S | G | H | D | G | C | F | W | M | - | - | - | - | - | - | - | - | - | - | -320 |
| D2VVS6_NAEGR | - | I | E | W | K | - | G | E | W | S | D | Y | C | P | R | W | S | - | - | - | P | E | L | R | K | Y | V | G | N | D | - | - | - | - | - | F | N | - | - | - | - | - | - | S | Q | D | G | I | F | Y | M | S | - | - | - | - | - | - | - | - | - | -259 |
| D2V3V2_NAEGR | - | G | E | L | E | S | G | D | Y | S | D | D | S | K | W | W | T | - | - | - | K | E | L | R | K | Y | V | G | H | E | - | - | - | - | - | S | - | - | - | - | - | - | - | K | I | D | G | I | F | F | L | T | L | G | A | L | V | K | Y | Y | T | -268 |
| Q5B5J7_EMENI | - | - | K | E | W | N | G | A | W | S | D | G | S | E | Q | W | T | P | Q | W | M | E | K | L | N | - | - | - | H | K | - | - | - | - | - | F | G | N | D | G | F | F | W | I | S | Y | E | D | L | L | K | K | Y | Q | H | F | D | R | T | R | L | -293 |
| E4ZY10_LEPMJ | - | - | T | E | W | T | G | R | W | S | D | G | S | E | E | W | T | A | E | W | M | Q | K | L | N | - | - | - | H | K | - | - | - | - | - | F | G | D | D | G | V | F | W | I | S | - | - | - | - | - | - | - | - | - | - | - | - | - | - | - | - | -273 |
| A8PIE5_COPC7 | - | - | G | E | W | K | G | A | W | S | D | G | S | K | E | W | T | P | E | W | L | Q | V | L | P | E | L | G | H | T | - | - | - | - | - | F | G | D | D | G | Q | F | V | M | E | Y | K | D | F | L | D | S | F | A | D | V | E | R | T | F | L | -296 |
| A8NBA6_COPC7 | - | - | S | E | W | T | G | P | W | A | D | G | S | K | E | W | D | S | E | L | V | K | I | L | P | E | L | G | H | Q | - | - | - | - | - | F | G | D | D | G | Q | F | V | M | E | Y | C | D | F | L | R | C | F | E | I | I | E | R | T | L | L | -293 |
| A8NBA1_COPC7 | - | - | Y | E | W | T | G | R | W | S | D | G | S | K | E | W | T | S | - | - | - | - | - | - | - | - | - | - | - | - | - | - | - | - | - | - | - | - | - | - | - | - | - | - | - | - | - | - | - | - | - | - | - | - | - | - | - | - | - | - | - | -249 |
| A8NBA2_COPC7 | - | - | S | E | W | G | G | P | W | S | D | G | S | K | E | W | - | - | - | - | - | - | - | - | - | - | - | - | - | - | - | - | - | - | - | - | - | - | - | - | - | - | - | - | - | - | - | - | - | - | - | - | - | - | - | - | - | - | - | - | - | -247 |
| A8NAE6_COPC7 | - | - | A | A | W | G | G | P | W | S | D | G | S | K | E | W | T | P | E | W | L | E | I | L | P | E | - | - | - | - | - | - | - | - | - | - | - | - | - | - | - | - | - | - | - | - | - | - | - | - | - | - | - | - | - | - | - | - | - | - | - | -285 |
| A8NAI9_COPC7 | - | I | A | K | W | N | G | R | W | G | D | G | S | K | E | W | T | E | L | G | R | E | E | R | E | E | I | L | R | V | - | - | - | - | - | M | E | - | - | - | - | - | - | - | - | - | - | - | - | - | - | - | - | - | - | - | - | - | - | - | - | -269 |
| G4TER7_PIRID | - | - | G | E | W | R | G | R | W | S | D | G | A | K | E | W | - | - | - | - | - | - | - | - | - | - | - | - | - | - | - | - | - | - | - | - | - | - | - | - | - | - | - | - | - | - | - | - | - | - | - | - | - | - | - | - | - | - | - | - | - | -253 |
| G4TER6_PIRID | - | - | G | E | W | K | G | R | W | S | D | G | S | N | E | W | - | - | - | - | - | - | - | - | - | - | - | - | - | - | - | - | - | - | - | - | - | - | - | - | - | - | - | - | - | - | - | - | - | - | - | - | - | - | - | - | - | - | - | - | - | -242 |
| G4TNA1_PIRID | - | - | N | E | W | K | G | R | W | S | D | G | S | K | E | W | - | - | - | - | - | - | - | - | - | - | - | - | - | - | - | - | - | - | - | - | - | - | - | - | - | - | - | - | - | - | - | - | - | - | - | - | - | - | - | - | - | - | - | - | - | -252 |
| D0MWB9_PHYIT | - | - | - | E | W | T | G | P | W | S | D | S | S | D | E | - | - | - | - | - | - | - | - | - | - | - | - | - | - | - | - | - | - | - | - | - | - | - | - | - | - | - | - | - | - | - | - | - | - | - | - | - | - | - | - | - | - | - | - | - | - | -249 |
| L1I9H5_GUITH | - | E | F | E | W | N | G | R | Y | S | D | K | S | E | L | W | T | P | E | L | K | S | Q | L | D | H | E | D | E | - | - | - | - | - | - | - | - | - | - | - | - | - | - | - | E | D | G | S | F | W | M | - | - | - | - | - | - | - | - | - | - | -261 |
| Q7N497_PHOLL | - | V | V | K | Y | K | G | P | Y | S | P | D | A | A | - | E | W | E | N | I | P | A | D | V | Q | N | D | I | F | Q | - | - | - | - | - | - | - | - | - | - | N | N | R | F | E | G | K | S | F | W | M | - | - | - | - | - | - | - | - | - | - | -225 |
| ZP_11152369 | - | V | A | N | Y | T | G | P | F | S | P | E | S | S | K | D | W | E | I | I | P | N | A | I | Q | K | E | L | F | E | - | - | - | - | - | - | - | - | - | - | V | D | R | F | K | K | T | T | F | W | M | - | - | - | - | - | - | - | - | - | - | -223 |
| A2FEL7_TRIVA | - | E | Q | E | W | K | G | D | W | S | A | E | S | S | M | W | K | K | Y | P | A | V | A | S | K | C | N | F | D | K | - | - | - | - | - | D | - | - | - | - | - | R | T | Q | T | D | G | V | F | W | M | - | - | - | - | - | - | - | - | - | - | -253 |
| A2D9A8_TRIVA | - | K | T | E | W | N | G | D | W | S | D | D | S | D | L | W | D | R | Y | P | A | V | K | N | R | C | K | F | E | K | - | - | - | - | - | - | - | - | - | - | - | - | - | A | D | D | G | M | F | W | I | - | - | - | - | - | - | - | - | - | - | -250 |
| A2FHB3_TRIVA | - | D | S | E | W | N | G | D | W | S | D | K | S | P | L | W | T | K | H | P | D | I | A | K | Q | L | G | H | T | D | - | - | - | - | - | - | - | - | - | - | - | - | - | V | D | D | G | M | F | W | I | T | - | - | - | - | - | - | - | - | - | -253 |
| A2DTE8_TRIVA | - | I | D | E | D | S | V | E | M | P | K | D | S | M | K | W | Q | E | N | K | D | L | K | D | L | C | H | L | S | P | - | - | - | - | - | - | - | - | - | - | - | - | - | T | D | D | G | T | F | W | I | - | - | - | - | - | - | - | - | - | - | -241 |
| A2E4X2_TRIVA | - | M | D | D | W | N | G | N | F | S | A | M | S | D | K | W | A | I | G | F | K | R | Q | V | G | Y | K | E | G | G | - | - | - | - | - | H | - | - | - | - | - | F | W | I | S | D | G | D | F | - | - | - | - | - | - | - | - | - | - | - | - | -247 |
| A2E1X1_TRIVA | - | - | - | - | - | - | - | - | - | - | - | - | - | - | - | - | - | - | - | - | - | - | - | - | - | - | - | - | - | - | - | - | - | - | - | - | - | - | - | - | - | - | - | - | - | - | - | - | - | - | - | - | - | - | - | - | - | - | - | - | - | -191 |
| TPR_PORGI | - | G | V | L | S | K | R | D | W | V | I | H | F | D | N | M | K | W | F | N | L | S | K | D | D | G | I | F | - | - | - | - | - | - | - | - | - | - | - | - | - | - | - | - | - | - | - | - | - | - | - | - | - | - | - | - | - | - | - | - | - | -274 |
| D5MF42_9BACT | - | - | - | - | - | - | - | - | - | - | - | - | - | - | - | - | - | - | - | - | - | - | - | - | - | - | - | - | - | - | - | - | - | - | - | - | - | - | - | - | - | - | - | - | - | - | - | - | - | - | - | - | - | - | - | - | - | - | - | - | - | -202 |
| R9H254_BACT4 | - | - | - | - | - | - | - | - | - | - | - | - | - | - | - | - | - | - | - | - | - | - | - | - | - | - | - | - | - | - | - | - | - | - | - | - | - | - | - | - | - | - | - | - | - | - | - | - | - | - | - | - | - | - | - | - | - | - | - | - | - | -189 |
| ZP_04847694 | - | - | - | - | - | - | - | - | - | - | - | - | - | - | - | - | - | - | - | - | - | - | - | - | - | - | - | - | - | - | - | - | - | - | - | - | - | - | - | - | - | - | - | - | - | - | - | - | - | - | - | - | - | - | - | - | - | - | - | - | - | -189 |
| D7IBA4_9BACE | - | - | - | - | - | - | - | - | - | - | - | - | - | - | - | - | - | - | - | - | - | - | - | - | - | - | - | - | - | - | - | - | - | - | - | - | - | - | - | - | - | - | - | - | - | - | - | - | - | - | - | - | - | - | - | - | - | - | - | - | - | -187 |
| ZP_09858577 | - | - | - | - | - | - | - | - | - | - | - | - | - | - | - | - | - | - | - | - | - | - | - | - | - | - | - | - | - | - | - | - | - | - | - | - | - | - | - | - | - | - | - | - | - | - | - | - | - | - | - | - | - | - | - | - | - | - | - | - | - | -187 |
| E5C759_9BACE | - | - | - | - | - | - | - | - | - | - | - | - | - | - | - | - | - | - | - | - | - | - | - | - | - | - | - | - | - | - | - | - | - | - | - | - | - | - | - | - | - | - | - | - | - | - | - | - | - | - | - | - | - | - | - | - | - | - | - | - | - | -189 |
| A7LTS0_BACO1 | - | - | - | - | - | - | - | - | - | - | - | - | - | - | - | - | - | - | - | - | - | - | - | - | - | - | - | - | - | - | - | - | - | - | - | - | - | - | - | - | - | - | - | - | - | - | - | - | - | - | - | - | - | - | - | - | - | - | - | - | - | -187 |
| Q8YQK8_NOSS1 | - | - | - | - | - | - | - | - | - | - | - | - | - | - | - | - | - | - | - | - | - | - | - | - | - | - | - | - | - | - | - | - | - | - | - | - | - | - | - | - | - | - | - | - | - | - | - | - | - | - | - | - | - | - | - | - | - | - | - | - | - | -197 |
| Q3MBX8_ANAVT | - | - | - | - | - | - | - | - | - | - | - | - | - | - | - | - | - | - | - | - | - | - | - | - | - | - | - | - | - | - | - | - | - | - | - | - | - | - | - | - | - | - | - | - | - | - | - | - | - | - | - | - | - | - | - | - | - | - | - | - | - | -202 |
| Q8YZT8_NOSS1 | - | G | A | N | D | - | - | - | - | - | - | - | - | - | - | - | - | - | - | - | - | - | - | - | - | - | - | - | - | - | - | - | - | - | - | - | - | - | - | - | - | - | - | - | - | - | - | - | - | - | - | - | - | - | - | - | - | - | - | - | - | -200 |
| K9UIG2_9CHRO | - | A | A | D | G | N | - | - | - | - | - | - | - | - | - | - | - | - | - | - | - | - | - | - | - | - | - | - | - | - | - | - | - | - | - | - | - | - | - | - | - | - | - | - | - | - | - | - | - | - | - | - | - | - | - | - | - | - | - | - | - | -212 |
| A4YRP8_BRASO | - | - | - | - | - | - | - | - | - | - | - | - | - | - | - | - | - | - | - | - | - | - | - | - | - | - | - | - | - | - | - | - | - | - | - | - | - | - | - | - | - | - | - | - | - | - | - | - | - | - | - | - | - | - | - | - | - | - | - | - | - | -217 |
| H0TAL7_9BRAD | - | - | - | - | - | - | - | - | - | - | - | - | - | - | - | - | - | - | - | - | - | - | - | - | - | - | - | - | - | - | - | - | - | - | - | - | - | - | - | - | - | - | - | - | - | - | - | - | - | - | - | - | - | - | - | - | - | - | - | - | - | -222 |
| H0SNL4_9BRAD | - | - | - | - | - | - | - | - | - | - | - | - | - | - | - | - | - | - | - | - | - | - | - | - | - | - | - | - | - | - | - | - | - | - | - | - | - | - | - | - | - | - | - | - | - | - | - | - | - | - | - | - | - | - | - | - | - | - | - | - | - | -224 |
| H0S4P6_9BRAD | - | - | - | - | - | - | - | - | - | - | - | - | - | - | - | - | - | - | - | - | - | - | - | - | - | - | - | - | - | - | - | - | - | - | - | - | - | - | - | - | - | - | - | - | - | - | - | - | - | - | - | - | - | - | - | - | - | - | - | - | - | -224 |
| A5EMK2_BRASB | - | - | - | - | - | - | - | - | - | - | - | - | - | - | - | - | - | - | - | - | - | - | - | - | - | - | - | - | - | - | - | - | - | - | - | - | - | - | - | - | - | - | - | - | - | - | - | - | - | - | - | - | - | - | - | - | - | - | - | - | - | -217 |
| M4ZBJ4_9BRAD | - | - | - | - | - | - | - | - | - | - | - | - | - | - | - | - | - | - | - | - | - | - | - | - | - | - | - | - | - | - | - | - | - | - | - | - | - | - | - | - | - | - | - | - | - | - | - | - | - | - | - | - | - | - | - | - | - | - | - | - | - | -226 |
| Q07SP7_RHOP5 | - | - | - | - | - | - | - | - | - | - | - | - | - | - | - | - | - | - | - | - | - | - | - | - | - | - | - | - | - | - | - | - | - | - | - | - | - | - | - | - | - | - | - | - | - | - | - | - | - | - | - | - | - | - | - | - | - | - | - | - | - | -210 |
| B0JUQ7_MICAN | - | - | - | - | - | - | - | - | - | - | - | - | - | - | - | - | - | - | - | - | - | - | - | - | - | - | - | - | - | - | - | - | - | - | - | - | - | - | - | - | - | - | - | - | - | - | - | - | - | - | - | - | - | - | - | - | - | - | - | - | - | -191 |
| K9WJ87_9CYAN | - | - | - | - | - | D | M | T | W | T | - | - | - | - | - | - | - | - | - | - | - | - | - | - | - | - | - | - | - | - | - | - | - | - | - | - | - | - | - | - | - | - | - | - | - | - | - | - | - | - | - | - | - | - | - | - | - | - | - | - | - | -193 |
| G6FUE0_9CYAN | - | P | G | I | I | E | L | S | W | N | Q | I | E | A | N | - | - | - | - | - | - | - | - | - | - | - | - | - | - | - | - | - | - | - | - | - | - | - | - | - | - | - | - | - | - | - | - | - | - | - | - | - | - | - | - | - | - | - | - | - | - | -214 |
| K9PHG5_9CYAN | - | - | - | - | - | - | - | - | - | - | - | - | - | - | - | - | - | - | - | - | - | - | - | - | - | - | - | - | - | - | - | - | - | - | - | - | - | - | - | - | - | - | - | - | - | - | - | - | - | - | - | - | - | - | - | - | - | - | - | - | - | -196 |
| Q8YXX3_NOSS1 | - | - | - | - | - | - | - | - | - | - | - | - | - | - | - | - | - | - | - | - | - | - | - | - | - | - | - | - | - | - | - | - | - | - | - | - | - | - | - | - | - | - | - | - | - | - | - | - | - | - | - | - | - | - | - | - | - | - | - | - | - | -211 |
| K9TN70_9CYAN | - | - | - | - | - | - | - | - | - | - | - | - | - | - | - | - | - | - | - | - | - | - | - | - | - | - | - | - | - | - | - | - | - | - | - | - | - | - | - | - | - | - | - | - | - | - | - | - | - | - | - | - | - | - | - | - | - | - | - | - | - | -214 |
| Q05QW3_9SYNE | - | - | - | - | - | - | - | - | - | - | - | - | - | - | - | - | - | - | - | - | - | - | - | - | - | - | - | - | - | - | - | - | - | - | - | - | - | - | - | - | - | - | - | - | - | - | - | - | - | - | - | - | - | - | - | - | - | - | - | - | - | -238 |
| Q05QW2_9SYNE | - | - | - | - | - | - | - | - | - | - | - | - | - | - | - | - | - | - | - | - | - | - | - | - | - | - | - | - | - | - | - | - | - | - | - | - | - | - | - | - | - | - | - | - | - | - | - | - | - | - | - | - | - | - | - | - | - | - | - | - | - | -205 |
| Q05QW4_9SYNE | - | - | - | - | - | - | - | - | - | - | - | - | - | - | - | - | - | - | - | - | - | - | - | - | - | - | - | - | - | - | - | - | - | - | - | - | - | - | - | - | - | - | - | - | - | - | - | - | - | - | - | - | - | - | - | - | - | - | - | - | - | -216 |
| E0UGX6_CYAP2 | - | - | - | - | - | - | - | - | - | - | - | - | - | - | - | - | - | - | - | - | - | - | - | - | - | - | - | - | - | - | - | - | - | - | - | - | - | - | - | - | - | - | - | - | - | - | - | - | - | - | - | - | - | - | - | - | - | - | - | - | - | -197 |
| K7WKF4_9NOST | - | W | - | - | - | - | - | - | - | - | - | - | - | - | - | - | - | - | - | - | - | - | - | - | - | - | - | - | - | - | - | - | - | - | - | - | - | - | - | - | - | - | - | - | - | - | - | - | - | - | - | - | - | - | - | - | - | - | - | - | - | -204 |
| E0UGY5_CYAP2 | - | - | - | - | - | - | - | - | - | - | - | - | - | - | - | - | - | - | - | - | - | - | - | - | - | - | - | - | - | - | - | - | - | - | - | - | - | - | - | - | - | - | - | - | - | - | - | - | - | - | - | - | - | - | - | - | - | - | - | - | - | -199 |
| A8LCE5_FRASN | - | L | E | W | H | D | W | D | G | L | K | P | A | L | - | - | - | - | - | - | - | - | - | - | - | - | - | - | - | - | - | - | - | - | - | - | - | - | - | - | - | - | - | - | - | - | - | - | - | - | - | - | - | - | - | - | - | - | - | - | - | -213 |
| D3CT47_9ACTO | - | L | V | W | R | Q | W | A | S | L | R | P | - | - | - | - | - | - | - | - | - | - | - | - | - | - | - | - | - | - | - | - | - | - | - | - | - | - | - | - | - | - | - | - | - | - | - | - | - | - | - | - | - | - | - | - | - | - | - | - | - | -215 |
| Q2J4J6_FRASC | - | E | R | W | Y | A | W | D | G | I | L | P | - | - | - | - | - | - | - | - | - | - | - | - | - | - | - | - | - | - | - | - | - | - | - | - | - | - | - | - | - | - | - | - | - | - | - | - | - | - | - | - | - | - | - | - | - | - | - | - | - | -235 |
| Q0RAZ2_FRAAA | - | V | R | W | Y | A | W | S | Q | V | V | - | - | - | - | - | - | - | - | - | - | - | - | - | - | - | - | - | - | - | - | - | - | - | - | - | - | - | - | - | - | - | - | - | - | - | - | - | - | - | - | - | - | - | - | - | - | - | - | - | - | -221 |
| E3J3F6_FRASU | - | T | A | W | Y | R | W | D | D | L | R | P | - | - | - | - | - | - | - | - | - | - | - | - | - | - | - | - | - | - | - | - | - | - | - | - | - | - | - | - | - | - | - | - | - | - | - | - | - | - | - | - | - | - | - | - | - | - | - | - | - | -217 |
| ZP_09168240 | - | V | R | W | Y | R | W | D | E | L | R | P | - | - | - | - | - | - | - | - | - | - | - | - | - | - | - | - | - | - | - | - | - | - | - | - | - | - | - | - | - | - | - | - | - | - | - | - | - | - | - | - | - | - | - | - | - | - | - | - | - | -228 |
| C7MDV9_BRAFD | - | - | - | - | - | - | - | - | - | - | - | - | - | - | - | - | - | - | - | - | - | - | - | - | - | - | - | - | - | - | - | - | - | - | - | - | - | - | - | - | - | - | - | - | - | - | - | - | - | - | - | - | - | - | - | - | - | - | - | - | - | -172 |
| ZP_10094989 | - | - | - | - | - | - | - | - | - | - | - | - | - | - | - | - | - | - | - | - | - | - | - | - | - | - | - | - | - | - | - | - | - | - | - | - | - | - | - | - | - | - | - | - | - | - | - | - | - | - | - | - | - | - | - | - | - | - | - | - | - | -172 |
| A4X268_SALTO | - | - | - | - | - | - | - | - | - | - | - | - | - | - | - | - | - | - | - | - | - | - | - | - | - | - | - | - | - | - | - | - | - | - | - | - | - | - | - | - | - | - | - | - | - | - | - | - | - | - | - | - | - | - | - | - | - | - | - | - | - | -231 |
| WP_043162213 | - | - | - | - | - | - | - | - | - | - | - | - | - | - | - | - | - | - | - | - | - | - | - | - | - | - | - | - | - | - | - | - | - | - | - | - | - | - | - | - | - | - | - | - | - | - | - | - | - | - | - | - | - | - | - | - | - | - | - | - | - | -231 |
| Q0BRV8_GRABC | - | - | - | - | - | - | - | - | - | - | - | - | - | - | - | - | - | - | - | - | - | - | - | - | - | - | - | - | - | - | - | - | - | - | - | - | - | - | - | - | - | - | - | - | - | - | - | - | - | - | - | - | - | - | - | - | - | - | - | - | - | -199 |
| A9B2P0_HERA2 | - | - | - | - | - | - | - | - | - | - | - | - | - | - | - | - | - | - | - | - | - | - | - | - | - | - | - | - | - | - | - | - | - | - | - | - | - | - | - | - | - | - | - | - | - | - | - | - | - | - | - | - | - | - | - | - | - | - | - | - | - | -190 |
| A9B3U8_HERA2 | - | - | - | - | - | - | - | - | - | - | - | - | - | - | - | - | - | - | - | - | - | - | - | - | - | - | - | - | - | - | - | - | - | - | - | - | - | - | - | - | - | - | - | - | - | - | - | - | - | - | - | - | - | - | - | - | - | - | - | - | - | -192 |
| A9B2A3_HERA2 | - | - | - | - | - | - | - | - | - | - | - | - | - | - | - | - | - | - | - | - | - | - | - | - | - | - | - | - | - | - | - | - | - | - | - | - | - | - | - | - | - | - | - | - | - | - | - | - | - | - | - | - | - | - | - | - | - | - | - | - | - | -216 |
| D6ZC85_SEGRD | - | - | - | - | - | - | - | - | - | - | - | - | - | - | - | - | - | - | - | - | - | - | - | - | - | - | - | - | - | - | - | - | - | - | - | - | - | - | - | - | - | - | - | - | - | - | - | - | - | - | - | - | - | - | - | - | - | - | - | - | - | -238 |
| E5XTT9_9ACTO | - | - | - | - | - | - | - | - | - | - | - | - | - | - | - | - | - | - | - | - | - | - | - | - | - | - | - | - | - | - | - | - | - | - | - | - | - | - | - | - | - | - | - | - | - | - | - | - | - | - | - | - | - | - | - | - | - | - | - | - | - | -238 |
| E5XTU0_9ACTO | - | - | - | - | - | - | - | - | - | - | - | - | - | - | - | - | - | - | - | - | - | - | - | - | - | - | - | - | - | - | - | - | - | - | - | - | - | - | - | - | - | - | - | - | - | - | - | - | - | - | - | - | - | - | - | - | - | - | - | - | - | -245 |
| D6B0I6_9ACTO | - | - | - | - | - | - | - | - | - | - | - | - | - | - | - | - | - | - | - | - | - | - | - | - | - | - | - | - | - | - | - | - | - | - | - | - | - | - | - | - | - | - | - | - | - | - | - | - | - | - | - | - | - | - | - | - | - | - | - | - | - | -207 |
| D9X719_STRVR | - | - | - | - | - | - | - | - | - | - | - | - | - | - | - | - | - | - | - | - | - | - | - | - | - | - | - | - | - | - | - | - | - | - | - | - | - | - | - | - | - | - | - | - | - | - | - | - | - | - | - | - | - | - | - | - | - | - | - | - | - | -211 |
| H6L010_SAPGL | - | - | - | - | - | - | - | - | - | - | - | - | - | - | - | - | - | - | - | - | - | - | - | - | - | - | - | - | - | - | - | - | - | - | - | - | - | - | - | - | - | - | - | - | - | - | - | - | - | - | - | - | - | - | - | - | - | - | - | - | - | -191 |
| K1ZP83_9BACT | - | G | S | D | A | V | D | D | G | T | F | W | M | - | - | - | - | - | - | - | - | - | - | - | - | - | - | - | - | - | - | - | - | - | - | - | - | - | - | - | - | - | - | - | - | - | - | - | - | - | - | - | - | - | - | - | - | - | - | - | - | -232 |
| ADGB_HUMAN | - | - | - | - | - | - | - | - | - | - | - | - | - | - | - | - | - | - | - | - | - | - | - | - | - | - | - | - | - | - | - | - | - | - | - | - | - | - | - | - | - | - | - | - | - | - | - | - | - | - | - | - | - | - | - | - | - | - | - | - | - | -250 |
| J3QMP3_MOUSE | - | - | - | - | - | - | - | - | - | - | - | - | - | - | - | - | - | - | - | - | - | - | - | - | - | - | - | - | - | - | - | - | - | - | - | - | - | - | - | - | - | - | - | - | - | - | - | - | - | - | - | - | - | - | - | - | - | - | - | - | - | -250 |
| H2XR46_CIOIN | - | - | - | - | - | - | - | - | - | - | - | - | - | - | - | - | - | - | - | - | - | - | - | - | - | - | - | - | - | - | - | - | - | - | - | - | - | - | - | - | - | - | - | - | - | - | - | - | - | - | - | - | - | - | - | - | - | - | - | - | - | -257 |
| CAN7_MOUSE | - | - | - | R | W | K | G | R | Y | S | E | N | D | V | K | N | W | T | P | E | L | Q | K | Y | L | N | - | - | - | - | - | - | - | - | - | F | D | P | R | T | A | Q | K | I | D | N | G | I | F | W | I | S | - | - | - | - | - | - | - | - | - | -263 |
| CAN7_HUMAN | - | - | - | R | W | K | G | R | Y | S | E | N | D | V | K | N | W | T | P | E | L | Q | K | Y | L | N | - | - | - | - | - | - | - | - | - | F | D | P | R | T | A | Q | K | I | D | N | G | I | F | W | I | S | - | - | - | - | - | - | - | - | - | -263 |
| E1BY84_CHICK | - | - | - | R | W | K | G | R | Y | S | E | N | D | T | R | S | W | T | P | D | L | Q | K | Y | L | N | - | - | - | - | - | - | - | - | - | F | D | P | R | T | A | Q | K | I | D | N | G | I | F | W | I | A | - | - | - | - | - | - | - | - | - | -263 |
| H9GX91_DANRE | - | - | - | R | W | K | G | S | Y | S | E | R | D | E | K | N | W | T | P | D | L | L | K | Y | L | N | - | - | - | - | - | - | - | - | - | F | D | P | K | T | A | Q | K | F | D | N | G | V | F | W | I | I | - | - | - | - | - | - | - | - | - | -263 |
| F6SGI6_CIOIN | - | - | - | R | W | K | G | N | Y | S | A | H | D | D | A | H | W | T | P | E | L | R | K | A | L | N | - | - | - | - | - | - | - | - | - | Y | D | N | K | Y | A | Q | E | F | D | N | G | V | F | W | I | - | - | - | - | - | - | - | - | - | - | -260 |
| W4Y195_STRPU | - | - | - | R | W | K | G | R | F | S | E | E | D | T | T | N | W | T | P | E | L | Q | K | A | L | N | - | - | - | - | - | - | - | - | - | F | D | P | K | N | A | Q | E | F | D | N | G | V | F | W | I | - | - | - | - | - | - | - | - | - | - | -236 |
| R7V270_CAPTE | - | - | - | R | W | K | G | N | F | S | D | F | D | T | V | H | W | T | P | E | M | E | K | A | L | S | - | - | - | - | - | - | - | - | - | Y | N | R | K | V | A | V | D | N | D | N | G | I | F | W | I | - | - | - | - | - | - | - | - | - | - | -261 |
| G4LXK3_SCHMA | - | - | - | - | - | - | - | - | - | - | - | - | - | - | - | - | - | - | - | - | - | - | - | - | - | - | - | - | - | - | - | - | - | - | - | - | - | - | - | - | - | - | - | - | - | - | - | - | - | - | - | - | - | - | - | - | - | - | - | - | - | -221 |
| T2M2K0_HYDVU | - | - | - | R | W | K | G | N | Y | S | D | S | D | T | V | N | W | T | L | S | L | E | N | A | L | G | - | - | - | - | - | - | - | - | - | Y | N | R | E | K | A | A | Q | N | D | N | G | V | F | W | I | N | - | - | - | - | - | - | - | - | - | -261 |
| B3RQK1_TRIAD | - | - | - | R | W | K | G | K | Y | S | E | - | - | - | - | - | - | - | - | - | - | - | - | - | - | - | - | - | - | - | - | - | - | - | - | - | - | - | - | - | - | - | - | - | - | - | - | - | - | - | - | - | - | - | - | - | - | - | - | - | - | -230 |
| I1CS59_RHIO9 | - | - | - | R | W | R | G | P | Y | S | H | L | D | S | N | I | W | T | S | E | L | M | E | A | L | C | - | - | - | - | - | - | - | - | - | F | D | P | D | A | A | E | K | N | D | D | G | I | F | W | I | - | - | - | - | - | - | - | - | - | - | -258 |
| I1GER3_AMPQE | - | - | - | R | W | K | G | S | F | S | E | R | D | A | T | N | W | T | P | E | L | K | R | A | L | N | - | - | - | - | - | - | - | - | - | Y | D | Q | S | L | A | L | Q | V | D | N | G | V | F | W | I | - | - | - | - | - | - | - | - | - | - | -258 |
| Q22143_CAEEL | - | - | - | R | W | K | G | N | F | S | D | K | D | K | V | N | W | T | A | K | M | K | N | A | L | A | - | - | - | - | - | - | - | - | - | F | D | P | E | V | A | A | E | K | D | D | G | I | F | W | I | D | - | - | - | - | - | - | - | - | - | -262 |
| L1JZN7_GUITH | - | - | - | R | W | R | G | E | F | S | - | - | - | - | - | - | - | - | - | - | - | - | - | - | - | - | - | - | - | - | - | - | - | - | - | - | - | - | - | - | - | - | - | - | - | - | - | - | - | - | - | - | - | - | - | - | - | - | - | - | - | -232 |
| D0NT34_PHYIT | - | - | - | S | W | K | G | P | Y | S | - | - | - | - | - | - | - | - | - | - | - | - | - | - | - | - | - | - | - | - | - | - | - | - | - | - | - | - | - | - | - | - | - | - | - | - | - | - | - | - | - | - | - | - | - | - | - | - | - | - | - | -250 |
| A0A064B913_ASPOZ | - | - | - | - | - | - | A | D | T | A | P | G | D | - | - | N | G | S | L | S | A | S | Q | D | L | P | - | - | - | - | - | - | - | - | - | H | N | P | - | - | - | P | S | F | E | P | G | T | F | W | M | - | - | - | - | - | - | - | - | - | - | -259 |
| A8N4X4_COPC7 | - | - | - | T | E | D | G | K | G | V | D | G | V | N | R | M | I | N | V | L | W | S | D | A | L | N | - | - | - | - | - | - | - | - | - | M | F | D | G | I | Y | L | S | W | - | - | - | - | - | - | - | - | - | - | - | - | - | - | - | - | - | -277 |
| C0H5K1_PLAF7 | - | - | - | R | W | K | G | R | Y | S | H | N | D | Y | E | T | W | T | K | E | L | Q | K | K | L | N | - | - | - | - | - | - | - | - | - | Y | S | L | E | K | A | I | S | K | D | D | G | C | F | W | I | - | - | - | - | - | - | - | - | - | - | -431 |
| K1REZ8_CRAGI | - | Y | T | I | W | K | G | P | W | N | Q | N | G | S | E | W | A | S | L | P | P | E | H | D | H | I | L | S | S | - | - | - | - | - | - | - | - | - | - | - | - | - | - | V | S | D | Q | E | F | I | M | A | - | - | - | - | - | - | - | - | - | -245 |
| F4NTM9_BATDJ | - | - | - | - | - | Q | S | S | S | V | S | H | T | S | P | D | W | P | S | I | L | A | K | E | R | K | Q | L | N | - | - | - | - | - | - | - | - | - | - | - | L | S | I | G | Q | D | G | Q | F | - | - | - | - | - | - | - | - | - | - | - | - | -271 |
| Q6F6K5_CAEEL | - | E | I | R | W | Q | V | P | I | G | K | N | I | L | I | N | - | - | - | - | - | - | - | - | - | - | - | - | - | - | - | - | - | - | - | - | - | - | - | - | - | - | - | - | - | - | - | - | - | - | - | - | - | - | - | - | - | - | - | - | - | -194 |
| A8HZK8_CHLRE | - | P | Y | N | V | L | E | A | R | A | V | N | D | N | I | R | L | G | S | G | E | W | D | Y | P | A | M | A | G | K | - | - | - | - | - | L | D | S | F | A | D | S | W | E | D | E | A | T | F | W | M | S | - | - | - | - | - | - | - | - | - | -253 |
| A8J6T6_CHLRE | - | - | - | E | W | K | G | D | W | S | D | A | S | P | Q | W | D | T | Y | A | G | V | A | E | A | L | E | F | R | R | E | G | A | A | G | G | G | G | A | G | T | S | G | R | R | D | G | S | F | W | M | - | - | - | - | - | - | - | - | - | - | -321 |
| Z9JR51_9MICO | - | - | - | - | - | A | G | S | P | P | E | V | R | V | D | P | S | D | - | - | - | - | - | - | - | - | - | - | - | - | - | - | - | - | - | - | - | - | - | - | - | - | - | - | - | - | - | - | - | - | - | - | - | - | - | - | - | - | - | - | - | -215 |
| F7ZZH9_CELGA | - | - | - | - | - | - | G | N | S | A | D | G | - | - | - | - | - | - | - | - | - | - | - | - | - | - | - | - | - | - | - | - | - | - | - | - | - | - | - | - | - | - | - | - | - | - | - | - | - | - | - | - | - | - | - | - | - | - | - | - | - | -202 |
| Q7NDU2_GLOVI | - | - | - | - | - | - | - | - | - | - | - | - | - | - | - | - | - | - | - | - | - | - | - | - | - | - | - | - | - | - | - | - | - | - | - | - | - | - | - | - | - | - | - | - | - | - | - | - | - | - | - | - | - | - | - | - | - | - | - | - | - | -172 |
| I6AT95_9BACT | - | - | - | - | - | - | - | - | - | - | - | - | - | - | - | - | - | - | - | - | - | - | - | - | - | - | - | - | - | - | - | - | - | - | - | - | - | - | - | - | - | - | - | - | - | - | - | - | - | - | - | - | - | - | - | - | - | - | - | - | - | -198 |
| C7MGJ8_BRAFD | - | - | - | - | - | - | - | - | - | - | - | - | - | - | - | - | - | - | - | - | - | - | - | - | - | - | - | - | - | - | - | - | - | - | - | - | - | - | - | - | - | - | - | - | - | - | - | - | - | - | - | - | - | - | - | - | - | - | - | - | - | -197 |
| D0WP73_9ACTO | - | - | - | A | H | A | Y | M | V | V | D | A | D | Q | N | G | V | T | L | A | N | P | - | - | - | - | - | - | - | - | - | - | - | - | - | - | - | - | - | - | - | - | - | - | - | - | - | - | - | - | - | - | - | - | - | - | - | - | - | - | - | -212 |
| B0C8L1_ACAM1 | - | Y | D | K | K | L | G | K | L | V | S | K | T | D | E | V | N | G | E | F | W | L | E | L | S | D | V | T | K | H | - | - | - | - | - | - | - | F | K | S | I | H | T | T | I | D | D | R | - | - | - | - | - | - | - | - | - | - | - | - | - | -225 |
| B3E5A8_GEOLS | - | - | - | - | - | - | - | - | - | - | - | - | - | - | - | - | - | - | - | - | - | - | - | - | - | - | - | - | - | - | - | - | - | - | - | - | - | - | - | - | - | - | - | - | - | - | - | - | - | - | - | - | - | - | - | - | - | - | - | - | - | -189 |
| D4YQ94_9MICO | - | - | - | - | - | S | A | E | G | E | P | R | R | T | D | L | F | L | S | A | A | Q | F | K | R | A | F | L | - | - | - | - | - | - | - | - | - | - | - | - | - | - | - | - | - | - | - | - | - | - | - | - | - | - | - | - | - | - | - | - | - | -221 |
| PAPA_CARPA | - | - | - | - | - | - | T | G | W | G | E | N | G | Y | I | R | I | K | R | G | T | G | N | S | Y | G | V | C | G | - | - | - | - | - | - | - | - | - | - | - | - | - | - | L | Y | T | S | S | F | Y | P | V | K | N | - | - | - | - | - | - | - | -212 |
| RIM13_YEAST | - | S | Y | E | Q | Y | L | K | N | F | K | Q | L | Y | L | N | W | N | Q | E | K | L | F | K | R | S | Q | V | L | H | F | R | Y | D | T | S | R | Y | N | K | F | S | I | V | A | D | K | P | L | F | H | L | V | N | N | S | K | V | T | E | T | -278 |

| I |  |  |  |  |  |  |  |  |  |  |  |  |  |  |  |  |  |  |  |  |  |  |  |  |  |  |  |  |  |  |  |  |  |  |  |  |  |  |  |  |  |  |  |  |  |  |  |  |  |  |  |  |  |  |  |  |  |  |  |  |  |  |
| --- | --- | --- | --- | --- | --- | --- | --- | --- | --- | --- | --- | --- | --- | --- | --- | --- | --- | --- | --- | --- | --- | --- | --- | --- | --- | --- | --- | --- | --- | --- | --- | --- | --- | --- | --- | --- | --- | --- | --- | --- | --- | --- | --- | --- | --- | --- | --- | --- | --- | --- | --- | --- | --- | --- | --- | --- | --- | --- | --- | --- | --- | --- |
| Q9N4B1_CAEEL | - | - | - | - | - | - | - | - | - | - | - | - |  |  |  |  |  |  |  |  |  |  |  |  |  |  |  |  |  |  |  |  |  |  |  |  |  |  |  |  |  |  |  |  |  |  |  |  |  |  |  |  |  |  |  |  |  |  |  |  |  | -254 |
| Q8IAA8_CAEEL | - | - | - | - | - | - | - | - | - | - | - | - |  |  |  |  |  |  |  |  |  |  |  |  |  |  |  |  |  |  |  |  |  |  |  |  |  |  |  |  |  |  |  |  |  |  |  |  |  |  |  |  |  |  |  |  |  |  |  |  |  | -254 |
| CAN_CAEEL | - | - | - | - | - | - | - | - | - | - | - | - |  |  |  |  |  |  |  |  |  |  |  |  |  |  |  |  |  |  |  |  |  |  |  |  |  |  |  |  |  |  |  |  |  |  |  |  |  |  |  |  |  |  |  |  |  |  |  |  |  | -254 |
| Q9U2B2_CAEEL | - | - | - | - | - | - | - | - | - | - | - | - |  |  |  |  |  |  |  |  |  |  |  |  |  |  |  |  |  |  |  |  |  |  |  |  |  |  |  |  |  |  |  |  |  |  |  |  |  |  |  |  |  |  |  |  |  |  |  |  |  | -255 |
| Q9N4B2_CAEEL | - | - | - | - | - | - | - | - | - | - | - | - |  |  |  |  |  |  |  |  |  |  |  |  |  |  |  |  |  |  |  |  |  |  |  |  |  |  |  |  |  |  |  |  |  |  |  |  |  |  |  |  |  |  |  |  |  |  |  |  |  | -253 |
| C3Y6P6_BRAFL | - | - | - | - | - | - | - | - | - | - | - | - |  |  |  |  |  |  |  |  |  |  |  |  |  |  |  |  |  |  |  |  |  |  |  |  |  |  |  |  |  |  |  |  |  |  |  |  |  |  |  |  |  |  |  |  |  |  |  |  |  | -271 |
| C3Y6N1_BRAFL | - | - | - | - | - | - | - | - | - | - | - | - |  |  |  |  |  |  |  |  |  |  |  |  |  |  |  |  |  |  |  |  |  |  |  |  |  |  |  |  |  |  |  |  |  |  |  |  |  |  |  |  |  |  |  |  |  |  |  |  |  | -231 |
| CANA_DROME | - | - | - | - | - | - | - | - | - | - | - | - |  |  |  |  |  |  |  |  |  |  |  |  |  |  |  |  |  |  |  |  |  |  |  |  |  |  |  |  |  |  |  |  |  |  |  |  |  |  |  |  |  |  |  |  |  |  |  |  |  | -258 |
| CANB_DROME | - | - | - | - | - | - | - | - | - | - | - | - |  |  |  |  |  |  |  |  |  |  |  |  |  |  |  |  |  |  |  |  |  |  |  |  |  |  |  |  |  |  |  |  |  |  |  |  |  |  |  |  |  |  |  |  |  |  |  |  |  | -258 |
| X1Y3H71_ANODA | - | - | - | - | - | - | - | - | - | - | - | - |  |  |  |  |  |  |  |  |  |  |  |  |  |  |  |  |  |  |  |  |  |  |  |  |  |  |  |  |  |  |  |  |  |  |  |  |  |  |  |  |  |  |  |  |  |  |  |  |  | -259 |
| Q5IW12_GECLA | - | - | - | - | - | - | - | - | - | - | - | - |  |  |  |  |  |  |  |  |  |  |  |  |  |  |  |  |  |  |  |  |  |  |  |  |  |  |  |  |  |  |  |  |  |  |  |  |  |  |  |  |  |  |  |  |  |  |  |  |  | -258 |
| K1Q056_CRAGI | - | - | - | - | - | - | - | - | - | - | - | - |  |  |  |  |  |  |  |  |  |  |  |  |  |  |  |  |  |  |  |  |  |  |  |  |  |  |  |  |  |  |  |  |  |  |  |  |  |  |  |  |  |  |  |  |  |  |  |  |  | -258 |
| G4V6C0_SCHMA | - | - | - | - | - | - | - | - | - | - | - | - |  |  |  |  |  |  |  |  |  |  |  |  |  |  |  |  |  |  |  |  |  |  |  |  |  |  |  |  |  |  |  |  |  |  |  |  |  |  |  |  |  |  |  |  |  |  |  |  |  | -234 |
| R7ULX9_CAPTE | - | - | - | - | - | - | - | - | - | - | - | - |  |  |  |  |  |  |  |  |  |  |  |  |  |  |  |  |  |  |  |  |  |  |  |  |  |  |  |  |  |  |  |  |  |  |  |  |  |  |  |  |  |  |  |  |  |  |  |  |  | -259 |
| W4Z5Q7_STRPU | - | R | V | D | M | C | H | L | S | A | E | A |  |  |  |  |  |  |  |  |  |  |  |  |  |  |  |  |  |  |  |  |  |  |  |  |  |  |  |  |  |  |  |  |  |  |  |  |  |  |  |  |  |  |  |  |  |  |  |  |  | -271 |
| R7VIB6_CAPTE | - | K | L | E | I | C | L | L | T | P | - | - |  |  |  |  |  |  |  |  |  |  |  |  |  |  |  |  |  |  |  |  |  |  |  |  |  |  |  |  |  |  |  |  |  |  |  |  |  |  |  |  |  |  |  |  |  |  |  |  |  | -276 |
| T2MJ45_HYDVU | - | R | V | E | V | C | M | L | S | P | D | S |  |  |  |  |  |  |  |  |  |  |  |  |  |  |  |  |  |  |  |  |  |  |  |  |  |  |  |  |  |  |  |  |  |  |  |  |  |  |  |  |  |  |  |  |  |  |  |  |  | -270 |
| R7UAB6_CAPTE | - | K | V | E | I | C | N | L | G | P | D | S |  |  |  |  |  |  |  |  |  |  |  |  |  |  |  |  |  |  |  |  |  |  |  |  |  |  |  |  |  |  |  |  |  |  |  |  |  |  |  |  |  |  |  |  |  |  |  |  |  | -278 |
| C3ZKN8_BRAFL | - | R | L | E | I | C | N | L | P | P | D | A |  |  |  |  |  |  |  |  |  |  |  |  |  |  |  |  |  |  |  |  |  |  |  |  |  |  |  |  |  |  |  |  |  |  |  |  |  |  |  |  |  |  |  |  |  |  |  |  |  | -280 |
| B3RJS5_TRIAD | - | T | L | E | I | C | M | L | S | P | D | S |  |  |  |  |  |  |  |  |  |  |  |  |  |  |  |  |  |  |  |  |  |  |  |  |  |  |  |  |  |  |  |  |  |  |  |  |  |  |  |  |  |  |  |  |  |  |  |  |  | -275 |
| H2YVF0_CIOSA | - | - | - | - | - | - | - | - | - | - | - | - |  |  |  |  |  |  |  |  |  |  |  |  |  |  |  |  |  |  |  |  |  |  |  |  |  |  |  |  |  |  |  |  |  |  |  |  |  |  |  |  |  |  |  |  |  |  |  |  |  | -250 |
| G4VAG2_SCHMA | - | - | - | - | - | - | - | - | - | - | - | - |  |  |  |  |  |  |  |  |  |  |  |  |  |  |  |  |  |  |  |  |  |  |  |  |  |  |  |  |  |  |  |  |  |  |  |  |  |  |  |  |  |  |  |  |  |  |  |  |  | -257 |
| Smp_157500 | - | - | - | - | - | - | - | - | - | - | - | - |  |  |  |  |  |  |  |  |  |  |  |  |  |  |  |  |  |  |  |  |  |  |  |  |  |  |  |  |  |  |  |  |  |  |  |  |  |  |  |  |  |  |  |  |  |  |  |  |  | -254 |
| Q7SYA9_DANRE | - | - | - | - | - | - | - | - | - | - | - | - |  |  |  |  |  |  |  |  |  |  |  |  |  |  |  |  |  |  |  |  |  |  |  |  |  |  |  |  |  |  |  |  |  |  |  |  |  |  |  |  |  |  |  |  |  |  |  |  |  | -253 |
| A5PMP1_DANRE | - | - | - | - | - | - | - | - | - | - | - | - |  |  |  |  |  |  |  |  |  |  |  |  |  |  |  |  |  |  |  |  |  |  |  |  |  |  |  |  |  |  |  |  |  |  |  |  |  |  |  |  |  |  |  |  |  |  |  |  |  | -253 |
| CANX_CHICK | - | - | - | - | - | - | - | - | - | - | - | - |  |  |  |  |  |  |  |  |  |  |  |  |  |  |  |  |  |  |  |  |  |  |  |  |  |  |  |  |  |  |  |  |  |  |  |  |  |  |  |  |  |  |  |  |  |  |  |  |  | -253 |
| CAN1_HUMAN | - | - | - | - | - | - | - | - | - | - | - | - |  |  |  |  |  |  |  |  |  |  |  |  |  |  |  |  |  |  |  |  |  |  |  |  |  |  |  |  |  |  |  |  |  |  |  |  |  |  |  |  |  |  |  |  |  |  |  |  |  | -253 |
| CAN1_MOUSE | - | - | - | - | - | - | - | - | - | - | - | - |  |  |  |  |  |  |  |  |  |  |  |  |  |  |  |  |  |  |  |  |  |  |  |  |  |  |  |  |  |  |  |  |  |  |  |  |  |  |  |  |  |  |  |  |  |  |  |  |  | -253 |
| O42133_CHICK | - | - | - | - | - | - | - | - | - | - | - | - |  |  |  |  |  |  |  |  |  |  |  |  |  |  |  |  |  |  |  |  |  |  |  |  |  |  |  |  |  |  |  |  |  |  |  |  |  |  |  |  |  |  |  |  |  |  |  |  |  | -253 |
| Q7ZUR1_DANRE | - | - | - | - | - | - | - | - | - | - | - | - |  |  |  |  |  |  |  |  |  |  |  |  |  |  |  |  |  |  |  |  |  |  |  |  |  |  |  |  |  |  |  |  |  |  |  |  |  |  |  |  |  |  |  |  |  |  |  |  |  | -253 |
| E9QFN0_DANRE | - | - | - | - | - | - | - | - | - | - | - | - |  |  |  |  |  |  |  |  |  |  |  |  |  |  |  |  |  |  |  |  |  |  |  |  |  |  |  |  |  |  |  |  |  |  |  |  |  |  |  |  |  |  |  |  |  |  |  |  |  | -253 |
| CAN2_HUMAN | - | - | - | - | - | - | - | - | - | - | - | - |  |  |  |  |  |  |  |  |  |  |  |  |  |  |  |  |  |  |  |  |  |  |  |  |  |  |  |  |  |  |  |  |  |  |  |  |  |  |  |  |  |  |  |  |  |  |  |  |  | -253 |
| CAN2_MOUSE | - | - | - | - | - | - | - | - | - | - | - | - |  |  |  |  |  |  |  |  |  |  |  |  |  |  |  |  |  |  |  |  |  |  |  |  |  |  |  |  |  |  |  |  |  |  |  |  |  |  |  |  |  |  |  |  |  |  |  |  |  | -253 |
| CAN2_CHICK | - | - | - | - | - | - | - | - | - | - | - | - |  |  |  |  |  |  |  |  |  |  |  |  |  |  |  |  |  |  |  |  |  |  |  |  |  |  |  |  |  |  |  |  |  |  |  |  |  |  |  |  |  |  |  |  |  |  |  |  |  | -253 |
| Q6DFZ8_DANRE | - | - | - | - | - | - | - | - | - | - | - | - |  |  |  |  |  |  |  |  |  |  |  |  |  |  |  |  |  |  |  |  |  |  |  |  |  |  |  |  |  |  |  |  |  |  |  |  |  |  |  |  |  |  |  |  |  |  |  |  |  | -253 |
| Q504F5_DANRE | - | - | - | - | - | - | - | - | - | - | - | - |  |  |  |  |  |  |  |  |  |  |  |  |  |  |  |  |  |  |  |  |  |  |  |  |  |  |  |  |  |  |  |  |  |  |  |  |  |  |  |  |  |  |  |  |  |  |  |  |  | -251 |
| Q5BLH5_DANRE | - | - | - | - | - | - | - | - | - | - | - | - |  |  |  |  |  |  |  |  |  |  |  |  |  |  |  |  |  |  |  |  |  |  |  |  |  |  |  |  |  |  |  |  |  |  |  |  |  |  |  |  |  |  |  |  |  |  |  |  |  | -251 |
| Q4VBH3_DANRE | - | - | - | - | - | - | - | - | - | - | - | - |  |  |  |  |  |  |  |  |  |  |  |  |  |  |  |  |  |  |  |  |  |  |  |  |  |  |  |  |  |  |  |  |  |  |  |  |  |  |  |  |  |  |  |  |  |  |  |  |  | -253 |
| CAN8_RAT | - | - | - | - | - | - | - | - | - | - | - | - |  |  |  |  |  |  |  |  |  |  |  |  |  |  |  |  |  |  |  |  |  |  |  |  |  |  |  |  |  |  |  |  |  |  |  |  |  |  |  |  |  |  |  |  |  |  |  |  |  | -253 |
| CAN8_MOUSE | - | - | - | - | - | - | - | - | - | - | - | - |  |  |  |  |  |  |  |  |  |  |  |  |  |  |  |  |  |  |  |  |  |  |  |  |  |  |  |  |  |  |  |  |  |  |  |  |  |  |  |  |  |  |  |  |  |  |  |  |  | -253 |
| CAN8_HUMAN | - | - | - | - | - | - | - | - | - | - | - | - |  |  |  |  |  |  |  |  |  |  |  |  |  |  |  |  |  |  |  |  |  |  |  |  |  |  |  |  |  |  |  |  |  |  |  |  |  |  |  |  |  |  |  |  |  |  |  |  |  | -253 |
| XP_426117 | - | - | - | - | - | - | - | - | - | - | - | - |  |  |  |  |  |  |  |  |  |  |  |  |  |  |  |  |  |  |  |  |  |  |  |  |  |  |  |  |  |  |  |  |  |  |  |  |  |  |  |  |  |  |  |  |  |  |  |  |  | -253 |
| CAN9_MOUSE | - | - | - | - | - | - | - | - | - | - | - | - |  |  |  |  |  |  |  |  |  |  |  |  |  |  |  |  |  |  |  |  |  |  |  |  |  |  |  |  |  |  |  |  |  |  |  |  |  |  |  |  |  |  |  |  |  |  |  |  |  | -254 |
| CAN9_HUMAN | - | - | - | - | - | - | - | - | - | - | - | - |  |  |  |  |  |  |  |  |  |  |  |  |  |  |  |  |  |  |  |  |  |  |  |  |  |  |  |  |  |  |  |  |  |  |  |  |  |  |  |  |  |  |  |  |  |  |  |  |  | -254 |
| E1C6M3_CHICK | - | - | - | - | - | - | - | - | - | - | - | - |  |  |  |  |  |  |  |  |  |  |  |  |  |  |  |  |  |  |  |  |  |  |  |  |  |  |  |  |  |  |  |  |  |  |  |  |  |  |  |  |  |  |  |  |  |  |  |  |  | -253 |
| Q6DG44_DANRE | - | - | - | - | - | - | - | - | - | - | - | - |  |  |  |  |  |  |  |  |  |  |  |  |  |  |  |  |  |  |  |  |  |  |  |  |  |  |  |  |  |  |  |  |  |  |  |  |  |  |  |  |  |  |  |  |  |  |  |  |  | -253 |
| CAN3_HUMAN | - | - | - | - | - | - | - | - | - | - | - | - |  |  |  |  |  |  |  |  |  |  |  |  |  |  |  |  |  |  |  |  |  |  |  |  |  |  |  |  |  |  |  |  |  |  |  |  |  |  |  |  |  |  |  |  |  |  |  |  |  | -302 |
| CAN3_MOUSE | - | - | - | - | - | - | - | - | - | - | - | - |  |  |  |  |  |  |  |  |  |  |  |  |  |  |  |  |  |  |  |  |  |  |  |  |  |  |  |  |  |  |  |  |  |  |  |  |  |  |  |  |  |  |  |  |  |  |  |  |  | -302 |
| CAN3_CHICK | - | - | - | - | - | - | - | - | - | - | - | - |  |  |  |  |  |  |  |  |  |  |  |  |  |  |  |  |  |  |  |  |  |  |  |  |  |  |  |  |  |  |  |  |  |  |  |  |  |  |  |  |  |  |  |  |  |  |  |  |  | -301 |
| Q66HU4_DANRE | - | K | I | E | I | C | N | L | T | P | D | A |  |  |  |  |  |  |  |  |  |  |  |  |  |  |  |  |  |  |  |  |  |  |  |  |  |  |  |  |  |  |  |  |  |  |  |  |  |  |  |  |  |  |  |  |  |  |  |  |  | -277 |
| F1R632_DANRE | - | - | - | - | - | - | - | - | - | - | - | - |  |  |  |  |  |  |  |  |  |  |  |  |  |  |  |  |  |  |  |  |  |  |  |  |  |  |  |  |  |  |  |  |  |  |  |  |  |  |  |  |  |  |  |  |  |  |  |  |  | -253 |
| CAN11_HUMAN | - | - | - | - | - | - | - | - | - | - | - | - |  |  |  |  |  |  |  |  |  |  |  |  |  |  |  |  |  |  |  |  |  |  |  |  |  |  |  |  |  |  |  |  |  |  |  |  |  |  |  |  |  |  |  |  |  |  |  |  |  | -253 |
| CAN11_MOUSE | - | - | - | - | - | - | - | - | - | - | - | - |  |  |  |  |  |  |  |  |  |  |  |  |  |  |  |  |  |  |  |  |  |  |  |  |  |  |  |  |  |  |  |  |  |  |  |  |  |  |  |  |  |  |  |  |  |  |  |  |  | -253 |
| CAN12_MOUSE | - | - | - | - | - | - | - | - | - | - | - | - |  |  |  |  |  |  |  |  |  |  |  |  |  |  |  |  |  |  |  |  |  |  |  |  |  |  |  |  |  |  |  |  |  |  |  |  |  |  |  |  |  |  |  |  |  |  |  |  |  | -249 |
| CAN12_HUMAN | - | - | - | - | - | - | - | - | - | - | - | - |  |  |  |  |  |  |  |  |  |  |  |  |  |  |  |  |  |  |  |  |  |  |  |  |  |  |  |  |  |  |  |  |  |  |  |  |  |  |  |  |  |  |  |  |  |  |  |  |  | -249 |
| ENSSARP00000008135 | - | - | - | - | - | - | - | - | - | - | - | - |  |  |  |  |  |  |  |  |  |  |  |  |  |  |  |  |  |  |  |  |  |  |  |  |  |  |  |  |  |  |  |  |  |  |  |  |  |  |  |  |  |  |  |  |  |  |  |  |  | -220 |
| ENSSARP00000010553 | - | - | - | - | - | - | - | - | - | - | - | - |  |  |  |  |  |  |  |  |  |  |  |  |  |  |  |  |  |  |  |  |  |  |  |  |  |  |  |  |  |  |  |  |  |  |  |  |  |  |  |  |  |  |  |  |  |  |  |  |  | -261 |
| A7E2G3_DANRE | - | T | V | E | M | C | S | V | D | P | D | S |  |  |  |  |  |  |  |  |  |  |  |  |  |  |  |  |  |  |  |  |  |  |  |  |  |  |  |  |  |  |  |  |  |  |  |  |  |  |  |  |  |  |  |  |  |  |  |  |  | -275 |
| Q6NW69_DANRE | - | L | M | E | V | C | H | L | S | E | D | - |  |  |  |  |  |  |  |  |  |  |  |  |  |  |  |  |  |  |  |  |  |  |  |  |  |  |  |  |  |  |  |  |  |  |  |  |  |  |  |  |  |  |  |  |  |  |  |  |  | -271 |
| CAN13_HUMAN | - | - | - | - | - | - | - | - | - | - | - | - |  |  |  |  |  |  |  |  |  |  |  |  |  |  |  |  |  |  |  |  |  |  |  |  |  |  |  |  |  |  |  |  |  |  |  |  |  |  |  |  |  |  |  |  |  |  |  |  |  | -255 |
| CAN13_MOUSE | - | - | - | - | - | - | - | - | - | - | - | - |  |  |  |  |  |  |  |  |  |  |  |  |  |  |  |  |  |  |  |  |  |  |  |  |  |  |  |  |  |  |  |  |  |  |  |  |  |  |  |  |  |  |  |  |  |  |  |  |  | -232 |
| E1BRR5_CHICK | - | - | - | - | - | - | - | - | - | - | - | - |  |  |  |  |  |  |  |  |  |  |  |  |  |  |  |  |  |  |  |  |  |  |  |  |  |  |  |  |  |  |  |  |  |  |  |  |  |  |  |  |  |  |  |  |  |  |  |  |  | -250 |
| E7F0H3_DANRE | - | S | I | D | I | C | C | L | C | P | D | - |  |  |  |  |  |  |  |  |  |  |  |  |  |  |  |  |  |  |  |  |  |  |  |  |  |  |  |  |  |  |  |  |  |  |  |  |  |  |  |  |  |  |  |  |  |  |  |  |  | -272 |
| Q1RLS9_DANRE | - | E | M | D | I | C | C | - | - | - | - | - |  |  |  |  |  |  |  |  |  |  |  |  |  |  |  |  |  |  |  |  |  |  |  |  |  |  |  |  |  |  |  |  |  |  |  |  |  |  |  |  |  |  |  |  |  |  |  |  |  | -268 |
| F1Q9Z1_DANRE | - | N | M | D | I | C | C | N | C | P | D | - |  |  |  |  |  |  |  |  |  |  |  |  |  |  |  |  |  |  |  |  |  |  |  |  |  |  |  |  |  |  |  |  |  |  |  |  |  |  |  |  |  |  |  |  |  |  |  |  |  | -268 |
| A4QNV6_DANRE | - | E | V | D | I | C | C | F | T | P | D | - |  |  |  |  |  |  |  |  |  |  |  |  |  |  |  |  |  |  |  |  |  |  |  |  |  |  |  |  |  |  |  |  |  |  |  |  |  |  |  |  |  |  |  |  |  |  |  |  |  | -272 |
| D6W573_HUMAN | - | L | L | V | I | C | K | L | T | - | - | - |  |  |  |  |  |  |  |  |  |  |  |  |  |  |  |  |  |  |  |  |  |  |  |  |  |  |  |  |  |  |  |  |  |  |  |  |  |  |  |  |  |  |  |  |  |  |  |  |  | -266 |
| R7UBC6_CAPTE | - | L | V | S | V | C | H | L | R | P | D | - |  |  |  |  |  |  |  |  |  |  |  |  |  |  |  |  |  |  |  |  |  |  |  |  |  |  |  |  |  |  |  |  |  |  |  |  |  |  |  |  |  |  |  |  |  |  |  |  |  | -287 |
| K1RCZ2_CRAGI | - | - | - | - | - | - | - | - | - | - | - | - |  |  |  |  |  |  |  |  |  |  |  |  |  |  |  |  |  |  |  |  |  |  |  |  |  |  |  |  |  |  |  |  |  |  |  |  |  |  |  |  |  |  |  |  |  |  |  |  |  | -269 |
| Q5IW13_GECLA | - | - | - | - | - | - | - | - | - | - | - | - |  |  |  |  |  |  |  |  |  |  |  |  |  |  |  |  |  |  |  |  |  |  |  |  |  |  |  |  |  |  |  |  |  |  |  |  |  |  |  |  |  |  |  |  |  |  |  |  |  | -261 |
| R7UUX7_CAPTE | - | T | A | Y | F | C | H | L | Y | P | D | A |  |  |  |  |  |  |  |  |  |  |  |  |  |  |  |  |  |  |  |  |  |  |  |  |  |  |  |  |  |  |  |  |  |  |  |  |  |  |  |  |  |  |  |  |  |  |  |  |  | -275 |
| K1Q505_CRAGI | - | - | - | - | - | - | - | - | - | - | - | - |  |  |  |  |  |  |  |  |  |  |  |  |  |  |  |  |  |  |  |  |  |  |  |  |  |  |  |  |  |  |  |  |  |  |  |  |  |  |  |  |  |  |  |  |  |  |  |  |  | -260 |
| R7UD02_CAPTE | - | T | V | Q | I | C | H | M | T | P | D | - |  |  |  |  |  |  |  |  |  |  |  |  |  |  |  |  |  |  |  |  |  |  |  |  |  |  |  |  |  |  |  |  |  |  |  |  |  |  |  |  |  |  |  |  |  |  |  |  |  | -276 |
| CAN6_HUMAN | - | - | - | - | - | - | - | - | - | - | - | - |  |  |  |  |  |  |  |  |  |  |  |  |  |  |  |  |  |  |  |  |  |  |  |  |  |  |  |  |  |  |  |  |  |  |  |  |  |  |  |  |  |  |  |  |  |  |  |  |  | -276 |
| CAN6_MOUSE | - | - | - | - | - | - | - | - | - | - | - | - |  |  |  |  |  |  |  |  |  |  |  |  |  |  |  |  |  |  |  |  |  |  |  |  |  |  |  |  |  |  |  |  |  |  |  |  |  |  |  |  |  |  |  |  |  |  |  |  |  | -276 |
| E1C312_CHICK | - | - | - | - | - | - | - | - | - | - | - | - |  |  |  |  |  |  |  |  |  |  |  |  |  |  |  |  |  |  |  |  |  |  |  |  |  |  |  |  |  |  |  |  |  |  |  |  |  |  |  |  |  |  |  |  |  |  |  |  |  | -276 |
| E7FDN3_DANRE | - | - | - | - | - | - | - | - | - | - | - | - |  |  |  |  |  |  |  |  |  |  |  |  |  |  |  |  |  |  |  |  |  |  |  |  |  |  |  |  |  |  |  |  |  |  |  |  |  |  |  |  |  |  |  |  |  |  |  |  |  | -276 |
| CAN5_HUMAN | - | - | - | - | - | - | - | - | - | - | - | - |  |  |  |  |  |  |  |  |  |  |  |  |  |  |  |  |  |  |  |  |  |  |  |  |  |  |  |  |  |  |  |  |  |  |  |  |  |  |  |  |  |  |  |  |  |  |  |  |  | -272 |
| CAN5_MOUSE | - | - | - | - | - | - | - | - | - | - | - | - |  |  |  |  |  |  |  |  |  |  |  |  |  |  |  |  |  |  |  |  |  |  |  |  |  |  |  |  |  |  |  |  |  |  |  |  |  |  |  |  |  |  |  |  |  |  |  |  |  | -276 |
| E1C292_CHICK | - | - | - | - | - | - | - | - | - | - | - | - |  |  |  |  |  |  |  |  |  |  |  |  |  |  |  |  |  |  |  |  |  |  |  |  |  |  |  |  |  |  |  |  |  |  |  |  |  |  |  |  |  |  |  |  |  |  |  |  |  | -276 |
| A0JMG3_DANRE | - | - | - | - | - | - | - | - | - | - | - | - |  |  |  |  |  |  |  |  |  |  |  |  |  |  |  |  |  |  |  |  |  |  |  |  |  |  |  |  |  |  |  |  |  |  |  |  |  |  |  |  |  |  |  |  |  |  |  |  |  | -275 |
| E7F7F1_DANRE | - | - | - | - | - | - | - | - | - | - | - | - |  |  |  |  |  |  |  |  |  |  |  |  |  |  |  |  |  |  |  |  |  |  |  |  |  |  |  |  |  |  |  |  |  |  |  |  |  |  |  |  |  |  |  |  |  |  |  |  |  | -275 |
| XP_006824412 | - | - | - | - | - | - | - | - | - | - | - | - |  |  |  |  |  |  |  |  |  |  |  |  |  |  |  |  |  |  |  |  |  |  |  |  |  |  |  |  |  |  |  |  |  |  |  |  |  |  |  |  |  |  |  |  |  |  |  |  |  | -270 |
| W4YJN2_STRPU | - | - | - | - | - | - | - | - | - | - | - | - |  |  |  |  |  |  |  |  |  |  |  |  |  |  |  |  |  |  |  |  |  |  |  |  |  |  |  |  |  |  |  |  |  |  |  |  |  |  |  |  |  |  |  |  |  |  |  |  |  | -273 |
| C3YJZ3_BRAFL | - | - | - | - | - | - | - | - | - | - | - | - |  |  |  |  |  |  |  |  |  |  |  |  |  |  |  |  |  |  |  |  |  |  |  |  |  |  |  |  |  |  |  |  |  |  |  |  |  |  |  |  |  |  |  |  |  |  |  |  |  | -278 |
| T2MIT4_HYDVU | - | - | - | - | - | - | - | - | - | - | - | - |  |  |  |  |  |  |  |  |  |  |  |  |  |  |  |  |  |  |  |  |  |  |  |  |  |  |  |  |  |  |  |  |  |  |  |  |  |  |  |  |  |  |  |  |  |  |  |  |  | -276 |
| XP_002154722 | - | - | - | - | - | - | - | - | - | - | - | - |  |  |  |  |  |  |  |  |  |  |  |  |  |  |  |  |  |  |  |  |  |  |  |  |  |  |  |  |  |  |  |  |  |  |  |  |  |  |  |  |  |  |  |  |  |  |  |  |  | -240 |
| R7VLA7_CAPTE | - | - | - | - | - | - | - | - | - | - | - | - |  |  |  |  |  |  |  |  |  |  |  |  |  |  |  |  |  |  |  |  |  |  |  |  |  |  |  |  |  |  |  |  |  |  |  |  |  |  |  |  |  |  |  |  |  |  |  |  |  | -276 |
| K1R1C6_CRAGI | - | - | - | - | - | - | - | - | - | - | - | - |  |  |  |  |  |  |  |  |  |  |  |  |  |  |  |  |  |  |  |  |  |  |  |  |  |  |  |  |  |  |  |  |  |  |  |  |  |  |  |  |  |  |  |  |  |  |  |  |  | -276 |
| B3RIE5_TRIAD | - | - | - | - | - | - | - | - | - | - | - | - |  |  |  |  |  |  |  |  |  |  |  |  |  |  |  |  |  |  |  |  |  |  |  |  |  |  |  |  |  |  |  |  |  |  |  |  |  |  |  |  |  |  |  |  |  |  |  |  |  | -274 |
| K1PW75_CRAGI | - | - | - | - | - | - | - | - | - | - | - | - |  |  |  |  |  |  |  |  |  |  |  |  |  |  |  |  |  |  |  |  |  |  |  |  |  |  |  |  |  |  |  |  |  |  |  |  |  |  |  |  |  |  |  |  |  |  |  |  |  | -272 |
| K1RSB8_CRAGI | - | - | - | - | - | - | - | - | - | - | - | - |  |  |  |  |  |  |  |  |  |  |  |  |  |  |  |  |  |  |  |  |  |  |  |  |  |  |  |  |  |  |  |  |  |  |  |  |  |  |  |  |  |  |  |  |  |  |  |  |  | -270 |
| K1RWI3_CRAGI | - | - | - | - | - | - | - | - | - | - | - | - |  |  |  |  |  |  |  |  |  |  |  |  |  |  |  |  |  |  |  |  |  |  |  |  |  |  |  |  |  |  |  |  |  |  |  |  |  |  |  |  |  |  |  |  |  |  |  |  |  | -273 |
| R7VGC2_CAPTE | - | - | - | - | - | - | - | - | - | - | - | - |  |  |  |  |  |  |  |  |  |  |  |  |  |  |  |  |  |  |  |  |  |  |  |  |  |  |  |  |  |  |  |  |  |  |  |  |  |  |  |  |  |  |  |  |  |  |  |  |  | -273 |
| K1QU26_CRAGI | - | - | - | - | - | - | - | - | - | - | - | - |  |  |  |  |  |  |  |  |  |  |  |  |  |  |  |  |  |  |  |  |  |  |  |  |  |  |  |  |  |  |  |  |  |  |  |  |  |  |  |  |  |  |  |  |  |  |  |  |  | -275 |
| CAN5_CAEEL | - | - | - | - | - | - | - | - | - | - | - | - |  |  |  |  |  |  |  |  |  |  |  |  |  |  |  |  |  |  |  |  |  |  |  |  |  |  |  |  |  |  |  |  |  |  |  |  |  |  |  |  |  |  |  |  |  |  |  |  |  | -284 |
| I1FGV4_AMPQE | - | - | - | - | - | - | - | - | - | - | - | - |  |  |  |  |  |  |  |  |  |  |  |  |  |  |  |  |  |  |  |  |  |  |  |  |  |  |  |  |  |  |  |  |  |  |  |  |  |  |  |  |  |  |  |  |  |  |  |  |  | -255 |
| K1PHX3_CRAGI | - | - | - | - | - | - | - | - | - | - | - | - |  |  |  |  |  |  |  |  |  |  |  |  |  |  |  |  |  |  |  |  |  |  |  |  |  |  |  |  |  |  |  |  |  |  |  |  |  |  |  |  |  |  |  |  |  |  |  |  |  | -254 |
| B3RNL8_TRIAD | - | - | - | - | - | - | - | - | - | - | - | - |  |  |  |  |  |  |  |  |  |  |  |  |  |  |  |  |  |  |  |  |  |  |  |  |  |  |  |  |  |  |  |  |  |  |  |  |  |  |  |  |  |  |  |  |  |  |  |  |  | -255 |
| X1Y3H7_ANODA | - | - | - | - | - | - | - | - | - | - | - | - |  |  |  |  |  |  |  |  |  |  |  |  |  |  |  |  |  |  |  |  |  |  |  |  |  |  |  |  |  |  |  |  |  |  |  |  |  |  |  |  |  |  |  |  |  |  |  |  |  | -265 |
| R7TWH9_CAPTE | - | E | V | T | I | C | T | T | G | P | D | - |  |  |  |  |  |  |  |  |  |  |  |  |  |  |  |  |  |  |  |  |  |  |  |  |  |  |  |  |  |  |  |  |  |  |  |  |  |  |  |  |  |  |  |  |  |  |  |  |  | -277 |
| K1R5E8_CRAGI | - | - | - | - | - | - | - | - | - | - | - | - |  |  |  |  |  |  |  |  |  |  |  |  |  |  |  |  |  |  |  |  |  |  |  |  |  |  |  |  |  |  |  |  |  |  |  |  |  |  |  |  |  |  |  |  |  |  |  |  |  | -258 |
| XP_006811804 | - | - | - | - | - | - | - | - | - | - | - | - |  |  |  |  |  |  |  |  |  |  |  |  |  |  |  |  |  |  |  |  |  |  |  |  |  |  |  |  |  |  |  |  |  |  |  |  |  |  |  |  |  |  |  |  |  |  |  |  |  | -254 |
| C3YB23_BRAFL | - | - | - | - | - | - | - | - | - | - | - | - |  |  |  |  |  |  |  |  |  |  |  |  |  |  |  |  |  |  |  |  |  |  |  |  |  |  |  |  |  |  |  |  |  |  |  |  |  |  |  |  |  |  |  |  |  |  |  |  |  | -263 |
| H2ZIF1_CIOSA | - | - | - | - | - | - | - | - | - | - | - | - |  |  |  |  |  |  |  |  |  |  |  |  |  |  |  |  |  |  |  |  |  |  |  |  |  |  |  |  |  |  |  |  |  |  |  |  |  |  |  |  |  |  |  |  |  |  |  |  |  | -253 |
| W4YV93_STRPU | - | - | - | - | - | - | - | - | - | - | - | - |  |  |  |  |  |  |  |  |  |  |  |  |  |  |  |  |  |  |  |  |  |  |  |  |  |  |  |  |  |  |  |  |  |  |  |  |  |  |  |  |  |  |  |  |  |  |  |  |  | -257 |
| CAN10_HUMAN | - | - | - | - | - | - | - | - | - | - | - | - |  |  |  |  |  |  |  |  |  |  |  |  |  |  |  |  |  |  |  |  |  |  |  |  |  |  |  |  |  |  |  |  |  |  |  |  |  |  |  |  |  |  |  |  |  |  |  |  |  | -262 |
| CAN10_MOUSE | - | - | - | - | - | - | - | - | - | - | - | - |  |  |  |  |  |  |  |  |  |  |  |  |  |  |  |  |  |  |  |  |  |  |  |  |  |  |  |  |  |  |  |  |  |  |  |  |  |  |  |  |  |  |  |  |  |  |  |  |  | -262 |
| XP_004943443 | - | - | - | - | - | - | - | - | - | - | - | - |  |  |  |  |  |  |  |  |  |  |  |  |  |  |  |  |  |  |  |  |  |  |  |  |  |  |  |  |  |  |  |  |  |  |  |  |  |  |  |  |  |  |  |  |  |  |  |  |  | -262 |
| F8W4K1_DANRE | - | - | - | - | - | - | - | - | - | - | - | - |  |  |  |  |  |  |  |  |  |  |  |  |  |  |  |  |  |  |  |  |  |  |  |  |  |  |  |  |  |  |  |  |  |  |  |  |  |  |  |  |  |  |  |  |  |  |  |  |  | -265 |
| CANC_DROME | - | - | - | - | - | - | - | - | - | - | - | - |  |  |  |  |  |  |  |  |  |  |  |  |  |  |  |  |  |  |  |  |  |  |  |  |  |  |  |  |  |  |  |  |  |  |  |  |  |  |  |  |  |  |  |  |  |  |  |  |  | -250 |
| C3ZKN9_BRAFL | - | - | - | - | - | - | - | - | - | - | - | - |  |  |  |  |  |  |  |  |  |  |  |  |  |  |  |  |  |  |  |  |  |  |  |  |  |  |  |  |  |  |  |  |  |  |  |  |  |  |  |  |  |  |  |  |  |  |  |  |  | -233 |
| G4V623_SCHMA | - | - | - | - | - | - | - | - | - | - | - | - |  |  |  |  |  |  |  |  |  |  |  |  |  |  |  |  |  |  |  |  |  |  |  |  |  |  |  |  |  |  |  |  |  |  |  |  |  |  |  |  |  |  |  |  |  |  |  |  |  | -275 |
| R7VK39_CAPTE | - | - | - | - | - | - | - | - | - | - | - | - |  |  |  |  |  |  |  |  |  |  |  |  |  |  |  |  |  |  |  |  |  |  |  |  |  |  |  |  |  |  |  |  |  |  |  |  |  |  |  |  |  |  |  |  |  |  |  |  |  | -226 |
| G4V693_SCHMA | - | - | - | - | - | - | - | - | - | - | - | - |  |  |  |  |  |  |  |  |  |  |  |  |  |  |  |  |  |  |  |  |  |  |  |  |  |  |  |  |  |  |  |  |  |  |  |  |  |  |  |  |  |  |  |  |  |  |  |  |  | -307 |
| R7UPU6_CAPTE | - | D | T | H | M | C | N | F | T | P | D | - |  |  |  |  |  |  |  |  |  |  |  |  |  |  |  |  |  |  |  |  |  |  |  |  |  |  |  |  |  |  |  |  |  |  |  |  |  |  |  |  |  |  |  |  |  |  |  |  |  | -276 |
| R7V817_CAPTE | - | - | - | - | - | - | - | - | - | - | - | - |  |  |  |  |  |  |  |  |  |  |  |  |  |  |  |  |  |  |  |  |  |  |  |  |  |  |  |  |  |  |  |  |  |  |  |  |  |  |  |  |  |  |  |  |  |  |  |  |  | -256 |
| R7UNL8_CAPTE | - | R | V | S | I | C | N | F | T | P | D | - |  |  |  |  |  |  |  |  |  |  |  |  |  |  |  |  |  |  |  |  |  |  |  |  |  |  |  |  |  |  |  |  |  |  |  |  |  |  |  |  |  |  |  |  |  |  |  |  |  | -268 |
| K1PJ80_CRAGI | - | - | - | - | - | - | - | - | - | - | - | - |  |  |  |  |  |  |  |  |  |  |  |  |  |  |  |  |  |  |  |  |  |  |  |  |  |  |  |  |  |  |  |  |  |  |  |  |  |  |  |  |  |  |  |  |  |  |  |  |  | -247 |
| K1PCX8_CRAGI | - | - | - | - | - | - | - | - | - | - | - | - |  |  |  |  |  |  |  |  |  |  |  |  |  |  |  |  |  |  |  |  |  |  |  |  |  |  |  |  |  |  |  |  |  |  |  |  |  |  |  |  |  |  |  |  |  |  |  |  |  | -247 |
| CAND_DROME | - | - | - | - | - | - | - | - | - | - | - | - |  |  |  |  |  |  |  |  |  |  |  |  |  |  |  |  |  |  |  |  |  |  |  |  |  |  |  |  |  |  |  |  |  |  |  |  |  |  |  |  |  |  |  |  |  |  |  |  |  | -261 |
| XP_002735765 | - | - | - | - | - | - | - | - | - | - | - | - |  |  |  |  |  |  |  |  |  |  |  |  |  |  |  |  |  |  |  |  |  |  |  |  |  |  |  |  |  |  |  |  |  |  |  |  |  |  |  |  |  |  |  |  |  |  |  |  |  | -264 |
| CAN15_HUMAN | - | - | - | - | - | - | - | - | - | - | - | - |  |  |  |  |  |  |  |  |  |  |  |  |  |  |  |  |  |  |  |  |  |  |  |  |  |  |  |  |  |  |  |  |  |  |  |  |  |  |  |  |  |  |  |  |  |  |  |  |  | -261 |
| CAN15_MOUSE | - | - | - | - | - | - | - | - | - | - | - | - |  |  |  |  |  |  |  |  |  |  |  |  |  |  |  |  |  |  |  |  |  |  |  |  |  |  |  |  |  |  |  |  |  |  |  |  |  |  |  |  |  |  |  |  |  |  |  |  |  | -262 |
| E1BTA8_CHICK | - | - | - | - | - | - | - | - | - | - | - | - |  |  |  |  |  |  |  |  |  |  |  |  |  |  |  |  |  |  |  |  |  |  |  |  |  |  |  |  |  |  |  |  |  |  |  |  |  |  |  |  |  |  |  |  |  |  |  |  |  | -262 |
| E7FAX5_DANRE | - | - | - | - | - | - | - | - | - | - | - | - |  |  |  |  |  |  |  |  |  |  |  |  |  |  |  |  |  |  |  |  |  |  |  |  |  |  |  |  |  |  |  |  |  |  |  |  |  |  |  |  |  |  |  |  |  |  |  |  |  | -262 |
| C3XWK8_BRAFL | - | - | - | - | - | - | - | - | - | - | - | - |  |  |  |  |  |  |  |  |  |  |  |  |  |  |  |  |  |  |  |  |  |  |  |  |  |  |  |  |  |  |  |  |  |  |  |  |  |  |  |  |  |  |  |  |  |  |  |  |  | -261 |
| W4YMU4_STRPU | - | - | - | - | - | - | - | - | - | - | - | - |  |  |  |  |  |  |  |  |  |  |  |  |  |  |  |  |  |  |  |  |  |  |  |  |  |  |  |  |  |  |  |  |  |  |  |  |  |  |  |  |  |  |  |  |  |  |  |  |  | -376 |
| K1PV74_CRAGI | - | - | - | - | - | - | - | - | - | - | - | - |  |  |  |  |  |  |  |  |  |  |  |  |  |  |  |  |  |  |  |  |  |  |  |  |  |  |  |  |  |  |  |  |  |  |  |  |  |  |  |  |  |  |  |  |  |  |  |  |  | -262 |
| R7TJF2_CAPTE | - | - | - | - | - | - | - | - | - | - | - | - |  |  |  |  |  |  |  |  |  |  |  |  |  |  |  |  |  |  |  |  |  |  |  |  |  |  |  |  |  |  |  |  |  |  |  |  |  |  |  |  |  |  |  |  |  |  |  |  |  | -267 |
| T2MI17_HYDVU | - | - | - | - | - | - | - | - | - | - | - | - |  |  |  |  |  |  |  |  |  |  |  |  |  |  |  |  |  |  |  |  |  |  |  |  |  |  |  |  |  |  |  |  |  |  |  |  |  |  |  |  |  |  |  |  |  |  |  |  |  | -260 |
| Q1RL55_CIOIN | - | - | - | - | - | - | - | - | - | - | - | - |  |  |  |  |  |  |  |  |  |  |  |  |  |  |  |  |  |  |  |  |  |  |  |  |  |  |  |  |  |  |  |  |  |  |  |  |  |  |  |  |  |  |  |  |  |  |  |  |  | -251 |
| I1EA30_AMPQE | - | - | - | - | - | - | - | - | - | - | - | - |  |  |  |  |  |  |  |  |  |  |  |  |  |  |  |  |  |  |  |  |  |  |  |  |  |  |  |  |  |  |  |  |  |  |  |  |  |  |  |  |  |  |  |  |  |  |  |  |  | -234 |
| G5EEZ6_CAEEL | - | - | - | - | - | - | - | - | - | - | - | - |  |  |  |  |  |  |  |  |  |  |  |  |  |  |  |  |  |  |  |  |  |  |  |  |  |  |  |  |  |  |  |  |  |  |  |  |  |  |  |  |  |  |  |  |  |  |  |  |  | -258 |
| O44903_CAEEL | - | - | - | - | - | - | - | - | - | - | - | - |  |  |  |  |  |  |  |  |  |  |  |  |  |  |  |  |  |  |  |  |  |  |  |  |  |  |  |  |  |  |  |  |  |  |  |  |  |  |  |  |  |  |  |  |  |  |  |  |  | -258 |
| O02260_CAEEL | - | - | - | - | - | - | - | - | - | - | - | - |  |  |  |  |  |  |  |  |  |  |  |  |  |  |  |  |  |  |  |  |  |  |  |  |  |  |  |  |  |  |  |  |  |  |  |  |  |  |  |  |  |  |  |  |  |  |  |  |  | -241 |
| O02259_CAEEL | - | - | - | - | - | - | - | - | - | - | - | - |  |  |  |  |  |  |  |  |  |  |  |  |  |  |  |  |  |  |  |  |  |  |  |  |  |  |  |  |  |  |  |  |  |  |  |  |  |  |  |  |  |  |  |  |  |  |  |  |  | -232 |
| C1MTG3_MICPC | - | - | - | - | - | - | - | - | - | - | - | - |  |  |  |  |  |  |  |  |  |  |  |  |  |  |  |  |  |  |  |  |  |  |  |  |  |  |  |  |  |  |  |  |  |  |  |  |  |  |  |  |  |  |  |  |  |  |  |  |  | -342 |
| C1E493_MICSR | - | - | - | - | - | - | - | - | - | - | - | - |  |  |  |  |  |  |  |  |  |  |  |  |  |  |  |  |  |  |  |  |  |  |  |  |  |  |  |  |  |  |  |  |  |  |  |  |  |  |  |  |  |  |  |  |  |  |  |  |  | -275 |
| Q012Z6_OSTTA | - | - | - | - | - | - | - | - | - | - | - | - |  |  |  |  |  |  |  |  |  |  |  |  |  |  |  |  |  |  |  |  |  |  |  |  |  |  |  |  |  |  |  |  |  |  |  |  |  |  |  |  |  |  |  |  |  |  |  |  |  | -263 |
| L1IGB6_GUITH | - | - | - | - | - | - | - | - | - | - | - | - |  |  |  |  |  |  |  |  |  |  |  |  |  |  |  |  |  |  |  |  |  |  |  |  |  |  |  |  |  |  |  |  |  |  |  |  |  |  |  |  |  |  |  |  |  |  |  |  |  | -250 |
| B7FWX6_PHATC | - | - | - | - | - | - | - | - | - | - | - | - |  |  |  |  |  |  |  |  |  |  |  |  |  |  |  |  |  |  |  |  |  |  |  |  |  |  |  |  |  |  |  |  |  |  |  |  |  |  |  |  |  |  |  |  |  |  |  |  |  | -279 |
| N9TPR9_ENTHI | - | - | - | - | - | - | - | - | - | - | - | - |  |  |  |  |  |  |  |  |  |  |  |  |  |  |  |  |  |  |  |  |  |  |  |  |  |  |  |  |  |  |  |  |  |  |  |  |  |  |  |  |  |  |  |  |  |  |  |  |  | -238 |
| G0QS43_ICHMG | - | - | - | - | - | - | - | - | - | - | - | - |  |  |  |  |  |  |  |  |  |  |  |  |  |  |  |  |  |  |  |  |  |  |  |  |  |  |  |  |  |  |  |  |  |  |  |  |  |  |  |  |  |  |  |  |  |  |  |  |  | -254 |
| D8LB20_ECTSI | - | - | - | - | - | - | - | - | - | - | - | - |  |  |  |  |  |  |  |  |  |  |  |  |  |  |  |  |  |  |  |  |  |  |  |  |  |  |  |  |  |  |  |  |  |  |  |  |  |  |  |  |  |  |  |  |  |  |  |  |  | -288 |
| Q9U0T9_LEIMA | - | - | - | - | - | - | - | - | - | - | - | - |  |  |  |  |  |  |  |  |  |  |  |  |  |  |  |  |  |  |  |  |  |  |  |  |  |  |  |  |  |  |  |  |  |  |  |  |  |  |  |  |  |  |  |  |  |  |  |  |  | -282 |
| Q4CXJ8_TRYCC | - | - | - | - | - | - | - | - | - | - | - | - |  |  |  |  |  |  |  |  |  |  |  |  |  |  |  |  |  |  |  |  |  |  |  |  |  |  |  |  |  |  |  |  |  |  |  |  |  |  |  |  |  |  |  |  |  |  |  |  |  | -281 |
| F4I0A4_ARATH | - | - | - | - | - | - | - | - | - | - | - | - |  |  |  |  |  |  |  |  |  |  |  |  |  |  |  |  |  |  |  |  |  |  |  |  |  |  |  |  |  |  |  |  |  |  |  |  |  |  |  |  |  |  |  |  |  |  |  |  |  | -256 |
| Q8RUQ1_MAIZE | - | - | - | - | - | - | - | - | - | - | - | - |  |  |  |  |  |  |  |  |  |  |  |  |  |  |  |  |  |  |  |  |  |  |  |  |  |  |  |  |  |  |  |  |  |  |  |  |  |  |  |  |  |  |  |  |  |  |  |  |  | -256 |
| D7FQ17_ECTSI | - | - | - | - | - | - | - | - | - | - | - | - |  |  |  |  |  |  |  |  |  |  |  |  |  |  |  |  |  |  |  |  |  |  |  |  |  |  |  |  |  |  |  |  |  |  |  |  |  |  |  |  |  |  |  |  |  |  |  |  |  | -273 |
| D0MZ44_PHYIT | - | - | - | - | - | - | - | - | - | - | - | - |  |  |  |  |  |  |  |  |  |  |  |  |  |  |  |  |  |  |  |  |  |  |  |  |  |  |  |  |  |  |  |  |  |  |  |  |  |  |  |  |  |  |  |  |  |  |  |  |  | -277 |
| L1JBM0_GUITH | - | - | - | - | - | - | - | - | - | - | - | - |  |  |  |  |  |  |  |  |  |  |  |  |  |  |  |  |  |  |  |  |  |  |  |  |  |  |  |  |  |  |  |  |  |  |  |  |  |  |  |  |  |  |  |  |  |  |  |  |  | -231 |
| L1J739_GUITH | - | - | - | - | - | - | - | - | - | - | - | - |  |  |  |  |  |  |  |  |  |  |  |  |  |  |  |  |  |  |  |  |  |  |  |  |  |  |  |  |  |  |  |  |  |  |  |  |  |  |  |  |  |  |  |  |  |  |  |  |  | -252 |
| L1J0U9_GUITH | - | - | - | - | - | - | - | - | - | - | - | - |  |  |  |  |  |  |  |  |  |  |  |  |  |  |  |  |  |  |  |  |  |  |  |  |  |  |  |  |  |  |  |  |  |  |  |  |  |  |  |  |  |  |  |  |  |  |  |  |  | -256 |
| L1J1Q4_GUITH | - | - | - | - | - | - | - | - | - | - | - | - |  |  |  |  |  |  |  |  |  |  |  |  |  |  |  |  |  |  |  |  |  |  |  |  |  |  |  |  |  |  |  |  |  |  |  |  |  |  |  |  |  |  |  |  |  |  |  |  |  | -274 |
| D0ND88_PHYIT | - | - | - | - | - | - | - | - | - | - | - | - |  |  |  |  |  |  |  |  |  |  |  |  |  |  |  |  |  |  |  |  |  |  |  |  |  |  |  |  |  |  |  |  |  |  |  |  |  |  |  |  |  |  |  |  |  |  |  |  |  | -320 |
| D2VVS6_NAEGR | - | - | - | - | - | - | - | - | - | - | - | - |  |  |  |  |  |  |  |  |  |  |  |  |  |  |  |  |  |  |  |  |  |  |  |  |  |  |  |  |  |  |  |  |  |  |  |  |  |  |  |  |  |  |  |  |  |  |  |  |  | -259 |
| D2V3V2_NAEGR | - | Q | I | Q | - | - | - | - | - | - | - | - |  |  |  |  |  |  |  |  |  |  |  |  |  |  |  |  |  |  |  |  |  |  |  |  |  |  |  |  |  |  |  |  |  |  |  |  |  |  |  |  |  |  |  |  |  |  |  |  |  | -271 |
| Q5B5J7_EMENI | - | F | - | - | - | - | - | - | - | - | - | - |  |  |  |  |  |  |  |  |  |  |  |  |  |  |  |  |  |  |  |  |  |  |  |  |  |  |  |  |  |  |  |  |  |  |  |  |  |  |  |  |  |  |  |  |  |  |  |  |  | -294 |
| E4ZY10_LEPMJ | - | - | - | - | - | - | - | - | - | - | - | - |  |  |  |  |  |  |  |  |  |  |  |  |  |  |  |  |  |  |  |  |  |  |  |  |  |  |  |  |  |  |  |  |  |  |  |  |  |  |  |  |  |  |  |  |  |  |  |  |  | -273 |
| A8PIE5_COPC7 | - | F | - | - | - | - | - | - | - | - | - | - |  |  |  |  |  |  |  |  |  |  |  |  |  |  |  |  |  |  |  |  |  |  |  |  |  |  |  |  |  |  |  |  |  |  |  |  |  |  |  |  |  |  |  |  |  |  |  |  |  | -297 |
| A8NBA6_COPC7 | - | - | - | - | - | - | - | - | - | - | - | - |  |  |  |  |  |  |  |  |  |  |  |  |  |  |  |  |  |  |  |  |  |  |  |  |  |  |  |  |  |  |  |  |  |  |  |  |  |  |  |  |  |  |  |  |  |  |  |  |  | -293 |
| A8NBA1_COPC7 | - | - | - | - | - | - | - | - | - | - | - | - |  |  |  |  |  |  |  |  |  |  |  |  |  |  |  |  |  |  |  |  |  |  |  |  |  |  |  |  |  |  |  |  |  |  |  |  |  |  |  |  |  |  |  |  |  |  |  |  |  | -249 |
| A8NBA2_COPC7 | - | - | - | - | - | - | - | - | - | - | - | - |  |  |  |  |  |  |  |  |  |  |  |  |  |  |  |  |  |  |  |  |  |  |  |  |  |  |  |  |  |  |  |  |  |  |  |  |  |  |  |  |  |  |  |  |  |  |  |  |  | -247 |
| A8NAE6_COPC7 | - | - | - | - | - | - | - | - | - | - | - | - |  |  |  |  |  |  |  |  |  |  |  |  |  |  |  |  |  |  |  |  |  |  |  |  |  |  |  |  |  |  |  |  |  |  |  |  |  |  |  |  |  |  |  |  |  |  |  |  |  | -285 |
| A8NAI9_COPC7 | - | - | - | - | - | - | - | - | - | - | - | - |  |  |  |  |  |  |  |  |  |  |  |  |  |  |  |  |  |  |  |  |  |  |  |  |  |  |  |  |  |  |  |  |  |  |  |  |  |  |  |  |  |  |  |  |  |  |  |  |  | -269 |
| G4TER7_PIRID | - | - | - | - | - | - | - | - | - | - | - | - |  |  |  |  |  |  |  |  |  |  |  |  |  |  |  |  |  |  |  |  |  |  |  |  |  |  |  |  |  |  |  |  |  |  |  |  |  |  |  |  |  |  |  |  |  |  |  |  |  | -253 |
| G4TER6_PIRID | - | - | - | - | - | - | - | - | - | - | - | - |  |  |  |  |  |  |  |  |  |  |  |  |  |  |  |  |  |  |  |  |  |  |  |  |  |  |  |  |  |  |  |  |  |  |  |  |  |  |  |  |  |  |  |  |  |  |  |  |  | -242 |
| G4TNA1_PIRID | - | - | - | - | - | - | - | - | - | - | - | - |  |  |  |  |  |  |  |  |  |  |  |  |  |  |  |  |  |  |  |  |  |  |  |  |  |  |  |  |  |  |  |  |  |  |  |  |  |  |  |  |  |  |  |  |  |  |  |  |  | -252 |
| D0MWB9_PHYIT | - | - | - | - | - | - | - | - | - | - | - | - |  |  |  |  |  |  |  |  |  |  |  |  |  |  |  |  |  |  |  |  |  |  |  |  |  |  |  |  |  |  |  |  |  |  |  |  |  |  |  |  |  |  |  |  |  |  |  |  |  | -249 |
| L1I9H5_GUITH | - | - | - | - | - | - | - | - | - | - | - | - |  |  |  |  |  |  |  |  |  |  |  |  |  |  |  |  |  |  |  |  |  |  |  |  |  |  |  |  |  |  |  |  |  |  |  |  |  |  |  |  |  |  |  |  |  |  |  |  |  | -261 |
| Q7N497_PHOLL | - | - | - | - | - | - | - | - | - | - | - | - |  |  |  |  |  |  |  |  |  |  |  |  |  |  |  |  |  |  |  |  |  |  |  |  |  |  |  |  |  |  |  |  |  |  |  |  |  |  |  |  |  |  |  |  |  |  |  |  |  | -225 |
| ZP_11152369 | - | - | - | - | - | - | - | - | - | - | - | - |  |  |  |  |  |  |  |  |  |  |  |  |  |  |  |  |  |  |  |  |  |  |  |  |  |  |  |  |  |  |  |  |  |  |  |  |  |  |  |  |  |  |  |  |  |  |  |  |  | -223 |
| A2FEL7_TRIVA | - | - | - | - | - | - | - | - | - | - | - | - |  |  |  |  |  |  |  |  |  |  |  |  |  |  |  |  |  |  |  |  |  |  |  |  |  |  |  |  |  |  |  |  |  |  |  |  |  |  |  |  |  |  |  |  |  |  |  |  |  | -253 |
| A2D9A8_TRIVA | - | - | - | - | - | - | - | - | - | - | - | - |  |  |  |  |  |  |  |  |  |  |  |  |  |  |  |  |  |  |  |  |  |  |  |  |  |  |  |  |  |  |  |  |  |  |  |  |  |  |  |  |  |  |  |  |  |  |  |  |  | -250 |
| A2FHB3_TRIVA | - | - | - | - | - | - | - | - | - | - | - | - |  |  |  |  |  |  |  |  |  |  |  |  |  |  |  |  |  |  |  |  |  |  |  |  |  |  |  |  |  |  |  |  |  |  |  |  |  |  |  |  |  |  |  |  |  |  |  |  |  | -253 |
| A2DTE8_TRIVA | - | - | - | - | - | - | - | - | - | - | - | - |  |  |  |  |  |  |  |  |  |  |  |  |  |  |  |  |  |  |  |  |  |  |  |  |  |  |  |  |  |  |  |  |  |  |  |  |  |  |  |  |  |  |  |  |  |  |  |  |  | -241 |
| A2E4X2_TRIVA | - | - | - | - | - | - | - | - | - | - | - | - |  |  |  |  |  |  |  |  |  |  |  |  |  |  |  |  |  |  |  |  |  |  |  |  |  |  |  |  |  |  |  |  |  |  |  |  |  |  |  |  |  |  |  |  |  |  |  |  |  | -247 |
| A2E1X1_TRIVA | - | - | - | - | - | - | - | - | - | - | - | - |  |  |  |  |  |  |  |  |  |  |  |  |  |  |  |  |  |  |  |  |  |  |  |  |  |  |  |  |  |  |  |  |  |  |  |  |  |  |  |  |  |  |  |  |  |  |  |  |  | -191 |
| TPR_PORGI | - | - | - | - | - | - | - | - | - | - | - | - |  |  |  |  |  |  |  |  |  |  |  |  |  |  |  |  |  |  |  |  |  |  |  |  |  |  |  |  |  |  |  |  |  |  |  |  |  |  |  |  |  |  |  |  |  |  |  |  |  | -274 |
| D5MF42_9BACT | - | - | - | - | - | - | - | - | - | - | - | - |  |  |  |  |  |  |  |  |  |  |  |  |  |  |  |  |  |  |  |  |  |  |  |  |  |  |  |  |  |  |  |  |  |  |  |  |  |  |  |  |  |  |  |  |  |  |  |  |  | -202 |
| R9H254_BACT4 | - | - | - | - | - | - | - | - | - | - | - | - |  |  |  |  |  |  |  |  |  |  |  |  |  |  |  |  |  |  |  |  |  |  |  |  |  |  |  |  |  |  |  |  |  |  |  |  |  |  |  |  |  |  |  |  |  |  |  |  |  | -189 |
| ZP_04847694 | - | - | - | - | - | - | - | - | - | - | - | - |  |  |  |  |  |  |  |  |  |  |  |  |  |  |  |  |  |  |  |  |  |  |  |  |  |  |  |  |  |  |  |  |  |  |  |  |  |  |  |  |  |  |  |  |  |  |  |  |  | -189 |
| D7IBA4_9BACE | - | - | - | - | - | - | - | - | - | - | - | - |  |  |  |  |  |  |  |  |  |  |  |  |  |  |  |  |  |  |  |  |  |  |  |  |  |  |  |  |  |  |  |  |  |  |  |  |  |  |  |  |  |  |  |  |  |  |  |  |  | -187 |
| ZP_09858577 | - | - | - | - | - | - | - | - | - | - | - | - |  |  |  |  |  |  |  |  |  |  |  |  |  |  |  |  |  |  |  |  |  |  |  |  |  |  |  |  |  |  |  |  |  |  |  |  |  |  |  |  |  |  |  |  |  |  |  |  |  | -187 |
| E5C759_9BACE | - | - | - | - | - | - | - | - | - | - | - | - |  |  |  |  |  |  |  |  |  |  |  |  |  |  |  |  |  |  |  |  |  |  |  |  |  |  |  |  |  |  |  |  |  |  |  |  |  |  |  |  |  |  |  |  |  |  |  |  |  | -189 |
| A7LTS0_BACO1 | - | - | - | - | - | - | - | - | - | - | - | - |  |  |  |  |  |  |  |  |  |  |  |  |  |  |  |  |  |  |  |  |  |  |  |  |  |  |  |  |  |  |  |  |  |  |  |  |  |  |  |  |  |  |  |  |  |  |  |  |  | -187 |
| Q8YQK8_NOSS1 | - | - | - | - | - | - | - | - | - | - | - | - |  |  |  |  |  |  |  |  |  |  |  |  |  |  |  |  |  |  |  |  |  |  |  |  |  |  |  |  |  |  |  |  |  |  |  |  |  |  |  |  |  |  |  |  |  |  |  |  |  | -197 |
| Q3MBX8_ANAVT | - | - | - | - | - | - | - | - | - | - | - | - |  |  |  |  |  |  |  |  |  |  |  |  |  |  |  |  |  |  |  |  |  |  |  |  |  |  |  |  |  |  |  |  |  |  |  |  |  |  |  |  |  |  |  |  |  |  |  |  |  | -202 |
| Q8YZT8_NOSS1 | - | - | - | - | - | - | - | - | - | - | - | - |  |  |  |  |  |  |  |  |  |  |  |  |  |  |  |  |  |  |  |  |  |  |  |  |  |  |  |  |  |  |  |  |  |  |  |  |  |  |  |  |  |  |  |  |  |  |  |  |  | -200 |
| K9UIG2_9CHRO | - | - | - | - | - | - | - | - | - | - | - | - |  |  |  |  |  |  |  |  |  |  |  |  |  |  |  |  |  |  |  |  |  |  |  |  |  |  |  |  |  |  |  |  |  |  |  |  |  |  |  |  |  |  |  |  |  |  |  |  |  | -212 |
| A4YRP8_BRASO | - | - | - | - | - | - | - | - | - | - | - | - |  |  |  |  |  |  |  |  |  |  |  |  |  |  |  |  |  |  |  |  |  |  |  |  |  |  |  |  |  |  |  |  |  |  |  |  |  |  |  |  |  |  |  |  |  |  |  |  |  | -217 |
| H0TAL7_9BRAD | - | - | - | - | - | - | - | - | - | - | - | - |  |  |  |  |  |  |  |  |  |  |  |  |  |  |  |  |  |  |  |  |  |  |  |  |  |  |  |  |  |  |  |  |  |  |  |  |  |  |  |  |  |  |  |  |  |  |  |  |  | -222 |
| H0SNL4_9BRAD | - | - | - | - | - | - | - | - | - | - | - | - |  |  |  |  |  |  |  |  |  |  |  |  |  |  |  |  |  |  |  |  |  |  |  |  |  |  |  |  |  |  |  |  |  |  |  |  |  |  |  |  |  |  |  |  |  |  |  |  |  | -224 |
| H0S4P6_9BRAD | - | - | - | - | - | - | - | - | - | - | - | - |  |  |  |  |  |  |  |  |  |  |  |  |  |  |  |  |  |  |  |  |  |  |  |  |  |  |  |  |  |  |  |  |  |  |  |  |  |  |  |  |  |  |  |  |  |  |  |  |  | -224 |
| A5EMK2_BRASB | - | - | - | - | - | - | - | - | - | - | - | - |  |  |  |  |  |  |  |  |  |  |  |  |  |  |  |  |  |  |  |  |  |  |  |  |  |  |  |  |  |  |  |  |  |  |  |  |  |  |  |  |  |  |  |  |  |  |  |  |  | -217 |
| M4ZBJ4_9BRAD | - | - | - | - | - | - | - | - | - | - | - | - |  |  |  |  |  |  |  |  |  |  |  |  |  |  |  |  |  |  |  |  |  |  |  |  |  |  |  |  |  |  |  |  |  |  |  |  |  |  |  |  |  |  |  |  |  |  |  |  |  | -226 |
| Q07SP7_RHOP5 | - | - | - | - | - | - | - | - | - | - | - | - |  |  |  |  |  |  |  |  |  |  |  |  |  |  |  |  |  |  |  |  |  |  |  |  |  |  |  |  |  |  |  |  |  |  |  |  |  |  |  |  |  |  |  |  |  |  |  |  |  | -210 |
| B0JUQ7_MICAN | - | - | - | - | - | - | - | - | - | - | - | - |  |  |  |  |  |  |  |  |  |  |  |  |  |  |  |  |  |  |  |  |  |  |  |  |  |  |  |  |  |  |  |  |  |  |  |  |  |  |  |  |  |  |  |  |  |  |  |  |  | -191 |
| K9WJ87_9CYAN | - | - | - | - | - | - | - | - | - | - | - | - |  |  |  |  |  |  |  |  |  |  |  |  |  |  |  |  |  |  |  |  |  |  |  |  |  |  |  |  |  |  |  |  |  |  |  |  |  |  |  |  |  |  |  |  |  |  |  |  |  | -193 |
| G6FUE0_9CYAN | - | - | - | - | - | - | - | - | - | - | - | - |  |  |  |  |  |  |  |  |  |  |  |  |  |  |  |  |  |  |  |  |  |  |  |  |  |  |  |  |  |  |  |  |  |  |  |  |  |  |  |  |  |  |  |  |  |  |  |  |  | -214 |
| K9PHG5_9CYAN | - | - | - | - | - | - | - | - | - | - | - | - |  |  |  |  |  |  |  |  |  |  |  |  |  |  |  |  |  |  |  |  |  |  |  |  |  |  |  |  |  |  |  |  |  |  |  |  |  |  |  |  |  |  |  |  |  |  |  |  |  | -196 |
| Q8YXX3_NOSS1 | - | - | - | - | - | - | - | - | - | - | - | - |  |  |  |  |  |  |  |  |  |  |  |  |  |  |  |  |  |  |  |  |  |  |  |  |  |  |  |  |  |  |  |  |  |  |  |  |  |  |  |  |  |  |  |  |  |  |  |  |  | -211 |
| K9TN70_9CYAN | - | - | - | - | - | - | - | - | - | - | - | - |  |  |  |  |  |  |  |  |  |  |  |  |  |  |  |  |  |  |  |  |  |  |  |  |  |  |  |  |  |  |  |  |  |  |  |  |  |  |  |  |  |  |  |  |  |  |  |  |  | -214 |
| Q05QW3_9SYNE | - | - | - | - | - | - | - | - | - | - | - | - |  |  |  |  |  |  |  |  |  |  |  |  |  |  |  |  |  |  |  |  |  |  |  |  |  |  |  |  |  |  |  |  |  |  |  |  |  |  |  |  |  |  |  |  |  |  |  |  |  | -238 |
| Q05QW2_9SYNE | - | - | - | - | - | - | - | - | - | - | - | - |  |  |  |  |  |  |  |  |  |  |  |  |  |  |  |  |  |  |  |  |  |  |  |  |  |  |  |  |  |  |  |  |  |  |  |  |  |  |  |  |  |  |  |  |  |  |  |  |  | -205 |
| Q05QW4_9SYNE | - | - | - | - | - | - | - | - | - | - | - | - |  |  |  |  |  |  |  |  |  |  |  |  |  |  |  |  |  |  |  |  |  |  |  |  |  |  |  |  |  |  |  |  |  |  |  |  |  |  |  |  |  |  |  |  |  |  |  |  |  | -216 |
| E0UGX6_CYAP2 | - | - | - | - | - | - | - | - | - | - | - | - |  |  |  |  |  |  |  |  |  |  |  |  |  |  |  |  |  |  |  |  |  |  |  |  |  |  |  |  |  |  |  |  |  |  |  |  |  |  |  |  |  |  |  |  |  |  |  |  |  | -197 |
| K7WKF4_9NOST | - | - | - | - | - | - | - | - | - | - | - | - |  |  |  |  |  |  |  |  |  |  |  |  |  |  |  |  |  |  |  |  |  |  |  |  |  |  |  |  |  |  |  |  |  |  |  |  |  |  |  |  |  |  |  |  |  |  |  |  |  | -204 |
| E0UGY5_CYAP2 | - | - | - | - | - | - | - | - | - | - | - | - |  |  |  |  |  |  |  |  |  |  |  |  |  |  |  |  |  |  |  |  |  |  |  |  |  |  |  |  |  |  |  |  |  |  |  |  |  |  |  |  |  |  |  |  |  |  |  |  |  | -199 |
| A8LCE5_FRASN | - | - | - | - | - | - | - | - | - | - | - | - |  |  |  |  |  |  |  |  |  |  |  |  |  |  |  |  |  |  |  |  |  |  |  |  |  |  |  |  |  |  |  |  |  |  |  |  |  |  |  |  |  |  |  |  |  |  |  |  |  | -213 |
| D3CT47_9ACTO | - | - | - | - | - | - | - | - | - | - | - | - |  |  |  |  |  |  |  |  |  |  |  |  |  |  |  |  |  |  |  |  |  |  |  |  |  |  |  |  |  |  |  |  |  |  |  |  |  |  |  |  |  |  |  |  |  |  |  |  |  | -215 |
| Q2J4J6_FRASC | - | - | - | - | - | - | - | - | - | - | - | - |  |  |  |  |  |  |  |  |  |  |  |  |  |  |  |  |  |  |  |  |  |  |  |  |  |  |  |  |  |  |  |  |  |  |  |  |  |  |  |  |  |  |  |  |  |  |  |  |  | -235 |
| Q0RAZ2_FRAAA | - | - | - | - | - | - | - | - | - | - | - | - |  |  |  |  |  |  |  |  |  |  |  |  |  |  |  |  |  |  |  |  |  |  |  |  |  |  |  |  |  |  |  |  |  |  |  |  |  |  |  |  |  |  |  |  |  |  |  |  |  | -221 |
| E3J3F6_FRASU | - | - | - | - | - | - | - | - | - | - | - | - |  |  |  |  |  |  |  |  |  |  |  |  |  |  |  |  |  |  |  |  |  |  |  |  |  |  |  |  |  |  |  |  |  |  |  |  |  |  |  |  |  |  |  |  |  |  |  |  |  | -217 |
| ZP_09168240 | - | - | - | - | - | - | - | - | - | - | - | - |  |  |  |  |  |  |  |  |  |  |  |  |  |  |  |  |  |  |  |  |  |  |  |  |  |  |  |  |  |  |  |  |  |  |  |  |  |  |  |  |  |  |  |  |  |  |  |  |  | -228 |
| C7MDV9_BRAFD | - | - | - | - | - | - | - | - | - | - | - | - |  |  |  |  |  |  |  |  |  |  |  |  |  |  |  |  |  |  |  |  |  |  |  |  |  |  |  |  |  |  |  |  |  |  |  |  |  |  |  |  |  |  |  |  |  |  |  |  |  | -172 |
| ZP_10094989 | - | - | - | - | - | - | - | - | - | - | - | - |  |  |  |  |  |  |  |  |  |  |  |  |  |  |  |  |  |  |  |  |  |  |  |  |  |  |  |  |  |  |  |  |  |  |  |  |  |  |  |  |  |  |  |  |  |  |  |  |  | -172 |
| A4X268_SALTO | - | - | - | - | - | - | - | - | - | - | - | - |  |  |  |  |  |  |  |  |  |  |  |  |  |  |  |  |  |  |  |  |  |  |  |  |  |  |  |  |  |  |  |  |  |  |  |  |  |  |  |  |  |  |  |  |  |  |  |  |  | -231 |
| WP_043162213 | - | - | - | - | - | - | - | - | - | - | - | - |  |  |  |  |  |  |  |  |  |  |  |  |  |  |  |  |  |  |  |  |  |  |  |  |  |  |  |  |  |  |  |  |  |  |  |  |  |  |  |  |  |  |  |  |  |  |  |  |  | -231 |
| Q0BRV8_GRABC | - | - | - | - | - | - | - | - | - | - | - | - |  |  |  |  |  |  |  |  |  |  |  |  |  |  |  |  |  |  |  |  |  |  |  |  |  |  |  |  |  |  |  |  |  |  |  |  |  |  |  |  |  |  |  |  |  |  |  |  |  | -199 |
| A9B2P0_HERA2 | - | - | - | - | - | - | - | - | - | - | - | - |  |  |  |  |  |  |  |  |  |  |  |  |  |  |  |  |  |  |  |  |  |  |  |  |  |  |  |  |  |  |  |  |  |  |  |  |  |  |  |  |  |  |  |  |  |  |  |  |  | -190 |
| A9B3U8_HERA2 | - | - | - | - | - | - | - | - | - | - | - | - |  |  |  |  |  |  |  |  |  |  |  |  |  |  |  |  |  |  |  |  |  |  |  |  |  |  |  |  |  |  |  |  |  |  |  |  |  |  |  |  |  |  |  |  |  |  |  |  |  | -192 |
| A9B2A3_HERA2 | - | - | - | - | - | - | - | - | - | - | - | - |  |  |  |  |  |  |  |  |  |  |  |  |  |  |  |  |  |  |  |  |  |  |  |  |  |  |  |  |  |  |  |  |  |  |  |  |  |  |  |  |  |  |  |  |  |  |  |  |  | -216 |
| D6ZC85_SEGRD | - | - | - | - | - | - | - | - | - | - | - | - |  |  |  |  |  |  |  |  |  |  |  |  |  |  |  |  |  |  |  |  |  |  |  |  |  |  |  |  |  |  |  |  |  |  |  |  |  |  |  |  |  |  |  |  |  |  |  |  |  | -238 |
| E5XTT9_9ACTO | - | - | - | - | - | - | - | - | - | - | - | - |  |  |  |  |  |  |  |  |  |  |  |  |  |  |  |  |  |  |  |  |  |  |  |  |  |  |  |  |  |  |  |  |  |  |  |  |  |  |  |  |  |  |  |  |  |  |  |  |  | -238 |
| E5XTU0_9ACTO | - | - | - | - | - | - | - | - | - | - | - | - |  |  |  |  |  |  |  |  |  |  |  |  |  |  |  |  |  |  |  |  |  |  |  |  |  |  |  |  |  |  |  |  |  |  |  |  |  |  |  |  |  |  |  |  |  |  |  |  |  | -245 |
| D6B0I6_9ACTO | - | - | - | - | - | - | - | - | - | - | - | - |  |  |  |  |  |  |  |  |  |  |  |  |  |  |  |  |  |  |  |  |  |  |  |  |  |  |  |  |  |  |  |  |  |  |  |  |  |  |  |  |  |  |  |  |  |  |  |  |  | -207 |
| D9X719_STRVR | - | - | - | - | - | - | - | - | - | - | - | - |  |  |  |  |  |  |  |  |  |  |  |  |  |  |  |  |  |  |  |  |  |  |  |  |  |  |  |  |  |  |  |  |  |  |  |  |  |  |  |  |  |  |  |  |  |  |  |  |  | -211 |
| H6L010_SAPGL | - | - | - | - | - | - | - | - | - | - | - | - |  |  |  |  |  |  |  |  |  |  |  |  |  |  |  |  |  |  |  |  |  |  |  |  |  |  |  |  |  |  |  |  |  |  |  |  |  |  |  |  |  |  |  |  |  |  |  |  |  | -191 |
| K1ZP83_9BACT | - | - | - | - | - | - | - | - | - | - | - | - |  |  |  |  |  |  |  |  |  |  |  |  |  |  |  |  |  |  |  |  |  |  |  |  |  |  |  |  |  |  |  |  |  |  |  |  |  |  |  |  |  |  |  |  |  |  |  |  |  | -232 |
| ADGB_HUMAN | - | - | - | - | - | - | - | - | - | - | - | - |  |  |  |  |  |  |  |  |  |  |  |  |  |  |  |  |  |  |  |  |  |  |  |  |  |  |  |  |  |  |  |  |  |  |  |  |  |  |  |  |  |  |  |  |  |  |  |  |  | -250 |
| J3QMP3_MOUSE | - | - | - | - | - | - | - | - | - | - | - | - |  |  |  |  |  |  |  |  |  |  |  |  |  |  |  |  |  |  |  |  |  |  |  |  |  |  |  |  |  |  |  |  |  |  |  |  |  |  |  |  |  |  |  |  |  |  |  |  |  | -250 |
| H2XR46_CIOIN | - | - | - | - | - | - | - | - | - | - | - | - |  |  |  |  |  |  |  |  |  |  |  |  |  |  |  |  |  |  |  |  |  |  |  |  |  |  |  |  |  |  |  |  |  |  |  |  |  |  |  |  |  |  |  |  |  |  |  |  |  | -257 |
| CAN7_MOUSE | - | - | - | - | - | - | - | - | - | - | - | - |  |  |  |  |  |  |  |  |  |  |  |  |  |  |  |  |  |  |  |  |  |  |  |  |  |  |  |  |  |  |  |  |  |  |  |  |  |  |  |  |  |  |  |  |  |  |  |  |  | -263 |
| CAN7_HUMAN | - | - | - | - | - | - | - | - | - | - | - | - |  |  |  |  |  |  |  |  |  |  |  |  |  |  |  |  |  |  |  |  |  |  |  |  |  |  |  |  |  |  |  |  |  |  |  |  |  |  |  |  |  |  |  |  |  |  |  |  |  | -263 |
| E1BY84_CHICK | - | - | - | - | - | - | - | - | - | - | - | - |  |  |  |  |  |  |  |  |  |  |  |  |  |  |  |  |  |  |  |  |  |  |  |  |  |  |  |  |  |  |  |  |  |  |  |  |  |  |  |  |  |  |  |  |  |  |  |  |  | -263 |
| H9GX91_DANRE | - | - | - | - | - | - | - | - | - | - | - | - |  |  |  |  |  |  |  |  |  |  |  |  |  |  |  |  |  |  |  |  |  |  |  |  |  |  |  |  |  |  |  |  |  |  |  |  |  |  |  |  |  |  |  |  |  |  |  |  |  | -263 |
| F6SGI6_CIOIN | - | - | - | - | - | - | - | - | - | - | - | - |  |  |  |  |  |  |  |  |  |  |  |  |  |  |  |  |  |  |  |  |  |  |  |  |  |  |  |  |  |  |  |  |  |  |  |  |  |  |  |  |  |  |  |  |  |  |  |  |  | -260 |
| W4Y195_STRPU | - | - | - | - | - | - | - | - | - | - | - | - |  |  |  |  |  |  |  |  |  |  |  |  |  |  |  |  |  |  |  |  |  |  |  |  |  |  |  |  |  |  |  |  |  |  |  |  |  |  |  |  |  |  |  |  |  |  |  |  |  | -236 |
| R7V270_CAPTE | - | - | - | - | - | - | - | - | - | - | - | - |  |  |  |  |  |  |  |  |  |  |  |  |  |  |  |  |  |  |  |  |  |  |  |  |  |  |  |  |  |  |  |  |  |  |  |  |  |  |  |  |  |  |  |  |  |  |  |  |  | -261 |
| G4LXK3_SCHMA | - | - | - | - | - | - | - | - | - | - | - | - |  |  |  |  |  |  |  |  |  |  |  |  |  |  |  |  |  |  |  |  |  |  |  |  |  |  |  |  |  |  |  |  |  |  |  |  |  |  |  |  |  |  |  |  |  |  |  |  |  | -221 |
| T2M2K0_HYDVU | - | - | - | - | - | - | - | - | - | - | - | - |  |  |  |  |  |  |  |  |  |  |  |  |  |  |  |  |  |  |  |  |  |  |  |  |  |  |  |  |  |  |  |  |  |  |  |  |  |  |  |  |  |  |  |  |  |  |  |  |  | -261 |
| B3RQK1_TRIAD | - | - | - | - | - | - | - | - | - | - | - | - |  |  |  |  |  |  |  |  |  |  |  |  |  |  |  |  |  |  |  |  |  |  |  |  |  |  |  |  |  |  |  |  |  |  |  |  |  |  |  |  |  |  |  |  |  |  |  |  |  | -230 |
| I1CS59_RHIO9 | - | - | - | - | - | - | - | - | - | - | - | - |  |  |  |  |  |  |  |  |  |  |  |  |  |  |  |  |  |  |  |  |  |  |  |  |  |  |  |  |  |  |  |  |  |  |  |  |  |  |  |  |  |  |  |  |  |  |  |  |  | -258 |
| I1GER3_AMPQE | - | - | - | - | - | - | - | - | - | - | - | - |  |  |  |  |  |  |  |  |  |  |  |  |  |  |  |  |  |  |  |  |  |  |  |  |  |  |  |  |  |  |  |  |  |  |  |  |  |  |  |  |  |  |  |  |  |  |  |  |  | -258 |
| Q22143_CAEEL | - | - | - | - | - | - | - | - | - | - | - | - |  |  |  |  |  |  |  |  |  |  |  |  |  |  |  |  |  |  |  |  |  |  |  |  |  |  |  |  |  |  |  |  |  |  |  |  |  |  |  |  |  |  |  |  |  |  |  |  |  | -262 |
| L1JZN7_GUITH | - | - | - | - | - | - | - | - | - | - | - | - |  |  |  |  |  |  |  |  |  |  |  |  |  |  |  |  |  |  |  |  |  |  |  |  |  |  |  |  |  |  |  |  |  |  |  |  |  |  |  |  |  |  |  |  |  |  |  |  |  | -232 |
| D0NT34_PHYIT | - | - | - | - | - | - | - | - | - | - | - | - |  |  |  |  |  |  |  |  |  |  |  |  |  |  |  |  |  |  |  |  |  |  |  |  |  |  |  |  |  |  |  |  |  |  |  |  |  |  |  |  |  |  |  |  |  |  |  |  |  | -250 |
| A0A064B913_ASPOZ | - | - | - | - | - | - | - | - | - | - | - | - |  |  |  |  |  |  |  |  |  |  |  |  |  |  |  |  |  |  |  |  |  |  |  |  |  |  |  |  |  |  |  |  |  |  |  |  |  |  |  |  |  |  |  |  |  |  |  |  |  | -259 |
| A8N4X4_COPC7 | - | - | - | - | - | - | - | - | - | - | - | - |  |  |  |  |  |  |  |  |  |  |  |  |  |  |  |  |  |  |  |  |  |  |  |  |  |  |  |  |  |  |  |  |  |  |  |  |  |  |  |  |  |  |  |  |  |  |  |  |  | -277 |
| C0H5K1_PLAF7 | - | - | - | - | - | - | - | - | - | - | - | - |  |  |  |  |  |  |  |  |  |  |  |  |  |  |  |  |  |  |  |  |  |  |  |  |  |  |  |  |  |  |  |  |  |  |  |  |  |  |  |  |  |  |  |  |  |  |  |  |  | -431 |
| K1REZ8_CRAGI | - | - | - | - | - | - | - | - | - | - | - | - |  |  |  |  |  |  |  |  |  |  |  |  |  |  |  |  |  |  |  |  |  |  |  |  |  |  |  |  |  |  |  |  |  |  |  |  |  |  |  |  |  |  |  |  |  |  |  |  |  | -245 |
| F4NTM9_BATDJ | - | - | - | - | - | - | - | - | - | - | - | - |  |  |  |  |  |  |  |  |  |  |  |  |  |  |  |  |  |  |  |  |  |  |  |  |  |  |  |  |  |  |  |  |  |  |  |  |  |  |  |  |  |  |  |  |  |  |  |  |  | -271 |
| Q6F6K5_CAEEL | - | - | - | - | - | - | - | - | - | - | - | - |  |  |  |  |  |  |  |  |  |  |  |  |  |  |  |  |  |  |  |  |  |  |  |  |  |  |  |  |  |  |  |  |  |  |  |  |  |  |  |  |  |  |  |  |  |  |  |  |  | -194 |
| A8HZK8_CHLRE | - | - | - | - | - | - | - | - | - | - | - | - |  |  |  |  |  |  |  |  |  |  |  |  |  |  |  |  |  |  |  |  |  |  |  |  |  |  |  |  |  |  |  |  |  |  |  |  |  |  |  |  |  |  |  |  |  |  |  |  |  | -253 |
| A8J6T6_CHLRE | - | - | - | - | - | - | - | - | - | - | - | - |  |  |  |  |  |  |  |  |  |  |  |  |  |  |  |  |  |  |  |  |  |  |  |  |  |  |  |  |  |  |  |  |  |  |  |  |  |  |  |  |  |  |  |  |  |  |  |  |  | -321 |
| Z9JR51_9MICO | - | - | - | - | - | - | - | - | - | - | - | - |  |  |  |  |  |  |  |  |  |  |  |  |  |  |  |  |  |  |  |  |  |  |  |  |  |  |  |  |  |  |  |  |  |  |  |  |  |  |  |  |  |  |  |  |  |  |  |  |  | -215 |
| F7ZZH9_CELGA | - | - | - | - | - | - | - | - | - | - | - | - |  |  |  |  |  |  |  |  |  |  |  |  |  |  |  |  |  |  |  |  |  |  |  |  |  |  |  |  |  |  |  |  |  |  |  |  |  |  |  |  |  |  |  |  |  |  |  |  |  | -202 |
| Q7NDU2_GLOVI | - | - | - | - | - | - | - | - | - | - | - | - |  |  |  |  |  |  |  |  |  |  |  |  |  |  |  |  |  |  |  |  |  |  |  |  |  |  |  |  |  |  |  |  |  |  |  |  |  |  |  |  |  |  |  |  |  |  |  |  |  | -172 |
| I6AT95_9BACT | - | - | - | - | - | - | - | - | - | - | - | - |  |  |  |  |  |  |  |  |  |  |  |  |  |  |  |  |  |  |  |  |  |  |  |  |  |  |  |  |  |  |  |  |  |  |  |  |  |  |  |  |  |  |  |  |  |  |  |  |  | -198 |
| C7MGJ8_BRAFD | - | - | - | - | - | - | - | - | - | - | - | - |  |  |  |  |  |  |  |  |  |  |  |  |  |  |  |  |  |  |  |  |  |  |  |  |  |  |  |  |  |  |  |  |  |  |  |  |  |  |  |  |  |  |  |  |  |  |  |  |  | -197 |
| D0WP73_9ACTO | - | - | - | - | - | - | - | - | - | - | - | - |  |  |  |  |  |  |  |  |  |  |  |  |  |  |  |  |  |  |  |  |  |  |  |  |  |  |  |  |  |  |  |  |  |  |  |  |  |  |  |  |  |  |  |  |  |  |  |  |  | -212 |
| B0C8L1_ACAM1 | - | - | - | - | - | - | - | - | - | - | - | - |  |  |  |  |  |  |  |  |  |  |  |  |  |  |  |  |  |  |  |  |  |  |  |  |  |  |  |  |  |  |  |  |  |  |  |  |  |  |  |  |  |  |  |  |  |  |  |  |  | -225 |
| B3E5A8_GEOLS | - | - | - | - | - | - | - | - | - | - | - | - |  |  |  |  |  |  |  |  |  |  |  |  |  |  |  |  |  |  |  |  |  |  |  |  |  |  |  |  |  |  |  |  |  |  |  |  |  |  |  |  |  |  |  |  |  |  |  |  |  | -189 |
| D4YQ94_9MICO | - | - | - | - | - | - | - | - | - | - | - | - |  |  |  |  |  |  |  |  |  |  |  |  |  |  |  |  |  |  |  |  |  |  |  |  |  |  |  |  |  |  |  |  |  |  |  |  |  |  |  |  |  |  |  |  |  |  |  |  |  | -221 |
| PAPA_CARPA | - | - | - | - | - | - | - | - | - | - | - | - |  |  |  |  |  |  |  |  |  |  |  |  |  |  |  |  |  |  |  |  |  |  |  |  |  |  |  |  |  |  |  |  |  |  |  |  |  |  |  |  |  |  |  |  |  |  |  |  |  | -212 |
| RIM13_YEAST | - | - | - | - | - | - | - | - | - | - | - | - |  |  |  |  |  |  |  |  |  |  |  |  |  |  |  |  |  |  |  |  |  |  |  |  |  |  |  |  |  |  |  |  |  |  |  |  |  |  |  |  |  |  |  |  |  |  |  |  |  | -278 |
